# Proteomic Analysis of Excretory-Secretory Products of *Heligmosomoides polygyrus* Assessed with Next-Generation Sequencing Transcriptomic Information

**DOI:** 10.1371/journal.pntd.0001370

**Published:** 2011-10-25

**Authors:** Yovany Moreno, Pierre-Paul Gros, Mifong Tam, Mariela Segura, Rajesh Valanparambil, Timothy G. Geary, Mary M. Stevenson

**Affiliations:** 1 Institute of Parasitology and Centre for Host Parasite Interactions, McGill University, Ste-Anne de Bellevue, Quebec, Canada; 2 Centre for the Study of Host Resistance and Centre for Host Parasite Interactions, The Research Institute of McGill University Health Centre and Department of Medicine, McGill University, Montreal, Quebec, Canada; George Washington University, United States of America

## Abstract

The murine parasite *Heligmosomoides polygyrus* is a convenient experimental model to study immune responses and pathology associated with gastrointestinal nematode infections. The excretory-secretory products (ESP) produced by this parasite have potent immunomodulatory activity, but the protein(s) responsible has not been defined. Identification of the protein composition of ESP derived from *H. polygyrus* and other relevant nematode species has been hampered by the lack of genomic sequence information required for proteomic analysis based on database searches. To overcome this, a transcriptome next generation sequencing (RNA-seq) *de novo* assembly containing 33,641 transcripts was generated, annotated, and used to interrogate mass spectrometry (MS) data derived from 1D-SDS PAGE and LC-MS/MS analysis of ESP. Using the database generated from the 6 open reading frames deduced from the RNA-seq assembly and conventional identification programs, 209 proteins were identified in ESP including homologues of vitellogenins, retinol- and fatty acid-binding proteins, globins, and the allergen V5/Tpx-1-related family of proteins. Several potential immunomodulators, such as macrophage migration inhibitory factor, cysteine protease inhibitors, galectins, C-type lectins, peroxiredoxin, and glutathione S-transferase, were also identified. Comparative analysis of protein annotations based on the RNA-seq assembly and proteomics revealed processes and proteins that may contribute to the functional specialization of ESP, including proteins involved in signalling pathways and in nutrient transport and/or uptake. Together, these findings provide important information that will help to illuminate molecular, biochemical, and in particular immunomodulatory aspects of host-*H. polygyrus* biology. In addition, the methods and analyses presented here are applicable to study biochemical and molecular aspects of the host-parasite relationship in species for which sequence information is not available.

## Author Summary

Gastrointestinal (GI) nematode infections are major causes of human and animal disease. Much of their morbidity is associated with establishment of chronic infections in the host, reflecting the deployment of mechanisms to evade and modulate the immune response. The molecules responsible for these activities are poorly known. The proteins released from nematode species as excretory-secretory products (ESP) have potent immunomodulatory effects. The murine parasite *Heligmosomoides bakeri* (*polygyrus*) has served as a model to understand several aspects related to GI nematode infections. Here, we aimed to identify the protein components of *H. polygyrus* ESP through a proteomic approach, but the lack of genomic sequence information for this organism limited our ability to identify proteins by relying on comparisons between experimental and database-predicted mass spectra. To overcome these difficulties, we used transcriptome next-generation sequencing and several bioinformatic tools to generate and annotate a sequence assembly for this parasite. We used this information to support the protein identification process. Among the 209 proteins identified, we delineated particular processes and proteins that define the functional specialization of ESP. This work provides valuable data to establish a path to identify and understand particular parasite proteins involved in the orchestration of immune evasion events.

## Introduction

Gastrointestinal (GI) nematode infections are major causes of disease in both humans and animals. Infections with *Ascaris lumbricoides*, hookworms (*Necator americanus* and *Ancylostoma duodenalis*), *Trichuris trichiura*, and *Strongyloides stercoralis* are highly prevalent in developing countries, affecting ∼1 billion people and posing a burden estimated at ∼2 M DALYs (Disability-adjusted life years) (http://apps.who.int/ghodata) [1]. GI nematodes usually establish chronic infections, surviving in the host for considerable periods of time. This characteristic reflects the ability of these parasites to evade and modulate the host immune response from the early stages of infection while optimizing both feeding and reproduction [2], [3]. As a result, in addition to their commonly associated effects on host physiology including malnutrition, growth stunting, and anaemia, infection with GI nematodes influences the development and/or severity of co-occurring infections and immune-mediated diseases such as malaria or type 1 diabetes, respectively [4], 5.

Infection with the nematode *Heligmosomoides polygyrus*, a natural GI pathogen of mice, has provided a convenient experimental model to understand the biology of GI nematodes and the pathology associated with chronic infections with this class of helminth parasites [6]. Primary infection with *H. polygyrus* induces a highly polarized Th2 immune response in mice; despite induction of this response, the parasite survives and establishes a chronic infection with the differentiation and activation of host cell types that mediate potent immunoregulatory mechanisms, such as regulatory T cells and alternatively activated macrophages (AAMΦs) [7], [8]. Recent studies indicate that these regulatory responses, especially regulatory T cells, can be stimulated by treatment with *H. polygyrus* excretory-secretory products (ESP) [9]–[12]. These observations suggest that this fraction of the proteome contains many of the immunomodulatory factors responsible for evasion of the host immune response, but the proteins in ESP that mediate these effects remain largely unknown.

The use of mass-spectrometry based proteomics has overcome many limitations in the analysis and identification of helminth-derived proteins in ESP [13]. In general, these analyses achieve a remarkable sensitivity in protein identification if either genome, transcriptome, or proteome sequence information is available to support the interrogation of experimentally obtained mass spectra with peptide matching algorithms in database search programs [14]. However, most of this sensitivity is lost when assignation is based on homology with proteins identified in other species, as is the case for *H. polygyrus* and almost all other relevant parasitic nematode species for which sequence information is not available [15]–[17].

To better understand the molecular mechanisms that lead to the activation and modulation of the host immune response by GI nematodes, we used transcriptome next generation sequencing (RNA-seq) technologies and several bioinformatic tools to overcome the limitations in the proteomic analysis of ESP from *H. polygyrus*. Illumina sequencing (www. illumina.com) was employed to generate transcriptomic sequence data in a rapid and cost-efficient way [18]. The transcriptome assembly was used to identify proteins in the ESP using an experimental proteomic approach.

## Methods

### Ethics statement

Animal procedures were conducted in accordance with the guidelines and policies of the Canadian Council on Animal Care and the principles set forth in the Guide for the Care and Use of Laboratory Animals, Animal Resources Centre, McGill University. The protocol was approved by the McGill University Animal Care Committee (Permit Number: 4543). All efforts were made to minimize discomfort and suffering to the animals during handling and manipulation.

### Parasites

*H. polygyrus* was maintained and propagated in male BALB/c mice (Charles River Laboratories, St. Constant, Canada) by oral gavage inoculation of 400–450 third-stage larvae (L3) as described [19]. Adult parasites were collected from the small intestine on day 21 post infection under a dissection microscope. Worms were washed extensively with sterile endotoxin-free PBS (Invitrogen, Burlington, ON, Canada) containing 80 µg/ml gentamicin (Schering, Montreal, QC, Canada), 100 U/ml penicillin G, 100 µg/ml streptomycin (Invitrogen), and 20 µg/ml polymyxin B (Sigma, St. Louis, MO). Mice were housed in the Animal Care Facility at the Research Institute of the McGill University Health Centre.

### RNA extraction, cDNA library preparation and Illumina sequencing

For RNA extraction, viable worms were harvested from 6 infected mice on day 21 post-infection, and ∼1000 adult female and male worms free of host tissue were selected and extensively washed. After resuspension in 0.5 ml PBS, 3.0 ml Trizol (Invitrogen) were added to the worm suspension. Worms were disrupted with a Polytron homogenizer at maximum speed for 3 min with the tube positioned on ice. Following centrifugation at 12,000× g for 10 min at 4°C, the clear upper phase was collected and extracted with chloroform. After centrifugation at 12,000× g for 10 min at 4°C, the upper aqueous phase was collected, and RNA was precipitated with isopropanol. RNA was centrifuged at 12,000× g for 10 min at 4°C. The RNA pellet was washed with 75% ethanol, followed by centrifugation at 7,500× g for 5 min, and the RNA pellet was dissolved in water. The 260/280 ratio of the sample was >1.6. The RNA samples were stored at −70°C and until sequencing at the McGill University and Génome Québec Innovation Centre.

Total RNA quality was verified on an RNA chip using an Agilent 2100 Bioanalyzer and quantified using a NanoDrop ND-1000 UV-VIS spectrophotometer (Thermo Fisher). A cDNA library was prepared from 5 µg total RNA using the mRNA-Seq Sample Preparation Kit (Illumina), according to the manufacturer's recommendations. Quality of the library was verified on a DNA 1000 chip using the Agilent 2100 Bioanalyzer and quantified by PicoGreen fluorimetry. The library was subjected to 108 single-read cycles of sequencing on an Illumina Genome Analyzer IIx as per the manufacturer's protocol. Cluster generation was performed on a c-Bot (Illumina) with a single read cluster generation kit. Sequencing was performed once using a 36 cycle sequencing kit v4.

### ESP preparation

ESP were prepared using a modification of previously described methods [9]. Briefly, adult worms were collected as described above, and viable worms were selected, washed, and cultured at a density of ∼1000 worms per ml of serum-free RPMI 1640 medium (Invitrogen) supplemented with 2% glucose (Sigma) and antibiotics for 36 h at 37°C. The supernatant was harvested, centrifuged at 8,000× g for 10 min to remove eggs and debris, and concentrated using an Amicon centrifugal filter device with a 3 kDa cut-off (Millipore, Billerica, MA). The protein concentration in ESP preparations was determined with a Bradford Reagent kit (Bio-Rad, Hercules, CA) according to the manufacturer's instructions. For proteomic analysis, a pooled sample of ESP prepared from 4 harvests of adult worms from a total of 40 mice was used. The 4 ESP preparations were pooled after their migration patterns on 4–20% acrylamide SDS-PAGE were confirmed to be similar. Pooled ESP was stored at −80°C until analysis at the McGill University and Génome Québec Innovation Centre.

### 1D electrophoresis and band excision

ESP were resuspended in loading buffer containing 2-mercaptoethanol, and ∼100 µg protein were separated by SDS-PAGE through a 3 cm gradient gel (7–15% acrylamide) as described [20]. Following gel staining with Coomassie Brilliant Blue G, the entire lane was subjected to automated band excision using the Picking Workstation ProXCISION (Perkin Elmer) to generate 15 bands per lane (5–7 pieces/line).

### Tryptic digestion and Liquid Chromatography – Tandem Mass Spectrometry (LC-MS/MS) analysis

Proteins from gel bands were subjected to reduction, cysteine-alkylation, and in-gel tryptic digestion in a MassPrep Workstation (Micromass, Manchester, UK) as previously described [20]. Twenty µl of the tryptic digest solution were injected on a Zorbax 300SB-C18 pre-column (5×0.3 mm, 5 µm) previously equilibrated with water containing acetonitrile (5%) and formic acid (0.1%) using the Micro Well-plate sampler and the IsoPump modules of an Agilent 1100 Series Nanoflow HPLC. Following washing for 5 min at 15 µl/min, the pre-column was back-flushed to a 75 µm i.d. PicoFrit column (New Objective, Woburn, MA) filled with 10 cm of BioBasic C18 packing (5 µm, 300 Å) by the acetonitrile gradient supplied by the Agilent series 1100 Nanopump to allow elution of the peptides towards the mass spectrometer at a flow rate of 200 ηl/min as described [20]. Eluted peptides were analyzed in a Q-TOF micro (Waters Micromass, Manchester, UK) equipped with a Nanosource modified with a nanospray adapter (New Objective, Woburn, MA). The MS survey scan was set to 1 s (0.1 s interscan) and recorded from 350 to 1,600 *m/z*. MS/MS scans were acquired from 50 to 1,990 *m/z*, scan time was 1.35 s, and the interscan interval was 0.15 s. Doubly and triply charged ions were selected for fragmentation with collision energies calculated using a linear curve from reference collision energies.

MS raw data from a single run were acquired on the Data Directed Analysis feature in the MassLynx (Micromass) software with a 1, 2, 4 duty cycle (1 sec in MS mode 2 peptides selected for fragmentation, maximum of 4 sec in MS/MS acquisition mode). MS/MS raw data were transferred from the Q-TOF Micro computer to a 50 terabyte server and automatically manipulated for generation of peaklists by employing Distiller version 2.3.2.0 (http://www.matrixscience.com/distiller.htmls) with peak picking parameters set at 5 for Signal Noise Ration (SNR) and at 0.4 for Correlation Threshold (CT). The peaklisted data were then searched by employing Mascot version 2.3.01 (http://www.matrixscience.com) and X! Tandem version 2007.01.01.1 (http://www.thegpm.org) against the 6 open reading frames (ORF) translation of the transcriptomic assembly (see below). Searches were restricted to up to 1 missed (trypsin) cleavage, fixed carbamidomethyl alkylation of cysteines, variable oxidation of methionine, 0.5 mass unit tolerance on parent and fragment ions, and monoisotopic. Scaffold (version Scaffold_2_05_02, Proteome Software Inc., Portland, OR) was used to validate MS/MS-based peptide and protein identifications. Peptide identifications were accepted if they could be established at greater than 95.0% probability as specified by the Peptide Prophet algorithm [21]. Protein identifications were accepted if they could be established at greater than 95.0% probability and contained at least 2 identified peptides. Protein probabilities were assigned by the Protein Prophet algorithm [22]. Proteins that contained similar peptides and could not be differentiated based on MS/MS analysis alone were grouped to satisfy the principles of parsimony.

### Bioinformatics

Reads from Illumina sequencing were trimmed in a process that consisted of search and clipping for adapter sequences, elimination of the first 16 bases of the reads to remove random hexamers, and quality trimming using a Q20 threshold on the 3′ end. The assembly was done with Velvet 1.0.13 with a kmer value set at 43 [23]. Oases 0.1.6 (http://www.ebi.ac.uk/~zerbino/oases/) was then used for final transcriptome assembly.

Loci generated from the Oases assembler were subjected to analysis by BLASTx and BLASTn to identify putative homologues in *C. elegans*, other parasitic nematodes, and organisms other than nematodes (e-value of ≤1e-05). Full assembly will be available at Nembase4 (http://www.nematodes.org/nembase4/) (Submission date: May 6^th^, 2011) [24].

Gene Ontology (GO) annotations were performed using BLAST2GO [25]. Mapping of GO terms was performed on the hits retrieved from the initial search with BLASTx for protein homologues against the NCBI non-redundant database with a minimum expected value of 1×10^−3^ and a high scoring segment pair cut-off of 33. The annotation algorithm was set with default parameters; pre-eValue-Hit-Filter of 1×10^−6^, annotation cut-off of 55, and GO weight of 5. Identification of enriched GO terms in the secretome dataset compared to the transcriptome was done by assessing *P* values from Fisher's exact tests applying robust false discovery rate (FDR) using the integrated framework Gossip [26].

InterProScan [27], [28] searches were performed using the built-in feature of BLAST2GO using the conceptual translation from the longest ORF of each locus. Enrichment analysis of exported InterPro terms in the ESP vs. transcriptome datasets was also performed by assessing adjusted *P* values to control for FDR from Fisher's exact tests run using FatiGO [29] on the integrative online platform Babelomics (http://babelomics.bioinfo.cipf.es) [30].

## Results

### Protein identification of H. polygyrus ESP using protein homologues for peptide assignation

To identify proteins in *H. polygyrus* ESP, ∼100 µg ESP were separated by SDS-PAGE. The entire lane was excised in 15 pieces, digested with trypsin, and analyzed by LC-MS/MS. A preliminary protein identification attempt was performed on the complete MS data set (10,227 spectra) using the protein sequences from nematodes in the UniProt database (taxonomy ID:6231, September 17, 2010) as a search source for the peptide matching algorithms. After validation with Scaffold (v. 2_05_02), 20 proteins were assigned (95% probability) with a total number of assigned spectra ranging between 2 to 12 and between 2 to 7 unique peptides assigned per sequence. Nineteen of 20 identified proteins were homologues of proteins from nematodes other than *H. polygyrus* (Table S1).

### A transcriptomic analysis of adult H. polygyrus

To provide a more suitable information source for the peptide assignation software and to increase the number of proteins identified in the *H. polygyrus* ESP, an RNA-seq analysis of this organism was carried out. Using the GAIIx platform from Illumina, ∼24.7 million reads of raw data, amounting to >2.7 Gbp, were obtained from *H. polygyrus* poly-A selected mRNA. Initial assembly was performed after the removal of adapters, random hexamer primer sequences, and quality control trimming using Velvet 1.0.13 [23], generating 76,616 contigs. Final assembly with Oases 0.1.6 resulted in 33,641 total transcripts (isoforms) in 29,918 loci (3723 alternative splice events) (Table 1). These values do not include sequences <100 bp, which were removed for downstream analysis.

**Table 1:** Summary of the transcriptomic assembly and ESP subset annotations.

**Assembly Statistics**		
Number of transcripts (Isoforms) >100 bp	33,641	
Number of Loci >100 bp	29,918	
Average length (SD)	561.0+(566.2)	
Blastx (E>10^−5^)		
*C. elegans*	18,816 (55.9%)	
*B. malayi*	15,338 (45.6%)	
non-redundant	19,196 (57.1%)	
**Total Transcriptome**		**ESP subset**
**(33,641 seqs)**		**(208 seqs)**
**GO annotation**		
Number of Seqs with at least 1 GO term (%)	14,094 (41.9)	113 (54.3)
**Total number of terms (unique)**	85,884 (6,647)	642 (306)
Cellular Part terms (unique)	17,180 (764)	99 (52)
*Number of Sequences* (%)	8,694 (25.8)	47 (22.6)
Molecular Function terms (unique)	25,715 (1,805)	210 (87)
*Number of Sequences* (%)	11,671 (34.7)	107 (51.4)
Biological Process terms (unique)	42,989 (4,078)	333 (167)
*Number of Sequences* (%)	11,296 (33.6)	89 (42.8)
**InterProScan (IPS) annotation**		
Seqs with at least 1 protein signature (%)	17,342 (51.5)	158 (76.0)
Annotated InterPro Entries	4,547	134
*Domains*	2,204	70
*Protein Families*	1,896	41
Seqs with annotated InterPro Entries (%)	12,126 (36)	157 (75.4)
*Seqs annotated with predicted domains* (%)	15,435 (45.9)	104 (50)
*Seqs annotated with protein families* (%)	5,760 (17.1)	70 (33.7)

Searching for protein homologues in the *H. polygyrus* assembly with BLASTx identified 18,816 (55.9%) transcripts sharing homology with proteins from *C. elegans* (E cut-off 1×10^−5^) and 15,338 (45.6%) with proteins from *Brugia malayi* (Table 1). Only 4 sequences were found to return mouse proteins as the first BLASTx output (E cut-off 1×10^−15^), indicating a low degree of host RNA contamination in the preparations.

Functional annotation using BLAST2GO allowed us to assign GO terms to 14,094 (41.9%) sequences; 764 different cellular component terms were assigned to 8,694 sequences, 1,805 molecular function terms to 11,671 sequences, and 4,078 biological process terms to 11,296 sequences (Table 1). The most frequently annotated GO terms within these three categories were “integral to membrane” (GO:0016021, 1,513 sequences), “protein binding” (GO:0005515; 3,782 sequences), and “embryonic development ending in birth or egg hatching” (GO:0009792, 2,239 sequences) (Tables 2 and S2).

**Table 2:** Most abundant GO terms mapped and annotated using Blast2GO in the transcriptome and ESP datasets.

Transcriptome					ESP subset		
GO code			GO Term	Seqs (%)	GO code	GO Term	Seqs (%)
**Cellular Component**	1	GO:0016021	integral to membrane	1,513 (4.5)	GO:0005737	cytoplasm	14 (6.7)
	2	GO:0005634	nucleus	1,129 (3.4)	GO:0005576	extracellular region	6 (2.9)
	3	GO:0005737	cytoplasm	990 (2.9)	GO:0005615	extracellular space	6 (2.9)
	4	GO:0016020	membrane	743 (2.2)	GO:0016021	integral to membrane	4 (1.9)
	5	GO:0005829	cytosol	669 (2.0)	GO:0016020	membrane	4 (1.9)
**Molecular Function**	1	GO:0005515	protein binding	3,782 (11.2)	GO:0005515	protein binding	43 (20.7)
	2	GO:0005524	ATP binding	1,327 (3.9)	GO:0008233	peptidase activity	8 (3.8)*
	3	GO:0005488	binding	937 (2.8)	GO:0020037	heme binding	7 (3.4)*
	4	GO:0008270	zinc ion binding	643 (1.9)	GO:0008237	metallopeptidase activity	7 (3.4)*
	5	GO:0046872	metal ion binding	590 (1.8)	GO:0019825	oxygen binding	7 (3.4)*
**Biological Process**	1	GO:0009792	embryonic development ending in birth or egg hatching	2,239 (6.7)	GO:0008340	determination of adult lifespan	28 (13.5)*
	2	GO:0002119	nematode larval development	1,655 (4.9)	GO:0009792	embryonic development ending in birth or egg hatching	27 (13.0)
	3	GO:0040010	positive regulation of growth rate	1,513 (4.5)	GO:0040010	positive regulation of growth rate	26 (12.5)*
	4	GO:0000003	reproduction	1,311 (3.9)	GO:0040011	locomotion	10 (4.8)
	5	GO:0040011	locomotion	992 (2.9)	GO:0000003	reproduction	10 (4.8)

*Indicates GO term enrichment in the ESP subset when compared to the transcriptome dataset (FDR<0.5).

**Figure 1:**
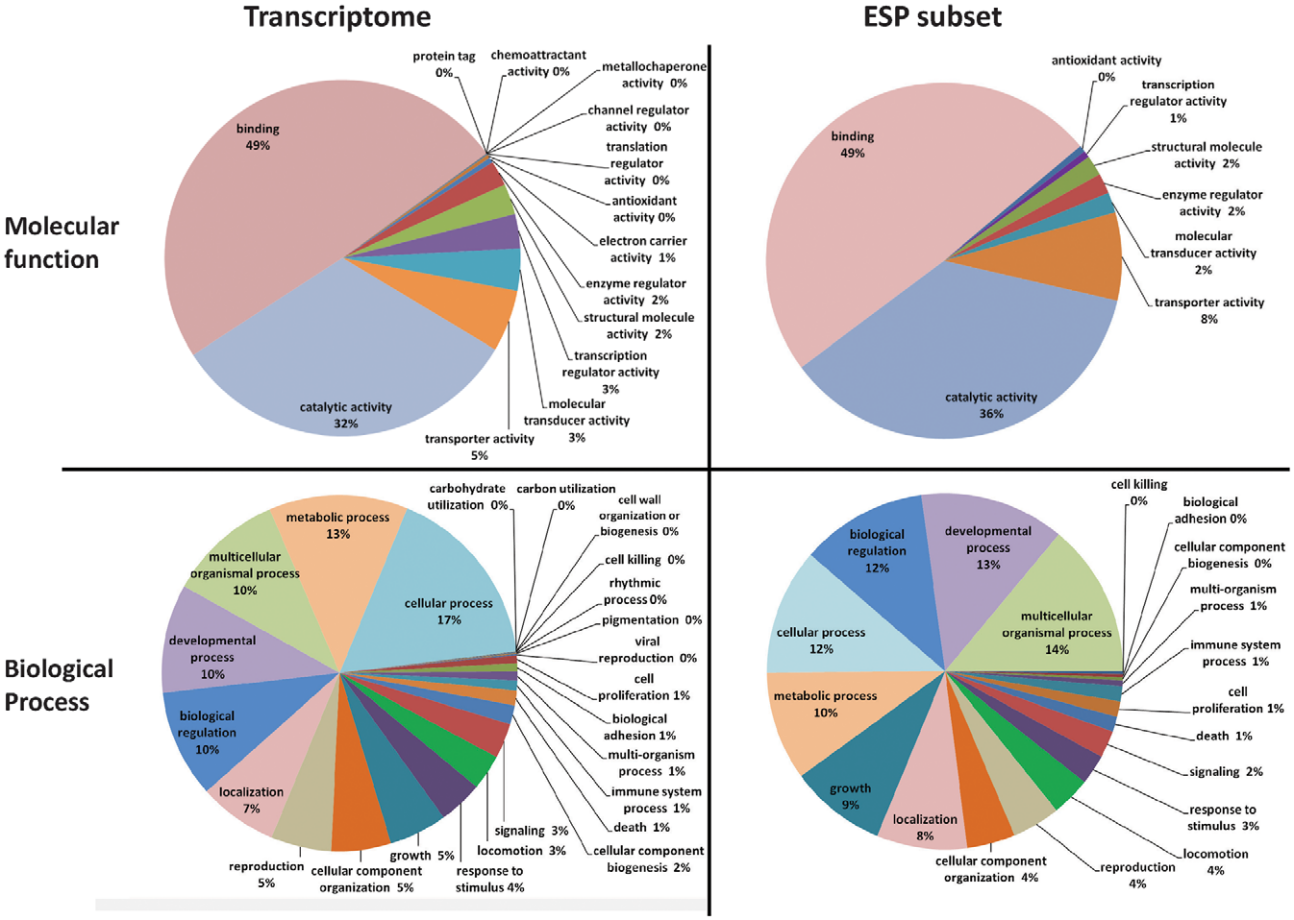
Molecular function and biological process GO terms (level 2) for the transcriptome and ESP subset.

Distribution of GO terms at level two indicated that “binding” (GO:0005488, 49% of annotated sequences) and “catalytic activity” (GO:0003824, 32%) were the two major molecular function categories (Figure 1, *left*). In the case of biological process, the most represented categories at level two were “cellular process” (GO:0009987, 17%), “metabolic process” (GO:0008152, 13%), “multicellular organismal process” (GO:0032501, 10%), “developmental process” (GO:0032502, 10%), and “biological regulation” (GO:0065007, 10%).

Functional domains and protein families were assigned to *H. polygyrus* transcripts using InterProScan [27], [28]; 17,342 (51.5%) sequences retrieved at least one protein signature, 2,204 different functional domains were predicted in 15,435 (45.9%) sequences, and 1,896 protein families in 5,760 sequences (17.1%) (Table 1). A protein kinase-like domain (IPR011009) was found in 360 sequences (1.1%), which represents the most frequently found predicted domain in the annotated dataset. In the case of protein families, the P-type, K/Mg/Cd/Cu/Zn/Na/Ca/Na/H-transporter family (IPR001757) was the most abundant, found on 80 sequences (0.2%) (Table 3).

**Table 3:** Five most abundant domains and families inferred from InterProScan in the transcriptome and ESP datasets.

Transcriptome				Secretome		
InterPro Accession		Name	Seqs (%)	InterPro Accession	Name	Seqs (%)
**Domains**						
1	IPR011009	Protein kinase-like domain	360 (1.1)	IPR014044	CAP domain	25 (12.0)*
2	IPR013783	Immunoglobulin-like fold	288 (0.9)	IPR006026	Peptidase, metallopeptidase	6 (2.9)*
3	IPR015943	WD40/YVTN repeat-like-containing domain	235 (0.7)	IPR000436	Sushi/SCR/CCP	5 (2.4)*
4	IPR015880	Zinc finger, C2H2-like	223 (0.7)	IPR001747	Lipid transport protein, N-terminal	5 (2.4)*
5	IPR016024	Armadillo-type fold	176 (0.5)	IPR008753	Peptidase M13	5 (2.4)*
**Families**						
1	IPR001757	ATPase, P-type, K/Mg/Cd/Cu/Zn/Na/Ca/Na/H-transporter	80 (0.2)	IPR001283	Allergen V5/Tpx-1-related	18 (8.7)*
2	IPR002198	Short-chain dehydrogenase/reductase SDR	69 (0.2)	IPR000718	Peptidase M13, neprilysin	8 (3.8)*
3	IPR001283	Allergen V5/Tpx-1-related	68 (0.2)	IPR001534	Transthyretin-like	5 (2.4)*
4	IPR020685	Tyrosine-protein kinase	61 (0.2)	IPR008632	Nematode fatty acid retinoid binding	3 (1.4)*
5	IPR020636	Calcium/calmodulin-dependent protein kinase-like	56 (0.2)	IPR009283	Apyrase	3 (1.4)*

*Indicates enrichment of the domain or the family in the ESP subset when compared to the transcriptome dataset (FDR<0.5).

### Use of transcriptomic data for assessing H. polygyrus ESP composition

The translation of the 6 ORFs of each transcript from the RNA-seq assembly was used as input for the matching algorithms in the protein identification software. Using this strategy, 209 proteins were identified with a total number of assigned spectra between 132 and 2 with 2 to 19 unique peptides assigned per sequence. It should be noted that one sequence appears twice as it was assigned to 2 different ORFs (Locus_541_Transcript_1/4_Confidence_0.692, frames 4 and 5) (Tables 4 and S3). Manual verification of peptide assignments showed that all the identified peptides group in a single ORF.

**Table 4:** Most abundant proteins based on the total spectra identified in ESP from adult *H. polygyrus*.

Protein Name	Blast2GO Seq. Description	Accession Numbers	P (%)	SC	UP	Closest Blastx Hit Description	Closest Blast HitSpecie	Closest Blast Hit Accession Number	Blastx Hit eValue
**L_272_T_4/4_C_0.700_3**	venom-allergen-like protein family member (vap-1)	L_272_T_4_0.700_3	100	132	19	two-domain activation associated secreted protein ASP4 precursor	*Ostertagia ostertagi*	CAO00417.1	2E-89
**L_566_T_2/6_C_0.476_3**	globin	L_566_T_2_0.476_3 L_566_T_3_0.571_3 L_566_T_4_0.524_3 L_566_T_5_0.762_2 L_566_T_6_0.762_3	100	82	5	Globin-like host-protective antigen	*Trichostrongylus colubriformis*	P27613.1	3E-30
**L_120_T_1/8_C_0.410_1**	secreted protein asp-2	L_120_T_1_0.410_1 L_120_T_5_0.436_1 L_120_T_8_0.462_1	100	74	6	secreted protein 4 precursor	*Ancylostoma caninum*	AAO63576.1	3E-26
**L_384_T_8/9_C_0.306_5**	globin	L_384_T_8_0.306_5	100	73	10	Globin-like host-protective antigen	*Trichostrongylus colubriformis*	P27613.1	3E-55
**L_2405_T_4/4_C_0.700_4**	secreted protein asp-2	L_2405_T_4_0.700_4	100	53	11	secreted protein 6 precursor	*Ancylostoma caninum*	AAO63578.1	8E-20
**L_2261_T_1/1_C_1.000_4**	globin	L_2261_T_1_1.000_4	100	47	7	Globin-like host-protective antigen	*Trichostrongylus colubriformis*	P27613.1	4E-39
**L_73_T_5/5_C_0.619_4**	vit-2	L_73_T_5_0.619_4	100	46	10	C. briggsae CBR-VIT-6 protein	*Caenorhabditis briggsae*	XP_002634040.1	6E-37
**L_94_T_3/4_C_0.429_4**	C. elegans protein confirmed by T evidence	L_94_T_3_0.429_4 L_94_T_4_0.429_1	100	46	8	LYSozyme family member (lys-8)	*Caenorhabditis elegans*	NP_495083.1	5E-30
**L_122_T_10/10_C_0.312_3**	vitellogenin structural genes (yolk protein genes) family member (vit-2)	L_122_T_10_0.312_3	100	45	10	VITellogenin structural genes (yolk protein genes) family member (vit-1)	*Caenorhabditis elegans*	NP_509305.1	1E-45
**L_2367_T_1/3_C_0.375_1**	ll20 15kda ladder antigen	L_2367_T_1_0.375_1	100	44	17	DVA-1 polyprotein	*Dictyocaulus viviparus*	Q24702.1	2E-145
**L_93_T_5/6_C_0.444_2**	vitellogenin structural genes (yolk protein genes) family member (vit-2)	L_93_T_5_0.444_2	100	43	10	VITellogenin structural genes (yolk protein genes) family member (vit-2)	*Caenorhabditis elegans*	NP_001123117.1	4E-53
**L_207_T_17/20_C_0.196_1**	briggsae cbr-vit-5 protein	L_207_T_17_0.196_1	100	41	7	VITellogenin structural genes (yolk protein genes) family member (vit-4)	*Caenorhabditis elegans*	NP_508612.1	3E-35
**L_290_T_3/3_C_0.833_3**	briggsae cbr-vit-2 protein	L_290_T_3_0.833_3	100	35	4	C. briggsae CBR-VIT-2 protein	*Caenorhabditis briggsae*	XP_002644638.1	4E-32
**L_211_T_5/5_C_0.765_5**	vitellogenin structural genes (yolk protein genes) family member (vit-6)	L_211_T_5_0.765_5	100	30	7	Vitellogenin-6	*Oscheius sp.* (strain CEW1)	Q94637.1	8E-39
**L_3_T_3/3_C_0.875_4**	vitellogenin structural genes (yolk protein genes) family member (vit-2)	L_3_T_3_0.875_4	100	28	7	Vitellogenin-6	*Oscheius sp.* (strain CEW1)	Q94637.1	6E-31
**L_2733_T_1/1_C_1.000_3**	globin	L_2733_T_1_1.000_3	100	27	7	Globin-like host-protective antigen	*Trichostrongylus colubriformis*	P27613.1	5E-56
**L_6303_T_1/1_C_1.000_1**	ancylostoma-secreted protein 1 precursor	L_6303_T_1_1.000_1	100	27	2	ancylostoma-secreted protein 1 precursor	*Ancylostoma duodenale*	AAD13339.1	2E-04
**L_927_T_1/2_C_1.000_6**	NA	L_927_T_1_1.000_6 L_927_T_2_1.000_4	100	25	2				
**L_160_T_5/5_C_0.733_4**	secreted protein asp-2	L_160_T_5_0.733_4	100	23	7	secreted protein 5 precursor	*Ancylostoma caninum*	AAO63577.1	3E-38
**L_407_T_1/2_C_1.000_5**	vitellogenin structural genes (yolk protein genes) family member (vit-6)	L_407_T_1_1.000_5	100	23	3	Vitellogenin-6	*Oscheius sp.* (strain CEW1)	Q94637.1	4E-15
**L_7106_T_1/1_C_1.000_2**	acetylcholinesterase 2	L_7106_T_1_1.000_2	100	23	14	putative neuromuscular acetylcholinesterase	*Dictyocaulus viviparus*	AAS49411.1	6E-169

Protein name identifiers are derived from the original transcriptome assembly nomenclature (L = locus, T = transcript, C = confidence) from which the conceptual translation frame number was added to the identifiers. **P (%)** = Protein identification probability (%); **SC** = Number of spectral counts; **UP** = Number of unique peptides.

Annotations from the non-redundant list of ESP hits (208 proteins) were extracted from the full transcriptome data set for further analysis. 642 GO terms could be annotated to sequences from the ESP subset (54.8% of the identified sequences), identifying 52 different cellular component terms in 47 (22.6%) sequences, 87 molecular functions in 107 (51.4%) sequences, and 167 biological processes in 89 (42.8%) sequences (Table 1).

At Level 2, within the molecular function category, 8 of the 14 terms initially found in the full transcriptome dataset were also found in the ESP subset. The GO terms “binding” (GO:0005488, 49% of annotated sequences) and “catalytic activity” (GO:0003824, 36%) were the most abundant terms in this category (Figure 1, *right*). In the biological process category, 19 of 25 terms were found in the ESP subset. Although the proportion of annotated terms in the ESP subset was slightly different than in the whole transcriptome dataset, the terms “multicellular organismal process” (GO:0032501, 14%), “developmental process” (GO:0032502, 13%), “biological regulation” (GO:0065007, 12%), “cellular process” (GO:0009987, 12%), and “metabolic process”( GO:0008152, 10%) were the most represented terms in both (Figure 1, left panels).

InterProScan hits assigned to the ESP subset predicted at least one protein signature for 158 (76.0%) sequences, identifying 70 functional domains in 104 (50.0%) sequences and 41 protein families for 70 (33.7%) sequences. The cysteine-rich secretory protein, antigen 5, and pathogenesis-related 1 protein (CAP) domain (IPR014044) with 25 (12.0%) sequences identified, was the most abundant domain in the ESP subset. The allergen V5/Tpx-1-related family (IPR001283), associated with the CAP domain, was the most prevalent found in the ESP subset (Table 3).

The proteins were organized according to the number of assigned spectra, indicative of protein abundance (Table 4) [31]. The most abundant hits organized in this manner were categorized into 3 main groups according to their annotated features. The first group is the proteins predicted to contain the CAP domain belonging to the allergen V5/Tpx-1-related family. This group of proteins is described in the annotation tables as homologues of venom allergen-like proteins (VAL), *A. caninum* secreted proteins, or activation-associated secreted proteins (ASP). The second group is composed of globin homologues. Proteins found within this group were annotated with the biological process GO term “oxygen transport” (GO:0015671) and the molecular function terms “heme binding” (GO:0020037), “oxygen transporter activity” (GO:0005344), and “oxygen binding” (GO:0019825). Although not predicted from the InterproScan in all these sequences, the globin-like domain (IPR009050) and globin family (IPR000971) were also annotated to some of these hits. The third group of most abundant proteins contains vitellogenin (Vtg) homologues. Most of these proteins are predicted to contain the characteristic Vtg open β-sheet (IPR15255, IPR 15817) domain as well as domains associated with lipid transport (IPR015819, IPR001747, and IPR015816) and GO terms associated with the molecular function of “protein binding” (GO:0005515) and the biological processes “embryonic development ending in birth or egg hatching” (GO:0009792), “determination of adult lifespan” (GO:0008340), and “positive regulation of growth rate” (GO:0040010) (Figure 1, right panels).

### Enrichment analysis delineates functional specialization of ESP

GO terms enrichment analysis using GOSSIP [26] identified terms that were over-represented in the ESP subset compared to the total transcriptome dataset (Table S4). Using adjusted *P*-values to control FDR (significance set at *p*<0.05) as criterion for statistical significance, 14 terms within the biological process category and 8 within the molecular function category were enriched in the ESP subset.

In accordance with the number of globin homologues found in the ESP subset, the biological process term “oxygen transport” (GO:0015671) and its parent “gas transport” (GO:0015669) were enriched in the ESP subset. Consistent with the globins and their putative role in oxygen transport via heme prosthetic groups, the molecular function terms “oxygen binding” (GO:0019825), “oxygen transporter activity” (GO:0005344), and “heme binding” (GO:00200037), together with their parent terms, “iron ion binding” (GO:0005506) and “tetrapyrrole binding” (GO:0046906), were also enriched in this subset.

Two other groups of hierarchically-related enriched biological process terms were delineated for their association with Vtg homologues in the ESP subset. The first group comprises the term “determination of adult lifespan” (GO:0008340) and its parent “multicellular organismal process” (GO:0032501). The second group consists of “positive regulation of growth rate” (GO:0040010) and parent terms “regulation of growth rate” (GO:0040009), “positive regulation of growth” (GO:0045927), and “regulation of growth” (GO:0040008). The identification of 3 homologues of glutathione-S-transferase also accounts for the enrichment of these terms.

In the molecular function category, two groups of enriched terms were associated with proteins of lower relative abundance. One group includes homologues of retinol and/or fatty acid binding protein as well as repetitive ladder antigens and Vtg homologues, which have the putative ability to bind and transport vitamin A and/or lipids. These proteins were annotated under the terms “retinol binding” (GO:0019841) and their parents, “retinoid binding” (GO:0005501), “isoprenoid binding” (GO:0019840), and “lipid binding” (GO:0008289). The other group is composed of certain proteases in the ESP subset, particularly several zinc metallopeptidase homologues. GO annotations in this group included the term “metallopeptidase activity” (GO:0008237) and the parent terms “peptidase activity acting on L-aminoacid peptides” (GO:0070011) and “peptidase activity” (GO:0008233).

In addition, the molecular function terms “intramolecular oxidoreductase activity” (GO:0016860) and “nucleoside diphosphate metabolic process” (GO:0009132) were also enriched in the ESP dataset. The first was associated with homologues of protein disulfide isomerase, triosephosphate isomerase (TPI), and macrophage migration inhibitory factor (MIF). The latter included homologues of nucleoside diphosphate kinases (NDPK), calcium activated nucleosidases, and ribonucleotide reductases (RNR).

Furthermore, InterPro domain enrichment analysis was performed using FatiGO [29] (Table S5). Likewise, adjusted *P*-values to control FDR were used as criteria for statistical significance (*p*<0.05); 23 domains and families were enriched in the ESP subset compared to the transcriptome dataset. Consistent with what was found in the enrichment analysis of GO terms, there was an enrichment of predicted families and domains associated with homologues of peptidases, globins, nucleosidases, glutathione-S-transferases, Vtg and retinol and/or fatty acid binding proteins. In addition, CAP domain (IPR014044) and its related allergen V5/Tpx-1-related family (IPR001283) and Ves allergen (IPR002413), along with the transthyretin-like family (IPR0001534), were enriched in the ESP dataset.

## Discussion

The ESP fraction of the proteome from parasitic nematodes is thought to contain many of the effector molecules that contribute in a direct or indirect way to establishment and survival within the host [32]. The *H. polygyrus*-mouse model is a convenient system for the study of human chronic gastrointestinal parasitism; potent immunomodulatory effects of ESP preparations from this parasite have been documented [9]–[12]. Specification of the protein composition of ESP is an important step toward compiling a comprehensive list of the proteins responsible for these effects. In addition, the transcriptomic analysis-based protein identification presented here highlights other aspects related to the biology of GI nematode infections that may illuminate new therapeutic strategies.

Proteomic approaches involving mass spectrometry have been applied for the characterization of ESP in several helminth species [33]. Protein identification in this manner has typically been empowered by the availability of information resulting from genome sequencing projects. Our preliminary results exemplify how the lack of this type of information and the reliance on sequences of protein homologues from different nematode species severely limit protein identification of *H. polygyrus* ESP; these factors would similarly limit such analyses from other unsequenced species.

To overcome this limitation, we sequenced the transcriptome of *H. polygyrus* using Illumina technology to provide the peptide matching software with the resulting RNA-seq *de novo* assembly. Next-generation sequencing technologies applied to the study of parasitic nematode transcriptomes offer an efficient way to understand how these organisms orchestrate their biochemical and molecular processes within the host [34]–[36]. However, we show here that its potential includes the use of this information to study specific aspects of the proteome. In particular, the *H. polygyrus* RNA-seq assembly was used as a reference for the identification of proteins present in the ESP.

Mass spectrometry-based proteomics has started to be exploited for the validation and/or correction of sequence datasets and associated annotations [37]. To a certain extent, this is the case for the present analysis. On the other hand, the overall output of the protein identification process is dependent on the searching space explored, in this case the 6 ORFs of the RNA-seq assembly. In addition to the sequence coverage, factors that may affect the quality of the *de novo* RNA-seq assembly include the performance of the assembly program as well as errors in individual reads during sequencing and genetic variation in the transcribed sequences, which complicates the recognition of sequence overlap during assembly [38]. How these factors and others (e.g., instrumental aspects of mass spectra acquisition) alter the final output has not been studied extensively. In practical terms, this imposes the need for further validation when using such a dataset for downstream analysis.

Comparison of frequencies and distribution of annotations provides a way to describe the degree of functional specialization of proteins in the ESP relative to the total transcriptome. GO terms enrichment analysis revealed how some of the components of the *H. polygyrus* ESP may be involved in processes associated with the transport and/or uptake of nutrients from the host as well as possible involvement in signalling pathways.

Globin homologues in the ESP were enriched in functional annotation categories related to oxygen and heme binding. Nematode globins are distantly related to those in vertebrates and are known or predicted to play a role in several processes, given their expression in different anatomical patterns and diversity in gene structure and amino acid sequence [39], [40]. Although a more precise understanding of the multiple functions of nematode globins is needed, it can be expected that their role in oxygen transport and supply must be critical in the low oxygen conditions of the host microenvironment, where the adult *H. polygyrus* attaches to and coils around the duodenal villi [41]. In this context, globin functions can vary from transport and delivery to oxygen sink depending on the affinity of oxygen binding. For example, the high oxygen affinity globin from *A. suum* has been proposed to prevent toxic effects of oxygen for this parasite [40], [42]. In addition, parasitic as well as free living nematodes are heme auxotrophs [43], and thus secreted globins may also participate as heme carriers for the supply of this prosthetic group required for many other biological processes.

Another group of enriched functions found in the ESP are related to binding of lipids and retinoids. Proteins associated with these functions are involved in the transport of these hydrophobic molecules as substrates for energy metabolism, membrane biosynthesis, and signalling [44]. Identified proteins in this group include homologues of nematode polyprotein allergens/antigens (NAR), fatty acid and retinol binding (FAR) proteins, and Vtg proteins. NAR and FAR proteins comprise classes of small (∼14 kDa and ∼20 kDa, respectively) lipid binding proteins from nematodes. NAR proteins bind both retinol and fatty acids; they are synthesized as repetitive polypeptides in tandem and are subsequently cleaved into multiple functionally similar proteins [45], [46]. FAR proteins exhibit higher affinity for retinol than for fatty acids [47]. In addition to a role in the acquisition of small lipids from the host or the microbiota, their role as parasite secreted proteins has been proposed to be the sequestration or delivery of signalling lipids to host cells [44]. Their possible role in sequestering vitamin A from the host has been associated with the pathology of parasitic nematode infections. Among these are visual impairment caused by infections with *Onchocerca volvulus* [47] and vitamin A deficiency in patients infected with *A. lumbricoides*, possibly due to malabsorption [48]. Sequestration of vitamin A may also contribute to immunomodulation as it is required for host adaptive immunity and is involved in the differentiation of CD4^+^ T helper (Th) cells and B cells. In particular, vitamin A deficiency leads to impaired intestinal immune responses, including antibody-mediated responses directed by Th2 cells [49], [50].

Vtg proteins form a highly diverse family in the large lipid transfer protein (LLTP) superfamily. In addition to the ESP from *H. polygyrus*, these proteins have also been identified in ESP from other parasitic GI nematodes [51], [52]. In *C. elegans*, Vtgs are implicated in the delivery of nutrients to support embryonic development, hence the enrichment of biological process terms associated with growth regulation. They are secreted from the intestine to the pseudocoelomic space where they transit through the gonadal basal lamina and then through the sheath pores for receptor-mediated oocyte endocytosis [53], [54]. Therefore, it is likely that their presence in ESP from parasitic GI nematodes is the result of egg release. However, the involvement of Vtg-like proteins in modulation of insect host immune responses [55]–[57] suggests a possible additional role in negotiation of the host-parasite interface.

Peptidase activity was another GO function enriched in the ESP protein set. Helminth proteases participate in the establishment, development, and maintenance of infection [58]. In *H. polygyrus*, developmental regulation of ESP-proteases suggest possible roles in exsheathment, invasion of the mucosa, and immune regulation during the larval stages, and feeding and migration during the adult stage [59]. Nothing is known about the substrate specificities of the *H. polygyrus* ESP-proteases. However, by analogy to the proteolytic cascade required for haemoglobin degradation by hookworms [60], several components of which were also identified in *A. caninum* ESP [52], the identified aspartyl, cysteine, and metalloproteinases from *H. polygyrus* are predicted to participate in degradation of host proteins acquired during tissue feeding.

The identification of enzymes involved in nucleotide metabolism suggests a possible role of ESP in modulation of host signalling pathways. Regulation of local levels of extracellular nucleotides could affect the activity of host purinergic receptors, which mediate a variety of cellular responses, including elements of the immune system [61]. Enzymes involved in nucleotide metabolism have previously been identified in ESP from parasitic nematodes [20], [62]–[64]. These include nucleoside diphosphate kinases, nucleosidases, and adenosine deaminases that participate in the formation of activators of purinergic receptors from ATP or UTP, such as AMP, UMP, adenosine, or inosine [61]. In addition, the homologue of ribonucleotide reductases in *H. polygyrus* ESP may contribute precursors for this pathway through the generation of deoxynucleotides from ribonucleotides.

In addition to proteins of interest based on comparison of GO annotation between datasets, homologues of ASP or VAL proteins were also highlighted for their abundance and number of isoforms identified. These proteins are characterized by the presence of the CAP domain (also known as SCP-like domain) and belong to the allergen V5/Tpx-1-related family of proteins, a group of evolutionarily related eukaryotic extracellular proteins whose function remains largely unknown [32], [65], [66]. InterPro terms associated with this domain and families were found to be enriched in the ESP dataset. Members of this family include cysteine-rich sperm proteins (CRISPs), insect venom allergens, and plant pathogenesis family-1 (PR-1) proteins. Reasons to suspect a role for these proteins at the nematode-host interface (including pathogenesis) include the rapid and specific release of *N. americanus* ASP-2 during the transition from larval to parasitic stages as well to their neutrophil chemoattractant activity [67], [68], and the angiogenic effects of several *O. volvulus* ASPs [69].

In addition to proteins highlighted on the basis of enrichment of functional annotation, other relevant proteins in *H. polygyrus* ESP include homologues of glycolytic and metabolic enzymes. Of particular interest are triosephosphate isomerase (TPI), fructose bisphosphate aldolase A (FBPA), and enolase (ENO), which have consistently been reported in nematode ESP, a pattern suggesting that their release cannot be simply due to worm death or damage during culture [32]. While the function of these proteins remains obscure in the context of host-nematode relationships, there is evidence of the association of these enzymes with host cell surface components and their involvement in functions unrelated to glycolysis, including microbial pathogenesis and autoimmune disorders [70]–[73].

Possible immunomodulators also include a homologue of MIF, a parasite protein that mimics a mammalian cytokine, which has been reported in many nematode ESPs. MIFs are usually associated with pro-inflammatory responses. However, in contrast to the mammalian cytokine, nematode MIF acts in a Th2 environment to induce AAMΦs [32], [74], [75]. In addition, the cysteine protease inhibitor (CPI) homologue identified in *H. polygyrus* ESP may modulate immune responses to unrelated antigens by inhibition of antigen processing and presentation by antigen presenting cells or by inhibition of T-cell proliferation, which may contribute to the state of cellular hypo-responsiveness characteristic of chronic parasitic nematode infections [76]–[78]. Also of interest are the previously characterized C-type lectins (CTL) from *H. polygyrus* and galectin homologues identified in the ESP in the present study [79]. Their role as immunomodulators is suggested by the involvement of these carbohydrate-binding proteins in a variety of immune functions [80]–[83] as well as the eosinophil attracting activity that has been reported for a galectin from *Haemonchus contortus* [84].

Finally, the presence of homologues of peroxiredoxin (PRX) and glutathione S-transferase (GST) in *H. polygyrus* ESP suggests a role for enzymes involved in detoxification of reactive oxygen species (ROS) released from the host [85], [86]. Other roles for these enzymes may include the induction of AAMΦs, as shown for a helminth PRX, promotion of Th2 immune responses, and the involvement of GST in heme transport and detoxification [87]–[89].

In conclusion, we employed the next-generation sequencing and proteomic approaches to gain insights into the transcriptome of adult *H. polygyrus* and used the dataset to identify protein components of the ESP. Comparison of functional annotation categories of the total transcriptome, which provides a picture of the total proteome, with those of the ESP subset allowed us to identify functions and associated proteins that may play a role at the host-parasite interface, where many events critical for success of the infection occur. The data presented here contribute to the identification of individual components that may be responsible for the immunomodulatory activity that has been reported for *H. polygyrus* ESP. Moreover, methods and analyses presented here are useful for the study of biochemical and molecular aspects of nematode biology in other species for which sequence information is not available.

## Supporting Information

Table S1 Proteins identified in *H. polygyrus* ESP through search in UniProt database taxon identifier 6231, Nematoda.
(PDF)

Table S2 *H. polygyrus* RNA-seq assembly automated annotation.
(PDF)

Table S3 Proteins identified in ESP from *H. polygyrus* through search on conceptual translation from RNA-seq assembly.
(PDF)

Table S4 Enrichment analysis of GO terms in the ESP dataset when compared to the transcriptome dataset.
(PDF)

Table S5 Enrichment analysis of InterPro domains and families in the ESP dataset versus the transcriptome dataset.
(PDF)
