# Supplementary material for: Proteomic Analysis of Excretory-Secretory Products of Heligmosomoides polygyrus Assessed with Next-Generation Sequencing Transcriptomic Information
Source: PLoS Negl Trop Dis. 2011 Oct 25;5(10):e1370. doi: 10.1371/journal.pntd.0001370 (PMC3201918; doi:10.1371/journal.pntd.0001370)
Supplement: Table S2 — H. polygyrus RNA-seq assembly automated annotation. (PDF) [file pntd.0001370.s002.pdf]

Table S2. *H. polygyrus* RNA-seq assembly automated annotation.									
		BLASTX							
Seq. Name	Seq. Length	#Hits Blastx (Max 20)	Closest Blastx Hit eValue	Accession Number	Similarity	Score	Alignment length	Positives	Blastx Hit description
Locus_1_Transcript_1/1_Conf_1.000	487	0							
Locus_10_Transcript_1/3_Conf_0.714	1415	0							
Locus_10_Transcript_2/3_Conf_0.714	1412	0							
Locus_10_Transcript_3/3_Conf_0.714	1412	0							
Locus_100_Transcript_1/1_Conf_1.000	670	20	1.95E-64	P51535.1	97	135.961	70	68	Myoglobin
Locus_1000_Transcript_1/1_Conf_1.000	1191	20	9.94E-145	XP_002641637.1	93	517.694	308	289	Hypothetical protein CBG09959
Locus_10000_Transcript_1/1_Conf_1.000	296	2	3.67E-04	XP_001893253.1	53	48.1358	89	48	hypothetical protein Bm1_08910
Locus_10001_Transcript_1/1_Conf_1.000	1203	20	2.20E-99	EFO22809.1	70	367.081	374	262	hypothetical protein LOAG_05677
Locus_10002_Transcript_1/1_Conf_1.000	1304	20	1.62E-159	CAB59919.1	83	567	386	324	hypothetical protein
Locus_10003_Transcript_1/1_Conf_1.000	517	20	3.87E-47	XP_002630442.1	73	191.045	156	114	Hypothetical protein CBG11175
Locus_10004_Transcript_1/1_Conf_1.000	2212	0							
Locus_10005_Transcript_1/1_Conf_1.000	226	0							
Locus_10006_Transcript_1/1_Conf_1.000	254	0							
Locus_10007_Transcript_1/1_Conf_1.000	140	17	1.84E-16	AAA69729.1	100	88.9669	43	43	immunoglobulin heavy chain
Locus_10008_Transcript_1/1_Conf_1.000	575	0							
Locus_10009_Transcript_1/1_Conf_1.000	1649	20	1.54E-168	NP_510624.1	76	597.43	509	391	hypothetical protein B0395.3

Locus_1001_Transcript_1/1_Conf_1.000	2115	20	0	XP_001892620.1	83	790.801	592	496	Vinculin family protein
Locus_10010_Transcript_1/1_Conf_1.000	416	0							
Locus_10011_Transcript_1/1_Conf_1.000	508	0							
Locus_10012_Transcript_1/1_Conf_1.000	401	3	4.34E-05	YP_002722239.1	39	51.2174	98	39	hypothetical protein BHWA1_02076
Locus_10013_Transcript_1/1_Conf_1.000	1561	20	1.93E-165	ACY40006.1	83	587.03	444	372	K+/cl- cotransporter protein 2, isoform a
Locus_10014_Transcript_1/1_Conf_1.000	543	20	3.06E-27	EFO17297.1	75	125.176	100	75	abhydrolase domain-containing protein 2
Locus_10015_Transcript_1/1_Conf_1.000	260	0							
Locus_10016_Transcript_1/1_Conf_1.000	2578	20	0	XP_002641770.1	77	798.89	844	650	C. briggsae CBR-EMB-9 protein
Locus_10017_Transcript_1/1_Conf_1.000	562	20	7.35E-75	XP_002631736.1	91	283.493	184	168	C. briggsae CBR-RAS-1 protein
Locus_10018_Transcript_1/1_Conf_1.000	179	0							
Locus_10019_Transcript_1/1_Conf_1.000	427	0							
Locus_1002_Transcript_1/1_Conf_1.000	306	2	4.72E-04	AAO63578.1	47	47.7506	69	33	secreted protein 6 precursor
Locus_10020_Transcript_1/1_Conf_1.000	561	4	3.36E-11	NP_001122450.1	41	72.0182	179	74	hypothetical protein F10G8.8
Locus_10021_Transcript_1/1_Conf_1.000	166	20	1.09E-24	XP_002923590.1	100	116.316	55	55	PREDICTED: DAZ-associated protein 1-like
Locus_10022_Transcript_1/1_Conf_1.000	484	4	9.71E-05	XP_002645151.1	56	50.0618	87	49	C. briggsae CBR-TNT-3 protein

Locus_10023_Transcript_1/1_Conf_1.000	914	20	3.68E-63	ACI49197.1	71	246.128	304	217	hypothetical protein Csp3_JD03.006
Locus_10024_Transcript_1/1_Conf_1.000	148	20	7.00E-11	NP_495256.1	86	70.4774	46	40	hypothetical protein F13H8.2
Locus_10025_Transcript_1/1_Conf_1.000	270	0							
Locus_10026_Transcript_1/1_Conf_1.000	604	20	2.55E-37	EFO24023.1	74	159.073	164	122	hypothetical protein LOAG_04465
Locus_10027_Transcript_1/1_Conf_1.000	139	0							
Locus_10028_Transcript_1/1_Conf_1.000	384	20	3.83E-46	XP_002630180.1	90	187.578	121	109	Hypothetical protein CBG00585
Locus_10029_Transcript_1/1_Conf_1.000	1272	20	1.43E-128	NP_493169.2	76	464.151	379	290	hypothetical protein T27F6.6
Locus_1003_Transcript_1/1_Conf_1.000	173	0							
Locus_10030_Transcript_1/2_Conf_1.000	820	20	1.05E-55	XP_002632396.1	97	221.09	114	111	C. briggsae CBR-UAF-2 protein
Locus_10030_Transcript_2/2_Conf_1.000	814	20	7.05E-97	XP_002632396.1	93	357.836	191	179	C. briggsae CBR-UAF-2 protein

Locus_10031_Transcript_1/1_Conf_1.000	805	5	2.46E-17	NP_741607.1	66	93.5893	145	97	hypothetical protein T07C12.12
Locus_10032_Transcript_1/1_Conf_1.000	867	20	1.39E-24	EFO23381.1	57	117.857	180	103	hypothetical protein LOAG_05102
Locus_10033_Transcript_1/1_Conf_1.000	997	20	3.89E-141	EFO24933.1	93	505.368	294	275	AGC/PKN protein kinase
Locus_10034_Transcript_1/1_Conf_1.000	1208	20	4.99E-59	NP_496704.1	80	233.032	183	147	Regulator of G protein Signaling family member (rgs-4)
Locus_10035_Transcript_1/1_Conf_1.000	512	20	1.61E-26	XP_001895780.1	78	122.479	97	76	transcription elongation factor B polypeptide 1
Locus_10036_Transcript_1/1_Conf_1.000	1041	20	5.84E-119	ACI49224.1	76	431.795	347	266	hypothetical protein Csp3_JD05.009
Locus_10037_Transcript_1/1_Conf_1.000	564	0							
Locus_10038_Transcript_1/1_Conf_1.000	674	0							
Locus_10039_Transcript_1/1_Conf_1.000	384	14	2.34E-11	XP_002827014.1	97	72.0182	73	71	PREDICTED: macrosialin-like isoform 3
Locus_1004_Transcript_1/1_Conf_1.000	855	20	3.92E-48	CBK19457.1	73	196.052	176	130	C. elegans protein K01A6.2e, partially confirmed by transcript evidence
Locus_10040_Transcript_1/2_Conf_1.000	419	20	1.21E-47	AAK62032.1	76	192.586	139	106	metalloprotease 1 precursor
Locus_10040_Transcript_2/2_Conf_1.000	419	20	1.21E-47	AAK62032.1	76	192.586	139	106	metalloprotease 1 precursor
Locus_10041_Transcript_1/1_Conf_1.000	543	20	4.13E-32	NP_492142.1	72	141.354	153	111	hypothetical protein T23G11.4
Locus_10042_Transcript_1/1_Conf_1.000	378	0							
Locus_10043_Transcript_1/1_Conf_1.000	326	0							
Locus_10044_Transcript_1/1_Conf_1.000	385	20	1.23E-36	NP_740947.2	73	155.992	126	92	hypothetical protein Y105E8A.19

Locus_10045_Transcript_1/1_Conf_1.000	1082	20	1.57E-138	AAC47238.1	83	496.893	360	301	non-muscle myosin heavy chain II
Locus_10046_Transcript_1/1_Conf_1.000	809	2	3.35E-06	EFO28415.1	40	56.6102	206	84	hypothetical protein LOAG_00061
Locus_10047_Transcript_1/1_Conf_1.000	456	20	2.94E-22	XP_001901176.1	76	108.227	95	73	Sec63 domain containing protein
Locus_10048_Transcript_1/1_Conf_1.000	1603	20	6.27E-135	XP_002649084.1	72	485.723	441	320	Hypothetical protein CBG22670
Locus_10049_Transcript_1/1_Conf_1.000	950	0							
Locus_1005_Transcript_1/1_Conf_1.000	2715	20	5.29E-58	NP_496514.1	56	231.106	401	228	yeast PAT1(protein associated with topo II) Related family member (patr-1)
Locus_10050_Transcript_1/1_Conf_1.000	527	0							
Locus_10051_Transcript_1/2_Conf_1.000	1023	20	7.32E-74	XP_002641487.1	63	281.952	341	217	Hypothetical protein CBG09778
Locus_10051_Transcript_2/2_Conf_1.000	1023	20	7.32E-74	XP_002641487.1	63	281.952	341	217	Hypothetical protein CBG09778
Locus_10052_Transcript_1/1_Conf_1.000	447	20	5.76E-50	NP_001009108.2	100	200.29	91	91	ribonuclease pancreatic precursor
Locus_10053_Transcript_1/1_Conf_1.000	1360	20	5.34E-60	AAK84590.1	71	236.498	180	129	AC084153_8Hypothetical protein Y22D7AL.16

Locus_10054_Transcript_1/1_Conf_1.000	1183	20	3.37E-36	EFO25544.1	76	157.147	119	91	hypothetical protein LOAG_02944
Locus_10055_Transcript_1/1_Conf_1.000	630	20	1.88E-65	XP_002633222.1	84	252.677	200	169	Hypothetical protein CBG05941
Locus_10056_Transcript_1/1_Conf_1.000	351	10	5.26E-19	P34288.4	58	97.4413	112	66	GTPase-activating protein pac-1
Locus_10057_Transcript_1/1_Conf_1.000	176	0							
Locus_10058_Transcript_1/1_Conf_1.000	236	0							
Locus_10059_Transcript_1/1_Conf_1.000	470	0							
Locus_1006_Transcript_1/3_Conf_0.583	142	0							
Locus_1006_Transcript_3/3_Conf_0.667	200	0							
Locus_10060_Transcript_1/1_Conf_1.000	997	20	1.61E-78	XP_002642022.1	80	297.36	215	174	Hypothetical protein CBG17958
Locus_10061_Transcript_1/1_Conf_1.000	769	20	1.13E-77	EFO22550.1	74	293.893	256	191	TATA binding protein associated factor
Locus_10062_Transcript_1/1_Conf_1.000	1124	20	1.05E-23	XP_001895613.1	54	115.546	183	100	Zinc finger, C2H2 type family protein

Locus_10063_Transcript_1/1_Conf_1.000	980	20	7.43E-105	XP_002638488.1	94	384.8	209	197	C. briggsae CBR-SEC-23 protein
Locus_10064_Transcript_1/1_Conf_1.000	170	20	2.35E-11	EFO21941.1	75	72.0182	52	39	hypothetical protein LOAG_06543
Locus_10065_Transcript_1/1_Conf_1.000	561	5	9.13E-17	EFO26231.1	70	90.5077	80	56	hypothetical protein LOAG_02257
Locus_10066_Transcript_1/1_Conf_1.000	1224	20	8.66E-75	XP_002637804.1	75	285.419	224	170	Hypothetical protein CBG04590
Locus_10067_Transcript_1/1_Conf_1.000	632	20	7.91E-64	NP_502658.1	95	247.284	134	128	hypothetical protein Y45F10D.4
Locus_10068_Transcript_1/1_Conf_1.000	195	0							
Locus_10069_Transcript_1/1_Conf_1.000	533	0							
Locus_1007_Transcript_1/1_Conf_1.000	192	0							
Locus_10070_Transcript_1/1_Conf_1.000	735	0							
Locus_10071_Transcript_1/1_Conf_1.000	910	20	1.44E-59	EFO15252.1	63	234.187	267	169	hypothetical protein LOAG_13260

Locus_10072_Transcript_1/1_Conf_1.000	951	8	7.01E-20	EFO20947.1	50	102.449	293	147	hypothetical protein LOAG_07543
Locus_10073_Transcript_1/1_Conf_1.000	718	7	7.32E-57	NP_492628.1	76	224.557	204	156	hypothetical protein Y106G6A.5
Locus_10074_Transcript_1/1_Conf_1.000	523	0							
Locus_10075_Transcript_1/1_Conf_1.000	285	19	3.35E-13	NP_510156.2	73	78.1814	56	41	hypothetical protein F11C1.4
Locus_10076_Transcript_1/1_Conf_1.000	521	3	1.97E-30	NP_504395.2	60	135.576	178	107	hypothetical protein Y47A7.1
Locus_10077_Transcript_1/1_Conf_1.000	130	0							
Locus_10078_Transcript_1/1_Conf_1.000	690	0							
Locus_10079_Transcript_1/1_Conf_1.000	743	3	1.27E-14	NP_001024476.1	50	84.3445	212	108	hypothetical protein C46H3.2
Locus_1008_Transcript_1/1_Conf_1.000	1080	20	1.98E-149	XP_002641166.1	90	533.102	347	313	C. briggsae CBR-EFT-1 protein

Locus_10080_Transcript_1/1_Conf_1.000	261	20	1.17E-42	BAG36500.1	100	176.022	87	87	unnamed protein product
Locus_10081_Transcript_1/1_Conf_1.000	596	20	1.03E-27	EFO26895.1	59	127.102	187	112	organic solute transporter alpha-like protein
Locus_10082_Transcript_1/1_Conf_1.000	816	20	1.38E-108	NP_510580.1	86	396.741	253	219	hypothetical protein F59D12.1
Locus_10083_Transcript_1/1_Conf_1.000	379	0							
Locus_10084_Transcript_1/2_Conf_1.000	589	20	3.54E-41	NP_503101.1	70	171.785	151	107	hypothetical protein C30H6.8
Locus_10084_Transcript_2/2_Conf_1.000	589	20	3.54E-41	NP_503101.1	70	171.785	151	107	hypothetical protein C30H6.8
Locus_10085_Transcript_1/1_Conf_1.000	133	6	7.54E-10	XP_002639323.1	81	67.0106	43	35	C. briggsae CBR-HUM-7 protein
Locus_10086_Transcript_1/1_Conf_1.000	249	2	1.84E-08	XP_002632297.1	57	62.3882	75	43	C. briggsae CBR-NOL-6 protein
Locus_10087_Transcript_1/1_Conf_1.000	405	2	2.60E-10	XP_001898097.1	47	68.5514	135	64	Asparaginase family protein
Locus_10088_Transcript_1/1_Conf_1.000	884	20	5.34E-64	NP_497461.1	63	248.825	238	151	PDGF/VEGF growth factor related family member (pvf-1)
Locus_10089_Transcript_1/1_Conf_1.000	366	3	1.56E-07	XP_001892279.1	63	59.3066	66	42	GrpE protein homolog, mitochondrial precursor
Locus_1009_Transcript_1/1_Conf_1.000	823	0							
Locus_10090_Transcript_1/2_Conf_1.000	1358	20	7.89E-88	NP_001040656.2	76	328.946	363	276	hypothetical protein F27D4.4

Locus_10090_Transcript_2/2_Conf_1.000	1358	20	7.88E-88	NP_001040656.2	76	328.946	363	276	hypothetical protein F27D4.4
Locus_10091_Transcript_1/1_Conf_1.000	431	20	7.76E-39	NP_001022773.1	85	163.31	122	104	Myc and Mondo-Like family member (mml-1)
Locus_10092_Transcript_1/1_Conf_1.000	508	20	5.34E-54	XP_001900544.1	84	213.772	149	126	Type III restriction enzyme, res subunit family protein
Locus_10093_Transcript_1/1_Conf_1.000	827	0							
Locus_10094_Transcript_1/1_Conf_1.000	804	20	2.88E-42	XP_002632817.1	82	176.407	111	92	C. briggsae CBR-CKA-1 protein
Locus_10095_Transcript_1/3_Conf_0.667	639	20	2.62E-86	NP_499324.1	86	322.013	200	173	4-HydroxyPhenylpyruvate Dioxygenase (HPD) family member (hpd-1)
Locus_10095_Transcript_2/3_Conf_0.667	639	20	9.97E-86	NP_499324.1	86	320.087	200	173	4-HydroxyPhenylpyruvate Dioxygenase (HPD) family member (hpd-1)
Locus_10095_Transcript_3/3_Conf_0.667	639	20	9.97E-86	NP_499324.1	86	320.087	200	173	4-HydroxyPhenylpyruvate Dioxygenase (HPD) family member (hpd-1)
Locus_10096_Transcript_1/1_Conf_1.000	957	2	5.27E-07	EFO20717.1	55	59.6918	145	81	hypothetical protein LOAG_07773

Locus_10097_Transcript_1/1_Conf_1.000	568	0							
Locus_10098_Transcript_1/1_Conf_1.000	712	3	4.12E-12	XP_002639131.1	44	75.8702	219	98	Hypothetical protein CBG14951
Locus_10099_Transcript_1/1_Conf_1.000	318	0							
Locus_101_Transcript_1/1_Conf_1.000	334	0							
Locus_1010_Transcript_1/1_Conf_1.000	901	20	1.01E-49	XP_002639673.1	83	201.445	139	116	Hypothetical protein CBG12391
Locus_10100_Transcript_1/1_Conf_1.000	767	20	3.99E-59	EFO23920.1	67	232.261	252	171	hypothetical protein LOAG_04569
Locus_10101_Transcript_1/1_Conf_1.000	672	16	6.14E-68	XP_002647278.1	72	261.151	224	163	C. briggsae CBR-KETN-1 protein
Locus_10102_Transcript_1/1_Conf_1.000	973	20	4.66E-67	XP_002646453.1	80	259.225	184	149	C. briggsae CBR-SMD-1 protein
Locus_10103_Transcript_1/1_Conf_1.000	330	0							
Locus_10104_Transcript_1/1_Conf_1.000	452	20	2.10E-60	XP_002630896.1	89	234.958	134	120	Hypothetical protein CBG02618
Locus_10105_Transcript_1/1_Conf_1.000	302	13	1.42E-16	NP_502242.2	64	89.3521	92	59	hypothetical protein F38H4.6
Locus_10106_Transcript_1/1_Conf_1.000	290	4	7.68E-10	XP_002639822.1	60	67.0106	87	53	C. briggsae CBR-APH-2 protein
Locus_10107_Transcript_1/1_Conf_1.000	128	0							
Locus_10108_Transcript_1/1_Conf_1.000	946	0							
Locus_10109_Transcript_1/1_Conf_1.000	1428	20	0	XP_002637424.1	80	646.351	471	381	Hypothetical protein CBG19133
Locus_1011_Transcript_1/1_Conf_1.000	1290	5	4.22E-35		48	153.68	406	197	DNA topoisomerase II
Locus_10110_Transcript_1/1_Conf_1.000	455	0							
Locus_10111_Transcript_1/1_Conf_1.000	184	0							

Locus_10112_Transcript_1/2_Conf_1.000	2126	20	1.47E-20	XP_002420141.1	48	106.301	458	221	ER to Golgi vesicle transport protein, putative; intracellular protein transport protein, putative
Locus_10112_Transcript_2/2_Conf_1.000	2099	20	1.45E-20	XP_002420141.1	48	106.301	458	221	ER to Golgi vesicle transport protein, putative; intracellular protein transport protein, putative
Locus_10113_Transcript_1/1_Conf_1.000	250	2	9.14E-08	ADG63133.1	75	60.077	57	43	calcium-dependent apyrase
Locus_10114_Transcript_1/1_Conf_1.000	371	0							
Locus_10115_Transcript_1/1_Conf_1.000	177	2	1.12E-05	EFO13974.1	65	53.1434	58	38	hypothetical protein LOAG_14552
Locus_10116_Transcript_1/1_Conf_1.000	642	6	3.92E-05	EEC79812.1	42	52.373	145	62	hypothetical protein OsI_21251
Locus_10117_Transcript_1/3_Conf_0.714	956	20	3.52E-112	XP_002634865.1	78	409.068	314	247	C. briggsae CBR-PLX-1 protein
Locus_10117_Transcript_2/3_Conf_0.714	956	20	7.10E-113	XP_002634865.1	78	411.379	314	248	C. briggsae CBR-PLX-1 protein
Locus_10117_Transcript_3/3_Conf_0.714	956	20	7.10E-113	XP_002634865.1	78	411.379	314	248	C. briggsae CBR-PLX-1 protein
Locus_10118_Transcript_1/1_Conf_1.000	515	20	3.30E-51	NP_741193.1	91	204.527	122	112	hypothetical protein C16A3.10
Locus_10119_Transcript_1/1_Conf_1.000	716	0							

Locus_1012_Transcript_1/1_Conf_1.000	609	20	5.29E-38	EFO28242.1	88	161.384	107	95	hypothetical protein LOAG_00237
Locus_10120_Transcript_1/1_Conf_1.000	502	20	1.54E-42	EFO18902.1	71	175.637	150	107	glycosyl hydrolase family 85 protein
Locus_10121_Transcript_1/1_Conf_1.000	567	20	2.24E-34	AAC47238.1	77	149.058	163	127	non-muscle myosin heavy chain II
Locus_10122_Transcript_1/1_Conf_1.000	596	20	1.93E-42	EFO22910.1	85	176.022	127	109	hypothetical protein LOAG_05577

Locus_10123_Transcript_1/1_Conf_1.000	1861	20	0	NP_001022671.1	90	808.135	478	433	hypothetical protein K08E3.5
Locus_10124_Transcript_1/1_Conf_1.000	201	0							
Locus_10125_Transcript_1/1_Conf_1.000	136	20	1.51E-18	1WZV	100	95.9005	44	44	CrystalStructure Of Ubch8
Locus_10126_Transcript_1/1_Conf_1.000	867	0							
Locus_10127_Transcript_1/1_Conf_1.000	821	0							
Locus_10128_Transcript_1/1_Conf_1.000	150	19	2.02E-18	XP_002344856.1	91	95.5153	48	44	PREDICTED: similar to hCG2042717
Locus_10129_Transcript_1/1_Conf_1.000	1173	20	4.94E-56	NP_504952.1	75	223.016	258	195	hypothetical protein F19F10.9
Locus_1013_Transcript_1/1_Conf_1.000	1634	20	3.28E-155	CAA84656.2	73	553.132	525	384	C. elegans protein F10F2.2, confirmed by transcript evidence
Locus_10130_Transcript_1/1_Conf_1.000	217	0							
Locus_10131_Transcript_1/1_Conf_1.000	1770	0							
Locus_10132_Transcript_1/1_Conf_1.000	219	0							
Locus_10133_Transcript_1/1_Conf_1.000	515	20	3.63E-74	XP_001897106.1	86	280.796	169	147	hypothetical protein
Locus_10134_Transcript_1/1_Conf_1.000	300	3	2.86E-25	NP_495991.1	72	118.242	100	72	hypothetical protein F42A8.1

Locus_10135_Transcript_1/1_Conf_1.000	553	20	3.09E-78	XP_002640158.1	82	294.664	183	151	C. briggsae CBR-TRE-1 protein
Locus_10136_Transcript_1/1_Conf_1.000	563	20	3.07E-89	NP_491857.1	95	331.257	180	172	RAB family member (rab-10)
Locus_10137_Transcript_1/1_Conf_1.000	1208	20	7.94E-65	ACZ54690.1	59	252.292	357	214	Uncoordinated protein 82, isoform h
Locus_10138_Transcript_1/1_Conf_1.000	445	20	8.91E-59	NP_503352.1	80	229.565	147	118	Flavin-containing MonoOxygenase family member (fmo-5)
Locus_10139_Transcript_1/1_Conf_1.000	620	20	2.03E-24	Q9NA80.3	51	116.316	225	115	Stress-activated map kinase-interacting protein 1 homolog
Locus_1014_Transcript_1/2_Conf_1.000	838	20	3.04E-90	CAR63714.1	88	335.88	226	201	hypothetical protein
Locus_1014_Transcript_2/2_Conf_1.000	869	20	4.25E-90	CAR63714.1	88	335.495	229	203	hypothetical protein
Locus_10140_Transcript_1/1_Conf_1.000	341	20	9.11E-16	EFO21559.1	58	86.6557	113	66	hypothetical protein LOAG_06930
Locus_10141_Transcript_1/3_Conf_0.400	422	0							
Locus_10141_Transcript_2/3_Conf_0.400	233	0							
Locus_10141_Transcript_3/3_Conf_0.400	433	0							
Locus_10142_Transcript_1/1_Conf_1.000	348	0							

Locus_10143_Transcript_1/1_Conf_1.000	598	20	1.58E-92	XP_002834351.1	100	342.428	164	164	PREDICTED: hypothetical protein LOC100438152
Locus_10144_Transcript_1/1_Conf_1.000	256	7	3.58E-07	NP_499886.1	50	58.151	76	38	hypothetical protein R02D3.7
Locus_10145_Transcript_1/1_Conf_1.000	851	20	1.94E-124	A8WVU9.2	92	449.514	274	254	Rho-associated protein kinase let-502
Locus_10146_Transcript_1/1_Conf_1.000	394	0							
Locus_10147_Transcript_1/1_Conf_1.000	294	0							
Locus_10148_Transcript_1/1_Conf_1.000	141	0							

Locus_10149_Transcript_1/1_Conf_1.000	1208	15	3.60E-33	XP_002638550.1	50	147.132	253	129	Hypothetical protein CBG05586
Locus_1015_Transcript_1/3_Conf_0.714	795	0							
Locus_1015_Transcript_2/3_Conf_0.429	535	0							
Locus_1015_Transcript_3/3_Conf_0.714	976	0							
Locus_10150_Transcript_1/1_Conf_1.000	385	0							
Locus_10151_Transcript_1/2_Conf_1.000	619	20	5.34E-33	XP_001900984.1	89	144.821	91	81	HMG box family protein
Locus_10151_Transcript_2/2_Conf_1.000	610	20	5.13E-33	XP_001900984.1	89	144.821	91	81	HMG box family protein
Locus_10152_Transcript_1/2_Conf_1.000	971	20	1.01E-37	EFO16052.1	60	161.77	178	108	hypothetical protein LOAG_12456
Locus_10152_Transcript_2/2_Conf_1.000	911	20	8.21E-39	XP_002633000.1	65	165.236	203	132	C. briggsae CBR-ICL-1 protein
Locus_10153_Transcript_1/2_Conf_1.000	1095	20	3.43E-48	CBB15981.1	74	196.823	169	126	C. elegans protein B0285.1c, partially confirmed by transcript evidence

Locus_10153_Transcript_2/2_Conf_1.000	1199	20	3.91E-48	CBB15981.1	74	196.823	169	126	C. elegans protein B0285.1c, partially confirmed by transcript evidence
Locus_10154_Transcript_1/1_Conf_1.000	752	6	6.13E-89	NP_501400.1	81	331.257	246	200	hypothetical protein F35H10.10
Locus_10155_Transcript_1/1_Conf_1.000	226	18	5.13E-22	NP_001021991.1	75	107.457	74	56	hypothetical protein C26D10.6
Locus_10156_Transcript_1/1_Conf_1.000	254	0							
Locus_10157_Transcript_1/1_Conf_1.000	421	20	1.44E-32	NP_497814.3	64	142.51	154	100	UNCoordinated family member (unc-79)
Locus_10158_Transcript_1/1_Conf_1.000	1123	20	2.83E-45	NP_494847.2	94	187.193	141	133	hypothetical protein F41C3.4
Locus_10159_Transcript_1/1_Conf_1.000	414	0							
Locus_1016_Transcript_1/1_Conf_1.000	706	20	1.75E-23	XP_001896558.1	61	113.62	126	78	Ser/Thr protein phosphatase family protein
Locus_10160_Transcript_1/1_Conf_1.000	164	20	2.07E-15	XP_001899041.1	87	85.5001	54	47	Transcription initiation factor IID, 18kD subunit family protein
Locus_10161_Transcript_1/2_Conf_1.000	360	0							
Locus_10161_Transcript_2/2_Conf_1.000	366	0							
Locus_10162_Transcript_1/1_Conf_1.000	562	0							
Locus_10163_Transcript_1/1_Conf_1.000	159	0							
Locus_10164_Transcript_1/1_Conf_1.000	385	0							
Locus_10165_Transcript_1/1_Conf_1.000	373	20	2.45E-24	NP_491935.1	96	115.161	62	60	G Protein, Gamma subunit family member (gpc-2)
Locus_10166_Transcript_1/1_Conf_1.000	482	20	1.49E-58	CAR63593.1	89	228.794	155	139	putative PQ loop repeat family protein
Locus_10167_Transcript_1/1_Conf_1.000	168	0							

Locus_10168_Transcript_1/1_Conf_1.000	1250	9	1.88E-40	CAA10033.1	49	171.4	430	212	DYS-1 protein
Locus_10169_Transcript_1/1_Conf_1.000	374	4	1.00E-09	CAA91482.3	71	66.6254	59	42	C. elegans protein T04F8.6, partially confirmed by transcript evidence
Locus_1017_Transcript_1/1_Conf_1.000	186	20	1.04E-14	XP_002631360.1	81	83.1889	61	50	Hypothetical protein CBG03195
Locus_10170_Transcript_1/1_Conf_1.000	398	20	1.48E-53	XP_002641446.1	92	212.231	127	118	Hypothetical protein CBG13315
Locus_10171_Transcript_1/1_Conf_1.000	369	0							
Locus_10172_Transcript_1/1_Conf_1.000	359	3	4.15E-08	XP_002634119.1	84	61.2326	57	48	Hypothetical protein CBG01673
Locus_10173_Transcript_1/1_Conf_1.000	700	20	4.63E-101	XP_002630114.1	96	371.318	233	224	C. briggsae CBR-PYR-1 protein
Locus_10174_Transcript_1/1_Conf_1.000	270	20	2.04E-39	EFO25642.1	98	165.236	89	88	hypothetical protein LOAG_02841
Locus_10175_Transcript_1/1_Conf_1.000	1220	20	3.40E-164	XP_001899489.1	81	582.408	411	337	Trehalose 6-phosphate synthase protein 1
Locus_10176_Transcript_1/1_Conf_1.000	226	0							
Locus_10177_Transcript_1/1_Conf_1.000	227	0							
Locus_10178_Transcript_1/1_Conf_1.000	765	20	3.80E-118	XP_002638375.1	86	428.328	254	219	Hypothetical protein CBG18580

Locus_10179_Transcript_1/1_Conf_1.000	541	20	1.26E-41	XP_002642775.1	75	172.94	148	112	C. briggsae CBR-NEX-1 protein
Locus_1018_Transcript_1/2_Conf_1.000	548	0							
Locus_1018_Transcript_2/2_Conf_1.000	599	0							
Locus_10180_Transcript_1/1_Conf_1.000	671	20	3.97E-43	NP_504518.3	69	178.718	202	141	Nuclear Hormone Receptor family member (nhr-142)
Locus_10181_Transcript_1/1_Conf_1.000	354	0							
Locus_10182_Transcript_1/1_Conf_1.000	183	0							
Locus_10183_Transcript_1/1_Conf_1.000	293	0							
Locus_10184_Transcript_1/1_Conf_1.000	276	20	6.99E-32	CBA11613.1	77	140.198	88	68	C. elegans protein F54F3.1c, partially confirmed by transcript evidence
Locus_10185_Transcript_1/1_Conf_1.000	314	5	3.96E-14	XP_002639081.1	67	81.2629	91	61	Hypothetical protein CBG14899
Locus_10186_Transcript_1/1_Conf_1.000	193	0							
Locus_10187_Transcript_1/1_Conf_1.000	491	0							
Locus_10188_Transcript_1/1_Conf_1.000	534	5	5.64E-23	NP_506872.1	72	110.923	102	74	hypothetical protein F28F8.5
Locus_10189_Transcript_1/1_Conf_1.000	670	20	1.55E-63	EFO27261.1	78	246.514	185	146	APB-1 protein
Locus_1019_Transcript_1/2_Conf_1.000	1234	20	4.97E-70	AAK84571.2	76	269.626	190	146	AC006685_2Hypothetical protein T13G4.2
Locus_1019_Transcript_2/2_Conf_1.000	1183	17	1.96E-60	AAK84571.2	82	237.654	151	124	AC006685_2Hypothetical protein T13G4.2
Locus_10190_Transcript_1/1_Conf_1.000	328	0							
Locus_10191_Transcript_1/1_Conf_1.000	591	20	1.11E-50	NP_506089.1	74	203.371	162	120	hypothetical protein R04F11.5
Locus_10192_Transcript_1/1_Conf_1.000	1275	2	1.25E-23	XP_002647382.1	47	115.546	425	201	Hypothetical protein CBG06447
Locus_10193_Transcript_1/1_Conf_1.000	338	20	2.40E-24	NP_001021246.1	88	115.161	71	63	Kinesin-Associated Protein family member (kap-1)
Locus_10194_Transcript_1/1_Conf_1.000	249	0							

Locus_10195_Transcript_1/1_Conf_1.000	1026	20	1.43E-77	XP_002636449.1	65	294.278	301	196	Hypothetical protein CBG23110
Locus_10196_Transcript_1/1_Conf_1.000	779	20	2.51E-48	XP_002630873.1	74	196.438	167	124	Hypothetical protein CBG02590
Locus_10197_Transcript_1/1_Conf_1.000	360	5	2.83E-41	NP_506424.2	84	171.4	119	100	Nose Resistant to Fluoxetine family member (nrf-5)
Locus_10198_Transcript_1/1_Conf_1.000	326	20	1.02E-30	XP_002639478.1	76	136.346	110	84	C. briggsae CBR-CYP-36A1 protein
Locus_10199_Transcript_1/1_Conf_1.000	1546	2	2.87E-12	XP_002638680.1	43	78.1814	314	138	Hypothetical protein CBG11872
Locus_102_Transcript_1/2_Conf_1.000	2142	20	0	XP_002636957.1	95	1261.13	713	683	C. briggsae CBR-EAT-6 protein
Locus_102_Transcript_2/2_Conf_1.000	1125	20	0	NP_506269.1	97	671.774	374	366	EATing: abnormal pharyngeal pumping family member (eat-6)

Locus_1020_Transcript_1/1_Conf_1.000	436	20	1.19E-31	XP_002636877.1	84	139.428	84	71	Hypothetical protein CBG09336
Locus_10200_Transcript_1/2_Conf_1.000	2648	20	0	NP_495537.1	77	899.042	731	568	HAlF transporter (PGP related) family member (haf-2)
Locus_10200_Transcript_2/2_Conf_1.000	635	4	1.34E-10	NP_495537.1	54	70.4774	113	62	HAlF transporter (PGP related) family member (haf-2)
Locus_10201_Transcript_1/1_Conf_1.000	238	0							
Locus_10202_Transcript_1/1_Conf_1.000	402	5	8.37E-25	NP_001022999.1	63	116.701	132	84	hypothetical protein ZK418.2
Locus_10203_Transcript_1/2_Conf_1.000	638	0							
Locus_10203_Transcript_2/2_Conf_1.000	641	0							
Locus_10204_Transcript_1/1_Conf_1.000	510	20	3.92E-65	NP_500247.2	89	250.751	144	129	hypothetical protein Y94H6A.8
Locus_10205_Transcript_1/1_Conf_1.000	362	20	3.59E-36	NP_493616.1	76	154.451	121	92	hypothetical protein F33H2.5
Locus_10206_Transcript_1/1_Conf_1.000	446	20	6.59E-62	XP_002642996.1	89	239.965	148	133	C. briggsae CBR-UNC-45 protein
Locus_10207_Transcript_1/2_Conf_1.000	360	9	3.47E-31	XP_002644619.1	91	137.887	90	82	C. briggsae CBR-LFI-1 protein
Locus_10207_Transcript_2/2_Conf_1.000	473	14	1.42E-32	NP_001024981.1	55	142.51	216	119	Lin-5 (Five) Interacting protein family member (lfi-1)
Locus_10208_Transcript_1/1_Conf_1.000	1004	20	3.23E-18	AAM98045.2	57	97.0561	106	61	Hypothetical protein C34H4.5
Locus_10209_Transcript_1/1_Conf_1.000	1087	20	8.04E-58	NP_491700.2	57	228.794	384	219	hypothetical protein F33D11.9

Locus_1021_Transcript_1/1_Conf_1.000	1285	20	1.70E-153	XP_002631561.1	80	546.969	364	293	C. briggsae CBR-GLN-2 protein
Locus_10210_Transcript_1/1_Conf_1.000	194	2	3.81E-09	EFO17919.1	73	64.6994	64	47	hypothetical protein LOAG_10578
Locus_10211_Transcript_1/3_Conf_0.667	576	4	2.00E-09	XP_002638722.1	71	66.2402	66	47	C. briggsae CBR-RLBP-1 protein
Locus_10211_Transcript_2/3_Conf_0.667	731	4	3.46E-09	XP_002638722.1	71	66.2402	66	47	C. briggsae CBR-RLBP-1 protein
Locus_10211_Transcript_3/3_Conf_0.667	731	4	3.46E-09	XP_002638722.1	71	66.2402	66	47	C. briggsae CBR-RLBP-1 protein
Locus_10212_Transcript_1/1_Conf_1.000	678	20	1.65E-68	XP_002763858.1	100	263.077	128	128	PREDICTED: ADP/ATP translocase 3-like, partial
Locus_10213_Transcript_1/1_Conf_1.000	256	0							
Locus_10214_Transcript_1/1_Conf_1.000	412	20	3.70E-33	NP_491412.1	92	144.436	85	79	Acyl-Coenzyme A Binding Protein family member (acbp-1)
Locus_10215_Transcript_1/1_Conf_1.000	400	20	7.83E-39	XP_002630251.1	78	163.31	123	96	Hypothetical protein CBG00670
Locus_10216_Transcript_1/2_Conf_1.000	1174	20	2.46E-47	NP_504203.1	56	194.126	296	168	hypothetical protein F32D1.9
Locus_10216_Transcript_2/2_Conf_1.000	1174	20	2.46E-47	NP_504203.1	56	194.126	296	168	hypothetical protein F32D1.9
Locus_10217_Transcript_1/1_Conf_1.000	262	0							
Locus_10218_Transcript_1/1_Conf_1.000	273	0							
Locus_10219_Transcript_1/1_Conf_1.000	283	20	5.18E-14	NP_001024551.1	60	80.8777	93	56	Nuclear Hormone Receptor family member (nhr-25)
Locus_1022_Transcript_1/1_Conf_1.000	499	0							
Locus_10220_Transcript_1/1_Conf_1.000	446	0							
Locus_10221_Transcript_1/1_Conf_1.000	766	0							
Locus_10222_Transcript_1/1_Conf_1.000	1189	20	1.65E-115	ACS36091.1	78	420.624	343	268	cysteine proteinase
Locus_10223_Transcript_1/1_Conf_1.000	389	0							
Locus_10224_Transcript_1/1_Conf_1.000	407	20	3.40E-10	EFO23403.1	56	68.1662	101	57	hypothetical protein LOAG_05077
Locus_10225_Transcript_1/1_Conf_1.000	144	20	1.67E-12	XP_002642817.1	79	75.8702	48	38	C. briggsae CBR-NAS-1 protein
Locus_10226_Transcript_1/1_Conf_1.000	1967	20	8.40E-124	XP_001901370.1	70	449.129	459	324	Lateral signaling target protein 2

Locus_10227_Transcript_1/1_Conf_1.000	270	20	1.12E-45	NP_001021871.1	100	186.037	90	90	UNCoordinated family member (unc-13)
Locus_10228_Transcript_1/1_Conf_1.000	307	18	5.21E-11	XP_002634644.1	60	70.8626	98	59	Hypothetical protein CBG03497
Locus_10229_Transcript_1/1_Conf_1.000	204	20	1.38E-11	NP_501554.2	72	72.7886	68	49	hypothetical protein D1046.5
Locus_1023_Transcript_1/1_Conf_1.000	844	20	2.37E-74	NP_508168.1	80	283.108	192	154	hypothetical protein F56F10.3
Locus_10230_Transcript_1/1_Conf_1.000	159	0							
Locus_10231_Transcript_1/1_Conf_1.000	325	0							
Locus_10232_Transcript_1/1_Conf_1.000	346	20	2.54E-21	NP_492549.2	62	105.145	117	73	hypothetical protein K07A1.9
Locus_10233_Transcript_1/1_Conf_1.000	532	20	7.38E-31	XP_002631098.1	63	137.117	158	100	Hypothetical protein CBG02872
Locus_10234_Transcript_1/1_Conf_1.000	149	0							
Locus_10235_Transcript_1/1_Conf_1.000	658	0							
Locus_10236_Transcript_1/1_Conf_1.000	353	20	7.83E-31	XP_001896501.1	78	136.732	99	78	Hypothetical subtilase-type proteinase F21H12.6 in chromosome II

Locus_10237_Transcript_1/1_Conf_1.000	176	20	1.46E-21	XP_002640668.1	94	105.916	58	55	C. briggsae CBR-UNC-54 protein
Locus_10238_Transcript_1/1_Conf_1.000	588	19	7.59E-52	XP_002640213.1	74	207.223	195	145	Hypothetical protein CBG12724
Locus_10239_Transcript_1/1_Conf_1.000	428	20	8.12E-12	AAO63577.1	53	73.559	145	77	secreted protein 5 precursor
Locus_1024_Transcript_1/1_Conf_1.000	1081	20	1.14E-88	XP_002633118.1	84	331.257	206	175	C. briggsae CBR-CAP-1 protein

Locus_10240_Transcript_1/1_Conf_1.000	926	20	1.80E-73	XP_002647138.1	91	280.411	164	150	Hypothetical protein CBG16436
Locus_10241_Transcript_1/1_Conf_1.000	289	0							
Locus_10242_Transcript_1/1_Conf_1.000	668	0							
Locus_10243_Transcript_1/1_Conf_1.000	283	20	3.12E-35	NP_509361.1	89	151.369	93	83	hypothetical protein K07E3.4
Locus_10244_Transcript_1/1_Conf_1.000	407	0							
Locus_10245_Transcript_1/1_Conf_1.000	972	20	1.41E-47	NP_493972.3	67	194.512	207	140	NucleOLar protein family member (nol-6)
Locus_10246_Transcript_1/1_Conf_1.000	768	6	3.68E-20	XP_002647225.1	47	102.834	255	122	Hypothetical protein CBG23815
Locus_10247_Transcript_1/1_Conf_1.000	592	20	4.23E-50	NP_497138.1	77	201.445	187	145	hypothetical protein F54C4.1
Locus_10248_Transcript_1/1_Conf_1.000	221	0							
Locus_10249_Transcript_1/1_Conf_1.000	538	20	3.27E-18	XP_001892089.1	57	95.1301	127	73	S-antigen protein precursor
Locus_1025_Transcript_1/1_Conf_1.000	796	3	1.42E-102	NP_501020.2	83	376.711	265	222	hypothetical protein C01B10.3
Locus_10250_Transcript_1/1_Conf_1.000	901	20	1.11E-88	NP_501399.1	66	330.872	321	215	Vacuolar H ATPase family member (vha-5)
Locus_10251_Transcript_1/1_Conf_1.000	312	20	1.55E-34	NP_501176.1	81	149.058	105	86	hypothetical protein T12B3.3
Locus_10252_Transcript_1/1_Conf_1.000	673	20	3.40E-34	XP_640893.1	57	149.058	221	126	hypothetical protein DDB_G0281219
Locus_10253_Transcript_1/1_Conf_1.000	307	0							
Locus_10254_Transcript_1/1_Conf_1.000	571	0							

Locus_10255_Transcript_1/1_Conf_1.000	213	20	2.74E-28	XP_002640693.1	94	128.257	69	65	Hypothetical protein CBG19758
Locus_10256_Transcript_1/1_Conf_1.000	493	0							
Locus_10257_Transcript_1/1_Conf_1.000	926	20	1.13E-75	XP_002641397.1	79	287.73	239	190	Hypothetical protein CBG13259
Locus_10258_Transcript_1/1_Conf_1.000	274	2	2.68E-07	ACZ64269.1	68	58.5362	83	57	astacin metalloprotease b
Locus_10259_Transcript_1/1_Conf_1.000	1718	20	1.54E-126	XP_002631305.1	62	457.988	576	361	C. briggsae CBR-DNA-2 protein
Locus_1026_Transcript_1/1_Conf_1.000	256	20	1.35E-38	BAC01681.1	96	162.54	85	82	immunoglobulin kappa light chain VLJ region
Locus_10260_Transcript_1/2_Conf_1.000	672	20	1.59E-15	XP_002637557.1	52	87.0409	185	97	Hypothetical protein CBG19289
Locus_10260_Transcript_2/2_Conf_1.000	284	0							
Locus_10261_Transcript_1/1_Conf_1.000	538	20	2.81E-70	AAG44663.1	100	268.085	131	131	AF260332_1DC33
Locus_10262_Transcript_1/1_Conf_1.000	616	20	2.88E-39	EFO21094.1	66	165.622	176	117	cyclophilin
Locus_10263_Transcript_1/1_Conf_1.000	1100	20	3.72E-18	NP_495696.2	61	97.0561	109	67	hypothetical protein K02C4.3
Locus_10264_Transcript_1/1_Conf_1.000	511	20	2.62E-77	XP_002630855.1	90	291.197	169	153	Hypothetical protein CBG02570

Locus_10265_Transcript_1/1_Conf_1.000	276	2	7.76E-15	NP_740947.2	65	83.5741	90	59	hypothetical protein Y105E8A.19
Locus_10266_Transcript_1/1_Conf_1.000	347	20	3.65E-44	NP_001022207.1	96	181.03	89	86	hypothetical protein F56D1.3
Locus_10267_Transcript_1/1_Conf_1.000	739	20	2.68E-57	NP_497019.1	67	226.098	250	168	hypothetical protein F57C2.5
Locus_10268_Transcript_1/1_Conf_1.000	198	0							
Locus_10269_Transcript_1/1_Conf_1.000	376	0							
Locus_1027_Transcript_1/1_Conf_1.000	488	2	6.05E-07	XP_002637168.1	65	57.3806	46	30	Hypothetical protein CBG09680
Locus_10270_Transcript_1/1_Conf_1.000	907	20	9.88E-69	XP_001898880.1	67	264.618	302	204	RhoGAP domain containing protein
Locus_10271_Transcript_1/1_Conf_1.000	197	0							
Locus_10272_Transcript_1/1_Conf_1.000	586	20	5.31E-37	XP_002646234.1	63	157.918	187	118	Hypothetical protein CBG11927
Locus_10273_Transcript_1/1_Conf_1.000	872	11	2.02E-23	AAM12418.1	53	114.005	226	121	spliced leader 30 kDa protein
Locus_10274_Transcript_1/1_Conf_1.000	329	0							
Locus_10275_Transcript_1/1_Conf_1.000	153	4	9.06E-19	EFO26119.1	97	96.6709	49	48	myotactin form A
Locus_10276_Transcript_1/1_Conf_1.000	424	11	1.29E-33	XP_001900740.1	73	145.976	131	96	BTB/POZ domain containing protein
Locus_10277_Transcript_1/1_Conf_1.000	542	20	2.01E-31	EFO18735.1	67	139.043	137	92	amidase
Locus_10278_Transcript_1/1_Conf_1.000	1362	20	1.52E-155	XP_002631669.1	82	553.903	455	374	C. briggsae CBR-MOG-4 protein
Locus_10279_Transcript_1/1_Conf_1.000	795	0							
Locus_1028_Transcript_1/1_Conf_1.000	779	20	2.12E-47	XP_002633078.1	66	193.356	224	149	Hypothetical protein CBG05761
Locus_10280_Transcript_1/1_Conf_1.000	535	0							
Locus_10281_Transcript_1/1_Conf_1.000	997	20	1.23E-86	EFO23979.1	70	324.324	334	234	metallopeptidase family M24 containing protein
Locus_10282_Transcript_1/1_Conf_1.000	435	4	2.50E-05	XP_843162.1	53	51.9878	91	49	proteophosphoglycan ppg4

Locus_10283_Transcript_1/1_Conf_1.000	508	20	1.52E-16	NP_491203.1	50	89.3521	171	87	human HUS1 related family member (hus-1)
Locus_10284_Transcript_1/1_Conf_1.000	1041	20	9.13E-72	XP_002633355.1	62	275.018	329	206	Hypothetical protein CBG06099
Locus_10285_Transcript_1/1_Conf_1.000	240	0							
Locus_10286_Transcript_1/1_Conf_1.000	327	20	4.56E-39	AAK31527.3	84	164.081	110	93	Neuronal symmetry protein 1, partially confirmed by transcript evidence
Locus_10287_Transcript_1/1_Conf_1.000	462	20	1.98E-74	XP_002643587.1	96	281.567	154	148	Hypothetical protein CBG16309
Locus_10288_Transcript_1/1_Conf_1.000	303	2	2.43E-08	NP_507813.1	58	62.003	73	43	hypothetical protein Y43F8C.13
Locus_10289_Transcript_1/1_Conf_1.000	215	0							
Locus_1029_Transcript_1/1_Conf_1.000	1027	20	2.13E-97	ACO15780.1	78	360.147	286	224	Lethal protein 767, isoform b, confirmed by transcript evidence
Locus_10290_Transcript_1/1_Conf_1.000	299	0							
Locus_10291_Transcript_1/1_Conf_1.000	141	0							
Locus_10292_Transcript_1/1_Conf_1.000	943	20	4.02E-76	XP_002644619.1	84	289.271	275	231	C. briggsae CBR-LFI-1 protein
Locus_10293_Transcript_1/2_Conf_1.000	2047	20	0	NP_001023608.1	88	915.99	564	498	CCR (yeast CCR4/NOT complex component) homolog family member (ccr-4)

Locus_10293_Transcript_2/2_Conf_1.000	2030	20	0	NP_001023608.1	88	915.99	564	498	CCR (yeast CCR4/NOT complex component) homolog family member (ccr-4)
Locus_10294_Transcript_1/1_Conf_1.000	364	14	3.26E-05	XP_002076888.1	59	51.6026	92	55	GD24585
Locus_10295_Transcript_1/1_Conf_1.000	806	20	7.06E-57	NP_507527.2	75	224.942	193	146	hypothetical protein Y59A8B.8
Locus_10296_Transcript_1/1_Conf_1.000	360	20	3.68E-57	NP_492775.2	86	224.172	119	103	LAMinin related. See also lmb- family member (lam-3)
Locus_10297_Transcript_1/1_Conf_1.000	198	0							
Locus_10298_Transcript_1/1_Conf_1.000	916	5	3.52E-05	XP_002029777.1	42	53.5286	213	91	GM25090
Locus_10299_Transcript_1/1_Conf_1.000	349	2	2.81E-12	NP_510633.1	69	75.0998	83	58	hypothetical protein ZK1073.2
Locus_103_Transcript_1/1_Conf_1.000	867	0							
Locus_1030_Transcript_1/2_Conf_1.000	1305	20	1.25E-18	XP_001894934.1	60	98.9821	128	77	chromobox protein homolog 3
Locus_1030_Transcript_2/2_Conf_1.000	1305	20	1.25E-18	XP_001894934.1	60	98.9821	128	77	chromobox protein homolog 3
Locus_10300_Transcript_1/2_Conf_1.000	308	0							
Locus_10300_Transcript_2/2_Conf_1.000	308	0							
Locus_10301_Transcript_1/1_Conf_1.000	464	0							
Locus_10302_Transcript_1/1_Conf_1.000	254	2	3.59E-07	NP_491456.2	71	58.151	56	40	hypothetical protein F57C9.6
Locus_10303_Transcript_1/1_Conf_1.000	167	2	2.44E-08	NP_500298.3	83	62.003	36	30	hypothetical protein Y67D8B.4
Locus_10304_Transcript_1/1_Conf_1.000	785	0							

Locus_10305_Transcript_1/1_Conf_1.000	456	0							
Locus_10306_Transcript_1/1_Conf_1.000	1038	0							
Locus_10307_Transcript_1/1_Conf_1.000	130	3	1.63E-04	NP_505377.2	77	49.2914	44	34	hypothetical protein B0222.9
Locus_10308_Transcript_1/2_Conf_1.000	1079	0							
Locus_10308_Transcript_2/2_Conf_1.000	1025	0							
Locus_10309_Transcript_1/1_Conf_1.000	398	4	7.16E-16	NP_501455.1	73	87.0409	71	52	Temporarily Assigned Gene name family member (tag-77)
Locus_1031_Transcript_1/1_Conf_1.000	650	20	1.13E-15	NP_499437.2	70	87.4261	92	65	hypothetical protein Y47D3A.29
Locus_10310_Transcript_1/1_Conf_1.000	1034	20	4.39E-50	EFO24890.1	60	202.986	242	146	RING finger protein 170
Locus_10311_Transcript_1/1_Conf_1.000	620	0							
Locus_10312_Transcript_1/1_Conf_1.000	314	20	6.74E-22	EFO15797.1	70	107.071	98	69	hypothetical protein LOAG_12712
Locus_10313_Transcript_1/1_Conf_1.000	315	20	1.27E-20	CAR63649.1	94	102.834	53	50	hypothetical protein
Locus_10314_Transcript_1/1_Conf_1.000	335	19	1.37E-19	ADI61822.1	64	99.3673	111	72	endonuclease-reverse transcriptase
Locus_10315_Transcript_1/1_Conf_1.000	313	0							
Locus_10316_Transcript_1/1_Conf_1.000	163	3	1.94E-05	XP_002634631.1	64	52.373	53	34	Hypothetical protein CBG18490
Locus_10317_Transcript_1/1_Conf_1.000	1241	0							

Locus_10318_Transcript_1/1_Conf_1.000	438	2	1.23E-04	NP_001023357.2	56	49.6766	73	41	hypothetical protein T04C4.1
Locus_10319_Transcript_1/1_Conf_1.000	281	0							
Locus_1032_Transcript_1/1_Conf_1.000	1008	20	4.63E-73	NP_506393.1	76	279.256	259	197	hypothetical protein T01D3.5
Locus_10320_Transcript_1/1_Conf_1.000	203	20	4.90E-25	XP_002648832.1	95	117.472	67	64	Hypothetical protein CBG16947
Locus_10321_Transcript_1/1_Conf_1.000	566	20	9.18E-57	2ON7	77	223.402	187	145	Structure Of Nagst-1
Locus_10322_Transcript_1/1_Conf_1.000	128	0							
Locus_10323_Transcript_1/1_Conf_1.000	131	20	1.29E-17	XP_002631390.1	97	92.8189	43	42	Hypothetical protein CBG03232
Locus_10324_Transcript_1/1_Conf_1.000	159	0							
Locus_10325_Transcript_1/1_Conf_1.000	1205	20	8.95E-101	NP_001024679.1	71	371.703	337	242	DiacylGlycerol Kinase family member (dgk-2)
Locus_10326_Transcript_1/1_Conf_1.000	862	20	3.09E-109	XP_002633235.1	87	399.053	273	240	Hypothetical protein CBG05956
Locus_10327_Transcript_1/1_Conf_1.000	398	20	1.58E-47	NP_492590.1	89	192.2	114	102	hypothetical protein K02A11.3
Locus_10328_Transcript_1/1_Conf_1.000	256	5	2.32E-06	XP_002645945.1	57	55.4546	84	48	C. briggsae CBR-GOB-1 protein

Locus_10329_Transcript_1/1_Conf_1.000	805	20	1.63E-53	NP_491160.1	75	213.772	179	135	SEParase family member (sep-1)
Locus_1033_Transcript_1/1_Conf_1.000	482	0							
Locus_10330_Transcript_1/1_Conf_1.000	129	0							
Locus_10331_Transcript_1/1_Conf_1.000	591	20	1.62E-25	NP_001024529.1	68	119.783	132	90	SLOwpoke potassium channel family member (slo-2)
Locus_10332_Transcript_1/1_Conf_1.000	304	0							
Locus_10333_Transcript_1/3_Conf_0.714	2944	20	3.56E-116	XP_694117.3	50	424.476	949	483	PREDICTED: KIAA1033-like
Locus_10333_Transcript_2/3_Conf_0.714	2944	20	6.08E-116	XP_694117.3	50	423.705	949	483	PREDICTED: KIAA1033-like
Locus_10333_Transcript_3/3_Conf_0.714	2944	20	6.08E-116	XP_694117.3	50	423.705	949	483	PREDICTED: KIAA1033-like
Locus_10334_Transcript_1/1_Conf_1.000	200	0							
Locus_10335_Transcript_1/1_Conf_1.000	404								
Locus_10336_Transcript_1/1_Conf_1.000	264	20	1.25E-20	XP_002636692.1	79	102.834	73	58	Hypothetical protein CBG23406
Locus_10337_Transcript_1/1_Conf_1.000	438	0							
Locus_10338_Transcript_1/1_Conf_1.000	998	4	7.36E-15	ACI49256.1	38	85.8853	372	145	hypothetical protein Csp3_JD07.006
Locus_10339_Transcript_1/1_Conf_1.000	610	6	2.31E-25	EFO24329.1	60	119.398	145	87	hypothetical protein LOAG_04162
Locus_1034_Transcript_1/1_Conf_1.000	1330	20	4.75E-122	CAA96571.1	66	442.58	427	282	parasite pepsinogen
Locus_10340_Transcript_1/1_Conf_1.000	175	20	1.73E-06	XP_002813545.1	72	55.8398	48	35	PREDICTED: histone-lysine N-methyltransferase SETMAR-like

Locus_10341_Transcript_1/2_Conf_1.000	765	20	1.05E-59	CBL43465.1	63	234.187	242	154	C. elegans protein Y55D9A.1d, confirmed by transcript evidence
Locus_10341_Transcript_2/2_Conf_1.000	765	20	2.76E-60	CBL43465.1	64	236.113	242	155	C. elegans protein Y55D9A.1d, confirmed by transcript evidence
Locus_10342_Transcript_1/1_Conf_1.000	777	3	4.48E-05	XP_787339.1	70	52.7582	54	38	PREDICTED: similar to replication factor C p37 subunit
Locus_10343_Transcript_1/2_Conf_1.000	526	20	3.25E-68	XP_002641367.1	84	261.151	193	163	C. briggsae CBR-GBF-1 protein
Locus_10343_Transcript_2/2_Conf_1.000	511	20	1.22E-66	XP_002641367.1	84	255.758	189	159	C. briggsae CBR-GBF-1 protein
Locus_10344_Transcript_1/1_Conf_1.000	727	20	9.02E-10	XP_001493987.1	57	68.1662	94	54	PREDICTED: similar to C-type lectin domain family 9, member A

Locus_10345_Transcript_1/1_Conf_1.000	459								
Locus_10346_Transcript_1/1_Conf_1.000	232	0							
Locus_10347_Transcript_1/1_Conf_1.000	213	0							
Locus_10348_Transcript_1/1_Conf_1.000	188	0							
Locus_10349_Transcript_1/1_Conf_1.000	1012	4	1.09E-05	CAP09614.1	39	55.4546	297	116	novel protein containing multiple sushi domains (SCR repeat)
Locus_1035_Transcript_1/4_Conf_0.400	669	20	4.49E-79	NP_502255.1	81	298.13	203	165	FATty acid desaturase family member (fat-6)
Locus_1035_Transcript_2/4_Conf_0.400	669	20	2.91E-78	NP_502255.1	81	295.434	201	164	FATty acid desaturase family member (fat-6)
Locus_1035_Transcript_3/4_Conf_0.400	1246	20	4.62E-140	NP_502255.1	81	502.286	339	277	FATty acid desaturase family member (fat-6)
Locus_1035_Transcript_4/4_Conf_0.400	1204	20	4.39E-140	NP_502255.1	81	502.286	339	277	FATty acid desaturase family member (fat-6)
Locus_10350_Transcript_1/1_Conf_1.000	2138	20	6.01E-147	NP_501725.2	61	526.168	683	421	hypothetical protein ZK1251.9
Locus_10351_Transcript_1/1_Conf_1.000	287	20	9.36E-32	NP_496465.1	86	139.813	92	80	hypothetical protein F54D5.12
Locus_10352_Transcript_1/1_Conf_1.000	334	0							
Locus_10353_Transcript_1/1_Conf_1.000	366	20	6.47E-46	XP_002640448.1	88	186.808	117	104	Hypothetical protein CBG08504
Locus_10354_Transcript_1/1_Conf_1.000	604	20	1.04E-22	XP_001902019.1	64	110.538	96	62	PRPF39 protein

Locus_10355_Transcript_1/1_Conf_1.000	153	20	4.34E-21	NP_497235.2	98	104.375	50	49	G-protein-coupled Receptor Kinase family member (grk-2)
Locus_10356_Transcript_1/1_Conf_1.000	137	0							
Locus_10357_Transcript_1/1_Conf_1.000	247	0							
Locus_10358_Transcript_1/1_Conf_1.000	301	20	3.86E-30	O61570.1	100	134.42	70	70	60S ribosomal protein L38
Locus_10359_Transcript_1/1_Conf_1.000	581	20	2.98E-77	NP_492474.2	82	291.582	193	160	hypothetical protein F14B4.1
Locus_1036_Transcript_1/1_Conf_1.000	1476	20	1.17E-39	XP_002646159.1	47	169.088	510	241	Hypothetical protein CBG08044
Locus_10360_Transcript_1/1_Conf_1.000	196	0							
Locus_10361_Transcript_1/1_Conf_1.000	1040	20	6.95E-72	NP_001129806.1	69	275.404	266	184	hypothetical protein C08H9.16
Locus_10362_Transcript_1/1_Conf_1.000	737	20	4.68E-62	XP_002639998.1	87	241.891	177	154	Hypothetical protein CBG10828
Locus_10363_Transcript_1/1_Conf_1.000	993	20	1.54E-89	EFO18920.1	78	333.954	283	222	hypothetical protein LOAG_09572
Locus_10364_Transcript_1/1_Conf_1.000	1147	20	9.17E-100	XP_002631224.1	67	368.237	385	260	Hypothetical protein CBG03022
Locus_10365_Transcript_1/1_Conf_1.000	196	0							

Locus_10366_Transcript_1/1_Conf_1.000	1067	20	1.32E-119	EAX03629.1	100	398.282	189	189	major histocompatibility complex, class II, DR alpha, isoform CRA_a
Locus_10367_Transcript_1/1_Conf_1.000	360	0							
Locus_10368_Transcript_1/3_Conf_0.333	518	3	4.61E-16	XP_001894994.1	53	87.8113	123	66	hypothetical protein Bm1_17685
Locus_10368_Transcript_2/3_Conf_0.333	857	2	7.46E-15	EFO17899.1	54	85.5001	182	100	hypothetical protein LOAG_10600
Locus_10368_Transcript_3/3_Conf_0.667	1374	3	1.82E-39	EFO17899.1	51	168.318	390	200	hypothetical protein LOAG_10600
Locus_10369_Transcript_1/1_Conf_1.000	333	20	2.42E-08	XP_002076646.1	66	62.003	71	47	GD15089
Locus_1037_Transcript_1/1_Conf_1.000	1779	20	1.87E-42	EFO25902.1	47	178.718	312	147	hypothetical protein LOAG_02587
Locus_10370_Transcript_1/1_Conf_1.000	509	20	6.55E-44	XP_002640526.1	72	180.259	170	123	Hypothetical protein CBG18688
Locus_10371_Transcript_1/1_Conf_1.000	261	2	2.63E-10	NP_741595.1	80	68.5514	36	29	hypothetical protein K06A4.7
Locus_10372_Transcript_1/1_Conf_1.000	288	20	4.64E-23	XP_002645469.1	77	110.923	93	72	C. briggsae CBR-CDD-1 protein
Locus_10373_Transcript_1/2_Conf_1.000	1858	20	2.07E-116	NP_499770.1	66	424.476	439	293	hypothetical protein F45G2.3
Locus_10373_Transcript_2/2_Conf_1.000	1786	20	5.73E-116	NP_499770.1	63	422.935	471	301	hypothetical protein F45G2.3
Locus_10374_Transcript_1/1_Conf_1.000	337	0							
Locus_10375_Transcript_1/1_Conf_1.000	848	20	5.54E-47	AAC46544.4	56	192.2	278	158	Hypothetical protein F48E8.4
Locus_10376_Transcript_1/1_Conf_1.000	476	20	8.04E-20	EFO24692.1	63	100.138	117	74	protein-tyrosine phosphatase
Locus_10377_Transcript_1/3_Conf_0.667	1121	20	1.97E-123	NP_001041037.1	83	446.817	268	223	GLYcosylation related family member (gly-10)

Locus_10377_Transcript_2/3_Conf_0.667	1097	20	2.66E-117	NP_001041037.1	82	426.402	260	215	GLYcosylation related family member (gly-10)
Locus_10377_Transcript_3/3_Conf_0.667	1121	20	1.51E-123	NP_001041037.1	83	447.203	268	223	GLYcosylation related family member (gly-10)
Locus_10378_Transcript_1/1_Conf_1.000	340	0							
Locus_10379_Transcript_1/2_Conf_1.000	404	20	9.80E-58	XP_002642327.1	95	226.098	120	114	C. briggsae CBR-TAT-3 protein
Locus_10379_Transcript_2/2_Conf_1.000	416	20	2.99E-67	XP_002642327.1	94	257.684	137	129	C. briggsae CBR-TAT-3 protein
Locus_1038_Transcript_1/2_Conf_1.000	511	2	3.85E-12	NP_505787.1	65	74.7146	98	64	hypothetical protein F58B4.4
Locus_1038_Transcript_2/2_Conf_1.000	523	2	4.22E-12	NP_505787.1	65	74.7146	98	64	hypothetical protein F58B4.4
Locus_10380_Transcript_1/1_Conf_1.000	429	20	5.03E-38	XP_002640436.1	70	160.614	140	99	Hypothetical protein CBG08488
Locus_10381_Transcript_1/2_Conf_1.000	260	0							
Locus_10381_Transcript_2/2_Conf_1.000	455	0							
Locus_10382_Transcript_1/1_Conf_1.000	526	20	8.34E-40	NP_872133.1	69	166.777	176	122	hypothetical protein F32D8.13
Locus_10383_Transcript_1/1_Conf_1.000	1145	20	8.89E-79	NP_001023510.1	62	298.516	325	202	hypothetical protein Y55F3AM.6
Locus_10384_Transcript_1/1_Conf_1.000	939	4	2.30E-23	XP_002639960.1	52	114.005	224	118	Hypothetical protein CBG10780
Locus_10385_Transcript_1/1_Conf_1.000	192	0							
Locus_10386_Transcript_1/2_Conf_1.000	676	6	2.08E-47	NP_496952.1	65	192.971	227	148	hypothetical protein K09E4.1

Locus_10386_Transcript_2/2_Conf_1.000	676	6	2.08E-47	NP_496952.1	65	192.971	227	148	hypothetical protein K09E4.1
Locus_10387_Transcript_1/1_Conf_1.000	720	20	1.18E-54	XP_002112403.1	70	217.238	188	133	hypothetical protein TRIADDRAFT_25844
Locus_10388_Transcript_1/1_Conf_1.000	935	20	3.03E-68	NP_497589.3	66	263.077	252	167	hypothetical protein Y71H2B.5
Locus_10389_Transcript_1/1_Conf_1.000	375	0							
Locus_1039_Transcript_1/1_Conf_1.000	260	0							
Locus_10390_Transcript_1/1_Conf_1.000	331	20	1.20E-39	ACJ65168.1	84	166.007	110	93	Uncoordinated protein 68, isoform b, partially confirmed by transcript evidence
Locus_10391_Transcript_1/1_Conf_1.000	239	0							
Locus_10392_Transcript_1/1_Conf_1.000	475	0							
Locus_10393_Transcript_1/1_Conf_1.000	371	20	7.37E-45	XP_002633716.1	81	183.341	119	97	C. briggsae CBR-GTA-1 protein
Locus_10394_Transcript_1/1_Conf_1.000	383	0							
Locus_10395_Transcript_1/1_Conf_1.000	985	20	6.24E-51	NP_505460.1	75	205.682	178	135	hypothetical protein C50F4.12
Locus_10396_Transcript_1/1_Conf_1.000	726	0							

Locus_10397_Transcript_1/3_Conf_0.714	1981	20	0	NP_001040912.1	78	675.241	508	398	hypothetical protein C08F8.2
Locus_10397_Transcript_2/3_Conf_0.714	1981	20	0	NP_001040912.1	78	675.241	508	398	hypothetical protein C08F8.2
Locus_10397_Transcript_3/3_Conf_0.714	1981	20	0	NP_001040912.1	78	675.241	508	398	hypothetical protein C08F8.2
Locus_10398_Transcript_1/1_Conf_1.000	1271	20	1.19E-66	NP_001021632.1	59	258.455	392	234	RaB GAP related family member (rbg-2)
Locus_10399_Transcript_1/1_Conf_1.000	143	0							
Locus_104_Transcript_1/1_Conf_1.000	885	20	5.71E-82	NP_497701.1	81	308.531	238	195	Conserved Regulator of Innate immunity family member (cri-3)
Locus_1040_Transcript_1/1_Conf_1.000	2567	20	0	XP_002647804.1	78	1044.65	860	678	Hypothetical protein CBG23578
Locus_10400_Transcript_1/1_Conf_1.000	628	2	6.29E-05	XP_002645190.1	45	51.6026	148	68	C. briggsae CBR-PPK-3 protein

Locus_10401_Transcript_1/1_Conf_1.000	1318	20	1.77E-145	NP_493982.1	82	520.39	399	328	hypothetical protein H17B01.1
Locus_10402_Transcript_1/1_Conf_1.000	355	20	5.24E-19	XP_001891914.1	66	97.4413	112	74	ThiF family protein
Locus_10403_Transcript_1/1_Conf_1.000	1042	20	5.60E-146	XP_002638849.1	84	521.546	347	293	Hypothetical protein CBG22061
Locus_10404_Transcript_1/1_Conf_1.000	404	20	1.29E-17	XP_001898675.1	61	92.8189	121	74	MGC82625 protein
Locus_10405_Transcript_1/1_Conf_1.000	598	20	1.18E-63	EFO20819.1	74	246.514	198	148	CK1/TTBKL protein kinase
Locus_10406_Transcript_1/1_Conf_1.000	1276	20	1.46E-72	NP_492469.1	71	278.1	256	183	Anterior PHarynx defective family member (aph-1)
Locus_10407_Transcript_1/1_Conf_1.000	185	20	3.46E-26	XP_001894250.1	88	121.324	61	54	Low-density lipoprotein receptor-related protein
Locus_10408_Transcript_1/1_Conf_1.000	822	20	4.74E-48	XP_001894112.1	68	195.667	235	162	RING finger domain containing protein
Locus_10409_Transcript_1/1_Conf_1.000	844	20	1.35E-53	XP_002642327.1	74	214.157	183	137	C. briggsae CBR-TAT-3 protein
Locus_1041_Transcript_1/2_Conf_1.000	668	20	1.48E-58	NP_496281.1	70	229.95	200	140	hypothetical protein C14A4.14
Locus_1041_Transcript_2/2_Conf_1.000	668	20	3.30E-58	NP_496281.1	69	228.794	200	139	hypothetical protein C14A4.14

Locus_10410_Transcript_1/1_Conf_1.000	354	11	4.31E-05	XP_647112.1	55	51.2174	78	43	N-terminal kinase-like protein
Locus_10411_Transcript_1/1_Conf_1.000	355	0							
Locus_10412_Transcript_1/1_Conf_1.000	533	20	1.70E-27	NP_494840.1	57	125.946	175	100	Temporarily Assigned Gene name family member (tag-151)
Locus_10413_Transcript_1/1_Conf_1.000	282	20	7.69E-34	CBW44372.1	90	146.747	94	85	C. elegans protein F21A10.2e, partially confirmed by transcript evidence
Locus_10414_Transcript_1/1_Conf_1.000	308	0							
Locus_10415_Transcript_1/1_Conf_1.000	975	0							
Locus_10416_Transcript_1/1_Conf_1.000	254	0							
Locus_10417_Transcript_1/1_Conf_1.000	836	20	2.25E-61	XP_002635871.1	66	239.965	271	180	Hypothetical protein CBG01087
Locus_10418_Transcript_1/1_Conf_1.000	275	0							
Locus_10419_Transcript_1/1_Conf_1.000	479	0							
Locus_1042_Transcript_1/1_Conf_1.000	1305	20	8.61E-52	NP_001024476.1	78	209.149	148	116	hypothetical protein C46H3.2
Locus_10420_Transcript_1/1_Conf_1.000	345	20	6.07E-15	AAD51334.1	79	83.9593	54	43	Kunitz type serine protease inhibitor
Locus_10421_Transcript_1/1_Conf_1.000	620	0							
Locus_10422_Transcript_1/1_Conf_1.000	475	0							
Locus_10423_Transcript_1/1_Conf_1.000	631	20	1.15E-46	XP_002645686.1	74	190.274	208	154	C. briggsae CBR-SYN-2 protein
Locus_10424_Transcript_1/1_Conf_1.000	294	0							
Locus_10425_Transcript_1/1_Conf_1.000	579	20	4.04E-66	AAK73885.2	79	254.603	192	153	AF003142_2Hypothetical protein F57C9.1b
Locus_10426_Transcript_1/1_Conf_1.000	613	0							
Locus_10427_Transcript_1/1_Conf_1.000	1274	20	2.53E-117	XP_002633543.1	72	426.787	383	276	Hypothetical protein CBG05410
Locus_10428_Transcript_1/1_Conf_1.000	816	20	4.40E-139	AAA72418.1	96	498.049	269	259	unnamed protein product
Locus_10429_Transcript_1/1_Conf_1.000	868	2	1.60E-04	XP_001897166.1	48	51.2174	125	60	hypothetical protein Bm1_28590
Locus_1043_Transcript_1/1_Conf_1.000	994	20	1.27E-75	XP_001897099.1	67	287.73	333	226	amidase
Locus_10430_Transcript_1/1_Conf_1.000	183	0							

Locus_10431_Transcript_1/1_Conf_1.000	505	0								
Locus_10432_Transcript_1/1_Conf_1.000	401	4	1.44E-08	XP_002634229.1	76	62.7734	55	42	C. briggsae CBR-UNC-26 protein
Locus_10433_Transcript_1/1_Conf_1.000	1434	5	9.23E-26	XP_001902031.1	49	122.865	248	122	hypothetical protein
Locus_10434_Transcript_1/2_Conf_1.000	1040	0								
Locus_10434_Transcript_2/2_Conf_1.000	1040	0								
Locus_10435_Transcript_1/1_Conf_1.000	618	4	2.17E-26	NP_502212.1	54	122.865	200	108	hypothetical protein ZK792.5
Locus_10436_Transcript_1/1_Conf_1.000	809								
Locus_10437_Transcript_1/1_Conf_1.000	287	0								
Locus_10438_Transcript_1/1_Conf_1.000	210	3	2.19E-09	ACI49256.1	73	65.4698	53	39	hypothetical protein Csp3_JD07.006
Locus_10439_Transcript_1/1_Conf_1.000	580	6	1.84E-10	XP_001902168.1	70	69.707	64	45	myotactin form B
Locus_1044_Transcript_1/1_Conf_1.000	684	4	5.28E-46	A8X3A7.2	65	188.348	241	157	Integrin alpha pat-2
Locus_10440_Transcript_1/1_Conf_1.000	391	0								
Locus_10441_Transcript_1/1_Conf_1.000	340	0								
Locus_10442_Transcript_1/4_Conf_0.167	403	13	8.62E-33	ZP_03149866.1	67	126.716	118	80	hypothetical protein G11MC16DRAFT_3625
Locus_10442_Transcript_2/4_Conf_0.167	763	20	1.04E-67	ACR38454.1	78	260.766	199	156	unknown
Locus_10442_Transcript_3/4_Conf_0.500	322	20	2.21E-33	ABO20854.1	92	145.206	78	72	hypothetical protein
Locus_10442_Transcript_4/4_Conf_0.722	757	19	6.26E-33	ABO20854.1	92	145.206	78	72	hypothetical protein
Locus_10443_Transcript_1/1_Conf_1.000	387	20	4.80E-57	EFO27005.1	94	223.787	128	121	intermediate filament protein
Locus_10444_Transcript_1/1_Conf_1.000	264	0								
Locus_10445_Transcript_1/1_Conf_1.000	267	6	1.48E-05	XP_002640597.1	67	52.7582	59	40	C. briggsae CBR-DLK-1 protein
Locus_10446_Transcript_1/1_Conf_1.000	317	0								
Locus_10447_Transcript_1/1_Conf_1.000	246	0								

Locus_10448_Transcript_1/1_Conf_1.000	835	20	1.40E-95	XP_002635350.1	81	353.599	249	204	Hypothetical protein CBG01521
Locus_10449_Transcript_1/1_Conf_1.000	403	0							
Locus_1045_Transcript_1/2_Conf_1.000	1283	20	2.37E-147	EFO18658.1	80	526.554	368	297	F-box domain-containing protein
Locus_1045_Transcript_2/2_Conf_1.000	1176	20	4.41E-121	EFO18658.1	81	439.113	303	247	F-box domain-containing protein
Locus_10450_Transcript_1/1_Conf_1.000	135	0							
Locus_10451_Transcript_1/1_Conf_1.000	401	7	2.36E-35	NP_495958.1	69	151.754	139	97	yeast SEC homolog family member (sec-5)
Locus_10452_Transcript_1/1_Conf_1.000	225	0							
Locus_10453_Transcript_1/1_Conf_1.000	228	0							
Locus_10454_Transcript_1/1_Conf_1.000	716	0							
Locus_10455_Transcript_1/1_Conf_1.000	795	20	1.48E-67	NP_495540.1	77	260.381	187	145	hypothetical protein F43E2.5
Locus_10456_Transcript_1/1_Conf_1.000	190	0							
Locus_10457_Transcript_1/1_Conf_1.000	194	0							
Locus_10458_Transcript_1/1_Conf_1.000	443	0							
Locus_10459_Transcript_1/2_Conf_1.000	849	20	5.89E-89	XP_002640838.1	88	331.643	200	176	C. briggsae CBR-UBC-20 protein
Locus_10459_Transcript_2/2_Conf_1.000	848	20	5.85E-89	XP_002640838.1	88	331.643	200	176	C. briggsae CBR-UBC-20 protein
Locus_1046_Transcript_1/1_Conf_1.000	1631	20	6.30E-82	EFO27811.1	56	309.686	501	281	3'-5' exonuclease

Locus_10460_Transcript_1/1_Conf_1.000	790	20	3.99E-49	NP_741083.1	85	199.134	132	113	ALdehyde deHydrogenase family member (alh-11)
Locus_10461_Transcript_1/1_Conf_1.000	279	20	1.78E-11	XP_002632331.1	67	72.4034	64	43	C. briggsae CBR-ROM-4 protein
Locus_10462_Transcript_1/2_Conf_1.000	798	20	1.40E-65	NP_496560.1	78	253.832	206	161	hypothetical protein Y17G7B.12
Locus_10462_Transcript_2/2_Conf_1.000	798	20	1.40E-65	NP_496560.1	78	253.832	206	161	hypothetical protein Y17G7B.12
Locus_10463_Transcript_1/1_Conf_1.000	608	20	1.06E-22	EFO18313.1	66	110.538	130	87	nuclear DNA-binding protein C1D
Locus_10464_Transcript_1/2_Conf_1.000	424	0							
Locus_10464_Transcript_2/2_Conf_1.000	424	0							
Locus_10465_Transcript_1/1_Conf_1.000	287	0							
Locus_10466_Transcript_1/1_Conf_1.000	366	0							
Locus_10467_Transcript_1/1_Conf_1.000	854	20	1.13E-63	NP_507692.1	63	247.669	282	178	hypothetical protein Y80D3A.9
Locus_10468_Transcript_1/1_Conf_1.000	659	20	1.15E-79	XP_002639222.1	78	300.056	233	184	Hypothetical protein CBG03773
Locus_10469_Transcript_1/1_Conf_1.000	238	20	4.29E-13	ZP_02737110.1	69	77.7962	69	48	Pyridoxal-5'-phosphate-dependent enzyme, beta subunit
Locus_1047_Transcript_1/1_Conf_1.000	2165	20	1.05E-90	EFO26769.1	53	339.347	744	399	hypothetical protein LOAG_01716
Locus_10470_Transcript_1/1_Conf_1.000	394	20	1.03E-46	XP_002630612.1	80	189.504	130	104	Hypothetical protein CBG02276
Locus_10471_Transcript_1/1_Conf_1.000	371	0							
Locus_10472_Transcript_1/1_Conf_1.000	280	2	4.54E-07	XP_002641621.1	64	57.7658	75	48	Hypothetical protein CBG09941

Locus_10473_Transcript_1/1_Conf_1.000	431	5	5.04E-30	XP_002810887.1	68	134.035	116	79	PREDICTED: cofilin-1-like
Locus_10474_Transcript_1/1_Conf_1.000	406	0							
Locus_10475_Transcript_1/1_Conf_1.000	179	0							
Locus_10476_Transcript_1/1_Conf_1.000	1826	20	1.27E-126	NP_499625.2	67	458.373	473	318	hypothetical protein Y49E10.20
Locus_10477_Transcript_1/1_Conf_1.000	254	0							
Locus_10478_Transcript_1/1_Conf_1.000	231	20	1.49E-13	EFO25902.1	68	79.337	72	49	hypothetical protein LOAG_02587
Locus_10479_Transcript_1/1_Conf_1.000	585	5	2.02E-36	CAG28316.1	71	155.992	162	116	spindle assembly abnormal protein 5, SAS-5
Locus_1048_Transcript_1/1_Conf_1.000	301	20	3.88E-14	EFO22751.1	88	81.2629	50	44	hypothetical protein LOAG_05734
Locus_10480_Transcript_1/1_Conf_1.000	408	20	2.11E-36	XP_002642327.1	68	155.221	151	103	C. briggsae CBR-TAT-3 protein
Locus_10481_Transcript_1/1_Conf_1.000	311	0							
Locus_10482_Transcript_1/1_Conf_1.000	344	20	1.88E-40	XP_001892739.1	89	168.703	103	92	Zinc finger, C2H2 type family protein
Locus_10483_Transcript_1/1_Conf_1.000	1178	5	8.58E-08	XP_002632964.1	37	62.7734	266	101	Hypothetical protein CBG21722
Locus_10484_Transcript_1/1_Conf_1.000	913	20	1.12E-11	XP_001899507.1	47	75.0998	242	114	hypothetical protein
Locus_10485_Transcript_1/1_Conf_1.000	487	2	6.61E-46	XP_002634524.1	78	186.808	146	115	Hypothetical protein CBG08318
Locus_10486_Transcript_1/1_Conf_1.000	336	1	9.80E-10	XP_002645528.1	72	66.6254	59	43	C. briggsae CBR-FLP-5 protein
Locus_10487_Transcript_1/1_Conf_1.000	225	0							
Locus_10488_Transcript_1/1_Conf_1.000	196	0							
Locus_10489_Transcript_1/1_Conf_1.000	447	1	3.66E-04	NP_495162.1	55	48.1358	79	44	hypothetical protein ZK1248.15

Locus_1049_Transcript_1/1_Conf_1.000	1318	20	3.34E-136	XP_002640719.1	91	489.574	284	259	C. briggsae CBR-EXC-4 protein
Locus_10490_Transcript_1/1_Conf_1.000	201	0							
Locus_10491_Transcript_1/1_Conf_1.000	368	20	3.23E-21	XP_002802296.1	100	104.76	50	50	PREDICTED: SH3 domain-binding glutamic acid-rich-like protein 3-like
Locus_10492_Transcript_1/1_Conf_1.000	189	0							
Locus_10493_Transcript_1/1_Conf_1.000	1062	20	7.25E-173	NP_498740.2	88	610.912	352	310	CaLPain family member (clp-1)
Locus_10494_Transcript_1/1_Conf_1.000	921	20	1.68E-63	NP_493578.1	92	247.284	139	129	Ubiquitin E2 (conjugating enzyme) variant family member (uev-1)
Locus_10495_Transcript_1/1_Conf_1.000	729	0							
Locus_10496_Transcript_1/1_Conf_1.000	357	0							
Locus_10497_Transcript_1/1_Conf_1.000	973	4	6.79E-34	EFO25502.1	53	149.058	252	134	F/Y-rich family protein
Locus_10498_Transcript_1/1_Conf_1.000	503	0							
Locus_10499_Transcript_1/1_Conf_1.000	767	20	9.76E-82	XP_002633444.1	85	307.375	188	160	Hypothetical protein CBG06212
Locus_105_Transcript_1/1_Conf_1.000	621	8	8.50E-23	NP_500399.2	66	110.923	120	80	hypothetical protein Y37E11AL.2

Locus_1050_Transcript_1/1_Conf_1.000	967	20	4.93E-61	XP_002630909.1	98	239.195	119	117	C. briggsae CBR-LGG-1 protein
Locus_10500_Transcript_1/1_Conf_1.000	279	20	5.67E-50	P00736.2	100	200.29	92	92	Complement C1r subcomponent
Locus_10501_Transcript_1/1_Conf_1.000	215	20	1.78E-19	XP_002639413.1	79	98.9821	68	54	C. briggsae CBR-CPS-6 protein
Locus_10502_Transcript_1/1_Conf_1.000	251	7	3.45E-31		92	137.887	76	70	hypothetical protein F09E10.7
Locus_10503_Transcript_1/1_Conf_1.000	709	20	1.51E-99	NP_496447.1	93	366.311	221	206	hypothetical protein W02B12.9
Locus_10504_Transcript_1/1_Conf_1.000	291	7	1.03E-14	NP_001041154.2	71	83.1889	77	55	hypothetical protein R31.2
Locus_10505_Transcript_1/1_Conf_1.000	789	20	6.41E-31	NP_498099.1	54	138.658	185	101	Caffeine Induced Death (S. pombe Cid) homolog family member (cid-1)
Locus_10506_Transcript_1/1_Conf_1.000	337	0							
Locus_10507_Transcript_1/1_Conf_1.000	549	0							

Locus_10508_Transcript_1/1_Conf_1.000	1312	20	1.22E-130	XP_002636654.1	82	471.085	357	294	C. briggsae CBR-CLH-6 protein
Locus_10509_Transcript_1/1_Conf_1.000	1024	20	6.82E-88	XP_001899663.1	68	328.561	335	231	Pyridine nucleotide-disulphide oxidoreductase family protein
Locus_1051_Transcript_1/1_Conf_1.000	258	20	1.39E-43	XP_002753304.1	100	179.104	85	85	PREDICTED: 60S ribosomal protein L4-like
Locus_10510_Transcript_1/1_Conf_1.000	145	0							
Locus_10511_Transcript_1/4_Conf_0.667	589	0							
Locus_10511_Transcript_2/4_Conf_0.667	604	0							
Locus_10511_Transcript_3/4_Conf_0.556	422	0							
Locus_10511_Transcript_4/4_Conf_0.667	605	0							
Locus_10512_Transcript_1/1_Conf_1.000	511	0							
Locus_10513_Transcript_1/2_Conf_1.000	756	20	2.87E-70	XP_001894547.1	73	269.24	234	171	Cyclin C
Locus_10513_Transcript_2/2_Conf_1.000	756	20	2.87E-70	XP_001894547.1	73	269.24	234	171	Cyclin C
Locus_10514_Transcript_1/1_Conf_1.000	444	0							
Locus_10515_Transcript_1/1_Conf_1.000	510	20	9.45E-27	AAC24231.1	64	123.25	135	87	cytoplasmic signalling transducer
Locus_10516_Transcript_1/1_Conf_1.000	433	4	5.01E-38	XP_002636981.1	83	160.614	124	103	C. briggsae CBR-LIN-33 protein
Locus_10517_Transcript_1/1_Conf_1.000	447	20	4.03E-19	XP_002635349.1	57	97.8265	140	81	Hypothetical protein CBG01520
Locus_10518_Transcript_1/1_Conf_1.000	1026	20	2.18E-126	XP_002639452.1	86	456.447	312	271	Hypothetical protein CBG04047
Locus_10519_Transcript_1/3_Conf_0.500	407	20	1.13E-55	NP_498300.2	97	208.764	96	94	MLP/CRP family (Muscle LIM Protein/Cysteine-rich Protein) family member (mlp-1)

Locus_10519_Transcript_2/3_Conf_0.500	495	20	4.98E-54	XP_002641170.1	97	213.772	97	95	C. briggsae CBR-MLP-1 protein
Locus_10519_Transcript_3/3_Conf_0.667	489	20	1.56E-63	NP_498300.2	98	245.358	113	111	MLP/CRP family (Muscle LIM Protein/Cysteine-rich Protein) family member (mlp-1)
Locus_1052_Transcript_1/1_Conf_1.000	715	20	3.95E-79	XP_002646318.1	91	298.516	193	176	C. briggsae CBR-RAB-8 protein
Locus_10520_Transcript_1/1_Conf_1.000	536	20	2.22E-11	XP_001942770.1	69	72.4034	85	59	PREDICTED: similar to CG32795 CG32795-PB
Locus_10521_Transcript_1/1_Conf_1.000	383	0							
Locus_10522_Transcript_1/1_Conf_1.000	523	0							
Locus_10523_Transcript_1/1_Conf_1.000	366	0							
Locus_10524_Transcript_1/1_Conf_1.000	387	0							
Locus_10525_Transcript_1/1_Conf_1.000	520	20	2.26E-18	XP_002432666.1	79	95.5153	67	53	5'-nucleotidase domain-containing protein, putative
Locus_10526_Transcript_1/1_Conf_1.000	579	20	2.08E-46	EFO16762.1	72	189.119	152	110	zinc knuckle family protein
Locus_10527_Transcript_1/1_Conf_1.000	264	0							
Locus_10528_Transcript_1/2_Conf_1.000	287	0							
Locus_10528_Transcript_2/2_Conf_1.000	811	20	6.60E-71	XP_002633717.1	84	271.552	189	159	Hypothetical protein CBG03401
Locus_10529_Transcript_1/1_Conf_1.000	484	0							
Locus_1053_Transcript_1/1_Conf_1.000	1542	20	7.52E-21	EFN82558.1	52	106.686	211	110	Choline transporter-like protein 1
Locus_10530_Transcript_1/1_Conf_1.000	308	20	3.58E-36	NP_509947.1	85	154.451	102	87	hypothetical protein T04F8.2
Locus_10531_Transcript_1/1_Conf_1.000	400	20	9.93E-26	XP_001901258.1	98	119.783	75	74	polyprotein

Locus_10532_Transcript_1/1_Conf_1.000	593	4	1.91E-34	EFO21187.1	72	149.443	204	147	hypothetical protein LOAG_07303
Locus_10533_Transcript_1/1_Conf_1.000	1129	2	1.32E-50	NP_500393.2	53	204.912	321	173	hypothetical protein Y37E11AL.5
Locus_10534_Transcript_1/1_Conf_1.000	371	0							
Locus_10535_Transcript_1/1_Conf_1.000	178	0							
Locus_10536_Transcript_1/1_Conf_1.000	168	20	5.07E-06	XP_001487335.1	65	54.299	52	34	hypothetical protein PGUG_00712
Locus_10537_Transcript_1/2_Conf_1.000	1172	20	2.15E-43	EFO24463.1	51	181.03	324	166	hypothetical protein LOAG_04022
Locus_10537_Transcript_2/2_Conf_1.000	1298	20	3.13E-54	EFO24463.1	50	217.238	407	207	hypothetical protein LOAG_04022
Locus_10538_Transcript_1/1_Conf_1.000	1087	0							
Locus_10539_Transcript_1/1_Conf_1.000	983	0							
Locus_1054_Transcript_1/1_Conf_1.000	395	20	2.91E-33	NP_001040743.1	71	144.821	126	90	hypothetical protein C17C3.1
Locus_10540_Transcript_1/1_Conf_1.000	741	20	3.17E-90	NP_502165.2	79	335.495	247	196	hypothetical protein C10C6.6
Locus_10541_Transcript_1/1_Conf_1.000	214	0							
Locus_10542_Transcript_1/1_Conf_1.000	520	0							

Locus_10543_Transcript_1/1_Conf_1.000	191	20	1.22E-23	XP_001893730.1	93	112.849	63	59	hypothetical protein
Locus_10544_Transcript_1/1_Conf_1.000	1113	20	1.19E-96	AAR86713.1	72	357.836	309	223	LIN-45 isoform 3
Locus_10545_Transcript_1/1_Conf_1.000	484	20	1.21E-63	CBJ25054.1	83	245.743	161	135	C. elegans protein C10C6.1b, confirmed by transcript evidence
Locus_10546_Transcript_1/1_Conf_1.000	644	20	5.76E-65	XP_002642138.1	73	251.136	230	169	C. briggsae CBR-HSE-5 protein
Locus_10547_Transcript_1/1_Conf_1.000	219	0							
Locus_10548_Transcript_1/2_Conf_1.000	593	20	2.56E-55	NP_001022992.1	76	218.779	198	151	CYtoKinesis defect family member (cyk-3)
Locus_10548_Transcript_2/2_Conf_1.000	593	20	4.22E-58	NP_001022992.1	75	228.024	198	150	CYtoKinesis defect family member (cyk-3)
Locus_10549_Transcript_1/1_Conf_1.000	853	0							
Locus_1055_Transcript_1/2_Conf_1.000	1953	20	1.42E-139	NP_495443.1	67	501.516	621	420	hypothetical protein ZK1127.9
Locus_1055_Transcript_2/2_Conf_1.000	1953	20	1.41E-139	NP_495443.1	67	501.516	621	420	hypothetical protein ZK1127.9

Locus_10550_Transcript_1/1_Conf_1.000	351	0							
Locus_10551_Transcript_1/1_Conf_1.000	138	0							
Locus_10552_Transcript_1/1_Conf_1.000	740	20	1.17E-36	NP_507534.1	59	157.532	206	122	hypothetical protein Y37H2A.1
Locus_10553_Transcript_1/1_Conf_1.000	769	20	2.77E-76	ADF56010.1	90	289.271	193	175	nicotinic acetylcholine receptor alpha subunit 63a
Locus_10554_Transcript_1/1_Conf_1.000	682	5	3.37E-61	NP_498608.1	76	238.81	192	146	hypothetical protein B0361.9
Locus_10555_Transcript_1/2_Conf_1.000	593	20	5.86E-76	XP_002634704.1	90	287.345	159	144	Hypothetical protein CBG19690
Locus_10555_Transcript_2/2_Conf_1.000	441	20	1.58E-47	XP_002634704.1	93	192.2	102	95	Hypothetical protein CBG19690
Locus_10556_Transcript_1/1_Conf_1.000	427	13	1.38E-19	NP_505729.1	67	99.3673	107	72	hypothetical protein F29F11.3
Locus_10557_Transcript_1/1_Conf_1.000	658	20	5.59E-26	XP_001893620.1	57	121.709	168	97	TAP C-terminal domain containing protein
Locus_10558_Transcript_1/1_Conf_1.000	599	20	3.70E-41	NP_491404.2	80	171.785	135	108	hypothetical protein D1007.16
Locus_10559_Transcript_1/1_Conf_1.000	475	20	2.33E-27	AAN10061.1	48	125.176	172	84	Kunitz-like protease inhibitor precursor
Locus_1056_Transcript_1/2_Conf_1.000	1610	3	1.16E-96	NP_495676.1	64	358.607	400	259	hypothetical protein DH11.2
Locus_1056_Transcript_2/2_Conf_1.000	383	0							
Locus_10560_Transcript_1/1_Conf_1.000	138	0							
Locus_10561_Transcript_1/1_Conf_1.000	389	20	1.95E-66	XP_002632356.1	94	254.988	128	121	Hypothetical protein CBG00370
Locus_10562_Transcript_1/1_Conf_1.000	421	20	4.59E-55	XP_002636678.1	87	217.238	138	121	C. briggsae CBR-CHE-11 protein
Locus_10563_Transcript_1/1_Conf_1.000	923	1	6.50E-07	XP_002647226.1	55	59.3066	68	38	Hypothetical protein CBG23816

Locus_10564_Transcript_1/1_Conf_1.000	693	20	1.07E-17	CAR63567.1	73	94.3597	64	47	putative COLlagen
Locus_10565_Transcript_1/1_Conf_1.000	436	20	1.27E-33	NP_497617.1	68	145.976	142	97	hypothetical protein Y71H2AM.20
Locus_10566_Transcript_1/1_Conf_1.000	393	14	8.21E-20	NP_496207.1	62	100.138	124	77	hypothetical protein T01E8.4
Locus_10567_Transcript_1/1_Conf_1.000	1264	19	1.32E-25	NP_001020993.1	41	122.094	452	187	Suppressor of PResenilin defect family member (spr-4)
Locus_10568_Transcript_1/1_Conf_1.000	894	5	1.73E-09	XP_002645552.1	42	67.781	182	78	Hypothetical protein CBG05235
Locus_10569_Transcript_1/1_Conf_1.000	587	20	2.71E-57	P34578.3	75	225.328	195	147	Rap guanine nucleotide exchange factor 1
Locus_1057_Transcript_1/1_Conf_1.000	637	20	3.22E-28	XP_001901523.1	95	129.028	66	63	U6 snRNA-associated Sm-like protein LSm5
Locus_10570_Transcript_1/2_Conf_1.000	359	1	3.51E-07	NP_502367.2	81	58.151	60	49	hypothetical protein F28D1.9
Locus_10570_Transcript_2/2_Conf_1.000	359	1	9.24E-08	NP_502367.2	81	60.077	60	49	hypothetical protein F28D1.9
Locus_10571_Transcript_1/1_Conf_1.000	199	2	1.22E-07	NP_505304.1	78	59.6918	42	33	TransThyretin-Related family domain family member (ttr-47)
Locus_10572_Transcript_1/1_Conf_1.000	226	20	7.16E-24	EFO24987.1	89	113.62	74	66	hypothetical protein LOAG_03502
Locus_10573_Transcript_1/1_Conf_1.000	461	20	1.89E-37	NP_500964.1	92	158.688	96	89	LSM Sm-like protein family member (lsm-8)
Locus_10574_Transcript_1/1_Conf_1.000	265	0							

Locus_10575_Transcript_1/1_Conf_1.000	215	20	2.83E-25	NP_491658.2	92	118.242	71	66	PaTched Related family member (ptr-10)
Locus_10576_Transcript_1/1_Conf_1.000	293	0							
Locus_10577_Transcript_1/1_Conf_1.000	434	3	2.12E-04	NP_001021684.1	66	48.9062	65	43	hypothetical protein Y105E8A.24
Locus_10578_Transcript_1/1_Conf_1.000	444	20	1.33E-46	NP_494807.1	75	189.119	160	120	Variable ABnormal morphology family member (vab-1)
Locus_10579_Transcript_1/1_Conf_1.000	804	20	9.79E-83	NP_509268.1	76	310.842	263	202	hypothetical protein F48E3.3
Locus_1058_Transcript_1/1_Conf_1.000	944	0							
Locus_10580_Transcript_1/1_Conf_1.000	466	20	6.02E-31	CAA75875.1	98	137.117	90	89	COL1A1 and PDGFB fusion transcript

Locus_10581_Transcript_1/2_Conf_1.000	538	0							
Locus_10581_Transcript_2/2_Conf_1.000	1550	20	5.99E-135	NP_001129871.1	90	485.723	302	272	related to yeast Vacuolar Protein Sorting factor family member (vps-26)
Locus_10582_Transcript_1/1_Conf_1.000	205	0							
Locus_10583_Transcript_1/1_Conf_1.000	374	20	1.34E-46	2WS2	84	189.119	124	105	The 2 AngstromStructure Of A Nu-Class Gst From Haemonchus Contortus
Locus_10584_Transcript_1/1_Conf_1.000	868	20	6.23E-33	NP_510225.2	61	145.591	187	115	hypothetical protein C49F8.2
Locus_10585_Transcript_1/1_Conf_1.000	350	0							
Locus_10586_Transcript_1/1_Conf_1.000	302	0							
Locus_10587_Transcript_1/1_Conf_1.000	701	20	2.69E-24	XP_002639987.1	73	116.316	96	71	C. briggsae CBR-AAP-1 protein
Locus_10588_Transcript_1/1_Conf_1.000	1545	1	3.76E-04	NP_496209.1	42	51.2174	224	95	hypothetical protein T01E8.5
Locus_10589_Transcript_1/1_Conf_1.000	171	0							
Locus_1059_Transcript_1/1_Conf_1.000	1512	20	1.62E-76	EFO22174.1	52	291.582	561	293	hypothetical protein LOAG_06316
Locus_10590_Transcript_1/1_Conf_1.000	167	0							
Locus_10591_Transcript_1/1_Conf_1.000	348	20	7.32E-45	XP_002641854.1	85	183.341	114	97	Hypothetical protein CBG16528
Locus_10592_Transcript_1/1_Conf_1.000	344	2	2.03E-10	NP_506363.1	64	68.9366	70	45	hypothetical protein C50B8.4
Locus_10593_Transcript_1/1_Conf_1.000	204	20	1.05E-27	XP_001900447.1	95	126.331	67	64	cyclin fold protein 1 variant b
Locus_10594_Transcript_1/1_Conf_1.000	676	20	3.31E-53	NP_491872.1	62	212.231	229	142	hypothetical protein C55B7.3
Locus_10595_Transcript_1/1_Conf_1.000	532	20	9.25E-58	XP_002631571.1	86	226.483	137	118	C. briggsae CBR-HOT-3 protein
Locus_10596_Transcript_1/1_Conf_1.000	908	0							
Locus_10597_Transcript_1/3_Conf_0.667	1299	20	5.39E-30	NP_498099.1	51	136.732	283	145	Caffeine Induced Death (S. pombe Cid) homolog family member (cid-1)
Locus_10597_Transcript_2/3_Conf_0.667	1140	20	8.12E-32	NP_498099.1	48	142.51	347	168	Caffeine Induced Death (S. pombe Cid) homolog family member (cid-1)
Locus_10597_Transcript_3/3_Conf_0.667	1281	20	9.63E-32	NP_498099.1	48	142.51	347	168	Caffeine Induced Death (S. pombe Cid) homolog family member (cid-1)
Locus_10598_Transcript_1/1_Conf_1.000	517	20	7.01E-65	NP_497837.1	89	249.98	169	151	Leucyl tRNA Synthetase family member (lrs-1)

Locus_10599_Transcript_1/1_Conf_1.000	921	20	4.31E-43	EFO17749.1	67	179.489	190	129	hypothetical protein LOAG_10750
Locus_106_Transcript_1/1_Conf_1.000	1858	20	0	ABC86956.1	98	882.863	493	484	protein disulfide isomerase
Locus_1060_Transcript_1/6_Conf_0.462	757	0							
Locus_1060_Transcript_2/6_Conf_0.154	387	0							
Locus_1060_Transcript_3/6_Conf_0.231	760	0							
Locus_1060_Transcript_4/6_Conf_0.385	760	0							
Locus_1060_Transcript_5/6_Conf_0.308	760	0							
Locus_1060_Transcript_6/6_Conf_0.615	778	0							
Locus_10600_Transcript_1/1_Conf_1.000	309	0							
Locus_10601_Transcript_1/1_Conf_1.000	361	20	5.54E-29	EFO20355.1	70	130.568	120	85	aminotransferase
Locus_10602_Transcript_1/1_Conf_1.000	668								
Locus_10603_Transcript_1/1_Conf_1.000	184	1	8.01E-07	NP_509241.1	63	56.9954	61	39	AuTophaGy (yeast Atg homolog) family member (atg-11)
Locus_10604_Transcript_1/1_Conf_1.000	728	0							
Locus_10605_Transcript_1/1_Conf_1.000	649	4	1.37E-29	XP_002639894.1	61	133.65	214	131	C. briggsae CBR-CDC-48.3 protein
Locus_10606_Transcript_1/1_Conf_1.000	557	11	8.65E-44	CAR63705.1	88	180.259	139	123	putative CREB Homolog family member
Locus_10607_Transcript_1/1_Conf_1.000	311	20	8.24E-25	XP_002642057.1	76	116.701	103	79	C. briggsae CBR-COL-89 protein
Locus_10608_Transcript_1/1_Conf_1.000	134	0							
Locus_10609_Transcript_1/1_Conf_1.000	424	0							
Locus_1061_Transcript_1/1_Conf_1.000	143	0							
Locus_10610_Transcript_1/1_Conf_1.000	673	20	1.72E-78	NP_497896.1	72	296.204	228	165	PDCD (mammalian ProgrammeD Cell Death protein) homolog family member (pdcd-2)
Locus_10611_Transcript_1/1_Conf_1.000	568	3	1.08E-12	NP_740803.1	46	77.0258	195	90	Nuclear Pore complex Protein family member (npp-13)
Locus_10612_Transcript_1/1_Conf_1.000	1135	20	5.05E-175	XP_002632772.1	87	618.231	383	334	C. briggsae CBR-TAT-2 protein

Locus_10613_Transcript_1/1_Conf_1.000	331	5	5.42E-08	XP_001637135.1	60	60.8474	74	45	predicted protein
Locus_10614_Transcript_1/1_Conf_1.000	155	0							
Locus_10615_Transcript_1/1_Conf_1.000	513	20	3.44E-24	EFO25156.1	76	114.775	96	73	hypothetical protein LOAG_03329
Locus_10616_Transcript_1/1_Conf_1.000	942	20	2.46E-41	NP_493396.1	54	173.711	303	164	hypothetical protein W04A4.5
Locus_10617_Transcript_1/1_Conf_1.000	986	20	1.53E-73	AAK62032.1	66	280.796	299	199	metalloprotease 1 precursor
Locus_10618_Transcript_1/1_Conf_1.000	654	16	4.64E-49	NP_741081.1	69	198.364	227	158	GEX Interacting protein family member (gei-16)
Locus_10619_Transcript_1/2_Conf_1.000	1872	20	1.26E-44	EFO20169.1	46	186.037	505	233	hypothetical protein LOAG_08321
Locus_10619_Transcript_2/2_Conf_1.000	212	0							
Locus_1062_Transcript_1/1_Conf_1.000	500	20	9.02E-64	NP_497837.1	87	246.128	166	146	Leucyl tRNA Synthetase family member (lrs-1)
Locus_10620_Transcript_1/1_Conf_1.000	515	20	1.58E-53	XP_002637051.1	80	212.231	144	116	C. briggsae CBR-TAG-315 protein
Locus_10621_Transcript_1/1_Conf_1.000	449	20	1.97E-66	NP_494777.1	87	254.988	149	130	MoLTing defective family member (mlt-7)
Locus_10622_Transcript_1/2_Conf_1.000	376	0							
Locus_10622_Transcript_2/2_Conf_1.000	376	0							
Locus_10623_Transcript_1/1_Conf_1.000	134	0							
Locus_10624_Transcript_1/1_Conf_1.000	134	2	1.13E-05	XP_002644763.1	79	53.1434	39	31	Hypothetical protein CBG14774
Locus_10625_Transcript_1/1_Conf_1.000	237	0							
Locus_10626_Transcript_1/1_Conf_1.000	243	2	2.51E-05	NP_507767.2	72	51.9878	55	40	hypothetical protein C14B4.2
Locus_10627_Transcript_1/1_Conf_1.000	493	0							
Locus_10628_Transcript_1/1_Conf_1.000	298	0							

Locus_10629_Transcript_1/1_Conf_1.000	299	20	3.50E-39	XP_002640690.1	88	164.466	99	88	Hypothetical protein CBG19753
Locus_1063_Transcript_1/2_Conf_1.000	392	20	7.20E-29	CAC51087.1	87	130.183	80	70	ES protein
Locus_1063_Transcript_2/2_Conf_1.000	794	20	1.70E-39	CAC51087.1	74	167.162	150	111	ES protein
Locus_10630_Transcript_1/1_Conf_1.000	375	20	2.78E-36	EFO20071.1	79	154.836	114	91	hypothetical protein LOAG_08420
Locus_10631_Transcript_1/1_Conf_1.000	710	20	4.53E-112	Q61YG1.2	88	407.912	236	209	Leishmanolysin-like peptidase
Locus_10632_Transcript_1/1_Conf_1.000	209	0							

Locus_10633_Transcript_1/1_Conf_1.000	231	20	3.43E-10	XP_001950127.1	77	68.1662	53	41	PREDICTED: similar to neuralized
Locus_10634_Transcript_1/1_Conf_1.000	151	0							
Locus_10635_Transcript_1/1_Conf_1.000	341	3	3.83E-14	XP_002631596.1	61	81.2629	107	66	Hypothetical protein CBG20777
Locus_10636_Transcript_1/1_Conf_1.000	2072	20	2.07E-128	ACI49231.1	62	464.537	636	395	hypothetical protein Csp3_JD05.016
Locus_10637_Transcript_1/1_Conf_1.000	776	20	1.23E-47	NP_504560.1	78	194.126	173	135	hypothetical protein F29G9.2
Locus_10638_Transcript_1/1_Conf_1.000	145	0							
Locus_10639_Transcript_1/1_Conf_1.000	354	0							
Locus_1064_Transcript_1/1_Conf_1.000	249	0							
Locus_10640_Transcript_1/1_Conf_1.000	675	20	1.27E-12	NP_001022292.1	72	77.411	81	59	hypothetical protein T01B7.5
Locus_10641_Transcript_1/5_Conf_0.167	440	0							
Locus_10641_Transcript_2/5_Conf_0.667	1651	20	5.81E-75	EFO23614.1	60	286.574	370	223	hypothetical protein LOAG_04870

Locus_10641_Transcript_3/5_Conf_0.667	1651	20	5.81E-75	EFO23614.1	60	286.574	370	223	hypothetical protein LOAG_04870
Locus_10641_Transcript_4/5_Conf_0.667	1651	20	5.81E-75	EFO23614.1	60	286.574	370	223	hypothetical protein LOAG_04870
Locus_10641_Transcript_5/5_Conf_0.667	1651	20	5.82E-75	EFO23614.1	60	286.574	370	223	hypothetical protein LOAG_04870
Locus_10642_Transcript_1/1_Conf_1.000	178	20	6.98E-24	XP_002632085.1	98	113.62	57	56	Hypothetical protein CBG17053
Locus_10643_Transcript_1/1_Conf_1.000	182	1	4.88E-04	NP_501925.2	68	47.7506	48	33	hypothetical protein F13B12.2
Locus_10644_Transcript_1/1_Conf_1.000	380	0							
Locus_10645_Transcript_1/1_Conf_1.000	182	0							
Locus_10646_Transcript_1/1_Conf_1.000	591	20	8.84E-40	NP_501552.1	77	167.162	150	116	hypothetical protein D1046.3
Locus_10647_Transcript_1/3_Conf_0.500	239	0							
Locus_10647_Transcript_2/3_Conf_0.333	1328	20	7.09E-110	XP_002646340.1	69	402.134	383	266	C. briggsae CBR-INX-15 protein
Locus_10647_Transcript_3/3_Conf_0.500	337	15	3.48E-07	NP_001021592.1	72	58.151	44	32	INneXin family member (inx-17)
Locus_10648_Transcript_1/1_Conf_1.000	988	19	1.32E-08	XP_001892028.1	50	65.0846	196	99	hypothetical protein Bm1_02630
Locus_10649_Transcript_1/1_Conf_1.000	145	0							
Locus_1065_Transcript_1/2_Conf_1.000	1061	20	1.57E-34	NP_505463.1	96	151.369	103	99	HIStone family member (his-35)

Locus_1065_Transcript_2/2_Conf_1.000	1061	20	8.30E-36	NP_505463.1	96	155.606	104	100	HIStone family member (his-35)
Locus_10650_Transcript_1/1_Conf_1.000	211	20	1.73E-14	XP_002428738.1	72	82.4185	70	51	Sex-determining protein fem-1, putative
Locus_10651_Transcript_1/1_Conf_1.000	577	18	1.69E-16	XP_002630479.1	60	89.7373	193	116	Hypothetical protein CBG11215
Locus_10652_Transcript_1/2_Conf_1.000	297	0							
Locus_10652_Transcript_2/2_Conf_1.000	286	0							
Locus_10653_Transcript_1/2_Conf_1.000	426	20	2.85E-57	NP_496822.2	85	224.557	141	121	hypothetical protein Y48C3A.14
Locus_10653_Transcript_2/2_Conf_1.000	397	20	6.20E-52	NP_496822.2	84	206.838	132	112	hypothetical protein Y48C3A.14
Locus_10654_Transcript_1/1_Conf_1.000	347	20	3.56E-15	XP_002429463.1	55	84.7297	106	59	set domain protein, putative

Locus_10655_Transcript_1/1_Conf_1.000	436	20	4.68E-28	AAC47238.1	73	127.487	114	84	non-muscle myosin heavy chain II
Locus_10656_Transcript_1/1_Conf_1.000	145	0							
Locus_10657_Transcript_1/1_Conf_1.000	153	0							
Locus_10658_Transcript_1/1_Conf_1.000	566	20	3.16E-49	XP_001901789.1	70	198.364	185	130	protein F41H10.5
Locus_1066_Transcript_1/1_Conf_1.000	781	20	2.95E-97	XP_002639719.1	91	358.992	199	182	Hypothetical protein CBG12445
Locus_10660_Transcript_1/1_Conf_1.000	353	20	1.33E-38	NP_496500.1	82	162.54	117	96	hypothetical protein VF13D12L.3
Locus_10661_Transcript_1/1_Conf_1.000	451	9	6.40E-17	XP_002632202.1	54	90.5077	121	66	Hypothetical protein CBG07069
Locus_10662_Transcript_1/1_Conf_1.000	607	20	2.58E-53	XP_002639587.1	75	212.231	183	138	Hypothetical protein CBG04225

Locus_10663_Transcript_1/1_Conf_1.000	242	20	4.58E-07	Q90WA0.1	63	57.7658	57	36	Textilinin-2
Locus_10664_Transcript_1/1_Conf_1.000	484	20	5.68E-13	NP_500023.1	64	77.411	94	61	hypothetical protein Y55F3AM.9
Locus_10665_Transcript_1/1_Conf_1.000	435	20	2.32E-27	NP_871792.1	90	125.176	81	73	hypothetical protein F08A10.1
Locus_10666_Transcript_1/1_Conf_1.000	468	20	8.99E-11	XP_001604140.1	63	70.0922	82	52	PREDICTED: similar to GM05135p
Locus_10667_Transcript_1/1_Conf_1.000	686	2	8.79E-17	NP_505881.3	63	91.2781	82	52	hypothetical protein T02E9.5
Locus_10668_Transcript_1/1_Conf_1.000	380	3	2.53E-05	AAK93849.3	49	51.9878	69	34	U12964_12Hypothetical protein F26F4.9a
Locus_10669_Transcript_1/1_Conf_1.000	514	20	4.20E-30	XP_001899440.1	62	134.42	159	99	CPSF A subunit region family protein
Locus_1067_Transcript_1/1_Conf_1.000	503	20	1.18E-42	NP_496168.1	78	176.022	140	110	hypothetical protein F52H3.5
Locus_10670_Transcript_1/1_Conf_1.000	660	1	7.82E-04	ZP_05807298.1	50	48.1358	81	41	glycosyl transferase family 51
Locus_10671_Transcript_1/1_Conf_1.000	370	0							
Locus_10672_Transcript_1/2_Conf_1.000	1034	0							
Locus_10672_Transcript_2/2_Conf_1.000	1034	0							
Locus_10673_Transcript_1/1_Conf_1.000	662	1	2.69E-04	YP_031621.1	50	49.6766	51	26	hypothetical protein FV3gorf43R
Locus_10674_Transcript_1/1_Conf_1.000	140	0							
Locus_10675_Transcript_1/1_Conf_1.000	698	2	1.79E-12	NP_497036.1	46	77.0258	150	70	hypothetical protein F19H8.2
Locus_10676_Transcript_1/1_Conf_1.000	903	20	1.21E-159	AAD39095.1	100	566.614	278	278	AF105337_1galectin GAL-1
Locus_10677_Transcript_1/1_Conf_1.000	243	0							
Locus_10678_Transcript_1/1_Conf_1.000	242	0							

Locus_10679_Transcript_1/1_Conf_1.000	149	20	4.23E-16	NP_502747.1	85	87.8113	48	41	hypothetical protein F55B11.1
Locus_1068_Transcript_1/1_Conf_1.000	1134	20	4.40E-78	NP_492519.2	74	296.204	315	236	hypothetical protein F30A10.3
Locus_10680_Transcript_1/1_Conf_1.000	783	20	7.66E-53	XP_002631256.1	66	211.46	245	164	C. briggsae CBR-CRN-3 protein
Locus_10681_Transcript_1/1_Conf_1.000	411	0							
Locus_10682_Transcript_1/1_Conf_1.000	1489	4	2.57E-10	NP_492568.1	46	71.633	179	83	Nuclear Pore complex Protein family member (npp-14)
Locus_10683_Transcript_1/1_Conf_1.000	483	0							
Locus_10684_Transcript_1/1_Conf_1.000	1015	3	3.53E-12	NP_499551.1	55	77.0258	177	99	Nuclear Pore complex Protein family member (npp-16)
Locus_10685_Transcript_1/1_Conf_1.000	262	0							
Locus_10686_Transcript_1/1_Conf_1.000	769	20	9.71E-13	NP_493412.2	55	78.1814	114	63	hypothetical protein Y37H9A.3
Locus_10687_Transcript_1/1_Conf_1.000	481	6	7.46E-18	XP_001899819.1	56	93.5893	141	79	protein ZK524.3
Locus_10688_Transcript_1/1_Conf_1.000	152	0							
Locus_10689_Transcript_1/1_Conf_1.000	886	7	1.72E-70	XP_002646401.1	69	270.396	321	223	C. briggsae CBR-DYS-1 protein

Locus_1069_Transcript_1/1_Conf_1.000	984	20	9.76E-121	XP_002637163.1	88	437.573	296	262	C. briggsae CBR-SQV-4 protein
Locus_10690_Transcript_1/1_Conf_1.000	409	20	5.97E-15	NP_508961.2	70	83.9593	74	52	CYtochrome P450 family member (cyp-43A1)
Locus_10691_Transcript_1/1_Conf_1.000	596	20	3.74E-70	NP_001021801.1	85	268.085	187	159	hypothetical protein Y6B3B.5
Locus_10692_Transcript_1/1_Conf_1.000	314	20	5.17E-14	ABO70952.1	96	80.8777	89	86	NADH dehydrogenase subunit 3
Locus_10693_Transcript_1/2_Conf_0.500	650	20	9.14E-50	AAK94765.1	64	200.675	208	135	GLY-2
Locus_10693_Transcript_2/2_Conf_0.500	650	20	9.14E-50	AAK94765.1	64	200.675	208	135	GLY-2
Locus_10694_Transcript_1/1_Conf_1.000	236	0							
Locus_10695_Transcript_1/1_Conf_1.000	401	7	1.59E-07	XP_002630536.1	56	59.3066	96	54	Hypothetical protein CBG12976

Locus_10696_Transcript_1/2_Conf_1.000	371	20	6.91E-35	NP_508412.1	92	150.214	84	78	Vacuolar H ATPase family member (vha-15)
Locus_10696_Transcript_2/2_Conf_1.000	238	17	1.17E-10	NP_508412.1	90	69.707	40	36	Vacuolar H ATPase family member (vha-15)
Locus_10697_Transcript_1/1_Conf_1.000	693	20	1.93E-67	NP_001122528.1	74	259.61	233	173	Cation transporting ATPase family member (catp-1)
Locus_10698_Transcript_1/1_Conf_1.000	429	0							
Locus_10699_Transcript_1/1_Conf_1.000	178	10	2.93E-22	ABD62754.1	91	108.227	58	53	Hc38
Locus_107_Transcript_1/2_Conf_1.000	4141	20	0	XP_002631199.1	79	1784.62	1356	1082	C. briggsae CBR-PTP-3 protein
Locus_107_Transcript_2/2_Conf_1.000	1751	20	0	XP_002631199.1	77	707.212	532	414	C. briggsae CBR-PTP-3 protein
Locus_1070_Transcript_1/1_Conf_1.000	271	0							

Locus_10700_Transcript_1/1_Conf_1.000	737	20	1.27E-67	XP_001099416.2	100	260.381	141	141	PREDICTED: profilin-1 isoform 1
Locus_10701_Transcript_1/1_Conf_1.000	935	0							
Locus_10702_Transcript_1/1_Conf_1.000	601	20	1.05E-75	NP_001023382.1	84	286.574	200	169	Amino Acid Transporter family member (aat-4)
Locus_10703_Transcript_1/1_Conf_1.000	663	20	2.26E-59	NP_498961.2	87	232.646	142	124	hypothetical protein ZK637.2
Locus_10704_Transcript_1/2_Conf_1.000	770	20	2.68E-87	NP_502782.2	88	325.865	212	188	hypothetical protein Y57G11C.34
Locus_10704_Transcript_2/2_Conf_1.000	770	20	2.67E-87	NP_502782.2	88	325.865	212	188	hypothetical protein Y57G11C.34
Locus_10705_Transcript_1/1_Conf_1.000	265	0							
Locus_10706_Transcript_1/1_Conf_1.000	1172	20	7.09E-63	XP_002630790.1	57	245.743	383	222	Hypothetical protein CBG02487
Locus_10707_Transcript_1/1_Conf_1.000	299	0							
Locus_10708_Transcript_1/3_Conf_0.714	971	20	1.86E-108	XP_002641258.1	79	396.741	323	256	Hypothetical protein CBG05169
Locus_10708_Transcript_2/3_Conf_0.714	969	20	7.81E-107	XP_002641258.1	78	391.349	322	254	Hypothetical protein CBG05169
Locus_10708_Transcript_3/3_Conf_0.714	971	20	1.86E-108	XP_002641258.1	79	396.741	323	257	Hypothetical protein CBG05169

Locus_10709_Transcript_1/1_Conf_1.000	132	20	4.71E-20	XP_002818641.1	100	100.908	43	43	PREDICTED: LOW QUALITY PROTEIN: zyxin-like
Locus_1071_Transcript_1/1_Conf_1.000	643	20	3.61E-59	XP_002637505.1	70	231.876	195	138	C. briggsae CBR-ASP-2 protein
Locus_10710_Transcript_1/1_Conf_1.000	966	20	2.85E-109	XP_002636903.1	91	399.438	241	220	C. briggsae CBR-PAS-6 protein
Locus_10711_Transcript_1/1_Conf_1.000	183	20	3.14E-19	CAR63581.1	100	98.2117	49	49	putative COLlagen
Locus_10712_Transcript_1/1_Conf_1.000	259	2	6.06E-23	NP_499479.2	97	110.538	70	68	hypothetical protein Y66D12A.24
Locus_10713_Transcript_1/1_Conf_1.000	148	0							
Locus_10714_Transcript_1/1_Conf_1.000	728	3	1.13E-36	XP_002630207.1	87	157.532	125	109	Hypothetical protein CBG00617
Locus_10715_Transcript_1/3_Conf_0.400	195	0							
Locus_10715_Transcript_2/3_Conf_0.400	392	2	8.84E-11	NP_495177.2	87	70.0922	48	42	hypothetical protein F59G1.4
Locus_10715_Transcript_3/3_Conf_0.600	433	2	8.90E-11	NP_495177.2	87	70.0922	48	42	hypothetical protein F59G1.4
Locus_10716_Transcript_1/1_Conf_1.000	186	0							
Locus_10717_Transcript_1/1_Conf_1.000	272	0							
Locus_10718_Transcript_1/1_Conf_1.000	341	7	3.95E-19	NP_500997.1	65	97.8265	110	72	hypothetical protein C01G5.8
Locus_10719_Transcript_1/1_Conf_1.000	945	10	4.83E-37	XP_002640248.1	59	159.458	300	179	Hypothetical protein CBG12766
Locus_1072_Transcript_1/2_Conf_1.000	2564	3	1.89E-33	NP_498678.3	48	149.443	305	148	abnormal cell LINeage family member (lin-13)

Locus_1072_Transcript_2/2_Conf_1.000	1280	20	4.40E-69	XP_002635275.1	73	266.544	234	171	Hypothetical protein CBG11519
Locus_10720_Transcript_1/1_Conf_1.000	251	1	1.61E-04	XP_002639641.1	76	49.2914	76	58	C. briggsae CBR-PRP-4 protein
Locus_10721_Transcript_1/1_Conf_1.000	458	20	1.56E-31	ACS36089.1	69	139.043	140	97	cysteine proteinase
Locus_10722_Transcript_1/1_Conf_1.000	321	3	2.54E-05	NP_506163.1	63	51.9878	60	38	CCCH-type zinc finger transcription factor family member (ccch-3)
Locus_10723_Transcript_1/1_Conf_1.000	352	20	2.20E-33	EFN83153.1	100	145.206	74	74	Histone H3.3
Locus_10724_Transcript_1/1_Conf_1.000	371	20	7.87E-47	EFO19031.1	86	189.889	123	107	hypothetical protein LOAG_09464
Locus_10725_Transcript_1/1_Conf_1.000	358	0							
Locus_10726_Transcript_1/1_Conf_1.000	676	20	3.26E-101	XP_002823818.1	100	371.703	218	218	PREDICTED: 60S ribosomal protein L6-like isoform 1
Locus_10727_Transcript_1/1_Conf_1.000	408	20	1.01E-54	CAA10033.1	87	216.083	136	119	DYS-1 protein
Locus_10728_Transcript_1/1_Conf_1.000	254	0							
Locus_10729_Transcript_1/1_Conf_1.000	301	20	2.88E-09	EFN62060.1	76	65.0846	47	36	tRNA (cytosine-5-)-methyltransferase

Locus_1073_Transcript_1/3_Conf_0.571	293	20	7.17E-08	XP_002639373.1	66	60.4622	51	34	C. briggsae CBR-CEJ-1 protein
Locus_1073_Transcript_2/3_Conf_0.571	306	20	4.12E-08	XP_002639373.1	57	61.2326	70	40	C. briggsae CBR-CEJ-1 protein
Locus_1073_Transcript_3/3_Conf_0.714	321	20	4.19E-08	XP_002639373.1	57	61.2326	70	40	C. briggsae CBR-CEJ-1 protein
Locus_10730_Transcript_1/1_Conf_1.000	129	2	1.25E-04	XP_002646625.1	66	49.6766	42	28	Hypothetical protein CBG11055
Locus_10731_Transcript_1/1_Conf_1.000	172	0							
Locus_10732_Transcript_1/1_Conf_1.000	653								
Locus_10733_Transcript_1/2_Conf_1.000	1085	20	5.61E-136	XP_002630855.1	92	488.419	284	263	Hypothetical protein CBG02570
Locus_10733_Transcript_2/2_Conf_1.000	1087	20	2.80E-135	XP_002630855.1	92	486.108	284	262	Hypothetical protein CBG02570
Locus_10734_Transcript_1/1_Conf_1.000	409	0							
Locus_10735_Transcript_1/1_Conf_1.000	311	20	6.10E-28	NP_505267.1	81	127.102	88	72	Regulator of Presynaptic Morphology family member (rpm-1)
Locus_10736_Transcript_1/1_Conf_1.000	927	4	1.67E-10	NP_505473.1	62	71.2478	110	69	hypothetical protein F25B3.6
Locus_10737_Transcript_1/1_Conf_1.000	902	20	1.16E-61	EFO21137.1	71	241.121	261	186	hypothetical protein LOAG_07352
Locus_10738_Transcript_1/1_Conf_1.000	137	0							
Locus_10739_Transcript_1/1_Conf_1.000	253	3	6.53E-17	NP_001076639.1	67	90.5077	89	60	TWiK family of potassium channels family member (twk-39)
Locus_1074_Transcript_1/2_Conf_1.000	1338	20	3.59E-85	EFO26413.1	69	320.087	289	202	F-box only protein 11

Locus_1074_Transcript_2/2_Conf_1.000	1332	20	3.57E-85	EFO26413.1	69	320.087	289	202	F-box only protein 11
Locus_10740_Transcript_1/1_Conf_1.000	843	20	2.53E-84	NP_508198.1	88	316.235	214	189	hypothetical protein F55A4.1
Locus_10741_Transcript_3/7_Conf_0.346	106	0							
Locus_10741_Transcript_4/7_Conf_0.346	107	0							
Locus_10741_Transcript_6/7_Conf_0.308	111	0							
Locus_10742_Transcript_1/1_Conf_1.000	314	20	3.01E-38	XP_002753447.1	98	161.384	91	90	PREDICTED: 40S ribosomal protein S27-like
Locus_10743_Transcript_1/1_Conf_1.000	1003	20	7.20E-10	XP_001849742.1	41	69.3218	294	122	conserved hypothetical protein
Locus_10744_Transcript_1/2_Conf_1.000	1228	20	1.39E-56	XP_002640319.1	68	224.942	241	166	C. briggsae CBR-DHS-1 protein
Locus_10744_Transcript_2/2_Conf_1.000	1228	20	8.17E-57	XP_002640319.1	69	225.713	241	167	C. briggsae CBR-DHS-1 protein
Locus_10745_Transcript_1/1_Conf_1.000	393	15	4.67E-07	XP_002639228.1	57	57.7658	105	60	Hypothetical protein CBG03783
Locus_10746_Transcript_1/1_Conf_1.000	685	20	1.73E-89	XP_002634879.1	85	332.798	218	186	Hypothetical protein CBG10547
Locus_10747_Transcript_1/1_Conf_1.000	476	0							
Locus_10748_Transcript_1/1_Conf_1.000	501	0							
Locus_10749_Transcript_1/1_Conf_1.000	357	3	1.02E-06	ABH10655.1	71	56.6102	52	37	FMRFamide-related peptide FLP-32 precursor
Locus_1075_Transcript_1/2_Conf_1.000	2643	20	3.43E-54	XP_001898464.1	50	218.394	385	196	Innexin inx-3
Locus_1075_Transcript_2/2_Conf_1.000	2484	20	3.19E-54	XP_001898464.1	50	218.394	385	196	Innexin inx-3
Locus_10750_Transcript_1/1_Conf_1.000	786	0							

Locus_10751_Transcript_1/1_Conf_1.000	241	0							
Locus_10752_Transcript_1/1_Conf_1.000	202	0							
Locus_10753_Transcript_1/1_Conf_1.000	257	0							
Locus_10754_Transcript_1/1_Conf_1.000	275	5	4.71E-20	XP_002642379.1	69	100.908	91	63	Hypothetical protein CBG18383
Locus_10755_Transcript_1/1_Conf_1.000	1057	20	5.08E-102	NP_496236.1	88	375.555	229	202	hypothetical protein T09F3.2
Locus_10756_Transcript_1/1_Conf_1.000	714	20	3.87E-34	NP_494539.1	57	149.058	231	132	hypothetical protein C16C8.18
Locus_10757_Transcript_1/1_Conf_1.000	437	0							
Locus_10758_Transcript_1/1_Conf_1.000	154	0							
Locus_10759_Transcript_1/1_Conf_1.000	194	20	1.39E-19	NP_509702.3	90	99.3673	52	47	UNCoordinated family member (unc-115)
Locus_1076_Transcript_1/1_Conf_1.000	462	20	1.39E-27	ABF69516.1	100	125.946	63	63	acidic ribosomal protein P1
Locus_10760_Transcript_1/1_Conf_1.000	325	0							
Locus_10761_Transcript_1/1_Conf_1.000	582	18	1.41E-58	XP_002633595.1	79	229.565	154	123	Hypothetical protein CBG05472
Locus_10762_Transcript_1/2_Conf_1.000	541	20	6.44E-46	NP_001041131.1	79	187.193	144	115	ADDucin family member (add-2)
Locus_10762_Transcript_2/2_Conf_1.000	521	20	5.71E-46	NP_001041131.1	79	187.193	144	115	ADDucin family member (add-2)
Locus_10763_Transcript_1/1_Conf_1.000	457	3	5.57E-05	NP_741332.1	81	50.8322	38	31	hypothetical protein F52C12.2
Locus_10764_Transcript_1/1_Conf_1.000	319	0							
Locus_10765_Transcript_1/1_Conf_1.000	309	3	1.46E-13	NP_493616.1	57	79.337	106	61	hypothetical protein F33H2.5
Locus_10766_Transcript_1/1_Conf_1.000	171	0							

Locus_10767_Transcript_1/1_Conf_1.000	346	20	2.00E-42	XP_002636771.1	90	175.252	102	92	C. briggsae CBR-ATN-1 protein
Locus_10768_Transcript_1/1_Conf_1.000	153	0							
Locus_10769_Transcript_1/1_Conf_1.000	914	20	1.59E-82	NP_493618.1	66	310.457	316	211	Deletions Of G-rich DNA family member (dog-1)
Locus_1077_Transcript_1/1_Conf_1.000	1873	20	2.01E-34	XP_001898097.1	47	152.14	461	218	Asparaginase family protein
Locus_10770_Transcript_1/2_Conf_1.000	464	1	8.23E-04	NP_502410.2	43	46.9802	150	65	hypothetical protein K08E7.6
Locus_10770_Transcript_2/2_Conf_1.000	416	0							
Locus_10771_Transcript_1/1_Conf_1.000	440	20	1.60E-28	XP_002631177.1	90	129.028	82	74	C. briggsae CBR-LSM-1 protein
Locus_10772_Transcript_1/1_Conf_1.000	352	0							
Locus_10773_Transcript_1/1_Conf_1.000	384	20	2.27E-14	EFO23261.1	58	82.0333	85	50	hypothetical protein LOAG_05224
Locus_10774_Transcript_1/1_Conf_1.000	582	20	1.33E-48	NP_506007.1	73	196.438	163	120	CYclin L family member (cyl-1)
Locus_10775_Transcript_1/1_Conf_1.000	235	0							
Locus_10776_Transcript_1/1_Conf_1.000	212	0							

Locus_10777_Transcript_1/2_Conf_1.000	454	20	5.36E-40	NP_509572.1	86	167.162	149	129	AdaPtin, Alpha chain (clathrin associated complex) family member (apa-2)
Locus_10777_Transcript_2/2_Conf_1.000	454	20	5.36E-40	NP_509572.1	86	167.162	149	129	AdaPtin, Alpha chain (clathrin associated complex) family member (apa-2)
Locus_10778_Transcript_1/1_Conf_1.000	967	20	8.19E-40	XP_002631527.1	71	168.703	145	104	Hypothetical protein CBG20693
Locus_10779_Transcript_1/1_Conf_1.000	815	0							
Locus_1078_Transcript_1/1_Conf_1.000	3058	20	1.14E-72	XP_002634123.1	50	280.026	638	324	Hypothetical protein CBG01680
Locus_10780_Transcript_1/2_Conf_1.000	280	0							
Locus_10780_Transcript_2/2_Conf_1.000	234	0							
Locus_10781_Transcript_1/2_Conf_1.000	572	20	2.19E-45	EFO27189.1	70	185.652	190	133	hypothetical protein LOAG_01295
Locus_10781_Transcript_2/2_Conf_1.000	566	20	6.59E-47	EFO27189.1	70	190.66	188	133	hypothetical protein LOAG_01295
Locus_10782_Transcript_1/1_Conf_1.000	376	20	2.62E-18	NP_001032980.2	64	95.1301	103	66	hypothetical protein T23H2.3
Locus_10783_Transcript_1/1_Conf_1.000	150	0							

Locus_10784_Transcript_1/1_Conf_1.000	196	20	8.68E-30	XP_002758725.1	100	133.265	65	65	PREDICTED: dolichyl-diphosphooligosaccharide--protein glycosyltransferase subunit 1-like isoform 2
Locus_10785_Transcript_1/1_Conf_1.000	430	0							
Locus_10786_Transcript_1/1_Conf_1.000	691	3	5.45E-14	XP_002631728.1	47	82.0333	223	107	Hypothetical protein CBG20930
Locus_10787_Transcript_1/1_Conf_1.000	185	0							
Locus_10788_Transcript_1/1_Conf_1.000	781	20	5.13E-25	NP_503033.1	68	119.013	115	79	Splicing FActor family member (sfa-1)
Locus_10789_Transcript_1/1_Conf_1.000	174	5	2.19E-09	XP_002634414.1	70	65.4698	57	40	C. briggsae CBR-EPI-1 protein
Locus_1079_Transcript_1/1_Conf_1.000	838	20	1.05E-66	XP_001896264.1	83	257.684	181	152	cyclophilin-type peptidyl-prolyl cis-trans isomerase-15, Bmcyp-5
Locus_10790_Transcript_1/1_Conf_1.000	241	3	3.63E-04	AAQ06435.1	70	48.1358	44	31	ABC6 protein
Locus_10791_Transcript_1/1_Conf_1.000	732	0							
Locus_10792_Transcript_1/1_Conf_1.000	139	0							

Locus_10793_Transcript_1/1_Conf_1.000	281	7	9.16E-08	NP_510369.2	55	60.077	79	44	CYtochrome P450 family member (cyp-13B2)
Locus_10794_Transcript_1/1_Conf_1.000	315	20	2.16E-20	XP_002633256.1	70	102.064	100	70	Hypothetical protein CBG05981
Locus_10795_Transcript_1/2_Conf_1.000	519	20	2.49E-33	XP_002636807.1	76	145.206	113	87	Hypothetical protein CBG09249
Locus_10795_Transcript_2/2_Conf_1.000	816	20	3.00E-79	XP_002636807.1	78	299.286	230	181	Hypothetical protein CBG09249
Locus_10796_Transcript_1/1_Conf_1.000	187	3	6.33E-04	NP_001122796.1	68	47.3654	50	34	Kinesin-Like Protein family member (klp-12)
Locus_10797_Transcript_1/1_Conf_1.000	275	13	2.68E-07	XP_001989288.1	61	58.5362	59	36	GH11645
Locus_10798_Transcript_1/1_Conf_1.000	215	20	3.69E-33	NP_001123995.1	100	144.436	71	71	cold shock domain-containing protein E1 isoform 3
Locus_10799_Transcript_1/1_Conf_1.000	496	5	1.92E-13	XP_001900701.1	55	78.9518	136	76	hypothetical protein Bm1_46195
Locus_108_Transcript_1/6_Conf_0.333	475	0							
Locus_108_Transcript_2/6_Conf_0.333	475	0							
Locus_108_Transcript_3/6_Conf_0.333	342	2	2.32E-06	XP_002648823.1	50	55.4546	60	30	Hypothetical protein CBG16936
Locus_108_Transcript_4/6_Conf_0.333	342	2	2.32E-06	XP_002648823.1	50	55.4546	60	30	Hypothetical protein CBG16936
Locus_108_Transcript_5/6_Conf_0.333	291	0							
Locus_108_Transcript_6/6_Conf_0.333	333	1	1.24E-04	XP_002648823.1	46	49.6766	60	28	Hypothetical protein CBG16936
Locus_1080_Transcript_1/1_Conf_1.000	1309	7	2.24E-108	NP_492018.1	66	397.127	474	313	UNCoordinated family member (unc-14)
Locus_10800_Transcript_1/1_Conf_1.000	502	20	7.67E-18	XP_002645115.1	73	93.5893	84	62	Hypothetical protein CBG16808
Locus_10801_Transcript_1/1_Conf_1.000	531	20	2.46E-18	XP_002634401.1	74	95.5153	82	61	C. briggsae CBR-PQN-55 protein

Locus_10802_Transcript_1/1_Conf_1.000	744	20	8.72E-80	NP_508573.1	82	300.827	195	160	Discoidin Domain Receptor family member (ddr-2)
Locus_10803_Transcript_1/1_Conf_1.000	216	0							
Locus_10804_Transcript_1/1_Conf_1.000	1267	20	1.57E-127	XP_002642404.1	75	460.685	398	299	Hypothetical protein CBG06798
Locus_10805_Transcript_1/1_Conf_1.000	295	20	1.74E-38	XP_002640025.1	89	162.155	97	87	Hypothetical protein CBG12497
Locus_10806_Transcript_1/1_Conf_1.000	257	20	5.90E-10	ACI49231.1	67	67.3958	80	54	hypothetical protein Csp3_JD05.016
Locus_10807_Transcript_1/1_Conf_1.000	144	0							
Locus_10808_Transcript_1/1_Conf_1.000	842	20	2.77E-91	NP_741746.1	80	339.347	255	204	Kinesin-Like Protein family member (klp-4)
Locus_10809_Transcript_1/1_Conf_1.000	138	9	1.20E-07	NP_499580.1	82	59.6918	45	37	hypothetical protein Y75B8A.7

Locus_1081_Transcript_1/2_Conf_1.000	268	14	5.81E-10	Q94637.1	63	67.3958	87	55	Vitellogenin-6
Locus_1081_Transcript_2/2_Conf_1.000	159	0							
Locus_10810_Transcript_1/1_Conf_1.000	713	0							
Locus_10811_Transcript_1/2_Conf_1.000	501	0							
Locus_10811_Transcript_2/2_Conf_1.000	501	0							
Locus_10812_Transcript_1/1_Conf_1.000	606	3	2.70E-10	EFO20116.1	59	69.3218	98	58	hypothetical protein LOAG_08374
Locus_10813_Transcript_1/1_Conf_1.000	365	0							
Locus_10814_Transcript_1/1_Conf_1.000	287	2	5.00E-09	NP_495751.1	94	64.3142	34	32	hypothetical protein F14F11.2
Locus_10815_Transcript_1/1_Conf_1.000	510	20	1.23E-50	NP_500615.1	87	202.601	125	109	hypothetical protein T22B11.2
Locus_10816_Transcript_1/1_Conf_1.000	319	2	3.56E-15	XP_001894519.1	67	84.7297	101	68	hypothetical protein Bm1_15295
Locus_10817_Transcript_1/1_Conf_1.000	659	20	7.27E-42	XP_002631454.1	74	174.481	154	115	Hypothetical protein CBG25152
Locus_10818_Transcript_1/1_Conf_1.000	842	12	1.39E-42	NP_499555.1	79	177.563	181	143	hypothetical protein Y56A3A.22
Locus_10819_Transcript_1/1_Conf_1.000	939	20	1.34E-55	XP_002644604.1	95	221.09	124	118	C. briggsae CBR-TSP-14 protein
Locus_1082_Transcript_1/1_Conf_1.000	577	3	4.92E-24	XP_002633740.1	60	114.775	179	109	Hypothetical protein CBG03425
Locus_10820_Transcript_1/1_Conf_1.000	548	4	7.64E-50	XP_002641200.1	80	200.29	170	137	Hypothetical protein CBG09061
Locus_10821_Transcript_1/1_Conf_1.000	283	20	6.52E-25	XP_001900378.1	75	117.087	94	71	DNA gyrase/topoisomerase IV, A subunit family protein
Locus_10822_Transcript_1/1_Conf_1.000	435	20	2.31E-51	NP_505697.2	84	204.912	143	121	hypothetical protein C35A5.7

Locus_10823_Transcript_1/1_Conf_1.000	756	20	1.69E-54	NP_872058.1	70	216.853	226	160	hypothetical protein 2RSSE.1
Locus_10824_Transcript_1/1_Conf_1.000	776	2	2.52E-08	NP_498032.1	47	63.5438	191	90	hypothetical protein F26F4.2
Locus_10825_Transcript_1/1_Conf_1.000	220	0							
Locus_10826_Transcript_1/1_Conf_1.000	833	20	8.78E-66	XP_001894435.1	71	254.603	238	170	Ser/Thr protein phosphatase family protein
Locus_10827_Transcript_1/1_Conf_1.000	451	20	7.54E-26	EFO21349.1	82	120.168	102	84	hypothetical protein LOAG_07141
Locus_10828_Transcript_1/4_Conf_0.625	958	0							
Locus_10828_Transcript_2/4_Conf_0.625	958	0							
Locus_10828_Transcript_3/4_Conf_0.625	621	0							
Locus_10828_Transcript_4/4_Conf_0.625	958	0							
Locus_10829_Transcript_1/2_Conf_1.000	717	20	4.77E-40	EFO15133.1	64	168.703	179	115	hypothetical protein LOAG_13378
Locus_10829_Transcript_2/2_Conf_1.000	719	20	4.77E-40	EFO15133.1	64	168.703	179	115	hypothetical protein LOAG_13378
Locus_1083_Transcript_1/1_Conf_1.000	151	0							
Locus_10830_Transcript_1/1_Conf_1.000	453	0							
Locus_10831_Transcript_1/2_Conf_1.000	194	3	1.54E-18	CAR63588.1	83	95.9005	65	54	hypothetical protein
Locus_10831_Transcript_2/2_Conf_1.000	513	10	2.29E-52	CAR63588.1	83	208.379	136	113	hypothetical protein
Locus_10832_Transcript_1/1_Conf_1.000	448	6	9.61E-13	EFO28040.1	69	76.6406	71	49	sema domain-containing protein
Locus_10833_Transcript_1/1_Conf_1.000	202	0							
Locus_10834_Transcript_1/1_Conf_1.000	561	20	2.43E-86	P29190.1	92	321.627	173	160	Phosphoenolpyruvate carboxylase
Locus_10835_Transcript_1/1_Conf_1.000	738	20	2.43E-50	NP_509589.1	79	202.986	147	117	hypothetical protein ZK899.2
Locus_10836_Transcript_1/1_Conf_1.000	848	0							
Locus_10837_Transcript_1/1_Conf_1.000	414	20	5.87E-39	AAS99337.1	94	163.696	97	92	putative calmodulin isoform a
Locus_10838_Transcript_1/1_Conf_1.000	688	0							
Locus_10839_Transcript_1/1_Conf_1.000	226	2	2.31E-06	NP_871818.1	81	55.4546	38	31	FMRF-Like Peptide family member (flp-33)
Locus_1084_Transcript_1/1_Conf_1.000	415	0							
Locus_10840_Transcript_1/1_Conf_1.000	311	5	1.77E-27	NP_872143.2	92	125.561	76	70	TropoMoDulin family member (tmd-2)
Locus_10841_Transcript_1/1_Conf_1.000	534	2	7.90E-09	XP_001893258.1	58	63.929	103	60	hypothetical protein Bm1_08935

Locus_10842_Transcript_1/2_Conf_1.000	1371	20	8.86E-87	NP_510462.2	59	325.479	465	278	EGR (Early Growth factor Response factor) Homolog family member (egrh-1)
Locus_10842_Transcript_2/2_Conf_1.000	1493	20	1.38E-88	NP_510462.2	59	331.643	471	282	EGR (Early Growth factor Response factor) Homolog family member (egrh-1)
Locus_10843_Transcript_1/1_Conf_1.000	342	3	1.26E-36	NP_495991.1	75	155.992	109	82	hypothetical protein F42A8.1
Locus_10844_Transcript_1/2_Conf_1.000	330	20	8.28E-41	NP_499203.1	82	169.859	108	89	TransThyretin-Related family domain family member (ttr-2)
Locus_10844_Transcript_2/2_Conf_1.000	330	20	8.28E-41	NP_499203.1	82	169.859	108	89	TransThyretin-Related family domain family member (ttr-2)
Locus_10845_Transcript_1/2_Conf_1.000	1363	2	1.33E-26	EFO27879.1	46	125.561	471	219	hypothetical protein LOAG_00601
Locus_10845_Transcript_2/2_Conf_1.000	1312	2	1.18E-24	EFO27879.1	46	119.013	452	208	hypothetical protein LOAG_00601
Locus_10846_Transcript_1/1_Conf_1.000	182	20	9.16E-19	XP_001898349.1	86	96.6709	60	52	calpain D
Locus_10847_Transcript_1/1_Conf_1.000	1188	20	1.44E-127	XP_001899692.1	81	460.685	382	310	WW domain containing protein

Locus_10848_Transcript_1/1_Conf_1.000	818	20	2.78E-109	NP_491881.2	90	399.053	232	209	NADH Ubiquinone Oxidoreductase family member (nuo-2)
Locus_10849_Transcript_1/1_Conf_1.000	319	20	8.74E-22	EFO20229.1	71	106.686	106	76	hypothetical protein LOAG_08261
Locus_1085_Transcript_1/4_Conf_0.667	1807	20	0	NP_502997.1	85	669.078	431	367	Methionine AminoPeptidase family member (map-2)
Locus_1085_Transcript_2/4_Conf_0.667	1807	20	0	NP_502997.1	85	669.078	431	367	Methionine AminoPeptidase family member (map-2)
Locus_1085_Transcript_3/4_Conf_0.667	1561	20	0	NP_502997.1	85	669.078	431	367	Methionine AminoPeptidase family member (map-2)

Locus_1085_Transcript_4/4_Conf_0.667	1807	20	0	NP_502997.1	85	669.078	431	367	Methionine AminoPeptidase family member (map-2)
Locus_10850_Transcript_1/1_Conf_1.000	321	0							
Locus_10851_Transcript_1/1_Conf_1.000	907	20	1.01E-113	NP_497751.1	78	414.075	304	239	hypothetical protein C14B1.10
Locus_10852_Transcript_1/1_Conf_1.000	371	0							
Locus_10853_Transcript_1/1_Conf_1.000	434	1	1.63E-04	XP_002642900.1	60	49.2914	78	47	Hypothetical protein CBG15174
Locus_10854_Transcript_1/1_Conf_1.000	177	0							
Locus_10855_Transcript_1/1_Conf_1.000	805	8	1.69E-34	NP_497963.1	57	150.599	241	139	Suppressor/Enhancer of Lin-12 family member (sel-13)
Locus_10856_Transcript_1/1_Conf_1.000	267	7	8.93E-27	AAL77521.1	87	123.25	71	62	L80003_1guanylyl cyclase
Locus_10857_Transcript_1/1_Conf_1.000	145	0							
Locus_10858_Transcript_1/1_Conf_1.000	1455	0							
Locus_10859_Transcript_1/1_Conf_1.000	329	20	3.27E-37	XP_002636129.1	84	157.918	108	91	C. briggsae CBR-ATGR-9 protein
Locus_1086_Transcript_1/1_Conf_1.000	1090	4	2.38E-09	EFO19781.1	47	67.781	224	107	hypothetical protein LOAG_08708
Locus_10860_Transcript_1/1_Conf_1.000	329	20	5.56E-45	XP_002637938.1	88	183.726	109	96	Hypothetical protein CBG04750
Locus_10861_Transcript_1/2_Conf_1.000	451	0							
Locus_10861_Transcript_2/2_Conf_1.000	460	0							

Locus_10862_Transcript_1/1_Conf_1.000	554	20	2.63E-53	AAP36737.1	100	211.846	105	105	Homo sapiens S100 calcium binding protein A11 (calgizzarin)
Locus_10863_Transcript_1/1_Conf_1.000	219	0							
Locus_10864_Transcript_1/1_Conf_1.000	401	0							
Locus_10865_Transcript_1/2_Conf_1.000	458	2	6.16E-04	XP_001592033.1	54	47.3654	104	57	hypothetical protein SS1G_07480
Locus_10865_Transcript_2/2_Conf_1.000	485	2	6.29E-04	XP_001592033.1	54	47.3654	104	57	hypothetical protein SS1G_07480
Locus_10866_Transcript_1/1_Conf_1.000	915	2	2.79E-10	XP_002631368.1	59	70.4774	147	88	Hypothetical protein CBG03210
Locus_10867_Transcript_1/1_Conf_1.000	873	20	1.53E-87	NP_001023891.2	75	327.02	291	219	HIF (hypoxia inducible factor) homolog family member (hif-1)
Locus_10868_Transcript_1/1_Conf_1.000	248	0							
Locus_10869_Transcript_1/1_Conf_1.000	264	20	8.11E-28	XP_002632407.1	82	126.716	88	73	Hypothetical protein CBG00431
Locus_1087_Transcript_1/1_Conf_1.000	375	20	3.50E-55	NP_502165.2	93	217.624	125	117	hypothetical protein C10C6.6
Locus_10870_Transcript_1/1_Conf_1.000	438	10	3.52E-76	NP_509848.2	97	287.345	143	139	hypothetical protein C09F12.2
Locus_10871_Transcript_1/1_Conf_1.000	409	7	2.57E-42	ABM91438.1	80	174.866	120	97	defective mitochondrial respiration family member protein 1

Locus_10872_Transcript_1/1_Conf_1.000	481	20	4.49E-39	NP_491212.1	67	164.081	154	104	INneXin family member (inx-13)
Locus_10873_Transcript_1/1_Conf_1.000	623	0							
Locus_10874_Transcript_1/1_Conf_1.000	960	20	5.97E-59	XP_001894229.1	61	232.261	326	199	Variant SH3 domain containing protein
Locus_10875_Transcript_1/1_Conf_1.000	587	20	3.20E-58	AAV28323.2	72	228.409	187	136	Hypothetical protein T15B7.1
Locus_10876_Transcript_1/1_Conf_1.000	228	0							
Locus_10877_Transcript_1/2_Conf_1.000	791	5	1.87E-22	XP_002639505.1	67	110.538	167	112	Hypothetical protein CBG04107
Locus_10877_Transcript_2/2_Conf_1.000	789	5	1.87E-22	XP_002639505.1	67	110.538	167	112	Hypothetical protein CBG04107
Locus_10878_Transcript_1/1_Conf_1.000	500	13	4.06E-56	XP_002632942.1	69	220.705	166	116	Hypothetical protein CBG21698
Locus_10879_Transcript_1/1_Conf_1.000	157	0							
Locus_1088_Transcript_1/1_Conf_1.000	140	0							
Locus_10880_Transcript_1/1_Conf_1.000	145	0							
Locus_10881_Transcript_1/5_Conf_0.636	1386	20	6.09E-51	CBG22751.1	63	206.453	206	130	C. elegans protein Y59A8B.13b, partially confirmed by transcript evidence
Locus_10881_Transcript_2/5_Conf_0.636	1213	20	8.59E-51	EFO25339.1	54	205.682	293	161	zinc finger protein
Locus_10881_Transcript_3/5_Conf_0.636	1358	20	1.01E-50	EFO25339.1	54	205.682	293	161	zinc finger protein
Locus_10881_Transcript_4/5_Conf_0.636	1386	20	7.96E-51	EFO25339.1	55	206.068	293	162	zinc finger protein
Locus_10881_Transcript_5/5_Conf_0.636	1386	20	1.04E-50	EFO25339.1	54	205.682	293	161	zinc finger protein
Locus_10882_Transcript_1/1_Conf_1.000	434	0							
Locus_10883_Transcript_1/1_Conf_1.000	175	0							
Locus_10884_Transcript_1/1_Conf_1.000	333	0							
Locus_10885_Transcript_1/1_Conf_1.000	444	20	4.29E-45	NP_499344.2	77	184.111	147	114	hypothetical protein Y39A1A.9
Locus_10886_Transcript_1/1_Conf_1.000	331	0							
Locus_10887_Transcript_1/1_Conf_1.000	284	2	2.18E-04	XP_002642364.1	70	48.9062	47	33	Hypothetical protein CBG18362

Locus_10888_Transcript_1/1_Conf_1.000	203	20	1.38E-27	EDM06125.1	100	125.946	62	62	rCG35185, isoform CRA_c
Locus_10889_Transcript_1/1_Conf_1.000	371	20	1.82E-27	XP_002637830.1	81	125.561	88	72	C. briggsae CBR-DHS-22 protein
Locus_1089_Transcript_1/1_Conf_1.000	1208	20	1.64E-86	XP_002648569.1	68	324.324	330	227	Hypothetical protein CBG24890
Locus_10890_Transcript_1/1_Conf_1.000	272	0							
Locus_10891_Transcript_1/1_Conf_1.000	621	4	3.81E-31	XP_002640017.1	54	138.658	217	119	Hypothetical protein CBG12487
Locus_10892_Transcript_1/2_Conf_1.000	1303	20	3.27E-43	XP_002633896.1	63	180.644	346	221	Hypothetical protein CBG19958
Locus_10892_Transcript_2/2_Conf_1.000	1303	20	2.50E-43	XP_002633896.1	63	181.03	346	221	Hypothetical protein CBG19958
Locus_10893_Transcript_1/1_Conf_1.000	295	20	5.83E-18	XP_002646426.1	78	93.9745	74	58	C. briggsae CBR-EYA-1 protein
Locus_10894_Transcript_1/2_Conf_1.000	271	20	8.09E-12	XP_002598195.1	62	73.559	83	52	hypothetical protein BRAFLDRAFT_261117
Locus_10894_Transcript_2/2_Conf_1.000	260	20	8.20E-12	ZP_01160100.1	64	73.559	76	49	Alkaline phosphatase
Locus_10895_Transcript_1/1_Conf_1.000	312	20	1.11E-24	XP_002647071.1	86	116.316	82	71	C. briggsae CBR-NIT-1 protein
Locus_10896_Transcript_1/1_Conf_1.000	921	20	1.19E-101	NP_496800.1	76	374.015	306	234	hypothetical protein W09H1.5
Locus_10897_Transcript_1/1_Conf_1.000	295	0							
Locus_10898_Transcript_1/1_Conf_1.000	548	20	6.74E-22	CAD21647.3	60	107.457	153	93	C. elegans protein T03F6.6, confirmed by transcript evidence
Locus_10899_Transcript_1/1_Conf_1.000	396	20	3.53E-39	XP_002647861.1	84	164.466	107	90	Hypothetical protein CBG23659
Locus_109_Transcript_1/8_Conf_0.100	106	7	1.16E-05	NP_501859.1	89	53.1434	37	33	RACK1 (mammalian Receptor of Activated C Kinase) homolog family member (rack-1)

Locus_109_Transcript_2/8_Conf_0.100	148	20	3.04E-06	NP_501859.1	85	55.0694	41	35	RACK1 (mammalian Receptor of Activated C Kinase) homolog family member (rack-1)
Locus_109_Transcript_3/8_Conf_0.450	837	20	1.85E-71	XP_002633718.1	97	273.478	134	131	C. briggsae CBR-RACK-1 protein
Locus_109_Transcript_4/8_Conf_0.450	837	20	6.37E-72	XP_002633718.1	95	275.018	138	132	C. briggsae CBR-RACK-1 protein
Locus_109_Transcript_5/8_Conf_0.100	283	20	1.78E-14	NP_501859.1	92	82.4185	54	50	RACK1 (mammalian Receptor of Activated C Kinase) homolog family member (rack-1)

Locus_109_Transcript_6/8_Conf_0.100	283	20	1.78E-14	NP_501859.1	92	82.4185	54	50	RACK1 (mammalian Receptor of Activated C Kinase) homolog family member (rack-1)
Locus_109_Transcript_7/8_Conf_0.300	842	20	4.35E-145	XP_002633718.1	93	518.079	280	263	C. briggsae CBR-RACK-1 protein
Locus_109_Transcript_8/8_Conf_0.350	948	20	1.85E-145	XP_002633718.1	92	519.62	284	264	C. briggsae CBR-RACK-1 protein
Locus_1090_Transcript_1/1_Conf_1.000	824	20	7.76E-75	CAM84573.1	65	284.648	302	198	intestinal prolyl carboxypeptidase 1
Locus_10900_Transcript_1/3_Conf_0.714	447	4	3.09E-11	NP_505417.1	60	71.633	121	73	hypothetical protein K07B1.6
Locus_10900_Transcript_2/3_Conf_0.429	322	0							
Locus_10900_Transcript_3/3_Conf_0.714	322	0							
Locus_10901_Transcript_1/1_Conf_1.000	224	0							
Locus_10902_Transcript_1/1_Conf_1.000	995	20	1.49E-100	XP_002636180.1	75	370.548	326	245	Hypothetical protein CBG01437
Locus_10903_Transcript_1/1_Conf_1.000	150	20	3.69E-20	XP_002914026.1	100	101.293	49	49	PREDICTED: polyadenylate-binding protein 1-like
Locus_10904_Transcript_1/1_Conf_1.000	1255	20	6.83E-67	XP_002640612.1	68	259.225	314	214	Hypothetical protein CBG08723

Locus_10905_Transcript_1/1_Conf_1.000	482	0							
Locus_10906_Transcript_1/1_Conf_1.000	1876	20	6.50E-134	XP_002645956.1	72	482.641	431	311	C. briggsae CBR-ENT-2 protein
Locus_10907_Transcript_1/1_Conf_1.000	423	3	1.88E-08	NP_495495.1	59	62.3882	83	49	hypothetical protein E04F6.6
Locus_10908_Transcript_1/1_Conf_1.000	1041	0							
Locus_10909_Transcript_1/1_Conf_1.000	1410	20	2.96E-69	CAA10033.1	65	267.314	369	240	DYS-1 protein
Locus_1091_Transcript_1/1_Conf_1.000	410	20	1.99E-10	EFO19957.1	60	68.9366	115	69	hypothetical protein LOAG_08536
Locus_10910_Transcript_1/1_Conf_1.000	730	20	9.18E-95	XP_002638788.1	88	350.517	205	181	Hypothetical protein CBG05143
Locus_10911_Transcript_1/1_Conf_1.000	706	20	3.86E-55	NP_491763.1	66	218.779	235	156	hypothetical protein T27A3.6
Locus_10912_Transcript_1/1_Conf_1.000	731	0							
Locus_10913_Transcript_1/1_Conf_1.000	691	0							
Locus_10914_Transcript_1/1_Conf_1.000	996	20	2.80E-14	XP_001463473.1	50	83.9593	106	53	hypothetical protein
Locus_10915_Transcript_1/1_Conf_1.000	417	0							
Locus_10916_Transcript_1/1_Conf_1.000	316	20	4.66E-15	EFO23801.1	62	84.3445	105	66	replication factor C
Locus_10917_Transcript_1/1_Conf_1.000	568	20	7.38E-62	NP_872022.1	76	240.35	188	143	CysTiNoSin (lysosomal protein) homolog family member (ctns-1)
Locus_10918_Transcript_1/1_Conf_1.000	559	2	2.35E-25	NP_498100.1	65	119.013	124	81	Poly(U) Polymerase family member (pup-2)
Locus_10919_Transcript_1/1_Conf_1.000	813	0							
Locus_1092_Transcript_1/1_Conf_1.000	2516	20	0	NP_001022033.1	86	1185.24	715	620	Threonyl tRNA Synthetase family member (trs-1)
Locus_10920_Transcript_1/1_Conf_1.000	156	0							
Locus_10921_Transcript_1/1_Conf_1.000	752	20	5.25E-48	ACI48997.1	67	195.282	233	157	hypothetical protein Cbre_JD01.004
Locus_10922_Transcript_1/1_Conf_1.000	1450	20	1.88E-151	NP_502613.2	73	540.421	489	359	QUInine non-avoider family member (qui-1)
Locus_10923_Transcript_1/1_Conf_1.000	638	3	1.55E-22	XP_002634007.1	72	110.153	92	67	Hypothetical protein CBG01539

Locus_10924_Transcript_1/1_Conf_1.000	603	20	1.31E-49	NP_501913.1	72	199.904	159	115	hypothetical protein R11A8.5
Locus_10925_Transcript_1/1_Conf_1.000	543	20	6.12E-60	XP_002639515.1	77	233.802	179	138	Hypothetical protein CBG04121
Locus_10926_Transcript_1/1_Conf_1.000	447	0							
Locus_10927_Transcript_1/1_Conf_1.000	278	3	2.12E-04	NP_490888.1	73	48.9062	41	30	Mitochondrial Processing Peptidase Alpha family member (mppa-1)
Locus_10928_Transcript_1/1_Conf_1.000	929	20	2.56E-43	NP_491522.1	56	180.259	308	174	phosphoinositide kinase AdAPter subunit family member (aap-1)
Locus_10929_Transcript_1/1_Conf_1.000	505	0							
Locus_1093_Transcript_1/1_Conf_1.000	785	20	5.45E-75	NP_671477.1	99	285.034	136	135	rRNA promoter binding protein
Locus_10930_Transcript_1/1_Conf_1.000	157	0							
Locus_10931_Transcript_1/1_Conf_1.000	1156	20	7.26E-145	XP_002638763.1	82	518.079	388	322	C. briggsae CBR-BLI-3 protein
Locus_10932_Transcript_1/1_Conf_1.000	163	0							
Locus_10933_Transcript_1/2_Conf_1.000	255	0							
Locus_10933_Transcript_2/2_Conf_1.000	209	0							
Locus_10934_Transcript_1/1_Conf_1.000	358	0							
Locus_10935_Transcript_1/1_Conf_1.000	554	20	1.51E-24	NP_500522.1	66	116.316	130	86	TransThyretin-Related family domain family member (ttr-20)
Locus_10936_Transcript_1/1_Conf_1.000	428	1	5.09E-14	NP_502212.1	63	80.8777	111	70	hypothetical protein ZK792.5
Locus_10937_Transcript_1/1_Conf_1.000	340	0							
Locus_10938_Transcript_1/1_Conf_1.000	784	20	5.50E-43	EFO19864.1	79	178.718	139	111	hypothetical protein LOAG_08632

Locus_10939_Transcript_1/1_Conf_1.000	411	20	2.91E-54	NP_001022641.1	83	214.542	136	113	LEThal family member (let-805)
Locus_1094_Transcript_1/1_Conf_1.000	533	20	4.89E-67	NP_500005.1	89	257.299	156	139	MAP kinase Activated protein Kinase family member (mak-2)
Locus_10940_Transcript_1/1_Conf_1.000	139	0							
Locus_10941_Transcript_1/1_Conf_1.000	181	2	1.68E-04	XP_001949404.1	71	49.2914	39	28	PREDICTED: similar to GA19830-PA
Locus_10942_Transcript_1/1_Conf_1.000	320	20	1.71E-17	ADI61822.1	61	92.4337	93	57	endonuclease-reverse transcriptase
Locus_10943_Transcript_1/1_Conf_1.000	1180	20	4.76E-99	XP_002642360.1	89	365.925	214	192	C. briggsae CBR-RAB-30 protein
Locus_10944_Transcript_1/1_Conf_1.000	835	0							
Locus_10945_Transcript_1/1_Conf_1.000	432	20	4.09E-48	2WS2	90	194.126	116	105	The 2 AngstromStructure Of A Nu-Class Gst From Haemonchus Contortus
Locus_10946_Transcript_1/1_Conf_1.000	281	0							
Locus_10947_Transcript_1/1_Conf_1.000	316	20	1.49E-29	NP_741839.2	69	132.494	105	73	hypothetical protein F48E3.8
Locus_10948_Transcript_1/1_Conf_1.000	134	0							
Locus_10949_Transcript_1/2_Conf_1.000	442	20	5.13E-22	AAO63576.1	56	107.457	150	85	secreted protein 4 precursor
Locus_10949_Transcript_2/2_Conf_1.000	442	20	6.69E-22	AAO63576.1	56	107.071	150	85	secreted protein 4 precursor
Locus_1095_Transcript_1/1_Conf_1.000	383	7	1.01E-38	XP_002639089.1	74	162.925	134	100	C. briggsae CBR-TAG-163 protein
Locus_10950_Transcript_1/2_Conf_1.000	671	20	2.58E-34	EFO27619.1	62	149.443	216	134	hypothetical protein LOAG_00870
Locus_10950_Transcript_2/2_Conf_1.000	487	6	1.10E-16	EFO27619.1	52	89.7373	155	81	hypothetical protein LOAG_00870
Locus_10951_Transcript_1/1_Conf_1.000	458	0							

Locus_10952_Transcript_1/1_Conf_1.000	320	20	1.82E-27	XP_513188.2	63	125.561	106	67	PREDICTED: complement component 1, q subcomponent, gamma polypeptide
Locus_10953_Transcript_1/1_Conf_1.000	1854	20	0	XP_002644381.1	86	868.226	578	498	C. briggsae CBR-SAX-3 protein
Locus_10954_Transcript_1/1_Conf_1.000	864	20	2.49E-66	XP_002640643.1	68	256.529	251	171	C. briggsae CBR-TAG-271 protein
Locus_10955_Transcript_1/4_Conf_0.429	538	20	7.17E-66	XP_508190.1	99	253.447	126	125	PREDICTED: interferon induced transmembrane protein 2 (1-8D) isoform 9
Locus_10955_Transcript_2/4_Conf_0.571	657	20	3.03E-64	AAX37033.1	99	248.825	125	124	interferon induced transmembrane protein 1
Locus_10955_Transcript_3/4_Conf_0.571	563	20	1.30E-63	AAP36548.1	100	246.128	133	133	Homo sapiens interferon induced transmembrane protein 3 (1-8U)
Locus_10955_Transcript_4/4_Conf_0.571	603	20	3.09E-75	XP_508190.1	97	285.034	146	142	PREDICTED: interferon induced transmembrane protein 2 (1-8D) isoform 9
Locus_10956_Transcript_1/1_Conf_1.000	908	20	3.74E-84	NP_495106.1	70	315.849	301	213	FUcosyl Transferase family member (fut-3)
Locus_10957_Transcript_1/1_Conf_1.000	290	20	3.69E-12	XP_002639821.1	61	74.7146	76	47	C. briggsae CBR-ERS-2 protein

Locus_10958_Transcript_1/2_Conf_1.000	314	20	7.70E-26	XP_002640523.1	82	120.168	81	67	Hypothetical protein CBG18685
Locus_10958_Transcript_2/2_Conf_1.000	385	20	7.51E-26	XP_002640523.1	82	120.168	81	67	Hypothetical protein CBG18685
Locus_10959_Transcript_1/1_Conf_1.000	311	20	1.18E-39	NP_001040812.1	85	166.007	103	88	hypothetical protein T27A1.5
Locus_1096_Transcript_1/1_Conf_1.000	1771	20	3.88E-40	NP_490728.2	74	171.014	213	158	TAF (TBP-associated transcription factor) family member (taf-4)
Locus_10960_Transcript_1/1_Conf_1.000	588	20	2.99E-40	XP_001898597.1	64	168.703	184	118	Steroid receptor seven-up type 2
Locus_10961_Transcript_1/1_Conf_1.000	665	0							
Locus_10962_Transcript_1/1_Conf_1.000	165	6	3.65E-04	NP_001020987.1	81	48.1358	38	31	UNCoordinated family member (unc-89)
Locus_10963_Transcript_1/1_Conf_1.000	323	12	1.53E-26	NP_001021703.1	87	122.479	107	94	EXOCyst component family member (exoc-8)
Locus_10964_Transcript_1/1_Conf_1.000	298	7	8.68E-14	NP_491337.1	70	80.1073	74	52	EXOCyst component family member (exoc-7)
Locus_10965_Transcript_1/1_Conf_1.000	240	0							

Locus_10966_Transcript_1/1_Conf_1.000	218	20	7.94E-28	XP_002929945.1	100	126.716	63	63	PREDICTED: endothelial differentiation-related factor 1-like
Locus_10967_Transcript_1/1_Conf_1.000	250	18	1.83E-24	XP_002636962.1	84	115.546	82	69	C. briggsae CBR-MAX-1 protein
Locus_10968_Transcript_1/1_Conf_1.000	179	20	1.27E-25	XP_002723429.1	100	119.398	59	59	PREDICTED: non-POU domain containing, octamer-binding-like
Locus_10969_Transcript_1/1_Conf_1.000	178	0							
Locus_1097_Transcript_1/1_Conf_1.000	1110	20	2.82E-114	CAT00983.1	80	416.387	336	271	C. elegans protein C34C6.2c, partially confirmed by transcript evidence
Locus_10970_Transcript_1/1_Conf_1.000	884	20	7.45E-66	EFO20497.1	64	254.988	297	191	serologically defined colon cancer antigen 1
Locus_10971_Transcript_1/1_Conf_1.000	179	20	3.58E-20	ACF22787.1	98	101.293	58	57	MDR-like ABC transporter
Locus_10972_Transcript_1/1_Conf_1.000	996	20	7.16E-111	NP_490929.1	95	404.831	252	240	mitochondrial ProHiBitin complex family member (phb-1)
Locus_10973_Transcript_1/1_Conf_1.000	528	0							
Locus_10974_Transcript_1/1_Conf_1.000	260	11	6.68E-30	XP_002631379.1	91	133.65	73	67	Hypothetical protein CBG03221
Locus_10975_Transcript_1/1_Conf_1.000	426	20	2.87E-33	NP_001129936.1	80	144.821	105	84	SMAll family member (sma-9)
Locus_10976_Transcript_1/1_Conf_1.000	132	3	2.59E-18	XP_002830627.1	93	95.1301	44	41	PREDICTED: hypothetical protein LOC100438967
Locus_10977_Transcript_1/1_Conf_1.000	482	4	5.71E-18	NP_503722.1	63	93.9745	105	67	hypothetical protein W02H5.2

Locus_10978_Transcript_1/1_Conf_1.000	694	20	1.79E-89	XP_002633161.1	77	332.798	231	179	C. briggsae CBR-TAG-224 protein
Locus_10979_Transcript_1/1_Conf_1.000	214	0							
Locus_1098_Transcript_1/2_Conf_1.000	1905	20	2.02E-98	NP_496095.1	63	364.77	401	256	WEE homolog family member (wee-1.3)
Locus_1098_Transcript_2/2_Conf_1.000	1905	20	2.02E-98	NP_496095.1	63	364.77	401	256	WEE homolog family member (wee-1.3)
Locus_10980_Transcript_1/1_Conf_1.000	247	0							
Locus_10981_Transcript_1/1_Conf_1.000	488	20	9.62E-21	NP_001040707.1	54	103.219	160	87	CDT (S. pombe CDC10 Dependent Transcript) homolog family member (cdt-1)
Locus_10982_Transcript_1/2_Conf_1.000	466	20	1.18E-10	XP_002641354.1	72	69.707	51	37	Hypothetical protein CBG13208
Locus_10982_Transcript_2/2_Conf_1.000	481	20	1.15E-10	XP_002641354.1	72	69.707	51	37	Hypothetical protein CBG13208
Locus_10983_Transcript_1/1_Conf_1.000	291	2	5.68E-05	NP_506635.1	55	50.8322	72	40	hypothetical protein Y50E8A.1
Locus_10984_Transcript_1/2_Conf_1.000	254	20	1.46E-08	NP_663214.1	70	62.7734	67	47	viral ubiquitin
Locus_10984_Transcript_2/2_Conf_1.000	204	20	2.52E-05	XP_321091.3	73	51.9878	46	34	AGAP001970-PA
Locus_10985_Transcript_1/1_Conf_1.000	680	20	4.15E-112	NP_492779.1	93	407.912	224	210	hypothetical protein B0511.6
Locus_10986_Transcript_1/1_Conf_1.000	612	2	5.99E-05	NP_035608.1	45	51.6026	122	55	small proline-rich protein 3
Locus_10987_Transcript_1/1_Conf_1.000	138	20	3.59E-20	XP_002928409.1	100	101.293	45	45	PREDICTED: heterogeneous nuclear ribonucleoprotein M-like

Locus_10988_Transcript_1/1_Conf_1.000	866	20	4.57E-44	EFO19733.1	60	182.57	243	148	hypothetical protein LOAG_08758
Locus_10989_Transcript_1/1_Conf_1.000	1446	20	1.50E-124	NP_501147.2	82	451.055	322	267	fatty acid ELOngation family member (elo-3)
Locus_1099_Transcript_1/2_Conf_1.000	833	20	1.14E-81	XP_002640453.1	93	307.375	186	173	C. briggsae CBR-RPL-17 protein
Locus_1099_Transcript_2/2_Conf_1.000	492	20	5.56E-37	NP_740781.1	89	157.147	91	81	Ribosomal Protein, Large subunit family member (rpl-17)
Locus_10990_Transcript_1/1_Conf_1.000	1051	20	1.23E-124	XP_002630800.1	84	450.669	268	227	Hypothetical protein CBG02500
Locus_10991_Transcript_1/1_Conf_1.000	331	1	1.63E-04	XP_001902364.1	60	49.2914	112	68	Myotubularin-related family protein
Locus_10992_Transcript_1/1_Conf_1.000	138	0							
Locus_10993_Transcript_1/1_Conf_1.000	1141	0							
Locus_10994_Transcript_1/1_Conf_1.000	479	0							
Locus_10995_Transcript_1/1_Conf_1.000	387	20	8.56E-22	EFO22859.1	96	106.686	61	59	hypothetical protein LOAG_05624
Locus_10996_Transcript_1/1_Conf_1.000	232	0							
Locus_10997_Transcript_1/2_Conf_1.000	182	0							
Locus_10997_Transcript_2/2_Conf_1.000	763	4	5.39E-32	NP_001122673.1	72	142.124	195	141	hypothetical protein B0353.1
Locus_10998_Transcript_1/1_Conf_1.000	406	4	1.09E-16	NP_001021652.1	57	89.7373	142	81	FANCI (Fanconi anemia complex component I) homolog family member (fnci-1)
Locus_10999_Transcript_1/1_Conf_1.000	464	20	2.69E-55	XP_002643394.1	84	218.009	148	125	Hypothetical protein CBG16008

Locus_11_Transcript_10/14_Conf_0.176	364	12	4.78E-09	NP_001023275.1	75	50.8322	45	34	VITellogenin structural genes (yolk protein genes) family member (vit-6)
Locus_11_Transcript_11/14_Conf_0.147	276	8	3.86E-06	NP_001023275.1	71	54.6842	46	33	VITellogenin structural genes (yolk protein genes) family member (vit-6)
Locus_11_Transcript_12/14_Conf_0.147	276	8	3.86E-06	NP_001023275.1	71	54.6842	46	33	VITellogenin structural genes (yolk protein genes) family member (vit-6)
Locus_11_Transcript_13/14_Conf_0.176	364	8	3.70E-07	Q94637.1	71	52.373	49	35	Vitellogenin-6
Locus_11_Transcript_14/14_Conf_0.176	364	20	4.36E-15	Q94637.1	76	68.5514	52	40	Vitellogenin-6
Locus_11_Transcript_5/14_Conf_0.088	407	20	4.57E-23	EFO25665.1	92	110.923	63	58	ribosomal protein S13
Locus_11_Transcript_6/14_Conf_0.059	354	20	4.58E-23	EFO25665.1	92	110.923	63	58	ribosomal protein S13
Locus_11_Transcript_7/14_Conf_0.176	364	20	1.11E-16	Q94637.1	78	73.9442	56	44	Vitellogenin-6

Locus_11_Transcript_8/14_Conf_0.088	448	20	6.38E-41	EFO25665.1	95	170.244	91	87	ribosomal protein S13
Locus_11_Transcript_9/14_Conf_0.088	448	20	6.38E-41	EFO25665.1	95	170.244	91	87	ribosomal protein S13
Locus_110_Transcript_1/3_Conf_0.833	457	20	1.56E-15	ADK91433.1	71	85.8853	73	52	c-type lectin 1
Locus_110_Transcript_2/3_Conf_0.722	452	20	1.58E-15	ADK91433.1	71	85.8853	73	52	c-type lectin 1
Locus_110_Transcript_3/3_Conf_0.889	394	20	1.60E-15	ADK91433.1	71	85.8853	73	52	c-type lectin 1

Locus_1100_Transcript_1/1_Conf_1.000	742	20	1.18E-44	XP_002644771.1	80	184.111	129	104	C. briggsae CBR-MIG-23 protein
Locus_11000_Transcript_1/1_Conf_1.000	752	20	1.68E-70	NP_001023373.1	75	270.011	240	180	Histidyl tRNA Synthetase family member (hrs-1)
Locus_11001_Transcript_1/1_Conf_1.000	481	20	2.93E-22	XP_001898035.1	56	108.227	176	99	hypothetical protein Bm1_32955
Locus_11002_Transcript_1/1_Conf_1.000	410	20	2.94E-38	XP_002643586.1	72	161.384	133	97	C. briggsae CBR-SYM-4 protein
Locus_11003_Transcript_1/1_Conf_1.000	284	6	1.56E-10	EFO21753.1	57	69.3218	97	56	hypothetical protein LOAG_06732
Locus_11004_Transcript_1/1_Conf_1.000	1122	20	1.72E-34	XP_002639508.1	52	151.369	382	199	Hypothetical protein CBG04113
Locus_11005_Transcript_1/1_Conf_1.000	156	0							
Locus_11006_Transcript_1/1_Conf_1.000	1610	20	6.01E-69	Q61CW2.3	62	266.544	361	225	Serine/threonine-protein kinase smg-1
Locus_11007_Transcript_1/1_Conf_1.000	421	20	1.82E-19	NP_502282.1	77	98.9821	85	66	hypothetical protein T12G3.4
Locus_11008_Transcript_1/1_Conf_1.000	524	2	1.11E-04	XP_422062.2	46	50.0618	102	47	PREDICTED: similar to mKIAA0236 protein
Locus_11009_Transcript_1/1_Conf_1.000	342	16	4.97E-25	XP_002646276.1	82	117.472	74	61	C. briggsae CBR-ABF-2 protein
Locus_1101_Transcript_1/10_Conf_0.250	306	20	3.70E-41	CAI17777.2	100	171.014	101	101	major histocompatibility complex, class I, A
Locus_1101_Transcript_10/10_Conf_0.321	716	20	7.08E-69	XP_002746334.1	88	208.764	150	132	PREDICTED: CHLA class I histocompatibility antigen, A-2 alpha chain-like

Locus_1101_Transcript_2/10_Conf_0.250	575	20	9.48E-30	CAQ09232.1	100	100.908	73	73	major histocompatibility complex, class I, C
Locus_1101_Transcript_3/10_Conf_0.321	368	20	3.26E-67	CBW44123.1	94	182.57	91	86	MHC class I antigen
Locus_1101_Transcript_4/10_Conf_0.286	246	20	5.43E-42	CBW44123.1	100	98.5969	37	37	MHC class I antigen
Locus_1101_Transcript_5/10_Conf_0.321	779	20	2.94E-76	ABQ95441.1	92	238.424	166	153	MHC class I antigen
Locus_1101_Transcript_6/10_Conf_0.107	465	20	3.27E-61	CAA34947.1	99	237.654	138	137	leukocyte antigen F

Locus_1101_Transcript_7/10_Conf_0.393	716	20	1.41E-60	CBM41445.1	100	167.933	76	76	MHC class I antigen
Locus_1101_Transcript_8/10_Conf_0.250	246	20	2.45E-42	CBW44123.1	100	98.5969	37	37	MHC class I antigen
Locus_1101_Transcript_9/10_Conf_0.250	718	20	3.53E-77	CAM24816.1	100	214.157	118	118	major histocompatibility complex, class I, C
Locus_11010_Transcript_1/1_Conf_1.000	303	0							
Locus_11011_Transcript_1/1_Conf_1.000	654	20	7.31E-95	NP_502560.1	86	350.517	217	187	FATty acid desaturase family member (fat-2)
Locus_11012_Transcript_1/1_Conf_1.000	643	20	1.23E-83	XP_002630183.1	89	313.153	212	189	C. briggsae CBR-FLI-1 protein
Locus_11013_Transcript_1/1_Conf_1.000	291	2	1.03E-06	NP_506151.1	70	56.6102	51	36	hypothetical protein C14C10.4
Locus_11014_Transcript_1/1_Conf_1.000	403								
Locus_11015_Transcript_1/1_Conf_1.000	590	2	1.49E-31	NP_497506.3	60	139.813	194	118	hypothetical protein Y48G9A.3

Locus_11016_Transcript_1/1_Conf_1.000	412	20	2.82E-57	NP_741553.1	88	224.557	136	120	ALdehyde deHydrogenase family member (alh-4)
Locus_11017_Transcript_1/1_Conf_1.000	1942	20	6.58E-105	XP_002644943.1	57	386.341	627	362	Hypothetical protein CBG10898
Locus_11018_Transcript_1/1_Conf_1.000	377	7	2.29E-14	AAC61752.1	57	82.0333	121	69	nuclear hormone receptor
Locus_11019_Transcript_1/1_Conf_1.000	442	20	1.35E-30	NP_498057.1	66	135.961	141	94	hypothetical protein C27F2.10
Locus_1102_Transcript_1/1_Conf_1.000	2206	20	0	XP_002633545.1	76	638.647	693	531	Hypothetical protein CBG05413
Locus_11020_Transcript_1/1_Conf_1.000	427	2	1.29E-33	XP_002636965.1	78	145.976	106	83	Hypothetical protein CBG09443
Locus_11021_Transcript_1/1_Conf_1.000	576	20	4.29E-36	EFO19461.1	90	154.836	90	81	hypothetical protein LOAG_09031
Locus_11022_Transcript_1/1_Conf_1.000	627	1	4.81E-05	XP_002645117.1	52	51.9878	93	49	C. briggsae CBR-XPC-1 protein
Locus_11023_Transcript_1/1_Conf_1.000	371	0							
Locus_11024_Transcript_1/1_Conf_1.000	441	20	6.03E-39	NP_872248.2	72	163.696	138	100	hypothetical protein R57.1
Locus_11025_Transcript_1/1_Conf_1.000	154	0							
Locus_11026_Transcript_1/1_Conf_1.000	376	0							
Locus_11027_Transcript_1/1_Conf_1.000	560	20	2.11E-50	NP_495951.2	84	202.216	136	115	hypothetical protein W07A12.4
Locus_11028_Transcript_1/1_Conf_1.000	338	9	3.60E-12	AAN61521.1	72	74.7146	66	48	301KDa_2 protein
Locus_11029_Transcript_1/1_Conf_1.000	259	20	3.22E-16	EFO26239.1	67	88.1965	85	57	hypothetical protein LOAG_02251
Locus_1103_Transcript_1/1_Conf_1.000	386	0							
Locus_11030_Transcript_1/1_Conf_1.000	572	0							
Locus_11031_Transcript_1/2_Conf_1.000	184	0							
Locus_11031_Transcript_2/2_Conf_1.000	435	0							

Locus_11032_Transcript_1/1_Conf_1.000	516	20	5.62E-22	NP_495931.2	56	107.457	156	88	hypothetical protein T24B8.7
Locus_11033_Transcript_1/1_Conf_1.000	168	4	8.36E-09	XP_002633975.1	75	63.5438	45	34	Hypothetical protein CBG20077
Locus_11034_Transcript_1/1_Conf_1.000	329	0							
Locus_11035_Transcript_1/1_Conf_1.000	433	0							
Locus_11036_Transcript_1/1_Conf_1.000	309	0							
Locus_11037_Transcript_1/1_Conf_1.000	729	20	4.08E-18	EFO19311.1	72	95.9005	83	60	hypothetical protein LOAG_09182
Locus_11038_Transcript_1/3_Conf_0.667	611	0							
Locus_11038_Transcript_2/3_Conf_0.667	808	0							
Locus_11038_Transcript_3/3_Conf_0.667	799	0							
Locus_11039_Transcript_1/1_Conf_1.000	795	20	1.57E-101	XP_002643079.1	91	373.244	224	204	C. briggsae CBR-ABCE-1 protein
Locus_1104_Transcript_1/1_Conf_1.000	695	20	1.52E-72	XP_002647023.1	92	276.559	150	139	C. briggsae CBR-MLC-3 protein
Locus_11040_Transcript_1/1_Conf_1.000	274	0							
Locus_11041_Transcript_1/1_Conf_1.000	232	0							

Locus_11042_Transcript_1/1_Conf_1.000	1063	20	1.56E-58	XP_002635593.1	81	231.106	161	132	Hypothetical protein CBG20581
Locus_11043_Transcript_1/1_Conf_1.000	543	20	7.55E-26	NP_498946.1	76	120.553	106	81	hypothetical protein F54F2.7
Locus_11044_Transcript_1/1_Conf_1.000	282	0							
Locus_11045_Transcript_1/1_Conf_1.000	248	0							
Locus_11046_Transcript_1/2_Conf_1.000	981	20	3.94E-122	NP_500161.1	84	442.195	326	275	hypothetical protein R05C11.3
Locus_11046_Transcript_2/2_Conf_1.000	981	20	3.94E-122	NP_500161.1	84	442.195	326	275	hypothetical protein R05C11.3
Locus_11047_Transcript_1/1_Conf_1.000	159	0							
Locus_11048_Transcript_1/1_Conf_1.000	377	0							
Locus_11049_Transcript_1/2_Conf_1.000	395	20	1.83E-19	XP_002640352.1	61	98.9821	116	71	C. briggsae CBR-ARI-1 protein
Locus_11049_Transcript_2/2_Conf_1.000	461	20	4.99E-22	XP_002640352.1	68	107.457	105	72	C. briggsae CBR-ARI-1 protein
Locus_1105_Transcript_1/1_Conf_1.000	223	0							
Locus_11050_Transcript_1/1_Conf_1.000	624	20	2.47E-78	NP_001122513.1	90	295.434	172	156	UNCoordinated family member (unc-57)
Locus_11051_Transcript_1/1_Conf_1.000	289	20	2.54E-29	NP_490714.1	92	131.724	96	89	hypothetical protein C53D5.5
Locus_11052_Transcript_1/1_Conf_1.000	167	0							
Locus_11053_Transcript_1/1_Conf_1.000	829	0							
Locus_11054_Transcript_1/1_Conf_1.000	245	20	2.66E-23	NP_510625.2	75	111.694	79	60	hypothetical protein T27A8.1
Locus_11055_Transcript_1/1_Conf_1.000	644	20	2.27E-53	XP_001899300.1	71	212.616	212	152	Negative elongation factor B homolog
Locus_11056_Transcript_1/1_Conf_1.000	183	10	6.56E-09	EFO27462.1	63	63.929	61	39	hypothetical protein LOAG_01021
Locus_11057_Transcript_1/1_Conf_1.000	299	20	1.02E-46	XP_002750287.1	100	189.504	94	94	PREDICTED: elongation factor 1-beta-like
Locus_11058_Transcript_1/1_Conf_1.000	488	20	5.77E-58	XP_002639764.1	80	226.868	153	123	Hypothetical protein CBG02210

Locus_11059_Transcript_1/1_Conf_1.000	975	20	2.06E-22	EFO20991.1	54	110.923	205	112	hypothetical protein LOAG_07497
Locus_1106_Transcript_1/1_Conf_1.000	460	20	1.15E-18	CBH29662.1	72	96.2857	88	64	C. elegans protein F44G4.4c, confirmed by transcript evidence
Locus_11060_Transcript_1/1_Conf_1.000	293	20	7.64E-18	XP_002631109.1	94	93.5893	52	49	Hypothetical protein CBG02884
Locus_11061_Transcript_1/1_Conf_1.000	720	20	1.51E-17	CAA10734.1	83	93.9745	66	55	centaurin beta 1A
Locus_11062_Transcript_1/1_Conf_1.000	588	20	9.82E-84	EFO22295.1	91	313.153	180	165	hypothetical protein LOAG_06193
Locus_11063_Transcript_1/2_Conf_1.000	906	20	3.34E-16	XP_001891812.1	60	90.1225	108	65	hypothetical protein Bm1_01480
Locus_11063_Transcript_2/2_Conf_1.000	840	20	2.93E-16	XP_001891812.1	60	90.1225	108	65	hypothetical protein Bm1_01480
Locus_11064_Transcript_1/1_Conf_1.000	235	0							
Locus_11065_Transcript_1/1_Conf_1.000	389	4	5.00E-30	NP_497649.1	87	134.035	85	74	hypothetical protein Y55D5A.4
Locus_11066_Transcript_1/1_Conf_1.000	336	3	5.74E-10	CAR63610.1	55	67.3958	93	52	putative IQ calmodulin-binding motif domain protein

Locus_11067_Transcript_1/2_Conf_1.000	1594	20	4.50E-101	NP_497068.2	69	373.244	389	271	TBC (Tre-2/Bub2/Cdc16) domain family member (tbc-20)
Locus_11067_Transcript_2/2_Conf_1.000	1588	20	9.98E-101	NP_497068.2	71	372.089	370	263	TBC (Tre-2/Bub2/Cdc16) domain family member (tbc-20)
Locus_11068_Transcript_1/1_Conf_1.000	312	20	4.36E-29	CAR63568.1	89	130.954	83	74	putative Tubulin-specific chaperone A
Locus_11069_Transcript_1/1_Conf_1.000	219	0							
Locus_1107_Transcript_1/2_Conf_1.000	2145	20	0	NP_509671.3	83	827.78	597	500	hypothetical protein ZC506.1
Locus_1107_Transcript_2/2_Conf_1.000	2009	20	0	NP_509671.3	81	854.744	645	527	hypothetical protein ZC506.1
Locus_11070_Transcript_1/1_Conf_1.000	569	20	7.71E-27	XP_001896497.1	66	124.02	126	84	pold2-prov protein
Locus_11071_Transcript_1/2_Conf_1.000	1137	0							
Locus_11071_Transcript_2/2_Conf_1.000	1135	0							
Locus_11072_Transcript_1/1_Conf_1.000	972	20	9.41E-60	NP_500420.3	62	234.958	329	204	hypothetical protein M02B7.5
Locus_11073_Transcript_1/1_Conf_1.000	330	20	7.08E-08	XP_001301920.1	68	60.4622	60	41	ankyrin repeat protein
Locus_11074_Transcript_1/1_Conf_1.000	418	0							
Locus_11075_Transcript_1/1_Conf_1.000	236	20	5.24E-27	XP_002643972.1	88	124.02	78	69	C. briggsae CBR-SLO-2 protein

Locus_11076_Transcript_1/1_Conf_1.000	729	20	6.66E-53	XP_002630580.1	61	211.46	244	151	Hypothetical protein CBG13036
Locus_11077_Transcript_1/1_Conf_1.000	1623	1	1.93E-06	XP_002416346.1	45	58.9214	199	90	conserved hypothetical protein
Locus_11078_Transcript_1/1_Conf_1.000	130	0							
Locus_11079_Transcript_1/1_Conf_1.000	1000								
Locus_1108_Transcript_1/1_Conf_1.000	1100	20	1.77E-36	EFO17908.1	51	157.918	293	151	hypothetical protein LOAG_10591
Locus_11080_Transcript_1/1_Conf_1.000	460	20	1.26E-57	XP_002639234.1	85	225.713	149	128	Hypothetical protein CBG03790
Locus_11081_Transcript_1/1_Conf_1.000	428	0							
Locus_11082_Transcript_1/1_Conf_1.000	146	0							
Locus_11083_Transcript_1/1_Conf_1.000	433	1	7.30E-05	XP_002638904.1	43	50.447	116	50	Hypothetical protein CBG22130
Locus_11084_Transcript_1/1_Conf_1.000	920	20	5.64E-128	XP_001897894.1	84	461.455	313	265	ancient conserved domain protein 4
Locus_11085_Transcript_1/1_Conf_1.000	177	0							
Locus_11086_Transcript_1/1_Conf_1.000	356	0							
Locus_11087_Transcript_1/1_Conf_1.000	1777	20	2.46E-135	NP_500645.1	63	487.263	541	341	Egl-1 suppressor/DiO uptake defective/raf enhancer family member (eor-1)
Locus_11088_Transcript_1/1_Conf_1.000	679	20	1.51E-37	XP_002631497.1	76	160.229	143	110	Hypothetical protein CBG20661
Locus_11089_Transcript_1/1_Conf_1.000	199	0							
Locus_1109_Transcript_1/4_Conf_0.556	596	20	4.78E-25	XP_002635171.1	91	118.242	67	61	Hypothetical protein CBG11408
Locus_1109_Transcript_2/4_Conf_0.667	2042	20	0	NP_001041134.1	93	769.615	433	406	hypothetical protein F58E10.3

Locus_1109_Transcript_3/4_Conf_0.222	1916	20	0	XP_002635171.1	94	660.218	366	346	Hypothetical protein CBG11408
Locus_1109_Transcript_4/4_Conf_0.667	2028	20	0	NP_001041134.1	93	769.615	433	406	hypothetical protein F58E10.3
Locus_11090_Transcript_1/1_Conf_1.000	1154	2	6.29E-48	NP_507188.3	48	196.052	391	191	hypothetical protein Y32B12B.2
Locus_11091_Transcript_1/1_Conf_1.000	592	2	1.52E-15	XP_002630921.1	76	86.6557	75	57	Hypothetical protein CBG02647
Locus_11092_Transcript_1/1_Conf_1.000	243	0							
Locus_11093_Transcript_1/2_Conf_1.000	661	0							
Locus_11093_Transcript_2/2_Conf_1.000	657	0							
Locus_11094_Transcript_1/2_Conf_1.000	514	3	7.44E-11	EFO17966.1	69	70.4774	66	46	hypothetical protein LOAG_10533
Locus_11094_Transcript_2/2_Conf_1.000	258	3	1.11E-08	EFO17966.1	69	63.1586	59	41	hypothetical protein LOAG_10533
Locus_11095_Transcript_1/1_Conf_1.000	376	0							
Locus_11096_Transcript_1/1_Conf_1.000	693	20	9.69E-19	XP_001895742.1	74	97.8265	97	72	KH domain containing protein
Locus_11097_Transcript_1/1_Conf_1.000	362	0							
Locus_11098_Transcript_1/1_Conf_1.000	455	7	2.42E-16	NP_509836.1	51	88.5817	155	80	VEGF (vascular endothelial growth factor) Receptor family member (ver-3)
Locus_11099_Transcript_1/1_Conf_1.000	249	20	4.81E-33	XP_002630901.1	86	144.05	87	75	Hypothetical protein CBG02625
Locus_111_Transcript_1/1_Conf_1.000	1415	20	0	XP_002647388.1	81	655.981	465	381	Hypothetical protein CBG06453
Locus_1110_Transcript_1/2_Conf_1.000	3146	5	6.29E-10	NP_498207.1	41	71.633	272	112	Germline-enhanced AT-HooK protein family member (gak-1)

Locus_1110_Transcript_2/2_Conf_1.000	3035	5	6.08E-10	NP_498207.1	41	71.633	272	112	Germline-enhanced AT-HooK protein family member (gak-1)
Locus_11100_Transcript_1/1_Conf_1.000	347	0							
Locus_11101_Transcript_1/1_Conf_1.000	466	20	5.58E-61	ACJ65180.1	87	236.884	154	135	Gaba b receptor subunit protein 2, confirmed by transcript evidence
Locus_11102_Transcript_1/1_Conf_1.000	713	7	1.62E-08	XP_002641190.1	66	63.929	54	36	Hypothetical protein CBG09051
Locus_11103_Transcript_1/1_Conf_1.000	150	0							
Locus_11104_Transcript_1/1_Conf_1.000	946	20	2.64E-51	XP_002641428.1	59	206.838	276	164	C. briggsae CBR-MAA-1 protein
Locus_11105_Transcript_1/1_Conf_1.000	595	0							
Locus_11106_Transcript_1/1_Conf_1.000	233	0							
Locus_11107_Transcript_1/1_Conf_1.000	302	20	2.75E-52	AAO85518.1	96	207.994	100	96	AF496634_1putative serine/threonine phosphatase
Locus_11108_Transcript_1/1_Conf_1.000	312	20	3.02E-30	EFO25269.1	91	134.806	95	87	HT004 protein
Locus_11109_Transcript_1/1_Conf_1.000	130	20	1.20E-15	NP_499263.1	93	86.2705	43	40	hypothetical protein T20G5.4
Locus_1111_Transcript_1/1_Conf_1.000	1166	4	4.48E-09	NP_498170.2	45	67.0106	244	111	hypothetical protein R02F2.7
Locus_11110_Transcript_1/1_Conf_1.000	175	20	1.16E-10	XP_002631813.1	86	69.707	44	38	Hypothetical protein CBG21034
Locus_11111_Transcript_1/1_Conf_1.000	311	0							
Locus_11112_Transcript_1/1_Conf_1.000	184	20	5.92E-18	CBA12962.1	78	93.9745	61	48	LEV-9 protein
Locus_11113_Transcript_1/1_Conf_1.000	1147	20	6.79E-103	XP_002642274.1	64	378.637	334	217	C. briggsae CBR-PDR-1 protein
Locus_11114_Transcript_1/1_Conf_1.000	405	20	1.52E-18	XP_001901920.1	88	95.9005	51	45	hypothetical protein Bm1_52260

Locus_11115_Transcript_1/1_Conf_1.000	558	20	1.98E-40	XP_002639086.1	89	169.088	106	95	C. briggsae CBR-VHA-10 protein
Locus_11116_Transcript_1/1_Conf_1.000	783	20	1.83E-46	XP_002642305.1	59	190.274	267	158	Hypothetical protein CBG18297
Locus_11117_Transcript_1/1_Conf_1.000	139	20	1.01E-14	P29190.1	94	83.1889	39	37	Phosphoenolpyruvate carboxylase
Locus_11118_Transcript_1/1_Conf_1.000	511	20	1.55E-37	NP_495841.1	65	159.073	163	106	hypothetical protein D2085.3
Locus_11119_Transcript_1/1_Conf_1.000	371	20	4.95E-33	XP_002631287.1	98	144.05	89	88	C. briggsae CBR-TNC-2 protein
Locus_1112_Transcript_1/1_Conf_1.000	557	3	3.21E-06	XP_002642465.1	59	55.4546	89	53	C. briggsae CBR-LIN-9 protein
Locus_11120_Transcript_1/1_Conf_1.000	490	20	2.20E-33	XP_001898147.1	76	145.206	125	96	SRB protein.
Locus_11121_Transcript_1/1_Conf_1.000	759	4	2.81E-65	NP_498072.1	67	252.677	248	168	hypothetical protein C45G9.5
Locus_11122_Transcript_1/1_Conf_1.000	570	20	4.50E-99	CAR63542.1	96	363.999	189	182	putative Pre-mRNA splicing protein
Locus_11123_Transcript_1/1_Conf_1.000	200	0							
Locus_11124_Transcript_1/1_Conf_1.000	593	0							
Locus_11125_Transcript_1/1_Conf_1.000	339	2	3.03E-11	XP_002629898.1	55	71.633	100	55	Hypothetical protein CBG21936
Locus_11126_Transcript_1/1_Conf_1.000	294	2	4.79E-04	XP_001554686.1	50	47.7506	101	51	hypothetical protein BC1G_06829

Locus_11127_Transcript_1/1_Conf_1.000	224	10	2.64E-26	NP_001022643.1	86	121.709	74	64	LEThal family member (let-805)
Locus_11128_Transcript_1/1_Conf_1.000	407	8	4.01E-19	EFO26895.1	61	97.8265	123	76	organic solute transporter alpha-like protein
Locus_11129_Transcript_1/1_Conf_1.000	155	0							
Locus_1113_Transcript_1/1_Conf_1.000	1578	20	0	CAR63639.1	98	686.797	354	347	hypothetical protein
Locus_11130_Transcript_1/2_Conf_1.000	525	0							
Locus_11130_Transcript_2/2_Conf_1.000	525	0							
Locus_11131_Transcript_1/1_Conf_1.000	447	0							
Locus_11132_Transcript_1/1_Conf_1.000	481	20	2.25E-14	EFO24820.1	62	82.0333	116	72	hypothetical protein LOAG_03664
Locus_11133_Transcript_1/2_Conf_1.000	1221	20	3.06E-80	XP_001898803.1	69	303.523	371	256	G-patch domain containing protein
Locus_11133_Transcript_2/2_Conf_1.000	1221	20	3.06E-80	XP_001898803.1	69	303.523	371	256	G-patch domain containing protein
Locus_11134_Transcript_1/1_Conf_1.000	584	20	9.06E-98	CAI40836.1	100	359.762	194	194	prosaposin

Locus_11135_Transcript_1/1_Conf_1.000	610	20	4.01E-78	NP_496338.1	91	294.664	183	167	hypothetical protein F59E10.3
Locus_11136_Transcript_1/1_Conf_1.000	532	4	1.27E-06	NP_493601.2	40	56.6102	190	76	FERM domain (protein4.1-ezrin-radixin-moesin) family member (frm-1)
Locus_11137_Transcript_1/1_Conf_1.000	461	20	3.09E-64	NP_502290.1	93	247.669	153	143	hypothetical protein F11A10.2
Locus_11138_Transcript_1/1_Conf_1.000	1540	20	2.51E-24	XP_001894571.1	48	118.242	400	192	UBX domain containing protein
Locus_11139_Transcript_1/1_Conf_1.000	338	4	7.01E-16	NP_491339.2	63	87.0409	105	67	hypothetical protein C43E11.11
Locus_1114_Transcript_1/1_Conf_1.000	541	1	2.48E-05	CAR63546.1	55	52.373	65	36	hypothetical protein
Locus_11140_Transcript_1/1_Conf_1.000	901	0							
Locus_11141_Transcript_1/1_Conf_1.000	497	20	2.54E-74	NP_491989.1	93	281.182	165	155	hypothetical protein C30F12.7
Locus_11142_Transcript_1/1_Conf_1.000	248	0							
Locus_11143_Transcript_1/1_Conf_1.000	333	20	8.57E-22	NP_499157.2	87	106.686	110	96	hypothetical protein T05G5.8
Locus_11144_Transcript_1/1_Conf_1.000	292	0							
Locus_11145_Transcript_1/1_Conf_1.000	506	0							

Locus_11146_Transcript_1/1_Conf_1.000	962	20	4.30E-41	XP_002631725.1	51	172.94	301	155	Hypothetical protein CBG20927
Locus_11147_Transcript_1/1_Conf_1.000	639	20	4.25E-36	XP_002630784.1	70	155.221	144	101	Hypothetical protein CBG02480
Locus_11148_Transcript_1/1_Conf_1.000	902	20	4.99E-81	NP_492213.1	79	305.449	221	176	Acyl protein THioesterase family member (ath-1)
Locus_11149_Transcript_1/1_Conf_1.000	215	0							
Locus_1115_Transcript_1/1_Conf_1.000	113	0							
Locus_11150_Transcript_1/1_Conf_1.000	1900	20	2.99E-102	EFO23042.1	57	377.481	622	356	hypothetical protein LOAG_05445
Locus_11151_Transcript_1/1_Conf_1.000	724	0							
Locus_11152_Transcript_1/1_Conf_1.000	910	20	1.41E-131	NP_502028.1	87	473.396	298	260	hypothetical protein ZK596.2
Locus_11153_Transcript_1/1_Conf_1.000	350	20	1.74E-46	EFO20855.1	88	188.734	115	102	hypothetical protein LOAG_07635
Locus_11154_Transcript_1/1_Conf_1.000	261	20	7.13E-24	XP_001900101.1	82	113.62	80	66	ADP-specific Phosphofructokinase/Glucokinase conserved region family protein
Locus_11155_Transcript_1/1_Conf_1.000	3521	20	2.71E-33	NP_491539.1	40	149.443	1174	475	SPindle Defective family member (spd-5)
Locus_11156_Transcript_1/1_Conf_1.000	162	20	8.40E-17	EFO20302.1	86	90.1225	53	46	hypothetical protein LOAG_08188
Locus_11157_Transcript_1/1_Conf_1.000	232	20	2.15E-12	NP_001022541.1	73	75.485	60	44	hypothetical protein ZK970.8
Locus_11158_Transcript_1/1_Conf_1.000	143	0							
Locus_11159_Transcript_1/1_Conf_1.000	238	0							
Locus_1116_Transcript_1/1_Conf_1.000	1338	20	2.04E-72	EFO21147.1	59	277.715	386	229	RNA binding protein
Locus_11160_Transcript_1/1_Conf_1.000	252	2	3.25E-08	NP_001023399.1	88	61.6178	34	30	hypothetical protein T23B5.4
Locus_11161_Transcript_1/1_Conf_1.000	636	20	2.34E-87	XP_002639436.1	84	325.479	209	177	Hypothetical protein CBG04029

Locus_11162_Transcript_1/3_Conf_0.667	681	0							
Locus_11162_Transcript_2/3_Conf_0.667	666	1	7.99E-04	ZP_04667639.1	42	48.1358	212	90	1,4-alpha-glucan branching enzyme
Locus_11162_Transcript_3/3_Conf_0.667	666	0							
Locus_11163_Transcript_1/1_Conf_1.000	608	20	3.56E-87	NP_497886.2	95	324.709	161	153	hypothetical protein R07E5.4
Locus_11164_Transcript_1/1_Conf_1.000	481	20	1.56E-15	XP_002638710.1	81	85.8853	59	48	Hypothetical protein CBG00291
Locus_11165_Transcript_1/1_Conf_1.000	180	2	7.53E-05	XP_002642697.1	59	50.447	59	35	C. briggsae CBR-NPP-15 protein
Locus_11166_Transcript_1/1_Conf_1.000	153	0							
Locus_11167_Transcript_1/1_Conf_1.000	253	20	6.73E-30	XP_002647042.1	86	133.65	84	73	Hypothetical protein CBG03560
Locus_11168_Transcript_1/1_Conf_1.000	559	20	5.32E-94	CAR63641.1	96	347.051	185	179	putative 26S proteasome regulatory chain 4
Locus_11169_Transcript_1/1_Conf_1.000	1069	8	4.80E-15	NP_497463.3	45	86.6557	288	131	hypothetical protein Y39A3CL.1
Locus_1117_Transcript_1/6_Conf_0.250	340	0							
Locus_1117_Transcript_3/6_Conf_0.708	1535	0							
Locus_1117_Transcript_4/6_Conf_0.542	1276	0							
Locus_1117_Transcript_5/6_Conf_0.667	1547	0							
Locus_1117_Transcript_6/6_Conf_0.667	1547	0							
Locus_11170_Transcript_1/1_Conf_1.000	667	0							
Locus_11171_Transcript_1/1_Conf_1.000	129	0							
Locus_11172_Transcript_1/1_Conf_1.000	314	0							
Locus_11173_Transcript_1/1_Conf_1.000	303	20	2.49E-37	NP_501339.2	88	158.303	99	88	neuRonal IGCAM family member (rig-4)
Locus_11174_Transcript_1/1_Conf_1.000	1037	20	3.15E-56	EFO24512.1	77	223.402	204	159	hypothetical protein LOAG_03972
Locus_11175_Transcript_1/1_Conf_1.000	404	20	1.37E-43	CAX65056.1	78	179.104	136	107	C. elegans protein F25C8.3d, partially confirmed by transcript evidence
Locus_11176_Transcript_1/1_Conf_1.000	409	20	3.04E-35	XP_002638650.1	77	151.369	135	105	C. briggsae CBR-TGT-2 protein
Locus_11177_Transcript_1/1_Conf_1.000	261	0							
Locus_11178_Transcript_1/1_Conf_1.000	209	0							
Locus_11179_Transcript_1/3_Conf_0.600	489	20	1.91E-61	CAR63538.1	75	238.424	162	123	putative MFP2
Locus_11179_Transcript_2/3_Conf_0.600	843	20	7.07E-103	CAR63538.1	79	377.867	249	198	putative MFP2
Locus_11179_Transcript_3/3_Conf_0.600	843	20	1.21E-102	CAR63538.1	79	377.096	249	198	putative MFP2

Locus_1118_Transcript_1/2_Conf_1.000	4155	20	0	ABA18180.1	90	1613.59	925	841	argonaute-like
Locus_1118_Transcript_2/2_Conf_1.000	4149	20	0	ABA18180.1	90	1613.59	925	841	argonaute-like
Locus_11180_Transcript_1/1_Conf_1.000	215	20	1.84E-16	XP_002642051.1	66	88.9669	72	48	Hypothetical protein CBG17988
Locus_11181_Transcript_1/1_Conf_1.000	181	5	2.49E-24	EFO16149.1	93	115.161	59	55	hypothetical protein LOAG_12360
Locus_11182_Transcript_1/1_Conf_1.000	974	20	2.56E-57	NP_498602.1	73	226.868	223	164	hypothetical protein B0361.6
Locus_11183_Transcript_1/1_Conf_1.000	173	0							
Locus_11184_Transcript_1/1_Conf_1.000	145	0							
Locus_11185_Transcript_1/1_Conf_1.000	988	20	3.98E-122	NP_508530.1	84	442.195	328	276	yeast SEC homolog family member (sec-3)
Locus_11186_Transcript_1/1_Conf_1.000	582	5	7.11E-10	XP_002637083.1	56	67.781	127	72	Hypothetical protein CBG09582
Locus_11187_Transcript_1/2_Conf_1.000	645	20	5.62E-76	NP_502165.2	87	287.73	219	192	hypothetical protein C10C6.6
Locus_11187_Transcript_2/2_Conf_1.000	645	20	5.62E-76	NP_502165.2	87	287.73	219	192	hypothetical protein C10C6.6
Locus_11188_Transcript_1/1_Conf_1.000	530	20	4.71E-14	EFO26499.1	95	81.2629	45	43	Cab39 protein
Locus_11189_Transcript_1/1_Conf_1.000	154	0							
Locus_1119_Transcript_1/1_Conf_1.000	521	20	2.57E-38	NP_001022966.1	93	161.77	116	109	hypothetical protein Y82E9BR.3

Locus_11190_Transcript_1/1_Conf_1.000	178	20	1.90E-13	EFO22275.1	80	78.9518	56	45	hypothetical protein LOAG_06211
Locus_11191_Transcript_1/1_Conf_1.000	228	0							
Locus_11192_Transcript_1/1_Conf_1.000	352	20	5.80E-26	NP_510321.1	69	120.553	91	63	Erythroid-Like Transcription factor family member (elt-3)
Locus_11193_Transcript_1/1_Conf_1.000	425	20	6.23E-20	XP_002640260.1	67	100.523	139	94	Hypothetical protein CBG12785
Locus_11194_Transcript_1/1_Conf_1.000	598	5	1.46E-29	XP_002639411.1	62	133.265	161	100	Hypothetical protein CBG04000
Locus_11195_Transcript_1/1_Conf_1.000	891	20	2.44E-64	ACO15781.1	72	249.98	238	172	Hypothetical protein F40G9.17
Locus_11196_Transcript_1/1_Conf_1.000	251	1	2.31E-27	NP_001024262.1	83	125.176	83	69	hypothetical protein Y51A2D.21
Locus_11197_Transcript_1/1_Conf_1.000	618	20	6.48E-55	ACJ65179.1	92	217.624	153	142	Hypothetical protein Y67D8C.10d
Locus_11198_Transcript_1/2_Conf_1.000	955	20	1.46E-33	NP_496477.2	50	147.902	296	150	hypothetical protein D1043.1
Locus_11198_Transcript_2/2_Conf_1.000	622	16	7.20E-22	NP_496477.2	53	107.842	186	99	hypothetical protein D1043.1
Locus_11199_Transcript_1/1_Conf_1.000	582	0							
Locus_112_Transcript_1/2_Conf_1.000	899	3	1.24E-15	XP_002630206.1	59	88.1965	144	86	Hypothetical protein CBG00616
Locus_112_Transcript_2/2_Conf_1.000	601	3	1.59E-15	XP_002630206.1	60	86.6557	141	85	Hypothetical protein CBG00616

Locus_1120_Transcript_1/1_Conf_1.000	249	0							
Locus_11200_Transcript_1/1_Conf_1.000	299	0							
Locus_11201_Transcript_1/4_Conf_0.571	334	20	7.74E-31	NP_498870.1	77	136.732	110	85	hypothetical protein C30C11.1
Locus_11201_Transcript_2/4_Conf_0.571	469	20	3.87E-38	NP_498870.1	79	160.999	116	92	hypothetical protein C30C11.1
Locus_11201_Transcript_3/4_Conf_0.429	334	20	7.74E-31	NP_498870.1	77	136.732	110	85	hypothetical protein C30C11.1
Locus_11201_Transcript_4/4_Conf_0.571	469	20	3.87E-38	NP_498870.1	79	160.999	116	92	hypothetical protein C30C11.1
Locus_11202_Transcript_1/1_Conf_1.000	191	0							
Locus_11203_Transcript_1/1_Conf_1.000	341	0							
Locus_11204_Transcript_1/1_Conf_1.000	232	0							
Locus_11205_Transcript_1/1_Conf_1.000	267	4	2.70E-07	XP_001894559.1	66	58.5362	56	37	Bromodomain containing protein
Locus_11206_Transcript_1/1_Conf_1.000	904	20	9.15E-83	NP_496402.1	82	311.227	220	182	hypothetical protein T06D8.5

Locus_11207_Transcript_1/1_Conf_1.000	581	20	3.48E-118	3K9M	100	427.557	193	193	Cathepsin B In Complex With Stefin A
Locus_11208_Transcript_1/1_Conf_1.000	899	0							
Locus_11209_Transcript_1/1_Conf_1.000	249	20	1.97E-10	XP_002845397.1	67	68.9366	73	49	phosphoethanolamine transferase 7
Locus_1121_Transcript_1/1_Conf_1.000	588	6	1.40E-21	NP_001023357.2	68	106.686	163	111	hypothetical protein T04C4.1
Locus_11210_Transcript_1/1_Conf_1.000	328	0							
Locus_11211_Transcript_1/2_Conf_1.000	204	0							
Locus_11211_Transcript_2/2_Conf_1.000	216	0							
Locus_11212_Transcript_1/1_Conf_1.000	170	0							
Locus_11213_Transcript_1/1_Conf_1.000	1483	20	3.34E-127	NP_506619.1	81	459.914	365	299	RFC (DNA replication factor) family member (rfc-1)
Locus_11214_Transcript_1/1_Conf_1.000	404	0							
Locus_11215_Transcript_1/1_Conf_1.000	355	0							
Locus_11216_Transcript_1/1_Conf_1.000	258	0							

Locus_11217_Transcript_1/1_Conf_1.000	628	20	3.73E-82	NP_508190.1	85	308.145	209	179	DiacylGlycerol Kinase family member (dgk-1)
Locus_11218_Transcript_1/1_Conf_1.000	1034	5	2.04E-39	XP_002641460.1	54	167.548	340	186	Hypothetical protein CBG13329
Locus_11219_Transcript_1/1_Conf_1.000	552	20	2.30E-57	Q09261.2	79	225.328	152	121	Uncharacterized protein C32A3.2
Locus_1122_Transcript_1/1_Conf_1.000	771	0							
Locus_11220_Transcript_1/1_Conf_1.000	236	20	9.30E-08	BAH16150.1	100	60.077	51	51	NADH dehydrogenase subunit 6
Locus_11221_Transcript_1/1_Conf_1.000	281	0							
Locus_11222_Transcript_1/1_Conf_1.000	242	20	1.92E-21	XP_002642159.1	78	105.531	79	62	Hypothetical protein CBG18119
Locus_11223_Transcript_1/1_Conf_1.000	1356	20	0	XP_002644007.1	90	706.057	410	371	Hypothetical protein CBG17397
Locus_11224_Transcript_1/1_Conf_1.000	188	0							
Locus_11225_Transcript_1/1_Conf_1.000	1096	2	7.91E-45	XP_002644320.1	52	185.652	330	174	Hypothetical protein CBG14129
Locus_11226_Transcript_1/1_Conf_1.000	586	20	5.11E-56	NP_498463.1	79	221.09	174	138	hypothetical protein C13B9.3
Locus_11227_Transcript_1/1_Conf_1.000	481	20	3.93E-43	XP_002640121.1	79	177.563	123	98	C. briggsae CBR-RBX-2 protein

Locus_11228_Transcript_1/1_Conf_1.000	1204	2	1.50E-47	NP_500551.2	52	194.897	412	215	hypothetical protein F55F10.1
Locus_11229_Transcript_1/1_Conf_1.000	566	0							
Locus_1123_Transcript_1/1_Conf_1.000	956	20	1.17E-107	ACI49193.1	75	394.045	317	239	hypothetical protein Csp3_JD03.002
Locus_11230_Transcript_1/1_Conf_1.000	523	7	1.31E-29	XP_001891702.1	65	132.88	132	87	Hypothetical 30.5 kDa protein ZK1321.3 in chromosome II, putative
Locus_11231_Transcript_1/1_Conf_1.000	287	7	9.11E-11	NP_504680.1	56	70.0922	94	53	hypothetical protein ZK1055.7
Locus_11232_Transcript_1/1_Conf_1.000	149	0							
Locus_11233_Transcript_1/1_Conf_1.000	340	20	2.38E-40	XP_002631516.1	83	168.318	112	94	Hypothetical protein CBG20680
Locus_11234_Transcript_1/1_Conf_1.000	988	20	1.03E-77	NP_499890.2	69	294.664	267	186	hypothetical protein T21D12.3
Locus_11235_Transcript_1/1_Conf_1.000	183	3	1.51E-05	XP_001901405.1	83	52.7582	37	31	Conserved hypothetical protein
Locus_11236_Transcript_1/1_Conf_1.000	1171	4	1.14E-28	NP_493404.2	50	132.109	320	160	hypothetical protein Y6B3B.9
Locus_11237_Transcript_1/1_Conf_1.000	170	0							
Locus_11238_Transcript_1/2_Conf_1.000	750	20	1.15E-103	NP_001021395.1	87	380.178	246	216	Receptor Mediated Endocytosis family member (rme-8)
Locus_11238_Transcript_2/2_Conf_1.000	538	20	1.65E-70	NP_001021395.1	88	268.855	176	155	Receptor Mediated Endocytosis family member (rme-8)
Locus_11239_Transcript_1/1_Conf_1.000	1041	20	1.40E-104	NP_001033378.1	78	384.03	315	247	Acyl CoA DeHydrogenase family member (acdh-11)
Locus_1124_Transcript_1/1_Conf_1.000	857	20	3.68E-30	NP_495106.1	72	136.346	102	74	FUcosyl Transferase family member (fut-3)
Locus_11240_Transcript_1/1_Conf_1.000	317	0							
Locus_11241_Transcript_1/1_Conf_1.000	604	20	3.56E-47	CAR63674.1	79	191.815	157	125	putative PeRoXisome assembly factor
Locus_11242_Transcript_1/1_Conf_1.000	947	20	0	NP_492020.1	99	672.159	315	314	hypothetical protein K10D3.4
Locus_11243_Transcript_1/1_Conf_1.000	340	0							
Locus_11244_Transcript_1/2_Conf_0.667	168	0							

Locus_11244_Transcript_2/2_Conf_0.667	168	0								
Locus_11245_Transcript_1/3_Conf_0.667	1205	20	3.06E-08	XP_001900670.1	46	64.3142	191	89	BRCA2 repeat family protein	
Locus_11245_Transcript_2/3_Conf_0.500	743	20	1.67E-06	YP_001158279.1	35	57.3806	202	72	hypothetical protein Strop_1435	
Locus_11245_Transcript_3/3_Conf_0.667	1151	20	3.48E-06	YP_001158279.1	35	57.3806	202	72	hypothetical protein Strop_1435	
Locus_11246_Transcript_1/1_Conf_1.000	671	20	2.31E-67	XP_002633298.1	78	259.225	209	165	C. briggsae CBR-PRX-14 protein	
Locus_11247_Transcript_1/1_Conf_1.000	526	20	8.93E-34	XP_001949820.1	63	146.747	152	96	PREDICTED: similar to conserved hypothetical protein	
Locus_11248_Transcript_1/1_Conf_1.000	377	0								
Locus_11249_Transcript_1/1_Conf_1.000	249	0								
Locus_1125_Transcript_1/1_Conf_1.000	253	3	3.35E-21	NP_001041068.1	80	104.76	73	59	hypothetical protein ZK822.5	
Locus_11250_Transcript_1/1_Conf_1.000	318	0								
Locus_11251_Transcript_1/1_Conf_1.000	334	3	1.62E-04	NP_492725.2	40	49.2914	84	34	hypothetical protein F25D7.5	
Locus_11252_Transcript_1/1_Conf_1.000	792	20	2.08E-29	XP_001892702.1	51	133.65	267	137	zyg-9 protein	
Locus_11253_Transcript_1/1_Conf_1.000	813	20	7.93E-40	NP_001022052.1	61	168.318	244	149	SREBP Cleavage activating Protein (SCAP) homolog family member (scp-1)	
Locus_11254_Transcript_1/1_Conf_1.000	387	0								
Locus_11255_Transcript_1/1_Conf_1.000	317	4	1.50E-05	NP_741096.1	55	52.7582	65	36	hypothetical protein W04B5.3	
Locus_11256_Transcript_1/1_Conf_1.000	1021	20	8.10E-57	NP_001023357.2	64	225.328	265	172	hypothetical protein T04C4.1	
Locus_11257_Transcript_1/1_Conf_1.000	891	0								

Locus_11258_Transcript_1/1_Conf_1.000	433	20	3.35E-42	AAA27985.1	90	174.481	102	92	alpha-collagen
Locus_11259_Transcript_1/1_Conf_1.000	338	10	1.16E-10	EFO17726.1	54	69.707	112	61	hypothetical protein LOAG_10772
Locus_1126_Transcript_1/3_Conf_0.500	1243	7	9.87E-42	NP_001123026.1	45	175.637	499	225	RETiculon protein family member (ret-1)
Locus_1126_Transcript_2/3_Conf_0.667	2209	20	2.04E-89	NP_506656.2	50	335.109	718	366	RETiculon protein family member (ret-1)
Locus_1126_Transcript_3/3_Conf_0.333	1027	20	2.30E-59	NP_506657.3	74	233.802	249	185	RETiculon protein family member (ret-1)
Locus_11260_Transcript_1/1_Conf_1.000	372	20	2.79E-44	NP_491777.3	80	181.415	118	95	hypothetical protein T09B4.8
Locus_11261_Transcript_1/1_Conf_1.000	509	20	2.09E-66		93	254.988	169	158	hypothetical protein Y18D10A.15 - Caenorhabditis elegans
Locus_11262_Transcript_1/1_Conf_1.000	692	20	2.18E-63	NP_001021719.1	73	246.128	218	160	hypothetical protein Y110A7A.9
Locus_11263_Transcript_1/1_Conf_1.000	135	0							
Locus_11264_Transcript_1/1_Conf_1.000	187	10	5.00E-09	NP_001022133.1	59	64.3142	62	37	hypothetical protein F32A5.1
Locus_11265_Transcript_1/1_Conf_1.000	195	20	8.70E-22	XP_002604687.1	85	106.686	64	55	hypothetical protein BRAFLDRAFT_282362
Locus_11266_Transcript_1/1_Conf_1.000	154	0							
Locus_11267_Transcript_1/1_Conf_1.000	381	20	5.58E-29	XP_002632484.1	73	130.568	126	93	Hypothetical protein CBG13719

Locus_11268_Transcript_1/1_Conf_1.000	238	3	3.63E-12	EFO17234.1	75	74.7146	62	47	RecQ helicase
Locus_11269_Transcript_1/1_Conf_1.000	142	0							
Locus_1127_Transcript_1/1_Conf_1.000	854	20	1.30E-35	NP_502208.1	61	154.451	173	107	AuTophaGy (yeast Atg homolog) family member (atg-4.2)
Locus_11270_Transcript_1/1_Conf_1.000	682	20	4.72E-55	ACI49113.1	86	218.394	131	113	hypothetical protein Cbre_JD14.001
Locus_11271_Transcript_1/1_Conf_1.000	186	0							
Locus_11272_Transcript_1/1_Conf_1.000	278	20	6.51E-46	BAE88120.1	100	186.808	92	92	unnamed protein product
Locus_11273_Transcript_1/1_Conf_1.000	250	20	2.74E-20	NP_490990.2	78	101.679	83	65	hypothetical protein Y23H5B.5
Locus_11274_Transcript_1/2_Conf_1.000	602	1	2.15E-04	EFO27879.1	43	49.6766	133	58	hypothetical protein LOAG_00601
Locus_11274_Transcript_2/2_Conf_1.000	602	1	2.15E-04	EFO27879.1	43	49.6766	133	58	hypothetical protein LOAG_00601
Locus_11275_Transcript_1/1_Conf_1.000	577	20	2.21E-24	XP_310936.4	79	115.931	84	67	AGAP000199-PA
Locus_11276_Transcript_1/1_Conf_1.000	133	0							
Locus_11277_Transcript_1/1_Conf_1.000	436	20	2.09E-44	XP_002640736.1	84	181.8	119	101	Hypothetical protein CBG19806
Locus_11278_Transcript_1/1_Conf_1.000	779	20	3.85E-65	NP_509509.1	83	252.292	167	140	hypothetical protein D1009.1

Locus_11279_Transcript_1/1_Conf_1.000	858	20	9.30E-82	NP_495015.1	78	307.76	257	202	NuDiX family member (ndx-6)
Locus_1128_Transcript_1/1_Conf_1.000	118	0							
Locus_11280_Transcript_1/1_Conf_1.000	153	20	3.44E-18	NP_491363.1	98	94.7449	50	49	Dynein Heavy Chain family member (dhc-1)
Locus_11281_Transcript_1/1_Conf_1.000	257	0							
Locus_11282_Transcript_1/1_Conf_1.000	709	15	8.25E-05	XP_002318326.1	39	51.6026	258	103	predicted protein
Locus_11283_Transcript_1/1_Conf_1.000	518	3	9.29E-17	NP_001129848.1	64	90.1225	109	70	hypothetical protein C27H2.2
Locus_11284_Transcript_1/1_Conf_1.000	257	0							
Locus_11285_Transcript_1/1_Conf_1.000	495	0							
Locus_11286_Transcript_1/1_Conf_1.000	227	3	7.16E-24	NP_503052.1	79	113.62	78	62	hypothetical protein C52D10.12
Locus_11287_Transcript_1/1_Conf_1.000	136	0							
Locus_11288_Transcript_1/1_Conf_1.000	804	20	9.06E-121	EFO21494.1	86	437.187	265	228	hypothetical protein LOAG_06995
Locus_11289_Transcript_1/1_Conf_1.000	328	20	2.85E-49	3LQZ	100	197.978	109	109	CrystalStructure Of Hla-Dp2
Locus_1129_Transcript_1/1_Conf_1.000	1118	20	1.32E-103	CAV31790.1	84	380.948	248	210	C. elegans protein Y105E8B.8c, confirmed by transcript evidence

Locus_11290_Transcript_1/1_Conf_1.000	292	0							
Locus_11291_Transcript_1/1_Conf_1.000	458	0							
Locus_11292_Transcript_1/1_Conf_1.000	151	5	7.45E-13	XP_002643017.1	84	77.0258	50	42	C. briggsae CBR-POLK-1 protein
Locus_11293_Transcript_1/1_Conf_1.000	288	20	2.46E-24	XP_002632657.1	84	115.161	85	72	C. briggsae CBR-TRA-3 protein
Locus_11294_Transcript_1/1_Conf_1.000	253	20	2.16E-28	XP_002644773.1	94	128.642	67	63	Hypothetical protein CBG14786
Locus_11295_Transcript_1/2_Conf_1.000	627	0							
Locus_11295_Transcript_2/2_Conf_1.000	335	1	2.77E-04	XP_001892902.1	52	48.521	71	37	ATPase, AAA family protein
Locus_11296_Transcript_1/1_Conf_1.000	278	0							
Locus_11297_Transcript_1/1_Conf_1.000	842	20	1.59E-46	XP_002644816.1	60	190.66	265	161	Hypothetical protein CBG14834
Locus_11298_Transcript_1/1_Conf_1.000	656	0							
Locus_11299_Transcript_1/1_Conf_1.000	1014	20	2.26E-51	NP_495391.1	81	207.223	159	130	hypothetical protein F31E8.4
Locus_113_Transcript_1/2_Conf_1.000	919	5	2.13E-26	NP_492097.1	63	124.02	261	166	hypothetical protein ZK524.4
Locus_113_Transcript_2/2_Conf_1.000	787	5	2.34E-25	NP_492097.1	63	120.168	259	164	hypothetical protein ZK524.4
Locus_1130_Transcript_1/1_Conf_1.000	612	4	1.29E-15	XP_002639202.1	65	87.0409	104	68	Hypothetical protein CBG03746
Locus_11300_Transcript_1/1_Conf_1.000	403	0							
Locus_11301_Transcript_1/1_Conf_1.000	408	20	5.31E-72	BAG62064.1	100	273.478	135	135	unnamed protein product
Locus_11302_Transcript_1/1_Conf_1.000	301	2	2.60E-18	NP_493074.2	69	95.1301	99	69	hypothetical protein R05D7.1
Locus_11303_Transcript_1/1_Conf_1.000	185	0							
Locus_11304_Transcript_1/1_Conf_1.000	214	0							
Locus_11305_Transcript_1/1_Conf_1.000	226	0							

Locus_11306_Transcript_1/1_Conf_1.000	276	20	1.03E-22	XP_001900667.1	92	102.064	57	53	Hypothetical 26.6 kDa protein T19C3.4 in chromosome III, putative
Locus_11307_Transcript_1/1_Conf_1.000	578	20	5.04E-53	EFO20071.1	77	211.075	171	133	hypothetical protein LOAG_08420
Locus_11308_Transcript_1/1_Conf_1.000	471	0							
Locus_11309_Transcript_1/1_Conf_1.000	653	20	1.66E-107	NP_508927.2	92	392.504	217	200	DihydroPYrimidine Dehydrogenase family member (dpyd-1)
Locus_1131_Transcript_1/1_Conf_1.000	1513	20	6.24E-137	XP_002635005.1	70	492.271	458	323	C. briggsae CBR-ERS-3 protein
Locus_11310_Transcript_1/1_Conf_1.000	477	20	2.81E-65	NP_001122979.1	96	251.136	132	127	hypothetical protein M03F8.3
Locus_11311_Transcript_1/1_Conf_1.000	357	20	3.50E-15	NP_493404.2	64	84.7297	113	73	hypothetical protein Y6B3B.9
Locus_11312_Transcript_1/1_Conf_1.000	252	20	2.65E-18	NP_001023351.1	67	95.1301	74	50	hypothetical protein T01G1.4
Locus_11313_Transcript_1/1_Conf_1.000	233	2	1.76E-14	XP_002643270.1	79	82.4185	62	49	Hypothetical protein CBG08142
Locus_11314_Transcript_1/1_Conf_1.000	329	20	2.86E-33	NP_495799.1	79	144.821	106	84	hypothetical protein T13H5.4
Locus_11315_Transcript_1/1_Conf_1.000	167	0							

Locus_11316_Transcript_1/1_Conf_1.000	557	20	2.48E-91	A8X4H1.2	91	338.191	185	170	Dual specificity tyrosine-phosphorylation-regulated kinase mbk-1
Locus_11317_Transcript_1/1_Conf_1.000	314	0							
Locus_11318_Transcript_1/1_Conf_1.000	375	0							
Locus_11319_Transcript_1/1_Conf_1.000	629	0							
Locus_1132_Transcript_1/1_Conf_1.000	992	20	8.54E-40	XP_001897511.1	77	168.703	130	101	hypothetical protein Bm1_30365
Locus_11320_Transcript_1/1_Conf_1.000	423	0							
Locus_11321_Transcript_1/1_Conf_1.000	252	0							
Locus_11322_Transcript_1/1_Conf_1.000	979	20	3.55E-123	AAA03517.1	91	445.662	320	293	kinesin-related protein
Locus_11323_Transcript_1/1_Conf_1.000	390	0							
Locus_11324_Transcript_1/1_Conf_1.000	643	20	1.06E-42	XP_002631498.1	62	177.178	169	106	C. briggsae CBR-JMJD-2 protein
Locus_11325_Transcript_1/1_Conf_1.000	372	20	1.70E-17	XP_002647384.1	92	92.4337	51	47	Hypothetical protein CBG06449
Locus_11326_Transcript_1/1_Conf_1.000	977	20	1.40E-95	XP_002631498.1	68	353.984	320	220	C. briggsae CBR-JMJD-2 protein
Locus_11327_Transcript_1/1_Conf_1.000	696	20	1.33E-116	NP_496968.1	93	422.935	231	217	hypothetical protein Y48B6A.12
Locus_11328_Transcript_1/1_Conf_1.000	471	0							
Locus_11329_Transcript_1/1_Conf_1.000	283	0							

Locus_1133_Transcript_1/1_Conf_1.000	457	20	3.79E-62	XP_002638971.1	93	240.736	133	125	C. briggsae CBR-RPL-27 protein
Locus_11330_Transcript_1/1_Conf_1.000	619	4	9.11E-33	EFO26206.1	60	144.05	170	103	hypothetical protein LOAG_02278
Locus_11331_Transcript_1/1_Conf_1.000	349	7	3.30E-29	ADI24623.1	77	131.339	119	92	Copine domain protein, atypical protein 2, isoform c, partially confirmed by transcript evidence
Locus_11332_Transcript_1/1_Conf_1.000	757	20	2.61E-55	CBA11992.1	61	219.55	250	154	endonuclease-reverse transcriptase HmRTE-e01
Locus_11333_Transcript_1/1_Conf_1.000	618	20	4.77E-74	NP_506610.1	79	281.182	203	162	Patterned Expression Site family member (pes-9)
Locus_11334_Transcript_1/2_Conf_1.000	141	0							
Locus_11334_Transcript_2/2_Conf_1.000	165	0							
Locus_11335_Transcript_1/1_Conf_1.000	299	0							
Locus_11336_Transcript_1/1_Conf_1.000	389	20	2.18E-17	NP_507807.1	80	92.0485	71	57	hypothetical protein Y43F8C.7
Locus_11337_Transcript_1/1_Conf_1.000	486	20	7.30E-53	NP_495988.1	79	209.92	161	128	Eukaryotic Initiation Factor family member (eif-3.F)
Locus_11338_Transcript_1/1_Conf_1.000	161	20	1.44E-08	XP_002643286.1	71	62.7734	53	38	Hypothetical protein CBG08162
Locus_11339_Transcript_1/1_Conf_1.000	559	3	9.28E-14	XP_002641618.1	56	80.4925	187	105	Hypothetical protein CBG09938
Locus_1134_Transcript_1/6_Conf_0.357	495	20	1.60E-52	NP_509242.1	77	208.764	164	127	hypothetical protein C07D8.6

Locus_1134_Transcript_2/6_Conf_0.357	537	20	1.83E-53	NP_509242.1	77	212.231	166	129	hypothetical protein C07D8.6
Locus_1134_Transcript_3/6_Conf_0.357	1046	20	6.96E-104	NP_509242.1	76	381.719	306	235	hypothetical protein C07D8.6
Locus_1134_Transcript_4/6_Conf_0.357	1046	20	6.96E-104	NP_509242.1	76	381.719	306	235	hypothetical protein C07D8.6
Locus_1134_Transcript_5/6_Conf_0.357	1046	20	1.07E-104	NP_509242.1	76	384.415	306	235	hypothetical protein C07D8.6
Locus_1134_Transcript_6/6_Conf_0.357	1046	20	1.07E-104	NP_509242.1	76	384.415	306	235	hypothetical protein C07D8.6
Locus_11340_Transcript_1/1_Conf_1.000	559	20	4.00E-41	NP_505918.2	66	171.4	197	131	Tubulin Tyrosine Ligase Like family member (ttll-5)
Locus_11341_Transcript_1/1_Conf_1.000	932								
Locus_11342_Transcript_1/1_Conf_1.000	323	0							
Locus_11343_Transcript_1/2_Conf_1.000	688	20	4.46E-93	XP_002630033.1	84	344.739	226	191	C. briggsae CBR-PUF-12 protein
Locus_11343_Transcript_2/2_Conf_1.000	688	20	7.60E-93	XP_002630033.1	84	343.969	226	191	C. briggsae CBR-PUF-12 protein
Locus_11344_Transcript_1/1_Conf_1.000	1079	20	6.98E-38	EFO17895.1	52	162.54	260	137	hypothetical protein LOAG_10603
Locus_11345_Transcript_1/1_Conf_1.000	223	0							
Locus_11346_Transcript_1/1_Conf_1.000	354	0							
Locus_11347_Transcript_1/1_Conf_1.000	550	0							
Locus_11348_Transcript_1/1_Conf_1.000	502	20	1.64E-52	XP_002636901.1	80	208.764	166	134	Hypothetical protein CBG09363
Locus_11349_Transcript_1/1_Conf_1.000	222	0							
Locus_1135_Transcript_1/1_Conf_1.000	997	20	1.40E-98	NP_498051.1	80	363.999	283	228	hypothetical protein C27F2.4
Locus_11350_Transcript_1/1_Conf_1.000	648	0							
Locus_11351_Transcript_1/1_Conf_1.000	250	0							
Locus_11352_Transcript_1/1_Conf_1.000	325	4	6.39E-41	EFO17984.1	84	170.244	107	90	hypothetical protein LOAG_10513
Locus_11353_Transcript_1/1_Conf_1.000	307	2	1.67E-17	XP_002631009.1	60	92.4337	113	68	Hypothetical protein CBG02761
Locus_11354_Transcript_1/1_Conf_1.000	377	20	1.52E-58	ADG29194.1	100	228.794	114	114	40S ribosomal protein S23

Locus_11355_Transcript_1/1_Conf_1.000	777	20	6.88E-22	EFO23574.1	59	108.612	126	75	hypothetical protein LOAG_04910
Locus_11356_Transcript_1/1_Conf_1.000	944	20	5.23E-92	NP_871684.1	87	342.043	231	201	COenzyme Q (ubiquinone) biosynthesis family member (coq-2)
Locus_11357_Transcript_1/1_Conf_1.000	178	20	2.94E-14	XP_002643972.1	81	81.6481	61	50	C. briggsae CBR-SLO-2 protein
Locus_11358_Transcript_1/1_Conf_1.000	438	0							
Locus_11359_Transcript_1/1_Conf_1.000	329	20	6.62E-22	NP_001041137.1	82	107.071	101	83	Alpha MANnosidase family member (aman-2)
Locus_1136_Transcript_1/1_Conf_1.000	1684	4	2.30E-34	NP_490917.3	55	151.754	207	114	hypothetical protein Y20F4.2
Locus_11360_Transcript_1/1_Conf_1.000	687	20	5.54E-35	EFO23859.1	63	151.754	183	116	hypothetical protein LOAG_04629
Locus_11361_Transcript_1/1_Conf_1.000	169	0							
Locus_11362_Transcript_1/1_Conf_1.000	359	0							
Locus_11363_Transcript_1/1_Conf_1.000	395	0							
Locus_11364_Transcript_1/2_Conf_1.000	691	20	3.40E-24	NP_509021.2	51	115.931	239	122	hypothetical protein C15H9.4
Locus_11364_Transcript_2/2_Conf_1.000	679	20	5.56E-24	XP_002644813.1	54	115.161	242	131	Hypothetical protein CBG14831
Locus_11365_Transcript_1/1_Conf_1.000	137	1	4.27E-05	NP_494994.1	62	51.2174	45	28	hypothetical protein ZK622.1
Locus_11366_Transcript_1/1_Conf_1.000	937	2	8.69E-23	NP_510225.2	52	112.079	317	167	hypothetical protein C49F8.2
Locus_11367_Transcript_1/1_Conf_1.000	404	2	2.07E-07	NP_509527.1	47	58.9214	120	57	Sodium:Neurotransmitter symporter Family family member (snf-12)
Locus_11368_Transcript_1/1_Conf_1.000	529	20	7.45E-44	XP_002642085.1	70	180.259	151	107	Hypothetical protein CBG18026

Locus_11369_Transcript_1/1_Conf_1.000	2162	20	0	NP_492839.4	82	769.615	580	476	Leucine-rich repeats, Ras-like domain, Kinase family member (lrk-1)
Locus_1137_Transcript_1/1_Conf_1.000	1703	20	0	NP_498568.1	86	863.988	560	484	fatty Acid CoA Synthetase family member (acs-16)
Locus_11370_Transcript_1/1_Conf_1.000	461	20	1.61E-20	NP_497688.2	62	102.449	134	84	hypothetical protein Y53G8AR.5
Locus_11371_Transcript_1/1_Conf_1.000	237	2	3.40E-10	XP_001898035.1	61	68.1662	77	47	hypothetical protein Bm1_32955
Locus_11372_Transcript_1/1_Conf_1.000	168	0							
Locus_11373_Transcript_1/3_Conf_0.500	171	0							
Locus_11373_Transcript_2/3_Conf_0.500	171	0							
Locus_11373_Transcript_3/3_Conf_0.667	171	0							
Locus_11374_Transcript_1/1_Conf_1.000	195	0							
Locus_11375_Transcript_1/1_Conf_1.000	753	14	1.86E-45	NP_506662.1	59	186.808	231	137	hypothetical protein ZC412.3
Locus_11376_Transcript_1/1_Conf_1.000	1024	4	7.69E-23	XP_002629901.1	52	112.464	252	132	Hypothetical protein CBG21942
Locus_11377_Transcript_1/1_Conf_1.000	733	2	2.67E-09	CAP34328.1	66	66.6254	59	39	Hypothetical protein CBG16019
Locus_11378_Transcript_1/1_Conf_1.000	233	0							
Locus_11379_Transcript_1/1_Conf_1.000	299	20	6.16E-44	NP_502268.1	89	180.259	99	89	TRNA Guanine Transglycosylase family member (tgt-1)
Locus_1138_Transcript_1/1_Conf_1.000	1471	20	0	XP_002640255.1	86	706.442	435	378	C. briggsae CBR-UNC-87 protein
Locus_11380_Transcript_1/1_Conf_1.000	252	9	2.25E-09	XP_001896064.1	69	65.4698	59	41	hypothetical protein Bm1_23035
Locus_11381_Transcript_1/1_Conf_1.000	361	3	1.02E-14	NP_491456.2	70	83.1889	75	53	hypothetical protein F57C9.6
Locus_11382_Transcript_1/1_Conf_1.000	354	2	4.31E-05	NP_508627.5	74	51.2174	39	29	Sensory AXon guidance family member (sax-1)
Locus_11383_Transcript_1/1_Conf_1.000	327	6	9.25E-16	NP_490834.1	60	86.6557	100	60	hypothetical protein Y48G8AL.1
Locus_11384_Transcript_1/1_Conf_1.000	198	0							
Locus_11385_Transcript_1/1_Conf_1.000	652	20	5.56E-79	XP_002633800.1	81	297.745	219	178	C. briggsae CBR-RBD-1 protein
Locus_11386_Transcript_1/1_Conf_1.000	1033	0							
Locus_11387_Transcript_1/1_Conf_1.000	568	20	5.10E-71	NP_493701.1	80	270.781	189	152	hypothetical protein K01A2.5

Locus_11388_Transcript_1/1_Conf_1.000	239	8	9.85E-26	NP_001040987.1	84	119.783	79	67	ALP/Enigma encoding family member (alp-1)
Locus_11389_Transcript_1/1_Conf_1.000	589	20	2.37E-37	NP_503059.2	88	159.073	92	81	hypothetical protein ZK550.3
Locus_1139_Transcript_1/1_Conf_1.000	1014	20	4.79E-102	EFO22864.1	81	375.555	274	223	3'-phosphoadenosine 5'-phosphosulfate synthase 2
Locus_11390_Transcript_1/1_Conf_1.000	178	0							
Locus_11391_Transcript_1/1_Conf_1.000	309	20	5.15E-35	XP_001900807.1	84	150.599	102	86	peptidyl-prolyl cis-trans isomerase, FKBP-type family protein
Locus_11392_Transcript_1/1_Conf_1.000	546	20	2.91E-41	XP_002646691.1	78	171.785	134	105	Hypothetical protein CBG13068
Locus_11393_Transcript_1/1_Conf_1.000	639	20	1.76E-90	EFO23380.1	92	335.88	193	178	BTB/POZ domain-containing protein
Locus_11394_Transcript_1/1_Conf_1.000	609	0							
Locus_11395_Transcript_1/1_Conf_1.000	289	0							
Locus_11396_Transcript_1/1_Conf_1.000	354	20	1.37E-43	XP_002630114.1	86	179.104	111	96	C. briggsae CBR-PYR-1 protein
Locus_11397_Transcript_1/1_Conf_1.000	243	1	2.42E-08	CAR63594.1	62	62.003	79	49	hypothetical protein
Locus_11398_Transcript_1/1_Conf_1.000	2886	20	1.65E-158	NP_510290.1	63	565.074	807	511	Germinal Center Kinase family member (gck-4)
Locus_11399_Transcript_1/1_Conf_1.000	787	0							
Locus_114_Transcript_1/1_Conf_1.000	1512	20	1.35E-123	NP_497506.3	64	447.973	540	346	hypothetical protein Y48G9A.3

Locus_1140_Transcript_1/1_Conf_1.000	961	20	1.04E-95	XP_002634374.1	100	354.369	177	177	C. briggsae CBR-ARF-3 protein
Locus_11400_Transcript_1/1_Conf_1.000	134	0							
Locus_11401_Transcript_1/1_Conf_1.000	1973	20	0	NP_499392.1	82	837.025	625	516	OXidative stress Induced family member (oxi-1)
Locus_11402_Transcript_1/1_Conf_1.000	562	20	1.13E-91	AAT02163.1	96	339.347	176	169	hypothetical protein L3ni56 isoform a
Locus_11403_Transcript_1/1_Conf_1.000	397	0							
Locus_11404_Transcript_1/1_Conf_1.000	485	0							
Locus_11405_Transcript_1/1_Conf_1.000	664	20	3.23E-98	XP_002646363.1	90	361.688	220	200	Hypothetical protein CBG12078
Locus_11406_Transcript_1/1_Conf_1.000	441	2	1.43E-48	CAR63706.1	93	195.667	138	129	putative FOS (B-Zip transcription factor) homolog family member
Locus_11407_Transcript_1/1_Conf_1.000	565	20	3.30E-22	EFO23808.1	61	108.612	136	84	hypothetical protein LOAG_04674
Locus_11408_Transcript_1/1_Conf_1.000	478	5	1.24E-04	XP_805431.1	43	49.6766	112	49	hypothetical protein
Locus_11409_Transcript_1/1_Conf_1.000	1904	20	0	NP_504245.3	72	738.799	636	464	hypothetical protein Y61A9LA.10
Locus_1141_Transcript_1/1_Conf_1.000	259	0							
Locus_11410_Transcript_1/1_Conf_1.000	613	20	4.47E-93	XP_001896600.1	93	344.354	183	172	peptidyl-prolyl cis-trans isomerase 11
Locus_11411_Transcript_1/1_Conf_1.000	363	0							
Locus_11412_Transcript_1/1_Conf_1.000	855	20	2.09E-25	CAZ65529.1	69	120.553	133	92	C. elegans protein T28C6.7b, partially confirmed by transcript evidence

Locus_11413_Transcript_1/1_Conf_1.000	523	20	5.46E-44	EFO22205.1	94	180.644	113	107	small Nuclear Ribonucleoprotein family member
Locus_11414_Transcript_1/1_Conf_1.000	673	20	3.30E-29	XP_002642913.1	58	132.494	176	103	C. briggsae CBR-SMI-1 protein
Locus_11415_Transcript_1/1_Conf_1.000	1268	9	6.57E-25	ABJ99064.1	44	119.783	427	192	Hypothetical protein W03A3.2
Locus_11416_Transcript_1/1_Conf_1.000	341	4	4.82E-25	XP_002635120.1	67	117.472	110	74	Hypothetical protein CBG11342
Locus_11417_Transcript_1/1_Conf_1.000	998	20	3.96E-85	XP_002631495.1	68	319.316	329	226	Hypothetical protein CBG20655
Locus_11418_Transcript_1/1_Conf_1.000	573	5	3.23E-36	EFO27097.1	72	155.221	131	95	hypothetical protein LOAG_01386
Locus_11419_Transcript_1/1_Conf_1.000	569	6	8.80E-31	NP_001122953.1	73	137.117	142	104	hypothetical protein F53H10.2
Locus_1142_Transcript_1/1_Conf_1.000	1041	20	2.18E-65	XP_001891854.1	65	253.832	353	230	Conserved oligomeric Golgi complex component 4
Locus_11420_Transcript_1/1_Conf_1.000	630	3	1.78E-31	NP_508493.1	60	139.813	215	131	protein KINase family member (kin-29)
Locus_11421_Transcript_1/1_Conf_1.000	326	20	8.40E-17	XP_001896013.1	66	90.1225	115	76	Protein kinase domain containing protein
Locus_11422_Transcript_1/1_Conf_1.000	256	20	5.31E-27	NP_001041301.1	82	124.02	85	70	hypothetical protein ZK54.1
Locus_11423_Transcript_1/1_Conf_1.000	240	0							
Locus_11424_Transcript_1/1_Conf_1.000	1067	20	1.45E-96	NP_510510.1	73	357.451	282	208	DHHC-types zinc finger transcription factor family member (dhhc-1)
Locus_11425_Transcript_1/1_Conf_1.000	142	0							
Locus_11426_Transcript_1/1_Conf_1.000	491	20	1.47E-45	NP_509186.3	72	185.652	165	120	Carbonic AnHydrase family member (cah-5)
Locus_11427_Transcript_1/1_Conf_1.000	133	0							
Locus_11428_Transcript_1/1_Conf_1.000	271	0							
Locus_11429_Transcript_1/1_Conf_1.000	289	0							

Locus_1143_Transcript_1/1_Conf_1.000	1662	20	1.56E-51	EFO23026.1	46	208.764	447	208	MYND finger family protein
Locus_11430_Transcript_1/1_Conf_1.000	481	0							
Locus_11431_Transcript_1/1_Conf_1.000	239	0							
Locus_11432_Transcript_1/1_Conf_1.000	299	0							
Locus_11433_Transcript_1/1_Conf_1.000	1881	20	0	XP_001896664.1	85	874.389	583	496	Protein kinase domain containing protein
Locus_11434_Transcript_1/1_Conf_1.000	908	20	3.88E-73	CAE12199.1	64	279.256	286	185	aspartyl protease precursor
Locus_11435_Transcript_1/1_Conf_1.000	854	20	3.38E-76	NP_508767.1	99	289.271	142	141	AdaPtin, Small chain (clathrin associated complex) family member (aps-2)
Locus_11436_Transcript_1/1_Conf_1.000	337	20	6.34E-17	Q61CX7.2	63	90.5077	96	61	Protein lin-28
Locus_11437_Transcript_1/1_Conf_1.000	268	4	2.07E-07	NP_498921.3	64	58.9214	88	57	hypothetical protein B0303.11
Locus_11438_Transcript_1/1_Conf_1.000	936	20	1.11E-102	EFO20614.1	80	377.481	302	243	hypothetical protein LOAG_07877

Locus_11439_Transcript_1/1_Conf_1.000	379	0								
Locus_1144_Transcript_1/1_Conf_1.000	856	20	3.76E-68	NP_497103.1	75	232.646	244	185	hypothetical protein Y53F4B.18	
Locus_11440_Transcript_1/2_Conf_1.000	350	0								
Locus_11440_Transcript_2/2_Conf_1.000	683	0								
Locus_11441_Transcript_1/1_Conf_1.000	942	20	5.78E-91	CAR97821.1	75	338.576	273	206	C. elegans protein F11C1.5d, partially confirmed by transcript evidence	
Locus_11442_Transcript_1/2_Conf_1.000	357	0								
Locus_11442_Transcript_2/2_Conf_1.000	300	0								
Locus_11443_Transcript_1/1_Conf_1.000	528	20	3.69E-51	XP_002636692.1	72	204.527	158	115	Hypothetical protein CBG23406	
Locus_11444_Transcript_1/1_Conf_1.000	376	0								
Locus_11445_Transcript_1/1_Conf_1.000	1420	20	0	CAR64255.1	86	659.062	454	391	protein RIO1 kinase	
Locus_11446_Transcript_1/1_Conf_1.000	570	20	6.16E-56	NP_497037.1	91	220.705	141	129	ARF-Like family member (arl-3)	
Locus_11447_Transcript_1/1_Conf_1.000	662	8	4.10E-45	XP_002639056.1	68	185.267	170	117	C. briggsae CBR-HOT-5 protein	
Locus_11448_Transcript_1/1_Conf_1.000	1248	20	0	XP_001895795.1	92	640.573	371	343	actin 1	
Locus_11449_Transcript_1/1_Conf_1.000	460	20	1.59E-52	NP_491634.1	85	208.764	154	131	hypothetical protein M01E11.2	
Locus_1145_Transcript_1/2_Conf_1.000	777	4	2.29E-17	NP_504957.2	55	93.5893	164	91	hypothetical protein C50E3.5	

Locus_1145_Transcript_2/2_Conf_1.000	1739	6	8.99E-50	NP_504957.2	54	202.986	396	217	hypothetical protein C50E3.5
Locus_11450_Transcript_1/1_Conf_1.000	474	0							
Locus_11451_Transcript_1/1_Conf_1.000	616	20	2.69E-53	AAX29387.1	100	212.231	156	156	ribosomal protein L29
Locus_11452_Transcript_1/1_Conf_1.000	741	5	1.56E-04	XP_001950169.1	38	50.8322	144	56	PREDICTED: similar to replication factors MCM
Locus_11453_Transcript_1/1_Conf_1.000	270	0							
Locus_11454_Transcript_1/1_Conf_1.000	399	0							
Locus_11455_Transcript_1/1_Conf_1.000	414	20	1.27E-25	XP_001233556.1	62	119.398	140	87	PREDICTED: hypothetical protein
Locus_11456_Transcript_1/1_Conf_1.000	513	20	6.42E-63	NP_871854.1	87	243.432	167	146	SCP(Small C-terminal domain Phosphatase)-Like phosphatase family member (scpl-3)
Locus_11457_Transcript_1/1_Conf_1.000	537	20	9.35E-74	NP_498688.1	84	279.641	178	151	Prolyl Carboxy Peptidase like family member (pcp-1)
Locus_11458_Transcript_1/1_Conf_1.000	189	5	7.96E-07	NP_506519.1	72	56.9954	51	37	hypothetical protein T16A9.5
Locus_11459_Transcript_1/1_Conf_1.000	237	0							
Locus_1146_Transcript_1/1_Conf_1.000	969	0							
Locus_11460_Transcript_1/1_Conf_1.000	914	20	2.21E-100	XP_001893912.1	81	369.777	274	222	mRNA capping enzyme, C-terminal domain containing protein
Locus_11461_Transcript_1/1_Conf_1.000	174	0							
Locus_11462_Transcript_1/1_Conf_1.000	942	4	1.31E-18	A8WZU5.2	48	98.2117	290	141	Fanconi-associated nuclease 1 homolog
Locus_11463_Transcript_1/1_Conf_1.000	421	20	6.46E-25	NP_001040864.1	69	117.087	107	74	SMAll family member (sma-4)
Locus_11464_Transcript_1/1_Conf_1.000	1249	20	3.51E-63	XP_001897220.1	58	246.899	397	232	P120
Locus_11465_Transcript_1/1_Conf_1.000	365	4	2.75E-12	XP_001895046.1	62	75.0998	118	74	hypothetical protein
Locus_11466_Transcript_1/1_Conf_1.000	225	0							

Locus_11467_Transcript_1/1_Conf_1.000	152	0							
Locus_11468_Transcript_1/1_Conf_1.000	414	20	2.18E-09	XP_002638816.1	55	65.4698	99	55	Hypothetical protein CBG22019
Locus_11469_Transcript_1/1_Conf_1.000	140	0							
Locus_1147_Transcript_1/2_Conf_1.000	375	20	2.88E-33	ABA00169.1	66	144.821	124	83	Plexin protein 1
Locus_1147_Transcript_2/2_Conf_1.000	375	20	2.88E-33	ABA00169.1	66	144.821	124	83	Plexin protein 1
Locus_11470_Transcript_1/1_Conf_1.000	650	20	3.71E-51	XP_002636678.1	66	205.297	216	144	C. briggsae CBR-CHE-11 protein
Locus_11471_Transcript_1/1_Conf_1.000	412	20	1.24E-12	NP_001020168.1	49	76.2554	159	79	coiled-coil domain-containing protein 93
Locus_11472_Transcript_1/1_Conf_1.000	1419	20	1.43E-79	XP_002645548.1	55	301.597	467	260	Hypothetical protein CBG05229
Locus_11473_Transcript_1/1_Conf_1.000	1166	20	7.30E-52	XP_001893792.1	50	209.149	375	189	Zinc finger, C2H2 type family protein
Locus_11474_Transcript_1/1_Conf_1.000	740	20	2.06E-41	XP_001900557.1	51	173.326	277	143	Bromodomain containing protein
Locus_11475_Transcript_1/1_Conf_1.000	357	0							
Locus_11476_Transcript_1/1_Conf_1.000	1183	20	2.21E-104	NP_501491.3	75	383.645	301	228	Heparan SulphoTransferase family member (hst-1)
Locus_11477_Transcript_1/1_Conf_1.000	239	8	9.26E-08	XP_002636450.1	75	60.077	48	36	Hypothetical protein CBG23111
Locus_11478_Transcript_1/1_Conf_1.000	177	0							
Locus_11479_Transcript_1/1_Conf_1.000	359	0							
Locus_1148_Transcript_1/1_Conf_1.000	324	0							

Locus_11480_Transcript_1/1_Conf_1.000	987	20	5.99E-94	XP_002632141.1	69	348.591	309	215	C. briggsae CBR-TRE-5 protein
Locus_11481_Transcript_1/1_Conf_1.000	284	2	2.48E-16	XP_002631194.1	78	88.5817	66	52	C. briggsae CBR-NLP-18 protein
Locus_11482_Transcript_1/2_Conf_1.000	501	0							
Locus_11482_Transcript_2/2_Conf_1.000	501	0							
Locus_11483_Transcript_1/2_Conf_1.000	480	20	2.56E-26	EFO22039.1	64	121.709	146	94	hypothetical protein LOAG_06449
Locus_11483_Transcript_2/2_Conf_1.000	480	20	3.34E-26	EFO22039.1	64	121.324	146	94	hypothetical protein LOAG_06449
Locus_11484_Transcript_1/1_Conf_1.000	841	0							
Locus_11485_Transcript_1/1_Conf_1.000	178	2	1.01E-06	XP_002630757.1	66	56.6102	59	39	Hypothetical protein CBG02451
Locus_11486_Transcript_1/1_Conf_1.000	193	0							
Locus_11487_Transcript_1/1_Conf_1.000	893	20	5.09E-62	ACI49172.1	67	242.276	262	176	hypothetical protein Csp3_JD01.004
Locus_11488_Transcript_1/1_Conf_1.000	385	20	3.72E-33	NP_490883.1	100	144.436	74	74	LSM Sm-like protein family member (lsm-6)

Locus_11489_Transcript_1/1_Conf_1.000	301	2	6.63E-06	NP_495103.1	69	53.9138	55	38	ZYGote defective: embryonic lethal family member (zyg-1)
Locus_1149_Transcript_1/1_Conf_1.000	1404	20	2.92E-85	NP_494767.1	58	320.472	466	273	hypothetical protein H20J04.2
Locus_11490_Transcript_1/1_Conf_1.000	441	20	4.61E-39	XP_002638772.1	73	164.081	142	105	C. briggsae CBR-PTR-21 protein
Locus_11491_Transcript_1/1_Conf_1.000	478	20	1.31E-38	XP_002643084.1	76	162.54	119	91	C. briggsae CBR-TAG-174 protein
Locus_11492_Transcript_1/1_Conf_1.000	689	20	8.68E-97	ABS50363.1	91	357.066	229	210	LIM domain protein variant
Locus_11493_Transcript_1/1_Conf_1.000	494	0							
Locus_11494_Transcript_1/1_Conf_1.000	580	0							
Locus_11495_Transcript_1/1_Conf_1.000	297	20	3.89E-22	NP_001024903.1	67	107.842	93	63	abnormal DAuer Formation family member (daf-9)
Locus_11496_Transcript_1/1_Conf_1.000	195	20	2.21E-25	XP_002640668.1	96	118.627	64	62	C. briggsae CBR-UNC-54 protein
Locus_11497_Transcript_1/1_Conf_1.000	448	4	4.92E-17	NP_740830.2	47	90.8929	184	88	hypothetical protein Y47G6A.29
Locus_11498_Transcript_1/1_Conf_1.000	146	0							
Locus_11499_Transcript_1/1_Conf_1.000	187	0							
Locus_115_Transcript_1/6_Conf_0.235	1107	20	0	CAR63627.1	99	652.129	326	324	putative Elongation FacTor

Locus_115_Transcript_2/6_Conf_0.235	770	20	5.37E-96	NP_001021266.1	72	354.755	306	222	UNCoordinated family member (unc-44)
Locus_115_Transcript_3/6_Conf_0.412	3662	20	0	NP_001021268.1	77	1289.63	1034	802	UNCoordinated family member (unc-44)
Locus_115_Transcript_4/6_Conf_0.118	1655	5	9.39E-41	NP_001021266.1	65	172.94	283	185	UNCoordinated family member (unc-44)
Locus_115_Transcript_5/6_Conf_0.412	3644	20	0	NP_500898.1	77	1278.46	1028	796	UNCoordinated family member (unc-44)
Locus_115_Transcript_6/6_Conf_0.353	2780	20	0	NP_492457.1	96	1558.12	852	824	Elongation FacTor family member (eft-2)
Locus_1150_Transcript_1/3_Conf_0.750	2164	2	6.08E-22	XP_002635564.1	44	110.923	347	156	C. briggsae CBR-TAM-1 protein
Locus_1150_Transcript_2/3_Conf_0.750	2188	2	6.17E-22	XP_002635564.1	44	110.923	347	156	C. briggsae CBR-TAM-1 protein
Locus_1150_Transcript_3/3_Conf_0.750	2176	2	6.13E-22	XP_002635564.1	44	110.923	347	156	C. briggsae CBR-TAM-1 protein
Locus_11500_Transcript_1/2_Conf_1.000	857	20	3.13E-13	XP_002646949.1	52	80.1073	177	93	C. briggsae CBR-APD-3 protein
Locus_11500_Transcript_2/2_Conf_1.000	670	20	1.47E-13	XP_002646949.1	52	80.4925	177	93	C. briggsae CBR-APD-3 protein
Locus_11501_Transcript_1/1_Conf_1.000	602	3	3.90E-22	NP_491469.2	52	108.612	200	104	hypothetical protein F25F8.1
Locus_11502_Transcript_1/2_Conf_1.000	830	20	2.27E-106	NP_001022869.1	84	389.423	256	217	hypothetical protein Y42G9A.4

Locus_11502_Transcript_2/2_Conf_1.000	767	20	7.67E-111	NP_001022869.1	92	404.06	235	218	hypothetical protein Y42G9A.4
Locus_11503_Transcript_1/1_Conf_1.000	512	20	1.20E-21	NP_001024866.1	55	106.301	177	98	hypothetical protein R57.1
Locus_11504_Transcript_1/4_Conf_0.333	548	20	4.24E-08	XP_001956624.1	50	61.6178	142	71	GF24494
Locus_11504_Transcript_2/4_Conf_0.333	720	19	3.97E-10	NP_496398.2	43	69.3218	246	107	hypothetical protein T06D8.1
Locus_11504_Transcript_3/4_Conf_0.222	702	0							
Locus_11504_Transcript_4/4_Conf_0.333	378	3	5.10E-06	NP_496398.2	52	54.299	97	51	hypothetical protein T06D8.1
Locus_11505_Transcript_1/1_Conf_1.000	398	0							
Locus_11506_Transcript_1/1_Conf_1.000	281	20	1.59E-52	XP_002930069.1	100	208.764	93	93	PREDICTED: casein kinase II subunit beta-like isoform 1

Locus_11507_Transcript_1/1_Conf_1.000	526	20	3.98E-58	AAF34687.1	81	227.639	175	143	AF223064_1putative microtubule severing protein katanin p60 subunit
Locus_11508_Transcript_1/1_Conf_1.000	816	20	2.24E-42	XP_002117005.1	55	176.792	240	134	hypothetical protein TRIADDRAFT_60996
Locus_11509_Transcript_1/1_Conf_1.000	307	1	4.72E-04	EFA84933.1	58	47.7506	58	34	hypothetical protein PPL_01926
Locus_1151_Transcript_1/3_Conf_0.667	2997	20	1.12E-165	XP_002630883.1	66	588.956	735	486	Hypothetical protein CBG02604
Locus_1151_Transcript_2/3_Conf_0.667	2997	20	6.54E-166	XP_002630883.1	66	589.726	735	487	Hypothetical protein CBG02604
Locus_1151_Transcript_3/3_Conf_0.667	2997	20	2.49E-165	XP_002630883.1	66	587.8	735	486	Hypothetical protein CBG02604
Locus_11510_Transcript_1/1_Conf_1.000	213	0							
Locus_11511_Transcript_1/1_Conf_1.000	227	0							
Locus_11512_Transcript_1/1_Conf_1.000	327	0							
Locus_11513_Transcript_1/2_Conf_1.000	1022	20	9.58E-50	XP_001309767.1	54	201.83	323	176	ankyrin repeat protein
Locus_11513_Transcript_2/2_Conf_1.000	1022	20	9.58E-50	XP_001309767.1	54	201.83	323	176	ankyrin repeat protein

Locus_11514_Transcript_1/1_Conf_1.000	828	20	6.47E-61	NP_499648.3	71	238.424	228	162	ELongator complex Protein Component family member (elpc-2)
Locus_11515_Transcript_1/1_Conf_1.000	128	0							
Locus_11516_Transcript_1/1_Conf_1.000	781	20	5.87E-21	EFO28351.1	65	105.531	111	73	2Fe-2S iron-sulfur cluster binding domain-containing protein
Locus_11517_Transcript_1/1_Conf_1.000	426	20	5.82E-10	XP_002595762.1	49	67.3958	143	71	hypothetical protein BRAFLDRAFT_200800
Locus_11518_Transcript_1/1_Conf_1.000	263	20	3.11E-21	EDL15956.1	85	63.929	40	34	procollagen, type I, alpha 1, isoform CRA_a
Locus_11519_Transcript_1/1_Conf_1.000	217	0							
Locus_1152_Transcript_1/4_Conf_0.750	1007	20	7.93E-41	AAN05752.1	71	172.17	147	105	heat shock protein 20
Locus_1152_Transcript_2/4_Conf_0.750	1007	20	1.00E-43	CAG25499.1	73	181.8	157	115	heat shock protein 20
Locus_1152_Transcript_3/4_Conf_0.450	584	20	3.38E-44	CAG25499.1	73	181.8	157	115	heat shock protein 20
Locus_1152_Transcript_4/4_Conf_0.750	993	20	9.81E-44	CAG25499.1	73	181.8	157	115	heat shock protein 20
Locus_11520_Transcript_1/2_Conf_1.000	426	0							
Locus_11520_Transcript_2/2_Conf_1.000	455	0							
Locus_11521_Transcript_1/1_Conf_1.000	337	0							
Locus_11522_Transcript_1/1_Conf_1.000	144	0							
Locus_11523_Transcript_1/1_Conf_1.000	619	3	2.18E-18	NP_498056.2	57	96.2857	141	81	hypothetical protein C27F2.7
Locus_11524_Transcript_1/1_Conf_1.000	293	20	3.64E-44	ACC44546.1	96	181.03	97	94	SPC-1
Locus_11525_Transcript_1/1_Conf_1.000	196	0							

Locus_11526_Transcript_1/1_Conf_1.000	227	20	1.10E-24	XP_002637346.1	84	116.316	75	63	Hypothetical protein CBG19044
Locus_11527_Transcript_1/1_Conf_1.000	426	0							
Locus_11528_Transcript_1/1_Conf_1.000	1200	20	1.01E-19	AAP48996.1	41	102.449	395	162	cellulosomal scaffoldin anchoring protein C
Locus_11529_Transcript_1/1_Conf_1.000	227	0							
Locus_1153_Transcript_1/1_Conf_1.000	784	4	4.38E-24	NP_508979.2	48	115.931	233	112	DAF-16/FOXO Controlled, germline Tumor affecting family member (dct-11)
Locus_11530_Transcript_1/1_Conf_1.000	595	5	2.82E-09	NP_505962.2	42	65.855	198	85	Nuclear Pore complex Protein family member (npp-22)
Locus_11531_Transcript_1/1_Conf_1.000	253	0							
Locus_11532_Transcript_1/1_Conf_1.000	197	20	6.47E-09	EFO25376.1	69	63.929	65	45	hypothetical protein LOAG_03111
Locus_11533_Transcript_1/1_Conf_1.000	534	0							
Locus_11534_Transcript_1/1_Conf_1.000	832	20	1.85E-31	NP_508153.1	58	140.584	178	105	hypothetical protein T04G9.4
Locus_11535_Transcript_1/1_Conf_1.000	570	20	2.05E-51	NP_504500.1	79	205.682	133	106	C-type LECtin family member (clec-1)
Locus_11536_Transcript_1/1_Conf_1.000	743	20	1.31E-27	NP_510604.1	95	127.487	65	62	hypothetical protein F59F4.2
Locus_11537_Transcript_1/1_Conf_1.000	305	1	6.19E-04	CAH23216.1	45	47.3654	97	44	putative L3 ES protein

Locus_11538_Transcript_1/1_Conf_1.000	594	20	9.92E-39	XP_002640158.1	86	163.696	110	95	C. briggsae CBR-TRE-1 protein
Locus_11539_Transcript_1/1_Conf_1.000	310	20	2.92E-30	XP_002645186.1	89	134.806	102	91	Hypothetical protein CBG16916
Locus_1154_Transcript_1/1_Conf_1.000	335	13	8.83E-35	NP_492132.1	79	149.828	108	86	Enhancer of Glp-One (glp-1) family member (ego-1)
Locus_11540_Transcript_1/1_Conf_1.000	262	20	4.93E-33	EFO21345.1	90	144.05	87	79	hypothetical protein LOAG_07143
Locus_11541_Transcript_1/1_Conf_1.000	151	0							
Locus_11542_Transcript_1/1_Conf_1.000	324	0							
Locus_11543_Transcript_1/1_Conf_1.000	790	20	2.29E-12	EFO26136.1	53	77.0258	195	104	hypothetical protein LOAG_02353
Locus_11544_Transcript_1/1_Conf_1.000	267	0							
Locus_11545_Transcript_1/1_Conf_1.000	138	15	1.84E-16	EAW72008.1	100	88.9669	39	39	natural killer cell group 7 sequence, isoform CRA_c
Locus_11546_Transcript_1/1_Conf_1.000	518	0							
Locus_11547_Transcript_1/1_Conf_1.000	237	0							
Locus_11548_Transcript_1/1_Conf_1.000	715	20	1.62E-48	XP_002637050.1	73	196.823	183	135	Hypothetical protein CBG09545
Locus_11549_Transcript_1/1_Conf_1.000	888	0							
Locus_1155_Transcript_1/1_Conf_1.000	519	20	3.46E-43	NP_492582.1	78	177.948	127	100	Farnesylated-proteins Converting Enzyme (FACE) family member (fce-1)
Locus_11550_Transcript_1/1_Conf_1.000	1195	20	1.16E-76	XP_002636710.1	67	291.582	317	214	C. briggsae CBR-TSFM-1 protein
Locus_11551_Transcript_1/1_Conf_1.000	295	0							
Locus_11552_Transcript_1/1_Conf_1.000	405	0							
Locus_11553_Transcript_1/1_Conf_1.000	388	20	2.67E-15	NP_495088.1	55	85.1149	127	71	hypothetical protein C17G10.1
Locus_11554_Transcript_1/1_Conf_1.000	582	20	6.19E-30	NP_490812.1	89	134.42	104	93	GLutaRedoXin family member (glrx-10)

Locus_11555_Transcript_1/1_Conf_1.000	1338	20	3.00E-132	XP_002633301.1	82	476.478	342	283	Hypothetical protein CBG06032
Locus_11556_Transcript_1/1_Conf_1.000	190	0							
Locus_11557_Transcript_1/1_Conf_1.000	225	0							
Locus_11558_Transcript_1/1_Conf_1.000	1590	20	6.37E-47	XP_002640788.1	45	193.356	552	252	C. briggsae CBR-FBXA-1 protein
Locus_11559_Transcript_1/1_Conf_1.000	262	20	2.70E-31	XP_001896078.1	90	138.272	83	75	A-macroglobulin complement component family protein
Locus_1156_Transcript_1/1_Conf_1.000	412	0							
Locus_11560_Transcript_1/1_Conf_1.000	777	12	2.84E-84	AAA96224.2	73	315.849	245	181	Hypothetical protein T07H6.4
Locus_11561_Transcript_1/1_Conf_1.000	143	9	2.74E-12	NP_001122601.1	91	75.0998	47	43	Multiple PDZ domain protein family member (mpz-1)
Locus_11562_Transcript_1/1_Conf_1.000	782	20	2.31E-134	AAX28980.1	100	482.256	243	243	ribosomal protein S3
Locus_11563_Transcript_1/1_Conf_1.000	294	20	1.39E-11	XP_002637534.1	64	72.7886	88	57	C. briggsae CBR-TRY-5 protein
Locus_11564_Transcript_1/1_Conf_1.000	1581	20	3.22E-75	EFO23010.1	56	287.345	536	305	hypothetical protein LOAG_05474

Locus_11565_Transcript_1/1_Conf_1.000	580								
Locus_11566_Transcript_1/1_Conf_1.000	424	20	2.60E-34	EFO19620.1	81	148.288	111	91	histone acetyltransferase
Locus_11567_Transcript_1/1_Conf_1.000	793	0							
Locus_11568_Transcript_1/1_Conf_1.000	674	20	3.72E-73	XP_002644648.1	79	278.485	224	179	Hypothetical protein CBG14624
Locus_11569_Transcript_1/1_Conf_1.000	1232	20	4.45E-95	NP_497827.1	75	352.829	282	213	GLyOxalase Domain containing family member (glod-4)
Locus_1157_Transcript_1/3_Conf_0.714	1297	2	2.51E-11	NP_499710.1	59	74.7146	148	88	hypothetical protein Y39E4B.6
Locus_1157_Transcript_2/3_Conf_0.571	1513	2	3.10E-11	NP_499710.1	59	74.7146	148	88	hypothetical protein Y39E4B.6
Locus_1157_Transcript_3/3_Conf_0.571	1235	20	2.12E-12	NP_499710.1	57	78.1814	215	124	hypothetical protein Y39E4B.6
Locus_11570_Transcript_1/1_Conf_1.000	713	0							
Locus_11571_Transcript_1/1_Conf_1.000	788	20	1.18E-77	XP_002637336.1	79	293.893	215	170	Hypothetical protein CBG19031
Locus_11572_Transcript_1/1_Conf_1.000	227	0							
Locus_11573_Transcript_1/1_Conf_1.000	157	0							
Locus_11574_Transcript_1/2_Conf_1.000	586	20	1.23E-17	NP_495042.2	50	93.5893	165	83	hypothetical protein F56D1.1
Locus_11574_Transcript_2/2_Conf_1.000	586	20	2.48E-18	XP_002630725.1	46	95.9005	156	73	Hypothetical protein CBG02409
Locus_11575_Transcript_1/1_Conf_1.000	437	20	1.79E-11	XP_002641852.1	77	72.4034	49	38	C. briggsae CBR-LARP-1 protein
Locus_11576_Transcript_1/1_Conf_1.000	215	0							
Locus_11577_Transcript_1/1_Conf_1.000	809	20	8.82E-124	XP_002630114.1	87	447.203	269	236	C. briggsae CBR-PYR-1 protein
Locus_11578_Transcript_1/2_Conf_1.000	479	2	1.62E-04	ZP_05026437.1	53	49.2914	113	61	DNA sulfur modification protein DndD
Locus_11578_Transcript_2/2_Conf_1.000	479	1	3.61E-04	XP_689920.3	54	48.1358	91	50	PREDICTED: cingulin-like
Locus_11579_Transcript_1/1_Conf_1.000	251	0							

Locus_1158_Transcript_1/1_Conf_1.000	762	20	2.88E-118	NP_491363.1	91	428.713	253	232	Dynein Heavy Chain family member (dhc-1)
Locus_11580_Transcript_1/1_Conf_1.000	289	10	2.73E-07	XP_002641560.1	51	58.5362	96	49	Hypothetical protein CBG09859
Locus_11581_Transcript_1/1_Conf_1.000	680	0							
Locus_11582_Transcript_1/1_Conf_1.000	204	0							
Locus_11583_Transcript_1/1_Conf_1.000	682	0							
Locus_11584_Transcript_1/1_Conf_1.000	727	20	1.01E-85	A8XKG6.2	83	320.472	242	203	DPYD_CAEBRRecName: Full=Probable dihydropyrimidine dehydrogenase
Locus_11585_Transcript_1/1_Conf_1.000	513	6	9.35E-30	CAR97833.1	69	133.265	134	93	C. elegans protein F46G10.5b, partially confirmed by transcript evidence
Locus_11586_Transcript_1/1_Conf_1.000	279	20	2.39E-32	XP_002639664.1	86	141.739	92	80	C. briggsae CBR-HUM-1 protein
Locus_11587_Transcript_1/1_Conf_1.000	274	0							
Locus_11588_Transcript_1/1_Conf_1.000	225	0							
Locus_11589_Transcript_1/1_Conf_1.000	225	2	5.88E-10	NP_492200.1	64	67.3958	71	46	hypothetical protein C54G4.5
Locus_1159_Transcript_1/1_Conf_1.000	1346	4	4.33E-38	NP_499275.2	63	163.696	242	154	hypothetical protein K10G9.2
Locus_11590_Transcript_1/1_Conf_1.000	208	0							
Locus_11591_Transcript_1/1_Conf_1.000	364	18	6.74E-35	NP_741076.1	78	150.214	120	94	GEX Interacting protein family member (gei-16)
Locus_11592_Transcript_1/1_Conf_1.000	773	20	7.15E-64	XP_002630082.1	90	248.054	153	138	Hypothetical protein CBG13461

Locus_11593_Transcript_1/1_Conf_1.000	275	20	1.08E-24	XP_002634119.1	84	116.316	71	60	Hypothetical protein CBG01673
Locus_11594_Transcript_1/1_Conf_1.000	370	20	1.49E-29	XP_002640278.1	85	132.494	85	73	Hypothetical protein CBG12804
Locus_11595_Transcript_1/1_Conf_1.000	365	20	5.00E-30	NP_498090.1	95	134.035	84	80	Temporarily Assigned Gene name family member (tag-262)
Locus_11596_Transcript_1/1_Conf_1.000	613	20	9.52E-19	XP_973445.1	51	97.4413	195	101	PREDICTED: similar to vesicular mannose-binding lectin
Locus_11597_Transcript_1/1_Conf_1.000	683	0							
Locus_11598_Transcript_1/1_Conf_1.000	179	0							
Locus_11599_Transcript_1/1_Conf_1.000	1092	20	1.34E-52	XP_002635267.1	69	211.46	266	185	C. briggsae CBR-LETM-1 protein
Locus_116_Transcript_1/1_Conf_1.000	550	20	3.26E-56	NP_498030.1	79	221.476	183	145	hypothetical protein F26F4.12
Locus_1160_Transcript_1/2_Conf_1.000	386	20	6.59E-14	EFO23006.1	70	80.4925	87	61	mast family protein
Locus_1160_Transcript_2/2_Conf_1.000	1120	20	4.47E-59	XP_001900462.1	74	233.032	222	165	CLASP1
Locus_11600_Transcript_1/1_Conf_1.000	497	1	5.59E-05	XP_001476928.1	53	50.8322	97	52	PREDICTED: hypothetical protein
Locus_11601_Transcript_1/1_Conf_1.000	201	0							
Locus_11602_Transcript_1/1_Conf_1.000	496	20	1.96E-42	XP_001902364.1	73	175.252	159	117	Myotubularin-related family protein
Locus_11603_Transcript_1/1_Conf_1.000	157	0							

Locus_11604_Transcript_1/1_Conf_1.000	351	7	4.30E-29	NP_001022438.1	87	130.954	97	85	Ligand-Gated ion Channel family member (lgc-35)
Locus_11605_Transcript_1/1_Conf_1.000	213	20	2.66E-15	NP_501562.1	73	85.1149	67	49	hypothetical protein C53B4.6
Locus_11606_Transcript_1/1_Conf_1.000	183	20	1.83E-27	2XDU	100	125.561	60	60	Structre Of Hsp90 With Small Molecule Inhibitor Bound
Locus_11607_Transcript_1/1_Conf_1.000	438	2	2.40E-08	NP_491834.1	55	62.003	101	56	MUTator family member (mut-2)
Locus_11608_Transcript_1/1_Conf_1.000	1236	20	5.27E-112	XP_002640687.1	75	409.068	352	264	Hypothetical protein CBG19749
Locus_11609_Transcript_1/1_Conf_1.000	348	20	5.58E-61	2I07	100	236.884	115	115	Human Complement Component C3b
Locus_1161_Transcript_1/1_Conf_1.000	881	20	1.63E-113	NP_001122649.1	77	413.305	294	229	abnormal cell LINeage family member (lin-29)
Locus_11610_Transcript_1/1_Conf_1.000	586	20	2.44E-66	NP_493947.1	80	255.373	194	157	hypothetical protein F56D12.6
Locus_11611_Transcript_1/1_Conf_1.000	488	20	2.79E-28	XP_001601941.1	66	128.257	136	91	PREDICTED: similar to rCG44212

Locus_11612_Transcript_1/1_Conf_1.000	358	20	3.73E-33	NP_001122663.1	73	144.436	115	85	hypothetical protein Y53F4B.39
Locus_11613_Transcript_1/1_Conf_1.000	409	20	4.86E-33	EFO26089.1	82	144.05	98	81	hypothetical protein LOAG_02401
Locus_11614_Transcript_1/1_Conf_1.000	369	20	3.01E-22	NP_505803.1	78	108.227	114	90	hypothetical protein F53B7.3
Locus_11615_Transcript_1/1_Conf_1.000	1161	20	1.48E-137	NP_491777.3	83	493.812	330	277	hypothetical protein T09B4.8
Locus_11616_Transcript_1/1_Conf_1.000	412	0							
Locus_11617_Transcript_1/1_Conf_1.000	149	20	2.40E-19	XP_002930267.1	100	98.5969	49	49	PREDICTED: histone H2A type 2-B-like isoform 1
Locus_11618_Transcript_1/1_Conf_1.000	233	3	1.59E-07	XP_002647560.1	69	59.3066	66	46	Hypothetical protein CBG06646
Locus_11619_Transcript_1/1_Conf_1.000	265	20	3.91E-14	XP_418636.2	80	81.2629	61	49	PREDICTED: similar to ABC transporter protein; P-glycoprotein
Locus_1162_Transcript_1/1_Conf_1.000	981	20	7.12E-156	XP_002641617.1	95	554.288	326	312	C. briggsae CBR-MOG-1 protein
Locus_11620_Transcript_1/1_Conf_1.000	1363	20	0	NP_509898.1	86	699.893	454	393	ACOnitase family member (aco-1)
Locus_11621_Transcript_1/2_Conf_1.000	378	20	2.50E-45	XP_002643138.1	84	184.882	123	104	Hypothetical protein CBG15316
Locus_11621_Transcript_2/2_Conf_1.000	384	20	2.03E-47	XP_002643138.1	85	191.815	123	105	Hypothetical protein CBG15316
Locus_11622_Transcript_1/1_Conf_1.000	297	20	1.74E-22	NP_499461.1	72	108.997	100	72	hypothetical protein T28D6.4
Locus_11623_Transcript_1/1_Conf_1.000	228	0							
Locus_11624_Transcript_1/1_Conf_1.000	244								
Locus_11625_Transcript_1/1_Conf_1.000	224	0							
Locus_11626_Transcript_1/1_Conf_1.000	138	0							
Locus_11627_Transcript_1/1_Conf_1.000	491	20	1.84E-64	XP_002630183.1	89	248.44	162	145	C. briggsae CBR-FLI-1 protein

Locus_11628_Transcript_1/1_Conf_1.000	230	20	1.95E-05	YP_743833.1	86	52.373	36	31	tRNA (5-carboxymethylaminomethyl-2-thiouridylate) synthase
Locus_11629_Transcript_1/1_Conf_1.000	626	0							
Locus_1163_Transcript_1/1_Conf_1.000	360	0							
Locus_11630_Transcript_1/1_Conf_1.000	132	0							
Locus_11631_Transcript_1/1_Conf_1.000	150	0							
Locus_11632_Transcript_1/1_Conf_1.000	342	2	1.45E-08	XP_002636650.1	52	62.7734	97	51	Hypothetical protein CBG23361
Locus_11633_Transcript_1/1_Conf_1.000	290	4	1.60E-15	XP_002633091.1	74	85.8853	78	58	Hypothetical protein CBG05778
Locus_11634_Transcript_1/1_Conf_1.000	879	20	8.66E-99	NP_500551.2	77	364.385	292	225	hypothetical protein F55F10.1
Locus_11635_Transcript_1/1_Conf_1.000	532	0							
Locus_11636_Transcript_1/1_Conf_1.000	507	16	1.38E-09	XP_002129449.1	50	66.2402	163	83	PREDICTED: hypothetical protein
Locus_11637_Transcript_1/1_Conf_1.000	389	0							
Locus_11638_Transcript_1/1_Conf_1.000	478	20	1.39E-64	XP_002633391.1	90	248.825	160	144	Hypothetical protein CBG06150
Locus_11639_Transcript_1/1_Conf_1.000	1464	20	0	NP_497853.1	97	892.493	488	477	hypothetical protein T08A11.2
Locus_1164_Transcript_1/2_Conf_1.000	401	20	3.41E-34	XP_002638772.1	70	147.902	133	94	C. briggsae CBR-PTR-21 protein

Locus_1164_Transcript_2/2_Conf_1.000	291	20	1.35E-22	NP_490751.1	75	109.383	91	69	PaTched Related family member (ptr-21)
Locus_11640_Transcript_1/1_Conf_1.000	629	0							
Locus_11641_Transcript_1/1_Conf_1.000	667	20	3.31E-58	EFO27604.1	74	228.794	221	164	hypothetical protein LOAG_00873
Locus_11642_Transcript_1/1_Conf_1.000	378	4	1.75E-14	XP_002633359.1	94	82.4185	50	47	Hypothetical protein CBG06103
Locus_11643_Transcript_1/1_Conf_1.000	134	4	3.74E-09	XP_002643366.1	79	64.6994	44	35	Hypothetical protein CBG15976
Locus_11644_Transcript_1/1_Conf_1.000	268	2	2.29E-06	XP_002636286.1	63	55.4546	73	46	Hypothetical protein CBG08576
Locus_11645_Transcript_1/1_Conf_1.000	823	0							
Locus_11646_Transcript_1/1_Conf_1.000	667	6	2.62E-55	XP_002637345.1	64	219.164	232	149	C. briggsae CBR-UNC-68 protein
Locus_11647_Transcript_1/2_Conf_1.000	1354	20	1.08E-145	NP_492121.1	72	521.161	456	330	Piwi (fruitfly) Related Gene family member (prg-1)
Locus_11647_Transcript_2/2_Conf_1.000	1354	20	1.08E-145	NP_492121.1	72	521.161	456	330	Piwi (fruitfly) Related Gene family member (prg-1)
Locus_11648_Transcript_1/1_Conf_1.000	479	0							
Locus_11649_Transcript_1/1_Conf_1.000	734	20	2.72E-62	EFO22517.1	73	242.662	204	149	DREV methyltransferase
Locus_1165_Transcript_1/1_Conf_1.000	412	20	6.92E-64	XP_002634177.1	93	246.514	132	124	C. briggsae CBR-RPL-20 protein
Locus_11650_Transcript_1/1_Conf_1.000	404	13	5.10E-06	XP_001900016.1	47	54.299	134	64	Leucine Rich Repeat family protein
Locus_11651_Transcript_1/1_Conf_1.000	452	15	7.58E-10	XP_001897754.1	49	67.0106	158	78	Poly

Locus_11652_Transcript_1/2_Conf_1.000	688	20	1.67E-23	XP_002646274.1	72	113.62	106	77	Hypothetical protein CBG11979
Locus_11652_Transcript_2/2_Conf_1.000	646	20	6.87E-74	XP_002646274.1	80	280.796	210	169	Hypothetical protein CBG11979
Locus_11653_Transcript_1/1_Conf_1.000	349	0							
Locus_11654_Transcript_1/1_Conf_1.000	1089	20	4.85E-95	XP_002645831.1	63	352.443	404	257	C. briggsae CBR-RBC-1 protein
Locus_11655_Transcript_1/1_Conf_1.000	294	20	5.43E-32	NP_001123000.1	84	140.584	96	81	hypothetical protein T05C3.6
Locus_11656_Transcript_1/1_Conf_1.000	335	20	8.56E-30	NP_507684.4	78	133.265	107	84	abnormal EMBroygenesis family member (emb-4)
Locus_11657_Transcript_1/1_Conf_1.000	291	4	2.23E-09	XP_002637600.1	72	65.4698	68	49	C. briggsae CBR-SULP-8 protein
Locus_11658_Transcript_1/1_Conf_1.000	1545	1	8.37E-04	XP_001604445.1	37	50.0618	354	132	PREDICTED: hypothetical protein
Locus_11659_Transcript_1/2_Conf_1.000	625	3	5.82E-13	CAR63732.1	62	67.3958	82	51	hypothetical protein
Locus_11659_Transcript_2/2_Conf_1.000	575	3	1.84E-15	CAR63732.1	51	86.2705	152	78	hypothetical protein
Locus_1166_Transcript_1/4_Conf_0.222	262	0							
Locus_1166_Transcript_2/4_Conf_0.222	900	1	9.95E-05	NP_505827.1	50	51.9878	102	52	hypothetical protein H19N07.3
Locus_1166_Transcript_3/4_Conf_0.222	892	20	1.48E-61	NP_491562.2	60	240.736	294	179	LEThal family member (let-526)
Locus_1166_Transcript_4/4_Conf_0.444	2031	0							
Locus_11660_Transcript_1/1_Conf_1.000	146	6	1.51E-13	NP_871966.2	89	79.337	48	43	hypothetical protein Y48B6A.6
Locus_11661_Transcript_1/1_Conf_1.000	256	0							
Locus_11662_Transcript_1/1_Conf_1.000	131	0							
Locus_11663_Transcript_1/1_Conf_1.000	770	20	1.68E-73	NP_501418.1	97	280.026	142	138	SQuashed Vulva family member (sqv-1)
Locus_11664_Transcript_1/1_Conf_1.000	427	20	7.56E-26	XP_002642911.1	83	120.168	77	64	Hypothetical protein CBG15186
Locus_11665_Transcript_1/1_Conf_1.000	1134	3	1.53E-30	CAA91299.3	46	138.272	400	187	C. elegans protein T01B7.6, confirmed by transcript evidence
Locus_11666_Transcript_1/1_Conf_1.000	174	20	1.78E-27	NP_001163801.1	100	125.561	57	57	hypothetical protein LOC401152 isoform 1
Locus_11667_Transcript_1/2_Conf_1.000	803	2	2.88E-18	NP_505117.2	68	96.6709	116	79	hypothetical protein K07C11.8
Locus_11667_Transcript_2/2_Conf_1.000	526	2	1.06E-18	NP_505117.2	68	96.6709	116	79	hypothetical protein K07C11.8

Locus_11668_Transcript_1/1_Conf_1.000	786	20	3.25E-43	XP_002640253.1	53	179.489	269	145	C. briggsae CBR-CYE-1 protein
Locus_11669_Transcript_1/1_Conf_1.000	434	5	9.75E-42	NP_495343.1	76	172.94	144	110	hypothetical protein C56C10.12
Locus_1167_Transcript_1/2_Conf_1.000	577	0							
Locus_1167_Transcript_2/2_Conf_1.000	577	0							
Locus_11670_Transcript_1/2_Conf_1.000	308	0							
Locus_11670_Transcript_2/2_Conf_1.000	307	0							
Locus_11671_Transcript_1/1_Conf_1.000	448	0							
Locus_11672_Transcript_1/1_Conf_1.000	611	20	7.54E-85	NP_491342.1	92	317.005	178	165	hypothetical protein C43E11.9
Locus_11673_Transcript_1/1_Conf_1.000	307								
Locus_11674_Transcript_1/1_Conf_1.000	328	20	4.75E-12	NP_495768.1	52	74.3294	117	61	hypothetical protein F49E12.1

Locus_11675_Transcript_1/1_Conf_1.000	238	20	2.97E-22	XP_002723288.1	82	108.227	63	52	PREDICTED: COX6B protein (predicted)-like
Locus_11676_Transcript_1/1_Conf_1.000	509	0							
Locus_11677_Transcript_1/1_Conf_1.000	260	20	2.39E-11	XP_002643075.1	58	72.0182	87	51	Hypothetical protein CBG22995
Locus_11678_Transcript_1/1_Conf_1.000	140	20	7.74E-15	NP_496814.1	93	83.5741	46	43	Member of AAA family binding CED-4 family member (mac-1)
Locus_11679_Transcript_1/1_Conf_1.000	370	1	1.11E-08	EFO23989.1	59	63.1586	91	54	hypothetical protein LOAG_04493
Locus_1168_Transcript_1/1_Conf_1.000	1984	20	9.92E-165	XP_002643237.1	62	585.104	650	405	C. briggsae CBR-IGCM-1 protein
Locus_11680_Transcript_1/1_Conf_1.000	1479	20	0	CBJ25106.1	86	700.664	494	425	C. elegans protein Y105C5B.21d, partially confirmed by transcript evidence
Locus_11681_Transcript_1/1_Conf_1.000	1026	20	5.25E-88	XP_002635050.1	65	328.946	360	234	Hypothetical protein CBG11250
Locus_11682_Transcript_1/1_Conf_1.000	818	20	3.81E-58	XP_002644069.1	69	229.18	198	137	C. briggsae CBR-PXN-2 protein
Locus_11683_Transcript_1/1_Conf_1.000	371	20	7.14E-40	NP_498263.2	80	166.777	110	88	ALdehyde deHydrogenase family member (alh-9)
Locus_11684_Transcript_1/1_Conf_1.000	133	0							
Locus_11685_Transcript_1/1_Conf_1.000	517	0							
Locus_11686_Transcript_1/1_Conf_1.000	451	2	1.25E-04	NP_501585.1	58	49.6766	58	34	hypothetical protein C01F6.2
Locus_11687_Transcript_1/1_Conf_1.000	921	20	4.58E-61	XP_001891577.1	82	239.195	172	142	Protein set-1
Locus_11688_Transcript_1/1_Conf_1.000	216	0							
Locus_11689_Transcript_1/1_Conf_1.000	126	0							
Locus_1169_Transcript_1/1_Conf_1.000	728	0							
Locus_11690_Transcript_1/1_Conf_1.000	447	2	1.94E-05	EFO16740.1	49	52.373	77	38	hypothetical protein LOAG_11764
Locus_11691_Transcript_1/1_Conf_1.000	1172	20	4.64E-147	XP_002645119.1	84	525.398	393	331	Hypothetical protein CBG16813
Locus_11692_Transcript_1/1_Conf_1.000	837	20	3.01E-13	NP_492529.2	46	80.1073	239	111	SET (trithorax/polycomb) domain containing family member (set-32)
Locus_11693_Transcript_1/1_Conf_1.000	347	6	7.94E-07	XP_002129449.1	46	56.9954	115	54	PREDICTED: hypothetical protein

Locus_11694_Transcript_1/1_Conf_1.000	271	0							
Locus_11695_Transcript_1/1_Conf_1.000	1172	20	1.03E-146	NP_507860.3	85	524.242	362	308	DeHydrogenases, Short chain family member (dhs-24)
Locus_11696_Transcript_1/1_Conf_1.000	390	20	4.06E-48	XP_002639529.1	85	194.126	130	111	Hypothetical protein CBG04158
Locus_11697_Transcript_1/1_Conf_1.000	226	3	2.91E-25	NP_500037.2	85	118.242	74	63	hypothetical protein Y55F3AR.1
Locus_11698_Transcript_1/1_Conf_1.000	446	20	8.17E-12	XP_002631215.1	56	73.559	96	54	C. briggsae CBR-ECT-2 protein
Locus_11699_Transcript_1/1_Conf_1.000	696	20	5.80E-96	EFO22056.1	86	354.369	232	200	hypothetical protein LOAG_06429
Locus_117_Transcript_1/1_Conf_1.000	191	0							
Locus_1170_Transcript_1/1_Conf_1.000	1015	0							

Locus_11700_Transcript_1/1_Conf_1.000	1894	20	1.95E-77	EFO28277.1	52	295.049	578	302	piwi domain-containing protein
Locus_11701_Transcript_1/1_Conf_1.000	150	5	3.46E-10	A8XKF2.2	83	68.1662	43	36	Choline transporter-like protein 1
Locus_11702_Transcript_1/1_Conf_1.000	231	4	4.47E-10	NP_492367.1	77	67.781	57	44	Glycogen Synthase Kinase Alpha subunit family member (gska-3)
Locus_11703_Transcript_1/1_Conf_1.000	551	20	3.87E-41	NP_001040716.1	69	171.4	183	127	hypothetical protein ZC328.3
Locus_11704_Transcript_1/1_Conf_1.000	210	0							
Locus_11705_Transcript_1/1_Conf_1.000	700	20	3.14E-65	NP_505697.2	73	252.292	234	172	hypothetical protein C35A5.7
Locus_11706_Transcript_1/1_Conf_1.000	757	8	5.54E-106	AAT02162.1	89	387.882	249	222	hypothetical protein L3ni51
Locus_11707_Transcript_1/1_Conf_1.000	682	20	7.46E-85	NP_496454.1	82	317.39	223	185	hypothetical protein B0334.3
Locus_11708_Transcript_1/1_Conf_1.000	344	1	2.10E-07	NP_001122817.1	67	58.9214	56	38	hypothetical protein Y54G2A.52
Locus_11709_Transcript_1/1_Conf_1.000	692	20	2.13E-34	XP_001898340.1	67	149.828	169	114	LD47622p
Locus_1171_Transcript_1/1_Conf_1.000	1900	20	7.84E-119	XP_002640096.1	71	432.565	440	313	C. briggsae CBR-PNK-1 protein
Locus_11710_Transcript_1/2_Conf_1.000	1819	20	2.14E-25	NP_504250.2	43	122.094	497	216	Suppressor Of Clr family member (soc-1)

Locus_11710_Transcript_2/2_Conf_1.000	1801	20	1.62E-25	NP_504250.2	44	122.479	496	219	Suppressor Of Clr family member (soc-1)
Locus_11711_Transcript_1/1_Conf_1.000	243	0							
Locus_11712_Transcript_1/1_Conf_1.000	164	5	1.88E-08	NP_497046.1	66	62.3882	53	35	UNCoordinated family member (unc-52)
Locus_11713_Transcript_1/1_Conf_1.000	375	7	1.95E-05	XP_001895231.1	88	52.373	34	30	Bucentaur or craniofacial development family protein
Locus_11714_Transcript_1/1_Conf_1.000	386	20	1.37E-11	XP_001902350.1	60	72.7886	119	72	Myosin XVIIIA
Locus_11715_Transcript_1/1_Conf_1.000	322	0							
Locus_11716_Transcript_1/1_Conf_1.000	320	0							
Locus_11717_Transcript_1/1_Conf_1.000	168	20	9.83E-26	BAH14701.1	100	119.783	55	55	unnamed protein product
Locus_11718_Transcript_1/1_Conf_1.000	982	20	1.68E-64	NP_492260.1	64	250.751	304	197	hypothetical protein F52B5.3
Locus_11719_Transcript_1/1_Conf_1.000	364	3	8.25E-25	NP_508493.1	74	116.701	122	91	protein KINase family member (kin-29)

Locus_1172_Transcript_1/1_Conf_1.000	503	20	1.34E-38	XP_002637575.1	75	162.54	148	111	C. briggsae CBR-EHBP-1 protein
Locus_11720_Transcript_1/1_Conf_1.000	150	0							
Locus_11721_Transcript_1/1_Conf_1.000	476	20	3.50E-15	XP_002199801.1	62	84.7297	86	54	PREDICTED: ELMO/CED-12 domain containing 1
Locus_11722_Transcript_1/1_Conf_1.000	805	20	1.43E-41	XP_002408201.1	62	174.096	265	165	Ran-binding protein, putative
Locus_11723_Transcript_1/1_Conf_1.000	569	0							
Locus_11724_Transcript_1/1_Conf_1.000	395	0							
Locus_11725_Transcript_1/2_Conf_1.000	635	20	4.02E-23	XP_001900825.1	78	112.079	89	70	CG3579-PA
Locus_11725_Transcript_2/2_Conf_1.000	635	20	4.02E-23	XP_001900825.1	78	112.079	89	70	CG3579-PA
Locus_11726_Transcript_1/2_Conf_1.000	1272	0							
Locus_11726_Transcript_2/2_Conf_1.000	1272	0							
Locus_11727_Transcript_1/1_Conf_1.000	303	20	5.03E-30	EFO21314.1	81	134.035	100	81	male sterility protein
Locus_11728_Transcript_1/1_Conf_1.000	222	6	2.64E-18	XP_001896269.1	85	95.1301	57	49	BTB/POZ domain containing protein
Locus_11729_Transcript_1/1_Conf_1.000	727	20	7.24E-76	XP_002647628.1	74	287.73	227	169	Hypothetical protein CBG06721
Locus_1173_Transcript_1/1_Conf_1.000	2987	20	0	XP_001900046.1	66	670.618	769	515	Kinesin motor domain containing protein
Locus_11730_Transcript_1/1_Conf_1.000	663	0							
Locus_11731_Transcript_1/1_Conf_1.000	353	4	1.75E-14	NP_496498.1	63	82.4185	99	63	hypothetical protein C47D12.8
Locus_11732_Transcript_1/1_Conf_1.000	1115	20	2.19E-74	CAX32485.1	67	283.878	359	243	C. elegans protein T20D3.11b, partially confirmed by transcript evidence
Locus_11733_Transcript_1/1_Conf_1.000	877	0							
Locus_11734_Transcript_1/1_Conf_1.000	469	2	8.13E-12	XP_002638516.1	46	73.559	139	65	Hypothetical protein CBG05542
Locus_11735_Transcript_1/1_Conf_1.000	332	20	8.89E-27	NP_496968.1	96	123.25	63	61	hypothetical protein Y48B6A.12

Locus_11736_Transcript_1/1_Conf_1.000	393	1	9.74E-05	NP_499759.1	72	50.0618	44	32	DNaJ domain (prokaryotic heat shock protein) family member (dnj-17)
Locus_11737_Transcript_1/1_Conf_1.000	608	20	3.48E-50	NP_497589.3	68	201.83	196	134	hypothetical protein Y71H2B.5
Locus_11738_Transcript_1/1_Conf_1.000	675	20	5.67E-37	EFO18191.1	71	158.303	168	120	hypothetical protein LOAG_10303
Locus_11739_Transcript_1/1_Conf_1.000	273	20	4.56E-23	EFO18946.1	74	110.923	90	67	hypothetical protein LOAG_09552
Locus_1174_Transcript_1/1_Conf_1.000	663	20	2.85E-70	XP_002640874.1	83	268.855	173	144	C. briggsae CBR-CCO-2 protein
Locus_11740_Transcript_1/1_Conf_1.000	202	0							
Locus_11741_Transcript_1/1_Conf_1.000	323	20	2.98E-38	NP_495409.3	86	161.384	108	93	hypothetical protein C44B7.10
Locus_11742_Transcript_1/1_Conf_1.000	553	20	1.81E-78	NP_493616.1	87	295.434	184	161	hypothetical protein F33H2.5
Locus_11743_Transcript_1/1_Conf_1.000	799	20	2.01E-72	EFO27128.1	77	276.559	244	188	cyclin domain-containing protein
Locus_11744_Transcript_1/1_Conf_1.000	154	20	3.01E-14	XP_002919374.1	100	81.6481	38	38	PREDICTED: pleckstrin homology domain-containing family O member 1-like
Locus_11745_Transcript_1/1_Conf_1.000	514	20	8.44E-39	XP_001894255.1	82	163.31	128	105	trafficking protein particle complex subunit 1

Locus_11746_Transcript_1/1_Conf_1.000	283	20	1.66E-20	XP_002192510.1	100	102.449	80	80	PREDICTED: heterogeneous nuclear ribonucleoprotein K
Locus_11747_Transcript_1/1_Conf_1.000	390	20	4.65E-36	NP_500022.1	71	154.066	127	91	Inner Mitochondrial Membrane Protease family member (immp-2)
Locus_11748_Transcript_1/1_Conf_1.000	251	20	2.63E-39	ADN23570.1	100	164.851	83	83	ubiquitin
Locus_11749_Transcript_1/1_Conf_1.000	509	20	5.75E-32	EFO22933.1	66	140.584	171	113	hypothetical protein LOAG_05552
Locus_1175_Transcript_1/1_Conf_1.000	807	20	2.18E-114	ACI49174.1	91	416.001	245	225	hypothetical protein Csp3_JD02.002
Locus_11750_Transcript_1/1_Conf_1.000	468	20	5.21E-51	NP_498688.1	72	203.756	153	111	Prolyl Carboxy Peptidase like family member (pcp-1)

Locus_11751_Transcript_1/1_Conf_1.000	205	20	7.53E-26	NP_495261.1	89	120.168	67	60	hypothetical protein F13H8.7
Locus_11752_Transcript_1/1_Conf_1.000	614	20	5.21E-25	NP_502214.2	53	118.242	186	100	hypothetical protein ZK792.7
Locus_11753_Transcript_1/1_Conf_1.000	800	20	2.58E-27	EFO17580.1	69	126.716	137	95	hypothetical protein LOAG_10920
Locus_11754_Transcript_1/1_Conf_1.000	589	3	2.01E-28	NP_493625.4	56	129.413	185	105	hypothetical protein F31C3.3
Locus_11755_Transcript_1/2_Conf_1.000	312	0							
Locus_11755_Transcript_2/2_Conf_1.000	279	0							
Locus_11756_Transcript_1/1_Conf_1.000	678	20	1.35E-78	NP_502573.1	78	296.59	223	176	hypothetical protein Y62E10A.6
Locus_11757_Transcript_1/1_Conf_1.000	303	0							
Locus_11758_Transcript_1/1_Conf_1.000	608	20	9.13E-67	XP_002641059.1	79	256.914	193	153	Hypothetical protein CBG22470
Locus_11759_Transcript_1/1_Conf_1.000	313	20	3.92E-54	XP_002760567.1	100	214.157	104	104	PREDICTED: 60S ribosomal protein L5-like isoform 1
Locus_1176_Transcript_1/2_Conf_1.000	708	20	1.49E-38	XP_001896392.1	63	163.696	215	136	Dual specificity phosphatase, catalytic domain containing protein
Locus_1176_Transcript_2/2_Conf_1.000	708	20	1.14E-38	XP_001896392.1	63	164.081	213	136	Dual specificity phosphatase, catalytic domain containing protein
Locus_11760_Transcript_1/1_Conf_1.000	1085	20	7.27E-160	CBI70492.1	93	567.77	361	337	vacuolar ATPase a subunit
Locus_11761_Transcript_1/2_Conf_1.000	263	0							
Locus_11761_Transcript_2/2_Conf_1.000	530	0							
Locus_11762_Transcript_1/1_Conf_1.000	273	6	1.37E-19	XP_002632822.1	70	99.3673	91	64	C. briggsae CBR-NHR-97 protein
Locus_11763_Transcript_1/1_Conf_1.000	217	0							

Locus_11764_Transcript_1/1_Conf_1.000	887	20	4.96E-110	XP_002636649.1	79	401.749	296	235	Hypothetical protein CBG23360
Locus_11765_Transcript_1/1_Conf_1.000	400	2	4.95E-09	XP_002639986.1	78	64.3142	42	33	Hypothetical protein CBG10814
Locus_11766_Transcript_1/1_Conf_1.000	307	0							
Locus_11767_Transcript_1/1_Conf_1.000	244	7	4.71E-12	NP_001122977.1	76	74.3294	80	61	Variable ABnormal morphology family member (vab-8)
Locus_11768_Transcript_1/1_Conf_1.000	1119	20	3.70E-138	CBL87054.2	86	495.738	311	270	C. elegans protein F54E4.1, partially confirmed by transcript evidence
Locus_11769_Transcript_1/1_Conf_1.000	545	14	2.77E-36	NP_001123010.1	68	155.221	157	107	related to yeast Vacuolar Protein Sorting factor family member (vps-39)
Locus_1177_Transcript_1/1_Conf_1.000	472	20	5.00E-62	XP_002646355.1	83	240.35	154	128	Hypothetical protein CBG12069
Locus_11770_Transcript_1/1_Conf_1.000	181	0							
Locus_11771_Transcript_1/2_Conf_1.000	272	0							
Locus_11771_Transcript_2/2_Conf_1.000	268	0							
Locus_11772_Transcript_1/1_Conf_1.000	628	1	1.22E-08	NP_001021391.1	56	63.929	144	81	hypothetical protein F16C3.3
Locus_11773_Transcript_1/1_Conf_1.000	434	3	1.92E-13	XP_002636820.1	58	78.9518	148	86	Hypothetical protein CBG09264
Locus_11774_Transcript_1/1_Conf_1.000	801	20	3.81E-87	NP_498566.3	84	325.479	234	198	hypothetical protein K04G7.11
Locus_11775_Transcript_1/1_Conf_1.000	188	0							
Locus_11776_Transcript_1/1_Conf_1.000	268	20	1.47E-37	XP_002641474.1	88	159.073	87	77	C. briggsae CBR-PTP-1 protein

Locus_11777_Transcript_1/1_Conf_1.000	410	20	2.91E-70	NP_501549.1	100	267.7	134	134	RAP homolog (vertebrate Rap GTPase family) family member (rap-1)
Locus_11778_Transcript_1/1_Conf_1.000	990	20	5.38E-26	NP_501282.1	49	122.865	289	143	hypothetical protein C33H5.8
Locus_11779_Transcript_1/1_Conf_1.000	314	0							
Locus_1178_Transcript_1/2_Conf_1.000	643	20	2.04E-78	NP_497574.2	89	295.819	174	156	hypothetical protein Y54F10AM.5
Locus_1178_Transcript_2/2_Conf_1.000	643	20	2.04E-78	NP_497574.2	89	295.819	174	156	hypothetical protein Y54F10AM.5
Locus_11780_Transcript_1/2_Conf_1.000	933	0							
Locus_11780_Transcript_2/2_Conf_1.000	581	0							
Locus_11781_Transcript_1/2_Conf_1.000	652	20	1.02E-56	XP_002638616.1	84	223.787	171	144	Hypothetical protein CBG05668
Locus_11781_Transcript_2/2_Conf_1.000	885	20	8.03E-60	XP_002638616.1	78	234.958	213	167	Hypothetical protein CBG05668
Locus_11782_Transcript_1/1_Conf_1.000	696	7	7.46E-35	XP_002641835.1	60	151.369	230	138	C. briggsae CBR-CYK-1 protein
Locus_11783_Transcript_1/1_Conf_1.000	518	20	6.39E-42	NP_494918.2	75	173.711	171	129	hypothetical protein T27F7.3
Locus_11784_Transcript_1/1_Conf_1.000	197	0							
Locus_11785_Transcript_1/1_Conf_1.000	217	0							
Locus_11786_Transcript_1/1_Conf_1.000	183	0							
Locus_11787_Transcript_1/1_Conf_1.000	245	0							
Locus_11788_Transcript_1/1_Conf_1.000	319	0							
Locus_11789_Transcript_1/1_Conf_1.000	309	20	7.71E-23	XP_002639369.1	66	110.153	96	64	C. briggsae CBR-ZIG-7 protein
Locus_1179_Transcript_1/1_Conf_1.000	2249	5	5.22E-24	XP_002636796.1	45	117.857	389	178	C. briggsae CBR-RIL-2 protein
Locus_11790_Transcript_1/1_Conf_1.000	279	0							

Locus_11791_Transcript_1/1_Conf_1.000	1017	20	1.02E-59	EFO16811.1	63	234.958	264	167	hypothetical protein LOAG_11692
Locus_11792_Transcript_1/1_Conf_1.000	188	0							
Locus_11793_Transcript_1/1_Conf_1.000	359	5	2.42E-24	XP_002636405.1	96	115.161	58	56	Hypothetical protein CBG23057
Locus_11794_Transcript_1/1_Conf_1.000	1503	20	0	NP_001024792.2	87	655.596	422	371	yeast SMF (divalent cation transporter) homolog family member (smf-1)
Locus_11795_Transcript_1/1_Conf_1.000	1026	20	3.84E-14	XP_002129449.1	47	83.5741	184	87	PREDICTED: hypothetical protein
Locus_11796_Transcript_1/1_Conf_1.000	432	2	5.59E-05	NP_740989.1	54	50.8322	141	77	hypothetical protein F07F6.8
Locus_11797_Transcript_1/1_Conf_1.000	164	0							
Locus_11798_Transcript_1/1_Conf_1.000	435	20	1.03E-67	XP_002644717.1	93	259.225	145	136	Hypothetical protein CBG14711
Locus_11799_Transcript_1/1_Conf_1.000	252	3	8.53E-17	XP_001898467.1	78	90.1225	80	63	FYVE zinc finger family protein
Locus_118_Transcript_1/1_Conf_1.000	471	0							
Locus_1180_Transcript_1/1_Conf_1.000	1449	20	1.23E-118	Q5QQ52.1	68	431.409	402	277	Xylosyltransferase sqv-6
Locus_11800_Transcript_1/1_Conf_1.000	266	0							
Locus_11801_Transcript_1/1_Conf_1.000	142	0							
Locus_11802_Transcript_1/1_Conf_1.000	487	0							
Locus_11803_Transcript_1/1_Conf_1.000	202	0							
Locus_11804_Transcript_1/1_Conf_1.000	316	5	5.17E-06	NP_001021675.1	71	54.299	63	45	hypothetical protein W10C8.6
Locus_11805_Transcript_1/1_Conf_1.000	152	0							
Locus_11806_Transcript_1/1_Conf_1.000	227	20	4.48E-26	NP_503148.1	90	120.939	66	60	hypothetical protein Y108G3AL.2
Locus_11807_Transcript_1/1_Conf_1.000	183	20	6.11E-23	XP_002640829.1	96	110.538	58	56	C. briggsae CBR-ADS-1 protein

Locus_11808_Transcript_1/2_Conf_1.000	449	2	8.42E-09	XP_002642114.1	48	63.5438	141	69	C. briggsae CBR-PQN-45 protein
Locus_11808_Transcript_2/2_Conf_1.000	449	2	1.75E-06	XP_002642114.1	48	55.8398	141	68	C. briggsae CBR-PQN-45 protein
Locus_11809_Transcript_1/1_Conf_1.000	372	0							
Locus_1181_Transcript_1/1_Conf_1.000	1290	20	5.04E-113	XP_002638266.1	87	412.535	275	240	C. briggsae CBR-HAF-3 protein
Locus_11810_Transcript_1/1_Conf_1.000	748	20	1.37E-40	NP_001022051.1	59	170.629	250	148	SREBP Cleavage activating Protein (SCAP) homolog family member (scp-1)
Locus_11811_Transcript_1/1_Conf_1.000	143	0							
Locus_11812_Transcript_1/2_Conf_1.000	367	0							
Locus_11812_Transcript_2/2_Conf_1.000	367	0							
Locus_11813_Transcript_1/1_Conf_1.000	604	20	9.93E-74	CAA86821.1	90	280.026	178	161	excretory/secretory antigen
Locus_11814_Transcript_1/1_Conf_1.000	1129	20	2.94E-50	XP_002634649.1	71	203.756	225	161	C. briggsae CBR-DGK-4 protein
Locus_11815_Transcript_1/1_Conf_1.000	319	7	1.71E-09	XP_001898068.1	69	65.855	71	49	Homeobox domain containing protein
Locus_11816_Transcript_1/1_Conf_1.000	291	4	2.63E-10	NP_491254.1	58	68.5514	80	47	hypothetical protein C50F2.4
Locus_11817_Transcript_1/1_Conf_1.000	143	0							
Locus_11818_Transcript_1/1_Conf_1.000	636	12	1.45E-20	NP_499391.1	76	103.605	86	66	hypothetical protein Y39A1C.1
Locus_11819_Transcript_1/1_Conf_1.000	232	12	6.03E-15	XP_002631617.1	72	83.9593	75	54	Hypothetical protein CBG20802
Locus_1182_Transcript_1/1_Conf_1.000	231	8	6.88E-19	XP_002631304.1	92	97.0561	53	49	Hypothetical protein CBG03118
Locus_11820_Transcript_1/1_Conf_1.000	283	0							
Locus_11821_Transcript_1/1_Conf_1.000	713	20	2.21E-98	EFO22809.1	88	362.459	237	210	hypothetical protein LOAG_05677
Locus_11822_Transcript_1/1_Conf_1.000	750	20	7.74E-92	XP_002630011.1	80	340.887	250	201	Hypothetical protein CBG13376

Locus_11823_Transcript_1/1_Conf_1.000	414	20	2.38E-40	NP_497425.1	72	168.318	136	99	SphingoMyelin Synthase family member (sms-3)
Locus_11824_Transcript_1/1_Conf_1.000	566	20	1.02E-23	XP_001902442.1	60	113.62	169	103	hypothetical protein Bm1_54925
Locus_11825_Transcript_1/1_Conf_1.000	823	20	4.09E-92	NP_505433.3	80	342.043	243	196	Heavy chain, Unconventional Myosin family member (hum-2)
Locus_11826_Transcript_1/1_Conf_1.000	300	0							
Locus_11827_Transcript_1/1_Conf_1.000	527	4	1.74E-37	CAB07693.3	74	159.073	131	97	C. elegans protein Y48E1B.7, partially confirmed by transcript evidence
Locus_11828_Transcript_1/2_Conf_1.000	831	20	2.39E-71	NP_493713.2	81	273.092	204	167	CalPoNin family member (cpn-2)
Locus_11828_Transcript_2/2_Conf_1.000	734	20	1.88E-71	NP_493713.2	81	273.092	204	167	CalPoNin family member (cpn-2)
Locus_11829_Transcript_1/1_Conf_1.000	607	20	2.77E-31	XP_002643253.1	75	139.043	128	96	C. briggsae CBR-AEX-3 protein
Locus_1183_Transcript_1/1_Conf_1.000	965	20	1.70E-37	XP_002189824.1	55	160.999	280	156	PREDICTED: centrosomal protein 97kDa
Locus_11830_Transcript_1/1_Conf_1.000	935	20	3.77E-119	NP_510560.2	81	432.18	304	249	Gut OBstructed or defective family member (gob-1)
Locus_11831_Transcript_1/1_Conf_1.000	183	20	6.53E-25	XP_002637927.1	96	117.087	61	59	Hypothetical protein CBG04739

Locus_11832_Transcript_1/1_Conf_1.000	339	0							
Locus_11833_Transcript_1/1_Conf_1.000	756	20	2.43E-77	EFO21654.1	76	292.738	249	191	hypothetical protein LOAG_06832
Locus_11834_Transcript_1/1_Conf_1.000	209	0							
Locus_11835_Transcript_1/1_Conf_1.000	281	0							
Locus_11836_Transcript_1/1_Conf_1.000	160	0							
Locus_11837_Transcript_1/1_Conf_1.000	1289	20	3.26E-104	XP_001898591.1	70	383.259	396	279	Polyprenyl synthetase family protein
Locus_11838_Transcript_1/1_Conf_1.000	765	20	4.93E-25	NP_496945.3	62	119.013	166	103	XRN (mouse/S. cerevisiae) ribonuclease related family member (xrn-1)
Locus_11839_Transcript_1/1_Conf_1.000	600	20	1.04E-99	AAV38432.1	100	366.311	173	173	tissue inhibitor of metalloproteinase 1 (erythroid potentiating activity, collagenase inhibitor)
Locus_1184_Transcript_1/1_Conf_1.000	621	20	6.01E-69	NP_001023176.1	91	264.233	157	143	hypothetical protein F33D4.8
Locus_11840_Transcript_1/1_Conf_1.000	493	17	4.43E-18	XP_002632297.1	56	94.3597	151	85	C. briggsae CBR-NOL-6 protein
Locus_11841_Transcript_1/1_Conf_1.000	441	0							
Locus_11842_Transcript_1/1_Conf_1.000	681	20	1.91E-72	XP_001896094.1	74	276.174	225	168	Protein kinase domain containing protein
Locus_11843_Transcript_1/1_Conf_1.000	133	6	9.22E-08	XP_002642608.1	82	60.077	41	34	C. briggsae CBR-LIG-4 protein
Locus_11844_Transcript_1/1_Conf_1.000	1224	0							
Locus_11845_Transcript_1/1_Conf_1.000	1117	20	6.22E-61	CAB01684.2	74	239.195	201	150	C. elegans protein C35C5.3a, confirmed by transcript evidence
Locus_11846_Transcript_1/1_Conf_1.000	234	0							

Locus_11847_Transcript_1/1_Conf_1.000	625	20	4.48E-96	EFO19031.1	91	354.369	208	190	hypothetical protein LOAG_09464
Locus_11848_Transcript_1/1_Conf_1.000	401	20	3.08E-35	XP_002639450.1	96	151.369	80	77	Hypothetical protein CBG04045
Locus_11849_Transcript_1/1_Conf_1.000	181	0							
Locus_1185_Transcript_1/1_Conf_1.000	2272	20	6.64E-43	XP_002644054.1	83	180.644	132	110	Hypothetical protein CBG17518
Locus_11850_Transcript_1/1_Conf_1.000	131	0							
Locus_11851_Transcript_1/1_Conf_1.000	495	0							
Locus_11852_Transcript_1/1_Conf_1.000	348	20	1.52E-50	NP_506259.2	92	202.216	115	106	hypothetical protein W07G4.3
Locus_11853_Transcript_1/1_Conf_1.000	889	4	1.98E-82	XP_002643042.1	77	310.071	289	223	Hypothetical protein CBG22957
Locus_11854_Transcript_1/1_Conf_1.000	410	3	7.49E-34	A8WRV1.2	75	146.747	128	96	Serine/threonine-protein kinase kin-29
Locus_11855_Transcript_1/1_Conf_1.000	368	0							
Locus_11856_Transcript_1/1_Conf_1.000	203	0							
Locus_11857_Transcript_1/1_Conf_1.000	222	20	2.02E-18	NP_001021770.1	92	95.5153	54	50	hypothetical protein Y47G6A.7
Locus_11858_Transcript_1/1_Conf_1.000	557	20	2.99E-20	NP_499961.2	73	102.064	82	60	Nuclear Hormone Receptor family member (nhr-76)
Locus_11859_Transcript_1/1_Conf_1.000	675	20	2.28E-70	NP_506561.1	87	269.24	170	149	CYclophyliN family member (cyn-1)

Locus_1186_Transcript_1/1_Conf_1.000	677	20	1.94E-61	NP_490749.1	92	239.58	166	153	hypothetical protein Y65B4BR.5
Locus_11860_Transcript_1/1_Conf_1.000	897	20	2.11E-31	XP_001892919.1	53	140.584	240	129	TPR Domain containing protein
Locus_11861_Transcript_1/1_Conf_1.000	524	20	3.22E-28	ACI49081.1	67	128.257	137	93	hypothetical protein Cbre_JD11.005
Locus_11862_Transcript_1/1_Conf_1.000	201	0							
Locus_11863_Transcript_1/1_Conf_1.000	297	0							
Locus_11864_Transcript_1/1_Conf_1.000	621	12	2.72E-45	NP_505034.1	79	185.652	204	163	hypothetical protein F40A3.7
Locus_11865_Transcript_1/1_Conf_1.000	2213	20	5.02E-88	XP_001893141.1	62	330.487	466	289	DEAD/DEAH box helicase family protein
Locus_11866_Transcript_1/1_Conf_1.000	650	2	2.68E-25	NP_492698.2	54	119.398	183	100	hypothetical protein ZC247.1
Locus_11867_Transcript_1/1_Conf_1.000	389	2	1.91E-13	NP_491039.1	55	78.9518	118	65	hypothetical protein Y71F9B.1
Locus_11868_Transcript_1/1_Conf_1.000	555	20	2.01E-16	NP_496567.2	48	89.3521	184	90	hypothetical protein Y17G7B.11
Locus_11869_Transcript_1/1_Conf_1.000	526	2	3.18E-23	XP_001895430.1	53	111.694	186	100	hypothetical protein Bm1_19875
Locus_1187_Transcript_1/1_Conf_1.000	825	0							
Locus_11870_Transcript_1/1_Conf_1.000	661	20	6.91E-85	NP_510667.2	87	317.39	217	189	TBC (Tre-2/Bub2/Cdc16) domain family member (tbc-4)
Locus_11871_Transcript_1/2_Conf_1.000	860	9	7.20E-42	XP_002642351.1	66	175.252	170	113	Hypothetical protein CBG18348
Locus_11871_Transcript_2/2_Conf_1.000	860	9	1.60E-41	XP_002642351.1	66	174.096	170	113	Hypothetical protein CBG18348
Locus_11872_Transcript_1/1_Conf_1.000	307	20	4.68E-36	NP_490795.2	82	154.066	102	84	Nematode AStacin protease family member (nas-30)
Locus_11873_Transcript_1/1_Conf_1.000	302	20	1.33E-30	XP_002634824.1	82	135.961	86	71	Hypothetical protein CBG13932

Locus_11874_Transcript_1/1_Conf_1.000	265	20	3.63E-36	NP_505689.1	91	154.451	87	80	hypothetical protein Y32F6B.1
Locus_11875_Transcript_1/1_Conf_1.000	764	20	1.17E-18	ABB53347.1	51	97.8265	183	95	secreted protein 5 precursor
Locus_11876_Transcript_1/1_Conf_1.000	221	0							
Locus_11877_Transcript_1/1_Conf_1.000	194	20	5.47E-24	NP_500274.2	92	114.005	63	58	hypothetical protein Y54G2A.4
Locus_11878_Transcript_1/1_Conf_1.000	333	9	8.87E-19	NP_509416.2	77	96.6709	103	80	hypothetical protein F55E10.7
Locus_11879_Transcript_1/1_Conf_1.000	141	0							
Locus_1188_Transcript_1/1_Conf_1.000	817	20	1.77E-47	XP_002637575.1	60	193.741	269	162	C. briggsae CBR-EHBP-1 protein
Locus_11880_Transcript_1/1_Conf_1.000	200	0							
Locus_11881_Transcript_1/1_Conf_1.000	615	20	7.66E-08	NP_001021233.1	76	61.2326	47	36	hypothetical protein D2045.9
Locus_11882_Transcript_1/1_Conf_1.000	1167	20	9.61E-153	NP_504650.1	78	544.273	389	304	hypothetical protein W01A11.1
Locus_11883_Transcript_1/1_Conf_1.000	481	0							
Locus_11884_Transcript_1/1_Conf_1.000	166	0							
Locus_11885_Transcript_1/1_Conf_1.000	1563	20	0	AAV27339.1	80	653.67	521	420	Hc38
Locus_11886_Transcript_1/1_Conf_1.000	434	0							
Locus_11887_Transcript_1/1_Conf_1.000	342	20	1.31E-33	NP_502290.1	98	145.976	70	69	hypothetical protein F11A10.2

Locus_11888_Transcript_1/1_Conf_1.000	429	20	8.58E-38	NP_001040974.1	81	159.844	141	115	hypothetical protein K09B11.9
Locus_11889_Transcript_1/1_Conf_1.000	491	20	1.63E-36	CAA21622.4	62	155.606	176	110	C. elegans protein Y43F8C.12, partially confirmed by transcript evidence
Locus_1189_Transcript_1/1_Conf_1.000	1045	20	6.25E-129	XP_002644986.1	86	464.922	316	273	C. briggsae CBR-TAG-320 protein
Locus_11890_Transcript_1/1_Conf_1.000	257	3	1.19E-10	XP_002632648.1	69	69.707	59	41	Hypothetical protein CBG21570
Locus_11891_Transcript_1/1_Conf_1.000	223	0							
Locus_11892_Transcript_1/1_Conf_1.000	646	11	3.33E-36	NP_501400.1	67	155.606	192	130	hypothetical protein F35H10.10
Locus_11893_Transcript_1/1_Conf_1.000	581	20	3.00E-61	XP_002648243.1	85	238.424	163	139	Hypothetical protein CBG24373
Locus_11894_Transcript_1/1_Conf_1.000	595	0							
Locus_11895_Transcript_1/1_Conf_1.000	681	3	1.54E-05	XP_002648983.1	83	53.9138	37	31	Hypothetical protein CBG21314
Locus_11896_Transcript_1/1_Conf_1.000	783	20	2.46E-51	NP_001024128.1	77	206.453	164	127	EEA1 (Early Endosome Antigen, Rab effector) homolog family member (eea-1)

Locus_11897_Transcript_1/1_Conf_1.000	550	20	5.55E-64	NP_491714.1	76	247.284	181	138	Aurora/Ipl1 Related kinase family member (air-2)
Locus_11898_Transcript_1/1_Conf_1.000	739	2	2.70E-41	NP_499274.3	61	172.94	212	131	Connector/eNhancer of KSR family member (cnk-1)
Locus_11899_Transcript_1/1_Conf_1.000	1098	20	3.42E-72	XP_001898576.1	64	276.559	303	195	Fructosamine kinase family protein
Locus_119_Transcript_1/10_Conf_0.167	708	20	9.80E-99	CAD22112.1	97	363.614	200	195	tubulin

Locus_119_Transcript_10/10_Conf_0.542	2840	20	0	XP_002646073.1	96	807.364	441	424	Hypothetical protein CBG07939
Locus_119_Transcript_2/10_Conf_0.167	666	20	5.04E-99	CAD22112.1	97	364.385	198	194	tubulin
Locus_119_Transcript_3/10_Conf_0.542	2940	20	0	XP_002646073.1	96	807.364	441	424	Hypothetical protein CBG07939

Locus_119_Transcript_4/10_Conf_0.542	2940	20	0	XP_002646073.1	96	807.364	441	424	Hypothetical protein CBG07939
Locus_119_Transcript_5/10_Conf_0.083	1395	20	4.88E-125	CBL87050.1	79	452.595	334	265	C. elegans protein C50F4.14b, confirmed by transcript evidence
Locus_119_Transcript_6/10_Conf_0.083	1395	20	4.88E-125	CBL87050.1	79	452.595	334	265	C. elegans protein C50F4.14b, confirmed by transcript evidence
Locus_119_Transcript_7/10_Conf_0.542	2840	20	0	XP_002646073.1	96	809.29	441	424	Hypothetical protein CBG07939
Locus_119_Transcript_8/10_Conf_0.292	1992	20	0	XP_002646073.1	95	655.596	360	344	Hypothetical protein CBG07939

Locus_119_Transcript_9/10_Conf_0.292	1992	20	0	XP_002646073.1	95	653.67	360	344	Hypothetical protein CBG07939
Locus_1190_Transcript_1/1_Conf_1.000	362	7	8.28E-25	NP_001022814.1	70	116.701	119	84	ODoRant response abnormal family member (odr-4)
Locus_11900_Transcript_1/3_Conf_0.600	666	20	6.19E-81	NP_491093.1	93	304.294	174	163	RNA Polymerase II (B) subunit family member (rpb-7)
Locus_11900_Transcript_2/3_Conf_0.400	353	20	2.59E-50	NP_491093.1	96	201.445	115	111	RNA Polymerase II (B) subunit family member (rpb-7)

Locus_11900_Transcript_3/3_Conf_0.600	646	20	5.80E-81	NP_491093.1	93	304.294	174	163	RNA Polymerase II (B) subunit family member (rpb-7)
Locus_11901_Transcript_1/1_Conf_1.000	293	2	6.25E-20	EFO16669.1	71	100.523	96	69	BTB/POZ domain-containing protein
Locus_11902_Transcript_1/2_Conf_1.000	277	0							
Locus_11902_Transcript_2/2_Conf_1.000	283	0							
Locus_11903_Transcript_1/1_Conf_1.000	347	0							
Locus_11904_Transcript_1/1_Conf_1.000	379	2	4.46E-10	XP_002630789.1	55	67.781	113	63	Hypothetical protein CBG02486
Locus_11905_Transcript_1/1_Conf_1.000	305	0							
Locus_11906_Transcript_1/1_Conf_1.000	134	0							
Locus_11907_Transcript_1/1_Conf_1.000	1126	20	5.49E-73	NP_491118.2	61	279.256	367	226	hypothetical protein Y54E10A.11
Locus_11908_Transcript_1/1_Conf_1.000	318	20	1.64E-28	NP_500157.2	75	129.028	106	80	Cleavage and Polyadenylation Specificity Factor family member (cpsf-1)
Locus_11909_Transcript_1/1_Conf_1.000	115	0							
Locus_1191_Transcript_1/1_Conf_1.000	455	20	1.20E-23	NP_509947.1	68	112.849	117	80	hypothetical protein T04F8.2
Locus_11910_Transcript_1/1_Conf_1.000	1299	20	8.65E-12	XP_002151267.1	50	76.2554	114	58	histone acetyltransferase, putative
Locus_11911_Transcript_1/1_Conf_1.000	185	0							
Locus_11912_Transcript_1/1_Conf_1.000	165	0							
Locus_11913_Transcript_1/1_Conf_1.000	696	20	1.15E-27	XP_001902469.1	54	127.487	231	125	G protein pathway suppressor 1

Locus_11914_Transcript_1/2_Conf_1.000	1426	20	3.73E-11	XP_002643523.1	45	74.3294	183	83	Hypothetical protein CBG16200
Locus_11914_Transcript_2/2_Conf_1.000	1401	20	8.09E-19	NP_741906.1	40	99.7525	509	206	Maternal Effect Sterile family member (mes-1)

Locus_11915_Transcript_1/1_Conf_1.000	1677	20	1.25E-40	AAA93318.1	56	172.555	278	156	serine/threonine kinase
Locus_11916_Transcript_1/2_Conf_1.000	763	0							
Locus_11916_Transcript_2/2_Conf_1.000	288	0							
Locus_11917_Transcript_1/1_Conf_1.000	299	0							
Locus_11918_Transcript_1/1_Conf_1.000	785	20	3.77E-76	XP_002639317.1	72	288.886	250	181	C. briggsae CBR-ABT-2 protein
Locus_11919_Transcript_1/1_Conf_1.000	305	0							
Locus_1192_Transcript_1/3_Conf_0.714	752	0							
Locus_1192_Transcript_2/3_Conf_0.714	804	0							
Locus_1192_Transcript_3/3_Conf_0.714	752	0							
Locus_11920_Transcript_1/1_Conf_1.000	836	20	1.31E-85	XP_002633781.1	73	320.472	278	205	Hypothetical protein CBG03472
Locus_11921_Transcript_1/2_Conf_1.000	492	20	2.95E-38	ACI49052.1	70	161.384	144	102	hypothetical protein Cbre_JD08.004
Locus_11921_Transcript_2/2_Conf_1.000	492	20	6.57E-38	ACI49052.1	70	160.229	144	102	hypothetical protein Cbre_JD08.004
Locus_11922_Transcript_1/1_Conf_1.000	996	20	1.01E-64	NP_504952.1	76	251.521	224	171	hypothetical protein F19F10.9
Locus_11923_Transcript_1/1_Conf_1.000	362	20	7.46E-42	NP_510509.2	88	173.326	94	83	hypothetical protein F09B12.3
Locus_11924_Transcript_1/1_Conf_1.000	175	0							

Locus_11925_Transcript_1/1_Conf_1.000	560	20	1.01E-60	ACI49101.1	77	236.498	189	146	hypothetical protein Cbre_JD13.003
Locus_11926_Transcript_1/1_Conf_1.000	498	3	8.29E-09	XP_002630376.1	67	63.5438	78	53	Hypothetical protein CBG04315
Locus_11927_Transcript_1/1_Conf_1.000	159	0							
Locus_11928_Transcript_1/2_Conf_1.000	638	0							
Locus_11928_Transcript_2/2_Conf_1.000	438	0							
Locus_11929_Transcript_1/1_Conf_1.000	884	0							
Locus_1193_Transcript_1/1_Conf_1.000	995	20	5.93E-166	ABC86838.1	93	587.8	331	311	depsiphilin
Locus_11930_Transcript_1/1_Conf_1.000	246	0							
Locus_11931_Transcript_1/1_Conf_1.000	175	4	7.26E-13	XP_002645979.1	78	77.0258	56	44	C. briggsae CBR-NHR-48 protein
Locus_11932_Transcript_1/1_Conf_1.000	482	0							
Locus_11933_Transcript_1/1_Conf_1.000	567	7	1.78E-15	XP_001898423.1	47	86.2705	189	90	Mediator complex subunit rgr-1
Locus_11934_Transcript_1/1_Conf_1.000	522	20	1.76E-34	XP_002642109.1	73	149.058	132	97	C. briggsae CBR-CDH-4 protein
Locus_11935_Transcript_1/2_Conf_1.000	339	0							
Locus_11935_Transcript_2/2_Conf_1.000	470	0							
Locus_11936_Transcript_1/1_Conf_1.000	681	0							
Locus_11937_Transcript_1/1_Conf_1.000	302	0							
Locus_11938_Transcript_1/1_Conf_1.000	315	5	9.10E-11	ADG63133.1	59	70.0922	105	62	calcium-dependent apyrase
Locus_11939_Transcript_1/1_Conf_1.000	704	20	4.72E-69	NP_508706.2	69	265.003	247	172	NCK (Non-Catalytic region of tyrosine Kinase) adaptor protein family member (nck-1)
Locus_1194_Transcript_1/1_Conf_1.000	1108	20	2.06E-24	ACI49169.1	62	117.857	148	92	hypothetical protein Csp3_JD01.001
Locus_11940_Transcript_1/1_Conf_1.000	311	9	5.01E-14	XP_002632974.1	61	80.8777	105	65	Hypothetical protein CBG21734

Locus_11941_Transcript_1/1_Conf_1.000	896	20	4.96E-57	XP_002630704.1	59	225.713	304	180	C. briggsae CBR-PINK-1 protein
Locus_11942_Transcript_1/1_Conf_1.000	556	20	5.06E-52	NP_501797.1	79	207.608	141	112	DiHydroPyrimidinase family member (dhp-2)
Locus_11943_Transcript_1/1_Conf_1.000	278	0							
Locus_11944_Transcript_1/1_Conf_1.000	362	0							
Locus_11945_Transcript_1/1_Conf_1.000	248	14	6.55E-14	ACT34056.1	92	80.4925	50	46	glutamate dehydrogenase
Locus_11946_Transcript_1/1_Conf_1.000	352	0							
Locus_11947_Transcript_1/1_Conf_1.000	390	20	1.04E-27	XP_002646193.1	72	126.331	103	75	Hypothetical protein CBG24498
Locus_11948_Transcript_1/2_Conf_1.000	1448	2	4.20E-18	XP_001891812.1	52	97.4413	218	114	hypothetical protein Bm1_01480
Locus_11948_Transcript_2/2_Conf_1.000	1448	2	4.20E-18	XP_001891812.1	52	97.4413	218	114	hypothetical protein Bm1_01480
Locus_11949_Transcript_1/1_Conf_1.000	359	3	1.02E-14	XP_002647546.1	60	83.1889	102	62	C. briggsae CBR-PME-5 protein
Locus_1195_Transcript_1/1_Conf_1.000	439	20	2.22E-54	NP_491454.2	84	214.927	132	111	hypothetical protein F54D7.2
Locus_11950_Transcript_1/1_Conf_1.000	538	20	3.93E-72	XP_002634910.1	84	274.248	172	145	Hypothetical protein CBG22508
Locus_11951_Transcript_1/1_Conf_1.000	728	0							

Locus_11952_Transcript_1/1_Conf_1.000	384	20	3.62E-12	NP_496283.1	60	74.7146	94	57	Cell-death-Related Nuclease family member (crn-3)
Locus_11953_Transcript_1/1_Conf_1.000	819	0							
Locus_11954_Transcript_1/1_Conf_1.000	330	0							
Locus_11955_Transcript_1/1_Conf_1.000	309	0							
Locus_11956_Transcript_1/1_Conf_1.000	228	0							
Locus_11957_Transcript_1/1_Conf_1.000	366	20	5.51E-21	XP_002634905.1	84	103.99	73	62	Hypothetical protein CBG22501
Locus_11958_Transcript_1/1_Conf_1.000	211	0							
Locus_11959_Transcript_1/1_Conf_1.000	454	20	3.72E-33	EFO18399.1	74	144.436	136	101	geranylgeranyl pyrophosphate synthetase
Locus_1196_Transcript_1/1_Conf_1.000	391	18	1.19E-15	NP_001122782.1	62	86.2705	123	77	hypothetical protein F58G6.9
Locus_11960_Transcript_1/1_Conf_1.000	147	7	2.49E-08	XP_002641319.1	72	62.003	48	35	Hypothetical protein CBG24620
Locus_11961_Transcript_1/1_Conf_1.000	430	20	7.46E-58	NP_491864.2	90	226.483	140	126	hypothetical protein K06A5.8
Locus_11962_Transcript_1/1_Conf_1.000	713	20	5.10E-87	XP_002637195.1	81	324.709	233	189	C. briggsae CBR-LIG-1 protein
Locus_11963_Transcript_1/1_Conf_1.000	198	20	9.61E-13	EFO27986.1	73	76.6406	60	44	hypothetical protein LOAG_00494

Locus_11964_Transcript_1/1_Conf_1.000	365	20	2.75E-20	XP_002639618.1	69	101.679	110	76	Hypothetical protein CBG12331
Locus_11965_Transcript_1/1_Conf_1.000	321	20	1.33E-46	NP_492433.1	87	189.119	106	93	HomoGentisate Oxidase family member (hgo-1)
Locus_11966_Transcript_1/1_Conf_1.000	352	20	2.36E-27	XP_002640036.1	74	125.176	115	86	Hypothetical protein CBG12508
Locus_11967_Transcript_1/1_Conf_1.000	551	0							
Locus_11968_Transcript_1/1_Conf_1.000	205	0							
Locus_11969_Transcript_1/1_Conf_1.000	303	20	1.08E-24	NP_508181.1	81	116.316	82	67	hypothetical protein F53H8.3
Locus_1197_Transcript_1/1_Conf_1.000	2172	6	6.52E-32	XP_002639900.1	44	144.05	510	227	C. briggsae CBR-APR-1 protein
Locus_11970_Transcript_1/1_Conf_1.000	713	3	9.84E-06	AAZ42336.1	66	54.6842	57	38	twitchin kinase
Locus_11971_Transcript_1/1_Conf_1.000	334	2	4.73E-04	XP_001894907.1	71	47.7506	45	32	RNA binding protein
Locus_11972_Transcript_1/1_Conf_1.000	1334	7	3.94E-92	XP_002631715.1	60	343.199	450	273	Hypothetical protein CBG20916
Locus_11973_Transcript_1/1_Conf_1.000	693	15	6.71E-20	CAN99691.2	50	101.679	222	112	C. elegans protein F54B3.1b, partially confirmed by transcript evidence
Locus_11974_Transcript_1/1_Conf_1.000	530	3	9.51E-07	Q60YF6.3	51	56.9954	102	53	Mediator of RNA polymerase II transcription subunit 29
Locus_11975_Transcript_1/1_Conf_1.000	278	0							
Locus_11976_Transcript_1/1_Conf_1.000	689	4	3.49E-13	NP_001023362.1	72	79.337	103	75	Temporarily Assigned Gene name family member (tag-261)

Locus_11977_Transcript_1/3_Conf_0.600	421	20	2.29E-30	NP_492796.1	72	135.191	117	85	hypothetical protein C34B2.6
Locus_11977_Transcript_2/3_Conf_0.600	526	20	3.51E-30	NP_492796.1	74	134.806	113	84	hypothetical protein C34B2.6

Locus_11977_Transcript_3/3_Conf_0.600	421	20	2.29E-30	NP_492796.1	72	135.191	117	85	hypothetical protein C34B2.6
Locus_11978_Transcript_1/1_Conf_1.000	387	20	2.16E-49	A8XV40.2	85	198.364	128	110	Probable spastin homolog spas-1
Locus_11979_Transcript_1/1_Conf_1.000	938	20	5.02E-79	XP_002636597.1	87	298.901	194	169	C. briggsae CBR-SKP-1 protein
Locus_1198_Transcript_1/1_Conf_1.000	1408	20	0	XP_002642332.1	91	777.704	468	429	Hypothetical protein CBG18327
Locus_11980_Transcript_1/1_Conf_1.000	231	8	2.99E-14	NP_502273.2	81	81.6481	74	60	UNCoordinated family member (unc-22)

Locus_11981_Transcript_1/1_Conf_1.000	217	3	8.24E-17	XP_001900400.1	73	90.1225	69	51	Zinc finger, C2H2 type family protein
Locus_11982_Transcript_1/1_Conf_1.000	363	20	2.10E-28	NP_507513.2	68	128.642	120	82	hypothetical protein E01B7.1
Locus_11983_Transcript_1/1_Conf_1.000	602	20	2.15E-12	XP_002633896.1	51	76.2554	208	107	Hypothetical protein CBG19958
Locus_11984_Transcript_1/1_Conf_1.000	178	0							
Locus_11985_Transcript_1/1_Conf_1.000	620	0							
Locus_11986_Transcript_1/1_Conf_1.000	281	0							
Locus_11987_Transcript_1/1_Conf_1.000	638	6	2.55E-25	EFO27989.1	56	119.398	148	83	hypothetical protein LOAG_00497
Locus_11988_Transcript_1/1_Conf_1.000	856	0							
Locus_11989_Transcript_1/2_Conf_1.000	558	0							
Locus_11989_Transcript_2/2_Conf_1.000	561	0							
Locus_1199_Transcript_1/2_Conf_1.000	2933	20	0	NP_001040844.1	68	730.709	792	540	Tousled-Like Kinase family member (tlk-1)
Locus_1199_Transcript_2/2_Conf_1.000	2912	20	0	NP_001040844.1	68	733.791	785	541	Tousled-Like Kinase family member (tlk-1)
Locus_11990_Transcript_1/1_Conf_1.000	453	0							
Locus_11991_Transcript_1/1_Conf_1.000	791	20	1.00E-92	NP_495958.1	80	343.969	257	208	yeast SEC homolog family member (sec-5)
Locus_11992_Transcript_1/1_Conf_1.000	374	0							
Locus_11993_Transcript_1/1_Conf_1.000	296	0							
Locus_11994_Transcript_1/1_Conf_1.000	292	0							
Locus_11995_Transcript_1/1_Conf_1.000	363	2	6.78E-11	XP_002635317.1	62	70.4774	80	50	C. briggsae CBR-TAG-196 protein
Locus_11996_Transcript_1/1_Conf_1.000	754	4	1.81E-24	NP_508542.1	85	117.087	121	103	hypothetical protein C15C7.5
Locus_11997_Transcript_1/1_Conf_1.000	614	20	2.09E-74	NP_491711.2	78	282.337	205	161	hypothetical protein C34G6.1
Locus_11998_Transcript_1/1_Conf_1.000	304	20	4.55E-31	XP_002424477.1	94	137.502	79	75	Surfeit locus protein, putative

Locus_11999_Transcript_1/1_Conf_1.000	236	0							
Locus_12_Transcript_1/1_Conf_1.000	1554	20	7.66E-106	NP_492481.1	95	389.037	208	199	RAB family member (rab-5)
Locus_120_Transcript_1/8_Conf_0.410	575	20	3.03E-26	AAO63576.1	54	122.094	199	108	secreted protein 4 precursor
Locus_120_Transcript_2/8_Conf_0.308	404	16	9.93E-10	AAO63576.1	51	66.6254	137	71	secreted protein 4 precursor
Locus_120_Transcript_3/8_Conf_0.179	166	0							
Locus_120_Transcript_4/8_Conf_0.179	179	0							
Locus_120_Transcript_5/8_Conf_0.436	584	20	5.42E-26	AAO63576.1	54	121.324	199	108	secreted protein 4 precursor
Locus_120_Transcript_6/8_Conf_0.462	584	20	3.51E-25	AAO63576.1	53	118.627	199	106	secreted protein 4 precursor
Locus_120_Transcript_8/8_Conf_0.462	584	20	3.18E-26	AAO63576.1	54	122.094	199	108	secreted protein 4 precursor
Locus_1200_Transcript_1/2_Conf_1.000	705	7	1.94E-06	XP_001897045.1	52	56.9954	159	84	hypothetical protein Bm1_27970
Locus_1200_Transcript_2/2_Conf_1.000	917	20	1.02E-28	NP_498671.2	80	131.724	83	67	Uridine PhosPhorylase family member (upp-1)
Locus_12000_Transcript_1/1_Conf_1.000	415	2	6.56E-06	XP_002637729.1	57	53.9138	84	48	Hypothetical protein CBG11595
Locus_12001_Transcript_1/1_Conf_1.000	1113	4	4.65E-16	EFO27437.1	45	90.1225	341	156	origin recognition complex subunit 5
Locus_12002_Transcript_1/1_Conf_1.000	573	0							
Locus_12003_Transcript_1/1_Conf_1.000	183	0							
Locus_12004_Transcript_1/1_Conf_1.000	140	0							
Locus_12005_Transcript_1/1_Conf_1.000	345	0							
Locus_12006_Transcript_1/1_Conf_1.000	249	0							
Locus_12007_Transcript_1/2_Conf_1.000	1111	1	1.82E-04	NP_498338.1	53	51.6026	76	41	hypothetical protein F42A10.5
Locus_12007_Transcript_2/2_Conf_1.000	1105	1	1.38E-04	NP_498338.1	55	51.9878	76	42	hypothetical protein F42A10.5
Locus_12008_Transcript_1/1_Conf_1.000	138	0							

Locus_12009_Transcript_1/1_Conf_1.000	805	20	3.42E-27	NP_501545.1	59	126.331	267	159	RADiation sensitivity abnormal/yeast RAD-related family member (rad-26)
Locus_1201_Transcript_1/1_Conf_1.000	930	20	1.95E-67	EFO26146.1	72	260.381	237	172	hypothetical protein LOAG_02340
Locus_12010_Transcript_1/1_Conf_1.000	634	5	6.44E-13	XP_002639170.1	72	78.1814	80	58	Hypothetical protein CBG03709
Locus_12011_Transcript_1/1_Conf_1.000	415	20	1.14E-50	CAA84656.2	84	202.601	132	112	C. elegans protein F10F2.2, confirmed by transcript evidence
Locus_12012_Transcript_1/1_Conf_1.000	621	0							
Locus_12013_Transcript_1/1_Conf_1.000	254	0							
Locus_12014_Transcript_1/2_Conf_1.000	440	3	2.03E-15	NP_492118.1	56	85.5001	146	82	hypothetical protein D2030.3
Locus_12014_Transcript_2/2_Conf_1.000	440	3	1.55E-15	NP_492118.1	56	85.8853	146	83	hypothetical protein D2030.3
Locus_12015_Transcript_1/1_Conf_1.000	428	20	5.04E-46	XP_002743343.1	100	187.193	96	96	PREDICTED: hypothetical protein LOC100413255
Locus_12016_Transcript_1/1_Conf_1.000	334	3	4.12E-16	XP_002631490.1	71	87.8113	70	50	C. briggsae CBR-EGG-3 protein
Locus_12017_Transcript_1/1_Conf_1.000	740	20	6.09E-110	NP_498645.1	80	400.979	246	198	MUscle Positioning family member (mup-4)
Locus_12018_Transcript_1/1_Conf_1.000	217	0							
Locus_12019_Transcript_1/1_Conf_1.000	392	0							
Locus_1202_Transcript_1/1_Conf_1.000	179	20	1.72E-22	NP_491853.2	94	108.997	57	54	SyNapTotagmin family member (snt-4)
Locus_12020_Transcript_1/1_Conf_1.000	152	0							

Locus_12021_Transcript_1/1_Conf_1.000	486	8	4.74E-44	XP_002631275.1	66	180.644	163	108	Hypothetical protein CBG03085
Locus_12022_Transcript_1/1_Conf_1.000	274	20	6.34E-33	NP_498117.3	86	143.665	91	79	Gro-1 OPeron gene family member (gop-1)
Locus_12023_Transcript_1/1_Conf_1.000	393	20	2.02E-18	NP_494788.2	58	95.5153	137	80	related to yeast Vacuolar Protein Sorting factor family member (vps-18)
Locus_12024_Transcript_1/1_Conf_1.000	305	0							
Locus_12025_Transcript_1/1_Conf_1.000	863	20	1.27E-62	BAE91269.1	100	244.202	116	116	unnamed protein product
Locus_12026_Transcript_1/1_Conf_1.000	309	20	2.32E-19	XP_002633895.1	69	98.5969	92	64	C. briggsae CBR-SAND-1 protein
Locus_12027_Transcript_1/1_Conf_1.000	162	0							
Locus_12028_Transcript_1/1_Conf_1.000	585	0							
Locus_12029_Transcript_1/1_Conf_1.000	529	0							
Locus_1203_Transcript_1/2_Conf_1.000	3600	20	1.36E-64	NP_497706.1	45	253.447	1002	459	hypothetical protein F59A2.6
Locus_1203_Transcript_2/2_Conf_1.000	3525	20	3.89E-56	NP_497706.1	44	225.328	984	436	hypothetical protein F59A2.6
Locus_12030_Transcript_1/1_Conf_1.000	430	0							
Locus_12031_Transcript_1/1_Conf_1.000	592	20	5.21E-24	XP_001892273.1	54	114.775	202	110	hypothetical protein
Locus_12032_Transcript_1/1_Conf_1.000	210	20	1.12E-13	XP_002755896.1	70	79.7221	68	48	PREDICTED: dnaJ homolog subfamily A member 3, mitochondrial
Locus_12033_Transcript_1/1_Conf_1.000	407	2	2.20E-09	NP_501176.1	80	65.4698	47	38	hypothetical protein T12B3.3
Locus_12034_Transcript_1/1_Conf_1.000	250	3	1.84E-16	AAC39119.1	67	88.9669	83	56	low-affinity peptide transporter
Locus_12035_Transcript_1/1_Conf_1.000	277	0							
Locus_12036_Transcript_1/1_Conf_1.000	1417	20	4.58E-78	NP_508977.1	57	296.59	402	230	hypothetical protein F13D11.1
Locus_12037_Transcript_1/1_Conf_1.000	269	9	6.66E-06	CBM41222.1	50	53.9138	63	32	C. elegans protein T10G3.3b, partially confirmed by transcript evidence
Locus_12038_Transcript_1/1_Conf_1.000	203	0							
Locus_12039_Transcript_1/1_Conf_1.000	574	10	1.54E-54	XP_002629691.1	74	216.083	194	144	Hypothetical protein CBG00912

Locus_1204_Transcript_1/2_Conf_1.000	4436	20	0	NP_001021649.1	81	1652.88	1207	982	HaMmeRhead embryonic lethal family member (hmr-1)
Locus_1204_Transcript_2/2_Conf_1.000	4558	20	0	NP_001021649.1	81	1652.88	1207	982	HaMmeRhead embryonic lethal family member (hmr-1)
Locus_12040_Transcript_1/1_Conf_1.000	296	0							
Locus_12041_Transcript_1/1_Conf_1.000	975	20	5.20E-167	XP_001898368.1	93	591.267	324	303	cpsf3-prov protein
Locus_12042_Transcript_1/1_Conf_1.000	824	8	3.52E-35	CAX51629.1	57	152.91	266	152	C. elegans protein C48B4.4d, partially confirmed by transcript evidence
Locus_12043_Transcript_1/1_Conf_1.000	794	20	1.16E-96	CAA45963.1	100	357.066	208	208	BBC1
Locus_12044_Transcript_1/1_Conf_1.000	835	20	4.68E-75	NP_495741.2	88	285.419	210	186	hypothetical protein K01C8.1
Locus_12045_Transcript_1/1_Conf_1.000	171	0							
Locus_12046_Transcript_1/1_Conf_1.000	308	20	7.46E-34	XP_002636068.1	79	146.747	102	81	Hypothetical protein CBG01308
Locus_12047_Transcript_1/1_Conf_1.000	967	20	2.03E-22	XP_001702729.1	70	110.923	100	70	ubiquitin-related modifier
Locus_12048_Transcript_1/1_Conf_1.000	380	0							

Locus_12049_Transcript_1/1_Conf_1.000	240	20	1.57E-15	NP_510155.3	73	85.8853	78	57	PIP Kinase family member (ppk-3)
Locus_1205_Transcript_1/1_Conf_1.000	205	7	3.40E-10	EFO26077.1	80	68.1662	51	41	leucine Rich Repeat family protein
Locus_12050_Transcript_1/1_Conf_1.000	2566	20	3.31E-179	XP_002639041.1	73	633.639	596	437	C. briggsae CBR-XNP-1 protein
Locus_12051_Transcript_1/1_Conf_1.000	750	20	1.14E-18	NP_499886.1	50	97.8265	196	98	hypothetical protein R02D3.7
Locus_12052_Transcript_1/1_Conf_1.000	645	20	6.67E-61	NP_498248.2	67	237.654	214	144	hypothetical protein Y37B11A.2
Locus_12053_Transcript_1/1_Conf_1.000	188	0							
Locus_12054_Transcript_1/1_Conf_1.000	687	20	8.18E-71	NP_504753.3	77	270.781	239	185	UNCoordinated family member (unc-68)
Locus_12055_Transcript_1/1_Conf_1.000	464	20	9.22E-72	XP_001900091.1	90	272.707	153	139	U5 small nuclear ribonucleoprotein 200 kDa helicase
Locus_12056_Transcript_1/1_Conf_1.000	688	20	2.90E-76	CAR63592.1	82	288.886	194	160	hypothetical protein

Locus_12057_Transcript_1/1_Conf_1.000	214	20	2.48E-21	NP_499949.2	87	105.145	71	62	Gut on EXterior family member (gex-2)
Locus_12058_Transcript_1/1_Conf_1.000	280	20	5.14E-43	NP_492127.1	95	177.178	93	89	Low-density lipoprotein Receptor Related family member (lrp-1)
Locus_12059_Transcript_1/1_Conf_1.000	619	20	4.21E-46	EFO25304.1	70	188.348	211	149	leucine Rich Repeat family protein
Locus_1206_Transcript_1/4_Conf_0.625	596	20	1.59E-20	XP_001895554.1	56	103.219	158	90	SWIRM domain containing protein
Locus_1206_Transcript_2/4_Conf_0.625	592	20	1.46E-26	XP_001895554.1	62	123.25	155	97	SWIRM domain containing protein
Locus_1206_Transcript_3/4_Conf_0.625	732	20	8.83E-29	EFO20641.1	63	131.339	154	98	hypothetical protein LOAG_07849

Locus_1206_Transcript_4/4_Conf_0.625	592	20	4.12E-29	EFO20641.1	63	131.724	156	99	hypothetical protein LOAG_07849
Locus_12060_Transcript_1/1_Conf_1.000	580	20	1.23E-14	XP_001900371.1	51	83.5741	198	102	hypothetical protein
Locus_12061_Transcript_1/1_Conf_1.000	152	0							
Locus_12062_Transcript_1/1_Conf_1.000	755	20	6.33E-78	NP_506083.1	77	294.664	254	196	hypothetical protein F55C5.8
Locus_12063_Transcript_1/1_Conf_1.000	264	9	6.24E-12	NP_001022773.1	58	73.9442	107	63	Myc and Mondo-Like family member (mml-1)
Locus_12064_Transcript_1/1_Conf_1.000	1005	20	2.87E-91	EFO27641.1	75	339.732	333	251	symplekin
Locus_12065_Transcript_1/1_Conf_1.000	586	0							
Locus_12066_Transcript_1/1_Conf_1.000	653	0							
Locus_12067_Transcript_1/1_Conf_1.000	386	20	1.24E-28	XP_002643566.1	69	129.413	114	79	Hypothetical protein CBG16268
Locus_12068_Transcript_1/1_Conf_1.000	446	2	3.93E-06	XP_001900477.1	64	54.6842	57	37	HMG box family protein
Locus_12069_Transcript_1/1_Conf_1.000	286	3	2.47E-24	XP_002631432.1	90	115.161	76	69	Hypothetical protein CBG03290
Locus_1207_Transcript_1/1_Conf_1.000	199	1	1.94E-05	XP_001624571.1	73	52.373	42	31	predicted protein
Locus_12070_Transcript_1/1_Conf_1.000	502	20	3.92E-30	XP_002629838.1	86	134.42	83	72	Hypothetical protein CBG18727
Locus_12071_Transcript_1/1_Conf_1.000	350	0							
Locus_12072_Transcript_1/2_Conf_1.000	1412	7	1.86E-47	XP_002635238.1	58	194.897	258	151	Hypothetical protein CBG11482
Locus_12072_Transcript_2/2_Conf_1.000	1388	7	1.08E-47	XP_002635238.1	56	195.667	279	159	Hypothetical protein CBG11482

Locus_12073_Transcript_1/1_Conf_1.000	1207	20	3.07E-117	XP_001897522.1	81	426.402	306	248	Zinc knuckle family protein
Locus_12074_Transcript_1/2_Conf_1.000	538	20	7.22E-42	XP_002399529.1	75	173.711	140	105	tpr repeat nuclear phosphoprotein, putative
Locus_12074_Transcript_2/2_Conf_1.000	776	4	6.81E-30	CAX51623.1	61	135.191	202	125	C. elegans protein B0464.2d, partially confirmed by transcript evidence
Locus_12075_Transcript_1/1_Conf_1.000	183	0							
Locus_12076_Transcript_1/1_Conf_1.000	147	0							
Locus_12077_Transcript_1/1_Conf_1.000	1383	20	5.17E-143	NP_496090.1	76	512.301	435	333	hypothetical protein ZK1320.9
Locus_12078_Transcript_1/1_Conf_1.000	483	20	4.19E-24	XP_001899004.1	65	114.39	122	80	co-chaperone protein HscB, mitochondrial precursor
Locus_12079_Transcript_1/1_Conf_1.000	193	0							
Locus_1208_Transcript_1/1_Conf_1.000	610	20	5.58E-88	NP_508413.1	96	327.405	191	185	Ribosomal Protein, Large subunit family member (rpl-11.2)
Locus_12080_Transcript_1/1_Conf_1.000	1569	20	4.79E-164	XP_002634686.1	74	582.408	522	390	Hypothetical protein CBG19672
Locus_12081_Transcript_1/1_Conf_1.000	803	20	1.19E-64	NP_502781.1	84	250.751	224	190	hypothetical protein Y57G11C.4
Locus_12082_Transcript_1/1_Conf_1.000	1005	20	9.39E-34	EFO13829.1	65	148.673	156	102	hypothetical protein LOAG_14699
Locus_12083_Transcript_1/1_Conf_1.000	460	20	1.15E-10	EFO17978.1	69	69.707	49	34	dual specificity phosphatase

Locus_12084_Transcript_1/1_Conf_1.000	301	0							
Locus_12085_Transcript_1/2_Conf_1.000	475	0							
Locus_12085_Transcript_2/2_Conf_1.000	489	0							
Locus_12086_Transcript_1/1_Conf_1.000	161	0							
Locus_12087_Transcript_1/1_Conf_1.000	464	0							
Locus_12088_Transcript_1/1_Conf_1.000	776	0							
Locus_12089_Transcript_1/1_Conf_1.000	290	0							
Locus_1209_Transcript_1/1_Conf_1.000	779	20	1.17E-37	NP_494880.1	74	160.999	204	151	COLlagen family member (col-73)
Locus_12090_Transcript_1/1_Conf_1.000	1134	20	2.51E-57	NP_497893.1	76	227.254	185	141	hypothetical protein R07E5.1
Locus_12091_Transcript_1/2_Conf_1.000	195	0							
Locus_12091_Transcript_2/2_Conf_1.000	397	0							
Locus_12092_Transcript_1/1_Conf_1.000	814	0							
Locus_12093_Transcript_1/1_Conf_1.000	309	0							
Locus_12094_Transcript_1/1_Conf_1.000	428	20	2.42E-48	EFO18868.1	84	194.897	130	110	hypothetical protein LOAG_09629
Locus_12095_Transcript_1/1_Conf_1.000	134	0							
Locus_12096_Transcript_1/1_Conf_1.000	559	3	1.69E-15	NP_500893.2	84	86.2705	50	42	hypothetical protein K02B2.1
Locus_12097_Transcript_1/2_Conf_1.000	1557	3	1.52E-53	NP_498952.1	68	215.312	199	136	hypothetical protein F44E2.4
Locus_12097_Transcript_2/2_Conf_1.000	1554	3	1.52E-53	NP_498952.1	68	215.312	199	136	hypothetical protein F44E2.4
Locus_12098_Transcript_1/1_Conf_1.000	1068	20	4.85E-100	NP_495424.2	75	369.007	299	226	hypothetical protein F22D3.6
Locus_12099_Transcript_1/1_Conf_1.000	426	0							
Locus_121_Transcript_1/7_Conf_0.375	150	0							
Locus_121_Transcript_2/7_Conf_0.375	156	0							
Locus_121_Transcript_3/7_Conf_0.375	310	0							
Locus_121_Transcript_4/7_Conf_0.667	423	0							
Locus_121_Transcript_5/7_Conf_0.167	162	0							
Locus_121_Transcript_6/7_Conf_0.375	259	0							
Locus_121_Transcript_7/7_Conf_0.375	150	0							
Locus_1210_Transcript_1/1_Conf_1.000	736	6	1.09E-26	NP_500907.1	63	124.405	135	86	Prion-like-(Q/N-rich)-domain-bearing protein family member (pqn-22)
Locus_12100_Transcript_1/1_Conf_1.000	360	0							
Locus_12101_Transcript_1/1_Conf_1.000	444	0							
Locus_12102_Transcript_1/1_Conf_1.000	532	20	3.16E-82	XP_002638801.1	89	307.76	177	159	Hypothetical protein CBG05158
Locus_12103_Transcript_1/1_Conf_1.000	1160	20	9.14E-55	XP_002632325.1	76	218.779	184	140	Hypothetical protein CBG00332

Locus_12104_Transcript_1/1_Conf_1.000	235	3	4.91E-25	XP_002644048.1	55	117.472	128	71	Hypothetical protein CBG17510
Locus_12105_Transcript_1/1_Conf_1.000	210	0							
Locus_12106_Transcript_1/1_Conf_1.000	438	20	8.76E-35	EFO24963.1	82	149.828	111	92	hypothetical protein LOAG_03520
Locus_12107_Transcript_1/1_Conf_1.000	358	20	9.17E-32	XP_002642780.1	75	139.813	115	87	Hypothetical protein CBG21174
Locus_12108_Transcript_1/1_Conf_1.000	287	20	1.31E-25	CAA22110.2	78	119.398	91	71	C. elegans protein Y75B8A.26, partially confirmed by transcript evidence
Locus_12109_Transcript_1/1_Conf_1.000	323	20	4.47E-18	XP_002637707.1	67	94.3597	107	72	Hypothetical protein CBG11572
Locus_1211_Transcript_1/1_Conf_1.000	1856	20	0	EFO19100.1	95	1095.49	605	575	U5 snRNP-specific protein
Locus_12110_Transcript_1/1_Conf_1.000	578	0							
Locus_12111_Transcript_1/1_Conf_1.000	406	0							
Locus_12112_Transcript_1/1_Conf_1.000	486	4	3.41E-26	XP_002639317.1	63	121.324	144	92	C. briggsae CBR-ABT-2 protein
Locus_12113_Transcript_1/1_Conf_1.000	390	4	2.10E-20	XP_002646179.1	65	102.064	125	82	Hypothetical protein CBG23745
Locus_12114_Transcript_1/1_Conf_1.000	1113	20	4.80E-21	XP_001892006.1	60	106.686	172	104	1300013D05Rik protein
Locus_12115_Transcript_1/1_Conf_1.000	182	0							

Locus_12116_Transcript_1/1_Conf_1.000	201	20	1.09E-24	NP_001822.2	100	116.316	66	66	clusterin isoform 1
Locus_12117_Transcript_1/1_Conf_1.000	1033	4	1.74E-22	ABS19466.2	46	111.309	305	142	Hypothetical protein F49D11.10
Locus_12118_Transcript_1/1_Conf_1.000	692	0							
Locus_12119_Transcript_1/1_Conf_1.000	657	0							
Locus_1212_Transcript_1/2_Conf_1.000	1732	6	1.80E-58	NP_494278.3	52	231.876	336	177	Prion-like-(Q/N-rich)-domain-bearing protein family member (pqn-42)
Locus_1212_Transcript_2/2_Conf_1.000	1179	6	4.07E-58	NP_494278.3	52	229.95	324	171	Prion-like-(Q/N-rich)-domain-bearing protein family member (pqn-42)
Locus_12120_Transcript_1/1_Conf_1.000	227	0							
Locus_12121_Transcript_1/1_Conf_1.000	257	20	4.07E-27	XP_001898425.1	89	124.405	85	76	RuvB-like 2
Locus_12122_Transcript_1/1_Conf_1.000	388	20	2.85E-09	NP_001007899.1	54	65.0846	96	52	methyltransferase like 9
Locus_12123_Transcript_1/1_Conf_1.000	343	20	2.72E-31	CBM41226.1	76	138.272	113	87	C. elegans protein T21C9.2b, confirmed by transcript evidence
Locus_12124_Transcript_1/1_Conf_1.000	408	20	5.32E-64	XP_002636978.1	92	246.899	134	124	Hypothetical protein CBG09461

Locus_12125_Transcript_1/1_Conf_1.000	496	20	9.42E-45	XP_001895465.1	70	182.956	164	115	EGF-like domain containing protein
Locus_12126_Transcript_1/1_Conf_1.000	236	20	1.34E-14	ADI61822.1	70	82.8037	77	54	endonuclease-reverse transcriptase
Locus_12127_Transcript_1/1_Conf_1.000	531	20	4.31E-47	XP_002633395.1	84	191.045	167	141	C. briggsae CBR-KIN-4 protein
Locus_12128_Transcript_1/1_Conf_1.000	510	20	3.03E-33	NP_504831.1	76	144.821	117	90	hypothetical protein C13F10.2
Locus_12129_Transcript_1/1_Conf_1.000	149	20	1.56E-10	NP_491508.2	79	69.3218	48	38	PHAryngeal gland Toxin-related family member (phat-1)
Locus_1213_Transcript_1/4_Conf_0.375	536	20	1.58E-30	XP_002630755.1	64	132.494	122	79	C. briggsae CBR-LYS-8 protein
Locus_1213_Transcript_2/4_Conf_0.250	234	0							
Locus_1213_Transcript_3/4_Conf_0.500	968	20	4.18E-60	XP_002630755.1	67	236.113	208	140	C. briggsae CBR-LYS-8 protein
Locus_1213_Transcript_4/4_Conf_0.500	962	20	1.28E-61	XP_002630755.1	67	241.121	206	140	C. briggsae CBR-LYS-8 protein
Locus_12130_Transcript_1/1_Conf_1.000	249	20	6.53E-14	XP_002631442.1	80	80.4925	81	65	Hypothetical protein CBG03304
Locus_12131_Transcript_1/1_Conf_1.000	592	0							
Locus_12132_Transcript_1/1_Conf_1.000	377	0							
Locus_12133_Transcript_1/1_Conf_1.000	411	0							
Locus_12134_Transcript_1/2_Conf_1.000	962								
Locus_12134_Transcript_2/2_Conf_1.000	978								
Locus_12135_Transcript_1/1_Conf_1.000	407	0							

Locus_12136_Transcript_1/1_Conf_1.000	880	20	1.23E-153	NP_494939.1	93	546.584	292	274	PhosphoLipase D family member (pld-1)
Locus_12137_Transcript_1/1_Conf_1.000	729	20	3.98E-45	NP_505512.3	65	152.14	195	128	AQuaPorin or aquaglyceroporin related family member (aqp-4)
Locus_12138_Transcript_1/1_Conf_1.000	757	20	1.05E-96	NP_502164.1	85	357.066	239	204	WHiTe (Drosophila) related ABC transporte family member (wht-2)
Locus_12139_Transcript_1/1_Conf_1.000	204	0							
Locus_1214_Transcript_1/2_Conf_1.000	493	20	5.93E-47	NP_499522.2	93	190.274	121	113	GBF1(Golgi-specific Brefeldin-A-resistant Factor 1) homolog family member (gbf-1)

Locus_1214_Transcript_2/2_Conf_1.000	493	20	5.93E-47	NP_499522.2	93	190.274	121	113	GBF1(Golgi-specific Brefeldin-A-resistant Factor 1) homolog family member (gbf-1)
Locus_12140_Transcript_1/1_Conf_1.000	1133	0							
Locus_12141_Transcript_1/1_Conf_1.000	409	5	3.38E-18	NP_491750.3	64	94.7449	134	87	hypothetical protein C27A12.9
Locus_12142_Transcript_1/1_Conf_1.000	463	1	8.49E-09	NP_498245.2	57	63.5438	142	81	related to Islet cell Diabetes Autoantigen family member (ida-1)
Locus_12143_Transcript_1/1_Conf_1.000	181	0							
Locus_12144_Transcript_1/1_Conf_1.000	1954	20	1.00E-60	NP_494841.3	49	239.58	643	316	hypothetical protein F10G7.10
Locus_12145_Transcript_1/1_Conf_1.000	751	20	2.86E-78	EFO16473.1	76	295.819	226	173	zinc finger protein
Locus_12146_Transcript_1/1_Conf_1.000	646	20	4.81E-35	XP_002640142.1	66	151.754	160	107	C. briggsae CBR-DBR-1 protein
Locus_12147_Transcript_1/1_Conf_1.000	558	20	2.26E-44	EFO25953.1	64	182.185	189	122	TK/Met protein kinase
Locus_12148_Transcript_1/1_Conf_1.000	345	20	2.90E-33	NP_498634.1	80	144.821	111	89	hypothetical protein T20B12.3

Locus_12149_Transcript_1/1_Conf_1.000	252	4	2.57E-13	XP_001894170.1	64	78.5666	82	53	Ribonucleases P/MRP protein subunit POP1 containing protein
Locus_1215_Transcript_1/3_Conf_0.600	1864	20	0	CAB40412.1	91	759.214	472	434	hexokinase
Locus_1215_Transcript_2/3_Conf_0.400	1728	20	0	CAB40412.1	94	750.74	448	423	hexokinase
Locus_1215_Transcript_3/3_Conf_0.600	1863	20	0	CAB40412.1	92	775.778	483	446	hexokinase
Locus_12150_Transcript_1/1_Conf_1.000	331	0							
Locus_12151_Transcript_1/1_Conf_1.000	575	0							
Locus_12152_Transcript_1/1_Conf_1.000	451	20	2.96E-30	NP_001041050.1	70	134.806	117	82	hypothetical protein Y57G11C.3
Locus_12153_Transcript_1/1_Conf_1.000	1755	20	1.86E-127	NP_496546.2	68	461.07	487	336	hypothetical protein W03C9.6
Locus_12154_Transcript_1/1_Conf_1.000	397	20	2.38E-11	XP_002638990.1	61	72.0182	88	54	Hypothetical protein CBG22236
Locus_12155_Transcript_1/1_Conf_1.000	503	20	1.71E-25	XP_002641945.1	64	119.013	121	78	C. briggsae CBR-TAG-198 protein
Locus_12156_Transcript_1/1_Conf_1.000	665	20	1.75E-27	XP_001894040.1	56	126.716	166	93	F-box domain containing protein
Locus_12157_Transcript_1/1_Conf_1.000	138	0							

Locus_12158_Transcript_1/1_Conf_1.000	875	20	2.11E-20	XP_002640302.1	48	103.99	286	139	Hypothetical protein CBG12832
Locus_12159_Transcript_1/1_Conf_1.000	1760	20	1.15E-169	XP_002634860.1	72	601.282	536	386	Hypothetical protein CBG13978
Locus_1216_Transcript_1/1_Conf_1.000	1826	20	1.18E-124	NP_509391.1	58	451.825	569	335	hypothetical protein M60.2
Locus_12160_Transcript_1/1_Conf_1.000	422	0							
Locus_12161_Transcript_1/2_Conf_1.000	925	20	2.16E-34	NP_499533.1	85	150.599	91	78	SR Protein (splicing factor) family member (rsp-8)
Locus_12161_Transcript_2/2_Conf_1.000	318	0							
Locus_12162_Transcript_1/1_Conf_1.000	454	13	4.12E-24	NP_492324.2	58	114.39	138	81	hypothetical protein R05D11.9
Locus_12163_Transcript_1/1_Conf_1.000	407	20	8.63E-22	EFO25480.1	65	106.686	117	77	hypothetical protein LOAG_03009
Locus_12164_Transcript_1/1_Conf_1.000	146	0							

Locus_12165_Transcript_1/1_Conf_1.000	128	20	7.83E-15	XP_001110023.2	100	83.5741	42	42	PREDICTED: sentrin-specific protease 3-like isoform 3
Locus_12166_Transcript_1/1_Conf_1.000	593	20	2.57E-47	NP_501908.2	67	192.2	182	123	hypothetical protein Y43C5B.3
Locus_12167_Transcript_1/1_Conf_1.000	470	20	3.59E-68	XP_001231438.1	100	260.766	148	148	PREDICTED: similar to ribosomal protein L26 isoform 1
Locus_12168_Transcript_1/1_Conf_1.000	313	0							
Locus_12169_Transcript_1/1_Conf_1.000	355	0							
Locus_1217_Transcript_1/1_Conf_1.000	1216	20	1.67E-70	EFO17798.1	60	271.166	346	210	hypothetical protein LOAG_10699
Locus_12170_Transcript_1/1_Conf_1.000	279	0							
Locus_12171_Transcript_1/1_Conf_1.000	442	0							
Locus_12172_Transcript_1/1_Conf_1.000	217	20	5.89E-23	NP_499273.3	84	110.538	71	60	hypothetical protein R01H10.7
Locus_12173_Transcript_1/1_Conf_1.000	251	3	2.25E-06	EFO15975.1	64	55.4546	68	44	hypothetical protein LOAG_12534
Locus_12174_Transcript_1/2_Conf_1.000	1249	2	1.31E-17	NP_507948.2	48	95.5153	179	87	DAF-16/FOXO Controlled, germline Tumor affecting family member (dct-10)
Locus_12174_Transcript_2/2_Conf_1.000	1249	2	2.64E-18	NP_507948.2	48	97.8265	179	87	DAF-16/FOXO Controlled, germline Tumor affecting family member (dct-10)
Locus_12175_Transcript_1/1_Conf_1.000	989	20	1.58E-78	XP_002641183.1	89	297.36	213	191	Hypothetical protein CBG09044
Locus_12176_Transcript_1/1_Conf_1.000	412	4	2.50E-05	XP_001900130.1	68	51.9878	44	30	hypothetical protein
Locus_12177_Transcript_1/1_Conf_1.000	403	0							

Locus_12178_Transcript_1/1_Conf_1.000	822	20	1.72E-90	XP_002640833.1	85	336.65	276	236	C. briggsae CBR-KLP-20 protein
Locus_12179_Transcript_1/1_Conf_1.000	566	0							
Locus_1218_Transcript_1/1_Conf_1.000	4106	20	0	XP_001893907.1	68	1054.66	1287	886	hypothetical protein
Locus_12180_Transcript_1/1_Conf_1.000	188	20	4.24E-08	P34569.4	60	61.2326	61	37	Kelch repeat-containing protein kel-10
Locus_12181_Transcript_1/1_Conf_1.000	371	20	3.43E-34	NP_497510.2	80	147.902	111	89	hypothetical protein C39B5.6
Locus_12182_Transcript_1/1_Conf_1.000	133	0							
Locus_12183_Transcript_1/1_Conf_1.000	593	2	2.06E-04	EFO24041.1	56	49.6766	55	31	hypothetical protein LOAG_04441
Locus_12184_Transcript_1/2_Conf_1.000	1770	20	1.13E-39	EFO23952.1	45	169.474	526	237	hypothetical protein LOAG_04537

Locus_12184_Transcript_2/2_Conf_1.000	1770	20	1.13E-39	EFO23952.1	45	169.474	526	237	hypothetical protein LOAG_04537
Locus_12185_Transcript_1/1_Conf_1.000	528	20	2.64E-57	NP_501339.2	77	224.942	171	132	neuRonal IGCAM family member (rig-4)
Locus_12186_Transcript_1/1_Conf_1.000	390	1	7.48E-10	NP_001129792.1	73	67.0106	56	41	hypothetical protein T23G11.11
Locus_12187_Transcript_1/1_Conf_1.000	896	3	1.12E-16	NP_500551.2	62	91.6633	293	183	hypothetical protein F55F10.1
Locus_12188_Transcript_1/1_Conf_1.000	299	0							
Locus_12189_Transcript_1/1_Conf_1.000	723	19	8.04E-27	NP_503051.1	72	124.79	123	89	FMRF-Like Peptide family member (flp-17)
Locus_1219_Transcript_1/1_Conf_1.000	177	0							
Locus_12190_Transcript_1/1_Conf_1.000	187	0							
Locus_12191_Transcript_1/2_Conf_1.000	1129	20	1.78E-55	XP_002636552.1	62	221.09	297	185	C. briggsae CBR-SULP-4 protein
Locus_12191_Transcript_2/2_Conf_1.000	494	20	2.78E-12	NP_505493.1	68	75.0998	74	51	SULfate Permease family member (sulp-8)
Locus_12192_Transcript_1/1_Conf_1.000	757	20	6.67E-43	ABC69942.1	63	178.333	190	120	STIP
Locus_12193_Transcript_1/1_Conf_1.000	425	20	5.82E-26	NP_494922.1	59	120.553	127	76	hypothetical protein F59A6.4
Locus_12194_Transcript_1/1_Conf_1.000	276	20	5.74E-18	NP_495183.1	80	93.9745	66	53	FRataxin (involved in human Friedrich's ataxia) Homolog family member (frh-1)

Locus_12195_Transcript_1/1_Conf_1.000	1120	20	1.15E-155	NP_497603.3	88	553.903	373	329	hypothetical protein Y71H2AM.9
Locus_12196_Transcript_1/1_Conf_1.000	315	20	1.59E-31	XP_002637541.1	78	139.043	104	82	C. briggsae CBR-HUM-2 protein
Locus_12197_Transcript_1/1_Conf_1.000	138	1	1.62E-04	NP_500104.1	81	49.2914	43	35	Nuclear Pore complex Protein family member (npp-8)
Locus_12198_Transcript_1/1_Conf_1.000	370	20	3.01E-22	XP_002632893.1	82	108.227	74	61	Hypothetical protein CBG15101
Locus_12199_Transcript_1/1_Conf_1.000	578	0							
Locus_122_Transcript_1/10_Conf_0.500	1018	20	3.88E-43	NP_509305.1	72	179.874	170	124	VITellogenin structural genes (yolk protein genes) family member (vit-1)
Locus_122_Transcript_10/10_Conf_0.312	573	20	1.00E-45	NP_509305.1	69	186.808	191	133	VITellogenin structural genes (yolk protein genes) family member (vit-1)
Locus_122_Transcript_2/10_Conf_0.500	1018	20	2.87E-46	NP_509305.1	72	190.274	182	132	VITellogenin structural genes (yolk protein genes) family member (vit-1)
Locus_122_Transcript_3/10_Conf_0.312	244	2	7.29E-05	NP_509305.1	77	50.447	45	35	VITellogenin structural genes (yolk protein genes) family member (vit-1)
Locus_122_Transcript_4/10_Conf_0.312	244	13	1.57E-07	NP_509305.1	73	59.3066	57	42	VITellogenin structural genes (yolk protein genes) family member (vit-1)

Locus_122_Transcript_5/10_Conf_0.281	1052	20	1.77E-30	NP_509305.1	75	137.887	119	90	VITellogenin structural genes (yolk protein genes) family member (vit-1)
Locus_122_Transcript_6/10_Conf_0.281	1094	20	1.39E-33	NP_509305.1	74	148.288	131	98	VITellogenin structural genes (yolk protein genes) family member (vit-1)
Locus_122_Transcript_7/10_Conf_0.281	1052	20	1.77E-30	NP_509305.1	75	137.887	119	90	VITellogenin structural genes (yolk protein genes) family member (vit-1)
Locus_122_Transcript_8/10_Conf_0.062	564	6	3.91E-07	NP_001040703.1	66	58.5362	74	49	bZIP transcription factor family member (zip-4)
Locus_122_Transcript_9/10_Conf_0.312	573	20	1.82E-47	NP_509305.1	69	192.586	198	138	VITellogenin structural genes (yolk protein genes) family member (vit-1)
Locus_1220_Transcript_1/2_Conf_1.000	486	20	1.82E-11	CAD26968.1	52	72.4034	106	56	transposase
Locus_1220_Transcript_2/2_Conf_1.000	317	20	7.44E-13	CAD26968.1	58	77.0258	89	52	transposase
Locus_12200_Transcript_1/1_Conf_1.000	194	0							
Locus_12201_Transcript_1/1_Conf_1.000	1099	20	4.97E-55	NP_496426.1	77	219.55	175	135	hypothetical protein B0491.1
Locus_12202_Transcript_1/1_Conf_1.000	241	20	1.16E-18	EFO26239.1	77	96.2857	71	55	hypothetical protein LOAG_02251
Locus_12203_Transcript_1/1_Conf_1.000	270	0							
Locus_12204_Transcript_1/1_Conf_1.000	291	4	4.19E-16	XP_002646250.1	65	87.8113	91	60	C. briggsae CBR-AEX-1 protein

Locus_12205_Transcript_1/1_Conf_1.000	528	20	2.38E-66	NP_498192.2	95	254.988	144	137	hypothetical protein F09F7.3
Locus_12206_Transcript_1/1_Conf_1.000	242	6	1.13E-05	XP_001897023.1	53	53.1434	76	41	RNA dependent RNA polymerase family protein
Locus_12207_Transcript_1/1_Conf_1.000	524	0							
Locus_12208_Transcript_1/1_Conf_1.000	562	20	1.65E-50	XP_002633700.1	77	202.601	173	134	Hypothetical protein CBG03383
Locus_12209_Transcript_1/1_Conf_1.000	293	20	2.29E-22	XP_002639062.1	70	108.612	95	67	Hypothetical protein CBG14873
Locus_1221_Transcript_1/1_Conf_1.000	260	0							
Locus_12210_Transcript_1/1_Conf_1.000	1963	20	3.52E-122	EFO20200.1	59	443.736	620	370	hypothetical protein LOAG_08289
Locus_12211_Transcript_1/1_Conf_1.000	252	0							
Locus_12212_Transcript_1/2_Conf_1.000	336	0							
Locus_12212_Transcript_2/2_Conf_1.000	833	20	2.50E-28	XP_001896132.1	78	130.183	108	85	hypothetical protein
Locus_12213_Transcript_1/1_Conf_1.000	686	0							
Locus_12214_Transcript_1/1_Conf_1.000	401	5	4.15E-40	XP_001898210.1	76	167.548	117	89	hypothetical protein Bm1_33815
Locus_12215_Transcript_1/1_Conf_1.000	391	20	2.82E-33		67	144.821	121	82	DNA topoisomerase II
Locus_12216_Transcript_1/3_Conf_0.429	449	20	4.44E-26	NP_491633.1	73	120.939	101	74	Kinesin-Like Protein family member (klp-15)
Locus_12216_Transcript_2/3_Conf_0.429	847	20	7.21E-63	NP_491633.1	63	244.973	274	173	Kinesin-Like Protein family member (klp-15)

Locus_12216_Transcript_3/3_Conf_0.286	416	20	2.48E-21	NP_491633.1	72	105.145	90	65	Kinesin-Like Protein family member (klp-15)
Locus_12217_Transcript_1/1_Conf_1.000	444	12	5.64E-21	NP_871630.1	69	103.99	123	85	Nuclear Hormone Receptor family member (nhr-80)
Locus_12218_Transcript_1/1_Conf_1.000	348	20	1.29E-41	NP_055217.2	100	172.555	82	82	cytochrome b-c1 complex subunit 8
Locus_12219_Transcript_1/1_Conf_1.000	274	2	1.73E-22	NP_498411.1	81	108.997	91	74	related to yeast Vacuolar Protein Sorting factor family member (vps-16)
Locus_1222_Transcript_1/1_Conf_1.000	1102	20	1.66E-167	XP_002647836.1	91	593.193	350	319	Hypothetical protein CBG23625
Locus_12220_Transcript_1/1_Conf_1.000	139	0							
Locus_12221_Transcript_1/1_Conf_1.000	643	20	4.70E-75	NP_493334.1	87	284.648	213	187	hypothetical protein M01E5.2
Locus_12222_Transcript_1/1_Conf_1.000	553	0							
Locus_12223_Transcript_1/1_Conf_1.000	549	20	5.34E-83	BAG61260.1	100	310.457	181	181	unnamed protein product
Locus_12224_Transcript_1/1_Conf_1.000	418	20	3.11E-27	XP_002637376.1	69	124.79	135	94	Hypothetical protein CBG19078

Locus_12225_Transcript_1/1_Conf_1.000	1107	20	1.02E-23	XP_001638909.1	49	115.546	236	117	predicted protein
Locus_12226_Transcript_1/1_Conf_1.000	766	20	3.96E-91	XP_002630470.1	77	338.576	259	201	Hypothetical protein CBG11204
Locus_12227_Transcript_1/1_Conf_1.000	147	0							
Locus_12228_Transcript_1/1_Conf_1.000	508	20	5.33E-62	NP_505267.1	80	240.35	168	136	Regulator of Presynaptic Morphology family member (rpm-1)
Locus_12229_Transcript_1/1_Conf_1.000	844	0							
Locus_1223_Transcript_1/1_Conf_1.000	286	18	3.12E-27	NP_499455.1	79	124.79	94	75	Tumorous Enhancer of Glp-1(gf) family member (teg-1)
Locus_12230_Transcript_1/1_Conf_1.000	147	0							
Locus_12231_Transcript_1/1_Conf_1.000	151	0							
Locus_12232_Transcript_1/1_Conf_1.000	405	0							
Locus_12233_Transcript_1/1_Conf_1.000	229	2	2.08E-15	BAE00363.1	97	85.5001	40	39	unnamed protein product
Locus_12234_Transcript_1/1_Conf_1.000	292	20	3.19E-40	XP_002642536.1	86	167.933	95	82	C. briggsae CBR-LRS-1 protein
Locus_12235_Transcript_1/1_Conf_1.000	958	20	4.24E-65	XP_001895121.1	61	252.677	309	191	hypothetical protein
Locus_12236_Transcript_1/1_Conf_1.000	1034	20	4.23E-178	AAB99830.2	93	628.246	332	309	thrombospondin
Locus_12237_Transcript_1/1_Conf_1.000	139	0							
Locus_12238_Transcript_1/1_Conf_1.000	195	20	6.90E-19	XP_001899845.1	88	97.0561	51	45	CG7224
Locus_12239_Transcript_1/1_Conf_1.000	168	2	7.08E-08	CAA21622.4	73	60.4622	56	41	C. elegans protein Y43F8C.12, partially confirmed by transcript evidence
Locus_1224_Transcript_1/1_Conf_1.000	2034	20	4.79E-29	AAO63577.1	47	134.42	250	118	secreted protein 5 precursor
Locus_12240_Transcript_1/1_Conf_1.000	208	0							

Locus_12241_Transcript_1/1_Conf_1.000	937	20	7.28E-62	NP_500367.1	61	241.891	318	196	hypothetical protein M57.2
Locus_12242_Transcript_1/1_Conf_1.000	689	20	1.08E-22	XP_002645890.1	51	110.923	179	93	Hypothetical protein CBG07640
Locus_12243_Transcript_1/1_Conf_1.000	389	4	7.76E-15	XP_002631400.1	76	83.5741	64	49	Hypothetical protein CBG03250
Locus_12244_Transcript_1/1_Conf_1.000	226	0							
Locus_12245_Transcript_1/1_Conf_1.000	325	0							
Locus_12246_Transcript_1/1_Conf_1.000	194	0							
Locus_12247_Transcript_1/1_Conf_1.000	240	0							
Locus_12248_Transcript_1/1_Conf_1.000	608	0							
Locus_12249_Transcript_1/1_Conf_1.000	260	8	1.23E-07	ABB53347.1	51	59.6918	81	42	secreted protein 5 precursor
Locus_1225_Transcript_1/3_Conf_0.750	707	15	4.82E-25	ACU24256.1	100	88.1965	41	41	unknown
Locus_1225_Transcript_2/3_Conf_0.750	772	19	1.93E-21	XP_002118239.1	81	68.5514	86	70	predicted protein
Locus_1225_Transcript_3/3_Conf_0.750	708	19	1.63E-21	XP_002118239.1	81	68.5514	86	70	predicted protein
Locus_12250_Transcript_1/1_Conf_1.000	992	20	4.85E-35	EFO19997.1	54	152.91	339	184	hypothetical protein LOAG_08495
Locus_12251_Transcript_1/1_Conf_1.000	743	0							
Locus_12252_Transcript_1/1_Conf_1.000	292	0							
Locus_12253_Transcript_1/1_Conf_1.000	168	2	1.48E-05	NP_509318.1	62	52.7582	54	34	ADAM (disintegrin plus metalloprotease) family member (adm-4)
Locus_12254_Transcript_1/1_Conf_1.000	253	20	2.24E-17	XP_001896328.1	94	92.0485	85	80	calcium ATPase
Locus_12255_Transcript_1/1_Conf_1.000	420	0							
Locus_12256_Transcript_1/1_Conf_1.000	321	0							
Locus_12257_Transcript_1/1_Conf_1.000	659	20	4.01E-24	CBI63237.1	57	115.546	203	117	C. elegans protein Y111B2A.4a, partially confirmed by transcript evidence
Locus_12258_Transcript_1/1_Conf_1.000	936	20	2.19E-58	EFO25095.1	65	230.335	252	165	hypothetical protein LOAG_03392
Locus_12259_Transcript_1/1_Conf_1.000	195	0							

Locus_1226_Transcript_1/3_Conf_0.667	2576	20	1.23E-157	XP_002640115.1	67	561.992	544	365	C. briggsae CBR-GLD-2 protein
Locus_1226_Transcript_2/3_Conf_0.667	2278	20	1.82E-149	XP_002640115.1	74	534.643	422	315	C. briggsae CBR-GLD-2 protein
Locus_1226_Transcript_3/3_Conf_0.667	2654	20	2.18E-149	XP_002640115.1	74	534.643	422	315	C. briggsae CBR-GLD-2 protein
Locus_12260_Transcript_1/1_Conf_1.000	130	0							
Locus_12261_Transcript_1/1_Conf_1.000	563	20	3.18E-56	ACI48999.1	85	173.326	113	97	hypothetical protein Cbre_JD01.006
Locus_12262_Transcript_1/1_Conf_1.000	559	20	4.53E-69	NP_495779.1	84	264.233	184	155	abnormal Eversion of VuLva family member (evl-20)
Locus_12263_Transcript_1/1_Conf_1.000	612	20	1.72E-52	CBL43463.1	73	209.534	165	122	C. elegans protein Y52B11C.1, partially confirmed by transcript evidence

Locus_12264_Transcript_1/1_Conf_1.000	212	20	3.60E-20	NP_497809.1	87	101.293	70	61	Heavy chain, Unconventional Myosin family member (hum-5)
Locus_12265_Transcript_1/1_Conf_1.000	214	0							
Locus_12266_Transcript_1/1_Conf_1.000	237	0							
Locus_12267_Transcript_1/1_Conf_1.000	568	20	1.35E-47	XP_002633481.1	93	192.971	103	96	C. briggsae CBR-PHY-2 protein
Locus_12268_Transcript_1/1_Conf_1.000	205	0							
Locus_12269_Transcript_1/1_Conf_1.000	386	20	2.33E-22	XP_001269594.1	75	72.0182	54	41	hypothetical protein ACLA_028940
Locus_1227_Transcript_1/1_Conf_1.000	326	0							
Locus_12270_Transcript_1/1_Conf_1.000	151	4	1.36E-06	NP_499647.1	95	56.225	46	44	TAF (TBP-associated transcription factor) family member (taf-7.2)
Locus_12271_Transcript_1/1_Conf_1.000	161	20	5.46E-16	NP_492067.1	88	87.4261	52	46	hypothetical protein F13G3.11
Locus_12272_Transcript_1/1_Conf_1.000	138	0							
Locus_12273_Transcript_1/1_Conf_1.000	437	20	7.64E-63	BAG59449.1	100	243.047	119	119	unnamed protein product
Locus_12274_Transcript_1/2_Conf_1.000	1580	20	0	XP_002647008.1	82	690.649	515	427	Hypothetical protein CBG23794
Locus_12274_Transcript_2/2_Conf_1.000	573	20	1.59E-59	NP_499267.1	81	232.646	186	151	hypothetical protein Y70G10A.3

Locus_12275_Transcript_1/1_Conf_1.000	954	20	6.19E-125	ACY39994.1	76	451.44	351	268	C. elegans FLN-2 protein, isoform d
Locus_12276_Transcript_1/1_Conf_1.000	235	20	4.92E-17	NP_506788.1	67	90.8929	77	52	CYtochrome P450 family member (cyp-34A2)
Locus_12277_Transcript_1/1_Conf_1.000	201	20	3.28E-29	XP_002819700.1	100	131.339	67	67	PREDICTED: stomatin-like protein 2-like isoform 2
Locus_12278_Transcript_1/1_Conf_1.000	159	20	7.90E-07	CAD99040.1	83	56.9954	37	31	putative scj1 protein
Locus_12279_Transcript_1/1_Conf_1.000	212	20	1.73E-22	EFO21588.1	82	108.997	70	58	coiled-coil domain-containing protein 101
Locus_1228_Transcript_1/1_Conf_1.000	1671	20	0	XP_002637091.1	89	723.391	475	424	Hypothetical protein CBG09590
Locus_12280_Transcript_1/1_Conf_1.000	214	0							
Locus_12281_Transcript_1/1_Conf_1.000	781	20	7.18E-19	NP_498090.1	48	98.5969	258	125	Temporarily Assigned Gene name family member (tag-262)
Locus_12282_Transcript_1/1_Conf_1.000	197	0							
Locus_12283_Transcript_1/1_Conf_1.000	293	0							
Locus_12284_Transcript_1/1_Conf_1.000	274	2	6.81E-19	XP_002636840.1	70	97.0561	91	64	Hypothetical protein CBG09291
Locus_12285_Transcript_1/1_Conf_1.000	1973	7	2.83E-95	NP_510336.2	53	354.369	617	330	TBC (Tre-2/Bub2/Cdc16) domain family member (tbc-19)
Locus_12286_Transcript_1/1_Conf_1.000	143	2	1.32E-06	XP_002632345.1	76	56.225	43	33	Hypothetical protein CBG00357
Locus_12287_Transcript_1/1_Conf_1.000	252	0							
Locus_12288_Transcript_1/1_Conf_1.000	171	0							
Locus_12289_Transcript_1/1_Conf_1.000	196	0							
Locus_1229_Transcript_1/1_Conf_1.000	2030	20	4.67E-101	XP_001894842.1	62	373.629	481	301	Dual specificity phosphatase, catalytic domain containing protein
Locus_12290_Transcript_1/1_Conf_1.000	188	4	2.17E-20	NP_505746.2	93	102.064	62	58	hypothetical protein F28H7.8
Locus_12291_Transcript_1/1_Conf_1.000	356	20	2.42E-40	NP_510442.2	93	168.318	90	84	hypothetical protein F40E10.6
Locus_12292_Transcript_1/1_Conf_1.000	530	0							
Locus_12293_Transcript_1/1_Conf_1.000	528	20	2.75E-30	XP_001897467.1	63	135.191	177	113	CAP-Gly domain containing protein
Locus_12294_Transcript_1/1_Conf_1.000	136	0							
Locus_12295_Transcript_1/1_Conf_1.000	143	0							

Locus_12296_Transcript_1/1_Conf_1.000	383	20	1.33E-30	NP_498702.1	83	135.961	123	103	Ribosomal Protein, Large subunit family member (rpl-35)
Locus_12297_Transcript_1/1_Conf_1.000	807	20	2.11E-101	NP_001023058.1	77	372.859	263	204	hypothetical protein C42C1.11
Locus_12298_Transcript_1/1_Conf_1.000	363	20	1.01E-30	NP_497603.3	87	136.346	85	74	hypothetical protein Y71H2AM.9
Locus_12299_Transcript_1/1_Conf_1.000	430	3	1.09E-16	XP_002639154.1	92	89.7373	55	51	Hypothetical protein CBG14981
Locus_123_Transcript_1/8_Conf_0.267	1728	20	8.41E-141	ADI54942.1	98	505.368	263	258	heat shock protein 70
Locus_123_Transcript_2/8_Conf_0.267	1728	20	8.41E-141	ADI54942.1	98	505.368	263	258	heat shock protein 70
Locus_123_Transcript_3/8_Conf_0.167	477	20	4.50E-47	BAI81971.1	100	190.66	99	99	heat shock protein 70
Locus_123_Transcript_4/8_Conf_0.167	519	20	6.08E-56	BAI81971.1	100	220.32	113	113	heat shock protein 70
Locus_123_Transcript_5/8_Conf_0.267	1739	20	0	XP_002632429.1	98	846.655	478	473	C. briggsae CBR-HSP-1 protein

Locus_123_Transcript_6/8_Conf_0.267	1739	20	0	XP_002632429.1	98	840.877	478	473	C. briggsae CBR-HSP-1 protein
Locus_123_Transcript_7/8_Conf_0.400	1830	20	0	NP_503068.1	98	1019.22	574	565	Heat Shock Protein family member (hsp-1)
Locus_123_Transcript_8/8_Conf_0.400	1872	20	0	NP_503068.1	98	1039.64	588	579	Heat Shock Protein family member (hsp-1)
Locus_1230_Transcript_1/4_Conf_0.300	1373	20	5.97E-67	XP_002632730.1	72	259.61	245	178	C. briggsae CBR-LST-3 protein
Locus_1230_Transcript_2/4_Conf_0.700	3319	20	9.72E-110	XP_001901214.1	58	403.29	787	462	SAP domain containing protein
Locus_1230_Transcript_3/4_Conf_0.700	3320	20	9.72E-110	XP_001901214.1	58	403.29	787	462	SAP domain containing protein
Locus_1230_Transcript_4/4_Conf_0.700	3320	20	9.72E-110	XP_001901214.1	58	403.29	787	462	SAP domain containing protein
Locus_12300_Transcript_1/1_Conf_1.000	340	20	1.55E-31	XP_002633760.1	63	139.043	117	74	C. briggsae CBR-GON-1 protein
Locus_12301_Transcript_1/1_Conf_1.000	231	0							
Locus_12302_Transcript_1/2_Conf_1.000	252	0							
Locus_12302_Transcript_2/2_Conf_1.000	387	0							
Locus_12303_Transcript_1/1_Conf_1.000	248	3	7.74E-15	XP_002639235.1	59	83.5741	82	49	Hypothetical protein CBG03793
Locus_12304_Transcript_1/1_Conf_1.000	763	20	1.32E-54	NP_001022234.1	69	217.238	237	164	hypothetical protein K05F1.6

Locus_12305_Transcript_1/1_Conf_1.000	552	2	1.32E-28	NP_505498.1	59	129.798	162	97	hypothetical protein ZK287.7
Locus_12306_Transcript_1/1_Conf_1.000	1228	20	8.71E-67	NP_001122711.1	52	258.84	405	213	CBP/p300 homolog family member (cbp-1)
Locus_12307_Transcript_1/2_Conf_1.000	668	20	6.91E-48	EFO24728.1	67	194.512	219	148	blistered cuticle protein 3
Locus_12307_Transcript_2/2_Conf_1.000	668	20	6.91E-48	EFO24728.1	67	194.512	219	148	blistered cuticle protein 3
Locus_12308_Transcript_1/1_Conf_1.000	309	0							
Locus_12309_Transcript_1/1_Conf_1.000	342	20	1.87E-56	AAZ82849.1	96	221.861	113	109	Cam kinase kinase protein 1, isoform b
Locus_1231_Transcript_1/1_Conf_1.000	712	20	1.62E-16	XP_001656054.1	48	90.5077	233	112	brain chitinase and chia
Locus_12310_Transcript_1/1_Conf_1.000	161	0							
Locus_12311_Transcript_1/1_Conf_1.000	585	0							
Locus_12312_Transcript_1/1_Conf_1.000	553	0							

Locus_12313_Transcript_1/2_Conf_1.000	1509	20	8.47E-110	NP_502354.2	73	402.134	334	244	yeast SEC homolog family member (sec-24.2)
Locus_12313_Transcript_2/2_Conf_1.000	1509	20	8.47E-110	NP_502354.2	73	402.134	334	244	yeast SEC homolog family member (sec-24.2)
Locus_12314_Transcript_1/1_Conf_1.000	243	2	1.16E-10	XP_002643753.1	76	69.707	64	49	C. briggsae CBR-PSSY-1 protein
Locus_12315_Transcript_1/1_Conf_1.000	235	4	5.44E-16	XP_002644415.1	66	87.4261	75	50	C. briggsae CBR-TSP-17 protein
Locus_12316_Transcript_1/1_Conf_1.000	309	0							
Locus_12317_Transcript_1/1_Conf_1.000	887	0							
Locus_12318_Transcript_1/1_Conf_1.000	507	0							
Locus_12319_Transcript_1/1_Conf_1.000	171	0							
Locus_1232_Transcript_1/1_Conf_1.000	520	20	3.83E-42	NP_510335.2	78	174.481	129	101	hypothetical protein C31E10.7
Locus_12320_Transcript_1/1_Conf_1.000	486	14	2.46E-08	XP_002648923.1	49	62.003	160	79	Hypothetical protein CBG17840
Locus_12321_Transcript_1/1_Conf_1.000	527	0							
Locus_12322_Transcript_1/1_Conf_1.000	612	20	5.54E-43	NP_499696.2	77	177.948	160	124	hypothetical protein ZK1010.3

Locus_12323_Transcript_1/1_Conf_1.000	685	20	4.40E-101	NP_001023295.1	95	371.318	196	187	UNCoordinated family member (unc-43)
Locus_12324_Transcript_1/1_Conf_1.000	241	5	4.90E-09	XP_001898554.1	84	64.3142	38	32	DOMON domain containing protein
Locus_12325_Transcript_1/1_Conf_1.000	264	20	1.64E-20	NP_001022870.1	79	102.449	87	69	hypothetical protein Y43F4A.1
Locus_12326_Transcript_1/1_Conf_1.000	592	20	2.17E-54	NP_504292.2	76	215.698	198	151	hypothetical protein C37H5.5
Locus_12327_Transcript_1/1_Conf_1.000	852	0							
Locus_12328_Transcript_1/1_Conf_1.000	354	4	2.35E-27	XP_002641855.1	72	125.176	106	77	Hypothetical protein CBG16529
Locus_12329_Transcript_1/1_Conf_1.000	181	0							
Locus_1233_Transcript_1/1_Conf_1.000	510	20	8.51E-44	ACI49119.1	85	179.874	115	98	hypothetical protein Cbre_JD14.007
Locus_12330_Transcript_1/1_Conf_1.000	683	20	8.43E-12	EFA08418.1	47	74.7146	223	106	hypothetical protein TcasGA2_TC006065
Locus_12331_Transcript_1/1_Conf_1.000	356	20	4.73E-28	XP_002074576.1	64	127.487	118	76	GK23146
Locus_12332_Transcript_1/1_Conf_1.000	232	1	1.81E-19	EFO17857.1	68	98.9821	77	53	CK1/TTBK protein kinase
Locus_12333_Transcript_1/1_Conf_1.000	814	4	1.72E-18	CAB54509.2	48	97.4413	215	104	C. elegans protein Y79H2A.3a, partially confirmed by transcript evidence
Locus_12334_Transcript_1/1_Conf_1.000	609	0							
Locus_12335_Transcript_1/1_Conf_1.000	194	4	4.20E-16	NP_497814.3	82	87.8113	56	46	UNCoordinated family member (unc-79)
Locus_12336_Transcript_1/1_Conf_1.000	419	3	1.27E-04	NP_001084750.1	60	49.6766	58	35	TSC22 domain family, member 2
Locus_12337_Transcript_1/1_Conf_1.000	342	20	6.46E-41	NP_491314.1	82	170.244	108	89	INneXin family member (inx-16)

Locus_12338_Transcript_1/1_Conf_1.000	165	0							
Locus_12339_Transcript_1/1_Conf_1.000	434	3	2.67E-23	XP_002647006.1	62	111.694	155	97	Hypothetical protein CBG22464
Locus_1234_Transcript_1/1_Conf_1.000	310	20	3.84E-14	XP_001847954.1	72	81.2629	74	54	shc transforming protein
Locus_12340_Transcript_1/1_Conf_1.000	248	0							
Locus_12341_Transcript_1/1_Conf_1.000	492	0							
Locus_12342_Transcript_1/1_Conf_1.000	646	15	9.65E-52	XP_002644812.1	66	207.223	219	146	Hypothetical protein CBG14830
Locus_12343_Transcript_1/1_Conf_1.000	210	20	7.76E-15	XP_002595182.1	68	83.5741	69	47	hypothetical protein BRAFLDRAFT_285565
Locus_12344_Transcript_1/1_Conf_1.000	165	0							
Locus_12345_Transcript_1/1_Conf_1.000	1449	20	6.31E-123	XP_002640311.1	68	445.662	510	347	C. briggsae CBR-LET-363 protein
Locus_12346_Transcript_1/1_Conf_1.000	866	20	2.24E-91	XP_002644316.1	74	339.732	289	214	C. briggsae CBR-TAG-312 protein
Locus_12347_Transcript_1/1_Conf_1.000	334	0							
Locus_12348_Transcript_1/1_Conf_1.000	682	20	8.31E-60	NP_497539.3	71	234.187	198	142	F-box A protein family member (fbxa-1)
Locus_12349_Transcript_1/1_Conf_1.000	326	20	2.21E-25	NP_496059.2	81	118.627	91	74	UDP-GlucuronosylTransferase family member (ugt-58)
Locus_1235_Transcript_1/1_Conf_1.000	728	20	2.20E-40	XP_002639923.1	59	169.859	251	149	C. briggsae CBR-JPH-1 protein
Locus_12350_Transcript_1/1_Conf_1.000	436	0							
Locus_12351_Transcript_1/1_Conf_1.000	247	0							
Locus_12352_Transcript_1/1_Conf_1.000	382	20	9.47E-45	NP_510091.2	90	182.956	114	103	hypothetical protein T18D3.3
Locus_12353_Transcript_1/1_Conf_1.000	367	0							

Locus_12354_Transcript_1/1_Conf_1.000	496	20	6.32E-41	XP_002641474.1	69	170.244	162	112	C. briggsae CBR-PTP-1 protein
Locus_12355_Transcript_1/1_Conf_1.000	751	20	2.84E-102	EFO15747.1	95	375.555	205	196	RPB-5 protein
Locus_12356_Transcript_1/1_Conf_1.000	1087	14	2.21E-23	EFO22475.1	49	114.39	314	154	PH domain-containing protein
Locus_12357_Transcript_1/1_Conf_1.000	390	20	6.31E-25	XP_002639764.1	85	117.087	101	86	Hypothetical protein CBG02210
Locus_12358_Transcript_1/1_Conf_1.000	875	20	7.48E-26	EFO26243.1	50	122.094	287	146	ku P80 DNA helicase
Locus_12359_Transcript_1/1_Conf_1.000	165	0							
Locus_1236_Transcript_1/1_Conf_1.000	1606	20	9.23E-78	EFO24996.1	65	295.819	423	276	hypothetical protein LOAG_03490
Locus_12360_Transcript_1/1_Conf_1.000	316	3	2.10E-07	CBM41222.1	51	58.9214	86	44	C. elegans protein T10G3.3b, partially confirmed by transcript evidence
Locus_12361_Transcript_1/1_Conf_1.000	939	20	2.43E-65	NP_001040941.1	72	253.447	202	147	PhosphatidylSerine Receptor family member (psr-1)
Locus_12362_Transcript_1/1_Conf_1.000	235	0							
Locus_12363_Transcript_1/1_Conf_1.000	274	20	1.67E-25	XP_002643815.1	78	119.013	90	71	Hypothetical protein CBG02030
Locus_12364_Transcript_1/1_Conf_1.000	510	0							
Locus_12365_Transcript_1/1_Conf_1.000	459	20	1.60E-28	XP_002639084.1	62	129.028	151	95	Hypothetical protein CBG14902
Locus_12366_Transcript_1/1_Conf_1.000	133	0							
Locus_12367_Transcript_1/1_Conf_1.000	492	8	7.07E-16	XP_002629751.1	86	87.0409	50	43	Hypothetical protein CBG00985

Locus_12368_Transcript_1/1_Conf_1.000	441	20	7.61E-42	XP_001899686.1	73	173.326	145	106	Abnormal embryonic partitioning of cytoplasm protein 3, isoform b
Locus_12369_Transcript_1/2_Conf_1.000	1028	20	2.59E-111	XP_002634540.1	85	406.371	265	226	C. briggsae CBR-EGL-21 protein
Locus_12369_Transcript_2/2_Conf_1.000	1028	20	2.59E-111	XP_002634540.1	85	406.371	265	226	C. briggsae CBR-EGL-21 protein
Locus_1237_Transcript_1/2_Conf_1.000	1379	20	5.70E-17	XP_002639868.1	43	93.5893	181	79	C. briggsae CBR-DAO-5 protein
Locus_1237_Transcript_2/2_Conf_1.000	1567	20	4.36E-16	XP_002639868.1	52	90.8929	165	87	C. briggsae CBR-DAO-5 protein
Locus_12370_Transcript_1/1_Conf_1.000	224	0							
Locus_12371_Transcript_1/1_Conf_1.000	363	0							
Locus_12372_Transcript_1/1_Conf_1.000	906	20	1.42E-91	NP_741241.1	73	340.502	302	222	Dipeptidyl Peptidase Four (IV) family member (dpf-6)
Locus_12373_Transcript_1/1_Conf_1.000	171	0							
Locus_12374_Transcript_1/1_Conf_1.000	516	20	1.52E-51	XP_002647980.1	72	205.682	172	125	C. briggsae CBR-PUS-1 protein
Locus_12375_Transcript_1/1_Conf_1.000	445	20	6.85E-43	NP_510130.2	85	176.792	105	90	hypothetical protein F22E10.5
Locus_12376_Transcript_1/1_Conf_1.000	154	20	1.18E-18	XP_002920956.1	100	96.2857	46	46	PREDICTED: RNA-binding protein 42-like isoform 2
Locus_12377_Transcript_1/1_Conf_1.000	373	0							
Locus_12378_Transcript_1/1_Conf_1.000	594	0							
Locus_12379_Transcript_1/1_Conf_1.000	132	4	4.42E-10	NP_001021692.1	88	67.781	42	37	EATing: abnormal pharyngeal pumping family member (eat-18)
Locus_1238_Transcript_1/1_Conf_1.000	1257	20	7.26E-101	XP_002630425.1	76	372.089	303	231	Hypothetical protein CBG11150

Locus_12380_Transcript_1/1_Conf_1.000	374	20	1.21E-39	XP_002642385.1	83	166.007	126	105	Hypothetical protein CBG18389
Locus_12381_Transcript_1/1_Conf_1.000	589	0							
Locus_12382_Transcript_1/1_Conf_1.000	213	15	2.66E-15	NP_001022735.1	73	85.1149	71	52	hypothetical protein R155.1
Locus_12383_Transcript_1/1_Conf_1.000	650	0							
Locus_12384_Transcript_1/1_Conf_1.000	694	20	1.83E-09	XP_002642093.1	52	67.0106	174	91	C. briggsae CBR-SMC-4 protein
Locus_12385_Transcript_1/1_Conf_1.000	304	0							
Locus_12386_Transcript_1/1_Conf_1.000	625	20	1.56E-56	NP_001023008.1	72	223.016	197	143	CULlin family member (cul-2)
Locus_12387_Transcript_1/1_Conf_1.000	897	20	3.44E-74	NP_495578.2	68	282.722	260	177	hypothetical protein ZK1290.5
Locus_12388_Transcript_1/1_Conf_1.000	226	1	1.27E-12	NP_499274.3	74	76.2554	67	50	Connector/eNhancer of KSR family member (cnk-1)
Locus_12389_Transcript_1/1_Conf_1.000	474	14	7.46E-50	EFO27980.1	79	199.904	158	125	hypothetical protein LOAG_00505
Locus_1239_Transcript_1/1_Conf_1.000	159	0							
Locus_12390_Transcript_1/1_Conf_1.000	329	0							
Locus_12391_Transcript_1/1_Conf_1.000	363	20	6.35E-09	XP_002176123.1	67	63.929	55	37	chromo domain-containing protein

Locus_12392_Transcript_1/1_Conf_1.000	353	20	9.25E-32	NP_509229.1	75	139.813	114	86	SUppressor of activated let-60 Ras family member (sur-5)
Locus_12393_Transcript_1/1_Conf_1.000	138	16	2.11E-12	XP_002631560.1	86	75.485	46	40	Hypothetical protein CBG20736
Locus_12394_Transcript_1/1_Conf_1.000	177	20	3.70E-25	NP_501715.1	96	117.857	58	56	Acetylcholine-gated Chloride Channel family member (acc-1)
Locus_12395_Transcript_1/1_Conf_1.000	502	20	5.42E-64	XP_001154691.1	99	246.899	125	124	PREDICTED: hypothetical protein isoform 2
Locus_12396_Transcript_1/1_Conf_1.000	202	20	1.29E-25	XP_002818734.1	100	119.398	58	58	PREDICTED: chondroitin sulfate glucuronyltransferase-like isoform 2
Locus_12397_Transcript_1/1_Conf_1.000	376	20	8.89E-59	NP_492127.1	89	229.565	126	113	Low-density lipoprotein Receptor Related family member (lrp-1)
Locus_12398_Transcript_1/1_Conf_1.000	177	0							
Locus_12399_Transcript_1/1_Conf_1.000	229	0							
Locus_124_Transcript_1/1_Conf_1.000	237	0							
Locus_1240_Transcript_1/1_Conf_1.000	2279	20	1.82E-32	XP_001901591.1	67	145.976	159	107	WH1 domain containing protein
Locus_12400_Transcript_1/1_Conf_1.000	980	20	1.81E-34	NP_490916.4	47	150.984	381	182	GTPase Activating Protein family member (gap-3)
Locus_12401_Transcript_1/1_Conf_1.000	1105	20	2.38E-97	XP_001895783.1	73	360.147	342	252	BTB/POZ domain containing protein 9

Locus_12402_Transcript_1/1_Conf_1.000	453	20	2.11E-36	XP_002645055.1	67	155.221	152	102	Hypothetical protein CBG16715
Locus_12403_Transcript_1/1_Conf_1.000	224	0							
Locus_12404_Transcript_1/1_Conf_1.000	197	0							
Locus_12405_Transcript_1/1_Conf_1.000	352	20	3.63E-36	NP_491357.1	88	154.451	93	82	hypothetical protein ZK973.3
Locus_12406_Transcript_1/2_Conf_1.000	635	20	1.69E-29	XP_001902631.1	56	133.265	211	119	ARID/BRIGHT DNA binding domain containing protein
Locus_12406_Transcript_2/2_Conf_1.000	292	20	3.66E-20	EFO18078.1	83	101.293	59	49	arid/bright DNA binding domain-containing protein
Locus_12407_Transcript_1/1_Conf_1.000	1137	20	2.25E-98	XP_002643956.1	64	363.614	380	246	Hypothetical protein CBG17315
Locus_12408_Transcript_1/1_Conf_1.000	181	20	1.12E-24	XP_002646598.1	94	116.316	59	56	Hypothetical protein CBG20482
Locus_12409_Transcript_1/1_Conf_1.000	1260	20	1.50E-21	XP_001364696.1	52	108.612	266	140	PREDICTED: similar to SCY1-like 3 (S. cerevisiae)
Locus_1241_Transcript_1/1_Conf_1.000	1559	5	6.60E-57	NP_493282.1	74	226.483	179	134	hypothetical protein W05B5.1
Locus_12410_Transcript_1/1_Conf_1.000	306	0							
Locus_12411_Transcript_1/1_Conf_1.000	520	3	3.06E-07	NP_491907.1	52	58.5362	88	46	Protein Phosphatase Four Regulatory subunit family member (ppfr-2)
Locus_12412_Transcript_1/1_Conf_1.000	254	1	2.25E-09	EFO23711.1	55	65.4698	84	47	hypothetical protein LOAG_04772
Locus_12413_Transcript_1/1_Conf_1.000	374	0							
Locus_12414_Transcript_1/1_Conf_1.000	714	20	4.38E-70	XP_002645904.1	76	268.47	217	167	C. briggsae CBR-MRP-5 protein
Locus_12415_Transcript_1/1_Conf_1.000	164	0							
Locus_12416_Transcript_1/1_Conf_1.000	438	20	5.15E-27	NP_492346.2	70	124.02	116	82	hypothetical protein F39H2.3

Locus_12417_Transcript_1/1_Conf_1.000	585	20	4.04E-61	EFO19790.1	74	238.039	197	147	exostosin-1
Locus_12418_Transcript_1/1_Conf_1.000	686	5	6.46E-44	NP_001023038.1	74	181.415	156	116	EXCretory canal abnormal family member (exc-5)
Locus_12419_Transcript_1/1_Conf_1.000	222	2	4.38E-05	CAR63563.1	80	51.2174	35	28	hypothetical protein
Locus_1242_Transcript_1/1_Conf_1.000	2496	20	0	NP_491559.1	88	840.877	572	509	Ezrin/Radixin/Moesin family member (erm-1)
Locus_12420_Transcript_1/1_Conf_1.000	713	20	9.99E-67	NP_001041123.2	68	257.299	238	163	hypothetical protein F55A11.6
Locus_12421_Transcript_1/1_Conf_1.000	139	0							
Locus_12422_Transcript_1/1_Conf_1.000	851	20	2.50E-55	XP_002629711.1	72	219.935	220	159	C. briggsae CBR-RHY-1 protein
Locus_12423_Transcript_1/2_Conf_1.000	729	20	1.03E-53	XP_002636561.1	64	214.157	214	139	Hypothetical protein CBG23253
Locus_12423_Transcript_2/2_Conf_1.000	526	20	2.69E-30	XP_002636561.1	61	135.191	147	91	Hypothetical protein CBG23253
Locus_12424_Transcript_1/1_Conf_1.000	692	0							
Locus_12425_Transcript_1/1_Conf_1.000	235	0							

Locus_12426_Transcript_1/1_Conf_1.000	488	20	1.13E-53	NP_497733.1	87	212.616	131	115	Protein Tyrosine Phosphatase family member (ptp-1)
Locus_12427_Transcript_1/2_Conf_1.000	470	2	2.07E-07	XP_001892427.1	70	58.9214	55	39	hypothetical protein Bm1_04745
Locus_12427_Transcript_2/2_Conf_1.000	632	2	6.80E-07	EFO16594.1	72	58.151	51	37	hypothetical protein LOAG_11910
Locus_12428_Transcript_1/1_Conf_1.000	237	0							
Locus_12429_Transcript_1/1_Conf_1.000	1179	20	3.74E-120	ACI49192.1	74	436.032	393	292	hypothetical protein Csp3_JD03.001
Locus_1243_Transcript_1/1_Conf_1.000	353	20	9.92E-18	BAE87571.1	60	93.2041	110	66	unnamed protein product
Locus_12430_Transcript_1/1_Conf_1.000	224	5	3.22E-24	NP_001022641.1	82	114.775	75	62	LEThal family member (let-805)
Locus_12431_Transcript_1/1_Conf_1.000	291	0							
Locus_12432_Transcript_1/1_Conf_1.000	342	20	1.65E-36	EFO24987.1	84	155.606	108	91	hypothetical protein LOAG_03502
Locus_12433_Transcript_1/1_Conf_1.000	289	4	3.33E-13	XP_002638865.1	72	78.1814	65	47	Hypothetical protein CBG22082
Locus_12434_Transcript_1/1_Conf_1.000	263	0							
Locus_12435_Transcript_1/1_Conf_1.000	145	0							
Locus_12436_Transcript_1/1_Conf_1.000	380	19	5.42E-24	NP_494788.2	69	114.005	133	93	related to yeast Vacuolar Protein Sorting factor family member (vps-18)
Locus_12437_Transcript_1/1_Conf_1.000	476	0							
Locus_12438_Transcript_1/1_Conf_1.000	139	0							
Locus_12439_Transcript_1/1_Conf_1.000	716	20	7.45E-78	AAN11402.1	75	294.278	235	178	secreted-protein 1 precursor
Locus_1244_Transcript_1/1_Conf_1.000	2806	20	1.07E-53	NP_741023.2	47	216.853	768	361	Nuclear Pore complex Protein family member (npp-21)
Locus_12440_Transcript_1/1_Conf_1.000	485	0							
Locus_12441_Transcript_1/1_Conf_1.000	243	0							
Locus_12442_Transcript_1/1_Conf_1.000	467	4	4.96E-09	EFO27500.1	50	64.3142	144	72	hypothetical protein LOAG_00978

Locus_12443_Transcript_1/1_Conf_1.000	477	20	1.36E-43	NP_501913.1	76	179.104	151	115	hypothetical protein R11A8.5
Locus_12444_Transcript_1/1_Conf_1.000	1002	20	2.97E-72	XP_002634026.1	83	276.559	187	156	C. briggsae CBR-PCT-1 protein
Locus_12445_Transcript_1/1_Conf_1.000	308	2	2.18E-25	XP_002631591.1	82	118.627	92	76	C. briggsae CBR-DRR-1 protein
Locus_12446_Transcript_1/1_Conf_1.000	895	6	6.11E-31	NP_001122672.1	58	139.043	202	119	Regulator of G protein Signaling family member (rgs-5)
Locus_12447_Transcript_1/1_Conf_1.000	280	0							
Locus_12448_Transcript_1/1_Conf_1.000	1022	20	1.89E-122	NP_503577.1	81	443.351	320	261	hypothetical protein F22F7.1
Locus_12449_Transcript_1/1_Conf_1.000	497	16	2.84E-33	XP_002832705.1	96	144.821	89	86	PREDICTED: hypothetical protein LOC100439353, partial
Locus_1245_Transcript_1/3_Conf_0.818	3794	20	1.53E-90	NP_741537.1	53	339.732	653	348	human MLK (Mixed Lineage Kinase) homolog family member (mlk-1)
Locus_1245_Transcript_2/3_Conf_0.818	3818	20	1.54E-90	NP_741537.1	53	339.732	653	348	human MLK (Mixed Lineage Kinase) homolog family member (mlk-1)
Locus_1245_Transcript_3/3_Conf_0.455	640	0							
Locus_12450_Transcript_1/1_Conf_1.000	505	2	1.94E-08	XP_002643645.1	78	62.3882	51	40	Hypothetical protein CBG16393

Locus_12451_Transcript_1/1_Conf_1.000	280	0								
Locus_12452_Transcript_1/1_Conf_1.000	670	0								
Locus_12453_Transcript_1/3_Conf_0.714	1973	20	3.64E-26	XP_001900501.1	51	124.79	291	150	hypothetical protein Bm1_45195
Locus_12453_Transcript_2/3_Conf_0.714	1973	20	3.64E-26	XP_001900501.1	51	124.79	291	150	hypothetical protein Bm1_45195
Locus_12453_Transcript_3/3_Conf_0.714	1973	20	3.64E-26	XP_001900501.1	51	124.79	291	150	hypothetical protein Bm1_45195
Locus_12454_Transcript_1/2_Conf_1.000	526	0								
Locus_12454_Transcript_2/2_Conf_1.000	532	0								
Locus_12455_Transcript_1/1_Conf_1.000	729	4	1.08E-10	XP_001894822.1	51	71.2478	176	91	SNF2 family N-terminal domain containing protein
Locus_12456_Transcript_1/1_Conf_1.000	386	20	1.66E-49	XP_002643641.1	89	198.749	128	115	C. briggsae CBR-IFA-3 protein

Locus_12457_Transcript_1/1_Conf_1.000	380	0							
Locus_12458_Transcript_1/1_Conf_1.000	132	0							
Locus_12459_Transcript_1/1_Conf_1.000	145	0							
Locus_1246_Transcript_1/2_Conf_1.000	920	1	1.54E-08	XP_002643611.1	48	64.6994	170	82	C. briggsae CBR-AIN-1 protein
Locus_1246_Transcript_2/2_Conf_1.000	842	1	2.25E-08	XP_002643611.1	44	63.929	165	74	C. briggsae CBR-AIN-1 protein
Locus_12460_Transcript_1/1_Conf_1.000	262	19	3.43E-10	XP_002633069.1	87	68.1662	40	35	C. briggsae CBR-TIN-9.1 protein
Locus_12461_Transcript_1/1_Conf_1.000	355	0							
Locus_12462_Transcript_1/1_Conf_1.000	665	20	1.47E-58	NP_492769.1	69	229.95	213	149	RTEL (mammalian Regulator of TELomere length) homolog family member (rtel-1)
Locus_12463_Transcript_1/1_Conf_1.000	274	0							
Locus_12464_Transcript_1/1_Conf_1.000	130	20	1.93E-05	XP_002576412.1	76	52.373	43	33	tyrosine kinase
Locus_12465_Transcript_1/1_Conf_1.000	621	0							
Locus_12466_Transcript_1/1_Conf_1.000	134	0							
Locus_12467_Transcript_1/1_Conf_1.000	279	0							
Locus_12468_Transcript_1/3_Conf_0.714	1279	20	2.22E-129	NP_499995.2	83	466.848	369	309	hypothetical protein Y55F3BL.2
Locus_12468_Transcript_2/3_Conf_0.714	1279	20	2.22E-129	NP_499995.2	83	466.848	369	309	hypothetical protein Y55F3BL.2
Locus_12468_Transcript_3/3_Conf_0.714	1279	20	2.22E-129	NP_499995.2	83	466.848	369	309	hypothetical protein Y55F3BL.2

Locus_12469_Transcript_1/1_Conf_1.000	447	0							
Locus_1247_Transcript_1/2_Conf_1.000	429	20	3.58E-60	ABJ97284.1	99	234.187	110	109	major sperm protein
Locus_1247_Transcript_2/2_Conf_1.000	534	20	4.57E-57	BAI81973.1	100	224.172	104	104	major sperm protein 1
Locus_12470_Transcript_1/1_Conf_1.000	390	20	4.20E-45	XP_002636522.1	86	184.111	115	99	Hypothetical protein CBG23203
Locus_12471_Transcript_1/1_Conf_1.000	405	20	8.61E-30	XP_002634543.1	70	133.265	134	94	C. briggsae CBR-CED-5 protein
Locus_12472_Transcript_1/1_Conf_1.000	479	19	1.01E-22	XP_002640716.1	59	109.768	152	90	Hypothetical protein CBG19783
Locus_12473_Transcript_1/1_Conf_1.000	875	20	8.77E-51	XP_001902022.1	64	204.912	293	188	hypothetical protein Bm1_52770
Locus_12474_Transcript_1/1_Conf_1.000	131	20	1.57E-15	XP_002742370.1	95	85.8853	43	41	PREDICTED: urocanase domain containing 1-like
Locus_12475_Transcript_1/1_Conf_1.000	540	20	2.80E-41	XP_001896506.1	68	171.785	179	123	Cullin family protein
Locus_12476_Transcript_1/1_Conf_1.000	212	20	5.55E-21	XP_002927000.1	100	103.99	49	49	PREDICTED: LOW QUALITY PROTEIN: lamin-A/C-like
Locus_12477_Transcript_1/1_Conf_1.000	152	0							
Locus_12478_Transcript_1/1_Conf_1.000	375	0							
Locus_12479_Transcript_1/1_Conf_1.000	1504	20	8.66E-14	BAA36710.1	42	83.1889	375	161	DEAD-Box Protein

Locus_1248_Transcript_1/1_Conf_1.000	465	5	1.34E-22	NP_497291.1	66	109.383	100	66	hypothetical protein F23H11.5
Locus_12480_Transcript_1/1_Conf_1.000	859	0							
Locus_12481_Transcript_1/1_Conf_1.000	877	20	1.91E-106	XP_002640662.1	85	389.808	275	235	Hypothetical protein CBG19722
Locus_12482_Transcript_1/1_Conf_1.000	179	0							
Locus_12483_Transcript_1/1_Conf_1.000	422	0							
Locus_12484_Transcript_1/1_Conf_1.000	1238	0							
Locus_12485_Transcript_1/1_Conf_1.000	595	5	8.44E-22	XP_002648817.1	60	107.457	123	74	Hypothetical protein CBG15628
Locus_12486_Transcript_1/1_Conf_1.000	1033	10	1.31E-86	NP_505475.2	88	324.324	218	192	hypothetical protein K09G1.1
Locus_12487_Transcript_1/1_Conf_1.000	325	20	3.89E-22	XP_001902301.1	84	107.842	73	62	ubiquitin-like protein
Locus_12488_Transcript_1/1_Conf_1.000	577	20	2.09E-07	XP_001122201.1	79	53.9138	43	34	PREDICTED: similar to Lysine-specific histone demethylase 1 (Amine oxidase flavin-containing domain protein 2) (BRAF35-HDAC complex protein BHC110)
Locus_12489_Transcript_1/1_Conf_1.000	453	0							
Locus_1249_Transcript_1/4_Conf_0.667	1470	20	0	NP_001021985.1	93	720.694	409	384	HELicase family member (hel-1)

Locus_1249_Transcript_2/4_Conf_0.222	961	20	1.50E-110	NP_001021986.1	91	403.675	230	210	HELicase family member (hel-1)
Locus_1249_Transcript_3/4_Conf_0.667	1470	20	0	NP_001021985.1	93	720.694	409	384	HELicase family member (hel-1)

Locus_1249_Transcript_4/4_Conf_0.667	1470	20	0	NP_001021985.1	93	720.694	409	384	HELicase family member (hel-1)
Locus_12490_Transcript_1/3_Conf_0.429	1555	20	1.44E-11	EAX02636.1	44	75.8702	310	137	glycosyltransferase 28 domain containing 1, isoform CRA_c
Locus_12490_Transcript_2/3_Conf_0.286	1364	20	1.21E-11	BAH13833.1	44	75.8702	310	137	unnamed protein product
Locus_12490_Transcript_3/3_Conf_0.571	274	0							
Locus_12491_Transcript_1/1_Conf_1.000	391	20	1.45E-37	BAA08309.1	80	159.073	120	96	ryanodine receptor
Locus_12492_Transcript_1/1_Conf_1.000	606	0							
Locus_12493_Transcript_1/1_Conf_1.000	278	5	8.25E-33	XP_002637129.1	85	143.28	91	78	Hypothetical protein CBG09631
Locus_12494_Transcript_1/1_Conf_1.000	352	20	1.48E-21	XP_001898517.1	63	105.916	117	74	DRIM protein

Locus_12495_Transcript_1/1_Conf_1.000	349	17	1.18E-10	NP_493130.2	51	69.707	133	68	hypothetical protein T09E11.6
Locus_12496_Transcript_1/2_Conf_1.000	237	20	2.51E-21	EFO19548.1	75	105.145	78	59	ubiquinone biosynthesis O-methyltransferase
Locus_12496_Transcript_2/2_Conf_1.000	243	20	8.02E-20	EFO19548.1	73	100.138	80	59	ubiquinone biosynthesis O-methyltransferase
Locus_12497_Transcript_1/1_Conf_1.000	706	15	3.19E-25	NP_501506.1	65	119.398	135	88	hypothetical protein F21D5.6
Locus_12498_Transcript_1/1_Conf_1.000	301	20	3.17E-24	NP_502574.1	70	114.775	99	70	MAternally affected Uncoordination family member (mau-8)
Locus_12499_Transcript_1/1_Conf_1.000	445	0							
Locus_125_Transcript_1/2_Conf_1.000	970	20	4.14E-108	AAC48257.3	90	395.586	252	228	Pcna (proliferating cell nuclear antigen) homolog protein 1, confirmed by transcript evidence
Locus_125_Transcript_2/2_Conf_1.000	970	20	4.14E-108	AAC48257.3	90	395.586	252	228	Pcna (proliferating cell nuclear antigen) homolog protein 1, confirmed by transcript evidence
Locus_1250_Transcript_1/1_Conf_1.000	1214	2	4.04E-08	XP_002640249.1	68	63.929	61	42	C. briggsae CBR-PQN-20 protein
Locus_12500_Transcript_1/1_Conf_1.000	399	0							
Locus_12501_Transcript_1/1_Conf_1.000	130	0							
Locus_12502_Transcript_1/1_Conf_1.000	170	20	1.68E-25	ACJ65168.1	98	119.013	56	55	Uncoordinated protein 68, isoform b, partially confirmed by transcript evidence
Locus_12503_Transcript_1/1_Conf_1.000	237	1	2.70E-07	NP_491118.2	52	58.5362	78	41	hypothetical protein Y54E10A.11

Locus_12504_Transcript_1/2_Conf_1.000	810	20	2.59E-67	NP_491541.1	78	259.61	187	146	hypothetical protein F56A3.2
Locus_12504_Transcript_2/2_Conf_1.000	781	20	2.43E-67	NP_491541.1	78	259.61	187	146	hypothetical protein F56A3.2
Locus_12505_Transcript_1/1_Conf_1.000	344	0							
Locus_12506_Transcript_1/1_Conf_1.000	175	0							
Locus_12507_Transcript_1/1_Conf_1.000	280	8	3.97E-11	XP_001894571.1	70	71.2478	62	44	UBX domain containing protein
Locus_12508_Transcript_1/1_Conf_1.000	931	20	8.44E-87	NP_497567.1	88	324.709	234	206	hypothetical protein Y54F10AL.1
Locus_12509_Transcript_1/1_Conf_1.000	232	0							
Locus_1251_Transcript_1/1_Conf_1.000	499	0							
Locus_12510_Transcript_1/1_Conf_1.000	564	0							
Locus_12511_Transcript_1/1_Conf_1.000	932	20	1.96E-59	NP_491606.2	60	233.802	316	192	hypothetical protein C09D4.4
Locus_12512_Transcript_1/1_Conf_1.000	201	20	1.25E-20	NP_499993.1	89	102.834	66	59	Enoyl-CoA Hydratase family member (ech-5)
Locus_12513_Transcript_1/1_Conf_1.000	1304	20	2.20E-140	NP_001040974.1	80	503.442	435	348	hypothetical protein K09B11.9
Locus_12514_Transcript_1/1_Conf_1.000	391	20	6.33E-09	AAA97925.1	79	63.929	39	31	hum-1
Locus_12515_Transcript_1/1_Conf_1.000	1130	20	2.58E-46	XP_001898245.1	57	190.66	379	217	Protein kinase domain containing protein
Locus_12516_Transcript_1/1_Conf_1.000	182	0							
Locus_12517_Transcript_1/1_Conf_1.000	130	0							

Locus_12518_Transcript_1/1_Conf_1.000	390	20	2.30E-51	NP_001022641.1	86	204.912	130	112	LEThal family member (let-805)
Locus_12519_Transcript_1/1_Conf_1.000	193	0							
Locus_1252_Transcript_1/5_Conf_0.333	1413	1	4.36E-04	XP_002639136.1	53	50.8322	128	68	C. briggsae CBR-TAG-63 protein
Locus_1252_Transcript_2/5_Conf_0.222	385	0							
Locus_1252_Transcript_3/5_Conf_0.222	1257	0							
Locus_1252_Transcript_4/5_Conf_0.222	2044	20	0	XP_002638874.1	84	843.958	571	483	Hypothetical protein CBG22093
Locus_1252_Transcript_5/5_Conf_0.333	1566	20	2.03E-05	XP_002813170.1	40	55.4546	230	92	PREDICTED: hypothetical protein LOC100442257
Locus_12520_Transcript_1/1_Conf_1.000	195	0							
Locus_12521_Transcript_1/1_Conf_1.000	332	8	1.86E-08	XP_002639634.1	62	62.3882	94	59	C. briggsae CBR-CLE-1 protein
Locus_12522_Transcript_1/1_Conf_1.000	210	4	3.86E-06	XP_001898470.1	55	54.6842	84	47	hypothetical protein Bm1_35105
Locus_12523_Transcript_1/1_Conf_1.000	662	2	5.74E-31	NP_505701.2	62	138.272	225	140	hypothetical protein F25D1.2
Locus_12524_Transcript_1/2_Conf_1.000	442	1	1.50E-05	YP_003009133.1	44	52.7582	92	41	Collagen triple helix repeat protein
Locus_12524_Transcript_2/2_Conf_1.000	365	1	4.71E-04	YP_003009133.1	44	47.7506	86	38	Collagen triple helix repeat protein
Locus_12525_Transcript_1/1_Conf_1.000	171	0							
Locus_12526_Transcript_1/1_Conf_1.000	129	0							
Locus_12527_Transcript_1/1_Conf_1.000	282	4	3.59E-15	XP_001895046.1	72	84.7297	70	51	hypothetical protein
Locus_12528_Transcript_1/1_Conf_1.000	679	20	2.76E-31	NP_492764.2	91	139.428	82	75	UBiquitin Conjugating enzyme family member (ubc-25)
Locus_12529_Transcript_1/1_Conf_1.000	134	16	1.68E-09	XP_001898554.1	75	65.855	45	34	DOMON domain containing protein
Locus_1253_Transcript_1/2_Conf_1.000	487	0							
Locus_1253_Transcript_2/2_Conf_1.000	487	1	1.49E-05	XP_002738888.1	41	52.7582	128	53	PREDICTED: selectin, platelet-like
Locus_12530_Transcript_1/1_Conf_1.000	367	0							
Locus_12531_Transcript_1/1_Conf_1.000	280	2	3.15E-08	XP_002630287.1	86	61.6178	44	38	Hypothetical protein CBG00720
Locus_12532_Transcript_1/1_Conf_1.000	145	0							
Locus_12533_Transcript_1/1_Conf_1.000	843	0							

Locus_12534_Transcript_1/1_Conf_1.000	733	20	1.57E-126	XP_002647010.1	93	456.062	242	227	C. briggsae CBR-COR-1 protein
Locus_12535_Transcript_1/1_Conf_1.000	787	0							
Locus_12536_Transcript_1/1_Conf_1.000	1674	2	1.88E-04	XP_002505186.1	38	52.373	236	90	predicted protein
Locus_12537_Transcript_1/1_Conf_1.000	553	20	2.04E-05	XP_790476.1	54	52.7582	130	71	PREDICTED: hypothetical protein
Locus_12538_Transcript_1/1_Conf_1.000	338	20	2.57E-18	EFA09973.1	64	95.1301	110	71	hypothetical protein TcasGA2_TC012131
Locus_12539_Transcript_1/1_Conf_1.000	438	8	1.11E-13	CBA11608.1	57	79.7221	137	79	C. elegans protein F45H7.2d, partially confirmed by transcript evidence
Locus_1254_Transcript_1/4_Conf_0.667	797	0							
Locus_1254_Transcript_2/4_Conf_0.444	271	0							
Locus_1254_Transcript_3/4_Conf_0.667	848	0							
Locus_1254_Transcript_4/4_Conf_0.667	788	0							
Locus_12540_Transcript_1/1_Conf_1.000	429	5	3.89E-06	XP_002636697.1	47	54.6842	141	67	Hypothetical protein CBG23415
Locus_12541_Transcript_1/1_Conf_1.000	2523	14	2.06E-08	XP_002666563.1	37	66.2402	325	121	PREDICTED: wu:fo94f09
Locus_12542_Transcript_1/1_Conf_1.000	391	20	1.26E-41	XP_002639098.1	78	172.555	129	101	C. briggsae CBR-UNC-40 protein
Locus_12543_Transcript_1/1_Conf_1.000	746	20	2.82E-62	CBM41226.1	66	242.662	263	175	C. elegans protein T21C9.2b, confirmed by transcript evidence
Locus_12544_Transcript_1/1_Conf_1.000	574	4	1.37E-10	XP_001900000.1	58	70.0922	92	54	60S ribosomal protein L44
Locus_12545_Transcript_1/1_Conf_1.000	313	20	4.23E-16	XP_002641332.1	61	87.8113	99	61	Hypothetical protein CBG13180
Locus_12546_Transcript_1/1_Conf_1.000	438	3	8.55E-06	NP_001023785.1	45	53.5286	150	68	hypothetical protein F11A3.2

Locus_12547_Transcript_1/1_Conf_1.000	994	20	1.13E-116	NP_494571.1	82	424.091	328	269	AdaPtin, Delta chain (clathrin associated complex) family member (apd-3)
Locus_12548_Transcript_1/1_Conf_1.000	890	20	5.58E-69	XP_002630482.1	65	265.388	306	200	Hypothetical protein CBG11218
Locus_12549_Transcript_1/1_Conf_1.000	225	3	2.64E-10	P0C656.2	62	68.5514	72	45	Mediator of RNA polymerase II transcription subunit 12
Locus_1255_Transcript_1/1_Conf_1.000	612	20	3.71E-47	XP_002642127.1	78	191.815	142	112	C. briggsae CBR-RNP-4 protein
Locus_12550_Transcript_1/1_Conf_1.000	709	20	3.10E-44	NP_001021008.1	72	182.57	184	133	PolyBRoMo domain containing family member (pbrm-1)
Locus_12551_Transcript_1/1_Conf_1.000	294	1	6.90E-11	NP_001129840.1	69	70.4774	63	44	hypothetical protein R08D7.4
Locus_12552_Transcript_1/1_Conf_1.000	544	5	5.05E-30	XP_002630594.1	63	134.42	195	123	C. briggsae CBR-AKA-1 protein
Locus_12553_Transcript_1/1_Conf_1.000	237	0							

Locus_12554_Transcript_1/1_Conf_1.000	1477	20	8.09E-158	XP_002641701.1	81	561.607	438	356	C. briggsae CBR-TBG-1 protein
Locus_12555_Transcript_1/1_Conf_1.000	302	0							
Locus_12556_Transcript_1/1_Conf_1.000	282	20	3.81E-41	XP_002630326.1	96	171.014	93	90	Hypothetical protein CBG04253
Locus_12557_Transcript_1/1_Conf_1.000	380	0							
Locus_12558_Transcript_1/1_Conf_1.000	273	0							
Locus_12559_Transcript_1/1_Conf_1.000	235	3	1.30E-09	XP_002639005.1	60	66.2402	80	48	Hypothetical protein CBG22251
Locus_1256_Transcript_1/1_Conf_1.000	1268	20	2.92E-57	NP_503731.1	63	227.254	269	171	hypothetical protein Y45G12B.2
Locus_12560_Transcript_1/1_Conf_1.000	594	0							
Locus_12561_Transcript_1/1_Conf_1.000	531	20	1.49E-23	EFO15643.1	89	112.849	68	61	K+ channel tetramerisation domain-containing protein
Locus_12562_Transcript_1/1_Conf_1.000	312	0							
Locus_12563_Transcript_1/1_Conf_1.000	295	20	4.15E-40	XP_002834805.1	100	167.548	86	86	PREDICTED: cytochrome c oxidase subunit 6C-like
Locus_12564_Transcript_1/1_Conf_1.000	813	0							
Locus_12565_Transcript_1/1_Conf_1.000	268	0							
Locus_12566_Transcript_1/2_Conf_1.000	634	0							
Locus_12566_Transcript_2/2_Conf_1.000	619	0							
Locus_12567_Transcript_1/1_Conf_1.000	194	0							
Locus_12568_Transcript_1/1_Conf_1.000	181	0							
Locus_12569_Transcript_1/1_Conf_1.000	130	0							
Locus_1257_Transcript_1/1_Conf_1.000	240	0							
Locus_12570_Transcript_1/1_Conf_1.000	257	0							
Locus_12571_Transcript_1/1_Conf_1.000	682	20	1.06E-22	EFO19565.1	60	110.923	108	65	hypothetical protein LOAG_08928
Locus_12572_Transcript_1/1_Conf_1.000	624	20	1.32E-55	XP_002630212.1	89	219.935	129	115	Hypothetical protein CBG00622
Locus_12573_Transcript_1/1_Conf_1.000	276	0							
Locus_12574_Transcript_1/1_Conf_1.000	1074	20	3.50E-98	NP_494995.1	72	362.844	362	261	Guanylyl CYclase family member (gcy-12)

Locus_12575_Transcript_1/1_Conf_1.000	984	20	5.53E-07	XP_001581848.1	42	59.6918	339	143	hypothetical protein
Locus_12576_Transcript_1/1_Conf_1.000	164	0							
Locus_12577_Transcript_1/1_Conf_1.000	351	20	6.66E-14	EFO26156.1	65	80.4925	106	69	hypothetical protein LOAG_02329
Locus_12578_Transcript_1/1_Conf_1.000	450	0							
Locus_12579_Transcript_1/1_Conf_1.000	465	1	1.04E-43	XP_001164327.1	95	129.798	86	82	PREDICTED: prolyl 4-hydroxylase, beta subunit isoform 5
Locus_1258_Transcript_1/1_Conf_1.000	1433	20	6.74E-61	XP_001900112.1	58	239.58	355	208	UV excision repair protein Rad23 containing protein
Locus_12580_Transcript_1/2_Conf_1.000	1040	20	3.04E-51	EFO25759.1	60	206.838	279	168	hypothetical protein LOAG_02720
Locus_12580_Transcript_2/2_Conf_1.000	1040	20	3.04E-51	EFO25759.1	60	206.838	279	168	hypothetical protein LOAG_02720
Locus_12581_Transcript_1/1_Conf_1.000	358	5	2.43E-16	XP_002632410.1	56	88.5817	121	68	Hypothetical protein CBG00435
Locus_12582_Transcript_1/1_Conf_1.000	278	0							
Locus_12583_Transcript_1/1_Conf_1.000	915	20	1.77E-73	XP_001896686.1	68	280.411	310	211	hypothetical protein
Locus_12584_Transcript_1/1_Conf_1.000	526	0							
Locus_12585_Transcript_1/1_Conf_1.000	296	2	2.71E-07	XP_002639374.1	57	58.5362	76	44	Hypothetical protein CBG03958
Locus_12586_Transcript_1/1_Conf_1.000	336	20	6.99E-24	NP_496444.1	67	113.62	106	72	Alanyl tRNA Synthetase family member (ars-1)

Locus_12587_Transcript_1/1_Conf_1.000	949	20	1.21E-112	XP_002646059.1	92	410.609	239	221	C. briggsae CBR-PMR-1 protein
Locus_12588_Transcript_1/1_Conf_1.000	398	2	3.09E-35	NP_500395.2	76	151.369	131	100	hypothetical protein Y37E11AL.6
Locus_12589_Transcript_1/1_Conf_1.000	131	0							
Locus_1259_Transcript_1/4_Conf_0.400	276	14	1.12E-05	NP_001174125.1	40	53.1434	88	36	Os04g0654400
Locus_1259_Transcript_2/4_Conf_0.500	399	20	1.14E-05	BAJ05299.1	39	53.1434	138	54	oocyte maturation arresting factor
Locus_1259_Transcript_3/4_Conf_0.300	399	0							
Locus_1259_Transcript_4/4_Conf_0.600	522	6	4.23E-04	ZP_06212340.1	46	48.1358	83	39	UreE urease accessory domain protein
Locus_12590_Transcript_1/1_Conf_1.000	2848	20	0	XP_001900373.1	71	875.159	832	597	TPR Domain containing protein
Locus_12591_Transcript_1/1_Conf_1.000	353	20	4.45E-26	XP_002640398.1	60	120.939	120	73	Hypothetical protein CBG08444
Locus_12592_Transcript_1/1_Conf_1.000	223	20	1.27E-20	XP_002637343.1	78	102.834	70	55	Hypothetical protein CBG19039
Locus_12593_Transcript_1/1_Conf_1.000	429	0							
Locus_12594_Transcript_1/1_Conf_1.000	290	0							

Locus_12595_Transcript_1/1_Conf_1.000	283	0							
Locus_12596_Transcript_1/1_Conf_1.000	423	20	1.64E-28	NP_497201.1	75	129.028	133	101	hypothetical protein Y55B1BR.4
Locus_12597_Transcript_1/1_Conf_1.000	1170	20	1.71E-125	NP_491652.1	75	453.751	395	299	hypothetical protein F55F8.2
Locus_12598_Transcript_1/1_Conf_1.000	771	20	1.03E-78	XP_001900477.1	79	297.36	240	190	HMG box family protein
Locus_12599_Transcript_1/1_Conf_1.000	220	0							
Locus_126_Transcript_1/1_Conf_1.000	2058	20	6.05E-80	XP_001896711.1	65	303.523	317	209	Dcp2, box A domain containing protein
Locus_1260_Transcript_1/1_Conf_1.000	1398	20	1.13E-129	XP_002635578.1	86	468.003	301	260	C. briggsae CBR-TAG-253 protein
Locus_12600_Transcript_1/1_Conf_1.000	1892	20	6.35E-20	XP_002647615.1	43	103.99	301	132	C. briggsae CBR-MES-4 protein
Locus_12601_Transcript_1/1_Conf_1.000	725	20	3.35E-57	XP_002634017.1	71	225.713	225	161	C. briggsae CBR-SPH-1 protein
Locus_12602_Transcript_1/1_Conf_1.000	459	4	5.91E-15	NP_490674.3	53	83.9593	156	84	hypothetical protein Y48G1C.8
Locus_12603_Transcript_1/1_Conf_1.000	623	20	2.45E-62	XP_002639152.1	75	242.276	196	148	Hypothetical protein CBG14979
Locus_12604_Transcript_1/1_Conf_1.000	368	20	6.91E-48	XP_002641646.1	84	193.356	123	104	C. briggsae CBR-CBP-1 protein

Locus_12605_Transcript_1/1_Conf_1.000	231	20	1.80E-35	NP_510459.1	98	152.14	76	75	EPsiN (endocytic protein) homolog family member (epn-1)
Locus_12606_Transcript_1/1_Conf_1.000	516	0							
Locus_12607_Transcript_1/1_Conf_1.000	379	0							
Locus_12608_Transcript_1/1_Conf_1.000	261	0							
Locus_12609_Transcript_1/1_Conf_1.000	199	20	6.64E-22	XP_002927828.1	100	107.071	50	50	PREDICTED: 60S ribosomal protein L17-like
Locus_1261_Transcript_1/3_Conf_0.625	360	20	1.24E-12	AAD51334.1	72	76.2554	59	43	Kunitz type serine protease inhibitor
Locus_1261_Transcript_2/3_Conf_0.250	1504	20	2.78E-137	NP_506041.1	74	493.426	430	319	hypothetical protein C13C4.5
Locus_1261_Transcript_3/3_Conf_0.625	1417	20	2.65E-118	XP_001898133.1	77	430.254	354	274	Innexin family protein
Locus_12610_Transcript_1/1_Conf_1.000	481	0							
Locus_12611_Transcript_1/1_Conf_1.000	411	20	6.33E-25	XP_002636649.1	60	117.087	124	75	Hypothetical protein CBG23360
Locus_12612_Transcript_1/1_Conf_1.000	764	0							
Locus_12613_Transcript_1/1_Conf_1.000	652	20	1.09E-66	NP_490879.2	75	256.914	203	154	hypothetical protein Y71G12B.17
Locus_12614_Transcript_1/1_Conf_1.000	371	0							

Locus_12615_Transcript_1/1_Conf_1.000	179	20	5.72E-18	XP_002813487.1	100	93.9745	44	44	PREDICTED: protein SEC13 homolog isoform 4
Locus_12616_Transcript_1/1_Conf_1.000	474	20	1.08E-24	XP_002630102.1	69	116.316	155	107	Hypothetical protein CBG13485
Locus_12617_Transcript_1/2_Conf_1.000	566	20	3.28E-38	EFO27624.1	69	161.77	129	90	hypothetical protein LOAG_00857
Locus_12617_Transcript_2/2_Conf_1.000	795	20	7.18E-38	EFO27624.1	69	161.77	129	90	hypothetical protein LOAG_00857
Locus_12618_Transcript_1/2_Conf_1.000	546	0							
Locus_12618_Transcript_2/2_Conf_1.000	254	0							
Locus_12619_Transcript_1/1_Conf_1.000	421	0							
Locus_1262_Transcript_1/1_Conf_1.000	1527	20	2.45E-72	XP_002635160.1	64	277.715	391	252	Hypothetical protein CBG11393
Locus_12620_Transcript_1/1_Conf_1.000	311	0							
Locus_12621_Transcript_1/1_Conf_1.000	638	0							
Locus_12622_Transcript_1/1_Conf_1.000	303	0							
Locus_12623_Transcript_1/1_Conf_1.000	393	0							
Locus_12624_Transcript_1/1_Conf_1.000	209	2	1.13E-05	NP_495673.2	79	53.1434	69	55	hypothetical protein DH11.4
Locus_12625_Transcript_1/1_Conf_1.000	401	0							
Locus_12626_Transcript_1/2_Conf_0.500	1445	2	4.07E-05	XP_002647016.1	38	54.299	338	129	Hypothetical protein CBG24039
Locus_12626_Transcript_2/2_Conf_0.500	919	0							
Locus_12627_Transcript_1/1_Conf_1.000	458	20	9.39E-45	XP_001895623.1	83	182.956	120	100	DNA-directed RNA polymerase II 33 kDa polypeptide
Locus_12628_Transcript_1/1_Conf_1.000	494	20	1.09E-08	ZP_07656264.1	53	63.1586	136	73	chromosome segregation protein SMC

Locus_12629_Transcript_1/1_Conf_1.000	870	2	7.21E-13	XP_002639099.1	44	78.9518	295	132	C. briggsae CBR-NPP-7 protein
Locus_1263_Transcript_1/1_Conf_1.000	979	0							
Locus_12630_Transcript_1/1_Conf_1.000	615	0							
Locus_12631_Transcript_1/1_Conf_1.000	314	3	6.77E-06	XP_002642902.1	47	53.9138	117	55	Hypothetical protein CBG15176
Locus_12632_Transcript_1/1_Conf_1.000	944	20	2.87E-90	NP_493664.2	77	336.265	271	210	hypothetical protein K10B4.3
Locus_12633_Transcript_1/1_Conf_1.000	1558	20	5.25E-155	NP_495455.2	84	552.362	390	331	3-Ketoacyl-coA Thiolase family member (kat-1)
Locus_12634_Transcript_1/1_Conf_1.000	273	20	2.10E-44	XP_002749580.1	100	181.8	89	89	PREDICTED: 60S ribosomal protein L10a-like isoform 1
Locus_12635_Transcript_1/1_Conf_1.000	743	6	2.48E-18	EFO22616.1	43	96.6709	341	147	TK/ABL protein kinase
Locus_12636_Transcript_1/1_Conf_1.000	1284	20	1.85E-91	XP_001895766.1	68	340.887	389	265	double-strand break repair protein mre-11

Locus_12637_Transcript_1/1_Conf_1.000	912	20	9.17E-22	CAX65090.1	55	108.612	177	98	C. elegans protein Y56A3A.4b, confirmed by transcript evidence
Locus_12638_Transcript_1/3_Conf_0.600	546	20	1.35E-38	XP_002643489.1	83	162.925	106	88	C. briggsae CBR-APA-2 protein
Locus_12638_Transcript_2/3_Conf_0.600	551	20	1.38E-38	XP_002643489.1	83	162.925	106	88	C. briggsae CBR-APA-2 protein

Locus_12638_Transcript_3/3_Conf_0.600	551	20	1.38E-38	XP_002643489.1	83	162.925	106	88	C. briggsae CBR-APA-2 protein
Locus_12639_Transcript_1/1_Conf_1.000	813	0							
Locus_1264_Transcript_1/2_Conf_1.000	674	20	9.52E-53	XP_002631659.1	90	210.69	116	105	C. briggsae CBR-MEX-1 protein
Locus_1264_Transcript_2/2_Conf_1.000	680	20	9.73E-53	XP_002631659.1	90	210.69	116	105	C. briggsae CBR-MEX-1 protein
Locus_12640_Transcript_1/1_Conf_1.000	597	20	5.71E-42	NP_001024929.1	69	174.481	191	132	Choline Kinase C family member (ckc-1)
Locus_12641_Transcript_1/2_Conf_1.000	244	0							
Locus_12641_Transcript_2/2_Conf_1.000	244	0							
Locus_12642_Transcript_1/1_Conf_1.000	398	0							
Locus_12643_Transcript_1/1_Conf_1.000	1113	20	6.49E-127	NP_001122812.1	79	458.373	359	287	TBC (Tre-2/Bub2/Cdc16) domain family member (tbc-9)
Locus_12644_Transcript_1/1_Conf_1.000	227	0							
Locus_12645_Transcript_1/1_Conf_1.000	475	20	9.70E-66	EFO26601.1	83	252.677	156	131	CK1/WORM6 protein kinase
Locus_12646_Transcript_1/1_Conf_1.000	283	0							
Locus_12647_Transcript_1/1_Conf_1.000	402	0							
Locus_12648_Transcript_1/1_Conf_1.000	1086	2	2.71E-13	XP_001900007.1	42	80.8777	288	121	hypothetical protein Bm1_42720
Locus_12649_Transcript_1/1_Conf_1.000	186	0							
Locus_1265_Transcript_1/2_Conf_0.333	2359	20	3.74E-113	XP_001898107.1	65	414.075	464	303	PDZ-domain protein scribble
Locus_1265_Transcript_2/2_Conf_0.667	1272	20	5.40E-144	NP_491319.1	90	515.383	323	292	proteasome Regulatory Particle, Non-ATPase-like family member (rpn-8)

Locus_12650_Transcript_1/1_Conf_1.000	190	2	1.66E-04	XP_002410801.1	56	49.2914	60	34	factor for adipocyte differentiation, putative
Locus_12651_Transcript_1/1_Conf_1.000	344	20	2.23E-25	NP_499570.1	87	118.627	79	69	ARp2/3 compleX component family member (arx-3)
Locus_12652_Transcript_1/1_Conf_1.000	438	0							
Locus_12653_Transcript_1/1_Conf_1.000	154	2	3.34E-05	XP_002643365.1	64	51.6026	57	37	C. briggsae CBR-PQN-34 protein
Locus_12654_Transcript_1/2_Conf_1.000	1788	20	0	NP_495749.1	80	681.404	552	447	mammalian NucleoSTemin (stem cell marker) related family member (nst-1)
Locus_12654_Transcript_2/2_Conf_1.000	1788	20	0	NP_495749.1	81	681.404	552	448	mammalian NucleoSTemin (stem cell marker) related family member (nst-1)
Locus_12655_Transcript_1/1_Conf_1.000	366	20	3.01E-35	NP_001129924.1	90	151.369	121	110	SLOwpoke potassium channel family member (slo-2)
Locus_12656_Transcript_1/1_Conf_1.000	256	0							
Locus_12657_Transcript_1/1_Conf_1.000	265	0							
Locus_12658_Transcript_1/1_Conf_1.000	411	6	2.41E-16	XP_001901424.1	72	88.5817	127	92	tRNA modification GTPase TrmE family protein
Locus_12659_Transcript_1/1_Conf_1.000	510	0							
Locus_1266_Transcript_1/1_Conf_1.000	900	20	2.63E-106	NP_001022302.1	92	389.423	259	239	MethylMalonic Aciduria type A protein family member (mmaa-1)
Locus_12660_Transcript_1/1_Conf_1.000	247	3	1.46E-05	NP_491382.1	75	52.7582	48	36	hypothetical protein Y119C1B.5
Locus_12661_Transcript_1/1_Conf_1.000	507	0							
Locus_12662_Transcript_1/1_Conf_1.000	694	20	5.13E-28	XP_001891849.1	58	128.642	173	102	Retinoblastoma-associated protein A domain containing protein
Locus_12663_Transcript_1/1_Conf_1.000	889	0							
Locus_12664_Transcript_1/1_Conf_1.000	133	0							
Locus_12665_Transcript_1/1_Conf_1.000	1434	20	1.51E-177	NP_506191.1	81	627.091	433	351	hypothetical protein F18E2.1
Locus_12666_Transcript_1/1_Conf_1.000	235	0							
Locus_12667_Transcript_1/1_Conf_1.000	321	0							

Locus_12668_Transcript_1/1_Conf_1.000	375	0							
Locus_12669_Transcript_1/1_Conf_1.000	340	0							
Locus_1267_Transcript_1/2_Conf_1.000	2235	20	0	XP_002640959.1	94	882.478	528	497	C. briggsae CBR-HSP-60 protein
Locus_1267_Transcript_2/2_Conf_1.000	937	0							
Locus_12670_Transcript_1/1_Conf_1.000	1490	0							
Locus_12671_Transcript_1/3_Conf_0.600	849	20	3.13E-90	NP_741744.2	78	335.88	274	214	hypothetical protein C15C7.7
Locus_12671_Transcript_2/3_Conf_0.600	773	20	2.07E-87	NP_741744.2	79	326.25	250	199	hypothetical protein C15C7.7

Locus_12671_Transcript_3/3_Conf_0.600	849	20	5.70E-92	NP_741744.2	78	341.658	274	216	hypothetical protein C15C7.7
Locus_12672_Transcript_1/1_Conf_1.000	394	0							
Locus_12673_Transcript_1/1_Conf_1.000	233	18	7.37E-13	NP_741861.2	83	77.0258	53	44	Nuclear Hormone Receptor family member (nhr-14)
Locus_12674_Transcript_1/1_Conf_1.000	701	13	5.22E-36	ACV31115.1	81	155.221	126	103	Hypothetical protein H06I04.6b
Locus_12675_Transcript_1/1_Conf_1.000	379	0							
Locus_12676_Transcript_1/1_Conf_1.000	210	0							
Locus_12677_Transcript_1/1_Conf_1.000	295	0							
Locus_12678_Transcript_1/1_Conf_1.000	438	20	1.58E-68	XP_002639744.1	83	261.922	142	118	C. briggsae CBR-LAM-3 protein
Locus_12679_Transcript_1/1_Conf_1.000	1321	20	0	NP_497570.1	86	693.345	416	359	hypothetical protein Y54F10AM.8
Locus_1268_Transcript_1/1_Conf_1.000	164	19	4.45E-18	XP_001894499.1	94	94.3597	51	48	unc-45 protein
Locus_12680_Transcript_1/1_Conf_1.000	241	3	1.86E-08	NP_508175.1	94	62.3882	38	36	Suppressor/Enhancer of Lin-12 family member (sel-12)
Locus_12681_Transcript_1/2_Conf_1.000	534	20	5.60E-47	XP_002634543.1	74	190.66	169	126	C. briggsae CBR-CED-5 protein

Locus_12681_Transcript_2/2_Conf_1.000	534	20	5.60E-47	XP_002634543.1	74	190.66	169	126	C. briggsae CBR-CED-5 protein
Locus_12682_Transcript_1/1_Conf_1.000	386	4	6.16E-20	EFO26621.1	53	100.523	151	81	AIDA-1b
Locus_12683_Transcript_1/1_Conf_1.000	316	0							
Locus_12684_Transcript_1/1_Conf_1.000	137	12	4.27E-05	XP_002609440.1	60	51.2174	43	26	hypothetical protein BRAFLDRAFT_93487
Locus_12685_Transcript_1/1_Conf_1.000	413	20	5.19E-19	CAL53402.1	59	97.4413	108	64	putative SMC protein (ISS)
Locus_12686_Transcript_1/1_Conf_1.000	133	0							
Locus_12687_Transcript_1/1_Conf_1.000	207	0							
Locus_12688_Transcript_1/1_Conf_1.000	219	0							
Locus_12689_Transcript_1/1_Conf_1.000	175	0							
Locus_1269_Transcript_1/1_Conf_1.000	803	20	5.86E-96	XP_002633299.1	83	354.755	240	200	Hypothetical protein CBG06029
Locus_12690_Transcript_1/1_Conf_1.000	250	0							
Locus_12691_Transcript_1/1_Conf_1.000	457	3	4.87E-09	NP_498641.1	53	64.3142	89	48	hypothetical protein H14A12.3
Locus_12692_Transcript_1/3_Conf_0.714	953	20	2.99E-79	NP_502288.1	80	299.671	211	170	hypothetical protein K08E4.6
Locus_12692_Transcript_2/3_Conf_0.714	953	20	6.03E-80	NP_502288.1	81	301.982	211	171	hypothetical protein K08E4.6
Locus_12692_Transcript_3/3_Conf_0.714	953	20	6.03E-80	NP_502288.1	81	301.982	211	171	hypothetical protein K08E4.6
Locus_12693_Transcript_1/1_Conf_1.000	546	4	2.00E-66	NP_001033357.2	82	255.373	181	150	DAF-16/FOXO Controlled, germline Tumor affecting family member (dct-6)
Locus_12694_Transcript_1/1_Conf_1.000	464	0							
Locus_12695_Transcript_1/1_Conf_1.000	242	0							
Locus_12696_Transcript_1/3_Conf_0.333	224	0							

Locus_12696_Transcript_2/3_Conf_0.667	751	0							
Locus_12696_Transcript_3/3_Conf_0.500	566	0							
Locus_12697_Transcript_1/1_Conf_1.000	224	2	9.73E-13	XP_002632085.1	88	76.6406	42	37	Hypothetical protein CBG17053
Locus_12698_Transcript_1/1_Conf_1.000	264	0							
Locus_12699_Transcript_1/1_Conf_1.000	500	20	9.04E-56	XP_002632430.1	81	219.55	166	136	Hypothetical protein CBG00458
Locus_127_Transcript_1/4_Conf_0.250	497	20	2.80E-81	DAA05878.1	98	304.294	151	148	TPA_inf: eukaryotic translation elongation factor 1A
Locus_127_Transcript_2/4_Conf_0.250	539	20	3.54E-81	DAA05878.1	98	304.294	151	148	TPA_inf: eukaryotic translation elongation factor 1A
Locus_127_Transcript_3/4_Conf_0.450	1010	20	6.93E-178	ABX79382.1	97	627.476	323	315	elongation factor 1 alpha
Locus_127_Transcript_4/4_Conf_0.450	1010	20	6.93E-178	ABX79382.1	97	627.476	323	315	elongation factor 1 alpha
Locus_1270_Transcript_1/1_Conf_1.000	1122	20	5.19E-23	NP_001023207.1	62	113.235	176	110	MiniBrain Kinase (Drosophila) homolog family member (mbk-2)
Locus_12700_Transcript_1/1_Conf_1.000	396	0							

Locus_12701_Transcript_1/1_Conf_1.000	600	16	2.13E-36	ACI49203.1	92	155.992	92	85	hypothetical protein Csp3_JD04.002
Locus_12702_Transcript_1/1_Conf_1.000	148	20	7.72E-18	NP_508515.2	93	93.5893	48	45	AQuaPorin or aquaglyceroporin related family member (aqp-7)
Locus_12703_Transcript_1/1_Conf_1.000	408	3	2.35E-11	NP_498613.2	53	72.0182	114	61	hypothetical protein F56C9.8
Locus_12704_Transcript_1/1_Conf_1.000	778	20	7.04E-59	XP_002639543.1	75	231.491	244	185	Hypothetical protein CBG04172
Locus_12705_Transcript_1/1_Conf_1.000	389	0							
Locus_12706_Transcript_1/1_Conf_1.000	474	0							
Locus_12707_Transcript_1/1_Conf_1.000	285	0							
Locus_12708_Transcript_1/1_Conf_1.000	632	20	3.99E-07	ABA06434.1	48	58.9214	129	63	telomerase reverse transcriptase
Locus_12709_Transcript_1/1_Conf_1.000	379	20	1.39E-11	ACM79010.1	64	72.7886	67	43	Kunitz protein 8
Locus_1271_Transcript_1/1_Conf_1.000	645	20	6.41E-80	NP_001022987.1	89	300.827	183	164	related to yeast Vacuolar Protein Sorting factor family member (vps-29)

Locus_12710_Transcript_1/1_Conf_1.000	230	20	8.14E-28	AAY84711.2	86	126.716	74	64	dual oxidase
Locus_12711_Transcript_1/1_Conf_1.000	1057	20	2.33E-22	XP_001894604.1	50	110.923	240	121	exonuclease family protein
Locus_12712_Transcript_1/1_Conf_1.000	239	0							
Locus_12713_Transcript_1/1_Conf_1.000	232	2	2.45E-08	NP_494772.1	71	62.003	66	47	NOT-Like (yeast CCR4/NOT complex component) family member (ntl-2)
Locus_12714_Transcript_1/1_Conf_1.000	397	20	3.31E-29	EFO19311.1	63	131.339	128	81	hypothetical protein LOAG_09182
Locus_12715_Transcript_1/1_Conf_1.000	203	11	1.34E-06	ADI61824.1	59	56.225	67	40	endonuclease-reverse transcriptase
Locus_12716_Transcript_1/1_Conf_1.000	1234	20	1.23E-177	CAA06162.1	89	627.091	397	354	CB-CYP-4

Locus_12717_Transcript_1/1_Conf_1.000	397	20	2.09E-07	XP_002630114.1	97	58.9214	34	33	C. briggsae CBR-PYR-1 protein
Locus_12718_Transcript_1/1_Conf_1.000	428	20	6.79E-51	NP_504643.1	80	203.371	142	115	ACyLtransferase-like family member (acl-8)
Locus_12719_Transcript_1/1_Conf_1.000	158	0							
Locus_1272_Transcript_1/1_Conf_1.000	689	20	2.36E-102	NP_498785.1	93	375.555	205	192	yeast PRP (splicing factor) related family member (prp-8)
Locus_12720_Transcript_1/1_Conf_1.000	294	0							
Locus_12721_Transcript_1/1_Conf_1.000	205	0							
Locus_12722_Transcript_1/1_Conf_1.000	353	0							
Locus_12723_Transcript_1/1_Conf_1.000	277	0							
Locus_12724_Transcript_1/1_Conf_1.000	158	0							
Locus_12725_Transcript_1/1_Conf_1.000	471	20	6.82E-27	NP_505188.3	60	123.635	160	96	PeroXidasiN (Drosophila peroxidase) homolog family member (pxn-1)

Locus_12726_Transcript_1/1_Conf_1.000	591	20	2.82E-62	NP_001023646.1	80	241.891	196	158	AKT kinase family member (akt-1)
Locus_12727_Transcript_1/1_Conf_1.000	882	20	3.52E-124	BAG63399.1	100	448.743	222	222	unnamed protein product
Locus_12728_Transcript_1/1_Conf_1.000	945	20	2.81E-61	EFA79727.1	64	239.965	266	171	hypothetical protein PPL_07418
Locus_12729_Transcript_1/1_Conf_1.000	170	0							
Locus_1273_Transcript_1/1_Conf_1.000	692	20	8.85E-65	XP_002631500.1	81	250.751	200	162	Hypothetical protein CBG20664
Locus_12730_Transcript_1/1_Conf_1.000	380	1	2.07E-07	NP_499670.1	75	58.9214	45	34	hypothetical protein Y37D8A.4
Locus_12731_Transcript_1/1_Conf_1.000	364	20	4.23E-29	NP_507527.2	83	130.954	112	93	hypothetical protein Y59A8B.8
Locus_12732_Transcript_1/1_Conf_1.000	168	0							
Locus_12733_Transcript_1/1_Conf_1.000	917	1	3.19E-06	XP_001900252.1	40	56.9954	333	134	hypothetical protein Bm1_43950

Locus_12734_Transcript_1/1_Conf_1.000	608	20	7.02E-51	NP_506230.1	84	204.142	157	132	hypothetical protein F54F3.4
Locus_12735_Transcript_1/1_Conf_1.000	133	0							
Locus_12736_Transcript_1/1_Conf_1.000	380	0							
Locus_12737_Transcript_1/1_Conf_1.000	361	0							
Locus_12738_Transcript_1/1_Conf_1.000	272	20	7.50E-42	XP_002637342.1	97	173.326	90	88	C. briggsae CBR-UNC-70 protein
Locus_12739_Transcript_1/1_Conf_1.000	333	1	1.24E-12	EFO14895.1	85	76.2554	49	42	CMGC/CDK/CDK9 protein kinase
Locus_1274_Transcript_1/1_Conf_1.000	520	20	1.19E-35	XP_002634275.1	68	152.91	168	115	Hypothetical protein CBG01851
Locus_12740_Transcript_1/1_Conf_1.000	367	3	2.66E-07	NP_001021652.1	46	58.5362	118	55	FANCI (Fanconi anemia complex component I) homolog family member (fnci-1)
Locus_12741_Transcript_1/1_Conf_1.000	290	0							
Locus_12742_Transcript_1/2_Conf_1.000	808	20	3.94E-23	XP_002640836.1	56	112.849	230	131	Hypothetical protein CBG15724
Locus_12742_Transcript_2/2_Conf_1.000	808	20	3.94E-23	XP_002640836.1	56	112.849	230	131	Hypothetical protein CBG15724
Locus_12743_Transcript_1/1_Conf_1.000	671	20	3.45E-63	XP_002639870.1	69	245.358	223	155	C. briggsae CBR-IRS-2 protein
Locus_12744_Transcript_1/1_Conf_1.000	537	20	6.55E-35	XP_002633331.1	70	150.599	139	98	Hypothetical protein CBG06070
Locus_12745_Transcript_1/1_Conf_1.000	600	0							

Locus_12746_Transcript_1/1_Conf_1.000	419	20	1.09E-72	NP_001122711.1	94	275.789	136	129	CBP/p300 homolog family member (cbp-1)
Locus_12747_Transcript_1/1_Conf_1.000	446	4	3.65E-28	XP_002634339.1	72	127.872	134	97	C. briggsae CBR-RIC-3 protein
Locus_12748_Transcript_1/1_Conf_1.000	133	0							
Locus_12749_Transcript_1/1_Conf_1.000	257	0							
Locus_1275_Transcript_1/2_Conf_1.000	212	6	1.16E-10	NP_001023274.1	64	69.707	70	45	VITellogenin structural genes (yolk protein genes) family member (vit-6)
Locus_1275_Transcript_2/2_Conf_1.000	205	6	3.40E-10	NP_001023274.1	64	68.1662	68	44	VITellogenin structural genes (yolk protein genes) family member (vit-6)
Locus_12750_Transcript_1/1_Conf_1.000	178	0							
Locus_12751_Transcript_1/1_Conf_1.000	271	7	1.25E-12	CAD44516.1	65	76.2554	89	58	VAB-10B protein
Locus_12752_Transcript_1/1_Conf_1.000	924	20	3.21E-46	XP_002636564.1	80	189.889	149	120	Hypothetical protein CBG23256
Locus_12753_Transcript_1/1_Conf_1.000	237	0							
Locus_12754_Transcript_1/1_Conf_1.000	601	20	1.06E-35	NP_496466.1	79	153.68	116	92	hypothetical protein F54D5.11
Locus_12755_Transcript_1/1_Conf_1.000	264	20	2.29E-14	NP_502268.1	64	82.0333	82	53	TRNA Guanine Transglycosylase family member (tgt-1)
Locus_12756_Transcript_1/2_Conf_1.000	695	4	3.31E-35	XP_002639709.1	62	152.525	202	127	Hypothetical protein CBG12430
Locus_12756_Transcript_2/2_Conf_1.000	695	4	2.54E-35	XP_002639709.1	63	152.91	202	128	Hypothetical protein CBG12430

Locus_12757_Transcript_1/1_Conf_1.000	219	3	3.70E-20	CAR63585.1	91	101.293	72	66	putative MAD-Like family member
Locus_12758_Transcript_1/1_Conf_1.000	433	20	1.50E-42	NP_499178.1	87	175.637	132	115	ARF-Like family member (arl-5)
Locus_12759_Transcript_1/1_Conf_1.000	417	20	1.54E-42	XP_001896370.1	80	175.637	138	111	hypothetical protein
Locus_1276_Transcript_1/1_Conf_1.000	221	4	2.74E-15	XP_002631558.1	75	85.1149	73	55	Hypothetical protein CBG20734
Locus_12760_Transcript_1/1_Conf_1.000	271	1	1.68E-17	EAW48404.1	100	92.4337	42	42	hCG2030753
Locus_12761_Transcript_1/1_Conf_1.000	304	20	1.42E-16	EFO22213.1	72	89.3521	101	73	arginine-tRNA-protein transferase
Locus_12762_Transcript_1/1_Conf_1.000	663	20	4.47E-15	XP_001897733.1	48	85.5001	219	107	hypothetical protein Bm1_31440
Locus_12763_Transcript_1/1_Conf_1.000	235	7	8.70E-06	XP_002631981.1	75	53.5286	37	28	Hypothetical protein CBG10259
Locus_12764_Transcript_1/1_Conf_1.000	246	1	4.71E-04	NP_505419.3	69	47.7506	42	29	hypothetical protein K07B1.7

Locus_12765_Transcript_1/1_Conf_1.000	676	20	4.44E-74	AAL06642.1	80	281.567	201	162	serine-threonine protein kinase
Locus_12766_Transcript_1/1_Conf_1.000	452	0							
Locus_12767_Transcript_1/1_Conf_1.000	158	9	5.29E-19	XP_002488965.1	91	97.4413	46	42	hypothetical protein SORBIDRAFT_1138s002030
Locus_12768_Transcript_1/1_Conf_1.000	215	16	1.97E-10	NP_493125.2	66	68.9366	63	42	hypothetical protein T09E11.9
Locus_12769_Transcript_1/1_Conf_1.000	387	0							
Locus_1277_Transcript_1/1_Conf_1.000	519	20	1.15E-46	XP_002633893.1	77	189.504	132	102	C. briggsae CBR-ELO-5 protein
Locus_12770_Transcript_1/1_Conf_1.000	748	20	2.09E-97	NP_001021052.1	88	359.377	218	192	UDP-GALactose 4-Epimerase family member (gale-1)
Locus_12771_Transcript_1/1_Conf_1.000	259	0							
Locus_12772_Transcript_1/1_Conf_1.000	198	0							
Locus_12773_Transcript_1/1_Conf_1.000	477	20	3.92E-67	EFO21314.1	87	257.299	159	139	male sterility protein
Locus_12774_Transcript_1/1_Conf_1.000	1144	20	3.39E-70	XP_002639112.1	57	270.011	411	236	Hypothetical protein CBG14931
Locus_12775_Transcript_1/1_Conf_1.000	282	20	7.24E-08	XP_002630318.1	71	60.4622	69	49	Hypothetical protein CBG04241
Locus_12776_Transcript_1/2_Conf_1.000	523	0							
Locus_12776_Transcript_2/2_Conf_1.000	450	0							
Locus_12777_Transcript_1/1_Conf_1.000	177	20	1.85E-08	ZP_04173150.1	60	62.3882	56	34	LPXTG-motif cell wall anchor domain protein
Locus_12778_Transcript_1/1_Conf_1.000	502	1	8.24E-04	XP_002123338.1	56	46.9802	79	45	PREDICTED: similar to phosphatidylinositol glycan anchor biosynthesis, class G
Locus_12779_Transcript_1/1_Conf_1.000	264	3	9.65E-05	AAD37914.1	51	50.0618	102	53	R151.8B protein
Locus_1278_Transcript_1/1_Conf_1.000	707	20	2.78E-37	XP_001898151.1	70	159.458	140	98	Conserved hypothetical protein
Locus_12780_Transcript_1/1_Conf_1.000	306	20	2.25E-22	NP_001022063.1	72	108.612	101	73	Acyl CoA DeHydrogenase family member (acdh-12)
Locus_12781_Transcript_1/1_Conf_1.000	397	20	1.63E-60	XP_002634622.1	93	235.343	132	123	Hypothetical protein CBG18478
Locus_12782_Transcript_1/1_Conf_1.000	138	3	4.69E-12	CBA10134.1	82	74.3294	47	39	DAF-16A transcription factor

Locus_12783_Transcript_1/1_Conf_1.000	740	6	5.80E-44	NP_001032983.1	57	181.8	277	160	hypothetical protein W05F2.4
Locus_12784_Transcript_1/1_Conf_1.000	386	0							
Locus_12785_Transcript_1/1_Conf_1.000	219	0							
Locus_12786_Transcript_1/1_Conf_1.000	535	0							
Locus_12787_Transcript_1/1_Conf_1.000	206	0							
Locus_12788_Transcript_1/2_Conf_1.000	3164	20	1.68E-103	NP_500284.2	55	382.489	732	409	Enhancer of EfL-1 mutant phenotype family member (eel-1)
Locus_12788_Transcript_2/2_Conf_1.000	3164	20	1.68E-103	NP_500284.2	55	382.489	732	409	Enhancer of EfL-1 mutant phenotype family member (eel-1)
Locus_12789_Transcript_1/1_Conf_1.000	1004	20	1.09E-82	NP_500551.2	75	311.227	305	229	hypothetical protein F55F10.1
Locus_1279_Transcript_1/1_Conf_1.000	970	20	4.07E-55	XP_002637784.1	59	219.55	326	194	C. briggsae CBR-RFC-1 protein
Locus_12790_Transcript_1/1_Conf_1.000	150	0							
Locus_12791_Transcript_1/2_Conf_1.000	754	20	1.10E-13	XP_002646295.1	47	81.2629	165	79	Hypothetical protein CBG12001
Locus_12791_Transcript_2/2_Conf_1.000	742	20	7.46E-15	XP_002646295.1	52	85.1149	175	91	Hypothetical protein CBG12001
Locus_12792_Transcript_1/1_Conf_1.000	494	4	2.13E-12	NP_502353.1	67	75.485	84	57	hypothetical protein ZC518.1
Locus_12793_Transcript_1/1_Conf_1.000	378	20	2.97E-30	XP_002631828.1	78	134.806	100	78	Hypothetical protein CBG17763
Locus_12794_Transcript_1/1_Conf_1.000	314	0							
Locus_12795_Transcript_1/1_Conf_1.000	405	0							
Locus_12796_Transcript_1/1_Conf_1.000	635	1	4.04E-07	EFO17551.1	67	58.9214	71	48	hypothetical protein LOAG_10948
Locus_12797_Transcript_1/1_Conf_1.000	710	20	9.01E-44	AAD00842.1	66	181.03	195	130	OvB8

Locus_12798_Transcript_1/1_Conf_1.000	917	4	1.46E-51	NP_510523.1	66	207.608	245	162	hypothetical protein T10B10.3
Locus_12799_Transcript_1/1_Conf_1.000	357	20	3.73E-25	XP_002630560.1	71	117.857	115	82	C. briggsae CBR-NPP-21 protein
Locus_128_Transcript_1/1_Conf_1.000	148	0							
Locus_1280_Transcript_1/1_Conf_1.000	519	20	2.25E-42	EFO20386.1	65	175.252	157	103	hypothetical protein LOAG_08104
Locus_12800_Transcript_1/1_Conf_1.000	373	0							
Locus_12801_Transcript_1/1_Conf_1.000	790	15	7.81E-37	NP_506593.2	57	158.303	240	139	hypothetical protein R02D5.3
Locus_12802_Transcript_1/1_Conf_1.000	296	20	1.48E-29	EFO21682.1	75	132.494	95	72	hypothetical protein LOAG_06805
Locus_12803_Transcript_1/1_Conf_1.000	265	0							
Locus_12804_Transcript_1/1_Conf_1.000	128	0							
Locus_12805_Transcript_1/1_Conf_1.000	184	0							
Locus_12806_Transcript_1/2_Conf_0.667	1081	20	2.19E-31	XP_002600898.1	59	140.969	191	113	hypothetical protein BRAFLDRAFT_215136
Locus_12806_Transcript_2/2_Conf_0.833	1482	20	3.43E-31	XP_002600898.1	59	140.969	191	113	hypothetical protein BRAFLDRAFT_215136
Locus_12807_Transcript_1/1_Conf_1.000	229	20	6.25E-20	XP_002645118.1	80	100.523	75	60	Hypothetical protein CBG16811
Locus_12808_Transcript_1/1_Conf_1.000	556	20	1.38E-41	XP_002632423.1	55	172.94	205	114	C. briggsae CBR-SNF-9 protein
Locus_12809_Transcript_1/1_Conf_1.000	958	20	1.46E-65	NP_498963.1	61	254.218	322	197	Temporarily Assigned Gene name family member (tag-256)
Locus_1281_Transcript_1/1_Conf_1.000	342	20	9.65E-37	XP_002633235.1	79	156.377	116	92	Hypothetical protein CBG05956
Locus_12810_Transcript_1/1_Conf_1.000	444	0							
Locus_12811_Transcript_1/1_Conf_1.000	247	10	3.14E-16	XP_001892618.1	74	88.1965	79	59	Acyltransferase family protein

Locus_12812_Transcript_1/1_Conf_1.000	357	19	3.49E-31	NP_496395.1	75	137.887	95	72	LIn-26 Related family member (lir-3)
Locus_12813_Transcript_1/2_Conf_1.000	321	0							
Locus_12813_Transcript_2/2_Conf_1.000	793	20	3.54E-29	XP_001902329.1	54	132.88	250	136	TPR Domain containing protein
Locus_12814_Transcript_1/1_Conf_1.000	402	0							
Locus_12815_Transcript_1/1_Conf_1.000	524	20	1.38E-71	XP_002638567.1	87	272.322	174	152	Hypothetical protein CBG05609
Locus_12816_Transcript_1/1_Conf_1.000	484	20	1.02E-78	XP_002640630.1	95	295.819	161	154	C. briggsae CBR-APG-1 protein
Locus_12817_Transcript_1/1_Conf_1.000	553	20	1.59E-66	EFO24958.1	83	255.758	183	153	hypothetical protein LOAG_03524
Locus_12818_Transcript_1/1_Conf_1.000	386	0							
Locus_12819_Transcript_1/3_Conf_0.667	1139	20	6.73E-119	ADE60689.1	80	431.795	309	250	Larp (rna binding la related protein) homolog protein 1, isoform a, partially confirmed by transcript evidence
Locus_12819_Transcript_2/3_Conf_0.333	438	20	2.70E-68	ADE60689.1	90	261.151	146	132	Larp (rna binding la related protein) homolog protein 1, isoform a, partially confirmed by transcript evidence
Locus_12819_Transcript_3/3_Conf_0.667	1322	20	1.37E-129	ADE60688.1	77	467.618	373	289	Larp (rna binding la related protein) homolog protein 1, isoform b, partially confirmed by transcript evidence
Locus_1282_Transcript_1/1_Conf_1.000	501	20	2.70E-47	NP_492215.1	77	191.43	167	129	hypothetical protein K04G2.6
Locus_12820_Transcript_1/1_Conf_1.000	134	0							
Locus_12821_Transcript_1/1_Conf_1.000	232	20	7.37E-13	NP_497336.3	79	77.0258	74	59	hypothetical protein Y71D11A.3
Locus_12822_Transcript_1/1_Conf_1.000	571	20	1.13E-25	NP_741242.1	72	120.168	100	72	Dipeptidyl Peptidase Four (IV) family member (dpf-6)
Locus_12823_Transcript_1/1_Conf_1.000	401	20	1.43E-40	NP_505591.1	78	169.088	133	104	hypothetical protein R04B5.5
Locus_12824_Transcript_1/1_Conf_1.000	155	0							

Locus_12825_Transcript_1/1_Conf_1.000	333	20	1.67E-33	AAB23867.2	79	145.591	110	87	UNC-5
Locus_12826_Transcript_1/1_Conf_1.000	876	0							
Locus_12827_Transcript_1/1_Conf_1.000	297	0							
Locus_12828_Transcript_1/1_Conf_1.000	134	20	6.59E-14	YP_003434088.1	93	80.4925	44	41	cytochrome c oxidase subunit III
Locus_12829_Transcript_1/1_Conf_1.000	537	20	7.24E-34	XP_002633416.1	67	147.132	178	120	Hypothetical protein CBG06181
Locus_1283_Transcript_1/1_Conf_1.000	1057	20	3.46E-50	XP_002633732.1	55	203.371	372	206	Hypothetical protein CBG03417
Locus_12830_Transcript_1/1_Conf_1.000	258	7	7.42E-13	CAA10033.1	71	77.0258	78	56	DYS-1 protein
Locus_12831_Transcript_1/1_Conf_1.000	249	0							
Locus_12832_Transcript_1/1_Conf_1.000	584	20	1.07E-82	XP_002639182.1	93	309.686	184	172	C. briggsae CBR-TAG-316 protein
Locus_12833_Transcript_1/1_Conf_1.000	317	0							
Locus_12834_Transcript_1/1_Conf_1.000	157	3	6.25E-20	CAR63690.1	92	100.523	52	48	putative Sna protein
Locus_12835_Transcript_1/1_Conf_1.000	282	4	1.61E-07	EFO25881.1	56	59.3066	96	54	SPRY domain-containing protein
Locus_12836_Transcript_1/2_Conf_1.000	760	20	2.91E-54	XP_002634768.1	74	216.083	243	182	C. briggsae CBR-HLH-30 protein

Locus_12836_Transcript_2/2_Conf_1.000	751	20	6.84E-56	XP_002634768.1	75	221.476	240	182	C. briggsae CBR-HLH-30 protein
Locus_12837_Transcript_1/1_Conf_1.000	159	0							
Locus_12838_Transcript_1/1_Conf_1.000	568	3	8.56E-10	NP_001023556.1	88	67.3958	42	37	Membrane Calcium ATPase family member (mca-3)
Locus_12839_Transcript_1/1_Conf_1.000	153	0							
Locus_1284_Transcript_1/2_Conf_1.000	1061	7	1.51E-37	NP_492729.1	54	161.384	231	126	hypothetical protein Y106G6H.4
Locus_1284_Transcript_2/2_Conf_1.000	920	7	1.38E-41	NP_492729.1	65	174.481	184	121	hypothetical protein Y106G6H.4
Locus_12840_Transcript_1/1_Conf_1.000	495	20	1.78E-35	AAC78238.2	89	152.14	102	91	Guanylyl cyclase protein 28, isoform d
Locus_12841_Transcript_1/1_Conf_1.000	1362	20	3.25E-174	NP_501303.1	83	615.92	416	347	hypothetical protein C06A6.5
Locus_12842_Transcript_1/1_Conf_1.000	152	0							
Locus_12843_Transcript_1/1_Conf_1.000	360	4	1.28E-17	XP_002639414.1	79	92.8189	73	58	C. briggsae CBR-EIF-3.H protein
Locus_12844_Transcript_1/1_Conf_1.000	1312	20	1.77E-129	XP_002633684.1	70	467.233	455	319	C. briggsae CBR-EEL-1 protein

Locus_12845_Transcript_1/1_Conf_1.000	845	20	6.15E-123	XP_002635231.1	88	444.506	280	248	Hypothetical protein CBG11474
Locus_12846_Transcript_1/1_Conf_1.000	333	20	6.51E-54	NP_001138309.1	100	213.386	110	110	keratin, type I cytoskeletal 17
Locus_12847_Transcript_1/1_Conf_1.000	134	2	7.30E-05	XP_002645884.1	69	50.447	43	30	Hypothetical protein CBG07632
Locus_12848_Transcript_1/1_Conf_1.000	269	0							
Locus_12849_Transcript_1/1_Conf_1.000	154	0							
Locus_1285_Transcript_1/1_Conf_1.000	2370	20	0	XP_002638699.1	93	1173.3	690	642	Hypothetical protein CBG11896
Locus_12850_Transcript_1/1_Conf_1.000	407	20	5.04E-38	EFO20415.1	69	160.614	142	99	hyaluronidase
Locus_12851_Transcript_1/2_Conf_1.000	475	20	4.25E-29	P34578.3	66	130.954	149	99	Rap guanine nucleotide exchange factor 1
Locus_12851_Transcript_2/2_Conf_1.000	881	20	4.51E-47	P34578.3	56	192.586	308	173	Rap guanine nucleotide exchange factor 1
Locus_12852_Transcript_1/1_Conf_1.000	133	0							
Locus_12853_Transcript_1/1_Conf_1.000	246	0							

Locus_12854_Transcript_1/1_Conf_1.000	228	2	3.93E-06	NP_490772.2	62	54.6842	74	46	hypothetical protein Y18H1A.3
Locus_12855_Transcript_1/1_Conf_1.000	687	2	5.04E-12	EFO22475.1	55	75.485	135	75	PH domain-containing protein
Locus_12856_Transcript_1/1_Conf_1.000	1229	20	7.08E-93	EFO21585.1	65	345.51	413	271	hypothetical protein LOAG_06905
Locus_12857_Transcript_1/1_Conf_1.000	158	0							
Locus_12858_Transcript_1/1_Conf_1.000	676	4	1.15E-21	XP_002640452.1	58	107.457	198	115	Hypothetical protein CBG08509
Locus_12859_Transcript_1/1_Conf_1.000	819	2	1.43E-12	XP_002639525.1	43	77.7962	252	110	Hypothetical protein CBG04150
Locus_1286_Transcript_1/3_Conf_0.714	2169	20	4.10E-127	XP_002636551.1	58	460.299	652	383	Hypothetical protein CBG23241
Locus_1286_Transcript_2/3_Conf_0.714	2169	20	1.84E-127	AAX34423.1	58	461.455	653	381	anion transporter SULP-5
Locus_1286_Transcript_3/3_Conf_0.714	2169	20	1.08E-127	XP_002636551.1	58	462.225	652	384	Hypothetical protein CBG23241
Locus_12860_Transcript_1/1_Conf_1.000	406	0							
Locus_12861_Transcript_1/1_Conf_1.000	288	0							
Locus_12862_Transcript_1/1_Conf_1.000	305	20	1.12E-29	XP_002639182.1	93	132.88	79	74	C. briggsae CBR-TAG-316 protein
Locus_12863_Transcript_1/1_Conf_1.000	472	20	4.25E-45	XP_001900983.1	85	184.111	132	113	La domain containing protein
Locus_12864_Transcript_1/1_Conf_1.000	322	0							
Locus_12865_Transcript_1/1_Conf_1.000	686	20	3.29E-72	NP_508530.1	75	275.404	228	173	yeast SEC homolog family member (sec-3)

Locus_12866_Transcript_1/1_Conf_1.000	449	20	2.49E-61	NP_496269.1	89	238.039	152	136	hypothetical protein R166.2
Locus_12867_Transcript_1/1_Conf_1.000	421	20	2.52E-53	EFO17199.1	90	211.46	117	106	histone methyltransferase
Locus_12868_Transcript_1/1_Conf_1.000	843	20	5.34E-151	XP_537399.1	99	537.724	266	265	PREDICTED: similar to 40S ribosomal protein S4, X isoform
Locus_12869_Transcript_1/1_Conf_1.000	327	20	2.77E-36	XP_002637153.1	84	154.836	104	88	Hypothetical protein CBG09655
Locus_1287_Transcript_1/1_Conf_1.000	255	20	4.96E-33	XP_002698352.1	87	144.05	85	74	PREDICTED: hypothetical protein
Locus_12870_Transcript_1/1_Conf_1.000	642	20	3.29E-36	XP_001899664.1	69	155.606	136	95	Smad1
Locus_12871_Transcript_1/1_Conf_1.000	298	20	7.77E-47	XP_002644145.1	92	189.889	98	91	C. briggsae CBR-DAF-12 protein
Locus_12872_Transcript_1/1_Conf_1.000	356	20	2.13E-12	CAB07705.2	76	75.485	52	40	C. elegans protein ZC101.1, partially confirmed by transcript evidence
Locus_12873_Transcript_1/1_Conf_1.000	788	0							
Locus_12874_Transcript_1/1_Conf_1.000	467	0							
Locus_12875_Transcript_1/1_Conf_1.000	285	18	5.00E-09	NP_501190.1	82	64.3142	39	32	Temporarily Assigned Gene name family member (tag-224)

Locus_12876_Transcript_1/1_Conf_1.000	637	20	1.40E-23	AAA51433.1	95	113.62	63	60	guanine nucleotide regulatory protein
Locus_12877_Transcript_1/1_Conf_1.000	1310	20	7.19E-107	XP_001897028.1	67	392.119	394	267	Peptidase family M1 containing protein
Locus_12878_Transcript_1/1_Conf_1.000	366	0							

Locus_12879_Transcript_1/1_Conf_1.000	844	20	1.28E-11	NP_497036.1	62	74.7146	91	57	hypothetical protein F19H8.2
Locus_1288_Transcript_1/1_Conf_1.000	804	20	1.22E-16	EFO16079.1	49	91.2781	186	92	hypothetical protein LOAG_12429
Locus_12880_Transcript_1/1_Conf_1.000	128	20	5.09E-06	ZP_01004740.1	66	54.299	45	30	ribosomal RNA large subunit methyltransferase J
Locus_12881_Transcript_1/1_Conf_1.000	1570	20	1.15E-133	XP_002643167.1	73	481.485	435	319	Hypothetical protein CBG15348
Locus_12882_Transcript_1/1_Conf_1.000	291	0							
Locus_12883_Transcript_1/1_Conf_1.000	1272	0							

Locus_12884_Transcript_1/1_Conf_1.000	202	20	2.06E-23	AAA18956.1	91	112.079	62	57	Sur-1 MAP kinase
Locus_12885_Transcript_1/1_Conf_1.000	375	20	1.30E-17	XP_001901741.1	66	92.8189	125	83	Eukaryotic-type DNA primase, large subunit family protein
Locus_12886_Transcript_1/1_Conf_1.000	348	20	3.28E-53	XP_002646356.1	91	211.075	116	106	Hypothetical protein CBG12070
Locus_12887_Transcript_1/1_Conf_1.000	475	4	5.92E-39	NP_491915.2	88	163.696	157	139	hypothetical protein ZC581.9
Locus_12888_Transcript_1/1_Conf_1.000	804	20	3.90E-31	NP_001184122.1	73	139.428	116	85	sestrin-3
Locus_12889_Transcript_1/1_Conf_1.000	681	20	3.65E-23	EFO19714.1	70	112.464	106	75	hypothetical protein LOAG_08776
Locus_1289_Transcript_1/1_Conf_1.000	357	20	2.50E-29	NP_499668.1	71	131.724	111	79	hypothetical protein Y37D8A.2
Locus_12890_Transcript_1/1_Conf_1.000	138	20	1.05E-11	NP_509287.1	88	73.1738	42	37	hypothetical protein C44E12.1
Locus_12891_Transcript_1/1_Conf_1.000	372	8	1.49E-29	EFO20596.1	97	132.494	69	67	MYND finger family protein
Locus_12892_Transcript_1/1_Conf_1.000	448	0							

Locus_12893_Transcript_1/1_Conf_1.000	241	19	1.12E-21	NP_492014.4	75	106.301	79	60	hypothetical protein F22D6.11
Locus_12894_Transcript_1/1_Conf_1.000	345	0							
Locus_12895_Transcript_1/1_Conf_1.000	544	4	2.87E-25	NP_508889.1	61	118.627	168	103	hypothetical protein Y34B4A.7
Locus_12896_Transcript_1/1_Conf_1.000	822	0							
Locus_12897_Transcript_1/1_Conf_1.000	1391	20	9.61E-73	XP_002641571.1	66	278.87	294	196	Hypothetical protein CBG09872
Locus_12898_Transcript_1/1_Conf_1.000	128	20	1.29E-17	XP_002813872.1	100	92.8189	42	42	PREDICTED: lactotransferrin-like isoform 2
Locus_12899_Transcript_1/1_Conf_1.000	608	3	1.30E-04	XP_968882.1	45	50.447	203	93	PREDICTED: similar to cp27, putative
Locus_129_Transcript_1/1_Conf_1.000	197	6	3.77E-25	NP_492295.1	95	117.857	65	62	hypothetical protein F10D11.6
Locus_1290_Transcript_1/1_Conf_1.000	304	1	2.35E-11	NP_490917.3	50	72.0182	104	53	hypothetical protein Y20F4.2
Locus_12900_Transcript_1/1_Conf_1.000	266	20	3.39E-42	NP_508537.1	100	174.481	88	88	SPeCtrin family member (spc-1)
Locus_12901_Transcript_1/1_Conf_1.000	148	0							
Locus_12902_Transcript_1/1_Conf_1.000	334	1	1.74E-06	XP_002803306.1	48	55.8398	92	45	PREDICTED: hypothetical protein LOC100426317
Locus_12903_Transcript_1/1_Conf_1.000	696	0							
Locus_12904_Transcript_1/1_Conf_1.000	601	16	9.29E-24	EFO26799.1	51	114.005	185	95	HMG box family protein
Locus_12905_Transcript_1/1_Conf_1.000	604	20	1.14E-69	NP_500088.1	85	266.544	174	148	hypothetical protein Y77E11A.1
Locus_12906_Transcript_1/1_Conf_1.000	209	0							
Locus_12907_Transcript_1/1_Conf_1.000	264	17	2.08E-07	CAA88613.1	45	58.9214	73	33	salivary gland secretion protein 4

Locus_12908_Transcript_1/1_Conf_1.000	189	20	1.11E-24	XP_001898310.1	95	116.316	62	59	DNA-directed RNA polymerase II largest subunit
Locus_12909_Transcript_1/1_Conf_1.000	165	20	1.38E-19	XP_002912840.1	94	99.3673	54	51	PREDICTED: LOW QUALITY PROTEIN: protein unc-13 homolog A-like
Locus_1291_Transcript_1/1_Conf_1.000	855	20	3.78E-51	NP_001122835.1	69	206.068	195	135	UNCoordinated family member (unc-22)
Locus_12910_Transcript_1/2_Conf_1.000	275	0							
Locus_12910_Transcript_2/2_Conf_1.000	248	0							
Locus_12911_Transcript_1/1_Conf_1.000	292	0							
Locus_12912_Transcript_1/1_Conf_1.000	696	20	2.32E-36	NP_001022303.1	80	156.377	114	92	MethylMalonic Aciduria type A protein family member (mmaa-1)
Locus_12913_Transcript_1/1_Conf_1.000	451	1	4.76E-04	XP_001901417.1	63	47.7506	66	42	hypothetical protein Bm1_49755
Locus_12914_Transcript_1/1_Conf_1.000	346	6	1.65E-20	CAR63710.1	86	102.449	65	56	putative C2 domain containing protein
Locus_12915_Transcript_1/1_Conf_1.000	161	0							
Locus_12916_Transcript_1/1_Conf_1.000	367	0							
Locus_12917_Transcript_1/2_Conf_1.000	988	0							
Locus_12917_Transcript_2/2_Conf_1.000	859	0							
Locus_12918_Transcript_1/1_Conf_1.000	190	15	1.04E-14	NP_496066.2	80	83.1889	60	48	Nuclear Hormone Receptor family member (nhr-19)
Locus_12919_Transcript_1/1_Conf_1.000	757	20	7.13E-45	NP_509296.1	63	184.882	257	164	hypothetical protein F27D9.2
Locus_1292_Transcript_1/2_Conf_1.000	1832	0							
Locus_1292_Transcript_2/2_Conf_1.000	1826	0							
Locus_12920_Transcript_1/1_Conf_1.000	376	3	4.02E-35	NP_502233.2	77	150.984	117	91	hypothetical protein H01G02.1
Locus_12921_Transcript_1/1_Conf_1.000	148	0							
Locus_12922_Transcript_1/1_Conf_1.000	229	0							
Locus_12923_Transcript_1/1_Conf_1.000	164	0							
Locus_12924_Transcript_1/1_Conf_1.000	383	0							

Locus_12925_Transcript_1/1_Conf_1.000	405	20	1.98E-26	NP_001119697.1	74	122.094	89	66	casein kinase II beta subunit
Locus_12926_Transcript_1/1_Conf_1.000	651	6	1.28E-91	CAR63586.1	89	339.732	217	195	hypothetical protein
Locus_12927_Transcript_1/1_Conf_1.000	355	20	1.34E-06	XP_001894185.1	46	56.225	107	50	Major Sperm Protein (MSP) cytoskeletal MSP
Locus_12928_Transcript_1/1_Conf_1.000	142	3	1.78E-11	NP_508432.1	85	72.4034	47	40	hypothetical protein F47G3.1
Locus_12929_Transcript_1/2_Conf_1.000	764	0							
Locus_12929_Transcript_2/2_Conf_1.000	383	0							
Locus_1293_Transcript_1/1_Conf_1.000	679	20	1.44E-80	XP_002632216.1	78	303.138	225	176	Hypothetical protein CBG07084
Locus_12930_Transcript_1/1_Conf_1.000	233	0							
Locus_12931_Transcript_1/1_Conf_1.000	208	0							
Locus_12932_Transcript_1/1_Conf_1.000	588	20	6.02E-49	XP_002631639.1	67	197.593	187	126	Hypothetical protein CBG20828
Locus_12933_Transcript_1/1_Conf_1.000	298	0							
Locus_12934_Transcript_1/1_Conf_1.000	135	0							

Locus_12935_Transcript_1/1_Conf_1.000	347	20	8.78E-06	NP_001128605.1	46	53.5286	123	57	breast cancer anti-estrogen resistance 1
Locus_12936_Transcript_1/1_Conf_1.000	213	7	2.17E-25	EFO23509.1	95	118.627	69	66	STAT protein
Locus_12937_Transcript_1/1_Conf_1.000	735	20	3.68E-83	XP_001898650.1	82	311.997	243	201	SD04925p
Locus_12938_Transcript_1/1_Conf_1.000	685	20	2.70E-66	NP_496968.1	80	255.758	185	149	hypothetical protein Y48B6A.12
Locus_12939_Transcript_1/1_Conf_1.000	133	0							

Locus_1294_Transcript_1/2_Conf_1.000	1511	20	6.79E-176	AAF00540.1	99	621.698	319	318	AF187691_1casein kinase I
Locus_1294_Transcript_2/2_Conf_1.000	1501	20	6.74E-176	AAF00540.1	99	621.698	319	318	AF187691_1casein kinase I
Locus_12940_Transcript_1/1_Conf_1.000	833	20	2.40E-55	XP_002635067.1	75	219.935	239	180	Hypothetical protein CBG11281
Locus_12941_Transcript_1/1_Conf_1.000	945	20	3.43E-67	XP_002629865.1	65	259.61	242	158	C. briggsae CBR-ZTF-2 protein
Locus_12942_Transcript_1/1_Conf_1.000	196	0							
Locus_12943_Transcript_1/1_Conf_1.000	729	20	5.63E-52	XP_002645831.1	65	208.379	230	151	C. briggsae CBR-RBC-1 protein
Locus_12944_Transcript_1/1_Conf_1.000	207	0							
Locus_12945_Transcript_1/1_Conf_1.000	307	0							
Locus_12946_Transcript_1/1_Conf_1.000	385	3	2.68E-15	AAT12422.1	73	85.1149	105	77	hypothetical protein isoform a

Locus_12947_Transcript_1/1_Conf_1.000	542	6	4.65E-20	NP_491731.2	72	101.293	86	62	hypothetical protein C06A5.3
Locus_12948_Transcript_1/1_Conf_1.000	483	20	4.46E-42	NP_500460.1	72	174.096	155	113	hypothetical protein W02C12.1
Locus_12949_Transcript_1/1_Conf_1.000	351	0							
Locus_1295_Transcript_1/1_Conf_1.000	564	20	2.04E-64	NP_001021182.1	82	248.825	188	155	hypothetical protein C24A1.3
Locus_12950_Transcript_1/1_Conf_1.000	369								
Locus_12951_Transcript_1/1_Conf_1.000	300	0							
Locus_12952_Transcript_1/1_Conf_1.000	449	3	6.67E-06	ZP_06280939.1	45	53.9138	79	36	LPXTG-motif cell wall anchor domain protein
Locus_12953_Transcript_1/1_Conf_1.000	356	20	7.32E-21	XP_002644943.1	81	103.605	111	90	Hypothetical protein CBG10898
Locus_12954_Transcript_1/1_Conf_1.000	918	20	2.25E-76	ACM46026.1	68	290.041	304	208	Temporarily assigned gene name protein 233, isoform a, partially confirmed by transcript evidence
Locus_12955_Transcript_1/1_Conf_1.000	178	0							
Locus_12956_Transcript_1/1_Conf_1.000	421	2	1.35E-06	XP_002641991.1	51	56.225	147	75	Hypothetical protein CBG09214
Locus_12957_Transcript_1/1_Conf_1.000	558	0							
Locus_12958_Transcript_1/1_Conf_1.000	210	20	9.47E-21	EAX00648.1	100	103.219	50	50	hCG23722
Locus_12959_Transcript_1/1_Conf_1.000	177	20	2.75E-12	XP_002629697.1	77	75.0998	59	46	C. briggsae CBR-AQP-2 protein
Locus_1296_Transcript_1/1_Conf_1.000	429	0							
Locus_12960_Transcript_1/1_Conf_1.000	167	0							
Locus_12961_Transcript_1/1_Conf_1.000	360	0							
Locus_12962_Transcript_1/1_Conf_1.000	329	20	7.80E-31	NP_493554.1	81	136.732	98	80	hypothetical protein Y54E5A.6
Locus_12963_Transcript_1/1_Conf_1.000	131	0							
Locus_12964_Transcript_1/1_Conf_1.000	302	7	2.12E-28	XP_002635247.1	73	128.642	99	73	Hypothetical protein CBG11491
Locus_12965_Transcript_1/1_Conf_1.000	436	2	7.01E-16	NP_504797.1	49	87.0409	144	71	hypothetical protein VC5.2
Locus_12966_Transcript_1/1_Conf_1.000	277	20	3.59E-28	NP_500389.1	85	127.872	90	77	hypothetical protein ZC416.1
Locus_12967_Transcript_1/1_Conf_1.000	326	1	2.22E-09	NP_502613.2	64	65.4698	73	47	QUInine non-avoider family member (qui-1)
Locus_12968_Transcript_1/1_Conf_1.000	495	0							

Locus_12969_Transcript_1/1_Conf_1.000	156	0							
Locus_1297_Transcript_1/1_Conf_1.000	957	20	6.30E-85	NP_492334.1	87	318.546	214	187	hypothetical protein F43G9.5
Locus_12970_Transcript_1/1_Conf_1.000	421	0							
Locus_12971_Transcript_1/1_Conf_1.000	564	20	1.29E-26	NP_505444.1	63	123.25	138	87	FMRF-Like Peptide family member (flp-6)
Locus_12972_Transcript_1/1_Conf_1.000	424	20	2.12E-52	XP_002643051.1	83	208.379	139	116	C. briggsae CBR-DPF-6 protein
Locus_12973_Transcript_1/1_Conf_1.000	899	20	4.40E-37	XP_001900313.1	61	159.458	219	134	Piwi domain containing protein
Locus_12974_Transcript_1/1_Conf_1.000	539	0							
Locus_12975_Transcript_1/1_Conf_1.000	790	20	3.57E-90	XP_001896878.1	80	335.495	250	201	vesicle-fusing ATPase
Locus_12976_Transcript_1/1_Conf_1.000	250	0							
Locus_12977_Transcript_1/1_Conf_1.000	604	4	1.78E-14	EFO26702.1	61	83.1889	142	88	GTP-ase activating protein for Arf containing protein
Locus_12978_Transcript_1/1_Conf_1.000	432	0							
Locus_12979_Transcript_1/1_Conf_1.000	171	20	5.38E-24	NP_001024141.1	98	114.005	56	55	Germinal Center Kinase family member (gck-1)
Locus_1298_Transcript_1/3_Conf_0.667	577	3	4.16E-23	NP_495792.1	65	111.694	123	81	hypothetical protein M05D6.6
Locus_1298_Transcript_2/3_Conf_0.667	565	3	1.34E-23	NP_495792.1	65	113.235	123	81	hypothetical protein M05D6.6
Locus_1298_Transcript_3/3_Conf_0.667	565	3	3.89E-23	NP_495792.1	65	111.694	123	81	hypothetical protein M05D6.6
Locus_12980_Transcript_1/1_Conf_1.000	458	20	5.36E-24	XP_001900529.1	56	114.005	141	79	CG17219-PA
Locus_12981_Transcript_1/1_Conf_1.000	698	20	1.60E-53	NP_502420.2	76	213.386	194	149	hypothetical protein C26H9A.2
Locus_12982_Transcript_1/1_Conf_1.000	1663	20	6.14E-149	NP_495532.2	80	532.332	454	366	hypothetical protein F21D12.3
Locus_12983_Transcript_1/1_Conf_1.000	644	2	4.93E-24	NP_001033409.1	65	115.161	152	100	hypothetical protein F29B9.12

Locus_12984_Transcript_1/1_Conf_1.000	256	20	7.18E-24	NP_001022680.1	83	113.62	77	64	hypothetical protein K11H3.6
Locus_12985_Transcript_1/1_Conf_1.000	182	0							
Locus_12986_Transcript_1/1_Conf_1.000	479	4	1.08E-08	NP_492518.1	88	63.1586	36	32	hypothetical protein F30A10.6
Locus_12987_Transcript_1/1_Conf_1.000	185	0							
Locus_12988_Transcript_1/1_Conf_1.000	149	20	1.08E-11	XP_002640991.1	75	73.1738	48	36	Hypothetical protein CBG11740
Locus_12989_Transcript_1/1_Conf_1.000	228	0							
Locus_1299_Transcript_1/1_Conf_1.000	1862	20	8.03E-60	NP_506181.2	56	236.498	420	238	hypothetical protein R11D1.10
Locus_12990_Transcript_1/1_Conf_1.000	1454	20	0	XP_001898240.1	84	707.212	483	410	XPA-binding protein 2
Locus_12991_Transcript_1/1_Conf_1.000	215	0							
Locus_12992_Transcript_1/1_Conf_1.000	162	0							
Locus_12993_Transcript_1/1_Conf_1.000	175	0							
Locus_12994_Transcript_1/1_Conf_1.000	391	0							
Locus_12995_Transcript_1/1_Conf_1.000	367	4	1.00E-30	XP_002639732.1	68	136.346	124	85	C. briggsae CBR-TAG-264 protein
Locus_12996_Transcript_1/1_Conf_1.000	878	20	3.34E-26	XP_001892273.1	48	123.25	294	144	hypothetical protein
Locus_12997_Transcript_1/1_Conf_1.000	879	0							
Locus_12998_Transcript_1/2_Conf_1.000	2488	20	4.26E-99	XP_002636770.1	56	367.466	651	365	Hypothetical protein CBG23501
Locus_12998_Transcript_2/2_Conf_1.000	2473	20	6.31E-95	XP_002636770.1	55	353.599	651	361	Hypothetical protein CBG23501
Locus_12999_Transcript_1/1_Conf_1.000	274	0							
Locus_13_Transcript_1/1_Conf_1.000	251	0							
Locus_130_Transcript_1/6_Conf_0.250	885	20	5.66E-114	XP_002634359.1	96	414.846	221	214	C. briggsae CBR-RPS-2 protein

Locus_130_Transcript_2/6_Conf_0.375	1992	20	4.50E-149	XP_002636413.1	78	533.102	392	307	Hypothetical protein CBG23066
Locus_130_Transcript_3/6_Conf_0.250	1031	20	1.47E-114	XP_002634359.1	96	417.157	222	215	C. briggsae CBR-RPS-2 protein
Locus_130_Transcript_4/6_Conf_0.375	2180	20	5.00E-149	XP_002636413.1	78	533.102	392	307	Hypothetical protein CBG23066
Locus_130_Transcript_5/6_Conf_0.250	1031	20	7.31E-114	XP_002634359.1	96	414.846	221	214	C. briggsae CBR-RPS-2 protein
Locus_130_Transcript_6/6_Conf_0.375	2180	20	5.00E-149	XP_002636413.1	78	533.102	392	307	Hypothetical protein CBG23066
Locus_1300_Transcript_1/1_Conf_1.000	983	0							
Locus_13000_Transcript_1/1_Conf_1.000	301	0							

Locus_13001_Transcript_1/1_Conf_1.000	391	20	1.30E-62	1LYA	100	242.276	117	117	CrystalStructures Of Native And Inhibited Forms Of Human Cathepsin D: Implications For Lysosomal Targeting And Drug Design
Locus_13002_Transcript_1/1_Conf_1.000	178	0							
Locus_13003_Transcript_1/1_Conf_1.000	299	20	1.13E-21	NP_495836.2	80	106.301	89	72	Variable ABnormal morphology family member (vab-9)
Locus_13004_Transcript_1/1_Conf_1.000	406	20	2.05E-23	ACI49193.1	84	112.079	72	61	hypothetical protein Csp3_JD03.002
Locus_13005_Transcript_1/1_Conf_1.000	248	0							
Locus_13006_Transcript_1/1_Conf_1.000	218	9	2.57E-10	CAD28559.2	81	68.5514	48	39	metalloprotease I
Locus_13007_Transcript_1/1_Conf_1.000	228	0							
Locus_13008_Transcript_1/1_Conf_1.000	433	20	8.29E-25	NP_504567.1	61	116.701	143	88	hypothetical protein R01B10.4
Locus_13009_Transcript_1/1_Conf_1.000	307	0							
Locus_1301_Transcript_1/1_Conf_1.000	1108	4	3.28E-38	XP_002643523.1	51	163.696	345	177	Hypothetical protein CBG16200
Locus_13010_Transcript_1/1_Conf_1.000	289	0							
Locus_13011_Transcript_1/1_Conf_1.000	628	2	1.34E-31	NP_506277.1	54	140.198	214	116	High Incidence of Males (increased X chromosome loss) family member (him-17)
Locus_13012_Transcript_1/1_Conf_1.000	296	0							
Locus_13013_Transcript_1/1_Conf_1.000	752								
Locus_13014_Transcript_1/1_Conf_1.000	203	0							
Locus_13015_Transcript_1/1_Conf_1.000	550	1	2.22E-04	XP_002637364.1	56	49.2914	69	39	Hypothetical protein CBG19064
Locus_13016_Transcript_1/1_Conf_1.000	175	0							
Locus_13017_Transcript_1/1_Conf_1.000	584	20	3.19E-18	ACJ01664.1	67	95.5153	81	55	DAF-12 C4
Locus_13018_Transcript_1/1_Conf_1.000	204	0							
Locus_13019_Transcript_1/1_Conf_1.000	276	0							
Locus_1302_Transcript_1/2_Conf_1.000	1289	0							
Locus_1302_Transcript_2/2_Conf_1.000	1279	0							
Locus_13020_Transcript_1/1_Conf_1.000	139	4	1.24E-12	NP_505034.1	73	76.2554	46	34	hypothetical protein F40A3.7
Locus_13021_Transcript_1/1_Conf_1.000	468	4	6.44E-09	XP_002630296.1	58	63.929	98	57	C. briggsae CBR-EMB-27 protein
Locus_13022_Transcript_1/1_Conf_1.000	645	3	2.83E-19	XP_001902425.1	58	99.3673	144	84	hypothetical protein Bm1_54835
Locus_13023_Transcript_1/1_Conf_1.000	327	0							

Locus_13024_Transcript_1/1_Conf_1.000	753	20	2.95E-67	XP_001899456.1	73	259.225	248	182	Tetraspanin family protein
Locus_13025_Transcript_1/1_Conf_1.000	365	20	1.12E-21	XP_002633316.1	78	106.301	85	67	C. briggsae CBR-GTL-2 protein
Locus_13026_Transcript_1/1_Conf_1.000	257	0							
Locus_13027_Transcript_1/1_Conf_1.000	741	20	9.52E-95	NP_494897.3	79	350.517	249	197	hypothetical protein R05F9.12
Locus_13028_Transcript_1/1_Conf_1.000	268	16	1.93E-13	XP_002629956.1	88	78.9518	45	40	C. briggsae CBR-MLT-7 protein
Locus_13029_Transcript_1/1_Conf_1.000	289	20	5.87E-18	NP_495990.1	63	93.9745	95	60	Tubulin Tyrosine Ligase Like family member (ttll-12)
Locus_1303_Transcript_1/1_Conf_1.000	1421	20	3.62E-91	NP_496549.1	94	340.117	190	179	RAB family member (rab-7)
Locus_13030_Transcript_1/1_Conf_1.000	263	0							
Locus_13031_Transcript_1/1_Conf_1.000	788	20	1.56E-45	ABB88219.1	72	187.193	168	121	Hypothetical protein Y48G1C.12
Locus_13032_Transcript_1/1_Conf_1.000	1755	20	1.26E-91	XP_001901778.1	65	342.043	430	281	Probable molybdopterin binding domain containing protein
Locus_13033_Transcript_1/1_Conf_1.000	1226	20	0	XP_001118006.2	100	673.315	327	327	PREDICTED: elongation factor 2
Locus_13034_Transcript_1/1_Conf_1.000	796	1	1.61E-05	CBI55964.1	42	54.299	156	66	unnamed protein product
Locus_13035_Transcript_1/1_Conf_1.000	848	0							

Locus_13036_Transcript_1/1_Conf_1.000	576	20	1.03E-05	YP_002522680.1	51	53.9138	127	66	hypothetical protein trd_1477
Locus_13037_Transcript_1/1_Conf_1.000	248	2	1.32E-06	NP_502588.1	59	56.225	74	44	NucleOLar protein family member (nol-9)
Locus_13038_Transcript_1/1_Conf_1.000	316	0							
Locus_13039_Transcript_1/1_Conf_1.000	480	20	3.12E-32	EFO26003.1	68	141.354	157	107	SPRY domain-containing protein
Locus_1304_Transcript_1/1_Conf_1.000	769	20	1.49E-29	XP_002648943.1	60	134.035	165	99	Hypothetical protein CBG21256
Locus_13040_Transcript_1/1_Conf_1.000	710	20	1.20E-96	NP_498704.1	85	356.681	233	199	COenzyme Q (ubiquinone) biosynthesis family member (coq-5)
Locus_13041_Transcript_1/1_Conf_1.000	297	0							
Locus_13042_Transcript_1/2_Conf_1.000	302	0							
Locus_13042_Transcript_2/2_Conf_1.000	441	0							
Locus_13043_Transcript_1/1_Conf_1.000	139	0							
Locus_13044_Transcript_1/2_Conf_1.000	238	0							
Locus_13044_Transcript_2/2_Conf_1.000	376	0							
Locus_13045_Transcript_1/1_Conf_1.000	878	20	7.09E-77	NP_505070.2	68	291.582	296	202	Valyl tRNA Synthetase family member (vrs-1)
Locus_13046_Transcript_1/1_Conf_1.000	567	0							
Locus_13047_Transcript_1/1_Conf_1.000	211	2	1.67E-09	EFO27604.1	71	65.855	70	50	hypothetical protein LOAG_00873
Locus_13048_Transcript_1/1_Conf_1.000	1552	20	0	XP_002647888.1	87	853.588	516	450	C. briggsae CBR-IRS-1 protein
Locus_13049_Transcript_1/1_Conf_1.000	393	6	1.19E-18	NP_001021427.1	82	96.2857	119	98	hypothetical protein F28H1.4

Locus_1305_Transcript_1/2_Conf_1.000	2638	19	4.39E-09	YP_867496.1	38	68.5514	565	218	Zinc finger-domain-containing protein
Locus_1305_Transcript_2/2_Conf_1.000	472	0							
Locus_13050_Transcript_1/1_Conf_1.000	355	20	1.47E-29	NP_503604.2	78	132.494	114	90	hypothetical protein F44C8.7
Locus_13051_Transcript_1/1_Conf_1.000	415	0							
Locus_13052_Transcript_1/1_Conf_1.000	1019	20	8.53E-91	NP_001021798.2	74	338.191	327	243	Arf-1 Guanine nucleotide Exchange Factor homolog family member (agef-1)
Locus_13053_Transcript_1/1_Conf_1.000	368	20	9.96E-55	BAA08392.1	100	216.083	107	107	vacuolar ATPase
Locus_13054_Transcript_1/1_Conf_1.000	501	5	7.60E-50	NP_496398.2	79	199.904	140	111	hypothetical protein T06D8.1
Locus_13055_Transcript_1/1_Conf_1.000	396	0							
Locus_13056_Transcript_1/1_Conf_1.000	305	8	9.52E-13	XP_001900282.1	68	76.6406	67	46	hypothetical protein Bm1_44100
Locus_13057_Transcript_1/1_Conf_1.000	838	20	4.39E-89	NP_001022911.1	76	332.028	279	214	HELicase family member (hel-308)
Locus_13058_Transcript_1/1_Conf_1.000	472	20	1.16E-50	NP_492624.2	77	202.601	158	122	hypothetical protein F52F12.7
Locus_13059_Transcript_1/1_Conf_1.000	146	3	8.27E-20	EEE74739.1	100	100.138	48	48	predicted protein

Locus_1306_Transcript_1/1_Conf_1.000	1387	20	5.91E-38	EFO24120.1	61	163.31	239	148	CAMK/TRBL protein kinase
Locus_13060_Transcript_1/1_Conf_1.000	144	0							
Locus_13061_Transcript_1/1_Conf_1.000	419	20	1.22E-39	NP_510447.1	74	166.007	139	103	hypothetical protein C05G5.1
Locus_13062_Transcript_1/1_Conf_1.000	721	20	1.55E-38	XP_002641616.1	60	163.696	252	152	C. briggsae CBR-ALX-1 protein
Locus_13063_Transcript_1/1_Conf_1.000	433	20	7.73E-39	NP_490837.2	79	163.31	129	103	GRainyHead (Drosophila transcription factor) homolog family member (grh-1)
Locus_13064_Transcript_1/1_Conf_1.000	181	0							
Locus_13065_Transcript_1/3_Conf_0.667	797	20	6.81E-105	NP_496077.1	80	384.415	268	216	hypothetical protein ZK1307.9
Locus_13065_Transcript_2/3_Conf_0.556	821	20	1.39E-108	NP_496077.1	81	396.741	274	222	hypothetical protein ZK1307.9
Locus_13065_Transcript_3/3_Conf_0.667	821	20	1.39E-108	NP_496077.1	81	396.741	274	222	hypothetical protein ZK1307.9
Locus_13066_Transcript_1/1_Conf_1.000	737	8	3.28E-23	XP_002642159.1	50	112.849	217	110	Hypothetical protein CBG18119
Locus_13067_Transcript_1/1_Conf_1.000	732	5	6.33E-27	A8WN14.3	57	125.176	248	143	E3 UFM1-protein ligase 1 homolog
Locus_13068_Transcript_1/2_Conf_1.000	343	20	1.45E-16	EFO27351.1	66	89.3521	89	59	hypothetical protein LOAG_01121
Locus_13068_Transcript_2/2_Conf_1.000	771	20	2.74E-31	EFO27351.1	65	139.813	144	95	hypothetical protein LOAG_01121
Locus_13069_Transcript_1/1_Conf_1.000	393	0							
Locus_1307_Transcript_1/1_Conf_1.000	498	20	1.22E-44	NP_500024.1	76	182.57	133	102	AuTophaGy (yeast Atg homolog) family member (atg-3)
Locus_13070_Transcript_1/2_Conf_1.000	1052	20	1.49E-37	EFO27069.1	58	161.384	203	119	Ulp1 protease

Locus_13070_Transcript_2/2_Conf_1.000	995	20	1.37E-37	EFO27069.1	58	161.384	203	119	Ulp1 protease
Locus_13071_Transcript_1/1_Conf_1.000	796	20	5.87E-40	EFO26083.1	76	168.703	142	108	hypothetical protein LOAG_02395
Locus_13072_Transcript_1/1_Conf_1.000	272	0							
Locus_13073_Transcript_1/1_Conf_1.000	168	3	3.29E-05	XP_002630736.1	66	51.6026	54	36	Hypothetical protein CBG02420
Locus_13074_Transcript_1/1_Conf_1.000	396	20	3.75E-57	NP_001021410.2	88	224.172	132	117	LIM domain family member (lim-9)
Locus_13075_Transcript_1/1_Conf_1.000	191	0							
Locus_13076_Transcript_1/1_Conf_1.000	837	0							
Locus_13077_Transcript_1/1_Conf_1.000	297	20	9.57E-21	AAF36480.1	88	103.219	61	54	glutathione S-transferase 2
Locus_13078_Transcript_1/1_Conf_1.000	887	20	3.17E-48	EFO25981.1	64	196.438	214	139	MBD3 protein
Locus_13079_Transcript_1/1_Conf_1.000	463	20	2.21E-41	EFO25969.1	77	171.785	148	115	abnormal epIthelia family member
Locus_1308_Transcript_1/1_Conf_1.000	353	2	2.71E-07	XP_002643980.1	53	58.5362	101	54	C. briggsae CBR-OCT-2 protein
Locus_13080_Transcript_1/1_Conf_1.000	521	3	6.82E-07	XP_002647552.1	55	57.3806	95	53	Hypothetical protein CBG06638
Locus_13081_Transcript_1/1_Conf_1.000	2063	20	0	EFO26190.1	76	647.121	510	390	BTB/POZ domain-containing protein 2
Locus_13082_Transcript_1/2_Conf_1.000	895	20	3.45E-50	EFO21942.1	75	202.986	160	121	kynurenine-oxoglutarate transaminase
Locus_13082_Transcript_2/2_Conf_1.000	895	20	3.45E-50	EFO21942.1	75	202.986	160	121	kynurenine-oxoglutarate transaminase
Locus_13083_Transcript_1/1_Conf_1.000	790	0							
Locus_13084_Transcript_1/1_Conf_1.000	193	2	7.92E-15	NP_498089.2	77	83.5741	62	48	hypothetical protein Y54H5A.2
Locus_13085_Transcript_1/1_Conf_1.000	880	20	6.31E-41	XP_001900446.1	95	172.17	86	82	MYND finger family protein
Locus_13086_Transcript_1/1_Conf_1.000	229	20	4.63E-15	NP_500708.1	71	84.3445	63	45	hypothetical protein C09B9.4
Locus_13087_Transcript_1/1_Conf_1.000	372	0							

Locus_13088_Transcript_1/1_Conf_1.000	479	20	1.50E-34	NP_503037.2	72	149.058	121	88	ITSN (intersectin) family member (itsn-1)
Locus_13089_Transcript_1/1_Conf_1.000	406	0							
Locus_1309_Transcript_1/1_Conf_1.000	2613	20	0	XP_002637775.1	90	640.958	390	351	C. briggsae CBR-UNC-112 protein
Locus_13090_Transcript_1/1_Conf_1.000	526	18	4.91E-24	XP_002643238.1	88	114.39	70	62	Hypothetical protein CBG08105
Locus_13091_Transcript_1/1_Conf_1.000	530	0							
Locus_13092_Transcript_1/1_Conf_1.000	334	0							
Locus_13093_Transcript_1/2_Conf_1.000	579	20	3.25E-10	NP_502107.1	65	62.7734	55	36	COLlagen family member (col-125)
Locus_13093_Transcript_2/2_Conf_1.000	579	20	1.49E-10	NP_502107.1	65	63.929	55	36	COLlagen family member (col-125)
Locus_13094_Transcript_1/1_Conf_1.000	176	20	1.20E-15	XP_001898544.1	84	86.2705	58	49	Probable methylenetetrahydrofolate reductase
Locus_13095_Transcript_1/1_Conf_1.000	305	0							
Locus_13096_Transcript_1/1_Conf_1.000	310	0							
Locus_13097_Transcript_1/1_Conf_1.000	460	20	1.19E-23	CAV31771.1	96	112.849	52	50	C. elegans protein F25D7.3b, partially confirmed by transcript evidence
Locus_13098_Transcript_1/1_Conf_1.000	473	5	2.97E-22	NP_497202.2	65	108.227	110	72	hypothetical protein F10C5.2
Locus_13099_Transcript_1/1_Conf_1.000	322	0							

Locus_131_Transcript_1/1_Conf_1.000	1566	20	2.64E-130	ADD54353.1	97	470.315	385	375	NADH dehydrogenase subunit 4
Locus_1310_Transcript_1/2_Conf_0.857	546	19	3.36E-05	EFO26634.1	40	51.9878	106	43	hypothetical protein LOAG_01858
Locus_1310_Transcript_2/2_Conf_0.857	516	5	1.07E-04	XP_539701.2	54	50.0618	55	30	PREDICTED: similar to CG31690-PB, isoform B
Locus_13100_Transcript_1/1_Conf_1.000	315	20	2.71E-39	NP_001041037.1	86	164.851	105	91	GLYcosylation related family member (gly-10)
Locus_13101_Transcript_1/1_Conf_1.000	440	20	9.96E-55	NP_508530.1	85	216.083	138	118	yeast SEC homolog family member (sec-3)
Locus_13102_Transcript_1/1_Conf_1.000	207	4	1.20E-15	XP_002642004.1	100	86.2705	41	41	C. briggsae CBR-TOH-1 protein
Locus_13103_Transcript_1/1_Conf_1.000	253	20	3.81E-33	XP_002640505.1	91	144.436	84	77	Hypothetical protein CBG13644
Locus_13104_Transcript_1/1_Conf_1.000	161	20	1.45E-21	CAB93148.1	97	79.337	35	34	HDJ2 protein
Locus_13105_Transcript_1/1_Conf_1.000	174	0							
Locus_13106_Transcript_1/1_Conf_1.000	579	2	1.19E-09	XP_002642979.1	80	67.0106	50	40	Hypothetical protein CBG15263

Locus_13107_Transcript_1/1_Conf_1.000	301	20	5.03E-38	XP_001899774.1	92	160.614	93	86	Guanine nucleotide exchange factor for Ras-like GTPases; N-terminal motif family protein
Locus_13108_Transcript_1/1_Conf_1.000	241	0							
Locus_13109_Transcript_1/1_Conf_1.000	1259	20	5.19E-107	NP_496341.1	68	392.504	393	268	EGG sterile (unfertilizable) family member (egg-3)
Locus_1311_Transcript_1/1_Conf_1.000	1046	18	4.94E-17	ABB53347.1	38	93.2041	306	119	secreted protein 5 precursor
Locus_13110_Transcript_1/1_Conf_1.000	328	8	3.17E-32	NP_001024859.1	79	141.354	110	87	hypothetical protein R11G1.6
Locus_13111_Transcript_1/1_Conf_1.000	219	0							
Locus_13112_Transcript_1/1_Conf_1.000	185	11	1.36E-14	XP_002631488.1	80	82.8037	61	49	C. briggsae CBR-PTR-8 protein
Locus_13113_Transcript_1/1_Conf_1.000	414	2	2.48E-21	XP_002633521.1	76	105.145	86	66	Hypothetical protein CBG05384
Locus_13114_Transcript_1/1_Conf_1.000	929	20	7.07E-102	NP_001024859.1	75	374.785	317	239	hypothetical protein R11G1.6
Locus_13115_Transcript_1/2_Conf_1.000	785	20	4.39E-16	XP_002158068.1	51	89.3521	176	90	PREDICTED: similar to Forkhead box protein J1
Locus_13115_Transcript_2/2_Conf_1.000	776	20	6.61E-17	XP_002158068.1	50	92.0485	173	87	PREDICTED: similar to Forkhead box protein J1

Locus_13116_Transcript_1/1_Conf_1.000	597	20	8.14E-81	CAB07859.3	83	303.523	206	173	C. elegans protein W09D10.2, partially confirmed by transcript evidence
Locus_13117_Transcript_1/1_Conf_1.000	234	0							
Locus_13118_Transcript_1/1_Conf_1.000	464	20	5.99E-63	NP_502809.2	88	243.432	153	135	hypothetical protein Y41E3.3
Locus_13119_Transcript_1/1_Conf_1.000	502	20	8.96E-59	XP_002630065.1	82	229.565	156	129	Hypothetical protein CBG13441
Locus_1312_Transcript_1/4_Conf_0.250	1458	5	7.02E-13	XP_002642206.1	40	80.1073	228	92	C. briggsae CBR-BRP-1 protein
Locus_1312_Transcript_2/4_Conf_0.250	1496	5	7.27E-13	XP_002642206.1	40	80.1073	228	92	C. briggsae CBR-BRP-1 protein
Locus_1312_Transcript_3/4_Conf_0.667	1503	5	7.33E-13	XP_002642206.1	40	80.1073	228	92	C. briggsae CBR-BRP-1 protein
Locus_1312_Transcript_4/4_Conf_0.667	1509	5	7.37E-13	XP_002642206.1	40	80.1073	228	92	C. briggsae CBR-BRP-1 protein
Locus_13120_Transcript_1/1_Conf_1.000	205	0							
Locus_13121_Transcript_1/1_Conf_1.000	261	0							
Locus_13122_Transcript_1/1_Conf_1.000	451	5	1.25E-12	XP_001901679.1	89	76.2554	46	41	Immunoglobulin I-set domain containing protein
Locus_13123_Transcript_1/1_Conf_1.000	750	20	7.85E-44	NP_001122771.1	59	181.415	251	149	hypothetical protein F13H10.4
Locus_13124_Transcript_1/1_Conf_1.000	610	20	5.60E-72	XP_002637344.1	83	274.248	181	152	C. briggsae CBR-ODC-1 protein
Locus_13125_Transcript_1/1_Conf_1.000	563	20	4.00E-97	EFB27138.1	100	357.451	175	175	hypothetical protein PANDA_001098

Locus_13126_Transcript_1/1_Conf_1.000	329	20	3.25E-53	EAW77587.1	100	211.075	109	109	annexin A2, isoform CRA_c
Locus_13127_Transcript_1/1_Conf_1.000	284	0							
Locus_13128_Transcript_1/2_Conf_1.000	417	0							
Locus_13128_Transcript_2/2_Conf_1.000	869	6	1.44E-21	XP_002635348.1	57	107.842	208	120	C. briggsae CBR-DNJ-22 protein
Locus_13129_Transcript_1/1_Conf_1.000	437	13	6.14E-12	XP_002412316.1	59	73.9442	105	62	conserved hypothetical protein
Locus_1313_Transcript_1/2_Conf_1.000	1167	20	1.16E-73	XP_002641912.1	82	281.567	242	200	C. briggsae CBR-RNP-7 protein
Locus_1313_Transcript_2/2_Conf_1.000	835	20	3.77E-16	XP_001896769.1	69	89.7373	95	66	hypothetical protein Bm1_26580
Locus_13130_Transcript_1/1_Conf_1.000	129	20	6.40E-09	XP_002603055.1	86	63.929	38	33	hypothetical protein BRAFLDRAFT_274883

Locus_13131_Transcript_1/1_Conf_1.000	261	0							
Locus_13132_Transcript_1/1_Conf_1.000	645	15	3.83E-16	XP_002646317.1	68	88.9669	147	101	Hypothetical protein CBG12024
Locus_13133_Transcript_1/1_Conf_1.000	281	2	1.01E-06	XP_002639296.1	54	56.6102	95	52	Hypothetical protein CBG03866
Locus_13134_Transcript_1/1_Conf_1.000	768	20	9.91E-58	XP_001893225.1	64	227.639	257	165	Myb-like DNA-binding domain containing protein
Locus_13135_Transcript_1/1_Conf_1.000	410	20	6.34E-33	XP_002632976.1	70	143.665	122	86	Hypothetical protein CBG21736
Locus_13136_Transcript_1/1_Conf_1.000	203	20	7.11E-08	NP_001079447.1	69	60.4622	56	39	phosphatidylinositol-specific phospholipase C, X domain containing 2
Locus_13137_Transcript_1/1_Conf_1.000	1025	0							
Locus_13138_Transcript_1/1_Conf_1.000	130	4	5.78E-18	NP_001022641.1	95	93.9745	43	41	LEThal family member (let-805)
Locus_13139_Transcript_1/1_Conf_1.000	239	20	4.41E-34	NP_496717.1	98	147.517	79	78	hypothetical protein Y46G5A.12
Locus_1314_Transcript_1/1_Conf_1.000	933	4	1.75E-07	NP_741911.1	65	61.2326	66	43	Suppressor of Constitutive Dauer formation family member (scd-1)
Locus_13140_Transcript_1/1_Conf_1.000	275	7	1.47E-05	NP_740998.1	63	52.7582	46	29	hypothetical protein C25H3.7
Locus_13141_Transcript_1/1_Conf_1.000	143	20	1.32E-06	CAB00874.2	63	56.225	47	30	C. elegans protein R09H10.5, partially confirmed by transcript evidence

Locus_13142_Transcript_1/1_Conf_1.000	148	8	7.72E-18	XP_002640908.1	95	93.5893	48	46	Hypothetical protein CBG00466
Locus_13143_Transcript_1/1_Conf_1.000	239	0							
Locus_13144_Transcript_1/1_Conf_1.000	312	20	4.20E-40		87	167.548	102	89	protein K10D6.2b
Locus_13145_Transcript_1/2_Conf_1.000	787	20	2.22E-92	XP_002631607.1	78	342.813	261	204	Hypothetical protein CBG20788
Locus_13145_Transcript_2/2_Conf_1.000	787	20	4.94E-92	XP_002631607.1	77	341.658	261	203	Hypothetical protein CBG20788
Locus_13146_Transcript_1/1_Conf_1.000	661	20	1.31E-67	XP_002641643.1	81	259.996	180	147	C. briggsae CBR-SQV-3 protein
Locus_13147_Transcript_1/1_Conf_1.000	181	0							
Locus_13148_Transcript_1/1_Conf_1.000	450	0							
Locus_13149_Transcript_1/1_Conf_1.000	598	1	8.06E-04	XP_002063859.1	40	47.7506	159	64	GK15899
Locus_1315_Transcript_1/1_Conf_1.000	1262	0							
Locus_13150_Transcript_1/2_Conf_1.000	374	0							
Locus_13150_Transcript_2/2_Conf_1.000	281	0							
Locus_13151_Transcript_1/1_Conf_1.000	720	20	3.04E-10	XP_001323102.1	48	69.707	235	115	viral A-type inclusion protein
Locus_13152_Transcript_1/1_Conf_1.000	288	2	1.66E-04	XP_001898158.1	53	49.2914	86	46	hypothetical protein Bm1_33560
Locus_13153_Transcript_1/2_Conf_1.000	1066	20	1.62E-71	NP_509394.1	78	274.248	224	175	hypothetical protein M60.7
Locus_13153_Transcript_2/2_Conf_1.000	1065	20	1.62E-71	NP_509394.1	78	274.248	224	175	hypothetical protein M60.7
Locus_13154_Transcript_1/1_Conf_1.000	331	20	3.85E-38	NP_492132.1	90	160.999	110	99	Enhancer of Glp-One (glp-1) family member (ego-1)
Locus_13155_Transcript_1/2_Conf_1.000	584	0							
Locus_13155_Transcript_2/2_Conf_1.000	584	0							
Locus_13156_Transcript_1/1_Conf_1.000	197	0							
Locus_13157_Transcript_1/1_Conf_1.000	135	20	1.24E-12	XP_002643051.1	88	76.2554	45	40	C. briggsae CBR-DPF-6 protein
Locus_13158_Transcript_1/1_Conf_1.000	168	0							

Locus_13159_Transcript_1/1_Conf_1.000	581	20	1.34E-77	NP_501968.2	83	292.738	193	161	Flavin-containing MonoOxygenase family member (fmo-1)
Locus_1316_Transcript_1/1_Conf_1.000	1659	20	0	CAD29445.1	90	844.729	488	440	protein disulfide isomerase 1
Locus_13160_Transcript_1/1_Conf_1.000	151	0							
Locus_13161_Transcript_1/1_Conf_1.000	533	2	3.56E-17	XP_002632168.1	64	91.6633	127	82	C. briggsae CBR-PQN-85 protein
Locus_13162_Transcript_1/1_Conf_1.000	369	20	2.47E-16	XP_001626861.1	67	88.5817	87	59	predicted protein
Locus_13163_Transcript_1/2_Conf_1.000	507	20	1.57E-21	XP_002926282.1	61	105.916	131	81	PREDICTED: poly(ADP-ribose) glycohydrolase-like
Locus_13163_Transcript_2/2_Conf_1.000	507	20	5.39E-22	XP_002926282.1	61	107.457	131	81	PREDICTED: poly(ADP-ribose) glycohydrolase-like
Locus_13164_Transcript_1/1_Conf_1.000	146	9	6.55E-17	XP_002805525.1	100	90.5077	42	42	PREDICTED: hypothetical protein LOC100426852
Locus_13165_Transcript_1/1_Conf_1.000	399	0							
Locus_13166_Transcript_1/1_Conf_1.000	472	20	7.26E-45	XP_002645831.1	72	183.341	162	117	C. briggsae CBR-RBC-1 protein
Locus_13167_Transcript_1/1_Conf_1.000	412	0							
Locus_13168_Transcript_1/1_Conf_1.000	195	1	2.08E-15	EAW79999.1	97	85.5001	40	39	MYC-associated zinc finger protein (purine-binding transcription factor), isoform CRA_f
Locus_13169_Transcript_1/1_Conf_1.000	539	0							
Locus_1317_Transcript_1/2_Conf_1.000	3302	20	0	NP_507869.1	68	815.839	899	615	UNCoordinated family member (unc-51)

Locus_1317_Transcript_2/2_Conf_1.000	3296	20	0	NP_507869.1	66	815.068	917	612	UNCoordinated family member (unc-51)
Locus_13170_Transcript_1/1_Conf_1.000	475	0							
Locus_13171_Transcript_1/1_Conf_1.000	438	0							
Locus_13172_Transcript_1/1_Conf_1.000	320	20	2.63E-18	XP_002635663.1	72	95.1301	79	57	Hypothetical protein CBG21858
Locus_13173_Transcript_1/1_Conf_1.000	823	20	1.19E-99	XP_002643926.1	87	367.081	244	214	C. briggsae CBR-SFXN-1.5 protein
Locus_13174_Transcript_1/1_Conf_1.000	445	0							
Locus_13175_Transcript_1/2_Conf_1.000	537	20	7.99E-41	NP_501090.1	72	170.244	166	121	hypothetical protein C06G3.9
Locus_13175_Transcript_2/2_Conf_1.000	537	20	3.04E-40	NP_501090.1	71	168.318	166	119	hypothetical protein C06G3.9
Locus_13176_Transcript_1/1_Conf_1.000	686	0							
Locus_13177_Transcript_1/1_Conf_1.000	390	20	6.95E-32	NP_501302.1	78	140.198	125	98	MVB (yeast MultiVesicular Body sorting factor) related family member (mvb-12)

Locus_13178_Transcript_1/1_Conf_1.000	1520	20	2.12E-84	XP_002636129.1	61	317.775	396	243	C. briggsae CBR-ATGR-9 protein
Locus_13179_Transcript_1/1_Conf_1.000	199	0							
Locus_1318_Transcript_1/1_Conf_1.000	553	20	1.31E-44	NP_741320.1	65	182.956	183	119	SET (trithorax/polycomb) domain containing family member (set-23)
Locus_13180_Transcript_1/1_Conf_1.000	843	20	6.24E-75	XP_002639128.1	78	285.034	223	174	C. briggsae CBR-SPG-7 protein
Locus_13181_Transcript_1/1_Conf_1.000	866	20	1.28E-62	XP_002637088.1	67	244.202	248	168	Hypothetical protein CBG09587
Locus_13182_Transcript_1/1_Conf_1.000	721	20	1.81E-79	NP_508983.2	75	299.671	253	192	CTBP (CtBP) transcriptional co-repressor homolog family member (ctbp-1)
Locus_13183_Transcript_1/1_Conf_1.000	267	0							
Locus_13184_Transcript_1/1_Conf_1.000	333	0							
Locus_13185_Transcript_1/1_Conf_1.000	425	0							
Locus_13186_Transcript_1/1_Conf_1.000	196	0							
Locus_13187_Transcript_1/1_Conf_1.000	807	0							
Locus_13188_Transcript_1/1_Conf_1.000	344	4	1.83E-11	NP_504395.2	56	72.4034	115	65	hypothetical protein Y47A7.1
Locus_13189_Transcript_1/2_Conf_1.000	280	2	1.24E-04	EFO19653.1	80	49.6766	41	33	pre-B cell enhancing factor
Locus_13189_Transcript_2/2_Conf_1.000	222	0							
Locus_1319_Transcript_1/2_Conf_1.000	999	20	6.04E-142	XP_002630817.1	88	508.064	311	276	C. briggsae CBR-LEC-5 protein
Locus_1319_Transcript_2/2_Conf_1.000	1023	20	1.27E-118	XP_002630817.1	79	430.639	319	254	C. briggsae CBR-LEC-5 protein
Locus_13190_Transcript_1/1_Conf_1.000	170	0							
Locus_13191_Transcript_1/1_Conf_1.000	217	0							
Locus_13192_Transcript_1/1_Conf_1.000	318	0							
Locus_13193_Transcript_1/1_Conf_1.000	269	20	1.25E-20	NP_001129924.1	80	102.834	89	72	SLOwpoke potassium channel family member (slo-2)
Locus_13194_Transcript_1/1_Conf_1.000	995	5	6.55E-56	EFO22136.1	64	222.246	273	176	hypothetical protein LOAG_06352
Locus_13195_Transcript_1/1_Conf_1.000	286	0							
Locus_13196_Transcript_1/1_Conf_1.000	332	0							

Locus_13197_Transcript_1/1_Conf_1.000	306	4	7.04E-08	XP_002637153.1	81	60.4622	37	30	Hypothetical protein CBG09655
Locus_13198_Transcript_1/1_Conf_1.000	220	20	3.03E-22	XP_002630183.1	80	108.227	73	59	C. briggsae CBR-FLI-1 protein
Locus_13199_Transcript_1/1_Conf_1.000	520	2	8.34E-05	XP_002644141.1	56	50.447	65	37	C. briggsae CBR-FLP-19 protein
Locus_132_Transcript_1/3_Conf_0.600	418	20	1.03E-30	XP_002635598.1	80	136.346	130	105	C. briggsae CBR-RPS-30 protein
Locus_132_Transcript_2/3_Conf_0.600	478	20	1.01E-30	XP_002635598.1	80	136.346	130	105	C. briggsae CBR-RPS-30 protein
Locus_132_Transcript_3/3_Conf_0.600	487	20	1.02E-30	XP_002635598.1	80	136.346	130	105	C. briggsae CBR-RPS-30 protein
Locus_1320_Transcript_1/1_Conf_1.000	360	4	2.50E-13	NP_504235.2	64	78.5666	117	75	Drosophila SOS homolog family member (sos-1)
Locus_13200_Transcript_1/1_Conf_1.000	247	0							
Locus_13201_Transcript_1/1_Conf_1.000	555	4	3.52E-37	NP_493486.2	83	158.303	130	108	hypothetical protein C49A1.10
Locus_13202_Transcript_1/1_Conf_1.000	345	0							
Locus_13203_Transcript_1/1_Conf_1.000	221	0							
Locus_13204_Transcript_1/1_Conf_1.000	451	6	6.85E-11	XP_002632899.1	79	70.4774	54	43	C. briggsae CBR-DAF-15 protein
Locus_13205_Transcript_1/2_Conf_1.000	231	20	2.07E-15	XP_001898420.1	82	85.5001	64	53	ATP-dependent DNA helicase, RecQ family protein
Locus_13205_Transcript_2/2_Conf_1.000	236	20	7.85E-15	XP_001898420.1	82	83.5741	63	52	ATP-dependent DNA helicase, RecQ family protein
Locus_13206_Transcript_1/1_Conf_1.000	218	0							
Locus_13207_Transcript_1/1_Conf_1.000	423	0							
Locus_13208_Transcript_1/1_Conf_1.000	616	20	2.70E-37	EFO15564.1	60	159.073	202	123	hypothetical protein LOAG_12944

Locus_13209_Transcript_1/1_Conf_1.000	358	20	2.76E-28	NP_505501.1	72	128.257	119	86	Temporarily Assigned Gene name family member (tag-120)
Locus_1321_Transcript_1/2_Conf_1.000	1618	20	6.21E-98	NP_500797.1	81	362.844	246	200	fatty acid ELOngation family member (elo-6)
Locus_1321_Transcript_2/2_Conf_1.000	1618	20	6.21E-98	NP_500797.1	81	362.844	246	200	fatty acid ELOngation family member (elo-6)
Locus_13210_Transcript_1/1_Conf_1.000	362	20	1.32E-38	NP_497625.3	87	162.54	101	88	hypothetical protein Y71H2AM.1
Locus_13211_Transcript_1/1_Conf_1.000	236	0							
Locus_13212_Transcript_1/1_Conf_1.000	201	0							
Locus_13213_Transcript_1/1_Conf_1.000	178	2	5.03E-06	XP_002639575.1	65	54.299	60	39	C. briggsae CBR-BAM-2 protein
Locus_13214_Transcript_1/1_Conf_1.000	663	20	1.86E-53	NP_001024840.1	74	213.001	213	159	hypothetical protein R07E4.1
Locus_13215_Transcript_1/1_Conf_1.000	606	20	1.43E-11	XP_002632653.1	46	73.559	188	88	Hypothetical protein CBG21575
Locus_13216_Transcript_1/1_Conf_1.000	418	0							
Locus_13217_Transcript_1/1_Conf_1.000	563	0							
Locus_13218_Transcript_1/1_Conf_1.000	320	0							
Locus_13219_Transcript_1/1_Conf_1.000	166	0							
Locus_1322_Transcript_1/1_Conf_1.000	1273	20	2.72E-95	NP_504655.1	91	353.599	202	184	DumPY : shorter than wild-type family member (dpy-11)
Locus_13220_Transcript_1/1_Conf_1.000	333	0							
Locus_13221_Transcript_1/1_Conf_1.000	400	20	2.90E-09	XP_002720026.1	53	65.0846	94	50	PREDICTED: heterochromatin protein 1-beta-like
Locus_13222_Transcript_1/1_Conf_1.000	369	0							
Locus_13223_Transcript_1/2_Conf_1.000	497	20	1.22E-68	ACY40004.1	87	262.307	162	142	K+/cl- cotransporter protein 2, isoform c

Locus_13223_Transcript_2/2_Conf_1.000	497	20	1.22E-68	ACY40004.1	87	262.307	162	142	K+/cl- cotransporter protein 2, isoform c
Locus_13224_Transcript_1/1_Conf_1.000	236	3	1.87E-08	NP_001024264.1	58	62.3882	77	45	hypothetical protein Y58G8A.4
Locus_13225_Transcript_1/1_Conf_1.000	303	0							
Locus_13226_Transcript_1/1_Conf_1.000	276	0							
Locus_13227_Transcript_1/1_Conf_1.000	377	20	2.37E-19	EFO22101.1	68	98.5969	95	65	hypothetical protein LOAG_06386
Locus_13228_Transcript_1/1_Conf_1.000	175	0							
Locus_13229_Transcript_1/1_Conf_1.000	352	20	5.64E-13	EFO19207.1	78	77.411	60	47	hypothetical protein LOAG_09288
Locus_1323_Transcript_1/2_Conf_1.000	261	20	5.83E-26	EFO20001.1	80	120.553	86	69	Sel1l protein
Locus_1323_Transcript_2/2_Conf_1.000	261	20	5.83E-26	EFO20001.1	80	120.553	86	69	Sel1l protein
Locus_13230_Transcript_1/1_Conf_1.000	304	2	7.26E-29	NP_508948.2	80	130.183	100	80	hypothetical protein T21F4.1
Locus_13231_Transcript_1/1_Conf_1.000	418	20	4.21E-16	XP_001893741.1	60	87.8113	110	67	LIM domain containing protein
Locus_13232_Transcript_1/1_Conf_1.000	305	4	7.03E-24	XP_002636973.1	68	113.62	101	69	Hypothetical protein CBG09454
Locus_13233_Transcript_1/1_Conf_1.000	178	0							
Locus_13234_Transcript_1/1_Conf_1.000	248	0							

Locus_13235_Transcript_1/1_Conf_1.000	542	20	1.52E-79	XP_002630154.1	95	298.901	160	153	C. briggsae CBR-ARL-1 protein
Locus_13236_Transcript_1/1_Conf_1.000	1407	20	4.01E-42	XP_002632130.1	75	177.178	185	140	C. briggsae CBR-COL-68 protein
Locus_13237_Transcript_1/1_Conf_1.000	413	20	4.99E-38	NP_491552.2	87	160.614	137	120	LEThal family member (let-363)
Locus_13238_Transcript_1/1_Conf_1.000	349	20	6.85E-43	XP_002760231.1	98	176.792	114	112	PREDICTED: prefoldin subunit 2-like
Locus_13239_Transcript_1/1_Conf_1.000	155	2	3.34E-05	NP_498595.1	66	51.6026	53	35	hypothetical protein T20H4.5
Locus_1324_Transcript_1/1_Conf_1.000	370	0							
Locus_13240_Transcript_1/1_Conf_1.000	134	0							
Locus_13241_Transcript_1/1_Conf_1.000	483	20	3.44E-18	XP_002639738.1	63	94.7449	101	64	Hypothetical protein CBG12466
Locus_13242_Transcript_1/1_Conf_1.000	391	20	6.31E-25	XP_002639033.1	77	117.087	93	72	Hypothetical protein CBG22282
Locus_13243_Transcript_1/1_Conf_1.000	748	4	5.58E-42	XP_001892872.1	71	175.252	170	121	hypothetical protein Bm1_07015
Locus_13244_Transcript_1/1_Conf_1.000	265	7	2.22E-09	XP_001901023.1	95	65.4698	40	38	hypothetical protein Bm1_47805
Locus_13245_Transcript_1/1_Conf_1.000	575	20	6.01E-83	NP_493544.2	90	310.457	181	164	hypothetical protein Y105E8B.3

Locus_13246_Transcript_1/2_Conf_1.000	558	10	3.30E-11	NP_499177.3	41	72.0182	198	82	hypothetical protein ZK632.7
Locus_13246_Transcript_2/2_Conf_1.000	522	14	9.41E-12	NP_499177.3	40	73.559	202	82	hypothetical protein ZK632.7
Locus_13247_Transcript_1/1_Conf_1.000	178	0							
Locus_13248_Transcript_1/1_Conf_1.000	424	1	2.15E-04	AAK18978.3	53	48.9062	125	67	Hypothetical protein T20F7.1
Locus_13249_Transcript_1/1_Conf_1.000	728	20	5.90E-94	AAV70493.1	82	347.821	230	190	acetylcholine/choline transporter
Locus_1325_Transcript_1/1_Conf_1.000	679	20	6.08E-63	XP_002631415.1	70	244.588	217	154	Hypothetical protein CBG03266
Locus_13250_Transcript_1/2_Conf_1.000	1727	20	0	XP_002640216.1	81	750.74	529	433	Hypothetical protein CBG12727
Locus_13250_Transcript_2/2_Conf_1.000	1023	20	1.19E-140	XP_002640216.1	83	503.827	323	271	Hypothetical protein CBG12727
Locus_13251_Transcript_1/1_Conf_1.000	401	0							
Locus_13252_Transcript_1/1_Conf_1.000	442	0							
Locus_13253_Transcript_1/1_Conf_1.000	185	0							
Locus_13254_Transcript_1/1_Conf_1.000	255	3	3.82E-09	XP_002640308.1	75	64.6994	57	43	Hypothetical protein CBG12847

Locus_13255_Transcript_1/2_Conf_1.000	251	20	1.55E-23	XP_001901686.1	83	112.464	83	69	Cohesin subunit SA-1
Locus_13255_Transcript_2/2_Conf_1.000	251	20	1.55E-23	XP_001901686.1	83	112.464	83	69	Cohesin subunit SA-1
Locus_13256_Transcript_1/1_Conf_1.000	325	0							
Locus_13257_Transcript_1/1_Conf_1.000	316	0							

Locus_13258_Transcript_1/1_Conf_1.000	690	20	7.06E-38	EFO24692.1	65	161.384	164	108	protein-tyrosine phosphatase
Locus_13259_Transcript_1/1_Conf_1.000	1507	20	3.66E-113	XP_002630297.1	72	413.305	410	299	C. briggsae CBR-PCH-2 protein
Locus_1326_Transcript_1/1_Conf_1.000	1316	20	1.64E-50	NP_499287.2	57	204.912	254	147	T BoX family member (tbx-8)
Locus_13260_Transcript_1/1_Conf_1.000	2594	3	7.10E-04	NP_001032352.2	51	51.2174	105	54	tripartite motif-containing protein 71
Locus_13261_Transcript_1/1_Conf_1.000	2647	12	7.76E-14	CAR63553.1	43	84.3445	252	110	putative major allergen
Locus_13262_Transcript_1/1_Conf_1.000	167	0							
Locus_13263_Transcript_1/1_Conf_1.000	482	20	3.83E-14	XP_001902194.1	57	81.2629	138	79	3'-5' exoribonuclease CSL4 homolog
Locus_13264_Transcript_1/1_Conf_1.000	441	20	1.49E-29	NP_497626.1	65	132.494	125	82	hypothetical protein Y71H2AR.1
Locus_13265_Transcript_1/1_Conf_1.000	746	0							
Locus_13266_Transcript_1/1_Conf_1.000	271	20	6.15E-36	CAR63585.1	95	153.68	88	84	putative MAD-Like family member
Locus_13267_Transcript_1/1_Conf_1.000	315	0							
Locus_13268_Transcript_1/1_Conf_1.000	311	5	1.40E-40	NP_495954.1	93	169.088	103	96	Regulator of HYpoxia-inducible factor (hif-1) family member (rhy-1)

Locus_13269_Transcript_1/1_Conf_1.000	875	20	5.87E-55	NP_498653.2	56	218.779	298	169	LIGase family member (lig-4)
Locus_1327_Transcript_1/1_Conf_1.000	1858	20	1.51E-50	EFO27065.1	54	205.682	362	196	hypothetical protein LOAG_01420
Locus_13270_Transcript_1/1_Conf_1.000	1352	20	6.71E-47	NP_499810.2	52	192.971	437	228	hypothetical protein T25C8.1
Locus_13271_Transcript_1/1_Conf_1.000	361	1	3.04E-27	NP_493572.2	68	124.79	118	81	hypothetical protein F39B2.8
Locus_13272_Transcript_1/1_Conf_1.000	849	20	2.30E-109	XP_002646355.1	81	399.438	283	230	Hypothetical protein CBG12069
Locus_13273_Transcript_1/1_Conf_1.000	306	0							
Locus_13274_Transcript_1/1_Conf_1.000	231	8	3.10E-11	AAT72456.1	72	71.633	59	43	FOG-3
Locus_13275_Transcript_1/1_Conf_1.000	727	2	2.61E-25	XP_002636407.1	63	119.783	157	99	Hypothetical protein CBG23059
Locus_13276_Transcript_1/1_Conf_1.000	303	20	3.49E-23	EFO20885.1	80	111.309	76	61	hypothetical protein LOAG_07603
Locus_13277_Transcript_1/1_Conf_1.000	247	0							
Locus_13278_Transcript_1/1_Conf_1.000	197	0							
Locus_13279_Transcript_1/1_Conf_1.000	1020	0							
Locus_1328_Transcript_1/2_Conf_0.500	1269	20	0	NP_509217.2	89	652.129	381	341	hypothetical protein F46H5.3
Locus_1328_Transcript_2/2_Conf_0.500	1269	20	0	NP_509217.2	89	649.047	381	340	hypothetical protein F46H5.3
Locus_13280_Transcript_1/1_Conf_1.000	233	10	1.88E-16	XP_002642915.1	68	88.9669	76	52	Hypothetical protein CBG15190
Locus_13281_Transcript_1/1_Conf_1.000	350	6	1.75E-22	XP_002636985.1	77	108.997	105	81	Hypothetical protein CBG09473
Locus_13282_Transcript_1/1_Conf_1.000	536	20	9.77E-84	NP_000230.1	100	312.768	148	148	lysozyme C precursor
Locus_13283_Transcript_1/1_Conf_1.000	142	9	7.98E-12	XP_002647278.1	82	73.559	46	38	C. briggsae CBR-KETN-1 protein

Locus_13284_Transcript_1/1_Conf_1.000	508	20	9.74E-56	NP_496100.1	79	219.55	173	137	hypothetical protein Y53C12B.1
Locus_13285_Transcript_1/1_Conf_1.000	564	20	9.16E-65	AAP36795.1	100	249.98	128	128	Homo sapiens CD59 antigen p18-20 (antigen identified by monoclonal antibodies 16.3A5, EJ16, EJ30, EL32 and G344)
Locus_13286_Transcript_1/1_Conf_1.000	588	12	1.14E-39	XP_002638395.1	58	166.777	201	117	Hypothetical protein CBG18604
Locus_13287_Transcript_1/2_Conf_1.000	1848	20	0	XP_001896443.1	79	755.747	568	451	DNA topoisomerase III beta-1
Locus_13287_Transcript_2/2_Conf_1.000	1848	20	0	XP_001896443.1	79	754.977	568	450	DNA topoisomerase III beta-1
Locus_13288_Transcript_1/1_Conf_1.000	156	20	1.31E-09	AAK31527.3	80	66.2402	52	42	Neuronal symmetry protein 1, partially confirmed by transcript evidence
Locus_13289_Transcript_1/1_Conf_1.000	488								
Locus_1329_Transcript_1/2_Conf_1.000	458	20	1.08E-24	AAX30301.1	96	116.316	55	53	unknown
Locus_1329_Transcript_2/2_Conf_1.000	469	20	1.86E-32	BAA10929.1	74	142.124	120	89	cytochrome P450 like_TBP
Locus_13290_Transcript_1/1_Conf_1.000	137	0							
Locus_13291_Transcript_1/1_Conf_1.000	146	20	1.56E-10	XP_002640635.1	76	69.3218	46	35	Hypothetical protein CBG08753
Locus_13292_Transcript_1/1_Conf_1.000	832	20	1.76E-82	NP_498112.1	74	310.071	241	179	PRotein arginine MethylTransferase family member (prmt-5)
Locus_13293_Transcript_1/1_Conf_1.000	460	20	1.31E-22	EFO19933.1	63	109.383	138	87	helix-loop-helix DNA-binding domain-containing protein
Locus_13294_Transcript_1/1_Conf_1.000	152	0							
Locus_13295_Transcript_1/1_Conf_1.000	315	0							
Locus_13296_Transcript_1/1_Conf_1.000	822	20	8.96E-39	NP_501996.2	54	164.851	282	155	UDP-GlucuronosylTransferase family member (ugt-54)

Locus_13297_Transcript_1/1_Conf_1.000	632	20	2.85E-21	CAR97823.1	54	105.916	166	91	C. elegans protein F26H11.2i, partially confirmed by transcript evidence
Locus_13298_Transcript_1/1_Conf_1.000	463	20	2.35E-59	XP_002634350.1	85	231.491	131	112	C. briggsae CBR-ILYS-4 protein
Locus_13299_Transcript_1/1_Conf_1.000	392	0							
Locus_133_Transcript_1/1_Conf_1.000	1211	20	3.47E-52	NP_493028.2	59	210.305	373	222	WAVE (actin cytoskeleton modulator) homolog family member (wve-1)
Locus_1330_Transcript_1/1_Conf_1.000	1727	20	0	NP_740787.2	78	707.597	556	438	hypothetical protein C53H9.2
Locus_13300_Transcript_1/1_Conf_1.000	624	0							
Locus_13301_Transcript_1/1_Conf_1.000	610	20	1.64E-55	NP_741176.2	67	219.55	210	141	hypothetical protein C23G10.7
Locus_13302_Transcript_1/1_Conf_1.000	185	0							
Locus_13303_Transcript_1/1_Conf_1.000	885	20	2.23E-110	NP_496279.1	84	402.905	266	224	Temporarily Assigned Gene name family member (tag-242)
Locus_13304_Transcript_1/1_Conf_1.000	815	20	7.38E-54	CAA92456.3	71	214.927	203	146	C. elegans protein C53B4.4b, partially confirmed by transcript evidence
Locus_13305_Transcript_1/1_Conf_1.000	484	20	9.99E-31	NP_490911.1	68	130.183	150	103	SCAMP (synaptic vesicle protein) homolog family member (scm-1)
Locus_13306_Transcript_1/1_Conf_1.000	153	0							
Locus_13307_Transcript_1/1_Conf_1.000	140	0							
Locus_13308_Transcript_1/1_Conf_1.000	177	0							
Locus_13309_Transcript_1/1_Conf_1.000	205	0							
Locus_1331_Transcript_1/1_Conf_1.000	811	20	3.27E-78	NP_509260.1	71	295.819	248	178	ACyLtransferase-like family member (acl-12)

Locus_13310_Transcript_1/1_Conf_1.000	389	20	7.21E-37	EFO23898.1	78	156.762	120	94	FolC protein
Locus_13311_Transcript_1/1_Conf_1.000	1583	20	9.77E-40	NP_498165.2	46	169.474	458	213	hypothetical protein F48E8.2
Locus_13312_Transcript_1/2_Conf_1.000	221	0							
Locus_13312_Transcript_2/2_Conf_1.000	173	0							
Locus_13313_Transcript_1/1_Conf_1.000	735	20	2.32E-61	NP_509122.1	70	239.58	244	173	hypothetical protein F38B6.4
Locus_13314_Transcript_1/1_Conf_1.000	241	7	3.37E-26	XP_002631168.1	84	121.324	70	59	C. briggsae CBR-PQM-1 protein
Locus_13315_Transcript_1/1_Conf_1.000	413	20	1.61E-36	NP_501320.2	74	155.606	135	100	eXPOrtin (nuclear export receptor) family member (xpo-3)
Locus_13316_Transcript_1/1_Conf_1.000	362	20	2.85E-17	AAO63576.1	59	91.6633	111	66	secreted protein 4 precursor
Locus_13317_Transcript_1/1_Conf_1.000	203	20	8.11E-12	NP_001021562.1	67	73.559	74	50	hypothetical protein K07A3.3
Locus_13318_Transcript_1/1_Conf_1.000	384	0							
Locus_13319_Transcript_1/1_Conf_1.000	572	0							
Locus_1332_Transcript_1/1_Conf_1.000	622	15	4.24E-06	XP_002637664.1	47	55.4546	143	68	Hypothetical protein CBG19420
Locus_13320_Transcript_1/1_Conf_1.000	279	0							
Locus_13321_Transcript_1/1_Conf_1.000	633	6	9.27E-20	NP_510700.3	57	100.908	227	131	Histone DeAcetylase family member (hda-4)
Locus_13322_Transcript_1/1_Conf_1.000	513	20	8.15E-42	XP_001951535.1	70	173.326	172	122	PREDICTED: similar to dynein heavy chain
Locus_13323_Transcript_1/1_Conf_1.000	184	3	3.71E-12	NP_491803.1	87	74.7146	48	42	SaPosin-like Protein family member (spp-15)
Locus_13324_Transcript_1/1_Conf_1.000	472	3	2.34E-35	NP_501123.1	74	151.754	157	117	COLlagen family member (col-113)
Locus_13325_Transcript_1/1_Conf_1.000	646	4	2.63E-41	XP_001897455.1	80	172.555	123	99	hypothetical protein
Locus_13326_Transcript_1/1_Conf_1.000	170	0							
Locus_13327_Transcript_1/2_Conf_1.000	1263	2	2.33E-22	NP_499581.2	54	111.309	211	116	hypothetical protein Y75B8A.8
Locus_13327_Transcript_2/2_Conf_1.000	1263	2	2.33E-22	NP_499581.2	54	111.309	211	116	hypothetical protein Y75B8A.8
Locus_13328_Transcript_1/1_Conf_1.000	185	0							

Locus_13329_Transcript_1/1_Conf_1.000	293	3	9.66E-13	CAA92756.3	75	76.6406	62	47	C. elegans protein T24B8.4, partially confirmed by transcript evidence
Locus_1333_Transcript_1/3_Conf_0.714	1891	20	0	XP_002640606.1	94	917.916	529	499	Hypothetical protein CBG08717
Locus_1333_Transcript_2/3_Conf_0.714	1891	20	0	XP_002640606.1	94	917.916	529	499	Hypothetical protein CBG08717
Locus_1333_Transcript_3/3_Conf_0.714	1891	20	0	XP_002640606.1	94	917.916	529	499	Hypothetical protein CBG08717
Locus_13330_Transcript_1/1_Conf_1.000	343	0							
Locus_13331_Transcript_1/1_Conf_1.000	547	2	1.15E-13	NP_001033437.1	60	80.1073	87	53	hypothetical protein W09G12.10
Locus_13332_Transcript_1/1_Conf_1.000	158	0							
Locus_13333_Transcript_1/1_Conf_1.000	129	0							

Locus_13334_Transcript_1/1_Conf_1.000	239	20	5.95E-39	BAG65521.1	100	163.696	79	79	unnamed protein product
Locus_13335_Transcript_1/1_Conf_1.000	153	0							
Locus_13336_Transcript_1/1_Conf_1.000	363	4	1.56E-23	XP_002631126.1	81	112.464	125	102	C. briggsae CBR-SHN-1 protein
Locus_13337_Transcript_1/1_Conf_1.000	164	0							
Locus_13338_Transcript_1/1_Conf_1.000	1913	20	8.31E-177	NP_491489.1	69	625.165	613	425	AminoPeptidase P family member (app-1)
Locus_13339_Transcript_1/1_Conf_1.000	136	0							
Locus_1334_Transcript_1/1_Conf_1.000	1090	20	3.95E-105	XP_002629729.1	71	385.956	325	231	C. briggsae CBR-TAG-124 protein
Locus_13340_Transcript_1/1_Conf_1.000	353	0							
Locus_13341_Transcript_1/1_Conf_1.000	1304	20	1.16E-157	NP_506859.2	79	560.836	437	347	hypothetical protein C06B8.7
Locus_13342_Transcript_1/1_Conf_1.000	606	2	6.63E-17	NP_500233.1	58	91.2781	110	64	hypothetical protein Y69A2AR.28
Locus_13343_Transcript_1/1_Conf_1.000	289	2	1.36E-06	XP_001894940.1	51	56.225	95	49	Trypsin family protein
Locus_13344_Transcript_1/1_Conf_1.000	139	0							
Locus_13345_Transcript_1/1_Conf_1.000	240	0							
Locus_13346_Transcript_1/2_Conf_1.000	501	6	5.29E-35	NP_495914.1	57	150.599	167	96	hypothetical protein C18E9.8
Locus_13346_Transcript_2/2_Conf_1.000	501	6	5.29E-35	NP_495914.1	57	150.599	167	96	hypothetical protein C18E9.8
Locus_13347_Transcript_1/1_Conf_1.000	174	0							

Locus_13348_Transcript_1/1_Conf_1.000	1009	20	5.79E-108	NP_497663.1	80	395.201	304	245	MEChanosensory abnormality family member (mec-12)
Locus_13349_Transcript_1/1_Conf_1.000	555	0							
Locus_1335_Transcript_1/1_Conf_1.000	570	20	9.13E-76	ACD88888.1	97	286.574	149	145	troponin C
Locus_13350_Transcript_1/1_Conf_1.000	145	0							
Locus_13351_Transcript_1/1_Conf_1.000	437	0							
Locus_13352_Transcript_1/2_Conf_1.000	233	20	7.88E-15	ZP_04113472.1	64	83.5741	76	49	LPXTG-motif cell wall anchor domain protein
Locus_13352_Transcript_2/2_Conf_1.000	676	20	6.09E-23	ZP_04113472.1	53	111.694	130	69	LPXTG-motif cell wall anchor domain protein
Locus_13353_Transcript_1/1_Conf_1.000	985	20	7.47E-121	NP_494897.3	75	437.958	328	248	hypothetical protein R05F9.12
Locus_13354_Transcript_1/1_Conf_1.000	377	0							
Locus_13355_Transcript_1/1_Conf_1.000	299	0							
Locus_13356_Transcript_1/1_Conf_1.000	228	0							
Locus_13357_Transcript_1/1_Conf_1.000	250	0							
Locus_13358_Transcript_1/4_Conf_0.375	367	20	6.44E-62	EFO26582.1	100	239.965	122	122	TATA-box binding protein
Locus_13358_Transcript_2/4_Conf_0.625	925	20	3.50E-93	XP_002642588.1	73	345.895	320	235	C. briggsae CBR-TBP-1 protein

Locus_13358_Transcript_3/4_Conf_0.625	925	20	3.50E-93	XP_002642588.1	73	345.895	320	235	C. briggsae CBR-TBP-1 protein
Locus_13358_Transcript_4/4_Conf_0.625	925	20	3.50E-93	XP_002642588.1	73	345.895	320	235	C. briggsae CBR-TBP-1 protein
Locus_13359_Transcript_1/1_Conf_1.000	154	4	9.08E-11	EFO21130.1	88	70.0922	42	37	hypothetical protein LOAG_07358
Locus_1336_Transcript_1/1_Conf_1.000	190	0							
Locus_13360_Transcript_1/1_Conf_1.000	219	0							
Locus_13361_Transcript_1/1_Conf_1.000	639	0							
Locus_13362_Transcript_1/1_Conf_1.000	717	20	1.29E-45	EFO23663.1	63	187.193	228	144	myosin xviiia
Locus_13363_Transcript_1/1_Conf_1.000	221	0							
Locus_13364_Transcript_1/1_Conf_1.000	380	20	9.53E-37	NP_492244.1	78	156.377	113	89	hypothetical protein ZK265.3
Locus_13365_Transcript_1/1_Conf_1.000	254	20	3.12E-27	NP_495261.1	83	124.79	80	67	hypothetical protein F13H8.7
Locus_13366_Transcript_1/1_Conf_1.000	161	16	3.20E-16	AAS49411.1	86	88.1965	51	44	putative neuromuscular acetylcholinesterase
Locus_13367_Transcript_1/1_Conf_1.000	325	6	1.52E-34	NP_492775.2	84	149.058	105	89	LAMinin related. See also lmb- family member (lam-3)
Locus_13368_Transcript_1/1_Conf_1.000	132	0							
Locus_13369_Transcript_1/1_Conf_1.000	785	20	3.74E-108	XP_002631013.1	88	395.201	260	230	C. briggsae CBR-PTR-18 protein
Locus_1337_Transcript_1/3_Conf_0.667	921	0							
Locus_1337_Transcript_2/3_Conf_0.667	967	0							
Locus_1337_Transcript_3/3_Conf_0.667	969	0							

Locus_13370_Transcript_1/1_Conf_1.000	451	20	5.38E-40	NP_496146.1	68	167.162	149	102	Ubiquitin Fusion Degradation (yeast UFD homolog) family member (ufd-3)
Locus_13371_Transcript_1/1_Conf_1.000	204	0							
Locus_13372_Transcript_1/1_Conf_1.000	395	20	1.54E-34	XP_002644371.1	72	149.058	129	94	C. briggsae CBR-DHS-27 protein
Locus_13373_Transcript_1/1_Conf_1.000	477	20	4.99E-38	XP_001898260.1	74	160.614	125	93	MSP domain containing protein
Locus_13374_Transcript_1/1_Conf_1.000	281	20	1.54E-47	BAG51482.1	100	192.2	88	88	unnamed protein product
Locus_13375_Transcript_1/1_Conf_1.000	730	20	1.87E-63	NP_510828.1	93	246.514	152	142	Myosin Light Chain family member (mlc-2)
Locus_13376_Transcript_1/1_Conf_1.000	271	0							
Locus_13377_Transcript_1/1_Conf_1.000	617	3	5.44E-38	XP_002631029.1	71	161.384	151	108	Hypothetical protein CBG02786
Locus_13378_Transcript_1/1_Conf_1.000	888	6	4.31E-45	XP_002641960.1	66	186.037	201	134	C. briggsae CBR-UNC-36 protein
Locus_13379_Transcript_1/1_Conf_1.000	236	2	7.37E-05	EFO22556.1	58	50.447	77	45	hypothetical protein LOAG_05929
Locus_1338_Transcript_1/1_Conf_1.000	535	18	7.08E-42	NP_001040754.1	65	173.711	177	116	DNaJ domain (prokaryotic heat shock protein) family member (dnj-8)
Locus_13380_Transcript_1/1_Conf_1.000	697	0							
Locus_13381_Transcript_1/1_Conf_1.000	348	0							
Locus_13382_Transcript_1/1_Conf_1.000	332	5	2.27E-22	NP_491451.2	64	108.612	109	70	2 (Zwei) IG-domain protein family member (zig-7)
Locus_13383_Transcript_1/1_Conf_1.000	1055	20	1.17E-74	AAK29933.3	67	284.648	308	208	Rab connectin related protein 2, partially confirmed by transcript evidence
Locus_13384_Transcript_1/1_Conf_1.000	379	0							
Locus_13385_Transcript_1/1_Conf_1.000	306	6	1.28E-25	AAT02161.1	80	119.398	76	61	hypothetical protein L3ni22
Locus_13386_Transcript_1/1_Conf_1.000	136	0							
Locus_13387_Transcript_1/1_Conf_1.000	1009	20	1.47E-127	NP_506318.1	81	460.299	301	245	CathePsin Z family member (cpz-2)
Locus_13388_Transcript_1/1_Conf_1.000	523	20	1.63E-64	XP_002631314.1	84	248.825	163	137	C. briggsae CBR-LDH-1 protein
Locus_13389_Transcript_1/1_Conf_1.000	177	0							

Locus_1339_Transcript_1/1_Conf_1.000	612	0							
Locus_13390_Transcript_1/1_Conf_1.000	175	20	2.66E-23	XP_002642408.1	92	111.694	57	53	C. briggsae CBR-NHL-1 protein
Locus_13391_Transcript_1/1_Conf_1.000	247	20	2.10E-28	XP_001900058.1	82	128.642	80	66	Immunoglobulin I-set domain containing protein
Locus_13392_Transcript_1/1_Conf_1.000	170	0							
Locus_13393_Transcript_1/1_Conf_1.000	1188	3	9.40E-95	XP_002639760.1	69	351.673	312	216	Hypothetical protein CBG02206
Locus_13394_Transcript_1/2_Conf_1.000	307	0							
Locus_13394_Transcript_2/2_Conf_1.000	310	0							
Locus_13395_Transcript_1/1_Conf_1.000	484	17	1.60E-07	NP_001133980.1	71	59.3066	45	32	SH2 domain-containing protein 4A
Locus_13396_Transcript_1/1_Conf_1.000	300	20	2.76E-44	EFO23652.1	92	181.415	99	92	vacuolar proton pump
Locus_13397_Transcript_1/1_Conf_1.000	172	0							
Locus_13398_Transcript_1/1_Conf_1.000	481	6	1.70E-62	XP_002636539.1	80	241.891	160	129	Hypothetical protein CBG23226
Locus_13399_Transcript_1/1_Conf_1.000	362	20	4.87E-17	XP_002630266.1	60	90.8929	126	76	Hypothetical protein CBG00693
Locus_134_Transcript_1/1_Conf_1.000	716	4	2.79E-16	NP_001022219.1	50	89.7373	182	92	hypothetical protein F59B10.4
Locus_1340_Transcript_1/5_Conf_0.444	1411	0							
Locus_1340_Transcript_2/5_Conf_0.556	1507	0							
Locus_1340_Transcript_3/5_Conf_0.444	1429	0							
Locus_1340_Transcript_4/5_Conf_0.556	1501	0							
Locus_1340_Transcript_5/5_Conf_0.556	1507	0							
Locus_13400_Transcript_1/1_Conf_1.000	477	3	1.79E-11	NP_494767.1	63	72.4034	112	71	hypothetical protein H20J04.2
Locus_13401_Transcript_1/2_Conf_1.000	847	20	2.05E-17	NP_510460.2	66	93.9745	83	55	MiniBrain Kinase (Drosophila) homolog family member (mbk-1)
Locus_13401_Transcript_2/2_Conf_1.000	771	20	1.73E-17	NP_510460.2	66	93.9745	83	55	MiniBrain Kinase (Drosophila) homolog family member (mbk-1)
Locus_13402_Transcript_1/1_Conf_1.000	450	0							

Locus_13403_Transcript_1/1_Conf_1.000	578	20	2.95E-53	XP_002643681.1	72	211.846	182	132	Hypothetical protein CBG01860
Locus_13404_Transcript_1/1_Conf_1.000	134	0							
Locus_13405_Transcript_1/1_Conf_1.000	967	20	5.27E-63	XP_002639862.1	63	245.743	320	204	C. briggsae CBR-CED-12 protein
Locus_13406_Transcript_1/1_Conf_1.000	208	0							
Locus_13407_Transcript_1/1_Conf_1.000	1122	8	3.04E-31	CAR63527.1	94	140.584	108	102	putative TolA protein
Locus_13408_Transcript_1/1_Conf_1.000	256	0							
Locus_13409_Transcript_1/1_Conf_1.000	607	20	2.74E-71	XP_002633090.1	95	271.937	164	156	C. briggsae CBR-CYN-12 protein
Locus_1341_Transcript_1/1_Conf_1.000	285	20	4.08E-19	XP_001898944.1	67	97.8265	94	63	Protein kinase domain containing protein
Locus_13410_Transcript_1/1_Conf_1.000	641	2	1.92E-04	DAA21469.1	36	50.0618	158	57	complement factor H precursor
Locus_13411_Transcript_1/1_Conf_1.000	186	0							

Locus_13412_Transcript_1/2_Conf_1.000	372	20	1.07E-19	NP_506362.1	64	99.7525	99	64	BIR (baculovirus inhibitory repeat) family member (bir-2)
Locus_13412_Transcript_2/2_Conf_1.000	644	20	3.31E-20	NP_506362.1	60	102.449	128	77	BIR (baculovirus inhibitory repeat) family member (bir-2)
Locus_13413_Transcript_1/1_Conf_1.000	251	0							
Locus_13414_Transcript_1/2_Conf_1.000	299	2	7.87E-07	XP_002647590.1	61	56.9954	77	47	Hypothetical protein CBG06678
Locus_13414_Transcript_2/2_Conf_1.000	367	2	1.32E-06	XP_002647590.1	61	56.225	77	47	Hypothetical protein CBG06678

Locus_13415_Transcript_1/1_Conf_1.000	643	20	1.46E-76	CAA21686.2	81	289.656	216	177	C. elegans protein Y54E2A.4, partially confirmed by transcript evidence
Locus_13416_Transcript_1/1_Conf_1.000	830	8	1.32E-13	EFO16199.1	50	81.2629	175	89	acetyltransferase
Locus_13417_Transcript_1/1_Conf_1.000	378	0							
Locus_13418_Transcript_1/1_Conf_1.000	250	0							
Locus_13419_Transcript_1/1_Conf_1.000	257	0							
Locus_1342_Transcript_1/1_Conf_1.000	454	20	5.72E-42	O01358.1	93	173.711	125	117	60S ribosomal protein L27a
Locus_13420_Transcript_1/1_Conf_1.000	302	0							
Locus_13421_Transcript_1/1_Conf_1.000	475	17	5.39E-16	ACI49188.1	67	87.4261	97	65	hypothetical protein Csp3_JD02.016
Locus_13422_Transcript_1/1_Conf_1.000	285	0							
Locus_13423_Transcript_1/1_Conf_1.000	196	0							
Locus_13424_Transcript_1/1_Conf_1.000	422	20	3.01E-14	XP_002660885.1	68	81.6481	88	60	PREDICTED: hypothetical protein
Locus_13425_Transcript_1/1_Conf_1.000	1300	20	8.71E-105	AAM18101.1	74	385.185	347	257	AF497823_1putative Na-H exchanger isoform 1
Locus_13426_Transcript_1/1_Conf_1.000	297	20	6.22E-12	ACD12702.1	85	73.9442	48	41	histone H1
Locus_13427_Transcript_1/1_Conf_1.000	331	0							
Locus_13428_Transcript_1/1_Conf_1.000	1601	20	4.84E-103	NP_508710.2	60	379.793	529	319	Multidrug Resistance Protein family member (mrp-6)
Locus_13429_Transcript_1/1_Conf_1.000	163	0							
Locus_1343_Transcript_1/1_Conf_1.000	499	20	2.38E-56	XP_002648101.1	85	221.476	152	130	C. briggsae CBR-DHS-13 protein
Locus_13430_Transcript_1/1_Conf_1.000	388	0							
Locus_13431_Transcript_1/2_Conf_1.000	551	20	4.41E-53	NP_499799.1	77	211.075	180	140	fatty Acid CoA Synthetase family member (acs-14)

Locus_13431_Transcript_2/2_Conf_1.000	534	20	8.92E-53	NP_499799.1	78	209.92	175	138	fatty Acid CoA Synthetase family member (acs-14)
Locus_13432_Transcript_1/1_Conf_1.000	945	3	5.54E-25	CAB03203.2	55	119.398	245	135	C. elegans protein M02B1.4, partially confirmed by transcript evidence
Locus_13433_Transcript_1/1_Conf_1.000	542	15	2.87E-70	XP_002642952.1	82	268.085	180	148	Hypothetical protein CBG15235
Locus_13434_Transcript_1/1_Conf_1.000	128	0							
Locus_13435_Transcript_1/1_Conf_1.000	402	20	2.33E-59	XP_002642823.1	92	231.491	131	121	Hypothetical protein CBG21221
Locus_13436_Transcript_1/1_Conf_1.000	370	0							
Locus_13437_Transcript_1/1_Conf_1.000	429	20	2.33E-43	XP_002639695.1	78	178.333	146	114	C. briggsae CBR-FCP-1 protein
Locus_13438_Transcript_1/1_Conf_1.000	654	20	7.25E-10	XP_002634943.1	44	68.1662	134	60	Hypothetical protein CBG22545
Locus_13439_Transcript_1/1_Conf_1.000	992	20	1.31E-173	ADF56003.1	99	613.224	310	309	nicotinic acetylcholine receptor alpha subunit 63b
Locus_1344_Transcript_1/1_Conf_1.000	1056	20	8.92E-115	Q94125.6	72	417.927	375	272	Phosphatidylinositol 3-kinase age-1

Locus_13440_Transcript_1/1_Conf_1.000	221	20	3.34E-29	XP_002629937.1	89	131.339	73	65	Hypothetical protein CBG03655
Locus_13441_Transcript_1/1_Conf_1.000	230	0							
Locus_13442_Transcript_1/1_Conf_1.000	163	0							
Locus_13443_Transcript_1/1_Conf_1.000	220	0							
Locus_13444_Transcript_1/1_Conf_1.000	701	10	2.06E-16	XP_001891682.1	47	90.1225	225	106	MRG family protein
Locus_13445_Transcript_1/1_Conf_1.000	225	0							
Locus_13446_Transcript_1/1_Conf_1.000	404	20	3.98E-59	XP_002630309.1	92	230.72	131	121	Hypothetical protein CBG00745
Locus_13447_Transcript_1/1_Conf_1.000	246	0							
Locus_13448_Transcript_1/1_Conf_1.000	481	0							
Locus_13449_Transcript_1/1_Conf_1.000	192	0							
Locus_1345_Transcript_1/1_Conf_1.000	288	0							
Locus_13450_Transcript_1/1_Conf_1.000	446	20	1.75E-22	XP_002635322.1	59	108.997	145	86	Hypothetical protein CBG01489
Locus_13451_Transcript_1/1_Conf_1.000	357	0							
Locus_13452_Transcript_1/1_Conf_1.000	459	4	2.75E-04	XP_002547545.1	42	48.521	147	62	predicted protein
Locus_13453_Transcript_1/1_Conf_1.000	255	0							
Locus_13454_Transcript_1/1_Conf_1.000	1224	20	0	NP_001021621.1	89	686.026	403	361	Nuclear/nucleolar GTP-binding Protein family member (ngp-1)
Locus_13455_Transcript_1/1_Conf_1.000	146	0							
Locus_13456_Transcript_1/1_Conf_1.000	403	2	1.59E-07	NP_509349.1	65	59.3066	58	38	hypothetical protein F45E1.1
Locus_13457_Transcript_1/1_Conf_1.000	600	20	2.88E-33	EFO23325.1	63	145.591	149	94	hypothetical protein LOAG_05163
Locus_13458_Transcript_1/1_Conf_1.000	870	0							
Locus_13459_Transcript_1/1_Conf_1.000	612	0							

Locus_1346_Transcript_1/3_Conf_0.714	1080	20	1.88E-91	NP_493246.1	68	340.502	407	278	hypothetical protein Y18D10A.8
Locus_1346_Transcript_2/3_Conf_0.714	1086	20	5.15E-97	NP_493246.1	69	358.992	407	281	hypothetical protein Y18D10A.8
Locus_1346_Transcript_3/3_Conf_0.714	1110	20	4.37E-99	NP_493246.1	70	365.925	407	287	hypothetical protein Y18D10A.8
Locus_13460_Transcript_1/1_Conf_1.000	475	0							
Locus_13461_Transcript_1/1_Conf_1.000	2719	20	0	XP_002638375.1	78	1257.66	963	754	Hypothetical protein CBG18580
Locus_13462_Transcript_1/1_Conf_1.000	216	5	8.82E-11	NP_001021862.1	57	70.0922	101	58	PMR-type Golgi ATPase family member (pmr-1)
Locus_13463_Transcript_1/1_Conf_1.000	1376	20	0	NP_740958.1	94	647.506	366	345	protein KINase family member (kin-1)
Locus_13464_Transcript_1/1_Conf_1.000	526	0							
Locus_13465_Transcript_1/1_Conf_1.000	319	0							

Locus_13466_Transcript_1/1_Conf_1.000	602	20	1.46E-85	AAU87825.2	88	319.316	186	165	Hypothetical protein C54E4.5
Locus_13467_Transcript_1/1_Conf_1.000	547	1	8.28E-04	XP_640723.1	62	47.3654	95	59	hypothetical protein DDB_G0281503
Locus_13468_Transcript_1/1_Conf_1.000	300	20	7.78E-31	EFO26621.1	81	136.732	93	76	AIDA-1b
Locus_13469_Transcript_1/1_Conf_1.000	701	20	3.22E-86	XP_001895373.1	81	322.013	233	189	PCI domain containing protein
Locus_1347_Transcript_1/1_Conf_1.000	1004	20	3.25E-103	A8WLV5.2	80	379.407	267	215	Probable eukaryotic translation initiation factor 3 subunit G
Locus_13470_Transcript_1/1_Conf_1.000	216	20	2.24E-14	ADF56007.1	95	82.0333	70	67	nicotinic acetylcholine receptor non-alpha subunit 29.3
Locus_13471_Transcript_1/1_Conf_1.000	383	20	8.61E-30	XP_002640484.1	90	133.265	85	77	C. briggsae CBR-TAF-1 protein
Locus_13472_Transcript_1/1_Conf_1.000	238	8	1.34E-06	CAR63562.1	67	56.225	78	53	putative COLlagen
Locus_13473_Transcript_1/1_Conf_1.000	144	0							
Locus_13474_Transcript_1/1_Conf_1.000	306	1	1.28E-09	EFO19473.1	67	66.2402	99	67	AGC/DMPK/GEK protein kinase
Locus_13475_Transcript_1/2_Conf_1.000	170	0							
Locus_13475_Transcript_2/2_Conf_1.000	991	0							
Locus_13476_Transcript_1/1_Conf_1.000	138	19	3.72E-09	EFO15479.1	91	64.6994	34	31	hypothetical protein LOAG_13032
Locus_13477_Transcript_1/1_Conf_1.000	564	7	4.05E-04	XP_002935260.1	42	48.521	171	72	PREDICTED: bromodomain-containing protein 3
Locus_13478_Transcript_1/1_Conf_1.000	352	0							

Locus_13479_Transcript_1/1_Conf_1.000	158	0							
Locus_1348_Transcript_1/1_Conf_1.000	289	0							
Locus_13480_Transcript_1/1_Conf_1.000	130	7	1.58E-07	NP_510233.1	73	59.3066	42	31	CYtochrome P450 family member (cyp-13B1)
Locus_13481_Transcript_1/1_Conf_1.000	128	0							
Locus_13482_Transcript_1/1_Conf_1.000	509	20	5.67E-80	XP_002633437.1	91	300.056	169	155	C. briggsae CBR-UNC-22 protein
Locus_13483_Transcript_1/1_Conf_1.000	380	20	7.05E-40	NP_497647.1	78	166.777	127	100	hypothetical protein Y55D5A.3
Locus_13484_Transcript_1/1_Conf_1.000	132	0							
Locus_13485_Transcript_1/1_Conf_1.000	838	20	2.89E-32	XP_001897363.1	52	143.28	226	119	hypothetical protein Bm1_29605
Locus_13486_Transcript_1/1_Conf_1.000	132	0							
Locus_13487_Transcript_1/1_Conf_1.000	354	20	6.18E-28	NP_001021940.1	70	127.102	121	85	hypothetical protein C08H9.3
Locus_13488_Transcript_1/1_Conf_1.000	224	0							
Locus_13489_Transcript_1/1_Conf_1.000	845	20	9.65E-68	NP_001021145.1	72	261.151	239	174	hypothetical protein C05D11.1
Locus_1349_Transcript_1/1_Conf_1.000	1533	3	3.63E-116	NP_502736.1	60	423.32	520	314	hypothetical protein Y64G10A.6
Locus_13490_Transcript_1/1_Conf_1.000	267	0							
Locus_13491_Transcript_1/1_Conf_1.000	305	0							
Locus_13492_Transcript_1/1_Conf_1.000	452	20	1.12E-53	NP_493642.1	86	212.616	150	129	hypothetical protein F23F1.6
Locus_13493_Transcript_1/1_Conf_1.000	1078	20	6.21E-95	EFO23108.1	66	352.058	363	243	Paf1 family protein
Locus_13494_Transcript_1/2_Conf_1.000	1385	20	0	NP_001021410.2	98	698.738	319	313	LIM domain family member (lim-9)
Locus_13494_Transcript_2/2_Conf_1.000	1385	20	0	NP_001021410.2	98	698.738	319	313	LIM domain family member (lim-9)
Locus_13495_Transcript_1/1_Conf_1.000	150	0							
Locus_13496_Transcript_1/2_Conf_1.000	230	20	2.01E-10	ACA30304.1	63	68.9366	74	47	serine protease

Locus_13496_Transcript_2/2_Conf_1.000	256	20	3.13E-11	ACA30304.1	64	71.633	78	50	serine protease
Locus_13497_Transcript_1/1_Conf_1.000	372	0							
Locus_13498_Transcript_1/1_Conf_1.000	153	0							
Locus_13499_Transcript_1/1_Conf_1.000	142	0							
Locus_135_Transcript_1/1_Conf_1.000	1283	20	1.43E-168	ABX79385.1	96	597.045	341	330	glyceraldehyde-3-phosphate dehydrogenase
Locus_1350_Transcript_1/1_Conf_1.000	1668	20	9.88E-139	XP_001900571.1	69	498.434	495	342	HIRA protein.
Locus_13500_Transcript_1/1_Conf_1.000	689	20	1.94E-80	NP_496210.2	85	302.753	235	200	hypothetical protein F54B3.3
Locus_13501_Transcript_1/1_Conf_1.000	363	1	4.71E-04	XP_758895.1	52	47.7506	67	35	hypothetical protein UM02748.1
Locus_13502_Transcript_1/1_Conf_1.000	330	0							
Locus_13503_Transcript_1/1_Conf_1.000	286	0							
Locus_13504_Transcript_1/1_Conf_1.000	340	0							
Locus_13505_Transcript_1/1_Conf_1.000	786	4	3.37E-32	XP_002630374.1	54	142.895	269	147	Hypothetical protein CBG04313
Locus_13506_Transcript_1/1_Conf_1.000	1287	1	4.67E-18	NP_491884.1	41	97.0561	449	185	hypothetical protein T10E9.2
Locus_13507_Transcript_1/1_Conf_1.000	220	0							

Locus_13508_Transcript_1/1_Conf_1.000	1148	20	1.34E-175	NP_509023.1	86	620.157	382	331	hypothetical protein C15H9.7
Locus_13509_Transcript_1/1_Conf_1.000	443	0							
Locus_1351_Transcript_1/1_Conf_1.000	2915	20	0	XP_002641964.1	98	1678.3	858	841	C. briggsae CBR-PRP-8 protein
Locus_13510_Transcript_1/1_Conf_1.000	865	2	9.01E-08	EFO28197.1	56	62.003	92	52	hypothetical protein LOAG_00279
Locus_13511_Transcript_1/1_Conf_1.000	182	0							
Locus_13512_Transcript_1/1_Conf_1.000	378	0							
Locus_13513_Transcript_1/1_Conf_1.000	148	0							
Locus_13514_Transcript_1/1_Conf_1.000	640	2	3.16E-15	NP_001041016.1	79	85.8853	63	50	hypothetical protein Y116A8C.30
Locus_13515_Transcript_1/1_Conf_1.000	170	0							
Locus_13516_Transcript_1/1_Conf_1.000	388	4	8.31E-09	NP_001022032.1	46	63.5438	121	56	TRRAP-like (transcription/transformation domain-associated protein) family member (trr-1)
Locus_13517_Transcript_1/1_Conf_1.000	573	0							
Locus_13518_Transcript_1/1_Conf_1.000	145	2	4.41E-05	XP_002642155.1	70	51.2174	48	34	C. briggsae CBR-GLR-1 protein
Locus_13519_Transcript_1/1_Conf_1.000	135	0							
Locus_1352_Transcript_1/1_Conf_1.000	587	20	9.11E-13	XP_002195631.1	58	77.411	129	76	PREDICTED: RAD51-like 3 (S. cerevisiae)

Locus_13520_Transcript_1/1_Conf_1.000	346	2	6.08E-07	NP_502515.1	53	57.3806	103	55	hypothetical protein C39E9.11
Locus_13521_Transcript_1/1_Conf_1.000	1096	20	1.21E-77	EFO19790.1	61	294.664	378	232	exostosin-1
Locus_13522_Transcript_1/1_Conf_1.000	441	20	3.66E-36	XP_002640257.1	67	154.451	149	100	Hypothetical protein CBG12782
Locus_13523_Transcript_1/1_Conf_1.000	527	20	6.82E-42	EFO20779.1	66	173.711	175	117	hypothetical protein LOAG_07709
Locus_13524_Transcript_1/1_Conf_1.000	385	20	2.49E-37	XP_002641238.1	74	158.303	133	99	C. briggsae CBR-THOC-2 protein
Locus_13525_Transcript_1/1_Conf_1.000	243	0							
Locus_13526_Transcript_1/1_Conf_1.000	487	0							
Locus_13527_Transcript_1/1_Conf_1.000	265	0							
Locus_13528_Transcript_1/1_Conf_1.000	492	0							
Locus_13529_Transcript_1/1_Conf_1.000	363	0							
Locus_1353_Transcript_2/3_Conf_0.667	798	20	8.63E-116	NP_001076672.1	89	420.624	249	224	hypothetical protein F01G4.6
Locus_1353_Transcript_3/3_Conf_0.667	798	20	8.64E-116	NP_001076672.1	89	420.624	249	224	hypothetical protein F01G4.6
Locus_13530_Transcript_1/1_Conf_1.000	955	1	6.55E-42	CAG28316.1	65	175.637	202	132	spindle assembly abnormal protein 5, SAS-5
Locus_13531_Transcript_1/2_Conf_1.000	470	0							
Locus_13531_Transcript_2/2_Conf_1.000	470	0							
Locus_13532_Transcript_1/1_Conf_1.000	331	2	2.78E-12	NP_508464.2	64	75.0998	96	62	hypothetical protein F52H2.7

Locus_13533_Transcript_1/1_Conf_1.000	475	3	2.68E-07	NP_001021054.1	48	58.5362	154	75	Suppressor with Morphological effect on Genitalia family member (smg-1)
Locus_13534_Transcript_1/1_Conf_1.000	486	0							
Locus_13535_Transcript_1/1_Conf_1.000	155	2	1.66E-04	XP_002634292.1	72	49.2914	51	37	C. briggsae CBR-UGT-54 protein
Locus_13536_Transcript_1/1_Conf_1.000	230	0							
Locus_13537_Transcript_1/1_Conf_1.000	400	20	9.23E-48	XP_001898337.1	82	192.971	133	110	oxidoreductase, short chain dehydrogenase/reductase family protein
Locus_13538_Transcript_1/1_Conf_1.000	895	20	2.30E-70	Q610N4.2	77	270.011	253	195	Transmembrane protein 104 homolog
Locus_13539_Transcript_1/1_Conf_1.000	381	0							
Locus_1354_Transcript_1/1_Conf_1.000	472	20	2.11E-44	XP_002634364.1	96	181.8	82	79	C. briggsae CBR-EXC-9 protein
Locus_13540_Transcript_1/1_Conf_1.000	438	20	4.04E-56	XP_002637802.1	94	220.705	137	130	C. briggsae CBR-HDA-1 protein
Locus_13541_Transcript_1/1_Conf_1.000	686	0							
Locus_13542_Transcript_1/1_Conf_1.000	161	0							
Locus_13543_Transcript_1/1_Conf_1.000	465	20	3.29E-37	XP_002631639.1	77	157.918	149	116	Hypothetical protein CBG20828
Locus_13544_Transcript_1/1_Conf_1.000	145	2	1.08E-11	NP_492142.1	85	73.1738	47	40	hypothetical protein T23G11.4
Locus_13545_Transcript_1/1_Conf_1.000	149	0							
Locus_13546_Transcript_1/1_Conf_1.000	145	0							
Locus_13547_Transcript_1/1_Conf_1.000	497	6	6.75E-43	NP_498388.1	69	176.792	166	116	hypothetical protein C56G2.3
Locus_13548_Transcript_1/1_Conf_1.000	688	20	1.54E-85	XP_002636924.1	81	319.701	229	186	C. briggsae CBR-ACL-6 protein
Locus_13549_Transcript_1/1_Conf_1.000	201	4	1.99E-26	XP_002632215.1	95	122.094	66	63	Hypothetical protein CBG07083
Locus_1355_Transcript_1/1_Conf_1.000	229	0							
Locus_13550_Transcript_1/1_Conf_1.000	248	0							
Locus_13551_Transcript_1/1_Conf_1.000	382	0							

Locus_13552_Transcript_1/1_Conf_1.000	570	20	3.37E-62	ACA81684.1	84	241.506	161	136	cholecystokinin receptor 2 splice isoform b
Locus_13553_Transcript_1/1_Conf_1.000	263	0							
Locus_13554_Transcript_1/1_Conf_1.000	631	20	2.10E-32	EFO24248.1	61	142.895	175	107	repair protein Rad1/Rec1/Rad17 containing protein
Locus_13555_Transcript_1/1_Conf_1.000	514	20	3.54E-37	CAR63581.1	77	157.918	143	111	putative COLlagen
Locus_13556_Transcript_1/1_Conf_1.000	295	7	5.45E-16	NP_490710.2	75	87.4261	80	60	hypothetical protein C53D5.2
Locus_13557_Transcript_1/1_Conf_1.000	268	20	1.64E-04	DAA32996.1	44	49.2914	83	37	ubiquitin-associated and SH3 domain-containing protein A
Locus_13558_Transcript_1/1_Conf_1.000	547	20	1.27E-12	XP_001704397.1	47	76.6406	141	67	Tenascin-X precursor
Locus_13559_Transcript_1/2_Conf_1.000	1350	20	9.68E-163	XP_002638613.1	83	577.785	407	340	C. briggsae CBR-PGP-9 protein
Locus_13559_Transcript_2/2_Conf_1.000	1350	20	9.68E-163	XP_002638613.1	83	577.785	407	340	C. briggsae CBR-PGP-9 protein

Locus_1356_Transcript_1/2_Conf_1.000	2814	20	0	NP_490865.3	78	1028.08	758	592	hypothetical protein Y92H12A.2
Locus_1356_Transcript_2/2_Conf_1.000	2754	20	0	NP_490865.3	80	1030.78	743	598	hypothetical protein Y92H12A.2
Locus_13560_Transcript_1/1_Conf_1.000	326	0							

Locus_13561_Transcript_1/1_Conf_1.000	1146	20	1.69E-69	EFO24820.1	66	267.7	296	196	hypothetical protein LOAG_03664
Locus_13562_Transcript_1/1_Conf_1.000	221	3	1.16E-05	NP_492441.1	52	53.1434	70	37	related to vertebrate MEF2 transcription factor family member (mef-2)
Locus_13563_Transcript_1/1_Conf_1.000	231	2	1.49E-05	XP_002630854.1	68	52.7582	50	34	C. briggsae CBR-RAN-5 protein
Locus_13564_Transcript_1/1_Conf_1.000	265	20	8.66E-30	XP_002629838.1	85	133.265	87	74	Hypothetical protein CBG18727
Locus_13565_Transcript_1/1_Conf_1.000	128	2	6.42E-09	XP_002636472.1	80	63.929	42	34	Hypothetical protein CBG23143
Locus_13566_Transcript_1/1_Conf_1.000	319	4	3.09E-35	CAR63626.1	89	151.369	108	97	hypothetical protein
Locus_13567_Transcript_1/1_Conf_1.000	347	0							
Locus_13568_Transcript_1/1_Conf_1.000	484	5	6.92E-19	NP_509847.1	60	97.0561	128	77	CLaudin-like in Caenorhabditis family member (clc-1)
Locus_13569_Transcript_1/1_Conf_1.000	243	0							
Locus_1357_Transcript_1/5_Conf_0.688	3471	20	9.63E-92	XP_002641165.1	57	343.584	600	347	C. briggsae CBR-NPP-10 protein
Locus_1357_Transcript_2/5_Conf_0.125	492	5	9.25E-08	XP_002641165.1	55	60.077	85	47	C. briggsae CBR-NPP-10 protein
Locus_1357_Transcript_3/5_Conf_0.188	1343	20	2.37E-36	ACZ54696.1	64	157.918	242	156	Nuclear pore complex protein protein 10, isoform c, partially confirmed by transcript evidence
Locus_1357_Transcript_4/5_Conf_0.562	2980	20	6.44E-81	XP_002641165.1	63	307.375	417	264	C. briggsae CBR-NPP-10 protein
Locus_1357_Transcript_5/5_Conf_0.688	3492	20	9.69E-92	XP_002641165.1	57	343.584	600	347	C. briggsae CBR-NPP-10 protein
Locus_13570_Transcript_1/1_Conf_1.000	221	20	6.73E-30	XP_002826714.1	100	133.65	63	63	PREDICTED: 60S ribosomal protein L21-like isoform 1
Locus_13571_Transcript_1/1_Conf_1.000	211	0							
Locus_13572_Transcript_1/1_Conf_1.000	591	1	2.87E-06	NP_504410.1	48	55.8398	108	52	hypothetical protein B0238.11
Locus_13573_Transcript_1/1_Conf_1.000	185	19	7.48E-13	NP_491270.2	79	77.0258	48	38	Low-density lipoprotein Receptor Related family member (lrp-2)
Locus_13574_Transcript_1/1_Conf_1.000	211	1	6.16E-04	XP_002646401.1	58	47.3654	70	41	C. briggsae CBR-DYS-1 protein

Locus_13575_Transcript_1/1_Conf_1.000	676	20	1.79E-107	NP_497185.1	93	392.504	223	208	Alkyl-Dihydroxyacetonephosphate Synthase family member (ads-1)
Locus_13576_Transcript_1/1_Conf_1.000	574	20	1.83E-39	EFO22852.1	65	166.007	194	127	WD domain-containing protein
Locus_13577_Transcript_1/3_Conf_0.667	1150	20	1.10E-177	XP_002641484.1	89	627.091	386	345	Hypothetical protein CBG09775
Locus_13577_Transcript_2/3_Conf_0.667	1150	20	3.00E-175	XP_002641484.1	89	619.002	385	344	Hypothetical protein CBG09775
Locus_13577_Transcript_3/3_Conf_0.667	1150	20	3.00E-175	XP_002641484.1	89	619.002	385	344	Hypothetical protein CBG09775
Locus_13578_Transcript_1/1_Conf_1.000	203	0							
Locus_13579_Transcript_1/2_Conf_1.000	631	20	3.13E-36	CBA11992.1	72	155.606	122	89	endonuclease-reverse transcriptase HmRTE-e01
Locus_13579_Transcript_2/2_Conf_1.000	313	2	6.77E-06	XP_002632164.1	68	53.9138	60	41	Hypothetical protein CBG07023
Locus_1358_Transcript_1/6_Conf_0.286	753	20	5.14E-104	NP_741155.2	95	381.333	227	216	hypothetical protein F01F1.12
Locus_1358_Transcript_2/6_Conf_0.286	711	20	1.34E-103	NP_741155.2	95	379.793	224	213	hypothetical protein F01F1.12
Locus_1358_Transcript_3/6_Conf_0.357	1287	20	2.85E-108	XP_002641199.1	94	396.741	228	215	Hypothetical protein CBG09060

Locus_1358_Transcript_4/6_Conf_0.357	1287	20	1.42E-107	XP_002641199.1	94	394.43	228	215	Hypothetical protein CBG09060
Locus_1358_Transcript_5/6_Conf_0.357	1288	20	6.04E-167	NP_001021240.1	94	591.652	361	340	hypothetical protein F01F1.12
Locus_1358_Transcript_6/6_Conf_0.357	1288	20	1.49E-165	NP_001021240.1	94	587.03	361	340	hypothetical protein F01F1.12
Locus_13580_Transcript_1/1_Conf_1.000	326	0							
Locus_13581_Transcript_1/1_Conf_1.000	685	0							
Locus_13582_Transcript_1/1_Conf_1.000	494	20	4.86E-41	XP_001901777.1	80	170.629	110	88	Melibiase family protein
Locus_13583_Transcript_1/1_Conf_1.000	175	0							
Locus_13584_Transcript_1/1_Conf_1.000	171	20	4.55E-23	AAF59456.4	96	110.923	57	55	Smg-associated and lethal protein 2, partially confirmed by transcript evidence
Locus_13585_Transcript_1/1_Conf_1.000	439	0							
Locus_13586_Transcript_1/1_Conf_1.000	502	0							
Locus_13588_Transcript_1/1_Conf_1.000	541	20	6.96E-08	ZP_04297130.1	45	60.8474	140	63	Cell divisionFtsK/SpoIIIE
Locus_13589_Transcript_1/1_Conf_1.000	316	5	7.46E-05	EFO12242.1	66	50.447	56	37	hypothetical protein LOAG_16291
Locus_1359_Transcript_1/5_Conf_0.444	887	20	5.77E-50	CBA11992.1	59	202.216	264	158	endonuclease-reverse transcriptase HmRTE-e01

Locus_1359_Transcript_2/5_Conf_0.444	673	13	2.21E-25	CBA11992.1	58	119.783	163	96	endonuclease-reverse transcriptase HmRTE-e01
Locus_1359_Transcript_3/5_Conf_0.222	271	0							
Locus_1359_Transcript_4/5_Conf_0.333	1219	20	9.52E-66	CBA11992.1	55	255.373	358	199	endonuclease-reverse transcriptase HmRTE-e01
Locus_1359_Transcript_5/5_Conf_0.444	953	20	5.03E-50	CBA11992.1	60	202.601	265	159	endonuclease-reverse transcriptase HmRTE-e01
Locus_13590_Transcript_1/1_Conf_1.000	189	0							
Locus_13591_Transcript_1/2_Conf_1.000	774	3	1.80E-22	NP_001022822.1	42	110.538	328	140	hypothetical protein Y111B2A.9
Locus_13591_Transcript_2/2_Conf_1.000	774	3	1.37E-22	NP_001022822.1	42	110.923	328	140	hypothetical protein Y111B2A.9
Locus_13592_Transcript_1/2_Conf_1.000	1066	20	6.87E-147	XP_001899040.1	92	524.628	336	310	vinculin
Locus_13592_Transcript_2/2_Conf_1.000	1066	20	6.87E-147	XP_001899040.1	92	524.628	336	310	vinculin
Locus_13593_Transcript_1/1_Conf_1.000	481	20	1.27E-33	XP_002636940.1	92	145.976	79	73	Hypothetical protein CBG09413
Locus_13594_Transcript_1/1_Conf_1.000	501	3	7.43E-13	CAR63585.1	76	77.0258	56	43	putative MAD-Like family member
Locus_13595_Transcript_1/2_Conf_1.000	152	0							
Locus_13595_Transcript_2/2_Conf_1.000	144	0							
Locus_13596_Transcript_1/1_Conf_1.000	172	0							
Locus_13597_Transcript_1/1_Conf_1.000	227	0							
Locus_13598_Transcript_1/1_Conf_1.000	454	20	1.52E-18	NP_509240.2	57	95.9005	142	81	TransThyretin-Related family domain family member (ttr-30)
Locus_13599_Transcript_1/1_Conf_1.000	230	0							
Locus_136_Transcript_1/2_Conf_1.000	645	0							
Locus_136_Transcript_2/2_Conf_1.000	907	0							
Locus_1360_Transcript_1/1_Conf_1.000	294	0							
Locus_13600_Transcript_1/1_Conf_1.000	358	0							
Locus_13601_Transcript_1/1_Conf_1.000	356	0							

Locus_13602_Transcript_1/1_Conf_1.000	177	20	4.83E-25	2XA7	100	117.472	58	58	Ap2 Clathrin Adaptor Core In Active Complex With Cargo Peptides
Locus_13603_Transcript_1/2_Conf_1.000	644	0							
Locus_13603_Transcript_2/2_Conf_1.000	641	0							
Locus_13604_Transcript_1/2_Conf_1.000	578	4	1.54E-09	XP_002641258.1	94	66.6254	35	33	Hypothetical protein CBG05169
Locus_13604_Transcript_2/2_Conf_1.000	488	4	9.98E-10	XP_002641258.1	94	66.6254	35	33	Hypothetical protein CBG05169
Locus_13605_Transcript_1/2_Conf_1.000	344	0							
Locus_13605_Transcript_2/2_Conf_1.000	344	0							
Locus_13606_Transcript_1/1_Conf_1.000	860	20	3.01E-56	CAA22108.2	63	223.016	300	189	C. elegans protein Y75B8A.24, partially confirmed by transcript evidence
Locus_13607_Transcript_1/1_Conf_1.000	405	20	3.48E-47	XP_002641892.1	83	191.045	130	109	Hypothetical protein CBG16586
Locus_13608_Transcript_1/1_Conf_1.000	891	20	1.22E-31	EFO23701.1	52	141.354	291	153	TPR Domain containing protein
Locus_13609_Transcript_1/1_Conf_1.000	880	20	3.50E-07	XP_692550.3	42	60.077	325	138	PREDICTED: si:dkey-230p4.1
Locus_1361_Transcript_1/1_Conf_1.000	1005	20	2.30E-56	NP_001022708.1	56	223.787	352	199	Inhibitor of NFKappaB Kinase Epsilon subunit homolog family member (ikke-1)
Locus_13610_Transcript_1/1_Conf_1.000	310	0							
Locus_13611_Transcript_1/1_Conf_1.000	837	20	1.75E-37	EFO26887.1	53	160.614	247	132	hypothetical protein LOAG_01595
Locus_13612_Transcript_1/1_Conf_1.000	450	0							

Locus_13613_Transcript_1/1_Conf_1.000	494	1	4.75E-04	EFO15567.1	48	47.7506	78	38	hypothetical protein LOAG_12942
Locus_13614_Transcript_1/1_Conf_1.000	728	2	9.00E-18	NP_502746.1	50	94.7449	253	128	hypothetical protein Y67A10A.9
Locus_13615_Transcript_1/1_Conf_1.000	416	20	1.35E-51	EFO20693.1	87	205.682	139	121	hypothetical protein LOAG_07794
Locus_13616_Transcript_1/1_Conf_1.000	237	0							
Locus_13617_Transcript_1/1_Conf_1.000	185	20	6.75E-22	EFO21414.1	91	107.071	61	56	hypothetical protein LOAG_07072
Locus_13618_Transcript_1/1_Conf_1.000	200	20	1.06E-19	EFO24572.1	78	99.7525	65	51	carboxyl transferase domain-containing protein
Locus_13619_Transcript_1/1_Conf_1.000	204	20	5.22E-27	CAR63646.1	88	124.02	67	59	putative Homeobox family member
Locus_1362_Transcript_1/1_Conf_1.000	135	2	3.27E-05	NP_492659.1	78	51.6026	41	32	hypothetical protein B0379.1
Locus_13620_Transcript_1/1_Conf_1.000	263	0							
Locus_13621_Transcript_1/1_Conf_1.000	395	20	2.21E-57	XP_002644998.1	89	224.942	131	117	C. briggsae CBR-LAM-2 protein
Locus_13622_Transcript_1/1_Conf_1.000	300	0							

Locus_13623_Transcript_1/1_Conf_1.000	214	20	6.34E-09	NP_491270.2	59	63.929	71	42	Low-density lipoprotein Receptor Related family member (lrp-2)
Locus_13624_Transcript_1/1_Conf_1.000	346	4	1.30E-33	XP_002641415.1	83	145.976	101	84	Hypothetical protein CBG13282
Locus_13625_Transcript_1/1_Conf_1.000	431	20	7.05E-24	XP_002631495.1	72	113.62	144	105	Hypothetical protein CBG20655
Locus_13626_Transcript_1/1_Conf_1.000	392	0							
Locus_13627_Transcript_1/1_Conf_1.000	983	20	3.23E-116	XP_002647755.1	75	422.55	328	247	C. briggsae CBR-ASP-1 protein
Locus_13628_Transcript_1/2_Conf_1.000	712	0							
Locus_13628_Transcript_2/2_Conf_1.000	1566	0							
Locus_13629_Transcript_1/1_Conf_1.000	904	20	6.12E-87	XP_002640327.1	70	325.094	301	213	C. briggsae CBR-GLH-1 protein
Locus_1363_Transcript_1/1_Conf_1.000	514	2	2.74E-05	XP_002631254.1	51	51.9878	116	60	Hypothetical protein CBG03058
Locus_13630_Transcript_1/1_Conf_1.000	624	0							
Locus_13631_Transcript_1/1_Conf_1.000	1361	1	2.13E-08	XP_001901492.1	44	65.0846	213	94	hypothetical protein
Locus_13632_Transcript_1/1_Conf_1.000	272	0							
Locus_13633_Transcript_1/1_Conf_1.000	329	2	2.98E-06	XP_002636840.1	68	55.0694	61	42	Hypothetical protein CBG09291

Locus_13634_Transcript_1/1_Conf_1.000	499	20	3.24E-29	NP_505373.1	57	131.339	153	88	hypothetical protein B0222.5
Locus_13635_Transcript_1/1_Conf_1.000	146	0							
Locus_13636_Transcript_1/1_Conf_1.000	192	0							
Locus_13637_Transcript_1/1_Conf_1.000	1292	20	0	AAR07614.1	99	801.586	408	406	UNC-18
Locus_13638_Transcript_1/1_Conf_1.000	159	0							
Locus_13639_Transcript_1/1_Conf_1.000	422	5	9.67E-13	XP_002645040.1	54	76.6406	142	77	C. briggsae CBR-CLC-5 protein
Locus_1364_Transcript_1/1_Conf_1.000	348	20	2.22E-17	NP_491097.3	75	92.0485	73	55	hypothetical protein Y54E10BR.1
Locus_13640_Transcript_1/1_Conf_1.000	181	0							
Locus_13641_Transcript_1/1_Conf_1.000	321	0							
Locus_13642_Transcript_1/1_Conf_1.000	559	20	2.13E-26	XP_002637392.1	64	122.479	145	93	Hypothetical protein CBG19099
Locus_13643_Transcript_1/1_Conf_1.000	179	0							
Locus_13644_Transcript_1/1_Conf_1.000	186	20	3.70E-12	XP_002631121.1	72	74.7146	58	42	Hypothetical protein CBG02899
Locus_13645_Transcript_1/1_Conf_1.000	172	20	2.41E-24	XP_002758725.1	100	115.161	57	57	PREDICTED: dolichyl-diphosphooligosaccharide--protein glycosyltransferase subunit 1-like isoform 2
Locus_13646_Transcript_1/2_Conf_1.000	504	0							
Locus_13646_Transcript_2/2_Conf_1.000	477	0							
Locus_13647_Transcript_1/1_Conf_1.000	170	0							
Locus_13648_Transcript_1/1_Conf_1.000	176	0							
Locus_13649_Transcript_1/1_Conf_1.000	229	0							
Locus_1365_Transcript_1/5_Conf_0.222	672	20	2.19E-65	AAG36874.1	83	252.677	182	152	AF242767_1SF2
Locus_1365_Transcript_2/5_Conf_0.333	534	0							
Locus_1365_Transcript_3/5_Conf_0.222	748	20	2.75E-65	AAG36874.1	83	252.677	182	152	AF242767_1SF2

Locus_1365_Transcript_4/5_Conf_0.556	1140	20	6.14E-72	AAG36874.1	83	275.789	198	165	AF242767_1SF2
Locus_1365_Transcript_5/5_Conf_0.556	975	20	4.83E-72	AAG36874.1	83	275.789	198	165	AF242767_1SF2
Locus_13650_Transcript_1/1_Conf_1.000	142	0							
Locus_13651_Transcript_1/1_Conf_1.000	395	20	1.26E-28	XP_001901742.1	77	129.413	94	73	PWI domain containing protein
Locus_13652_Transcript_1/1_Conf_1.000	377	20	6.63E-38	NP_001023236.1	80	160.229	125	101	hypothetical protein F56H11.1
Locus_13653_Transcript_1/1_Conf_1.000	459	14	8.77E-35	XP_002640654.1	72	149.828	153	111	Hypothetical protein CBG19712
Locus_13654_Transcript_1/1_Conf_1.000	339	0							
Locus_13655_Transcript_1/1_Conf_1.000	366	20	6.28E-33	NP_001022691.1	87	143.665	94	82	AVEugle (Drosophila eye differentation) homolog family member (ave-1)
Locus_13656_Transcript_1/1_Conf_1.000	258	4	5.50E-08	EFO22604.1	67	60.8474	76	51	RPN-3 protein
Locus_13657_Transcript_1/1_Conf_1.000	171	5	9.20E-16	NP_495035.1	83	86.6557	55	46	DeHydrogenases, Short chain family member (dhs-5)
Locus_13658_Transcript_1/1_Conf_1.000	952	20	5.77E-115	XP_002645892.1	82	418.313	317	260	Hypothetical protein CBG07642
Locus_13659_Transcript_1/1_Conf_1.000	721	2	1.78E-10	EFO16155.1	50	70.4774	145	73	hypothetical protein LOAG_12354
Locus_1366_Transcript_1/1_Conf_1.000	362	5	3.72E-25	CAR63528.1	96	117.857	64	62	hypothetical protein

Locus_13660_Transcript_1/1_Conf_1.000	420	20	1.64E-36	XP_002636220.1	70	155.606	128	90	Hypothetical protein CBG12141
Locus_13661_Transcript_1/1_Conf_1.000	131	0							
Locus_13662_Transcript_1/1_Conf_1.000	314	0							
Locus_13663_Transcript_1/1_Conf_1.000	194	0							
Locus_13664_Transcript_1/1_Conf_1.000	957	2	1.10E-04	XP_001270159.1	40	51.9878	275	112	GAS2 domain protein
Locus_13665_Transcript_1/2_Conf_1.000	1756	20	1.06E-114	XP_001898777.1	73	418.698	343	251	daf-18 protein

Locus_13665_Transcript_2/2_Conf_1.000	1666	20	9.93E-115	XP_001898777.1	73	418.698	343	251	daf-18 protein
Locus_13666_Transcript_1/1_Conf_1.000	138	0							
Locus_13667_Transcript_1/1_Conf_1.000	152	0							
Locus_13668_Transcript_1/1_Conf_1.000	760	20	5.15E-43	NP_001123112.1	78	178.718	119	94	hypothetical protein C17H11.6
Locus_13669_Transcript_1/1_Conf_1.000	381	20	8.63E-22	XP_001899513.1	66	106.686	116	77	AIDA-1b
Locus_1367_Transcript_1/3_Conf_0.714	1556	0							
Locus_1367_Transcript_2/3_Conf_0.571	1020	0							
Locus_1367_Transcript_3/3_Conf_0.714	1553	0							
Locus_13670_Transcript_1/1_Conf_1.000	493	20	4.09E-64	EFO26413.1	91	247.284	135	123	F-box only protein 11
Locus_13671_Transcript_1/1_Conf_1.000	130	0							
Locus_13672_Transcript_1/1_Conf_1.000	231	0							
Locus_13673_Transcript_1/1_Conf_1.000	138	0							
Locus_13674_Transcript_1/1_Conf_1.000	379	20	1.24E-36	NP_509879.2	81	155.992	125	102	hypothetical protein C03A3.2
Locus_13675_Transcript_1/1_Conf_1.000	268	0							
Locus_13676_Transcript_1/1_Conf_1.000	328	0							
Locus_13677_Transcript_1/1_Conf_1.000	237	5	4.58E-23	XP_002631799.1	81	110.923	79	64	Hypothetical protein CBG21018
Locus_13678_Transcript_1/1_Conf_1.000	452	7	2.35E-19	CAB60494.2	63	98.5969	136	87	C. elegans protein Y39C12A.1, partially confirmed by transcript evidence

Locus_13679_Transcript_1/1_Conf_1.000	846	20	1.45E-63	NP_503370.1	65	247.284	238	155	hypothetical protein K10C9.3
Locus_1368_Transcript_1/1_Conf_1.000	838	20	2.77E-67	XP_002630597.1	73	259.61	236	174	Hypothetical protein CBG13057
Locus_13680_Transcript_1/1_Conf_1.000	145	20	1.08E-11	XP_002630906.1	75	73.1738	48	36	Hypothetical protein CBG02630
Locus_13681_Transcript_1/1_Conf_1.000	609	0							
Locus_13682_Transcript_1/1_Conf_1.000	312	0							
Locus_13683_Transcript_1/1_Conf_1.000	553	20	4.53E-21	EFO23361.1	59	104.76	140	83	cyclin domain-containing protein
Locus_13684_Transcript_1/1_Conf_1.000	203	0							
Locus_13685_Transcript_1/1_Conf_1.000	410	20	1.46E-45	XP_001896252.1	82	185.652	134	110	MED7 protein
Locus_13686_Transcript_1/1_Conf_1.000	280	20	3.46E-31	XP_001897202.1	86	137.887	93	80	CG8090-PA
Locus_13687_Transcript_1/1_Conf_1.000	274	2	1.37E-11	NP_495410.4	62	72.7886	82	51	hypothetical protein C44B7.11
Locus_13688_Transcript_1/1_Conf_1.000	308	0							
Locus_13689_Transcript_1/1_Conf_1.000	593	20	1.71E-67	XP_002641317.1	80	259.225	200	160	C. briggsae CBR-PAD-2 protein
Locus_1369_Transcript_1/1_Conf_1.000	616	20	3.87E-60	NP_496176.1	74	234.958	203	151	beta-LACTamase domain containing family member (lact-2)
Locus_13690_Transcript_1/1_Conf_1.000	1069	20	8.71E-33	XP_002647217.1	68	145.591	208	142	Hypothetical protein CBG22401
Locus_13691_Transcript_1/1_Conf_1.000	279	20	2.04E-15	EFO22882.1	76	85.5001	60	46	TK/FER protein kinase
Locus_13692_Transcript_1/1_Conf_1.000	707	20	4.59E-32	XP_002643629.1	88	142.124	124	110	C. briggsae CBR-LET-2 protein

Locus_13693_Transcript_1/1_Conf_1.000	551	0								
Locus_13694_Transcript_1/1_Conf_1.000	461	20	2.08E-44	NP_510159.1	83	181.8	118	98	hypothetical protein F11C1.1	
Locus_13695_Transcript_1/1_Conf_1.000	633	20	3.04E-71	XP_001902705.1	86	271.937	181	157	Ras-related protein Rab-11B	
Locus_13696_Transcript_1/1_Conf_1.000	525	20	1.05E-50	NP_491216.2	76	202.986	171	130	Resistance to Inhibitors of Cholinesterase family member (ric-19)	
Locus_13697_Transcript_1/1_Conf_1.000	734	20	5.14E-53	NP_493372.1	70	211.846	220	155	NuDiX family member (ndx-8)	
Locus_13698_Transcript_1/2_Conf_1.000	296	0								
Locus_13698_Transcript_2/2_Conf_1.000	308	0								
Locus_13699_Transcript_1/2_Conf_1.000	783	20	9.29E-75	AAN11401.1	69	284.263	259	181	metalloprotease 1 precursor	
Locus_13699_Transcript_2/2_Conf_1.000	783	20	4.17E-75	AAN11401.1	70	285.419	259	182	metalloprotease 1 precursor	
Locus_137_Transcript_10/15_Conf_0.375	380	20	1.56E-55	ABM92348.1	99	218.779	106	105	beta tubulin isotype 1	
Locus_137_Transcript_11/15_Conf_0.375	380	20	1.56E-55	ABM92348.1	99	218.779	106	105	beta tubulin isotype 1	
Locus_137_Transcript_12/15_Conf_0.375	380	20	1.56E-55	ABM92348.1	99	218.779	106	105	beta tubulin isotype 1	

Locus_137_Transcript_13/15_Conf_0.375	380	20	1.56E-55	ABM92348.1	99	218.779	106	105	beta tubulin isotype 1
Locus_137_Transcript_14/15_Conf_0.375	380	20	1.56E-55	ABM92348.1	99	218.779	106	105	beta tubulin isotype 1
Locus_137_Transcript_15/15_Conf_0.375	380	20	1.56E-55	ABM92348.1	99	218.779	106	105	beta tubulin isotype 1
Locus_137_Transcript_2/15_Conf_1.000	1082	20	1.66E-180	AAP20434.1	99	636.336	333	332	beta-tubulin isotype 1
Locus_137_Transcript_3/15_Conf_1.000	1082	20	0	AAP20434.1	99	638.262	333	332	beta-tubulin isotype 1
Locus_137_Transcript_4/15_Conf_1.000	167	20	4.89E-25	ACS50329.1	100	117.472	55	55	beta-tubulin
Locus_137_Transcript_5/15_Conf_1.000	221	20	9.41E-24	ABM92348.1	98	113.235	53	52	beta tubulin isotype 1
Locus_137_Transcript_6/15_Conf_0.375	380	20	1.56E-55	ABM92348.1	99	218.779	106	105	beta tubulin isotype 1
Locus_137_Transcript_7/15_Conf_0.375	380	20	1.56E-55	ABM92348.1	99	218.779	106	105	beta tubulin isotype 1

Locus_137_Transcript_8/15_Conf_0.375	380	20	1.56E-55	ABM92348.1	99	218.779	106	105	beta tubulin isotype 1
Locus_137_Transcript_9/15_Conf_0.375	380	20	1.56E-55	ABM92348.1	99	218.779	106	105	beta tubulin isotype 1
Locus_1370_Transcript_1/2_Conf_1.000	1722	20	3.34E-89	NP_505054.1	71	333.954	292	210	hypothetical protein Y97E10AL.2
Locus_1370_Transcript_2/2_Conf_1.000	1722	20	3.34E-89	NP_505054.1	71	333.954	292	210	hypothetical protein Y97E10AL.2
Locus_13700_Transcript_1/1_Conf_1.000	141	0							
Locus_13701_Transcript_1/1_Conf_1.000	173	0							
Locus_13702_Transcript_1/1_Conf_1.000	512	0							
Locus_13703_Transcript_1/1_Conf_1.000	155	2	2.39E-11	NP_501239.3	72	72.0182	51	37	hypothetical protein F45E4.5
Locus_13704_Transcript_1/1_Conf_1.000	367	19	1.97E-10	XP_002631639.1	55	68.9366	103	57	Hypothetical protein CBG20828
Locus_13705_Transcript_1/1_Conf_1.000	405	20	1.42E-40	NP_496968.1	83	169.088	135	113	hypothetical protein Y48B6A.12
Locus_13706_Transcript_1/1_Conf_1.000	237	0							
Locus_13707_Transcript_1/1_Conf_1.000	357	0							
Locus_13708_Transcript_1/1_Conf_1.000	137	0							
Locus_13709_Transcript_1/1_Conf_1.000	465	20	1.92E-61	NP_491989.1	91	238.424	136	124	hypothetical protein C30F12.7

Locus_1371_Transcript_1/1_Conf_1.000	488	20	6.86E-35	XP_002636128.1	70	150.214	134	94	Hypothetical protein CBG01378
Locus_13710_Transcript_1/1_Conf_1.000	128	20	1.58E-07	AAD01617.2	92	59.3066	42	39	cyclophilin-16
Locus_13711_Transcript_1/1_Conf_1.000	407	0							
Locus_13712_Transcript_1/1_Conf_1.000	341	20	3.13E-24	EFO24293.1	80	114.775	85	68	hypothetical protein LOAG_04190
Locus_13713_Transcript_1/1_Conf_1.000	177	0							
Locus_13714_Transcript_1/1_Conf_1.000	145	0							
Locus_13715_Transcript_1/1_Conf_1.000	309	14	1.72E-14	CAD44516.1	59	82.4185	102	61	VAB-10B protein
Locus_13716_Transcript_1/1_Conf_1.000	954	20	4.09E-60	NP_500156.2	78	236.113	184	145	XPC (Xeroderma Pigmentosum group C) DNA repair gene homolog family member (xpc-1)
Locus_13717_Transcript_1/1_Conf_1.000	461	20	1.35E-51	XP_002631622.1	76	205.682	151	116	Hypothetical protein CBG20808
Locus_13718_Transcript_1/1_Conf_1.000	498	0							
Locus_13719_Transcript_1/1_Conf_1.000	180	0							
Locus_1372_Transcript_1/1_Conf_1.000	1716	20	1.61E-91	EFO25336.1	68	341.658	317	218	EBF3-S
Locus_13720_Transcript_1/1_Conf_1.000	864	20	6.66E-11	EFN66847.1	49	72.4034	131	65	hypothetical protein EAG_08499
Locus_13721_Transcript_1/1_Conf_1.000	230	0							
Locus_13722_Transcript_1/1_Conf_1.000	464	8	1.30E-25	NP_507480.1	69	119.398	129	90	TWiK family of potassium channels family member (twk-33)
Locus_13723_Transcript_1/1_Conf_1.000	391	0							

Locus_13724_Transcript_1/1_Conf_1.000	382	3	1.34E-06	XP_002643776.1	67	56.225	43	29	C. briggsae CBR-FLP-2 protein
Locus_13725_Transcript_1/1_Conf_1.000	152	20	4.67E-15	NP_505261.2	84	84.3445	50	42	hypothetical protein B0507.2
Locus_13726_Transcript_1/1_Conf_1.000	254	0							
Locus_13727_Transcript_1/1_Conf_1.000	657	1	1.21E-12	XP_002647382.1	45	77.411	200	91	Hypothetical protein CBG06447
Locus_13728_Transcript_1/1_Conf_1.000	244	0							
Locus_13729_Transcript_1/1_Conf_1.000	1080	20	4.78E-95	AAA97973.2	64	352.443	363	233	Hypothetical protein F21C10.7
Locus_1373_Transcript_1/1_Conf_1.000	993	20	6.49E-88	NP_001122884.1	69	328.561	291	203	Temporarily Assigned Gene name family member (tag-333)
Locus_13730_Transcript_1/1_Conf_1.000	460	16	3.47E-15	NP_495156.1	66	84.7297	112	74	TBC (Tre-2/Bub2/Cdc16) domain family member (tbc-2)
Locus_13731_Transcript_1/1_Conf_1.000	397	2	1.40E-11	XP_002640811.1	52	72.7886	116	61	Hypothetical protein CBG15695
Locus_13732_Transcript_1/1_Conf_1.000	180	0							
Locus_13733_Transcript_1/1_Conf_1.000	256	0							
Locus_13734_Transcript_1/1_Conf_1.000	220	6	1.46E-08	EFO21023.1	70	62.7734	51	36	hypothetical protein LOAG_07466
Locus_13735_Transcript_1/1_Conf_1.000	770	0							
Locus_13736_Transcript_1/1_Conf_1.000	187	5	2.16E-28	NP_502312.2	98	128.642	62	61	Neurexin Like receptor family member (nlr-1)
Locus_13737_Transcript_1/1_Conf_1.000	158	20	1.07E-11	CAQ48388.2	80	73.1738	51	41	C. elegans protein C26H9A.1b, confirmed by transcript evidence
Locus_13738_Transcript_1/1_Conf_1.000	948	19	1.20E-35	NP_001129935.1	55	154.836	229	128	SMAll family member (sma-9)
Locus_13739_Transcript_1/1_Conf_1.000	376	20	9.52E-53	XP_001086913.2	100	209.534	103	103	PREDICTED: translocon-associated protein subunit delta isoform 2
Locus_1374_Transcript_1/2_Conf_1.000	513	1	3.58E-05	CAR63546.1	52	51.6026	93	49	hypothetical protein
Locus_1374_Transcript_2/2_Conf_1.000	404	1	3.31E-05	CAR63546.1	52	51.6026	93	49	hypothetical protein
Locus_13740_Transcript_1/1_Conf_1.000	975	13	5.06E-29	XP_002634908.1	89	132.88	85	76	Hypothetical protein CBG22506
Locus_13741_Transcript_1/1_Conf_1.000	253	0							
Locus_13742_Transcript_1/1_Conf_1.000	404	0							
Locus_13743_Transcript_1/1_Conf_1.000	134	0							
Locus_13744_Transcript_1/1_Conf_1.000	454	0							
Locus_13745_Transcript_1/1_Conf_1.000	198	2	4.33E-05	NP_497805.2	86	51.2174	36	31	hypothetical protein C36E8.4

Locus_13746_Transcript_1/1_Conf_1.000	413	20	2.25E-22	NP_001153506.1	73	108.612	87	64	uridine-cytidine kinase 2
Locus_13747_Transcript_1/2_Conf_1.000	273	0							
Locus_13747_Transcript_2/2_Conf_1.000	273	0							
Locus_13748_Transcript_1/1_Conf_1.000	357	0							
Locus_13749_Transcript_1/1_Conf_1.000	547	0							
Locus_1375_Transcript_1/4_Conf_0.667	3239	2	4.99E-10	XP_001891820.1	52	72.0182	114	60	GRAM domain containing protein
Locus_1375_Transcript_2/4_Conf_0.667	3293	2	5.08E-10	XP_001891820.1	52	72.0182	114	60	GRAM domain containing protein
Locus_1375_Transcript_3/4_Conf_0.667	3293	2	5.08E-10	XP_001891820.1	52	72.0182	114	60	GRAM domain containing protein
Locus_1375_Transcript_4/4_Conf_0.667	3293	2	5.08E-10	XP_001891820.1	52	72.0182	114	60	GRAM domain containing protein
Locus_13750_Transcript_1/1_Conf_1.000	134	0							
Locus_13751_Transcript_1/1_Conf_1.000	332	4	1.17E-10	NP_498501.1	62	69.707	114	71	hypothetical protein T07E3.4
Locus_13752_Transcript_1/1_Conf_1.000	227	0							
Locus_13753_Transcript_1/3_Conf_0.667	916	20	1.38E-118	XP_002639923.1	89	430.254	257	230	C. briggsae CBR-JPH-1 protein
Locus_13753_Transcript_2/3_Conf_0.667	865	20	5.30E-109	XP_002639923.1	89	398.282	239	213	C. briggsae CBR-JPH-1 protein
Locus_13753_Transcript_3/3_Conf_0.667	865	20	5.30E-109	XP_002639923.1	89	398.282	239	213	C. briggsae CBR-JPH-1 protein
Locus_13754_Transcript_1/1_Conf_1.000	135	0							
Locus_13755_Transcript_1/1_Conf_1.000	691	20	5.60E-35	XP_002631614.1	57	151.754	238	136	C. briggsae CBR-MAC-1 protein
Locus_13756_Transcript_1/1_Conf_1.000	211	0							
Locus_13757_Transcript_1/1_Conf_1.000	1383	10	1.47E-113	ACP34146.1	70	414.461	435	306	Hypothetical protein F56B3.4a
Locus_13758_Transcript_1/1_Conf_1.000	534	9	2.54E-07	XP_002639002.1	62	58.9214	56	35	Hypothetical protein CBG22248
Locus_13759_Transcript_1/1_Conf_1.000	303	0							
Locus_1376_Transcript_1/1_Conf_1.000	526	2	3.26E-60	CAR63590.1	72	234.572	170	123	hypothetical protein
Locus_13760_Transcript_1/1_Conf_1.000	340	0							
Locus_13761_Transcript_1/1_Conf_1.000	1057	0							

Locus_13762_Transcript_1/1_Conf_1.000	429	20	5.57E-29	EFO19478.1	78	130.568	96	75	U5 snRNP-associated protein
Locus_13763_Transcript_1/1_Conf_1.000	336	20	1.66E-33	XP_002631318.1	79	145.591	106	84	Hypothetical protein CBG03140
Locus_13764_Transcript_1/1_Conf_1.000	452	20	6.84E-19	NP_491248.1	90	97.0561	55	50	hypothetical protein T03F1.1
Locus_13765_Transcript_1/1_Conf_1.000	977	20	7.97E-75	CAR63535.1	67	285.034	309	208	putative transcription factor yin yang 2
Locus_13766_Transcript_1/1_Conf_1.000	439	20	2.45E-53	XP_002631343.1	88	211.46	143	127	C. briggsae CBR-SGN-1 protein
Locus_13767_Transcript_1/1_Conf_1.000	984	20	5.42E-79	XP_002631146.1	64	298.901	343	221	Hypothetical protein CBG02930
Locus_13768_Transcript_1/1_Conf_1.000	356	6	1.25E-12	NP_741018.1	65	76.2554	85	56	Homeobox family member (ceh-38)
Locus_13769_Transcript_1/1_Conf_1.000	810	20	8.08E-61	XP_002630102.1	69	238.039	270	187	Hypothetical protein CBG13485
Locus_1377_Transcript_1/1_Conf_1.000	2561	4	1.15E-14	XP_002638877.1	38	87.0409	708	272	Hypothetical protein CBG22096
Locus_13770_Transcript_1/1_Conf_1.000	256	0							

Locus_13771_Transcript_1/1_Conf_1.000	942	20	7.05E-97	NP_001129916.1	77	358.221	294	227	TWiK family of potassium channels family member (twk-46)
Locus_13772_Transcript_1/1_Conf_1.000	235	0							
Locus_13773_Transcript_1/1_Conf_1.000	143	20	2.56E-18	XP_002816314.1	100	95.1301	47	47	PREDICTED: PRELI domain-containing protein 1, mitochondrial-like isoform 2
Locus_13774_Transcript_1/1_Conf_1.000	930	20	3.52E-109	NP_494780.1	84	399.053	301	254	Temporarily Assigned Gene name family member (tag-231)
Locus_13775_Transcript_1/1_Conf_1.000	232	20	1.06E-27	NP_497182.2	89	126.331	76	68	hypothetical protein Y50D7A.2
Locus_13776_Transcript_1/1_Conf_1.000	466	0							
Locus_13777_Transcript_1/1_Conf_1.000	268	4	1.17E-10	NP_497704.1	57	69.707	88	51	hypothetical protein F59A2.5
Locus_13778_Transcript_1/1_Conf_1.000	543	20	4.59E-15	XP_421797.2	63	84.7297	110	70	PREDICTED: hypothetical protein
Locus_13779_Transcript_1/1_Conf_1.000	130	3	1.99E-10	NP_491646.3	80	68.9366	42	34	hypothetical protein F46F11.7
Locus_1378_Transcript_1/1_Conf_1.000	170	0							
Locus_13780_Transcript_1/1_Conf_1.000	495	4	1.84E-32	NP_497919.1	73	142.124	110	81	hypothetical protein M88.2
Locus_13781_Transcript_1/1_Conf_1.000	334	0							
Locus_13782_Transcript_1/1_Conf_1.000	157	0							
Locus_13783_Transcript_1/1_Conf_1.000	436	20	4.38E-26	XP_002821384.1	100	120.939	62	62	PREDICTED: 60S acidic ribosomal protein P2-like
Locus_13784_Transcript_1/1_Conf_1.000	550	20	5.08E-25	NP_491930.1	76	117.857	90	69	DiHydroOrotate Dehydrogenas family member (dhod-1)
Locus_13785_Transcript_1/1_Conf_1.000	444	20	7.37E-21	XP_002639995.1	62	103.605	147	92	C. briggsae CBR-ELPC-1 protein
Locus_13786_Transcript_1/1_Conf_1.000	406	3	2.79E-04	A8WRV1.2	56	48.521	81	46	Serine/threonine-protein kinase kin-29
Locus_13787_Transcript_1/1_Conf_1.000	478	0							

Locus_13788_Transcript_1/1_Conf_1.000	692	20	1.49E-120	AAL06642.1	97	436.032	230	225	serine-threonine protein kinase
Locus_13789_Transcript_1/1_Conf_1.000	1181	0							
Locus_1379_Transcript_1/1_Conf_1.000	700	0							
Locus_13790_Transcript_1/1_Conf_1.000	572	20	1.12E-49	CBA11992.1	68	199.904	187	128	endonuclease-reverse transcriptase HmRTE-e01
Locus_13791_Transcript_1/1_Conf_1.000	437	4	1.04E-27	NP_001023419.1	78	126.331	104	82	hypothetical protein W03F8.10
Locus_13792_Transcript_1/1_Conf_1.000	690	5	1.80E-33	XP_002641216.1	64	146.747	167	107	Hypothetical protein CBG09080
Locus_13793_Transcript_1/1_Conf_1.000	214	0							
Locus_13794_Transcript_1/1_Conf_1.000	266	0							
Locus_13795_Transcript_1/1_Conf_1.000	1514	20	9.32E-133	Q24702.1	75	478.404	453	342	DVA-1 polyprotein
Locus_13796_Transcript_1/1_Conf_1.000	164	0							
Locus_13797_Transcript_1/1_Conf_1.000	355	20	3.08E-11	NP_001122952.1	67	71.633	70	47	hypothetical protein F53H10.2
Locus_13798_Transcript_1/1_Conf_1.000	232	0							
Locus_13799_Transcript_1/1_Conf_1.000	525	20	4.24E-68	NP_509350.2	96	260.766	125	120	hypothetical protein C34D10.2

Locus_138_Transcript_1/1_Conf_1.000	2455	20	3.09E-110	EFO16992.1	80	404.445	298	241	CCR4-NOT transcription complex component
Locus_1380_Transcript_1/2_Conf_1.000	1408	20	5.30E-119	NP_492252.1	71	432.565	410	292	hypothetical protein T01G9.2
Locus_1380_Transcript_2/2_Conf_1.000	1408	20	2.63E-118	NP_492252.1	70	430.254	410	291	hypothetical protein T01G9.2
Locus_13800_Transcript_1/1_Conf_1.000	365	20	1.07E-56	XP_001135032.1	99	222.631	121	120	PREDICTED: similar to Ribosomal protein L14
Locus_13801_Transcript_1/1_Conf_1.000	424	0							
Locus_13802_Transcript_1/1_Conf_1.000	148	0							
Locus_13803_Transcript_1/1_Conf_1.000	372	4	3.54E-23	NP_493560.1	77	111.309	87	67	hypothetical protein K05C4.11
Locus_13804_Transcript_1/1_Conf_1.000	177	0							
Locus_13805_Transcript_1/1_Conf_1.000	179	0							
Locus_13806_Transcript_1/2_Conf_1.000	360	1	2.77E-04	XP_002634943.1	44	48.521	109	48	Hypothetical protein CBG22545
Locus_13806_Transcript_2/2_Conf_1.000	1075	20	5.55E-11	XP_002634943.1	42	73.1738	198	85	Hypothetical protein CBG22545
Locus_13807_Transcript_1/1_Conf_1.000	432	0							
Locus_13808_Transcript_1/1_Conf_1.000	533	4	3.53E-41	XP_002636840.1	70	171.4	178	126	Hypothetical protein CBG09291
Locus_13809_Transcript_1/1_Conf_1.000	134	0							
Locus_1381_Transcript_1/1_Conf_1.000	541	0							
Locus_13810_Transcript_1/1_Conf_1.000	171	2	4.73E-04	CAX65058.1	66	47.7506	48	32	C. elegans protein F55D12.2b, confirmed by transcript evidence
Locus_13811_Transcript_1/1_Conf_1.000	1124	20	2.78E-101	NP_001021584.1	74	373.244	285	212	EXOnuclease family member (exo-3)
Locus_13812_Transcript_1/1_Conf_1.000	199	1	2.15E-04	XP_002643566.1	73	48.9062	46	34	Hypothetical protein CBG16268
Locus_13813_Transcript_1/1_Conf_1.000	165	0							
Locus_13814_Transcript_1/1_Conf_1.000	982	2	1.04E-13	NP_504241.1	75	82.0333	68	51	hypothetical protein Y61A9LA.3
Locus_13815_Transcript_1/1_Conf_1.000	665	20	4.07E-109	NP_510020.2	91	397.897	221	203	hypothetical protein W03G11.3
Locus_13816_Transcript_1/1_Conf_1.000	1056	20	4.60E-103	NP_490719.1	71	379.022	343	244	hypothetical protein Y48G1A.3
Locus_13817_Transcript_1/1_Conf_1.000	401	0							
Locus_13818_Transcript_1/1_Conf_1.000	192	0							
Locus_13819_Transcript_1/1_Conf_1.000	726	2	6.69E-05	XP_001901708.1	47	51.9878	134	63	F-box domain containing protein

Locus_1382_Transcript_1/1_Conf_1.000	672	20	1.80E-51	XP_002644536.1	88	206.453	125	110	C. briggsae CBR-DPY-23 protein
Locus_13820_Transcript_1/2_Conf_1.000	434	20	8.58E-22	NP_497868.1	79	106.686	74	59	Temporarily Assigned Gene name family member (tag-131)
Locus_13820_Transcript_2/2_Conf_1.000	434	20	8.58E-22	NP_497868.1	79	106.686	74	59	Temporarily Assigned Gene name family member (tag-131)
Locus_13821_Transcript_1/1_Conf_1.000	332	0							
Locus_13822_Transcript_1/1_Conf_1.000	682	20	4.67E-87		90	324.709	199	180	hypothetical protein C25H3.9 - Caenorhabditis elegans
Locus_13823_Transcript_1/1_Conf_1.000	142	4	6.99E-08	NP_500284.2	84	60.4622	39	33	Enhancer of EfL-1 mutant phenotype family member (eel-1)
Locus_13824_Transcript_1/1_Conf_1.000	660	0							
Locus_13825_Transcript_1/1_Conf_1.000	651	20	1.20E-65	ADE60684.1	78	253.447	182	142	Diacylglycerol kinase protein 4, isoform b, partially confirmed by transcript evidence
Locus_13826_Transcript_1/1_Conf_1.000	757	20	7.03E-93	NP_496968.1	90	344.354	202	183	hypothetical protein Y48B6A.12
Locus_13827_Transcript_1/1_Conf_1.000	327	0							
Locus_13828_Transcript_1/1_Conf_1.000	1116	20	1.07E-129	NP_497714.2	83	467.618	304	254	Protein PHosphatase family member (pph-6)
Locus_13829_Transcript_1/1_Conf_1.000	561	20	8.25E-18	XP_392369.1	97	93.9745	43	42	PREDICTED: similar to Protein yippee-like 1
Locus_1383_Transcript_1/1_Conf_1.000	1683	0							
Locus_13830_Transcript_1/1_Conf_1.000	213	0							
Locus_13831_Transcript_1/1_Conf_1.000	418	20	3.56E-23	XP_001893552.1	75	111.309	86	65	haloacid dehalogenase-like hydrolase domain containing 2, putative
Locus_13832_Transcript_1/1_Conf_1.000	353	20	2.87E-41	3MNG	100	171.4	85	85	Wild Type Human Prxv With Dtt Bound As A Competitive Inhibitor

Locus_13833_Transcript_1/1_Conf_1.000	229	20	2.45E-32	NP_741109.1	90	141.739	75	68	FERM domain (protein4.1-ezrin-radixin-moesin) family member (frm-8)
Locus_13834_Transcript_1/1_Conf_1.000	296	20	4.02E-35	XP_002632369.1	87	150.984	98	86	C. briggsae CBR-PMP-4 protein
Locus_13835_Transcript_1/1_Conf_1.000	667	0							
Locus_13836_Transcript_1/1_Conf_1.000	332	2	1.48E-05	XP_002643723.1	62	52.7582	53	33	Hypothetical protein CBG01914
Locus_13837_Transcript_1/1_Conf_1.000	880	20	2.17E-33	XP_001984068.1	84	147.132	82	69	GH15211
Locus_13838_Transcript_1/1_Conf_1.000	858	20	2.08E-73	XP_001894604.1	67	280.026	273	185	exonuclease family protein
Locus_13839_Transcript_1/1_Conf_1.000	657	0							
Locus_1384_Transcript_1/1_Conf_1.000	466	20	3.86E-62	BAG54741.1	100	240.736	146	146	unnamed protein product
Locus_13840_Transcript_1/1_Conf_1.000	150	0							
Locus_13841_Transcript_1/2_Conf_1.000	295	0							
Locus_13841_Transcript_2/2_Conf_1.000	295	0							
Locus_13842_Transcript_1/1_Conf_1.000	557	20	9.60E-27	NP_495774.1	58	123.635	176	103	hypothetical protein F14E5.4
Locus_13843_Transcript_1/1_Conf_1.000	377	20	1.75E-30	NP_509361.1	81	135.576	124	101	hypothetical protein K07E3.4
Locus_13844_Transcript_1/1_Conf_1.000	179	3	9.46E-13	NP_495112.1	77	76.6406	57	44	hypothetical protein C25H3.11

Locus_13845_Transcript_1/1_Conf_1.000	298	20	1.09E-24	XP_002632973.1	78	116.316	84	66	Hypothetical protein CBG21733
Locus_13846_Transcript_1/1_Conf_1.000	461	0							
Locus_13847_Transcript_1/1_Conf_1.000	397	0							
Locus_13848_Transcript_1/1_Conf_1.000	142	0							
Locus_13849_Transcript_1/1_Conf_1.000	131	5	3.29E-05	AAK71410.2	63	51.6026	41	26	AC024881_1Hypothetical protein Y97E10B.1
Locus_1385_Transcript_1/1_Conf_1.000	537	3	2.68E-04	XP_002939048.1	47	48.9062	130	62	PREDICTED: macrophage mannose receptor 1-like
Locus_13850_Transcript_1/2_Conf_1.000	385	20	1.37E-27	XP_002128408.1	93	125.946	91	85	PREDICTED: similar to U6 snRNA-associated Sm-like protein LSm3
Locus_13850_Transcript_2/2_Conf_1.000	385	20	1.37E-27	XP_002128408.1	93	125.946	91	85	PREDICTED: similar to U6 snRNA-associated Sm-like protein LSm3
Locus_13851_Transcript_1/2_Conf_1.000	585	20	3.31E-55	XP_002632393.1	73	218.394	192	142	Hypothetical protein CBG00415
Locus_13851_Transcript_2/2_Conf_1.000	585	20	3.31E-55	XP_002632393.1	73	218.394	192	142	Hypothetical protein CBG00415
Locus_13852_Transcript_1/1_Conf_1.000	430	20	1.72E-46	NP_001020988.1	81	188.734	137	111	UNCoordinated family member (unc-89)
Locus_13853_Transcript_1/2_Conf_1.000	598	20	2.85E-25	XP_002740677.1	70	119.013	104	73	PREDICTED: cyclin-dependent kinase 2-like
Locus_13853_Transcript_2/2_Conf_1.000	276	0							
Locus_13854_Transcript_1/1_Conf_1.000	259	20	3.42E-34	NP_492433.1	86	147.902	86	74	HomoGentisate Oxidase family member (hgo-1)
Locus_13855_Transcript_1/1_Conf_1.000	1287	20	3.91E-57	NP_001020983.1	57	226.868	405	233	TOLl (Drosophila) family member (tol-1)
Locus_13856_Transcript_1/1_Conf_1.000	453	20	7.75E-31	XP_002648358.1	84	136.732	92	78	Hypothetical protein CBG24564
Locus_13857_Transcript_1/1_Conf_1.000	212	0							

Locus_13858_Transcript_1/1_Conf_1.000	243	20	3.05E-19	XP_002632237.1	81	98.2117	64	52	Hypothetical protein CBG07111
Locus_13859_Transcript_1/1_Conf_1.000	609	20	9.41E-11	XP_001899546.1	44	70.8626	207	93	hypothetical protein
Locus_1386_Transcript_1/1_Conf_1.000	1651	20	2.99E-180	XP_002639432.1	74	636.336	483	361	C. briggsae CBR-TYR-4 protein
Locus_13860_Transcript_1/1_Conf_1.000	677	20	1.95E-37	NP_504260.1	65	159.844	177	116	TAF (TBP-associated transcription factor) family member (taf-10)
Locus_13861_Transcript_1/1_Conf_1.000	217	20	6.31E-17	DAA16569.1	100	90.5077	72	72	heat shock protein HSP 90-beta
Locus_13862_Transcript_1/1_Conf_1.000	313	0							
Locus_13863_Transcript_1/1_Conf_1.000	762	20	1.68E-142	NP_001164609.1	100	509.22	239	239	clusterin isoform 3
Locus_13864_Transcript_1/1_Conf_1.000	552	0							
Locus_13865_Transcript_1/1_Conf_1.000	268	0							
Locus_13866_Transcript_1/1_Conf_1.000	440	20	8.78E-27	XP_002633460.1	71	123.25	142	101	Hypothetical protein CBG06228
Locus_13867_Transcript_1/1_Conf_1.000	1178	20	3.95E-37	NP_492228.1	61	160.229	210	129	MAternally affected Uncoordination family member (mau-2)
Locus_13868_Transcript_1/1_Conf_1.000	246	4	1.91E-13	NP_497420.1	77	78.9518	61	47	hypothetical protein Y67D2.6

Locus_13869_Transcript_1/1_Conf_1.000	157	20	3.11E-11	NP_491902.3	88	71.633	44	39	hypothetical protein D2092.5
Locus_1387_Transcript_1/3_Conf_0.286	322	0							
Locus_1387_Transcript_2/3_Conf_0.714	1422	20	5.15E-37	NP_502566.1	47	160.229	381	182	Muscle EXcess family member (mex-5)
Locus_1387_Transcript_3/3_Conf_0.714	1422	20	5.15E-37	NP_502566.1	47	160.229	381	182	Muscle EXcess family member (mex-5)
Locus_13870_Transcript_1/1_Conf_1.000	399	2	2.62E-18	XP_002642086.1	78	95.1301	132	104	Hypothetical protein CBG18027
Locus_13871_Transcript_1/1_Conf_1.000	227	0							
Locus_13872_Transcript_1/1_Conf_1.000	233	20	1.87E-32	NP_502000.1	90	142.124	76	69	hypothetical protein C06G8.1
Locus_13873_Transcript_1/1_Conf_1.000	672	20	1.73E-62	NP_492446.3	76	243.047	221	168	hypothetical protein ZK858.1
Locus_13874_Transcript_1/1_Conf_1.000	379	20	2.51E-29	XP_002644672.1	69	131.724	126	87	C. briggsae CBR-VPS-41 protein
Locus_13875_Transcript_1/1_Conf_1.000	204	0							
Locus_13876_Transcript_1/1_Conf_1.000	911	20	1.89E-35	NP_509877.2	54	154.066	266	144	hypothetical protein C34F6.10
Locus_13877_Transcript_1/1_Conf_1.000	671	0							
Locus_13878_Transcript_1/1_Conf_1.000	302	20	5.59E-21	XP_002632832.1	53	103.99	143	76	Hypothetical protein CBG15024

Locus_13879_Transcript_1/1_Conf_1.000	425	20	6.64E-30	NP_001040880.1	79	133.65	89	71	hypothetical protein Y39A3CL.4
Locus_1388_Transcript_1/1_Conf_1.000	941	0							
Locus_13880_Transcript_1/1_Conf_1.000	410	0							
Locus_13881_Transcript_1/1_Conf_1.000	358	20	1.51E-34	Q9BI88.2	74	149.058	109	81	UPF0587 protein F46B6.12
Locus_13882_Transcript_1/1_Conf_1.000	520	20	8.83E-39	NP_001021589.1	64	163.31	171	111	hypothetical protein R11A5.4
Locus_13883_Transcript_1/1_Conf_1.000	185	0							
Locus_13884_Transcript_1/1_Conf_1.000	655	0							
Locus_13885_Transcript_1/1_Conf_1.000	213	0							
Locus_13886_Transcript_1/1_Conf_1.000	345	2	7.44E-05	XP_002647702.1	55	50.447	93	52	Hypothetical protein CBG17891
Locus_13887_Transcript_1/1_Conf_1.000	176	0							
Locus_13888_Transcript_1/1_Conf_1.000	497	1	2.77E-04	ABI21593.1	53	39.2762	45	24	hypothetical protein
Locus_13889_Transcript_1/1_Conf_1.000	309	20	2.74E-28	EFO23898.1	74	128.257	99	74	FolC protein
Locus_1389_Transcript_1/1_Conf_1.000	567	20	2.84E-13	XP_002637364.1	50	78.9518	183	93	Hypothetical protein CBG19064
Locus_13890_Transcript_1/1_Conf_1.000	692	0							
Locus_13891_Transcript_1/1_Conf_1.000	162	0							
Locus_13892_Transcript_1/1_Conf_1.000	203	0							
Locus_13893_Transcript_1/1_Conf_1.000	295	20	5.80E-34	XP_002636626.1	90	147.132	98	89	C. briggsae CBR-AMAN-2 protein
Locus_13894_Transcript_1/1_Conf_1.000	392	20	2.02E-31	XP_001898856.1	77	138.658	129	100	cation channel family protein
Locus_13895_Transcript_1/1_Conf_1.000	814	0							

Locus_13896_Transcript_1/1_Conf_1.000	324	20	7.55E-42	XP_002629880.1	84	173.326	104	88	Hypothetical protein CBG21917
Locus_13897_Transcript_1/1_Conf_1.000	347	0							
Locus_13898_Transcript_1/1_Conf_1.000	498	4	1.19E-39	NP_498063.1	71	166.007	151	108	hypothetical protein R144.11
Locus_13899_Transcript_1/1_Conf_1.000	395	20	1.22E-31	XP_002642778.1	70	139.428	131	93	C. briggsae CBR-PRMT-5 protein
Locus_139_Transcript_1/1_Conf_1.000	1544	20	8.25E-44	XP_002647887.1	61	182.956	239	148	Hypothetical protein CBG23753
Locus_1390_Transcript_1/2_Conf_1.000	711	5	1.67E-21	XP_002641213.1	77	107.071	80	62	C. briggsae CBR-MDT-15 protein
Locus_1390_Transcript_2/2_Conf_1.000	893	9	4.64E-39	XP_001902687.1	67	166.007	154	104	hypothetical protein

Locus_13900_Transcript_1/1_Conf_1.000	797	9	1.41E-17	NP_491541.1	64	94.3597	102	66	hypothetical protein F56A3.2
Locus_13901_Transcript_1/1_Conf_1.000	315	20	6.52E-09	XP_002113412.1	57	63.929	97	56	hypothetical protein TRIADDRAFT_57554
Locus_13902_Transcript_1/2_Conf_1.000	899	20	1.09E-112	XP_002631657.1	88	410.609	261	230	Hypothetical protein CBG20848
Locus_13902_Transcript_2/2_Conf_1.000	874	20	5.41E-109	XP_002631657.1	88	398.282	252	222	Hypothetical protein CBG20848
Locus_13903_Transcript_1/1_Conf_1.000	186	0							
Locus_13904_Transcript_1/2_Conf_1.000	251	0							
Locus_13904_Transcript_2/2_Conf_1.000	257	0							
Locus_13905_Transcript_1/2_Conf_1.000	627	20	4.31E-38	XP_002640628.1	60	161.77	200	120	C. briggsae CBR-EAT-18 protein
Locus_13905_Transcript_2/2_Conf_1.000	483	20	3.30E-45	XP_002640628.1	78	184.496	152	120	C. briggsae CBR-EAT-18 protein
Locus_13906_Transcript_1/1_Conf_1.000	648	20	2.51E-23	NP_001129749.1	95	112.849	60	57	abnormal cell LINeage family member (lin-10)
Locus_13907_Transcript_1/1_Conf_1.000	484	20	7.31E-61	XP_002642567.1	89	236.498	161	144	C. briggsae CBR-UNC-79 protein
Locus_13908_Transcript_1/1_Conf_1.000	132	0							
Locus_13909_Transcript_1/1_Conf_1.000	200	0							
Locus_1391_Transcript_1/1_Conf_1.000	311	0							
Locus_13910_Transcript_1/1_Conf_1.000	264	0							
Locus_13911_Transcript_1/1_Conf_1.000	293	20	6.04E-23	AAN05752.1	75	110.538	95	72	heat shock protein 20
Locus_13912_Transcript_1/1_Conf_1.000	318	20	2.80E-28	XP_002645500.1	70	128.257	106	75	Hypothetical protein CBG22750
Locus_13913_Transcript_1/1_Conf_1.000	372	20	2.73E-07	NP_491642.2	53	58.5362	129	69	hypothetical protein F46F11.9
Locus_13914_Transcript_1/1_Conf_1.000	368	0							
Locus_13915_Transcript_1/1_Conf_1.000	589	20	6.00E-57	XP_002644776.1	74	224.172	199	149	Hypothetical protein CBG14789
Locus_13916_Transcript_1/1_Conf_1.000	458	0							
Locus_13917_Transcript_1/1_Conf_1.000	131	0							
Locus_13918_Transcript_1/1_Conf_1.000	580	20	2.62E-65	NP_508919.2	77	251.906	192	148	hypothetical protein C03F11.3
Locus_13919_Transcript_1/1_Conf_1.000	207	0							
Locus_1392_Transcript_1/1_Conf_1.000	946	20	4.19E-65	XP_001623486.1	65	252.677	310	204	predicted protein

Locus_13920_Transcript_1/1_Conf_1.000	755	5	3.21E-05	XP_001895157.1	63	53.1434	55	35	TAZ zinc finger family protein
Locus_13921_Transcript_1/1_Conf_1.000	1344	20	7.87E-165	XP_002636651.1	78	584.719	451	353	C. briggsae CBR-DRS-2 protein
Locus_13922_Transcript_1/1_Conf_1.000	264	0							
Locus_13923_Transcript_1/1_Conf_1.000	325	0							
Locus_13924_Transcript_1/1_Conf_1.000	616	20	1.29E-39	XP_002642330.1	72	166.777	153	111	Hypothetical protein CBG18325
Locus_13925_Transcript_1/1_Conf_1.000	191	0							
Locus_13926_Transcript_1/1_Conf_1.000	254	20	1.04E-38	A8XJL7.2	97	162.925	84	82	Serine/threonine-protein kinase sax-1
Locus_13927_Transcript_1/2_Conf_1.000	1105	20	1.87E-110	XP_002634178.1	79	403.675	311	246	Hypothetical protein CBG01746
Locus_13927_Transcript_2/2_Conf_1.000	1106	20	2.45E-110	XP_002634178.1	79	403.29	311	246	Hypothetical protein CBG01746
Locus_13928_Transcript_1/1_Conf_1.000	833	1	3.29E-04	XP_002405770.1	40	50.0618	170	68	secreted mucin MUC17, putative
Locus_13929_Transcript_1/1_Conf_1.000	213	0							
Locus_1393_Transcript_1/1_Conf_1.000	509	2	1.33E-04	NP_683559.2	42	49.6766	118	50	RNA recognition motif (RRM)-containing protein
Locus_13930_Transcript_1/1_Conf_1.000	703	20	1.54E-19	NP_001076765.1	70	100.523	100	70	Phasmid Socket Absent family member (psa-3)

Locus_13931_Transcript_1/1_Conf_1.000	712	20	2.78E-101	NP_001024342.1	92	372.089	237	219	Aboc, EXpulsion defective family member (aex-3)
Locus_13932_Transcript_1/1_Conf_1.000	458	20	3.95E-43	EFO23801.1	79	177.563	144	114	replication factor C
Locus_13933_Transcript_1/1_Conf_1.000	275	2	1.24E-12	NP_495888.2	62	76.2554	88	55	hypothetical protein T07D4.1
Locus_13934_Transcript_1/1_Conf_1.000	348	20	4.60E-39	NP_491924.1	79	164.081	116	92	hypothetical protein W02D3.4
Locus_13935_Transcript_1/1_Conf_1.000	195	0							
Locus_13936_Transcript_1/1_Conf_1.000	145	2	1.46E-08	NP_501792.2	70	62.7734	47	33	abnormal GONad development family member (gon-1)
Locus_13937_Transcript_1/1_Conf_1.000	151	0							
Locus_13938_Transcript_1/1_Conf_1.000	594	0							
Locus_13939_Transcript_1/1_Conf_1.000	205	10	3.63E-12	XP_002639106.1	73	74.7146	63	46	Hypothetical protein CBG14925
Locus_1394_Transcript_1/1_Conf_1.000	936	20	7.42E-107	NP_501650.1	76	391.349	301	230	hypothetical protein C10C5.3
Locus_13940_Transcript_1/1_Conf_1.000	455	0							
Locus_13941_Transcript_1/1_Conf_1.000	427	20	4.60E-15	NP_495055.1	57	84.3445	143	82	hypothetical protein ZK177.8
Locus_13942_Transcript_1/1_Conf_1.000	184	0							
Locus_13944_Transcript_1/1_Conf_1.000	185	4	1.84E-11	XP_002630706.1	74	72.4034	63	47	C. briggsae CBR-MOG-5 protein
Locus_13945_Transcript_1/1_Conf_1.000	260	3	6.93E-19	XP_002638537.1	90	97.0561	52	47	Hypothetical protein CBG05572
Locus_13946_Transcript_1/1_Conf_1.000	534	0							

Locus_13947_Transcript_1/1_Conf_1.000	398	20	9.86E-66	EFO27677.1	96	252.677	130	126	hypothetical protein LOAG_00808
Locus_13948_Transcript_1/1_Conf_1.000	324	0							
Locus_13949_Transcript_1/1_Conf_1.000	151	0							
Locus_1395_Transcript_1/2_Conf_1.000	820	20	1.15E-62	NP_498755.1	81	244.202	178	145	hypothetical protein K12H4.4
Locus_1395_Transcript_2/2_Conf_1.000	821	20	1.15E-62	NP_498755.1	81	244.202	178	145	hypothetical protein K12H4.4
Locus_13950_Transcript_1/2_Conf_1.000	431	20	2.96E-30	XP_002641823.1	70	134.806	144	101	Hypothetical protein CBG16490
Locus_13950_Transcript_2/2_Conf_1.000	423	20	1.13E-29	XP_002641823.1	70	132.88	141	99	Hypothetical protein CBG16490
Locus_13951_Transcript_1/1_Conf_1.000	537	0							
Locus_13952_Transcript_1/1_Conf_1.000	145	0							
Locus_13953_Transcript_1/1_Conf_1.000	170	0							
Locus_13954_Transcript_1/1_Conf_1.000	461	20	3.14E-16	XP_002612934.1	55	88.1965	102	57	hypothetical protein BRAFLDRAFT_278667
Locus_13955_Transcript_1/1_Conf_1.000	887	20	8.92E-43	XP_002665162.1	55	178.333	271	150	PREDICTED: kelch-like 10-like
Locus_13956_Transcript_1/1_Conf_1.000	440	0							
Locus_13957_Transcript_1/1_Conf_1.000	135	4	7.50E-18	EFO16626.1	100	93.5893	44	44	INX-1 protein
Locus_13958_Transcript_1/1_Conf_1.000	141	0							

Locus_13959_Transcript_1/1_Conf_1.000	216	20	2.56E-18	XP_002640829.1	75	95.1301	70	53	C. briggsae CBR-ADS-1 protein
Locus_1396_Transcript_1/1_Conf_1.000	1615	6	2.82E-50	EFO24916.1	48	204.527	512	246	hypothetical protein LOAG_03568
Locus_13960_Transcript_1/1_Conf_1.000	240	0							
Locus_13961_Transcript_1/1_Conf_1.000	399	20	4.43E-50	NP_001022450.1	86	200.675	132	114	hypothetical protein Y48E1A.1
Locus_13962_Transcript_1/1_Conf_1.000	170	0							
Locus_13963_Transcript_1/1_Conf_1.000	425	20	7.53E-58	EFO26325.1	85	226.483	140	119	hypothetical protein LOAG_02163
Locus_13964_Transcript_1/1_Conf_1.000	186	0							
Locus_13965_Transcript_1/1_Conf_1.000	131	20	6.39E-17	XP_002713121.1	100	90.5077	43	43	PREDICTED: beta-catenin

Locus_13966_Transcript_1/3_Conf_0.500	1025	20	1.53E-55	NP_510746.2	69	221.09	199	138	hypothetical protein K02H8.1
Locus_13966_Transcript_2/3_Conf_0.333	627	20	1.06E-60	NP_001129932.1	70	236.884	211	149	hypothetical protein K02H8.1
Locus_13966_Transcript_3/3_Conf_0.500	692	8	1.26E-10	NP_001129931.1	60	70.8626	83	50	hypothetical protein K02H8.1
Locus_13967_Transcript_1/1_Conf_1.000	207	0							
Locus_13968_Transcript_1/1_Conf_1.000	237	20	4.15E-16	XP_002632592.1	71	87.8113	78	56	C. briggsae CBR-EPS-8 protein
Locus_13969_Transcript_1/1_Conf_1.000	477	20	2.56E-42	CAN99691.2	65	174.866	167	110	C. elegans protein F54B3.1b, partially confirmed by transcript evidence
Locus_1397_Transcript_1/2_Conf_1.000	3125	20	4.35E-104	NP_001041165.1	56	384.415	707	401	hypothetical protein T09E8.1
Locus_1397_Transcript_2/2_Conf_1.000	1860	20	6.13E-68	XP_002636951.1	64	263.462	357	230	Hypothetical protein CBG09427
Locus_13970_Transcript_1/1_Conf_1.000	162	3	8.69E-14	NP_508964.2	90	80.1073	52	47	Regulator of G protein Signaling family member (rgs-6)
Locus_13971_Transcript_1/1_Conf_1.000	134	0							
Locus_13972_Transcript_1/1_Conf_1.000	379	1	8.70E-06	XP_001892617.1	55	53.5286	58	32	Protein kinase domain containing protein
Locus_13973_Transcript_1/1_Conf_1.000	980	0							
Locus_13974_Transcript_1/1_Conf_1.000	259	0							
Locus_13975_Transcript_1/1_Conf_1.000	256	5	1.11E-08	XP_002639252.1	94	63.1586	39	37	C. briggsae CBR-GFI-2 protein
Locus_13976_Transcript_1/1_Conf_1.000	226	0							
Locus_13977_Transcript_1/1_Conf_1.000	334	2	5.40E-08	NP_495385.1	78	60.8474	42	33	hypothetical protein F41G3.1

Locus_13978_Transcript_1/1_Conf_1.000	155	20	6.48E-25	NP_001158888.1	100	117.087	51	51	L-lactate dehydrogenase A chain isoform 5
Locus_13979_Transcript_1/1_Conf_1.000	214	20	1.50E-34	XP_002927231.1	100	149.058	71	71	PREDICTED: tubulin alpha-1B chain-like
Locus_1398_Transcript_1/2_Conf_1.000	1828	20	7.68E-132	NP_502079.1	70	475.707	431	303	IntraMembrane Protease (IMPAS) family member (imp-2)
Locus_1398_Transcript_2/2_Conf_1.000	1809	20	5.85E-128	NP_502079.1	74	389.037	313	234	IntraMembrane Protease (IMPAS) family member (imp-2)
Locus_13980_Transcript_1/1_Conf_1.000	139	0							
Locus_13981_Transcript_1/1_Conf_1.000	308	4	1.20E-15	XP_002639235.1	69	86.2705	65	45	Hypothetical protein CBG03793

Locus_13982_Transcript_1/1_Conf_1.000	388	20	2.91E-54	EFO19497.1	89	214.542	128	115	DNA polymerase epsilon catalytic subunit
Locus_13983_Transcript_1/1_Conf_1.000	248	9	1.01E-14	NP_506678.2	65	83.1889	78	51	hypothetical protein W09D12.1
Locus_13984_Transcript_1/1_Conf_1.000	337	0							
Locus_13985_Transcript_1/1_Conf_1.000	361	0							
Locus_13986_Transcript_1/1_Conf_1.000	735	20	9.50E-39	XP_002641664.1	64	164.466	216	139	Hypothetical protein CBG09992
Locus_13987_Transcript_1/1_Conf_1.000	444	20	1.38E-51	XP_002640280.1	83	205.682	121	101	Hypothetical protein CBG12806
Locus_13988_Transcript_1/1_Conf_1.000	839	20	1.17E-49	NP_494767.1	58	201.06	286	166	hypothetical protein H20J04.2

Locus_13989_Transcript_1/1_Conf_1.000	1524	20	5.44E-72		54	276.559	508	278	hypothetical protein Y106G6D.5
Locus_1399_Transcript_1/3_Conf_0.500	1802	4	1.41E-37	NP_001122735.1	57	162.54	407	236	hypothetical protein Y56A3A.7
Locus_1399_Transcript_2/3_Conf_0.625	1579	5	2.03E-45	NP_001122735.1	58	188.348	448	261	hypothetical protein Y56A3A.7
Locus_1399_Transcript_3/3_Conf_0.500	1870	5	2.52E-45	NP_001122735.1	58	188.348	448	261	hypothetical protein Y56A3A.7
Locus_13990_Transcript_1/1_Conf_1.000	220	0							
Locus_13991_Transcript_1/1_Conf_1.000	335	0							
Locus_13992_Transcript_1/1_Conf_1.000	729	20	1.47E-84	XP_002647042.1	76	316.62	247	190	Hypothetical protein CBG03560
Locus_13993_Transcript_1/1_Conf_1.000	162	0							
Locus_13994_Transcript_1/1_Conf_1.000	357	20	2.75E-44	EFO19977.1	91	181.415	117	107	hypothetical protein LOAG_08511
Locus_13995_Transcript_1/1_Conf_1.000	151	20	8.78E-22	XP_002638784.1	96	106.686	50	48	Hypothetical protein CBG05137
Locus_13996_Transcript_1/1_Conf_1.000	576	20	2.79E-19	NP_508889.1	52	98.9821	155	82	hypothetical protein Y34B4A.7
Locus_13997_Transcript_1/1_Conf_1.000	978	20	1.86E-39	EFO19488.1	60	167.548	233	142	hypothetical protein LOAG_09005
Locus_13998_Transcript_1/1_Conf_1.000	211	0							
Locus_13999_Transcript_1/1_Conf_1.000	402	4	4.78E-04	XP_001899942.1	54	47.7506	97	53	hypothetical protein Bm1_42390

Locus_14_Transcript_1/1_Conf_1.000	2309	20	0	XP_002641511.1	94	973.77	599	569	C. briggsae CBR-CHC-1 protein
Locus_140_Transcript_1/1_Conf_1.000	1282	20	0	AAS55927.1	96	765.763	399	386	cytoskeletal beta actin
Locus_1400_Transcript_1/1_Conf_1.000	893	0							
Locus_14000_Transcript_1/1_Conf_1.000	349	20	2.52E-37	XP_002636973.1	86	158.303	115	99	Hypothetical protein CBG09454
Locus_14001_Transcript_1/1_Conf_1.000	259	20	1.31E-17	XP_002641885.1	73	92.8189	86	63	Hypothetical protein CBG16572

Locus_14002_Transcript_1/1_Conf_1.000	960	20	2.10E-138	XP_001155090.1	98	381.333	197	195	PREDICTED: similar to ANXA2 protein
Locus_14003_Transcript_1/1_Conf_1.000	335	0							
Locus_14004_Transcript_1/1_Conf_1.000	191	0							
Locus_14005_Transcript_1/1_Conf_1.000	151	1	6.32E-04	XP_002642982.1	58	47.3654	50	29	Hypothetical protein CBG15266
Locus_14006_Transcript_1/1_Conf_1.000	148	0							
Locus_14007_Transcript_1/1_Conf_1.000	266	1	8.17E-04	XP_001901545.1	64	46.9802	54	35	hypothetical protein Bm1_50385
Locus_14008_Transcript_1/1_Conf_1.000	574	20	1.17E-70	XP_001895692.1	74	269.626	204	152	jmjC domain containing protein
Locus_14009_Transcript_1/1_Conf_1.000	139	20	9.14E-16	EFO23246.1	95	86.6557	45	43	degenerin unc-8
Locus_1401_Transcript_1/1_Conf_1.000	1167	20	1.73E-85	XP_002641620.1	60	320.857	392	239	C. briggsae CBR-BUB-1 protein
Locus_14010_Transcript_1/1_Conf_1.000	388	0							
Locus_14011_Transcript_1/1_Conf_1.000	448	0							
Locus_14012_Transcript_1/1_Conf_1.000	423	0							
Locus_14013_Transcript_1/1_Conf_1.000	161	0							

Locus_14014_Transcript_1/1_Conf_1.000	619	20	3.19E-86	XP_001896258.1	81	321.627	205	167	Cytoplasmic polyadenylation element binding protein 1
Locus_14015_Transcript_1/1_Conf_1.000	669	0							
Locus_14016_Transcript_1/3_Conf_0.600	335	1	2.51E-05	NP_491118.2	56	51.9878	110	62	hypothetical protein Y54E10A.11
Locus_14016_Transcript_2/3_Conf_0.600	342	1	4.38E-05	NP_491118.2	55	51.2174	110	61	hypothetical protein Y54E10A.11
Locus_14016_Transcript_3/3_Conf_0.600	335	1	4.28E-05	NP_491118.2	55	51.2174	110	61	hypothetical protein Y54E10A.11
Locus_14017_Transcript_1/1_Conf_1.000	440	10	3.79E-46	NP_001129848.1	79	187.578	144	114	hypothetical protein C27H2.2
Locus_14018_Transcript_1/1_Conf_1.000	476	20	3.16E-24	XP_317488.4	76	114.775	89	68	AGAP007978-PA
Locus_14019_Transcript_1/1_Conf_1.000	161	0							
Locus_1402_Transcript_1/1_Conf_1.000	560	20	1.72E-60	XP_002639413.1	85	235.728	151	129	C. briggsae CBR-CPS-6 protein
Locus_14020_Transcript_1/1_Conf_1.000	270	6	5.60E-13	AAK31527.3	57	77.411	113	65	Neuronal symmetry protein 1, partially confirmed by transcript evidence
Locus_14021_Transcript_1/1_Conf_1.000	149	20	1.66E-12	XP_001899592.1	85	75.8702	48	41	C2 domain containing protein
Locus_14022_Transcript_1/1_Conf_1.000	128	0							
Locus_14023_Transcript_1/1_Conf_1.000	458	20	2.94E-22	NP_498986.2	68	108.227	128	88	hypothetical protein R08D7.5
Locus_14024_Transcript_1/1_Conf_1.000	333	18	1.24E-28	NP_506118.1	81	129.413	90	73	hypothetical protein C54D10.5
Locus_14025_Transcript_1/1_Conf_1.000	395	20	1.27E-20	XP_002638798.1	74	102.834	111	83	C. briggsae CBR-PIF-1 protein
Locus_14026_Transcript_1/1_Conf_1.000	173	0							
Locus_14027_Transcript_1/1_Conf_1.000	194	20	2.30E-14	XP_002646723.1	75	82.0333	58	44	C. briggsae CBR-CLH-3 protein
Locus_14028_Transcript_1/1_Conf_1.000	146	0							
Locus_14029_Transcript_1/1_Conf_1.000	496	0							

Locus_1403_Transcript_1/1_Conf_1.000	302	2	4.30E-05	XP_002642297.1	58	51.2174	51	30	Hypothetical protein CBG18289
Locus_14030_Transcript_1/1_Conf_1.000	148	0							
Locus_14031_Transcript_1/1_Conf_1.000	283	0							
Locus_14032_Transcript_1/1_Conf_1.000	392	20	4.50E-39	EFO23299.1	73	164.081	130	96	hypothetical protein LOAG_05186
Locus_14033_Transcript_1/2_Conf_1.000	1212	20	1.14E-95	XP_002636970.1	60	354.755	446	271	Hypothetical protein CBG09451
Locus_14033_Transcript_2/2_Conf_1.000	1212	20	1.14E-95	XP_002636970.1	60	354.755	446	271	Hypothetical protein CBG09451
Locus_14034_Transcript_1/1_Conf_1.000	1029	20	8.42E-86	XP_002634139.1	73	321.627	300	221	Hypothetical protein CBG01700
Locus_14035_Transcript_1/1_Conf_1.000	270	20	7.54E-26	EFO21211.1	78	120.168	89	70	hypothetical protein LOAG_07280
Locus_14036_Transcript_1/1_Conf_1.000	496	0							
Locus_14037_Transcript_1/1_Conf_1.000	147	20	2.17E-20	3IE3	100	102.064	48	48	l Basis ForThe Binding OfThe Anti-Cancer Compound 6-(7-Nitro-2
Locus_14038_Transcript_1/1_Conf_1.000	595	20	1.85E-69	XP_002630445.1	81	265.774	180	146	Hypothetical protein CBG11178
Locus_14039_Transcript_1/1_Conf_1.000	950	7	6.37E-21	XP_001894575.1	57	105.916	158	91	MGC80099 protein
Locus_1404_Transcript_1/1_Conf_1.000	302	20	2.33E-43	AAO85518.1	100	178.333	87	87	AF496634_1putative serine/threonine phosphatase
Locus_14040_Transcript_1/1_Conf_1.000	399	20	5.44E-32	ABQ01576.1	72	140.584	109	79	scramblase 1
Locus_14041_Transcript_1/1_Conf_1.000	320	0							

Locus_14042_Transcript_1/1_Conf_1.000	184	20	2.17E-28	EFB20679.1	100	128.642	60	60	hypothetical protein PANDA_013768
Locus_14043_Transcript_1/1_Conf_1.000	131	0							
Locus_14044_Transcript_1/1_Conf_1.000	430	20	2.31E-67	AAO85518.1	98	258.07	127	125	AF496634_1putative serine/threonine phosphatase
Locus_14045_Transcript_1/1_Conf_1.000	194	6	2.30E-14	NP_510479.1	93	82.0333	63	59	Multidrug Resistance Protein family member (mrp-5)
Locus_14046_Transcript_1/1_Conf_1.000	172	20	2.19E-09	XP_001897369.1	75	65.4698	52	39	Protein kinase domain containing protein
Locus_14047_Transcript_1/1_Conf_1.000	165	3	2.88E-09	XP_002646710.1	74	65.0846	43	32	C. briggsae CBR-MAOC-1 protein
Locus_14048_Transcript_1/1_Conf_1.000	1358	20	6.92E-76	NP_001041223.1	67	289.271	300	201	mammalian VAV (oncogene) related family member (vav-1)
Locus_14049_Transcript_1/1_Conf_1.000	531	4	1.18E-12	NP_500378.1	65	76.6406	78	51	TAF (TBP-associated transcription factor) family member (taf-2)
Locus_1405_Transcript_1/1_Conf_1.000	1277	20	6.07E-103	EFO28398.1	72	379.022	425	307	hypothetical protein LOAG_00087
Locus_14050_Transcript_1/1_Conf_1.000	190	0							
Locus_14051_Transcript_1/1_Conf_1.000	934	3	1.81E-12	XP_001896205.1	57	77.7962	90	52	hypothetical protein Bm1_23740
Locus_14052_Transcript_1/1_Conf_1.000	146	0							

Locus_14053_Transcript_1/1_Conf_1.000	192	20	5.66E-21	NP_493596.1	100	103.99	53	53	UNCoordinated family member (unc-54)
Locus_14054_Transcript_1/1_Conf_1.000	267	0							
Locus_14055_Transcript_1/1_Conf_1.000	204	0							
Locus_14056_Transcript_1/1_Conf_1.000	811	20	9.91E-51	NP_001024382.1	83	204.527	157	131	DiacylGlycerol Kinase family member (dgk-1)
Locus_14057_Transcript_1/1_Conf_1.000	177	1	3.60E-04	NP_492172.1	71	48.1358	49	35	Aspergillus NUclear Division related family member (nud-2)
Locus_14058_Transcript_1/1_Conf_1.000	408	0							
Locus_14059_Transcript_1/1_Conf_1.000	309	19	5.93E-07	CAB40845.1	52	57.3806	75	39	conidiospore surface protein
Locus_1406_Transcript_1/1_Conf_1.000	1498	20	1.06E-96	XP_001899812.1	67	358.607	410	276	hypothetical protein Bm1_41765
Locus_14060_Transcript_1/1_Conf_1.000	534	20	1.25E-38	XP_001897852.1	56	162.925	177	100	notch-like transmembrane receptor

Locus_14061_Transcript_1/1_Conf_1.000	410	0							
Locus_14062_Transcript_1/1_Conf_1.000	139	1	2.58E-10	AAT12422.1	83	68.5514	42	35	hypothetical protein isoform a
Locus_14063_Transcript_1/1_Conf_1.000	479	5	9.18E-08	XP_002638736.1	56	60.077	90	51	C. briggsae CBR-EGO-1 protein
Locus_14064_Transcript_1/1_Conf_1.000	260	20	8.17E-28	NP_498761.1	86	126.716	86	74	DiCer Related family member (dcr-1)
Locus_14065_Transcript_1/1_Conf_1.000	161	8	1.95E-05	NP_001020988.1	84	52.373	53	45	UNCoordinated family member (unc-89)
Locus_14066_Transcript_1/1_Conf_1.000	355	0							
Locus_14067_Transcript_1/1_Conf_1.000	634	20	4.15E-28	XP_002641256.1	85	128.642	82	70	Hypothetical protein CBG05167
Locus_14068_Transcript_1/1_Conf_1.000	177	0							
Locus_14069_Transcript_1/1_Conf_1.000	914	20	1.64E-87	EFO27866.1	76	327.02	283	216	GTPase activating protein and VPS9 domains 1
Locus_1407_Transcript_1/2_Conf_1.000	706	20	1.49E-107	CAR63530.1	98	392.889	197	195	putative synaptic vesicle 2-related protein
Locus_1407_Transcript_2/2_Conf_1.000	861	20	2.97E-128	CAR63530.1	97	462.225	243	236	putative synaptic vesicle 2-related protein
Locus_14070_Transcript_1/1_Conf_1.000	387	20	2.84E-33	XP_002642740.1	79	144.821	112	89	Hypothetical protein CBG21119
Locus_14071_Transcript_1/1_Conf_1.000	372	0							
Locus_14072_Transcript_1/1_Conf_1.000	293	6	5.12E-14	XP_002639127.1	71	80.8777	83	59	Hypothetical protein CBG14946

Locus_14073_Transcript_1/1_Conf_1.000	478	20	1.76E-67	XP_002912365.1	100	258.455	129	129	PREDICTED: 60S ribosomal protein L23a-like
Locus_14074_Transcript_1/1_Conf_1.000	345	9	1.39E-35	XP_002641524.1	87	152.525	87	76	Hypothetical protein CBG09820
Locus_14075_Transcript_1/1_Conf_1.000	364	0							
Locus_14076_Transcript_1/1_Conf_1.000	483	2	1.40E-19	XP_002639186.1	57	99.3673	173	99	C. briggsae CBR-LEM-3 protein
Locus_14077_Transcript_1/1_Conf_1.000	351	0							
Locus_14078_Transcript_1/1_Conf_1.000	285	0							
Locus_14079_Transcript_1/1_Conf_1.000	415	20	3.57E-28	NP_001022052.1	64	127.872	134	87	SREBP Cleavage activating Protein (SCAP) homolog family member (scp-1)
Locus_1408_Transcript_1/2_Conf_1.000	317	20	1.23E-15	P82968.1	79	86.2705	53	42	Four-domain proteases inhibitor
Locus_1408_Transcript_2/2_Conf_1.000	316	20	2.09E-15	P82968.1	79	85.5001	53	42	Four-domain proteases inhibitor
Locus_14080_Transcript_1/1_Conf_1.000	471	20	1.98E-42	XP_002648986.1	76	175.252	160	122	C. briggsae CBR-SAX-2 protein
Locus_14081_Transcript_1/1_Conf_1.000	383	20	4.01E-19	XP_395372.2	59	97.8265	127	76	PREDICTED: similar to CG7565-PA
Locus_14082_Transcript_1/1_Conf_1.000	622	20	2.62E-56	XP_001894163.1	76	222.246	193	148	hypothetical protein Bm1_13490
Locus_14083_Transcript_1/2_Conf_1.000	649	20	1.19E-49	NP_502541.2	78	200.29	149	117	hypothetical protein JC8.2
Locus_14083_Transcript_2/2_Conf_1.000	626	20	1.08E-49	NP_502541.2	78	200.29	149	117	hypothetical protein JC8.2
Locus_14084_Transcript_1/1_Conf_1.000	812	20	2.86E-82	XP_002631390.1	84	309.301	258	217	Hypothetical protein CBG03232

Locus_14085_Transcript_1/1_Conf_1.000	162	0							
Locus_14086_Transcript_1/1_Conf_1.000	630	0							
Locus_14087_Transcript_1/2_Conf_1.000	428	0							
Locus_14087_Transcript_2/2_Conf_1.000	428	0							
Locus_14088_Transcript_1/1_Conf_1.000	672	20	4.02E-35	CAB16467.2	63	152.14	180	115	C. elegans protein W10C6.1, partially confirmed by transcript evidence
Locus_14089_Transcript_1/1_Conf_1.000	254	20	5.89E-26	EFO25124.1	80	120.553	83	67	bromodomain containing protein
Locus_1409_Transcript_1/1_Conf_1.000	616	20	3.45E-109	BAG61974.1	100	397.897	192	192	unnamed protein product
Locus_14090_Transcript_1/1_Conf_1.000	224	13	8.50E-17	XP_002641658.1	74	90.1225	71	53	Hypothetical protein CBG09986
Locus_14091_Transcript_1/1_Conf_1.000	225	0							
Locus_14092_Transcript_1/1_Conf_1.000	246	0							
Locus_14093_Transcript_1/1_Conf_1.000	439	20	1.15E-18	XP_002646219.1	65	96.2857	101	66	Hypothetical protein CBG11908
Locus_14094_Transcript_1/1_Conf_1.000	144	20	6.35E-12	CAF98408.1	83	73.9442	48	40	unnamed protein product
Locus_14095_Transcript_1/1_Conf_1.000	157	0							
Locus_14096_Transcript_1/2_Conf_1.000	621	20	5.51E-22	XP_002632140.1	74	108.227	105	78	C. briggsae CBR-SPTL-1 protein
Locus_14096_Transcript_2/2_Conf_1.000	260	0							
Locus_14097_Transcript_1/1_Conf_1.000	356	9	4.05E-49	XP_519474.2	96	126.716	62	60	PREDICTED: hepatocellular carcinoma-associated antigen 112
Locus_14098_Transcript_1/1_Conf_1.000	165	0							

Locus_14099_Transcript_1/1_Conf_1.000	275	20	6.39E-09	XP_001893875.1	61	63.929	81	50	Bromodomain associated family protein
Locus_141_Transcript_1/1_Conf_1.000	2163	20	4.62E-163	NP_872090.1	74	579.711	527	391	Lateral Signaling Target family member (lst-4)
Locus_1410_Transcript_1/1_Conf_1.000	200	0							
Locus_14100_Transcript_1/1_Conf_1.000	488	20	1.44E-08	NP_001021459.2	44	62.7734	159	71	hypothetical protein F36H2.3
Locus_14101_Transcript_1/1_Conf_1.000	212	0							
Locus_14102_Transcript_1/1_Conf_1.000	270	0							
Locus_14103_Transcript_1/1_Conf_1.000	439	20	2.29E-59	NP_004543.1	100	231.491	106	106	NADH dehydrogenase ubiquinone iron-sulfur protein 5
Locus_14104_Transcript_1/1_Conf_1.000	1783	20	2.65E-12	XP_002661927.1	41	78.5666	439	182	PREDICTED: hypothetical protein
Locus_14105_Transcript_1/1_Conf_1.000	369	0							
Locus_14106_Transcript_1/1_Conf_1.000	133	0							
Locus_14107_Transcript_1/1_Conf_1.000	132	0							
Locus_14108_Transcript_1/1_Conf_1.000	621	15	1.29E-87	NP_495267.1	89	326.25	191	171	hypothetical protein C29F5.1
Locus_14109_Transcript_1/1_Conf_1.000	222	0							
Locus_1411_Transcript_1/1_Conf_1.000	987	20	2.23E-48	XP_002634004.1	80	197.208	180	144	Hypothetical protein CBG20111
Locus_14110_Transcript_1/1_Conf_1.000	449	0							

Locus_14111_Transcript_1/1_Conf_1.000	339	20	2.17E-17	EFO24980.1	72	92.0485	79	57	hypothetical protein LOAG_03505
Locus_14112_Transcript_1/1_Conf_1.000	978	0							
Locus_14113_Transcript_1/1_Conf_1.000	667	20	5.18E-25	NP_502063.1	45	94.3597	163	74	hypothetical protein Y5F2A.4
Locus_14114_Transcript_1/1_Conf_1.000	189	20	7.69E-10	XP_002640423.1	73	67.0106	56	41	Hypothetical protein CBG08474
Locus_14115_Transcript_1/1_Conf_1.000	363	20	1.04E-35	XP_002631218.1	81	152.91	121	99	Hypothetical protein CBG03015
Locus_14116_Transcript_1/1_Conf_1.000	777	5	3.29E-32	NP_496398.2	76	142.895	102	78	hypothetical protein T06D8.1
Locus_14117_Transcript_1/1_Conf_1.000	336	0							
Locus_14118_Transcript_1/1_Conf_1.000	328	20	3.74E-33	ACD88896.1	81	144.436	91	74	conserved hypothetical protein
Locus_14119_Transcript_1/1_Conf_1.000	332	0							
Locus_1412_Transcript_1/1_Conf_1.000	1338	20	1.66E-90	XP_001894513.1	68	337.806	363	247	DZF family protein

Locus_14120_Transcript_1/1_Conf_1.000	442	20	1.25E-44	XP_002633800.1	79	182.57	147	117	C. briggsae CBR-RBD-1 protein
Locus_14121_Transcript_1/1_Conf_1.000	473	0							
Locus_14122_Transcript_1/1_Conf_1.000	652	20	1.56E-81	NP_001122814.1	82	306.22	217	178	STAT transcription factor family member (sta-1)
Locus_14123_Transcript_1/1_Conf_1.000	282	20	1.32E-09	CAJ98743.1	77	66.2402	54	42	serine/threonine phosphatase
Locus_14124_Transcript_1/1_Conf_1.000	267	0							
Locus_14125_Transcript_1/1_Conf_1.000	543	20	3.59E-60	NP_505435.1	84	234.572	175	148	hypothetical protein F36D4.2
Locus_14126_Transcript_1/1_Conf_1.000	289	0							
Locus_14127_Transcript_1/1_Conf_1.000	464	20	5.25E-59	NP_494756.1	84	230.335	154	130	hypothetical protein C01F1.1
Locus_14128_Transcript_1/1_Conf_1.000	310	20	2.03E-23	NP_493573.1	73	112.079	100	73	hypothetical protein F39B2.7
Locus_14129_Transcript_1/1_Conf_1.000	223	0							
Locus_1413_Transcript_1/1_Conf_1.000	1479	6	1.00E-14	NP_001129872.1	46	86.2705	366	169	hypothetical protein T23B5.3
Locus_14130_Transcript_1/1_Conf_1.000	566	20	3.59E-69	CBA12962.1	75	264.618	200	151	LEV-9 protein
Locus_14131_Transcript_1/1_Conf_1.000	313	0							
Locus_14132_Transcript_1/1_Conf_1.000	453	20	3.59E-36	NP_509859.1	73	154.451	118	87	hypothetical protein R08B4.3
Locus_14133_Transcript_1/1_Conf_1.000	132	0							

Locus_14134_Transcript_1/1_Conf_1.000	968	20	1.30E-61	XP_001896873.1	69	241.121	267	185	Origin recognition complex subunit 1
Locus_14135_Transcript_1/1_Conf_1.000	585	0							
Locus_14136_Transcript_1/1_Conf_1.000	1051	20	5.42E-80	NP_001023527.1	67	302.368	298	202	hypothetical protein Y57G11C.43
Locus_14137_Transcript_1/1_Conf_1.000	442	20	4.75E-52	AAX37111.1	100	207.223	118	118	small nuclear ribonucleoprotein D2 polypeptide
Locus_14138_Transcript_1/1_Conf_1.000	375	7	2.62E-10	ADI24623.1	64	68.5514	70	45	Copine domain protein, atypical protein 2, isoform c, partially confirmed by transcript evidence
Locus_14139_Transcript_1/1_Conf_1.000	963	20	2.30E-34	EFO26310.1	75	150.599	129	97	rap/ran-GAP family protein
Locus_1414_Transcript_1/1_Conf_1.000	790	20	5.30E-110	NP_001129887.1	86	401.364	262	227	SEC(selenocysteine)-tRNA Synthase family member (secs-1)
Locus_14140_Transcript_1/1_Conf_1.000	139	20	1.67E-17	BAG60581.1	100	92.4337	46	46	unnamed protein product
Locus_14141_Transcript_1/1_Conf_1.000	264								
Locus_14142_Transcript_1/1_Conf_1.000	448	20	9.61E-13	XP_002674131.1	55	76.6406	86	48	hypothetical protein NAEGRDRAFT_80725
Locus_14143_Transcript_1/1_Conf_1.000	168	0							
Locus_14144_Transcript_1/1_Conf_1.000	391	3	6.54E-14	NP_500183.2	75	80.4925	60	45	hypothetical protein M70.4
Locus_14145_Transcript_1/1_Conf_1.000	260	0							
Locus_14146_Transcript_1/1_Conf_1.000	266	20	1.13E-21	XP_002645292.1	73	106.301	88	65	C. briggsae CBR-SNF-12 protein
Locus_14147_Transcript_1/1_Conf_1.000	208	0							
Locus_14148_Transcript_1/1_Conf_1.000	1051	20	1.11E-08	XP_002635706.1	39	65.4698	215	85	Hypothetical protein CBG22445

Locus_14149_Transcript_1/1_Conf_1.000	366	4	2.09E-36	NP_001033357.2	79	155.221	118	94	DAF-16/FOXO Controlled, germline Tumor affecting family member (dct-6)
Locus_1415_Transcript_1/1_Conf_1.000	1034	20	9.68E-82	NP_492732.2	72	308.145	321	233	yeast SEC homolog family member (sec-8)
Locus_14150_Transcript_1/1_Conf_1.000	190	0							
Locus_14151_Transcript_1/1_Conf_1.000	146	13	1.98E-05	XP_001901649.1	74	52.373	47	35	WGR domain containing protein
Locus_14152_Transcript_1/1_Conf_1.000	138	0							
Locus_14153_Transcript_1/1_Conf_1.000	139	4	1.61E-12	CAA86821.1	95	75.8702	43	41	excretory/secretory antigen
Locus_14154_Transcript_1/1_Conf_1.000	342	0							
Locus_14155_Transcript_1/1_Conf_1.000	154	0							
Locus_14156_Transcript_1/1_Conf_1.000	525	20	9.48E-60	NP_492590.1	79	233.032	162	129	hypothetical protein K02A11.3
Locus_14157_Transcript_1/1_Conf_1.000	673	20	3.64E-36	EFO26611.1	60	155.606	220	134	hypothetical protein LOAG_01867
Locus_14158_Transcript_1/1_Conf_1.000	219	0							
Locus_14159_Transcript_1/1_Conf_1.000	234	20	2.88E-17	NP_501645.1	75	91.6633	69	52	hypothetical protein T20D3.8
Locus_1416_Transcript_1/1_Conf_1.000	508	2	1.40E-62	XP_001145371.1	98	242.276	118	116	PREDICTED: hypothetical protein
Locus_14160_Transcript_1/1_Conf_1.000	577	20	2.25E-69	NP_496170.1	80	265.388	191	154	LEThal family member (let-268)
Locus_14161_Transcript_1/1_Conf_1.000	800	20	2.77E-13	XP_001902864.1	65	80.1073	87	57	hypothetical protein Bm1_57035
Locus_14162_Transcript_1/1_Conf_1.000	842	20	4.07E-26	XP_002641927.1	63	122.865	138	88	Hypothetical protein CBG16626
Locus_14163_Transcript_1/1_Conf_1.000	128	20	1.48E-13	NP_503406.2	92	79.337	42	39	SyNapTotagmin family member (snt-3)
Locus_14164_Transcript_1/1_Conf_1.000	169	0							
Locus_14165_Transcript_1/1_Conf_1.000	254	0							
Locus_14166_Transcript_1/1_Conf_1.000	194	0							
Locus_14167_Transcript_1/1_Conf_1.000	226	4	5.13E-22	XP_002631267.1	80	107.457	75	60	C. briggsae CBR-PRO-1 protein
Locus_14168_Transcript_1/1_Conf_1.000	593	0							
Locus_14169_Transcript_1/1_Conf_1.000	198	0							

Locus_1417_Transcript_1/1_Conf_1.000	1934	20	1.35E-17	CAX12544.1	44	96.2857	258	114	nuclear oncoprotein skia
Locus_14170_Transcript_1/2_Conf_1.000	291	0							
Locus_14170_Transcript_2/2_Conf_1.000	333	0							
Locus_14171_Transcript_1/1_Conf_1.000	156	1	1.76E-22	XP_002343179.1	100	108.997	51	51	PREDICTED: hypothetical protein XP_002343179
Locus_14172_Transcript_1/1_Conf_1.000	570	3	9.97E-06	XP_002637821.1	51	53.9138	152	78	Hypothetical protein CBG04610
Locus_14173_Transcript_1/1_Conf_1.000	469	0							
Locus_14174_Transcript_1/1_Conf_1.000	322	11	1.70E-25	XP_002634211.1	83	119.013	90	75	C. briggsae CBR-MAU-8 protein
Locus_14175_Transcript_1/1_Conf_1.000	452	20	3.39E-26	NP_491902.3	69	121.324	120	83	hypothetical protein D2092.5
Locus_14176_Transcript_1/1_Conf_1.000	404	20	7.07E-32	NP_741383.1	74	140.198	111	83	tYrosinyl tRNA Synthetase family member (yrs-1)
Locus_14177_Transcript_1/1_Conf_1.000	164	0							
Locus_14178_Transcript_1/1_Conf_1.000	405	20	2.42E-32	NP_495663.1	72	141.739	134	97	REV1 (translesion DNA polymerase) homolog family member (rev-1)
Locus_14179_Transcript_1/1_Conf_1.000	913	20	8.32E-15	EFO17283.1	60	85.5001	202	122	hypothetical protein LOAG_11216
Locus_1418_Transcript_1/1_Conf_1.000	881	2	7.01E-24	NP_502443.2	51	115.546	257	133	hypothetical protein F07C6.4
Locus_14180_Transcript_1/1_Conf_1.000	370	20	1.89E-24	EFO19846.1	79	115.546	92	73	cleavage and polyadenylation specificity factor subunit 2
Locus_14181_Transcript_1/1_Conf_1.000	382	5	1.52E-18	XP_002643698.1	74	95.9005	78	58	Hypothetical protein CBG01887
Locus_14182_Transcript_1/1_Conf_1.000	694	20	1.65E-26	XP_001900728.1	75	123.635	96	72	RhoGAP domain containing protein
Locus_14183_Transcript_1/1_Conf_1.000	337	20	3.01E-51	AAD00776.1	89	204.527	112	100	2,5 dihydroxyphenylacetate oxidase
Locus_14184_Transcript_1/1_Conf_1.000	266	20	1.17E-42	XP_002347521.1	100	176.022	84	84	PREDICTED: hypothetical protein XP_002347521

Locus_14185_Transcript_1/1_Conf_1.000	194	2	9.06E-11	XP_002638788.1	84	70.0922	46	39	Hypothetical protein CBG05143
Locus_14186_Transcript_1/1_Conf_1.000	233	0							
Locus_14187_Transcript_1/1_Conf_1.000	408	0							
Locus_14188_Transcript_1/1_Conf_1.000	480	20	4.36E-26	XP_002634144.1	77	120.939	131	101	Hypothetical protein CBG01705
Locus_14189_Transcript_1/1_Conf_1.000	224	0							
Locus_1419_Transcript_1/1_Conf_1.000	588	20	5.41E-66	NP_499418.1	86	254.218	153	133	hypothetical protein Y48A6B.7
Locus_14190_Transcript_1/1_Conf_1.000	129	0							
Locus_14191_Transcript_1/1_Conf_1.000	335	0							
Locus_14192_Transcript_1/1_Conf_1.000	631	20	2.08E-21	XP_002633194.1	58	84.7297	105	61	Hypothetical protein CBG05911
Locus_14193_Transcript_1/2_Conf_1.000	631	2	5.39E-04	EFO27009.1	71	48.521	35	25	hypothetical protein LOAG_01466
Locus_14193_Transcript_2/2_Conf_1.000	571	2	4.19E-04	EFO27009.1	71	48.521	35	25	hypothetical protein LOAG_01466
Locus_14194_Transcript_1/1_Conf_1.000	358	4	1.56E-39	NP_496830.2	83	165.622	119	99	hypothetical protein Y48E1C.4
Locus_14195_Transcript_1/1_Conf_1.000	313	0							
Locus_14196_Transcript_1/1_Conf_1.000	194	0							
Locus_14197_Transcript_1/1_Conf_1.000	468	20	5.92E-71	AAC38987.1	91	270.011	156	142	P-glycoprotein
Locus_14198_Transcript_1/1_Conf_1.000	545	20	1.46E-45	XP_002640390.1	72	186.037	182	132	Hypothetical protein CBG08435
Locus_14199_Transcript_1/1_Conf_1.000	221	6	1.97E-05	XP_002639496.1	69	52.373	66	46	Hypothetical protein CBG04097

Locus_142_Transcript_1/1_Conf_1.000	1491	20	1.49E-66	NP_496763.1	59	258.455	337	200	CYclin D family member (cyd-1)
Locus_1420_Transcript_1/1_Conf_1.000	487	20	8.63E-46	NP_510298.1	68	186.422	160	110	AMP-Activated Kinase Beta subunit family member (aakb-1)
Locus_14200_Transcript_1/1_Conf_1.000	256	0							
Locus_14201_Transcript_1/1_Conf_1.000	154	0							
Locus_14202_Transcript_1/1_Conf_1.000	1023	1	1.96E-10	NP_498298.1	41	71.2478	321	134	hypothetical protein F25B5.2
Locus_14203_Transcript_1/1_Conf_1.000	202	0							
Locus_14204_Transcript_1/1_Conf_1.000	935	20	6.54E-63	NP_491315.1	64	245.358	304	197	INneXin family member (inx-17)
Locus_14205_Transcript_1/1_Conf_1.000	282	5	8.86E-06	XP_002631238.1	62	53.5286	64	40	Hypothetical protein CBG03041
Locus_14206_Transcript_1/1_Conf_1.000	685	20	4.59E-74	1TW9	84	281.567	204	172	Glutathione Transferase-2
Locus_14207_Transcript_1/1_Conf_1.000	424	0							
Locus_14208_Transcript_1/1_Conf_1.000	185	20	1.83E-27	XP_002914026.1	100	125.561	61	61	PREDICTED: polyadenylate-binding protein 1-like
Locus_14209_Transcript_1/1_Conf_1.000	362	0							
Locus_1421_Transcript_1/1_Conf_1.000	603	0							
Locus_14210_Transcript_1/1_Conf_1.000	264	20	1.21E-31	XP_001894470.1	86	139.428	87	75	GCD14 protein
Locus_14211_Transcript_1/1_Conf_1.000	468	9	9.54E-37	NP_499059.2	60	156.377	168	102	hypothetical protein K04H4.2

Locus_14212_Transcript_1/1_Conf_1.000	341	0							
Locus_14213_Transcript_1/1_Conf_1.000	901	20	9.16E-59	XP_002638852.1	63	231.491	290	184	Hypothetical protein CBG22064
Locus_14214_Transcript_1/1_Conf_1.000	204	0							
Locus_14215_Transcript_1/1_Conf_1.000	388	20	5.52E-37	NP_001040927.1	75	157.147	128	96	hypothetical protein C47A4.2
Locus_14216_Transcript_1/1_Conf_1.000	172	0							
Locus_14217_Transcript_1/1_Conf_1.000	274	20	1.42E-16	XP_002641416.1	78	89.3521	65	51	C. briggsae CBR-SBP-1 protein
Locus_14218_Transcript_1/1_Conf_1.000	222	0							
Locus_14219_Transcript_1/1_Conf_1.000	227	20	6.06E-23	XP_002606421.1	89	110.538	73	65	hypothetical protein BRAFLDRAFT_67673
Locus_1422_Transcript_1/1_Conf_1.000	865	20	3.31E-119	NP_871640.1	84	432.18	288	242	Glycyl tRNA Synthetase family member (grs-1)
Locus_14220_Transcript_1/1_Conf_1.000	364	20	6.06E-52	NP_001023252.1	91	206.838	120	110	Transbilayer Amphipath Transporters (subfamily IV P-type ATPase) family member (tat-2)
Locus_14221_Transcript_1/1_Conf_1.000	204	2	1.09E-08	NP_499474.1	85	63.1586	35	30	BED-type zinc finger transcription factor family member (bed-2)
Locus_14222_Transcript_1/1_Conf_1.000	396	20	4.73E-68	XP_002927413.1	100	260.381	131	131	PREDICTED: eukaryotic translation initiation factor 3 subunit C-like
Locus_14223_Transcript_1/1_Conf_1.000	390	6	4.53E-15	EFO16157.1	52	84.3445	142	74	ribonuclease P/MRP protein subunit POP1 containing protein

Locus_14224_Transcript_1/1_Conf_1.000	329	20	1.13E-29	XP_001900386.1	80	132.88	105	84	Phosphatidylcholine:ceramide cholinephosphotransferase 3, putative
Locus_14225_Transcript_1/1_Conf_1.000	266	17	1.86E-40	EFO27230.1	97	168.703	88	86	fasciclin domain-containing protein
Locus_14226_Transcript_1/1_Conf_1.000	432	2	6.60E-06	NP_499664.2	48	53.9138	116	56	hypothetical protein BE10.2
Locus_14227_Transcript_1/1_Conf_1.000	400	9	1.35E-06	XP_002630073.1	52	56.225	91	48	Hypothetical protein CBG13452
Locus_14228_Transcript_1/1_Conf_1.000	187	0							
Locus_14229_Transcript_1/1_Conf_1.000	197	0							
Locus_1423_Transcript_1/1_Conf_1.000	1472	20	1.30E-115	NP_001024665.1	67	421.394	449	303	SynDaPiN (synaptic dynamin binding protein) homolog family member (sdpn-1)
Locus_14230_Transcript_1/1_Conf_1.000	443	20	6.07E-15	XP_001520430.1	62	83.9593	80	50	PREDICTED: similar to N-acetylglucosaminyltransferase V, partial
Locus_14231_Transcript_1/1_Conf_1.000	505	0							
Locus_14232_Transcript_1/1_Conf_1.000	285	20	2.31E-22	NP_491075.2	77	108.612	94	73	hypothetical protein Y71F9AL.1
Locus_14233_Transcript_1/1_Conf_1.000	523	0							
Locus_14234_Transcript_1/1_Conf_1.000	741	20	2.38E-37	XP_001900728.1	51	159.844	269	139	RhoGAP domain containing protein
Locus_14235_Transcript_1/1_Conf_1.000	805	20	8.46E-10	XP_862879.1	44	68.5514	218	97	PREDICTED: similar to Syntaxin-18 isoform 4

Locus_14236_Transcript_1/1_Conf_1.000	250	20	2.09E-36	AAH02110.2	98	155.221	75	74	Rpl24 protein
Locus_14237_Transcript_1/1_Conf_1.000	283	3	4.99E-25	NP_001041206.1	75	117.472	96	72	hypothetical protein ZC487.1
Locus_14238_Transcript_1/1_Conf_1.000	361	20	8.32E-09	EFO24006.1	61	63.5438	72	44	hypothetical protein LOAG_04478
Locus_14239_Transcript_1/3_Conf_0.600	780	20	5.95E-90	XP_002631584.1	82	334.724	264	219	Hypothetical protein CBG20762
Locus_14239_Transcript_2/3_Conf_0.600	781	20	5.95E-90	XP_002631584.1	82	334.724	264	219	Hypothetical protein CBG20762
Locus_14239_Transcript_3/3_Conf_0.600	781	20	5.95E-90	XP_002631584.1	82	334.724	264	219	Hypothetical protein CBG20762
Locus_1424_Transcript_1/2_Conf_1.000	1073	5	1.70E-36	XP_002641061.1	72	157.918	154	112	Hypothetical protein CBG22473
Locus_1424_Transcript_2/2_Conf_1.000	1152	5	1.90E-36	XP_002641061.1	72	157.918	154	112	Hypothetical protein CBG22473
Locus_14240_Transcript_1/1_Conf_1.000	765	20	1.20E-55	XP_002631403.1	70	220.705	220	154	Hypothetical protein CBG03253
Locus_14241_Transcript_1/1_Conf_1.000	824	0							
Locus_14242_Transcript_1/1_Conf_1.000	364	0							
Locus_14243_Transcript_1/1_Conf_1.000	284	0							
Locus_14244_Transcript_1/1_Conf_1.000	202	4	2.61E-10	NP_499313.2	70	68.5514	67	47	hypothetical protein F43D9.1

Locus_14245_Transcript_1/1_Conf_1.000	373	20	6.17E-68	BAG59411.1	100	259.996	119	119	unnamed protein product
Locus_14246_Transcript_1/1_Conf_1.000	337	20	6.47E-62	CAA68491.1	100	239.965	112	112	glutathione peroxidase
Locus_14247_Transcript_1/1_Conf_1.000	326	0							

Locus_14248_Transcript_1/1_Conf_1.000	198	20	5.07E-30	XP_002923753.1	100	134.035	65	65	PREDICTED: rRNA 2'-O-methyltransferase fibrillarin-like
Locus_14249_Transcript_1/1_Conf_1.000	741	20	2.65E-12	XP_002416765.1	49	76.6406	147	73	Sin3p-Rpd3p histone-deacetylase complex, DNA binding subunit, putative
Locus_1425_Transcript_1/1_Conf_1.000	356	7	2.79E-04	XP_002782358.1	69	48.521	42	29	conserved hypothetical protein
Locus_14250_Transcript_1/1_Conf_1.000	483	0							
Locus_14251_Transcript_1/1_Conf_1.000	377	20	3.87E-54	XP_002638687.1	93	214.157	124	116	Hypothetical protein CBG11882
Locus_14252_Transcript_1/1_Conf_1.000	317	0							
Locus_14253_Transcript_1/1_Conf_1.000	258	0							
Locus_14254_Transcript_1/2_Conf_1.000	464	0							
Locus_14254_Transcript_2/2_Conf_1.000	351	0							
Locus_14255_Transcript_1/1_Conf_1.000	253	0							
Locus_14256_Transcript_1/1_Conf_1.000	181	0							
Locus_14257_Transcript_1/1_Conf_1.000	626	0							
Locus_14258_Transcript_1/1_Conf_1.000	465	20	3.63E-44	NP_490714.1	87	181.03	107	94	hypothetical protein C53D5.5
Locus_14259_Transcript_1/1_Conf_1.000	546	20	6.91E-51	XP_002631157.1	85	203.756	144	123	C. briggsae CBR-RPN-9 protein
Locus_1426_Transcript_1/1_Conf_1.000	180	0							
Locus_14260_Transcript_1/1_Conf_1.000	131	0							
Locus_14261_Transcript_1/1_Conf_1.000	195	0							
Locus_14262_Transcript_1/1_Conf_1.000	354	0							
Locus_14263_Transcript_1/1_Conf_1.000	341	20	5.14E-35	XP_002639310.1	87	150.599	113	99	Hypothetical protein CBG03881
Locus_14264_Transcript_1/1_Conf_1.000	178	0							
Locus_14265_Transcript_1/1_Conf_1.000	154	0							
Locus_14266_Transcript_1/1_Conf_1.000	1401	20	4.04E-111	XP_002631389.1	75	406.371	319	241	C. briggsae CBR-OOC-5 protein
Locus_14267_Transcript_1/1_Conf_1.000	676	1	7.48E-05	NP_505951.1	68	51.6026	89	61	hypothetical protein T27F2.4
Locus_14268_Transcript_1/1_Conf_1.000	138	0							
Locus_14269_Transcript_1/1_Conf_1.000	363	3	1.01E-06	NP_507800.2	70	56.6102	50	35	hypothetical protein B0399.1

Locus_1427_Transcript_1/1_Conf_1.000	1976	20	9.60E-144	NP_001033327.1	71	515.383	500	359	CAN cell Migration defective family member (cam-1)
Locus_14270_Transcript_1/1_Conf_1.000	175	14	4.71E-12	NP_505743.1	78	74.3294	56	44	hypothetical protein F28H7.3
Locus_14271_Transcript_1/1_Conf_1.000	284	0							
Locus_14272_Transcript_1/1_Conf_1.000	203	0							
Locus_14273_Transcript_1/1_Conf_1.000	212	6	9.51E-05	NP_001122663.1	59	50.0618	66	39	hypothetical protein Y53F4B.39
Locus_14274_Transcript_1/1_Conf_1.000	416	20	5.15E-35	XP_002634297.1	87	150.599	109	95	Hypothetical protein CBG17635
Locus_14275_Transcript_1/1_Conf_1.000	408	0							
Locus_14276_Transcript_1/1_Conf_1.000	725	20	2.45E-100	NP_491653.2	84	369.007	241	203	hypothetical protein F55F8.3
Locus_14277_Transcript_1/1_Conf_1.000	222	0							
Locus_14278_Transcript_1/2_Conf_1.000	202	0							
Locus_14278_Transcript_2/2_Conf_1.000	154	0							
Locus_14279_Transcript_1/1_Conf_1.000	182	0							
Locus_1428_Transcript_1/1_Conf_1.000	226	0							
Locus_14280_Transcript_1/1_Conf_1.000	301	0							
Locus_14281_Transcript_1/1_Conf_1.000	910	20	2.16E-39	EFO22896.1	62	167.162	260	163	hypothetical protein LOAG_05591
Locus_14282_Transcript_1/1_Conf_1.000	461	20	3.68E-41	CAD44516.1	76	171.014	152	117	VAB-10B protein

Locus_14283_Transcript_1/1_Conf_1.000	177	7	4.39E-10	NP_509266.1	72	67.781	58	42	hypothetical protein F48E3.2
Locus_14284_Transcript_1/1_Conf_1.000	582	4	6.42E-19	NP_491128.4	57	97.8265	192	111	Conserved Oligomeric Golgi (COG) Component family member (cogc-1)
Locus_14285_Transcript_1/1_Conf_1.000	460	20	2.48E-13	XP_001380323.1	53	78.5666	132	71	PREDICTED: hypothetical protein
Locus_14286_Transcript_1/1_Conf_1.000	622	0							
Locus_14287_Transcript_1/1_Conf_1.000	263	0							
Locus_14288_Transcript_1/1_Conf_1.000	407	20	2.34E-27	XP_002630954.1	69	125.176	143	100	C. briggsae CBR-TAG-184 protein
Locus_14289_Transcript_1/1_Conf_1.000	249	20	4.99E-22	XP_001902912.1	82	107.457	80	66	aminotransferase, class V family protein
Locus_1429_Transcript_1/1_Conf_1.000	524	20	2.06E-67	CAR63531.1	93	258.455	147	138	putative Prion-like-(Q/N-rich)-domain-bearing protein
Locus_14290_Transcript_1/1_Conf_1.000	193	20	1.49E-13	XP_002762172.1	100	79.337	38	38	PREDICTED: 26S protease regulatory subunit 6B
Locus_14291_Transcript_1/1_Conf_1.000	329	0							
Locus_14292_Transcript_1/2_Conf_1.000	922	20	5.23E-65	XP_002648431.1	84	252.292	217	183	C. briggsae CBR-LPD-8 protein
Locus_14292_Transcript_2/2_Conf_1.000	922	20	5.23E-65	XP_002648431.1	84	252.292	217	183	C. briggsae CBR-LPD-8 protein
Locus_14293_Transcript_1/2_Conf_1.000	442	3	9.07E-11	NP_001023436.1	55	70.0922	128	71	hypothetical protein Y37E11AL.3
Locus_14293_Transcript_2/2_Conf_1.000	442	3	9.07E-11	NP_001023436.1	55	70.0922	128	71	hypothetical protein Y37E11AL.3
Locus_14294_Transcript_1/1_Conf_1.000	458	4	2.50E-13	XP_002640105.1	52	78.5666	149	78	C. briggsae CBR-MUT-2 protein
Locus_14295_Transcript_1/1_Conf_1.000	551	20	1.02E-25	NP_498886.2	53	120.168	163	88	hypothetical protein C06E1.3
Locus_14296_Transcript_1/1_Conf_1.000	372	0							
Locus_14297_Transcript_1/1_Conf_1.000	279	1	2.85E-09	EAX01292.1	72	65.0846	47	34	hCG1648136
Locus_14298_Transcript_1/1_Conf_1.000	177	0							
Locus_14299_Transcript_1/1_Conf_1.000	185	0							

Locus_143_Transcript_1/4_Conf_0.727	3170	20	0	AAM73861.1	85	1433.31	1045	889	AF457202_1putative serine/threonine protein kinase
Locus_143_Transcript_2/4_Conf_0.727	3347	20	0	AAM73859.1	91	1497.26	963	884	AF457200_1putative serine/threonine protein kinase
Locus_143_Transcript_3/4_Conf_0.727	3251	20	0	AAM73861.1	91	1519.6	1030	940	AF457202_1putative serine/threonine protein kinase
Locus_143_Transcript_4/4_Conf_0.727	3125	20	0	AAM73861.1	86	1439.86	1030	895	AF457202_1putative serine/threonine protein kinase
Locus_1430_Transcript_1/1_Conf_1.000	539	3	3.83E-43	XP_002633338.1	77	177.948	144	112	Hypothetical protein CBG06077
Locus_14300_Transcript_1/1_Conf_1.000	206	0							
Locus_14301_Transcript_1/1_Conf_1.000	205	0							
Locus_14302_Transcript_1/1_Conf_1.000	265	0							
Locus_14303_Transcript_1/1_Conf_1.000	1465	20	1.09E-21	NP_001090490.1	66	109.383	89	59	inhibitor of growth family, member 1-like
Locus_14304_Transcript_1/1_Conf_1.000	146	17	1.37E-14	A8WQT4.2	92	82.8037	42	39	Calcium uptake protein 1 homolog
Locus_14305_Transcript_1/1_Conf_1.000	247	1	7.26E-05	NP_502613.2	56	50.447	80	45	QUInine non-avoider family member (qui-1)
Locus_14306_Transcript_1/1_Conf_1.000	838	20	2.56E-113	XP_002640628.1	84	412.535	279	236	C. briggsae CBR-EAT-18 protein
Locus_14307_Transcript_1/1_Conf_1.000	377	0							
Locus_14308_Transcript_1/1_Conf_1.000	323	5	1.03E-06	XP_001893560.1	74	56.6102	47	35	hypothetical protein Bm1_10440
Locus_14309_Transcript_1/1_Conf_1.000	224	0							

Locus_1431_Transcript_1/1_Conf_1.000	755	20	3.72E-70	XP_002640472.1	82	268.855	214	177	C. briggsae CBR-RPS-6 protein
Locus_14310_Transcript_1/2_Conf_1.000	997	20	1.02E-56	NP_741459.2	61	224.942	326	202	WASP (actin cytoskeleton modulator) homolog family member (wsp-1)
Locus_14310_Transcript_2/2_Conf_1.000	988	20	1.44E-55	NP_741459.2	60	221.09	323	197	WASP (actin cytoskeleton modulator) homolog family member (wsp-1)
Locus_14311_Transcript_1/1_Conf_1.000	172	0							
Locus_14312_Transcript_1/1_Conf_1.000	174	0							
Locus_14313_Transcript_1/1_Conf_1.000	500	3	8.50E-38	NP_493074.2	77	159.844	132	102	hypothetical protein R05D7.1
Locus_14314_Transcript_1/1_Conf_1.000	144	0							
Locus_14315_Transcript_1/1_Conf_1.000	256	2	1.72E-09	EFO18967.1	61	65.855	85	52	LIM domain-containing protein
Locus_14316_Transcript_1/1_Conf_1.000	624	0							

Locus_14317_Transcript_1/1_Conf_1.000	238	20	5.43E-08	XP_002930758.1	100	60.8474	45	45	PREDICTED: melanoma-associated antigen D1-like
Locus_14318_Transcript_1/2_Conf_1.000	651	0							
Locus_14318_Transcript_2/2_Conf_1.000	636	0							
Locus_14319_Transcript_1/1_Conf_1.000	1110	20	1.27E-106	NP_497939.2	76	390.963	341	260	Suppressor/Enhancer of Lin-12 family member (sel-2)
Locus_1432_Transcript_1/1_Conf_1.000	1709	20	3.59E-168	NP_502152.3	77	596.275	470	362	CYtochrome P450 family member (cyp-31A2)
Locus_14320_Transcript_1/2_Conf_1.000	352	20	1.06E-19	XP_001900152.1	62	99.7525	114	71	Actin family protein
Locus_14320_Transcript_2/2_Conf_1.000	427	20	2.69E-23	XP_002629986.1	81	111.694	79	64	Hypothetical protein CBG13348
Locus_14321_Transcript_1/1_Conf_1.000	853	20	1.81E-37	NP_496498.1	68	160.614	183	125	hypothetical protein C47D12.8
Locus_14322_Transcript_1/1_Conf_1.000	973	20	1.76E-82	XP_002638826.1	73	310.457	326	240	Hypothetical protein CBG22031
Locus_14323_Transcript_1/1_Conf_1.000	512	20	9.41E-43	ACI49172.1	68	176.407	168	115	hypothetical protein Csp3_JD01.004
Locus_14324_Transcript_1/1_Conf_1.000	156	0							
Locus_14325_Transcript_1/1_Conf_1.000	235	0							
Locus_14326_Transcript_1/1_Conf_1.000	772	0							
Locus_14327_Transcript_1/1_Conf_1.000	469	4	2.70E-15	XP_002639461.1	62	85.1149	91	57	Hypothetical protein CBG04056
Locus_14328_Transcript_1/1_Conf_1.000	213	0							
Locus_14329_Transcript_1/1_Conf_1.000	620	0							
Locus_1433_Transcript_1/1_Conf_1.000	202	20	8.38E-17	XP_002634622.1	77	90.1225	67	52	Hypothetical protein CBG18478
Locus_14330_Transcript_1/1_Conf_1.000	534	0							
Locus_14331_Transcript_1/1_Conf_1.000	229	0							

Locus_14332_Transcript_1/1_Conf_1.000	261	20	3.41E-34	NP_504245.3	95	147.902	86	82	hypothetical protein Y61A9LA.10
Locus_14333_Transcript_1/1_Conf_1.000	188	0							
Locus_14334_Transcript_1/1_Conf_1.000	816	20	8.97E-100	NP_001022287.1	79	367.466	272	216	hypothetical protein R53.1
Locus_14335_Transcript_1/1_Conf_1.000	368	20	9.69E-34	XP_002639434.1	82	146.362	112	92	Hypothetical protein CBG04027
Locus_14336_Transcript_1/1_Conf_1.000	442	20	9.91E-50	AAB59518.1	100	199.519	100	100	apolipoprotein E
Locus_14337_Transcript_1/1_Conf_1.000	578	20	7.50E-65	NP_001023936.1	81	250.366	192	157	hypothetical protein F46B6.6
Locus_14338_Transcript_1/1_Conf_1.000	180	0							
Locus_14339_Transcript_1/1_Conf_1.000	165	0							
Locus_1434_Transcript_1/2_Conf_1.000	1010	1	2.68E-04	XP_001899348.1	64	50.8322	54	35	Probable coatomer beta' subunit

Locus_1434_Transcript_2/2_Conf_1.000	959	1	2.45E-04	XP_001899348.1	64	50.8322	54	35	Probable coatomer beta' subunit
Locus_14340_Transcript_1/1_Conf_1.000	341	20	2.30E-51	XP_002638788.1	91	204.912	113	103	Hypothetical protein CBG05143
Locus_14341_Transcript_1/1_Conf_1.000	574	0							
Locus_14342_Transcript_1/1_Conf_1.000	192	0							
Locus_14343_Transcript_1/1_Conf_1.000	293	6	9.98E-18	Q8I0P7.3	75	93.2041	84	63	Probable 3',5'-cyclic phosphodiesterase pde-3
Locus_14344_Transcript_1/1_Conf_1.000	355	0							
Locus_14345_Transcript_1/1_Conf_1.000	305	6	8.61E-14	ADI24640.1	57	80.1073	96	55	Hypothetical protein C56E6.9
Locus_14346_Transcript_1/1_Conf_1.000	175	0							
Locus_14347_Transcript_1/1_Conf_1.000	221	20	9.07E-35	XP_002759394.1	100	149.828	73	73	PREDICTED: polyadenylate-binding protein 1-like
Locus_14348_Transcript_1/1_Conf_1.000	217	20	1.45E-13	ADI61822.1	68	79.337	69	47	endonuclease-reverse transcriptase
Locus_14349_Transcript_1/1_Conf_1.000	144	0							
Locus_1435_Transcript_1/1_Conf_1.000	718	0							
Locus_14350_Transcript_1/1_Conf_1.000	485	20	1.26E-28	NP_001076607.1	69	129.413	116	81	hypothetical protein K12C11.6
Locus_14351_Transcript_1/1_Conf_1.000	260	0							
Locus_14352_Transcript_1/1_Conf_1.000	723	20	7.17E-76	AAA28103.1	95	287.73	185	176	ras protein
Locus_14353_Transcript_1/2_Conf_1.000	663	20	1.15E-79	BAD92215.1	100	300.056	145	145	HLA class II histocompatibility antigen, DP beta chain precursor variant

Locus_14353_Transcript_2/2_Conf_1.000	553	20	7.35E-80	BAD92215.1	100	300.056	145	145	HLA class II histocompatibility antigen, DP beta chain precursor variant
Locus_14354_Transcript_1/1_Conf_1.000	258	20	9.94E-42	BAI45345.1	100	172.94	85	85	mannosidase, alpha, class 2B, member 1
Locus_14355_Transcript_1/1_Conf_1.000	513	3	3.78E-47	EFO27774.1	67	191.045	171	116	rap/ran-GAP family protein
Locus_14356_Transcript_1/1_Conf_1.000	686	20	5.95E-98	XP_001894292.1	87	360.918	226	198	cyclin-dependent kinase 7 homolog
Locus_14357_Transcript_1/1_Conf_1.000	544	20	1.08E-48	NP_504578.1	72	196.438	180	131	hypothetical protein F17A9.4
Locus_14358_Transcript_1/1_Conf_1.000	268	20	6.86E-19	NP_505920.1	72	97.0561	87	63	hypothetical protein C55A6.6
Locus_14359_Transcript_1/1_Conf_1.000	905	2	5.43E-43	NP_492185.2	59	179.104	295	175	SMG-associated and Lethal family member (smgl-1)
Locus_1436_Transcript_1/2_Conf_1.000	1214	20	1.17E-124	XP_002639724.1	84	451.055	354	299	C. briggsae CBR-MEL-26 protein

Locus_1436_Transcript_2/2_Conf_1.000	2114	20	2.42E-124	XP_002639724.1	84	451.055	354	299	C. briggsae CBR-MEL-26 protein
Locus_14360_Transcript_1/1_Conf_1.000	239	20	4.71E-36	XP_002640668.1	97	154.066	79	77	C. briggsae CBR-UNC-54 protein
Locus_14361_Transcript_1/1_Conf_1.000	534	8	2.37E-13	NP_496318.1	52	78.9518	171	89	ECT2 (mammalian Rho GEF) homolog family member (ect-2)
Locus_14362_Transcript_1/1_Conf_1.000	203	0							
Locus_14363_Transcript_1/1_Conf_1.000	333	2	2.25E-38	NP_498952.1	80	161.77	111	89	hypothetical protein F44E2.4
Locus_14364_Transcript_1/1_Conf_1.000	225	0							
Locus_14365_Transcript_1/1_Conf_1.000	262	20	1.76E-06	CAA85409.2	64	55.8398	45	29	C. elegans protein D2089.2, partially confirmed by transcript evidence
Locus_14366_Transcript_1/1_Conf_1.000	373	11	4.47E-26	NP_001021875.1	68	120.939	126	86	Leucyl tRNA Synthetase family member (lrs-2)
Locus_14367_Transcript_1/1_Conf_1.000	164	0							
Locus_14368_Transcript_1/1_Conf_1.000	269	0							
Locus_14369_Transcript_1/1_Conf_1.000	697	20	2.42E-09	XP_002631402.1	50	66.6254	180	90	Hypothetical protein CBG03252
Locus_1437_Transcript_1/1_Conf_1.000	955	20	3.37E-30	AAA92672.1	68	136.732	147	101	HMG CoA synthase
Locus_14370_Transcript_1/1_Conf_1.000	374	2	4.65E-07	XP_002647555.1	75	57.7658	48	36	Hypothetical protein CBG06641
Locus_14371_Transcript_1/1_Conf_1.000	709	0							
Locus_14372_Transcript_1/1_Conf_1.000	152	0							
Locus_14373_Transcript_1/1_Conf_1.000	132	0							
Locus_14374_Transcript_1/1_Conf_1.000	1714	20	3.62E-152	NP_496363.1	74	543.117	575	427	LEThal family member (let-858)

Locus_14375_Transcript_1/1_Conf_1.000	527	20	2.87E-32	NP_499126.1	63	141.739	157	99	DNA PRImase homolog family member (pri-1)
Locus_14376_Transcript_1/1_Conf_1.000	143	0							
Locus_14377_Transcript_1/1_Conf_1.000	180	19	3.97E-22	NP_498645.1	86	107.842	59	51	MUscle Positioning family member (mup-4)
Locus_14378_Transcript_1/1_Conf_1.000	562	20	8.46E-47	NP_001022572.1	69	190.274	195	136	Elongation Factor Kinase family member (efk-1)
Locus_14379_Transcript_1/1_Conf_1.000	271	0							
Locus_1438_Transcript_1/1_Conf_1.000	219	20	1.04E-38	XP_002762091.1	100	162.925	73	73	PREDICTED: glucose-6-phosphate isomerase like, partial
Locus_14380_Transcript_1/1_Conf_1.000	417	0							
Locus_14381_Transcript_1/1_Conf_1.000	525	0							
Locus_14382_Transcript_1/1_Conf_1.000	248	3	9.49E-05	NP_508523.1	55	50.0618	83	46	Temporarily Assigned Gene name family member (tag-312)
Locus_14383_Transcript_1/1_Conf_1.000	509	20	1.84E-46	NP_001021940.1	72	188.734	168	122	hypothetical protein C08H9.3
Locus_14384_Transcript_1/1_Conf_1.000	401	0							
Locus_14385_Transcript_1/1_Conf_1.000	231	20	2.89E-25	XP_002639432.1	80	118.242	77	62	C. briggsae CBR-TYR-4 protein
Locus_14386_Transcript_1/1_Conf_1.000	652	20	7.03E-74	XP_001901411.1	70	280.796	223	157	Thioredoxin family protein
Locus_14387_Transcript_1/1_Conf_1.000	155	0							

Locus_14388_Transcript_1/1_Conf_1.000	705	20	2.85E-66	NP_001122776.1	87	255.758	148	130	hypothetical protein F35G2.1
Locus_14389_Transcript_1/1_Conf_1.000	598	20	9.67E-66	XP_001900850.1	79	253.447	198	157	ERCC4 domain containing protein
Locus_1439_Transcript_1/1_Conf_1.000	561	20	4.50E-40	XP_002639226.1	96	167.933	91	88	Hypothetical protein CBG03777
Locus_14390_Transcript_1/1_Conf_1.000	500	20	4.24E-21	Q61SU7.3	60	104.375	141	85	ATP-dependent RNA helicase SUV3 homolog
Locus_14391_Transcript_1/1_Conf_1.000	264	20	2.06E-31	XP_002646681.1	91	138.658	85	78	C. briggsae CBR-PRX-5 protein
Locus_14392_Transcript_1/1_Conf_1.000	1037	20	3.77E-17		46	93.5893	283	131	hypothetical protein ZC13.1
Locus_14393_Transcript_1/2_Conf_1.000	398	0							
Locus_14393_Transcript_2/2_Conf_1.000	398	0							
Locus_14394_Transcript_1/1_Conf_1.000	563	20	2.38E-49	XP_001901731.1	72	198.749	188	136	Guanine nucleotide-binding protein alpha-3 subunit

Locus_14395_Transcript_1/1_Conf_1.000	258	0							
Locus_14396_Transcript_1/1_Conf_1.000	458	0							
Locus_14397_Transcript_1/1_Conf_1.000	278	0							
Locus_14398_Transcript_1/1_Conf_1.000	133	20	2.50E-13	NP_001023736.1	90	78.5666	44	40	Na/H eXchanger family member (nhx-3)
Locus_14399_Transcript_1/1_Conf_1.000	139	0							
Locus_144_Transcript_1/1_Conf_1.000	1533	20	0	XP_001895190.1	81	681.404	483	396	Receptor family ligand binding region containing protein
Locus_1440_Transcript_1/1_Conf_1.000	1065	20	1.82E-123	XP_002637266.1	78	446.817	330	260	Hypothetical protein CBG18949
Locus_14400_Transcript_1/1_Conf_1.000	172	0							
Locus_14401_Transcript_1/1_Conf_1.000	262	0							
Locus_14402_Transcript_1/1_Conf_1.000	369	20	6.96E-11	NP_493178.1	67	70.4774	78	53	hypothetical protein W02D9.3
Locus_14403_Transcript_1/1_Conf_1.000	208	0							
Locus_14404_Transcript_1/1_Conf_1.000	767	0							
Locus_14405_Transcript_1/3_Conf_0.667	518	1	2.71E-08	NP_001122478.1	74	62.003	43	32	hypothetical protein F45H11.3
Locus_14405_Transcript_2/3_Conf_0.667	525	1	2.89E-08	NP_001122478.1	74	62.003	43	32	hypothetical protein F45H11.3
Locus_14405_Transcript_3/3_Conf_0.667	534	1	3.00E-08	NP_001122478.1	74	62.003	43	32	hypothetical protein F45H11.3
Locus_14406_Transcript_1/1_Conf_1.000	238	0							
Locus_14407_Transcript_1/1_Conf_1.000	243	0							
Locus_14408_Transcript_1/2_Conf_1.000	509	20	3.35E-48	NP_499553.1	85	194.512	123	105	yeast CCR4 Associated Factor family member (ccf-1)

Locus_14408_Transcript_2/2_Conf_1.000	509	20	3.35E-48	NP_499553.1	85	194.512	123	105	yeast CCR4 Associated Factor family member (ccf-1)
Locus_14409_Transcript_1/1_Conf_1.000	351	5	1.14E-13	XP_002639084.1	65	79.7221	88	58	Hypothetical protein CBG14902
Locus_1441_Transcript_1/1_Conf_1.000	856	20	8.07E-86	NP_499133.1	98	321.242	162	160	UBiquitin Conjugating enzyme family member (ubc-7)
Locus_14410_Transcript_1/1_Conf_1.000	432	20	6.54E-38	NP_498982.1	80	160.229	111	89	hypothetical protein R08D7.1
Locus_14411_Transcript_1/1_Conf_1.000	1595	20	0	XP_002643273.1	85	742.265	523	447	C. briggsae CBR-MRP-1 protein
Locus_14412_Transcript_1/1_Conf_1.000	539	18	5.31E-69	XP_002640063.1	90	263.848	166	151	Hypothetical protein CBG12545
Locus_14413_Transcript_1/1_Conf_1.000	767	20	1.62E-52	EFO24017.1	65	210.305	238	157	hypothetical protein LOAG_04469
Locus_14414_Transcript_1/1_Conf_1.000	1089	4	2.69E-53	NP_509401.2	54	213.772	339	184	hypothetical protein R09F10.3
Locus_14415_Transcript_1/1_Conf_1.000	310	0							
Locus_14416_Transcript_1/1_Conf_1.000	128	0							
Locus_14417_Transcript_1/1_Conf_1.000	257	19	5.50E-24	EFO18138.1	83	114.005	85	71	hypothetical protein LOAG_10361

Locus_14418_Transcript_1/1_Conf_1.000	318	4	2.55E-05	XP_001895677.1	86	51.9878	36	31	Actin family protein
Locus_14419_Transcript_1/1_Conf_1.000	451	20	2.25E-62	EFO28147.1	98	241.506	120	118	pre-mRNA splicing factor PRP8
Locus_1442_Transcript_1/1_Conf_1.000	1047	20	1.13E-45	NP_495323.1	56	188.348	352	199	Temporarily Assigned Gene name family member (tag-184)
Locus_14420_Transcript_1/1_Conf_1.000	152	2	8.52E-09	XP_002629821.1	81	63.5438	48	39	C. briggsae CBR-SQV-8 protein
Locus_14421_Transcript_1/2_Conf_1.000	448	0							
Locus_14421_Transcript_2/2_Conf_1.000	448	0							
Locus_14422_Transcript_1/1_Conf_1.000	168	20	5.60E-13	XP_001607068.1	81	77.411	60	49	PREDICTED: similar to polyribonucleotide nucleotidyltransferase
Locus_14423_Transcript_1/1_Conf_1.000	664	2	1.44E-11	NP_492348.1	51	73.559	117	60	hypothetical protein K07A12.2
Locus_14424_Transcript_1/1_Conf_1.000	502	20	1.03E-22	XP_002639569.1	82	109.768	112	92	Hypothetical protein CBG04200
Locus_14425_Transcript_1/2_Conf_1.000	666	20	4.07E-32	XP_002640276.1	72	142.124	177	128	Hypothetical protein CBG12801
Locus_14425_Transcript_2/2_Conf_1.000	587	20	1.11E-34	XP_002640276.1	72	150.214	170	123	Hypothetical protein CBG12801
Locus_14426_Transcript_1/1_Conf_1.000	558	0							
Locus_14427_Transcript_1/1_Conf_1.000	813	20	5.00E-18	XP_001623641.1	37	95.9005	264	99	predicted protein
Locus_14428_Transcript_1/1_Conf_1.000	264	0							
Locus_14429_Transcript_1/1_Conf_1.000	573	20	8.19E-48	CBA11992.1	62	193.741	191	120	endonuclease-reverse transcriptase HmRTE-e01
Locus_1443_Transcript_1/1_Conf_1.000	681	20	1.58E-34	NP_498150.1	64	150.214	170	110	hypothetical protein Y32H12A.7
Locus_14430_Transcript_1/1_Conf_1.000	277	4	3.61E-12	XP_001896695.1	62	74.7146	91	57	Hr1 repeat family protein
Locus_14431_Transcript_1/1_Conf_1.000	337	0							

Locus_14432_Transcript_1/1_Conf_1.000	241	3	2.42E-16	EFO23234.1	69	88.5817	66	46	hypothetical protein LOAG_05253
Locus_14433_Transcript_1/1_Conf_1.000	219	20	4.50E-34	EAW69389.1	100	147.517	71	71	LSM7 homolog, U6 small nuclear RNA associated (S. cerevisiae), isoform CRA_a
Locus_14434_Transcript_1/1_Conf_1.000	261	2	1.14E-05	XP_002634568.1	84	53.1434	52	44	Hypothetical protein CBG08374
Locus_14435_Transcript_1/1_Conf_1.000	270	20	5.04E-30	XP_001898305.1	79	134.035	87	69	Transmembrane 9 superfamily protein member 2 precursor, putative
Locus_14436_Transcript_1/1_Conf_1.000	281	0							
Locus_14437_Transcript_1/1_Conf_1.000	338	0							
Locus_14438_Transcript_1/1_Conf_1.000	165	7	1.17E-18	XP_002639508.1	96	96.2857	51	49	Hypothetical protein CBG04113
Locus_14439_Transcript_1/1_Conf_1.000	221	20	1.50E-29	NP_494777.1	90	132.494	73	66	MoLTing defective family member (mlt-7)
Locus_1444_Transcript_1/2_Conf_1.000	800	20	2.46E-78	NP_498360.2	81	296.204	227	184	hypothetical protein T12A2.7
Locus_1444_Transcript_2/2_Conf_1.000	800	20	2.46E-78	NP_498360.2	81	296.204	227	184	hypothetical protein T12A2.7
Locus_14440_Transcript_1/1_Conf_1.000	394	20	9.94E-50	EFO26261.1	87	199.519	131	115	hypothetical protein LOAG_02219
Locus_14441_Transcript_1/1_Conf_1.000	251	3	4.49E-39	XP_002830960.1	95	164.081	83	79	PREDICTED: hypothetical protein LOC100438111
Locus_14442_Transcript_1/1_Conf_1.000	323	20	5.25E-43	XP_002633186.1	92	177.178	104	96	Hypothetical protein CBG05900
Locus_14443_Transcript_1/1_Conf_1.000	1117	20	4.32E-54	NP_741217.1	67	216.468	223	151	EXOSome (multiexonuclease complex) component family member (exos-9)
Locus_14444_Transcript_1/2_Conf_1.000	898	8	9.44E-48	NP_871881.1	70	194.897	244	172	hypothetical protein F30F8.9
Locus_14444_Transcript_2/2_Conf_1.000	568	10	7.43E-38	NP_492165.1	65	160.614	186	121	hypothetical protein F30F8.9

Locus_14445_Transcript_1/1_Conf_1.000	218	20	2.02E-23	XP_001898626.1	88	112.079	72	64	Pyruvate kinase, muscle isozyme
Locus_14446_Transcript_1/2_Conf_1.000	597	0							
Locus_14446_Transcript_2/2_Conf_1.000	511	0							
Locus_14447_Transcript_1/1_Conf_1.000	161	0							
Locus_14448_Transcript_1/1_Conf_1.000	129	0							
Locus_14449_Transcript_1/1_Conf_1.000	218	0							
Locus_1445_Transcript_1/2_Conf_1.000	2002	20	6.72E-93	XP_002643215.1	68	342.813	326	223	Hypothetical protein CBG08080
Locus_1445_Transcript_2/2_Conf_1.000	1973	20	4.66E-98	XP_002643215.1	66	363.614	365	242	Hypothetical protein CBG08080
Locus_14450_Transcript_1/1_Conf_1.000	186	0							
Locus_14451_Transcript_1/1_Conf_1.000	148	0							
Locus_14452_Transcript_1/1_Conf_1.000	200	20	2.89E-17	XP_002634283.1	78	91.6633	65	51	C. briggsae CBR-HRS-1 protein
Locus_14453_Transcript_1/1_Conf_1.000	922	20	4.39E-96	NP_509283.1	76	355.525	273	208	APYrase family member (apy-1)
Locus_14454_Transcript_1/1_Conf_1.000	130	2	9.57E-05	NP_498688.1	67	50.0618	43	29	Prolyl Carboxy Peptidase like family member (pcp-1)
Locus_14455_Transcript_1/1_Conf_1.000	886								
Locus_14456_Transcript_1/1_Conf_1.000	422	0							
Locus_14457_Transcript_1/1_Conf_1.000	572	20	6.34E-69	NP_505519.3	81	263.848	186	152	UNCoordinated family member (unc-42)
Locus_14458_Transcript_1/1_Conf_1.000	469	0							
Locus_14459_Transcript_1/1_Conf_1.000	477	2	1.08E-08	XP_002637934.1	48	63.1586	152	74	Hypothetical protein CBG04746

Locus_1446_Transcript_1/2_Conf_1.000	1659	20	3.77E-160	XP_001896639.1	93	557.755	309	289	hypothetical protein
Locus_1446_Transcript_2/2_Conf_1.000	1595	20	8.91E-166	XP_001896639.1	92	588.186	329	305	hypothetical protein
Locus_14460_Transcript_1/1_Conf_1.000	381	20	5.05E-30	NP_498764.1	70	134.035	125	88	GTP exchange factor for ARFs family member (grp-1)
Locus_14461_Transcript_1/1_Conf_1.000	270	0							
Locus_14462_Transcript_1/1_Conf_1.000	540	0							
Locus_14463_Transcript_1/1_Conf_1.000	490	0							
Locus_14464_Transcript_1/1_Conf_1.000	137	0							
Locus_14465_Transcript_1/1_Conf_1.000	258	20	8.74E-22	NP_506083.1	77	106.686	76	59	hypothetical protein F55C5.8
Locus_14466_Transcript_1/1_Conf_1.000	324	0							
Locus_14467_Transcript_1/1_Conf_1.000	166	20	2.44E-08	ACO51818.1	70	62.003	55	39	Rho GDP-dissociation inhibitor 2
Locus_14468_Transcript_1/1_Conf_1.000	262	0							

Locus_14469_Transcript_1/2_Conf_1.000	948	20	4.99E-58	XP_002638726.1	73	229.18	240	176	Hypothetical protein CBG00309
Locus_14469_Transcript_2/2_Conf_1.000	948	20	4.99E-58	XP_002638726.1	73	229.18	240	176	Hypothetical protein CBG00309
Locus_1447_Transcript_1/1_Conf_1.000	615	0							
Locus_14470_Transcript_1/1_Conf_1.000	151	0							
Locus_14471_Transcript_1/1_Conf_1.000	158	0							
Locus_14472_Transcript_1/1_Conf_1.000	132	0							
Locus_14473_Transcript_1/1_Conf_1.000	158	20	3.00E-22	XP_002814804.1	100	108.227	52	52	PREDICTED: insulin-like growth factor-binding protein 7-like
Locus_14474_Transcript_1/1_Conf_1.000	387	0							
Locus_14475_Transcript_1/1_Conf_1.000	206	0							
Locus_14476_Transcript_1/1_Conf_1.000	536	20	3.08E-37	EFO23539.1	71	158.303	156	111	hypothetical protein LOAG_04946
Locus_14477_Transcript_1/1_Conf_1.000	294	0							
Locus_14478_Transcript_1/1_Conf_1.000	231	0							
Locus_14479_Transcript_1/1_Conf_1.000	207	0							
Locus_1448_Transcript_1/1_Conf_1.000	618	20	9.05E-57	XP_002630759.1	68	223.787	206	141	Hypothetical protein CBG02453
Locus_14480_Transcript_1/1_Conf_1.000	890	20	1.99E-66	EFO26982.1	90	256.914	152	138	PAR-6 protein

Locus_14481_Transcript_1/1_Conf_1.000	708	20	8.65E-71	NP_509361.1	87	270.781	195	171	hypothetical protein K07E3.4
Locus_14482_Transcript_1/1_Conf_1.000	165	0							
Locus_14483_Transcript_1/1_Conf_1.000	264	3	8.72E-06	NP_504274.2	70	53.5286	50	35	UDP-GlucuronosylTransferase family member (ugt-61)
Locus_14484_Transcript_1/1_Conf_1.000	183	20	5.92E-18	NP_001022451.1	92	93.9745	54	50	hypothetical protein Y48E1A.1
Locus_14485_Transcript_1/1_Conf_1.000	244	20	6.79E-19	XP_001641772.1	74	97.0561	79	59	predicted protein
Locus_14486_Transcript_1/1_Conf_1.000	215	6	5.19E-11	AAL56427.1	77	70.8626	54	42	AF370722_1intracellular globin
Locus_14487_Transcript_1/1_Conf_1.000	416	0							
Locus_14488_Transcript_1/1_Conf_1.000	484	0							
Locus_14489_Transcript_1/1_Conf_1.000	630	20	6.93E-60	XP_002637479.1	72	234.187	211	152	Hypothetical protein CBG19196
Locus_1449_Transcript_1/1_Conf_1.000	667	0							
Locus_14490_Transcript_1/1_Conf_1.000	248	3	1.91E-13	XP_002647006.1	75	78.9518	64	48	Hypothetical protein CBG22464
Locus_14491_Transcript_1/1_Conf_1.000	261	1	4.34E-05	XP_001899770.1	54	51.2174	82	45	hAT family dimerisation domain containing protein
Locus_14492_Transcript_1/1_Conf_1.000	314	1	2.85E-04	NP_497581.3	50	48.521	81	41	hypothetical protein Y54F10AR.2
Locus_14493_Transcript_1/1_Conf_1.000	221	0							
Locus_14494_Transcript_1/2_Conf_1.000	160	0							
Locus_14494_Transcript_2/2_Conf_1.000	347	0							
Locus_14495_Transcript_1/1_Conf_1.000	183	5	3.47E-18	NP_001023620.1	81	94.7449	60	49	hypothetical protein ZK792.4
Locus_14496_Transcript_1/1_Conf_1.000	602	20	8.42E-17	EFO27042.1	46	90.8929	189	88	hypothetical protein LOAG_01440
Locus_14497_Transcript_1/1_Conf_1.000	386	0							
Locus_14498_Transcript_1/1_Conf_1.000	249	20	4.99E-22	XP_002427316.1	70	107.457	81	57	transportin-2, putative
Locus_14499_Transcript_1/1_Conf_1.000	200	0							

Locus_145_Transcript_1/3_Conf_0.600	897	0							
Locus_145_Transcript_2/3_Conf_0.600	1436	20	1.17E-12	NP_001021459.2	37	79.337	359	134	hypothetical protein F36H2.3
Locus_145_Transcript_3/3_Conf_0.600	908	0							
Locus_1450_Transcript_1/1_Conf_1.000	1424	20	0	NP_741215.1	83	681.789	436	366	NADH Ubiquinone Oxidoreductase family member (nuo-4)
Locus_14500_Transcript_1/1_Conf_1.000	417	20	9.98E-34	EFO21654.1	70	146.362	137	97	hypothetical protein LOAG_06832
Locus_14501_Transcript_1/1_Conf_1.000	401	3	1.39E-19	NP_507813.1	62	99.3673	116	72	hypothetical protein Y43F8C.13
Locus_14502_Transcript_1/1_Conf_1.000	264	9	5.65E-05	NP_504738.1	60	50.8322	70	42	COLlagen family member (col-143)
Locus_14503_Transcript_1/1_Conf_1.000	478	4	5.56E-13	EFO24423.1	63	77.411	119	76	hypothetical protein LOAG_04059
Locus_14504_Transcript_1/1_Conf_1.000	900	20	1.11E-80	NP_503421.1	73	304.294	278	204	Hydroxy-Acyl-CoA Dehydrogenase family member (hacd-1)
Locus_14505_Transcript_1/1_Conf_1.000	466	20	1.74E-54	AAD45606.1	92	215.312	124	115	galectin 3
Locus_14506_Transcript_1/1_Conf_1.000	139	0							
Locus_14507_Transcript_1/1_Conf_1.000	351	20	1.69E-33	NP_496144.1	94	145.591	87	82	hypothetical protein C05C10.3
Locus_14508_Transcript_1/1_Conf_1.000	190	20	6.07E-23	CAR63575.1	87	110.538	63	55	hypothetical protein
Locus_14509_Transcript_1/1_Conf_1.000	285	0							

Locus_1451_Transcript_1/2_Conf_1.000	1673	20	5.98E-35	XP_002635620.1	61	153.68	212	130	Hypothetical protein CBG21813
Locus_1451_Transcript_2/2_Conf_1.000	1673	20	5.98E-35	XP_002635620.1	61	153.68	212	130	Hypothetical protein CBG21813
Locus_14510_Transcript_1/1_Conf_1.000	137	2	2.11E-12	CAE17761.2	91	75.485	36	33	C. elegans protein C48D5.3, confirmed by transcript evidence
Locus_14511_Transcript_1/1_Conf_1.000	309	0							
Locus_14512_Transcript_1/1_Conf_1.000	409	20	1.50E-58	AAH71725.1	97	228.794	131	128	IGL@ protein
Locus_14513_Transcript_1/1_Conf_1.000	712	20	3.36E-30	NP_499239.1	78	135.961	85	67	hypothetical protein T16H12.4
Locus_14514_Transcript_1/1_Conf_1.000	938	20	1.05E-68	NP_499903.3	62	264.618	314	197	Defecation Suppressor of Clk-1 family member (dsc-4)
Locus_14515_Transcript_1/1_Conf_1.000	139	0							
Locus_14516_Transcript_1/1_Conf_1.000	556	0							
Locus_14517_Transcript_1/1_Conf_1.000	344	20	1.27E-12	EFO18793.1	69	76.2554	75	52	hypothetical protein LOAG_09702
Locus_14518_Transcript_1/1_Conf_1.000	149	0							
Locus_14519_Transcript_1/1_Conf_1.000	197	0							
Locus_1452_Transcript_1/1_Conf_1.000	545	20	1.93E-13	XP_001902112.1	95	79.337	40	38	Iron-sulfur cluster assembly accessory protein
Locus_14520_Transcript_1/1_Conf_1.000	607	0							
Locus_14521_Transcript_1/1_Conf_1.000	208	20	1.33E-06	XP_002739131.1	59	56.225	67	40	PREDICTED: major facilitator superfamily domain containing 5-like
Locus_14522_Transcript_1/1_Conf_1.000	565	4	3.64E-29	NP_510486.2	54	131.724	172	93	hypothetical protein F20D1.3

Locus_14523_Transcript_1/2_Conf_1.000	692	3	6.45E-07	EFO21590.1	43	58.5362	211	92	hypothetical protein LOAG_06899
Locus_14523_Transcript_2/2_Conf_1.000	692	4	2.89E-07	EFO21590.1	44	59.6918	211	93	hypothetical protein LOAG_06899
Locus_14524_Transcript_1/1_Conf_1.000	577	20	9.81E-57	XP_002629673.1	82	223.402	149	123	Hypothetical protein CBG00891
Locus_14525_Transcript_1/1_Conf_1.000	444	20	1.99E-50	NP_492361.2	78	201.83	148	116	RNA Polymerase II (B) subunit family member (rpb-3)
Locus_14526_Transcript_1/1_Conf_1.000	241	2	3.87E-14	XP_002643403.1	75	81.2629	58	44	Hypothetical protein CBG16021
Locus_14527_Transcript_1/1_Conf_1.000	210	0							
Locus_14528_Transcript_1/1_Conf_1.000	287	5	5.72E-05	XP_002637333.1	65	50.8322	49	32	C. briggsae CBR-COL-142 protein
Locus_14529_Transcript_1/1_Conf_1.000	684	20	6.82E-86	NP_499668.1	79	320.857	228	181	hypothetical protein Y37D8A.2
Locus_1453_Transcript_1/2_Conf_1.000	1438	20	7.44E-84	NP_504551.3	82	315.849	224	184	Protein Carboxymethyltransferase family member (pcm-1)
Locus_1453_Transcript_2/2_Conf_1.000	1482	20	7.78E-84	NP_504551.3	82	315.849	224	184	Protein Carboxymethyltransferase family member (pcm-1)
Locus_14530_Transcript_1/1_Conf_1.000	242	20	2.67E-23	XP_002709090.1	83	111.694	80	67	PREDICTED: N-acetyltransferase 10
Locus_14531_Transcript_1/1_Conf_1.000	235	0							
Locus_14532_Transcript_1/1_Conf_1.000	357	0							
Locus_14533_Transcript_1/1_Conf_1.000	299	20	5.05E-38	EFO27760.1	83	160.614	93	78	glycosyl hydrolase family 31 protein
Locus_14534_Transcript_1/1_Conf_1.000	239	20	6.39E-25	XP_002644187.1	87	117.087	73	64	Hypothetical protein CBG17169
Locus_14535_Transcript_1/1_Conf_1.000	221	0							
Locus_14536_Transcript_1/1_Conf_1.000	235	0							
Locus_14537_Transcript_1/2_Conf_1.000	1575	20	4.02E-94	NP_001022260.1	59	350.132	545	323	Initiation Factor 4G (eIF4G) family member (ifg-1)
Locus_14537_Transcript_2/2_Conf_1.000	1581	20	3.09E-94	NP_001022260.1	59	350.517	545	322	Initiation Factor 4G (eIF4G) family member (ifg-1)

Locus_14538_Transcript_1/1_Conf_1.000	180	5	8.59E-09	CAA16382.5	66	63.5438	60	40	C. elegans protein Y45F10D.7, partially confirmed by transcript evidence
Locus_14539_Transcript_1/1_Conf_1.000	303	20	4.25E-29	NP_509429.1	89	130.954	74	66	Temporarily Assigned Gene name family member (tag-123)
Locus_1454_Transcript_1/2_Conf_1.000	821	20	6.98E-68	XP_002635374.1	68	261.536	286	195	C. briggsae CBR-TAG-117 protein
Locus_1454_Transcript_2/2_Conf_1.000	1175	20	6.21E-75	XP_002635374.1	60	285.804	427	257	C. briggsae CBR-TAG-117 protein
Locus_14540_Transcript_1/1_Conf_1.000	730	20	7.01E-103	NP_001021145.1	85	377.481	240	206	hypothetical protein C05D11.1
Locus_14541_Transcript_1/1_Conf_1.000	189	20	4.80E-28	ACJ65168.1	96	127.487	62	60	Uncoordinated protein 68, isoform b, partially confirmed by transcript evidence
Locus_14542_Transcript_1/3_Conf_0.667	1307	20	1.48E-128	XP_001898423.1	74	464.151	434	323	Mediator complex subunit rgr-1
Locus_14542_Transcript_2/3_Conf_0.667	1467	20	1.24E-142	XP_001898423.1	73	511.146	490	360	Mediator complex subunit rgr-1
Locus_14542_Transcript_3/3_Conf_0.667	1467	20	1.24E-142	XP_001898423.1	73	511.146	490	360	Mediator complex subunit rgr-1
Locus_14543_Transcript_1/1_Conf_1.000	352	20	1.02E-38	XP_002631360.1	78	162.925	123	97	Hypothetical protein CBG03195

Locus_14544_Transcript_1/1_Conf_1.000	391	20	2.15E-49	AAN11401.1	81	198.364	128	104	metalloprotease 1 precursor
Locus_14545_Transcript_1/1_Conf_1.000	1738	20	1.46E-100	NP_001123105.1	64	371.703	487	312	CDC-42 Guanine nucleotide Exchange Factor family member (cgef-1)
Locus_14546_Transcript_1/1_Conf_1.000	315	20	1.60E-23	EFO20067.1	68	112.464	112	77	hypothetical protein LOAG_08425
Locus_14547_Transcript_1/1_Conf_1.000	283	20	3.71E-12	XP_002120612.1	60	74.7146	80	48	PREDICTED: similar to ATPase, aminophospholipid transporter (APLT), class I, type 8A, member 1, partial
Locus_14548_Transcript_1/1_Conf_1.000	191	0							
Locus_14549_Transcript_1/1_Conf_1.000	161	0							
Locus_1455_Transcript_1/1_Conf_1.000	496	20	8.78E-59	NP_497791.1	81	229.565	153	124	NITrilase family member (nit-1)
Locus_14550_Transcript_1/2_Conf_1.000	1052	20	2.34E-91	NP_500791.2	75	340.117	324	244	SAND endocytosis protein family member (sand-1)
Locus_14550_Transcript_2/2_Conf_1.000	459	20	3.46E-23	NP_500791.2	71	111.309	121	86	SAND endocytosis protein family member (sand-1)
Locus_14551_Transcript_1/1_Conf_1.000	302	20	2.28E-06	XP_002003728.1	49	55.4546	99	49	GI18068
Locus_14552_Transcript_1/1_Conf_1.000	152	3	1.50E-05	NP_494220.2	68	52.7582	50	34	hypothetical protein F54A3.1
Locus_14553_Transcript_1/1_Conf_1.000	238	20	3.75E-17	YP_003797541.1	80	91.2781	71	57	ATP-dependent protease La
Locus_14554_Transcript_1/1_Conf_1.000	525	20	2.97E-37	EFO26437.1	70	158.303	170	120	CK1 protein kinase
Locus_14555_Transcript_1/1_Conf_1.000	448	14	5.77E-42	NP_501544.1	79	173.711	121	96	hypothetical protein C27B7.2
Locus_14556_Transcript_1/1_Conf_1.000	258	0							
Locus_14557_Transcript_1/1_Conf_1.000	245	0							
Locus_14558_Transcript_1/1_Conf_1.000	247	2	1.19E-15	EFO24527.1	66	86.2705	80	53	hypothetical protein LOAG_03957
Locus_14559_Transcript_1/1_Conf_1.000	170	0							
Locus_1456_Transcript_1/1_Conf_1.000	302	1	1.48E-05	XP_002129449.1	53	52.7582	102	55	PREDICTED: hypothetical protein
Locus_14560_Transcript_1/1_Conf_1.000	640	20	3.23E-60	EFO27810.1	74	235.343	206	153	plasmid Maintenance protein containing protein
Locus_14561_Transcript_1/1_Conf_1.000	795	0							

Locus_14562_Transcript_1/1_Conf_1.000	1201	4	2.28E-88	XP_002635496.1	70	330.487	375	264	Hypothetical protein CBG08796
Locus_14563_Transcript_1/1_Conf_1.000	184	0							
Locus_14564_Transcript_1/1_Conf_1.000	552	20	5.32E-46	XP_002647377.1	93	187.578	133	124	Hypothetical protein CBG06437
Locus_14565_Transcript_1/1_Conf_1.000	423	20	8.97E-27	NP_502633.1	72	123.25	114	83	UDP-GlucuronosylTransferase family member (ugt-22)
Locus_14566_Transcript_1/2_Conf_1.000	228	0							
Locus_14566_Transcript_2/2_Conf_1.000	746	0							
Locus_14567_Transcript_1/1_Conf_1.000	303	0							
Locus_14568_Transcript_1/1_Conf_1.000	848	20	4.71E-30	3N9L	67	135.961	151	102	Cekdm7a From C.Elegans
Locus_14569_Transcript_1/1_Conf_1.000	340	0							
Locus_1457_Transcript_1/1_Conf_1.000	1198	20	7.36E-55	NP_741338.1	65	219.164	236	154	hypothetical protein Y69A2AR.7
Locus_14570_Transcript_1/1_Conf_1.000	138	6	2.41E-16	NP_872196.2	95	88.5817	46	44	hypothetical protein D1054.9
Locus_14571_Transcript_1/1_Conf_1.000	497	14	3.99E-19	NP_499088.1	72	97.8265	81	59	Neuropeptide-Like Protein family member (nlp-36)
Locus_14572_Transcript_1/1_Conf_1.000	380	0							
Locus_14573_Transcript_1/1_Conf_1.000	509	0							
Locus_14574_Transcript_1/2_Conf_1.000	619	20	1.51E-19	XP_001645325.1	47	100.138	204	97	hypothetical protein Kpol_1058p4

Locus_14574_Transcript_2/2_Conf_1.000	412	15	4.56E-07	XP_001645325.1	44	57.7658	134	60	hypothetical protein Kpol_1058p4
Locus_14575_Transcript_1/1_Conf_1.000	237	4	1.06E-11	EFO22550.1	67	73.1738	77	52	TATA binding protein associated factor
Locus_14576_Transcript_1/1_Conf_1.000	630	0							
Locus_14577_Transcript_1/1_Conf_1.000	654	20	1.57E-97	NP_498645.1	83	359.377	218	183	MUscle Positioning family member (mup-4)
Locus_14578_Transcript_1/1_Conf_1.000	178	8	3.84E-14	NP_504908.1	81	81.2629	59	48	hypothetical protein F18E3.7
Locus_14579_Transcript_1/1_Conf_1.000	1211	20	2.81E-94	XP_002640721.1	62	350.132	392	245	Hypothetical protein CBG19789
Locus_1458_Transcript_1/2_Conf_1.000	1009	20	3.08E-101	NP_492354.1	86	372.859	236	205	Proteasome Beta Subunit family member (pbs-7)
Locus_1458_Transcript_2/2_Conf_1.000	2245	20	3.79E-83	NP_741955.2	71	314.309	320	229	hypothetical protein H18N23.2

Locus_14580_Transcript_1/1_Conf_1.000	305	20	7.75E-31	XP_001891560.1	98	136.732	67	66	DNA-directed RNA polymerase II 7.6 kDa polypeptide
Locus_14581_Transcript_1/2_Conf_1.000	461	0							
Locus_14581_Transcript_2/2_Conf_1.000	460	0							
Locus_14582_Transcript_1/1_Conf_1.000	559	0							
Locus_14583_Transcript_1/1_Conf_1.000	429	20	4.12E-32	NP_495516.2	78	140.969	127	100	hypothetical protein F32A5.8
Locus_14584_Transcript_1/3_Conf_0.600	245	0							
Locus_14584_Transcript_2/3_Conf_0.400	376	0							
Locus_14584_Transcript_3/3_Conf_0.600	245	0							
Locus_14585_Transcript_1/1_Conf_1.000	198	0							
Locus_14586_Transcript_1/1_Conf_1.000	222	20	1.26E-28	NP_496968.1	94	129.413	74	70	hypothetical protein Y48B6A.12
Locus_14587_Transcript_1/1_Conf_1.000	448	20	1.12E-61	XP_002641528.1	84	239.195	149	126	Hypothetical protein CBG09825
Locus_14588_Transcript_1/1_Conf_1.000	277	20	2.11E-12	EFO21272.1	62	75.485	74	46	hypothetical protein LOAG_07212
Locus_14589_Transcript_1/1_Conf_1.000	195	0							
Locus_1459_Transcript_1/1_Conf_1.000	229	20	2.61E-34	XP_002925190.1	100	148.288	73	73	PREDICTED: 40S ribosomal protein S13-like
Locus_14590_Transcript_1/1_Conf_1.000	227	0							
Locus_14591_Transcript_1/2_Conf_1.000	233	0							
Locus_14591_Transcript_2/2_Conf_1.000	430	20	6.78E-43	NP_001021848.1	82	176.792	107	88	hypothetical protein ZC334.7
Locus_14592_Transcript_1/1_Conf_1.000	191	0							

Locus_14593_Transcript_1/1_Conf_1.000	254	20	2.74E-15	CAA84704.4	87	85.1149	57	50	C. elegans protein F43D9.3a, confirmed by transcript evidence
Locus_14594_Transcript_1/1_Conf_1.000	325	0							
Locus_14595_Transcript_1/1_Conf_1.000	379	20	1.46E-61	XP_002831613.1	100	238.81	114	114	PREDICTED: LOW QUALITY PROTEIN: ubiquitin-like modifier-activating enzyme 1-like
Locus_14596_Transcript_1/1_Conf_1.000	152	0							
Locus_14597_Transcript_1/1_Conf_1.000	154	0							
Locus_14598_Transcript_1/1_Conf_1.000	209	0							
Locus_14599_Transcript_1/1_Conf_1.000	1985	20	0	NP_001041007.1	71	662.529	655	468	Juxtamembrane domain-Associated Catenin family member (jac-1)
Locus_146_Transcript_1/1_Conf_1.000	2367	20	0	XP_002639688.1	93	1111.29	626	586	C. briggsae CBR-FASN-1 protein
Locus_1460_Transcript_1/1_Conf_1.000	294	4	8.71E-14	XP_002646111.1	72	80.1073	69	50	C. briggsae CBR-PAD-1 protein
Locus_14600_Transcript_1/2_Conf_1.000	657	6	2.00E-07	ZP_02160840.1	45	60.077	159	72	hypothetical protein KAOT1_18882
Locus_14600_Transcript_2/2_Conf_1.000	657	3	2.00E-07	ZP_02160840.1	45	60.077	159	72	hypothetical protein KAOT1_18882
Locus_14601_Transcript_1/1_Conf_1.000	128	0							
Locus_14602_Transcript_1/1_Conf_1.000	245	0							
Locus_14603_Transcript_1/1_Conf_1.000	450	20	2.76E-36	NP_494905.2	74	154.836	151	112	MeDiaTor family member (mdt-4)
Locus_14604_Transcript_1/1_Conf_1.000	245	4	2.04E-23	NP_741205.1	96	112.079	61	59	PDF (arthropod Pigment Dispersing Factor) homolog family member (pdf-1)
Locus_14605_Transcript_1/1_Conf_1.000	317	0							
Locus_14606_Transcript_1/1_Conf_1.000	280	20	4.22E-29	NP_501217.2	82	130.954	93	77	hypothetical protein R05G6.10
Locus_14607_Transcript_1/1_Conf_1.000	143	0							
Locus_14608_Transcript_1/1_Conf_1.000	250	0							

Locus_14609_Transcript_1/1_Conf_1.000	1321	2	6.15E-05	NP_495931.2	46	53.5286	213	98	hypothetical protein T24B8.7
Locus_1461_Transcript_1/2_Conf_1.000	909	20	5.33E-30	NP_498126.1	73	135.961	137	101	hypothetical protein ZC395.10
Locus_1461_Transcript_2/2_Conf_1.000	894	20	5.17E-30	NP_498126.1	73	135.961	137	101	hypothetical protein ZC395.10
Locus_14610_Transcript_1/1_Conf_1.000	442	20	2.81E-28	XP_002643824.1	69	128.257	123	86	Hypothetical protein CBG02040
Locus_14611_Transcript_1/1_Conf_1.000	312	20	6.10E-23	XP_001902677.1	76	110.538	97	74	5'-nucleotidase, cytosolic III
Locus_14612_Transcript_1/1_Conf_1.000	427	0							
Locus_14613_Transcript_1/1_Conf_1.000	136	0							
Locus_14614_Transcript_1/1_Conf_1.000	143	20	4.99E-22	BAG61003.1	100	107.457	47	47	unnamed protein product
Locus_14615_Transcript_1/1_Conf_1.000	383	2	4.42E-26	XP_002631382.1	69	120.939	126	87	Hypothetical protein CBG03224
Locus_14616_Transcript_1/2_Conf_1.000	210	9	1.12E-05	XP_002634231.1	74	53.1434	47	35	C. briggsae CBR-TTR-45 protein
Locus_14616_Transcript_2/2_Conf_1.000	216	19	9.13E-08	XP_002634231.1	76	60.077	47	36	C. briggsae CBR-TTR-45 protein
Locus_14617_Transcript_1/1_Conf_1.000	388	0							
Locus_14618_Transcript_1/1_Conf_1.000	177	0							
Locus_14619_Transcript_1/1_Conf_1.000	310	20	3.72E-09	CAF95080.1	61	64.6994	91	56	unnamed protein product
Locus_1462_Transcript_1/1_Conf_1.000	1438	8	1.77E-85	ADM53747.1	62	321.242	339	213	Zinc finger transcription factor family protein 12, isoform c
Locus_14620_Transcript_1/2_Conf_1.000	404	20	1.17E-18	XP_002591927.1	58	96.2857	134	78	hypothetical protein BRAFLDRAFT_265245
Locus_14620_Transcript_2/2_Conf_1.000	404	20	1.17E-18	XP_002591927.1	58	96.2857	134	78	hypothetical protein BRAFLDRAFT_265245

Locus_14621_Transcript_1/1_Conf_1.000	170	20	5.41E-16	ABE91946.1	88	87.4261	53	47	Ras GTPase; Sigma-54 factor, interaction region
Locus_14622_Transcript_1/1_Conf_1.000	804	0							
Locus_14623_Transcript_1/1_Conf_1.000	554	0							
Locus_14624_Transcript_1/1_Conf_1.000	167	0							
Locus_14625_Transcript_1/1_Conf_1.000	439	20	5.62E-74	NP_501665.1	97	280.026	144	141	hypothetical protein F59B8.2
Locus_14626_Transcript_1/1_Conf_1.000	149	5	1.11E-16	NP_491711.2	87	89.7373	49	43	hypothetical protein C34G6.1
Locus_14627_Transcript_1/1_Conf_1.000	977	20	2.32E-74	AAV41897.1	65	283.493	319	210	SAX-2
Locus_14628_Transcript_1/1_Conf_1.000	524	0							
Locus_14629_Transcript_1/1_Conf_1.000	585	20	1.60E-33	NP_496652.1	73	146.362	134	98	Temporarily Assigned Gene name family member (tag-281)
Locus_1463_Transcript_1/1_Conf_1.000	2295	20	0	NP_510527.1	79	679.093	535	423	hypothetical protein H03A11.1
Locus_14630_Transcript_1/1_Conf_1.000	294	20	1.25E-36	CAB57358.1	85	155.992	95	81	microsomal aminopeptidase
Locus_14631_Transcript_1/1_Conf_1.000	184	0							
Locus_14632_Transcript_1/1_Conf_1.000	282	6	3.97E-14	NP_495914.1	68	81.2629	74	51	hypothetical protein C18E9.8
Locus_14633_Transcript_1/1_Conf_1.000	418	0							
Locus_14634_Transcript_1/1_Conf_1.000	232	4	3.19E-24	XP_002630898.1	91	114.775	62	57	Hypothetical protein CBG02620
Locus_14635_Transcript_1/1_Conf_1.000	328	2	1.42E-32	NP_504605.1	77	142.51	109	84	hypothetical protein K12B6.4
Locus_14636_Transcript_1/1_Conf_1.000	181	0							
Locus_14637_Transcript_1/1_Conf_1.000	306	0							
Locus_14638_Transcript_1/1_Conf_1.000	671	20	1.49E-82	CAC10528.1	80	309.686	227	182	putative inositol 1,4,5-trisphosphate receptor

Locus_14639_Transcript_1/1_Conf_1.000	189	0							
Locus_1464_Transcript_2/4_Conf_0.667	3362	1	7.52E-09	NP_509922.2	35	68.1662	600	214	hypothetical protein F29G6.3
Locus_1464_Transcript_3/4_Conf_0.250	2174	0							
Locus_1464_Transcript_4/4_Conf_0.667	3317	1	9.05E-07	NP_509922.2	35	61.2326	579	208	hypothetical protein F29G6.3
Locus_14640_Transcript_1/1_Conf_1.000	164	0							
Locus_14641_Transcript_1/1_Conf_1.000	226	0							
Locus_14642_Transcript_1/1_Conf_1.000	255	0							
Locus_14643_Transcript_1/1_Conf_1.000	512	0							
Locus_14644_Transcript_1/1_Conf_1.000	196	20	2.45E-16	NP_494786.4	93	88.5817	63	59	TWiK family of potassium channels family member (twk-2)
Locus_14645_Transcript_1/1_Conf_1.000	246	1	3.61E-04	NP_509416.2	71	48.1358	42	30	hypothetical protein F55E10.7
Locus_14646_Transcript_1/1_Conf_1.000	428	20	5.18E-67	XP_002634991.1	99	256.914	125	124	Hypothetical protein CBG13531
Locus_14647_Transcript_1/1_Conf_1.000	576	0							
Locus_14648_Transcript_1/1_Conf_1.000	548	20	4.22E-24	XP_002642540.1	88	114.775	76	67	Hypothetical protein CBG20163
Locus_14649_Transcript_1/1_Conf_1.000	254	5	2.03E-10	NP_508729.2	66	68.9366	80	53	hypothetical protein C52B9.4
Locus_1465_Transcript_1/1_Conf_1.000	2135	20	2.59E-174	NP_499577.1	67	617.076	682	458	hypothetical protein Y75B8A.4
Locus_14650_Transcript_1/1_Conf_1.000	776	20	6.76E-54	XP_002640868.1	79	214.927	157	125	Hypothetical protein CBG15759
Locus_14651_Transcript_1/1_Conf_1.000	1026	20	1.30E-78	NP_492757.2	79	297.745	239	189	ToLLIp homolog family member (tli-1)

Locus_14652_Transcript_1/1_Conf_1.000	378	20	2.12E-36	BAG58382.1	100	155.221	74	74	unnamed protein product
Locus_14653_Transcript_1/1_Conf_1.000	153	0							
Locus_14654_Transcript_1/1_Conf_1.000	627	20	8.02E-85	ACO11472.1	83	317.005	201	167	Beta-ureidopropionase
Locus_14655_Transcript_1/1_Conf_1.000	140	3	8.57E-06	XP_002640263.1	67	53.5286	46	31	Hypothetical protein CBG12788
Locus_14656_Transcript_1/1_Conf_1.000	446	20	1.86E-56	XP_001894541.1	83	221.861	148	124	LIM domain containing protein
Locus_14657_Transcript_1/1_Conf_1.000	427	0							
Locus_14658_Transcript_1/1_Conf_1.000	366	6	5.89E-23	XP_002633327.1	86	110.538	90	78	C. briggsae CBR-DNJ-1 protein
Locus_14659_Transcript_1/1_Conf_1.000	156	0							
Locus_1466_Transcript_1/1_Conf_1.000	383	0							
Locus_14660_Transcript_1/1_Conf_1.000	349	0							
Locus_14661_Transcript_1/1_Conf_1.000	436	20	1.30E-62	CAL49442.3	91	242.276	144	132	C. elegans protein F36H1.2c, partially confirmed by transcript evidence
Locus_14662_Transcript_1/1_Conf_1.000	534	20	1.43E-42	EFO22680.1	77	176.022	126	98	hypothetical protein LOAG_05804
Locus_14663_Transcript_1/1_Conf_1.000	190	0							
Locus_14664_Transcript_1/1_Conf_1.000	152	0							
Locus_14665_Transcript_1/1_Conf_1.000	160	0							
Locus_14666_Transcript_1/1_Conf_1.000	503	20	2.87E-73	XP_001892755.1	89	277.715	167	149	TBC domain containing protein
Locus_14667_Transcript_1/1_Conf_1.000	623	20	1.87E-56	CAB66478.1	100	180.644	91	91	hypothetical protein
Locus_14668_Transcript_1/1_Conf_1.000	319	0							
Locus_14669_Transcript_1/1_Conf_1.000	372	0							

Locus_1467_Transcript_1/3_Conf_0.714	3136	20	9.44E-99	EFO28201.1	61	366.696	472	292	hypothetical protein LOAG_00283
Locus_1467_Transcript_2/3_Conf_0.714	3142	20	9.46E-99	EFO28201.1	61	366.696	472	292	hypothetical protein LOAG_00283
Locus_1467_Transcript_3/3_Conf_0.714	3142	20	9.46E-99	EFO28201.1	61	366.696	472	292	hypothetical protein LOAG_00283
Locus_14670_Transcript_1/1_Conf_1.000	466	20	1.80E-51	NP_001023266.1	81	205.297	156	127	UNCoordinated family member (unc-26)
Locus_14671_Transcript_1/2_Conf_1.000	435	20	9.64E-66	XP_002633333.1	92	252.677	145	134	Hypothetical protein CBG06072
Locus_14671_Transcript_2/2_Conf_1.000	435	20	9.64E-66	XP_002633333.1	92	252.677	145	134	Hypothetical protein CBG06072
Locus_14672_Transcript_1/1_Conf_1.000	223	0							
Locus_14673_Transcript_1/1_Conf_1.000	381	0							
Locus_14674_Transcript_1/1_Conf_1.000	216	20	1.27E-33	XP_002913180.1	100	145.976	72	72	PREDICTED: cyclin-dependent kinase 2-associated protein 1-like
Locus_14675_Transcript_1/1_Conf_1.000	300	20	2.19E-33	XP_002822876.1	100	145.206	71	71	PREDICTED: protein C10-like isoform 2
Locus_14676_Transcript_1/1_Conf_1.000	671	20	2.37E-88	CAA91463.2	86	328.946	223	192	C. elegans protein F42E11.1a, partially confirmed by transcript evidence
Locus_14677_Transcript_1/1_Conf_1.000	291	20	1.39E-27	NP_493472.1	75	125.946	92	69	hypothetical protein C37A5.1
Locus_14678_Transcript_1/2_Conf_1.000	430	20	3.50E-23	XP_002631584.1	74	111.309	108	80	Hypothetical protein CBG20762
Locus_14678_Transcript_2/2_Conf_1.000	430	20	2.96E-22	XP_002631584.1	73	108.227	108	79	Hypothetical protein CBG20762
Locus_14679_Transcript_1/1_Conf_1.000	268	0							
Locus_1468_Transcript_1/1_Conf_1.000	1016	0							
Locus_14680_Transcript_1/1_Conf_1.000	181	0							

Locus_14681_Transcript_1/1_Conf_1.000	316	20	8.77E-22	BAE75796.1	70	106.686	98	69	Slc26a6 A
Locus_14682_Transcript_1/1_Conf_1.000	304	0							
Locus_14683_Transcript_1/1_Conf_1.000	169	0							
Locus_14684_Transcript_1/1_Conf_1.000	612	20	1.70E-92	XP_002644330.1	90	342.428	203	183	C. briggsae CBR-SPC-1 protein
Locus_14685_Transcript_1/1_Conf_1.000	177	9	8.00E-12	EFO23504.1	71	73.559	59	42	TK protein kinase
Locus_14686_Transcript_1/1_Conf_1.000	487	20	1.38E-51	NP_502547.2	73	205.682	170	125	hypothetical protein JC8.5
Locus_14687_Transcript_1/1_Conf_1.000	724	20	8.06E-19	EFN63743.1	49	98.2117	220	109	RCC1 domain-containing protein 1
Locus_14688_Transcript_1/1_Conf_1.000	603	20	2.23E-41	NP_001023110.1	64	172.555	197	128	Homolog Of ELAC2 (cancer susceptibility locus) family member (hoe-1)
Locus_14689_Transcript_1/1_Conf_1.000	272	0							
Locus_1469_Transcript_1/1_Conf_1.000	498	0							
Locus_14690_Transcript_1/1_Conf_1.000	175	0							
Locus_14691_Transcript_1/1_Conf_1.000	132	0							

Locus_14692_Transcript_1/1_Conf_1.000	1236	20	6.00E-124	XP_002643227.1	74	448.743	423	315	C. briggsae CBR-SEL-12 protein
Locus_14693_Transcript_1/1_Conf_1.000	222	0							
Locus_14694_Transcript_1/1_Conf_1.000	206	0							
Locus_14695_Transcript_1/1_Conf_1.000	865								
Locus_14696_Transcript_1/1_Conf_1.000	197	0							
Locus_14697_Transcript_1/1_Conf_1.000	179	0							
Locus_14698_Transcript_1/1_Conf_1.000	423	4	9.97E-10	NP_493393.1	57	66.6254	135	78	hypothetical protein W09C5.7
Locus_14699_Transcript_1/1_Conf_1.000	602	20	4.11E-72	NP_001122494.1	84	274.633	179	152	TOMosyn synaptic protein family member (tom-1)
Locus_147_Transcript_1/1_Conf_1.000	1067	20	5.14E-110	XP_002642221.1	86	402.134	251	216	Hypothetical protein CBG18195
Locus_1470_Transcript_1/3_Conf_0.500	529	0							
Locus_1470_Transcript_2/3_Conf_0.500	516	0							
Locus_1470_Transcript_3/3_Conf_0.667	516	0							
Locus_14700_Transcript_1/1_Conf_1.000	471	2	3.74E-25	XP_002642486.1	61	117.857	127	78	Hypothetical protein CBG06905
Locus_14701_Transcript_1/1_Conf_1.000	283	3	1.04E-14	NP_001024665.1	86	83.1889	53	46	SynDaPiN (synaptic dynamin binding protein) homolog family member (sdpn-1)
Locus_14702_Transcript_1/1_Conf_1.000	144	0							
Locus_14703_Transcript_1/1_Conf_1.000	400	0							

Locus_14704_Transcript_1/1_Conf_1.000	881	20	2.61E-95	XP_002642921.1	83	352.829	296	248	Hypothetical protein CBG15197
Locus_14705_Transcript_1/1_Conf_1.000	1243	20	3.17E-165	NP_499162.2	83	585.874	405	338	hypothetical protein R10E11.3
Locus_14706_Transcript_1/1_Conf_1.000	1024	20	1.04E-11	CBH12501.1	45	75.485	341	156	kinesin K39, putative
Locus_14707_Transcript_1/4_Conf_0.250	239	0							
Locus_14707_Transcript_2/4_Conf_0.625	1388	20	1.72E-29	XP_001897896.1	45	135.191	397	181	RNA binding motif protein 21
Locus_14707_Transcript_3/4_Conf_0.625	1399	20	1.02E-29	XP_001897896.1	45	135.961	401	183	RNA binding motif protein 21

Locus_14707_Transcript_4/4_Conf_0.625	1399	20	1.02E-29	XP_001897896.1	45	135.961	401	183	RNA binding motif protein 21
Locus_14708_Transcript_1/1_Conf_1.000	467	0							
Locus_14709_Transcript_1/1_Conf_1.000	570	3	1.59E-11	NP_494749.2	56	73.1738	88	50	hypothetical protein C16A11.7
Locus_1471_Transcript_1/1_Conf_1.000	3092	20	1.17E-133	CAA78521.1	74	482.641	523	388	hypothetical polypeptide II
Locus_14710_Transcript_1/1_Conf_1.000	143	0							
Locus_14711_Transcript_1/1_Conf_1.000	197	0							
Locus_14712_Transcript_1/1_Conf_1.000	353	0							
Locus_14713_Transcript_1/1_Conf_1.000	756	20	1.37E-96	NP_504476.1	87	356.681	224	197	Cell-death-Related Nuclease family member (crn-2)
Locus_14714_Transcript_1/1_Conf_1.000	605	0							
Locus_14715_Transcript_1/1_Conf_1.000	804	20	3.06E-60	XP_002639513.1	61	236.113	265	164	Hypothetical protein CBG04119
Locus_14716_Transcript_1/1_Conf_1.000	413	0							
Locus_14717_Transcript_1/1_Conf_1.000	1313	20	0	NP_001023732.1	93	780.015	433	406	EGg Laying defective family member (egl-3)
Locus_14718_Transcript_1/1_Conf_1.000	173	0							
Locus_14719_Transcript_1/1_Conf_1.000	453	20	2.94E-38	XP_002640798.1	77	161.384	109	85	C. briggsae CBR-NHR-121 protein
Locus_1472_Transcript_1/1_Conf_1.000	384	0							
Locus_14720_Transcript_1/1_Conf_1.000	307	0							
Locus_14721_Transcript_1/1_Conf_1.000	575	20	7.65E-54	XP_002113404.1	73	213.772	160	117	hypothetical protein TRIADDRAFT_57530
Locus_14722_Transcript_1/1_Conf_1.000	254	0							
Locus_14723_Transcript_1/1_Conf_1.000	238	0							
Locus_14724_Transcript_1/1_Conf_1.000	610	20	3.81E-20	EFO20693.1	55	102.064	167	93	hypothetical protein LOAG_07794
Locus_14725_Transcript_1/1_Conf_1.000	263	0							

Locus_14726_Transcript_1/1_Conf_1.000	359	20	2.96E-22	NP_001041045.1	63	108.227	119	76	COenzyme Q (ubiquinone) biosynthesis family member (coq-3)
Locus_14727_Transcript_1/1_Conf_1.000	1012	20	1.28E-139	NP_494712.1	94	500.36	313	297	proteasome Regulatory Particle, Non-ATPase-like family member (rpn-11)
Locus_14728_Transcript_1/1_Conf_1.000	413	0							
Locus_14729_Transcript_1/1_Conf_1.000	137	20	2.85E-17	XP_001103566.2	100	91.6633	45	45	PREDICTED: fas-activated serine/threonine kinase
Locus_1473_Transcript_1/1_Conf_1.000	945	20	2.50E-126	XP_002633756.1	83	456.062	301	252	C. briggsae CBR-DHP-2 protein
Locus_14730_Transcript_1/1_Conf_1.000	147	0							
Locus_14731_Transcript_1/1_Conf_1.000	271	0							
Locus_14732_Transcript_1/1_Conf_1.000	333	13	6.81E-11	NP_491252.1	64	70.4774	62	40	AntiBacterial Factor related family member (abf-2)
Locus_14733_Transcript_1/1_Conf_1.000	418	20	3.30E-53	NP_493616.1	83	211.075	139	116	hypothetical protein F33H2.5
Locus_14734_Transcript_1/2_Conf_1.000	740	20	7.79E-65	NP_501092.1	73	251.136	228	167	Conserved Oligomeric Golgi (COG) Component family member (cogc-2)

Locus_14734_Transcript_2/2_Conf_1.000	740	20	7.79E-65	NP_501092.1	73	251.136	228	167	Conserved Oligomeric Golgi (COG) Component family member (cogc-2)
Locus_14735_Transcript_1/1_Conf_1.000	613	0							
Locus_14736_Transcript_1/1_Conf_1.000	311	20	4.67E-28	EFO27919.1	92	127.487	76	70	hypothetical protein LOAG_00556
Locus_14737_Transcript_1/1_Conf_1.000	425	20	2.28E-30	XP_002637013.1	65	135.191	146	96	C. briggsae CBR-NID-1 protein
Locus_14738_Transcript_1/1_Conf_1.000	142	20	4.23E-13	XP_002645201.1	91	77.7962	46	42	C. briggsae CBR-BCAT-1 protein
Locus_14739_Transcript_1/1_Conf_1.000	307	0							
Locus_1474_Transcript_1/2_Conf_1.000	1463	0							
Locus_1474_Transcript_2/2_Conf_1.000	1433	0							
Locus_14740_Transcript_1/1_Conf_1.000	814	20	4.47E-59	EFO26681.1	64	232.261	278	179	glycosyl transferase
Locus_14741_Transcript_1/1_Conf_1.000	448	20	8.91E-43	NP_001021685.1	94	176.407	100	94	ZO-1 (Zonula Occludens tight junctional protein) Ortholog family member (zoo-1)
Locus_14742_Transcript_1/1_Conf_1.000	544	20	1.09E-24	ADI24640.1	59	116.701	175	104	Hypothetical protein C56E6.9
Locus_14743_Transcript_1/1_Conf_1.000	219	20	5.89E-26	EFO13953.1	91	120.553	72	66	hypothetical protein LOAG_14573
Locus_14744_Transcript_1/1_Conf_1.000	402	0							
Locus_14745_Transcript_1/1_Conf_1.000	298	0							
Locus_14746_Transcript_1/1_Conf_1.000	234	0							
Locus_14747_Transcript_1/1_Conf_1.000	193	0							
Locus_14748_Transcript_1/1_Conf_1.000	182	0							
Locus_14749_Transcript_1/1_Conf_1.000	141	0							

Locus_1475_Transcript_1/1_Conf_1.000	826	20	9.88E-70	EFO26807.1	81	267.7	202	164	wnt-4 protein
Locus_14750_Transcript_1/1_Conf_1.000	261	0							
Locus_14751_Transcript_1/1_Conf_1.000	666	0							
Locus_14752_Transcript_1/1_Conf_1.000	281	2	8.57E-06	XP_002645909.1	64	53.5286	51	33	Hypothetical protein CBG07665
Locus_14753_Transcript_1/1_Conf_1.000	173	20	1.28E-25	ADM64582.1	100	119.398	56	56	ribosomal protein L27
Locus_14754_Transcript_1/1_Conf_1.000	177	0							
Locus_14755_Transcript_1/2_Conf_1.000	265	0							
Locus_14755_Transcript_2/2_Conf_1.000	265	0							
Locus_14756_Transcript_1/1_Conf_1.000	412	10	1.23E-28	NP_001123020.1	69	129.413	105	73	hypothetical protein T23B12.8
Locus_14757_Transcript_1/2_Conf_1.000	426	20	1.17E-18	EFO16664.1	53	96.2857	183	98	lenep protein
Locus_14757_Transcript_2/2_Conf_1.000	339	20	3.58E-20	XP_002629740.1	66	101.293	106	71	Hypothetical protein CBG00973
Locus_14758_Transcript_1/1_Conf_1.000	302	20	6.35E-41	NP_509597.1	91	170.244	100	91	GTPase Activating Protein family member (gap-2)

Locus_14759_Transcript_1/1_Conf_1.000	1202	20	4.81E-155	NP_495846.1	83	551.977	369	307	hypothetical protein C34C6.4
Locus_1476_Transcript_1/2_Conf_1.000	1330	0							
Locus_1476_Transcript_2/2_Conf_1.000	1330	0							
Locus_14760_Transcript_1/2_Conf_1.000	247	0							
Locus_14760_Transcript_2/2_Conf_1.000	360	0							
Locus_14761_Transcript_1/1_Conf_1.000	423	20	1.86E-40	NP_492533.2	74	168.703	136	101	IRoquois subclass of homeoboX family member (irx-1)
Locus_14762_Transcript_1/1_Conf_1.000	285	0							
Locus_14763_Transcript_1/1_Conf_1.000	651	20	5.29E-21	XP_002740067.1	58	105.145	140	82	PREDICTED: RETRotransposon-like family member (retr-1)-like, partial
Locus_14764_Transcript_1/1_Conf_1.000	356	20	7.04E-40	Q5K2C1.1	88	166.777	109	96	Alpha,alpha-trehalose-phosphate synthase UDP-forming 2
Locus_14765_Transcript_1/1_Conf_1.000	236	2	8.68E-14	XP_002647167.1	69	80.1073	78	54	Hypothetical protein CBG22317
Locus_14766_Transcript_1/2_Conf_1.000	517	4	1.29E-26	NP_506375.1	71	122.865	116	83	hypothetical protein F53F4.11
Locus_14766_Transcript_2/2_Conf_1.000	517	4	1.29E-26	NP_506375.1	71	122.865	116	83	hypothetical protein F53F4.11
Locus_14767_Transcript_1/1_Conf_1.000	677	0							
Locus_14768_Transcript_1/1_Conf_1.000	144	0							
Locus_14769_Transcript_1/1_Conf_1.000	590	20	9.54E-71	NP_001041018.1	80	270.011	196	158	hypothetical protein Y11D7A.3

Locus_1477_Transcript_1/3_Conf_0.714	3328	20	0	XP_002635268.1	69	780.015	789	547	Hypothetical protein CBG11512
Locus_1477_Transcript_2/3_Conf_0.714	3352	20	0	XP_002635268.1	70	778.474	790	556	Hypothetical protein CBG11512
Locus_1477_Transcript_3/3_Conf_0.714	3334	20	0	XP_002635268.1	69	781.556	790	548	Hypothetical protein CBG11512
Locus_14770_Transcript_1/1_Conf_1.000	536	20	2.21E-35	EFO24456.1	79	152.14	102	81	hypothetical protein LOAG_04032
Locus_14771_Transcript_1/1_Conf_1.000	228	11	1.03E-06	NP_001020992.1	76	56.6102	42	32	hypothetical protein C09D4.1

Locus_14772_Transcript_1/1_Conf_1.000	231	20	8.38E-33	NP_500265.2	93	143.28	76	71	hypothetical protein Y54G2A.17
Locus_14773_Transcript_1/1_Conf_1.000	214	0							
Locus_14774_Transcript_1/2_Conf_1.000	618	20	3.76E-95	NP_508927.2	85	351.288	218	187	DihydroPYrimidine Dehydrogenase family member (dpyd-1)
Locus_14774_Transcript_2/2_Conf_1.000	618	20	1.43E-94	NP_508927.2	85	349.362	218	187	DihydroPYrimidine Dehydrogenase family member (dpyd-1)
Locus_14775_Transcript_1/2_Conf_1.000	492	0							
Locus_14775_Transcript_2/2_Conf_1.000	479	0							
Locus_14776_Transcript_1/1_Conf_1.000	414	20	3.10E-56	XP_002642176.1	87	221.09	137	120	Hypothetical protein CBG18143
Locus_14777_Transcript_1/1_Conf_1.000	401	5	3.09E-19	NP_492332.1	64	98.2117	91	59	hypothetical protein F43G9.4
Locus_14778_Transcript_1/1_Conf_1.000	583	0							
Locus_14779_Transcript_1/1_Conf_1.000	527	0							
Locus_1478_Transcript_1/2_Conf_1.000	1381	20	1.25E-88	NP_001016570.1	66	331.643	322	213	aurora kinase A

Locus_1478_Transcript_2/2_Conf_1.000	1324	20	3.90E-92	NP_001081565.1	72	343.199	290	209	serine/threonine-protein kinase 6
Locus_14780_Transcript_1/1_Conf_1.000	927	2	6.57E-07	NP_502860.2	42	59.3066	185	78	hypothetical protein Y73F8A.25
Locus_14781_Transcript_1/1_Conf_1.000	493	0							
Locus_14782_Transcript_1/1_Conf_1.000	234	20	5.44E-16	AAK29933.3	71	87.4261	76	54	Rab connectin related protein 2, partially confirmed by transcript evidence
Locus_14783_Transcript_1/1_Conf_1.000	353	20	6.19E-36	XP_002646888.1	75	153.68	123	93	C. briggsae CBR-PTC-3 protein
Locus_14784_Transcript_1/1_Conf_1.000	250	20	1.40E-40	BAI45831.1	100	169.088	83	83	lipolysis stimulated lipoprotein receptor
Locus_14785_Transcript_1/1_Conf_1.000	186	0							
Locus_14786_Transcript_1/1_Conf_1.000	249	0							
Locus_14787_Transcript_1/1_Conf_1.000	169	0							
Locus_14788_Transcript_1/1_Conf_1.000	406	0							
Locus_14789_Transcript_1/1_Conf_1.000	658	0							
Locus_1479_Transcript_1/1_Conf_1.000	651	0							
Locus_14790_Transcript_1/1_Conf_1.000	357	20	6.98E-56	ACZ54691.1	96	219.935	118	114	Uncoordinated protein 82, isoform f
Locus_14791_Transcript_1/1_Conf_1.000	222	0							
Locus_14792_Transcript_1/1_Conf_1.000	640	19	4.40E-33	XP_002631813.1	54	145.206	208	114	Hypothetical protein CBG21034
Locus_14793_Transcript_1/1_Conf_1.000	1754	20	8.87E-138	NP_740853.2	79	495.352	435	347	TOMosyn synaptic protein family member (tom-1)
Locus_14794_Transcript_1/1_Conf_1.000	521	4	1.51E-30	XP_002631397.1	63	135.961	150	95	C. briggsae CBR-DEP-1 protein
Locus_14795_Transcript_1/1_Conf_1.000	222	0							
Locus_14796_Transcript_1/1_Conf_1.000	220	0							
Locus_14797_Transcript_1/1_Conf_1.000	496	0							

Locus_14798_Transcript_1/1_Conf_1.000	673	20	1.48E-37	XP_002629759.1	59	160.229	223	133	Hypothetical protein CBG00995
Locus_14799_Transcript_1/1_Conf_1.000	273	5	2.43E-08	XP_002634384.1	52	62.003	88	46	C. briggsae CBR-ELPC-4 protein
Locus_148_Transcript_1/4_Conf_0.545	557	0							
Locus_148_Transcript_2/4_Conf_0.545	553	0							
Locus_148_Transcript_3/4_Conf_0.545	359	0							
Locus_148_Transcript_4/4_Conf_0.545	563	0							
Locus_1480_Transcript_1/1_Conf_1.000	1606	20	0	NP_496376.1	94	885.174	474	447	NADH Ubiquinone Oxidoreductase family member (nuo-1)
Locus_14800_Transcript_1/1_Conf_1.000	258	20	1.94E-29	EFO25600.1	100	132.109	66	66	beige/BEACH domain-containing protein
Locus_14801_Transcript_1/1_Conf_1.000	1658	20	2.37E-84	NP_503398.1	62	317.775	452	281	hypothetical protein C39F7.5
Locus_14802_Transcript_1/2_Conf_1.000	1197	20	4.94E-168	AAV68383.1	85	595.119	391	335	antigen h11
Locus_14802_Transcript_2/2_Conf_1.000	1090	20	2.00E-157	CAB57358.1	86	559.681	355	307	microsomal aminopeptidase
Locus_14803_Transcript_1/1_Conf_1.000	1065	20	5.00E-89	NP_001022488.1	62	332.413	370	230	UNCoordinated family member (unc-52)
Locus_14804_Transcript_1/1_Conf_1.000	545	20	1.98E-34	EFO15307.1	72	149.058	133	97	hypothetical protein LOAG_13207
Locus_14805_Transcript_1/1_Conf_1.000	278	0							
Locus_14806_Transcript_1/1_Conf_1.000	205	0							
Locus_14807_Transcript_1/1_Conf_1.000	422	4	1.45E-08	NP_493601.2	54	62.7734	75	41	FERM domain (protein4.1-ezrin-radixin-moesin) family member (frm-1)
Locus_14808_Transcript_1/1_Conf_1.000	187	0							
Locus_14809_Transcript_1/2_Conf_1.000	180	0							
Locus_14809_Transcript_2/2_Conf_1.000	309	0							
Locus_1481_Transcript_1/1_Conf_1.000	400	7	5.06E-46	NP_501821.2	84	187.193	132	111	hypothetical protein T04B2.5
Locus_14810_Transcript_1/3_Conf_0.667	634	20	2.18E-61	CAA22108.2	75	239.195	213	161	C. elegans protein Y75B8A.24, partially confirmed by transcript evidence

Locus_14810_Transcript_2/3_Conf_0.500	962	20	9.12E-92	XP_002647048.1	75	341.273	325	244	Hypothetical protein CBG03566
Locus_14810_Transcript_3/3_Conf_0.667	1108	20	1.30E-111	XP_002647048.1	75	407.527	374	283	Hypothetical protein CBG03566
Locus_14811_Transcript_1/1_Conf_1.000	395	20	1.35E-38	NP_001023370.1	71	162.54	130	93	ALP/Enigma encoding family member (alp-1)
Locus_14812_Transcript_1/1_Conf_1.000	285	0							
Locus_14813_Transcript_1/1_Conf_1.000	185	2	3.37E-05	NP_497903.1	62	51.6026	59	37	anNEXin family member (nex-3)
Locus_14814_Transcript_1/1_Conf_1.000	574	0							
Locus_14815_Transcript_1/1_Conf_1.000	175	19	9.49E-13	XP_002636796.1	75	76.6406	57	43	C. briggsae CBR-RIL-2 protein
Locus_14816_Transcript_1/1_Conf_1.000	592	20	1.72E-43	EFN62425.1	80	179.489	125	101	Nuclear factor 1 A-type
Locus_14817_Transcript_1/1_Conf_1.000	741	0							
Locus_14818_Transcript_1/1_Conf_1.000	222	0							
Locus_14819_Transcript_1/1_Conf_1.000	206	4	6.41E-09	XP_001892631.1	83	63.929	65	54	hypothetical protein Bm1_05795
Locus_1482_Transcript_1/1_Conf_1.000	919	20	5.63E-19	EFO24485.1	76	99.3673	84	64	hypothetical protein LOAG_03999
Locus_14820_Transcript_1/1_Conf_1.000	616	20	1.13E-51	XP_002630011.1	79	206.838	179	142	Hypothetical protein CBG13376
Locus_14821_Transcript_1/1_Conf_1.000	269	20	1.33E-30	XP_002644858.1	81	135.961	86	70	Hypothetical protein CBG05031
Locus_14822_Transcript_1/1_Conf_1.000	144	0							
Locus_14823_Transcript_1/1_Conf_1.000	625	20	1.08E-41	EFO25741.1	75	173.711	150	113	hypothetical protein LOAG_02746
Locus_14824_Transcript_1/1_Conf_1.000	614	20	9.80E-32	NP_001041088.1	57	140.584	194	111	hypothetical protein C27A7.3

Locus_14825_Transcript_1/1_Conf_1.000	638	20	2.01E-46	NP_741492.1	64	189.504	214	139	hypothetical protein T12G3.8
Locus_14826_Transcript_1/1_Conf_1.000	454	20	2.31E-59	NP_498636.2	84	231.491	151	128	hypothetical protein T20B12.1
Locus_14827_Transcript_1/1_Conf_1.000	519	0							
Locus_14828_Transcript_1/1_Conf_1.000	357	20	1.08E-32	XP_002630470.1	73	142.895	116	85	Hypothetical protein CBG11204
Locus_14829_Transcript_1/1_Conf_1.000	193	0							
Locus_1483_Transcript_1/2_Conf_1.000	1709	20	7.27E-36	NP_001021928.1	60	156.762	264	160	Maternal Effect Lethal family member (mel-11)
Locus_1483_Transcript_2/2_Conf_1.000	1442	20	1.30E-19	NP_001021929.1	63	102.449	166	105	Maternal Effect Lethal family member (mel-11)
Locus_14830_Transcript_1/1_Conf_1.000	154	0							
Locus_14831_Transcript_1/1_Conf_1.000	154	0							
Locus_14832_Transcript_1/1_Conf_1.000	266	0							
Locus_14833_Transcript_1/1_Conf_1.000	720	20	1.11E-44	EFO25648.1	63	184.111	209	133	platelet-activating factor acetylhydrolase
Locus_14834_Transcript_1/1_Conf_1.000	471	20	3.75E-17	XP_001896547.1	64	91.2781	102	66	Transcription factor TFIIB repeat family protein
Locus_14835_Transcript_1/1_Conf_1.000	142	3	2.17E-09	XP_002631490.1	77	65.4698	45	35	C. briggsae CBR-EGG-3 protein
Locus_14836_Transcript_1/1_Conf_1.000	194	0							
Locus_14837_Transcript_1/1_Conf_1.000	193	0							
Locus_14838_Transcript_1/1_Conf_1.000	306	20	1.61E-20	NP_510072.1	69	102.449	99	69	hypothetical protein F42F12.4

Locus_14839_Transcript_1/2_Conf_1.000	605	20	2.35E-91	XP_002643185.1	97	338.576	168	164	Hypothetical protein CBG24154
Locus_14839_Transcript_2/2_Conf_1.000	501	20	3.17E-72	EFO25025.1	98	274.248	133	131	NADH-ubiquinone oxidoreductase 23 kDa subunit
Locus_1484_Transcript_1/1_Conf_1.000	1577	20	2.28E-89	NP_001022864.1	68	334.339	363	250	hypothetical protein Y42G9A.3
Locus_14840_Transcript_1/2_Conf_1.000	386	20	6.39E-09	XP_641859.1	53	63.929	128	69	hypothetical protein DDB_G0279091
Locus_14840_Transcript_2/2_Conf_1.000	212	0							
Locus_14841_Transcript_1/1_Conf_1.000	251	0							
Locus_14842_Transcript_1/1_Conf_1.000	366	13	3.31E-35	XP_849940.1	96	128.642	65	63	PREDICTED: similar to Small EDRK-rich factor 2 (4F5rel) isoform 2
Locus_14843_Transcript_1/1_Conf_1.000	310	0							
Locus_14844_Transcript_1/1_Conf_1.000	139	0							
Locus_14845_Transcript_1/1_Conf_1.000	399	20	2.79E-28	XP_002548749.1	83	128.257	101	84	ADP-ribosylation factor

Locus_14846_Transcript_1/2_Conf_1.000	478	20	7.46E-34	CAD56609.2	66	146.747	142	94	C. elegans protein Y39A1A.5, confirmed by transcript evidence
Locus_14846_Transcript_2/2_Conf_1.000	478	20	7.46E-34	CAD56609.2	66	146.747	142	94	C. elegans protein Y39A1A.5, confirmed by transcript evidence
Locus_14847_Transcript_1/1_Conf_1.000	532	0							
Locus_14848_Transcript_1/1_Conf_1.000	267	0							
Locus_14849_Transcript_1/1_Conf_1.000	134	0							
Locus_1485_Transcript_1/1_Conf_1.000	483	20	2.13E-52	NP_495985.1	84	208.379	152	129	Temporarily Assigned Gene name family member (tag-175)
Locus_14850_Transcript_1/1_Conf_1.000	555	20	2.41E-70	XP_002633243.1	95	268.47	141	135	Hypothetical protein CBG05964
Locus_14851_Transcript_1/1_Conf_1.000	154	0							
Locus_14852_Transcript_1/1_Conf_1.000	1020	20	2.27E-75	EFA02360.1	62	286.96	378	238	hypothetical protein TcasGA2_TC008035
Locus_14853_Transcript_1/1_Conf_1.000	353	20	1.38E-27	XP_002647028.1	76	125.946	115	88	C. briggsae CBR-EMB-8 protein
Locus_14854_Transcript_1/1_Conf_1.000	175	0							
Locus_14855_Transcript_1/1_Conf_1.000	207	5	4.14E-08	XP_002641493.1	86	61.2326	38	33	Hypothetical protein CBG09784

Locus_14856_Transcript_1/1_Conf_1.000	615	20	4.91E-47	XP_001899238.1	60	191.43	208	126	Low-density lipoprotein receptor repeat class B containing protein
Locus_14857_Transcript_1/1_Conf_1.000	296	0							
Locus_14858_Transcript_1/1_Conf_1.000	317	2	5.17E-06	XP_002642239.1	57	54.299	71	41	Hypothetical protein CBG18221
Locus_14859_Transcript_1/1_Conf_1.000	232	0							
Locus_1486_Transcript_1/3_Conf_0.667	1060	20	6.71E-78	EFO24879.1	67	295.434	325	218	hypothetical protein LOAG_03607
Locus_1486_Transcript_2/3_Conf_0.667	1134	20	9.79E-78	EFO24879.1	67	295.049	325	218	hypothetical protein LOAG_03607
Locus_1486_Transcript_3/3_Conf_0.667	1060	20	8.77E-78	EFO24879.1	67	295.049	325	218	hypothetical protein LOAG_03607
Locus_14860_Transcript_1/1_Conf_1.000	1048	20	1.52E-82	ACF74367.1	66	310.842	364	243	Hypothetical protein R13F6.4d
Locus_14861_Transcript_1/1_Conf_1.000	163	0							
Locus_14862_Transcript_1/1_Conf_1.000	254	11	2.17E-20	NP_001122684.1	76	102.064	76	58	UNCoordinated family member (unc-103)
Locus_14863_Transcript_1/1_Conf_1.000	154	0							
Locus_14864_Transcript_1/1_Conf_1.000	230	20	4.17E-32	XP_002638742.1	94	140.969	76	72	C. briggsae CBR-PDE-6 protein
Locus_14865_Transcript_1/1_Conf_1.000	132	0							
Locus_14866_Transcript_1/1_Conf_1.000	517	20	1.48E-30	NP_510020.2	69	135.961	141	98	hypothetical protein W03G11.3
Locus_14867_Transcript_1/1_Conf_1.000	179	0							
Locus_14868_Transcript_1/1_Conf_1.000	257	4	8.80E-14	EFO27378.1	76	80.1073	64	49	hypothetical protein LOAG_01103
Locus_14869_Transcript_1/1_Conf_1.000	140	0							
Locus_1487_Transcript_1/1_Conf_1.000	227	0							

Locus_14870_Transcript_1/1_Conf_1.000	140	16	1.37E-11	AAP36667.1	100	72.7886	36	36	Homo sapiens vesicle-associated membrane protein 8 (endobrevin)
Locus_14871_Transcript_1/1_Conf_1.000	665	0							
Locus_14872_Transcript_1/1_Conf_1.000	288	0							
Locus_14873_Transcript_1/1_Conf_1.000	144	1	2.57E-21	EAX03766.1	100	105.145	47	47	high mobility group AT-hook 1, isoform CRA_e
Locus_14874_Transcript_1/1_Conf_1.000	754	20	7.31E-42	CAA90662.2	62	174.866	205	129	C. elegans protein R166.1, partially confirmed by transcript evidence
Locus_14875_Transcript_1/1_Conf_1.000	169	3	1.58E-07	XP_002642375.1	70	59.3066	50	35	Hypothetical protein CBG18379
Locus_14876_Transcript_1/1_Conf_1.000	228	0							
Locus_14877_Transcript_1/1_Conf_1.000	147	0							
Locus_14878_Transcript_1/1_Conf_1.000	280	3	2.40E-16	NP_740874.1	81	88.5817	55	45	hypothetical protein F57B10.14
Locus_14879_Transcript_1/1_Conf_1.000	413	20	1.72E-30	NP_503523.2	73	135.576	126	93	hypothetical protein Y32G9A.8
Locus_1488_Transcript_1/1_Conf_1.000	1213	20	4.78E-118	NP_492517.2	86	429.098	322	279	STomatin-Like family member (stl-1)

Locus_14880_Transcript_1/1_Conf_1.000	212	20	1.73E-14	EFO26533.1	81	82.4185	58	47	hypothetical protein LOAG_01953
Locus_14881_Transcript_1/1_Conf_1.000	274	2	3.74E-09	NP_492101.1	61	64.6994	89	55	hypothetical protein T28F4.4
Locus_14882_Transcript_1/1_Conf_1.000	189	2	2.39E-19	NP_503543.1	85	98.5969	63	54	hypothetical protein T08B1.1
Locus_14883_Transcript_1/1_Conf_1.000	139	0							
Locus_14884_Transcript_1/1_Conf_1.000	359	0							
Locus_14885_Transcript_1/2_Conf_1.000	641	20	1.88E-84	EFO20001.1	84	315.849	213	181	Sel1l protein
Locus_14885_Transcript_2/2_Conf_1.000	641	20	1.88E-84	EFO20001.1	84	315.849	213	181	Sel1l protein
Locus_14886_Transcript_1/1_Conf_1.000	235	0							
Locus_14887_Transcript_1/1_Conf_1.000	256	20	7.41E-29	XP_002810117.1	100	130.183	73	73	PREDICTED: keratinocyte-associated protein 2-like isoform 1
Locus_14888_Transcript_1/1_Conf_1.000	134	0							
Locus_14889_Transcript_1/1_Conf_1.000	328	2	6.86E-11	NP_509148.2	86	70.4774	45	39	hypothetical protein F41B4.1
Locus_1489_Transcript_1/1_Conf_1.000	552	20	3.32E-56	EFO21483.1	81	221.476	155	126	inner mitochondrial membrane protease family member
Locus_14890_Transcript_1/1_Conf_1.000	295	0							
Locus_14891_Transcript_1/1_Conf_1.000	138	0							
Locus_14892_Transcript_1/1_Conf_1.000	243	20	3.36E-26	EFO16124.1	84	121.324	79	67	protein-tyrosine phosphatase
Locus_14893_Transcript_1/1_Conf_1.000	466								
Locus_14894_Transcript_1/1_Conf_1.000	745	20	7.59E-100	ACY40006.1	86	367.466	250	217	K+/cl- cotransporter protein 2, isoform a
Locus_14895_Transcript_1/1_Conf_1.000	197	0							

Locus_14896_Transcript_1/1_Conf_1.000	236	2	4.31E-13	NP_501715.1	74	77.7962	77	57	Acetylcholine-gated Chloride Channel family member (acc-1)
Locus_14897_Transcript_1/1_Conf_1.000	222	2	5.90E-10	NP_508526.2	81	67.3958	43	35	TWiK family of potassium channels family member (twk-16)
Locus_14898_Transcript_1/1_Conf_1.000	384	20	3.84E-38	CAA88959.2	75	160.999	119	90	C. elegans protein M03C11.2, partially confirmed by transcript evidence
Locus_14899_Transcript_1/2_Conf_1.000	584	0							
Locus_14899_Transcript_2/2_Conf_1.000	432	0							
Locus_149_Transcript_1/1_Conf_1.000	747	20	3.16E-45	XP_001896912.1	70	186.037	189	134	hypothetical protein
Locus_1490_Transcript_1/2_Conf_1.000	2340	7	2.01E-34	NP_001122680.1	57	152.525	190	109	hypothetical protein C29E4.13
Locus_1490_Transcript_2/2_Conf_1.000	2340	7	2.01E-34	NP_001122680.1	57	152.525	190	109	hypothetical protein C29E4.13
Locus_14900_Transcript_1/1_Conf_1.000	422	0							
Locus_14901_Transcript_1/1_Conf_1.000	205	4	1.74E-06	XP_001894167.1	75	55.8398	48	36	Rad9 family protein
Locus_14902_Transcript_1/1_Conf_1.000	176	0							
Locus_14903_Transcript_1/1_Conf_1.000	321	0							
Locus_14904_Transcript_1/1_Conf_1.000	185	0							
Locus_14905_Transcript_1/2_Conf_1.000	246	0							
Locus_14905_Transcript_2/2_Conf_1.000	255	0							
Locus_14906_Transcript_1/1_Conf_1.000	403	0							
Locus_14907_Transcript_1/1_Conf_1.000	144	20	2.49E-16	NP_499865.1	93	88.5817	48	45	hypothetical protein F29C4.6
Locus_14908_Transcript_1/1_Conf_1.000	507	0							
Locus_14909_Transcript_1/1_Conf_1.000	262	20	1.48E-29	NP_496100.1	87	132.494	87	76	hypothetical protein Y53C12B.1

Locus_1491_Transcript_1/2_Conf_1.000	286	20	6.74E-14	NP_501767.1	77	80.4925	66	51	Sperm-Specific family, class P family member (ssp-10)
Locus_1491_Transcript_2/2_Conf_1.000	284	20	6.76E-14	NP_501767.1	77	80.4925	66	51	Sperm-Specific family, class P family member (ssp-10)
Locus_14910_Transcript_1/1_Conf_1.000	227	0							
Locus_14911_Transcript_1/1_Conf_1.000	405	0							
Locus_14912_Transcript_1/1_Conf_1.000	230	0							
Locus_14913_Transcript_1/1_Conf_1.000	136	0							
Locus_14914_Transcript_1/1_Conf_1.000	330	20	2.04E-31	NP_001022715.1	87	138.658	109	95	ALIX (Apoptosis-linked gene 2 interacting protein X) homolog family member (alx-1)
Locus_14915_Transcript_1/1_Conf_1.000	349	0							
Locus_14916_Transcript_1/1_Conf_1.000	827	3	2.67E-91	CAR63525.1	78	339.347	265	207	hypothetical protein
Locus_14917_Transcript_1/1_Conf_1.000	514	0							
Locus_14918_Transcript_1/1_Conf_1.000	518	0							
Locus_14919_Transcript_1/1_Conf_1.000	289	20	4.33E-29	XP_002920245.1	100	130.954	61	61	PREDICTED: STIP1 homology and U box-containing protein 1-like
Locus_1492_Transcript_1/1_Conf_1.000	657	20	9.15E-53	NP_001040786.1	66	210.69	218	144	RNase H family member (rnh-1.0)

Locus_14920_Transcript_1/1_Conf_1.000	168	9	7.55E-18	ACI49073.1	92	93.5893	52	48	hypothetical protein Cbre_JD10.005
Locus_14921_Transcript_1/1_Conf_1.000	293	1	4.35E-05	NP_498089.2	48	51.2174	110	53	hypothetical protein Y54H5A.2
Locus_14922_Transcript_1/1_Conf_1.000	469	20	7.09E-24	NP_496299.1	64	113.62	117	75	beta-LACTamase domain containing family member (lact-3)
Locus_14923_Transcript_1/1_Conf_1.000	618	20	3.78E-71	EFO14607.1	78	271.552	205	160	TRE-2 protein
Locus_14924_Transcript_1/1_Conf_1.000	788	20	1.70E-84	EFO16211.1	73	316.62	261	193	oxidoreductase
Locus_14925_Transcript_1/1_Conf_1.000	680	2	3.62E-07	NP_502401.1	50	59.3066	136	69	HSN Abnormal Migration family member (ham-1)
Locus_14926_Transcript_1/1_Conf_1.000	362	20	1.78E-43	XP_002631054.1	89	178.718	107	96	C. briggsae CBR-BTF-1 protein
Locus_14927_Transcript_1/1_Conf_1.000	299	2	4.60E-15	NP_498014.2	64	84.3445	90	58	COenzyme Q (ubiquinone) biosynthesis family member (coq-8)
Locus_14928_Transcript_1/1_Conf_1.000	595	20	1.13E-42	XP_002647701.1	65	176.792	201	131	Hypothetical protein CBG17890
Locus_14929_Transcript_1/2_Conf_1.000	323	0							
Locus_14929_Transcript_2/2_Conf_1.000	323	0							
Locus_1493_Transcript_1/1_Conf_1.000	1584	20	2.64E-77	XP_002647056.1	55	294.278	454	250	Hypothetical protein CBG03577
Locus_14930_Transcript_1/1_Conf_1.000	144	20	7.74E-18	XP_002815470.1	100	93.5893	47	47	PREDICTED: t-complex protein 1 subunit epsilon-like
Locus_14931_Transcript_1/1_Conf_1.000	662	20	2.08E-81	CAD44515.1	84	305.834	220	186	VAB-10A protein
Locus_14932_Transcript_1/1_Conf_1.000	217	0							
Locus_14933_Transcript_1/1_Conf_1.000	437	5	8.28E-17	XP_002642061.1	65	90.1225	137	90	Hypothetical protein CBG17999
Locus_14934_Transcript_1/1_Conf_1.000	142	0							
Locus_14935_Transcript_1/1_Conf_1.000	303	0							
Locus_14936_Transcript_1/1_Conf_1.000	354	11	2.00E-10	EFO17518.1	56	68.9366	96	54	hypothetical protein LOAG_10981
Locus_14937_Transcript_1/1_Conf_1.000	215	1	1.62E-04	EFO24319.1	67	49.2914	49	33	hypothetical protein LOAG_04165

Locus_14938_Transcript_1/1_Conf_1.000	443	20	7.17E-16	EFO24199.1	57	87.0409	108	62	hypothetical protein LOAG_04289
Locus_14939_Transcript_1/1_Conf_1.000	322	2	3.00E-14	NP_491418.1	68	81.6481	74	51	hypothetical protein B0041.5
Locus_1494_Transcript_1/4_Conf_0.444	946	20	2.32E-31	XP_001896932.1	80	140.584	105	85	PDZ domain containing protein
Locus_1494_Transcript_2/4_Conf_0.444	1817	20	0	CAP24558.1	89	716.457	419	376	C. briggsae CBR-LIN-53 protein
Locus_1494_Transcript_3/4_Conf_0.333	1078	20	1.01E-31	EFO21602.1	79	132.494	107	85	hypothetical protein LOAG_06884

Locus_1494_Transcript_4/4_Conf_0.444	1817	20	0	CAP24558.1	89	716.457	419	376	C. briggsae CBR-LIN-53 protein
Locus_14940_Transcript_1/1_Conf_1.000	462	0							
Locus_14941_Transcript_1/1_Conf_1.000	277	0							
Locus_14942_Transcript_1/1_Conf_1.000	1104	7	6.14E-45	NP_493565.1	58	186.037	284	167	hypothetical protein K05C4.7
Locus_14943_Transcript_1/1_Conf_1.000	312	5	1.12E-08	NP_506662.1	54	63.1586	98	53	hypothetical protein ZC412.3
Locus_14944_Transcript_1/1_Conf_1.000	710	20	9.22E-81	NP_492436.1	78	303.908	241	189	hypothetical protein W06D4.4
Locus_14945_Transcript_1/1_Conf_1.000	566	20	2.99E-15	XP_002633158.1	86	85.5001	46	40	C. briggsae CBR-UNC-82 protein
Locus_14946_Transcript_1/1_Conf_1.000	297	4	5.99E-23	NP_741662.1	68	110.538	99	68	TRNA Guanine Transglycosylase family member (tgt-2)
Locus_14947_Transcript_1/1_Conf_1.000	170	0							
Locus_14948_Transcript_1/1_Conf_1.000	767	20	3.05E-67	NP_001021875.1	68	259.225	257	177	Leucyl tRNA Synthetase family member (lrs-2)

Locus_14949_Transcript_1/1_Conf_1.000	727	20	5.12E-05	XP_001950365.1	45	52.373	139	63	PREDICTED: similar to IRA1 protein
Locus_1495_Transcript_1/1_Conf_1.000	293	6	1.18E-18	CAD44516.1	74	96.2857	81	60	VAB-10B protein
Locus_14950_Transcript_1/1_Conf_1.000	612	20	9.71E-64	NP_001021995.1	77	246.899	181	140	hypothetical protein C27D6.11
Locus_14951_Transcript_1/1_Conf_1.000	679	20	2.11E-39	NP_495410.4	58	166.392	234	138	hypothetical protein C44B7.11
Locus_14952_Transcript_1/1_Conf_1.000	545	20	5.98E-23	XP_002641267.1	62	110.923	141	88	Hypothetical protein CBG05179
Locus_14953_Transcript_1/1_Conf_1.000	289	5	1.95E-21	NP_498783.1	72	105.531	91	66	hypothetical protein C50C3.7
Locus_14954_Transcript_1/1_Conf_1.000	188	20	6.29E-28	XP_002923753.1	100	127.102	62	62	PREDICTED: rRNA 2'-O-methyltransferase fibrillarin-like
Locus_14955_Transcript_1/1_Conf_1.000	195	16	6.90E-19	NP_500647.2	82	97.0561	64	53	Dipeptidyl Peptidase Four (IV) family member (dpf-5)
Locus_14956_Transcript_1/1_Conf_1.000	482	2	9.46E-05	AAO63578.1	37	50.0618	141	53	secreted protein 6 precursor

Locus_14957_Transcript_1/1_Conf_1.000	254	6	2.24E-25	NP_492295.1	88	118.627	75	66	hypothetical protein F10D11.6
Locus_14958_Transcript_1/1_Conf_1.000	508	20	1.71E-76	ADD13547.1	90	288.5	169	153	Inositol triphosphate receptor protein 1, isoform h
Locus_14959_Transcript_1/1_Conf_1.000	450	20	1.91E-53	XP_002638883.1	78	211.846	144	113	Hypothetical protein CBG22103
Locus_1496_Transcript_1/1_Conf_1.000	295	0							
Locus_14960_Transcript_1/1_Conf_1.000	339	2	3.59E-04	NP_001040692.2	50	48.1358	89	45	hypothetical protein T28B8.3
Locus_14961_Transcript_1/1_Conf_1.000	307	0							
Locus_14962_Transcript_1/1_Conf_1.000	423	0							
Locus_14963_Transcript_1/1_Conf_1.000	213	0							
Locus_14964_Transcript_1/1_Conf_1.000	161	0							
Locus_14965_Transcript_1/1_Conf_1.000	304	5	6.17E-12	CAB55048.3	56	73.9442	88	50	C. elegans protein Y50E8A.5, partially confirmed by transcript evidence
Locus_14966_Transcript_1/1_Conf_1.000	689	3	6.17E-10	NP_491340.2	49	68.5514	191	94	histone METhyltransferase-like family member (met-1)
Locus_14967_Transcript_1/1_Conf_1.000	148	0							
Locus_14968_Transcript_1/1_Conf_1.000	382	0							
Locus_14969_Transcript_1/1_Conf_1.000	324	5	2.34E-51	NP_740906.1	93	204.912	108	101	hypothetical protein F26E4.7
Locus_1497_Transcript_1/1_Conf_1.000	230	20	1.35E-06	XP_002643187.1	78	56.225	41	32	Hypothetical protein CBG24156
Locus_14970_Transcript_1/1_Conf_1.000	174	0							

Locus_14971_Transcript_1/1_Conf_1.000	242	20	1.32E-38	ADG03678.1	100	162.54	80	80	alpha actinin 4 short isoform
Locus_14972_Transcript_1/1_Conf_1.000	619	4	3.56E-53	NP_001033357.2	67	211.846	206	140	DAF-16/FOXO Controlled, germline Tumor affecting family member (dct-6)
Locus_14973_Transcript_1/1_Conf_1.000	143	20	9.41E-21	XP_002642408.1	100	103.219	47	47	C. briggsae CBR-NHL-1 protein
Locus_14974_Transcript_1/1_Conf_1.000	456	0							
Locus_14975_Transcript_1/1_Conf_1.000	869	20	2.14E-33	EFO16257.1	68	147.132	150	102	hypothetical protein LOAG_12250
Locus_14976_Transcript_1/1_Conf_1.000	158	5	1.82E-19	NP_001022619.1	88	98.9821	52	46	NorDiHydroGuaiaretic acid resistant family member (ndg-4)
Locus_14977_Transcript_1/1_Conf_1.000	761	0							
Locus_14978_Transcript_1/1_Conf_1.000	326	0							
Locus_14979_Transcript_1/1_Conf_1.000	400	0							
Locus_1498_Transcript_1/2_Conf_1.000	472	20	4.44E-10	P34473.3	59	67.781	89	53	Uncharacterized protein F58A4.6
Locus_1498_Transcript_2/2_Conf_1.000	472	20	3.40E-10	P34473.3	59	68.1662	89	53	Uncharacterized protein F58A4.6
Locus_14980_Transcript_1/1_Conf_1.000	398	2	4.21E-08	NP_001122443.1	91	61.2326	36	33	hypothetical protein F02E9.5
Locus_14981_Transcript_1/1_Conf_1.000	223	20	2.47E-16	XP_001902364.1	79	88.5817	74	59	Myotubularin-related family protein
Locus_14982_Transcript_1/1_Conf_1.000	419	0							
Locus_14983_Transcript_1/1_Conf_1.000	348	20	2.21E-33	CAR63547.1	72	145.206	118	86	putative COLlagen

Locus_14984_Transcript_1/1_Conf_1.000	310	20	2.17E-25	NP_001020988.1	83	118.627	103	86	UNCoordinated family member (unc-89)
Locus_14985_Transcript_1/1_Conf_1.000	236	0							
Locus_14986_Transcript_1/1_Conf_1.000	293	0							
Locus_14987_Transcript_1/1_Conf_1.000	241	0							
Locus_14988_Transcript_1/1_Conf_1.000	391	20	1.39E-48	XP_002632959.1	85	195.667	128	109	C. briggsae CBR-RAD-26 protein
Locus_14989_Transcript_1/1_Conf_1.000	314	20	3.02E-30	NP_499492.1	78	134.806	102	80	hypothetical protein Y66D12A.10
Locus_1499_Transcript_1/1_Conf_1.000	1283	20	7.71E-106	EFO24882.1	68	388.652	435	300	hypothetical protein LOAG_03599
Locus_14990_Transcript_1/1_Conf_1.000	163	0							
Locus_14991_Transcript_1/1_Conf_1.000	437	0							
Locus_14992_Transcript_1/1_Conf_1.000	251	20	6.10E-20	XP_002644341.1	71	100.523	85	61	Hypothetical protein CBG14163
Locus_14993_Transcript_1/1_Conf_1.000	374	20	2.53E-29	XP_002641856.1	69	131.724	116	81	Hypothetical protein CBG16531
Locus_14994_Transcript_1/1_Conf_1.000	475	0							
Locus_14995_Transcript_1/1_Conf_1.000	398	0							
Locus_14996_Transcript_1/1_Conf_1.000	144	0							
Locus_14997_Transcript_1/1_Conf_1.000	605	20	3.35E-21	XP_001950752.1	57	105.531	207	119	PREDICTED: similar to galactokinase 1
Locus_14998_Transcript_1/1_Conf_1.000	226	0							
Locus_14999_Transcript_1/1_Conf_1.000	380	20	9.55E-29	XP_002639263.1	83	129.798	84	70	C. briggsae CBR-MOM-5 protein
Locus_15_Transcript_1/3_Conf_0.429	424	0							
Locus_15_Transcript_2/3_Conf_0.429	598	0							
Locus_15_Transcript_3/3_Conf_0.500	349	0							
Locus_150_Transcript_1/6_Conf_0.500	1513	20	0	Q9N358.3	88	641.728	414	368	T-complex protein 1 subunitTheta
Locus_150_Transcript_2/6_Conf_0.571	1550	20	0	Q9N358.3	91	805.053	503	461	T-complex protein 1 subunitTheta

Locus_150_Transcript_3/6_Conf_0.571	1550	20	0	Q9N358.3	91	803.512	503	461	T-complex protein 1 subunitTheta
Locus_150_Transcript_4/6_Conf_0.571	1550	20	0	Q9N358.3	91	801.971	503	461	T-complex protein 1 subunitTheta
Locus_150_Transcript_5/6_Conf_0.143	2282	20	3.04E-104	XP_001900116.1	54	384.415	763	415	SMC proteins Flexible Hinge Domain containing protein
Locus_150_Transcript_6/6_Conf_0.571	1550	20	0	Q9N358.3	91	803.512	503	461	T-complex protein 1 subunitTheta
Locus_1500_Transcript_1/1_Conf_1.000	1364	20	0	NP_508841.1	99	758.444	376	375	ACTin family member (act-4)
Locus_15000_Transcript_1/1_Conf_1.000	136	0							
Locus_15001_Transcript_1/1_Conf_1.000	307	0							
Locus_15002_Transcript_1/1_Conf_1.000	180	0							
Locus_15003_Transcript_1/1_Conf_1.000	305	7	1.57E-15	XP_001894313.1	63	85.8853	86	55	NLI interacting factor-like phosphatase family protein
Locus_15004_Transcript_1/1_Conf_1.000	235	0							
Locus_15005_Transcript_1/1_Conf_1.000	571	0							
Locus_15006_Transcript_1/1_Conf_1.000	222	0							
Locus_15007_Transcript_1/1_Conf_1.000	267	0							
Locus_15008_Transcript_1/1_Conf_1.000	184	1	3.60E-07	EFO17857.1	67	58.151	61	41	CK1/TTBK protein kinase

Locus_15009_Transcript_1/1_Conf_1.000	1054	20	3.08E-91	XP_002640663.1	68	339.732	332	228	C. briggsae CBR-DOG-1 protein
Locus_1501_Transcript_1/3_Conf_0.667	1640	20	1.59E-16	ACX37244.1	40	92.4337	404	164	eIF4G
Locus_1501_Transcript_2/3_Conf_0.667	1634	20	2.07E-16	ACX37244.1	40	92.0485	402	164	eIF4G
Locus_1501_Transcript_3/3_Conf_0.333	366	0							
Locus_15010_Transcript_1/1_Conf_1.000	331	20	6.74E-59	BAF84240.1	100	229.95	109	109	unnamed protein product
Locus_15011_Transcript_1/1_Conf_1.000	345	2	8.18E-20	XP_001897339.1	65	100.138	101	66	Negative elongation factor A homolog
Locus_15012_Transcript_1/1_Conf_1.000	315	0							
Locus_15013_Transcript_1/1_Conf_1.000	479	20	1.51E-18	EFO24760.1	82	95.9005	76	63	hypothetical protein LOAG_03731
Locus_15014_Transcript_1/1_Conf_1.000	900	16	8.69E-09	XP_001921732.1	45	65.4698	170	77	PREDICTED: zonadhesin-like
Locus_15015_Transcript_1/1_Conf_1.000	239	0							
Locus_15016_Transcript_1/1_Conf_1.000	409	20	3.38E-18	XP_001362642.1	46	94.7449	122	57	PREDICTED: similar to OTTHUMP00000028801
Locus_15017_Transcript_1/1_Conf_1.000	406	6	2.20E-09	EFO22525.1	58	65.4698	77	45	hypothetical protein LOAG_05962
Locus_15018_Transcript_1/1_Conf_1.000	979	20	3.96E-90	NP_001076724.1	70	335.88	327	230	Na-K-Cl Cotransporter homolog family member (nkcc-1)
Locus_15019_Transcript_1/1_Conf_1.000	489	20	9.46E-61	XP_002637665.1	90	236.113	151	137	C. briggsae CBR-APT-10 protein

Locus_1502_Transcript_1/1_Conf_1.000	576	20	2.00E-25	XP_001901168.1	78	119.398	111	87	Groucho/TLE N-terminal Q-rich domain containing protein
Locus_15020_Transcript_1/1_Conf_1.000	615	9	9.37E-06	AAD13340.1	42	54.299	187	79	ancylostoma secreted protein 1 precursor
Locus_15021_Transcript_1/1_Conf_1.000	778	1	2.44E-27	NP_497506.3	55	126.716	246	137	hypothetical protein Y48G9A.3
Locus_15022_Transcript_1/1_Conf_1.000	391	0							
Locus_15023_Transcript_1/1_Conf_1.000	889	20	2.31E-115	NP_741249.1	82	419.468	297	245	hypothetical protein C02F5.7
Locus_15024_Transcript_1/1_Conf_1.000	252	20	1.36E-14	XP_002643359.1	57	82.8037	101	58	Hypothetical protein CBG15962
Locus_15025_Transcript_1/3_Conf_0.667	924	20	1.06E-49	NP_498711.1	62	201.445	199	124	TYRosinase family member (tyr-1)
Locus_15025_Transcript_2/3_Conf_0.667	924	20	1.06E-49	NP_498711.1	62	201.445	199	124	TYRosinase family member (tyr-1)
Locus_15025_Transcript_3/3_Conf_0.667	924	20	1.06E-49	NP_498711.1	62	201.445	199	124	TYRosinase family member (tyr-1)
Locus_15026_Transcript_1/1_Conf_1.000	544	4	2.11E-44	XP_002641787.1	71	182.185	152	109	Hypothetical protein CBG10134
Locus_15027_Transcript_1/1_Conf_1.000	130	2	1.09E-08	NP_491911.3	79	63.1586	43	34	hypothetical protein ZC581.3
Locus_15028_Transcript_1/1_Conf_1.000	1009	1	7.76E-20	NP_495158.3	45	102.449	320	147	hypothetical protein ZK1248.13
Locus_15029_Transcript_1/1_Conf_1.000	422	20	4.43E-58	ABS45067.1	94	227.254	140	132	latrophilin-like protein 2
Locus_1503_Transcript_1/1_Conf_1.000	2692	20	0	NP_741616.2	65	1031.55	913	597	CRiM (Cysteine RIch motor neuron protein) homolog family member (crm-1)
Locus_15030_Transcript_1/1_Conf_1.000	563	0							
Locus_15031_Transcript_1/1_Conf_1.000	479	20	2.56E-42	NP_501928.1	74	174.866	139	103	hypothetical protein F13B12.4
Locus_15032_Transcript_1/1_Conf_1.000	693	6	7.33E-59	NP_502752.1	64	231.106	211	137	hypothetical protein LLC1.2
Locus_15033_Transcript_1/1_Conf_1.000	156	0							
Locus_15034_Transcript_1/1_Conf_1.000	147	20	6.31E-20	XP_002833008.1	100	100.523	48	48	PREDICTED: heme-binding protein 2-like isoform 2
Locus_15035_Transcript_1/1_Conf_1.000	589	5	1.75E-40	NP_505776.1	85	169.474	149	127	hypothetical protein F32D8.5
Locus_15036_Transcript_1/1_Conf_1.000	843	2	5.95E-09	EFO24331.1	64	65.855	78	50	hypothetical protein LOAG_04146
Locus_15037_Transcript_1/1_Conf_1.000	193	20	4.97E-09	XP_002737095.1	70	64.3142	57	40	PREDICTED: glutathione S-transferase kappa 1-like

Locus_15038_Transcript_1/1_Conf_1.000	280	2	3.59E-12	XP_002631029.1	68	74.7146	67	46	Hypothetical protein CBG02786
Locus_15039_Transcript_1/1_Conf_1.000	288	20	3.32E-29	XP_002633438.1	82	131.339	95	78	Hypothetical protein CBG06206
Locus_1504_Transcript_1/1_Conf_1.000	1163	20	9.59E-129	XP_002636865.1	74	464.537	390	292	Hypothetical protein CBG09322
Locus_15040_Transcript_1/1_Conf_1.000	488	0							
Locus_15041_Transcript_1/1_Conf_1.000	257	20	6.11E-07	XP_002159471.1	50	57.3806	91	46	PREDICTED: similar to alkaline phosphatase, liver/bone/kidney, partial
Locus_15042_Transcript_1/1_Conf_1.000	303	0							
Locus_15043_Transcript_1/1_Conf_1.000	294	20	3.66E-12	NP_495668.1	56	74.7146	98	55	hypothetical protein ZK669.2
Locus_15044_Transcript_1/2_Conf_1.000	720	0							
Locus_15044_Transcript_2/2_Conf_1.000	485	0							

Locus_15045_Transcript_1/1_Conf_1.000	686	19	1.50E-16	XP_002648013.1	45	90.5077	212	97	Hypothetical protein CBG23963
Locus_15046_Transcript_1/1_Conf_1.000	331	20	1.45E-53	NP_491646.3	87	212.231	109	95	hypothetical protein F46F11.7
Locus_15047_Transcript_1/2_Conf_1.000	557	20	1.13E-51	XP_002647223.1	73	206.453	180	132	Hypothetical protein CBG23813
Locus_15047_Transcript_2/2_Conf_1.000	555	20	1.13E-51	XP_002647223.1	74	206.453	175	130	Hypothetical protein CBG23813
Locus_15048_Transcript_1/1_Conf_1.000	436	20	4.84E-25	XP_002972906.1	68	117.472	125	85	hypothetical protein SELMODRAFT_98330
Locus_15049_Transcript_1/1_Conf_1.000	557	20	1.85E-70	XP_002630469.1	86	268.855	166	143	C. briggsae CBR-SQV-7 protein
Locus_1505_Transcript_1/1_Conf_1.000	451	20	5.37E-48	XP_002640609.1	85	193.741	149	128	C. briggsae CBR-PRI-2 protein
Locus_15050_Transcript_1/1_Conf_1.000	327	0							
Locus_15051_Transcript_1/1_Conf_1.000	192	0							
Locus_15052_Transcript_1/1_Conf_1.000	303	0							
Locus_15053_Transcript_1/1_Conf_1.000	746	0							
Locus_15054_Transcript_1/1_Conf_1.000	340	6	3.71E-09	CBM41222.1	55	64.6994	90	50	C. elegans protein T10G3.3b, partially confirmed by transcript evidence
Locus_15055_Transcript_1/1_Conf_1.000	377	20	1.17E-26	XP_002641207.1	70	122.865	108	76	Hypothetical protein CBG09068
Locus_15056_Transcript_1/1_Conf_1.000	748	0							
Locus_15057_Transcript_1/1_Conf_1.000	198	13	1.81E-11	EFO18099.1	63	72.4034	63	40	hypothetical protein LOAG_10400
Locus_15058_Transcript_1/1_Conf_1.000	140	0							

Locus_15059_Transcript_1/1_Conf_1.000	439	20	1.55E-23	NP_509583.1	71	112.464	112	80	hypothetical protein B0272.4
Locus_1506_Transcript_1/1_Conf_1.000	419	20	7.93E-23	XP_002636649.1	80	110.153	70	56	Hypothetical protein CBG23360
Locus_15060_Transcript_1/1_Conf_1.000	129	2	2.35E-11	XP_002640624.1	88	72.0182	42	37	Hypothetical protein CBG08742
Locus_15061_Transcript_1/1_Conf_1.000	512	20	2.54E-64	XP_002641212.1	83	248.054	159	132	Hypothetical protein CBG09074
Locus_15062_Transcript_1/1_Conf_1.000	520	6	7.54E-06	XP_002598663.1	42	53.9138	124	53	hypothetical protein BRAFLDRAFT_67064
Locus_15063_Transcript_1/1_Conf_1.000	289	0							
Locus_15064_Transcript_1/1_Conf_1.000	388	20	1.91E-21	NP_497585.1	68	105.531	123	84	G Protein, Alpha subunit family member (gpa-17)

Locus_15065_Transcript_1/1_Conf_1.000	572	20	3.89E-26	CAA94882.2	60	121.709	193	117	C. elegans protein B0024.10, confirmed by transcript evidence
Locus_15066_Transcript_1/1_Conf_1.000	205	5	7.28E-29	CAR63715.1	100	130.183	67	67	putative NHL (ring finger b-box coiled coil) domain containing family member
Locus_15067_Transcript_1/1_Conf_1.000	533	20	8.44E-27	XP_002639475.1	61	123.635	172	105	Hypothetical protein CBG04073
Locus_15068_Transcript_1/1_Conf_1.000	237	6	3.88E-14	ACJ65180.1	73	81.2629	79	58	Gaba b receptor subunit protein 2, confirmed by transcript evidence
Locus_15069_Transcript_1/1_Conf_1.000	303	20	8.57E-30	XP_001898220.1	83	133.265	100	83	multidrug Resistance Protein family member (mrp-5)
Locus_1507_Transcript_1/2_Conf_1.000	1909	8	2.19E-44	XP_002641295.1	63	185.267	282	180	Hypothetical protein CBG05210
Locus_1507_Transcript_2/2_Conf_1.000	1215	8	1.21E-44	XP_002641295.1	63	185.267	282	180	Hypothetical protein CBG05210
Locus_15070_Transcript_1/1_Conf_1.000	163	20	2.53E-13	NP_493802.2	85	78.5666	54	46	SyNapTotagmin family member (snt-6)
Locus_15071_Transcript_1/1_Conf_1.000	140	0							
Locus_15072_Transcript_1/1_Conf_1.000	308	20	1.41E-24	AAL15593.1	72	115.931	103	75	AF322256_14Sim15
Locus_15073_Transcript_1/1_Conf_1.000	414	0							

Locus_15074_Transcript_1/1_Conf_1.000	513	20	5.10E-52	NP_496057.1	83	207.223	155	130	EXCretory canal abnormal family member (exc-7)
Locus_15075_Transcript_1/1_Conf_1.000	181	13	7.75E-18	NP_001021395.1	88	93.5893	52	46	Receptor Mediated Endocytosis family member (rme-8)
Locus_15076_Transcript_1/1_Conf_1.000	410	0							
Locus_15077_Transcript_1/1_Conf_1.000	755	20	4.72E-57	NP_499177.3	68	225.328	252	173	hypothetical protein ZK632.7
Locus_15078_Transcript_1/1_Conf_1.000	181	0							
Locus_15079_Transcript_1/1_Conf_1.000	186	0							
Locus_1508_Transcript_1/1_Conf_1.000	1272	20	3.15E-51	XP_002641264.1	72	207.223	211	152	C. briggsae CBR-DNJ-24 protein
Locus_15080_Transcript_1/1_Conf_1.000	286	3	2.24E-17	XP_002645893.1	78	92.0485	92	72	Hypothetical protein CBG07646
Locus_15081_Transcript_1/1_Conf_1.000	1005	20	3.31E-63	XP_001902019.1	62	246.514	295	184	PRPF39 protein
Locus_15082_Transcript_1/1_Conf_1.000	278	20	1.85E-08	CBI63218.1	76	62.3882	52	40	C. elegans protein F46G10.7c, confirmed by transcript evidence
Locus_15083_Transcript_1/1_Conf_1.000	425	1	1.65E-04	EFN74469.1	45	49.2914	81	37	hypothetical protein EAG_09297
Locus_15084_Transcript_1/1_Conf_1.000	350	0							
Locus_15085_Transcript_1/1_Conf_1.000	467	20	1.94E-29	XP_002639128.1	84	132.109	88	74	C. briggsae CBR-SPG-7 protein
Locus_15086_Transcript_1/1_Conf_1.000	810	0							
Locus_15087_Transcript_1/1_Conf_1.000	169	20	8.61E-22	NP_001076639.1	96	106.686	55	53	TWiK family of potassium channels family member (twk-39)
Locus_15088_Transcript_1/1_Conf_1.000	238	0							
Locus_15089_Transcript_1/1_Conf_1.000	138	0							
Locus_1509_Transcript_1/1_Conf_1.000	1131	20	1.05E-156	NP_001022735.1	84	557.37	376	316	hypothetical protein R155.1

Locus_15090_Transcript_1/1_Conf_1.000	500	20	7.90E-60	NP_509931.1	98	233.032	116	114	abnormal cell MIGration family member (mig-2)
Locus_15091_Transcript_1/1_Conf_1.000	290	0							
Locus_15092_Transcript_1/1_Conf_1.000	262	20	2.22E-17	XP_002632484.1	75	92.0485	70	53	Hypothetical protein CBG13719
Locus_15093_Transcript_1/1_Conf_1.000	389	0							
Locus_15094_Transcript_1/1_Conf_1.000	162	20	5.27E-11	NP_501217.2	81	70.8626	54	44	hypothetical protein R05G6.10
Locus_15095_Transcript_1/1_Conf_1.000	183	0							
Locus_15096_Transcript_1/1_Conf_1.000	409	0							
Locus_15097_Transcript_1/1_Conf_1.000	298	0							
Locus_15098_Transcript_1/1_Conf_1.000	183	20	9.15E-11	XP_002640025.1	87	70.0922	40	35	Hypothetical protein CBG12497
Locus_15099_Transcript_1/1_Conf_1.000	159	1	3.32E-05	EFO20471.1	69	51.6026	53	37	hypothetical protein LOAG_08020
Locus_151_Transcript_1/1_Conf_1.000	814	0							
Locus_1510_Transcript_1/1_Conf_1.000	1331	20	2.01E-88	XP_002639683.1	62	330.872	444	277	C. briggsae CBR-GEI-11 protein
Locus_15100_Transcript_1/1_Conf_1.000	128	2	3.65E-04	NP_499641.2	64	48.1358	37	24	hypothetical protein Y111B2A.10
Locus_15101_Transcript_1/1_Conf_1.000	879	20	5.20E-19	XP_002637508.1	75	99.3673	79	60	C. briggsae CBR-TAG-232 protein
Locus_15102_Transcript_1/1_Conf_1.000	314	0							
Locus_15103_Transcript_1/1_Conf_1.000	523	0							
Locus_15104_Transcript_1/1_Conf_1.000	350	20	1.87E-32	NP_508492.1	71	142.124	116	83	hypothetical protein Y71H10B.1

Locus_15105_Transcript_1/1_Conf_1.000	315	20	9.35E-32	AAL72637.1	73	139.813	105	77	AF411588_1inositol polyphosphate 5-phosphatase type I
Locus_15106_Transcript_1/1_Conf_1.000	326	20	3.40E-26	NP_492719.1	85	121.324	70	60	hypothetical protein F45H11.5
Locus_15107_Transcript_1/1_Conf_1.000	185	0							
Locus_15108_Transcript_1/1_Conf_1.000	196	0							
Locus_15109_Transcript_1/1_Conf_1.000	955	20	5.47E-89	CAB05198.3	80	332.028	240	193	C. elegans protein F52B11.2, partially confirmed by transcript evidence
Locus_1511_Transcript_1/2_Conf_1.000	1744	3	1.11E-23	XP_001898374.1	48	116.316	346	168	hypothetical protein Bm1_34635
Locus_1511_Transcript_2/2_Conf_1.000	1645	20	4.06E-28	NP_504644.1	80	130.954	94	76	ACyLtransferase-like family member (acl-9)
Locus_15110_Transcript_1/1_Conf_1.000	381	20	1.41E-48	XP_002648358.1	88	195.667	125	111	Hypothetical protein CBG24564
Locus_15111_Transcript_1/1_Conf_1.000	316	20	2.99E-54	CAI15061.1	100	214.542	105	105	guanylate kinase 1
Locus_15112_Transcript_1/1_Conf_1.000	176	0							
Locus_15113_Transcript_1/2_Conf_1.000	448	0							
Locus_15113_Transcript_2/2_Conf_1.000	472	0							
Locus_15114_Transcript_1/1_Conf_1.000	316	20	1.49E-37	NP_741746.1	98	159.073	105	103	Kinesin-Like Protein family member (klp-4)
Locus_15115_Transcript_1/1_Conf_1.000	1793	20	3.41E-76	NP_872042.1	58	290.812	499	294	hypothetical protein C27H5.2
Locus_15116_Transcript_1/1_Conf_1.000	352	2	3.09E-11	NP_495375.3	53	71.633	115	61	hypothetical protein T14B4.1
Locus_15117_Transcript_1/1_Conf_1.000	366	20	6.98E-16	EFO23847.1	72	87.0409	75	54	hypothetical protein LOAG_04634

Locus_15118_Transcript_1/1_Conf_1.000	157	20	1.50E-05	XP_002004206.1	69	52.7582	46	32	GI19788
Locus_15119_Transcript_1/1_Conf_1.000	271	8	9.89E-10	NP_491880.1	60	66.6254	82	50	hypothetical protein T10E9.6
Locus_1512_Transcript_1/1_Conf_1.000	2516	20	0	EFO26683.1	88	916.376	614	545	elongation factor Tu GTP binding domain-containing protein
Locus_15120_Transcript_1/1_Conf_1.000	263	5	3.66E-20	NP_001123142.1	74	101.293	81	60	related to yeast Vacuolar Protein Sorting factor family member (vps-41)
Locus_15121_Transcript_1/1_Conf_1.000	172	20	3.15E-24	XP_002631669.1	100	114.775	56	56	C. briggsae CBR-MOG-4 protein
Locus_15122_Transcript_1/3_Conf_0.571	674	0							
Locus_15122_Transcript_2/3_Conf_0.714	790	0							
Locus_15122_Transcript_3/3_Conf_0.714	789	0							
Locus_15123_Transcript_1/1_Conf_1.000	385	0							

Locus_15124_Transcript_1/1_Conf_1.000	1032	20	1.25E-121	XP_002643017.1	76	440.654	340	260	C. briggsae CBR-POLK-1 protein
Locus_15125_Transcript_1/1_Conf_1.000	620	2	5.89E-40	XP_002634811.1	77	167.933	120	93	Hypothetical protein CBG13916
Locus_15126_Transcript_1/1_Conf_1.000	697	20	1.28E-10	XP_002642710.1	70	70.8626	75	53	C. briggsae CBR-MCM-5 protein
Locus_15127_Transcript_1/1_Conf_1.000	297	0							
Locus_15128_Transcript_1/1_Conf_1.000	613	20	3.43E-85	XP_002645218.1	94	318.161	191	180	Hypothetical protein CBG00076
Locus_15129_Transcript_1/1_Conf_1.000	172	3	3.38E-10	XP_002642486.1	78	68.1662	55	43	Hypothetical protein CBG06905
Locus_1513_Transcript_1/2_Conf_1.000	593	0							
Locus_1513_Transcript_2/2_Conf_1.000	2315	20	1.12E-170	XP_002639813.1	63	605.134	665	422	C. briggsae CBR-WTS-1 protein
Locus_15130_Transcript_1/1_Conf_1.000	308	3	1.47E-05	XP_001607761.1	82	52.7582	34	28	PREDICTED: similar to ENSANGP00000004989
Locus_15131_Transcript_1/1_Conf_1.000	152	0							
Locus_15132_Transcript_1/1_Conf_1.000	184	0							
Locus_15133_Transcript_1/1_Conf_1.000	358	0							
Locus_15134_Transcript_1/1_Conf_1.000	171	20	4.41E-18	CAL38390.1	100	94.3597	45	45	hypothetical protein
Locus_15135_Transcript_1/1_Conf_1.000	421	20	1.35E-14	EFO20607.1	74	82.8037	71	53	hypothetical protein LOAG_07879
Locus_15136_Transcript_1/1_Conf_1.000	606	20	5.51E-80	XP_002636870.1	85	300.827	201	172	Hypothetical protein CBG09328
Locus_15137_Transcript_1/1_Conf_1.000	204	0							
Locus_15138_Transcript_1/1_Conf_1.000	255	0							
Locus_15139_Transcript_1/1_Conf_1.000	168	0							
Locus_1514_Transcript_1/1_Conf_1.000	1282	20	1.42E-83	NP_503108.1	81	314.694	230	187	hypothetical protein C06A12.3
Locus_15140_Transcript_1/1_Conf_1.000	293	0							
Locus_15141_Transcript_1/1_Conf_1.000	137	0							

Locus_15142_Transcript_1/1_Conf_1.000	768	20	5.13E-30	XP_002647111.1	63	135.576	158	100	C. briggsae CBR-HEH-1 protein
Locus_15143_Transcript_1/1_Conf_1.000	117	0							
Locus_15144_Transcript_1/1_Conf_1.000	470	0							
Locus_15145_Transcript_1/1_Conf_1.000	271	0							
Locus_15146_Transcript_1/1_Conf_1.000	348	0							
Locus_15147_Transcript_1/1_Conf_1.000	186	0							
Locus_15148_Transcript_1/1_Conf_1.000	303	20	1.67E-25	XP_001177751.1	81	119.013	101	82	PREDICTED: hypothetical protein
Locus_15149_Transcript_1/1_Conf_1.000	406	6	3.50E-23	XP_001898115.1	64	111.309	134	87	hypothetical protein
Locus_1515_Transcript_1/3_Conf_0.500	242	0							
Locus_1515_Transcript_2/3_Conf_0.667	352	0							
Locus_1515_Transcript_3/3_Conf_0.667	298	0							
Locus_15150_Transcript_1/1_Conf_1.000	137	0							
Locus_15151_Transcript_1/1_Conf_1.000	1100	20	1.29E-95	XP_002648242.1	86	354.369	227	196	C. briggsae CBR-SRF-3 protein
Locus_15152_Transcript_1/1_Conf_1.000	496	0							
Locus_15153_Transcript_1/1_Conf_1.000	479	0							
Locus_15154_Transcript_1/1_Conf_1.000	390	20	2.23E-38	XP_002832332.1	75	161.77	134	101	PREDICTED: host cell factor 1-like
Locus_15155_Transcript_1/1_Conf_1.000	684	0							
Locus_15156_Transcript_1/1_Conf_1.000	377	20	7.56E-42	AAH29482.1	100	173.326	83	83	Hypothetical protein HSPC152
Locus_15157_Transcript_1/1_Conf_1.000	553	6	2.26E-12	XP_002630815.1	53	75.8702	145	77	Hypothetical protein CBG02516
Locus_15158_Transcript_1/1_Conf_1.000	253	0							
Locus_15159_Transcript_1/1_Conf_1.000	985	20	1.74E-93	NP_496492.1	78	347.051	338	266	hypothetical protein C47D12.2
Locus_1516_Transcript_1/1_Conf_1.000	225	5	1.77E-14	XP_002631813.1	68	82.4185	73	50	Hypothetical protein CBG21034

Locus_15160_Transcript_1/1_Conf_1.000	420	10	5.47E-24	NP_500401.1	81	114.005	121	99	hypothetical protein Y37E11AL.1
Locus_15161_Transcript_1/1_Conf_1.000	338	14	6.78E-11		62	70.4774	85	53	hypothetical protein Y105C5B.d
Locus_15162_Transcript_1/1_Conf_1.000	185	0							
Locus_15163_Transcript_1/1_Conf_1.000	671	20	3.61E-20	XP_002630952.1	54	102.449	173	94	C. briggsae CBR-ABCX-1 protein
Locus_15164_Transcript_1/1_Conf_1.000	564	20	1.73E-55	NP_500586.2	81	219.164	185	150	hypothetical protein ZK185.2
Locus_15165_Transcript_1/1_Conf_1.000	220	0							
Locus_15166_Transcript_1/1_Conf_1.000	379	20	1.10E-16	EFO21580.1	66	89.7373	75	50	hypothetical protein LOAG_06910
Locus_15167_Transcript_1/1_Conf_1.000	440	20	5.49E-37	NP_504599.2	86	157.147	104	90	Myotonic dystrophy-Related, Cdc42-binding Kinase homolog family member (mrck-1)
Locus_15168_Transcript_1/1_Conf_1.000	264	4	1.53E-26	NP_491513.2	81	122.479	88	72	Formin HOmology Domain family member (fhod-1)
Locus_15169_Transcript_1/2_Conf_1.000	226	0							
Locus_15169_Transcript_2/2_Conf_1.000	127	0							
Locus_1517_Transcript_1/1_Conf_1.000	1362	20	4.44E-131	XP_002631365.1	69	472.626	459	321	C. briggsae CBR-RHA-1 protein
Locus_15170_Transcript_1/1_Conf_1.000	492	20	5.96E-31	EFO14143.1	64	137.117	164	106	hypothetical protein LOAG_14379
Locus_15171_Transcript_1/1_Conf_1.000	342	5	1.23E-07	XP_001892437.1	64	59.6918	64	41	Gem-associated protein 7
Locus_15172_Transcript_1/1_Conf_1.000	406	20	2.13E-20	NP_503751.1	63	102.064	119	75	hypothetical protein R05D8.7

Locus_15173_Transcript_1/1_Conf_1.000	186	4	5.52E-16	XP_002630757.1	77	87.4261	62	48	Hypothetical protein CBG02451
Locus_15174_Transcript_1/1_Conf_1.000	187	0							
Locus_15175_Transcript_1/1_Conf_1.000	418	1	3.23E-08	NP_491852.1	52	61.6178	125	65	hypothetical protein F37E3.3
Locus_15176_Transcript_1/1_Conf_1.000	709	5	4.35E-22	XP_002645124.1	60	108.997	130	78	Hypothetical protein CBG16818
Locus_15177_Transcript_1/1_Conf_1.000	281	0							
Locus_15178_Transcript_1/1_Conf_1.000	314	0							
Locus_15179_Transcript_1/1_Conf_1.000	169	0							
Locus_1518_Transcript_1/1_Conf_1.000	1195	20	7.42E-132	XP_002632159.1	89	474.937	304	271	Hypothetical protein CBG07018
Locus_15180_Transcript_1/1_Conf_1.000	1008	20	4.42E-116	XP_002638881.1	79	422.165	334	264	C. briggsae CBR-VPS-34 protein
Locus_15181_Transcript_1/1_Conf_1.000	146	0							
Locus_15182_Transcript_1/1_Conf_1.000	731	20	6.69E-37	NP_506278.1	89	158.303	87	78	hypothetical protein T09E8.3
Locus_15183_Transcript_1/1_Conf_1.000	704	2	1.95E-14	EFO28233.1	48	83.5741	246	120	TTK protein kinase
Locus_15184_Transcript_1/1_Conf_1.000	194	0							
Locus_15185_Transcript_1/1_Conf_1.000	321	0							
Locus_15186_Transcript_1/1_Conf_1.000	141	0							
Locus_15187_Transcript_1/1_Conf_1.000	262	0							
Locus_15188_Transcript_1/1_Conf_1.000	232	0							
Locus_15189_Transcript_1/1_Conf_1.000	401	20	1.62E-68	XP_002640864.1	96	261.922	133	129	C. briggsae CBR-UNC-25 protein
Locus_1519_Transcript_1/2_Conf_1.000	935	20	7.67E-88	NP_505412.2	85	328.176	206	176	ACyLtransferase-like family member (acl-14)
Locus_1519_Transcript_2/2_Conf_1.000	1107	20	2.40E-73	NP_505413.1	82	280.411	180	149	hypothetical protein K07B1.4

Locus_15190_Transcript_1/1_Conf_1.000	290	20	3.44E-18	XP_002818294.1	100	94.7449	96	96	PREDICTED: collagen alpha-2(I) chain-like
Locus_15191_Transcript_1/1_Conf_1.000	279	20	1.77E-27	XP_001900518.1	79	125.561	92	73	3'5'-cyclic nucleotide phosphodiesterase family protein
Locus_15192_Transcript_1/1_Conf_1.000	325	20	9.27E-24	AAP41124.1	78	113.235	84	66	aldose reductase
Locus_15193_Transcript_1/1_Conf_1.000	678	20	3.26E-24	XP_002640845.1	52	115.931	224	118	C. briggsae CBR-CDH-1 protein
Locus_15194_Transcript_1/2_Conf_1.000	283	20	1.32E-17	XP_002630522.1	63	92.8189	97	62	Hypothetical protein CBG12960
Locus_15194_Transcript_2/2_Conf_1.000	283	20	1.32E-17	XP_002630522.1	63	92.8189	97	62	Hypothetical protein CBG12960
Locus_15195_Transcript_1/1_Conf_1.000	265	0							
Locus_15196_Transcript_1/1_Conf_1.000	342	0							
Locus_15197_Transcript_1/1_Conf_1.000	132	20	2.87E-09	AAC72298.1	83	65.0846	43	36	reverse transcriptase
Locus_15198_Transcript_1/1_Conf_1.000	764	20	2.35E-11	EFN52362.1	43	73.559	250	109	hypothetical protein CHLNCDRAFT_138782
Locus_15199_Transcript_1/1_Conf_1.000	139	0							

Locus_152_Transcript_1/1_Conf_1.000	305	20	1.29E-09	XP_002122773.1	63	66.2402	63	40	PREDICTED: similar to glucosidase, beta, acid
Locus_1520_Transcript_1/1_Conf_1.000	357	20	2.04E-31	EFO22150.1	78	138.658	105	82	SET domain-containing protein
Locus_15200_Transcript_1/1_Conf_1.000	254	20	2.40E-19	XP_001998853.1	73	98.5969	76	56	GI23404
Locus_15201_Transcript_1/1_Conf_1.000	1292	20	3.09E-78	XP_001896605.1	62	296.975	402	251	HYPOTHETICAL 59.2KD PROTEIN IN PFK26-SGA1 INTERGENIC REGION, putative
Locus_15202_Transcript_1/1_Conf_1.000	179	0							
Locus_15203_Transcript_1/1_Conf_1.000	383	20	5.96E-31	NP_505237.1	68	137.117	126	86	human CLN (neuronal ceroid lipofuscinosis) related family member (cln-3.3)
Locus_15204_Transcript_1/1_Conf_1.000	989	20	7.11E-79	XP_001897597.1	84	298.516	200	169	Lung seven transmembrane receptor family protein
Locus_15205_Transcript_1/1_Conf_1.000	609	20	8.39E-60	EFO27604.1	75	233.802	203	153	hypothetical protein LOAG_00873
Locus_15206_Transcript_1/1_Conf_1.000	478	20	8.51E-38	EFO19811.1	75	159.844	132	99	hemimethylated DNA binding domain-containing protein
Locus_15207_Transcript_1/1_Conf_1.000	136	4	1.02E-06	XP_001896000.1	79	56.6102	44	35	hypothetical protein
Locus_15208_Transcript_1/1_Conf_1.000	277	20	2.24E-38	NP_492839.4	92	161.77	91	84	Leucine-rich repeats, Ras-like domain, Kinase family member (lrk-1)
Locus_15209_Transcript_1/2_Conf_1.000	1065	20	2.24E-97	XP_002646179.1	73	360.147	322	238	Hypothetical protein CBG23745

Locus_15209_Transcript_2/2_Conf_1.000	948	20	1.26E-77	XP_002646179.1	63	294.278	322	204	Hypothetical protein CBG23745
Locus_1521_Transcript_1/1_Conf_1.000	1019	20	3.84E-83	NP_496604.1	72	312.768	266	193	hypothetical protein Y57A10A.26
Locus_15210_Transcript_1/1_Conf_1.000	210	0							
Locus_15211_Transcript_1/1_Conf_1.000	352	20	1.21E-23	XP_002630751.1	63	112.849	114	72	Hypothetical protein CBG02443
Locus_15212_Transcript_1/1_Conf_1.000	460	5	4.54E-07	XP_002643565.1	66	57.7658	54	36	Hypothetical protein CBG16267
Locus_15213_Transcript_1/1_Conf_1.000	209	0							
Locus_15214_Transcript_1/1_Conf_1.000	138	0							
Locus_15215_Transcript_1/1_Conf_1.000	156	0							
Locus_15216_Transcript_1/1_Conf_1.000	471	20	8.26E-57	NP_500720.1	87	223.016	147	128	IsoValeryl-CoA Dehydrogenase family member (ivd-1)
Locus_15217_Transcript_1/1_Conf_1.000	529	20	1.61E-30	XP_002633897.1	66	135.961	168	112	Hypothetical protein CBG19959
Locus_15218_Transcript_1/1_Conf_1.000	182	0							
Locus_15219_Transcript_1/1_Conf_1.000	145	20	2.18E-20	XP_002759346.1	100	102.064	48	48	PREDICTED: eukaryotic translation initiation factor 3 subunit E
Locus_1522_Transcript_1/2_Conf_1.000	2182	20	7.02E-26	ZP_02995497.1	54	124.02	127	69	hypothetical protein CLOSPO_02619
Locus_1522_Transcript_2/2_Conf_1.000	2188	20	7.04E-26	ZP_02995497.1	54	124.02	127	69	hypothetical protein CLOSPO_02619
Locus_15220_Transcript_1/1_Conf_1.000	246	20	1.23E-20	XP_002646069.1	85	102.834	67	57	C. briggsae CBR-GALE-1 protein
Locus_15221_Transcript_1/2_Conf_1.000	343	0							
Locus_15221_Transcript_2/2_Conf_1.000	207	0							
Locus_15222_Transcript_1/1_Conf_1.000	436	20	5.16E-35	XP_001900114.1	70	150.599	132	93	MGC14327-like protein
Locus_15223_Transcript_1/2_Conf_1.000	431	20	1.01E-38	NP_498705.1	92	162.925	93	86	Temporarily Assigned Gene name family member (tag-277)

Locus_15223_Transcript_2/2_Conf_1.000	386	20	1.01E-38	NP_498705.1	92	162.925	93	86	Temporarily Assigned Gene name family member (tag-277)
Locus_15224_Transcript_1/1_Conf_1.000	224	0							
Locus_15225_Transcript_1/2_Conf_1.000	896	20	8.53E-33	NP_491838.1	74	145.206	114	85	hypothetical protein K04F10.7
Locus_15225_Transcript_2/2_Conf_1.000	896	20	8.53E-33	NP_491838.1	74	145.206	114	85	hypothetical protein K04F10.7
Locus_15226_Transcript_1/1_Conf_1.000	235	12	2.61E-18	NP_502380.1	85	95.1301	63	54	hypothetical protein C25G4.6
Locus_15227_Transcript_1/1_Conf_1.000	305	20	1.51E-26	XP_002832428.1	100	122.479	58	58	PREDICTED: neudesin-like
Locus_15228_Transcript_1/1_Conf_1.000	212	20	2.33E-19	EFO17543.1	87	98.5969	57	50	aspartate aminotransferase
Locus_15229_Transcript_1/1_Conf_1.000	359	16	6.35E-33	XP_002639055.1	82	143.665	103	85	C. briggsae CBR-GCY-28 protein
Locus_1523_Transcript_1/1_Conf_1.000	1218	20	9.22E-61	NP_498568.1	83	238.81	160	134	fatty Acid CoA Synthetase family member (acs-16)
Locus_15230_Transcript_1/1_Conf_1.000	190	20	2.56E-13	NP_498621.1	81	78.5666	64	52	GOlgi Snap Receptor complex member family member (gosr-1)
Locus_15231_Transcript_1/1_Conf_1.000	133	0							
Locus_15232_Transcript_1/1_Conf_1.000	725	20	1.23E-51	XP_001892945.1	71	207.223	239	170	exosome complex exonuclease RRP40
Locus_15233_Transcript_1/1_Conf_1.000	169	0							
Locus_15234_Transcript_1/1_Conf_1.000	491	0							
Locus_15235_Transcript_1/1_Conf_1.000	385	20	1.55E-55	NP_491165.2	99	218.779	128	127	human SPG (spastic paraplegia) family member (spg-7)

Locus_15236_Transcript_1/1_Conf_1.000	129	6	5.61E-05	ZP_01471425.1	79	50.8322	39	31	short-chain dehydrogenase/reductase (SDR) superfamily protein
Locus_15237_Transcript_1/1_Conf_1.000	385	20	7.24E-37	NP_491915.2	82	156.762	124	102	hypothetical protein ZC581.9
Locus_15238_Transcript_1/1_Conf_1.000	251	20	6.95E-32	XP_002644493.1	96	140.198	83	80	Hypothetical protein CBG14380
Locus_15239_Transcript_1/1_Conf_1.000	446	20	5.22E-59	XP_002633481.1	86	230.335	147	127	C. briggsae CBR-PHY-2 protein
Locus_1524_Transcript_1/2_Conf_1.000	607	3	8.62E-33	CAR63637.1	75	144.05	115	87	hypothetical protein
Locus_1524_Transcript_2/2_Conf_1.000	247	0							
Locus_15240_Transcript_1/1_Conf_1.000	223	0							
Locus_15241_Transcript_1/1_Conf_1.000	1252	0							
Locus_15242_Transcript_1/1_Conf_1.000	216	13	3.95E-19	XP_002639744.1	81	97.8265	70	57	C. briggsae CBR-LAM-3 protein
Locus_15243_Transcript_1/1_Conf_1.000	259	0							
Locus_15244_Transcript_1/1_Conf_1.000	959	20	2.20E-29	NP_001076617.1	63	134.035	131	83	hypothetical protein W01A8.8
Locus_15245_Transcript_1/1_Conf_1.000	346	16	3.02E-06	XP_002636866.1	66	55.0694	66	44	Hypothetical protein CBG09323
Locus_15246_Transcript_1/1_Conf_1.000	619	20	2.23E-55	CAO00417.1	71	219.164	190	135	two-domain activation associated secreted protein ASP4 precursor
Locus_15247_Transcript_1/1_Conf_1.000	489	20	2.24E-78	XP_002643972.1	96	294.664	162	156	C. briggsae CBR-SLO-2 protein
Locus_15248_Transcript_1/2_Conf_1.000	403	0							
Locus_15248_Transcript_2/2_Conf_1.000	397	0							

Locus_15249_Transcript_1/1_Conf_1.000	1090	20	3.36E-96	NP_501359.1	72	356.295	359	262	hypothetical protein F42G8.6
Locus_1525_Transcript_1/1_Conf_1.000	1322	20	4.25E-14	XP_001899731.1	81	83.9593	55	45	Protein cec-1.
Locus_15250_Transcript_1/1_Conf_1.000	365	20	1.08E-24	NP_495704.1	81	116.316	72	59	CysTiNoSin (lysosomal protein) homolog family member (ctns-1)
Locus_15251_Transcript_1/1_Conf_1.000	146	3	1.56E-10	XP_002641998.1	92	69.3218	39	36	Hypothetical protein CBG09222
Locus_15252_Transcript_1/1_Conf_1.000	247	20	8.01E-12	EFO25925.1	75	73.559	64	48	SMC protein Flexible Hinge Domain containing protein
Locus_15253_Transcript_1/1_Conf_1.000	575	0							
Locus_15254_Transcript_1/1_Conf_1.000	533	20	2.81E-22	ACS37721.1	51	108.612	175	90	C-type lectin-1
Locus_15255_Transcript_1/1_Conf_1.000	387	20	1.66E-41	A8XST1.2	76	172.17	128	98	Post-GPI attachment to proteins factor 2
Locus_15256_Transcript_1/1_Conf_1.000	307	0							
Locus_15257_Transcript_1/2_Conf_1.000	1142	4	5.86E-06	NP_505144.2	52	56.6102	119	62	hypothetical protein W02D7.6
Locus_15257_Transcript_2/2_Conf_1.000	1142	4	5.86E-06	NP_505144.2	52	56.6102	119	62	hypothetical protein W02D7.6
Locus_15258_Transcript_1/1_Conf_1.000	139	0							
Locus_15259_Transcript_1/1_Conf_1.000	190	0							
Locus_1526_Transcript_1/2_Conf_1.000	1264	20	1.90E-32	NP_507998.1	61	144.821	233	143	hypothetical protein F53F8.5
Locus_1526_Transcript_2/2_Conf_1.000	1177	20	2.04E-17	NP_507998.1	50	94.7449	233	118	hypothetical protein F53F8.5

Locus_15260_Transcript_1/1_Conf_1.000	256	20	5.70E-13	XP_002645055.1	54	77.411	82	45	Hypothetical protein CBG16715
Locus_15261_Transcript_1/1_Conf_1.000	227	0							
Locus_15262_Transcript_1/1_Conf_1.000	258	0							
Locus_15263_Transcript_1/1_Conf_1.000	329	0							
Locus_15264_Transcript_1/1_Conf_1.000	520	20	1.19E-43	XP_002630483.1	83	179.489	109	91	Hypothetical protein CBG11219
Locus_15265_Transcript_1/1_Conf_1.000	944	20	2.52E-70	EFO21354.1	67	270.011	313	210	hypothetical protein LOAG_07131
Locus_15266_Transcript_1/1_Conf_1.000	379	20	1.57E-47	XP_002634501.1	87	192.2	127	111	C. briggsae CBR-SMGL-2 protein
Locus_15267_Transcript_1/1_Conf_1.000	130	0							
Locus_15268_Transcript_1/1_Conf_1.000	499	20	2.91E-46	XP_001899325.1	72	187.963	165	120	GDP-fucose protein O-fucosyltransferase 2 precursor
Locus_15269_Transcript_1/1_Conf_1.000	438	20	7.40E-42	XP_002645831.1	77	173.326	142	110	C. briggsae CBR-RBC-1 protein
Locus_1527_Transcript_1/1_Conf_1.000	582	0							
Locus_15270_Transcript_1/1_Conf_1.000	203	0							
Locus_15271_Transcript_1/1_Conf_1.000	495	17	8.33E-09	EFN69196.1	60	63.5438	86	52	hypothetical protein EAG_13720
Locus_15272_Transcript_1/1_Conf_1.000	177	0							
Locus_15273_Transcript_1/1_Conf_1.000	449	13	7.11E-16	NP_496492.1	70	87.0409	75	53	hypothetical protein C47D12.2
Locus_15274_Transcript_1/1_Conf_1.000	284	3	2.10E-15	NP_497247.2	56	85.5001	93	53	GEX Interacting protein family member (gei-16)
Locus_15275_Transcript_1/1_Conf_1.000	235	0							

Locus_15276_Transcript_1/1_Conf_1.000	458	20	2.95E-06	NP_499181.1	54	55.0694	85	46	hypothetical protein ZK632.10
Locus_15277_Transcript_1/1_Conf_1.000	502	20	1.53E-50	NP_495899.1	81	202.216	140	114	hypothetical protein T21B10.1
Locus_15278_Transcript_1/1_Conf_1.000	272	0							
Locus_15279_Transcript_1/1_Conf_1.000	205	1	8.10E-04	NP_001023900.1	66	46.9802	66	44	hypothetical protein F39G3.5
Locus_1528_Transcript_1/1_Conf_1.000	430	20	2.86E-25	NP_001122693.1	73	118.242	117	86	POLK (DNA polymerase kappa) homolog family member (polk-1)
Locus_15280_Transcript_1/1_Conf_1.000	442	20	1.06E-35	XP_002643964.1	72	152.91	137	100	C. briggsae CBR-HUM-4 protein
Locus_15281_Transcript_1/2_Conf_1.000	1053	20	2.59E-114	NP_501835.2	73	416.387	356	263	DIS3 (yeast disjunction abnormal) exonuclease homolog family member (dis-3)
Locus_15281_Transcript_2/2_Conf_1.000	1011	20	6.26E-86	NP_501835.2	71	322.013	300	213	DIS3 (yeast disjunction abnormal) exonuclease homolog family member (dis-3)
Locus_15282_Transcript_1/1_Conf_1.000	243	0							
Locus_15283_Transcript_1/1_Conf_1.000	988	10	2.17E-35	ABF71718.3	69	154.066	297	205	Collagen protein 46, partially confirmed by transcript evidence
Locus_15284_Transcript_1/2_Conf_1.000	682	20	8.56E-73	EFO23808.1	78	277.33	227	179	hypothetical protein LOAG_04674
Locus_15284_Transcript_2/2_Conf_1.000	682	20	8.56E-73	EFO23808.1	78	277.33	227	179	hypothetical protein LOAG_04674
Locus_15285_Transcript_1/1_Conf_1.000	259	0							
Locus_15286_Transcript_1/1_Conf_1.000	1200	20	5.45E-74	XP_001896288.1	62	282.722	351	220	mbt repeat family protein
Locus_15287_Transcript_1/1_Conf_1.000	221	0							
Locus_15288_Transcript_1/1_Conf_1.000	227	20	2.24E-09	XP_002647659.1	70	65.4698	47	33	Hypothetical protein CBG06766
Locus_15289_Transcript_1/1_Conf_1.000	384	20	7.29E-13	ADI24665.1	63	77.0258	77	49	Hypothetical protein Y38C1AA.13

Locus_1529_Transcript_1/1_Conf_1.000	435	20	1.83E-32	XP_002641208.1	70	142.124	125	88	C. briggsae CBR-DPF-4 protein
Locus_15290_Transcript_1/1_Conf_1.000	199	0							
Locus_15291_Transcript_1/2_Conf_1.000	413	0							
Locus_15291_Transcript_2/2_Conf_1.000	413	0							
Locus_15292_Transcript_1/1_Conf_1.000	160	0							
Locus_15293_Transcript_1/1_Conf_1.000	810	20	6.95E-121	NP_510603.1	88	437.573	269	239	hypothetical protein F59F4.1
Locus_15294_Transcript_1/1_Conf_1.000	356	4	3.29E-13	XP_001895370.1	64	78.1814	116	75	hypothetical protein Bm1_19575
Locus_15295_Transcript_1/1_Conf_1.000	218	0							
Locus_15296_Transcript_1/1_Conf_1.000	147	0							
Locus_15297_Transcript_1/1_Conf_1.000	139	0							
Locus_15298_Transcript_1/1_Conf_1.000	367	20	9.98E-47	NP_001023627.1	89	189.504	119	106	Na/H eXchanger family member (nhx-9)
Locus_15299_Transcript_1/1_Conf_1.000	183	0							
Locus_153_Transcript_1/3_Conf_0.333	166	0							
Locus_153_Transcript_2/3_Conf_0.833	411	20	2.59E-15	NP_508612.1	68	81.6481	80	55	VITellogenin structural genes (yolk protein genes) family member (vit-4)
Locus_153_Transcript_3/3_Conf_0.833	402	20	5.75E-15	NP_508612.1	69	80.4925	78	54	VITellogenin structural genes (yolk protein genes) family member (vit-4)
Locus_1530_Transcript_1/1_Conf_1.000	509	5	2.94E-36	NP_496819.1	65	154.836	159	104	hypothetical protein Y48C3A.20
Locus_15300_Transcript_1/1_Conf_1.000	252	20	2.65E-26	XP_002629711.1	79	121.709	83	66	C. briggsae CBR-RHY-1 protein
Locus_15301_Transcript_1/1_Conf_1.000	226	0							
Locus_15302_Transcript_1/1_Conf_1.000	304	0							
Locus_15303_Transcript_1/1_Conf_1.000	203	20	1.48E-13	NP_001022166.1	79	79.337	59	47	Temporarily Assigned Gene name family member (tag-169)
Locus_15304_Transcript_1/1_Conf_1.000	157	0							
Locus_15305_Transcript_1/1_Conf_1.000	575	20	3.46E-30	XP_002631433.1	64	135.191	153	99	C. briggsae CBR-REV-1 protein
Locus_15306_Transcript_1/1_Conf_1.000	153	20	2.16E-20	XP_002829638.1	100	102.064	51	51	PREDICTED: prostate tumor-overexpressed gene 1 protein-like
Locus_15307_Transcript_1/1_Conf_1.000	154	3	8.79E-06	XP_002641552.1	68	53.5286	47	32	C. briggsae CBR-RBC-2 protein

Locus_15308_Transcript_1/1_Conf_1.000	342	0							
Locus_15309_Transcript_1/1_Conf_1.000	136	0							
Locus_1531_Transcript_1/3_Conf_0.714	691	0							
Locus_1531_Transcript_2/3_Conf_0.714	1342	4	3.80E-18	NP_872051.1	50	97.4413	213	107	hypothetical protein Y17G7B.20
Locus_1531_Transcript_3/3_Conf_0.714	615	0							
Locus_15310_Transcript_1/1_Conf_1.000	439	20	3.67E-41	NP_498122.2	80	171.014	134	108	abnormal GROwth rate family member (gro-1)
Locus_15311_Transcript_1/1_Conf_1.000	280	20	8.51E-30	XP_002634229.1	80	133.265	90	72	C. briggsae CBR-UNC-26 protein
Locus_15312_Transcript_1/1_Conf_1.000	142	0							
Locus_15313_Transcript_1/1_Conf_1.000	145	1	1.28E-04	XP_002638613.1	86	49.6766	43	37	C. briggsae CBR-PGP-9 protein
Locus_15314_Transcript_1/1_Conf_1.000	533	20	4.76E-46	NP_502386.1	73	187.578	171	126	hypothetical protein T04A11.2
Locus_15315_Transcript_1/1_Conf_1.000	395	20	8.21E-20	NP_494781.1	74	100.138	86	64	hypothetical protein ZK430.7
Locus_15316_Transcript_1/1_Conf_1.000	342	0							
Locus_15317_Transcript_1/1_Conf_1.000	384	0							
Locus_15318_Transcript_1/1_Conf_1.000	797	20	1.60E-45	EFO17307.1	59	187.193	278	165	hypothetical protein LOAG_11192
Locus_15319_Transcript_1/1_Conf_1.000	395	2	3.45E-18	NP_500196.2	77	94.7449	72	56	hypothetical protein Y38F2AR.3
Locus_1532_Transcript_1/1_Conf_1.000	755	20	1.09E-37	CAB71314.1	70	160.999	136	96	galectin
Locus_15320_Transcript_1/1_Conf_1.000	198	0							

Locus_15321_Transcript_1/1_Conf_1.000	263	19	8.75E-06	XP_002934628.1	52	53.5286	71	37	PREDICTED: regenerating islet-derived protein 4-like
Locus_15322_Transcript_1/1_Conf_1.000	383	20	1.28E-41	XP_002636935.1	84	172.555	126	106	C. briggsae CBR-UIG-1 protein
Locus_15323_Transcript_1/1_Conf_1.000	226	0							
Locus_15324_Transcript_1/1_Conf_1.000	390	8	5.72E-18	NP_001021405.1	82	93.9745	114	94	asparagiNyl tRNA Synthetase family member (nrs-1)
Locus_15325_Transcript_1/1_Conf_1.000	271	5	2.20E-09	EFO23808.1	57	65.4698	91	52	hypothetical protein LOAG_04674
Locus_15326_Transcript_1/1_Conf_1.000	165	0							
Locus_15327_Transcript_1/1_Conf_1.000	556	3	1.75E-28	NP_506256.3	72	129.413	177	128	CaDHerin family member (cdh-6)
Locus_15328_Transcript_1/1_Conf_1.000	200	0							
Locus_15329_Transcript_1/1_Conf_1.000	529	20	1.04E-29	XP_002640799.1	65	133.265	155	101	Hypothetical protein CBG15679
Locus_1533_Transcript_1/3_Conf_0.333	409	0							
Locus_1533_Transcript_3/3_Conf_0.667	418	0							
Locus_15330_Transcript_1/1_Conf_1.000	370	0							
Locus_15331_Transcript_1/1_Conf_1.000	609	20	2.54E-40	NP_495248.1	80	169.088	131	106	hypothetical protein T24H7.3
Locus_15332_Transcript_1/1_Conf_1.000	313	20	4.67E-23	EFO18094.1	82	110.923	64	53	hypothetical protein LOAG_10404
Locus_15333_Transcript_1/1_Conf_1.000	194	0							
Locus_15334_Transcript_1/1_Conf_1.000	296	20	7.07E-40	XP_002644104.1	90	166.777	97	88	C. briggsae CBR-PLC-1 protein
Locus_15335_Transcript_1/1_Conf_1.000	558	20	3.29E-19	XP_392522.2	49	98.5969	199	98	PREDICTED: similar to CG3894-PA, isoform A
Locus_15336_Transcript_1/1_Conf_1.000	213	0							
Locus_15337_Transcript_1/1_Conf_1.000	279	4	3.03E-19	NP_492839.4	78	98.2117	71	56	Leucine-rich repeats, Ras-like domain, Kinase family member (lrk-1)
Locus_15338_Transcript_1/1_Conf_1.000	385	20	1.01E-30	EFO16233.1	78	136.346	124	97	variant SH3 domain-containing protein
Locus_15339_Transcript_1/1_Conf_1.000	340	20	1.72E-14	AAN10061.1	72	82.4185	54	39	Kunitz-like protease inhibitor precursor
Locus_1534_Transcript_1/1_Conf_1.000	253	0							

Locus_15340_Transcript_1/1_Conf_1.000	291	20	8.14E-28	NP_500084.2	79	126.716	97	77	hypothetical protein Y77E11A.2
Locus_15341_Transcript_1/1_Conf_1.000	1280	3	4.49E-05	EFO24673.1	58	53.9138	84	49	hypothetical protein LOAG_03814
Locus_15342_Transcript_1/1_Conf_1.000	169	3	4.01E-11		75	71.2478	56	42	myoblast city protein homolog
Locus_15343_Transcript_1/1_Conf_1.000	539	0							
Locus_15344_Transcript_1/1_Conf_1.000	151	0							
Locus_15345_Transcript_1/1_Conf_1.000	443	0							
Locus_15346_Transcript_1/1_Conf_1.000	427	20	2.52E-21	NP_001022683.1	67	105.145	123	83	hypothetical protein M01A8.2
Locus_15347_Transcript_1/1_Conf_1.000	247	0							
Locus_15348_Transcript_1/1_Conf_1.000	142	0							
Locus_15349_Transcript_1/1_Conf_1.000	418	0							
Locus_1535_Transcript_1/1_Conf_1.000	1998	20	0	EFO18359.1	78	710.294	534	421	hypothetical protein LOAG_10137
Locus_15350_Transcript_1/1_Conf_1.000	298	0							
Locus_15351_Transcript_1/1_Conf_1.000	179	0							
Locus_15352_Transcript_1/1_Conf_1.000	195	20	5.09E-30	EAX09318.1	100	134.035	64	64	collagen, type VI, alpha 2, isoform CRA_c
Locus_15353_Transcript_1/1_Conf_1.000	267	0							
Locus_15354_Transcript_1/1_Conf_1.000	369	0							
Locus_15355_Transcript_1/1_Conf_1.000	517	0							
Locus_15356_Transcript_1/1_Conf_1.000	150	0							
Locus_15357_Transcript_1/1_Conf_1.000	364	6	1.31E-30	XP_002631359.1	74	135.961	118	88	Hypothetical protein CBG03194
Locus_15358_Transcript_1/1_Conf_1.000	179	0							
Locus_15359_Transcript_1/1_Conf_1.000	245	0							
Locus_1536_Transcript_1/1_Conf_1.000	279	0							

Locus_15360_Transcript_1/1_Conf_1.000	237	0								
Locus_15361_Transcript_1/1_Conf_1.000	169	0								
Locus_15362_Transcript_1/1_Conf_1.000	552	20	5.67E-56	XP_002923059.1	100	220.705	106	106	PREDICTED: LOW QUALITY PROTEIN: cellular retinoic acid-binding protein 1-like
Locus_15363_Transcript_1/1_Conf_1.000	1027	0								
Locus_15364_Transcript_1/1_Conf_1.000	1141	20	2.52E-33	XP_002647599.1	54	147.517	381	206	C. briggsae CBR-EEA-1 protein
Locus_15365_Transcript_1/1_Conf_1.000	217	0								
Locus_15366_Transcript_1/1_Conf_1.000	286	0								
Locus_15367_Transcript_1/1_Conf_1.000	439	20	6.94E-32	XP_002639196.1	64	140.198	137	89	Hypothetical protein CBG03739
Locus_15368_Transcript_1/1_Conf_1.000	812	20	2.08E-32	XP_001900821.1	57	143.665	236	135	hypothetical protein Bm1_46790
Locus_15369_Transcript_1/1_Conf_1.000	148	0								
Locus_1537_Transcript_1/1_Conf_1.000	322	0								
Locus_15370_Transcript_1/1_Conf_1.000	405	0								
Locus_15371_Transcript_1/1_Conf_1.000	234	0								
Locus_15372_Transcript_1/1_Conf_1.000	412	20	3.30E-16	XP_002712017.1	60	77.0258	92	56	PREDICTED: thiamin pyrophosphokinase 1-like
Locus_15373_Transcript_1/1_Conf_1.000	180	20	3.49E-10	CAG04255.1	71	68.1662	56	40	unnamed protein product
Locus_15374_Transcript_1/1_Conf_1.000	842	4	6.53E-08	XP_002644385.1	53	62.3882	91	49	Hypothetical protein CBG14225
Locus_15375_Transcript_1/1_Conf_1.000	148	0								
Locus_15376_Transcript_1/1_Conf_1.000	238	0								
Locus_15377_Transcript_1/1_Conf_1.000	210	2	1.24E-04	NP_502000.1	62	49.6766	69	43	hypothetical protein C06G8.1

Locus_15378_Transcript_1/1_Conf_1.000	631	20	2.07E-80	XP_002632140.1	86	302.368	192	167	C. briggsae CBR-SPTL-1 protein
Locus_15379_Transcript_1/1_Conf_1.000	484	20	5.97E-71	XP_002914026.1	100	270.011	161	161	PREDICTED: polyadenylate-binding protein 1-like
Locus_1538_Transcript_1/1_Conf_1.000	1115	0							
Locus_15380_Transcript_1/1_Conf_1.000	420	1	2.16E-04	NP_497921.1	42	48.9062	123	52	hypothetical protein M88.3
Locus_15381_Transcript_1/1_Conf_1.000	515	20	2.09E-13	EFO16274.1	49	78.9518	148	73	hypothetical protein LOAG_12234
Locus_15382_Transcript_1/1_Conf_1.000	259	0							
Locus_15383_Transcript_1/1_Conf_1.000	191	0							
Locus_15384_Transcript_1/1_Conf_1.000	195	0							
Locus_15385_Transcript_1/1_Conf_1.000	492	5	1.29E-17	NP_508573.1	60	92.8189	147	89	Discoidin Domain Receptor family member (ddr-2)
Locus_15386_Transcript_1/1_Conf_1.000	928	9	7.42E-51	NP_492132.1	56	205.297	331	186	Enhancer of Glp-One (glp-1) family member (ego-1)
Locus_15387_Transcript_1/1_Conf_1.000	285	0							
Locus_15388_Transcript_1/1_Conf_1.000	506	20	3.41E-21	Q17678.6	71	104.76	102	73	UPF0632 protein C05G5.5

Locus_15389_Transcript_1/1_Conf_1.000	212	20	5.56E-13	3LTV	100	77.411	37	37	Mouse-Human Sod1 Chimera
Locus_1539_Transcript_1/1_Conf_1.000	501	20	4.01E-67	XP_002634908.1	83	257.299	161	135	Hypothetical protein CBG22506
Locus_15390_Transcript_1/1_Conf_1.000	326	20	1.98E-58	XP_002831943.1	100	228.409	108	108	PREDICTED: 60S ribosomal protein L36a-like isoform 1
Locus_15391_Transcript_1/1_Conf_1.000	830	20	9.86E-102	P34288.4	83	374.015	275	230	GTPase-activating protein pac-1
Locus_15392_Transcript_1/1_Conf_1.000	539	20	4.23E-50	NP_506859.2	79	201.06	177	140	hypothetical protein C06B8.7
Locus_15393_Transcript_1/1_Conf_1.000	615	20	1.47E-67	EFO23641.1	76	259.61	210	161	hypothetical protein LOAG_04841
Locus_15394_Transcript_1/1_Conf_1.000	329	0							

Locus_15395_Transcript_1/1_Conf_1.000	481	20	1.81E-64	XP_002630872.1	92	248.44	140	129	Hypothetical protein CBG02589
Locus_15396_Transcript_1/1_Conf_1.000	365	20	4.69E-20	EFO25500.1	78	100.908	80	63	STE/STE20/TAO protein kinase
Locus_15397_Transcript_1/1_Conf_1.000	150	0							
Locus_15398_Transcript_1/1_Conf_1.000	303	6	1.24E-12	CAA10033.1	67	76.2554	100	67	DYS-1 protein
Locus_15399_Transcript_1/1_Conf_1.000	312	20	1.90E-24	NP_506256.3	66	115.546	104	69	CaDHerin family member (cdh-6)
Locus_154_Transcript_1/1_Conf_1.000	366	20	1.07E-32	XP_001897933.1	77	142.895	115	89	RE01069p
Locus_1540_Transcript_1/1_Conf_1.000	534	0							

Locus_15400_Transcript_1/1_Conf_1.000	669	20	9.29E-101	CAR63641.1	97	370.163	206	200	putative 26S proteasome regulatory chain 4
Locus_15401_Transcript_1/1_Conf_1.000	401	0							
Locus_15402_Transcript_1/1_Conf_1.000	1057	20	7.41E-69	NP_493723.3	85	265.388	199	170	hypothetical protein C03H5.2
Locus_15403_Transcript_1/1_Conf_1.000	217	0							
Locus_15404_Transcript_1/1_Conf_1.000	194	0							
Locus_15405_Transcript_1/1_Conf_1.000	273	0							
Locus_15406_Transcript_1/1_Conf_1.000	105	0							
Locus_15407_Transcript_1/1_Conf_1.000	617	0							
Locus_15408_Transcript_1/1_Conf_1.000	214	20	6.55E-14	EFO25803.1	84	80.4925	53	45	enoyl-CoA hydratase/isomerase
Locus_15409_Transcript_1/1_Conf_1.000	313	20	1.78E-06	XP_002940157.1	56	55.8398	83	47	PREDICTED: PI-PLC X domain-containing protein 2-like
Locus_1541_Transcript_1/1_Conf_1.000	4728	20	0	NP_494819.3	57	843.958	1579	910	abnormal DYe Filling family member (dyf-14)
Locus_15410_Transcript_1/1_Conf_1.000	336	0							
Locus_15411_Transcript_1/1_Conf_1.000	250	0							
Locus_15412_Transcript_1/1_Conf_1.000	668	20	6.68E-51	NP_001041018.1	72	204.527	200	144	hypothetical protein Y11D7A.3
Locus_15413_Transcript_1/1_Conf_1.000	315	20	5.32E-19	NP_498593.1	74	97.4413	103	77	hypothetical protein R151.10
Locus_15414_Transcript_1/1_Conf_1.000	205	0							
Locus_15415_Transcript_1/2_Conf_1.000	201	1	5.63E-05	XP_002638777.1	65	50.8322	38	25	C. briggsae CBR-NAS-30 protein
Locus_15415_Transcript_2/2_Conf_1.000	336	5	5.92E-23	XP_002638777.1	71	110.538	107	76	C. briggsae CBR-NAS-30 protein

Locus_15416_Transcript_1/1_Conf_1.000	280	4	2.32E-19	CBM41243.1	68	98.5969	89	61	C. elegans protein Y39B6A.43c, partially confirmed by transcript evidence
Locus_15417_Transcript_1/1_Conf_1.000	452	0							
Locus_15418_Transcript_1/1_Conf_1.000	286	20	7.45E-13	XP_002637159.1	78	77.0258	82	64	C. briggsae CBR-AQP-6 protein
Locus_15419_Transcript_1/1_Conf_1.000	179	0							
Locus_1542_Transcript_1/3_Conf_0.556	533	20	3.37E-84	XP_002638552.1	94	314.309	170	161	C. briggsae CBR-RPL-2 protein
Locus_1542_Transcript_2/3_Conf_0.778	633	20	3.02E-87	XP_002638552.1	94	325.094	193	182	C. briggsae CBR-RPL-2 protein
Locus_1542_Transcript_3/3_Conf_0.667	633	20	3.02E-87	XP_002638552.1	94	325.094	193	182	C. briggsae CBR-RPL-2 protein
Locus_15420_Transcript_1/1_Conf_1.000	264	0							
Locus_15421_Transcript_1/1_Conf_1.000	420	0							
Locus_15422_Transcript_1/1_Conf_1.000	457	0							
Locus_15423_Transcript_1/1_Conf_1.000	151	0							
Locus_15424_Transcript_1/1_Conf_1.000	131	20	2.13E-12	XP_002641501.1	93	75.485	43	40	Hypothetical protein CBG09795
Locus_15425_Transcript_1/1_Conf_1.000	312	0							
Locus_15426_Transcript_1/1_Conf_1.000	251	3	2.03E-23	XP_002642237.1	80	112.079	83	67	Hypothetical protein CBG18219
Locus_15427_Transcript_1/1_Conf_1.000	379	0							
Locus_15428_Transcript_1/1_Conf_1.000	167	0							
Locus_15429_Transcript_1/1_Conf_1.000	310	0							
Locus_1543_Transcript_1/1_Conf_1.000	1050	20	2.03E-119	XP_002642557.1	78	433.335	343	268	Hypothetical protein CBG20194
Locus_15430_Transcript_1/1_Conf_1.000	449	10	1.58E-31	CBK55579.1	68	139.043	132	91	C. elegans protein C25G4.3b, confirmed by transcript evidence

Locus_15431_Transcript_1/1_Conf_1.000	515	19	5.14E-20	XP_002639725.1	63	100.908	146	92	Hypothetical protein CBG12452
Locus_15432_Transcript_1/1_Conf_1.000	284	20	4.66E-31	EFO15717.1	84	137.502	93	79	hypothetical protein LOAG_12793
Locus_15433_Transcript_1/1_Conf_1.000	270	3	5.23E-19	NP_501219.1	69	97.4413	81	56	hypothetical protein R05G6.1
Locus_15434_Transcript_1/1_Conf_1.000	311	20	1.18E-39	XP_002641970.1	91	166.007	92	84	Hypothetical protein CBG16677
Locus_15435_Transcript_1/2_Conf_1.000	1069	3	4.90E-60	NP_509393.3	89	236.113	157	140	hypothetical protein M60.6
Locus_15435_Transcript_2/2_Conf_1.000	239	3	3.07E-19	NP_509393.3	86	98.2117	66	57	hypothetical protein M60.6
Locus_15436_Transcript_1/1_Conf_1.000	155	0							
Locus_15437_Transcript_1/1_Conf_1.000	215	0							
Locus_15438_Transcript_1/1_Conf_1.000	807	0							
Locus_15439_Transcript_1/2_Conf_1.000	550	20	5.35E-75	NP_741437.1	88	283.878	182	161	DEnse Body family member (deb-1)
Locus_15439_Transcript_2/2_Conf_1.000	555	20	3.25E-75	NP_741437.1	88	284.648	184	162	DEnse Body family member (deb-1)
Locus_1544_Transcript_1/1_Conf_1.000	310	0							
Locus_15440_Transcript_1/1_Conf_1.000	185	0							
Locus_15441_Transcript_1/1_Conf_1.000	425	20	1.25E-44	XP_002645045.1	78	182.57	132	104	C. briggsae CBR-MEK-1 protein
Locus_15442_Transcript_1/1_Conf_1.000	150	0							
Locus_15443_Transcript_1/1_Conf_1.000	322	20	1.26E-12	NP_001024271.1	70	76.2554	71	50	CHeckpoint Kinase family member (chk-2)
Locus_15444_Transcript_1/1_Conf_1.000	138	20	5.92E-15	ADD20480.1	95	83.9593	45	43	hypothetical conserved protein
Locus_15445_Transcript_1/1_Conf_1.000	320	20	9.02E-27	NP_506082.2	75	123.25	104	79	hypothetical protein F55C5.7

Locus_15446_Transcript_1/1_Conf_1.000	664	20	2.10E-81	EAX09318.1	100	305.834	166	166	collagen, type VI, alpha 2, isoform CRA_c
Locus_15447_Transcript_1/2_Conf_1.000	328	0							
Locus_15447_Transcript_2/2_Conf_1.000	328	0							
Locus_15448_Transcript_1/1_Conf_1.000	203	20	2.69E-15	XP_002643273.1	86	85.1149	67	58	C. briggsae CBR-MRP-1 protein
Locus_15449_Transcript_1/1_Conf_1.000	497	20	6.33E-33	XP_001896024.1	68	143.665	150	102	DNA ligase III
Locus_1545_Transcript_1/1_Conf_1.000	865	20	1.18E-116	XP_002637027.1	84	423.705	257	218	Hypothetical protein CBG09520
Locus_15450_Transcript_1/1_Conf_1.000	355	0							
Locus_15451_Transcript_1/2_Conf_1.000	424	20	7.63E-10	XP_001892560.1	66	67.0106	69	46	GPI transamidase subunit PIG-U family protein
Locus_15451_Transcript_2/2_Conf_1.000	409	20	5.78E-10	XP_001892560.1	66	67.3958	69	46	GPI transamidase subunit PIG-U family protein
Locus_15452_Transcript_1/1_Conf_1.000	883	20	4.00E-27	XP_002636475.1	60	126.331	192	117	Hypothetical protein CBG23146
Locus_15453_Transcript_1/1_Conf_1.000	281	0							
Locus_15454_Transcript_1/1_Conf_1.000	157	0							
Locus_15455_Transcript_1/1_Conf_1.000	268	0							
Locus_15456_Transcript_1/1_Conf_1.000	577	20	2.78E-27	XP_002641517.1	86	125.561	79	68	Hypothetical protein CBG09813
Locus_15457_Transcript_1/1_Conf_1.000	342	20	8.42E-49	XP_002634599.1	89	196.438	112	100	Hypothetical protein CBG08414

Locus_15458_Transcript_1/1_Conf_1.000	365	20	3.92E-59	ABM84473.1	100	230.72	121	121	cathepsin D (lysosomal aspartyl peptidase)
Locus_15459_Transcript_1/1_Conf_1.000	134	0							
Locus_1546_Transcript_1/1_Conf_1.000	961	6	1.92E-49	NP_492029.2	93	200.675	117	109	hypothetical protein M05B5.2
Locus_15460_Transcript_1/1_Conf_1.000	292	0							
Locus_15461_Transcript_1/1_Conf_1.000	305	20	1.33E-22	ACO15791.1	73	109.383	100	73	Hypothetical protein Y71F9B.9b
Locus_15462_Transcript_1/1_Conf_1.000	141	0							
Locus_15463_Transcript_1/1_Conf_1.000	260	2	1.49E-29	NP_500538.2	93	132.494	86	80	Ligand-Gated ion Channel family member (lgc-22)
Locus_15464_Transcript_1/1_Conf_1.000	286	0							
Locus_15465_Transcript_1/1_Conf_1.000	194	0							
Locus_15466_Transcript_1/2_Conf_1.000	409	9	1.24E-28	NP_506123.2	74	129.413	99	74	hypothetical protein C54D10.10
Locus_15466_Transcript_2/2_Conf_1.000	416	9	1.23E-28	NP_506123.2	74	129.413	99	74	hypothetical protein C54D10.10
Locus_15467_Transcript_1/1_Conf_1.000	381	20	7.07E-16	NP_001023850.1	79	87.0409	98	78	LEThal family member (let-413)

Locus_15468_Transcript_1/1_Conf_1.000	141	20	3.24E-13	XP_002925604.1	100	78.1814	36	36	PREDICTED: receptor-type tyrosine-protein phosphatase F-like isoform 2
Locus_15469_Transcript_1/1_Conf_1.000	485	0							
Locus_1547_Transcript_1/1_Conf_1.000	550	2	1.26E-07	XP_002644421.1	64	60.077	74	48	C. briggsae CBR-NLP-1 protein
Locus_15470_Transcript_1/1_Conf_1.000	166	0							
Locus_15471_Transcript_1/1_Conf_1.000	220	0							
Locus_15472_Transcript_1/1_Conf_1.000	171	0							
Locus_15473_Transcript_1/1_Conf_1.000	224	0							
Locus_15474_Transcript_1/1_Conf_1.000	595	20	1.93E-50	NP_506382.1	93	202.601	141	132	LETM1 (Leucine zipper, EF-hand, TransMembrane mitochondrial protein) homolog family member (letm-1)
Locus_15475_Transcript_1/1_Conf_1.000	278	0							
Locus_15476_Transcript_1/1_Conf_1.000	594	1	3.11E-08	XP_002813353.1	55	62.3882	83	46	PREDICTED: hypothetical protein LOC100439541
Locus_15477_Transcript_1/1_Conf_1.000	187	2	6.53E-09	NP_504708.1	80	63.929	57	46	hypothetical protein T25F10.3
Locus_15478_Transcript_1/1_Conf_1.000	325	1	7.52E-04	AAB61392.1	59	45.4394	54	32	transposase
Locus_15479_Transcript_1/1_Conf_1.000	249	0							
Locus_1548_Transcript_1/1_Conf_1.000	1383	20	6.32E-141	NP_510669.1	86	505.368	322	277	hypothetical protein F38E9.5
Locus_15480_Transcript_1/1_Conf_1.000	265	0							
Locus_15481_Transcript_1/1_Conf_1.000	267	0							
Locus_15482_Transcript_1/1_Conf_1.000	338	3	2.66E-15	NP_497943.2	61	85.1149	116	71	hypothetical protein F10F2.4

Locus_15483_Transcript_1/1_Conf_1.000	291	20	1.06E-43	NP_499783.1	96	179.489	96	93	Cyclin-Dependent Kinase family member (cdk-5)
Locus_15484_Transcript_1/1_Conf_1.000	348	3	7.86E-31	NP_500393.2	75	136.732	115	87	hypothetical protein Y37E11AL.5
Locus_15485_Transcript_1/1_Conf_1.000	145	2	1.62E-07	XP_002642776.1	80	59.3066	46	37	C. briggsae CBR-WRS-2 protein
Locus_15486_Transcript_1/1_Conf_1.000	409	0							
Locus_15487_Transcript_1/1_Conf_1.000	1319	20	1.15E-144	XP_002636212.1	74	517.694	438	327	Hypothetical protein CBG12133
Locus_15488_Transcript_1/1_Conf_1.000	251	5	4.52E-15	XP_002629829.1	68	84.3445	83	57	C. briggsae CBR-PLC-3 protein
Locus_15489_Transcript_1/2_Conf_1.000	751	20	3.50E-68	NP_499676.1	82	262.307	177	146	hypothetical protein Y37D8A.10

Locus_15489_Transcript_2/2_Conf_1.000	573	20	1.88E-68	NP_499676.1	82	262.307	177	146	hypothetical protein Y37D8A.10
Locus_1549_Transcript_1/2_Conf_1.000	1206	20	5.20E-141	XP_002631017.1	81	505.368	402	326	Hypothetical protein CBG02769
Locus_1549_Transcript_2/2_Conf_1.000	1206	20	5.20E-141	XP_002631017.1	80	505.368	402	324	Hypothetical protein CBG02769
Locus_15490_Transcript_1/1_Conf_1.000	266	0							
Locus_15491_Transcript_1/1_Conf_1.000	302	0							
Locus_15492_Transcript_1/1_Conf_1.000	247	0							
Locus_15493_Transcript_1/1_Conf_1.000	314	0							
Locus_15494_Transcript_1/1_Conf_1.000	284	16	7.21E-24	XP_002646276.1	74	113.62	85	63	C. briggsae CBR-ABF-2 protein
Locus_15495_Transcript_1/1_Conf_1.000	1353	20	0	NP_740900.1	86	639.802	450	389	hypothetical protein T08G11.1
Locus_15496_Transcript_1/1_Conf_1.000	378	20	2.88E-25	NP_498264.1	80	118.242	82	66	Dipeptidyl Peptidase Four (IV) family member (dpf-4)
Locus_15497_Transcript_1/1_Conf_1.000	158	0							
Locus_15498_Transcript_1/1_Conf_1.000	140	20	5.92E-15	XP_001098677.2	100	83.9593	38	38	PREDICTED: serine incorporator 2
Locus_15499_Transcript_1/1_Conf_1.000	372	0							

Locus_155_Transcript_1/1_Conf_1.000	842	20	6.83E-82	XP_002636957.1	96	308.145	150	145	C. briggsae CBR-EAT-6 protein
Locus_1550_Transcript_1/1_Conf_1.000	301	15	8.94E-11	XP_002634040.1	66	70.0922	104	69	C. briggsae CBR-VIT-6 protein
Locus_15500_Transcript_1/1_Conf_1.000	419	5	1.82E-27	ACT20210.1	67	125.561	139	94	Hypothetical protein Y34B4A.8b
Locus_15501_Transcript_1/2_Conf_1.000	509	0							
Locus_15501_Transcript_2/2_Conf_1.000	509	0							
Locus_15502_Transcript_1/1_Conf_1.000	300	20	2.19E-25	XP_001891853.1	78	118.627	97	76	Patched family protein
Locus_15503_Transcript_1/1_Conf_1.000	438	20	4.50E-31	NP_001032972.1	76	137.502	98	75	hypothetical protein C36B1.14
Locus_15504_Transcript_1/1_Conf_1.000	647	0							
Locus_15505_Transcript_1/1_Conf_1.000	940	20	6.54E-103	NP_492779.1	92	378.252	240	223	hypothetical protein B0511.6

Locus_15506_Transcript_1/1_Conf_1.000	191	20	1.03E-30	CAX12872.1	100	136.346	63	63	major histocompatibility complex, class I, F
Locus_15507_Transcript_1/1_Conf_1.000	278	0							
Locus_15508_Transcript_1/1_Conf_1.000	254								
Locus_15509_Transcript_1/2_Conf_1.000	235	6	2.28E-22	EFO24879.1	76	108.612	78	60	hypothetical protein LOAG_03607
Locus_15509_Transcript_2/2_Conf_1.000	235	6	2.28E-22	EFO24879.1	76	108.612	78	60	hypothetical protein LOAG_03607
Locus_1551_Transcript_1/1_Conf_1.000	689	2	3.27E-11	XP_002637606.1	53	72.7886	116	62	Hypothetical protein CBG19344
Locus_15510_Transcript_1/1_Conf_1.000	150	0							
Locus_15511_Transcript_1/1_Conf_1.000	326	0							
Locus_15512_Transcript_1/1_Conf_1.000	371	1	1.31E-09	NP_001022291.1	58	66.2402	102	60	hypothetical protein T01B7.5
Locus_15513_Transcript_1/1_Conf_1.000	454	0							
Locus_15514_Transcript_1/1_Conf_1.000	528	20	1.50E-44	NP_493617.2	86	182.57	119	103	hypothetical protein F33H2.6
Locus_15515_Transcript_1/1_Conf_1.000	667	20	7.79E-92	BAB32114.1	92	340.502	200	185	unnamed protein product
Locus_15516_Transcript_1/1_Conf_1.000	135	0							
Locus_15517_Transcript_1/1_Conf_1.000	439	2	9.45E-05	AAX33674.1	39	50.0618	113	45	plus agglutinin
Locus_15518_Transcript_1/1_Conf_1.000	552	20	9.39E-43	NP_497829.1	84	176.792	118	100	hypothetical protein C16C10.8

Locus_15519_Transcript_1/2_Conf_1.000	702	20	7.80E-64	NP_492861.1	76	247.669	238	183	hypothetical protein Y52B11A.10
Locus_15519_Transcript_2/2_Conf_1.000	702	20	7.80E-64	NP_492861.1	76	247.669	238	183	hypothetical protein Y52B11A.10
Locus_1552_Transcript_1/1_Conf_1.000	415	0							
Locus_15520_Transcript_1/1_Conf_1.000	332	0							
Locus_15521_Transcript_1/1_Conf_1.000	671	0							
Locus_15522_Transcript_1/1_Conf_1.000	551	0							
Locus_15523_Transcript_1/1_Conf_1.000	269	6	3.39E-26	NP_502119.2	86	121.324	79	68	hypothetical protein F54D1.6
Locus_15524_Transcript_1/1_Conf_1.000	324	20	6.42E-25	NP_491660.1	79	117.087	94	75	hypothetical protein F55F8.9
Locus_15525_Transcript_1/1_Conf_1.000	138	0							
Locus_15526_Transcript_1/1_Conf_1.000	271	0							
Locus_15527_Transcript_1/1_Conf_1.000	501	0							
Locus_15528_Transcript_1/1_Conf_1.000	284	0							
Locus_15529_Transcript_1/1_Conf_1.000	391	0							
Locus_1553_Transcript_1/1_Conf_1.000	2756	20	0	NP_741743.1	93	922.924	532	495	Propionyl Coenzyme A Carboxylase Beta subunit family member (pccb-1)
Locus_15530_Transcript_1/1_Conf_1.000	285	0							
Locus_15531_Transcript_1/1_Conf_1.000	381	20	1.24E-44	XP_002640754.1	84	182.57	123	104	Hypothetical protein CBG24191
Locus_15532_Transcript_1/1_Conf_1.000	252	0							
Locus_15533_Transcript_1/1_Conf_1.000	278	20	5.18E-27	XP_002635871.1	86	124.02	88	76	Hypothetical protein CBG01087
Locus_15534_Transcript_1/1_Conf_1.000	273	0							
Locus_15535_Transcript_1/1_Conf_1.000	361	0							
Locus_15536_Transcript_1/1_Conf_1.000	220	0							

Locus_15537_Transcript_1/1_Conf_1.000	129	20	2.78E-12	EFO25124.1	90	75.0998	42	38	bromodomain containing protein
Locus_15538_Transcript_1/1_Conf_1.000	411	20	1.19E-31	XP_002631305.1	67	139.428	139	94	C. briggsae CBR-DNA-2 protein
Locus_15539_Transcript_1/1_Conf_1.000	135	0							
Locus_1554_Transcript_1/1_Conf_1.000	1072	3	5.95E-98	NP_499903.3	70	362.073	357	252	Defecation Suppressor of Clk-1 family member (dsc-4)
Locus_15540_Transcript_1/1_Conf_1.000	216	0							
Locus_15541_Transcript_1/1_Conf_1.000	636	20	1.59E-51	NP_508877.1	73	206.453	176	130	hypothetical protein T03G11.4
Locus_15542_Transcript_1/1_Conf_1.000	131	0							
Locus_15543_Transcript_1/1_Conf_1.000	151	7	5.70E-13	XP_001900069.1	85	77.411	49	42	protein C18H9.7
Locus_15544_Transcript_1/1_Conf_1.000	394	0							
Locus_15545_Transcript_1/1_Conf_1.000	479	6	1.67E-17	CBM41243.1	60	92.4337	120	72	C. elegans protein Y39B6A.43c, partially confirmed by transcript evidence
Locus_15546_Transcript_1/2_Conf_1.000	363	20	2.82E-49	NP_498923.1	93	197.978	108	101	hypothetical protein B0303.15

Locus_15546_Transcript_2/2_Conf_1.000	345	20	2.61E-50	NP_498923.1	92	201.445	113	104	hypothetical protein B0303.15
Locus_15547_Transcript_1/1_Conf_1.000	138	0							
Locus_15548_Transcript_1/1_Conf_1.000	342	0							
Locus_15549_Transcript_1/1_Conf_1.000	665	20	8.05E-57	NP_741241.1	67	224.172	221	150	Dipeptidyl Peptidase Four (IV) family member (dpf-6)
Locus_1555_Transcript_1/1_Conf_1.000	237	20	5.78E-26	NP_491399.1	85	120.553	78	67	Ribosomal Protein, Large subunit family member (rpl-24.1)
Locus_15550_Transcript_1/1_Conf_1.000	249	20	3.23E-29	NP_509693.1	85	131.339	81	69	hypothetical protein W07E11.1
Locus_15551_Transcript_1/1_Conf_1.000	669	0							
Locus_15552_Transcript_1/1_Conf_1.000	186	20	2.73E-23	NP_497696.2	91	111.694	62	57	Pim (mammalian oncogene) Related Kinase family member (prk-2)
Locus_15553_Transcript_1/1_Conf_1.000	464	20	1.05E-67	NP_505493.1	90	259.225	154	140	SULfate Permease family member (sulp-8)
Locus_15554_Transcript_1/1_Conf_1.000	149	0							
Locus_15555_Transcript_1/1_Conf_1.000	852	20	8.57E-88	EFO26794.1	75	327.791	280	211	galactoside-binding lectin family protein
Locus_15556_Transcript_1/1_Conf_1.000	405	0							
Locus_15557_Transcript_1/1_Conf_1.000	597	20	7.64E-71	NP_496968.1	82	270.396	188	155	hypothetical protein Y48B6A.12

Locus_15558_Transcript_1/2_Conf_1.000	267	0							
Locus_15558_Transcript_2/2_Conf_1.000	290	0							
Locus_15559_Transcript_1/1_Conf_1.000	741	5	5.18E-08	XP_001897464.1	51	62.3882	107	55	hypothetical protein Bm1_30125
Locus_1556_Transcript_1/1_Conf_1.000	1175	20	3.30E-68	NP_504349.1	79	263.462	216	172	hypothetical protein K11D12.5
Locus_15560_Transcript_1/1_Conf_1.000	534	20	3.76E-35	EFO27810.1	70	151.369	144	101	plasmid Maintenance protein containing protein
Locus_15561_Transcript_1/1_Conf_1.000	501	0							
Locus_15562_Transcript_1/1_Conf_1.000	289	20	3.80E-25	XP_002632959.1	77	117.857	93	72	C. briggsae CBR-RAD-26 protein
Locus_15563_Transcript_1/1_Conf_1.000	414	0							
Locus_15564_Transcript_1/1_Conf_1.000	718	20	1.74E-66	NP_001021021.1	80	256.529	189	152	hypothetical protein C32F10.8
Locus_15565_Transcript_1/1_Conf_1.000	179	0							
Locus_15566_Transcript_1/1_Conf_1.000	164	20	1.21E-15	XP_001900591.1	88	86.2705	53	47	Blistered cuticle protein 3
Locus_15567_Transcript_1/1_Conf_1.000	582	20	2.07E-09	XP_001895583.1	73	66.2402	49	36	Surfeit locus protein 4 homolog
Locus_15568_Transcript_1/1_Conf_1.000	614								

Locus_15569_Transcript_1/1_Conf_1.000	294	0							
Locus_1557_Transcript_1/1_Conf_1.000	1009	0							
Locus_15570_Transcript_1/1_Conf_1.000	195	3	3.93E-06	NP_504274.2	82	54.6842	39	32	UDP-GlucuronosylTransferase family member (ugt-61)
Locus_15571_Transcript_1/1_Conf_1.000	1578	20	1.67E-140	EFO23974.1	71	504.212	471	337	hypothetical protein LOAG_04507
Locus_15572_Transcript_1/1_Conf_1.000	338	10	5.52E-29	NP_001021303.1	69	130.568	108	75	Nuclear Hormone Receptor family member (nhr-105)
Locus_15573_Transcript_1/1_Conf_1.000	328	20	8.58E-46	XP_001111129.1	100	186.422	103	103	PREDICTED: CD63 antigen isoform 1
Locus_15574_Transcript_1/1_Conf_1.000	204	20	7.07E-16	NP_508272.1	88	87.0409	52	46	human SEDL (spondyloepiphyseal dysplasia tarda) related family member (sedl-1)
Locus_15575_Transcript_1/1_Conf_1.000	768	20	4.71E-92	NP_507487.1	84	341.658	252	214	P-GlycoProtein related family member (pgp-9)
Locus_15576_Transcript_1/1_Conf_1.000	402	20	1.09E-32	XP_002632873.1	69	142.895	132	92	Hypothetical protein CBG15077
Locus_15577_Transcript_1/1_Conf_1.000	557	6	6.25E-10	CAD44516.1	55	67.781	184	102	VAB-10B protein
Locus_15578_Transcript_1/1_Conf_1.000	167	0							
Locus_15579_Transcript_1/1_Conf_1.000	353	0							
Locus_1558_Transcript_1/1_Conf_1.000	3022	17	1.53E-21	EFO26551.1	60	110.153	157	95	hypothetical protein LOAG_01926
Locus_15580_Transcript_1/1_Conf_1.000	197	0							
Locus_15581_Transcript_1/1_Conf_1.000	160	0							
Locus_15582_Transcript_1/1_Conf_1.000	696	20	4.08E-41	XP_002640039.1	76	172.17	170	130	Hypothetical protein CBG12511
Locus_15583_Transcript_1/1_Conf_1.000	207	0							
Locus_15584_Transcript_1/1_Conf_1.000	426	0							
Locus_15585_Transcript_1/1_Conf_1.000	135	3	1.28E-09	NP_001041140.1	84	66.2402	44	37	hypothetical protein F58H1.7
Locus_15586_Transcript_1/1_Conf_1.000	302	0							

Locus_15587_Transcript_1/1_Conf_1.000	290	20	1.76E-22	XP_001897819.1	88	108.997	79	70	Surfeit locus protein 5 containing protein
Locus_15588_Transcript_1/1_Conf_1.000	270	2	9.05E-39	CAH93159.1	100	135.961	67	67	hypothetical protein
Locus_15589_Transcript_1/1_Conf_1.000	235	0							
Locus_1559_Transcript_1/1_Conf_1.000	2882	20	0	EFO24169.1	70	836.639	887	627	hypothetical protein LOAG_04317
Locus_15590_Transcript_1/1_Conf_1.000	172	0							
Locus_15591_Transcript_1/1_Conf_1.000	133	0							
Locus_15592_Transcript_1/1_Conf_1.000	222	20	6.50E-25	NP_494991.1	92	117.087	69	64	Phosphoethanolamine MethyTransferase family member (pmt-1)
Locus_15593_Transcript_1/1_Conf_1.000	196	0							
Locus_15594_Transcript_1/1_Conf_1.000	265	0							
Locus_15595_Transcript_1/1_Conf_1.000	491	20	1.56E-55	XP_002633256.1	80	218.779	155	125	Hypothetical protein CBG05981
Locus_15596_Transcript_1/1_Conf_1.000	487	0							
Locus_15597_Transcript_1/1_Conf_1.000	300	0							
Locus_15598_Transcript_1/1_Conf_1.000	638	20	1.02E-82	XP_002630211.1	86	310.071	213	184	C. briggsae CBR-FRS-2 protein
Locus_15599_Transcript_1/1_Conf_1.000	500	0							
Locus_156_Transcript_1/2_Conf_1.000	391	0							
Locus_156_Transcript_2/2_Conf_1.000	366	0							
Locus_1560_Transcript_1/1_Conf_1.000	331	0							

Locus_15600_Transcript_1/1_Conf_1.000	327	20	3.16E-40	EFO23513.1	85	167.933	108	92	CMGC/CDK/CRK7 protein kinase
Locus_15601_Transcript_1/1_Conf_1.000	173	0							
Locus_15602_Transcript_1/1_Conf_1.000	378	0							
Locus_15603_Transcript_1/1_Conf_1.000	312	0							
Locus_15604_Transcript_1/1_Conf_1.000	301	0							
Locus_15605_Transcript_1/1_Conf_1.000	184	0							
Locus_15606_Transcript_1/1_Conf_1.000	379	20	1.73E-46	P51535.1	90	188.734	113	102	Myoglobin
Locus_15607_Transcript_1/1_Conf_1.000	396	0							
Locus_15608_Transcript_1/1_Conf_1.000	509	20	1.20E-13	XP_001897610.1	67	79.7221	87	59	SNARE-associated protein Snapin
Locus_15609_Transcript_1/1_Conf_1.000	438	2	1.61E-04	XP_002067139.1	52	49.2914	110	58	GK24176
Locus_1561_Transcript_1/1_Conf_1.000	1253	20	3.52E-71	NP_495686.2	72	273.478	266	194	hypothetical protein T05H10.1
Locus_15610_Transcript_1/1_Conf_1.000	188	0							
Locus_15611_Transcript_1/1_Conf_1.000	232	20	5.83E-18	NP_499865.1	75	93.9745	79	60	hypothetical protein F29C4.6
Locus_15612_Transcript_1/1_Conf_1.000	140	0							
Locus_15613_Transcript_1/1_Conf_1.000	229	0							
Locus_15614_Transcript_1/1_Conf_1.000	398	20	4.17E-40	XP_002632119.1	77	167.548	134	104	Hypothetical protein CBG06974
Locus_15615_Transcript_1/1_Conf_1.000	921	20	1.96E-112	NP_490973.2	90	409.838	255	231	hypothetical protein F23C8.5
Locus_15616_Transcript_1/1_Conf_1.000	135	0							
Locus_15617_Transcript_1/1_Conf_1.000	343	0							
Locus_15618_Transcript_1/1_Conf_1.000	391	2	1.66E-33	NP_496432.2	69	145.591	123	86	hypothetical protein C05D12.1
Locus_15619_Transcript_1/1_Conf_1.000	553	20	1.50E-48	XP_002635580.1	70	196.052	151	106	C. briggsae CBR-PPN-1 protein

Locus_1562_Transcript_1/1_Conf_1.000	359	0								
Locus_15620_Transcript_1/1_Conf_1.000	839	0								
Locus_15621_Transcript_1/1_Conf_1.000	278	0								
Locus_15622_Transcript_1/1_Conf_1.000	282	0								
Locus_15623_Transcript_1/1_Conf_1.000	473	20	4.26E-37	XP_001894688.1	66	157.532	155	103	Protein-tyrosine phosphatase containing protein	
Locus_15624_Transcript_1/1_Conf_1.000	347	20	3.29E-53	NP_001122975.1	98	211.075	114	112	hypothetical protein K10C8.3	
Locus_15625_Transcript_1/1_Conf_1.000	326	0								
Locus_15626_Transcript_1/1_Conf_1.000	277	0								
Locus_15627_Transcript_1/1_Conf_1.000	558	0								
Locus_15628_Transcript_1/1_Conf_1.000	715									
Locus_15629_Transcript_1/1_Conf_1.000	245	0								
Locus_1563_Transcript_1/2_Conf_1.000	3044	20	1.65E-148		56	531.946	842	475	hypothetical protein H15N14.1a - Caenorhabditis elegans	
Locus_1563_Transcript_2/2_Conf_1.000	3038	20	4.94E-153		56	546.969	842	474	hypothetical protein H15N14.1a - Caenorhabditis elegans	
Locus_15630_Transcript_1/2_Conf_1.000	280	0								
Locus_15630_Transcript_2/2_Conf_1.000	280	0								
Locus_15631_Transcript_1/1_Conf_1.000	242	0								
Locus_15632_Transcript_1/2_Conf_1.000	834	20	8.20E-96	XP_002631134.1	86	354.369	229	199	Hypothetical protein CBG02918	
Locus_15632_Transcript_2/2_Conf_1.000	232	20	7.60E-26	NP_496427.1	74	120.168	95	71	hypothetical protein B0491.7	
Locus_15633_Transcript_1/1_Conf_1.000	145	20	2.58E-13	NP_499028.1	85	78.5666	48	41	NHL (ring finger b-box coiled coil) domain containing family member (nhl-1)	
Locus_15634_Transcript_1/1_Conf_1.000	282	0								
Locus_15635_Transcript_1/1_Conf_1.000	410	0								
Locus_15636_Transcript_1/2_Conf_1.000	695	20	4.12E-94	NP_493832.1	84	348.206	231	196	Acyl CoA DeHydrogenase family member (acdh-9)	
Locus_15636_Transcript_2/2_Conf_1.000	695	20	1.41E-94	NP_493832.1	84	349.747	231	196	Acyl CoA DeHydrogenase family member (acdh-9)	
Locus_15637_Transcript_1/1_Conf_1.000	385	20	1.42E-24	NP_001122663.1	76	115.931	91	70	hypothetical protein Y53F4B.39	
Locus_15638_Transcript_1/1_Conf_1.000	309	0								
Locus_15639_Transcript_1/1_Conf_1.000	542	0								

Locus_1564_Transcript_1/1_Conf_1.000	785	20	5.12E-57	XP_001899636.1	71	225.328	207	147	Chain A, Two Seven-Bladed Beta-Propeller Domains Revealed By The Structure Of A C. Elegans Homologue Of Yeast Actin Interacting Protein 1, putative
Locus_15640_Transcript_1/1_Conf_1.000	266	0							
Locus_15641_Transcript_1/1_Conf_1.000	279	0							
Locus_15642_Transcript_1/1_Conf_1.000	426	20	2.94E-62	XP_001896500.1	87	241.121	139	122	leucyl-tRNA synthetase
Locus_15643_Transcript_1/1_Conf_1.000	319	20	6.63E-54	EFO21255.1	97	213.386	106	103	hypothetical protein LOAG_07233
Locus_15644_Transcript_1/1_Conf_1.000	618	20	1.35E-44	NP_497787.1	88	183.341	109	97	Paralysed Arrest at Two-fold family member (pat-3)
Locus_15645_Transcript_1/1_Conf_1.000	527	0							
Locus_15646_Transcript_1/1_Conf_1.000	266	0							
Locus_15647_Transcript_1/1_Conf_1.000	332	8	2.78E-04	NP_001174833.1	59	48.521	54	32	Os06g0534900
Locus_15648_Transcript_1/1_Conf_1.000	290	0							
Locus_15649_Transcript_1/1_Conf_1.000	495	20	5.74E-26	XP_002646337.1	55	120.553	170	95	Hypothetical protein CBG12050
Locus_1565_Transcript_1/1_Conf_1.000	2446	20	8.87E-25	XP_001464299.1	41	120.553	806	333	kinesin K39
Locus_15650_Transcript_1/1_Conf_1.000	849	3	1.93E-23	NP_001022400.1	45	114.005	284	130	Polarity and Osmotic sensitivity Defect family member (pod-2)
Locus_15651_Transcript_1/1_Conf_1.000	568	20	8.14E-69	NP_001129905.1	80	263.462	190	152	hypothetical protein T03D8.6
Locus_15652_Transcript_1/1_Conf_1.000	200	0							
Locus_15653_Transcript_1/1_Conf_1.000	209	0							
Locus_15654_Transcript_1/1_Conf_1.000	784	20	2.91E-52	XP_002639833.1	64	209.534	219	141	C. briggsae CBR-MET-1 protein
Locus_15655_Transcript_1/1_Conf_1.000	159	0							

Locus_15656_Transcript_1/2_Conf_1.000	592	20	1.22E-73	NP_499759.1	78	279.641	198	156	DNaJ domain (prokaryotic heat shock protein) family member (dnj-17)
Locus_15656_Transcript_2/2_Conf_1.000	744	20	2.53E-95	NP_499759.1	81	352.443	248	201	DNaJ domain (prokaryotic heat shock protein) family member (dnj-17)
Locus_15657_Transcript_1/1_Conf_1.000	428	20	9.86E-34	XP_001901272.1	73	146.362	143	105	DEAD
Locus_15658_Transcript_1/1_Conf_1.000	214	0							
Locus_15659_Transcript_1/1_Conf_1.000	188	0							
Locus_1566_Transcript_1/2_Conf_1.000	979	0							
Locus_1566_Transcript_2/2_Conf_1.000	1193	0							
Locus_15660_Transcript_1/1_Conf_1.000	446	2	5.29E-11	XP_001902408.1	50	70.8626	162	81	hypothetical protein Bm1_54750
Locus_15661_Transcript_1/1_Conf_1.000	678	20	7.40E-77	XP_001892908.1	86	290.812	195	169	K+ channel tetramerisation domain containing protein
Locus_15662_Transcript_1/1_Conf_1.000	444	20	3.18E-40	XP_001899240.1	76	167.933	131	100	Tetraspanin family protein
Locus_15663_Transcript_1/1_Conf_1.000	152	20	4.82E-20	XP_002927000.1	100	100.908	50	50	PREDICTED: LOW QUALITY PROTEIN: lamin-A/C-like
Locus_15664_Transcript_1/1_Conf_1.000	515	0							
Locus_15665_Transcript_1/1_Conf_1.000	440	0							
Locus_15666_Transcript_1/1_Conf_1.000	329	0							

Locus_15667_Transcript_1/1_Conf_1.000	144	20	5.93E-18	XP_002687731.1	100	93.9745	47	47	PREDICTED: myosin, heavy chain 9, non-muscle
Locus_15668_Transcript_1/1_Conf_1.000	504	20	3.38E-53	XP_002645216.1	87	211.075	143	125	Hypothetical protein CBG00074
Locus_15669_Transcript_1/1_Conf_1.000	155	0							
Locus_1567_Transcript_1/1_Conf_1.000	255	20	2.47E-24	CAG25499.1	76	115.161	85	65	heat shock protein 20
Locus_15670_Transcript_1/1_Conf_1.000	383	6	1.13E-13	XP_002644887.1	61	79.7221	92	57	C. briggsae CBR-OPT-2 protein
Locus_15671_Transcript_1/1_Conf_1.000	167	0							
Locus_15672_Transcript_1/1_Conf_1.000	174	20	5.73E-26	NP_502576.1	100	120.553	58	58	RAB family member (rab-19)

Locus_15673_Transcript_1/1_Conf_1.000	265	20	1.26E-12	EFO23269.1	83	76.2554	53	44	vacuolar protein sorting-associated protein 45
Locus_15674_Transcript_1/1_Conf_1.000	134	0							
Locus_15675_Transcript_1/1_Conf_1.000	285	0							
Locus_15676_Transcript_1/1_Conf_1.000	735	15	4.99E-72	XP_002631027.1	78	275.018	230	181	Hypothetical protein CBG02784
Locus_15677_Transcript_1/1_Conf_1.000	183	20	1.12E-08	2G64	87	63.1586	39	34	Structure Of Caenorhabditis Elegans 6-Pyruvoyl Tetrahydropterin Synthase
Locus_15678_Transcript_1/1_Conf_1.000	421	0							
Locus_15679_Transcript_1/1_Conf_1.000	436	0							
Locus_1568_Transcript_1/1_Conf_1.000	1301	20	5.29E-94	NP_498963.1	79	349.362	254	202	Temporarily Assigned Gene name family member (tag-256)
Locus_15680_Transcript_1/1_Conf_1.000	310	0							
Locus_15681_Transcript_1/1_Conf_1.000	168	0							
Locus_15682_Transcript_1/1_Conf_1.000	381	20	1.19E-55	XP_002630466.1	92	219.164	126	116	Hypothetical protein CBG11199

Locus_15683_Transcript_1/1_Conf_1.000	253	20	1.50E-29	AAV83778.1	100	132.494	83	83	HSD48
Locus_15684_Transcript_1/1_Conf_1.000	289	2	1.60E-07	XP_002630551.1	75	59.3066	49	37	Hypothetical protein CBG12993
Locus_15685_Transcript_1/1_Conf_1.000	420	20	4.60E-47	XP_002629647.1	95	190.66	103	98	C. briggsae CBR-VHA-9 protein
Locus_15686_Transcript_1/1_Conf_1.000	149	0							
Locus_15687_Transcript_1/1_Conf_1.000	655	0							
Locus_15688_Transcript_1/1_Conf_1.000	183	0							
Locus_15689_Transcript_1/1_Conf_1.000	302	20	6.83E-19	NP_741553.1	71	97.0561	100	71	ALdehyde deHydrogenase family member (alh-4)
Locus_1569_Transcript_1/1_Conf_1.000	478	0							
Locus_15690_Transcript_1/1_Conf_1.000	339	0							
Locus_15691_Transcript_1/1_Conf_1.000	1207	14	3.37E-31	NP_504556.3	45	140.584	279	128	Chondroitin ProteoGlycan family member (cpg-4)
Locus_15692_Transcript_1/1_Conf_1.000	172	0							
Locus_15693_Transcript_1/1_Conf_1.000	800	20	1.11E-70	XP_001900427.1	70	270.781	251	176	Histone acetyltransferase type B catalytic subunit
Locus_15694_Transcript_1/1_Conf_1.000	465	5	2.62E-18	XP_001902197.1	61	95.1301	117	72	hypothetical protein Bm1_53660
Locus_15695_Transcript_1/1_Conf_1.000	203	0							
Locus_15696_Transcript_1/1_Conf_1.000	646	20	1.96E-28	NP_509836.1	56	129.798	208	117	VEGF (vascular endothelial growth factor) Receptor family member (ver-3)
Locus_15697_Transcript_1/1_Conf_1.000	843								
Locus_15698_Transcript_1/1_Conf_1.000	279	0							
Locus_15699_Transcript_1/1_Conf_1.000	295	20	8.13E-20	XP_001895480.1	65	100.138	96	63	Immunoglobulin I-set domain containing protein

Locus_157_Transcript_1/1_Conf_1.000	1611	20	2.46E-163	XP_002642099.1	88	580.096	383	339	Hypothetical protein CBG18040
Locus_1570_Transcript_1/1_Conf_1.000	574	20	1.07E-55	ACO56244.1	74	219.935	169	126	putative DEAD-box RNA helicase
Locus_15700_Transcript_1/1_Conf_1.000	198	0							
Locus_15701_Transcript_1/1_Conf_1.000	382	20	2.86E-25	XP_002638481.1	76	118.242	126	96	Hypothetical protein CBG12909
Locus_15702_Transcript_1/1_Conf_1.000	494	20	3.56E-76	BAC56579.1	100	287.345	144	144	similar to ribosomal protein S14
Locus_15703_Transcript_1/1_Conf_1.000	476	20	3.50E-07	EFA75185.1	45	58.151	91	41	Scar1
Locus_15704_Transcript_1/1_Conf_1.000	484	5	4.21E-08	XP_002737158.1	72	61.2326	69	50	PREDICTED: intracisternal A particle-promoted polypeptide-like

Locus_15705_Transcript_1/1_Conf_1.000	752	20	3.40E-55	ADI61822.1	62	219.164	240	151	endonuclease-reverse transcriptase
Locus_15706_Transcript_1/1_Conf_1.000	301	3	1.25E-12	EFO20394.1	73	76.2554	60	44	hypothetical protein LOAG_08094
Locus_15707_Transcript_1/1_Conf_1.000	230	0							
Locus_15708_Transcript_1/1_Conf_1.000	263	20	2.54E-13	NP_001123686.1	62	78.5666	79	49	inhibitor of kappa light polypeptide gene enhancer in B-cells, kinase complex-associated protein
Locus_15709_Transcript_1/1_Conf_1.000	297	20	5.60E-29	XP_001892618.1	76	130.568	98	75	Acyltransferase family protein
Locus_1571_Transcript_1/1_Conf_1.000	542	20	2.87E-70	XP_001072155.2	99	268.085	140	139	PREDICTED: histone cluster 2, H3c2-like
Locus_15710_Transcript_1/1_Conf_1.000	587	0							
Locus_15711_Transcript_1/1_Conf_1.000	266	0							
Locus_15712_Transcript_1/1_Conf_1.000	230	20	6.72E-06	AAN10061.1	65	52.373	38	25	Kunitz-like protease inhibitor precursor

Locus_15713_Transcript_1/2_Conf_1.000	372	2	3.33E-05	CAE17836.3	64	51.6026	51	33	C. elegans protein F46F2.5, partially confirmed by transcript evidence
Locus_15713_Transcript_2/2_Conf_1.000	385	0							
Locus_15714_Transcript_1/1_Conf_1.000	370	12	1.50E-13	NP_001023075.1	59	79.337	96	57	inorganic PYroPhosphatase family member (pyp-1)
Locus_15715_Transcript_1/1_Conf_1.000	841	0							
Locus_15716_Transcript_1/1_Conf_1.000	138	20	1.61E-12	EFO19409.1	94	75.8702	39	37	hypothetical protein LOAG_09084
Locus_15717_Transcript_1/1_Conf_1.000	715	20	1.28E-61	CBL87054.2	68	240.35	245	169	C. elegans protein F54E4.1, partially confirmed by transcript evidence
Locus_15718_Transcript_1/1_Conf_1.000	610	20	1.71E-28	EFO24963.1	70	129.798	121	85	hypothetical protein LOAG_03520
Locus_15719_Transcript_1/1_Conf_1.000	752	0							
Locus_1572_Transcript_1/1_Conf_1.000	784	20	4.75E-87	NP_495701.2	73	325.094	265	195	IRE1 kinase related family member (ire-1)
Locus_15720_Transcript_1/1_Conf_1.000	500	20	7.43E-42	NP_495514.1	95	173.326	92	88	LSM Sm-like protein family member (lsm-4)
Locus_15721_Transcript_1/1_Conf_1.000	706	0							

Locus_15722_Transcript_1/1_Conf_1.000	206	3	1.02E-06	NP_496984.1	87	56.6102	39	34	hypothetical protein R06A4.8
Locus_15723_Transcript_1/1_Conf_1.000	464	6	2.31E-14	EFO19081.1	76	82.0333	56	43	hypothetical protein LOAG_09412
Locus_15724_Transcript_1/1_Conf_1.000	186	0							
Locus_15725_Transcript_1/1_Conf_1.000	667	0							
Locus_15726_Transcript_1/1_Conf_1.000	360	0							
Locus_15727_Transcript_1/1_Conf_1.000	704	20	3.89E-47	XP_002635830.1	53	192.2	228	123	Hypothetical protein CBG10495
Locus_15728_Transcript_1/2_Conf_1.000	1048	20	1.38E-51	XP_002738625.1	66	207.994	227	150	PREDICTED: 5-nucleotidase domain containing 2-like
Locus_15728_Transcript_2/2_Conf_1.000	584	20	7.53E-44	NP_499552.1	88	180.644	108	96	hypothetical protein Y56A3A.18
Locus_15729_Transcript_1/1_Conf_1.000	188	0							
Locus_1573_Transcript_1/1_Conf_1.000	648	3	1.37E-45	NP_505986.1	59	186.808	222	132	hypothetical protein R13H4.5
Locus_15730_Transcript_1/1_Conf_1.000	448	20	5.62E-21	XP_002634599.1	86	103.99	59	51	Hypothetical protein CBG08414
Locus_15731_Transcript_1/1_Conf_1.000	333	1	8.33E-09	NP_741828.1	69	63.5438	65	45	hypothetical protein C54D1.7
Locus_15732_Transcript_1/1_Conf_1.000	150	20	2.39E-19	XP_002831109.1	100	98.5969	49	49	PREDICTED: LOW QUALITY PROTEIN: myosin 9-like
Locus_15733_Transcript_1/1_Conf_1.000	549	0							

Locus_15734_Transcript_1/1_Conf_1.000	724	20	8.53E-53	XP_002633447.1	66	211.075	209	140	Hypothetical protein CBG06215
Locus_15735_Transcript_1/1_Conf_1.000	395	1	7.97E-07	EFO23365.1	56	56.9954	105	59	hypothetical protein LOAG_05120
Locus_15736_Transcript_1/1_Conf_1.000	340	0							
Locus_15737_Transcript_1/1_Conf_1.000	240	0							
Locus_15738_Transcript_1/1_Conf_1.000	567	0							
Locus_15739_Transcript_1/1_Conf_1.000	176	0							
Locus_1574_Transcript_1/2_Conf_1.000	981	20	1.41E-111	CAR63577.1	85	407.142	266	227	putative Thyroglobulin type-1 repeat family protein
Locus_1574_Transcript_2/2_Conf_1.000	529	1	2.49E-23	CAR63577.1	78	112.079	111	87	putative Thyroglobulin type-1 repeat family protein
Locus_15740_Transcript_1/1_Conf_1.000	418	13	9.05E-27	NP_501395.1	67	123.25	138	93	hypothetical protein K07H8.1
Locus_15741_Transcript_1/1_Conf_1.000	255	8	7.21E-08	CAR63553.1	55	60.4622	79	44	putative major allergen
Locus_15742_Transcript_1/1_Conf_1.000	233	0							
Locus_15743_Transcript_1/1_Conf_1.000	384	20	2.12E-20	NP_001041018.1	78	102.064	70	55	hypothetical protein Y11D7A.3
Locus_15744_Transcript_1/2_Conf_1.000	862	20	1.36E-56	XP_001897063.1	64	224.172	269	174	Bromodomain containing protein
Locus_15744_Transcript_2/2_Conf_1.000	553	20	2.65E-37	XP_001897063.1	63	158.688	184	117	Bromodomain containing protein
Locus_15745_Transcript_1/1_Conf_1.000	504	20	3.29E-32	XP_002922306.1	100	141.354	70	70	PREDICTED: triosephosphate isomerase-like, partial
Locus_15746_Transcript_1/1_Conf_1.000	545	20	7.55E-26	XP_001920862.2	62	120.553	159	99	PREDICTED: zgc:172302
Locus_15747_Transcript_1/1_Conf_1.000	235	0							
Locus_15748_Transcript_1/1_Conf_1.000	356	0							

Locus_15749_Transcript_1/1_Conf_1.000	318	3	3.93E-14	XP_002635581.1	76	81.2629	56	43	Hypothetical protein CBG20567
Locus_1575_Transcript_1/1_Conf_1.000	399	20	8.43E-17	NP_500284.2	97	90.1225	43	42	Enhancer of EfL-1 mutant phenotype family member (eel-1)
Locus_15750_Transcript_1/1_Conf_1.000	231	20	9.61E-21	XP_002923753.1	100	103.219	50	50	PREDICTED: rRNA 2'-O-methyltransferase fibrillarin-like
Locus_15751_Transcript_1/1_Conf_1.000	901	5	2.29E-09	XP_001896365.1	44	67.3958	227	100	hypothetical protein Bm1_24535
Locus_15752_Transcript_1/1_Conf_1.000	188	0							
Locus_15753_Transcript_1/1_Conf_1.000	469	0							
Locus_15754_Transcript_1/1_Conf_1.000	379	20	4.59E-23	XP_001898623.1	70	110.923	96	68	Piwi domain containing protein
Locus_15755_Transcript_1/2_Conf_1.000	583	20	7.01E-58	NP_495708.1	75	227.254	180	135	hypothetical protein F10B5.2
Locus_15755_Transcript_2/2_Conf_1.000	532	20	2.69E-57	NP_495708.1	78	224.942	162	127	hypothetical protein F10B5.2
Locus_15756_Transcript_1/1_Conf_1.000	167	0							
Locus_15757_Transcript_1/2_Conf_1.000	391	20	5.90E-23	NP_504643.1	86	110.538	69	60	ACyLtransferase-like family member (acl-8)
Locus_15757_Transcript_2/2_Conf_1.000	388	20	1.27E-41	NP_504643.1	88	172.555	104	92	ACyLtransferase-like family member (acl-8)
Locus_15758_Transcript_1/1_Conf_1.000	289	0							
Locus_15759_Transcript_1/1_Conf_1.000	134	0							
Locus_1576_Transcript_1/1_Conf_1.000	936	0							
Locus_15760_Transcript_1/1_Conf_1.000	174	0							
Locus_15761_Transcript_1/2_Conf_1.000	1313	18	6.06E-21	NP_499234.1	54	106.686	243	133	Enhancer of Ksr-1 Lethality family member (ekl-6)
Locus_15761_Transcript_2/2_Conf_1.000	222	0							
Locus_15762_Transcript_1/1_Conf_1.000	305	2	7.29E-13	XP_002649028.1	67	77.0258	65	44	Hypothetical protein CBG21482
Locus_15763_Transcript_1/1_Conf_1.000	186	0							
Locus_15764_Transcript_1/1_Conf_1.000	554	20	3.53E-74	NP_001021681.1	85	281.182	183	156	hypothetical protein Y105E8A.10
Locus_15765_Transcript_1/1_Conf_1.000	209	0							
Locus_15766_Transcript_1/1_Conf_1.000	412	20	3.24E-37	XP_002641220.1	82	157.918	120	99	Hypothetical protein CBG09084
Locus_15767_Transcript_1/1_Conf_1.000	266	0							
Locus_15768_Transcript_1/2_Conf_1.000	515	0							
Locus_15768_Transcript_2/2_Conf_1.000	464	0							

Locus_15769_Transcript_1/1_Conf_1.000	483	20	1.67E-89	XP_002818294.1	100	331.643	161	161	PREDICTED: collagen alpha-2(I) chain-like
Locus_1577_Transcript_1/4_Conf_0.786	2137	20	4.05E-10	XP_001660048.1	57	71.633	100	57	hypothetical protein AaeL_AAEL009430
Locus_1577_Transcript_2/4_Conf_0.786	2121	20	1.29E-08	XP_001660048.1	55	66.6254	102	57	hypothetical protein AaeL_AAEL009430
Locus_1577_Transcript_3/4_Conf_0.214	397	20	2.64E-10	EFO16022.1	61	68.5514	80	49	hypothetical protein LOAG_12486
Locus_1577_Transcript_4/4_Conf_0.786	2115	20	4.00E-10	XP_001660048.1	57	71.633	100	57	hypothetical protein AaeL_AAEL009430
Locus_15770_Transcript_1/1_Conf_1.000	431	0							
Locus_15771_Transcript_1/1_Conf_1.000	172	7	3.74E-09	NP_491336.2	80	64.6994	50	40	NuDiX family member (ndx-7)
Locus_15772_Transcript_1/1_Conf_1.000	265	20	1.06E-11	XP_002638233.1	65	73.1738	87	57	Hypothetical protein CBG15892
Locus_15773_Transcript_1/1_Conf_1.000	183	2	1.01E-17	NP_492177.2	81	93.2041	55	45	hypothetical protein R11A5.7
Locus_15774_Transcript_1/1_Conf_1.000	570	20	2.14E-16	EFO22249.1	72	89.3521	72	52	hypothetical protein LOAG_06236
Locus_15775_Transcript_1/1_Conf_1.000	136	0							

Locus_15776_Transcript_1/1_Conf_1.000	439	20	6.31E-17	XP_002108517.1	69	90.5077	83	58	hypothetical protein TRIADDRAFT_51503
Locus_15777_Transcript_1/1_Conf_1.000	270	0							
Locus_15778_Transcript_1/1_Conf_1.000	189	0							
Locus_15779_Transcript_1/1_Conf_1.000	458	20	9.72E-42	NP_077275.1	100	172.94	91	91	pancreatic progenitor cell differentiation and proliferation factor
Locus_1578_Transcript_1/1_Conf_1.000	1339	20	1.62E-69	EFO20046.1	86	268.085	159	138	hypothetical protein LOAG_08445
Locus_15780_Transcript_1/1_Conf_1.000	163	20	4.03E-19	EFO17870.1	100	97.8265	48	48	calmodulin-like protein
Locus_15781_Transcript_1/1_Conf_1.000	212	20	2.57E-34	XP_001142642.1	100	148.288	70	70	PREDICTED: Tu translation elongation factor, mitochondrial isoform 1
Locus_15782_Transcript_1/1_Conf_1.000	467	0							
Locus_15783_Transcript_1/1_Conf_1.000	292	20	1.58E-47	BAG60492.1	100	192.2	92	92	unnamed protein product
Locus_15784_Transcript_1/1_Conf_1.000	383	20	2.25E-46	XP_002631604.1	96	188.348	96	93	Hypothetical protein CBG20785
Locus_15785_Transcript_1/1_Conf_1.000	336	20	5.16E-35	XP_002630326.1	83	150.599	104	87	Hypothetical protein CBG04253
Locus_15786_Transcript_1/1_Conf_1.000	555	20	2.35E-41	NP_001024131.1	64	172.17	187	121	Peroxisomal Membrane Protein related family member (pmp-5)
Locus_15787_Transcript_1/1_Conf_1.000	141	0							
Locus_15788_Transcript_1/1_Conf_1.000	130	20	2.87E-17	3BJ5	100	91.6633	43	43	Alternative Conformations OfThe X Region Of Human Protein Disulphide-Isomerase Modulate Exposure OfThe Substrate Binding B' Domain

Locus_15789_Transcript_1/1_Conf_1.000	637	20	3.77E-61	XP_002631214.1	86	238.424	157	136	C. briggsae CBR-MPZ-1 protein
Locus_1579_Transcript_1/1_Conf_1.000	970	20	1.49E-57	XP_002646929.1	91	227.639	129	118	Hypothetical protein CBG19634
Locus_15790_Transcript_1/1_Conf_1.000	300	20	6.15E-36	XP_002641343.1	88	153.68	99	88	C. briggsae CBR-DPY-18 protein
Locus_15791_Transcript_1/1_Conf_1.000	505	20	1.69E-20	XP_002735371.1	55	102.449	165	91	PREDICTED: hypothetical protein
Locus_15792_Transcript_1/1_Conf_1.000	279	0							
Locus_15793_Transcript_1/1_Conf_1.000	498	2	9.44E-21	XP_002632353.1	54	103.219	180	98	Hypothetical protein CBG00367
Locus_15794_Transcript_1/1_Conf_1.000	496	3	2.12E-04	XP_001639273.1	45	48.9062	70	32	predicted protein
Locus_15795_Transcript_1/1_Conf_1.000	231	0							
Locus_15796_Transcript_1/1_Conf_1.000	702	20	2.91E-18	XP_001902854.1	52	96.2857	192	100	cytoplasmic intermediate filament protein
Locus_15797_Transcript_1/1_Conf_1.000	368	20	3.09E-56	CAB05601.2	96	221.09	122	118	C. elegans protein R11A5.1b, partially confirmed by transcript evidence
Locus_15798_Transcript_1/1_Conf_1.000	147	0							
Locus_15799_Transcript_1/1_Conf_1.000	233	0							
Locus_158_Transcript_1/3_Conf_0.727	486	20	5.46E-16	XP_002641290.1	47	87.4261	147	70	Hypothetical protein CBG05204
Locus_158_Transcript_2/3_Conf_0.636	476	20	5.96E-15	XP_002641290.1	46	83.9593	147	69	Hypothetical protein CBG05204
Locus_158_Transcript_3/3_Conf_0.818	500	20	5.36E-16	XP_002641290.1	47	87.4261	147	70	Hypothetical protein CBG05204
Locus_1580_Transcript_1/2_Conf_1.000	1109	0							
Locus_1580_Transcript_2/2_Conf_1.000	1115	0							

Locus_15800_Transcript_1/2_Conf_1.000	842	20	5.85E-41	CAX51676.1	62	172.17	222	139	C. elegans protein F56A8.9, confirmed by transcript evidence
Locus_15800_Transcript_2/2_Conf_1.000	700	20	3.38E-35	CAX51676.1	68	152.525	161	110	C. elegans protein F56A8.9, confirmed by transcript evidence
Locus_15801_Transcript_1/1_Conf_1.000	256	2	9.75E-05	XP_002639508.1	58	50.0618	67	39	Hypothetical protein CBG04113
Locus_15802_Transcript_1/1_Conf_1.000	149	3	2.33E-06	AAG12204.1	68	55.4546	41	28	AF287482_5Orf122
Locus_15803_Transcript_1/1_Conf_1.000	251	0							
Locus_15804_Transcript_1/1_Conf_1.000	215	0							
Locus_15805_Transcript_1/1_Conf_1.000	139	0							
Locus_15806_Transcript_1/1_Conf_1.000	164	20	4.45E-18	Q8I0P7.3	94	94.3597	50	47	Probable 3'
Locus_15807_Transcript_1/1_Conf_1.000	329	15	1.69E-09	XP_002641343.1	67	65.855	67	45	C. briggsae CBR-DPY-18 protein
Locus_15808_Transcript_1/1_Conf_1.000	287	20	6.91E-43	ADD13547.1	96	176.792	93	90	Inositol triphosphate receptor protein 1, isoform h
Locus_15809_Transcript_1/1_Conf_1.000	238	1	1.80E-11	CAB07859.3	94	72.4034	36	34	C. elegans protein W09D10.2, partially confirmed by transcript evidence
Locus_1581_Transcript_1/1_Conf_1.000	1270	20	2.83E-177	EFO21688.1	87	625.935	422	369	phosphatidylinositol 3
Locus_15810_Transcript_1/1_Conf_1.000	815	20	9.58E-78	XP_002636833.1	72	294.278	273	197	C. briggsae CBR-PST-1 protein
Locus_15811_Transcript_1/1_Conf_1.000	239	0							

Locus_15812_Transcript_1/1_Conf_1.000	352	20	2.80E-12	XP_002812714.1	100	75.0998	35	35	PREDICTED: collagen alpha-1(III) chain-like isoform 2
Locus_15813_Transcript_1/1_Conf_1.000	355	0							
Locus_15814_Transcript_1/1_Conf_1.000	532	0							
Locus_15815_Transcript_1/1_Conf_1.000	497	20	3.36E-34	XP_002631025.1	72	147.902	122	89	Hypothetical protein CBG02782
Locus_15816_Transcript_1/1_Conf_1.000	279	0							
Locus_15817_Transcript_1/2_Conf_1.000	258	0							
Locus_15817_Transcript_2/2_Conf_1.000	252	0							
Locus_15818_Transcript_1/1_Conf_1.000	292	2	4.96E-09	NP_001032977.1	72	64.3142	50	36	hypothetical protein K02A11.4
Locus_15819_Transcript_1/1_Conf_1.000	491	20	4.01E-27	EFO20863.1	65	124.405	166	109	hypothetical protein LOAG_07626
Locus_1582_Transcript_1/1_Conf_1.000	407	0							
Locus_15820_Transcript_1/1_Conf_1.000	416	0							
Locus_15821_Transcript_1/1_Conf_1.000	474	20	1.99E-10	XP_001900444.1	45	68.9366	130	59	Calponin homology
Locus_15822_Transcript_1/1_Conf_1.000	443	20	2.81E-28	EFO20355.1	69	128.257	143	100	aminotransferase
Locus_15823_Transcript_1/2_Conf_1.000	398	20	5.61E-53	NP_493832.1	90	210.305	132	120	Acyl CoA DeHydrogenase family member (acdh-9)
Locus_15823_Transcript_2/2_Conf_1.000	398	20	5.61E-53	NP_493832.1	90	210.305	132	120	Acyl CoA DeHydrogenase family member (acdh-9)

Locus_15824_Transcript_1/1_Conf_1.000	448	20	2.66E-71	XP_002638706.1	97	271.166	144	140	Hypothetical protein CBG00285
Locus_15825_Transcript_1/1_Conf_1.000	567	0							
Locus_15826_Transcript_1/1_Conf_1.000	532	0							
Locus_15827_Transcript_1/1_Conf_1.000	381	20	1.33E-30	CAB03414.3	69	135.961	132	92	Hypothetical protein T23G11.5a
Locus_15828_Transcript_1/1_Conf_1.000	521	5	3.06E-15	XP_002636819.1	81	85.1149	55	45	C. briggsae CBR-DHS-17 protein
Locus_15829_Transcript_1/1_Conf_1.000	265	20	7.55E-42	XP_002813776.1	100	173.326	82	82	PREDICTED: guanine nucleotide-binding protein G(i) subunit alpha-2-like
Locus_1583_Transcript_1/1_Conf_1.000	398	0							
Locus_15830_Transcript_1/1_Conf_1.000	283	0							
Locus_15831_Transcript_1/1_Conf_1.000	535	0							
Locus_15832_Transcript_1/1_Conf_1.000	473	20	4.89E-25	XP_002646472.1	74	117.472	127	95	C. briggsae CBR-NSF-1 protein
Locus_15833_Transcript_1/1_Conf_1.000	178	0							
Locus_15834_Transcript_1/1_Conf_1.000	131	0							
Locus_15835_Transcript_1/1_Conf_1.000	263	11	9.35E-08	XP_002633270.1	71	60.077	74	53	C. briggsae CBR-TTR-17 protein
Locus_15836_Transcript_1/1_Conf_1.000	773	20	5.10E-78	XP_001893993.1	83	295.049	213	177	Flavoprotein
Locus_15837_Transcript_1/1_Conf_1.000	329	0							
Locus_15838_Transcript_1/1_Conf_1.000	314	20	7.68E-34	NP_492127.1	89	146.747	98	88	Low-density lipoprotein Receptor Related family member (lrp-1)
Locus_15839_Transcript_1/1_Conf_1.000	800	20	3.22E-62	XP_001900232.1	67	242.662	268	181	transportin-SR

Locus_1584_Transcript_1/1_Conf_1.000	625	0							
Locus_15840_Transcript_1/1_Conf_1.000	503	0							
Locus_15841_Transcript_1/1_Conf_1.000	196	0							
Locus_15842_Transcript_1/1_Conf_1.000	392	14	9.10E-24	EFO22879.1	67	113.235	118	80	hypothetical protein LOAG_05602
Locus_15843_Transcript_1/1_Conf_1.000	297	0							
Locus_15844_Transcript_1/1_Conf_1.000	687	20	2.38E-70	AAF81283.1	81	269.24	190	155	glutathione S-transferase
Locus_15845_Transcript_1/1_Conf_1.000	136	0							
Locus_15846_Transcript_1/1_Conf_1.000	557	4	1.47E-51	XP_002641427.1	77	206.068	188	145	Hypothetical protein CBG13296
Locus_15847_Transcript_1/1_Conf_1.000	441	20	1.94E-37	XP_002750116.1	100	158.688	93	93	PREDICTED: vimentin-like isoform 2
Locus_15848_Transcript_1/1_Conf_1.000	411	0							
Locus_15849_Transcript_1/1_Conf_1.000	187	20	3.96E-14	3KM6	100	81.2629	39	39	CrystalStructure OfThe Human Gst Pi C47sY108V DOUBLE Mutant In Complex WithThe Ethacrynic Acid-Glutathione Conjugate
Locus_1585_Transcript_1/1_Conf_1.000	2712	20	0	NP_495269.1	75	837.795	712	541	hypothetical protein C32D5.3
Locus_15850_Transcript_1/1_Conf_1.000	267	0							
Locus_15851_Transcript_1/1_Conf_1.000	112	0							
Locus_15852_Transcript_1/1_Conf_1.000	181	0							
Locus_15853_Transcript_1/1_Conf_1.000	142	0							
Locus_15854_Transcript_1/1_Conf_1.000	447	13	1.29E-41	NP_499880.3	80	172.555	136	110	hypothetical protein R02D3.3
Locus_15855_Transcript_1/1_Conf_1.000	215	0							
Locus_15856_Transcript_1/1_Conf_1.000	397	0							
Locus_15857_Transcript_1/2_Conf_1.000	167	0							
Locus_15857_Transcript_2/2_Conf_1.000	205	0							
Locus_15858_Transcript_1/1_Conf_1.000	1007	20	1.45E-151	XP_002638687.1	88	540.036	328	290	Hypothetical protein CBG11882
Locus_15859_Transcript_1/1_Conf_1.000	261	0							

Locus_1586_Transcript_1/1_Conf_1.000	453	12	2.12E-20	NP_495986.3	68	102.064	110	75	EATing: abnormal pharyngeal pumping family member (eat-3)
Locus_15860_Transcript_1/1_Conf_1.000	358	20	2.60E-10	XP_001895583.1	75	68.5514	49	37	Surfeit locus protein 4 homolog
Locus_15861_Transcript_1/1_Conf_1.000	467	20	4.00E-59	XP_002631703.1	93	230.72	125	117	Hypothetical protein CBG20902
Locus_15862_Transcript_1/1_Conf_1.000	351	0							
Locus_15863_Transcript_1/2_Conf_1.000	346	0							
Locus_15863_Transcript_2/2_Conf_1.000	343	0							
Locus_15864_Transcript_1/1_Conf_1.000	1083	20	1.82E-110	NP_504268.1	75	403.675	354	269	PeRoXisome assembly factor family member (prx-6)
Locus_15865_Transcript_1/1_Conf_1.000	145	20	4.25E-16	NP_497837.1	91	87.8113	48	44	Leucyl tRNA Synthetase family member (lrs-1)

Locus_15866_Transcript_1/1_Conf_1.000	614	20	1.19E-61	XP_001891579.1	73	239.965	205	151	ImpB/MucB/SamB family protein
Locus_15867_Transcript_1/1_Conf_1.000	194	5	3.22E-08	AAF43009.1	70	61.6178	57	40	DNAse II homolog F09G8.2
Locus_15868_Transcript_1/1_Conf_1.000	173	0							
Locus_15869_Transcript_1/1_Conf_1.000	213	20	5.01E-14	XP_002643380.1	75	80.8777	65	49	Hypothetical protein CBG15993
Locus_1587_Transcript_1/1_Conf_1.000	657	20	2.01E-100	XP_002643349.1	91	369.007	217	198	Hypothetical protein CBG15946
Locus_15870_Transcript_1/1_Conf_1.000	580	0							
Locus_15871_Transcript_1/1_Conf_1.000	334	0							
Locus_15872_Transcript_1/2_Conf_1.000	571	0							
Locus_15872_Transcript_2/2_Conf_1.000	567	0							
Locus_15873_Transcript_1/1_Conf_1.000	130	0							
Locus_15874_Transcript_1/2_Conf_1.000	366	0							
Locus_15874_Transcript_2/2_Conf_1.000	427	0							
Locus_15875_Transcript_1/1_Conf_1.000	391	20	2.48E-21	EFO22018.1	65	105.145	129	84	hypothetical protein LOAG_06465
Locus_15876_Transcript_1/1_Conf_1.000	325	3	5.11E-06	XP_002639283.1	51	54.299	111	57	C. briggsae CBR-NAS-5 protein
Locus_15877_Transcript_1/1_Conf_1.000	271	20	2.35E-11	NP_491197.2	67	72.0182	85	57	SeMaPhorin related family member (smp-2)
Locus_15878_Transcript_1/1_Conf_1.000	753	20	2.50E-58	XP_002645084.1	75	229.565	186	141	Hypothetical protein CBG16758
Locus_15879_Transcript_1/1_Conf_1.000	1170	0							
Locus_1588_Transcript_1/2_Conf_1.000	1627	20	2.40E-65	EFO13989.1	74	254.603	229	170	hypothetical protein LOAG_14538
Locus_1588_Transcript_2/2_Conf_1.000	1612	20	2.37E-65	EFO13989.1	74	254.603	229	170	hypothetical protein LOAG_14538
Locus_15880_Transcript_1/1_Conf_1.000	331	4	2.97E-14		61	81.6481	105	65	myoblast city protein homolog
Locus_15881_Transcript_1/1_Conf_1.000	200	0							

Locus_15882_Transcript_1/1_Conf_1.000	157	10	2.23E-09	XP_001902320.1	68	65.4698	50	34	Protein-tyrosine phosphatase containing protein
Locus_15883_Transcript_1/1_Conf_1.000	419	20	3.89E-62	XP_001897890.1	86	240.736	139	120	Serine/threonine protein phosphatase PP1-alpha catalytic subunit
Locus_15884_Transcript_1/1_Conf_1.000	369	20	1.59E-39	NP_497814.3	72	165.622	122	89	UNCoordinated family member (unc-79)
Locus_15885_Transcript_1/1_Conf_1.000	147	0							
Locus_15886_Transcript_1/1_Conf_1.000	368	20	2.91E-38	EFO25550.1	80	161.384	114	92	nucleolar protein 10
Locus_15887_Transcript_1/1_Conf_1.000	214	20	3.24E-21	ABJ99064.1	83	104.76	71	59	Hypothetical protein W03A3.2
Locus_15888_Transcript_1/1_Conf_1.000	328	2	1.21E-15	XP_002648567.1	58	86.2705	103	60	Hypothetical protein CBG24887
Locus_15889_Transcript_1/1_Conf_1.000	330	0							
Locus_1589_Transcript_1/1_Conf_1.000	1848	8	1.74E-06	XP_002647483.1	41	59.3066	388	161	Hypothetical protein CBG06557
Locus_15890_Transcript_1/1_Conf_1.000	3179	20	0	XP_002646198.1	64	877.085	1095	710	Hypothetical protein CBG04359
Locus_15891_Transcript_1/2_Conf_1.000	309	0							
Locus_15891_Transcript_2/2_Conf_1.000	309	0							
Locus_15892_Transcript_1/1_Conf_1.000	616	20	2.86E-71	XP_002629988.1	80	271.937	206	166	C. briggsae CBR-NRF-6 protein
Locus_15893_Transcript_1/1_Conf_1.000	171	0							
Locus_15894_Transcript_1/1_Conf_1.000	280	20	1.05E-11	CBW48338.1	61	73.1738	93	57	Hypothetical protein C27A7.1d
Locus_15895_Transcript_1/1_Conf_1.000	161	0							
Locus_15896_Transcript_1/1_Conf_1.000	799	20	5.56E-22	BAB68205.1	68	108.997	117	80	keratin-like protein
Locus_15897_Transcript_1/1_Conf_1.000	145	0							
Locus_15898_Transcript_1/1_Conf_1.000	141	0							
Locus_15899_Transcript_1/1_Conf_1.000	239	0							

Locus_159_Transcript_1/1_Conf_1.000	1797	20	0	XP_002641211.1	91	829.706	538	493	C. briggsae CBR-CCT-6 protein
Locus_1590_Transcript_1/1_Conf_1.000	826	20	9.23E-76	NP_492339.1	88	287.73	184	163	CalPoNin family member (cpn-1)
Locus_15900_Transcript_1/1_Conf_1.000	450	0							
Locus_15901_Transcript_1/1_Conf_1.000	250	20	2.24E-22	XP_001892089.1	81	108.612	79	64	S-antigen protein precursor
Locus_15902_Transcript_1/1_Conf_1.000	458	20	7.39E-66	ACI49151.1	90	253.062	151	137	hypothetical protein Cbre_JD20.006
Locus_15903_Transcript_1/1_Conf_1.000	300	20	1.52E-18	NP_493981.1	77	95.9005	70	54	hypothetical protein H17B01.1
Locus_15904_Transcript_1/1_Conf_1.000	135	0							
Locus_15905_Transcript_1/1_Conf_1.000	381	0							
Locus_15906_Transcript_1/1_Conf_1.000	150	0							
Locus_15907_Transcript_1/1_Conf_1.000	461	0							
Locus_15908_Transcript_1/1_Conf_1.000	420	2	4.06E-11	NP_491994.2	70	71.2478	62	44	hypothetical protein H06O01.2
Locus_15909_Transcript_1/1_Conf_1.000	559	20	8.26E-71	NP_499212.1	89	270.011	183	164	Masculinisation Of Germline family member (mog-1)
Locus_1591_Transcript_1/1_Conf_1.000	628	20	1.33E-55	XP_002630918.1	89	219.935	155	138	Hypothetical protein CBG02643

Locus_15910_Transcript_1/1_Conf_1.000	278	20	2.49E-29	XP_001892891.1	82	131.724	92	76	cullin homolog 3
Locus_15911_Transcript_1/1_Conf_1.000	224	20	2.10E-07	XP_539278.2	74	58.9214	50	37	PREDICTED: similar to 2-aminoadipic 6-semialdehyde dehydrogenase isoform 1
Locus_15912_Transcript_1/1_Conf_1.000	182	4	1.37E-22	NP_503059.2	93	109.383	60	56	hypothetical protein ZK550.3
Locus_15913_Transcript_1/1_Conf_1.000	392	20	1.34E-59	EFO27533.1	90	232.261	130	117	hypothetical protein LOAG_00947
Locus_15914_Transcript_1/1_Conf_1.000	222	20	2.46E-32	XP_002634980.1	97	141.739	74	72	C. briggsae CBR-ARK-1 protein
Locus_15915_Transcript_1/1_Conf_1.000	310	20	5.17E-19	NP_497751.1	69	97.4413	102	71	hypothetical protein C14B1.10
Locus_15916_Transcript_1/1_Conf_1.000	561	20	1.22E-53	XP_002640778.1	85	213.001	171	147	C. briggsae CBR-FRL-1 protein

Locus_15917_Transcript_1/1_Conf_1.000	551	20	2.13E-31	AAO63576.1	58	139.043	179	105	secreted protein 4 precursor
Locus_15918_Transcript_1/1_Conf_1.000	138	20	1.97E-10	NP_505688.1	82	68.9366	46	38	Cdc-42 Related Protein family member (crp-1)
Locus_15919_Transcript_1/1_Conf_1.000	782	4	9.06E-22	NP_001022206.1	48	108.227	251	122	hypothetical protein F56D1.2
Locus_1592_Transcript_1/1_Conf_1.000	716	20	1.72E-74	XP_002649016.1	82	283.108	207	171	Hypothetical protein CBG21464
Locus_15920_Transcript_1/1_Conf_1.000	303	12	4.14E-08	BAB68205.1	69	61.2326	66	46	keratin-like protein
Locus_15921_Transcript_1/1_Conf_1.000	177	0							
Locus_15922_Transcript_1/1_Conf_1.000	139	0							
Locus_15923_Transcript_1/1_Conf_1.000	253	0							
Locus_15924_Transcript_1/1_Conf_1.000	151	4	2.17E-04	XP_002755675.1	98	48.9062	50	49	PREDICTED: uncharacterized protein C11orf24-like
Locus_15925_Transcript_1/1_Conf_1.000	305	0							
Locus_15926_Transcript_1/1_Conf_1.000	453	0							
Locus_15927_Transcript_1/1_Conf_1.000	1028	20	5.36E-16	XP_002158612.1	53	89.7373	137	73	PREDICTED: hypothetical protein, partial
Locus_15928_Transcript_1/1_Conf_1.000	287	2	3.57E-15	XP_001897687.1	68	84.7297	85	58	Zinc finger, C2H2 type family protein
Locus_15929_Transcript_1/1_Conf_1.000	312	0							
Locus_1593_Transcript_1/1_Conf_1.000	261	0							
Locus_15930_Transcript_1/1_Conf_1.000	502	3	4.50E-18	NP_497077.2	85	94.3597	69	59	hypothetical protein R05H10.2
Locus_15931_Transcript_1/1_Conf_1.000	192	5	3.80E-17	XP_002636415.1	81	91.2781	60	49	Hypothetical protein CBG23068
Locus_15932_Transcript_1/1_Conf_1.000	361	20	7.46E-42	NP_491219.1	89	173.326	119	106	hypothetical protein C32E8.3
Locus_15933_Transcript_1/1_Conf_1.000	262	4	6.05E-15	XP_002646198.1	80	83.9593	67	54	Hypothetical protein CBG04359
Locus_15934_Transcript_1/1_Conf_1.000	452	20	3.48E-47	NP_498398.1	77	191.045	150	116	LEThal family member (let-716)

Locus_15935_Transcript_1/1_Conf_1.000	587	3	8.25E-14	XP_002640845.1	48	80.8777	211	103	C. briggsae CBR-CDH-1 protein
Locus_15936_Transcript_1/1_Conf_1.000	233	0							
Locus_15937_Transcript_1/1_Conf_1.000	135	0							
Locus_15938_Transcript_1/1_Conf_1.000	443	0							
Locus_15939_Transcript_1/1_Conf_1.000	217	0							
Locus_1594_Transcript_1/2_Conf_1.000	812	20	6.55E-95	NP_741371.2	92	351.288	234	217	Ribosomal Protein, Large subunit family member (rpl-7A)
Locus_1594_Transcript_2/2_Conf_1.000	812	20	6.55E-95	NP_741371.2	92	351.288	234	217	Ribosomal Protein, Large subunit family member (rpl-7A)
Locus_15940_Transcript_1/2_Conf_1.000	587	3	2.24E-27	NP_508028.2	89	125.946	69	62	hypothetical protein F31D4.9
Locus_15940_Transcript_2/2_Conf_1.000	587	3	2.24E-27	NP_508028.2	89	125.946	69	62	hypothetical protein F31D4.9
Locus_15941_Transcript_1/1_Conf_1.000	363	0							
Locus_15942_Transcript_1/1_Conf_1.000	160	20	3.54E-15	EFO21962.1	87	84.7297	48	42	TK/EGFR protein kinase
Locus_15943_Transcript_1/1_Conf_1.000	421	2	2.55E-13	NP_491418.1	75	78.5666	66	50	hypothetical protein B0041.5
Locus_15944_Transcript_1/1_Conf_1.000	1391	20	1.05E-127	XP_002647378.1	96	461.455	300	288	C. briggsae CBR-TTB-1 protein
Locus_15945_Transcript_1/1_Conf_1.000	459	20	1.14E-50	XP_002643561.1	83	202.601	156	131	Hypothetical protein CBG16259
Locus_15946_Transcript_1/1_Conf_1.000	235	0							

Locus_15947_Transcript_1/1_Conf_1.000	421	20	4.92E-41	NP_491082.2	92	170.629	96	89	hypothetical protein Y71F9AR.2
Locus_15948_Transcript_1/1_Conf_1.000	784	0							
Locus_15949_Transcript_1/1_Conf_1.000	623	20	3.00E-52	EFO17715.1	72	208.764	193	140	oxidoreductase
Locus_1595_Transcript_1/2_Conf_1.000	1643	20	1.37E-76	XP_002636911.1	61	291.967	345	213	Hypothetical protein CBG09375
Locus_1595_Transcript_2/2_Conf_1.000	1583	20	1.26E-79	NP_504461.1	67	301.982	325	218	hypothetical protein C24G6.8
Locus_15950_Transcript_1/1_Conf_1.000	371	0							
Locus_15951_Transcript_1/1_Conf_1.000	635	20	4.08E-92	XP_002642342.1	91	341.273	210	192	Hypothetical protein CBG18338
Locus_15952_Transcript_1/1_Conf_1.000	321	2	1.18E-18	NP_504827.2	64	96.2857	102	66	hypothetical protein F09G2.1
Locus_15953_Transcript_1/1_Conf_1.000	196	0							
Locus_15954_Transcript_1/1_Conf_1.000	410	0							
Locus_15955_Transcript_1/1_Conf_1.000	150	0							
Locus_15956_Transcript_1/1_Conf_1.000	421	0							
Locus_15957_Transcript_1/1_Conf_1.000	289	20	5.44E-48	XP_002816866.1	100	193.741	96	96	PREDICTED: 60S ribosomal protein L10a-like
Locus_15958_Transcript_1/1_Conf_1.000	171	0							
Locus_15959_Transcript_1/1_Conf_1.000	482	6	3.24E-13	XP_001900333.1	53	78.1814	145	77	Anaphase promoting complex subunit 8 / cdc23 family protein
Locus_1596_Transcript_1/1_Conf_1.000	3277	20	0	NP_509658.1	75	1268.06	1107	841	Multidrug Resistance Protein family member (mrp-4)

Locus_15960_Transcript_1/1_Conf_1.000	308	2	1.37E-11	XP_001899300.1	62	72.7886	83	52	Negative elongation factor B homolog
Locus_15961_Transcript_1/1_Conf_1.000	151	0							
Locus_15962_Transcript_1/1_Conf_1.000	431	20	6.57E-38	XP_002636980.1	78	160.229	119	93	Hypothetical protein CBG09463
Locus_15963_Transcript_1/1_Conf_1.000	301	0							
Locus_15964_Transcript_1/1_Conf_1.000	224	20	3.03E-06	XP_001635789.1	60	55.0694	70	42	predicted protein
Locus_15965_Transcript_1/1_Conf_1.000	567	3	1.16E-06	XP_002642235.1	51	56.9954	74	38	C. briggsae CBR-BRD-1 protein
Locus_15966_Transcript_1/1_Conf_1.000	352	0							
Locus_15967_Transcript_1/1_Conf_1.000	569	20	9.07E-44	EFO17782.1	72	180.259	128	93	hypothetical protein LOAG_10716
Locus_15968_Transcript_1/1_Conf_1.000	241	0							
Locus_15969_Transcript_1/1_Conf_1.000	656	20	3.47E-20	NP_001123073.1	50	102.449	204	104	hypothetical protein ZC250.5
Locus_1597_Transcript_1/1_Conf_1.000	227	20	5.87E-18	NP_491756.2	76	93.9745	72	55	hypothetical protein ZK484.6
Locus_15970_Transcript_1/1_Conf_1.000	370	20	1.48E-61	XP_002634817.1	95	238.81	123	118	C. briggsae CBR-CLP-7 protein
Locus_15971_Transcript_1/1_Conf_1.000	144	0							
Locus_15972_Transcript_1/1_Conf_1.000	150	0							

Locus_15973_Transcript_1/1_Conf_1.000	186	20	7.68E-26	NP_001132929.1	100	120.168	62	62	B-cell receptor-associated protein 31 isoform a
Locus_15974_Transcript_1/1_Conf_1.000	370	0							
Locus_15975_Transcript_1/1_Conf_1.000	213	0							
Locus_15976_Transcript_1/1_Conf_1.000	243	0							
Locus_15977_Transcript_1/1_Conf_1.000	170	0							
Locus_15978_Transcript_1/1_Conf_1.000	150	0							
Locus_15979_Transcript_1/1_Conf_1.000	199	0							
Locus_1598_Transcript_1/1_Conf_1.000	1524	20	4.39E-122	XP_002639175.1	78	442.965	385	303	C. briggsae CBR-ILE-1 protein
Locus_15980_Transcript_1/1_Conf_1.000	379	20	2.51E-37	XP_002632404.1	78	158.303	125	98	Hypothetical protein CBG00428
Locus_15981_Transcript_1/1_Conf_1.000	181	0							
Locus_15982_Transcript_1/1_Conf_1.000	379	20	8.54E-70	XP_002927427.1	100	266.159	126	126	PREDICTED: transcription elongation factor SPT5-like isoform 2

Locus_15983_Transcript_1/1_Conf_1.000	170	20	1.68E-25	XP_002811271.1	100	119.013	56	56	PREDICTED: nuclear migration protein nudC-like
Locus_15984_Transcript_1/1_Conf_1.000	194	0							
Locus_15985_Transcript_1/2_Conf_1.000	611	0							
Locus_15985_Transcript_2/2_Conf_1.000	592	0							
Locus_15986_Transcript_1/1_Conf_1.000	995	11	1.86E-18	NP_492646.1	51	97.8265	180	93	Feminization Of Germline family member (fog-3)
Locus_15987_Transcript_1/1_Conf_1.000	280	0							
Locus_15988_Transcript_1/1_Conf_1.000	214	0							
Locus_15989_Transcript_1/1_Conf_1.000	152	0							
Locus_1599_Transcript_1/1_Conf_1.000	1238	20	1.47E-146	EFO21722.1	75	523.857	413	312	A-macroglobulin complement component family protein
Locus_15990_Transcript_1/1_Conf_1.000	238	20	8.93E-19	XP_002637127.1	85	96.6709	78	67	Hypothetical protein CBG09629
Locus_15991_Transcript_1/1_Conf_1.000	171	20	2.87E-09	NP_497334.1	71	65.0846	56	40	INverted Formin/formin Three-related family member (inft-1)
Locus_15992_Transcript_1/1_Conf_1.000	210	0							
Locus_15993_Transcript_1/1_Conf_1.000	303	2	2.05E-15	EFO18627.1	77	85.5001	88	68	hypothetical protein LOAG_09870
Locus_15994_Transcript_1/1_Conf_1.000	171	0							
Locus_15995_Transcript_1/1_Conf_1.000	562	0							
Locus_15996_Transcript_1/1_Conf_1.000	256	20	1.88E-32	NP_493778.1	83	142.124	85	71	hypothetical protein K07E8.7
Locus_15997_Transcript_1/1_Conf_1.000	288	0							
Locus_15998_Transcript_1/1_Conf_1.000	294	0							
Locus_15999_Transcript_1/1_Conf_1.000	514	4	2.32E-04	XP_002647225.1	43	48.9062	162	71	Hypothetical protein CBG23815
Locus_16_Transcript_1/2_Conf_1.000	549	2	4.94E-04	AAD13339.1	52	48.1358	65	34	ancylostoma-secreted protein 1 precursor
Locus_16_Transcript_2/2_Conf_1.000	225	2	6.30E-04	AAN11402.1	55	47.3654	54	30	secreted-protein 1 precursor
Locus_160_Transcript_1/5_Conf_0.133	609	20	4.80E-31	NP_001122684.1	96	138.272	95	92	UNCoordinated family member (unc-103)
Locus_160_Transcript_2/5_Conf_0.467	537	20	3.36E-31	AAO63577.1	60	138.272	160	97	secreted protein 5 precursor
Locus_160_Transcript_3/5_Conf_0.600	740	20	8.11E-38	AAO63577.1	59	161.384	192	115	secreted protein 5 precursor

Locus_160_Transcript_4/5_Conf_0.733	791	20	2.43E-38	AAO63577.1	60	163.31	192	116	secreted protein 5 precursor
Locus_160_Transcript_5/5_Conf_0.733	740	20	2.79E-38	AAO63577.1	59	162.925	192	115	secreted protein 5 precursor
Locus_1600_Transcript_1/1_Conf_1.000	2966	20	4.13E-96	NP_490758.1	46	357.836	921	431	Synthetic secondary siRNA-deficient ArGOnaute mutant family member (sago-2)
Locus_16000_Transcript_1/1_Conf_1.000	326	0							
Locus_16001_Transcript_1/1_Conf_1.000	239	20	1.05E-27	NP_501689.1	82	126.331	79	65	fatty acid ELOngation family member (elo-1)
Locus_16002_Transcript_1/1_Conf_1.000	342	0							
Locus_16003_Transcript_1/1_Conf_1.000	350	0							
Locus_16004_Transcript_1/1_Conf_1.000	561	20	9.00E-65	AAN11401.1	78	249.98	184	145	metalloprotease 1 precursor
Locus_16005_Transcript_1/1_Conf_1.000	128	0							
Locus_16006_Transcript_1/1_Conf_1.000	1297	20	0	NP_506128.1	95	787.719	412	393	AcTiniN family member (atn-1)

Locus_16007_Transcript_1/1_Conf_1.000	511	20	2.95E-12	XP_001182427.1	54	75.0998	111	60	PREDICTED: similar to mannose receptor, partial
Locus_16008_Transcript_1/1_Conf_1.000	287	0							
Locus_16009_Transcript_1/1_Conf_1.000	145	0							
Locus_1601_Transcript_1/1_Conf_1.000	1644	5	4.07E-20	XP_002640451.1	45	104.375	320	144	Hypothetical protein CBG08508
Locus_16010_Transcript_1/1_Conf_1.000	695	5	1.11E-14	EFO21657.1	52	84.3445	161	85	hypothetical protein LOAG_06828
Locus_16011_Transcript_1/1_Conf_1.000	211	20	1.24E-20	NP_497751.1	81	102.834	70	57	hypothetical protein C14B1.10
Locus_16012_Transcript_1/1_Conf_1.000	269	20	5.96E-39	EFO21248.1	93	163.696	89	83	CMGC/CDK/CDK5 protein kinase
Locus_16013_Transcript_1/1_Conf_1.000	330	20	1.33E-06	XP_001764884.1	57	56.225	92	53	predicted protein
Locus_16014_Transcript_1/1_Conf_1.000	598	20	1.62E-12	XP_001896971.1	64	76.6406	102	66	hypothetical protein Bm1_27600
Locus_16015_Transcript_1/1_Conf_1.000	515	20	3.43E-40	NP_509417.2	81	167.933	118	96	hypothetical protein F40B5.2
Locus_16016_Transcript_1/1_Conf_1.000	734	20	1.36E-29	CAB07368.3	53	134.035	258	138	C. elegans protein F22G12.4, confirmed by transcript evidence

Locus_16017_Transcript_1/1_Conf_1.000	750	20	5.17E-104	NP_492752.1	93	381.333	218	203	hypothetical protein F59C6.5
Locus_16018_Transcript_1/1_Conf_1.000	1576	4	1.12E-27	XP_002641592.1	52	129.413	288	151	Hypothetical protein CBG09896
Locus_16019_Transcript_1/1_Conf_1.000	173	0							
Locus_1602_Transcript_1/1_Conf_1.000	580	20	5.34E-26	XP_001899692.1	62	121.324	135	85	WW domain containing protein
Locus_16020_Transcript_1/1_Conf_1.000	287	20	2.82E-28	NP_001022571.1	94	128.257	69	65	NeuroFibroMatosis homolog family member (nfm-1)
Locus_16021_Transcript_1/1_Conf_1.000	478	0							
Locus_16022_Transcript_1/1_Conf_1.000	485	20	3.08E-51	XP_002633387.1	81	204.527	149	121	C. briggsae CBR-TWK-25 protein
Locus_16023_Transcript_1/1_Conf_1.000	147	0							
Locus_16024_Transcript_1/1_Conf_1.000	586	0							
Locus_16025_Transcript_1/1_Conf_1.000	590	20	1.39E-29	NP_001122554.1	61	133.265	198	121	hypothetical protein Y71G12A.2
Locus_16026_Transcript_1/1_Conf_1.000	423	12	1.22E-07	XP_001744572.1	48	59.6918	117	57	hypothetical protein
Locus_16027_Transcript_1/1_Conf_1.000	187	2	2.84E-04	EFO23538.1	63	48.521	60	38	hypothetical protein LOAG_04945
Locus_16028_Transcript_1/1_Conf_1.000	317	5	8.21E-20	XP_002643599.1	65	100.138	113	74	C. briggsae CBR-HDA-4 protein
Locus_16029_Transcript_1/1_Conf_1.000	379	0							
Locus_1603_Transcript_1/1_Conf_1.000	656	20	1.78E-16	XP_002634226.1	53	90.1225	143	77	Hypothetical protein CBG01796
Locus_16030_Transcript_1/1_Conf_1.000	225	0							
Locus_16031_Transcript_1/1_Conf_1.000	670	20	2.03E-55	NP_001024971.1	74	219.55	224	167	abnormal cell MIGration family member (mig-15)
Locus_16032_Transcript_1/1_Conf_1.000	277	2	1.92E-05	EFO19241.1	52	52.373	88	46	hypothetical protein LOAG_09252
Locus_16033_Transcript_1/1_Conf_1.000	411	4	1.97E-18	XP_002633725.1	53	95.5153	142	76	Hypothetical protein CBG03410
Locus_16034_Transcript_1/1_Conf_1.000	270	0							

Locus_16035_Transcript_1/1_Conf_1.000	136	20	6.15E-12	EFO13686.1	86	73.9442	44	38	CAMK/TSSK protein kinase
Locus_16036_Transcript_1/1_Conf_1.000	258	20	2.22E-25	XP_001902207.1	92	118.627	76	70	Hypothetical GTP-binding protein C02F5.3 in chromosome III, putative
Locus_16037_Transcript_1/1_Conf_1.000	860	0							
Locus_16038_Transcript_1/1_Conf_1.000	476	20	9.52E-13	CAA84693.3	64	76.6406	88	57	C. elegans protein D2045.2, partially confirmed by transcript evidence
Locus_16039_Transcript_1/1_Conf_1.000	1002	20	5.14E-141	XP_002629752.1	90	504.982	334	302	Hypothetical protein CBG00986
Locus_1604_Transcript_1/1_Conf_1.000	350	20	3.51E-47	XP_002630779.1	88	191.045	113	100	Hypothetical protein CBG02475
Locus_16040_Transcript_1/1_Conf_1.000	130	0							
Locus_16041_Transcript_1/1_Conf_1.000	417	20	4.49E-26	NP_508948.2	83	120.939	96	80	hypothetical protein T21F4.1
Locus_16042_Transcript_1/1_Conf_1.000	295	1	1.44E-08	CAL22705.2	62	62.7734	93	58	C. elegans protein K03H1.13, partially confirmed by transcript evidence
Locus_16043_Transcript_1/1_Conf_1.000	479	0							
Locus_16044_Transcript_1/1_Conf_1.000	560	0							
Locus_16045_Transcript_1/1_Conf_1.000	291	20	1.09E-48	XP_001110023.2	100	196.052	97	97	PREDICTED: sentrin-specific protease 3-like isoform 3
Locus_16046_Transcript_1/1_Conf_1.000	247	10	4.38E-26	XP_002633733.1	87	120.939	78	68	Hypothetical protein CBG03418
Locus_16047_Transcript_1/2_Conf_1.000	643	6	1.58E-38	NP_509272.1	66	163.31	204	136	hypothetical protein F46C8.3
Locus_16047_Transcript_2/2_Conf_1.000	788	6	1.91E-35	NP_509272.1	68	153.68	175	120	hypothetical protein F46C8.3
Locus_16048_Transcript_1/1_Conf_1.000	545	17	4.60E-07	XP_396560.3	50	58.151	106	53	PREDICTED: similar to CG1402-PA
Locus_16049_Transcript_1/1_Conf_1.000	168	0							

Locus_1605_Transcript_1/1_Conf_1.000	719	20	2.44E-44	NP_494841.3	63	182.956	174	111	hypothetical protein F10G7.10
Locus_16050_Transcript_1/1_Conf_1.000	783	20	2.39E-38	NP_001022548.1	58	163.31	210	123	hypothetical protein F25B5.6
Locus_16051_Transcript_1/2_Conf_1.000	759	20	6.87E-88	ACY39993.1	76	327.791	252	192	C. elegans FLN-2 protein, isoform a, partially confirmed by transcript evidence
Locus_16051_Transcript_2/2_Conf_1.000	443	20	7.37E-37	ACY39994.1	90	156.762	92	83	C. elegans FLN-2 protein, isoform d
Locus_16052_Transcript_1/1_Conf_1.000	291	0							
Locus_16053_Transcript_1/1_Conf_1.000	396	0							
Locus_16054_Transcript_1/1_Conf_1.000	469	20	9.14E-72	NP_000032.1	100	272.707	137	137	apolipoprotein E precursor
Locus_16055_Transcript_1/1_Conf_1.000	310	19	1.32E-22	XP_002646997.1	72	109.383	97	70	Hypothetical protein CBG22453
Locus_16056_Transcript_1/1_Conf_1.000	456	3	2.26E-06	XP_001893605.1	43	55.4546	150	65	Trypsin family protein
Locus_16057_Transcript_1/1_Conf_1.000	281	0							
Locus_16058_Transcript_1/1_Conf_1.000	312	20	2.25E-09	NP_500767.1	55	65.4698	95	53	hypothetical protein F36H12.3
Locus_16059_Transcript_1/1_Conf_1.000	365	0							
Locus_1606_Transcript_1/2_Conf_1.000	1071	20	3.18E-67	XP_002640805.1	65	259.996	264	173	Hypothetical protein CBG15687

Locus_1606_Transcript_2/2_Conf_1.000	987	20	5.46E-71	NP_497690.1	68	272.322	263	180	hypothetical protein Y53G8AR.9
Locus_16060_Transcript_1/1_Conf_1.000	1121	20	4.50E-152	XP_002639404.1	85	541.962	370	317	Hypothetical protein CBG03992
Locus_16061_Transcript_1/1_Conf_1.000	199	0							
Locus_16062_Transcript_1/1_Conf_1.000	174	0							
Locus_16063_Transcript_1/1_Conf_1.000	253	0							
Locus_16064_Transcript_1/1_Conf_1.000	382	2	5.80E-10	NP_491209.3	89	67.3958	39	35	LARP (RNA binding La related protein) homolog family member (larp-5)
Locus_16065_Transcript_1/1_Conf_1.000	212	0							
Locus_16066_Transcript_1/1_Conf_1.000	220	4	1.23E-07	NP_509283.1	82	59.6918	41	34	APYrase family member (apy-1)
Locus_16067_Transcript_1/1_Conf_1.000	220	0							
Locus_16068_Transcript_1/1_Conf_1.000	212	0							
Locus_16069_Transcript_1/1_Conf_1.000	316	0							
Locus_1607_Transcript_1/3_Conf_0.714	3501	20	1.99E-145	NP_001022562.1	50	521.931	1152	585	Suppressor/Enhancer of Lin-12 family member (sel-5)
Locus_1607_Transcript_2/3_Conf_0.714	3411	20	1.58E-147	NP_001022562.1	50	528.865	1145	578	Suppressor/Enhancer of Lin-12 family member (sel-5)
Locus_1607_Transcript_3/3_Conf_0.714	3417	20	6.03E-147	NP_001022562.1	50	526.939	1147	580	Suppressor/Enhancer of Lin-12 family member (sel-5)
Locus_16070_Transcript_1/1_Conf_1.000	217	20	1.83E-24	NP_005006.3	100	115.546	72	72	mitochondrial inner membrane protein OXA1L
Locus_16071_Transcript_1/1_Conf_1.000	147	0							
Locus_16072_Transcript_1/1_Conf_1.000	287	0							
Locus_16073_Transcript_1/1_Conf_1.000	247	20	1.61E-28	AAL06641.1	100	129.028	82	82	serine-threonine protein kinase

Locus_16074_Transcript_1/1_Conf_1.000	197	2	5.85E-10	NP_506090.1	97	67.3958	38	37	hypothetical protein R04F11.2
Locus_16075_Transcript_1/1_Conf_1.000	268	20	3.40E-18	XP_002636870.1	80	94.7449	71	57	Hypothetical protein CBG09328
Locus_16076_Transcript_1/1_Conf_1.000	1189	20	1.49E-108	EDL03932.1	97	397.512	206	200	mCG15678, isoform CRA_c
Locus_16077_Transcript_1/1_Conf_1.000	598	20	5.88E-55	XP_002639150.1	78	217.624	188	147	C. briggsae CBR-ROM-5 protein
Locus_16078_Transcript_1/1_Conf_1.000	317	3	4.96E-33	XP_002645845.1	82	144.05	104	86	Hypothetical protein CBG07579
Locus_16079_Transcript_1/1_Conf_1.000	511	2	4.71E-10	NP_505787.1	65	67.781	101	66	hypothetical protein F58B4.4
Locus_1608_Transcript_1/1_Conf_1.000	350	3	2.30E-06	NP_491341.1	63	55.4546	63	40	MUS (Drosophila mutagen sensitive) related family member (mus-81)
Locus_16080_Transcript_1/1_Conf_1.000	483	20	1.34E-46	NP_001020956.1	86	189.119	124	107	related to yeast Vacuolar Protein Sorting factor family member (vps-34)
Locus_16081_Transcript_1/1_Conf_1.000	254	0							
Locus_16082_Transcript_1/1_Conf_1.000	236	0							
Locus_16083_Transcript_1/1_Conf_1.000	267	20	2.52E-21	CAA72916.2	73	105.145	88	65	serine protease inhibitor-like protein

Locus_16084_Transcript_1/1_Conf_1.000	215	5	1.24E-04	EFN81442.1	65	49.6766	44	29	Protein TRS85-like protein
Locus_16085_Transcript_1/1_Conf_1.000	186	0							
Locus_16086_Transcript_1/1_Conf_1.000	730	0							
Locus_16087_Transcript_1/1_Conf_1.000	533	20	3.89E-56	NP_510385.1	77	221.09	176	137	hypothetical protein C11H1.3
Locus_16088_Transcript_1/1_Conf_1.000	320	0							
Locus_16089_Transcript_1/1_Conf_1.000	226	6	1.60E-07	EFO26306.1	80	59.3066	40	32	hypothetical protein LOAG_02178
Locus_1609_Transcript_1/1_Conf_1.000	1841	20	3.46E-39	EFO23299.1	58	167.933	225	132	hypothetical protein LOAG_05186
Locus_16090_Transcript_1/1_Conf_1.000	231	20	6.44E-17	XP_002632463.1	91	90.5077	71	65	Hypothetical protein CBG13687
Locus_16091_Transcript_1/1_Conf_1.000	159	0							
Locus_16092_Transcript_1/1_Conf_1.000	253	0							
Locus_16093_Transcript_1/1_Conf_1.000	325	0							
Locus_16094_Transcript_1/1_Conf_1.000	227	0							
Locus_16095_Transcript_1/1_Conf_1.000	149	0							
Locus_16096_Transcript_1/1_Conf_1.000	359	3	4.57E-15	NP_501984.1	60	84.3445	120	73	Na/Ca eXchangers family member (ncx-3)
Locus_16097_Transcript_1/1_Conf_1.000	605	20	7.06E-96	XP_002645162.1	85	353.599	205	176	Hypothetical protein CBG16872
Locus_16098_Transcript_1/1_Conf_1.000	406	2	7.09E-08	NP_501059.1	86	60.4622	36	31	hypothetical protein T05A12.3
Locus_16099_Transcript_1/1_Conf_1.000	150	0							
Locus_161_Transcript_1/1_Conf_1.000	941	0							
Locus_1610_Transcript_1/2_Conf_1.000	901	20	1.73E-41	NP_498985.3	57	174.096	273	158	hypothetical protein R08D7.4
Locus_1610_Transcript_2/2_Conf_1.000	854	20	9.61E-39	NP_498985.3	57	164.851	264	152	hypothetical protein R08D7.4
Locus_16100_Transcript_1/1_Conf_1.000	150	20	7.96E-15	XP_002634817.1	89	83.5741	48	43	C. briggsae CBR-CLP-7 protein
Locus_16101_Transcript_1/1_Conf_1.000	466	0							
Locus_16102_Transcript_1/1_Conf_1.000	529	0							
Locus_16103_Transcript_1/1_Conf_1.000	214	0							

Locus_16104_Transcript_1/1_Conf_1.000	528	20	1.30E-88	XP_002638687.1	96	328.946	175	168	Hypothetical protein CBG11882
Locus_16105_Transcript_1/1_Conf_1.000	335	20	3.59E-36	XP_002636068.1	76	154.451	111	85	Hypothetical protein CBG01308
Locus_16106_Transcript_1/1_Conf_1.000	235	0							
Locus_16107_Transcript_1/1_Conf_1.000	301	10	2.11E-36	CAR63539.1	78	155.221	97	76	hypothetical protein
Locus_16108_Transcript_1/1_Conf_1.000	523	20	8.45E-37	XP_001894795.1	77	156.762	119	92	Aspartyl aminopeptidase
Locus_16109_Transcript_1/1_Conf_1.000	152	8	6.51E-17	NP_495365.1	92	90.5077	50	46	RaPsYn family member (rpy-1)
Locus_1611_Transcript_1/1_Conf_1.000	884	20	2.24E-62	P0C5E6.2	70	243.432	244	171	Mediator of RNA polymerase II transcription subunit 8
Locus_16110_Transcript_1/1_Conf_1.000	354	20	2.36E-11	XP_001112673.1	71	72.0182	56	40	PREDICTED: UPF0587 protein C1orf123-like isoform 3

Locus_16111_Transcript_1/1_Conf_1.000	549	16	1.73E-57	NP_497035.3	83	225.713	169	141	Trehalose 6-Phosphate Synthase family member (tps-2)
Locus_16112_Transcript_1/1_Conf_1.000	801	20	1.30E-10	XP_002802251.1	43	71.2478	303	132	PREDICTED: rootletin-like
Locus_16113_Transcript_1/1_Conf_1.000	334	0							
Locus_16114_Transcript_1/1_Conf_1.000	189	20	2.72E-23	XP_002822850.1	100	111.694	62	62	PREDICTED: CD9 antigen-like
Locus_16115_Transcript_1/1_Conf_1.000	258	20	2.80E-36	NP_497814.3	94	154.836	85	80	UNCoordinated family member (unc-79)
Locus_16116_Transcript_1/1_Conf_1.000	194	20	6.94E-11	XP_001422216.1	67	70.4774	64	43	predicted protein
Locus_16117_Transcript_1/1_Conf_1.000	593	20	2.64E-60	NP_493583.1	85	235.343	168	143	SeMaPhorin related family member (smp-1)
Locus_16118_Transcript_1/1_Conf_1.000	155	0							
Locus_16119_Transcript_1/1_Conf_1.000	480	20	6.09E-28	XP_640893.1	61	127.102	159	97	hypothetical protein DDB_G0281219

Locus_1612_Transcript_1/1_Conf_1.000	282	20	6.29E-36	NP_001041183.1	85	153.68	95	81	Na/Ca eXchangers family member (ncx-1)
Locus_16120_Transcript_1/1_Conf_1.000	370	20	4.08E-11	XP_001983598.1	79	71.2478	49	39	GH15492
Locus_16121_Transcript_1/1_Conf_1.000	177	20	7.97E-28	EAW98190.1	100	126.716	59	59	cytochrome c oxidase subunit VIa polypeptide 1, isoform CRA_a
Locus_16122_Transcript_1/1_Conf_1.000	434	0							
Locus_16123_Transcript_1/1_Conf_1.000	137	1	4.72E-04	NP_494220.2	69	47.7506	43	30	hypothetical protein F54A3.1
Locus_16124_Transcript_1/1_Conf_1.000	331	0							
Locus_16125_Transcript_1/2_Conf_1.000	293	0							
Locus_16125_Transcript_2/2_Conf_1.000	293	0							
Locus_16126_Transcript_1/1_Conf_1.000	284	0							
Locus_16127_Transcript_1/1_Conf_1.000	627	20	2.97E-63	XP_002635377.1	78	245.358	210	165	Hypothetical protein CBG00768
Locus_16128_Transcript_1/2_Conf_1.000	817	20	2.90E-82	ADG63133.1	71	309.301	269	193	calcium-dependent apyrase
Locus_16128_Transcript_2/2_Conf_1.000	817	20	3.79E-82	ADG63133.1	71	308.916	269	193	calcium-dependent apyrase
Locus_16129_Transcript_1/1_Conf_1.000	220	2	3.04E-06	XP_001900855.1	61	55.0694	67	41	hypothetical protein Bm1_46970
Locus_1613_Transcript_1/1_Conf_1.000	301	6	2.60E-18	AAT02162.1	97	95.1301	43	42	hypothetical protein L3ni51
Locus_16130_Transcript_1/1_Conf_1.000	505	2	8.72E-09	XP_002640245.1	61	63.5438	123	76	Hypothetical protein CBG12763

Locus_16131_Transcript_1/1_Conf_1.000	287	20	2.81E-44	EFO21966.1	97	181.415	94	92	pre-mRNA splicing factor ATP-dependent RNA helicase
Locus_16132_Transcript_1/1_Conf_1.000	496	4	9.51E-13	XP_002639360.1	58	76.6406	167	97	Hypothetical protein CBG03940
Locus_16133_Transcript_1/1_Conf_1.000	218	4	1.07E-24	CAR63710.1	88	116.316	71	63	putative C2 domain containing protein
Locus_16134_Transcript_1/1_Conf_1.000	257	0							
Locus_16135_Transcript_1/1_Conf_1.000	261	0							
Locus_16136_Transcript_1/1_Conf_1.000	936	20	2.39E-97	XP_002639541.1	79	359.762	284	227	Hypothetical protein CBG04170
Locus_16137_Transcript_1/1_Conf_1.000	228	0							
Locus_16138_Transcript_1/1_Conf_1.000	515	20	5.27E-49	NP_498761.1	79	197.208	174	138	DiCer Related family member (dcr-1)
Locus_16139_Transcript_1/1_Conf_1.000	158	0							
Locus_1614_Transcript_1/1_Conf_1.000	2487	20	0	XP_002639688.1	91	1379.39	828	761	C. briggsae CBR-FASN-1 protein

Locus_16140_Transcript_1/1_Conf_1.000	499	20	3.94E-43	CAD24794.1	93	177.563	94	88	HOM-C transcription factor
Locus_16141_Transcript_1/1_Conf_1.000	461	20	5.46E-61	XP_002634990.1	86	236.884	152	131	Hypothetical protein CBG13530
Locus_16142_Transcript_1/1_Conf_1.000	324	20	4.04E-11	AAD49436.1	64	71.2478	109	70	AF083642_1P-glycoprotein
Locus_16143_Transcript_1/1_Conf_1.000	302	0							
Locus_16144_Transcript_1/1_Conf_1.000	170	0							
Locus_16145_Transcript_1/1_Conf_1.000	368	20	6.72E-27	NP_497575.2	69	123.635	121	84	Homeobox family member (ceh-44)
Locus_16146_Transcript_1/1_Conf_1.000	201	0							
Locus_16147_Transcript_1/1_Conf_1.000	257	0							
Locus_16148_Transcript_1/1_Conf_1.000	591	2	8.30E-30	XP_002638587.1	61	134.035	201	123	Hypothetical protein CBG05635
Locus_16149_Transcript_1/1_Conf_1.000	359	0							
Locus_1615_Transcript_1/1_Conf_1.000	937	20	2.65E-88	XP_002647046.1	72	329.717	309	225	Hypothetical protein CBG03564
Locus_16150_Transcript_1/1_Conf_1.000	302	0							
Locus_16151_Transcript_1/1_Conf_1.000	260	20	4.18E-32	XP_001902960.1	96	140.969	86	83	Rap/ran-GAP family protein
Locus_16152_Transcript_1/1_Conf_1.000	270	19	3.67E-23	YP_173415.1	100	77.411	36	36	hypothetical protein NitaMp073
Locus_16153_Transcript_1/1_Conf_1.000	375	7	9.02E-11	NP_001021225.1	48	70.0922	154	74	CEll Death abnormality family member (ced-7)
Locus_16154_Transcript_1/1_Conf_1.000	174	0							
Locus_16155_Transcript_1/1_Conf_1.000	328	7	3.19E-08	XP_001897687.1	60	61.6178	84	51	Zinc finger, C2H2 type family protein
Locus_16156_Transcript_1/1_Conf_1.000	147	0							

Locus_16157_Transcript_1/1_Conf_1.000	225	20	6.28E-12	XP_002612934.1	78	73.9442	60	47	hypothetical protein BRAFLDRAFT_278667
Locus_16158_Transcript_1/1_Conf_1.000	175	0							
Locus_16159_Transcript_1/1_Conf_1.000	137	0							
Locus_1616_Transcript_1/1_Conf_1.000	480	20	2.02E-31	NP_001041137.1	67	138.658	156	105	Alpha MANnosidase family member (aman-2)
Locus_16160_Transcript_1/1_Conf_1.000	350	6	2.53E-21	XP_002641319.1	73	105.145	113	83	Hypothetical protein CBG24620
Locus_16161_Transcript_1/1_Conf_1.000	152	0							
Locus_16162_Transcript_1/1_Conf_1.000	259	0							
Locus_16163_Transcript_1/1_Conf_1.000	160	0							
Locus_16164_Transcript_1/1_Conf_1.000	166	0							
Locus_16165_Transcript_1/1_Conf_1.000	369	3	6.10E-07	NP_496477.2	57	57.3806	103	59	hypothetical protein D1043.1
Locus_16166_Transcript_1/1_Conf_1.000	249	20	1.67E-09	CAA22110.2	62	65.855	82	51	C. elegans protein Y75B8A.26, partially confirmed by transcript evidence
Locus_16167_Transcript_1/1_Conf_1.000	223	4	1.50E-13	NP_493592.1	69	79.337	72	50	hypothetical protein F32A7.4
Locus_16168_Transcript_1/1_Conf_1.000	133	20	5.05E-14	NP_492007.1	93	80.8777	44	41	NADH Ubiquinone oxidoreductase Fe-S protein family member (nduf-6)
Locus_16169_Transcript_1/1_Conf_1.000	493	0							
Locus_1617_Transcript_1/1_Conf_1.000	675	20	1.81E-67	CAN99708.2	93	259.61	176	164	C. elegans protein Y54G11A.10a, confirmed by transcript evidence
Locus_16170_Transcript_1/1_Conf_1.000	967	20	1.55E-131	CAA60046.1	96	473.396	261	251	cytoplasmic intermediate filament protein
Locus_16171_Transcript_1/1_Conf_1.000	196	20	3.00E-14	NP_491127.1	76	81.6481	65	50	hypothetical protein Y54E10A.3
Locus_16172_Transcript_1/1_Conf_1.000	209	0							
Locus_16173_Transcript_1/1_Conf_1.000	380	4	1.42E-40	NP_505267.1	88	169.088	127	112	Regulator of Presynaptic Morphology family member (rpm-1)
Locus_16174_Transcript_1/1_Conf_1.000	334	0							
Locus_16175_Transcript_1/1_Conf_1.000	143	0							

Locus_16176_Transcript_1/1_Conf_1.000	734	20	7.93E-54	AAA68328.2	66	214.542	239	158	Hypothetical protein F35D2.4
Locus_16177_Transcript_1/1_Conf_1.000	453	20	3.03E-51	XP_002638651.1	84	204.527	132	112	Hypothetical protein CBG23705
Locus_16178_Transcript_1/1_Conf_1.000	157	0							
Locus_16179_Transcript_1/1_Conf_1.000	258	20	8.45E-25	EFO26733.1	77	116.701	83	64	GTP1/OBG family protein
Locus_1618_Transcript_1/2_Conf_1.000	731	2	1.15E-04	XP_002642452.1	76	51.2174	38	29	Hypothetical protein CBG06862
Locus_1618_Transcript_2/2_Conf_1.000	731	2	1.15E-04	XP_002642452.1	76	51.2174	38	29	Hypothetical protein CBG06862
Locus_16180_Transcript_1/2_Conf_1.000	608	0							
Locus_16180_Transcript_2/2_Conf_1.000	562	0							
Locus_16181_Transcript_1/1_Conf_1.000	221	0							
Locus_16182_Transcript_1/1_Conf_1.000	596	4	2.64E-07	EFO21213.1	49	59.3066	161	80	hypothetical protein LOAG_07275
Locus_16183_Transcript_1/1_Conf_1.000	394	16	4.65E-23	XP_001900825.1	64	110.923	113	73	CG3579-PA
Locus_16184_Transcript_1/1_Conf_1.000	442	0							
Locus_16185_Transcript_1/1_Conf_1.000	388	0							
Locus_16186_Transcript_1/1_Conf_1.000	272	4	9.23E-16	NP_495925.1	63	86.6557	90	57	hypothetical protein F44G4.8
Locus_16187_Transcript_1/1_Conf_1.000	544	20	2.04E-55	XP_002646278.1	72	218.779	177	128	Hypothetical protein CBG11983
Locus_16188_Transcript_1/1_Conf_1.000	240	1	2.46E-05	XP_002764298.1	69	46.595	39	27	PREDICTED: hypothetical protein LOC100405620, partial
Locus_16189_Transcript_1/1_Conf_1.000	473	0							
Locus_1619_Transcript_1/4_Conf_0.571	2187	20	0	XP_002640691.1	82	891.338	676	561	Hypothetical protein CBG19756
Locus_1619_Transcript_2/4_Conf_0.571	2176	20	0	XP_002640691.1	82	891.338	676	561	Hypothetical protein CBG19756
Locus_1619_Transcript_3/4_Conf_0.571	2264	20	0	XP_002640691.1	81	893.264	701	571	Hypothetical protein CBG19756

Locus_1619_Transcript_4/4_Conf_0.571	2176	20	0	XP_002640691.1	82	891.338	676	561	Hypothetical protein CBG19756
Locus_16190_Transcript_1/1_Conf_1.000	199	3	1.70E-09	CAA22108.2	98	65.855	50	49	C. elegans protein Y75B8A.24, partially confirmed by transcript evidence
Locus_16191_Transcript_1/1_Conf_1.000	254	20	8.53E-17		74	90.1225	74	55	myoblast city protein homolog
Locus_16192_Transcript_1/1_Conf_1.000	460	0							
Locus_16193_Transcript_1/1_Conf_1.000	147	0							
Locus_16194_Transcript_1/1_Conf_1.000	300	0							
Locus_16195_Transcript_1/1_Conf_1.000	232	0							
Locus_16196_Transcript_1/1_Conf_1.000	138	0							
Locus_16197_Transcript_1/1_Conf_1.000	906	8	1.07E-22	XP_002636857.1	49	111.694	239	118	Hypothetical protein CBG09313
Locus_16198_Transcript_1/1_Conf_1.000	207	20	8.30E-25	XP_002643153.1	97	116.701	68	66	C. briggsae CBR-RHA-2 protein
Locus_16199_Transcript_1/1_Conf_1.000	189	0							
Locus_162_Transcript_1/8_Conf_0.083	303	0							
Locus_162_Transcript_2/8_Conf_0.083	261	0							
Locus_162_Transcript_3/8_Conf_0.333	1458	20	9.49E-10	XP_001753823.1	32	69.707	359	117	predicted protein
Locus_162_Transcript_4/8_Conf_0.333	1500	20	9.87E-10	XP_001753823.1	32	69.707	359	117	predicted protein
Locus_162_Transcript_5/8_Conf_0.292	1567	6	3.13E-06	XP_001753823.1	31	58.151	370	115	predicted protein
Locus_162_Transcript_6/8_Conf_0.292	1567	6	3.13E-06	XP_001753823.1	31	58.151	370	115	predicted protein
Locus_162_Transcript_7/8_Conf_0.333	1488	6	2.94E-06	XP_001753823.1	31	58.151	370	115	predicted protein
Locus_162_Transcript_8/8_Conf_0.333	1446	6	2.82E-06	XP_001753823.1	31	58.151	370	115	predicted protein
Locus_1620_Transcript_1/3_Conf_0.333	245	6	1.85E-08	ACF74367.1	64	62.3882	82	53	Hypothetical protein R13F6.4d
Locus_1620_Transcript_2/3_Conf_0.667	2034	20	1.01E-95	XP_002641869.1	55	355.91	628	349	C. briggsae CBR-TEN-1 protein
Locus_1620_Transcript_3/3_Conf_0.667	2034	20	1.01E-95	XP_002641869.1	55	355.91	628	349	C. briggsae CBR-TEN-1 protein
Locus_16200_Transcript_1/1_Conf_1.000	316	20	5.49E-24	XP_002732970.1	75	114.005	101	76	PREDICTED: Cell-death-Related Nuclease family member (crn-2)-like

Locus_16201_Transcript_1/2_Conf_1.000	313	4	1.27E-12	NP_498871.2	57	76.2554	102	59	hypothetical protein F42H10.5
Locus_16201_Transcript_2/2_Conf_1.000	313	4	1.27E-12	NP_498871.2	57	76.2554	102	59	hypothetical protein F42H10.5
Locus_16202_Transcript_1/1_Conf_1.000	134	0							
Locus_16203_Transcript_1/1_Conf_1.000	1426	20	1.81E-82	EFO22071.1	61	311.227	479	295	hypothetical protein LOAG_06415
Locus_16204_Transcript_1/1_Conf_1.000	253	0							
Locus_16205_Transcript_1/1_Conf_1.000	144	0							
Locus_16206_Transcript_1/1_Conf_1.000	202	0							
Locus_16207_Transcript_1/1_Conf_1.000	378	20	6.65E-14	EFO28351.1	80	80.4925	51	41	2Fe-2S iron-sulfur cluster binding domain-containing protein
Locus_16208_Transcript_1/1_Conf_1.000	469	20	1.22E-07	XP_001892000.1	76	59.6918	47	36	linker histone H1 and H5 family protein
Locus_16209_Transcript_1/1_Conf_1.000	481	0							
Locus_1621_Transcript_1/2_Conf_1.000	985	20	1.15E-12	XP_001897369.1	82	78.5666	52	43	Protein kinase domain containing protein
Locus_1621_Transcript_2/2_Conf_1.000	435	20	3.68E-49	XP_001897369.1	79	197.593	142	113	Protein kinase domain containing protein
Locus_16210_Transcript_1/1_Conf_1.000	303	20	5.38E-24	XP_002632297.1	73	114.005	87	64	C. briggsae CBR-NOL-6 protein
Locus_16211_Transcript_1/1_Conf_1.000	533	20	3.79E-27	XP_002640768.1	69	124.79	136	95	C. briggsae CBR-HYL-1 protein

Locus_16212_Transcript_1/1_Conf_1.000	603	11	9.44E-16	EFO22836.1	47	87.4261	189	90	hypothetical protein LOAG_05647
Locus_16213_Transcript_1/1_Conf_1.000	2075	20	9.21E-44	EFO25547.1	50	183.341	352	176	hypothetical protein LOAG_02935
Locus_16214_Transcript_1/1_Conf_1.000	380	20	1.25E-20	NP_502406.1	77	102.834	77	60	Heat Shock factor Binding protein family member (hsb-1)
Locus_16215_Transcript_1/1_Conf_1.000	523	20	2.10E-11	NP_001123062.1	48	72.4034	101	49	hypothetical protein Y69H2.3
Locus_16216_Transcript_1/1_Conf_1.000	827	0							
Locus_16217_Transcript_1/1_Conf_1.000	272	0							
Locus_16218_Transcript_1/1_Conf_1.000	198	1	3.66E-04	ACI49251.1	72	48.1358	44	32	hypothetical protein Csp3_JD07.001
Locus_16219_Transcript_1/1_Conf_1.000	149	0							
Locus_1622_Transcript_1/1_Conf_1.000	508	20	3.10E-86	AAH00553.2	100	320.857	154	154	PLD3 protein

Locus_16220_Transcript_1/1_Conf_1.000	358	20	2.59E-18	AAB23867.2	80	95.1301	77	62	UNC-5
Locus_16221_Transcript_1/1_Conf_1.000	406	0							
Locus_16222_Transcript_1/1_Conf_1.000	968	20	1.94E-166	NP_492476.1	92	589.341	322	299	hypothetical protein F14B4.3
Locus_16223_Transcript_1/1_Conf_1.000	1019	20	3.23E-98	NP_502432.1	77	362.844	339	262	RBD (RNA binding domain) protein family member (rbd-1)
Locus_16224_Transcript_1/1_Conf_1.000	192	8	4.33E-21	CAR63588.1	82	104.375	63	52	hypothetical protein
Locus_16225_Transcript_1/1_Conf_1.000	239	0							
Locus_16226_Transcript_1/1_Conf_1.000	149	0							
Locus_16227_Transcript_1/1_Conf_1.000	147	5	6.78E-06	XP_001898552.1	71	53.9138	49	35	hypothetical protein Bm1_35525
Locus_16228_Transcript_1/1_Conf_1.000	1087	20	2.99E-137	XP_002637530.1	89	492.656	312	278	C. briggsae CBR-UCP-4 protein
Locus_16229_Transcript_1/1_Conf_1.000	141	0							
Locus_1623_Transcript_1/1_Conf_1.000	722	20	6.00E-83	XP_002644219.1	88	311.227	177	157	Hypothetical protein CBG17217
Locus_16230_Transcript_1/1_Conf_1.000	210	20	7.76E-15	NP_496490.2	72	83.5741	69	50	NEPrilysin metallopeptidase family member (nep-1)
Locus_16231_Transcript_1/1_Conf_1.000	185	20	1.72E-25	AAA03517.1	98	119.013	61	60	kinesin-related protein
Locus_16232_Transcript_1/1_Conf_1.000	400	0							
Locus_16233_Transcript_1/1_Conf_1.000	225	0							
Locus_16234_Transcript_1/1_Conf_1.000	332	0							
Locus_16235_Transcript_1/1_Conf_1.000	236	0							

Locus_16236_Transcript_1/1_Conf_1.000	440	0							
Locus_16237_Transcript_1/1_Conf_1.000	188	0							
Locus_16238_Transcript_1/1_Conf_1.000	215	5	3.59E-20	AAD01960.1	88	101.293	70	62	UNC-45
Locus_16239_Transcript_1/1_Conf_1.000	303	2	7.31E-05	AAB63465.1	74	50.447	35	26	O-linked GlcNAc transferase
Locus_1624_Transcript_1/1_Conf_1.000	623	3	1.11E-14	NP_492344.2	93	83.9593	44	41	FMRF-Like Peptide family member (flp-22)
Locus_16240_Transcript_1/1_Conf_1.000	293	20	3.79E-17	NP_491902.3	69	91.2781	94	65	hypothetical protein D2092.5
Locus_16241_Transcript_1/1_Conf_1.000	142	0							
Locus_16242_Transcript_1/1_Conf_1.000	129	0							
Locus_16243_Transcript_1/1_Conf_1.000	423	0							
Locus_16244_Transcript_1/1_Conf_1.000	510	20	3.22E-59	ACI49130.1	82	231.106	173	142	hypothetical protein Cbre_JD16.002
Locus_16245_Transcript_1/2_Conf_1.000	1365	20	2.45E-129	XP_002631212.1	77	466.848	386	298	C. briggsae CBR-ECH-4 protein
Locus_16245_Transcript_2/2_Conf_1.000	1428	20	2.59E-129	XP_002631212.1	77	466.848	386	298	C. briggsae CBR-ECH-4 protein
Locus_16246_Transcript_1/1_Conf_1.000	129	0							
Locus_16247_Transcript_1/1_Conf_1.000	178	1	1.12E-05	CBA11992.1	79	53.1434	44	35	endonuclease-reverse transcriptase HmRTE-e01

Locus_16248_Transcript_1/1_Conf_1.000	231	20	1.81E-19	3HOF	97	98.9821	48	47	Structure Of Macrophage Migration Inhibitory Factor (Mif) With Caffeic Acid At 1.9a Resolution
Locus_16249_Transcript_1/1_Conf_1.000	148	0							
Locus_1625_Transcript_1/1_Conf_1.000	321	20	5.63E-29	XP_001901345.1	79	130.568	104	83	Beta-tubulin cofactor D family protein
Locus_16250_Transcript_1/1_Conf_1.000	566	20	3.64E-29	XP_002634284.1	88	131.724	81	72	Hypothetical protein CBG17617
Locus_16251_Transcript_1/1_Conf_1.000	407	20	2.76E-36	NP_495979.1	74	154.836	135	100	hypothetical protein C01G6.7
Locus_16252_Transcript_1/2_Conf_1.000	517	20	5.36E-50	XP_002632657.1	82	162.925	109	90	C. briggsae CBR-TRA-3 protein
Locus_16252_Transcript_2/2_Conf_1.000	419	20	3.20E-48	XP_002632657.1	79	194.512	139	111	C. briggsae CBR-TRA-3 protein
Locus_16253_Transcript_1/1_Conf_1.000	152	0							
Locus_16254_Transcript_1/1_Conf_1.000	202	0							
Locus_16255_Transcript_1/2_Conf_1.000	332	0							
Locus_16255_Transcript_2/2_Conf_1.000	332	0							

Locus_16256_Transcript_1/1_Conf_1.000	247	20	2.75E-20	NP_001040934.1	73	101.679	78	57	hypothetical protein F13E9.13
Locus_16257_Transcript_1/1_Conf_1.000	624	1	1.06E-04	NP_497287.2	42	50.8322	126	54	hypothetical protein F23H11.2
Locus_16258_Transcript_1/1_Conf_1.000	477	4	1.65E-49	NP_506653.1	84	198.749	158	134	hypothetical protein W06A7.2
Locus_16259_Transcript_1/1_Conf_1.000	132	0							
Locus_1626_Transcript_1/1_Conf_1.000	249	20	7.71E-15	AAD51334.1	86	83.5741	50	43	Kunitz type serine protease inhibitor
Locus_16260_Transcript_1/1_Conf_1.000	129	0							
Locus_16261_Transcript_1/1_Conf_1.000	163	0							
Locus_16262_Transcript_1/1_Conf_1.000	372	3	1.22E-07	ABS19466.2	59	59.6918	91	54	Hypothetical protein F49D11.10
Locus_16263_Transcript_1/1_Conf_1.000	437	15	9.80E-10	AAN11402.1	51	66.6254	105	54	secreted-protein 1 precursor
Locus_16264_Transcript_1/1_Conf_1.000	280	0							
Locus_16265_Transcript_1/1_Conf_1.000	207	20	6.81E-19	XP_002634252.1	83	97.0561	65	54	Hypothetical protein CBG01823
Locus_16266_Transcript_1/1_Conf_1.000	391	0							
Locus_16267_Transcript_1/1_Conf_1.000	337	20	2.94E-22	XP_002647284.1	70	108.227	114	80	Hypothetical protein CBG06324
Locus_16268_Transcript_1/1_Conf_1.000	276	0							
Locus_16269_Transcript_1/1_Conf_1.000	147	0							
Locus_1627_Transcript_1/1_Conf_1.000	421	20	7.41E-13	NP_497719.1	63	77.0258	98	62	GLYcosylation related family member (gly-14)
Locus_16270_Transcript_1/1_Conf_1.000	187	3	2.17E-12	ACI49212.1	74	75.485	62	46	hypothetical protein Csp3_JD04.011
Locus_16271_Transcript_1/1_Conf_1.000	412	20	2.31E-51	NP_497659.1	82	204.912	136	112	hypothetical protein Y53G8B.2
Locus_16272_Transcript_1/1_Conf_1.000	135	0							
Locus_16273_Transcript_1/1_Conf_1.000	192	20	1.03E-14	BAG59226.1	100	83.1889	63	63	unnamed protein product
Locus_16274_Transcript_1/1_Conf_1.000	198	20	1.94E-05	XP_001121729.1	66	52.373	53	35	PREDICTED: similar to CG10979-PA
Locus_16275_Transcript_1/1_Conf_1.000	185	20	7.47E-21	XP_002630326.1	90	103.605	61	55	Hypothetical protein CBG04253
Locus_16276_Transcript_1/1_Conf_1.000	205	0							

Locus_16277_Transcript_1/1_Conf_1.000	297	20	1.98E-50	XP_002643622.1	94	201.83	98	93	C. briggsae CBR-NAS-39 protein
Locus_16278_Transcript_1/1_Conf_1.000	159	0							
Locus_16279_Transcript_1/1_Conf_1.000	294	20	3.63E-36	NP_001022234.1	86	154.451	99	86	hypothetical protein K05F1.6
Locus_1628_Transcript_1/2_Conf_1.000	375	0							
Locus_1628_Transcript_2/2_Conf_1.000	262	0							
Locus_16280_Transcript_1/1_Conf_1.000	657	20	2.39E-85	XP_002643273.1	85	318.931	218	187	C. briggsae CBR-MRP-1 protein
Locus_16281_Transcript_1/1_Conf_1.000	287	0							
Locus_16282_Transcript_1/2_Conf_1.000	328	20	5.04E-38	XP_001891848.1	81	160.614	99	81	cytoplasmic polyadenylation element binding protein 3
Locus_16282_Transcript_2/2_Conf_1.000	328	20	5.04E-38	XP_001891848.1	81	160.614	99	81	cytoplasmic polyadenylation element binding protein 3
Locus_16283_Transcript_1/1_Conf_1.000	554	3	5.87E-53	NP_497506.3	72	210.69	193	139	hypothetical protein Y48G9A.3
Locus_16284_Transcript_1/1_Conf_1.000	225	0							
Locus_16285_Transcript_1/1_Conf_1.000	404	20	8.62E-38	XP_002633240.1	92	159.844	99	92	C. briggsae CBR-LSM-7 protein
Locus_16286_Transcript_1/1_Conf_1.000	421	20	6.84E-59	XP_002635072.1	92	229.95	140	130	C. briggsae CBR-TMD-2 protein
Locus_16287_Transcript_1/1_Conf_1.000	167	0							
Locus_16288_Transcript_1/1_Conf_1.000	156	3	3.01E-06	NP_499405.4	68	55.0694	47	32	2 (Zwei) IG-domain protein family member (zig-5)
Locus_16289_Transcript_1/1_Conf_1.000	195	1	6.27E-04	XP_001896346.1	62	47.3654	53	33	laminin alpha chain

Locus_1629_Transcript_1/1_Conf_1.000	1366	20	4.49E-107	XP_001895824.1	67	392.889	419	282	SH2 domain containing protein
Locus_16290_Transcript_1/1_Conf_1.000	168	10	6.40E-09	NP_508180.1	81	63.929	49	40	hypothetical protein F35H12.1
Locus_16291_Transcript_1/1_Conf_1.000	242	20	5.74E-34	XP_001368223.1	100	147.132	72	72	PREDICTED: similar to ribosomal protein S21
Locus_16292_Transcript_1/1_Conf_1.000	362	0							
Locus_16293_Transcript_1/1_Conf_1.000	326	20	8.06E-44	XP_002633498.1	88	179.874	108	96	Hypothetical protein CBG06270
Locus_16294_Transcript_1/1_Conf_1.000	141	0							
Locus_16295_Transcript_1/1_Conf_1.000	500	0							
Locus_16296_Transcript_1/1_Conf_1.000	991	20	2.79E-99	EFO27969.1	81	366.311	323	263	hypothetical protein LOAG_00509
Locus_16297_Transcript_1/1_Conf_1.000	230	0							
Locus_16298_Transcript_1/1_Conf_1.000	162	2	4.62E-07	NP_505112.1	68	57.7658	54	37	hypothetical protein F20D6.11
Locus_16299_Transcript_1/1_Conf_1.000	339	0							
Locus_163_Transcript_1/1_Conf_1.000	3210	20	0	NP_495343.1	68	718.768	770	525	hypothetical protein C56C10.12
Locus_1630_Transcript_1/2_Conf_1.000	1919	20	4.83E-108	XP_001898078.1	57	396.741	647	374	Ubiquitin carboxyl-terminal hydrolase family protein
Locus_1630_Transcript_2/2_Conf_1.000	1898	20	1.38E-107	XP_001898078.1	57	395.201	647	370	Ubiquitin carboxyl-terminal hydrolase family protein
Locus_16300_Transcript_1/1_Conf_1.000	382	4	6.38E-25	NP_497649.1	73	117.087	106	78	hypothetical protein Y55D5A.4
Locus_16301_Transcript_1/1_Conf_1.000	502	20	4.48E-34	EFO18114.1	61	147.517	164	101	hypothetical protein LOAG_10384

Locus_16302_Transcript_1/2_Conf_1.000	546	20	5.17E-14	NP_501018.1	67	81.2629	78	53	Dicer Related Helicase family member (drh-1)
Locus_16302_Transcript_2/2_Conf_1.000	487	20	7.33E-37	XP_002633533.1	63	156.762	161	103	C. briggsae CBR-DRH-1 protein
Locus_16303_Transcript_1/1_Conf_1.000	270	20	2.12E-20	NP_496477.2	75	102.064	86	65	hypothetical protein D1043.1
Locus_16304_Transcript_1/1_Conf_1.000	572	6	1.94E-17	XP_002638739.1	58	92.8189	129	76	Hypothetical protein CBG18539
Locus_16305_Transcript_1/1_Conf_1.000	264	0							
Locus_16306_Transcript_1/1_Conf_1.000	129	0							
Locus_16307_Transcript_1/1_Conf_1.000	254	0							
Locus_16308_Transcript_1/2_Conf_1.000	723	20	5.34E-55	NP_491590.1	75	218.394	182	138	hypothetical protein F27C1.6
Locus_16308_Transcript_2/2_Conf_1.000	726	20	1.42E-55	NP_491590.1	75	220.32	182	138	hypothetical protein F27C1.6
Locus_16309_Transcript_1/1_Conf_1.000	577	0							
Locus_1631_Transcript_1/1_Conf_1.000	1308	20	4.49E-125	NP_495181.2	72	452.595	374	271	Ceramide Glucosyl Transferase family member (cgt-3)
Locus_16310_Transcript_1/1_Conf_1.000	289	5	1.31E-17	NP_498650.2	68	92.8189	92	63	CLocK (biological timing) abnormality family member (clk-2)

Locus_16311_Transcript_1/1_Conf_1.000	323	20	8.97E-35	NP_503027.1	92	149.828	82	76	Small Nuclear Ribonucleoprotein family member (snr-1)
Locus_16312_Transcript_1/1_Conf_1.000	253	0							
Locus_16313_Transcript_1/1_Conf_1.000	131	20	1.29E-09	ADN00780.1	83	66.2402	43	36	class 3 lipase protein
Locus_16314_Transcript_1/1_Conf_1.000	174	0							
Locus_16315_Transcript_1/1_Conf_1.000	356	20	2.05E-39	ACT20206.1	84	165.236	109	92	Hypothetical protein F55F1.5
Locus_16316_Transcript_1/1_Conf_1.000	199	0							
Locus_16317_Transcript_1/1_Conf_1.000	731	20	2.09E-22	XP_002641983.1	48	110.153	218	105	Hypothetical protein CBG09205
Locus_16318_Transcript_1/1_Conf_1.000	197	20	6.03E-23	EFO18009.1	87	110.538	64	56	hypothetical protein LOAG_10489
Locus_16319_Transcript_1/1_Conf_1.000	1397	20	0	NP_491859.1	91	656.366	394	360	Acyl CoA DeHydrogenase family member (acdh-3)

Locus_1632_Transcript_1/1_Conf_1.000	447	20	3.51E-31	NP_491662.1	85	137.887	94	80	DNaJ domain (prokaryotic heat shock protein) family member (dnj-21)
Locus_16320_Transcript_1/1_Conf_1.000	1301	20	1.81E-118	XP_002634047.1	85	430.639	363	312	Hypothetical protein CBG01586
Locus_16321_Transcript_1/1_Conf_1.000	813	20	8.13E-69	NP_502174.2	69	264.618	245	170	hypothetical protein M04B2.2
Locus_16322_Transcript_1/1_Conf_1.000	174	0							
Locus_16323_Transcript_1/1_Conf_1.000	173	2	2.27E-06	NP_506118.1	75	55.4546	45	34	hypothetical protein C54D10.5
Locus_16324_Transcript_1/1_Conf_1.000	541	20	1.99E-71	2JG8	100	271.937	132	132	CrystallographicStructure Of Human C1q Globular Heads Complexed To Phosphatidyl-Serine
Locus_16325_Transcript_1/1_Conf_1.000	159	20	6.04E-15	NP_504512.1	88	83.9593	52	46	hypothetical protein F44E7.2
Locus_16326_Transcript_1/1_Conf_1.000	465	0							
Locus_16327_Transcript_1/1_Conf_1.000	201	0							
Locus_16328_Transcript_1/1_Conf_1.000	255	0							
Locus_16329_Transcript_1/1_Conf_1.000	429	2	6.18E-20	NP_500393.2	65	100.523	114	75	hypothetical protein Y37E11AL.5
Locus_1633_Transcript_1/1_Conf_1.000	250	0							
Locus_16330_Transcript_1/1_Conf_1.000	1137	20	2.54E-25	XP_001900089.1	49	120.939	305	150	hypothetical protein Bm1_43135
Locus_16331_Transcript_1/1_Conf_1.000	518	20	3.75E-34	XP_001901404.1	71	147.902	129	92	ribonuclease P protein subunit p29

Locus_16332_Transcript_1/1_Conf_1.000	1073	20	9.37E-27	EFO25858.1	50	125.561	234	119	low-density lipoprotein receptor repeat class B containing protein
Locus_16333_Transcript_1/1_Conf_1.000	141	20	1.08E-16	XP_001896000.1	95	89.7373	46	44	hypothetical protein
Locus_16334_Transcript_1/2_Conf_1.000	487	20	3.91E-14	EFO25573.1	58	81.2629	93	54	RING finger protein 20

Locus_16334_Transcript_2/2_Conf_1.000	487	20	3.91E-14	EFO25573.1	58	81.2629	93	54	RING finger protein 20
Locus_16335_Transcript_1/1_Conf_1.000	206	20	9.25E-08	CBN79121.1	63	60.077	65	41	conserved unknown protein
Locus_16336_Transcript_1/1_Conf_1.000	202	0							
Locus_16337_Transcript_1/1_Conf_1.000	336	20	1.66E-33	XP_001899441.1	83	145.591	112	93	Xeroderma Pigmentosum Group E Complementing protein
Locus_16338_Transcript_1/1_Conf_1.000	389	20	2.17E-41	NP_001024707.1	79	171.785	121	96	UNCoordinated family member (unc-84)

Locus_16339_Transcript_1/1_Conf_1.000	633	20	6.18E-40	AAL34093.1	69	167.933	164	114	SKR-1
Locus_1634_Transcript_1/2_Conf_1.000	1756	20	2.51E-165	NP_491749.2	88	491.5	280	247	ARI (ubiquitin ligase Ariadne) homolog family member (ari-1)
Locus_1634_Transcript_2/2_Conf_1.000	1752	20	4.98E-173	NP_491749.2	83	612.453	385	320	ARI (ubiquitin ligase Ariadne) homolog family member (ari-1)
Locus_16340_Transcript_1/1_Conf_1.000	170	0							
Locus_16341_Transcript_1/1_Conf_1.000	347	0							
Locus_16342_Transcript_1/1_Conf_1.000	159	0							
Locus_16343_Transcript_1/1_Conf_1.000	560	11	9.94E-08	YP_002478296.1	47	60.4622	180	86	Transposase and inactivated derivatives-like protein
Locus_16344_Transcript_1/1_Conf_1.000	292	0							
Locus_16345_Transcript_1/1_Conf_1.000	505	20	2.97E-41	EFO24017.1	73	171.4	168	123	hypothetical protein LOAG_04469
Locus_16346_Transcript_1/2_Conf_1.000	758	20	2.30E-27	EFO16973.1	59	126.716	238	141	calpain
Locus_16346_Transcript_2/2_Conf_1.000	2555	20	0	EFO16973.1	77	950.658	795	618	calpain
Locus_16347_Transcript_1/1_Conf_1.000	354	0							
Locus_16348_Transcript_1/1_Conf_1.000	333	0							
Locus_16349_Transcript_1/1_Conf_1.000	542	2	3.57E-12	NP_740830.2	47	75.0998	219	104	hypothetical protein Y47G6A.29
Locus_1635_Transcript_1/1_Conf_1.000	1696	20	8.02E-11	NP_038835.2	46	73.559	212	98	serine/threonine-protein kinase PLK3
Locus_16350_Transcript_1/1_Conf_1.000	595	20	1.44E-29	CAZ65501.2	58	133.265	200	117	C. elegans protein F46C3.3c, partially confirmed by transcript evidence

Locus_16351_Transcript_1/1_Conf_1.000	187	5	3.83E-09	XP_001896129.1	70	64.6994	62	44	Hypothetical 216.3 kDa protein R06F6.8 in chromosome II, putative
Locus_16352_Transcript_1/1_Conf_1.000	198	0							
Locus_16353_Transcript_1/1_Conf_1.000	799	2	5.04E-15	NP_510529.1	44	85.8853	284	126	hypothetical protein F39B1.1
Locus_16354_Transcript_1/1_Conf_1.000	180	0							
Locus_16355_Transcript_1/1_Conf_1.000	198	20	1.17E-26	XP_001898798.1	92	122.865	65	60	glucose-6-phosphate dehydrogenase
Locus_16356_Transcript_1/2_Conf_1.000	545	20	2.42E-40	NP_506573.1	80	168.703	165	132	hypothetical protein C15H11.9
Locus_16356_Transcript_2/2_Conf_1.000	544	20	2.42E-40	NP_506573.1	80	168.703	165	132	hypothetical protein C15H11.9
Locus_16357_Transcript_1/1_Conf_1.000	472	20	1.96E-66	NP_495840.1	91	254.988	147	134	hypothetical protein D2085.6
Locus_16358_Transcript_1/1_Conf_1.000	212	0							
Locus_16359_Transcript_1/1_Conf_1.000	961	20	6.58E-66	XP_002636770.1	66	255.373	289	193	Hypothetical protein CBG23501
Locus_1636_Transcript_1/1_Conf_1.000	2059	20	0	XP_002642663.1	82	752.281	518	425	C. briggsae CBR-NCL-1 protein
Locus_16360_Transcript_1/2_Conf_1.000	873	20	3.81E-46	NP_495437.2	78	189.504	151	118	hypothetical protein C52E12.4
Locus_16360_Transcript_2/2_Conf_1.000	896	20	1.55E-50	NP_495437.2	79	204.142	158	126	hypothetical protein C52E12.4

Locus_16361_Transcript_1/1_Conf_1.000	283	20	3.13E-27	XP_001900557.1	86	124.79	94	81	Bromodomain containing protein
Locus_16362_Transcript_1/1_Conf_1.000	197	0							
Locus_16363_Transcript_1/1_Conf_1.000	477	2	1.42E-08	XP_002631345.1	80	62.7734	41	33	Hypothetical protein CBG03175
Locus_16364_Transcript_1/1_Conf_1.000	146	0							
Locus_16365_Transcript_1/1_Conf_1.000	158	0							
Locus_16366_Transcript_1/1_Conf_1.000	373	20	1.75E-46	XP_002630247.1	86	188.734	124	107	Hypothetical protein CBG00666
Locus_16367_Transcript_1/1_Conf_1.000	524	20	8.37E-69	NP_001041257.1	90	263.077	171	155	Regulator of G protein Signaling family member (rgs-7)
Locus_16368_Transcript_1/1_Conf_1.000	496	0							
Locus_16369_Transcript_1/1_Conf_1.000	739	20	2.74E-94	NP_500379.1	82	348.977	245	201	hypothetical protein Y37E11B.5
Locus_1637_Transcript_1/1_Conf_1.000	132	0							
Locus_16370_Transcript_1/1_Conf_1.000	298	0							
Locus_16371_Transcript_1/1_Conf_1.000	363	20	3.34E-34	XP_002636220.1	78	147.902	107	84	Hypothetical protein CBG12141
Locus_16372_Transcript_1/1_Conf_1.000	189	0							
Locus_16373_Transcript_1/1_Conf_1.000	913	7	4.95E-76	CBK19481.1	79	288.886	210	167	C. elegans protein Y105C5A.15a, partially confirmed by transcript evidence
Locus_16374_Transcript_1/1_Conf_1.000	298	3	3.90E-06	NP_495170.1	51	54.6842	77	40	Temporarily Assigned Gene name family member (tag-234)
Locus_16375_Transcript_1/1_Conf_1.000	171	16	5.06E-06	NP_502508.1	69	54.299	39	27	SCP-Like extracellular protein family member (scl-6)
Locus_16376_Transcript_1/1_Conf_1.000	517	20	5.45E-17	XP_002638473.1	56	90.8929	175	98	Hypothetical protein CBG12901
Locus_16377_Transcript_1/1_Conf_1.000	267	1	4.77E-04	NP_001021551.1	66	47.7506	48	32	hypothetical protein K07A1.15
Locus_16378_Transcript_1/1_Conf_1.000	226	0							
Locus_16379_Transcript_1/1_Conf_1.000	162	2	1.10E-08	XP_002645729.1	92	63.1586	53	49	Hypothetical protein CBG07396
Locus_1638_Transcript_1/1_Conf_1.000	2637	20	2.90E-170	XP_001893842.1	62	603.979	675	419	hypothetical protein
Locus_16380_Transcript_1/1_Conf_1.000	196	20	2.01E-18	NP_501419.2	76	95.5153	65	50	hypothetical protein D2096.3

Locus_16381_Transcript_1/1_Conf_1.000	380	20	1.42E-40	NP_508799.1	86	169.088	111	96	hypothetical protein F16H11.1
Locus_16382_Transcript_1/1_Conf_1.000	545	0							
Locus_16383_Transcript_1/1_Conf_1.000	523	20	1.22E-27	NP_493278.2	78	126.331	98	77	Neuropeptide-Like Protein family member (nlp-15)
Locus_16384_Transcript_1/1_Conf_1.000	255	0							
Locus_16385_Transcript_1/1_Conf_1.000	383	20	7.82E-15	XP_002312307.1	65	83.5741	84	55	predicted protein
Locus_16386_Transcript_1/1_Conf_1.000	281	20	3.35E-18	NP_498265.1	68	94.7449	94	64	hypothetical protein F01F1.9
Locus_16387_Transcript_1/1_Conf_1.000	568	20	2.70E-80	XP_001896559.1	84	301.597	191	161	hypothetical protein
Locus_16388_Transcript_1/1_Conf_1.000	617	5	9.05E-09	NP_495641.1	49	64.3142	139	69	CKI family (Cyclin-dependent Kinase Inhibitor) family member (cki-1)
Locus_16389_Transcript_1/1_Conf_1.000	176	0							
Locus_1639_Transcript_1/2_Conf_1.000	537	20	4.22E-58	XP_002631526.1	75	227.639	177	133	C. briggsae CBR-MIG-14 protein
Locus_1639_Transcript_2/2_Conf_1.000	537	20	8.49E-59	XP_002631526.1	75	229.95	177	134	C. briggsae CBR-MIG-14 protein
Locus_16390_Transcript_1/1_Conf_1.000	392	0							
Locus_16391_Transcript_1/1_Conf_1.000	505	20	1.21E-34	XP_002645124.1	68	149.443	164	113	Hypothetical protein CBG16818

Locus_16392_Transcript_1/2_Conf_1.000	1400	20	3.91E-98	NP_741868.1	59	363.229	475	282	Apical Junction Molecule family member (ajm-1)
Locus_16392_Transcript_2/2_Conf_1.000	1435	20	4.34E-92	XP_002645282.1	58	343.199	484	282	C. briggsae CBR-AJM-1 protein
Locus_16393_Transcript_1/1_Conf_1.000	366	20	9.13E-08	ACI49172.1	51	60.077	124	64	hypothetical protein Csp3_JD01.004
Locus_16394_Transcript_1/1_Conf_1.000	471	20	5.41E-24	XP_002425539.1	90	114.005	55	50	Early growth response protein, putative
Locus_16395_Transcript_1/1_Conf_1.000	516	20	1.09E-33	NP_492769.1	65	146.362	167	109	RTEL (mammalian Regulator of TELomere length) homolog family member (rtel-1)
Locus_16396_Transcript_1/1_Conf_1.000	283	0							
Locus_16397_Transcript_1/1_Conf_1.000	280	0							
Locus_16398_Transcript_1/1_Conf_1.000	525	0							
Locus_16399_Transcript_1/1_Conf_1.000	467	20	5.84E-18	XP_001897531.1	55	93.9745	154	86	WW domain containing protein
Locus_164_Transcript_1/2_Conf_1.000	1686	20	7.35E-57	NP_499083.2	48	226.483	496	241	hypothetical protein B0464.6
Locus_164_Transcript_2/2_Conf_1.000	1686	20	2.14E-56	NP_499083.2	51	224.942	428	220	hypothetical protein B0464.6
Locus_1640_Transcript_1/1_Conf_1.000	441	20	8.46E-17	XP_002641151.1	52	90.1225	144	75	Hypothetical protein CBG09003
Locus_16400_Transcript_1/1_Conf_1.000	545	3	3.52E-07	XP_002636981.1	86	58.5362	36	31	C. briggsae CBR-LIN-33 protein
Locus_16401_Transcript_1/1_Conf_1.000	237	0							
Locus_16402_Transcript_1/1_Conf_1.000	231	3	2.89E-25	XP_002641969.1	81	118.242	76	62	Hypothetical protein CBG16676
Locus_16403_Transcript_1/1_Conf_1.000	688	2	5.76E-16	XP_002635026.1	63	88.5817	136	87	Hypothetical protein CBG13570

Locus_16404_Transcript_1/1_Conf_1.000	348	0								
Locus_16405_Transcript_1/1_Conf_1.000	198	7	1.76E-06	XP_002642555.1	54	55.8398	77	42	C. briggsae CBR-UNC-103.2 protein
Locus_16406_Transcript_1/1_Conf_1.000	274	0							
Locus_16407_Transcript_1/1_Conf_1.000	302	0							
Locus_16408_Transcript_1/1_Conf_1.000	290	20	1.95E-29	XP_001902375.1	80	132.109	95	76	Protein kinase domain containing protein
Locus_16409_Transcript_1/1_Conf_1.000	653	0							
Locus_1641_Transcript_1/1_Conf_1.000	1954	20	0	XP_002642110.1	94	876.315	538	509	C. briggsae CBR-CCT-5 protein
Locus_16410_Transcript_1/1_Conf_1.000	291	0							
Locus_16411_Transcript_1/1_Conf_1.000	269	0							
Locus_16412_Transcript_1/1_Conf_1.000	245	4	1.85E-16	NP_509518.1	77	88.9669	70	54	hypothetical protein C24A3.2
Locus_16413_Transcript_1/1_Conf_1.000	340	20	2.99E-59	ACJ65168.1	99	231.106	113	112	Uncoordinated protein 68, isoform b, partially confirmed by transcript evidence
Locus_16414_Transcript_1/1_Conf_1.000	617	3	2.00E-40	EFO25429.1	58	169.474	206	120	hypothetical protein LOAG_03053
Locus_16415_Transcript_1/1_Conf_1.000	467	20	5.61E-45	NP_491182.2	75	183.726	160	120	LiPid Depleted family member (lpd-3)
Locus_16416_Transcript_1/1_Conf_1.000	236	9	1.53E-10	NP_493057.1	70	69.3218	79	56	RNA-dependent RNA polymerase Family family member (rrf-2)
Locus_16417_Transcript_1/1_Conf_1.000	538	6	1.15E-39	XP_002647538.1	71	166.392	130	93	Hypothetical protein CBG06620
Locus_16418_Transcript_1/1_Conf_1.000	155	0							
Locus_16419_Transcript_1/1_Conf_1.000	227	0							

Locus_1642_Transcript_1/1_Conf_1.000	454	20	4.82E-57	XP_002647797.1	90	223.787	125	113	Hypothetical protein CBG23568
Locus_16420_Transcript_1/1_Conf_1.000	222	0							
Locus_16421_Transcript_1/1_Conf_1.000	268	0							
Locus_16422_Transcript_1/1_Conf_1.000	356	3	4.45E-10	NP_508551.1	64	67.781	104	67	hypothetical protein F28B4.3
Locus_16423_Transcript_1/1_Conf_1.000	389	20	7.84E-34	XP_002828455.1	89	136.346	76	68	PREDICTED: LOW QUALITY PROTEIN: ornithine decarboxylase antizyme 1-like
Locus_16424_Transcript_1/1_Conf_1.000	241	0							
Locus_16425_Transcript_1/1_Conf_1.000	387	0							
Locus_16426_Transcript_1/1_Conf_1.000	458	20	2.83E-41	EFO23504.1	77	171.4	157	121	TK protein kinase
Locus_16427_Transcript_1/1_Conf_1.000	220	0							
Locus_16428_Transcript_1/1_Conf_1.000	230	1	4.81E-04	XP_002898487.1	66	47.7506	53	35	microtubule -associated protein EB1
Locus_16429_Transcript_1/1_Conf_1.000	222	0							
Locus_1643_Transcript_1/1_Conf_1.000	629	5	2.81E-21	NP_001022775.1	88	105.916	85	75	hypothetical protein T20G5.14
Locus_16430_Transcript_1/2_Conf_1.000	792	0							
Locus_16430_Transcript_2/2_Conf_1.000	299	0							
Locus_16431_Transcript_1/1_Conf_1.000	180	0							
Locus_16432_Transcript_1/1_Conf_1.000	563	20	2.55E-35	XP_002426892.1	60	152.14	193	116	myst histone acetyltransferase, putative
Locus_16433_Transcript_1/1_Conf_1.000	275	20	3.85E-30	XP_002646095.1	83	134.42	90	75	C. briggsae CBR-HMR-1 protein
Locus_16434_Transcript_1/1_Conf_1.000	393	20	1.40E-11	P48460.4	70	72.7886	64	45	Putative serine/threonine-protein phosphatase C27B7.6
Locus_16435_Transcript_1/1_Conf_1.000	240	0							
Locus_16436_Transcript_1/1_Conf_1.000	550	0							
Locus_16437_Transcript_1/1_Conf_1.000	161	0							

Locus_16438_Transcript_1/2_Conf_1.000	1275	20	1.68E-36	EFO20862.1	50	158.303	289	147	zinc finger protein
Locus_16438_Transcript_2/2_Conf_1.000	1268	20	5.72E-37	EFO20862.1	46	159.844	365	169	zinc finger protein
Locus_16439_Transcript_1/1_Conf_1.000	309	20	1.27E-33	NP_497786.2	89	145.976	100	89	MethylMalonylCoA Mutase homolog family member (mmcm-1)
Locus_1644_Transcript_1/3_Conf_0.556	972	20	1.18E-86	XP_002644841.1	74	324.324	240	178	C. briggsae CBR-HSP-43 protein
Locus_1644_Transcript_3/3_Conf_0.444	941	20	1.12E-86	XP_002644841.1	74	324.324	240	178	C. briggsae CBR-HSP-43 protein
Locus_16440_Transcript_1/1_Conf_1.000	176	0							
Locus_16441_Transcript_1/1_Conf_1.000	312	0							
Locus_16442_Transcript_1/1_Conf_1.000	516	0							
Locus_16443_Transcript_1/1_Conf_1.000	476	0							
Locus_16444_Transcript_1/2_Conf_1.000	603	5	1.04E-38	XP_001895905.1	64	163.696	201	130	Fanconi anemia complementation group D2 protein, isoform 2, putative
Locus_16444_Transcript_2/2_Conf_1.000	399	5	7.37E-21	EFO26102.1	64	103.605	113	73	hypothetical protein LOAG_02385

Locus_16445_Transcript_1/1_Conf_1.000	621	20	3.54E-53	XP_001891594.1	88	211.846	140	124	MH1 domain containing protein
Locus_16446_Transcript_1/1_Conf_1.000	352	0							
Locus_16447_Transcript_1/1_Conf_1.000	523	20	2.52E-65	XP_002645826.1	81	251.521	174	142	Hypothetical protein CBG07557
Locus_16448_Transcript_1/1_Conf_1.000	134	20	6.17E-12	NP_493554.1	86	73.9442	44	38	hypothetical protein Y54E5A.6
Locus_16449_Transcript_1/1_Conf_1.000	358	2	2.68E-15	NP_495433.1	67	85.1149	74	50	hypothetical protein C15F1.2
Locus_1645_Transcript_1/2_Conf_1.000	890	20	2.71E-31	XP_002630755.1	54	140.198	224	122	C. briggsae CBR-LYS-8 protein
Locus_1645_Transcript_2/2_Conf_1.000	890	20	2.71E-31	XP_002630755.1	54	140.198	224	122	C. briggsae CBR-LYS-8 protein
Locus_16450_Transcript_1/1_Conf_1.000	232	0							
Locus_16451_Transcript_1/1_Conf_1.000	246	0							
Locus_16452_Transcript_1/1_Conf_1.000	234	0							
Locus_16453_Transcript_1/1_Conf_1.000	135	0							
Locus_16454_Transcript_1/1_Conf_1.000	712	0							
Locus_16455_Transcript_1/1_Conf_1.000	250	0							
Locus_16456_Transcript_1/1_Conf_1.000	799	20	7.92E-69	NP_495441.1	87	264.618	178	155	hypothetical protein ZK1127.6
Locus_16457_Transcript_1/1_Conf_1.000	138	0							
Locus_16458_Transcript_1/1_Conf_1.000	135	0							
Locus_16459_Transcript_1/1_Conf_1.000	145	0							

Locus_1646_Transcript_1/1_Conf_1.000	1004	20	3.83E-120	NP_504638.1	86	435.647	274	237	hypothetical protein T05H4.5
Locus_16460_Transcript_1/1_Conf_1.000	154	0							
Locus_16461_Transcript_1/1_Conf_1.000	321	0							
Locus_16462_Transcript_1/1_Conf_1.000	840	20	5.20E-106	XP_002637794.1	84	388.267	264	224	C. briggsae CBR-PES-9 protein
Locus_16463_Transcript_1/1_Conf_1.000	450	3	2.32E-59	XP_002634053.1	84	231.491	150	127	Hypothetical protein CBG01593
Locus_16464_Transcript_1/1_Conf_1.000	1058	20	3.19E-96	XP_002639898.1	78	356.295	296	233	Hypothetical protein CBG08220
Locus_16465_Transcript_1/1_Conf_1.000	332	0							
Locus_16466_Transcript_1/1_Conf_1.000	172	20	1.51E-26	XP_001091804.2	100	122.479	57	57	PREDICTED: hypothetical protein LOC703501
Locus_16467_Transcript_1/1_Conf_1.000	474	2	1.80E-11	XP_002647749.1	72	72.4034	47	34	C. briggsae CBR-NLP-42 protein
Locus_16468_Transcript_1/1_Conf_1.000	238	0							
Locus_16469_Transcript_1/1_Conf_1.000	1098	20	8.99E-81	NP_507118.1	57	305.064	365	209	O-ACyltransferase homolog family member (oac-6)
Locus_1647_Transcript_1/2_Conf_1.000	882	20	7.20E-69	NP_501459.1	77	265.003	195	151	Homolog of Yeast Longevity gene family member (hyl-1)

Locus_1647_Transcript_2/2_Conf_1.000	803	20	1.15E-62	NP_501459.1	89	216.083	126	113	Homolog of Yeast Longevity gene family member (hyl-1)
Locus_16470_Transcript_1/1_Conf_1.000	552	20	1.16E-08	XP_001896107.1	46	63.5438	144	67	hypothetical protein Bm1_23250
Locus_16471_Transcript_1/1_Conf_1.000	215	0							
Locus_16472_Transcript_1/1_Conf_1.000	208	2	5.75E-26	XP_002636221.1	95	120.553	68	65	Hypothetical protein CBG12142
Locus_16473_Transcript_1/1_Conf_1.000	775	20	1.85E-35	NP_001040994.1	70	153.68	131	92	WaRThog (hedgehog-like family) family member (wrt-5)
Locus_16474_Transcript_1/1_Conf_1.000	193	19	1.54E-26	XP_002645128.1	92	122.479	64	59	C. briggsae CBR-NSY-4 protein
Locus_16475_Transcript_1/1_Conf_1.000	135	0							
Locus_16476_Transcript_1/1_Conf_1.000	865	20	5.44E-138	XP_002646763.1	90	494.582	278	252	C. briggsae CBR-GSY-1 protein
Locus_16477_Transcript_1/1_Conf_1.000	268	8	2.89E-09	XP_001895179.1	88	65.0846	35	31	Suppressor of forked protein
Locus_16478_Transcript_1/1_Conf_1.000	664	0							
Locus_16479_Transcript_1/1_Conf_1.000	181	0							
Locus_1648_Transcript_1/1_Conf_1.000	1218	20	1.05E-72	NP_499022.1	61	278.485	357	220	hypothetical protein ZK512.5
Locus_16480_Transcript_1/1_Conf_1.000	412	20	3.94E-51	NP_499895.2	86	204.142	137	119	hypothetical protein T21D12.9
Locus_16481_Transcript_1/1_Conf_1.000	301	20	2.11E-36	XP_001895201.1	89	155.221	99	89	protein phosphatase 1 regulatory inhibitor subunit 16A, putative
Locus_16482_Transcript_1/1_Conf_1.000	308	3	1.67E-09	XP_001896335.1	50	65.855	108	55	helix-turn-helix, Psq domain containing protein
Locus_16483_Transcript_1/1_Conf_1.000	189	20	6.71E-22	XP_002630112.1	88	107.071	63	56	Hypothetical protein CBG00513

Locus_16484_Transcript_1/1_Conf_1.000	334	20	6.57E-22	NP_001021889.1	67	107.071	102	69	hypothetical protein B0252.3
Locus_16485_Transcript_1/1_Conf_1.000	167	20	2.70E-07	XP_002648412.1	66	58.5362	53	35	C. briggsae CBR-PQE-1 protein
Locus_16486_Transcript_1/1_Conf_1.000	314	0							
Locus_16487_Transcript_1/1_Conf_1.000	516	1	2.37E-04	NP_506662.1	43	48.9062	142	62	hypothetical protein ZC412.3
Locus_16488_Transcript_1/1_Conf_1.000	537	0							
Locus_16489_Transcript_1/1_Conf_1.000	626	0							
Locus_1649_Transcript_1/1_Conf_1.000	2463	20	3.08E-17	XP_509666.2	37	95.5153	498	189	PREDICTED: similar to KIAA0853 protein
Locus_16490_Transcript_1/1_Conf_1.000	310	2	4.53E-15	NP_502613.2	72	84.3445	93	67	QUInine non-avoider family member (qui-1)
Locus_16491_Transcript_1/1_Conf_1.000	362	20	4.09E-40	XP_001902741.1	75	167.548	120	90	DNA topoisomerase III
Locus_16492_Transcript_1/1_Conf_1.000	205	0							
Locus_16493_Transcript_1/1_Conf_1.000	246	0							
Locus_16494_Transcript_1/1_Conf_1.000	398	5	5.86E-18	NP_494916.1	65	93.9745	133	87	SHC (Src Homology domain C-terminal) adaptor homolog family member (shc-2)
Locus_16495_Transcript_1/1_Conf_1.000	325	0							
Locus_16496_Transcript_1/1_Conf_1.000	631	20	4.68E-32	NP_503727.1	52	141.739	198	104	hypothetical protein W02G9.4
Locus_16497_Transcript_1/1_Conf_1.000	232	0							
Locus_16498_Transcript_1/1_Conf_1.000	300	20	2.19E-25	O96650.1	82	118.627	87	72	Thymidylate synthase

Locus_16499_Transcript_1/1_Conf_1.000	518	20	1.05E-52	NP_509229.1	75	209.534	174	132	SUppressor of activated let-60 Ras family member (sur-5)
Locus_165_Transcript_1/1_Conf_1.000	3162	20	0	NP_496136.1	81	1140.56	915	743	hypothetical protein C08H9.2
Locus_1650_Transcript_1/1_Conf_1.000	845	20	3.08E-90	NP_497876.2	78	335.88	277	218	Heparan Sulfate-glucuronic acid-5-Epimerase family member (hse-5)
Locus_16500_Transcript_1/1_Conf_1.000	330	0							
Locus_16501_Transcript_1/1_Conf_1.000	963	20	3.78E-45	CAA84693.3	56	186.422	312	175	C. elegans protein D2045.2, partially confirmed by transcript evidence
Locus_16502_Transcript_1/1_Conf_1.000	442	0							
Locus_16503_Transcript_1/1_Conf_1.000	307	0							
Locus_16504_Transcript_1/1_Conf_1.000	392	8	9.44E-13	NP_001076703.1	68	76.6406	117	80	hypothetical protein T11G6.2
Locus_16505_Transcript_1/1_Conf_1.000	284	0							
Locus_16506_Transcript_1/1_Conf_1.000	753	20	5.60E-42	XP_002645124.1	66	175.252	162	107	Hypothetical protein CBG16818
Locus_16507_Transcript_1/1_Conf_1.000	273	0							
Locus_16508_Transcript_1/1_Conf_1.000	219	1	1.56E-10	EFO25846.1	69	69.3218	71	49	hypothetical protein LOAG_02637
Locus_16509_Transcript_1/1_Conf_1.000	158	0							

Locus_1651_Transcript_1/2_Conf_1.000	544	0							
Locus_1651_Transcript_2/2_Conf_1.000	550	0							
Locus_16510_Transcript_1/1_Conf_1.000	241	20	6.59E-22	XP_002644770.1	84	107.071	78	66	C. briggsae CBR-ACS-17 protein
Locus_16511_Transcript_1/1_Conf_1.000	748	20	2.33E-64	AAK68292.3	70	249.595	261	184	U28943_4Clc-type chloride channel protein 3, isoform b, confirmed by transcript evidence
Locus_16512_Transcript_1/1_Conf_1.000	218	0							
Locus_16513_Transcript_1/1_Conf_1.000	255	20	3.22E-24	NP_494384.2	75	114.775	85	64	PaTChed family member (ptc-3)
Locus_16514_Transcript_1/1_Conf_1.000	450	20	1.42E-32	EFO16233.1	67	142.51	140	94	variant SH3 domain-containing protein
Locus_16515_Transcript_1/1_Conf_1.000	273	20	6.56E-30	XP_002753198.1	96	133.65	87	84	PREDICTED: poly(rC)-binding protein 2-like isoform 2
Locus_16516_Transcript_1/1_Conf_1.000	148	0							
Locus_16517_Transcript_1/1_Conf_1.000	803	0							
Locus_16518_Transcript_1/1_Conf_1.000	288	0							
Locus_16519_Transcript_1/1_Conf_1.000	540	20	6.20E-65	XP_002640715.1	83	250.366	178	149	C. briggsae CBR-VPS-28 protein
Locus_1652_Transcript_1/1_Conf_1.000	1166	20	2.22E-133	XP_002633423.1	77	479.945	335	260	C. briggsae CBR-FAT-6 protein
Locus_16520_Transcript_1/1_Conf_1.000	906	20	5.97E-82	EFO25806.1	74	308.531	299	223	hypothetical protein LOAG_02677
Locus_16521_Transcript_1/1_Conf_1.000	597	0							
Locus_16522_Transcript_1/1_Conf_1.000	279	0							

Locus_16523_Transcript_1/1_Conf_1.000	411	5	4.09E-40	NP_506132.1	72	167.548	123	89	hypothetical protein W04D2.4
Locus_16524_Transcript_1/1_Conf_1.000	138	0							
Locus_16525_Transcript_1/1_Conf_1.000	347	20	4.59E-55	EFO25295.1	95	217.238	115	110	STE/STE20/KHS protein kinase
Locus_16526_Transcript_1/1_Conf_1.000	801	20	5.24E-28	XP_001900507.1	49	129.028	241	120	Protein set-2.
Locus_16527_Transcript_1/1_Conf_1.000	261	18	1.39E-11	AAS91567.1	82	72.7886	45	37	transformer-1
Locus_16528_Transcript_1/1_Conf_1.000	148	2	3.36E-05	EFO19039.1	84	51.6026	46	39	Tln1 protein
Locus_16529_Transcript_1/1_Conf_1.000	696	20	1.67E-26	NP_497348.3	78	123.635	94	74	hypothetical protein Y119D3B.12
Locus_1653_Transcript_1/1_Conf_1.000	1106	20	4.52E-64	CAZ65529.1	68	249.595	317	216	C. elegans protein T28C6.7b, partially confirmed by transcript evidence
Locus_16530_Transcript_1/1_Conf_1.000	482	8	5.65E-58	XP_002637142.1	88	226.868	137	121	Hypothetical protein CBG09644
Locus_16531_Transcript_1/1_Conf_1.000	626	20	3.82E-71	NP_491354.1	78	271.552	188	148	AcetylCholine Receptor family member (acr-6)
Locus_16532_Transcript_1/2_Conf_1.000	448	2	4.18E-08	XP_002631722.1	49	61.2326	145	72	C. briggsae CBR-SINH-1 protein
Locus_16532_Transcript_2/2_Conf_1.000	412	1	6.16E-04	XP_002631722.1	54	47.3654	97	53	C. briggsae CBR-SINH-1 protein
Locus_16533_Transcript_1/1_Conf_1.000	377	0							
Locus_16534_Transcript_1/1_Conf_1.000	334	20	3.95E-51	XP_002758725.1	100	204.142	107	107	PREDICTED: dolichyl-diphosphooligosaccharide--protein glycosyltransferase subunit 1-like isoform 2
Locus_16535_Transcript_1/1_Conf_1.000	327	0							
Locus_16536_Transcript_1/1_Conf_1.000	168	0							
Locus_16537_Transcript_1/1_Conf_1.000	148	2	8.26E-12	NP_001022051.1	80	73.559	47	38	SREBP Cleavage activating Protein (SCAP) homolog family member (scp-1)

Locus_16538_Transcript_1/1_Conf_1.000	246	2	2.50E-05	XP_002634825.1	63	51.9878	61	39	Hypothetical protein CBG13933
Locus_16539_Transcript_1/1_Conf_1.000	262	0							
Locus_1654_Transcript_1/1_Conf_1.000	262	0							
Locus_16540_Transcript_1/1_Conf_1.000	177	0							
Locus_16541_Transcript_1/1_Conf_1.000	539	20	2.03E-52	NP_506007.1	83	208.764	143	119	CYclin L family member (cyl-1)
Locus_16542_Transcript_1/1_Conf_1.000	341	6	7.22E-13	EFO19489.1	56	77.0258	113	64	IQ calmodulin-binding domain-containing protein
Locus_16543_Transcript_1/1_Conf_1.000	464	11	1.75E-38	NP_001122650.1	67	162.155	159	107	PaTChed family member (ptc-3)
Locus_16544_Transcript_1/1_Conf_1.000	146	2	2.50E-08	CAD19995.2	82	62.003	39	32	metalloprotease III
Locus_16545_Transcript_1/1_Conf_1.000	278	0							
Locus_16546_Transcript_1/1_Conf_1.000	140	0							
Locus_16547_Transcript_1/1_Conf_1.000	136	4	5.04E-06	XP_001895407.1	79	54.299	43	34	Phospholipase D. Active site motif family protein
Locus_16548_Transcript_1/1_Conf_1.000	225	4	4.66E-07	NP_495463.2	61	57.7658	73	45	hypothetical protein T02G5.2
Locus_16549_Transcript_1/1_Conf_1.000	283	0							
Locus_1655_Transcript_1/1_Conf_1.000	248	0							
Locus_16550_Transcript_1/1_Conf_1.000	343	0							
Locus_16551_Transcript_1/1_Conf_1.000	247	3	2.84E-17	CAO72177.1	69	91.6633	83	58	hexosaminidase
Locus_16552_Transcript_1/1_Conf_1.000	273	0							
Locus_16553_Transcript_1/1_Conf_1.000	243	4	1.57E-07	EFO27452.1	77	59.3066	81	63	hypothetical protein LOAG_01023
Locus_16554_Transcript_1/1_Conf_1.000	348	20	2.68E-55	NP_740906.1	98	218.009	115	113	hypothetical protein F26E4.7
Locus_16555_Transcript_1/2_Conf_1.000	383	0							
Locus_16555_Transcript_2/2_Conf_1.000	393	0							
Locus_16556_Transcript_1/1_Conf_1.000	187	2	1.27E-12	NP_507767.2	88	76.2554	60	53	hypothetical protein C14B4.2

Locus_16557_Transcript_1/1_Conf_1.000	213	20	2.75E-20	NP_491664.1	81	101.679	70	57	UNCoordinated family member (unc-40)
Locus_16558_Transcript_1/1_Conf_1.000	489	0							
Locus_16559_Transcript_1/1_Conf_1.000	396	0							
Locus_1656_Transcript_1/1_Conf_1.000	488	0							
Locus_16560_Transcript_1/1_Conf_1.000	364	0							
Locus_16561_Transcript_1/1_Conf_1.000	230	0							
Locus_16562_Transcript_1/1_Conf_1.000	131	0							
Locus_16563_Transcript_1/1_Conf_1.000	238	20	5.06E-22	P10253.3	100	107.457	52	52	Lysosomal alpha-glucosidase
Locus_16564_Transcript_1/1_Conf_1.000	190	0							
Locus_16565_Transcript_1/1_Conf_1.000	626	20	2.35E-20	XP_002636128.1	79	102.834	88	70	Hypothetical protein CBG01378
Locus_16566_Transcript_1/1_Conf_1.000	792	20	7.54E-72	XP_002637669.1	76	274.633	208	159	Hypothetical protein CBG19425
Locus_16567_Transcript_1/1_Conf_1.000	233	0							

Locus_16568_Transcript_1/1_Conf_1.000	486	20	2.00E-26	XP_002639142.1	55	122.094	168	94	Hypothetical protein CBG14966
Locus_16569_Transcript_1/1_Conf_1.000	267	0							
Locus_1657_Transcript_1/1_Conf_1.000	261	0							
Locus_16570_Transcript_1/1_Conf_1.000	385	20	5.91E-47	NP_505312.1	85	190.274	128	110	hypothetical protein T19A5.1
Locus_16571_Transcript_1/1_Conf_1.000	198	0							
Locus_16572_Transcript_1/1_Conf_1.000	568	20	3.24E-25	EFO22800.1	54	118.627	182	100	hypothetical protein LOAG_05685
Locus_16573_Transcript_1/1_Conf_1.000	170	0							
Locus_16574_Transcript_1/1_Conf_1.000	158	0							
Locus_16575_Transcript_1/1_Conf_1.000	192	0							
Locus_16576_Transcript_1/1_Conf_1.000	457	0							
Locus_16577_Transcript_1/1_Conf_1.000	615	2	2.46E-06	XP_001899613.1	62	56.225	70	44	hypothetical protein Bm1_40770
Locus_16578_Transcript_1/1_Conf_1.000	182	0							
Locus_16579_Transcript_1/1_Conf_1.000	453	20	6.50E-62	AAH62437.1	100	239.965	115	115	Cytochrome c oxidase subunit IV isoform 1
Locus_1658_Transcript_1/1_Conf_1.000	172	0							
Locus_16580_Transcript_1/1_Conf_1.000	282	0							
Locus_16581_Transcript_1/1_Conf_1.000	166	0							
Locus_16582_Transcript_1/1_Conf_1.000	359	20	2.50E-21	NP_491427.1	75	105.145	99	75	hypothetical protein W09C3.4
Locus_16583_Transcript_1/1_Conf_1.000	532	20	8.35E-67	NP_502120.1	80	256.529	177	142	hypothetical protein B0035.16
Locus_16584_Transcript_1/1_Conf_1.000	454	10	9.81E-26	XP_002638736.1	66	119.783	130	87	C. briggsae CBR-EGO-1 protein
Locus_16585_Transcript_1/1_Conf_1.000	502	20	5.47E-32	EFO21215.1	64	140.584	157	101	AMP-binding enzyme family protein

Locus_16586_Transcript_1/1_Conf_1.000	168	20	9.83E-26	XP_002807155.1	100	119.783	55	55	PREDICTED: LOW QUALITY PROTEIN: poly(rC)-binding protein 2-like
Locus_16587_Transcript_1/1_Conf_1.000	177	20	3.03E-27	XP_002760341.1	100	124.79	58	58	PREDICTED: glutamine synthetase
Locus_16588_Transcript_1/1_Conf_1.000	443	0							
Locus_16589_Transcript_1/1_Conf_1.000	293	20	1.87E-40	NP_491513.2	93	168.703	96	90	Formin HOmology Domain family member (fhod-1)
Locus_1659_Transcript_1/1_Conf_1.000	296	0							
Locus_16590_Transcript_1/1_Conf_1.000	277	0							
Locus_16591_Transcript_1/1_Conf_1.000	216	4	2.65E-15	XP_002633968.1	83	85.1149	56	47	C. briggsae CBR-MCA-1 protein
Locus_16592_Transcript_1/1_Conf_1.000	154	0							
Locus_16593_Transcript_1/1_Conf_1.000	546	20	7.66E-42	XP_001190458.1	69	173.711	178	123	PREDICTED: similar to ATP-binding cassette, sub-family C (CFTR/MRP), member 4
Locus_16594_Transcript_1/1_Conf_1.000	134	0							
Locus_16595_Transcript_1/1_Conf_1.000	249	0							
Locus_16596_Transcript_1/1_Conf_1.000	220	0							
Locus_16597_Transcript_1/1_Conf_1.000	267	20	2.27E-30	NP_740900.1	84	135.191	88	74	hypothetical protein T08G11.1
Locus_16598_Transcript_1/1_Conf_1.000	217	0							
Locus_16599_Transcript_1/1_Conf_1.000	226	0							
Locus_166_Transcript_1/1_Conf_1.000	1411	20	4.24E-92	XP_002645826.1	58	343.199	425	250	Hypothetical protein CBG07557
Locus_1660_Transcript_1/3_Conf_0.400	1995	20	0	XP_002638688.1	83	749.969	514	431	Hypothetical protein CBG11883

Locus_1660_Transcript_2/3_Conf_0.600	2103	20	0	XP_002638688.1	83	749.969	514	431	Hypothetical protein CBG11883
Locus_1660_Transcript_3/3_Conf_0.400	1696	20	5.39E-116	BAI79333.1	62	422.935	548	343	mitochondrial elongation factor G2
Locus_16600_Transcript_1/1_Conf_1.000	142	2	5.35E-08	XP_002638375.1	85	60.8474	34	29	Hypothetical protein CBG18580
Locus_16601_Transcript_1/1_Conf_1.000	549	0							
Locus_16602_Transcript_1/1_Conf_1.000	263	0							
Locus_16603_Transcript_1/1_Conf_1.000	294	20	5.83E-18	EFN72462.1	82	93.9745	73	60	Copper homeostasis protein cutC-like protein
Locus_16604_Transcript_1/2_Conf_1.000	825	4	2.99E-50	XP_002631532.1	78	202.986	193	152	Hypothetical protein CBG20698
Locus_16604_Transcript_2/2_Conf_1.000	825	4	6.02E-51	XP_002631532.1	79	205.297	193	153	Hypothetical protein CBG20698
Locus_16605_Transcript_1/2_Conf_1.000	528	0							
Locus_16605_Transcript_2/2_Conf_1.000	542	0							
Locus_16606_Transcript_1/1_Conf_1.000	687	20	6.75E-41	NP_502212.1	60	171.4	225	137	hypothetical protein ZK792.5
Locus_16607_Transcript_1/1_Conf_1.000	450	20	4.01E-19	NP_495338.1	56	97.8265	151	85	hypothetical protein C56C10.9
Locus_16608_Transcript_1/1_Conf_1.000	253	0							
Locus_16609_Transcript_1/2_Conf_1.000	637	0							
Locus_16609_Transcript_2/2_Conf_1.000	637	0							
Locus_1661_Transcript_1/1_Conf_1.000	286	0							
Locus_16610_Transcript_1/1_Conf_1.000	286	0							
Locus_16611_Transcript_1/1_Conf_1.000	285	0							
Locus_16612_Transcript_1/1_Conf_1.000	388	20	3.24E-37	NP_490840.2	76	157.918	130	99	DAP (Death-Associated Protein) Kinase homolog family member (dapk-1)
Locus_16613_Transcript_1/1_Conf_1.000	361	20	1.98E-18	XP_002647178.1	63	95.5153	119	75	Hypothetical protein CBG22337

Locus_16614_Transcript_1/1_Conf_1.000	382	20	2.19E-33	NP_741467.1	81	145.206	94	77	hypothetical protein C09G4.2
Locus_16615_Transcript_1/1_Conf_1.000	368	20	4.81E-33	EFO27702.1	79	144.05	124	98	roadblock/LC7 domain-containing protein
Locus_16616_Transcript_1/1_Conf_1.000	142	0							
Locus_16617_Transcript_1/1_Conf_1.000	314	20	2.63E-50	XP_002927596.1	100	201.445	96	96	PREDICTED: myosin regulatory light polypeptide 9-like
Locus_16618_Transcript_1/1_Conf_1.000	322	0							
Locus_16619_Transcript_1/1_Conf_1.000	133	0							
Locus_1662_Transcript_1/1_Conf_1.000	701	20	3.48E-48	XP_002632763.1	85	195.667	140	119	Hypothetical protein CBG11654
Locus_16620_Transcript_1/1_Conf_1.000	396	0							
Locus_16621_Transcript_1/1_Conf_1.000	381	4	3.74E-25	NP_496802.2	63	117.857	128	81	BTAF (TBP-associated factor) homolog family member (btf-1)
Locus_16622_Transcript_1/1_Conf_1.000	441	20	4.45E-50	EFO26644.1	78	200.675	143	112	hypothetical protein LOAG_01839
Locus_16623_Transcript_1/1_Conf_1.000	821	1	9.29E-04	XP_002635323.1	45	48.521	231	104	Hypothetical protein CBG01490

Locus_16624_Transcript_1/1_Conf_1.000	144	20	1.20E-10	XP_002601536.1	80	69.707	42	34	hypothetical protein BRAFLDRAFT_230606
Locus_16625_Transcript_1/1_Conf_1.000	423	3	1.76E-06	EFO22932.1	47	55.8398	120	57	hypothetical protein LOAG_05551
Locus_16626_Transcript_1/1_Conf_1.000	431	9	5.78E-18	A8X9H4.2	56	93.9745	120	68	Chondroitin proteoglycan 4
Locus_16627_Transcript_1/1_Conf_1.000	156	0							
Locus_16628_Transcript_1/1_Conf_1.000	164	0							
Locus_16629_Transcript_1/1_Conf_1.000	145	0							
Locus_1663_Transcript_1/1_Conf_1.000	2720	20	0	XP_002645952.1	96	1395.56	758	733	C. briggsae CBR-DYN-1 protein
Locus_16630_Transcript_1/1_Conf_1.000	659	20	1.27E-78	NP_508515.2	79	296.59	214	170	AQuaPorin or aquaglyceroporin related family member (aqp-7)
Locus_16631_Transcript_1/1_Conf_1.000	145	0							
Locus_16632_Transcript_1/1_Conf_1.000	519	5	2.68E-11	NP_503141.1	63	72.0182	101	64	hypothetical protein W03F9.10

Locus_16633_Transcript_1/1_Conf_1.000	638	20	2.86E-85	NP_501266.1	89	318.546	201	180	hypothetical protein C50F7.4
Locus_16634_Transcript_1/1_Conf_1.000	226	0							
Locus_16635_Transcript_1/1_Conf_1.000	1290	6	2.91E-52	XP_002631435.1	64	210.69	223	143	Hypothetical protein CBG03297
Locus_16636_Transcript_1/1_Conf_1.000	535	0							
Locus_16637_Transcript_1/1_Conf_1.000	245	0							
Locus_16638_Transcript_1/1_Conf_1.000	315	0							
Locus_16639_Transcript_1/1_Conf_1.000	141	18	2.94E-06	EFO23788.1	76	55.0694	42	32	hypothetical protein LOAG_04700
Locus_1664_Transcript_1/7_Conf_0.167	335	0							
Locus_1664_Transcript_2/7_Conf_0.667	1320	20	8.55E-132	NP_501877.2	80	474.937	362	290	hypothetical protein F01G10.7
Locus_1664_Transcript_3/7_Conf_0.667	1333	20	1.93E-131	NP_501877.2	80	473.781	362	290	hypothetical protein F01G10.7
Locus_1664_Transcript_4/7_Conf_0.556	1048	20	1.11E-125	NP_501877.2	79	454.136	353	282	hypothetical protein F01G10.7
Locus_1664_Transcript_5/7_Conf_0.556	1333	20	2.98E-132	NP_501877.2	80	476.478	362	290	hypothetical protein F01G10.7

Locus_1664_Transcript_6/7_Conf_0.611	1333	20	1.02E-132	NP_501877.2	80	478.019	362	291	hypothetical protein F01G10.7
Locus_1664_Transcript_7/7_Conf_0.667	1333	20	8.67E-132	NP_501877.2	80	474.937	362	290	hypothetical protein F01G10.7
Locus_16640_Transcript_1/1_Conf_1.000	475	20	4.07E-64	XP_002633392.1	85	247.284	158	135	C. briggsae CBR-WHT-2 protein
Locus_16641_Transcript_1/1_Conf_1.000	245	0							
Locus_16642_Transcript_1/1_Conf_1.000	617	2	1.99E-56	NP_001024385.1	77	222.631	192	148	hypothetical protein C09F12.3
Locus_16643_Transcript_1/1_Conf_1.000	345	20	1.18E-18	NP_740939.1	81	96.2857	72	59	hypothetical protein Y105E8A.11
Locus_16644_Transcript_1/1_Conf_1.000	270	2	2.69E-07	XP_002646257.1	58	58.5362	91	53	Hypothetical protein CBG11961
Locus_16645_Transcript_1/2_Conf_1.000	368	1	5.00E-14	CAR63617.1	79	80.8777	64	51	hypothetical protein
Locus_16645_Transcript_2/2_Conf_1.000	368	1	5.00E-14	CAR63617.1	79	80.8777	64	51	hypothetical protein
Locus_16646_Transcript_1/1_Conf_1.000	507	20	5.06E-12	EFO27069.1	65	74.3294	70	46	Ulp1 protease
Locus_16647_Transcript_1/1_Conf_1.000	216	20	5.34E-16	XP_002639908.1	72	87.4261	66	48	Hypothetical protein CBG08236
Locus_16648_Transcript_1/1_Conf_1.000	484	20	5.81E-50	NP_001129769.1	76	200.29	156	120	DAP (Death Associated Protein kinase) Like Kinase family member (dlk-1)

Locus_16649_Transcript_1/1_Conf_1.000	550	20	8.09E-31	XP_001897975.1	59	137.117	176	104	SOH1 family protein
Locus_1665_Transcript_1/1_Conf_1.000	545	4	2.10E-68	XP_002631447.1	86	261.922	160	139	Hypothetical protein CBG03309
Locus_16650_Transcript_1/1_Conf_1.000	271	0							
Locus_16651_Transcript_1/1_Conf_1.000	1146	20	1.43E-52	NP_509671.3	59	211.46	300	178	hypothetical protein ZC506.1
Locus_16652_Transcript_1/1_Conf_1.000	329	4	1.79E-43	NP_001022254.1	88	178.718	104	92	hypothetical protein M05D6.9
Locus_16653_Transcript_1/1_Conf_1.000	246	20	1.23E-28	XP_002634963.1	89	129.413	73	65	C. briggsae CBR-DIS-3 protein
Locus_16654_Transcript_1/1_Conf_1.000	445	0							
Locus_16655_Transcript_1/1_Conf_1.000	647	20	6.70E-45	XP_002661818.1	64	184.496	212	137	PREDICTED: Gap-Pol polyprotein-like
Locus_16656_Transcript_1/1_Conf_1.000	491	0							
Locus_16657_Transcript_1/1_Conf_1.000	269	20	4.02E-19	XP_002636816.1	61	97.8265	86	53	Hypothetical protein CBG09260
Locus_16658_Transcript_1/1_Conf_1.000	213	0							
Locus_16659_Transcript_1/1_Conf_1.000	142	0							
Locus_1666_Transcript_1/1_Conf_1.000	280	20	1.56E-15	XP_002634963.1	64	85.8853	93	60	C. briggsae CBR-DIS-3 protein
Locus_16660_Transcript_1/1_Conf_1.000	372	20	7.39E-21	NP_509237.1	69	103.605	102	71	SaPosin-like Protein family member (spp-4)
Locus_16661_Transcript_1/1_Conf_1.000	270	0							
Locus_16662_Transcript_1/1_Conf_1.000	301	0							
Locus_16663_Transcript_1/1_Conf_1.000	162	0							
Locus_16664_Transcript_1/1_Conf_1.000	155	2	8.79E-06	EFO27087.1	74	53.5286	51	38	hypothetical protein LOAG_01392
Locus_16665_Transcript_1/1_Conf_1.000	139	6	3.36E-10	NP_498641.1	83	68.1662	42	35	hypothetical protein H14A12.3
Locus_16666_Transcript_1/1_Conf_1.000	175	0							

Locus_16667_Transcript_1/1_Conf_1.000	504	20	8.23E-92	2I07	100	339.347	159	159	Human Complement Component C3b
Locus_16668_Transcript_1/1_Conf_1.000	575	20	9.63E-65	NP_504498.2	76	249.98	193	148	hypothetical protein F25B4.7
Locus_16669_Transcript_1/1_Conf_1.000	152	20	6.29E-20	NP_871645.1	98	100.523	50	49	hypothetical protein C28H8.11
Locus_1667_Transcript_1/1_Conf_1.000	1874	20	3.31E-154	XP_002643742.1	70	550.051	582	409	Hypothetical protein CBG01937
Locus_16670_Transcript_1/1_Conf_1.000	575	0							
Locus_16671_Transcript_1/1_Conf_1.000	590	5	1.30E-27	CAB04569.3	60	126.716	182	110	C. elegans protein K05C4.5, confirmed by transcript evidence
Locus_16672_Transcript_1/1_Conf_1.000	560	20	1.73E-52	NP_490726.1	77	209.149	179	139	hypothetical protein R119.3

Locus_16673_Transcript_1/1_Conf_1.000	260	0							
Locus_16674_Transcript_1/1_Conf_1.000	426	20	3.37E-50	XP_001897105.1	81	201.06	133	109	protein phosphatase 2A, regulatory subunit B'
Locus_16675_Transcript_1/1_Conf_1.000	140	6	2.49E-05	EFO25333.1	62	51.9878	48	30	hypothetical protein LOAG_03154
Locus_16676_Transcript_1/1_Conf_1.000	409	0							
Locus_16677_Transcript_1/1_Conf_1.000	221	13	5.91E-18	ACV31107.1	85	93.9745	67	57	Hypothetical protein F22D3.2b
Locus_16678_Transcript_1/2_Conf_1.000	546	20	1.35E-46	EFO28378.1	78	189.504	137	107	mediator complex subunit 31
Locus_16678_Transcript_2/2_Conf_1.000	545	20	1.01E-46	EFO28378.1	78	189.889	137	107	mediator complex subunit 31
Locus_16679_Transcript_1/1_Conf_1.000	923	10	1.19E-101	EFO20752.1	75	374.015	292	219	AMOP domain-containing protein
Locus_1668_Transcript_1/2_Conf_1.000	1303	20	9.77E-64	NP_492076.1	68	248.825	302	207	hypothetical protein F52A8.6
Locus_1668_Transcript_2/2_Conf_1.000	1116	20	5.62E-62	NP_001040668.1	81	242.662	200	163	hypothetical protein F52A8.6
Locus_16680_Transcript_1/1_Conf_1.000	230	20	9.29E-32	NP_502751.1	89	139.813	76	68	TRAnsformer : XX animals transformed into males family member (tra-3)
Locus_16681_Transcript_1/1_Conf_1.000	650	0							

Locus_16682_Transcript_1/1_Conf_1.000	197	0							
Locus_16683_Transcript_1/1_Conf_1.000	275	20	8.53E-46	XP_002834824.1	100	186.422	91	91	PREDICTED: LOW QUALITY PROTEIN: guanine nucleotide-binding protein subunit beta-2-like 1-like
Locus_16684_Transcript_1/1_Conf_1.000	284	20	9.72E-29	EFO16325.1	69	129.798	100	69	hypothetical protein LOAG_12180
Locus_16685_Transcript_1/1_Conf_1.000	507	20	3.05E-33	EFO21515.1	64	144.821	170	109	guanylate kinase
Locus_16686_Transcript_1/1_Conf_1.000	206	2	1.29E-09	NP_495911.3	63	66.2402	66	42	hypothetical protein C18E9.5
Locus_16687_Transcript_1/1_Conf_1.000	286	0							
Locus_16688_Transcript_1/1_Conf_1.000	1233	20	9.46E-37	XP_001892593.1	54	159.073	340	185	KH domain containing protein
Locus_16689_Transcript_1/1_Conf_1.000	429	0							
Locus_1669_Transcript_1/2_Conf_1.000	1412	20	9.12E-95	NP_497100.1	63	352.058	419	264	human Activating Signal Cointegrator homolog family member (asc-1)
Locus_1669_Transcript_2/2_Conf_1.000	1412	20	9.12E-95	NP_497100.1	63	352.058	419	264	human Activating Signal Cointegrator homolog family member (asc-1)
Locus_16690_Transcript_1/1_Conf_1.000	210	20	2.85E-17	XP_002645317.1	75	91.6633	69	52	C. briggsae CBR-TRK-1 protein
Locus_16691_Transcript_1/1_Conf_1.000	346	20	4.31E-45	CAR63665.1	92	184.111	98	91	hypothetical protein
Locus_16692_Transcript_1/1_Conf_1.000	132	20	2.87E-09	A8XJL7.2	88	65.0846	43	38	Serine/threonine-protein kinase sax-1
Locus_16693_Transcript_1/1_Conf_1.000	181	0							
Locus_16694_Transcript_1/1_Conf_1.000	375	0							
Locus_16695_Transcript_1/1_Conf_1.000	568	20	5.69E-38	EFO25439.1	70	160.999	161	113	srpr protein
Locus_16696_Transcript_1/1_Conf_1.000	246	0							
Locus_16697_Transcript_1/1_Conf_1.000	291	0							
Locus_16698_Transcript_1/1_Conf_1.000	280	0							

Locus_16699_Transcript_1/1_Conf_1.000	301	20	3.99E-35	EFO21757.1	85	150.984	99	85	hypothetical protein LOAG_06731
Locus_167_Transcript_1/1_Conf_1.000	243	0							
Locus_1670_Transcript_1/1_Conf_1.000	195	13	2.15E-12	NP_493741.1	71	75.485	59	42	hypothetical protein W10D9.5
Locus_16700_Transcript_1/1_Conf_1.000	235	0							
Locus_16701_Transcript_1/1_Conf_1.000	146	20	6.35E-12	NP_510249.2	91	73.9442	36	33	TREhalase family member (tre-4)
Locus_16702_Transcript_1/1_Conf_1.000	255	0							
Locus_16703_Transcript_1/2_Conf_1.000	264	0							
Locus_16703_Transcript_2/2_Conf_1.000	131	0							
Locus_16704_Transcript_1/1_Conf_1.000	220	20	3.12E-35	ACC44768.1	100	151.369	72	72	APA-2
Locus_16705_Transcript_1/1_Conf_1.000	343	3	1.55E-10	NP_001021445.1	60	69.3218	106	64	DAP (Death Associated Protein kinase) Like Kinase family member (dlk-1)
Locus_16706_Transcript_1/1_Conf_1.000	284	0							
Locus_16707_Transcript_1/1_Conf_1.000	197	0							
Locus_16708_Transcript_1/1_Conf_1.000	330	20	1.16E-34	NP_496959.1	89	149.443	107	96	EATing: abnormal pharyngeal pumping family member (eat-2)

Locus_16709_Transcript_1/1_Conf_1.000	533	6	7.18E-10	NP_001021054.1	50	67.3958	174	87	Suppressor with Morphological effect on Genitalia family member (smg-1)
Locus_1671_Transcript_1/1_Conf_1.000	215	1	1.24E-04	XP_002405836.1	64	49.6766	34	22	conserved hypothetical protein
Locus_16710_Transcript_1/1_Conf_1.000	148	0							
Locus_16711_Transcript_1/1_Conf_1.000	727	2	5.20E-66	XP_002639917.1	73	254.988	241	176	C. briggsae CBR-SMGL-1 protein
Locus_16712_Transcript_1/1_Conf_1.000	160	5	2.80E-12	EFO19924.1	79	75.0998	53	42	hypothetical protein LOAG_08567
Locus_16713_Transcript_1/1_Conf_1.000	303	0							
Locus_16714_Transcript_1/1_Conf_1.000	151	0							
Locus_16715_Transcript_1/1_Conf_1.000	354	0							
Locus_16716_Transcript_1/1_Conf_1.000	337	20	1.45E-37	NP_492905.1	97	159.073	88	86	hypothetical protein H25P06.1
Locus_16717_Transcript_1/1_Conf_1.000	302	20	1.57E-31	XP_001152859.1	98	139.043	67	66	PREDICTED: hypothetical protein
Locus_16718_Transcript_1/1_Conf_1.000	495	0							
Locus_16719_Transcript_1/1_Conf_1.000	147	20	5.92E-10	XP_001896664.1	81	67.3958	48	39	Protein kinase domain containing protein
Locus_1672_Transcript_1/1_Conf_1.000	490	0							
Locus_16720_Transcript_1/1_Conf_1.000	134	0							
Locus_16721_Transcript_1/1_Conf_1.000	396	20	1.82E-27	EFO21962.1	58	125.561	130	76	TK/EGFR protein kinase
Locus_16722_Transcript_1/1_Conf_1.000	174	0							
Locus_16723_Transcript_1/1_Conf_1.000	396	20	3.18E-56	AAA36105.1	97	221.09	106	103	gamma-interferon-inducible protein precursor
Locus_16724_Transcript_1/1_Conf_1.000	137	0							
Locus_16725_Transcript_1/1_Conf_1.000	166	0							
Locus_16726_Transcript_1/1_Conf_1.000	384	20	3.84E-38	NP_507509.3	79	160.999	126	100	hypothetical protein C06H5.6
Locus_16727_Transcript_1/1_Conf_1.000	482	0							
Locus_16728_Transcript_1/1_Conf_1.000	235	4	1.64E-12	NP_872065.1	82	75.8702	45	37	hypothetical protein F35C11.7

Locus_16729_Transcript_1/1_Conf_1.000	495	20	2.50E-21	NP_493658.2	70	105.145	131	93	hypothetical protein C50D2.8
Locus_1673_Transcript_1/1_Conf_1.000	907	0							
Locus_16730_Transcript_1/1_Conf_1.000	690	0							
Locus_16731_Transcript_1/1_Conf_1.000	178	0							
Locus_16732_Transcript_1/1_Conf_1.000	460	20	6.55E-06	XP_392160.2	48	53.9138	134	65	PREDICTED: similar to ubiquitin specific protease 3
Locus_16733_Transcript_1/1_Conf_1.000	341	20	3.00E-51	XP_001509789.1	100	204.527	112	112	PREDICTED: hypothetical protein, partial
Locus_16734_Transcript_1/1_Conf_1.000	237	0							
Locus_16735_Transcript_1/1_Conf_1.000	262	4	1.44E-16	NP_001122565.1	60	89.3521	107	65	Variable ABnormal morphology family member (vab-10)
Locus_16736_Transcript_1/2_Conf_1.000	619	3	2.17E-26	NP_491462.2	71	122.865	92	66	C-type LECtin family member (clec-90)
Locus_16736_Transcript_2/2_Conf_1.000	619	3	4.84E-26	NP_491462.2	71	121.709	92	66	C-type LECtin family member (clec-90)
Locus_16737_Transcript_1/1_Conf_1.000	309	0							
Locus_16738_Transcript_1/1_Conf_1.000	332	0							
Locus_16739_Transcript_1/1_Conf_1.000	1007	3	1.19E-28	NP_492325.2	49	131.724	308	153	hypothetical protein R05D11.6
Locus_1674_Transcript_1/1_Conf_1.000	843	0							
Locus_16740_Transcript_1/1_Conf_1.000	177	2	1.16E-10	NP_495620.1	75	69.707	58	44	hypothetical protein B0495.9
Locus_16741_Transcript_1/1_Conf_1.000	152	0							
Locus_16742_Transcript_1/1_Conf_1.000	250	0							
Locus_16743_Transcript_1/1_Conf_1.000	168	0							
Locus_16744_Transcript_1/1_Conf_1.000	322	20	1.30E-25	XP_001894170.1	66	119.398	106	70	Ribonucleases P/MRP protein subunit POP1 containing protein
Locus_16745_Transcript_1/1_Conf_1.000	174	0							

Locus_16746_Transcript_1/1_Conf_1.000	258	20	8.70E-38	XP_001898882.1	92	159.844	85	79	casein kinase 1, delta
Locus_16747_Transcript_1/1_Conf_1.000	1057	20	2.17E-153	XP_001901272.1	91	546.199	352	322	DEAD
Locus_16748_Transcript_1/1_Conf_1.000	240	20	7.82E-07	EFN81401.1	65	56.9954	61	40	Putative AC transposase
Locus_16749_Transcript_1/1_Conf_1.000	264	0							
Locus_1675_Transcript_1/1_Conf_1.000	230	1	1.49E-05	ACV86995.1	74	52.7582	39	29	gut-associated larval antigen-1
Locus_16750_Transcript_1/1_Conf_1.000	191	0							
Locus_16751_Transcript_1/1_Conf_1.000	293	0							
Locus_16752_Transcript_1/1_Conf_1.000	145	0							
Locus_16753_Transcript_1/1_Conf_1.000	364	20	1.15E-26	NP_506077.1	70	122.865	121	85	hypothetical protein F55C5.2
Locus_16754_Transcript_1/1_Conf_1.000	192	0							
Locus_16755_Transcript_1/1_Conf_1.000	150	0							
Locus_16756_Transcript_1/1_Conf_1.000	518	3	6.24E-05	ZP_07577412.1	48	50.8322	131	64	chromosome segregation protein SMC
Locus_16757_Transcript_1/1_Conf_1.000	138	20	1.36E-19	XP_002812342.1	100	99.3673	45	45	PREDICTED: ornithine decarboxylase-like
Locus_16758_Transcript_1/1_Conf_1.000	173	0							
Locus_16759_Transcript_1/1_Conf_1.000	1278	20	2.29E-17	NP_492467.1	55	94.7449	219	122	mammalian ASPM (Abnormal SPindles and primary Microcephaly) homolog family member (aspm-1)
Locus_1676_Transcript_1/1_Conf_1.000	1539	20	0	NP_491363.1	88	835.869	513	455	Dynein Heavy Chain family member (dhc-1)
Locus_16760_Transcript_1/1_Conf_1.000	174	0							

Locus_16761_Transcript_1/1_Conf_1.000	704	20	2.18E-82	XP_002645723.1	81	309.301	222	180	C. briggsae CBR-GCK-4 protein
Locus_16762_Transcript_1/1_Conf_1.000	300	6	1.37E-27	XP_541499.2	89	125.946	75	67	PREDICTED: similar to Orphan sodium- and chloride-dependent neurotransmitter transporter NTT5 (Solute carrier family 6 member 16)
Locus_16763_Transcript_1/1_Conf_1.000	188	0							
Locus_16764_Transcript_1/1_Conf_1.000	226	4	2.92E-09	XP_001899164.1	67	65.0846	62	42	Leucine Rich Repeat family protein
Locus_16765_Transcript_1/1_Conf_1.000	311	0							
Locus_16766_Transcript_1/1_Conf_1.000	1066	20	1.13E-149	XP_002641970.1	87	533.872	354	309	Hypothetical protein CBG16677
Locus_16767_Transcript_1/1_Conf_1.000	465	20	2.62E-18	EFO24621.1	66	95.1301	101	67	DNA repair protein Rad4 containing protein
Locus_16768_Transcript_1/1_Conf_1.000	540	20	2.30E-27	XP_002633766.1	70	125.561	151	106	Hypothetical protein CBG03455
Locus_16769_Transcript_1/1_Conf_1.000	263	2	1.49E-05	EFO27866.1	52	52.7582	89	47	GTPase activating protein and VPS9 domains 1
Locus_1677_Transcript_1/1_Conf_1.000	935	0							
Locus_16770_Transcript_1/1_Conf_1.000	145	20	6.33E-20	XP_522295.2	100	100.523	48	48	PREDICTED: T-cell, immune regulator 1
Locus_16771_Transcript_1/1_Conf_1.000	2206	11	2.15E-06	EDV09471.1	33	59.3066	520	173	conserved hypothetical protein
Locus_16772_Transcript_1/1_Conf_1.000	165	0							
Locus_16773_Transcript_1/1_Conf_1.000	971	0							
Locus_16774_Transcript_1/1_Conf_1.000	463	0							

Locus_16775_Transcript_1/1_Conf_1.000	197	20	3.30E-29	CAG33263.1	100	131.339	65	65	PSMB10
Locus_16776_Transcript_1/1_Conf_1.000	400	0							
Locus_16777_Transcript_1/1_Conf_1.000	147	20	3.96E-14	NP_495614.3	87	81.2629	49	43	Na/H eXchanger family member (nhx-2)
Locus_16778_Transcript_1/1_Conf_1.000	286	0							
Locus_16779_Transcript_1/1_Conf_1.000	368	0							
Locus_1678_Transcript_1/1_Conf_1.000	372	20	8.73E-22	XP_001901465.1	65	106.686	123	80	steroid hormone receptor
Locus_16780_Transcript_1/1_Conf_1.000	160	0							
Locus_16781_Transcript_1/1_Conf_1.000	309	1	2.76E-04	XP_001648563.1	58	48.521	72	42	bone morphogenetic protein receptor type II, putative
Locus_16782_Transcript_1/1_Conf_1.000	353	2	1.34E-06	XP_001894613.1	53	56.225	95	51	hypothetical protein
Locus_16783_Transcript_1/1_Conf_1.000	456	0							
Locus_16784_Transcript_1/1_Conf_1.000	478	0							
Locus_16785_Transcript_1/1_Conf_1.000	232	0							
Locus_16786_Transcript_1/1_Conf_1.000	196	0							
Locus_16787_Transcript_1/1_Conf_1.000	339	0							

Locus_16788_Transcript_1/1_Conf_1.000	717	20	2.08E-19	NP_001024724.2	49	100.138	202	99	EGg Laying defective family member (egl-15)
Locus_16789_Transcript_1/1_Conf_1.000	407	0							
Locus_1679_Transcript_1/2_Conf_1.000	1523	0							
Locus_1679_Transcript_2/2_Conf_1.000	1286	0							
Locus_16790_Transcript_1/1_Conf_1.000	295	20	2.71E-15	CBA11612.1	53	85.1149	104	56	C. elegans protein F54F3.1b, partially confirmed by transcript evidence
Locus_16791_Transcript_1/1_Conf_1.000	305	2	9.16E-32	NP_499205.1	73	139.813	101	74	hypothetical protein K03H1.5

Locus_16792_Transcript_1/1_Conf_1.000	256	0							
Locus_16793_Transcript_1/1_Conf_1.000	156	20	1.40E-11	CBL43465.1	88	72.7886	51	45	C. elegans protein Y55D9A.1d, confirmed by transcript evidence
Locus_16794_Transcript_1/1_Conf_1.000	333	0							
Locus_16795_Transcript_1/1_Conf_1.000	228	11	1.11E-08	NP_001024812.1	76	63.1586	38	29	hypothetical protein M163.8
Locus_16796_Transcript_1/1_Conf_1.000	369	20	8.72E-38	NP_741200.1	78	159.844	123	97	DIsplaced Gonad family member (dig-1)
Locus_16797_Transcript_1/1_Conf_1.000	188	20	1.40E-27	XP_002945331.1	100	125.946	62	62	PREDICTED: heat shock cognate 71 kDa protein-like, partial
Locus_16798_Transcript_1/1_Conf_1.000	175	0							
Locus_16799_Transcript_1/1_Conf_1.000	311	20	4.36E-34	XP_001897050.1	80	147.517	103	83	Endonuclease III-like protein 1
Locus_168_Transcript_1/3_Conf_0.273	427	0							
Locus_168_Transcript_2/3_Conf_0.364	569	0							
Locus_168_Transcript_3/3_Conf_0.818	764	7	8.08E-12	XP_001895031.1	71	75.0998	57	41	hypothetical protein Bm1_17870
Locus_1680_Transcript_1/1_Conf_1.000	1281	20	5.50E-120	XP_002646302.1	68	435.647	416	284	Hypothetical protein CBG12009
Locus_16800_Transcript_1/1_Conf_1.000	283	0							
Locus_16801_Transcript_1/1_Conf_1.000	144	0							
Locus_16802_Transcript_1/1_Conf_1.000	420	20	3.21E-16	ADI61822.1	58	88.1965	92	54	endonuclease-reverse transcriptase
Locus_16803_Transcript_1/1_Conf_1.000	252	0							
Locus_16804_Transcript_1/1_Conf_1.000	267	20	2.96E-38	XP_002632657.1	90	161.384	86	78	C. briggsae CBR-TRA-3 protein
Locus_16805_Transcript_1/1_Conf_1.000	322	20	1.26E-12	EFO18611.1	71	76.2554	71	51	hypothetical protein LOAG_09883
Locus_16806_Transcript_1/1_Conf_1.000	156	0							
Locus_16807_Transcript_1/1_Conf_1.000	184	1	6.35E-04	XP_002636985.1	61	47.3654	52	32	Hypothetical protein CBG09473
Locus_16808_Transcript_1/1_Conf_1.000	555	5	2.69E-45	XP_002645672.1	71	185.267	184	132	Hypothetical protein CBG07319
Locus_16809_Transcript_1/1_Conf_1.000	310	0							
Locus_1681_Transcript_1/1_Conf_1.000	140	2	7.75E-07	XP_002630428.1	75	56.9954	41	31	Hypothetical protein CBG11154

Locus_16810_Transcript_1/1_Conf_1.000	301	2	9.58E-05	NP_498634.1	51	50.0618	97	50	hypothetical protein T20B12.3
Locus_16811_Transcript_1/1_Conf_1.000	129	0							
Locus_16812_Transcript_1/1_Conf_1.000	851	5	2.07E-17	XP_001893623.1	48	93.9745	283	137	hypothetical protein Bm1_10755
Locus_16813_Transcript_1/1_Conf_1.000	363	0							
Locus_16814_Transcript_1/1_Conf_1.000	655	19	1.01E-27	NP_495774.1	51	127.487	199	103	hypothetical protein F14E5.4
Locus_16815_Transcript_1/1_Conf_1.000	249	20	1.45E-29	AAV40104.1	89	132.494	82	73	immunoglobulin mu heavy chain
Locus_16816_Transcript_1/1_Conf_1.000	136	0							
Locus_16817_Transcript_1/1_Conf_1.000	166	0							
Locus_16818_Transcript_1/1_Conf_1.000	171	19	1.29E-09	NP_504227.3	82	66.2402	40	33	hypothetical protein Y47D7A.14
Locus_16819_Transcript_1/1_Conf_1.000	173	20	3.61E-20	NP_498777.2	92	101.293	57	53	hypothetical protein C14B9.8
Locus_1682_Transcript_1/1_Conf_1.000	1194	3	4.08E-05	XP_001953944.1	37	53.9138	312	118	GF18021
Locus_16820_Transcript_1/1_Conf_1.000	404	6	3.91E-06	XP_002639968.1	51	54.6842	106	55	Hypothetical protein CBG10792
Locus_16821_Transcript_1/1_Conf_1.000	235	0							
Locus_16822_Transcript_1/1_Conf_1.000	407	2	1.02E-06	NP_505659.1	59	56.6102	54	32	hypothetical protein K07C5.3
Locus_16823_Transcript_1/1_Conf_1.000	272	0							
Locus_16824_Transcript_1/1_Conf_1.000	591	4	4.14E-13	EFO18237.1	51	78.5666	198	101	hypothetical protein LOAG_10257
Locus_16825_Transcript_1/1_Conf_1.000	301	0							
Locus_16826_Transcript_1/1_Conf_1.000	188	20	2.40E-11	ZP_04173150.1	65	72.0182	60	39	LPXTG-motif cell wall anchor domain protein
Locus_16827_Transcript_1/1_Conf_1.000	310	0							
Locus_16828_Transcript_1/1_Conf_1.000	642	0							
Locus_16829_Transcript_1/1_Conf_1.000	501	3	2.24E-09	NP_490949.3	79	65.4698	54	43	ABC Transporter family member (abt-2)

Locus_1683_Transcript_1/1_Conf_1.000	642	20	5.27E-26	NP_499343.2	80	121.709	88	71	hypothetical protein Y39A1A.8
Locus_16830_Transcript_1/1_Conf_1.000	371	1	6.44E-49	AAG23172.1	100	196.823	103	103	AF269289_1unknown
Locus_16831_Transcript_1/1_Conf_1.000	381	20	1.11E-69	BAG58223.1	100	265.774	126	126	unnamed protein product
Locus_16832_Transcript_1/2_Conf_1.000	327	0							
Locus_16832_Transcript_2/2_Conf_1.000	281	0							
Locus_16833_Transcript_1/1_Conf_1.000	567	2	5.74E-06	EFO22036.1	45	54.6842	140	64	hypothetical protein LOAG_06451
Locus_16834_Transcript_1/1_Conf_1.000	154	0							
Locus_16835_Transcript_1/1_Conf_1.000	237	0							
Locus_16836_Transcript_1/1_Conf_1.000	272	0							
Locus_16837_Transcript_1/1_Conf_1.000	174	0							
Locus_16838_Transcript_1/1_Conf_1.000	318	20	1.22E-23	NP_001122601.1	79	112.849	92	73	Multiple PDZ domain protein family member (mpz-1)
Locus_16839_Transcript_1/1_Conf_1.000	165	20	6.00E-15	CBL43465.1	90	83.9593	54	49	C. elegans protein Y55D9A.1d, confirmed by transcript evidence
Locus_1684_Transcript_1/1_Conf_1.000	482	0							

Locus_16840_Transcript_1/1_Conf_1.000	270	5	1.09E-08	XP_002630534.1	62	63.1586	82	51	Hypothetical protein CBG12974
Locus_16841_Transcript_1/1_Conf_1.000	246	0							
Locus_16842_Transcript_1/1_Conf_1.000	242	0							
Locus_16843_Transcript_1/1_Conf_1.000	668	20	8.94E-80	NP_490949.3	84	300.442	216	182	ABC Transporter family member (abt-2)
Locus_16844_Transcript_1/2_Conf_1.000	791	20	2.44E-101	NP_491572.2	92	227.254	128	118	AdaPtin, Mu/medium chain (clathrin associated complex) family member (apm-1)
Locus_16844_Transcript_2/2_Conf_1.000	763	20	3.30E-106	NP_491572.2	92	388.652	227	210	AdaPtin, Mu/medium chain (clathrin associated complex) family member (apm-1)
Locus_16845_Transcript_1/1_Conf_1.000	128	0							
Locus_16846_Transcript_1/1_Conf_1.000	372	20	2.69E-47	NP_001122835.1	90	191.43	124	112	UNCoordinated family member (unc-22)
Locus_16847_Transcript_1/1_Conf_1.000	136	0							
Locus_16848_Transcript_1/1_Conf_1.000	281	0							
Locus_16849_Transcript_1/1_Conf_1.000	398	20	5.85E-26	NP_505052.2	69	120.553	114	79	hypothetical protein F26D11.1
Locus_1685_Transcript_1/1_Conf_1.000	239	0							
Locus_16850_Transcript_1/1_Conf_1.000	405	11	4.43E-18	NP_499495.1	66	94.3597	124	82	hypothetical protein Y66D12A.7
Locus_16851_Transcript_1/1_Conf_1.000	180	0							
Locus_16852_Transcript_1/1_Conf_1.000	228	2	1.03E-06	XP_002639895.1	74	56.6102	47	35	Hypothetical protein CBG08217

Locus_16853_Transcript_1/1_Conf_1.000	330	20	3.36E-42	NP_508504.2	87	174.481	113	99	Non-muscle MYosin family member (nmy-1)
Locus_16854_Transcript_1/1_Conf_1.000	668	20	1.78E-128	CAI40836.1	100	462.225	222	222	prosaposin
Locus_16855_Transcript_1/1_Conf_1.000	132	0							
Locus_16856_Transcript_1/1_Conf_1.000	311	0							
Locus_16857_Transcript_1/1_Conf_1.000	356	20	5.07E-22	NP_496542.1	64	107.457	117	76	hypothetical protein ZK930.1
Locus_16858_Transcript_1/1_Conf_1.000	176	0							
Locus_16859_Transcript_1/1_Conf_1.000	734	20	1.01E-32	NP_503501.1	85	144.436	99	85	DeHydrogenases, Short chain family member (dhs-13)
Locus_1686_Transcript_1/1_Conf_1.000	572	0							
Locus_16860_Transcript_1/1_Conf_1.000	267	20	2.14E-12	EFN74509.1	61	75.485	83	51	hypothetical protein EAG_13603

Locus_16861_Transcript_1/1_Conf_1.000	354	20	3.16E-40	NP_493945.1	83	167.933	116	97	hypothetical protein Y57G7A.10
Locus_16862_Transcript_1/1_Conf_1.000	145	0							
Locus_16863_Transcript_1/1_Conf_1.000	290	0							
Locus_16864_Transcript_1/1_Conf_1.000	233	0							
Locus_16865_Transcript_1/1_Conf_1.000	137	0							
Locus_16866_Transcript_1/1_Conf_1.000	677	4	1.15E-13	XP_001897722.1	50	80.8777	153	78	hypothetical protein Bm1_31385
Locus_16867_Transcript_1/1_Conf_1.000	143	1	7.98E-12	AAK21295.1	100	73.559	35	35	NM-4
Locus_16868_Transcript_1/1_Conf_1.000	392	0							
Locus_16869_Transcript_1/1_Conf_1.000	426	0							
Locus_1687_Transcript_1/1_Conf_1.000	173	0							
Locus_16870_Transcript_1/1_Conf_1.000	207	0							
Locus_16871_Transcript_1/1_Conf_1.000	828	20	4.30E-89	XP_002645934.1	76	332.028	277	212	Hypothetical protein CBG07699
Locus_16872_Transcript_1/1_Conf_1.000	425	20	8.91E-51	XP_002633498.1	89	202.986	126	113	Hypothetical protein CBG06270
Locus_16873_Transcript_1/1_Conf_1.000	244	0							
Locus_16874_Transcript_1/1_Conf_1.000	1032	9	9.19E-32	NP_001023892.1	50	142.124	258	130	HIF (hypoxia inducible factor) homolog family member (hif-1)
Locus_16875_Transcript_1/1_Conf_1.000	1322	20	1.40E-49	EFO24754.1	56	201.83	334	190	hypothetical protein LOAG_03725
Locus_16876_Transcript_1/2_Conf_1.000	406	20	3.86E-38	CBK19479.1	88	160.999	96	85	C. elegans protein W08D2.5b, confirmed by transcript evidence
Locus_16876_Transcript_2/2_Conf_1.000	360	20	3.83E-38	CBK19479.1	88	160.999	96	85	C. elegans protein W08D2.5b, confirmed by transcript evidence
Locus_16877_Transcript_1/1_Conf_1.000	201	0							
Locus_16878_Transcript_1/1_Conf_1.000	154	0							
Locus_16879_Transcript_1/1_Conf_1.000	332	20	7.52E-26	XP_002629741.1	78	120.168	71	56	Hypothetical protein CBG00975
Locus_1688_Transcript_1/2_Conf_1.000	690	0							
Locus_1688_Transcript_2/2_Conf_1.000	684	0							

Locus_16880_Transcript_1/3_Conf_0.600	412	3	2.50E-13	XP_002647980.1	75	78.5666	60	45	C. briggsae CBR-PUS-1 protein
Locus_16880_Transcript_2/3_Conf_0.400	433	3	2.50E-13	XP_002647980.1	75	78.5666	60	45	C. briggsae CBR-PUS-1 protein
Locus_16880_Transcript_3/3_Conf_0.600	503	3	2.55E-13	XP_002647980.1	75	78.5666	60	45	C. briggsae CBR-PUS-1 protein
Locus_16881_Transcript_1/1_Conf_1.000	285	20	8.49E-25	ACO14988.1	86	116.701	69	60	Transcription elongation factor B polypeptide 1
Locus_16882_Transcript_1/1_Conf_1.000	577	20	5.40E-47	NP_495087.1	70	191.045	171	121	hypothetical protein C17G10.2
Locus_16883_Transcript_1/1_Conf_1.000	220	6	4.84E-12	NP_492113.2	75	74.3294	54	41	CHitin Synthase family member (chs-1)
Locus_16884_Transcript_1/1_Conf_1.000	527	3	4.94E-08	XP_001900523.1	53	61.2326	104	56	RWD domain containing protein
Locus_16885_Transcript_1/1_Conf_1.000	498	20	1.04E-35	NP_501024.1	69	152.91	168	116	hypothetical protein C01B10.8
Locus_16886_Transcript_1/1_Conf_1.000	622	4	1.90E-14	XP_001893304.1	66	83.1889	99	66	hypothetical protein
Locus_16887_Transcript_1/1_Conf_1.000	417	3	2.56E-13	XP_002639547.1	57	78.5666	142	82	Hypothetical protein CBG04176
Locus_16888_Transcript_1/1_Conf_1.000	980	20	7.08E-39	XP_001900253.1	63	165.622	218	138	hypothetical protein Bm1_43955
Locus_16889_Transcript_1/1_Conf_1.000	233	0							
Locus_1689_Transcript_1/1_Conf_1.000	1518	20	0	XP_002642074.1	85	679.863	455	389	Hypothetical protein CBG18013
Locus_16890_Transcript_1/1_Conf_1.000	649	20	1.97E-44	XP_001891895.1	64	182.956	190	123	metallo-beta-lactamase superfamily protein
Locus_16891_Transcript_1/1_Conf_1.000	665	0							
Locus_16892_Transcript_1/1_Conf_1.000	159	0							
Locus_16893_Transcript_1/1_Conf_1.000	246	2	6.57E-06	NP_001076772.1	75	53.9138	45	34	GEX Interacting protein family member (gei-3)
Locus_16894_Transcript_1/1_Conf_1.000	178	0							

Locus_16895_Transcript_1/1_Conf_1.000	244	0							
Locus_16896_Transcript_1/1_Conf_1.000	1169	20	0	ACT34056.1	91	648.277	389	356	glutamate dehydrogenase
Locus_16897_Transcript_1/1_Conf_1.000	182	0							
Locus_16898_Transcript_1/1_Conf_1.000	300	20	4.27E-29	ACI49079.1	89	130.954	84	75	hypothetical protein Cbre_JD11.003
Locus_16899_Transcript_1/1_Conf_1.000	761	8	6.79E-11	NP_494977.1	66	72.0182	68	45	hypothetical protein C34F11.1
Locus_169_Transcript_1/2_Conf_1.000	749	20	1.78E-64	CAD20464.1	91	249.98	177	162	putative ES protein F7
Locus_169_Transcript_2/2_Conf_1.000	579	20	4.64E-46	CAD20464.1	92	187.963	139	128	putative ES protein F7

Locus_1690_Transcript_1/1_Conf_1.000	1454	20	0	XP_001895390.1	96	643.269	330	317	Serine/threonine protein phosphatase F56C9.1 in chromosomeIII, putative
Locus_16900_Transcript_1/1_Conf_1.000	267	2	1.25E-12	NP_001021809.1	63	76.2554	88	56	Branching AbnorMal family member (bam-2)
Locus_16901_Transcript_1/1_Conf_1.000	378	0							
Locus_16902_Transcript_1/1_Conf_1.000	980	20	5.89E-102	EFO18398.1	74	375.17	313	233	Prp18 domain-containing protein
Locus_16903_Transcript_1/1_Conf_1.000	515	20	6.62E-68	NP_001023148.1	84	259.996	173	146	ForKHead transcription factor family member (fkh-7)
Locus_16904_Transcript_1/1_Conf_1.000	474	0							
Locus_16905_Transcript_1/1_Conf_1.000	1429	20	1.74E-55		57	192.971	277	158	hypothetical protein F13H8.5 - Caenorhabditis elegans
Locus_16906_Transcript_1/1_Conf_1.000	140	0							
Locus_16907_Transcript_1/1_Conf_1.000	230	20	5.64E-29	XP_002637520.1	86	130.568	76	66	Hypothetical protein CBG19243
Locus_16908_Transcript_1/1_Conf_1.000	220	1	1.51E-05	XP_002639022.1	48	52.7582	74	36	Hypothetical protein CBG22271
Locus_16909_Transcript_1/1_Conf_1.000	171	0							
Locus_1691_Transcript_1/2_Conf_1.000	1782	20	0	NP_497043.2	73	662.144	596	436	Amino acid Transporter GlycoProtein subunit family member (atgp-2)
Locus_1691_Transcript_2/2_Conf_1.000	2014	20	0	NP_497043.2	72	739.184	661	482	Amino acid Transporter GlycoProtein subunit family member (atgp-2)

Locus_16910_Transcript_1/1_Conf_1.000	255	20	1.22E-23	XP_001901338.1	78	112.849	84	66	Bloom's syndrome protein homolog
Locus_16911_Transcript_1/1_Conf_1.000	130	20	5.41E-16	XP_002747286.1	100	87.4261	42	42	PREDICTED: endoplasmic reticulum-Golgi intermediate compartment protein 3 isoform 2
Locus_16912_Transcript_1/2_Conf_1.000	249	0							
Locus_16912_Transcript_2/2_Conf_1.000	249	0							
Locus_16913_Transcript_1/1_Conf_1.000	398	20	7.94E-07	NP_001021756.1	55	56.9954	79	44	UDP-GlucuronosylTransferase family member (ugt-31)
Locus_16914_Transcript_1/1_Conf_1.000	165	0							
Locus_16915_Transcript_1/1_Conf_1.000	479	0							
Locus_16916_Transcript_1/1_Conf_1.000	326	20	3.52E-23	XP_002630450.1	73	111.309	87	64	C. briggsae CBR-ASM-1 protein
Locus_16917_Transcript_1/1_Conf_1.000	222	4	2.47E-16	XP_002642663.1	76	88.5817	73	56	C. briggsae CBR-NCL-1 protein
Locus_16918_Transcript_1/1_Conf_1.000	502	0							
Locus_16919_Transcript_1/1_Conf_1.000	175	0							

Locus_1692_Transcript_1/1_Conf_1.000	975	20	5.35E-71	NP_001022317.1	72	272.322	329	238	abnormal cell MIGration family member (mig-5)
Locus_16920_Transcript_1/1_Conf_1.000	174	20	7.52E-10	XP_002640523.1	77	67.0106	45	35	Hypothetical protein CBG18685
Locus_16921_Transcript_1/1_Conf_1.000	143	20	1.11E-13	EAW56733.1	92	79.7221	40	37	similar to zinc finger protein 569, isoform CRA_a
Locus_16922_Transcript_1/1_Conf_1.000	133	0							
Locus_16923_Transcript_1/1_Conf_1.000	184	0							
Locus_16924_Transcript_1/1_Conf_1.000	426	0							
Locus_16925_Transcript_1/1_Conf_1.000	236	1	5.42E-24	XP_002343485.1	100	114.005	48	48	PREDICTED: hypothetical protein XP_002343485
Locus_16926_Transcript_1/1_Conf_1.000	296	0							
Locus_16927_Transcript_1/1_Conf_1.000	398	2	1.60E-15	NP_492266.2	56	85.8853	117	66	hypothetical protein C34B7.2
Locus_16928_Transcript_1/1_Conf_1.000	295	4	5.11E-14	XP_002637429.1	65	80.8777	95	62	Hypothetical protein CBG19140
Locus_16929_Transcript_1/1_Conf_1.000	405	2	7.84E-07	EFO23509.1	64	56.9954	71	46	STAT protein
Locus_1693_Transcript_1/1_Conf_1.000	2313	20	1.67E-41	XP_001657404.1	56	176.022	266	151	ubiquitin-conjugating enzyme E2-230k
Locus_16930_Transcript_1/1_Conf_1.000	1595	20	1.68E-39	XP_002647371.1	56	168.703	294	166	Hypothetical protein CBG06428
Locus_16931_Transcript_1/1_Conf_1.000	144	0							

Locus_16932_Transcript_1/1_Conf_1.000	435	20	3.33E-50	NP_507912.2	77	201.06	146	113	MoLTing defective family member (mlt-4)
Locus_16933_Transcript_1/1_Conf_1.000	287	3	1.40E-19	NP_491915.2	80	99.3673	78	63	hypothetical protein ZC581.9
Locus_16934_Transcript_1/1_Conf_1.000	265	0							
Locus_16935_Transcript_1/2_Conf_1.000	625	20	3.98E-36	NP_491579.1	62	155.221	205	128	hypothetical protein F55A12.1
Locus_16935_Transcript_2/2_Conf_1.000	625	20	3.98E-36	NP_491579.1	62	155.221	205	128	hypothetical protein F55A12.1
Locus_16936_Transcript_1/1_Conf_1.000	324	0							
Locus_16937_Transcript_1/1_Conf_1.000	419	20	1.94E-45	NP_495666.3	79	185.267	138	110	Temporarily Assigned Gene name family member (tag-341)
Locus_16938_Transcript_1/1_Conf_1.000	665	20	2.05E-52	NP_497324.3	68	209.534	209	143	hypothetical protein BE0003N10.1
Locus_16939_Transcript_1/1_Conf_1.000	268	0							
Locus_1694_Transcript_1/1_Conf_1.000	156	0							
Locus_16940_Transcript_1/1_Conf_1.000	468	20	9.24E-32	NP_491891.1	64	139.813	155	100	XPG (Xeroderma Pigmentosum group G) DNA repair gene homolog family member (xpg-1)
Locus_16941_Transcript_1/1_Conf_1.000	416	17	3.84E-06	XP_002629756.1	65	54.6842	55	36	Hypothetical protein CBG00991
Locus_16942_Transcript_1/1_Conf_1.000	426	20	2.42E-48	CAX65056.1	83	194.897	140	117	C. elegans protein F25C8.3d, partially confirmed by transcript evidence
Locus_16943_Transcript_1/1_Conf_1.000	164	0							
Locus_16944_Transcript_1/1_Conf_1.000	267	19	5.10E-06	CAR63735.1	66	54.299	36	24	hypothetical protein
Locus_16945_Transcript_1/1_Conf_1.000	951	0							
Locus_16946_Transcript_1/1_Conf_1.000	183	0							
Locus_16947_Transcript_1/1_Conf_1.000	338	20	1.89E-45	NP_501399.1	91	185.267	111	102	Vacuolar H ATPase family member (vha-5)
Locus_16948_Transcript_1/1_Conf_1.000	185	0							
Locus_16949_Transcript_1/1_Conf_1.000	239	0							
Locus_1695_Transcript_1/1_Conf_1.000	403	0							

Locus_16950_Transcript_1/1_Conf_1.000	536	20	1.51E-76	AAF66614.1	87	288.886	177	155	AF142441_1H+/oligopeptide symporter OPT-3
Locus_16951_Transcript_1/1_Conf_1.000	725	13	2.75E-43	NP_001040915.1	62	179.489	236	147	hypothetical protein C27D8.3
Locus_16952_Transcript_1/1_Conf_1.000	417	0							
Locus_16953_Transcript_1/1_Conf_1.000	157	0							
Locus_16954_Transcript_1/1_Conf_1.000	273	0							
Locus_16955_Transcript_1/1_Conf_1.000	228	0							
Locus_16956_Transcript_1/1_Conf_1.000	359	0							
Locus_16957_Transcript_1/1_Conf_1.000	230	20	4.80E-12	XP_002642125.1	68	74.3294	76	52	Hypothetical protein CBG18072
Locus_16958_Transcript_1/1_Conf_1.000	434	14	8.33E-09	NP_510664.1	78	63.5438	144	113	LEThal family member (let-2)
Locus_16959_Transcript_1/1_Conf_1.000	317	0							
Locus_1696_Transcript_1/1_Conf_1.000	661	20	3.44E-68	NP_509342.1	90	261.922	154	140	SEx Muscle abnormal family member (sem-5)
Locus_16960_Transcript_1/3_Conf_0.667	641	0							
Locus_16960_Transcript_2/3_Conf_0.667	681	0							
Locus_16960_Transcript_3/3_Conf_0.667	681	0							
Locus_16961_Transcript_1/1_Conf_1.000	353	0							
Locus_16962_Transcript_1/1_Conf_1.000	203	0							
Locus_16963_Transcript_1/1_Conf_1.000	135	0							
Locus_16964_Transcript_1/1_Conf_1.000	306	0							
Locus_16965_Transcript_1/1_Conf_1.000	128	20	2.21E-09	ABU48855.1	78	65.4698	42	33	heparan sulfate 3-O sulfotransferase isoform a
Locus_16966_Transcript_1/1_Conf_1.000	144	0							
Locus_16967_Transcript_1/1_Conf_1.000	169	0							
Locus_16968_Transcript_1/1_Conf_1.000	618	20	1.24E-13	AAG24090.4	46	80.4925	187	87	Hypothetical protein F31F4.11
Locus_16969_Transcript_1/1_Conf_1.000	627	0							
Locus_1697_Transcript_1/2_Conf_1.000	538	1	9.22E-05	XP_001899906.1	56	50.447	66	37	MOZ/SAS family protein

Locus_1697_Transcript_2/2_Conf_1.000	998	20	3.77E-19	XP_002639480.1	50	100.138	150	76	Hypothetical protein CBG04078
Locus_16970_Transcript_1/1_Conf_1.000	234	0							
Locus_16971_Transcript_1/1_Conf_1.000	271	20	4.25E-45	XP_002810085.1	100	184.111	89	89	PREDICTED: lamin-A/C-like
Locus_16972_Transcript_1/1_Conf_1.000	631	7	3.73E-05	EER44235.1	49	52.373	134	66	calmodulin-binding coil-coil protein
Locus_16973_Transcript_1/1_Conf_1.000	221	20	1.15E-29	CAZ39159.1	94	132.88	72	68	C. elegans protein C01C7.1b, partially confirmed by transcript evidence
Locus_16974_Transcript_1/1_Conf_1.000	638	3	1.63E-64	XP_002634053.1	77	249.595	212	165	Hypothetical protein CBG01593

Locus_16975_Transcript_1/1_Conf_1.000	755	20	3.70E-86	ADK47995.1	100	322.013	158	158	c-myc promoter-binding protein 1
Locus_16976_Transcript_1/1_Conf_1.000	156	0							
Locus_16977_Transcript_1/1_Conf_1.000	620	20	8.43E-71	XP_002632331.1	83	270.396	200	166	C. briggsae CBR-ROM-4 protein
Locus_16978_Transcript_1/1_Conf_1.000	165	0							
Locus_16979_Transcript_1/1_Conf_1.000	374	2	7.65E-18	NP_498388.1	66	93.5893	83	55	hypothetical protein C56G2.3
Locus_1698_Transcript_1/1_Conf_1.000	341	0							
Locus_16980_Transcript_1/1_Conf_1.000	733	20	1.27E-43	XP_002641461.1	77	180.644	157	121	C. briggsae CBR-WAH-1 protein
Locus_16981_Transcript_1/1_Conf_1.000	203	1	3.47E-11	AAC06295.1	84	60.8474	45	38	hypothetical protein jmo23
Locus_16982_Transcript_1/1_Conf_1.000	248	0							
Locus_16983_Transcript_1/1_Conf_1.000	253	0							
Locus_16984_Transcript_1/1_Conf_1.000	601	7	9.59E-29	ACY39994.1	58	130.568	202	118	C. elegans FLN-2 protein, isoform d
Locus_16985_Transcript_1/1_Conf_1.000	859	20	1.10E-90	NP_496170.1	89	337.421	191	170	LEThal family member (let-268)
Locus_16986_Transcript_1/1_Conf_1.000	696	20	5.15E-44	NP_001040927.1	65	181.8	217	143	hypothetical protein C47A4.2
Locus_16987_Transcript_1/1_Conf_1.000	180	0							
Locus_16988_Transcript_1/1_Conf_1.000	547	0							
Locus_16989_Transcript_1/1_Conf_1.000	373	20	7.32E-53	NP_501979.3	89	209.92	124	111	hypothetical protein R102.4

Locus_1699_Transcript_1/1_Conf_1.000	1480	20	0	XP_002636638.1	97	818.92	435	423	C. briggsae CBR-RPT-1 protein
Locus_16990_Transcript_1/1_Conf_1.000	460	0							
Locus_16991_Transcript_1/1_Conf_1.000	605	2	4.84E-20	NP_496323.1	51	101.679	158	82	SET (trithorax/polycomb) domain containing family member (set-14)
Locus_16992_Transcript_1/1_Conf_1.000	240	0							
Locus_16993_Transcript_1/1_Conf_1.000	447	20	7.09E-24	NP_001022170.1	72	113.62	95	69	UNCoordinated family member (unc-53)
Locus_16994_Transcript_1/1_Conf_1.000	164	6	6.67E-06	XP_002643836.1	67	53.9138	55	37	Hypothetical protein CBG02060
Locus_16995_Transcript_1/1_Conf_1.000	355	20	9.28E-08	XP_002158963.1	50	60.077	91	46	PREDICTED: similar to Rps2e protein

Locus_16996_Transcript_1/1_Conf_1.000	246	20	3.45E-39	AAP88768.1	100	164.466	78	78	cytochrome c oxidase subunit VIIb
Locus_16997_Transcript_1/1_Conf_1.000	279	0							
Locus_16998_Transcript_1/1_Conf_1.000	271	0							
Locus_16999_Transcript_1/1_Conf_1.000	296	0							
Locus_17_Transcript_17/30_Conf_1.000	270	20	3.60E-44	ACR36970.1	96	181.03	89	86	unknown
Locus_17_Transcript_18/30_Conf_1.000	263	1	9.35E-08	XP_002467304.1	77	60.077	44	34	hypothetical protein SORBIDRAFT_01g024221
Locus_17_Transcript_19/30_Conf_1.000	263	1	3.21E-08	XP_002467304.1	75	61.6178	48	36	hypothetical protein SORBIDRAFT_01g024221
Locus_17_Transcript_20/30_Conf_1.000	270	20	3.60E-44	ACR36970.1	96	181.03	89	86	unknown
Locus_17_Transcript_21/30_Conf_0.019	363	11	2.48E-29	BAB33421.1	92	131.724	68	63	putative senescence-associated protein
Locus_17_Transcript_22/30_Conf_0.287	1283	20	1.46E-80	BAB33421.1	71	304.679	260	185	putative senescence-associated protein
Locus_17_Transcript_23/30_Conf_0.065	426	13	1.61E-12	XP_002638702.1	63	60.8474	61	39	Hypothetical protein CBG11901
Locus_17_Transcript_24/30_Conf_0.315	1384	20	1.07E-63	XP_001028745.1	67	248.825	246	167	hypothetical protein TTHERM_02141640
Locus_17_Transcript_25/30_Conf_0.361	1485	20	4.31E-58	XP_001028745.1	67	230.335	224	152	hypothetical protein TTHERM_02141640
Locus_17_Transcript_26/30_Conf_0.361	1527	20	7.64E-58	XP_001028745.1	67	229.565	222	150	hypothetical protein TTHERM_02141640
Locus_17_Transcript_27/30_Conf_0.296	1059	20	2.65E-58	XP_001028745.1	67	230.335	224	152	hypothetical protein TTHERM_02141640
Locus_17_Transcript_28/30_Conf_0.056	300	1	1.54E-12	XP_002638702.1	63	60.8474	61	39	Hypothetical protein CBG11901
Locus_17_Transcript_29/30_Conf_0.343	1387	20	3.94E-58	XP_001028745.1	67	230.335	224	152	hypothetical protein TTHERM_02141640
Locus_17_Transcript_30/30_Conf_0.565	2123	20	6.76E-58	XP_001028745.1	67	230.335	224	152	hypothetical protein TTHERM_02141640
Locus_170_Transcript_1/1_Conf_1.000	530	9	1.03E-69	AAR25200.1	90	266.159	162	147	surface-associated antigen 1
Locus_1700_Transcript_1/3_Conf_0.600	1828	20	2.03E-124	NP_741191.1	78	451.055	346	270	hypothetical protein C56G2.1
Locus_1700_Transcript_2/3_Conf_0.600	2035	20	1.03E-132	NP_741190.1	70	478.789	430	305	hypothetical protein C56G2.1
Locus_1700_Transcript_3/3_Conf_0.600	1832	20	2.03E-124	NP_741191.1	78	451.055	346	270	hypothetical protein C56G2.1
Locus_17000_Transcript_1/1_Conf_1.000	404	20	2.13E-20	ACI48997.1	62	102.064	136	85	hypothetical protein Cbre_JD01.004
Locus_17001_Transcript_1/1_Conf_1.000	175	0							
Locus_17002_Transcript_1/1_Conf_1.000	589	20	2.08E-25	NP_499759.1	75	119.398	109	82	DNaJ domain (prokaryotic heat shock protein) family member (dnj-17)

Locus_17003_Transcript_1/1_Conf_1.000	681	20	3.67E-116	NP_492127.1	93	421.394	226	211	Low-density lipoprotein Receptor Related family member (lrp-1)
Locus_17004_Transcript_1/1_Conf_1.000	214	0							
Locus_17005_Transcript_1/1_Conf_1.000	622	20	1.15E-19	NP_001040945.1	54	100.523	200	108	human HnRNP A1 homolog family member (hrp-1)
Locus_17006_Transcript_1/1_Conf_1.000	208	0							
Locus_17007_Transcript_1/1_Conf_1.000	345	0							
Locus_17008_Transcript_1/1_Conf_1.000	241	0							
Locus_17009_Transcript_1/1_Conf_1.000	415	20	4.54E-07	XP_002189413.1	59	57.7658	62	37	PREDICTED: putative U5 snRNP-specific 40 kDa protein, partial
Locus_1701_Transcript_1/1_Conf_1.000	502	0							
Locus_17010_Transcript_1/1_Conf_1.000	674	0							
Locus_17011_Transcript_1/1_Conf_1.000	245	4	2.68E-07	NP_495342.2	61	58.5362	81	50	related to yeast Vacuolar Protein Sorting factor family member (vps-33.2)
Locus_17012_Transcript_1/2_Conf_1.000	396	0							
Locus_17012_Transcript_2/2_Conf_1.000	481	0							
Locus_17013_Transcript_1/2_Conf_1.000	733	20	3.06E-114	XP_862070.1	99	415.231	202	201	PREDICTED: similar to Tubulin alpha-6 chain (Alpha-tubulin 6) (Alpha-tubulin isotype M-alpha-6) isoform 6
Locus_17013_Transcript_2/2_Conf_1.000	835	20	3.92E-114	XP_862070.1	99	415.231	202	201	PREDICTED: similar to Tubulin alpha-6 chain (Alpha-tubulin 6) (Alpha-tubulin isotype M-alpha-6) isoform 6
Locus_17014_Transcript_1/1_Conf_1.000	316	0							

Locus_17015_Transcript_1/1_Conf_1.000	287	20	3.57E-23	EFO16535.1	76	111.309	94	72	hypothetical protein LOAG_11971
Locus_17016_Transcript_1/1_Conf_1.000	451	0							
Locus_17017_Transcript_1/1_Conf_1.000	415	0							
Locus_17018_Transcript_1/1_Conf_1.000	320	0							
Locus_17019_Transcript_1/1_Conf_1.000	276	0							
Locus_1702_Transcript_1/1_Conf_1.000	1029	0							
Locus_17020_Transcript_1/1_Conf_1.000	171	0							
Locus_17021_Transcript_1/1_Conf_1.000	239	2	2.79E-04	NP_492616.1	52	48.521	82	43	Nematode AStacin protease family member (nas-5)
Locus_17022_Transcript_1/1_Conf_1.000	279	20	9.05E-48	XP_002763564.1	100	192.971	92	92	PREDICTED: transmembrane emp24 domain-containing protein 9-like
Locus_17023_Transcript_1/1_Conf_1.000	470	20	1.03E-14	EFO28126.1	60	83.1889	135	82	hypothetical protein LOAG_00360
Locus_17024_Transcript_1/1_Conf_1.000	311	20	5.17E-19	NP_740774.1	81	97.4413	77	63	hypothetical protein Y48G1A.4
Locus_17025_Transcript_1/1_Conf_1.000	362	20	1.24E-20	ACI49149.1	63	102.834	121	77	hypothetical protein Cbre_JD20.004
Locus_17026_Transcript_1/1_Conf_1.000	213	0							
Locus_17027_Transcript_1/1_Conf_1.000	455	20	3.27E-21	XP_002199753.1	57	104.76	119	69	PREDICTED: similar to FLJ14007
Locus_17028_Transcript_1/1_Conf_1.000	177	11	7.73E-15	XP_001901295.1	80	83.5741	56	45	Nematode astacin protease protein 30
Locus_17029_Transcript_1/2_Conf_1.000	752	0							

Locus_17029_Transcript_2/2_Conf_1.000	752	0							
Locus_1703_Transcript_1/1_Conf_1.000	468	20	1.62E-60	XP_002645153.1	91	235.343	156	143	C. briggsae CBR-SFXN-2 protein
Locus_17030_Transcript_1/1_Conf_1.000	387	20	2.66E-23	NP_491806.2	78	111.694	89	70	LiPid Depleted family member (lpd-2)
Locus_17031_Transcript_1/1_Conf_1.000	146	1	8.31E-04	XP_001893741.1	63	46.9802	49	31	LIM domain containing protein
Locus_17032_Transcript_1/1_Conf_1.000	552	0							
Locus_17033_Transcript_1/1_Conf_1.000	148	20	6.33E-12	XP_002823973.1	100	73.9442	34	34	PREDICTED: ADP-ribosylation factor-like protein 6-interacting protein 4-like isoform 5
Locus_17034_Transcript_1/1_Conf_1.000	150	0							
Locus_17035_Transcript_1/1_Conf_1.000	584	20	1.53E-20	NP_491574.1	64	103.219	135	87	hypothetical protein F55A12.8
Locus_17036_Transcript_1/1_Conf_1.000	158	20	3.92E-22	BAF83756.1	100	107.842	52	52	unnamed protein product
Locus_17037_Transcript_1/1_Conf_1.000	158	0							
Locus_17038_Transcript_1/1_Conf_1.000	267	20	1.06E-11	XP_002641218.1	64	73.1738	77	50	Hypothetical protein CBG09082
Locus_17039_Transcript_1/1_Conf_1.000	330	20	2.66E-47	NP_001122450.1	90	191.43	110	100	hypothetical protein F10G8.8
Locus_1704_Transcript_1/1_Conf_1.000	555	2	3.53E-29	NP_001022723.2	59	131.724	156	93	TENeurin family member (ten-1)
Locus_17040_Transcript_1/1_Conf_1.000	406	20	4.26E-37	EFO28414.1	71	157.532	133	95	hypothetical protein LOAG_00060
Locus_17041_Transcript_1/1_Conf_1.000	256	3	1.11E-24	AAC39119.1	73	116.316	78	57	low-affinity peptide transporter
Locus_17042_Transcript_1/1_Conf_1.000	166	0							
Locus_17043_Transcript_1/1_Conf_1.000	443	0							

Locus_17044_Transcript_1/1_Conf_1.000	308	20	3.70E-41	XP_002630183.1	91	171.014	102	93	C. briggsae CBR-FLI-1 protein
Locus_17045_Transcript_1/2_Conf_1.000	594	0							
Locus_17045_Transcript_2/2_Conf_1.000	594	0							
Locus_17046_Transcript_1/1_Conf_1.000	486	20	1.13E-45	CAA30855.1	91	186.037	137	125	myosin heavy chain 2
Locus_17047_Transcript_1/1_Conf_1.000	235	0							
Locus_17048_Transcript_1/1_Conf_1.000	176	0							
Locus_17049_Transcript_1/2_Conf_1.000	464	2	3.69E-04	NP_495257.3	46	48.1358	111	52	Biotin Protein Ligase family member (bpl-1)
Locus_17049_Transcript_2/2_Conf_1.000	464	2	3.69E-04	NP_495257.3	46	48.1358	111	52	Biotin Protein Ligase family member (bpl-1)
Locus_1705_Transcript_1/1_Conf_1.000	496	0							
Locus_17050_Transcript_1/1_Conf_1.000	354	20	7.32E-21	XP_002634722.1	67	103.605	101	68	Hypothetical protein CBG24065
Locus_17051_Transcript_1/1_Conf_1.000	132	20	1.98E-18	NP_001087890.2	100	95.5153	43	43	LIM domain only 4, gene 1
Locus_17052_Transcript_1/1_Conf_1.000	401	20	9.21E-56	XP_002645894.1	90	219.55	133	121	Hypothetical protein CBG07647
Locus_17053_Transcript_1/1_Conf_1.000	351	20	4.93E-17	NP_496909.1	67	90.8929	101	68	ING (mammalian INhibitor of Growth) homolog family member (ing-3)
Locus_17054_Transcript_1/1_Conf_1.000	1012	20	6.94E-77	XP_002637110.1	76	291.967	257	197	Hypothetical protein CBG09610
Locus_17055_Transcript_1/1_Conf_1.000	175	0							
Locus_17056_Transcript_1/1_Conf_1.000	224	0							
Locus_17057_Transcript_1/1_Conf_1.000	404	0							
Locus_17058_Transcript_1/1_Conf_1.000	502	20	4.51E-10	XP_975003.1	49	67.781	163	80	PREDICTED: similar to CG31751 CG31751-PA
Locus_17059_Transcript_1/1_Conf_1.000	233	0							

Locus_1706_Transcript_1/2_Conf_1.000	1467	20	1.87E-122	XP_002633783.1	72	444.121	425	309	Hypothetical protein CBG03474
Locus_1706_Transcript_2/2_Conf_1.000	1453	20	2.96E-115	XP_002633783.1	69	330.102	347	242	Hypothetical protein CBG03474
Locus_17060_Transcript_1/1_Conf_1.000	430	3	1.93E-05	XP_002644943.1	44	52.373	142	63	Hypothetical protein CBG10898
Locus_17061_Transcript_1/1_Conf_1.000	849	4	3.20E-10	NP_493646.1	56	70.0922	144	82	hypothetical protein F23F1.9
Locus_17062_Transcript_1/1_Conf_1.000	243	20	1.01E-22	NP_501127.2	96	109.768	57	55	RNA Polymerase, Class III (C) family member (rpc-1)
Locus_17063_Transcript_1/1_Conf_1.000	524	13	3.52E-67	XP_002640548.1	93	257.684	174	163	Hypothetical protein CBG15812
Locus_17064_Transcript_1/1_Conf_1.000	230	20	6.05E-15	XP_002647188.1	79	83.9593	59	47	Hypothetical protein CBG22355
Locus_17065_Transcript_1/1_Conf_1.000	665	4	5.10E-19	NP_001040707.1	60	98.5969	211	128	CDT (S. pombe CDC10 Dependent Transcript) homolog family member (cdt-1)
Locus_17066_Transcript_1/1_Conf_1.000	367	0							
Locus_17067_Transcript_1/1_Conf_1.000	432	0							
Locus_17068_Transcript_1/1_Conf_1.000	183	20	6.34E-12	XP_002634144.1	76	73.9442	60	46	Hypothetical protein CBG01705
Locus_17069_Transcript_1/1_Conf_1.000	193	0							

Locus_1707_Transcript_1/1_Conf_1.000	415	20	3.44E-39	NP_499099.1	74	164.466	136	101	hypothetical protein ZK1098.7
Locus_17070_Transcript_1/1_Conf_1.000	149	0							
Locus_17071_Transcript_1/1_Conf_1.000	339	2	1.61E-04	EFO20756.1	49	49.2914	104	51	nucleolar RNA-associated protein alpha
Locus_17072_Transcript_1/1_Conf_1.000	179	20	2.11E-12	XP_002637265.1	76	75.485	59	45	C. briggsae CBR-CPT-6 protein
Locus_17073_Transcript_1/1_Conf_1.000	255	0							
Locus_17074_Transcript_1/1_Conf_1.000	252	4	6.33E-12	NP_509145.2	68	73.9442	76	52	AuTophaGy (yeast Atg homolog) family member (atg-2)
Locus_17075_Transcript_1/1_Conf_1.000	370	0							
Locus_17076_Transcript_1/1_Conf_1.000	302	20	6.83E-19	XP_002640837.1	78	97.0561	64	50	Hypothetical protein CBG15725
Locus_17077_Transcript_1/1_Conf_1.000	268	20	8.06E-36	XP_001891652.1	97	153.295	79	77	myosin heavy chain, nonmuscle type 1
Locus_17078_Transcript_1/1_Conf_1.000	321	0							
Locus_17079_Transcript_1/1_Conf_1.000	157	4	1.49E-13	EFO21662.1	82	79.337	51	42	hypothetical protein LOAG_06825
Locus_1708_Transcript_1/1_Conf_1.000	460	0							
Locus_17080_Transcript_1/1_Conf_1.000	306	20	2.48E-37	NP_497182.2	88	158.303	101	89	hypothetical protein Y50D7A.2
Locus_17081_Transcript_1/1_Conf_1.000	188	0							
Locus_17082_Transcript_1/1_Conf_1.000	317	4	1.11E-24	XP_002644860.1	71	116.316	113	81	Hypothetical protein CBG05033
Locus_17083_Transcript_1/1_Conf_1.000	168	0							
Locus_17084_Transcript_1/1_Conf_1.000	736	20	3.75E-27	EFO22480.1	54	125.946	235	127	BSD domain-containing protein
Locus_17085_Transcript_1/1_Conf_1.000	256	0							
Locus_17086_Transcript_1/1_Conf_1.000	147	0							
Locus_17087_Transcript_1/1_Conf_1.000	149	0							
Locus_17088_Transcript_1/1_Conf_1.000	294	0							
Locus_17089_Transcript_1/1_Conf_1.000	286	0							
Locus_1709_Transcript_1/1_Conf_1.000	824	0							
Locus_17090_Transcript_1/1_Conf_1.000	485	0							
Locus_17091_Transcript_1/1_Conf_1.000	207	0							
Locus_17092_Transcript_1/1_Conf_1.000	214	0							
Locus_17093_Transcript_1/1_Conf_1.000	244	8	8.85E-27	XP_002636220.1	79	123.25	73	58	Hypothetical protein CBG12141
Locus_17094_Transcript_1/1_Conf_1.000	243	0							
Locus_17095_Transcript_1/1_Conf_1.000	195	0							
Locus_17096_Transcript_1/1_Conf_1.000	327	0							
Locus_17097_Transcript_1/1_Conf_1.000	155	0							

Locus_17098_Transcript_1/4_Conf_0.333	587	20	2.99E-64	XP_001892307.1	77	248.44	191	148	ATP-dependent DNA helicase, RecQ family protein
Locus_17098_Transcript_2/4_Conf_0.444	1113	20	7.48E-99	XP_002642560.1	72	365.155	315	227	C. briggsae CBR-RCQ-5 protein
Locus_17098_Transcript_3/4_Conf_0.444	1113	20	7.48E-99	XP_002642560.1	72	365.155	315	227	C. briggsae CBR-RCQ-5 protein
Locus_17098_Transcript_4/4_Conf_0.333	587	20	2.99E-64	XP_001892307.1	77	248.44	191	148	ATP-dependent DNA helicase, RecQ family protein
Locus_17099_Transcript_1/1_Conf_1.000	941	20	6.97E-113	XP_002641585.1	81	411.379	302	245	Hypothetical protein CBG09887
Locus_171_Transcript_1/1_Conf_1.000	677	20	3.67E-36	XP_001896629.1	57	155.606	229	131	U-box domain containing protein
Locus_1710_Transcript_1/1_Conf_1.000	271	20	8.37E-09	XP_001899631.1	57	63.5438	90	52	WD-repeat protein 26
Locus_17100_Transcript_1/1_Conf_1.000	157	0							
Locus_17101_Transcript_1/1_Conf_1.000	515	20	2.54E-35	NP_001021671.2	68	151.754	168	115	hypothetical protein W09G3.7
Locus_17102_Transcript_1/1_Conf_1.000	517	20	1.39E-12	XP_002049622.1	47	76.2554	166	79	GJ21697
Locus_17103_Transcript_1/1_Conf_1.000	280	0							
Locus_17104_Transcript_1/1_Conf_1.000	777	20	3.48E-66	CBL87054.2	69	255.758	262	181	C. elegans protein F54E4.1, partially confirmed by transcript evidence
Locus_17105_Transcript_1/1_Conf_1.000	232	0							

Locus_17106_Transcript_1/1_Conf_1.000	714	20	1.83E-76	XP_001893036.1	83	289.656	210	176	SMC family, C-terminal domain containing protein
Locus_17107_Transcript_1/1_Conf_1.000	287	20	1.66E-20	ADI61822.1	67	102.449	90	61	endonuclease-reverse transcriptase
Locus_17108_Transcript_1/1_Conf_1.000	280	4	1.84E-08	XP_001895740.1	68	62.3882	73	50	hypothetical protein Bm1_21430
Locus_17109_Transcript_1/1_Conf_1.000	465	20	2.62E-26	XP_001149667.1	72	121.709	115	83	PREDICTED: transcription factor AP-2 beta (activating enhancer binding protein 2 beta) isoform 1
Locus_1711_Transcript_1/1_Conf_1.000	878	20	5.15E-144	EFO23330.1	94	514.612	287	270	GTP-binding regulatory protein alpha chain
Locus_17110_Transcript_1/1_Conf_1.000	536	1	5.86E-04	NP_001022760.1	62	47.7506	54	34	hypothetical protein T16G12.9
Locus_17111_Transcript_1/1_Conf_1.000	680	20	3.02E-62	XP_002630829.1	76	242.276	227	174	C. briggsae CBR-VPS-35 protein
Locus_17112_Transcript_1/2_Conf_1.000	347	0							
Locus_17112_Transcript_2/2_Conf_1.000	345	0							
Locus_17113_Transcript_1/1_Conf_1.000	157	20	2.09E-07	XP_002157644.1	78	58.9214	51	40	PREDICTED: similar to predicted protein, partial
Locus_17114_Transcript_1/1_Conf_1.000	239	3	1.17E-10	NP_491138.2	63	69.707	79	50	SULfate Permease family member (sulp-6)
Locus_17115_Transcript_1/1_Conf_1.000	1047	20	9.30E-40	NP_001022032.1	54	168.703	333	181	TRRAP-like (transcription/transformation domain-associated protein) family member (trr-1)
Locus_17116_Transcript_1/1_Conf_1.000	143	3	1.61E-04	XP_002631235.1	66	49.2914	54	36	C. briggsae CBR-PTB-1 protein
Locus_17117_Transcript_1/1_Conf_1.000	157	0							

Locus_17118_Transcript_1/1_Conf_1.000	206	20	7.78E-31	XP_002762172.1	100	136.732	68	68	PREDICTED: 26S protease regulatory subunit 6B
Locus_17119_Transcript_1/1_Conf_1.000	1297	13	1.05E-25	XP_001895099.1	47	122.479	350	165	hypothetical protein Bm1_18210
Locus_1712_Transcript_1/1_Conf_1.000	1375	20	1.69E-61	EFO21998.1	50	241.506	385	194	RWD domain-containing protein
Locus_17120_Transcript_1/1_Conf_1.000	350	9	9.26E-40	XP_002635876.1	80	166.392	116	93	C. briggsae CBR-PTR-16 protein
Locus_17121_Transcript_1/1_Conf_1.000	279	4	1.90E-29	XP_002642450.1	79	132.109	92	73	Hypothetical protein CBG06858
Locus_17122_Transcript_1/1_Conf_1.000	391	20	5.92E-15	ADI61811.1	58	83.9593	111	65	endonuclease-reverse transcriptase
Locus_17123_Transcript_1/1_Conf_1.000	577	20	1.06E-18	NP_495324.2	80	97.0561	63	51	human WRN (Werner's syndrome) related family member (wrn-1)
Locus_17124_Transcript_1/1_Conf_1.000	530	20	4.20E-63	NP_496444.1	78	244.202	175	138	Alanyl tRNA Synthetase family member (ars-1)
Locus_17125_Transcript_1/1_Conf_1.000	223	0							
Locus_17126_Transcript_1/1_Conf_1.000	961	0							
Locus_17127_Transcript_1/1_Conf_1.000	461	0							
Locus_17128_Transcript_1/1_Conf_1.000	476	0							
Locus_17129_Transcript_1/1_Conf_1.000	360	0							

Locus_1713_Transcript_1/1_Conf_1.000	2758	20	0	XP_002646632.1	84	1382.47	926	783	C. briggsae CBR-UNC-52 protein
Locus_17130_Transcript_1/1_Conf_1.000	200	20	2.89E-17	XP_002636925.1	83	91.6633	68	57	C. briggsae CBR-RHR-1 protein
Locus_17131_Transcript_1/1_Conf_1.000	198	0							
Locus_17132_Transcript_1/1_Conf_1.000	503	4	3.44E-18	XP_002630955.1	59	94.7449	166	98	C. briggsae CBR-WRN-1 protein
Locus_17133_Transcript_1/3_Conf_0.333	483	2	4.66E-07	AAO63576.1	40	57.7658	154	62	secreted protein 4 precursor
Locus_17133_Transcript_2/3_Conf_0.667	687	4	1.29E-07	ABB53347.1	41	60.8474	214	89	secreted protein 5 precursor
Locus_17133_Transcript_3/3_Conf_0.500	175	0							
Locus_17134_Transcript_1/1_Conf_1.000	162	0							
Locus_17135_Transcript_1/1_Conf_1.000	196	5	3.54E-15	XP_002638527.1	76	84.7297	65	50	C. briggsae CBR-UNC-80 protein
Locus_17136_Transcript_1/1_Conf_1.000	255	2	1.04E-06	XP_002643159.1	63	56.6102	65	41	C. briggsae CBR-VPS-33.1 protein
Locus_17137_Transcript_1/2_Conf_1.000	260	8	2.90E-25	EFO25373.1	76	118.242	89	68	laterally symmetric protein 2
Locus_17137_Transcript_2/2_Conf_1.000	270	8	3.06E-27	EFO25373.1	77	124.79	92	71	laterally symmetric protein 2
Locus_17138_Transcript_1/1_Conf_1.000	382	8	3.87E-22	EFO20275.1	64	107.842	126	81	hypothetical protein LOAG_08213

Locus_17139_Transcript_1/1_Conf_1.000	1633	20	1.05E-60	XP_001893645.1	67	239.195	238	161	MGC79624 protein
Locus_1714_Transcript_1/1_Conf_1.000	1104	20	1.32E-63	NP_496151.1	74	248.054	213	158	hypothetical protein R53.2
Locus_17140_Transcript_1/1_Conf_1.000	640	0							
Locus_17141_Transcript_1/1_Conf_1.000	278	0							
Locus_17142_Transcript_1/1_Conf_1.000	400	20	1.34E-22	XP_002641332.1	60	109.383	138	84	Hypothetical protein CBG13180
Locus_17143_Transcript_1/1_Conf_1.000	821								
Locus_17144_Transcript_1/1_Conf_1.000	297	2	8.40E-09	XP_002636395.1	53	63.5438	97	52	Hypothetical protein CBG08700
Locus_17145_Transcript_1/1_Conf_1.000	413	20	1.57E-07	EFN69864.1	56	59.3066	89	50	Serine/threonine-protein kinase grp
Locus_17146_Transcript_1/1_Conf_1.000	402	1	2.80E-04	NP_508445.1	76	48.521	34	26	hypothetical protein T07D1.3
Locus_17147_Transcript_1/1_Conf_1.000	162	0							
Locus_17148_Transcript_1/1_Conf_1.000	143	0							
Locus_17149_Transcript_1/1_Conf_1.000	219	4	4.10E-11	NP_495673.2	94	71.2478	36	34	hypothetical protein DH11.4

Locus_1715_Transcript_1/1_Conf_1.000	936	20	2.38E-113	NP_505736.2	77	412.92	311	242	yeast MRE recombination/repair homolog family member (mre-11)
Locus_17150_Transcript_1/1_Conf_1.000	379	0							
Locus_17151_Transcript_1/1_Conf_1.000	390	3	1.18E-39	NP_001021158.1	86	166.007	104	90	UDP-GlucuronosylTransferase family member (ugt-60)
Locus_17152_Transcript_1/1_Conf_1.000	188	0							
Locus_17153_Transcript_1/1_Conf_1.000	249	20	1.19E-15	NP_495614.3	84	86.2705	58	49	Na/H eXchanger family member (nhx-2)
Locus_17154_Transcript_1/1_Conf_1.000	822	20	2.12E-64	EFO21353.1	70	249.98	269	190	oxidoreductase
Locus_17155_Transcript_1/1_Conf_1.000	250	0							
Locus_17156_Transcript_1/1_Conf_1.000	406	20	1.28E-33	NP_503029.2	80	145.976	117	94	hypothetical protein Y116A8C.27
Locus_17157_Transcript_1/1_Conf_1.000	133	0							

Locus_17158_Transcript_1/1_Conf_1.000	331	20	9.83E-26	XP_002634557.1	87	119.783	95	83	C. briggsae CBR-TAX-6 protein
Locus_17159_Transcript_1/1_Conf_1.000	260	0							
Locus_1716_Transcript_1/1_Conf_1.000	931	20	2.18E-42	XP_001897236.1	62	177.178	200	124	Zinc finger, C2H2 type family protein
Locus_17160_Transcript_1/1_Conf_1.000	351	2	2.43E-32	NP_506678.2	74	141.739	116	86	hypothetical protein W09D12.1
Locus_17161_Transcript_1/1_Conf_1.000	276	2	2.26E-06	XP_002639005.1	49	55.4546	91	45	Hypothetical protein CBG22251
Locus_17162_Transcript_1/1_Conf_1.000	349	0							
Locus_17163_Transcript_1/1_Conf_1.000	126	0							
Locus_17164_Transcript_1/2_Conf_1.000	596	20	2.79E-41	NP_499333.1	63	172.17	198	126	hypothetical protein Y45F3A.9
Locus_17164_Transcript_2/2_Conf_1.000	406	20	1.52E-26	NP_499333.1	65	122.479	135	89	hypothetical protein Y45F3A.9

Locus_17165_Transcript_1/1_Conf_1.000	211	20	1.15E-34	XP_002928912.1	100	149.443	70	70	PREDICTED: tubulin beta chain-like
Locus_17166_Transcript_1/1_Conf_1.000	158	0							
Locus_17167_Transcript_1/1_Conf_1.000	381	0							
Locus_17168_Transcript_1/1_Conf_1.000	272	4	4.59E-07	XP_001894916.1	55	57.7658	70	39	Zinc finger, C2H2 type family protein
Locus_17169_Transcript_1/1_Conf_1.000	148	0							
Locus_1717_Transcript_1/1_Conf_1.000	545	20	8.81E-67	NP_496402.1	82	256.529	175	144	hypothetical protein T06D8.5
Locus_17170_Transcript_1/1_Conf_1.000	128	20	1.43E-16	NP_001024858.2	95	89.3521	42	40	Sensory AXon guidance family member (sax-1)
Locus_17171_Transcript_1/2_Conf_1.000	313	20	4.66E-31	NP_500367.1	76	137.502	104	80	hypothetical protein M57.2

Locus_17171_Transcript_2/2_Conf_1.000	329	20	1.86E-32	NP_500367.1	76	142.124	110	84	hypothetical protein M57.2
Locus_17172_Transcript_1/1_Conf_1.000	448	20	9.83E-50	NP_499387.1	74	199.519	149	111	hypothetical protein R17.3
Locus_17173_Transcript_1/1_Conf_1.000	216	20	1.71E-22	NP_498418.2	88	108.997	69	61	hypothetical protein T26A5.5
Locus_17174_Transcript_1/1_Conf_1.000	525	20	4.04E-10	NP_001022699.1	52	68.1662	186	98	Ring Finger Protein, UBC associated family member (rfp-1)
Locus_17175_Transcript_1/1_Conf_1.000	204	0							
Locus_17176_Transcript_1/1_Conf_1.000	522	20	4.22E-12	XP_001273939.1	48	74.7146	166	81	sulfur metabolism regulator SkpA, putative
Locus_17177_Transcript_1/1_Conf_1.000	460	20	3.34E-26	XP_002632532.1	88	121.324	104	92	C. briggsae CBR-DPY-4 protein
Locus_17178_Transcript_1/1_Conf_1.000	516	20	1.16E-67	NP_498121.1	82	259.225	168	138	yeast HAP (HydroxylAminoPurine sensitivity) related family member (hap-1)
Locus_17179_Transcript_1/1_Conf_1.000	553	0							
Locus_1718_Transcript_1/2_Conf_1.000	867	20	5.97E-68	XP_001898186.1	75	261.922	188	142	AMME syndrome candidate gene 1 protein homolog
Locus_1718_Transcript_2/2_Conf_1.000	312	0							
Locus_17180_Transcript_1/1_Conf_1.000	541	0							
Locus_17181_Transcript_1/1_Conf_1.000	206	1	4.75E-04	XP_002631426.1	63	47.7506	49	31	Hypothetical protein CBG03282

Locus_17182_Transcript_1/1_Conf_1.000	168	20	2.13E-12	NP_001021239.1	74	75.485	54	40	Endo-b-N-acetylGlucosaminidase family member (eng-1)
Locus_17183_Transcript_1/1_Conf_1.000	360	0							
Locus_17184_Transcript_1/1_Conf_1.000	999	20	4.77E-30	XP_002636702.1	52	136.346	333	175	C. briggsae CBR-RAD-50 protein
Locus_17185_Transcript_1/1_Conf_1.000	129	0							
Locus_17186_Transcript_1/1_Conf_1.000	197	0							
Locus_17187_Transcript_1/1_Conf_1.000	405	0							
Locus_17188_Transcript_1/1_Conf_1.000	368	20	6.04E-52	XP_860709.1	93	206.838	118	110	PREDICTED: hypothetical protein XP_855616 isoform 2
Locus_17189_Transcript_1/1_Conf_1.000	430	20	2.27E-22	XP_002635357.1	72	108.612	92	67	C. briggsae CBR-CLN-3.3 protein
Locus_1719_Transcript_1/1_Conf_1.000	722	20	5.92E-14	EFO20048.1	51	82.0333	201	103	hypothetical protein LOAG_08441
Locus_17190_Transcript_1/1_Conf_1.000	135	0							
Locus_17191_Transcript_1/1_Conf_1.000	212	1	1.67E-09	EDL85946.1	71	65.855	53	38	rCG37353, isoform CRA_a
Locus_17192_Transcript_1/1_Conf_1.000	250	0							
Locus_17193_Transcript_1/1_Conf_1.000	309	0							
Locus_17194_Transcript_1/1_Conf_1.000	146	0							
Locus_17195_Transcript_1/1_Conf_1.000	659	20	1.32E-51	NP_498494.2	65	206.838	221	145	Central Region Assembly in meiosis abnormal family member (cra-1)
Locus_17196_Transcript_1/1_Conf_1.000	353	0							

Locus_17197_Transcript_1/1_Conf_1.000	510	20	1.27E-47	NP_505616.2	85	192.586	132	113	CULlin family member (cul-5)
Locus_17198_Transcript_1/1_Conf_1.000	419	0							
Locus_17199_Transcript_1/1_Conf_1.000	647	0							
Locus_172_Transcript_1/1_Conf_1.000	1053	5	5.84E-66	NP_497742.2	56	255.758	316	177	hypothetical protein C46F11.5
Locus_1720_Transcript_1/1_Conf_1.000	1258	20	5.42E-72	NP_496786.2	61	276.174	351	217	hypothetical protein F29C12.3
Locus_17200_Transcript_1/1_Conf_1.000	146	0							
Locus_17201_Transcript_1/1_Conf_1.000	351	0							
Locus_17202_Transcript_1/1_Conf_1.000	158	1	4.81E-04	NP_505683.1	70	47.7506	41	29	Poly-A Polymerase family member (pap-1)
Locus_17203_Transcript_1/1_Conf_1.000	794	20	4.67E-70	XP_002629667.1	73	163.31	140	103	C. briggsae CBR-TAG-165 protein
Locus_17204_Transcript_1/1_Conf_1.000	640	20	3.59E-43	AAO39166.1	92	178.718	98	91	nuclear receptor NHR-1
Locus_17205_Transcript_1/1_Conf_1.000	155	20	2.82E-12	XP_001895196.1	95	75.0998	47	45	Nematode cuticle collagen N-terminal domain containing protein

Locus_17206_Transcript_1/1_Conf_1.000	590	20	4.02E-61	BAG65301.1	100	238.039	106	106	unnamed protein product
Locus_17207_Transcript_1/1_Conf_1.000	232	0							
Locus_17208_Transcript_1/1_Conf_1.000	258	0							
Locus_17209_Transcript_1/1_Conf_1.000	415	2	5.55E-05	XP_002634505.1	62	50.8322	72	45	Hypothetical protein CBG08295
Locus_1721_Transcript_1/1_Conf_1.000	819	20	1.12E-41	AAK31453.4	64	174.481	209	135	Hypothetical protein C25H3.8
Locus_17210_Transcript_1/1_Conf_1.000	235	0							
Locus_17211_Transcript_1/1_Conf_1.000	354	20	6.19E-20	AAN34967.1	69	100.523	98	68	thioredoxin 2
Locus_17212_Transcript_1/1_Conf_1.000	275	0							
Locus_17213_Transcript_1/2_Conf_1.000	1049	2	2.56E-05	XP_843163.1	36	54.299	302	109	proteophosphoglycan 5
Locus_17213_Transcript_2/2_Conf_1.000	1049	2	2.56E-05	XP_843163.1	36	54.299	302	109	proteophosphoglycan 5
Locus_17214_Transcript_1/1_Conf_1.000	200	2	1.26E-04	NP_492157.2	56	49.6766	53	30	hypothetical protein D2005.6
Locus_17215_Transcript_1/1_Conf_1.000	559	4	2.52E-27	XP_002640034.1	61	125.561	180	111	C. briggsae CBR-EDC-3 protein
Locus_17216_Transcript_1/1_Conf_1.000	567	12	1.03E-71	NP_496205.2	83	273.092	188	157	PhosphoLipase C family member (plc-3)
Locus_17217_Transcript_1/1_Conf_1.000	249	20	1.00E-30	NP_508993.2	91	136.346	82	75	fatty Acid CoA Synthetase family member (acs-17)

Locus_17218_Transcript_1/1_Conf_1.000	187	4	1.61E-15	XP_002629798.1	83	85.8853	62	52	Hypothetical protein CBG01041
Locus_17219_Transcript_1/1_Conf_1.000	193	0							
Locus_1722_Transcript_1/2_Conf_1.000	1358	20	1.03E-79	EFO27010.1	79	301.982	215	170	uracil-DNA glycosylase
Locus_1722_Transcript_2/2_Conf_1.000	1148	20	8.07E-80	EFO27010.1	79	301.982	215	170	uracil-DNA glycosylase
Locus_17220_Transcript_1/1_Conf_1.000	134	0							
Locus_17221_Transcript_1/1_Conf_1.000	505	20	2.98E-25	EFO28229.1	83	118.242	72	60	hypothetical protein LOAG_00257
Locus_17222_Transcript_1/1_Conf_1.000	339	0							
Locus_17223_Transcript_1/1_Conf_1.000	154	6	3.81E-09	NP_509110.1	94	64.6994	36	34	TransThyretin-Related family domain family member (ttr-6)
Locus_17224_Transcript_1/1_Conf_1.000	172	0							
Locus_17225_Transcript_1/1_Conf_1.000	313	20	4.36E-29	XP_002640716.1	75	130.954	104	78	Hypothetical protein CBG19783
Locus_17226_Transcript_1/1_Conf_1.000	460	20	2.29E-59	NP_001022488.1	85	231.491	127	109	UNCoordinated family member (unc-52)
Locus_17227_Transcript_1/1_Conf_1.000	172	0							
Locus_17228_Transcript_1/1_Conf_1.000	188	0							
Locus_17229_Transcript_1/1_Conf_1.000	303	20	1.20E-23	XP_002636715.1	76	112.849	97	74	Hypothetical protein CBG23434
Locus_1723_Transcript_1/1_Conf_1.000	1662	20	2.87E-122	NP_500364.2	72	443.736	424	307	Tumor Susceptibility Gene homolog family member (tsg-101)
Locus_17230_Transcript_1/1_Conf_1.000	299	20	4.90E-25	NP_496829.1	73	117.472	94	69	hypothetical protein Y48E1C.2

Locus_17231_Transcript_1/1_Conf_1.000	163	20	1.02E-22	XP_002925888.1	100	109.768	54	54	PREDICTED: eukaryotic translation initiation factor 3 subunit K-like
Locus_17232_Transcript_1/1_Conf_1.000	628	20	1.91E-94	NP_505734.2	93	348.977	193	181	Protein PHosphatase family member (pph-1)
Locus_17233_Transcript_1/1_Conf_1.000	197	0							
Locus_17234_Transcript_1/1_Conf_1.000	309	20	2.55E-42	CAX65056.1	91	174.866	102	93	C. elegans protein F25C8.3d, partially confirmed by transcript evidence
Locus_17235_Transcript_1/1_Conf_1.000	149	0							
Locus_17236_Transcript_1/1_Conf_1.000	229	0							
Locus_17237_Transcript_1/1_Conf_1.000	1166	20	1.00E-101	XP_001898107.1	67	374.785	378	257	PDZ-domain protein scribble
Locus_17238_Transcript_1/1_Conf_1.000	349	0							
Locus_17239_Transcript_1/1_Conf_1.000	375	0							

Locus_1724_Transcript_1/1_Conf_1.000	3328	20	0	XP_002640935.1	73	919.842	830	607	C. briggsae CBR-SSL-1 protein
Locus_17240_Transcript_1/1_Conf_1.000	167	0							
Locus_17241_Transcript_1/1_Conf_1.000	154	14	2.23E-17	XP_002642067.1	92	92.0485	51	47	Hypothetical protein CBG18006
Locus_17242_Transcript_1/1_Conf_1.000	451	0							
Locus_17243_Transcript_1/1_Conf_1.000	322	7	6.25E-12	XP_002630536.1	57	73.9442	107	62	Hypothetical protein CBG12976
Locus_17244_Transcript_1/1_Conf_1.000	1201	20	4.64E-49	NP_491336.2	62	199.904	240	151	NuDiX family member (ndx-7)
Locus_17245_Transcript_1/1_Conf_1.000	431	0							
Locus_17246_Transcript_1/1_Conf_1.000	262	0							
Locus_17247_Transcript_1/1_Conf_1.000	224	20	3.45E-18	NP_491222.2	74	94.7449	74	55	hypothetical protein C32E8.9
Locus_17248_Transcript_1/1_Conf_1.000	506	2	2.89E-12	NP_509145.2	53	75.0998	129	69	AuTophaGy (yeast Atg homolog) family member (atg-2)
Locus_17249_Transcript_1/1_Conf_1.000	460	0							
Locus_1725_Transcript_1/2_Conf_1.000	2486	20	3.22E-139	XP_002639606.1	60	500.745	690	414	C. briggsae CBR-FRM-4 protein

Locus_1725_Transcript_2/2_Conf_1.000	2435	20	3.25E-136	XP_002639606.1	61	490.73	667	410	C. briggsae CBR-FRM-4 protein
Locus_17250_Transcript_1/1_Conf_1.000	477	0							
Locus_17251_Transcript_1/1_Conf_1.000	332	3	7.82E-07	NP_495954.1	62	56.9954	102	64	Regulator of HYpoxia-inducible factor (hif-1) family member (rhy-1)
Locus_17252_Transcript_1/1_Conf_1.000	495	20	1.36E-35	NP_505508.1	74	152.525	136	101	hypothetical protein F40F9.6
Locus_17253_Transcript_1/1_Conf_1.000	443	20	1.71E-17	NP_509794.2	100	92.4337	45	45	honeybee MBlk-1 Related factor family member (mbr-1)
Locus_17254_Transcript_1/1_Conf_1.000	320	2	1.11E-08	XP_002633778.1	60	63.1586	96	58	Hypothetical protein CBG03469
Locus_17255_Transcript_1/1_Conf_1.000	465	5	1.18E-10	XP_001900440.1	63	69.707	87	55	hypothetical protein Bm1_44890
Locus_17256_Transcript_1/1_Conf_1.000	164	3	2.29E-14	NP_493603.1	86	82.0333	52	45	PaTched Related family member (ptr-23)
Locus_17257_Transcript_1/1_Conf_1.000	716	0							
Locus_17258_Transcript_1/1_Conf_1.000	169	6	4.00E-19	XP_001899075.1	92	97.8265	55	51	hypothetical protein Bm1_38110
Locus_17259_Transcript_1/1_Conf_1.000	418	20	3.67E-36	NP_740853.2	77	154.451	122	95	TOMosyn synaptic protein family member (tom-1)
Locus_1726_Transcript_1/1_Conf_1.000	1901	20	0	NP_508550.1	87	830.091	544	476	Ral GDS-Like family member (rgl-1)

Locus_17260_Transcript_1/1_Conf_1.000	217	0							
Locus_17261_Transcript_1/1_Conf_1.000	476	20	1.42E-16	XP_002641832.1	55	89.3521	158	88	Hypothetical protein CBG16501
Locus_17262_Transcript_1/1_Conf_1.000	308	20	4.37E-42	EFN83153.1	100	174.096	90	90	Histone H3.3
Locus_17263_Transcript_1/1_Conf_1.000	203	20	1.47E-29	XP_002762066.1	100	132.494	64	64	PREDICTED: transmembrane protein 147-like
Locus_17264_Transcript_1/1_Conf_1.000	374	20	2.44E-48	XP_002642870.1	88	194.897	120	106	Hypothetical protein CBG15140
Locus_17265_Transcript_1/1_Conf_1.000	161	20	1.39E-11	NP_886553.3	100	72.7886	34	34	eukaryotic translation initiation factor 4 gamma 1 isoform 1
Locus_17266_Transcript_1/2_Conf_1.000	384	0							
Locus_17266_Transcript_2/2_Conf_1.000	380	0							
Locus_17267_Transcript_1/1_Conf_1.000	305	1	6.19E-04	XP_002637243.1	64	47.3654	59	38	Hypothetical protein CBG18919
Locus_17268_Transcript_1/1_Conf_1.000	220	0							
Locus_17269_Transcript_1/1_Conf_1.000	130	0							
Locus_1727_Transcript_1/1_Conf_1.000	761	20	2.48E-37	XP_002631715.1	63	159.844	181	115	Hypothetical protein CBG20916
Locus_17270_Transcript_1/1_Conf_1.000	233	0							
Locus_17271_Transcript_1/1_Conf_1.000	372	20	1.75E-30	AAN11401.1	68	135.576	122	84	metalloprotease 1 precursor

Locus_17272_Transcript_1/1_Conf_1.000	238	20	2.34E-35	XP_002634104.1	100	151.754	78	78	Hypothetical protein CBG01656
Locus_17273_Transcript_1/1_Conf_1.000	293	3	6.28E-04	ZP_04116816.1	59	47.3654	54	32	Collagen triple helix repeat domain protein
Locus_17274_Transcript_1/1_Conf_1.000	207	20	1.08E-24	NP_612634.3	100	116.316	57	57	synaptotagmin-8
Locus_17275_Transcript_1/1_Conf_1.000	154	4	4.22E-08	EFO20018.1	68	61.2326	50	34	negative elongation factor C/D
Locus_17276_Transcript_1/1_Conf_1.000	241	20	7.26E-29	XP_002637169.1	93	130.183	77	72	C. briggsae CBR-MRE-11 protein

Locus_17277_Transcript_1/1_Conf_1.000	604	20	8.40E-73	XP_002646357.1	80	276.944	186	149	Hypothetical protein CBG12071
Locus_17278_Transcript_1/1_Conf_1.000	225	20	1.07E-27	NP_502964.2	87	126.331	74	65	hypothetical protein Y51H4A.7
Locus_17279_Transcript_1/1_Conf_1.000	285	1	2.84E-04	XP_002638582.1	58	48.521	79	46	Hypothetical protein CBG05629
Locus_1728_Transcript_1/1_Conf_1.000	230	2	5.85E-18	AAQ18782.1	94	93.9745	51	48	GATA transcription factor
Locus_17280_Transcript_1/1_Conf_1.000	186	0							
Locus_17281_Transcript_1/1_Conf_1.000	130	0							
Locus_17282_Transcript_1/1_Conf_1.000	547	0							
Locus_17283_Transcript_1/1_Conf_1.000	714	20	3.81E-90	NP_501853.1	87	335.109	248	216	TetraSPanin family member (tsp-12)
Locus_17284_Transcript_1/1_Conf_1.000	309	6	3.58E-28	XP_002633574.1	71	127.872	103	74	Hypothetical protein CBG05448
Locus_17285_Transcript_1/1_Conf_1.000	348	20	5.09E-30	NP_495375.3	84	134.035	94	79	hypothetical protein T14B4.1
Locus_17286_Transcript_1/1_Conf_1.000	429	2	2.69E-07	NP_493320.1	50	58.5362	102	51	hypothetical protein ZK1053.2
Locus_17287_Transcript_1/1_Conf_1.000	400	0							
Locus_17288_Transcript_1/1_Conf_1.000	480	0							
Locus_17289_Transcript_1/1_Conf_1.000	169	20	1.28E-25	XP_002801133.1	100	119.398	55	55	PREDICTED: lysosomal alpha-mannosidase-like
Locus_1729_Transcript_1/1_Conf_1.000	428	0							
Locus_17290_Transcript_1/1_Conf_1.000	141	1	3.59E-04	XP_002634634.1	63	48.1358	44	28	Hypothetical protein CBG18497
Locus_17291_Transcript_1/1_Conf_1.000	217	0							
Locus_17292_Transcript_1/1_Conf_1.000	548	20	6.44E-65	EFO19031.1	83	250.366	176	147	hypothetical protein LOAG_09464
Locus_17293_Transcript_1/1_Conf_1.000	214	0							
Locus_17294_Transcript_1/1_Conf_1.000	294	20	4.43E-42	EFO26082.1	91	174.096	98	90	hypothetical protein LOAG_02394
Locus_17295_Transcript_1/1_Conf_1.000	311	20	1.01E-22	NP_491887.1	70	109.768	98	69	hypothetical protein F57B10.8
Locus_17296_Transcript_1/1_Conf_1.000	152	0							

Locus_17297_Transcript_1/1_Conf_1.000	159	20	4.17E-24	XP_002816748.1	100	114.39	53	53	PREDICTED: LOW QUALITY PROTEIN: large proline-rich protein BAT2-like
Locus_17298_Transcript_1/1_Conf_1.000	362	0							
Locus_17299_Transcript_1/1_Conf_1.000	238	0							
Locus_173_Transcript_1/2_Conf_1.000	2157	7	3.84E-08	XP_002632964.1	40	65.0846	151	61	Hypothetical protein CBG21722
Locus_173_Transcript_2/2_Conf_1.000	1791	4	2.04E-12	XP_002632964.1	35	78.9518	261	92	Hypothetical protein CBG21722
Locus_1730_Transcript_1/1_Conf_1.000	1908	20	0	XP_002629713.1	83	681.404	564	473	Hypothetical protein CBG00943
Locus_17300_Transcript_1/2_Conf_1.000	1008	20	1.53E-84	XP_002633358.1	76	317.39	244	187	C. briggsae CBR-ACL-3 protein

Locus_17300_Transcript_2/2_Conf_1.000	1005	20	1.53E-84	XP_002633358.1	76	317.39	244	187	C. briggsae CBR-ACL-3 protein
Locus_17301_Transcript_1/1_Conf_1.000	258	0							
Locus_17302_Transcript_1/1_Conf_1.000	153	0							
Locus_17303_Transcript_1/1_Conf_1.000	397	20	7.62E-34	EFO23539.1	74	146.747	131	97	hypothetical protein LOAG_04946
Locus_17304_Transcript_1/1_Conf_1.000	332	20	2.75E-44	XP_001896744.1	83	181.415	110	92	Trehalase family protein
Locus_17305_Transcript_1/1_Conf_1.000	153	0							
Locus_17306_Transcript_1/1_Conf_1.000	447	8	1.09E-48	XP_002644403.1	84	196.052	148	125	Hypothetical protein CBG14248
Locus_17307_Transcript_1/1_Conf_1.000	375	4	9.59E-29	NP_491019.1	67	129.798	124	84	hypothetical protein F40E3.5
Locus_17308_Transcript_1/1_Conf_1.000	543	0							
Locus_17309_Transcript_1/1_Conf_1.000	150	20	7.98E-07	XP_002640915.1	71	56.9954	49	35	Hypothetical protein CBG00476
Locus_1731_Transcript_1/1_Conf_1.000	1129	20	1.57E-35	XP_002640023.1	65	154.836	206	135	Hypothetical protein CBG12495
Locus_17310_Transcript_1/1_Conf_1.000	171	20	1.52E-18	XP_002643095.1	85	95.9005	56	48	C. briggsae CBR-GLY-11 protein

Locus_17311_Transcript_1/1_Conf_1.000	190	20	1.83E-19	BAF83766.1	100	98.9821	60	60	unnamed protein product
Locus_17312_Transcript_1/1_Conf_1.000	414	20	5.89E-31	XP_002647605.1	75	137.117	105	79	Hypothetical protein CBG06696
Locus_17313_Transcript_1/1_Conf_1.000	469	20	8.67E-22	XP_002640707.1	68	106.686	112	77	Hypothetical protein CBG19773
Locus_17314_Transcript_1/1_Conf_1.000	265	4	1.10E-16	CBI63237.1	63	89.7373	104	66	C. elegans protein Y111B2A.4a, partially confirmed by transcript evidence
Locus_17315_Transcript_1/1_Conf_1.000	287	20	4.51E-18	EFO23906.1	69	94.3597	85	59	hypothetical protein LOAG_04576
Locus_17316_Transcript_1/1_Conf_1.000	437	2	3.49E-07	XP_002636583.1	51	58.151	146	75	Hypothetical protein CBG23277
Locus_17317_Transcript_1/1_Conf_1.000	160	0							
Locus_17318_Transcript_1/1_Conf_1.000	245	0							
Locus_17319_Transcript_1/1_Conf_1.000	199	0							
Locus_1732_Transcript_1/1_Conf_1.000	2295	20	5.32E-165	NP_501339.2	60	586.26	740	447	neuRonal IGCAM family member (rig-4)
Locus_17320_Transcript_1/1_Conf_1.000	383	0							
Locus_17321_Transcript_1/1_Conf_1.000	400	0							
Locus_17322_Transcript_1/1_Conf_1.000	160	10	1.35E-06	XP_001902385.1	72	56.225	48	35	Patched family protein
Locus_17323_Transcript_1/1_Conf_1.000	153	0							
Locus_17324_Transcript_1/1_Conf_1.000	345	20	1.22E-23	NP_502129.1	85	112.849	70	60	hypothetical protein B0035.5
Locus_17325_Transcript_1/1_Conf_1.000	137	0							
Locus_17326_Transcript_1/1_Conf_1.000	656	20	1.04E-40	EFO23575.1	89	170.629	104	93	proteasome regulatory particle
Locus_17327_Transcript_1/1_Conf_1.000	307	0							

Locus_17328_Transcript_1/1_Conf_1.000	291	5	3.01E-06	AAX47336.1	65	55.0694	52	34	beta-catenin
Locus_17329_Transcript_1/1_Conf_1.000	294	0							
Locus_1733_Transcript_1/1_Conf_1.000	406	0							
Locus_17330_Transcript_1/1_Conf_1.000	155								
Locus_17331_Transcript_1/1_Conf_1.000	281	20	1.19E-15	EFO20952.1	70	86.2705	68	48	endonuclease/Exonuclease/phosphatase
Locus_17332_Transcript_1/2_Conf_1.000	435	0							
Locus_17332_Transcript_2/2_Conf_1.000	444	0							
Locus_17333_Transcript_1/1_Conf_1.000	251	20	2.31E-27	CAR63587.1	87	125.176	79	69	putative BolA-like family protein
Locus_17334_Transcript_1/1_Conf_1.000	480	20	3.67E-49	XP_002634049.1	75	197.593	158	120	Hypothetical protein CBG01588
Locus_17335_Transcript_1/1_Conf_1.000	199	20	2.07E-07	ACI49169.1	65	58.9214	64	42	hypothetical protein Csp3_JD01.001
Locus_17336_Transcript_1/1_Conf_1.000	185	0							
Locus_17337_Transcript_1/1_Conf_1.000	441	0							
Locus_17338_Transcript_1/1_Conf_1.000	538	20	1.56E-28	EFO23663.1	60	129.413	181	110	myosin xviiia
Locus_17339_Transcript_1/1_Conf_1.000	245	0							
Locus_1734_Transcript_1/2_Conf_1.000	262	0							
Locus_1734_Transcript_2/2_Conf_1.000	181	0							
Locus_17340_Transcript_1/1_Conf_1.000	155	0							
Locus_17341_Transcript_1/1_Conf_1.000	175	20	3.73E-09	XP_002633845.1	77	64.6994	48	37	C. briggsae CBR-BEC-1 protein
Locus_17342_Transcript_1/1_Conf_1.000	749	20	2.92E-91	XP_002630786.1	85	338.961	227	195	Hypothetical protein CBG02483
Locus_17343_Transcript_1/1_Conf_1.000	258	20	2.63E-18	BAD88409.1	66	95.1301	87	58	multidrug resistance-associated protein

Locus_17344_Transcript_1/1_Conf_1.000	387	20	7.76E-15	XP_001626861.1	67	83.5741	84	57	predicted protein
Locus_17345_Transcript_1/1_Conf_1.000	337	20	3.71E-25	CBA11992.1	70	117.857	111	78	endonuclease-reverse transcriptase HmRTE-e01
Locus_17346_Transcript_1/1_Conf_1.000	859	20	3.05E-125	XP_002643856.1	90	452.21	286	260	C. briggsae CBR-AAT-3 protein
Locus_17347_Transcript_1/1_Conf_1.000	145	1	1.56E-18	EAW60095.1	100	95.9005	44	44	hCG2041804
Locus_17348_Transcript_1/1_Conf_1.000	306	0							
Locus_17349_Transcript_1/1_Conf_1.000	189	2	1.00E-09	XP_002635377.1	91	66.6254	34	31	Hypothetical protein CBG00768
Locus_1735_Transcript_1/3_Conf_0.400	1343	20	0	NP_492790.1	84	675.241	448	377	hypothetical protein B0511.12
Locus_1735_Transcript_2/3_Conf_0.400	606	20	8.07E-31	XP_002640257.1	74	137.502	109	81	Hypothetical protein CBG12782
Locus_1735_Transcript_3/3_Conf_0.600	2986	20	0	NP_492790.1	74	1149.04	994	736	hypothetical protein B0511.12
Locus_17350_Transcript_1/1_Conf_1.000	185	0							
Locus_17351_Transcript_1/1_Conf_1.000	303	20	1.37E-27	NP_492590.1	69	125.946	101	70	hypothetical protein K02A11.3
Locus_17352_Transcript_1/1_Conf_1.000	479	20	2.09E-44	XP_002640631.1	73	181.8	165	121	Hypothetical protein CBG08749
Locus_17353_Transcript_1/1_Conf_1.000	252	20	1.45E-16	NP_496282.2	69	89.3521	83	58	hypothetical protein C14A4.3
Locus_17354_Transcript_1/1_Conf_1.000	251	0							
Locus_17355_Transcript_1/1_Conf_1.000	739	20	1.38E-45	EFO17241.1	58	187.193	234	138	hypothetical protein LOAG_11260
Locus_17356_Transcript_1/1_Conf_1.000	518	7	1.74E-31	XP_002647546.1	58	139.043	171	100	C. briggsae CBR-PME-5 protein
Locus_17357_Transcript_1/1_Conf_1.000	586	0							
Locus_17358_Transcript_1/1_Conf_1.000	535	7	4.18E-18	CAA84693.3	55	94.7449	177	98	C. elegans protein D2045.2, partially confirmed by transcript evidence
Locus_17359_Transcript_1/1_Conf_1.000	513	0							

Locus_1736_Transcript_1/1_Conf_1.000	774	20	1.67E-36	EFO16781.1	55	157.147	252	140	bone morphogenic protein 6
Locus_17360_Transcript_1/1_Conf_1.000	187	1	9.76E-05	ABD39382.1	75	50.0618	61	46	putative galectin protein 140
Locus_17361_Transcript_1/1_Conf_1.000	282	0							
Locus_17362_Transcript_1/1_Conf_1.000	168	0							
Locus_17363_Transcript_1/1_Conf_1.000	173	0							
Locus_17364_Transcript_1/1_Conf_1.000	224	0							
Locus_17365_Transcript_1/1_Conf_1.000	279	20	2.48E-29	XP_002646489.1	86	131.724	97	84	Hypothetical protein CBG19471
Locus_17366_Transcript_1/1_Conf_1.000	390	20	5.54E-13	NP_001022669.1	72	77.411	74	54	hypothetical protein K08E3.10
Locus_17367_Transcript_1/1_Conf_1.000	486	0							
Locus_17368_Transcript_1/1_Conf_1.000	441	20	3.41E-42	NP_508216.1	78	174.481	146	115	Sorting NeXin family member (snx-1)
Locus_17369_Transcript_1/1_Conf_1.000	274	20	2.18E-25	XP_002634480.1	73	118.627	90	66	Hypothetical protein CBG10739
Locus_1737_Transcript_1/5_Conf_0.333	911	20	2.81E-51	CAT00091.1	99	198.749	104	103	translationally controlled tumor protein
Locus_1737_Transcript_2/5_Conf_0.167	658	0							

Locus_1737_Transcript_3/5_Conf_0.417	859	20	4.31E-87	CAT00091.1	98	325.479	181	178	translationally controlled tumor protein
Locus_1737_Transcript_4/5_Conf_0.500	1311	20	8.29E-87	CAT00091.1	98	325.479	181	178	translationally controlled tumor protein
Locus_1737_Transcript_5/5_Conf_0.333	875	20	7.75E-132	XP_002639809.1	97	474.167	264	257	C. briggsae CBR-RPA-0 protein
Locus_17370_Transcript_1/1_Conf_1.000	750	5	7.55E-63	NP_495112.1	69	244.588	263	182	hypothetical protein C25H3.11
Locus_17371_Transcript_1/1_Conf_1.000	193	0							
Locus_17372_Transcript_1/1_Conf_1.000	135	0							
Locus_17373_Transcript_1/1_Conf_1.000	315	15	2.73E-15	XP_002646997.1	60	85.1149	104	63	Hypothetical protein CBG22453
Locus_17374_Transcript_1/1_Conf_1.000	527	20	4.80E-104	XP_001101364.2	100	380.178	175	175	PREDICTED: SPARC
Locus_17375_Transcript_1/1_Conf_1.000	221	0							
Locus_17376_Transcript_1/2_Conf_1.000	890	20	2.35E-67	XP_002633534.1	82	259.996	223	183	Hypothetical protein CBG05401
Locus_17376_Transcript_2/2_Conf_1.000	890	20	2.35E-67	XP_002633534.1	82	259.996	223	183	Hypothetical protein CBG05401
Locus_17377_Transcript_1/1_Conf_1.000	293	20	6.23E-28	XP_002630862.1	77	127.102	98	76	C. briggsae CBR-RRT-2 protein
Locus_17378_Transcript_1/1_Conf_1.000	486	20	1.87E-32	NP_499394.2	68	142.124	159	109	hypothetical protein C44B9.1

Locus_17379_Transcript_1/1_Conf_1.000	213	20	4.10E-16	NP_504274.2	77	87.8113	70	54	UDP-GlucuronosylTransferase family member (ugt-61)
Locus_1738_Transcript_1/1_Conf_1.000	1269	20	0	XP_002638687.1	96	796.964	422	409	Hypothetical protein CBG11882
Locus_17380_Transcript_1/1_Conf_1.000	1266	20	5.97E-10	XP_001602928.1	45	70.0922	328	148	PREDICTED: similar to GA21383-PA
Locus_17381_Transcript_1/1_Conf_1.000	243	0							
Locus_17382_Transcript_1/1_Conf_1.000	374	8	3.80E-09	XP_002631036.1	55	64.6994	114	63	Hypothetical protein CBG02793
Locus_17383_Transcript_1/1_Conf_1.000	356	0							
Locus_17384_Transcript_1/1_Conf_1.000	483	20	5.85E-26	EFO25878.1	70	120.553	140	98	RDD family protein
Locus_17385_Transcript_1/1_Conf_1.000	155	0							
Locus_17386_Transcript_1/1_Conf_1.000	356	0							
Locus_17387_Transcript_1/1_Conf_1.000	226								
Locus_17388_Transcript_1/2_Conf_1.000	631	0							
Locus_17388_Transcript_2/2_Conf_1.000	617	0							
Locus_17389_Transcript_1/1_Conf_1.000	178	0							
Locus_1739_Transcript_1/1_Conf_1.000	586	20	5.84E-52	NP_501020.2	93	207.608	115	107	hypothetical protein C01B10.3
Locus_17390_Transcript_1/1_Conf_1.000	222	0							
Locus_17391_Transcript_1/1_Conf_1.000	184	0							
Locus_17392_Transcript_1/1_Conf_1.000	302	2	1.02E-06	XP_002645841.1	64	56.6102	62	40	Hypothetical protein CBG07574

Locus_17393_Transcript_1/1_Conf_1.000	464	20	1.26E-69	A8X3A7.2	89	259.225	142	127	Integrin alpha pat-2
Locus_17394_Transcript_1/1_Conf_1.000	908	20	2.75E-111	XP_002631326.1	74	405.986	303	227	C. briggsae CBR-FUT-1 protein
Locus_17395_Transcript_1/1_Conf_1.000	416	6	3.22E-37	Q20548.2	76	157.918	138	106	Probable RING finger protein 207 homolog
Locus_17396_Transcript_1/1_Conf_1.000	791	1	3.66E-10	EFO16945.1	46	69.707	215	101	hypothetical protein LOAG_11558
Locus_17397_Transcript_1/1_Conf_1.000	452	20	9.12E-64	NP_492483.1	85	246.128	150	128	hypothetical protein F26H9.5
Locus_17398_Transcript_1/1_Conf_1.000	594	0							
Locus_17399_Transcript_1/1_Conf_1.000	333	4	8.33E-09	NP_496398.2	66	63.5438	65	43	hypothetical protein T06D8.1
Locus_174_Transcript_1/8_Conf_0.600	1809	20	7.07E-29	XP_002631180.1	56	133.65	253	142	C. briggsae CBR-NASP-1 protein
Locus_174_Transcript_2/8_Conf_0.560	1797	20	7.00E-29	XP_002631180.1	56	133.65	253	142	C. briggsae CBR-NASP-1 protein
Locus_174_Transcript_3/8_Conf_0.400	935	17	6.01E-08	NP_506298.1	58	62.7734	95	56	NASP (human Nuclear Autoantigenic Sperm Protein) homolog family member (nasp-2)
Locus_174_Transcript_4/8_Conf_0.560	1809	20	1.21E-28	XP_002631180.1	55	132.88	253	141	C. briggsae CBR-NASP-1 protein
Locus_174_Transcript_5/8_Conf_0.560	1816	20	7.10E-29	XP_002631180.1	56	133.65	253	142	C. briggsae CBR-NASP-1 protein
Locus_174_Transcript_6/8_Conf_0.360	935	17	6.01E-08	NP_506298.1	58	62.7734	95	56	NASP (human Nuclear Autoantigenic Sperm Protein) homolog family member (nasp-2)

Locus_174_Transcript_7/8_Conf_0.200	445	20	7.64E-10	NP_506298.1	60	67.0106	98	59	NASP (human Nuclear Autoantigenic Sperm Protein) homolog family member (nasp-2)
Locus_174_Transcript_8/8_Conf_0.560	1809	20	7.07E-29	XP_002631180.1	56	133.65	253	142	C. briggsae CBR-NASP-1 protein
Locus_1740_Transcript_1/1_Conf_1.000	759	20	9.04E-56	XP_002637954.1	68	221.09	238	164	Hypothetical protein CBG04771
Locus_17400_Transcript_1/1_Conf_1.000	262	20	5.26E-35	ACJ65168.1	94	150.599	87	82	Uncoordinated protein 68, isoform b, partially confirmed by transcript evidence
Locus_17401_Transcript_1/1_Conf_1.000	244	2	9.83E-10	XP_002642848.1	72	66.6254	76	55	Hypothetical protein CBG15114
Locus_17402_Transcript_1/1_Conf_1.000	220	20	3.03E-22	XP_002648832.1	82	108.227	73	60	Hypothetical protein CBG16947

Locus_17403_Transcript_1/1_Conf_1.000	281	20	6.73E-35	NP_495838.2	84	150.214	93	79	PYRimidine biosynthesis family member (pyr-1)
Locus_17404_Transcript_1/1_Conf_1.000	935	20	2.62E-112	XP_002646922.1	78	409.453	311	245	Hypothetical protein CBG19625
Locus_17405_Transcript_1/1_Conf_1.000	345	20	7.12E-40	NP_497319.1	85	166.777	114	97	hypothetical protein Y46E12BL.2
Locus_17406_Transcript_1/1_Conf_1.000	920	20	3.39E-40	XP_002633792.1	89	169.859	86	77	Hypothetical protein CBG03484
Locus_17407_Transcript_1/1_Conf_1.000	151	0							
Locus_17408_Transcript_1/1_Conf_1.000	167	20	7.59E-10	EFN89993.1	67	67.0106	53	36	Probable cytochrome P450 6a13
Locus_17409_Transcript_1/1_Conf_1.000	446	2	1.03E-22	XP_002631708.1	82	109.768	87	72	Hypothetical protein CBG20908
Locus_1741_Transcript_1/1_Conf_1.000	1858	20	0	XP_002630288.1	92	828.55	547	507	C. briggsae CBR-CCT-1 protein
Locus_17410_Transcript_1/1_Conf_1.000	239	0							

Locus_17411_Transcript_1/1_Conf_1.000	227	5	5.70E-05	NP_001021268.1	72	50.8322	40	29	UNCoordinated family member (unc-44)
Locus_17412_Transcript_1/1_Conf_1.000	423	20	1.58E-23	NP_001041046.1	61	112.464	120	74	hypothetical protein Y57G11C.22
Locus_17413_Transcript_1/1_Conf_1.000	130	0							
Locus_17414_Transcript_1/1_Conf_1.000	437	0							
Locus_17415_Transcript_1/1_Conf_1.000	221	0							
Locus_17416_Transcript_1/1_Conf_1.000	393	9	1.19E-18	CAB60377.4	59	96.2857	134	80	C. elegans protein Y46G5A.29, partially confirmed by transcript evidence
Locus_17417_Transcript_1/1_Conf_1.000	529	0							
Locus_17418_Transcript_1/1_Conf_1.000	205	0							
Locus_17419_Transcript_1/1_Conf_1.000	446	20	1.16E-58	XP_002642359.1	85	229.18	148	127	Hypothetical protein CBG18356
Locus_1742_Transcript_1/1_Conf_1.000	452	1	2.79E-04	XP_002648823.1	42	48.521	85	36	Hypothetical protein CBG16936
Locus_17420_Transcript_1/1_Conf_1.000	205	0							
Locus_17421_Transcript_1/1_Conf_1.000	206	2	1.25E-04	EFO26312.1	65	49.6766	41	27	hypothetical protein LOAG_02167
Locus_17422_Transcript_1/1_Conf_1.000	149	20	7.98E-15	NP_001129924.1	81	83.5741	49	40	SLOwpoke potassium channel family member (slo-2)
Locus_17423_Transcript_1/1_Conf_1.000	269	0							

Locus_17424_Transcript_1/1_Conf_1.000	146	20	3.71E-20	XP_002832350.1	100	101.293	48	48	PREDICTED: LOW QUALITY PROTEIN: filamin-A-like
Locus_17425_Transcript_1/1_Conf_1.000	411	13	3.23E-45	XP_002639996.1	78	184.496	136	107	Hypothetical protein CBG10826
Locus_17426_Transcript_1/1_Conf_1.000	345	20	5.28E-35	NP_001023496.1	75	150.599	114	86	L1 CAM ADhesion molecule homolog family member (lad-2)
Locus_17427_Transcript_1/1_Conf_1.000	649	4	1.99E-12	XP_002640451.1	68	76.6406	63	43	Hypothetical protein CBG08508
Locus_17428_Transcript_1/1_Conf_1.000	274	0							
Locus_17429_Transcript_1/2_Conf_1.000	322	0							
Locus_17429_Transcript_2/2_Conf_1.000	349	0							
Locus_1743_Transcript_1/1_Conf_1.000	1269	5	3.85E-33	NP_740830.2	55	147.132	246	137	hypothetical protein Y47G6A.29
Locus_17430_Transcript_1/1_Conf_1.000	219	20	5.70E-29	XP_002638487.1	95	130.568	73	70	Hypothetical protein CBG12917

Locus_17431_Transcript_1/1_Conf_1.000	143	20	5.34E-16	NP_501984.1	93	87.4261	47	44	Na/Ca eXchangers family member (ncx-3)
Locus_17432_Transcript_1/1_Conf_1.000	193	20	5.30E-19	XP_002405101.1	87	97.4413	62	54	protein 4.1G, putative
Locus_17433_Transcript_1/1_Conf_1.000	814	20	2.23E-42	NP_740987.2	55	176.792	268	149	hypothetical protein C27D6.4
Locus_17434_Transcript_1/1_Conf_1.000	216	0							
Locus_17435_Transcript_1/1_Conf_1.000	613	0							
Locus_17436_Transcript_1/1_Conf_1.000	390	4	1.04E-35	NP_504440.2	65	152.91	147	97	hypothetical protein F08F3.1
Locus_17437_Transcript_1/1_Conf_1.000	142	0							
Locus_17438_Transcript_1/1_Conf_1.000	130	0							
Locus_17439_Transcript_1/1_Conf_1.000	176	0							
Locus_1744_Transcript_1/1_Conf_1.000	1178	20	3.00E-61	EFO16596.1	65	240.35	279	183	hypothetical protein LOAG_11909
Locus_17440_Transcript_1/1_Conf_1.000	1197	20	6.74E-133	XP_002637705.1	86	478.404	312	271	Hypothetical protein CBG11570
Locus_17441_Transcript_1/1_Conf_1.000	196	20	1.21E-23	XP_002828959.1	93	112.849	62	58	PREDICTED: hypothetical protein LOC100434366
Locus_17442_Transcript_1/1_Conf_1.000	967	20	7.93E-152	XP_001900845.1	92	540.806	322	297	Beta-centractin

Locus_17443_Transcript_1/1_Conf_1.000	150	20	3.57E-15	XP_002821493.1	100	84.7297	39	39	PREDICTED: LOW QUALITY PROTEIN: serine/threonine-protein phosphatase PP1-alpha catalytic subunit-like
Locus_17444_Transcript_1/1_Conf_1.000	195	0							
Locus_17445_Transcript_1/1_Conf_1.000	404	20	3.19E-16	EFO22662.1	68	88.1965	82	56	exostosin family protein
Locus_17446_Transcript_1/1_Conf_1.000	154	4	1.83E-11	XP_002646726.1	74	72.4034	50	37	Hypothetical protein CBG13109
Locus_17447_Transcript_1/1_Conf_1.000	133	20	2.77E-12	NP_001040856.1	90	75.0998	43	39	PAPS (adenosine 3'-phosphate 5'-phosphosulfate) Transporter family member (pst-2)
Locus_17448_Transcript_1/1_Conf_1.000	189	0							
Locus_17449_Transcript_1/1_Conf_1.000	595	0							
Locus_1745_Transcript_1/1_Conf_1.000	371	0							
Locus_17450_Transcript_1/1_Conf_1.000	602	3	6.17E-52	XP_002643880.1	67	207.608	200	134	Hypothetical protein CBG02118
Locus_17451_Transcript_1/1_Conf_1.000	893	0							
Locus_17452_Transcript_1/1_Conf_1.000	455	0							
Locus_17453_Transcript_1/1_Conf_1.000	168	0							
Locus_17454_Transcript_1/1_Conf_1.000	531	1	3.21E-26	CAR63592.1	65	121.709	141	93	hypothetical protein
Locus_17455_Transcript_1/1_Conf_1.000	367	20	4.34E-42	XP_002630208.1	83	174.096	117	98	C. briggsae CBR-LACT-4 protein
Locus_17456_Transcript_1/1_Conf_1.000	195	6	7.37E-21	NP_001021988.1	79	103.605	63	50	Epithelial Fusion Failure family member (eff-1)
Locus_17457_Transcript_1/1_Conf_1.000	230	0							
Locus_17458_Transcript_1/1_Conf_1.000	217	4	1.04E-27	NP_492127.1	93	126.331	72	67	Low-density lipoprotein Receptor Related family member (lrp-1)
Locus_17459_Transcript_1/1_Conf_1.000	128	2	2.98E-06	AAV28323.2	70	55.0694	40	28	Hypothetical protein T15B7.1
Locus_1746_Transcript_1/1_Conf_1.000	488	1	8.18E-04	NP_001024088.1	46	46.9802	116	54	hypothetical protein T01C3.11

Locus_17460_Transcript_1/1_Conf_1.000	769	20	2.62E-42	XP_002639655.1	80	176.407	118	95	Hypothetical protein CBG12371
Locus_17461_Transcript_1/1_Conf_1.000	203	15	3.08E-19	XP_002641899.1	79	98.2117	68	54	C. briggsae CBR-NHR-10 protein
Locus_17462_Transcript_1/1_Conf_1.000	209	20	5.78E-10	NP_497641.2	67	67.3958	68	46	CaDHerin family member (cdh-1)
Locus_17463_Transcript_1/1_Conf_1.000	134	20	7.29E-13	NP_495437.2	90	77.0258	44	40	hypothetical protein C52E12.4
Locus_17464_Transcript_1/1_Conf_1.000	290	20	1.26E-20	XP_002645050.1	79	102.834	96	76	Hypothetical protein CBG16703
Locus_17465_Transcript_1/1_Conf_1.000	199	4	7.87E-15	XP_002644812.1	72	83.5741	65	47	Hypothetical protein CBG14830
Locus_17466_Transcript_1/1_Conf_1.000	1242	20	4.24E-85	XP_002640804.1	88	319.701	197	174	C. briggsae CBR-RAL-1 protein
Locus_17467_Transcript_1/1_Conf_1.000	499	20	6.32E-25	XP_002631305.1	74	117.087	108	80	C. briggsae CBR-DNA-2 protein
Locus_17468_Transcript_1/2_Conf_1.000	367	20	1.35E-35	CAA72915.1	79	152.525	116	92	serine protease inhibitor-like protein

Locus_17468_Transcript_2/2_Conf_1.000	366	20	8.76E-35	CAA72915.1	79	149.828	116	92	serine protease inhibitor-like protein
Locus_17469_Transcript_1/1_Conf_1.000	247	0							
Locus_1747_Transcript_1/1_Conf_1.000	1175	20	1.04E-122	XP_002637936.1	80	444.506	333	267	Hypothetical protein CBG04748
Locus_17470_Transcript_1/1_Conf_1.000	192	0							
Locus_17471_Transcript_1/1_Conf_1.000	249	5	8.26E-09		66	63.5438	63	42	myoblast city protein homolog
Locus_17472_Transcript_1/1_Conf_1.000	339	0							
Locus_17473_Transcript_1/1_Conf_1.000	283	20	7.69E-34	NP_500460.1	80	146.747	93	75	hypothetical protein W02C12.1
Locus_17474_Transcript_1/1_Conf_1.000	177	0							
Locus_17475_Transcript_1/1_Conf_1.000	281	2	1.97E-10	XP_002632596.1	64	68.9366	64	41	Hypothetical protein CBG23720
Locus_17476_Transcript_1/1_Conf_1.000	361	20	1.72E-30	NP_498089.2	76	135.576	115	88	hypothetical protein Y54H5A.2
Locus_17477_Transcript_1/1_Conf_1.000	756	20	4.13E-85	NP_509008.2	80	318.546	230	186	Inositol Polyphosphate 5-Phosphatase family member (ipp-5)
Locus_17478_Transcript_1/1_Conf_1.000	149	20	1.90E-16	NP_509898.1	91	88.9669	49	45	ACOnitase family member (aco-1)
Locus_17479_Transcript_1/1_Conf_1.000	178	0							
Locus_1748_Transcript_1/1_Conf_1.000	1113	20	2.16E-05	XP_001897183.1	64	54.6842	62	40	HMG box family protein
Locus_17480_Transcript_1/1_Conf_1.000	407	3	2.28E-06	NP_001123076.1	48	55.4546	129	62	hypothetical protein ZC455.1
Locus_17481_Transcript_1/1_Conf_1.000	132	0							
Locus_17482_Transcript_1/1_Conf_1.000	260	0							
Locus_17483_Transcript_1/1_Conf_1.000	320	0							
Locus_17484_Transcript_1/1_Conf_1.000	452	6	1.48E-05	AAA74035.1	58	52.7582	70	41	CV-FLP-1A

Locus_17485_Transcript_1/1_Conf_1.000	429	20	4.67E-60	NP_493778.1	85	233.802	142	121	hypothetical protein K07E8.7
Locus_17486_Transcript_1/1_Conf_1.000	361	20	1.41E-24	NP_001033391.1	64	115.931	125	81	GLutaRedoXin family member (glrx-22)
Locus_17487_Transcript_1/1_Conf_1.000	514	20	2.90E-39	XP_002647238.1	71	164.851	171	123	Hypothetical protein CBG24582
Locus_17488_Transcript_1/1_Conf_1.000	144	0							
Locus_17489_Transcript_1/1_Conf_1.000	188	0							
Locus_1749_Transcript_1/2_Conf_1.000	1200	20	3.19E-74	XP_001894031.1	64	283.493	318	205	Protein kinase domain containing protein
Locus_1749_Transcript_2/2_Conf_1.000	1200	20	3.19E-74	XP_001894031.1	64	283.493	318	205	Protein kinase domain containing protein
Locus_17490_Transcript_1/1_Conf_1.000	664	0							
Locus_17491_Transcript_1/1_Conf_1.000	189	0							
Locus_17492_Transcript_1/1_Conf_1.000	448	20	2.60E-26	A8XZU0.2	63	121.709	147	93	Probable cytosolic Fe-S cluster assembly factor oxy-4
Locus_17493_Transcript_1/1_Conf_1.000	179	1	3.60E-04	NP_496205.2	61	48.1358	54	33	PhosphoLipase C family member (plc-3)
Locus_17494_Transcript_1/1_Conf_1.000	395	20	3.01E-22	NP_741496.2	71	108.227	97	69	hypothetical protein Y10G11A.1
Locus_17495_Transcript_1/1_Conf_1.000	396	6	4.34E-21	XP_002634643.1	61	104.375	129	79	C. briggsae CBR-GEI-9 protein
Locus_17496_Transcript_1/1_Conf_1.000	308	0							
Locus_17497_Transcript_1/1_Conf_1.000	286	20	9.43E-08	AAI24420.1	58	60.077	79	46	Dgcr8 protein
Locus_17498_Transcript_1/1_Conf_1.000	789	20	5.98E-37	NP_505460.1	67	158.688	161	109	hypothetical protein C50F4.12
Locus_17499_Transcript_1/1_Conf_1.000	427	3	1.74E-38	XP_002645893.1	76	162.155	142	108	Hypothetical protein CBG07646

Locus_175_Transcript_1/2_Conf_1.000	2404	20	4.16E-51	XP_002642145.1	41	207.994	898	370	C. briggsae CBR-EVL-14 protein
Locus_175_Transcript_2/2_Conf_1.000	2404	20	2.70E-50	XP_002642145.1	41	205.297	898	369	C. briggsae CBR-EVL-14 protein
Locus_1750_Transcript_1/1_Conf_1.000	1086	20	7.74E-77	NP_498713.3	71	291.967	271	195	hypothetical protein ZK688.9
Locus_17500_Transcript_1/1_Conf_1.000	137	0							
Locus_17501_Transcript_1/1_Conf_1.000	395	20	1.60E-23	EFO22018.1	82	112.464	74	61	hypothetical protein LOAG_06465
Locus_17502_Transcript_1/1_Conf_1.000	422	4	1.40E-11	XP_002632757.1	66	72.7886	72	48	C. briggsae CBR-EOR-1 protein
Locus_17503_Transcript_1/1_Conf_1.000	647	20	4.46E-65	XP_002646632.1	74	251.521	216	160	C. briggsae CBR-UNC-52 protein
Locus_17504_Transcript_1/1_Conf_1.000	238	0							
Locus_17505_Transcript_1/1_Conf_1.000	194	0							
Locus_17506_Transcript_1/1_Conf_1.000	306	0							
Locus_17507_Transcript_1/1_Conf_1.000	178	2	3.48E-07	NP_504750.2	81	58.151	58	47	hypothetical protein K11C4.2
Locus_17508_Transcript_1/1_Conf_1.000	236	0							
Locus_17509_Transcript_1/1_Conf_1.000	176	0							
Locus_1751_Transcript_1/1_Conf_1.000	1317	7	5.52E-22	CAB07693.3	65	110.153	119	78	C. elegans protein Y48E1B.7, partially confirmed by transcript evidence
Locus_17510_Transcript_1/1_Conf_1.000	315	4	7.93E-23	NP_505399.1	71	110.153	104	74	hypothetical protein ZK682.2
Locus_17511_Transcript_1/1_Conf_1.000	163	0							
Locus_17512_Transcript_1/1_Conf_1.000	256	20	9.10E-11	XP_001122011.1	57	70.0922	89	51	PREDICTED: similar to easter CG4920-PA
Locus_17513_Transcript_1/1_Conf_1.000	248	0							
Locus_17514_Transcript_1/1_Conf_1.000	191	0							
Locus_17515_Transcript_1/1_Conf_1.000	397	0							
Locus_17516_Transcript_1/1_Conf_1.000	250	0							
Locus_17517_Transcript_1/1_Conf_1.000	357	20	1.28E-25	XP_002630721.1	66	119.398	133	88	C. briggsae CBR-CLR-1 protein
Locus_17518_Transcript_1/1_Conf_1.000	480	0							

Locus_17519_Transcript_1/1_Conf_1.000	1301	2	6.61E-20	NP_505209.2	48	103.219	428	206	hypothetical protein F25G6.9
Locus_1752_Transcript_1/1_Conf_1.000	761	20	1.87E-93	CBW44400.1	74	346.28	280	208	C. elegans protein Y59A8B.22a, confirmed by transcript evidence
Locus_17520_Transcript_1/1_Conf_1.000	301								
Locus_17521_Transcript_1/1_Conf_1.000	394	0							
Locus_17522_Transcript_1/1_Conf_1.000	265	0							
Locus_17523_Transcript_1/1_Conf_1.000	131	0							
Locus_17524_Transcript_1/1_Conf_1.000	336	0							
Locus_17525_Transcript_1/1_Conf_1.000	518	3	1.39E-04	EEQ88268.1	42	49.6766	155	66	hypothetical protein BDCG_03388
Locus_17526_Transcript_1/1_Conf_1.000	1007	20	3.31E-63	EFO15384.1	68	246.514	240	165	hypothetical protein LOAG_13128
Locus_17527_Transcript_1/1_Conf_1.000	169	3	4.58E-15	NP_001033381.1	84	84.3445	52	44	hypothetical protein Y71H2AM.15
Locus_17528_Transcript_1/1_Conf_1.000	293	1	8.20E-04	NP_509315.1	72	46.9802	37	27	hypothetical protein ZK154.4
Locus_17529_Transcript_1/1_Conf_1.000	538	20	1.79E-16	NP_001022401.1	70	89.3521	98	69	Polarity and Osmotic sensitivity Defect family member (pod-2)
Locus_1753_Transcript_1/1_Conf_1.000	288	6	4.36E-05	XP_002433635.1	68	51.2174	45	31	protein tyrosine phosphatase, putative
Locus_17530_Transcript_1/1_Conf_1.000	563	0							
Locus_17531_Transcript_1/1_Conf_1.000	354	20	1.52E-26	XP_002631704.1	73	122.479	101	74	Hypothetical protein CBG20903
Locus_17532_Transcript_1/1_Conf_1.000	179	5	1.23E-20	EFO22429.1	93	102.834	59	55	hypothetical protein LOAG_06054
Locus_17533_Transcript_1/1_Conf_1.000	480	0							
Locus_17534_Transcript_1/1_Conf_1.000	403	3	8.71E-06	ACI49231.1	66	53.5286	50	33	hypothetical protein Csp3_JD05.016
Locus_17535_Transcript_1/1_Conf_1.000	829	20	9.66E-57	CAA91288.2	60	224.557	255	155	C. elegans protein K08F8.5b, partially confirmed by transcript evidence

Locus_17536_Transcript_1/1_Conf_1.000	266	20	1.29E-25	NP_502170.3	75	119.398	86	65	TWiK family of potassium channels family member (twk-25)
Locus_17537_Transcript_1/1_Conf_1.000	331	0							
Locus_17538_Transcript_1/1_Conf_1.000	143	0							
Locus_17539_Transcript_1/1_Conf_1.000	250	20	1.65E-41	CAV31771.1	96	172.17	83	80	C. elegans protein F25D7.3b, partially confirmed by transcript evidence
Locus_1754_Transcript_1/2_Conf_1.000	2556	20	0	NP_740776.1	87	1202.96	713	627	WW domain Protein (E3 ubiquitin ligase) family member (wwp-1)
Locus_1754_Transcript_2/2_Conf_1.000	2589	20	0	NP_740776.1	84	1228.39	800	677	WW domain Protein (E3 ubiquitin ligase) family member (wwp-1)
Locus_17540_Transcript_1/1_Conf_1.000	270	0							
Locus_17541_Transcript_1/1_Conf_1.000	427	0							
Locus_17542_Transcript_1/1_Conf_1.000	768	5	2.12E-76	CAR63603.1	95	289.656	169	161	putative somatostatin receptor
Locus_17543_Transcript_1/1_Conf_1.000	163	0							
Locus_17544_Transcript_1/1_Conf_1.000	134	20	2.77E-12	NP_741757.1	92	75.0998	42	39	Endophilin-Related Protein family member (erp-1)
Locus_17545_Transcript_1/1_Conf_1.000	324	0							
Locus_17546_Transcript_1/1_Conf_1.000	392	20	1.55E-23	XP_002637289.1	75	112.464	89	67	Hypothetical protein CBG18976
Locus_17547_Transcript_1/1_Conf_1.000	165	0							
Locus_17548_Transcript_1/1_Conf_1.000	308	20	1.96E-34	NP_491390.2	85	148.673	100	85	hypothetical protein D1007.15

Locus_17549_Transcript_1/1_Conf_1.000	493	20	1.55E-71	XP_002642701.1	92	271.937	162	150	C. briggsae CBR-GLY-3 protein
Locus_1755_Transcript_1/1_Conf_1.000	1189	20	4.53E-81	NP_495808.2	85	306.22	200	171	STCH (truncated HSP) family member (stc-1)
Locus_17550_Transcript_1/1_Conf_1.000	630	6	1.45E-49	NP_001021494.1	69	199.904	196	136	hypothetical protein F55A12.2
Locus_17551_Transcript_1/1_Conf_1.000	228	0							
Locus_17552_Transcript_1/1_Conf_1.000	241	15	2.19E-17	NP_001122650.1	69	92.0485	79	55	PaTChed family member (ptc-3)
Locus_17553_Transcript_1/1_Conf_1.000	432	20	3.05E-27	NP_001041274.2	69	124.79	134	93	hypothetical protein K11G12.6
Locus_17554_Transcript_1/1_Conf_1.000	184	20	2.83E-28	ABW22635.1	100	128.257	61	61	GABA receptor subunit
Locus_17555_Transcript_1/1_Conf_1.000	155	0							
Locus_17556_Transcript_1/1_Conf_1.000	308	0							
Locus_17557_Transcript_1/1_Conf_1.000	193	0							
Locus_17558_Transcript_1/1_Conf_1.000	371	0							
Locus_17559_Transcript_1/1_Conf_1.000	345	4	6.94E-11	NP_001122607.1	65	70.4774	79	52	NURF (nucleosome remodeling factor) complex homolog family member (nurf-1)
Locus_1756_Transcript_1/1_Conf_1.000	1250	20	9.00E-160	NP_493742.1	78	567.77	447	350	NucleOLar protein family member (nol-1)

Locus_17560_Transcript_1/1_Conf_1.000	267	2	3.77E-09	NP_491354.1	61	64.6994	97	60	AcetylCholine Receptor family member (acr-6)
Locus_17561_Transcript_1/1_Conf_1.000	414	13	1.27E-25	CAA21686.2	67	119.398	135	91	C. elegans protein Y54E2A.4, partially confirmed by transcript evidence
Locus_17562_Transcript_1/1_Conf_1.000	173	0							
Locus_17563_Transcript_1/1_Conf_1.000	156	0							
Locus_17564_Transcript_1/1_Conf_1.000	157	2	6.71E-06	NP_498385.2	64	53.9138	51	33	hypothetical protein C56G2.4
Locus_17565_Transcript_1/1_Conf_1.000	444	20	2.87E-49	XP_002643809.1	78	197.978	147	115	Hypothetical protein CBG02021
Locus_17566_Transcript_1/1_Conf_1.000	220	5	3.03E-14	NP_510542.2	72	81.6481	66	48	Temporarily Assigned Gene name family member (tag-97)
Locus_17567_Transcript_1/1_Conf_1.000	291	5	3.56E-07	XP_001895488.1	59	58.151	92	55	hypothetical protein
Locus_17568_Transcript_1/1_Conf_1.000	584	0							
Locus_17569_Transcript_1/1_Conf_1.000	326	0							
Locus_1757_Transcript_1/6_Conf_0.167	656	20	1.19E-36	AAV28323.2	58	157.147	173	102	Hypothetical protein T15B7.1
Locus_1757_Transcript_2/6_Conf_0.500	2537	20	0	NP_497786.2	91	1217.99	729	670	MethylMalonylCoA Mutase homolog family member (mmcm-1)
Locus_1757_Transcript_3/6_Conf_0.583	2181	20	0	XP_002647077.1	92	905.205	521	480	C. briggsae CBR-MMCM-1 protein
Locus_1757_Transcript_4/6_Conf_0.417	2523	20	0	NP_497786.2	91	1223.38	748	682	MethylMalonylCoA Mutase homolog family member (mmcm-1)
Locus_1757_Transcript_5/6_Conf_0.583	3076	20	0	NP_497786.2	91	1215.68	729	669	MethylMalonylCoA Mutase homolog family member (mmcm-1)
Locus_1757_Transcript_6/6_Conf_0.583	3076	20	0	NP_497786.2	91	1217.99	729	670	MethylMalonylCoA Mutase homolog family member (mmcm-1)

Locus_17570_Transcript_1/1_Conf_1.000	496	20	1.66E-41	NP_741237.1	89	172.17	105	94	CaLPain family member (clp-1)
Locus_17571_Transcript_1/1_Conf_1.000	212	0							
Locus_17572_Transcript_1/1_Conf_1.000	420	20	2.15E-20	XP_002640915.1	69	102.064	103	72	Hypothetical protein CBG00476
Locus_17573_Transcript_1/1_Conf_1.000	243	0							
Locus_17574_Transcript_1/1_Conf_1.000	165	20	6.86E-11	NP_510625.2	80	70.4774	50	40	hypothetical protein T27A8.1
Locus_17575_Transcript_1/1_Conf_1.000	214	0							
Locus_17576_Transcript_1/1_Conf_1.000	149	0							
Locus_17577_Transcript_1/1_Conf_1.000	212	0							
Locus_17578_Transcript_1/1_Conf_1.000	543	0							
Locus_17579_Transcript_1/1_Conf_1.000	313	0							
Locus_1758_Transcript_1/1_Conf_1.000	616	20	1.25E-50	EFO16392.1	86	203.371	123	107	VCP protein
Locus_17580_Transcript_1/1_Conf_1.000	197	20	2.81E-12	EFO28029.1	95	75.0998	43	41	AP-1 complex subunit mu-1
Locus_17581_Transcript_1/1_Conf_1.000	169	20	1.85E-24	ABS45067.1	96	115.546	56	54	latrophilin-like protein 2
Locus_17582_Transcript_1/1_Conf_1.000	171	3	1.73E-14	NP_498952.1	76	82.4185	56	43	hypothetical protein F44E2.4

Locus_17583_Transcript_1/1_Conf_1.000	191	20	8.74E-30	ADK36640.1	100	133.265	63	63	L-lactate dehydrogenase B chain
Locus_17584_Transcript_1/1_Conf_1.000	429	20	2.66E-47	CAQ48388.2	86	191.43	143	123	C. elegans protein C26H9A.1b, confirmed by transcript evidence
Locus_17585_Transcript_1/1_Conf_1.000	196	0							
Locus_17586_Transcript_1/1_Conf_1.000	281	20	3.84E-14	NP_494219.3	59	81.2629	114	68	hypothetical protein F54A3.2
Locus_17587_Transcript_1/1_Conf_1.000	933	20	1.61E-69	XP_002631319.1	64	267.314	328	213	Hypothetical protein CBG03141
Locus_17588_Transcript_1/1_Conf_1.000	1091	20	8.47E-124	XP_002632173.1	88	447.973	296	262	Hypothetical protein CBG07032
Locus_17589_Transcript_1/1_Conf_1.000	292	0							
Locus_1759_Transcript_1/3_Conf_0.400	362	20	8.27E-33	NP_498590.4	87	143.28	105	92	hypothetical protein R151.6
Locus_1759_Transcript_2/3_Conf_0.600	573	20	1.27E-32	NP_498590.4	87	143.28	105	92	hypothetical protein R151.6
Locus_1759_Transcript_3/3_Conf_0.600	802	20	2.68E-32	NP_498590.4	87	143.28	105	92	hypothetical protein R151.6
Locus_17590_Transcript_1/1_Conf_1.000	184	0							
Locus_17591_Transcript_1/1_Conf_1.000	316	0							

Locus_17592_Transcript_1/1_Conf_1.000	137	0							
Locus_17593_Transcript_1/1_Conf_1.000	353	0							
Locus_17594_Transcript_1/1_Conf_1.000	255	0							
Locus_17595_Transcript_1/1_Conf_1.000	147	0							
Locus_17596_Transcript_1/1_Conf_1.000	156	0							
Locus_17597_Transcript_1/1_Conf_1.000	325	20	3.42E-10	XP_001636049.1	64	68.1662	98	63	predicted protein
Locus_17598_Transcript_1/1_Conf_1.000	142	20	5.35E-08	YP_001328864.1	80	60.8474	46	37	tRNA modification GTPase TrmE
Locus_17599_Transcript_1/1_Conf_1.000	132	20	2.12E-12	XP_002641552.1	86	75.485	44	38	C. briggsae CBR-RBC-2 protein
Locus_176_Transcript_1/1_Conf_1.000	1563	20	1.49E-141	XP_002642495.1	75	507.679	460	345	C. briggsae CBR-MIG-22 protein
Locus_1760_Transcript_1/1_Conf_1.000	1196	20	2.70E-49	EFO26991.1	60	200.675	261	159	hypothetical protein LOAG_01487
Locus_17600_Transcript_1/1_Conf_1.000	151	0							
Locus_17601_Transcript_1/1_Conf_1.000	267	2	3.40E-18	NP_492624.2	82	94.7449	69	57	hypothetical protein F52F12.7
Locus_17602_Transcript_1/1_Conf_1.000	245	2	1.79E-11	XP_002641411.1	65	72.4034	83	54	Hypothetical protein CBG13276
Locus_17603_Transcript_1/1_Conf_1.000	990	20	3.49E-25	NP_508831.3	56	120.168	168	95	hypothetical protein T07F12.2
Locus_17604_Transcript_1/1_Conf_1.000	212	0							
Locus_17605_Transcript_1/1_Conf_1.000	222	0							

Locus_17606_Transcript_1/1_Conf_1.000	150	6	4.37E-13	NP_001033327.1	84	77.7962	45	38	CAN cell Migration defective family member (cam-1)
Locus_17607_Transcript_1/1_Conf_1.000	489	20	4.61E-07	NP_492328.1	51	57.7658	122	63	yeast Enhancer of DeCapping homolog family member (edc-3)
Locus_17608_Transcript_1/1_Conf_1.000	400	20	1.47E-61	NP_506145.1	94	238.81	131	124	hypothetical protein E02A10.1
Locus_17609_Transcript_1/1_Conf_1.000	245	0							
Locus_1761_Transcript_1/1_Conf_1.000	781	0							
Locus_17610_Transcript_1/1_Conf_1.000	292	0							
Locus_17611_Transcript_1/1_Conf_1.000	286	0							
Locus_17612_Transcript_1/1_Conf_1.000	558								
Locus_17613_Transcript_1/1_Conf_1.000	340	20	2.74E-20	CAA91288.2	62	101.679	101	63	C. elegans protein K08F8.5b, partially confirmed by transcript evidence
Locus_17614_Transcript_1/1_Conf_1.000	265	1	6.24E-12	CAR63599.1	69	73.9442	65	45	hypothetical protein
Locus_17615_Transcript_1/1_Conf_1.000	132	0							
Locus_17616_Transcript_1/1_Conf_1.000	178	0							
Locus_17617_Transcript_1/1_Conf_1.000	278	0							
Locus_17618_Transcript_1/1_Conf_1.000	184	0							
Locus_17619_Transcript_1/1_Conf_1.000	467	1	8.46E-09	XP_002635121.1	42	63.5438	173	74	Hypothetical protein CBG11345
Locus_1762_Transcript_1/4_Conf_0.700	1967	1	2.36E-09	XP_002640249.1	35	68.9366	403	145	C. briggsae CBR-PQN-20 protein
Locus_1762_Transcript_2/4_Conf_0.700	1943	1	2.33E-09	XP_002640249.1	35	68.9366	403	145	C. briggsae CBR-PQN-20 protein
Locus_1762_Transcript_3/4_Conf_0.700	1943	1	2.33E-09	XP_002640249.1	35	68.9366	403	145	C. briggsae CBR-PQN-20 protein
Locus_1762_Transcript_4/4_Conf_0.700	1943	1	2.33E-09	XP_002640249.1	35	68.9366	403	145	C. briggsae CBR-PQN-20 protein
Locus_17620_Transcript_1/1_Conf_1.000	528	20	7.43E-52	AAN61521.1	70	206.838	175	124	301KDa_2 protein

Locus_17621_Transcript_1/1_Conf_1.000	140	20	8.83E-19	XP_002832350.1	100	96.6709	46	46	PREDICTED: LOW QUALITY PROTEIN: filamin-A-like
Locus_17622_Transcript_1/1_Conf_1.000	336	20	2.92E-38	NP_494753.3	87	161.384	97	85	hypothetical protein C01F1.6
Locus_17623_Transcript_1/1_Conf_1.000	216	0							
Locus_17624_Transcript_1/1_Conf_1.000	415	20	8.78E-35	AAN11401.1	71	149.828	127	91	metalloprotease 1 precursor
Locus_17625_Transcript_1/1_Conf_1.000	474	20	2.41E-32	XP_002634336.1	64	141.739	154	99	C. briggsae CBR-KLP-10 protein
Locus_17626_Transcript_1/1_Conf_1.000	168	2	4.01E-11	XP_002636973.1	72	71.2478	55	40	Hypothetical protein CBG09454

Locus_17627_Transcript_1/1_Conf_1.000	298	20	2.06E-31	EFO21606.1	81	138.658	99	81	PDZ-domain-containing protein scribble
Locus_17628_Transcript_1/1_Conf_1.000	345	0							
Locus_17629_Transcript_1/1_Conf_1.000	153	0							
Locus_1763_Transcript_1/1_Conf_1.000	844	4	1.37E-21	NP_508166.1	49	107.842	259	129	ImmunoGlobulin-like Cell adhesion Molecule family member (igcm-1)
Locus_17630_Transcript_1/1_Conf_1.000	400	20	1.21E-23	XP_002637712.1	63	112.849	133	84	C. briggsae CBR-RAP-2 protein
Locus_17631_Transcript_1/1_Conf_1.000	154	0							
Locus_17632_Transcript_1/1_Conf_1.000	301	20	7.06E-24	NP_001122600.1	84	113.62	76	64	Multiple PDZ domain protein family member (mpz-1)
Locus_17633_Transcript_1/1_Conf_1.000	655	0							
Locus_17634_Transcript_1/1_Conf_1.000	241	20	4.42E-18	NP_499704.1	73	94.3597	80	59	CYtochrome P450 family member (cyp-13A11)
Locus_17635_Transcript_1/1_Conf_1.000	262	0							

Locus_17636_Transcript_1/1_Conf_1.000	275	20	4.86E-33	NP_492476.1	85	144.05	91	78	hypothetical protein F14B4.3
Locus_17637_Transcript_1/1_Conf_1.000	141	0							
Locus_17638_Transcript_1/1_Conf_1.000	150	0							
Locus_17639_Transcript_1/1_Conf_1.000	454	4	2.59E-18	EFO26050.1	59	95.1301	131	78	PEK/PEK protein kinase
Locus_1764_Transcript_1/1_Conf_1.000	1018	20	3.49E-60	XP_002642911.1	66	236.498	317	211	Hypothetical protein CBG15186
Locus_17640_Transcript_1/1_Conf_1.000	189	20	2.46E-24	XP_001896329.1	96	115.161	62	60	calcium ATPase
Locus_17641_Transcript_1/1_Conf_1.000	172	20	2.27E-14	P00736.2	100	82.0333	36	36	Complement C1r subcomponent
Locus_17642_Transcript_1/1_Conf_1.000	681	6	1.26E-15	CAN99691.2	49	87.4261	206	101	C. elegans protein F54B3.1b, partially confirmed by transcript evidence
Locus_17643_Transcript_1/1_Conf_1.000	193	2	7.18E-08	NP_495415.2	72	60.4622	44	32	Acid SphingoMyelinase family member (asm-1)
Locus_17644_Transcript_1/1_Conf_1.000	324	20	4.44E-26	XP_002639976.1	73	120.939	109	80	C. briggsae CBR-KIN-32 protein
Locus_17645_Transcript_1/1_Conf_1.000	150	3	3.02E-14	CAA86821.1	87	81.6481	49	43	excretory/secretory antigen
Locus_17646_Transcript_1/1_Conf_1.000	129	8	8.63E-14	NP_001020988.1	93	80.1073	43	40	UNCoordinated family member (unc-89)
Locus_17647_Transcript_1/1_Conf_1.000	163	4	2.71E-07	XP_002640991.1	84	58.5362	53	45	Hypothetical protein CBG11740
Locus_17648_Transcript_1/1_Conf_1.000	153	0							
Locus_17649_Transcript_1/1_Conf_1.000	229	0							
Locus_1765_Transcript_1/1_Conf_1.000	375	20	2.89E-17	NP_001094784.1	100	91.6633	65	65	transmembrane protein 176B isoform b
Locus_17650_Transcript_1/1_Conf_1.000	239	0							
Locus_17651_Transcript_1/1_Conf_1.000	423	7	6.24E-12	EFO21962.1	63	73.9442	91	58	TK/EGFR protein kinase

Locus_17652_Transcript_1/1_Conf_1.000	159	20	1.14E-21	XP_001118006.2	100	106.301	52	52	PREDICTED: elongation factor 2
Locus_17653_Transcript_1/1_Conf_1.000	520	3	1.19E-51	NP_501400.1	90	206.068	174	158	hypothetical protein F35H10.10
Locus_17654_Transcript_1/1_Conf_1.000	131	0							
Locus_17655_Transcript_1/1_Conf_1.000	391	0							
Locus_17656_Transcript_1/1_Conf_1.000	373	20	3.65E-36	XP_002634229.1	77	154.451	123	95	C. briggsae CBR-UNC-26 protein
Locus_17657_Transcript_1/1_Conf_1.000	720	20	1.25E-88	XP_002636978.1	88	330.102	238	211	Hypothetical protein CBG09461
Locus_17658_Transcript_1/1_Conf_1.000	361	0							
Locus_17659_Transcript_1/1_Conf_1.000	830	20	1.30E-61	XP_002631397.1	70	240.736	249	176	C. briggsae CBR-DEP-1 protein
Locus_1766_Transcript_1/1_Conf_1.000	435	2	1.84E-24	NP_499357.2	73	115.546	94	69	hypothetical protein Y39A1A.20
Locus_17660_Transcript_1/1_Conf_1.000	427	0							
Locus_17661_Transcript_1/1_Conf_1.000	140	0							
Locus_17662_Transcript_1/1_Conf_1.000	220	0							
Locus_17663_Transcript_1/1_Conf_1.000	321	0							
Locus_17664_Transcript_1/1_Conf_1.000	778	20	1.32E-73	XP_002647977.1	69	280.411	265	183	Hypothetical protein CBG23894
Locus_17665_Transcript_1/1_Conf_1.000	596	20	2.85E-86	XP_002641537.1	92	321.627	182	169	Hypothetical protein CBG09836
Locus_17666_Transcript_1/1_Conf_1.000	1766	1	3.42E-04	YP_003834.1	57	51.6026	88	51	unknown
Locus_17667_Transcript_1/1_Conf_1.000	191	1	8.78E-14	NP_494276.2	78	80.1073	64	50	Variable ABnormal morphology family member (vab-19)

Locus_17668_Transcript_1/1_Conf_1.000	293	11	8.43E-25	EFO27980.1	76	116.701	97	74	hypothetical protein LOAG_00505
Locus_17669_Transcript_1/1_Conf_1.000	209	20	2.12E-20	XP_002630579.1	91	102.064	69	63	C. briggsae CBR-NHX-2 protein
Locus_1767_Transcript_1/2_Conf_1.000	1257	20	1.88E-40	XP_001366252.1	64	171.4	207	134	PREDICTED: similar to Ubiquitin specific peptidase 3
Locus_1767_Transcript_2/2_Conf_1.000	1018	20	8.62E-51	XP_002110506.1	62	205.297	258	161	hypothetical protein TRIADDRAFT_22897
Locus_17670_Transcript_1/1_Conf_1.000	214	0							
Locus_17671_Transcript_1/1_Conf_1.000	260	0							
Locus_17672_Transcript_1/2_Conf_1.000	565	20	9.83E-51	EFO22315.1	75	203.371	176	133	hypothetical protein LOAG_06169
Locus_17672_Transcript_2/2_Conf_1.000	402	20	1.99E-34	XP_002639921.1	71	148.673	131	94	Hypothetical protein CBG08253
Locus_17673_Transcript_1/1_Conf_1.000	438	0							
Locus_17674_Transcript_1/1_Conf_1.000	333	20	3.24E-45	EFO17249.1	92	184.496	106	98	RNA polymerase II

Locus_17675_Transcript_1/1_Conf_1.000	604	20	8.31E-113	XP_002639744.1	91	409.838	199	183	C. briggsae CBR-LAM-3 protein
Locus_17676_Transcript_1/1_Conf_1.000	742	20	5.57E-103	NP_491257.1	88	377.867	241	214	FK506-Binding protein family member (fkb-5)
Locus_17677_Transcript_1/1_Conf_1.000	177	20	1.19E-23	AAC27893.1	93	112.849	58	54	ornithine decarboxylase
Locus_17678_Transcript_1/1_Conf_1.000	170	20	3.74E-25	XP_002922306.1	100	117.857	56	56	PREDICTED: triosephosphate isomerase-like, partial
Locus_17679_Transcript_1/1_Conf_1.000	461	20	1.19E-15	XP_869306.2	73	86.2705	76	56	PREDICTED: hypothetical protein isoform 1
Locus_1768_Transcript_10/12_Conf_0.588	1767	20	3.07E-154	XP_001897032.1	99	550.051	288	286	polyubiquitin precursor
Locus_1768_Transcript_11/12_Conf_0.559	1767	20	3.07E-154	XP_001897032.1	99	550.051	288	286	polyubiquitin precursor
Locus_1768_Transcript_12/12_Conf_0.588	1767	20	3.07E-154	XP_001897032.1	99	550.051	288	286	polyubiquitin precursor

Locus_1768_Transcript_3/12_Conf_0.353	957	20	1.29E-154	XP_001897032.1	99	550.051	288	286	polyubiquitin precursor
Locus_1768_Transcript_4/12_Conf_0.324	915	20	3.86E-153	XP_001897032.1	99	545.043	285	283	polyubiquitin precursor
Locus_1768_Transcript_5/12_Conf_0.471	1298	20	3.73E-140	XP_001897032.1	99	502.671	264	262	polyubiquitin precursor
Locus_1768_Transcript_6/12_Conf_0.500	1343	20	2.14E-154	XP_001897032.1	99	550.051	288	286	polyubiquitin precursor
Locus_1768_Transcript_7/12_Conf_0.500	1343	20	3.92E-140	XP_001897032.1	99	502.671	264	262	polyubiquitin precursor
Locus_1768_Transcript_8/12_Conf_0.235	818	20	2.50E-134	XP_001897032.1	99	482.256	253	251	polyubiquitin precursor
Locus_1768_Transcript_9/12_Conf_0.088	349	20	1.29E-57	EFO21508.1	100	225.713	116	116	ubiquitin C II
Locus_17680_Transcript_1/1_Conf_1.000	298	20	2.06E-23	XP_002633309.1	76	112.079	98	75	Hypothetical protein CBG06044
Locus_17681_Transcript_1/1_Conf_1.000	241	0							

Locus_17682_Transcript_1/1_Conf_1.000	371	20	7.20E-08	XP_002818294.1	98	60.4622	75	74	PREDICTED: collagen alpha-2(I) chain-like
Locus_17683_Transcript_1/1_Conf_1.000	887	4	2.13E-36	XP_001901821.1	52	157.147	310	163	hypothetical protein Bm1_51765
Locus_17684_Transcript_1/2_Conf_1.000	526	2	9.18E-55	XP_002633375.1	71	216.468	172	123	Hypothetical protein CBG06123
Locus_17684_Transcript_2/2_Conf_1.000	357	2	1.46E-37	XP_002633375.1	74	159.073	116	86	Hypothetical protein CBG06123
Locus_17685_Transcript_1/1_Conf_1.000	214	20	1.41E-24	XP_002631304.1	91	115.931	71	65	Hypothetical protein CBG03118
Locus_17686_Transcript_1/1_Conf_1.000	615	0							
Locus_17687_Transcript_1/1_Conf_1.000	249	1	1.19E-15	AAT07449.1	78	86.2705	61	48	putative protein
Locus_17688_Transcript_1/1_Conf_1.000	396	0							
Locus_17689_Transcript_1/1_Conf_1.000	313	20	3.45E-26	XP_002637058.1	74	121.324	104	77	C. briggsae CBR-TTR-19 protein
Locus_1769_Transcript_1/2_Conf_1.000	231	0							
Locus_1769_Transcript_2/2_Conf_1.000	257	0							
Locus_17690_Transcript_1/1_Conf_1.000	223	0							
Locus_17691_Transcript_1/2_Conf_1.000	249	0							
Locus_17691_Transcript_2/2_Conf_1.000	250	0							
Locus_17692_Transcript_1/1_Conf_1.000	550	20	2.19E-44	XP_002637648.1	64	182.185	182	118	C. briggsae CBR-FSHR-1 protein
Locus_17693_Transcript_1/1_Conf_1.000	156	0							
Locus_17694_Transcript_1/1_Conf_1.000	832	20	8.14E-96	XP_002634173.1	79	354.369	275	219	C. briggsae CBR-HOE-1 protein
Locus_17695_Transcript_1/1_Conf_1.000	853	20	1.40E-130	NP_497814.3	88	469.929	281	250	UNCoordinated family member (unc-79)
Locus_17696_Transcript_1/1_Conf_1.000	291	2	4.06E-11	EFO26621.1	51	71.2478	96	49	AIDA-1b
Locus_17697_Transcript_1/1_Conf_1.000	159	20	3.55E-07	XP_002414302.1	82	58.151	40	33	multidrug resistance protein, putative
Locus_17698_Transcript_1/1_Conf_1.000	137	0							

Locus_17699_Transcript_1/1_Conf_1.000	965	20	3.60E-96	NP_001021501.1	77	355.91	292	227	UNCoordinated family member (unc-73)
Locus_177_Transcript_1/1_Conf_1.000	1587	20	4.84E-180	NP_506706.1	80	635.565	490	394	yeast MCM (licensing factor) related family member (mcm-3)
Locus_1770_Transcript_1/1_Conf_1.000	3320	20	0	NP_494782.1	64	870.537	1102	716	hypothetical protein ZK430.1
Locus_17700_Transcript_1/1_Conf_1.000	189	0							
Locus_17701_Transcript_1/1_Conf_1.000	189	20	2.38E-27	XP_002638537.1	96	125.176	62	60	Hypothetical protein CBG05572
Locus_17702_Transcript_1/1_Conf_1.000	408	1	2.51E-05	XP_002639683.1	71	51.9878	38	27	C. briggsae CBR-GEI-11 protein
Locus_17703_Transcript_1/1_Conf_1.000	329	0							
Locus_17704_Transcript_1/1_Conf_1.000	377	9	6.20E-36	XP_002638473.1	80	153.68	124	100	Hypothetical protein CBG12901
Locus_17705_Transcript_1/1_Conf_1.000	562	3	6.32E-18	NP_510840.2	74	94.3597	79	59	hypothetical protein F31A3.5
Locus_17706_Transcript_1/1_Conf_1.000	237	0							
Locus_17707_Transcript_1/1_Conf_1.000	426	0							
Locus_17708_Transcript_1/1_Conf_1.000	172	20	1.16E-26	XP_002834627.1	100	122.865	57	57	PREDICTED: collagen alpha-2(VI) chain-like, partial
Locus_17709_Transcript_1/1_Conf_1.000	661	20	1.06E-101	XP_002647328.1	91	373.244	219	200	C. briggsae CBR-EGL-8 protein

Locus_1771_Transcript_1/1_Conf_1.000	329	0							
Locus_17710_Transcript_1/2_Conf_1.000	356	20	1.58E-15	ADD13547.1	77	85.8853	67	52	Inositol triphosphate receptor protein 1, isoform h
Locus_17710_Transcript_2/2_Conf_1.000	250	0							
Locus_17711_Transcript_1/1_Conf_1.000	151	3	3.83E-09	XP_002637315.1	84	64.6994	50	42	Hypothetical protein CBG19004
Locus_17712_Transcript_1/2_Conf_1.000	553	2	1.57E-05	XP_002642641.1	46	53.1434	158	74	C. briggsae CBR-LIN-13 protein
Locus_17712_Transcript_2/2_Conf_1.000	547	2	1.51E-05	XP_002642641.1	46	53.1434	158	74	C. briggsae CBR-LIN-13 protein
Locus_17713_Transcript_1/1_Conf_1.000	204	20	5.41E-16	ZP_03131362.1	91	87.4261	46	42	protein of unknown function DUF255
Locus_17714_Transcript_1/1_Conf_1.000	771	20	1.25E-100	XP_002631092.1	83	370.163	253	210	Hypothetical protein CBG02865
Locus_17715_Transcript_1/1_Conf_1.000	223	0							
Locus_17716_Transcript_1/1_Conf_1.000	510	20	1.54E-77	XP_001897606.1	89	291.967	169	151	Adaptin or adaptin-related protein protein 7
Locus_17717_Transcript_1/1_Conf_1.000	200	0							

Locus_17718_Transcript_1/1_Conf_1.000	159	0							
Locus_17719_Transcript_1/1_Conf_1.000	269	16	2.88E-17	XP_002634836.1	65	91.6633	89	58	C. briggsae CBR-JTR-1 protein
Locus_1772_Transcript_1/1_Conf_1.000	1975	20	1.59E-138	XP_002643974.1	71	498.049	481	344	Hypothetical protein CBG17350
Locus_17720_Transcript_1/1_Conf_1.000	246	20	5.00E-30	EFO23722.1	92	134.035	81	75	hypothetical protein LOAG_04763
Locus_17721_Transcript_1/1_Conf_1.000	442	20	2.20E-65	XP_002643286.1	84	251.521	146	124	Hypothetical protein CBG08162
Locus_17722_Transcript_1/1_Conf_1.000	319	0							
Locus_17723_Transcript_1/1_Conf_1.000	186	0							
Locus_17724_Transcript_1/1_Conf_1.000	234	20	2.68E-39	ACZ64268.1	93	164.851	77	72	astacin metalloprotease a
Locus_17725_Transcript_1/1_Conf_1.000	280	0							
Locus_17726_Transcript_1/1_Conf_1.000	423	4	4.92E-25	NP_501044.1	81	117.472	69	56	hypothetical protein C17H12.11
Locus_17727_Transcript_1/1_Conf_1.000	167	0							
Locus_17728_Transcript_1/1_Conf_1.000	1300	5	6.73E-73	XP_001901141.1	57	279.256	375	216	hypothetical protein Bm1_48395
Locus_17729_Transcript_1/1_Conf_1.000	492	20	1.20E-23	AAK31527.3	67	112.849	139	94	Neuronal symmetry protein 1, partially confirmed by transcript evidence
Locus_1773_Transcript_1/1_Conf_1.000	771	20	5.51E-40	NP_001122441.1	60	168.703	230	138	hypothetical protein F02E9.10
Locus_17730_Transcript_1/1_Conf_1.000	540	20	2.16E-17	XP_002631113.1	55	92.4337	158	88	C. briggsae CBR-ARS-1 protein
Locus_17731_Transcript_1/1_Conf_1.000	330	5	1.86E-16	NP_001122928.1	90	88.9669	54	49	hypothetical protein F23H12.3
Locus_17732_Transcript_1/1_Conf_1.000	153	0							
Locus_17733_Transcript_1/1_Conf_1.000	216	0							
Locus_17734_Transcript_1/1_Conf_1.000	272	20	4.01E-19	XP_001897385.1	70	97.8265	91	64	hypothetical protein Bm1_29715
Locus_17735_Transcript_1/1_Conf_1.000	160	1	4.79E-04	XP_001893275.1	70	47.7506	48	34	LMBR1-like conserved region family protein
Locus_17736_Transcript_1/1_Conf_1.000	258	0							
Locus_17737_Transcript_1/1_Conf_1.000	359	0							
Locus_17738_Transcript_1/1_Conf_1.000	374	0							
Locus_17739_Transcript_1/1_Conf_1.000	145	0							
Locus_1774_Transcript_1/1_Conf_1.000	988	20	3.94E-45	ADN00783.1	100	186.422	96	96	collagen protein

Locus_17740_Transcript_1/1_Conf_1.000	506	20	3.40E-29	NP_492763.1	62	131.339	136	85	hypothetical protein F25H2.7
Locus_17741_Transcript_1/1_Conf_1.000	447	20	3.09E-11	XP_002600120.1	54	71.633	147	80	hypothetical protein BRAFLDRAFT_118238
Locus_17742_Transcript_1/1_Conf_1.000	194	0							
Locus_17743_Transcript_1/1_Conf_1.000	254	20	4.37E-21	NP_001129835.1	96	104.375	78	75	Novel Channel type/putative Nematode CAlcium channel family member (nca-2)
Locus_17744_Transcript_1/1_Conf_1.000	234	0							
Locus_17745_Transcript_1/1_Conf_1.000	150	0							
Locus_17746_Transcript_1/1_Conf_1.000	381								
Locus_17747_Transcript_1/1_Conf_1.000	256	20	1.83E-19	NP_001024053.1	86	98.9821	60	52	UNCoordinated family member (unc-70)
Locus_17748_Transcript_1/1_Conf_1.000	310	20	4.54E-07	YP_001701724.1	51	57.7658	101	52	hypothetical protein MAB_0978
Locus_17749_Transcript_1/1_Conf_1.000	220	0							
Locus_1775_Transcript_1/1_Conf_1.000	1334	20	1.49E-115	XP_002641312.1	85	421.009	288	245	C. briggsae CBR-UNC-50 protein
Locus_17750_Transcript_1/1_Conf_1.000	343	0							
Locus_17751_Transcript_1/1_Conf_1.000	180	0							

Locus_17752_Transcript_1/1_Conf_1.000	331	20	1.02E-14	AAC28140.1	59	59.6918	69	41	mariner transposase
Locus_17753_Transcript_1/1_Conf_1.000	307	2	6.59E-06	NP_499757.2	71	53.9138	38	27	hypothetical protein T03F6.4
Locus_17754_Transcript_1/1_Conf_1.000	194	0							
Locus_17755_Transcript_1/1_Conf_1.000	301	0							
Locus_17756_Transcript_1/1_Conf_1.000	219	2	5.16E-22	NP_001024385.1	85	107.457	68	58	hypothetical protein C09F12.3
Locus_17757_Transcript_1/1_Conf_1.000	576	0							
Locus_17758_Transcript_1/1_Conf_1.000	369	20	3.56E-23	XP_002634520.1	66	111.309	99	66	Hypothetical protein CBG08313
Locus_17759_Transcript_1/1_Conf_1.000	270	0							
Locus_1776_Transcript_1/1_Conf_1.000	532	1	5.89E-04	NP_999656.1	61	47.7506	65	40	kinesin-like protein KIF15
Locus_17760_Transcript_1/1_Conf_1.000	449	5	7.12E-08	ABB53347.1	52	60.4622	97	51	secreted protein 5 precursor
Locus_17761_Transcript_1/1_Conf_1.000	249	0							
Locus_17762_Transcript_1/1_Conf_1.000	402	20	3.99E-51	XP_002914026.1	100	204.142	117	117	PREDICTED: polyadenylate-binding protein 1-like
Locus_17763_Transcript_1/1_Conf_1.000	287	0							
Locus_17764_Transcript_1/1_Conf_1.000	441	20	2.53E-37	NP_505424.2	82	158.303	102	84	TransThyretin-Related family domain family member (ttr-40)
Locus_17765_Transcript_1/1_Conf_1.000	239	2	8.67E-06	ADM53755.1	55	53.5286	74	41	Hypothetical protein ZK1127.13

Locus_17766_Transcript_1/1_Conf_1.000	550	20	2.50E-48	ACD47109.1	93	195.282	126	118	profilin
Locus_17767_Transcript_1/2_Conf_1.000	415	20	7.41E-42	ACK57928.1	78	173.326	133	105	microsomal aminopeptidase H11
Locus_17767_Transcript_2/2_Conf_1.000	546	20	1.81E-59	AAV68383.1	81	232.261	181	148	antigen h11
Locus_17768_Transcript_1/1_Conf_1.000	307	20	4.36E-50	XP_002825166.1	100	200.675	100	100	PREDICTED: cytoplasmic dynein 1 heavy chain 1-like, partial
Locus_17769_Transcript_1/1_Conf_1.000	175	0							
Locus_1777_Transcript_1/2_Conf_1.000	1187	20	2.61E-121	NP_497799.1	94	439.884	242	228	MuSashI (fly neural) family member (msi-1)
Locus_1777_Transcript_2/2_Conf_1.000	1188	20	2.62E-121	NP_497799.1	94	439.884	242	228	MuSashI (fly neural) family member (msi-1)
Locus_17770_Transcript_1/1_Conf_1.000	141	0							
Locus_17771_Transcript_1/1_Conf_1.000	712	0							
Locus_17772_Transcript_1/1_Conf_1.000	633	3	2.99E-10	XP_002637670.1	65	69.3218	67	44	C. briggsae CBR-RDE-1 protein
Locus_17773_Transcript_1/1_Conf_1.000	1214	4	1.10E-106	NP_501362.2	76	391.349	357	272	hypothetical protein F42G8.5

Locus_17774_Transcript_1/1_Conf_1.000	213	3	1.56E-07	NP_491050.3	56	59.3066	74	42	InTestinal Neurexin-like family member (itx-1)
Locus_17775_Transcript_1/1_Conf_1.000	159	0							
Locus_17776_Transcript_1/1_Conf_1.000	188	20	8.21E-28	NP_496814.1	98	126.716	62	61	Member of AAA family binding CED-4 family member (mac-1)
Locus_17777_Transcript_1/1_Conf_1.000	621	20	3.77E-63	XP_002644784.1	73	244.973	194	142	Hypothetical protein CBG14799
Locus_17778_Transcript_1/1_Conf_1.000	445	1	8.19E-04	XP_002635917.1	56	46.9802	69	39	Hypothetical protein CBG01142
Locus_17779_Transcript_1/1_Conf_1.000	391	0							
Locus_1778_Transcript_1/1_Conf_1.000	524	20	2.62E-38	EFO22634.1	91	161.77	95	87	fibronectin type III domain-containing protein
Locus_17780_Transcript_1/1_Conf_1.000	205	0							
Locus_17781_Transcript_1/1_Conf_1.000	173	0							
Locus_17782_Transcript_1/1_Conf_1.000	924	14	2.38E-33	NP_500590.2	53	147.132	291	155	hypothetical protein F28E10.4
Locus_17783_Transcript_1/1_Conf_1.000	636	20	3.75E-77	NP_490992.2	79	291.582	211	168	hypothetical protein Y23H5B.4
Locus_17784_Transcript_1/1_Conf_1.000	691	20	4.08E-86	XP_002644575.1	85	321.627	204	175	Hypothetical protein CBG14518
Locus_17785_Transcript_1/1_Conf_1.000	483	20	5.10E-38	XP_001896380.1	76	160.614	164	125	XAP5 protein
Locus_17786_Transcript_1/1_Conf_1.000	200	0							
Locus_17787_Transcript_1/1_Conf_1.000	308	0							
Locus_17788_Transcript_1/1_Conf_1.000	277	4	1.37E-11	XP_001900186.1	62	72.7886	94	59	hypothetical protein
Locus_17789_Transcript_1/1_Conf_1.000	238	0							
Locus_1779_Transcript_1/1_Conf_1.000	274	4	8.91E-11	NP_492777.1	70	70.0922	71	50	hypothetical protein C43H8.2
Locus_17790_Transcript_1/1_Conf_1.000	166	0							
Locus_17791_Transcript_1/1_Conf_1.000	444	20	1.17E-26	XP_002640105.1	63	122.865	149	95	C. briggsae CBR-MUT-2 protein
Locus_17792_Transcript_1/1_Conf_1.000	340	20	2.91E-38	NP_497918.1	81	161.384	112	91	UDP-GlucuronosylTransferase family member (ugt-62)
Locus_17793_Transcript_1/1_Conf_1.000	488	20	4.44E-42	NP_502445.1	71	174.096	160	114	hypothetical protein M02B1.3

Locus_17794_Transcript_1/1_Conf_1.000	310	20	3.80E-46	XP_002643972.1	95	187.578	103	98	C. briggsae CBR-SLO-2 protein
Locus_17795_Transcript_1/1_Conf_1.000	178	20	5.53E-21	NP_502367.2	89	103.99	59	53	hypothetical protein F28D1.9
Locus_17796_Transcript_1/1_Conf_1.000	213	1	5.03E-06	NP_505001.1	64	54.299	64	41	hypothetical protein T05B11.1
Locus_17797_Transcript_1/1_Conf_1.000	469	20	9.28E-16	XP_002129449.1	50	86.6557	160	80	PREDICTED: hypothetical protein
Locus_17798_Transcript_1/1_Conf_1.000	399	20	1.02E-46	NP_497261.2	88	189.504	114	101	SyNapTotagmin family member (snt-2)
Locus_17799_Transcript_1/1_Conf_1.000	289	9	2.82E-12	EFO27735.1	58	75.0998	97	57	leucine Rich Repeat family protein
Locus_178_Transcript_1/2_Conf_1.000	1557	20	0	NP_491955.1	94	790.801	437	414	hypothetical protein K02F2.2

Locus_178_Transcript_2/2_Conf_1.000	1557	20	0	NP_491955.1	94	790.801	437	414	hypothetical protein K02F2.2
Locus_1780_Transcript_1/1_Conf_1.000	1590	20	3.63E-26	XP_002130953.1	44	124.405	547	241	PREDICTED: similar to chromosome 10 open reading frame 119
Locus_17800_Transcript_1/1_Conf_1.000	240	0							
Locus_17801_Transcript_1/1_Conf_1.000	247	9	3.25E-21	NP_871810.1	92	104.76	56	52	HAlF transporter (PGP related) family member (haf-9)
Locus_17802_Transcript_1/1_Conf_1.000	170	0							
Locus_17803_Transcript_1/1_Conf_1.000	419	14	1.45E-16	NP_510686.4	61	89.3521	112	69	hypothetical protein F59C12.3
Locus_17804_Transcript_1/1_Conf_1.000	341	20	4.32E-58	XP_002760341.1	100	227.254	110	110	PREDICTED: glutamine synthetase
Locus_17805_Transcript_1/1_Conf_1.000	353	5	8.68E-22	XP_002631039.1	73	106.686	82	60	Hypothetical protein CBG02799
Locus_17806_Transcript_1/2_Conf_1.000	334	20	5.19E-27	AAN11401.1	69	124.02	108	75	metalloprotease 1 precursor
Locus_17806_Transcript_2/2_Conf_1.000	352	20	1.25E-28	AAN11401.1	68	129.413	114	78	metalloprotease 1 precursor
Locus_17807_Transcript_1/1_Conf_1.000	691	0							
Locus_17808_Transcript_1/1_Conf_1.000	912	2	1.97E-32	CAR63629.1	57	144.05	259	150	hypothetical protein
Locus_17809_Transcript_1/1_Conf_1.000	426	20	2.87E-25	NP_872207.1	61	118.242	150	92	hypothetical protein Y69H2.14
Locus_1781_Transcript_1/1_Conf_1.000	230	0							

Locus_17810_Transcript_1/1_Conf_1.000	323	20	1.29E-41	EFO20030.1	89	172.555	107	96	toll and interleukin 1 receptor domain-containing protein isoform f
Locus_17811_Transcript_1/2_Conf_1.000	327	20	5.23E-27	XP_002640811.1	71	124.02	108	77	Hypothetical protein CBG15695
Locus_17811_Transcript_2/2_Conf_1.000	327	20	5.23E-27	XP_002640811.1	71	124.02	108	77	Hypothetical protein CBG15695
Locus_17812_Transcript_1/1_Conf_1.000	137	0							
Locus_17813_Transcript_1/1_Conf_1.000	162	0							
Locus_17814_Transcript_1/1_Conf_1.000	224	0							
Locus_17815_Transcript_1/1_Conf_1.000	318	0							
Locus_17816_Transcript_1/1_Conf_1.000	527	0							
Locus_17817_Transcript_1/1_Conf_1.000	304	0							
Locus_17818_Transcript_1/1_Conf_1.000	238	0							
Locus_17819_Transcript_1/1_Conf_1.000	239	0							
Locus_1782_Transcript_1/1_Conf_1.000	404	20	1.15E-74	AAH86899.1	100	282.337	133	133	Ribosomal protein S8
Locus_17820_Transcript_1/1_Conf_1.000	352	20	2.61E-26	NP_502060.1	73	121.709	108	79	TransThyretin-Related family domain family member (ttr-16)
Locus_17821_Transcript_1/1_Conf_1.000	376	0							
Locus_17822_Transcript_1/1_Conf_1.000	402	20	8.04E-52	NP_510711.2	86	206.453	135	117	AMP-Activated Kinase family member (aak-2)
Locus_17823_Transcript_1/1_Conf_1.000	265	0							
Locus_17824_Transcript_1/1_Conf_1.000	142	0							
Locus_17825_Transcript_1/1_Conf_1.000	191	0							
Locus_17826_Transcript_1/1_Conf_1.000	755	4	1.18E-15	NP_509620.2	48	87.8113	175	84	hypothetical protein C11E4.6
Locus_17827_Transcript_1/1_Conf_1.000	412	1	2.66E-23	NP_502795.2	72	111.694	90	65	Homolog of Hedgehog AcylTransferase family member (hhat-2)
Locus_17828_Transcript_1/1_Conf_1.000	181	0							
Locus_17829_Transcript_1/1_Conf_1.000	361	20	2.19E-09	EFN83858.1	75	65.4698	52	39	Histone-lysine N-methyltransferase SETMAR
Locus_1783_Transcript_1/1_Conf_1.000	1706	20	0	NP_001022078.1	91	894.419	524	479	ALdehyde deHydrogenase family member (alh-8)
Locus_17830_Transcript_1/1_Conf_1.000	559	20	3.87E-36	EFO24024.1	97	154.836	81	79	CMGC/DYRK/HIPK protein kinase
Locus_17831_Transcript_1/1_Conf_1.000	202	0							

Locus_17832_Transcript_1/1_Conf_1.000	171	20	6.58E-22	XP_002823622.1	100	107.071	48	48	PREDICTED: decorin-like isoform 1
Locus_17833_Transcript_1/1_Conf_1.000	171	0							
Locus_17834_Transcript_1/1_Conf_1.000	144	20	1.46E-08	BAA95178.1	71	62.7734	49	35	hgPTPR2Ab
Locus_17835_Transcript_1/1_Conf_1.000	547	20	3.21E-48	NP_490889.1	82	194.897	140	115	hypothetical protein Y71G12B.10
Locus_17836_Transcript_1/1_Conf_1.000	156	0							
Locus_17837_Transcript_1/1_Conf_1.000	155	2	1.89E-08	NP_495090.1	82	62.3882	50	41	hypothetical protein C17G10.7
Locus_17838_Transcript_1/1_Conf_1.000	466	20	4.04E-27	NP_001021801.1	87	124.405	74	65	hypothetical protein Y6B3B.5

Locus_17839_Transcript_1/1_Conf_1.000	1289	20	2.39E-147	NP_740931.2	79	526.554	433	345	hypothetical protein Y105E8A.3
Locus_1784_Transcript_1/1_Conf_1.000	2433	20	0	XP_002647083.1	86	1048.88	678	584	Hypothetical protein CBG03613
Locus_17840_Transcript_1/1_Conf_1.000	158	0							
Locus_17841_Transcript_1/1_Conf_1.000	342	20	1.07E-19	XP_001649524.1	70	99.7525	103	73	multiple ankyrin repeats single kh domain protein
Locus_17842_Transcript_1/1_Conf_1.000	670	20	5.32E-64	NP_001041144.1	83	248.054	171	143	hypothetical protein H27A22.1
Locus_17843_Transcript_1/1_Conf_1.000	383	20	1.97E-42	NP_492423.2	81	175.252	127	103	FCP1 (yeast TFIIF-interacting CTD phosphatase subunit) homolog family member (fcp-1)
Locus_17844_Transcript_1/1_Conf_1.000	199	20	1.26E-12	NP_008817.1	100	76.2554	38	38	ATP synthase subunit epsilon, mitochondrial
Locus_17845_Transcript_1/1_Conf_1.000	502	20	1.71E-09	XP_001635605.1	55	65.855	108	60	predicted protein
Locus_17846_Transcript_1/1_Conf_1.000	152	0							

Locus_17847_Transcript_1/1_Conf_1.000	258	20	2.23E-17	EAW98190.1	100	92.0485	38	38	cytochrome c oxidase subunit VIa polypeptide 1, isoform CRA_a
Locus_17848_Transcript_1/1_Conf_1.000	351	20	1.05E-43	XP_002640379.1	87	179.489	116	102	C. briggsae CBR-OPT-3 protein
Locus_17849_Transcript_1/1_Conf_1.000	277	0							
Locus_1785_Transcript_1/1_Conf_1.000	162	2	1.34E-06	NP_500107.1	70	56.225	54	38	hypothetical protein Y41D4B.11
Locus_17850_Transcript_1/1_Conf_1.000	785	20	1.28E-124	CBW48341.1	91	449.899	261	239	Hypothetical protein C27H6.1c
Locus_17851_Transcript_1/1_Conf_1.000	329	20	2.77E-28	XP_002640543.1	77	128.257	94	73	C. briggsae CBR-SCRM-1 protein
Locus_17852_Transcript_1/1_Conf_1.000	683	20	5.81E-29	NP_501200.1	53	131.724	226	121	ROD (Drosophila RoughDeal) homolog family member (rod-1)
Locus_17853_Transcript_1/1_Conf_1.000	542	5	3.01E-19	Q9NA80.3	54	98.5969	186	101	Stress-activated map kinase-interacting protein 1 homolog
Locus_17854_Transcript_1/1_Conf_1.000	329	20	3.51E-23	NP_001041035.1	70	111.309	90	63	Temporarily Assigned Gene name family member (tag-208)
Locus_17855_Transcript_1/1_Conf_1.000	310	0							
Locus_17856_Transcript_1/1_Conf_1.000	317	4	5.32E-19	NP_492775.2	65	97.4413	105	69	LAMinin related. See also lmb- family member (lam-3)
Locus_17857_Transcript_1/1_Conf_1.000	562	20	2.75E-13	EFO20139.1	88	78.9518	42	37	peripheral plasma membrane protein CASK
Locus_17858_Transcript_1/1_Conf_1.000	646	4	4.05E-58	CAR63536.1	71	228.409	185	132	hypothetical protein
Locus_17859_Transcript_1/1_Conf_1.000	241	20	2.86E-17	EFO20687.1	83	91.6633	60	50	hypothetical protein LOAG_07803
Locus_1786_Transcript_1/2_Conf_1.000	507	20	1.20E-13	EFO25573.1	62	79.7221	150	94	RING finger protein 20
Locus_1786_Transcript_2/2_Conf_1.000	507	20	1.20E-13	EFO25573.1	62	79.7221	150	94	RING finger protein 20
Locus_17860_Transcript_1/1_Conf_1.000	178	5	1.37E-11	NP_510744.1	75	72.7886	60	45	hypothetical protein F52G3.1

Locus_17861_Transcript_1/1_Conf_1.000	284	16	2.11E-07	XP_002643990.1	54	58.9214	95	52	C. briggsae CBR-VHL-1 protein
Locus_17862_Transcript_1/1_Conf_1.000	168	0							
Locus_17863_Transcript_1/1_Conf_1.000	242	2	3.62E-12	NP_490776.2	75	74.7146	58	44	hypothetical protein Y18H1A.9
Locus_17864_Transcript_1/1_Conf_1.000	435	20	2.22E-70	XP_002631669.1	95	268.085	144	137	C. briggsae CBR-MOG-4 protein
Locus_17865_Transcript_1/1_Conf_1.000	155	0							
Locus_17866_Transcript_1/1_Conf_1.000	153	0							
Locus_17867_Transcript_1/1_Conf_1.000	303	0							
Locus_17868_Transcript_1/1_Conf_1.000	671	0							
Locus_17869_Transcript_1/1_Conf_1.000	273	2	5.05E-14	NP_502337.1	95	80.8777	42	40	hypothetical protein C42C1.10
Locus_1787_Transcript_1/1_Conf_1.000	1162	20	5.07E-29	XP_001898268.1	53	133.265	214	114	Eukaryotic-type carbonic anhydrase family protein
Locus_17870_Transcript_1/1_Conf_1.000	444	20	8.97E-35	XP_001902632.1	80	149.828	130	104	Arid4a protein
Locus_17871_Transcript_1/1_Conf_1.000	365	0							
Locus_17872_Transcript_1/1_Conf_1.000	260	0							
Locus_17873_Transcript_1/1_Conf_1.000	160	0							
Locus_17874_Transcript_1/1_Conf_1.000	253	0							
Locus_17875_Transcript_1/1_Conf_1.000	342	20	4.98E-17	XP_002640122.1	75	90.8929	100	75	C. briggsae CBR-NCBP-1 protein
Locus_17876_Transcript_1/1_Conf_1.000	1007	20	5.07E-88	CAA16382.5	63	328.946	360	228	C. elegans protein Y45F10D.7, partially confirmed by transcript evidence
Locus_17877_Transcript_1/1_Conf_1.000	265	0							

Locus_17878_Transcript_1/1_Conf_1.000	201	20	1.63E-20	XP_002834351.1	100	102.449	43	43	PREDICTED: hypothetical protein LOC100438152
Locus_17879_Transcript_1/2_Conf_1.000	521	0							
Locus_17879_Transcript_2/2_Conf_1.000	521	0							
Locus_1788_Transcript_1/1_Conf_1.000	962	20	2.24E-106	EFO22691.1	83	389.808	291	244	hypothetical protein LOAG_05796
Locus_17880_Transcript_1/1_Conf_1.000	494	4	2.75E-44	CAR63586.1	90	181.415	102	92	hypothetical protein
Locus_17881_Transcript_1/1_Conf_1.000	702	4	3.20E-33	XP_002645822.1	56	145.976	235	133	Hypothetical protein CBG07553
Locus_17882_Transcript_1/1_Conf_1.000	247	0							
Locus_17883_Transcript_1/1_Conf_1.000	433	20	4.85E-33	XP_002639673.1	76	144.05	145	111	Hypothetical protein CBG12391
Locus_17884_Transcript_1/1_Conf_1.000	316	20	7.63E-42	NP_501792.2	89	173.326	99	89	abnormal GONad development family member (gon-1)

Locus_17885_Transcript_1/1_Conf_1.000	431	20	1.68E-25	XP_001901813.1	64	119.013	117	75	rad50 family protein
Locus_17886_Transcript_1/1_Conf_1.000	226	20	1.70E-33	NP_001021324.1	97	145.591	74	72	hypothetical protein C10G6.1
Locus_17887_Transcript_1/1_Conf_1.000	402	0							
Locus_17888_Transcript_1/1_Conf_1.000	291	20	2.61E-34	XP_001895873.1	80	148.288	96	77	hypothetical protein
Locus_17889_Transcript_1/1_Conf_1.000	471	3	2.05E-31	NP_741656.2	63	138.658	156	99	hypothetical protein Y39B6A.41
Locus_1789_Transcript_1/1_Conf_1.000	468	20	2.44E-24	XP_002631600.1	58	115.161	153	89	Hypothetical protein CBG20781
Locus_17890_Transcript_1/1_Conf_1.000	204	2	4.59E-07	XP_002634011.1	60	57.7658	65	39	Hypothetical protein CBG01544
Locus_17891_Transcript_1/1_Conf_1.000	274	5	1.98E-26	NP_001024333.1	81	122.094	87	71	Olfactory LeaRNing defective family member (olrn-1)
Locus_17892_Transcript_1/1_Conf_1.000	300	0							
Locus_17893_Transcript_1/1_Conf_1.000	253	0							
Locus_17894_Transcript_1/2_Conf_1.000	323	0							
Locus_17894_Transcript_2/2_Conf_1.000	204	0							
Locus_17895_Transcript_1/1_Conf_1.000	302	0							
Locus_17896_Transcript_1/1_Conf_1.000	706	20	3.62E-45	CAA21636.3	91	185.652	124	113	C. elegans protein Y38F1A.3, partially confirmed by transcript evidence
Locus_17897_Transcript_1/1_Conf_1.000	216	0							
Locus_17898_Transcript_1/1_Conf_1.000	360	0							
Locus_17899_Transcript_1/1_Conf_1.000	319	20	2.30E-22	NP_492340.1	69	108.612	94	65	hypothetical protein F43G9.10

Locus_179_Transcript_1/2_Conf_1.000	421	20	1.06E-43	Q07160.1	81	179.489	119	97	Heat shock protein homolog
Locus_179_Transcript_2/2_Conf_1.000	182	20	1.90E-24	CAG25499.1	95	115.546	60	57	heat shock protein 20
Locus_1790_Transcript_1/6_Conf_0.350	442	20	2.01E-18	AAD51334.1	70	95.5153	74	52	Kunitz type serine protease inhibitor
Locus_1790_Transcript_2/6_Conf_0.350	400	20	2.01E-18	AAD51334.1	70	95.5153	74	52	Kunitz type serine protease inhibitor
Locus_1790_Transcript_3/6_Conf_0.200	676	20	4.51E-18	AAD51334.1	70	95.5153	74	52	Kunitz type serine protease inhibitor
Locus_1790_Transcript_4/6_Conf_0.200	676	20	4.51E-18	AAD51334.1	70	95.5153	74	52	Kunitz type serine protease inhibitor
Locus_1790_Transcript_5/6_Conf_0.400	556	20	2.80E-18	AAD51334.1	70	95.5153	74	52	Kunitz type serine protease inhibitor

Locus_1790_Transcript_6/6_Conf_0.400	556	20	2.80E-18	AAD51334.1	70	95.5153	74	52	Kunitz type serine protease inhibitor
Locus_17900_Transcript_1/1_Conf_1.000	566	2	4.77E-13	XP_002640628.1	73	78.1814	57	42	C. briggsae CBR-EAT-18 protein
Locus_17901_Transcript_1/1_Conf_1.000	157	1	3.33E-05	XP_002637780.1	68	51.6026	50	34	Hypothetical protein CBG04563
Locus_17902_Transcript_1/1_Conf_1.000	368	14	4.09E-16	XP_002415665.1	61	87.8113	118	72	conserved hypothetical protein
Locus_17903_Transcript_1/1_Conf_1.000	370	5	3.68E-20	NP_492775.2	75	101.293	84	63	LAMinin related. See also lmb- family member (lam-3)
Locus_17904_Transcript_1/1_Conf_1.000	832	20	2.92E-69	XP_002632892.1	70	266.159	297	208	Hypothetical protein CBG15100
Locus_17905_Transcript_1/1_Conf_1.000	169	20	1.16E-18	XP_002743627.1	87	96.2857	56	49	PREDICTED: hypothetical protein LOC100405423
Locus_17906_Transcript_1/1_Conf_1.000	310	1	1.91E-05	EFO26102.1	46	52.373	101	47	hypothetical protein LOAG_02385
Locus_17907_Transcript_1/1_Conf_1.000	274	0							
Locus_17908_Transcript_1/1_Conf_1.000	565	20	2.36E-28	XP_002642471.1	67	129.028	136	92	Hypothetical protein CBG06887
Locus_17909_Transcript_1/1_Conf_1.000	135	0							
Locus_1791_Transcript_1/1_Conf_1.000	290	20	2.39E-11	NP_001122454.1	77	72.0182	48	37	abnormal ThermoTaXis family member (ttx-7)
Locus_17910_Transcript_1/1_Conf_1.000	935	20	4.02E-129	XP_002634971.1	89	465.307	284	255	Hypothetical protein CBG13507
Locus_17911_Transcript_1/1_Conf_1.000	229	0							
Locus_17912_Transcript_1/1_Conf_1.000	214	0							
Locus_17913_Transcript_1/1_Conf_1.000	597	0							
Locus_17914_Transcript_1/4_Conf_0.625	490	0							
Locus_17914_Transcript_2/4_Conf_0.625	490	0							
Locus_17914_Transcript_3/4_Conf_0.625	497	0							
Locus_17914_Transcript_4/4_Conf_0.625	497	0							

Locus_17915_Transcript_1/1_Conf_1.000	377	3	2.54E-05	EFO27305.1	45	51.9878	127	58	hypothetical protein LOAG_01169
Locus_17916_Transcript_1/1_Conf_1.000	899	20	6.13E-39	ABD39382.1	86	165.622	133	115	putative galectin protein 140
Locus_17917_Transcript_1/1_Conf_1.000	242	0							
Locus_17918_Transcript_1/1_Conf_1.000	559	20	1.23E-74	XP_002630326.1	84	282.722	186	158	Hypothetical protein CBG04253
Locus_17919_Transcript_1/1_Conf_1.000	252	20	1.97E-13	XP_002634749.1	67	78.9518	81	55	Hypothetical protein CBG21079
Locus_1792_Transcript_1/1_Conf_1.000	1361	5	6.18E-16	XP_001896257.1	51	90.1225	193	100	hypothetical protein Bm1_24000
Locus_17920_Transcript_1/1_Conf_1.000	143	0							
Locus_17921_Transcript_1/1_Conf_1.000	159	3	7.90E-07	XP_002637600.1	77	56.9954	53	41	C. briggsae CBR-SULP-8 protein
Locus_17922_Transcript_1/1_Conf_1.000	145	1	1.67E-04	XP_001897983.1	68	49.2914	48	33	RIKEN cDNA 2700097O09
Locus_17923_Transcript_1/1_Conf_1.000	358	0							
Locus_17924_Transcript_1/1_Conf_1.000	183	0							
Locus_17925_Transcript_1/1_Conf_1.000	136	0							
Locus_17926_Transcript_1/1_Conf_1.000	142	0							
Locus_17927_Transcript_1/1_Conf_1.000	156	2	3.68E-04	XP_001512989.1	57	48.1358	52	30	PREDICTED: similar to protocadherin 19
Locus_17928_Transcript_1/1_Conf_1.000	775	20	1.78E-54	EFO21392.1	69	216.853	230	159	hypothetical protein LOAG_07098
Locus_17929_Transcript_1/2_Conf_1.000	332	20	1.09E-16	EFO13838.1	90	89.7373	64	58	hypothetical protein LOAG_14688
Locus_17929_Transcript_2/2_Conf_1.000	332	20	1.09E-16	EFO13838.1	90	89.7373	64	58	hypothetical protein LOAG_14688
Locus_1793_Transcript_1/1_Conf_1.000	341	0							
Locus_17930_Transcript_1/1_Conf_1.000	150	18	2.74E-15	NP_502587.2	89	85.1149	49	44	Pelle/IL-1 receptor associated Kinase (IRAK) family member (pik-1)
Locus_17931_Transcript_1/1_Conf_1.000	505	15	3.77E-20	EFO23262.1	60	101.293	116	70	hypothetical protein LOAG_05225

Locus_17932_Transcript_1/1_Conf_1.000	282	0							
Locus_17933_Transcript_1/1_Conf_1.000	445	20	2.18E-73	XP_002638687.1	92	278.1	148	137	Hypothetical protein CBG11882
Locus_17934_Transcript_1/1_Conf_1.000	429	20	4.37E-66	NP_001040875.1	91	253.832	143	131	hypothetical protein T23G5.2
Locus_17935_Transcript_1/1_Conf_1.000	134	0							
Locus_17936_Transcript_1/1_Conf_1.000	263	1	5.12E-14	XP_001166450.1	84	80.8777	66	56	PREDICTED: hypothetical protein
Locus_17937_Transcript_1/1_Conf_1.000	276	0							
Locus_17938_Transcript_1/1_Conf_1.000	148	0							
Locus_17939_Transcript_1/1_Conf_1.000	260	5	6.71E-14	CAA84706.3	63	80.4925	87	55	C. elegans protein C38H2.1, partially confirmed by transcript evidence
Locus_1794_Transcript_1/1_Conf_1.000	1468	20	4.89E-22	AAN05752.1	64	110.538	128	83	heat shock protein 20
Locus_17940_Transcript_1/1_Conf_1.000	591	0							
Locus_17941_Transcript_1/1_Conf_1.000	470	0							
Locus_17942_Transcript_1/1_Conf_1.000	158	0							
Locus_17943_Transcript_1/1_Conf_1.000	259	20	4.04E-35	AAB37835.2	94	150.984	86	81	Dnaj domain (prokaryotic heat shock protein) protein 9, partially confirmed by transcript evidence
Locus_17944_Transcript_1/1_Conf_1.000	665	20	3.18E-37	XP_002639077.1	59	159.073	208	124	Hypothetical protein CBG14895
Locus_17945_Transcript_1/1_Conf_1.000	316	3	1.40E-27	NP_507767.2	79	125.946	107	85	hypothetical protein C14B4.2
Locus_17946_Transcript_1/1_Conf_1.000	374	20	5.46E-32	NP_001076652.1	94	140.584	74	70	NOT-Like (yeast CCR4/NOT complex component) family member (ntl-3)
Locus_17947_Transcript_1/1_Conf_1.000	276	5	1.79E-11	XP_002630778.1	79	72.4034	44	35	Hypothetical protein CBG02473
Locus_17948_Transcript_1/1_Conf_1.000	200	0							
Locus_17949_Transcript_1/1_Conf_1.000	128	0							
Locus_1795_Transcript_1/1_Conf_1.000	799	20	6.53E-47	EFO24294.1	62	191.815	253	157	hypothetical protein LOAG_04191
Locus_17950_Transcript_1/1_Conf_1.000	283	0							
Locus_17951_Transcript_1/1_Conf_1.000	636	0							

Locus_17952_Transcript_1/1_Conf_1.000	456	20	5.51E-45	NP_491087.1	85	183.726	151	129	MAP kinase kinase or Erk Kinase family member (mek-2)
Locus_17953_Transcript_1/1_Conf_1.000	351	20	3.39E-42	NP_491246.4	80	174.481	113	91	COenzyme Q (ubiquinone) biosynthesis family member (coq-4)
Locus_17954_Transcript_1/1_Conf_1.000	599	20	2.71E-84	XP_002643253.1	87	315.079	199	175	C. briggsae CBR-AEX-3 protein
Locus_17955_Transcript_1/1_Conf_1.000	147	0							
Locus_17956_Transcript_1/1_Conf_1.000	237	20	3.63E-12	NP_493027.1	71	74.7146	82	59	hypothetical protein R06C1.2
Locus_17957_Transcript_1/1_Conf_1.000	138	0							
Locus_17958_Transcript_1/1_Conf_1.000	192	0							
Locus_17959_Transcript_1/1_Conf_1.000	143	0							
Locus_1796_Transcript_1/2_Conf_1.000	2501	20	0	XP_002642411.1	73	819.305	754	553	C. briggsae CBR-INA-1 protein

Locus_1796_Transcript_2/2_Conf_1.000	2501	20	0	XP_002642411.1	73	819.305	754	553	C. briggsae CBR-INA-1 protein
Locus_17960_Transcript_1/1_Conf_1.000	309	20	9.16E-08	XP_002129690.1	49	60.077	73	36	PREDICTED: similar to C-type lectin domain family 4 member F (C-type lectin superfamily member 13) (C-type lectin 13) (Kupffer cell receptor)
Locus_17961_Transcript_1/1_Conf_1.000	343	0							
Locus_17962_Transcript_1/1_Conf_1.000	230	3	1.03E-14	XP_002638987.1	73	83.1889	76	56	Hypothetical protein CBG22233
Locus_17963_Transcript_1/1_Conf_1.000	274	20	7.47E-42	XP_002641621.1	90	173.326	91	82	Hypothetical protein CBG09941
Locus_17964_Transcript_1/1_Conf_1.000	249	0							
Locus_17965_Transcript_1/1_Conf_1.000	271	0							
Locus_17966_Transcript_1/1_Conf_1.000	259	20	8.45E-25	XP_002647748.1	80	116.701	86	69	C. briggsae CBR-WRS-1 protein
Locus_17967_Transcript_1/1_Conf_1.000	678	20	4.12E-27	NP_492329.1	62	125.561	142	89	hypothetical protein F43G9.13
Locus_17968_Transcript_1/1_Conf_1.000	179	20	9.76E-18	XP_002633968.1	87	93.2041	57	50	C. briggsae CBR-MCA-1 protein
Locus_17969_Transcript_1/1_Conf_1.000	146	20	8.86E-14	EFO20394.1	87	80.1073	48	42	hypothetical protein LOAG_08094
Locus_1797_Transcript_1/1_Conf_1.000	986	20	2.35E-82	XP_002636883.1	73	310.071	275	202	Hypothetical protein CBG09344
Locus_17970_Transcript_1/1_Conf_1.000	460	0							
Locus_17971_Transcript_1/1_Conf_1.000	135	0							

Locus_17972_Transcript_1/1_Conf_1.000	318	6	5.10E-38	XP_002636221.1	80	160.614	106	85	Hypothetical protein CBG12142
Locus_17973_Transcript_1/1_Conf_1.000	249	7	2.10E-20	EFO19192.1	74	102.064	83	62	MYND finger family protein
Locus_17974_Transcript_1/1_Conf_1.000	231	0							
Locus_17975_Transcript_1/1_Conf_1.000	157	0							
Locus_17976_Transcript_1/1_Conf_1.000	367	20	1.84E-16	NP_490726.1	76	88.9669	69	53	hypothetical protein R119.3
Locus_17977_Transcript_1/1_Conf_1.000	150	20	6.29E-20	P01023.3	100	100.523	49	49	Alpha-2-macroglobulin
Locus_17978_Transcript_1/1_Conf_1.000	171	1	4.28E-05	EFO22577.1	69	51.2174	55	38	hypothetical protein LOAG_05911
Locus_17979_Transcript_1/1_Conf_1.000	245	20	2.11E-28	XP_001897997.1	89	128.642	79	71	anion exchange protein

Locus_1798_Transcript_1/3_Conf_0.750	1313	20	2.79E-42	ADK91433.1	68	177.563	173	118	c-type lectin 1
Locus_1798_Transcript_2/3_Conf_0.750	1469	20	4.26E-42	ADK91433.1	68	177.178	173	119	c-type lectin 1

Locus_1798_Transcript_3/3_Conf_0.750	1469	20	1.12E-42	ADK91433.1	68	179.104	173	119	c-type lectin 1
Locus_17980_Transcript_1/1_Conf_1.000	180	0							
Locus_17981_Transcript_1/1_Conf_1.000	243	1	9.52E-05	XP_001895050.1	57	50.0618	54	31	hypothetical protein Bm1_17960
Locus_17982_Transcript_1/1_Conf_1.000	250	3	6.13E-04	XP_002634125.1	58	47.3654	62	36	C. briggsae CBR-MSH-5 protein
Locus_17983_Transcript_1/1_Conf_1.000	460	0							
Locus_17984_Transcript_1/1_Conf_1.000	185	4	4.24E-16	XP_002633677.1	80	87.8113	60	48	C. briggsae CBR-LAD-2 protein
Locus_17985_Transcript_1/1_Conf_1.000	426	20	1.75E-14	ACN62126.1	54	82.4185	142	78	tryptophan decarboxylase
Locus_17986_Transcript_1/1_Conf_1.000	156	20	1.10E-16	XP_002635876.1	94	89.7373	51	48	C. briggsae CBR-PTR-16 protein
Locus_17987_Transcript_1/1_Conf_1.000	143	0							
Locus_17988_Transcript_1/1_Conf_1.000	377	20	1.94E-21	NP_497480.2	65	105.531	127	83	POLH (DNA polymerase eta) homolog family member (polh-1)

Locus_17989_Transcript_1/1_Conf_1.000	374	20	3.41E-42	NP_001123000.1	77	174.481	124	96	hypothetical protein T05C3.6
Locus_1799_Transcript_1/1_Conf_1.000	489	20	1.92E-45	XP_001901624.1	73	185.267	148	109	valyl-tRNA synthetase family protein
Locus_17990_Transcript_1/1_Conf_1.000	263	0							
Locus_17991_Transcript_1/1_Conf_1.000	270	20	1.74E-14	XP_002131575.1	68	82.4185	88	60	PREDICTED: mitogen-activated protein kinase kinase
Locus_17992_Transcript_1/1_Conf_1.000	242	0							
Locus_17993_Transcript_1/1_Conf_1.000	360	5	7.28E-13	ADI24623.1	54	77.0258	113	62	Copine domain protein, atypical protein 2, isoform c, partially confirmed by transcript evidence
Locus_17994_Transcript_1/1_Conf_1.000	324	0							
Locus_17995_Transcript_1/1_Conf_1.000	327	0							
Locus_17996_Transcript_1/1_Conf_1.000	671	0							
Locus_17997_Transcript_1/1_Conf_1.000	251	0							
Locus_17998_Transcript_1/1_Conf_1.000	478	2	5.38E-08	XP_002631107.1	50	60.8474	122	62	Hypothetical protein CBG02882
Locus_17999_Transcript_1/1_Conf_1.000	180	20	1.41E-27	XP_002922896.1	100	125.946	60	60	PREDICTED: macrophage-capping protein-like
Locus_18_Transcript_1/1_Conf_1.000	1840	20	0	NP_492114.2	87	783.482	513	450	hypothetical protein T25G3.3
Locus_180_Transcript_1/6_Conf_0.111	1363	20	3.42E-115	XP_002640295.1	90	419.853	252	228	Hypothetical protein CBG12821

Locus_180_Transcript_2/6_Conf_0.111	1363	20	3.42E-115	XP_002640295.1	90	419.853	252	228	Hypothetical protein CBG12821
Locus_180_Transcript_3/6_Conf_0.278	738	12	5.45E-26	CAJ45481.1	68	122.094	122	84	legumain
Locus_180_Transcript_4/6_Conf_0.278	780	8	6.70E-25	CAJ45481.1	67	118.627	122	82	legumain
Locus_180_Transcript_5/6_Conf_0.389	2216	20	1.60E-134	CAJ45481.1	72	484.952	404	294	legumain
Locus_180_Transcript_6/6_Conf_0.389	2174	20	1.41E-135	CAJ45481.1	73	488.419	404	296	legumain
Locus_1800_Transcript_1/1_Conf_1.000	424	5	8.44E-09	XP_002631731.1	94	63.5438	52	49	Hypothetical protein CBG20933
Locus_18000_Transcript_1/1_Conf_1.000	589	20	8.19E-22	NP_491362.1	55	107.457	187	104	hypothetical protein ZK973.1
Locus_18001_Transcript_1/1_Conf_1.000	414	20	2.23E-38	NP_497589.3	81	161.77	138	113	hypothetical protein Y71H2B.5

Locus_18002_Transcript_1/1_Conf_1.000	303	20	6.16E-20	XP_001899247.1	81	100.523	100	81	Probable nucleolar GTP-binding protein 1.
Locus_18003_Transcript_1/1_Conf_1.000	128	0							
Locus_18004_Transcript_1/1_Conf_1.000	153	7	5.32E-11	XP_001897512.1	87	70.8626	41	36	Patched family protein
Locus_18005_Transcript_1/2_Conf_1.000	400	3	7.91E-07	CBX25202.1	46	56.9954	110	51	Hypothetical protein Y17G7B.23a
Locus_18005_Transcript_2/2_Conf_1.000	400	3	7.91E-07	CBX25202.1	46	56.9954	110	51	Hypothetical protein Y17G7B.23a
Locus_18006_Transcript_1/1_Conf_1.000	135	4	4.26E-13	XP_002637318.1	82	77.7962	45	37	Hypothetical protein CBG19009
Locus_18007_Transcript_1/1_Conf_1.000	596	20	7.36E-34	XP_002640693.1	58	147.517	204	119	Hypothetical protein CBG19758
Locus_18008_Transcript_1/1_Conf_1.000	377	20	5.24E-43	XP_002646355.1	88	177.178	109	96	Hypothetical protein CBG12069
Locus_18009_Transcript_1/1_Conf_1.000	441	6	1.60E-15	NP_497868.1	56	85.8853	138	78	Temporarily Assigned Gene name family member (tag-131)
Locus_1801_Transcript_1/1_Conf_1.000	700	20	2.76E-45	EFO22561.1	73	186.037	169	124	hypothetical protein LOAG_05921
Locus_18010_Transcript_1/1_Conf_1.000	621	20	1.17E-64	NP_495666.3	80	249.98	212	170	Temporarily Assigned Gene name family member (tag-341)
Locus_18011_Transcript_1/1_Conf_1.000	441	0							
Locus_18012_Transcript_1/1_Conf_1.000	1068	5	1.65E-15	NP_001022123.1	46	88.1965	273	126	hypothetical protein F28C6.4
Locus_18013_Transcript_1/1_Conf_1.000	169	20	6.17E-20	XP_002641994.1	96	100.523	56	54	Hypothetical protein CBG09217
Locus_18014_Transcript_1/1_Conf_1.000	151	2	3.35E-05	NP_508866.1	75	51.6026	53	40	Unidentified Vitellogenin-linked Transcript family member (uvt-3)
Locus_18015_Transcript_1/1_Conf_1.000	226	0							

Locus_18016_Transcript_1/1_Conf_1.000	632	20	2.95E-10	BAF47075.1	53	69.3218	136	73	AP2 homolog
Locus_18017_Transcript_1/1_Conf_1.000	469	20	1.09E-40	EFO20767.1	73	169.474	156	114	Smad1
Locus_18018_Transcript_1/1_Conf_1.000	285	0							
Locus_18019_Transcript_1/1_Conf_1.000	662	5	7.05E-13	XP_002639135.1	50	78.1814	216	109	C. briggsae CBR-SEP-1 protein
Locus_1802_Transcript_1/1_Conf_1.000	145	0							

Locus_18020_Transcript_1/1_Conf_1.000	205	20	4.42E-26	XP_002832350.1	100	120.939	68	68	PREDICTED: LOW QUALITY PROTEIN: filamin-A-like
Locus_18021_Transcript_1/1_Conf_1.000	412	20	8.25E-33	NP_505116.1	66	143.28	137	91	hypothetical protein K07C11.7
Locus_18022_Transcript_1/1_Conf_1.000	269	20	1.95E-16	NP_001122749.1	70	83.1889	80	56	hypothetical protein ZK643.3
Locus_18023_Transcript_1/1_Conf_1.000	176	2	1.28E-09	XP_002637557.1	75	66.2402	56	42	Hypothetical protein CBG19289
Locus_18024_Transcript_1/1_Conf_1.000	168	20	7.08E-08	XP_002643075.1	79	60.4622	44	35	Hypothetical protein CBG22995
Locus_18025_Transcript_1/1_Conf_1.000	231	0							
Locus_18026_Transcript_1/1_Conf_1.000	222	20	3.01E-30	BAE90469.1	90	134.806	72	65	unnamed protein product
Locus_18027_Transcript_1/1_Conf_1.000	1774	20	0	NP_508475.2	79	697.582	587	468	Inner Membrane of MiTochondria protein homolog family member (immt-1)
Locus_18028_Transcript_1/1_Conf_1.000	378	0							
Locus_18029_Transcript_1/1_Conf_1.000	177	7	1.97E-10	AAN61521.1	70	68.9366	58	41	301KDa_2 protein
Locus_1803_Transcript_1/1_Conf_1.000	922	20	9.02E-25	XP_002639695.1	72	118.627	108	78	C. briggsae CBR-FCP-1 protein
Locus_18030_Transcript_1/1_Conf_1.000	130	0							
Locus_18031_Transcript_1/1_Conf_1.000	135	0							
Locus_18032_Transcript_1/1_Conf_1.000	131	0							
Locus_18033_Transcript_1/1_Conf_1.000	265	0							
Locus_18034_Transcript_1/1_Conf_1.000	384	0							
Locus_18035_Transcript_1/1_Conf_1.000	185	0							
Locus_18036_Transcript_1/1_Conf_1.000	259	0							

Locus_18037_Transcript_1/1_Conf_1.000	168	0							
Locus_18038_Transcript_1/1_Conf_1.000	276	0							
Locus_18039_Transcript_1/1_Conf_1.000	432	2	2.27E-06	NP_495102.1	59	55.4546	74	44	hypothetical protein F59E12.11
Locus_1804_Transcript_1/6_Conf_0.762	1122	0							
Locus_1804_Transcript_2/6_Conf_0.762	1119	0							
Locus_1804_Transcript_3/6_Conf_0.762	1140	0							
Locus_1804_Transcript_4/6_Conf_0.762	1161	0							
Locus_1804_Transcript_5/6_Conf_0.762	1119	0							
Locus_1804_Transcript_6/6_Conf_0.762	1128	0							
Locus_18040_Transcript_1/1_Conf_1.000	369	0							
Locus_18041_Transcript_1/1_Conf_1.000	243	0							
Locus_18042_Transcript_1/1_Conf_1.000	256	0							
Locus_18043_Transcript_1/1_Conf_1.000	368	20	4.82E-25	3N9L	66	117.472	117	78	Cekdm7a From C.Elegans
Locus_18044_Transcript_1/1_Conf_1.000	285	0							
Locus_18045_Transcript_1/1_Conf_1.000	549	20	2.42E-51	NP_492132.1	73	205.297	178	131	Enhancer of Glp-One (glp-1) family member (ego-1)
Locus_18046_Transcript_1/1_Conf_1.000	424	20	1.43E-40	XP_002634303.1	76	169.088	141	108	C. briggsae CBR-NCX-3 protein
Locus_18047_Transcript_1/1_Conf_1.000	548	20	1.87E-64	NP_493426.2	87	248.825	183	160	TAF (TBP-associated transcription factor) family member (taf-1)
Locus_18048_Transcript_1/1_Conf_1.000	818	20	1.36E-39	CBW48397.1	72	167.548	157	114	Hypothetical protein F54C8.7e
Locus_18049_Transcript_1/1_Conf_1.000	702	20	2.98E-47	XP_002640651.1	65	192.586	231	152	Hypothetical protein CBG19709

Locus_1805_Transcript_1/1_Conf_1.000	1075	20	1.21E-90	EFO19288.1	96	337.806	179	173	RAB14 protein
Locus_18050_Transcript_1/1_Conf_1.000	451	4	3.07E-19	XP_002634268.1	55	98.2117	148	82	Hypothetical protein CBG01840
Locus_18051_Transcript_1/1_Conf_1.000	551	20	2.29E-17	XP_002123175.1	69	92.4337	92	64	PREDICTED: similar to predicted protein
Locus_18052_Transcript_1/1_Conf_1.000	244	0							
Locus_18053_Transcript_1/1_Conf_1.000	488	0							
Locus_18054_Transcript_1/1_Conf_1.000	265	0							
Locus_18055_Transcript_1/1_Conf_1.000	285	0							
Locus_18056_Transcript_1/2_Conf_1.000	457	20	1.33E-06	EFA09420.1	50	56.225	120	60	hypothetical protein TcasGA2_TC005222

Locus_18056_Transcript_2/2_Conf_1.000	457	20	1.33E-06	EFA09420.1	50	56.225	120	60	hypothetical protein TcasGA2_TC005222
Locus_18057_Transcript_1/1_Conf_1.000	347	20	3.54E-31	XP_001900176.1	89	137.887	82	73	LIM domain containing protein
Locus_18058_Transcript_1/1_Conf_1.000	182	0							
Locus_18059_Transcript_1/1_Conf_1.000	287	20	2.09E-23	NP_498250.3	74	112.079	95	71	POLQ (DNA polymerase theta) homolog family member (polq-1)
Locus_1806_Transcript_1/1_Conf_1.000	896	0							
Locus_18060_Transcript_1/1_Conf_1.000	153	18	1.50E-05	CBL43432.1	68	52.7582	47	32	C. elegans protein F01D4.1b, partially confirmed by transcript evidence
Locus_18061_Transcript_1/1_Conf_1.000	610	2	1.71E-12	NP_506085.2	45	76.6406	217	99	FERM domain (protein4.1-ezrin-radixin-moesin) family member (frm-7)
Locus_18062_Transcript_1/1_Conf_1.000	220	20	2.92E-33	XP_519230.2	100	144.821	72	72	PREDICTED: actin related protein 2/3 complex subunit 1B
Locus_18063_Transcript_1/1_Conf_1.000	525	20	1.47E-44	XP_002638481.1	70	182.57	175	123	Hypothetical protein CBG12909
Locus_18064_Transcript_1/1_Conf_1.000	306	0							
Locus_18065_Transcript_1/1_Conf_1.000	227	4	1.07E-11	NP_492746.2	94	73.1738	38	36	Temporarily Assigned Gene name family member (tag-68)
Locus_18066_Transcript_1/1_Conf_1.000	289	0							
Locus_18067_Transcript_1/1_Conf_1.000	732	0							
Locus_18068_Transcript_1/1_Conf_1.000	288	1	4.50E-10	NP_490992.2	79	67.781	48	38	hypothetical protein Y23H5B.4
Locus_18069_Transcript_1/1_Conf_1.000	246	0							

Locus_1807_Transcript_1/1_Conf_1.000	1724	20	0	XP_002634023.1	77	666.381	563	434	Hypothetical protein CBG01556
Locus_18070_Transcript_1/2_Conf_1.000	760	20	6.47E-62	NP_495796.1	74	241.506	225	168	RNase H family member (rnh-2)
Locus_18070_Transcript_2/2_Conf_1.000	760	20	4.95E-62	NP_495796.1	74	241.891	225	168	RNase H family member (rnh-2)
Locus_18071_Transcript_1/1_Conf_1.000	335	0							
Locus_18072_Transcript_1/1_Conf_1.000	174	20	1.85E-08	XP_002761583.1	93	62.3882	46	43	PREDICTED: 40S ribosomal protein S15-like
Locus_18073_Transcript_1/1_Conf_1.000	167	0							
Locus_18074_Transcript_1/1_Conf_1.000	320	2	1.26E-12	XP_002642676.1	57	76.2554	105	60	Hypothetical protein CBG12260
Locus_18075_Transcript_1/1_Conf_1.000	144	20	9.16E-11	AAL11454.1	88	70.0922	36	32	Skp1
Locus_18076_Transcript_1/1_Conf_1.000	165	0							
Locus_18077_Transcript_1/1_Conf_1.000	320	20	1.64E-28	XP_001894370.1	69	129.028	105	73	Immunoglobulin I-set domain containing protein
Locus_18078_Transcript_1/1_Conf_1.000	208	0							

Locus_18079_Transcript_1/1_Conf_1.000	178	20	4.39E-10	XP_002831205.1	100	67.781	34	34	PREDICTED: cyclic AMP-dependent transcription factor ATF-4-like
Locus_1808_Transcript_1/1_Conf_1.000	598	20	2.75E-20	XP_002638769.1	56	102.449	180	101	Hypothetical protein CBG05113
Locus_18080_Transcript_1/2_Conf_1.000	273	0							
Locus_18080_Transcript_2/2_Conf_1.000	252	0							
Locus_18081_Transcript_1/1_Conf_1.000	389	20	3.56E-52	XP_002648958.1	82	207.608	127	105	Hypothetical protein CBG21275
Locus_18082_Transcript_1/1_Conf_1.000	164	0							
Locus_18083_Transcript_1/1_Conf_1.000	310	0							
Locus_18084_Transcript_1/1_Conf_1.000	246	0							
Locus_18085_Transcript_1/1_Conf_1.000	156	0							
Locus_18086_Transcript_1/1_Conf_1.000	277	4	1.79E-19	NP_509455.3	73	98.9821	75	55	hypothetical protein F18E9.3
Locus_18087_Transcript_1/1_Conf_1.000	297	20	1.57E-39	XP_002639744.1	87	165.622	86	75	C. briggsae CBR-LAM-3 protein
Locus_18088_Transcript_1/1_Conf_1.000	146	0							
Locus_18089_Transcript_1/1_Conf_1.000	839	20	1.01E-93	XP_002634766.1	73	347.436	286	209	Hypothetical protein CBG05362
Locus_1809_Transcript_1/1_Conf_1.000	459	20	5.31E-40	XP_001898512.1	84	167.162	105	89	Protein kinase domain containing protein
Locus_18090_Transcript_1/1_Conf_1.000	187	0							
Locus_18091_Transcript_1/1_Conf_1.000	321	0							
Locus_18092_Transcript_1/1_Conf_1.000	1377	0							
Locus_18093_Transcript_1/2_Conf_1.000	732	20	8.83E-29	XP_968473.1	58	131.339	179	104	PREDICTED: similar to CG10343 CG10343-PA
Locus_18093_Transcript_2/2_Conf_1.000	720	20	8.51E-29	XP_968473.1	58	131.339	179	104	PREDICTED: similar to CG10343 CG10343-PA
Locus_18094_Transcript_1/1_Conf_1.000	237	20	3.51E-15	XP_002636807.1	74	84.7297	71	53	Hypothetical protein CBG09249
Locus_18095_Transcript_1/1_Conf_1.000	495	1	7.05E-08	NP_491118.2	46	60.4622	132	62	hypothetical protein Y54E10A.11
Locus_18096_Transcript_1/1_Conf_1.000	141	20	6.11E-12	XP_001998550.1	79	73.9442	44	35	GI23574

Locus_18097_Transcript_1/1_Conf_1.000	316	20	9.02E-43	XP_002638878.1	90	176.407	105	95	C. briggsae CBR-CSN-2 protein
Locus_18098_Transcript_1/1_Conf_1.000	521	0							
Locus_18099_Transcript_1/1_Conf_1.000	289	0							
Locus_181_Transcript_1/1_Conf_1.000	513	20	3.01E-20	NP_500698.1	79	101.679	81	64	Sperm-Specific family, class Q family member (ssq-2)
Locus_1810_Transcript_1/1_Conf_1.000	305	15	2.18E-33	NP_498273.1	85	145.206	90	77	hypothetical protein F01F1.2
Locus_18100_Transcript_1/1_Conf_1.000	234	0							
Locus_18101_Transcript_1/1_Conf_1.000	290	0							
Locus_18102_Transcript_1/1_Conf_1.000	269	20	3.90E-06	XP_002739735.1	49	54.6842	89	44	PREDICTED: UDP-Gal:betaGlcNAc beta 1,3-galactosyltransferase 1-like
Locus_18103_Transcript_1/1_Conf_1.000	249	20	6.30E-25	EFO27053.1	79	117.087	82	65	glutaminase
Locus_18104_Transcript_1/1_Conf_1.000	502	3	7.81E-71	CAR63658.1	83	269.626	165	138	hypothetical protein
Locus_18105_Transcript_1/1_Conf_1.000	265	4	2.29E-14	NP_492775.2	75	82.0333	69	52	LAMinin related. See also lmb- family member (lam-3)
Locus_18106_Transcript_1/1_Conf_1.000	548	8	1.00E-33	NP_500822.2	57	146.747	194	112	UNCoordinated family member (unc-5)
Locus_18107_Transcript_1/1_Conf_1.000	547	20	1.50E-21	NP_741042.1	98	106.301	53	52	human PTB (hnRNP) homolog family member (ptb-1)
Locus_18108_Transcript_1/1_Conf_1.000	203	0							
Locus_18109_Transcript_1/1_Conf_1.000	229	20	9.34E-16	NP_001041296.1	80	86.6557	47	38	COLlagen family member (col-181)

Locus_1811_Transcript_1/1_Conf_1.000	1312	20	6.88E-41	NP_491745.1	45	172.94	452	206	hypothetical protein C27A12.2
Locus_18110_Transcript_1/1_Conf_1.000	230	4	4.20E-08	EFO25668.1	64	61.2326	71	46	hypothetical protein LOAG_02820
Locus_18111_Transcript_1/1_Conf_1.000	252	20	6.55E-09	NP_499664.2	60	63.929	83	50	hypothetical protein BE10.2
Locus_18112_Transcript_1/1_Conf_1.000	203	0							
Locus_18113_Transcript_1/1_Conf_1.000	555	0							
Locus_18114_Transcript_1/1_Conf_1.000	158	0							
Locus_18115_Transcript_1/1_Conf_1.000	194	0							
Locus_18116_Transcript_1/1_Conf_1.000	401	20	6.89E-19	EFO28349.1	60	97.0561	105	63	major sperm protein
Locus_18117_Transcript_1/1_Conf_1.000	184	20	1.36E-14	NP_499749.1	87	82.8037	55	48	Aspergillus NUclear Division related family member (nud-1)
Locus_18118_Transcript_1/1_Conf_1.000	453	20	7.48E-42	XP_002640831.1	81	173.326	137	111	Hypothetical protein CBG15718
Locus_18119_Transcript_1/1_Conf_1.000	231	7	8.71E-14	XP_002630536.1	71	80.1073	70	50	Hypothetical protein CBG12976
Locus_1812_Transcript_1/1_Conf_1.000	161	0							
Locus_18120_Transcript_1/1_Conf_1.000	1040	17	5.98E-15	NP_499886.1	49	86.2705	135	67	hypothetical protein R02D3.7

Locus_18121_Transcript_1/1_Conf_1.000	758	20	2.03E-07	XP_002735551.1	44	60.4622	249	111	PREDICTED: janus kinase and microtubule interacting protein 1-like
Locus_18122_Transcript_1/1_Conf_1.000	243	0							
Locus_18123_Transcript_1/2_Conf_1.000	404	11	3.04E-67	ACZ64266.1	91	257.684	134	122	serine protease inhibitor
Locus_18123_Transcript_2/2_Conf_1.000	404	11	3.04E-67	ACZ64266.1	91	257.684	134	122	serine protease inhibitor
Locus_18124_Transcript_1/1_Conf_1.000	171	0							
Locus_18125_Transcript_1/1_Conf_1.000	216	0							
Locus_18126_Transcript_1/1_Conf_1.000	131	0							
Locus_18127_Transcript_1/1_Conf_1.000	372	2	3.92E-22	NP_501557.2	79	107.842	82	65	hypothetical protein C53B4.3
Locus_18128_Transcript_1/1_Conf_1.000	144	0							
Locus_18129_Transcript_1/1_Conf_1.000	136	0							
Locus_1813_Transcript_1/1_Conf_1.000	1352	0							
Locus_18130_Transcript_1/1_Conf_1.000	168	0							
Locus_18131_Transcript_1/2_Conf_0.333	152	0							
Locus_18131_Transcript_2/2_Conf_0.667	151	0							
Locus_18132_Transcript_1/1_Conf_1.000	191	20	3.33E-21	YP_002725716.1	88	104.76	63	56	NADH dehydrogenase subunit 1
Locus_18133_Transcript_1/1_Conf_1.000	271	0							
Locus_18134_Transcript_1/1_Conf_1.000	282	0							
Locus_18135_Transcript_1/1_Conf_1.000	232	0							
Locus_18136_Transcript_1/1_Conf_1.000	375	0							
Locus_18137_Transcript_1/1_Conf_1.000	351	0							

Locus_18138_Transcript_1/1_Conf_1.000	218	20	2.65E-15	XP_002635166.1	72	85.1149	72	52	Hypothetical protein CBG11402
Locus_18139_Transcript_1/1_Conf_1.000	162	0							
Locus_1814_Transcript_1/1_Conf_1.000	635	20	2.93E-74	NP_492761.1	92	281.952	153	141	hypothetical protein F25H2.5
Locus_18140_Transcript_1/1_Conf_1.000	153	5	7.67E-18	XP_002634716.1	91	93.5893	49	45	C. briggsae CBR-EXC-5 protein
Locus_18141_Transcript_1/1_Conf_1.000	1032	20	8.76E-83	AAX34423.1	67	311.612	336	228	anion transporter SULP-5
Locus_18142_Transcript_1/1_Conf_1.000	211	20	6.14E-20	XP_002645743.1	80	100.523	70	56	C. briggsae CBR-UNC-84 protein
Locus_18143_Transcript_1/1_Conf_1.000	140	0							
Locus_18144_Transcript_1/1_Conf_1.000	505	20	2.34E-62	NP_509572.1	84	241.506	156	132	AdaPtin, Alpha chain (clathrin associated complex) family member (apa-2)
Locus_18145_Transcript_1/1_Conf_1.000	146	0							

Locus_18146_Transcript_1/1_Conf_1.000	363	20	4.21E-45	NP_507972.1	84	184.111	122	103	hypothetical protein F26F2.7
Locus_18147_Transcript_1/1_Conf_1.000	721	20	3.18E-92	CBL43435.1	89	342.043	212	189	C. elegans protein F32F2.1e, partially confirmed by transcript evidence
Locus_18148_Transcript_1/1_Conf_1.000	494	20	2.05E-31	XP_002639518.1	61	138.658	153	94	Hypothetical protein CBG04124
Locus_18149_Transcript_1/1_Conf_1.000	147	2	3.84E-09	NP_505266.1	80	64.6994	47	38	hypothetical protein C01B7.5
Locus_1815_Transcript_1/1_Conf_1.000	1411	20	4.72E-67	XP_002630353.1	67	259.996	267	179	Hypothetical protein CBG04283
Locus_18150_Transcript_1/1_Conf_1.000	673	0							
Locus_18151_Transcript_1/1_Conf_1.000	217	0							
Locus_18152_Transcript_1/1_Conf_1.000	437	0							
Locus_18153_Transcript_1/1_Conf_1.000	145	0							
Locus_18154_Transcript_1/1_Conf_1.000	171	2	2.12E-12	XP_002342418.1	100	75.485	50	50	PREDICTED: hypothetical protein XP_002342418
Locus_18155_Transcript_1/1_Conf_1.000	395	20	7.63E-42	EFO26003.1	72	173.326	132	96	SPRY domain-containing protein
Locus_18156_Transcript_1/1_Conf_1.000	566	20	5.94E-64	NP_505071.1	77	247.284	188	145	hypothetical protein ZC513.5
Locus_18157_Transcript_1/1_Conf_1.000	860	20	1.77E-40	XP_002639601.1	66	170.629	247	165	Hypothetical protein CBG12310
Locus_18158_Transcript_1/1_Conf_1.000	227	0							
Locus_18159_Transcript_1/1_Conf_1.000	176	0							
Locus_1816_Transcript_1/1_Conf_1.000	1544	20	1.64E-92	EFO22955.1	58	344.739	470	273	MIZ zinc finger family protein
Locus_18160_Transcript_1/1_Conf_1.000	420	20	4.16E-48	XP_001900297.1	82	194.126	138	114	Importin-beta N-terminal domain containing protein

Locus_18161_Transcript_1/1_Conf_1.000	610	20	6.65E-57	AAC35506.1	81	224.172	158	129	putative actin-binding protein UNC-115
Locus_18162_Transcript_1/1_Conf_1.000	301	0							
Locus_18163_Transcript_1/1_Conf_1.000	201	0							
Locus_18164_Transcript_1/1_Conf_1.000	347	2	4.50E-10	XP_002636871.1	55	67.781	96	53	Hypothetical protein CBG09330
Locus_18165_Transcript_1/1_Conf_1.000	255	3	4.52E-10	XP_002633836.1	75	67.781	83	63	Hypothetical protein CBG19874
Locus_18166_Transcript_1/1_Conf_1.000	427	0							
Locus_18167_Transcript_1/1_Conf_1.000	167	0							
Locus_18168_Transcript_1/1_Conf_1.000	341	7	9.71E-26	CAX65089.1	78	119.783	91	71	C. elegans protein Y54G11A.17, confirmed by transcript evidence
Locus_18169_Transcript_1/1_Conf_1.000	184	0							
Locus_1817_Transcript_1/2_Conf_1.000	475	0							
Locus_1817_Transcript_2/2_Conf_1.000	478	0							
Locus_18170_Transcript_1/1_Conf_1.000	299	20	9.23E-32	NP_502747.1	78	139.813	99	78	hypothetical protein F55B11.1
Locus_18171_Transcript_1/1_Conf_1.000	961	20	1.79E-71	XP_002638881.1	64	273.863	319	205	C. briggsae CBR-VPS-34 protein
Locus_18172_Transcript_1/1_Conf_1.000	150	0							
Locus_18173_Transcript_1/1_Conf_1.000	413	20	7.67E-55	AAT76298.1	94	216.468	115	109	FMRFamide-related peptide FLP-18 precursor
Locus_18174_Transcript_1/1_Conf_1.000	169	0							
Locus_18175_Transcript_1/1_Conf_1.000	336	20	2.03E-31	NP_496483.1	79	138.658	110	87	hypothetical protein F07A11.5
Locus_18176_Transcript_1/1_Conf_1.000	144	0							
Locus_18177_Transcript_1/1_Conf_1.000	186	20	2.17E-12	XP_002635781.1	78	75.485	61	48	Hypothetical protein CBG10437
Locus_18178_Transcript_1/1_Conf_1.000	190	0							

Locus_18179_Transcript_1/1_Conf_1.000	289	20	9.08E-11	XP_001898051.1	61	70.0922	94	58	Ran-binding protein 7
Locus_1818_Transcript_1/1_Conf_1.000	3002	20	0	AAO85274.1	86	983.4	658	570	chondroitin disaccharide polymerase
Locus_18180_Transcript_1/1_Conf_1.000	192	0							
Locus_18181_Transcript_1/1_Conf_1.000	128	2	1.13E-05	NP_498470.1	76	53.1434	42	32	hypothetical protein F37A4.5
Locus_18182_Transcript_1/1_Conf_1.000	314	0							
Locus_18183_Transcript_1/1_Conf_1.000	157	0							
Locus_18184_Transcript_1/1_Conf_1.000	340	0							
Locus_18185_Transcript_1/1_Conf_1.000	152	0							
Locus_18186_Transcript_1/1_Conf_1.000	278	0							
Locus_18187_Transcript_1/1_Conf_1.000	460	2	5.73E-10	XP_002642255.1	71	67.3958	56	40	C. briggsae CBR-FLP-15 protein
Locus_18188_Transcript_1/1_Conf_1.000	132	0							
Locus_18189_Transcript_1/1_Conf_1.000	428	20	5.06E-30	NP_506393.1	66	134.035	145	96	hypothetical protein T01D3.5
Locus_1819_Transcript_1/1_Conf_1.000	3580	20	0	NP_508280.1	87	1718.75	1112	972	hypothetical protein D1005.1
Locus_18190_Transcript_1/1_Conf_1.000	155	0							
Locus_18191_Transcript_1/1_Conf_1.000	157	0							
Locus_18192_Transcript_1/1_Conf_1.000	336	0							

Locus_18193_Transcript_1/1_Conf_1.000	251	20	1.90E-13	XP_002635372.1	84	78.9518	52	44	C. briggsae CBR-RPM-1 protein
Locus_18194_Transcript_1/1_Conf_1.000	229	4	3.93E-06	EFO14601.1	72	54.6842	47	34	hypothetical protein LOAG_13915
Locus_18195_Transcript_1/1_Conf_1.000	151	0							
Locus_18196_Transcript_1/1_Conf_1.000	232	7	7.61E-18	CAX65068.1	73	93.5893	76	56	C. elegans protein K08C7.3d, confirmed by transcript evidence
Locus_18197_Transcript_1/1_Conf_1.000	246	4	8.82E-27	XP_002647365.1	85	123.25	82	70	C. briggsae CBR-SNT-5 protein
Locus_18198_Transcript_1/1_Conf_1.000	722	1	3.72E-08	NP_494776.2	54	62.7734	113	62	hypothetical protein B0286.1
Locus_18199_Transcript_1/1_Conf_1.000	308	0							
Locus_182_Transcript_1/1_Conf_1.000	498	20	1.25E-73	XP_002634875.1	88	278.87	166	147	Hypothetical protein CBG10541
Locus_1820_Transcript_1/1_Conf_1.000	603	20	3.10E-59	NP_492480.1	76	231.876	195	149	hypothetical protein F26H9.4
Locus_18200_Transcript_1/1_Conf_1.000	314	1	1.28E-04	XP_002642931.1	60	49.6766	70	42	C. briggsae CBR-ALH-11 protein
Locus_18201_Transcript_1/1_Conf_1.000	201	20	5.42E-24	NP_004533.1	100	114.005	50	50	NADH dehydrogenase ubiquinone 1 alpha subcomplex subunit 3
Locus_18202_Transcript_1/1_Conf_1.000	162	0							

Locus_18203_Transcript_1/1_Conf_1.000	243	20	1.41E-32	NP_001023645.1	92	142.51	80	74	AKT kinase family member (akt-1)
Locus_18204_Transcript_1/1_Conf_1.000	488	20	1.81E-43	XP_001896664.1	75	178.718	162	122	Protein kinase domain containing protein
Locus_18205_Transcript_1/1_Conf_1.000	272	0							
Locus_18206_Transcript_1/1_Conf_1.000	164	0							
Locus_18207_Transcript_1/1_Conf_1.000	853	20	6.05E-25	NP_510852.1	62	119.013	156	97	hypothetical protein T23E7.2
Locus_18208_Transcript_1/1_Conf_1.000	1381	0							
Locus_18209_Transcript_1/1_Conf_1.000	148	0							
Locus_1821_Transcript_1/1_Conf_1.000	1478	9	1.05E-48	NP_493601.2	46	199.134	515	238	FERM domain (protein4.1-ezrin-radixin-moesin) family member (frm-1)
Locus_18210_Transcript_1/1_Conf_1.000	237	20	7.82E-15	NP_491437.1	69	83.5741	79	55	UDP-GlucuronosylTransferase family member (ugt-28)
Locus_18211_Transcript_1/1_Conf_1.000	136	0							
Locus_18212_Transcript_1/1_Conf_1.000	420	20	6.47E-17	AAD13339.1	50	90.5077	126	63	ancylostoma-secreted protein 1 precursor
Locus_18213_Transcript_1/1_Conf_1.000	483	20	9.27E-48	XP_001897051.1	73	192.971	160	118	RNA polymerase Rpb1, domain 1 family protein
Locus_18214_Transcript_1/1_Conf_1.000	184	0							
Locus_18215_Transcript_1/1_Conf_1.000	135	0							
Locus_18216_Transcript_1/1_Conf_1.000	144	0							

Locus_18217_Transcript_1/1_Conf_1.000	425	20	2.85E-65	NP_502218.1	96	251.136	141	136	hypothetical protein T22B3.2
Locus_18218_Transcript_1/1_Conf_1.000	377	20	2.98E-30	XP_002641557.1	79	134.806	93	74	C. briggsae CBR-PRK-2 protein
Locus_18219_Transcript_1/1_Conf_1.000	503	20	5.41E-72	XP_002646621.1	89	273.478	160	143	Hypothetical protein CBG20508
Locus_1822_Transcript_1/1_Conf_1.000	777	20	5.22E-54	XP_001896728.1	71	215.312	224	160	Rhomboid family protein
Locus_18220_Transcript_1/1_Conf_1.000	228	0							
Locus_18221_Transcript_1/1_Conf_1.000	191	0							
Locus_18222_Transcript_1/1_Conf_1.000	435	3	3.15E-16	XP_002634303.1	62	88.1965	135	85	C. briggsae CBR-NCX-3 protein
Locus_18223_Transcript_1/1_Conf_1.000	166	0							
Locus_18224_Transcript_1/1_Conf_1.000	323	20	1.94E-21	XP_002633194.1	71	105.531	100	71	Hypothetical protein CBG05911
Locus_18225_Transcript_1/1_Conf_1.000	165	20	2.69E-15	NP_506520.1	82	85.1149	52	43	hypothetical protein T16A9.4
Locus_18226_Transcript_1/1_Conf_1.000	158	20	1.88E-24	XP_002930821.1	100	115.546	52	52	PREDICTED: splicing factor U2AF 35 kDa subunit-like
Locus_18227_Transcript_1/1_Conf_1.000	234	0							
Locus_18228_Transcript_1/2_Conf_1.000	835	20	1.12E-68	XP_783119.1	74	264.233	281	208	PREDICTED: similar to mFLJ00229 protein

Locus_18228_Transcript_2/2_Conf_1.000	835	20	1.12E-68	XP_783119.1	74	264.233	281	208	PREDICTED: similar to mFLJ00229 protein
Locus_18229_Transcript_1/1_Conf_1.000	386	0							
Locus_1823_Transcript_1/1_Conf_1.000	593	4	6.35E-30	XP_002640094.1	60	134.42	180	108	Hypothetical protein CBG12581
Locus_18230_Transcript_1/1_Conf_1.000	203	0							
Locus_18231_Transcript_1/1_Conf_1.000	288	0							
Locus_18232_Transcript_1/1_Conf_1.000	268	20	5.99E-23	NP_502118.4	71	110.538	89	64	Gon-Two Like (TRP subfamily) family member (gtl-2)
Locus_18233_Transcript_1/1_Conf_1.000	418	0							
Locus_18234_Transcript_1/1_Conf_1.000	233	20	7.09E-32	CBA11613.1	93	140.198	77	72	C. elegans protein F54F3.1c, partially confirmed by transcript evidence
Locus_18235_Transcript_1/1_Conf_1.000	380	5	2.34E-51	NP_001123175.1	87	204.912	120	105	ImmunoGlobulin-like Cell adhesion Molecule family member (igcm-3)
Locus_18236_Transcript_1/1_Conf_1.000	324	4	9.29E-16	EFO17810.1	71	86.6557	108	77	hypothetical protein LOAG_10687
Locus_18237_Transcript_1/1_Conf_1.000	146	2	1.67E-04	AAC31568.1	60	49.2914	48	29	putative zinc metallopeptidase
Locus_18238_Transcript_1/1_Conf_1.000	492	20	3.72E-41	NP_494918.2	66	171.014	168	111	hypothetical protein T27F7.3
Locus_18239_Transcript_1/1_Conf_1.000	203	20	9.87E-26	EDL12480.1	96	119.783	63	61	eukaryotic translation initiation factor 5A, isoform CRA_d
Locus_1824_Transcript_1/2_Conf_1.000	343	0							

Locus_1824_Transcript_2/2_Conf_1.000	341	0							
Locus_18240_Transcript_1/1_Conf_1.000	734	5	1.32E-24	NP_505973.1	46	117.472	263	123	hypothetical protein F55A11.7
Locus_18241_Transcript_1/1_Conf_1.000	199	0							
Locus_18242_Transcript_1/1_Conf_1.000	339	20	1.27E-17	XP_002632168.1	66	92.8189	112	74	C. briggsae CBR-PQN-85 protein
Locus_18243_Transcript_1/1_Conf_1.000	171	20	3.17E-08	XP_002633325.1	94	61.6178	35	33	Hypothetical protein CBG06062
Locus_18244_Transcript_1/1_Conf_1.000	312	0							
Locus_18245_Transcript_1/1_Conf_1.000	136	0							
Locus_18246_Transcript_1/1_Conf_1.000	480	5	7.00E-08	EFO27664.1	42	60.4622	137	58	BUB protein kinase
Locus_18247_Transcript_1/1_Conf_1.000	323	3	2.71E-23	NP_507767.2	71	111.694	105	75	hypothetical protein C14B4.2
Locus_18248_Transcript_1/1_Conf_1.000	176	20	3.16E-08	EFO19751.1	79	61.6178	48	38	prefoldin subunit 4
Locus_18249_Transcript_1/1_Conf_1.000	275	1	1.25E-04	XP_001901066.1	72	49.6766	40	29	Alpha-catulin
Locus_1825_Transcript_1/1_Conf_1.000	393	20	5.47E-40	ADD10740.1	100	167.162	81	81	ribosomal protein S26

Locus_18250_Transcript_1/1_Conf_1.000	137	0							
Locus_18251_Transcript_1/1_Conf_1.000	102	0							
Locus_18252_Transcript_1/1_Conf_1.000	455	20	1.16E-34	XP_001950864.1	72	149.443	152	110	PREDICTED: similar to werner syndrome helicase
Locus_18253_Transcript_1/1_Conf_1.000	632	2	1.98E-06	NP_506680.1	86	56.6102	37	32	HIstone H1 Like family member (hil-1)
Locus_18254_Transcript_1/1_Conf_1.000	391	2	2.25E-06	XP_002641416.1	53	55.4546	92	49	C. briggsae CBR-SBP-1 protein
Locus_18255_Transcript_1/1_Conf_1.000	305	20	2.48E-45	NP_509268.1	93	184.882	101	94	hypothetical protein F48E3.3
Locus_18256_Transcript_1/1_Conf_1.000	174	0							
Locus_18257_Transcript_1/1_Conf_1.000	211	0							
Locus_18258_Transcript_1/1_Conf_1.000	421	20	1.10E-40	NP_491776.1	85	169.474	119	102	hypothetical protein T09B4.7
Locus_18259_Transcript_1/1_Conf_1.000	692	20	2.68E-45	NP_500997.1	64	186.037	234	150	hypothetical protein C01G5.8
Locus_1826_Transcript_1/1_Conf_1.000	366	20	3.10E-48	CAA21573.2	96	194.512	106	102	C. elegans protein Y106G6H.3, confirmed by transcript evidence
Locus_18260_Transcript_1/1_Conf_1.000	318	0							
Locus_18261_Transcript_1/1_Conf_1.000	612	20	2.74E-50	XP_001891673.1	69	202.216	207	143	hypothetical protein Bm1_00760
Locus_18262_Transcript_1/1_Conf_1.000	514	2	3.24E-06	NP_001021459.2	46	55.0694	135	63	hypothetical protein F36H2.3
Locus_18263_Transcript_1/1_Conf_1.000	616	0							
Locus_18264_Transcript_1/1_Conf_1.000	177	0							
Locus_18265_Transcript_1/1_Conf_1.000	206	20	7.28E-29	EFO28029.1	95	130.183	68	65	AP-1 complex subunit mu-1
Locus_18266_Transcript_1/1_Conf_1.000	293	20	2.54E-21	NP_497959.1	78	105.145	95	75	hypothetical protein T04A8.6
Locus_18267_Transcript_1/1_Conf_1.000	189	0							
Locus_18268_Transcript_1/1_Conf_1.000	299	0							
Locus_18269_Transcript_1/1_Conf_1.000	694	20	3.75E-71	A8XYX2.2	74	271.937	223	166	Cap-specific mRNA (nucleoside-2'-O-)-methyltransferase 1A

Locus_1827_Transcript_1/1_Conf_1.000	1883	20	0	NP_499065.1	83	726.087	483	404	hypothetical protein C38C10.2
Locus_18270_Transcript_1/1_Conf_1.000	176	0							
Locus_18271_Transcript_1/1_Conf_1.000	1567	10	2.31E-25	XP_002632478.1	51	121.709	267	137	Hypothetical protein CBG13713
Locus_18272_Transcript_1/1_Conf_1.000	316	0							
Locus_18273_Transcript_1/1_Conf_1.000	397	0							
Locus_18274_Transcript_1/1_Conf_1.000	379	4	6.65E-14	NP_498113.1	73	80.4925	64	47	hypothetical protein C34E10.11
Locus_18275_Transcript_1/1_Conf_1.000	162	20	8.99E-11	XP_002634244.1	98	70.0922	54	53	C. briggsae CBR-LEV-1 protein
Locus_18276_Transcript_1/1_Conf_1.000	219	20	8.21E-36	CAR63550.1	97	153.295	72	70	putative Mediator of RNA polymerase II transcription subunit 6
Locus_18277_Transcript_1/1_Conf_1.000	296	20	4.63E-07	EFO20608.1	66	57.7658	72	48	ran-binding protein 7

Locus_18278_Transcript_1/1_Conf_1.000	201	20	3.39E-26	XP_002633097.1	95	121.324	66	63	C. briggsae CBR-RPC-1 protein
Locus_18279_Transcript_1/1_Conf_1.000	302	0							
Locus_1828_Transcript_1/1_Conf_1.000	1144	20	8.91E-71	NP_491332.1	66	271.937	355	237	NeurABin family member (nab-1)
Locus_18280_Transcript_1/1_Conf_1.000	297	0							
Locus_18281_Transcript_1/1_Conf_1.000	241	5	1.74E-06	XP_001900528.1	71	55.8398	60	43	GRIP domain containing protein
Locus_18282_Transcript_1/1_Conf_1.000	208	1	1.16E-10	NP_492417.2	72	69.707	54	39	Fatty Acid SyNthase family member (fasn-1)
Locus_18283_Transcript_1/1_Conf_1.000	318	20	2.99E-38	XP_002632600.1	87	161.384	105	92	Hypothetical protein CBG21499
Locus_18284_Transcript_1/1_Conf_1.000	129	0							
Locus_18285_Transcript_1/1_Conf_1.000	144	20	1.37E-14	XP_625155.1	82	82.8037	47	39	PREDICTED: similar to WD repeat domain 19, partial
Locus_18286_Transcript_1/2_Conf_1.000	868	20	3.63E-57	XP_002645108.1	76	226.098	165	126	Hypothetical protein CBG16797
Locus_18286_Transcript_2/2_Conf_1.000	725	20	2.56E-57	XP_002645108.1	76	226.098	165	126	Hypothetical protein CBG16797
Locus_18287_Transcript_1/1_Conf_1.000	141	0							
Locus_18288_Transcript_1/1_Conf_1.000	171	0							
Locus_18289_Transcript_1/1_Conf_1.000	243	0							
Locus_1829_Transcript_1/1_Conf_1.000	244	20	3.85E-14	NP_001033569.1	66	81.2629	81	54	SULfate Permease family member (sulp-7)
Locus_18290_Transcript_1/1_Conf_1.000	402	2	8.41E-09	EFO20920.1	52	63.5438	113	59	hypothetical protein LOAG_07566
Locus_18291_Transcript_1/1_Conf_1.000	993	20	1.49E-108	XP_002634112.1	76	397.127	317	244	C. briggsae CBR-COGC-8 protein
Locus_18292_Transcript_1/1_Conf_1.000	609	20	6.90E-46	XP_002646253.1	66	187.578	201	133	C. briggsae CBR-PRG-1 protein
Locus_18293_Transcript_1/1_Conf_1.000	345	0							
Locus_18294_Transcript_1/1_Conf_1.000	394	0							
Locus_18295_Transcript_1/1_Conf_1.000	154	0							
Locus_18296_Transcript_1/1_Conf_1.000	189	0							
Locus_18297_Transcript_1/1_Conf_1.000	460	20	9.35E-45	XP_002635106.1	78	182.956	136	107	C. briggsae CBR-TAG-225 protein
Locus_18298_Transcript_1/1_Conf_1.000	252	20	3.82E-25	EFO16377.1	76	117.857	82	63	hypothetical protein LOAG_12131
Locus_18299_Transcript_1/1_Conf_1.000	266	0							

Locus_183_Transcript_1/1_Conf_1.000	441	20	1.18E-26		70	122.865	148	104	hypothetical protein C52B9.7 - Caenorhabditis elegans
Locus_1830_Transcript_1/1_Conf_1.000	1067	4	2.00E-29	NP_491642.2	52	134.42	235	123	hypothetical protein F46F11.9
Locus_18300_Transcript_1/1_Conf_1.000	337	20	6.54E-22	AAG15121.1	66	107.071	108	72	nuclear receptor NHR-22
Locus_18301_Transcript_1/1_Conf_1.000	201	0							
Locus_18302_Transcript_1/1_Conf_1.000	389	0							
Locus_18303_Transcript_1/1_Conf_1.000	603	20	8.76E-46	XP_002644506.1	61	187.193	200	122	Hypothetical protein CBG14396
Locus_18304_Transcript_1/1_Conf_1.000	163	0							
Locus_18305_Transcript_1/1_Conf_1.000	219	0							
Locus_18306_Transcript_1/1_Conf_1.000	705	20	1.43E-33	XP_002639559.1	78	147.132	114	90	C. briggsae CBR-MEK-2 protein
Locus_18307_Transcript_1/1_Conf_1.000	263	0							
Locus_18308_Transcript_1/1_Conf_1.000	692	8	2.07E-13	XP_002636857.1	51	80.1073	201	103	Hypothetical protein CBG09313
Locus_18309_Transcript_1/1_Conf_1.000	283	0							
Locus_1831_Transcript_1/1_Conf_1.000	664	0							
Locus_18310_Transcript_1/1_Conf_1.000	239	3	6.01E-07	NP_741458.2	61	57.3806	60	37	P38 Map Kinase family member (pmk-2)
Locus_18311_Transcript_1/1_Conf_1.000	290	0							
Locus_18312_Transcript_1/1_Conf_1.000	365	20	8.86E-11	XP_002611624.1	63	70.0922	76	48	hypothetical protein BRAFLDRAFT_117138
Locus_18313_Transcript_1/1_Conf_1.000	427	20	1.91E-61	AAK62032.1	85	238.424	141	121	metalloprotease 1 precursor

Locus_18314_Transcript_1/1_Conf_1.000	339	20	8.55E-06	YP_001805161.1	59	53.5286	84	50	hypothetical protein cce_3747
Locus_18315_Transcript_1/1_Conf_1.000	447	20	2.14E-12	NP_495978.1	72	75.485	68	49	Temporarily Assigned Gene name family member (tag-165)
Locus_18316_Transcript_1/1_Conf_1.000	669	20	7.45E-58	NP_502862.1	73	227.639	197	144	hypothetical protein Y73F8A.26
Locus_18317_Transcript_1/2_Conf_1.000	431	20	8.58E-38	NP_505382.2	69	159.844	143	99	hypothetical protein C24B5.4
Locus_18317_Transcript_2/2_Conf_1.000	431	20	1.01E-38	XP_002637502.1	68	162.925	143	98	Hypothetical protein CBG19223
Locus_18318_Transcript_1/1_Conf_1.000	319	20	4.96E-17	NP_497641.2	67	90.8929	92	62	CaDHerin family member (cdh-1)
Locus_18319_Transcript_1/1_Conf_1.000	247	0							
Locus_1832_Transcript_1/1_Conf_1.000	225	1	5.70E-05	XP_002156800.1	71	50.8322	38	27	PREDICTED: similar to tyrosyl-DNA phodphodiesterase 1
Locus_18320_Transcript_1/1_Conf_1.000	231	0							
Locus_18321_Transcript_1/1_Conf_1.000	1602	7	3.02E-12	XP_002640169.1	35	78.1814	452	162	Hypothetical protein CBG12671

Locus_18322_Transcript_1/1_Conf_1.000	197	20	2.45E-16	XP_002631921.1	97	88.5817	46	45	Hypothetical protein CBG07909
Locus_18323_Transcript_1/1_Conf_1.000	378	20	1.16E-42	NP_001022488.1	92	176.022	103	95	UNCoordinated family member (unc-52)
Locus_18324_Transcript_1/1_Conf_1.000	146	0							
Locus_18325_Transcript_1/1_Conf_1.000	255	0							
Locus_18326_Transcript_1/1_Conf_1.000	139	2	9.48E-05	CAD20812.1	63	50.0618	44	28	SXC2 protein
Locus_18327_Transcript_1/1_Conf_1.000	400	20	2.01E-18	XP_001900715.1	67	95.5153	121	82	hypothetical protein
Locus_18328_Transcript_1/1_Conf_1.000	174	20	8.56E-22	XP_002919926.1	100	106.686	50	50	PREDICTED: hypothetical protein LOC100478075
Locus_18329_Transcript_1/1_Conf_1.000	128	0							
Locus_1833_Transcript_1/1_Conf_1.000	1541	4	6.63E-09	NP_491929.1	41	67.0106	275	115	Suppressor with Morphological effect on Genitalia family member (smg-5)
Locus_18330_Transcript_1/1_Conf_1.000	380	20	5.59E-37	XP_002629723.1	78	157.147	118	93	C. briggsae CBR-UGT-58 protein
Locus_18331_Transcript_1/1_Conf_1.000	195	8	1.44E-08	NP_492568.1	72	62.7734	50	36	Nuclear Pore complex Protein family member (npp-14)
Locus_18332_Transcript_1/1_Conf_1.000	330	20	2.84E-49	P01023.3	100	197.978	99	99	Alpha-2-macroglobulin
Locus_18333_Transcript_1/1_Conf_1.000	334	0							
Locus_18334_Transcript_1/1_Conf_1.000	794	0							
Locus_18335_Transcript_1/1_Conf_1.000	155	0							
Locus_18336_Transcript_1/1_Conf_1.000	241	20	3.60E-36	BAI46776.1	100	154.451	80	80	keratin 8
Locus_18337_Transcript_1/1_Conf_1.000	590	0							

Locus_18338_Transcript_1/1_Conf_1.000	436	20	6.97E-32	NP_500551.2	64	140.198	148	96	hypothetical protein F55F10.1
Locus_18339_Transcript_1/1_Conf_1.000	228	20	1.18E-10	NP_500215.2	63	69.707	71	45	hypothetical protein Y69A2AR.17
Locus_1834_Transcript_1/1_Conf_1.000	254	0							
Locus_18340_Transcript_1/1_Conf_1.000	208	1	1.98E-18	XP_001142623.1	100	95.5153	43	43	PREDICTED: similar to lysyl hydroxylase isoform 5
Locus_18341_Transcript_1/1_Conf_1.000	271	0							
Locus_18342_Transcript_1/1_Conf_1.000	167	0							
Locus_18343_Transcript_1/1_Conf_1.000	229	0							
Locus_18344_Transcript_1/1_Conf_1.000	155	0							
Locus_18345_Transcript_1/1_Conf_1.000	250	0							
Locus_18346_Transcript_1/1_Conf_1.000	237	0							
Locus_18347_Transcript_1/1_Conf_1.000	328	0							
Locus_18348_Transcript_1/1_Conf_1.000	354	0							
Locus_18349_Transcript_1/1_Conf_1.000	260	0							
Locus_1835_Transcript_1/3_Conf_0.714	1177	20	7.73E-25	XP_001892966.1	64	119.398	155	100	KH domain containing protein
Locus_1835_Transcript_2/3_Conf_0.714	1342	20	2.08E-24	XP_001892966.1	69	118.242	135	94	KH domain containing protein

Locus_1835_Transcript_3/3_Conf_0.714	1336	20	9.28E-25	XP_001892966.1	64	119.398	155	100	KH domain containing protein
Locus_18350_Transcript_1/1_Conf_1.000	171	20	6.35E-25	XP_002801692.1	98	117.087	56	55	PREDICTED: hypothetical protein LOC100430805
Locus_18351_Transcript_1/1_Conf_1.000	268	20	2.61E-18	NP_507972.1	63	95.1301	93	59	hypothetical protein F26F2.7
Locus_18352_Transcript_1/1_Conf_1.000	134	0							
Locus_18353_Transcript_1/1_Conf_1.000	249	20	2.16E-33	XP_002630855.1	92	145.206	83	77	Hypothetical protein CBG02570
Locus_18354_Transcript_1/1_Conf_1.000	693	20	9.47E-99	BAA08309.1	91	363.614	231	212	ryanodine receptor
Locus_18355_Transcript_1/1_Conf_1.000	163	20	1.03E-14	NP_508515.2	87	83.1889	47	41	AQuaPorin or aquaglyceroporin related family member (aqp-7)
Locus_18356_Transcript_1/1_Conf_1.000	375	20	1.06E-35	XP_001899245.1	98	152.91	76	75	Dumpy : shorter than wild-type protein 23, isoform b
Locus_18357_Transcript_1/1_Conf_1.000	158	0							
Locus_18358_Transcript_1/1_Conf_1.000	272	0							

Locus_18359_Transcript_1/1_Conf_1.000	238	20	7.04E-32	XP_002189607.1	93	140.198	78	73	PREDICTED: similar to cytochrome b5 reductase 3
Locus_1836_Transcript_1/1_Conf_1.000	950	20	2.40E-52	EFO25070.1	61	210.305	281	172	protein-L-isoaspartate O-methyltransferase domain-containing protein 2
Locus_18360_Transcript_1/1_Conf_1.000	523	7	7.33E-65	ABO36652.1	84	249.98	177	149	latrophilin-like protein 2
Locus_18361_Transcript_1/2_Conf_1.000	442	4	2.23E-09	XP_002631099.1	59	65.4698	82	49	C. briggsae CBR-OOC-3 protein
Locus_18361_Transcript_2/2_Conf_1.000	442	4	2.92E-09	XP_002631099.1	59	65.0846	82	49	C. briggsae CBR-OOC-3 protein
Locus_18362_Transcript_1/1_Conf_1.000	169	0							
Locus_18363_Transcript_1/1_Conf_1.000	157	0							
Locus_18364_Transcript_1/1_Conf_1.000	284	0							
Locus_18365_Transcript_1/1_Conf_1.000	348	20	8.13E-28	XP_002633875.1	66	126.716	113	75	C. briggsae CBR-FEM-1 protein
Locus_18366_Transcript_1/1_Conf_1.000	204	0							
Locus_18367_Transcript_1/1_Conf_1.000	632	20	1.45E-49	NP_001021522.1	74	199.904	161	120	hypothetical protein F59C6.12
Locus_18368_Transcript_1/1_Conf_1.000	534	5	6.05E-09	NP_505360.3	49	64.3142	177	87	hypothetical protein F21C10.9
Locus_18369_Transcript_1/1_Conf_1.000	188	5	1.04E-06	NP_496976.2	59	56.6102	62	37	hypothetical protein Y54G11A.7
Locus_1837_Transcript_1/2_Conf_1.000	2063	20	1.46E-158	NP_505864.1	72	564.688	475	344	GLYcosylation related family member (gly-20)
Locus_1837_Transcript_2/2_Conf_1.000	2063	20	1.46E-158	NP_505864.1	72	564.688	475	344	GLYcosylation related family member (gly-20)

Locus_18370_Transcript_1/1_Conf_1.000	406	20	3.70E-65	NP_495706.2	92	250.751	135	125	hypothetical protein F10B5.8
Locus_18371_Transcript_1/1_Conf_1.000	276	11	8.01E-20	ADD13547.1	78	100.138	93	73	Inositol triphosphate receptor protein 1, isoform h
Locus_18372_Transcript_1/1_Conf_1.000	484	20	7.85E-47	NP_502633.1	74	189.889	155	115	UDP-GlucuronosylTransferase family member (ugt-22)
Locus_18373_Transcript_1/1_Conf_1.000	154	20	6.70E-22	DAA31575.1	100	107.071	51	51	cold shock domain containing E1, RNA-binding
Locus_18374_Transcript_1/1_Conf_1.000	396	0							
Locus_18375_Transcript_1/1_Conf_1.000	234	20	1.21E-15	EFO20381.1	70	86.2705	74	52	ZIP Zinc transporter
Locus_18376_Transcript_1/1_Conf_1.000	169	2	1.43E-08	NP_494782.1	76	62.7734	52	40	hypothetical protein ZK430.1
Locus_18377_Transcript_1/1_Conf_1.000	151	0							
Locus_18378_Transcript_1/1_Conf_1.000	137	0							
Locus_18379_Transcript_1/1_Conf_1.000	242	20	5.56E-29	XP_002640509.1	92	130.568	79	73	C. briggsae CBR-RGA-2 protein
Locus_1838_Transcript_1/1_Conf_1.000	490	20	7.83E-23	XP_002643840.1	77	110.153	105	81	Hypothetical protein CBG02064
Locus_18380_Transcript_1/2_Conf_1.000	329	0							
Locus_18380_Transcript_2/2_Conf_1.000	345	0							
Locus_18381_Transcript_1/1_Conf_1.000	189	0							
Locus_18382_Transcript_1/1_Conf_1.000	491	20	9.51E-45	NP_508165.2	72	182.956	160	116	hypothetical protein F39H12.3
Locus_18383_Transcript_1/1_Conf_1.000	316	20	2.22E-49	XP_001166832.1	100	198.364	98	98	PREDICTED: similar to interferon-gamma isoform 2
Locus_18384_Transcript_1/1_Conf_1.000	375	1	1.30E-09	EFO24319.1	63	66.2402	74	47	hypothetical protein LOAG_04165

Locus_18385_Transcript_1/1_Conf_1.000	425	20	5.07E-38	XP_002633709.1	71	160.614	138	98	Hypothetical protein CBG03392
Locus_18386_Transcript_1/1_Conf_1.000	313	0							
Locus_18387_Transcript_1/1_Conf_1.000	149	0							
Locus_18388_Transcript_1/1_Conf_1.000	491	0							
Locus_18389_Transcript_1/1_Conf_1.000	194	0							
Locus_1839_Transcript_1/1_Conf_1.000	791	20	1.50E-80	NP_001122411.1	82	303.523	234	194	SCP(Small C-terminal domain Phosphatase)-Like phosphatase family member (scpl-1)
Locus_18390_Transcript_1/1_Conf_1.000	320	0							
Locus_18391_Transcript_1/1_Conf_1.000	708	20	7.46E-123	NP_501709.2	97	443.736	235	230	Membrane Calcium ATPase family member (mca-1)
Locus_18392_Transcript_1/1_Conf_1.000	206	0							
Locus_18393_Transcript_1/1_Conf_1.000	548	8	6.68E-54	NP_499047.1	96	213.772	105	101	FOrm-homology and ZInc finger domains family member (fozi-1)
Locus_18394_Transcript_1/1_Conf_1.000	214	20	8.25E-25	NP_510672.2	88	116.701	71	63	Nematode AStacin protease family member (nas-39)
Locus_18395_Transcript_1/1_Conf_1.000	128	0							
Locus_18396_Transcript_1/1_Conf_1.000	139	0							
Locus_18397_Transcript_1/1_Conf_1.000	252	6	3.04E-06	XP_002634634.1	54	55.0694	70	38	Hypothetical protein CBG18497
Locus_18398_Transcript_1/1_Conf_1.000	265	4	4.94E-09	XP_002639468.1	80	64.3142	46	37	Hypothetical protein CBG04063
Locus_18399_Transcript_1/1_Conf_1.000	148	20	7.99E-15	NP_498248.2	91	83.5741	47	43	hypothetical protein Y37B11A.2

Locus_184_Transcript_1/2_Conf_1.000	2838	20	0	NP_500815.2	55	671.774	916	506	Receptor Mediated Endocytosis family member (rme-2)
Locus_184_Transcript_2/2_Conf_1.000	2784	20	0	NP_500815.2	54	676.781	924	508	Receptor Mediated Endocytosis family member (rme-2)
Locus_1840_Transcript_1/1_Conf_1.000	1042	20	1.55E-79	XP_002646388.1	81	300.827	243	198	Hypothetical protein CBG15355
Locus_18400_Transcript_1/1_Conf_1.000	236	0							
Locus_18401_Transcript_1/1_Conf_1.000	236	20	2.53E-13	NP_506352.1	61	78.5666	77	47	TransThyretin-Related family domain family member (ttr-44)
Locus_18402_Transcript_1/1_Conf_1.000	387	0							
Locus_18403_Transcript_1/1_Conf_1.000	133	17	1.02E-06	NP_001024294.1	79	56.6102	44	35	hypothetical protein Y97E10AR.2
Locus_18404_Transcript_1/1_Conf_1.000	215	0							
Locus_18405_Transcript_1/3_Conf_0.625	370	0							
Locus_18405_Transcript_2/3_Conf_0.250	547	4	3.04E-06	XP_392462.3	58	55.4546	129	76	PREDICTED: similar to Stretchin-Mlck CG18255-PA, isoform A
Locus_18405_Transcript_3/3_Conf_0.625	502	20	1.45E-08	XP_392462.3	54	62.7734	164	90	PREDICTED: similar to Stretchin-Mlck CG18255-PA, isoform A
Locus_18406_Transcript_1/1_Conf_1.000	142	1	1.61E-04	AAO63576.1	67	49.2914	46	31	secreted protein 4 precursor
Locus_18407_Transcript_1/1_Conf_1.000	343	0							
Locus_18408_Transcript_1/1_Conf_1.000	435	5	8.24E-33	NP_501257.1	90	143.28	99	90	hypothetical protein F33D4.5

Locus_18409_Transcript_1/1_Conf_1.000	327	4	8.94E-19	NP_492524.2	72	96.6709	79	57	hypothetical protein F30A10.10
Locus_1841_Transcript_1/1_Conf_1.000	909	20	3.90E-73	AAV38629.1	100	279.256	138	138	cellular retinoic acid binding protein 2
Locus_18410_Transcript_1/1_Conf_1.000	203	0							
Locus_18411_Transcript_1/1_Conf_1.000	393	0							
Locus_18412_Transcript_1/1_Conf_1.000	130	20	1.57E-15	XP_575592.2	100	85.8853	43	43	PREDICTED: poly(rC) binding protein 1-like
Locus_18413_Transcript_1/1_Conf_1.000	360	3	8.61E-06	CBW44362.1	51	53.5286	129	67	C. elegans protein C33A11.4b, partially confirmed by transcript evidence
Locus_18414_Transcript_1/1_Conf_1.000	257	0							
Locus_18415_Transcript_1/1_Conf_1.000	230	0							
Locus_18416_Transcript_1/1_Conf_1.000	376	3	4.45E-34	NP_499680.1	83	147.517	98	82	UNCoordinated family member (unc-71)
Locus_18417_Transcript_1/1_Conf_1.000	638	0							
Locus_18418_Transcript_1/1_Conf_1.000	511	20	3.23E-43	XP_001901812.1	66	177.948	177	118	RNase3 domain containing protein
Locus_18419_Transcript_1/1_Conf_1.000	712	8	2.86E-05	XP_002169885.1	42	53.1434	202	85	PREDICTED: similar to cytosolic beta-N-acetylglucosaminidase
Locus_1842_Transcript_1/2_Conf_1.000	1016	20	7.76E-52	NP_491159.2	72	208.764	172	125	hypothetical protein K03E5.1
Locus_1842_Transcript_2/2_Conf_1.000	1553	20	8.45E-105	NP_491159.2	72	385.571	320	231	hypothetical protein K03E5.1

Locus_18420_Transcript_1/1_Conf_1.000	344	20	2.89E-49	EFO18549.1	82	197.978	113	93	hypothetical protein LOAG_09946
Locus_18421_Transcript_1/1_Conf_1.000	473	0							
Locus_18422_Transcript_1/1_Conf_1.000	616	20	1.03E-36	EFO23679.1	73	157.147	186	137	hypothetical protein LOAG_04806
Locus_18423_Transcript_1/1_Conf_1.000	764								
Locus_18424_Transcript_1/1_Conf_1.000	305	6	7.01E-32	NP_500982.2	83	140.198	95	79	hypothetical protein Y73B6BL.24
Locus_18425_Transcript_1/1_Conf_1.000	298	0							
Locus_18426_Transcript_1/1_Conf_1.000	693	20	2.00E-48	XP_002631178.1	60	196.438	246	149	C. briggsae CBR-MAT-2 protein
Locus_18427_Transcript_1/1_Conf_1.000	254	0							
Locus_18428_Transcript_1/1_Conf_1.000	1002	20	1.47E-71	CAA22108.2	64	274.248	335	217	C. elegans protein Y75B8A.24, partially confirmed by transcript evidence
Locus_18429_Transcript_1/1_Conf_1.000	409	2	1.33E-06	XP_002632194.1	61	56.225	70	43	Hypothetical protein CBG07060
Locus_1843_Transcript_1/1_Conf_1.000	159	0							
Locus_18430_Transcript_1/1_Conf_1.000	375	2	1.76E-06	NP_497334.1	50	55.8398	114	57	INverted Formin/formin Three-related family member (inft-1)
Locus_18431_Transcript_1/1_Conf_1.000	387	0							
Locus_18432_Transcript_1/1_Conf_1.000	148	0							
Locus_18433_Transcript_1/1_Conf_1.000	233	0							
Locus_18434_Transcript_1/1_Conf_1.000	211	2	2.77E-04	XP_001892872.1	53	48.521	62	33	hypothetical protein Bm1_07015
Locus_18435_Transcript_1/1_Conf_1.000	444	20	1.33E-54	NP_495436.1	88	215.698	144	127	SQuashed Vulva family member (sqv-7)
Locus_18436_Transcript_1/1_Conf_1.000	132	0							

Locus_18437_Transcript_1/1_Conf_1.000	342	20	5.13E-30	AAP36875.1	100	134.035	83	83	Homo sapiens apolipoprotein C-I
Locus_18438_Transcript_1/1_Conf_1.000	439	0							
Locus_18439_Transcript_1/1_Conf_1.000	432	6	1.62E-12		53	75.8702	142	76	hypothetical protein T28B8.4
Locus_1844_Transcript_1/1_Conf_1.000	530	6	1.88E-63	ABS50363.1	90	245.358	175	159	LIM domain protein variant
Locus_18440_Transcript_1/1_Conf_1.000	211	3	8.31E-09	XP_001901164.1	63	63.5438	69	44	hypothetical protein Bm1_48510
Locus_18441_Transcript_1/1_Conf_1.000	158	0							
Locus_18442_Transcript_1/1_Conf_1.000	177	0							
Locus_18443_Transcript_1/1_Conf_1.000	167	0							
Locus_18444_Transcript_1/1_Conf_1.000	493	0							
Locus_18445_Transcript_1/1_Conf_1.000	196	20	1.82E-19	EFO20545.1	94	98.9821	52	49	beta-centractin
Locus_18446_Transcript_1/1_Conf_1.000	288	20	1.34E-38	XP_002640011.1	90	162.54	95	86	Hypothetical protein CBG12481
Locus_18447_Transcript_1/1_Conf_1.000	237	0							
Locus_18448_Transcript_1/1_Conf_1.000	288	20	4.06E-27	XP_002646691.1	85	124.405	87	74	Hypothetical protein CBG13068
Locus_18449_Transcript_1/1_Conf_1.000	155	0							
Locus_1845_Transcript_1/1_Conf_1.000	1282	20	1.08E-99	NP_499274.3	67	368.237	386	259	Connector/eNhancer of KSR family member (cnk-1)
Locus_18450_Transcript_1/1_Conf_1.000	272	5	6.38E-25	NP_504292.2	84	117.087	88	74	hypothetical protein C37H5.5
Locus_18451_Transcript_1/1_Conf_1.000	298	4	7.34E-13	XP_002631639.1	67	77.0258	58	39	Hypothetical protein CBG20828
Locus_18452_Transcript_1/1_Conf_1.000	136	20	1.73E-06	XP_786652.1	77	55.8398	44	34	PREDICTED: similar to ENSANGP00000028235
Locus_18453_Transcript_1/1_Conf_1.000	147	0							

Locus_18454_Transcript_1/1_Conf_1.000	345	0							
Locus_18455_Transcript_1/1_Conf_1.000	584	2	7.58E-20	EFO20351.1	56	100.908	179	102	phosphatidylinositol 3
Locus_18456_Transcript_1/1_Conf_1.000	800	20	9.75E-43	NP_498636.2	66	177.948	248	165	hypothetical protein T20B12.1
Locus_18457_Transcript_1/1_Conf_1.000	245	0							
Locus_18458_Transcript_1/1_Conf_1.000	143	20	1.19E-07	NP_491930.1	77	59.6918	48	37	DiHydroOrotate Dehydrogenas family member (dhod-1)
Locus_18459_Transcript_1/1_Conf_1.000	328	20	1.34E-14	NP_498936.1	95	82.8037	42	40	hypothetical protein K02D10.1
Locus_1846_Transcript_1/1_Conf_1.000	1645	20	3.33E-139	NP_508777.1	73	499.975	438	324	hypothetical protein C07A12.7
Locus_18460_Transcript_1/1_Conf_1.000	149	0							
Locus_18461_Transcript_1/1_Conf_1.000	283	2	4.25E-08	NP_001022400.1	56	61.2326	81	46	Polarity and Osmotic sensitivity Defect family member (pod-2)
Locus_18462_Transcript_1/1_Conf_1.000	397	20	8.98E-43	XP_002636807.1	76	176.407	131	100	Hypothetical protein CBG09249
Locus_18463_Transcript_1/1_Conf_1.000	306	0							
Locus_18464_Transcript_1/1_Conf_1.000	434	20	2.50E-13	CAR63705.1	100	78.5666	38	38	putative CREB Homolog family member
Locus_18465_Transcript_1/1_Conf_1.000	551	20	3.07E-38	XP_002631239.1	71	161.77	179	128	C. briggsae CBR-LACT-3 protein
Locus_18466_Transcript_1/1_Conf_1.000	200	4	3.31E-05	CBL87058.1	57	51.6026	61	35	C. elegans protein T23F1.7c, partially confirmed by transcript evidence
Locus_18467_Transcript_1/1_Conf_1.000	155	0							

Locus_18468_Transcript_1/1_Conf_1.000	576	20	4.26E-60	CAA72916.2	77	234.572	188	145	serine protease inhibitor-like protein
Locus_18469_Transcript_1/1_Conf_1.000	162	20	7.61E-10	EFO26418.1	78	67.0106	50	39	hypothetical protein LOAG_02063
Locus_1847_Transcript_10/11_Conf_0.481	1586	20	0	BAG64279.1	94	862.448	480	453	unnamed protein product
Locus_1847_Transcript_11/11_Conf_0.556	1275	20	0	BAG62883.1	97	729.169	381	373	unnamed protein product
Locus_1847_Transcript_2/11_Conf_0.444	1251	20	0	AAB21181.1	97	701.049	372	364	immunoglobulin heavy chain
Locus_1847_Transcript_3/11_Conf_0.296	521	20	4.05E-76	AAO17822.1	91	287.345	173	159	anti-rabies SOJB immunoglobulin heavy chain
Locus_1847_Transcript_4/11_Conf_0.259	801	20	4.02E-129	ACN59874.1	99	464.922	222	221	chimeric anti-human type VII collagen immunoglobulin G1
Locus_1847_Transcript_5/11_Conf_0.556	1297	20	0	BAG62883.1	97	729.169	381	373	unnamed protein product
Locus_1847_Transcript_6/11_Conf_0.074	355	20	6.33E-57	ABI74358.1	91	223.402	121	111	immunoglobulin heavy chain variable region
Locus_1847_Transcript_7/11_Conf_0.074	316	20	3.20E-48	AAM75843.1	91	194.512	109	100	immunoglobulin heavy chain variable region

Locus_1847_Transcript_8/11_Conf_0.481	1266	20	0	BAG62883.1	95	691.804	381	362	unnamed protein product
Locus_1847_Transcript_9/11_Conf_0.481	1263	20	0	BAG62883.1	92	649.432	381	353	unnamed protein product
Locus_18470_Transcript_1/1_Conf_1.000	170	0							
Locus_18471_Transcript_1/1_Conf_1.000	306	0							
Locus_18472_Transcript_1/1_Conf_1.000	600	18	2.71E-07	XP_002631917.1	82	59.3066	46	38	Hypothetical protein CBG07905
Locus_18473_Transcript_1/1_Conf_1.000	312	0							
Locus_18474_Transcript_1/1_Conf_1.000	268	2	1.64E-04	NP_491537.1	55	49.2914	76	42	HoloCentric chromosome binding Protein family member (hcp-6)
Locus_18475_Transcript_1/1_Conf_1.000	189	0							
Locus_18476_Transcript_1/1_Conf_1.000	227	0							
Locus_18477_Transcript_1/1_Conf_1.000	1197	20	1.96E-124	NP_001129829.1	74	450.284	367	275	hypothetical protein Y46G5A.19
Locus_18478_Transcript_1/1_Conf_1.000	146	0							
Locus_18479_Transcript_1/1_Conf_1.000	431	0							
Locus_1848_Transcript_1/1_Conf_1.000	1393	20	1.12E-121	NP_490872.1	70	441.425	393	277	BT (Bacillus thuringiensis) toxin REsistant family member (bre-4)
Locus_18480_Transcript_1/1_Conf_1.000	350	20	6.87E-35	BAD88409.1	81	150.214	116	94	multidrug resistance-associated protein
Locus_18481_Transcript_1/1_Conf_1.000	159	20	7.12E-24	XP_001113553.2	100	113.62	52	52	PREDICTED: complement factor B isoform 1
Locus_18482_Transcript_1/1_Conf_1.000	410	1	9.20E-16	CAR63626.1	67	86.6557	77	52	hypothetical protein
Locus_18483_Transcript_1/1_Conf_1.000	1244	20	1.74E-163	NP_001122531.1	80	580.096	415	334	hypothetical protein Y105E8A.23

Locus_18484_Transcript_1/1_Conf_1.000	153	1	6.30E-04	XP_002634303.1	71	47.3654	52	37	C. briggsae CBR-NCX-3 protein
Locus_18485_Transcript_1/1_Conf_1.000	470	20	2.68E-39	NP_499652.1	76	164.851	131	100	hypothetical protein Y111B2A.20
Locus_18486_Transcript_1/1_Conf_1.000	271	3	1.58E-07	XP_002640845.1	55	59.3066	80	44	C. briggsae CBR-CDH-1 protein
Locus_18487_Transcript_1/1_Conf_1.000	128	0							
Locus_18488_Transcript_1/1_Conf_1.000	422	0							
Locus_18489_Transcript_1/1_Conf_1.000	234	20	2.88E-25	NP_494775.1	86	118.242	67	58	ForKHead transcription factor family member (fkh-6)
Locus_1849_Transcript_1/1_Conf_1.000	273	20	3.48E-31	XP_002412610.1	80	137.887	90	72	sorbitol dehydrogenase, putative

Locus_18490_Transcript_1/1_Conf_1.000	342	20	4.49E-34	XP_002641355.1	78	147.517	114	89	C. briggsae CBR-GLY-9 protein
Locus_18491_Transcript_1/1_Conf_1.000	491	20	2.25E-70	NP_496487.1	85	268.085	163	139	hypothetical protein ZK20.4
Locus_18492_Transcript_1/1_Conf_1.000	195	0							
Locus_18493_Transcript_1/1_Conf_1.000	317	7	2.82E-20	XP_002633023.1	78	101.679	74	58	Hypothetical protein CBG21797
Locus_18494_Transcript_1/2_Conf_1.000	416	0							
Locus_18494_Transcript_2/2_Conf_1.000	412	0							
Locus_18495_Transcript_1/1_Conf_1.000	243	0							
Locus_18496_Transcript_1/1_Conf_1.000	402	9	5.63E-13	NP_509538.1	75	77.411	66	50	Apical Junction Molecule family member (ajm-1)
Locus_18497_Transcript_1/1_Conf_1.000	254	0							
Locus_18498_Transcript_1/1_Conf_1.000	334	20	5.35E-40	XP_001894893.1	82	167.162	111	92	MiaB-like tRNA modifying enzyme, archaeal-type family protein
Locus_18499_Transcript_1/1_Conf_1.000	171	0							
Locus_185_Transcript_1/1_Conf_1.000	326	20	1.30E-17	XP_002645122.1	82	92.8189	74	61	Hypothetical protein CBG16816
Locus_1850_Transcript_1/1_Conf_1.000	773	20	6.28E-52	NP_505642.1	59	208.379	250	148	LYSozyme family member (lys-1)
Locus_18500_Transcript_1/1_Conf_1.000	204	0							
Locus_18501_Transcript_1/1_Conf_1.000	367	0							
Locus_18502_Transcript_1/1_Conf_1.000	452	20	7.75E-47	XP_002631100.1	85	189.889	124	106	Hypothetical protein CBG02874
Locus_18503_Transcript_1/1_Conf_1.000	229	0							
Locus_18504_Transcript_1/1_Conf_1.000	442	18	5.46E-32	NP_001023978.1	85	140.584	101	86	hypothetical protein F55C10.5
Locus_18505_Transcript_1/1_Conf_1.000	640	8	3.10E-87	NP_509030.2	95	325.094	169	161	hypothetical protein Y72A10A.1
Locus_18506_Transcript_1/1_Conf_1.000	203	3	4.60E-15	NP_001022279.1	88	84.3445	67	59	Serpentine Receptor, class AB (class A-like) family member (srab-14)

Locus_18507_Transcript_1/1_Conf_1.000	467	20	1.70E-17	XP_002629686.1	61	92.4337	129	79	Hypothetical protein CBG00906
Locus_18508_Transcript_1/1_Conf_1.000	783	20	1.18E-45	NP_506271.1	56	187.578	265	151	C-type LECtin family member (clec-41)
Locus_18509_Transcript_1/1_Conf_1.000	234	0							
Locus_1851_Transcript_1/1_Conf_1.000	2707	20	2.62E-166	XP_002639375.1	59	590.882	908	543	C. briggsae CBR-LET-502 protein
Locus_18510_Transcript_1/1_Conf_1.000	659	6	8.94E-16	EFO27431.1	48	87.8113	235	115	hypothetical protein LOAG_01051
Locus_18511_Transcript_1/1_Conf_1.000	506	20	2.75E-79	XP_002634766.1	87	297.745	170	149	Hypothetical protein CBG05362
Locus_18512_Transcript_1/1_Conf_1.000	148	0							
Locus_18513_Transcript_1/1_Conf_1.000	275	0							
Locus_18514_Transcript_1/1_Conf_1.000	165	20	8.39E-09	EFO16065.1	70	63.5438	51	36	asparagine synthase
Locus_18515_Transcript_1/1_Conf_1.000	765	20	3.01E-115	AAK31527.3	90	418.698	237	214	Neuronal symmetry protein 1, partially confirmed by transcript evidence
Locus_18516_Transcript_1/1_Conf_1.000	370	20	1.64E-52	NP_741809.1	87	208.764	123	108	Beta Carbonic Anhydrase family member (bca-1)
Locus_18517_Transcript_1/1_Conf_1.000	253	0							
Locus_18518_Transcript_1/1_Conf_1.000	171	20	1.73E-22	XP_002634622.1	94	108.997	57	54	Hypothetical protein CBG18478
Locus_18519_Transcript_1/1_Conf_1.000	172	0							
Locus_1852_Transcript_1/1_Conf_1.000	1393	20	0	NP_501804.1	88	647.121	395	350	Seryl tRNA Synthetase family member (srs-2)

Locus_18520_Transcript_1/1_Conf_1.000	203	20	2.36E-11	BAD92295.1	100	72.0182	52	52	HLA-DPA1 protein variant
Locus_18521_Transcript_1/1_Conf_1.000	344	20	4.34E-37	NP_495793.2	78	157.532	113	89	Gamma Butyrobetaine Hydroxylase family member (gbh-2)
Locus_18522_Transcript_1/1_Conf_1.000	275	20	3.05E-27	CAB05601.2	94	124.79	71	67	C. elegans protein R11A5.1b, partially confirmed by transcript evidence
Locus_18523_Transcript_1/1_Conf_1.000	269	20	5.60E-29	AAK62032.1	78	130.568	89	70	metalloprotease 1 precursor
Locus_18524_Transcript_1/1_Conf_1.000	255	20	3.01E-30	NP_741217.1	90	134.806	84	76	EXOSome (multiexonuclease complex) component family member (exos-9)
Locus_18525_Transcript_1/1_Conf_1.000	452	20	1.21E-07	1N0R	48	59.6918	126	61	Designed Ankyrin Repeat Protein With Four Identical Consensus Repeats
Locus_18526_Transcript_1/1_Conf_1.000	228	0							
Locus_18527_Transcript_1/1_Conf_1.000	225	0							
Locus_18528_Transcript_1/1_Conf_1.000	294	0							
Locus_18529_Transcript_1/1_Conf_1.000	525	20	2.06E-22	XP_001898275.1	51	108.997	199	102	GRIP domain containing protein
Locus_1853_Transcript_1/1_Conf_1.000	1125	20	1.04E-63	XP_001897956.1	62	248.44	311	195	UBA/TS-N domain containing protein

Locus_18530_Transcript_1/1_Conf_1.000	743	20	1.92E-87	NP_001021798.2	80	326.25	245	198	Arf-1 Guanine nucleotide Exchange Factor homolog family member (agef-1)
Locus_18531_Transcript_1/1_Conf_1.000	344	0							
Locus_18532_Transcript_1/1_Conf_1.000	695	4	7.66E-24	XP_002641244.1	53	114.775	222	119	Hypothetical protein CBG09112
Locus_18533_Transcript_1/1_Conf_1.000	469	2	9.63E-05	XP_002643723.1	62	50.0618	64	40	Hypothetical protein CBG01914
Locus_18534_Transcript_1/1_Conf_1.000	321	20	7.12E-24	NP_499704.1	66	113.62	105	70	CYtochrome P450 family member (cyp-13A11)
Locus_18535_Transcript_1/1_Conf_1.000	223	0							
Locus_18536_Transcript_1/1_Conf_1.000	889	20	2.55E-13	AAD31839.1	46	80.4925	171	80	AF132291_1ancylostoma-secreted protein 1 precursor
Locus_18537_Transcript_1/1_Conf_1.000	431	0							
Locus_18538_Transcript_1/1_Conf_1.000	403	20	1.62E-52	AAS99337.1	100	208.764	103	103	putative calmodulin isoform a
Locus_18539_Transcript_1/1_Conf_1.000	254	20	4.22E-24	CAM25761.1	100	114.39	72	72	major histocompatibility complex, class II, DP alpha 1
Locus_1854_Transcript_1/1_Conf_1.000	764	0							
Locus_18540_Transcript_1/1_Conf_1.000	292	20	3.79E-17	EFO14797.1	80	91.2781	67	54	hypothetical protein LOAG_13718
Locus_18541_Transcript_1/1_Conf_1.000	346	0							
Locus_18542_Transcript_1/1_Conf_1.000	169	20	2.87E-17	NP_001022911.1	92	91.6633	56	52	HELicase family member (hel-308)
Locus_18543_Transcript_1/1_Conf_1.000	262	20	3.00E-22	NP_499515.1	77	108.227	87	67	hypothetical protein Y41C4A.8

Locus_18544_Transcript_1/2_Conf_1.000	450	20	1.78E-51	EFO23808.1	78	205.297	151	118	hypothetical protein LOAG_04674
Locus_18544_Transcript_2/2_Conf_1.000	450	20	1.78E-51	EFO23808.1	78	205.297	151	118	hypothetical protein LOAG_04674
Locus_18545_Transcript_1/1_Conf_1.000	141	20	5.00E-14	EFO26621.1	91	80.8777	45	41	AIDA-1b

Locus_18546_Transcript_1/1_Conf_1.000	205	17	4.01E-11	CAA93884.2	64	71.2478	68	44	C. elegans protein ZK1067.2, partially confirmed by transcript evidence
Locus_18547_Transcript_1/1_Conf_1.000	179	0							
Locus_18548_Transcript_1/1_Conf_1.000	213	0							
Locus_18549_Transcript_1/1_Conf_1.000	256	0							
Locus_1855_Transcript_1/2_Conf_1.000	1479	1	1.00E-06	XP_002631725.1	42	59.6918	194	83	Hypothetical protein CBG20927
Locus_1855_Transcript_2/2_Conf_1.000	1479	1	1.00E-06	XP_002631725.1	42	59.6918	194	83	Hypothetical protein CBG20927
Locus_18550_Transcript_1/1_Conf_1.000	286	20	9.38E-24	NP_498534.2	84	113.235	70	59	ectopic membrane RuFfLes in embryo family member (rfl-1)
Locus_18551_Transcript_1/1_Conf_1.000	254	1	3.72E-04	XP_002649031.1	64	48.1358	64	41	Hypothetical protein CBG21486
Locus_18552_Transcript_1/2_Conf_1.000	242	0							
Locus_18552_Transcript_2/2_Conf_1.000	242	0							
Locus_18553_Transcript_1/1_Conf_1.000	148	0							
Locus_18554_Transcript_1/1_Conf_1.000	347	20	6.93E-19	NP_491099.1	83	97.0561	68	57	ARp2/3 compleX component family member (arx-7)
Locus_18555_Transcript_1/1_Conf_1.000	360	20	5.55E-29	EFO20693.1	71	130.568	119	85	hypothetical protein LOAG_07794
Locus_18556_Transcript_1/1_Conf_1.000	327	20	3.05E-43	XP_002798643.1	100	177.948	108	108	PREDICTED: keratin, type II cytoskeletal 8 isoform 3
Locus_18557_Transcript_1/1_Conf_1.000	418	20	7.82E-71	XP_002646356.1	96	269.626	139	134	Hypothetical protein CBG12070
Locus_18558_Transcript_1/1_Conf_1.000	131	20	1.86E-16	XP_002640864.1	100	88.9669	43	43	C. briggsae CBR-UNC-25 protein

Locus_18559_Transcript_1/1_Conf_1.000	160	0							
Locus_1856_Transcript_1/3_Conf_0.714	2144	20	0	XP_002639673.1	82	907.131	699	580	Hypothetical protein CBG12391
Locus_1856_Transcript_2/3_Conf_0.714	2144	20	0	XP_002639673.1	82	907.131	699	580	Hypothetical protein CBG12391
Locus_1856_Transcript_3/3_Conf_0.714	2144	20	0	XP_002639673.1	82	907.131	699	580	Hypothetical protein CBG12391
Locus_18560_Transcript_1/1_Conf_1.000	361	0							
Locus_18561_Transcript_1/1_Conf_1.000	811								
Locus_18562_Transcript_1/1_Conf_1.000	313	20	4.68E-15	AAD51334.1	66	84.3445	80	53	Kunitz type serine protease inhibitor
Locus_18563_Transcript_1/1_Conf_1.000	457	0							
Locus_18564_Transcript_1/1_Conf_1.000	139	2	8.57E-06	XP_002632655.1	78	53.5286	38	30	Hypothetical protein CBG21578
Locus_18565_Transcript_1/1_Conf_1.000	471	2	5.07E-14	NP_499928.1	51	80.8777	127	66	hypothetical protein Y66H1A.5
Locus_18566_Transcript_1/1_Conf_1.000	273	0							
Locus_18567_Transcript_1/1_Conf_1.000	193	2	3.11E-11	XP_002631732.1	65	71.633	64	42	Hypothetical protein CBG20934
Locus_18568_Transcript_1/1_Conf_1.000	219	5	1.55E-26	XP_002641766.1	88	122.479	71	63	Hypothetical protein CBG10108
Locus_18569_Transcript_1/1_Conf_1.000	266	0							
Locus_1857_Transcript_1/1_Conf_1.000	306	20	1.85E-16	NP_498551.3	63	88.9669	86	55	Chondroitin ProteoGlycan family member (cpg-2)
Locus_18570_Transcript_1/1_Conf_1.000	302	20	2.12E-20	XP_002635231.1	69	102.064	98	68	Hypothetical protein CBG11474
Locus_18571_Transcript_1/1_Conf_1.000	210	10	1.73E-22	NP_001129927.1	90	108.997	66	60	PhosphoLipase C family member (plc-1)
Locus_18572_Transcript_1/1_Conf_1.000	460	2	2.11E-04	NP_497502.2	50	48.9062	118	59	hypothetical protein Y48G9A.11
Locus_18573_Transcript_1/1_Conf_1.000	296	20	1.30E-25	NP_490949.3	75	119.398	94	71	ABC Transporter family member (abt-2)
Locus_18574_Transcript_1/1_Conf_1.000	257	0							
Locus_18575_Transcript_1/1_Conf_1.000	193	0							

Locus_18576_Transcript_1/1_Conf_1.000	989	20	1.40E-50	NP_503359.2	54	204.527	333	183	SQuashed Vulva family member (sqv-6)
Locus_18577_Transcript_1/1_Conf_1.000	244	0							
Locus_18578_Transcript_1/1_Conf_1.000	234	0							
Locus_18579_Transcript_1/1_Conf_1.000	752	0							
Locus_1858_Transcript_1/1_Conf_1.000	1420	20	3.88E-85	NP_496416.1	58	320.087	488	285	hypothetical protein T24F1.2
Locus_18580_Transcript_1/1_Conf_1.000	172	9	1.79E-19	CAA21636.3	94	98.9821	57	54	C. elegans protein Y38F1A.3, partially confirmed by transcript evidence
Locus_18581_Transcript_1/1_Conf_1.000	294	20	3.18E-32	NP_499664.2	79	141.354	96	76	hypothetical protein BE10.2
Locus_18582_Transcript_1/1_Conf_1.000	173	20	2.87E-09	NP_494824.1	75	65.0846	57	43	hypothetical protein T05A7.6
Locus_18583_Transcript_1/2_Conf_1.000	660	20	8.05E-17	NP_498219.1	58	91.2781	113	66	hypothetical protein F54E7.9
Locus_18583_Transcript_2/2_Conf_1.000	660	20	3.49E-20	NP_498219.1	58	102.449	113	66	hypothetical protein F54E7.9
Locus_18584_Transcript_1/1_Conf_1.000	341	20	6.96E-24	XP_002636068.1	72	113.62	98	71	Hypothetical protein CBG01308
Locus_18585_Transcript_1/1_Conf_1.000	537	0							
Locus_18586_Transcript_1/1_Conf_1.000	298	20	2.14E-12	AAM22023.2	75	75.485	60	45	L15188_2Hypothetical protein C14B9.10
Locus_18587_Transcript_1/1_Conf_1.000	1050	20	1.16E-114	XP_002645256.1	83	417.542	294	245	Hypothetical protein CBG00135
Locus_18588_Transcript_1/1_Conf_1.000	135	4	8.88E-11	XP_002643942.1	84	70.0922	45	38	C. briggsae CBR-LEV-8 protein
Locus_18589_Transcript_1/1_Conf_1.000	224	0							

Locus_1859_Transcript_1/1_Conf_1.000	1479	20	0	XP_002644843.1	90	682.559	409	369	Hypothetical protein CBG05011
Locus_18590_Transcript_1/5_Conf_0.400	203	0							
Locus_18590_Transcript_2/5_Conf_0.400	254	0							
Locus_18590_Transcript_4/5_Conf_0.600	380	0							
Locus_18590_Transcript_5/5_Conf_0.467	245	0							
Locus_18591_Transcript_1/1_Conf_1.000	132	0							
Locus_18592_Transcript_1/1_Conf_1.000	557	20	6.46E-15	XP_001989392.1	55	84.3445	126	70	GH11701
Locus_18593_Transcript_1/1_Conf_1.000	361	20	4.28E-05	NP_741539.2	50	51.2174	120	60	hypothetical protein C18C4.5
Locus_18594_Transcript_1/1_Conf_1.000	182	2	5.04E-09	AAM45145.1	86	64.3142	43	37	AF278680_1L3B25
Locus_18595_Transcript_1/1_Conf_1.000	141	3	6.76E-11	NP_510494.2	84	70.4774	44	37	hypothetical protein C18B12.6
Locus_18596_Transcript_1/1_Conf_1.000	232	0							
Locus_18597_Transcript_1/1_Conf_1.000	130	20	8.34E-17	XP_001114147.2	100	90.1225	43	43	PREDICTED: uncharacterized protein C1orf43-like isoform 3
Locus_18598_Transcript_1/1_Conf_1.000	319	20	4.34E-21	NP_001022673.1	74	104.375	105	78	NADC (Na+-coupled dicarboxylate transporter) family member (nac-3)
Locus_18599_Transcript_1/1_Conf_1.000	229	0							
Locus_186_Transcript_1/3_Conf_0.500	738	0							
Locus_186_Transcript_2/3_Conf_0.600	930	0							
Locus_186_Transcript_3/3_Conf_0.700	806	0							

Locus_1860_Transcript_1/1_Conf_1.000	647	20	4.35E-36	XP_001899156.1	75	155.221	150	113	Nuclear hormone receptor family member nhr-41
Locus_18600_Transcript_1/1_Conf_1.000	222	0							
Locus_18601_Transcript_1/1_Conf_1.000	278	2	4.27E-05	XP_002642236.1	60	51.2174	68	41	Hypothetical protein CBG18218
Locus_18602_Transcript_1/1_Conf_1.000	196	0							
Locus_18603_Transcript_1/1_Conf_1.000	213	20	1.90E-29	NP_493755.1	97	132.109	70	68	MoLTing defective family member (mlt-10)
Locus_18604_Transcript_1/1_Conf_1.000	157	20	1.26E-12	EAW97458.1	100	76.2554	36	36	decorin, isoform CRA_g
Locus_18605_Transcript_1/2_Conf_1.000	444	0							
Locus_18605_Transcript_2/2_Conf_1.000	444	0							
Locus_18606_Transcript_1/1_Conf_1.000	158	1	1.27E-04	XP_002632108.1	78	49.6766	52	41	C. briggsae CBR-PTR-3 protein
Locus_18607_Transcript_1/1_Conf_1.000	387	20	1.27E-33	XP_002630605.1	89	145.976	122	109	Hypothetical protein CBG02267
Locus_18608_Transcript_1/1_Conf_1.000	169	0							
Locus_18609_Transcript_1/1_Conf_1.000	247	0							

Locus_1861_Transcript_1/1_Conf_1.000	450	20	4.69E-52	EFO22655.1	79	207.223	134	106	hypothetical protein LOAG_05826
Locus_18610_Transcript_1/1_Conf_1.000	1013	20	1.75E-112	NP_505849.2	79	410.223	297	236	hypothetical protein T19B10.3
Locus_18611_Transcript_1/1_Conf_1.000	165	0							
Locus_18612_Transcript_1/1_Conf_1.000	224	20	2.64E-18	XP_002639616.1	86	95.1301	60	52	C. briggsae CBR-APM-1 protein
Locus_18613_Transcript_1/1_Conf_1.000	466	5	3.19E-32	XP_002634225.1	66	141.354	150	99	Hypothetical protein CBG01795
Locus_18614_Transcript_1/1_Conf_1.000	172	0							
Locus_18615_Transcript_1/1_Conf_1.000	182	0							
Locus_18616_Transcript_1/1_Conf_1.000	443	5	9.39E-08	NP_510705.1	80	60.077	40	32	hypothetical protein C10E2.6
Locus_18617_Transcript_1/1_Conf_1.000	702	20	2.95E-79	NP_001023875.1	93	298.901	175	163	hypothetical protein F32D8.14
Locus_18618_Transcript_1/2_Conf_1.000	405	0							
Locus_18618_Transcript_2/2_Conf_1.000	428	0							
Locus_18619_Transcript_1/1_Conf_1.000	340	20	1.96E-18	XP_002631544.1	54	95.5153	109	59	Hypothetical protein CBG20713
Locus_1862_Transcript_1/1_Conf_1.000	650	20	1.74E-16	XP_002608187.1	55	90.1225	157	87	hypothetical protein BRAFLDRAFT_125861
Locus_18620_Transcript_1/1_Conf_1.000	239	0							
Locus_18621_Transcript_1/1_Conf_1.000	136	0							
Locus_18622_Transcript_1/1_Conf_1.000	273	0							
Locus_18623_Transcript_1/1_Conf_1.000	146	0							
Locus_18624_Transcript_1/1_Conf_1.000	274	20	5.20E-27	NP_499949.2	90	124.02	90	81	Gut on EXterior family member (gex-2)

Locus_18625_Transcript_1/1_Conf_1.000	224	0							
Locus_18626_Transcript_1/1_Conf_1.000	138	4	5.02E-14	NP_492772.1	93	80.8777	45	42	SET (trithorax/polycomb) domain containing family member (set-18)
Locus_18627_Transcript_1/1_Conf_1.000	369	20	2.08E-39	NP_499603.1	83	165.236	115	96	Protein PHosphatase family member (pph-4.1)
Locus_18628_Transcript_1/1_Conf_1.000	464	20	1.64E-44	NP_496483.1	73	182.185	154	113	hypothetical protein F07A11.5
Locus_18629_Transcript_1/1_Conf_1.000	189	0							
Locus_1863_Transcript_1/1_Conf_1.000	1423	20	2.56E-36	XP_001891682.1	50	157.918	392	198	MRG family protein
Locus_18630_Transcript_1/1_Conf_1.000	143	0							
Locus_18631_Transcript_1/1_Conf_1.000	330	20	1.25E-12	EFA06146.1	59	76.2554	96	57	hypothetical protein TcasGA2_TC008990
Locus_18632_Transcript_1/1_Conf_1.000	319	2	2.81E-20	NP_001024902.1	68	101.679	95	65	hypothetical protein T10B10.4
Locus_18633_Transcript_1/1_Conf_1.000	529	20	1.00E-48	NP_492169.2	72	196.438	161	117	TAF (TBP-associated transcription factor) family member (taf-5)
Locus_18634_Transcript_1/1_Conf_1.000	244	0							
Locus_18635_Transcript_1/1_Conf_1.000	144	20	1.12E-16	NP_492775.2	85	89.7373	47	40	LAMinin related. See also lmb- family member (lam-3)
Locus_18636_Transcript_1/1_Conf_1.000	136	0							
Locus_18637_Transcript_1/1_Conf_1.000	130	0							
Locus_18638_Transcript_1/1_Conf_1.000	134	0							
Locus_18639_Transcript_1/1_Conf_1.000	227	0							

Locus_1864_Transcript_1/1_Conf_1.000	1321	20	8.83E-20	XP_002639715.1	61	102.834	129	79	Hypothetical protein CBG12437
Locus_18640_Transcript_1/1_Conf_1.000	304	20	1.37E-35	NP_510447.1	87	152.525	100	87	hypothetical protein C05G5.1
Locus_18641_Transcript_1/1_Conf_1.000	318	7	3.53E-39	XP_002645055.1	80	164.466	106	85	Hypothetical protein CBG16715
Locus_18642_Transcript_1/1_Conf_1.000	130	0							
Locus_18643_Transcript_1/1_Conf_1.000	341	20	2.09E-36	NP_506047.5	79	155.221	113	90	abnormal CHEmotaxis family member (che-11)
Locus_18644_Transcript_1/1_Conf_1.000	171	0							
Locus_18645_Transcript_1/1_Conf_1.000	427	0							
Locus_18646_Transcript_1/1_Conf_1.000	874	0							
Locus_18647_Transcript_1/1_Conf_1.000	473	20	1.12E-37	EFO25106.1	66	159.458	156	104	hypothetical protein LOAG_03373
Locus_18648_Transcript_1/1_Conf_1.000	123	0							
Locus_18649_Transcript_1/1_Conf_1.000	233	0							
Locus_1865_Transcript_1/2_Conf_0.500	1726	20	1.21E-38	CAR63549.1	86	166.007	99	86	hypothetical protein
Locus_1865_Transcript_2/2_Conf_0.750	2307	20	6.91E-88	CAR63549.1	80	330.102	231	185	hypothetical protein
Locus_18650_Transcript_1/1_Conf_1.000	162	0							
Locus_18651_Transcript_1/1_Conf_1.000	143	0							
Locus_18652_Transcript_1/1_Conf_1.000	492	20	4.40E-42	NP_495617.1	73	174.096	156	114	hypothetical protein B0495.2
Locus_18653_Transcript_1/1_Conf_1.000	244	0							
Locus_18654_Transcript_1/1_Conf_1.000	342	2	4.98E-17	XP_002633839.1	61	90.8929	106	65	Hypothetical protein CBG19878
Locus_18655_Transcript_1/1_Conf_1.000	930	20	3.13E-49	EFO16234.1	60	199.904	303	183	pold2-prov protein
Locus_18656_Transcript_1/1_Conf_1.000	252	20	2.74E-15	AAV88076.1	69	85.1149	78	54	putative retrotransposon polyprotein
Locus_18657_Transcript_1/1_Conf_1.000	258	1	1.15E-05	XP_002646401.1	59	53.1434	86	51	C. briggsae CBR-DYS-1 protein
Locus_18658_Transcript_1/1_Conf_1.000	206	4	3.64E-04	XP_002636748.1	73	48.1358	46	34	Hypothetical protein CBG23475
Locus_18659_Transcript_1/1_Conf_1.000	142	0							

Locus_1866_Transcript_1/2_Conf_1.000	1271	3	1.25E-07	AAO33571.1	50	62.3882	109	55	Thr-Ser protein
Locus_1866_Transcript_2/2_Conf_1.000	1208	3	1.17E-07	AAO33571.1	50	62.3882	109	55	Thr-Ser protein
Locus_18660_Transcript_1/1_Conf_1.000	360	20	1.20E-15	NP_491604.3	56	86.2705	119	67	hypothetical protein T05E8.3
Locus_18661_Transcript_1/1_Conf_1.000	425	0							
Locus_18662_Transcript_1/1_Conf_1.000	190	20	9.36E-24	CAB55030.2	91	113.235	61	56	C. elegans protein Y57A10A.10, partially confirmed by transcript evidence
Locus_18663_Transcript_1/1_Conf_1.000	254	0							
Locus_18664_Transcript_1/1_Conf_1.000	522	4	5.33E-15	NP_001041216.1	59	84.3445	104	62	hypothetical protein C05D9.9
Locus_18665_Transcript_1/1_Conf_1.000	129	0							
Locus_18666_Transcript_1/1_Conf_1.000	219	0							
Locus_18667_Transcript_1/1_Conf_1.000	571	5	5.95E-59	NP_001122951.1	85	230.72	157	135	Rod-like lethal, DYe-filling defective family member (rdy-2)
Locus_18668_Transcript_1/1_Conf_1.000	146	0							
Locus_18669_Transcript_1/1_Conf_1.000	479	20	5.51E-45	XP_002637016.1	75	183.726	141	107	C. briggsae CBR-TTR-27 protein
Locus_1867_Transcript_1/2_Conf_1.000	304	2	2.05E-07	XP_002645104.1	58	58.9214	55	32	Hypothetical protein CBG16791
Locus_1867_Transcript_2/2_Conf_1.000	482	2	2.03E-15	XP_002645104.1	67	85.5001	100	67	Hypothetical protein CBG16791
Locus_18670_Transcript_1/1_Conf_1.000	487	5	3.66E-12	NP_741607.1	51	74.7146	140	72	hypothetical protein T07C12.12
Locus_18671_Transcript_1/1_Conf_1.000	155	11	3.56E-15	XP_002631133.1	84	84.7297	50	42	C. briggsae CBR-CLH-2 protein
Locus_18672_Transcript_1/1_Conf_1.000	217	0							
Locus_18673_Transcript_1/1_Conf_1.000	160	0							
Locus_18674_Transcript_1/1_Conf_1.000	488	1	6.26E-04	XP_001551642.1	47	47.3654	109	52	predicted protein
Locus_18675_Transcript_1/1_Conf_1.000	129	4	2.28E-06	NP_506824.1	76	55.4546	39	30	hypothetical protein T06E6.1
Locus_18676_Transcript_1/1_Conf_1.000	312	20	3.68E-36	XP_002634196.1	87	154.451	99	87	Hypothetical protein CBG01765
Locus_18677_Transcript_1/1_Conf_1.000	416	20	7.38E-58	XP_002641543.1	85	226.483	138	118	Hypothetical protein CBG09842
Locus_18678_Transcript_1/1_Conf_1.000	231	0							
Locus_18679_Transcript_1/1_Conf_1.000	549	0							

Locus_1868_Transcript_1/2_Conf_1.000	1785	20	4.00E-85	CAC42336.2	65	320.472	360	234	C. elegans protein T06E6.2b, confirmed by transcript evidence
Locus_1868_Transcript_2/2_Conf_1.000	1785	20	4.01E-85	CAC42336.2	65	320.472	360	234	C. elegans protein T06E6.2b, confirmed by transcript evidence
Locus_18680_Transcript_1/1_Conf_1.000	476	0							
Locus_18681_Transcript_1/1_Conf_1.000	235	2	2.98E-22	XP_002632209.1	91	108.227	61	56	Hypothetical protein CBG07076
Locus_18682_Transcript_1/1_Conf_1.000	1030	20	1.48E-90	NP_505918.2	72	337.421	309	224	Tubulin Tyrosine Ligase Like family member (ttll-5)
Locus_18683_Transcript_1/1_Conf_1.000	463	3	1.19E-10	EFO18993.1	63	69.707	103	65	hypothetical protein LOAG_09502
Locus_18684_Transcript_1/1_Conf_1.000	241	20	1.79E-27	XP_001901477.1	85	125.561	78	67	Homeobox domain containing protein
Locus_18685_Transcript_1/1_Conf_1.000	226	0							
Locus_18686_Transcript_1/1_Conf_1.000	257	0							
Locus_18687_Transcript_1/1_Conf_1.000	277	0							
Locus_18688_Transcript_1/1_Conf_1.000	166	20	1.25E-20	EFO23546.1	98	102.834	52	51	PDZ domain-containing protein
Locus_18689_Transcript_1/1_Conf_1.000	321	0							
Locus_1869_Transcript_1/2_Conf_1.000	1835	20	2.71E-52	AAC72298.1	51	211.46	479	245	reverse transcriptase
Locus_1869_Transcript_2/2_Conf_1.000	292	1	7.69E-11	ZP_06734949.1	88	63.929	54	48	hypothetical protein NEIELOOT_01783
Locus_18690_Transcript_1/1_Conf_1.000	168	0							
Locus_18691_Transcript_1/1_Conf_1.000	499	20	1.78E-19	EFN65839.1	57	98.9821	149	85	Craniofacial development protein 2
Locus_18692_Transcript_1/1_Conf_1.000	341	0							
Locus_18693_Transcript_1/1_Conf_1.000	172	0							

Locus_18694_Transcript_1/1_Conf_1.000	137	20	1.01E-14	BAB33325.1	82	83.1889	45	37	KIAA1655 protein
Locus_18695_Transcript_1/1_Conf_1.000	201	2	9.61E-05	XP_002630279.1	61	50.0618	62	38	Hypothetical protein CBG00710
Locus_18696_Transcript_1/1_Conf_1.000	547	20	3.56E-39	XP_001902364.1	65	164.851	182	120	Myotubularin-related family protein
Locus_18697_Transcript_1/1_Conf_1.000	446	20	1.75E-30	XP_002631488.1	63	135.576	152	97	C. briggsae CBR-PTR-8 protein
Locus_18698_Transcript_1/1_Conf_1.000	174	0							
Locus_18699_Transcript_1/1_Conf_1.000	262	0							
Locus_187_Transcript_1/1_Conf_1.000	1760	20	1.83E-66	XP_002638761.1	86	258.455	207	180	C. briggsae CBR-MEX-3 protein
Locus_1870_Transcript_1/1_Conf_1.000	289	20	1.44E-32	NP_741546.3	86	142.51	94	81	PAPS (adenosine 3'-phosphate 5'-phosphosulfate) Transporter family member (pst-1)
Locus_18700_Transcript_1/1_Conf_1.000	256	9	4.51E-18	NP_498134.1	72	94.3597	83	60	hypothetical protein F52C9.7

Locus_18701_Transcript_1/1_Conf_1.000	197	20	1.17E-34	3MQL	100	149.443	65	65	CrystalStructure OfThe Fibronectin 6fni1-2fnii7fni Fragment
Locus_18702_Transcript_1/1_Conf_1.000	215	0							
Locus_18703_Transcript_1/1_Conf_1.000	129	0							
Locus_18704_Transcript_1/1_Conf_1.000	604	20	1.41E-11	NP_001121593.1	47	73.559	184	87	sinc finger protein Ci-ZF(C2H2)-148
Locus_18705_Transcript_1/1_Conf_1.000	410	20	1.05E-27	XP_002635372.1	66	126.331	137	91	C. briggsae CBR-RPM-1 protein
Locus_18706_Transcript_1/1_Conf_1.000	539	0							
Locus_18707_Transcript_1/1_Conf_1.000	619	20	4.07E-49	AAF98588.2	67	198.364	167	112	Trehalase protein 2, confirmed by transcript evidence
Locus_18708_Transcript_1/1_Conf_1.000	217	20	2.93E-14	XP_002640651.1	83	81.6481	60	50	Hypothetical protein CBG19709
Locus_18709_Transcript_1/1_Conf_1.000	374	0							
Locus_1871_Transcript_1/1_Conf_1.000	1713	20	0	NP_510705.1	78	666.766	563	441	hypothetical protein C10E2.6
Locus_18710_Transcript_1/1_Conf_1.000	415	20	7.16E-45	XP_002633760.1	82	183.341	121	100	C. briggsae CBR-GON-1 protein
Locus_18711_Transcript_1/1_Conf_1.000	326	4	2.06E-31	XP_002640784.1	76	138.658	101	77	Hypothetical protein CBG15659
Locus_18712_Transcript_1/1_Conf_1.000	191	20	2.02E-18	NP_504228.2	88	95.5153	53	47	human GoNadotropin-Releasing hormone Receptor (GnRHR) related family member (gnrr-5)

Locus_18713_Transcript_1/1_Conf_1.000	923	20	1.15E-96	XP_002641314.1	78	357.451	292	230	Hypothetical protein CBG24614
Locus_18714_Transcript_1/1_Conf_1.000	154	0							
Locus_18715_Transcript_1/1_Conf_1.000	139	0							
Locus_18716_Transcript_1/1_Conf_1.000	218	0							
Locus_18717_Transcript_1/1_Conf_1.000	317	20	2.47E-24	NP_509760.2	73	115.161	105	77	Temporarily Assigned Gene name family member (tag-289)
Locus_18718_Transcript_1/1_Conf_1.000	148	2	1.37E-06	XP_002640681.1	70	56.225	48	34	Hypothetical protein CBG19743
Locus_18719_Transcript_1/1_Conf_1.000	134	1	1.52E-10	EAW50568.1	100	69.3218	44	44	hCG1990620
Locus_1872_Transcript_1/1_Conf_1.000	795	20	3.24E-22	XP_001900476.1	70	109.768	104	73	DNA / pantothenate metabolism flavoprotein
Locus_18720_Transcript_1/1_Conf_1.000	184	5	4.68E-23	CAB04449.3	90	110.923	61	55	C. elegans protein F53A2.1, partially confirmed by transcript evidence
Locus_18721_Transcript_1/1_Conf_1.000	177	0							
Locus_18722_Transcript_1/1_Conf_1.000	249	2	1.51E-10	XP_001892617.1	62	69.3218	81	51	Protein kinase domain containing protein
Locus_18723_Transcript_1/1_Conf_1.000	766	9	2.80E-12	NP_499886.1	46	76.6406	187	87	hypothetical protein R02D3.7
Locus_18724_Transcript_1/1_Conf_1.000	154	20	1.65E-20	XP_002799187.1	100	102.449	51	51	PREDICTED: protein disulfide-isomerase A6 isoform 2
Locus_18725_Transcript_1/1_Conf_1.000	214	20	7.72E-23	XP_001891673.1	87	110.153	71	62	hypothetical protein Bm1_00760
Locus_18726_Transcript_1/1_Conf_1.000	158	0							

Locus_18727_Transcript_1/1_Conf_1.000	230	2	2.82E-04	XP_002641697.1	66	48.521	42	28	C. briggsae CBR-PRI-1 protein
Locus_18728_Transcript_1/1_Conf_1.000	149	0							
Locus_18729_Transcript_1/1_Conf_1.000	379	0							
Locus_1873_Transcript_1/2_Conf_1.000	1373	0							
Locus_1873_Transcript_2/2_Conf_1.000	1385	0							
Locus_18730_Transcript_1/1_Conf_1.000	176	0							
Locus_18731_Transcript_1/1_Conf_1.000	488	0							
Locus_18732_Transcript_1/1_Conf_1.000	281	0							
Locus_18733_Transcript_1/1_Conf_1.000	296	3	6.20E-36	CAR63525.1	84	153.68	98	83	hypothetical protein
Locus_18734_Transcript_1/1_Conf_1.000	1167	20	2.35E-58	NP_498041.1	57	230.72	313	181	SET (trithorax/polycomb) domain containing family member (set-2)
Locus_18735_Transcript_1/1_Conf_1.000	298	20	5.95E-47	NP_509871.2	92	190.274	98	91	Heparan SulphoTransferase family member (hst-2)
Locus_18736_Transcript_1/1_Conf_1.000	508	0							
Locus_18737_Transcript_1/1_Conf_1.000	208	20	1.47E-21	AAB65324.3	83	105.916	65	54	Hypothetical protein F40A3.5
Locus_18738_Transcript_1/1_Conf_1.000	305	20	3.86E-22	XP_002635372.1	67	107.842	106	72	C. briggsae CBR-RPM-1 protein
Locus_18739_Transcript_1/1_Conf_1.000	393	20	8.19E-28	NP_001040743.1	68	126.716	130	89	hypothetical protein C17C3.1
Locus_1874_Transcript_1/1_Conf_1.000	985	3	2.65E-17	NP_001122695.1	44	93.9745	327	146	hypothetical protein F34D10.9
Locus_18740_Transcript_1/1_Conf_1.000	172	20	2.95E-22	NP_503409.1	92	108.227	57	53	SyNapTotagmin family member (snt-5)

Locus_18741_Transcript_1/1_Conf_1.000	302	20	1.33E-30	NP_001021871.1	77	135.961	105	81	UNCoordinated family member (unc-13)
Locus_18742_Transcript_1/1_Conf_1.000	547	20	3.95E-22	NP_501018.1	62	108.227	130	81	Dicer Related Helicase family member (drh-1)
Locus_18743_Transcript_1/1_Conf_1.000	305	3	4.58E-07	NP_505360.3	54	57.7658	102	56	hypothetical protein F21C10.9
Locus_18744_Transcript_1/1_Conf_1.000	518	0							
Locus_18745_Transcript_1/1_Conf_1.000	226	0							
Locus_18746_Transcript_1/1_Conf_1.000	363	0							
Locus_18747_Transcript_1/1_Conf_1.000	242	20	6.15E-28	NP_498711.1	85	127.102	80	68	TYRosinase family member (tyr-1)
Locus_18748_Transcript_1/1_Conf_1.000	374	0							
Locus_18749_Transcript_1/1_Conf_1.000	462	20	3.67E-28	NP_491439.1	68	127.872	135	92	hypothetical protein C10H11.8
Locus_1875_Transcript_1/1_Conf_1.000	1225	20	6.90E-165	AAK28335.1	88	584.719	365	324	glycogen synthase
Locus_18750_Transcript_1/1_Conf_1.000	164	0							
Locus_18751_Transcript_1/1_Conf_1.000	194	0							
Locus_18752_Transcript_1/1_Conf_1.000	268	7	6.66E-06	XP_002644661.1	55	53.9138	88	49	Hypothetical protein CBG14642
Locus_18753_Transcript_1/1_Conf_1.000	174	0							
Locus_18754_Transcript_1/1_Conf_1.000	155	20	4.50E-18	CBI70492.1	98	94.3597	51	50	vacuolar ATPase a subunit
Locus_18755_Transcript_1/1_Conf_1.000	336	0							

Locus_18756_Transcript_1/1_Conf_1.000	497	20	2.73E-60	NP_001122508.1	85	234.572	165	141	DeHydrogenases, Short chain family member (dhs-3)
Locus_18757_Transcript_1/1_Conf_1.000	692	20	2.17E-79	XP_002633915.1	74	299.286	225	167	Hypothetical protein CBG19981
Locus_18758_Transcript_1/1_Conf_1.000	207	2	6.39E-09	XP_002645082.1	83	63.929	48	40	C. briggsae CBR-DHS-29 protein
Locus_18759_Transcript_1/1_Conf_1.000	290	2	8.23E-04	NP_001122656.1	58	46.9802	96	56	hypothetical protein Y46G5A.18
Locus_1876_Transcript_1/1_Conf_1.000	323	0							
Locus_18760_Transcript_1/1_Conf_1.000	200	16	1.48E-13	NP_490834.1	75	79.337	65	49	hypothetical protein Y48G8AL.1
Locus_18761_Transcript_1/1_Conf_1.000	595	4	1.54E-23	XP_002639320.1	60	113.235	136	82	C. briggsae CBR-DCT-9 protein
Locus_18762_Transcript_1/1_Conf_1.000	270	0							
Locus_18763_Transcript_1/1_Conf_1.000	1067	20	0	ACJ65168.1	94	653.284	354	336	Uncoordinated protein 68, isoform b, partially confirmed by transcript evidence
Locus_18764_Transcript_1/1_Conf_1.000	400	20	9.96E-18	XP_002640827.1	70	93.2041	93	66	Hypothetical protein CBG15714
Locus_18765_Transcript_1/1_Conf_1.000	224	0							
Locus_18766_Transcript_1/1_Conf_1.000	301	9	1.92E-21	NP_492082.2	69	105.531	100	69	hypothetical protein F07A5.4
Locus_18767_Transcript_1/1_Conf_1.000	288	0							
Locus_18768_Transcript_1/1_Conf_1.000	157	0							
Locus_18769_Transcript_1/1_Conf_1.000	169	0							
Locus_1877_Transcript_1/1_Conf_1.000	1054	20	4.77E-76	NP_501805.1	68	289.271	321	221	hypothetical protein F44D12.1
Locus_18770_Transcript_1/1_Conf_1.000	318	0							
Locus_18771_Transcript_1/1_Conf_1.000	157	0							
Locus_18772_Transcript_1/1_Conf_1.000	185	0							

Locus_18773_Transcript_1/1_Conf_1.000	903	20	1.04E-102	XP_002640962.1	75	377.481	294	222	Hypothetical protein CBG11705
Locus_18774_Transcript_1/1_Conf_1.000	128	4	7.35E-05	ABI52804.1	76	50.447	38	29	mitochondrial associated endoribonuclease MAR1-isochorismatase superfamily
Locus_18775_Transcript_1/1_Conf_1.000	246	2	4.26E-05	XP_002631065.1	75	51.2174	69	52	C. briggsae CBR-SFXN-1.4 protein
Locus_18776_Transcript_1/1_Conf_1.000	581	0							
Locus_18777_Transcript_1/1_Conf_1.000	204	1	8.11E-04	XP_002634811.1	52	46.9802	55	29	Hypothetical protein CBG13916
Locus_18778_Transcript_1/1_Conf_1.000	155	13	2.02E-10	AAS47831.1	78	68.9366	46	36	metalloprotease IV
Locus_18779_Transcript_1/1_Conf_1.000	315	0							
Locus_1878_Transcript_1/2_Conf_1.000	401	20	5.24E-43	XP_002637058.1	80	177.178	128	103	C. briggsae CBR-TTR-19 protein
Locus_1878_Transcript_2/2_Conf_1.000	469	20	8.05E-44	XP_002637058.1	80	179.874	130	105	C. briggsae CBR-TTR-19 protein
Locus_18780_Transcript_1/1_Conf_1.000	294	20	1.30E-17	XP_002642870.1	66	92.8189	78	52	Hypothetical protein CBG15140
Locus_18781_Transcript_1/1_Conf_1.000	144	0							
Locus_18782_Transcript_1/1_Conf_1.000	306	0							
Locus_18783_Transcript_1/1_Conf_1.000	169	20	1.09E-16	NP_510342.1	80	89.7373	56	45	hypothetical protein C33G3.4
Locus_18784_Transcript_1/1_Conf_1.000	509	20	7.77E-29	XP_002629716.1	59	130.183	170	101	Hypothetical protein CBG00946
Locus_18785_Transcript_1/1_Conf_1.000	566	20	8.95E-28	P0C5E7.1	55	127.102	195	109	Phosphatidylinositol 3-kinase age-1

Locus_18786_Transcript_1/1_Conf_1.000	373	20	1.77E-06	XP_001661184.1	73	55.8398	41	30	beta-1,3-n-acetylglucosaminyltransferase radical fringe (o-fucosylpeptide 3-beta-n-acetylglucosaminyltransferase)
Locus_18787_Transcript_1/1_Conf_1.000	180	20	5.37E-19	ACJ65168.1	100	97.4413	59	59	Uncoordinated protein 68, isoform b, partially confirmed by transcript evidence
Locus_18788_Transcript_1/1_Conf_1.000	133	20	2.19E-17	EFO20074.1	97	92.0485	43	42	KIN protein
Locus_18789_Transcript_1/1_Conf_1.000	457	3	3.03E-35	NP_496398.2	71	151.369	133	95	hypothetical protein T06D8.1
Locus_1879_Transcript_1/1_Conf_1.000	946	6	2.09E-56	XP_002636983.1	68	223.787	257	176	C. briggsae CBR-PQN-22 protein
Locus_18790_Transcript_1/1_Conf_1.000	245	0							
Locus_18791_Transcript_1/1_Conf_1.000	610	20	1.50E-24	XP_001900249.1	67	116.701	127	86	Ctr copper transporter family protein
Locus_18792_Transcript_1/1_Conf_1.000	223	0							
Locus_18793_Transcript_1/1_Conf_1.000	297	4	6.01E-15	EFO26610.1	68	83.9593	77	53	hypothetical protein LOAG_01866

Locus_18794_Transcript_1/1_Conf_1.000	369	20	6.91E-35	3O47	100	150.214	72	72	CrystalStructure Of Arfgap1-Arf1 Fusion Protein
Locus_18795_Transcript_1/1_Conf_1.000	130	0							
Locus_18796_Transcript_1/1_Conf_1.000	546	0							
Locus_18797_Transcript_1/1_Conf_1.000	181	20	7.75E-18	AAD00776.1	88	93.5893	53	47	2,5 dihydroxyphenylacetate oxidase
Locus_18798_Transcript_1/1_Conf_1.000	724	7	4.09E-55	CBA11611.1	74	218.779	246	184	C. elegans protein F53B7.2b, partially confirmed by transcript evidence
Locus_18799_Transcript_1/1_Conf_1.000	245	20	9.14E-32	EFO18852.1	93	139.813	81	76	RNA polymerase III 128kD subunit-PA
Locus_188_Transcript_1/1_Conf_1.000	311	0							
Locus_1880_Transcript_1/1_Conf_1.000	519	0							
Locus_18800_Transcript_1/1_Conf_1.000	314	4	3.14E-11	EFO28277.1	69	71.633	59	41	piwi domain-containing protein
Locus_18801_Transcript_1/1_Conf_1.000	273	20	7.29E-13	XP_002647311.1	72	77.0258	75	54	Hypothetical protein CBG06352
Locus_18802_Transcript_1/1_Conf_1.000	128	0							
Locus_18803_Transcript_1/1_Conf_1.000	205	0							
Locus_18804_Transcript_1/1_Conf_1.000	129	6	2.43E-08	NP_001076639.1	97	62.003	39	38	TWiK family of potassium channels family member (twk-39)

Locus_18805_Transcript_1/1_Conf_1.000	199	6	3.00E-06	NP_508151.1	62	55.0694	62	39	Intracellular LEctin family member (ile-2)
Locus_18806_Transcript_1/1_Conf_1.000	412	0							
Locus_18807_Transcript_1/1_Conf_1.000	843	4	1.01E-24	NP_001040707.1	59	118.242	197	118	CDT (S. pombe CDC10 Dependent Transcript) homolog family member (cdt-1)
Locus_18808_Transcript_1/1_Conf_1.000	222	20	1.54E-34	3O78	100	149.058	73	73	TheStructure Of Ca2+ Sensor (Case-12)
Locus_18809_Transcript_1/1_Conf_1.000	222	5	9.11E-11	XP_002641407.1	65	70.0922	69	45	C. briggsae CBR-SUCH-1 protein
Locus_1881_Transcript_1/2_Conf_0.750	133	0							
Locus_1881_Transcript_2/2_Conf_0.958	166	5	1.13E-05	XP_001348421.2	67	53.1434	55	37	conserved Plasmodium protein, unknown function
Locus_18810_Transcript_1/1_Conf_1.000	155	0							
Locus_18811_Transcript_1/1_Conf_1.000	361	20	1.78E-27	NP_502580.2	85	125.561	92	79	hypothetical protein Y62E10A.13
Locus_18812_Transcript_1/1_Conf_1.000	323	0							
Locus_18813_Transcript_1/1_Conf_1.000	365	20	1.76E-59	ABD77505.1	98	231.876	120	118	60S ribosomal protein L34-like protein
Locus_18814_Transcript_1/1_Conf_1.000	327	0							

Locus_18815_Transcript_1/1_Conf_1.000	375	0							
Locus_18816_Transcript_1/1_Conf_1.000	300	0							
Locus_18817_Transcript_1/1_Conf_1.000	472	20	4.40E-42	NP_497903.1	82	174.096	128	105	anNEXin family member (nex-3)
Locus_18818_Transcript_1/1_Conf_1.000	168	0							
Locus_18819_Transcript_1/1_Conf_1.000	140	0							
Locus_1882_Transcript_1/1_Conf_1.000	250	0							
Locus_18820_Transcript_1/1_Conf_1.000	164	0							
Locus_18821_Transcript_1/1_Conf_1.000	210	0							
Locus_18822_Transcript_1/1_Conf_1.000	230	8	2.63E-10	NP_502123.1	71	68.5514	60	43	hypothetical protein B0035.13
Locus_18823_Transcript_1/1_Conf_1.000	135	0							
Locus_18824_Transcript_1/1_Conf_1.000	178	0							
Locus_18825_Transcript_1/1_Conf_1.000	140	0							
Locus_18826_Transcript_1/1_Conf_1.000	189	20	3.68E-20	3ICH	100	101.293	63	63	CrystalStructure Of Cyclophilin B At 1.2 A Resolution
Locus_18827_Transcript_1/1_Conf_1.000	143	3	8.00E-04	AAB42328.2	81	46.9802	38	31	Hypothetical protein C32E8.11
Locus_18828_Transcript_1/1_Conf_1.000	768	4	3.70E-04	XP_002099137.1	41	49.6766	181	75	GE23539
Locus_18829_Transcript_1/1_Conf_1.000	378	20	7.05E-40	XP_002642051.1	74	166.777	126	94	Hypothetical protein CBG17988
Locus_1883_Transcript_1/2_Conf_1.000	2056	20	3.03E-156	NP_501180.1	70	556.984	531	375	hypothetical protein C48A7.2

Locus_1883_Transcript_2/2_Conf_1.000	1981	20	1.93E-160	NP_501180.1	74	570.852	506	375	hypothetical protein C48A7.2
Locus_18830_Transcript_1/1_Conf_1.000	154	0							
Locus_18831_Transcript_1/1_Conf_1.000	168	0							
Locus_18832_Transcript_1/1_Conf_1.000	334	0							
Locus_18833_Transcript_1/1_Conf_1.000	258	0							
Locus_18834_Transcript_1/1_Conf_1.000	180	0							
Locus_18835_Transcript_1/1_Conf_1.000	627	0							
Locus_18836_Transcript_1/1_Conf_1.000	190	0							
Locus_18837_Transcript_1/2_Conf_1.000	370	0							
Locus_18837_Transcript_2/2_Conf_1.000	369	0							
Locus_18838_Transcript_1/1_Conf_1.000	167	0							
Locus_18839_Transcript_1/1_Conf_1.000	423	0							
Locus_1884_Transcript_1/1_Conf_1.000	1248	20	1.37E-83	XP_002634010.1	57	314.694	433	248	C. briggsae CBR-RABS-5 protein
Locus_18840_Transcript_1/1_Conf_1.000	176	0							
Locus_18841_Transcript_1/1_Conf_1.000	577	20	1.04E-74	NP_491574.1	85	283.108	195	167	hypothetical protein F55A12.8
Locus_18842_Transcript_1/1_Conf_1.000	432	6	4.28E-05	AAD13339.1	52	51.2174	76	40	ancylostoma-secreted protein 1 precursor
Locus_18843_Transcript_1/1_Conf_1.000	261	6	1.60E-07	XP_002630534.1	69	59.3066	66	46	Hypothetical protein CBG12974
Locus_18844_Transcript_1/1_Conf_1.000	146	20	1.08E-11	NP_500458.1	81	73.1738	48	39	hypothetical protein F47C12.1
Locus_18845_Transcript_1/1_Conf_1.000	625	2	1.33E-15	XP_001892397.1	49	87.0409	187	93	hypothetical protein Bm1_04595
Locus_18846_Transcript_1/1_Conf_1.000	210	0							
Locus_18847_Transcript_1/1_Conf_1.000	129	0							
Locus_18848_Transcript_1/1_Conf_1.000	128	0							
Locus_18849_Transcript_1/1_Conf_1.000	148	20	1.41E-11	NP_502513.1	93	72.7886	43	40	hypothetical protein C39E9.10
Locus_1885_Transcript_1/1_Conf_1.000	626	20	3.49E-32	XP_001895135.1	62	142.124	180	112	replication factor A 73 kDa subunit
Locus_18850_Transcript_1/1_Conf_1.000	130	0							
Locus_18851_Transcript_1/1_Conf_1.000	419	20	4.17E-56	XP_001894250.1	87	220.705	139	122	Low-density lipoprotein receptor-related protein
Locus_18852_Transcript_1/1_Conf_1.000	305	4	2.05E-23	NP_001021638.1	71	112.079	101	72	hypothetical protein T23G11.6
Locus_18853_Transcript_1/1_Conf_1.000	353	0							

Locus_18854_Transcript_1/1_Conf_1.000	128	16	3.52E-07	NP_501934.3	69	58.151	42	29	hypothetical protein F01D4.3
Locus_18855_Transcript_1/1_Conf_1.000	640	20	1.09E-108	NP_491193.2	98	396.356	215	211	Calcium Channel, Beta subunit family member (ccb-1)
Locus_18856_Transcript_1/1_Conf_1.000	451	20	2.59E-26	NP_501549.1	98	121.709	65	64	RAP homolog (vertebrate Rap GTPase family) family member (rap-1)
Locus_18857_Transcript_1/1_Conf_1.000	212	10	1.33E-06	CAN86632.2	63	56.225	69	44	C. elegans protein Y48C3A.18b, confirmed by transcript evidence
Locus_18858_Transcript_1/1_Conf_1.000	437	0							
Locus_18859_Transcript_1/1_Conf_1.000	338	20	4.49E-55	AAL06640.1	95	217.238	112	107	serine-threonine protein kinase
Locus_1886_Transcript_1/1_Conf_1.000	1882	20	0	XP_002647048.1	89	956.822	596	534	Hypothetical protein CBG03566
Locus_18860_Transcript_1/1_Conf_1.000	196	4	2.72E-07	NP_001021444.1	59	58.5362	59	35	DAP (Death Associated Protein kinase) Like Kinase family member (dlk-1)
Locus_18861_Transcript_1/1_Conf_1.000	255	5	5.89E-18	NP_510539.1	80	93.9745	62	50	hypothetical protein Y15E3A.4
Locus_18862_Transcript_1/1_Conf_1.000	172	0							
Locus_18863_Transcript_1/1_Conf_1.000	349	0							

Locus_18864_Transcript_1/1_Conf_1.000	803	20	9.24E-25	XP_421314.2	51	118.242	230	118	PREDICTED: hypothetical protein
Locus_18865_Transcript_1/1_Conf_1.000	209	0							
Locus_18866_Transcript_1/2_Conf_1.000	261	0							
Locus_18866_Transcript_2/2_Conf_1.000	520	0							
Locus_18867_Transcript_1/1_Conf_1.000	390	0							
Locus_18868_Transcript_1/1_Conf_1.000	271	0							
Locus_18869_Transcript_1/1_Conf_1.000	217	0							
Locus_1887_Transcript_1/1_Conf_1.000	553	20	5.48E-59	XP_002632482.1	82	230.72	160	132	Hypothetical protein CBG13717
Locus_18870_Transcript_1/1_Conf_1.000	442	20	2.29E-38	XP_002630861.1	73	161.77	139	102	Hypothetical protein CBG02576
Locus_18871_Transcript_1/1_Conf_1.000	141	0							
Locus_18872_Transcript_1/1_Conf_1.000	265	6	8.15E-12	XP_001899774.1	76	73.559	71	54	Guanine nucleotide exchange factor for Ras-like GTPases; N-terminal motif family protein
Locus_18873_Transcript_1/1_Conf_1.000	198	0							
Locus_18874_Transcript_1/1_Conf_1.000	178	0							
Locus_18875_Transcript_1/1_Conf_1.000	163	4	7.61E-10	NP_499922.1	75	67.0106	53	40	Kinase, GLH-Binding family member (kgb-1)
Locus_18876_Transcript_1/1_Conf_1.000	450	20	1.33E-38	NP_495410.4	75	162.54	149	112	hypothetical protein C44B7.11
Locus_18877_Transcript_1/1_Conf_1.000	206	0							
Locus_18878_Transcript_1/1_Conf_1.000	253	20	3.96E-14	ACI49197.1	61	81.2629	80	49	hypothetical protein Csp3_JD03.006
Locus_18879_Transcript_1/1_Conf_1.000	180	0							
Locus_1888_Transcript_1/3_Conf_0.667	522	0							
Locus_1888_Transcript_2/3_Conf_0.667	493	0							
Locus_1888_Transcript_3/3_Conf_0.667	514	0							
Locus_18880_Transcript_1/1_Conf_1.000	361	2	3.27E-05	XP_001898007.1	69	51.6026	53	37	Origin recognition complex subunit 5
Locus_18881_Transcript_1/1_Conf_1.000	391	20	8.52E-22	XP_001898421.1	63	106.686	132	84	NG,NG-dimethylarginine dimethylaminohydrolase 1
Locus_18882_Transcript_1/1_Conf_1.000	208	0							
Locus_18883_Transcript_1/1_Conf_1.000	143	0							
Locus_18884_Transcript_1/1_Conf_1.000	228	20	6.66E-30	XP_001154014.1	97	133.65	69	67	PREDICTED: similar to COX4I1 protein isoform 1
Locus_18885_Transcript_1/1_Conf_1.000	446	2	6.25E-12	XP_002640218.1	59	73.9442	98	58	C. briggsae CBR-ZTF-4 protein

Locus_18886_Transcript_1/1_Conf_1.000	134	0							
Locus_18887_Transcript_1/1_Conf_1.000	206	0							
Locus_18888_Transcript_1/1_Conf_1.000	151	0							
Locus_18889_Transcript_1/1_Conf_1.000	283	20	5.71E-21	XP_002647214.1	71	103.99	94	67	Hypothetical protein CBG22398
Locus_1889_Transcript_1/2_Conf_1.000	1149	20	3.44E-163	XP_002643004.1	91	578.941	359	329	Hypothetical protein CBG24278
Locus_1889_Transcript_2/2_Conf_1.000	1149	20	1.70E-162	XP_002643004.1	91	576.63	359	328	Hypothetical protein CBG24278
Locus_18890_Transcript_1/1_Conf_1.000	416	20	1.27E-25	XP_002631207.1	74	119.398	123	92	Hypothetical protein CBG03003
Locus_18891_Transcript_1/1_Conf_1.000	164	5	3.19E-16	NP_496398.2	77	88.1965	54	42	hypothetical protein T06D8.1
Locus_18892_Transcript_1/1_Conf_1.000	194	0							
Locus_18893_Transcript_1/1_Conf_1.000	207	1	5.38E-24	AAQ15253.1	100	114.005	68	68	AF370417_1PP13850
Locus_18894_Transcript_1/1_Conf_1.000	275	0							
Locus_18895_Transcript_1/1_Conf_1.000	146	0							
Locus_18896_Transcript_1/1_Conf_1.000	395	5	1.23E-07	ABB53347.1	53	59.6918	86	46	secreted protein 5 precursor
Locus_18897_Transcript_1/1_Conf_1.000	264	0							
Locus_18898_Transcript_1/1_Conf_1.000	785	20	1.97E-16	NP_494956.1	49	90.5077	256	127	Serpentine Receptor, class B (beta) family member (srb-16)
Locus_18899_Transcript_1/1_Conf_1.000	192	20	4.19E-24	NP_001122009.1	85	114.39	63	54	host cell factor C1b
Locus_189_Transcript_1/3_Conf_0.750	1516	20	1.56E-95	XP_002636254.1	58	354.755	462	272	C. briggsae CBR-EGG-1 protein
Locus_189_Transcript_2/3_Conf_0.750	1748	20	1.86E-95	XP_002636254.1	58	354.755	462	272	C. briggsae CBR-EGG-1 protein
Locus_189_Transcript_3/3_Conf_0.750	1516	20	1.56E-95	XP_002636254.1	58	354.755	462	272	C. briggsae CBR-EGG-1 protein
Locus_1890_Transcript_1/1_Conf_1.000	1332	20	2.26E-156	XP_002643220.1	84	556.599	405	343	C. briggsae CBR-ARR-1 protein

Locus_18900_Transcript_1/1_Conf_1.000	290	20	3.66E-36	NP_509087.1	84	154.451	97	82	OligoPeptide Transporter family member (opt-2)
Locus_18901_Transcript_1/1_Conf_1.000	256	0							
Locus_18902_Transcript_1/1_Conf_1.000	477	20	4.23E-37	NP_495010.2	76	157.532	151	115	hypothetical protein F09E5.2
Locus_18903_Transcript_1/1_Conf_1.000	175	0							
Locus_18904_Transcript_1/1_Conf_1.000	311	20	4.68E-20	XP_002630767.1	78	100.908	83	65	C. briggsae CBR-ZYG-1 protein
Locus_18905_Transcript_1/1_Conf_1.000	296	16	1.75E-14		60	82.4185	92	56	hypothetical protein Y105C5B.d
Locus_18906_Transcript_1/1_Conf_1.000	456	0							
Locus_18907_Transcript_1/1_Conf_1.000	255	0							
Locus_18908_Transcript_1/2_Conf_1.000	966	0							
Locus_18908_Transcript_2/2_Conf_1.000	966	0							
Locus_18909_Transcript_1/1_Conf_1.000	259	1	8.22E-04	AAZ67106.1	68	46.9802	47	32	transposase
Locus_1891_Transcript_1/2_Conf_1.000	741	0							
Locus_1891_Transcript_2/2_Conf_1.000	664	0							
Locus_18910_Transcript_1/1_Conf_1.000	521	20	3.94E-63	NP_493371.1	81	244.202	170	139	hypothetical protein Y48G10A.1
Locus_18911_Transcript_1/1_Conf_1.000	208	0							
Locus_18912_Transcript_1/1_Conf_1.000	535	0							
Locus_18913_Transcript_1/1_Conf_1.000	289	0							
Locus_18914_Transcript_1/1_Conf_1.000	738	20	2.68E-57	XP_001473804.1	71	226.098	199	142	PREDICTED: similar to B cell antigen receptor Ig beta associated protein 1
Locus_18915_Transcript_1/1_Conf_1.000	162	0							
Locus_18916_Transcript_1/1_Conf_1.000	180	20	1.98E-13	Q8MNV0.2	74	78.9518	59	44	Probable spastin homolog spas-1

Locus_18917_Transcript_1/1_Conf_1.000	272	20	4.11E-48	XP_002804983.1	100	194.126	90	90	PREDICTED: isocitrate dehydrogenase
Locus_18918_Transcript_1/1_Conf_1.000	192	5	3.33E-05	EFO24938.1	62	51.6026	61	38	hypothetical protein LOAG_03545
Locus_18919_Transcript_1/1_Conf_1.000	1537	20	0	NP_495285.1	83	665.611	456	381	abnormal cell LINeage family member (lin-23)

Locus_1892_Transcript_1/1_Conf_1.000	1204	20	3.45E-52	AAC47238.1	63	210.305	323	204	non-muscle myosin heavy chain II
Locus_18920_Transcript_1/1_Conf_1.000	313	0							
Locus_18921_Transcript_1/1_Conf_1.000	131	3	1.86E-08	XP_002809210.1	80	62.3882	35	28	PREDICTED: myosin regulatory light chain 12B-like
Locus_18922_Transcript_1/1_Conf_1.000	474	3	3.63E-04	XP_001202730.1	51	48.1358	107	55	PREDICTED: similar to zinc finger protein, partial
Locus_18923_Transcript_1/1_Conf_1.000	221	0							
Locus_18924_Transcript_1/1_Conf_1.000	193	0							
Locus_18925_Transcript_1/1_Conf_1.000	222	0							
Locus_18926_Transcript_1/1_Conf_1.000	541	20	9.05E-24	NP_500084.2	83	113.62	79	66	hypothetical protein Y77E11A.2
Locus_18927_Transcript_1/1_Conf_1.000	303	0							
Locus_18928_Transcript_1/1_Conf_1.000	356	0							
Locus_18929_Transcript_1/1_Conf_1.000	447	2	1.22E-07	EFO20888.1	79	59.6918	44	35	hypothetical protein LOAG_07599
Locus_1893_Transcript_1/1_Conf_1.000	308	0							
Locus_18930_Transcript_1/1_Conf_1.000	438	0							
Locus_18931_Transcript_1/1_Conf_1.000	129	0							
Locus_18932_Transcript_1/1_Conf_1.000	274	0							
Locus_18933_Transcript_1/1_Conf_1.000	787	20	2.90E-92	NP_492031.1	82	342.428	263	217	hypothetical protein M05B5.6
Locus_18934_Transcript_1/1_Conf_1.000	280	0							

Locus_18935_Transcript_1/1_Conf_1.000	252	20	4.23E-16	CAA06162.1	82	87.8113	62	51	CB-CYP-4
Locus_18936_Transcript_1/1_Conf_1.000	136	0							
Locus_18937_Transcript_1/1_Conf_1.000	183	0							
Locus_18938_Transcript_1/1_Conf_1.000	205	5	1.92E-21	NP_506678.2	85	105.531	67	57	hypothetical protein W09D12.1
Locus_18939_Transcript_1/1_Conf_1.000	135	0							
Locus_1894_Transcript_1/1_Conf_1.000	1168	20	2.41E-95	NP_508455.2	93	353.599	197	184	hypothetical protein K02E10.1
Locus_18940_Transcript_1/1_Conf_1.000	254	9	6.98E-19	NP_493701.1	78	97.0561	60	47	hypothetical protein K01A2.5
Locus_18941_Transcript_1/1_Conf_1.000	204	0							
Locus_18942_Transcript_1/1_Conf_1.000	1232	20	9.30E-85	ABU49430.1	67	318.546	376	254	PRO-2
Locus_18943_Transcript_1/1_Conf_1.000	201	7	4.76E-12	EFO19031.1	87	74.3294	49	43	hypothetical protein LOAG_09464
Locus_18944_Transcript_1/1_Conf_1.000	578	20	2.51E-44	XP_002644494.1	67	182.185	196	133	Hypothetical protein CBG14382
Locus_18945_Transcript_1/1_Conf_1.000	690	20	3.88E-36	XP_002646140.1	76	155.606	121	92	Hypothetical protein CBG08020
Locus_18946_Transcript_1/1_Conf_1.000	329	5	2.50E-37	NP_493560.1	84	158.303	111	94	hypothetical protein K05C4.11
Locus_18947_Transcript_1/1_Conf_1.000	272	6	2.43E-16	NP_494828.1	75	88.5817	72	54	hypothetical protein T05A7.1
Locus_18948_Transcript_1/1_Conf_1.000	280	20	1.19E-15	NP_001123161.1	58	86.2705	87	51	hypothetical protein K03A11.6

Locus_18949_Transcript_1/1_Conf_1.000	331	20	2.33E-35	XP_002641709.1	80	151.754	110	88	C. briggsae CBR-CED-7 protein
Locus_1895_Transcript_1/1_Conf_1.000	623	20	3.37E-104	AAR07614.1	98	381.333	191	189	UNC-18
Locus_18950_Transcript_1/1_Conf_1.000	304	20	7.01E-32	NP_509110.1	89	140.198	76	68	TransThyretin-Related family domain family member (ttr-6)
Locus_18951_Transcript_1/1_Conf_1.000	230	17	1.77E-06	NP_001122780.1	56	55.8398	75	42	hypothetical protein F41H10.6
Locus_18952_Transcript_1/1_Conf_1.000	399	0							
Locus_18953_Transcript_1/1_Conf_1.000	155	0							
Locus_18954_Transcript_1/1_Conf_1.000	217	8	1.04E-27	CAR81372.1	91	126.331	70	64	C. elegans protein F57B7.2b, partially confirmed by transcript evidence
Locus_18955_Transcript_1/1_Conf_1.000	258	20	5.11E-30	NP_872081.1	88	134.035	84	74	Acyl CoA DeHydrogenase family member (acdh-13)
Locus_18956_Transcript_1/1_Conf_1.000	134	15	1.47E-13	NP_500715.1	88	79.337	43	38	hypothetical protein R13H9.5
Locus_18957_Transcript_1/1_Conf_1.000	549	20	3.76E-28	NP_501123.1	74	128.257	152	113	COLlagen family member (col-113)

Locus_18958_Transcript_1/1_Conf_1.000	153	20	1.15E-13	NP_001122771.1	80	79.7221	50	40	hypothetical protein F13H10.4
Locus_18959_Transcript_1/1_Conf_1.000	146	0							
Locus_1896_Transcript_1/1_Conf_1.000	793	20	3.27E-75	NP_507513.2	70	285.804	272	193	hypothetical protein E01B7.1
Locus_18960_Transcript_1/1_Conf_1.000	742	20	6.47E-43	NP_497604.2	59	178.333	254	150	SET (trithorax/polycomb) domain containing family member (set-27)
Locus_18961_Transcript_1/1_Conf_1.000	211	0							
Locus_18962_Transcript_1/1_Conf_1.000	158	0							
Locus_18963_Transcript_1/1_Conf_1.000	329	20	6.19E-20	XP_002633531.1	76	100.523	98	75	Hypothetical protein CBG05398
Locus_18964_Transcript_1/1_Conf_1.000	251	0							
Locus_18965_Transcript_1/1_Conf_1.000	173	0							
Locus_18966_Transcript_1/1_Conf_1.000	384	6	3.36E-34	ABH10656.1	92	147.902	83	77	FMRFamide-related peptide FLP-11 precursor
Locus_18967_Transcript_1/1_Conf_1.000	158	20	7.66E-10	XP_002633316.1	75	67.0106	52	39	C. briggsae CBR-GTL-2 protein
Locus_18968_Transcript_1/1_Conf_1.000	159	0							
Locus_18969_Transcript_1/1_Conf_1.000	187	0							
Locus_1897_Transcript_1/1_Conf_1.000	428	0							
Locus_18970_Transcript_1/1_Conf_1.000	480	20	3.92E-51	NP_497319.1	78	204.142	157	124	hypothetical protein Y46E12BL.2
Locus_18971_Transcript_1/1_Conf_1.000	310	0							
Locus_18972_Transcript_1/1_Conf_1.000	460	20	1.88E-61	XP_002753304.1	100	238.424	153	153	PREDICTED: 60S ribosomal protein L4-like

Locus_18973_Transcript_1/1_Conf_1.000	368	20	1.07E-32	ADK09905.1	100	142.895	122	122	non-muscle myosin IIA
Locus_18974_Transcript_1/1_Conf_1.000	289	0							
Locus_18975_Transcript_1/1_Conf_1.000	217	0							
Locus_18976_Transcript_1/1_Conf_1.000	198	0							
Locus_18977_Transcript_1/1_Conf_1.000	258	20	7.14E-32	NP_504498.2	91	140.198	81	74	hypothetical protein F25B4.7
Locus_18978_Transcript_1/1_Conf_1.000	226	0							
Locus_18979_Transcript_1/1_Conf_1.000	526	20	3.13E-79	NP_493549.1	84	297.745	175	148	hypothetical protein Y54E5A.1
Locus_1898_Transcript_1/1_Conf_1.000	1979	20	2.90E-140	NP_493982.1	75	503.827	436	327	hypothetical protein H17B01.1
Locus_18980_Transcript_1/1_Conf_1.000	132	0							
Locus_18981_Transcript_1/1_Conf_1.000	157	0							
Locus_18982_Transcript_1/1_Conf_1.000	273	0							

Locus_18983_Transcript_1/1_Conf_1.000	382	20	3.35E-66	XP_002630298.1	96	254.218	127	123	C. briggsae CBR-LTD-1 protein
Locus_18984_Transcript_1/1_Conf_1.000	153	0							
Locus_18985_Transcript_1/1_Conf_1.000	233	0							
Locus_18986_Transcript_1/1_Conf_1.000	238	2	1.02E-06	NP_506256.3	57	56.6102	77	44	CaDHerin family member (cdh-6)
Locus_18987_Transcript_1/1_Conf_1.000	129	0							
Locus_18988_Transcript_1/1_Conf_1.000	231	0							
Locus_18989_Transcript_1/1_Conf_1.000	136	20	5.56E-13	NP_501214.1	95	77.411	40	38	hypothetical protein R05G6.4
Locus_1899_Transcript_1/1_Conf_1.000	292	0							
Locus_18990_Transcript_1/1_Conf_1.000	453	20	1.46E-37	NP_508493.1	70	159.073	151	106	protein KINase family member (kin-29)
Locus_18991_Transcript_1/1_Conf_1.000	268	0							
Locus_18992_Transcript_1/1_Conf_1.000	311	0							
Locus_18993_Transcript_1/1_Conf_1.000	137	0							
Locus_18994_Transcript_1/1_Conf_1.000	196	0							
Locus_18995_Transcript_1/1_Conf_1.000	141	0							
Locus_18996_Transcript_1/1_Conf_1.000	371	7	3.23E-16	XP_002646731.1	56	88.1965	127	72	C. briggsae CBR-ADA-2 protein
Locus_18997_Transcript_1/1_Conf_1.000	365	0							
Locus_18998_Transcript_1/1_Conf_1.000	252	0							
Locus_18999_Transcript_1/1_Conf_1.000	139	0							
Locus_19_Transcript_1/1_Conf_1.000	1371	20	0	NP_498775.2	85	668.692	442	377	hypothetical protein C14B9.2
Locus_190_Transcript_1/1_Conf_1.000	356	0							
Locus_1900_Transcript_1/6_Conf_0.467	1098	20	1.17E-88	CBW38507.1	75	331.257	283	213	calcium-activated apyrase
Locus_1900_Transcript_2/6_Conf_0.600	1098	20	2.79E-90	CBW38507.1	75	336.65	283	215	calcium-activated apyrase

Locus_1900_Transcript_3/6_Conf_0.600	908	20	1.73E-89	CBW38507.1	75	333.569	283	213	calcium-activated apyrase
Locus_1900_Transcript_4/6_Conf_0.667	1098	20	2.36E-89	CBW38507.1	75	333.569	283	214	calcium-activated apyrase
Locus_1900_Transcript_5/6_Conf_0.467	1098	20	8.96E-89	CBW38507.1	75	331.643	283	213	calcium-activated apyrase
Locus_1900_Transcript_6/6_Conf_0.667	1098	20	2.36E-89	CBW38507.1	75	333.569	283	214	calcium-activated apyrase
Locus_19000_Transcript_1/1_Conf_1.000	165	20	1.34E-14	XP_002758248.1	100	82.8037	38	38	PREDICTED: calcium-regulated heat stable protein 1-like
Locus_19001_Transcript_1/1_Conf_1.000	450	20	1.29E-25	XP_002638288.1	60	119.398	149	90	Hypothetical protein CBG22835
Locus_19002_Transcript_1/1_Conf_1.000	141	0							
Locus_19003_Transcript_1/1_Conf_1.000	409	4	1.36E-43	XP_002638941.1	87	179.104	135	118	Hypothetical protein CBG22168
Locus_19004_Transcript_1/1_Conf_1.000	602	0							
Locus_19005_Transcript_1/1_Conf_1.000	283	20	3.96E-22	XP_002639142.1	77	107.842	94	73	Hypothetical protein CBG14966
Locus_19006_Transcript_1/1_Conf_1.000	158	20	1.59E-15	XP_002632333.1	94	85.8853	52	49	Hypothetical protein CBG00341

Locus_19007_Transcript_1/1_Conf_1.000	183	18	3.37E-05	YP_001854253.1	77	51.6026	35	27	heat shock protein DnaJ family protein
Locus_19008_Transcript_1/1_Conf_1.000	163	20	1.69E-17	NP_495675.1	85	92.4337	54	46	hypothetical protein DH11.1
Locus_19009_Transcript_1/1_Conf_1.000	168	0							
Locus_1901_Transcript_1/1_Conf_1.000	1440	20	0	XP_002647082.1	94	657.522	375	355	C. briggsae CBR-RPL-3 protein
Locus_19010_Transcript_1/1_Conf_1.000	742	20	1.59E-41	XP_001892576.1	84	173.711	125	105	Voltage-gated calcium channel, T-type, alpha subunit. C. elegans cca-1 ortholog
Locus_19011_Transcript_1/1_Conf_1.000	177	20	5.34E-24	NP_001021973.1	98	114.005	58	57	RAB family member (rab-3)

Locus_19012_Transcript_1/1_Conf_1.000	477	20	1.35E-51	EFO24598.1	81	205.682	144	117	hypothetical protein LOAG_03887
Locus_19013_Transcript_1/1_Conf_1.000	169	20	1.09E-16	XP_002642806.1	91	89.7373	48	44	C. briggsae CBR-CDK-5 protein
Locus_19014_Transcript_1/1_Conf_1.000	333	2	1.61E-28	NP_492185.2	78	129.028	97	76	SMG-associated and Lethal family member (smgl-1)
Locus_19015_Transcript_1/1_Conf_1.000	170	0							
Locus_19016_Transcript_1/1_Conf_1.000	453	20	5.00E-46	XP_002640189.1	72	187.193	148	107	Hypothetical protein CBG12692
Locus_19017_Transcript_1/3_Conf_0.600	1013	0							
Locus_19017_Transcript_2/3_Conf_0.600	455	0							
Locus_19017_Transcript_3/3_Conf_0.600	1037	0							
Locus_19018_Transcript_1/1_Conf_1.000	335	11	1.73E-22	EFO13548.1	73	108.997	98	72	hypothetical protein LOAG_14980
Locus_19019_Transcript_1/1_Conf_1.000	362	3	1.08E-32	XP_001892606.1	83	142.895	108	90	hypothetical protein Bm1_05665
Locus_1902_Transcript_1/3_Conf_0.714	1454	20	1.45E-26	NP_498156.1	51	125.561	235	122	hypothetical protein W03A5.4

Locus_1902_Transcript_2/3_Conf_0.714	1475	20	8.67E-27	XP_002641265.1	50	126.331	251	128	Hypothetical protein CBG05177
Locus_1902_Transcript_3/3_Conf_0.714	1448	20	1.44E-26	NP_498156.1	51	125.561	235	122	hypothetical protein W03A5.4
Locus_19020_Transcript_1/1_Conf_1.000	141	0							
Locus_19021_Transcript_1/1_Conf_1.000	325	0							
Locus_19022_Transcript_1/1_Conf_1.000	255	20	4.67E-07	ACX53644.1	55	57.7658	79	44	polyprotein
Locus_19023_Transcript_1/1_Conf_1.000	243	0							
Locus_19024_Transcript_1/1_Conf_1.000	1185	20	7.73E-65	EFO16078.1	63	252.292	349	220	hypothetical protein LOAG_12430
Locus_19025_Transcript_1/1_Conf_1.000	427	2	4.01E-27	XP_002636178.1	81	124.405	92	75	Hypothetical protein CBG01435
Locus_19026_Transcript_1/1_Conf_1.000	224	0							
Locus_19027_Transcript_1/1_Conf_1.000	639	4	2.19E-16	XP_002632958.1	74	89.7373	74	55	Hypothetical protein CBG21714
Locus_19028_Transcript_1/1_Conf_1.000	419	0							
Locus_19029_Transcript_1/1_Conf_1.000	513	20	1.49E-35	XP_002631317.1	77	152.525	112	87	Hypothetical protein CBG03139
Locus_1903_Transcript_1/1_Conf_1.000	1542	20	5.35E-160	XP_002630721.1	69	568.926	533	373	C. briggsae CBR-CLR-1 protein
Locus_19030_Transcript_1/1_Conf_1.000	302	0							
Locus_19031_Transcript_1/1_Conf_1.000	314	20	1.96E-21	XP_394116.3	68	105.531	104	71	PREDICTED: similar to Aromatic-L-amino-acid decarboxylase (AADC) (DOPA decarboxylase) (DDC)
Locus_19032_Transcript_1/1_Conf_1.000	212	0							
Locus_19033_Transcript_1/1_Conf_1.000	355	1	8.13E-04	XP_001897852.1	45	46.9802	126	57	notch-like transmembrane receptor
Locus_19034_Transcript_1/1_Conf_1.000	153	9	7.19E-08	XP_002646475.1	72	60.4622	50	36	Hypothetical protein CBG19448
Locus_19035_Transcript_1/1_Conf_1.000	188	0							
Locus_19036_Transcript_1/1_Conf_1.000	311	20	2.84E-17	NP_508551.1	59	91.6633	107	64	hypothetical protein F28B4.3

Locus_19037_Transcript_1/1_Conf_1.000	176	19	6.57E-14	XP_002811165.1	100	80.4925	38	38	PREDICTED: MARCKS-related protein-like
Locus_19038_Transcript_1/1_Conf_1.000	368	20	2.81E-41	XP_002638487.1	91	171.4	121	111	Hypothetical protein CBG12917
Locus_19039_Transcript_1/1_Conf_1.000	330	3	3.27E-21	NP_497897.1	82	104.76	91	75	PDCD (mammalian ProgrammeD Cell Death protein) homolog family member (pdcd-2)
Locus_1904_Transcript_1/1_Conf_1.000	806	20	4.33E-14	NP_507645.2	38	82.8037	248	96	hypothetical protein Y69H2.3
Locus_19040_Transcript_1/1_Conf_1.000	212	2	1.73E-06	XP_002637606.1	65	55.8398	52	34	Hypothetical protein CBG19344
Locus_19041_Transcript_1/1_Conf_1.000	511	20	1.84E-30	NP_498290.1	74	135.576	135	100	hypothetical protein F25B5.5
Locus_19042_Transcript_1/1_Conf_1.000	496	5	2.25E-38	NP_001022759.1	78	161.77	126	99	hypothetical protein T12D8.9
Locus_19043_Transcript_1/1_Conf_1.000	240	20	5.93E-39	XP_001084827.2	100	163.696	80	80	PREDICTED: serpin H1
Locus_19044_Transcript_1/1_Conf_1.000	318	20	4.04E-35	NP_498808.2	96	150.984	75	72	hypothetical protein C02F5.3

Locus_19045_Transcript_1/1_Conf_1.000	464	20	1.00E-17	XP_002648990.1	77	93.2041	72	56	Hypothetical protein CBG21322
Locus_19046_Transcript_1/1_Conf_1.000	276	20	5.93E-23	EFO22951.1	73	110.538	91	67	hypothetical protein LOAG_05534
Locus_19047_Transcript_1/1_Conf_1.000	338	13	1.36E-35	CBK19475.1	86	152.525	106	92	C. elegans protein T12G3.2e, confirmed by transcript evidence
Locus_19048_Transcript_1/1_Conf_1.000	250	0							
Locus_19049_Transcript_1/1_Conf_1.000	203	0							
Locus_1905_Transcript_1/1_Conf_1.000	495	0							
Locus_19050_Transcript_1/1_Conf_1.000	540	20	1.98E-87	NP_508184.2	92	325.094	180	166	AdaPtin, Mu/medium chain (clathrin associated complex) family member (apm-3)
Locus_19051_Transcript_1/1_Conf_1.000	323	20	4.48E-10	ABG23688.1	53	67.781	106	57	RNA-dependent RNA polymerase
Locus_19052_Transcript_1/1_Conf_1.000	890	20	7.51E-82	NP_492524.2	71	308.145	297	211	hypothetical protein F30A10.10
Locus_19053_Transcript_1/1_Conf_1.000	435	4	3.02E-43	NP_503059.2	85	177.948	111	95	hypothetical protein ZK550.3
Locus_19054_Transcript_1/2_Conf_1.000	375	0							
Locus_19054_Transcript_2/2_Conf_1.000	296	0							
Locus_19055_Transcript_1/1_Conf_1.000	162	6	1.58E-23	XP_530140.2	96	112.464	54	52	PREDICTED: hypothetical protein
Locus_19056_Transcript_1/1_Conf_1.000	483	0							
Locus_19057_Transcript_1/1_Conf_1.000	317	20	4.97E-25	NP_497482.1	78	117.472	89	70	hypothetical protein F53A3.7
Locus_19058_Transcript_1/1_Conf_1.000	171	0							
Locus_19059_Transcript_1/1_Conf_1.000	230	0							
Locus_1906_Transcript_1/1_Conf_1.000	712	20	2.23E-66	EFO22922.1	91	256.144	147	135	eukaryotic translation initiation factor 2 subunit 2
Locus_19060_Transcript_1/1_Conf_1.000	317	20	2.73E-15	EFO22683.1	68	85.1149	82	56	STE/STE11/MEKK2 protein kinase
Locus_19061_Transcript_1/1_Conf_1.000	136	0							
Locus_19062_Transcript_1/1_Conf_1.000	727	20	2.59E-49	NP_507155.1	57	199.519	224	129	hypothetical protein F26D2.13
Locus_19063_Transcript_1/1_Conf_1.000	197	0							

Locus_19064_Transcript_1/1_Conf_1.000	275	20	5.04E-22	NP_491855.1	84	107.457	66	56	Nuclear Pore complex Protein family member (npp-12)
Locus_19065_Transcript_1/1_Conf_1.000	717	20	5.02E-90	XP_002643890.1	81	334.724	243	198	Hypothetical protein CBG02142
Locus_19066_Transcript_1/1_Conf_1.000	273	20	3.38E-18	XP_002642446.1	74	94.7449	86	64	Hypothetical protein CBG06851
Locus_19067_Transcript_1/1_Conf_1.000	260	0							
Locus_19068_Transcript_1/1_Conf_1.000	149	0							
Locus_19069_Transcript_1/1_Conf_1.000	141	0							
Locus_1907_Transcript_1/5_Conf_0.308	747	20	6.15E-49	XP_001892438.1	82	198.364	137	113	60S ribosomal protein L23a
Locus_1907_Transcript_2/5_Conf_0.538	751	20	6.20E-49	XP_001892438.1	82	198.364	137	113	60S ribosomal protein L23a
Locus_1907_Transcript_3/5_Conf_0.462	751	20	6.20E-49	XP_001892438.1	82	198.364	137	113	60S ribosomal protein L23a

Locus_1907_Transcript_4/5_Conf_0.462	747	20	6.15E-49	XP_001892438.1	82	198.364	137	113	60S ribosomal protein L23a
Locus_1907_Transcript_5/5_Conf_0.308	543	20	2.85E-49	XP_001892438.1	82	198.364	137	113	60S ribosomal protein L23a
Locus_19070_Transcript_1/1_Conf_1.000	278	20	1.19E-47	EFO25348.1	100	192.586	92	92	rab family protein 3
Locus_19071_Transcript_1/1_Conf_1.000	308	0							
Locus_19072_Transcript_1/1_Conf_1.000	144	6	7.26E-08	XP_002639125.1	78	60.4622	47	37	C. briggsae CBR-FER-1 protein
Locus_19073_Transcript_1/1_Conf_1.000	653	20	2.08E-17	XP_001894228.1	61	93.2041	180	110	hypothetical protein
Locus_19074_Transcript_1/1_Conf_1.000	632	0							
Locus_19075_Transcript_1/1_Conf_1.000	492	20	1.61E-52	NP_001021681.1	73	208.764	172	126	hypothetical protein Y105E8A.10
Locus_19076_Transcript_1/1_Conf_1.000	238	8	1.74E-30	XP_002638768.1	90	135.576	76	69	Hypothetical protein CBG05111
Locus_19077_Transcript_1/1_Conf_1.000	188	0							
Locus_19078_Transcript_1/1_Conf_1.000	151	0							
Locus_19079_Transcript_1/1_Conf_1.000	294	20	1.75E-30	XP_002648619.1	78	135.576	98	77	Hypothetical protein CBG24962

Locus_1908_Transcript_1/2_Conf_1.000	1127	20	5.63E-102	AAM82155.1	69	375.555	324	224	cysteine proteinase
Locus_1908_Transcript_2/2_Conf_1.000	928	20	1.76E-60	AAM82155.1	67	237.269	209	141	cysteine proteinase
Locus_19080_Transcript_1/1_Conf_1.000	256	0							
Locus_19081_Transcript_1/1_Conf_1.000	134	0							
Locus_19082_Transcript_1/1_Conf_1.000	960	7	5.85E-14	XP_002640822.1	41	82.8037	286	119	Hypothetical protein CBG15709
Locus_19083_Transcript_1/1_Conf_1.000	429	5	2.60E-10	XP_002639923.1	85	68.5514	42	36	C. briggsae CBR-JPH-1 protein
Locus_19084_Transcript_1/1_Conf_1.000	230	0							
Locus_19085_Transcript_1/1_Conf_1.000	337	0							
Locus_19086_Transcript_1/1_Conf_1.000	193	20	4.96E-17	EAW84750.1	100	90.8929	63	63	coatomer protein complex, subunit epsilon, isoform CRA_c
Locus_19087_Transcript_1/1_Conf_1.000	200	20	4.60E-23	XP_001900213.1	90	110.923	66	60	Hypothetical oxidoreductase F56D1.5
Locus_19088_Transcript_1/1_Conf_1.000	235	1	2.07E-07	EFO24426.1	62	58.9214	67	42	hypothetical protein LOAG_04062
Locus_19089_Transcript_1/1_Conf_1.000	137	0							
Locus_1909_Transcript_1/1_Conf_1.000	317	4	1.11E-24	XP_002638813.1	71	116.316	106	76	C. briggsae CBR-ATM-1 protein
Locus_19090_Transcript_1/1_Conf_1.000	175	0							
Locus_19091_Transcript_1/1_Conf_1.000	209	0							
Locus_19092_Transcript_1/1_Conf_1.000	183	20	3.57E-31	EAW65357.1	100	137.887	60	60	filamin B, beta (actin binding protein 278), isoform CRA_e
Locus_19093_Transcript_1/1_Conf_1.000	525	20	4.70E-59	NP_496910.1	78	230.72	174	137	Histone DeAcetylase family member (hda-6)

Locus_19094_Transcript_1/1_Conf_1.000	275	20	1.52E-18	XP_786833.2	83	95.9005	68	57	PREDICTED: similar to MGC89020 protein
Locus_19095_Transcript_1/1_Conf_1.000	641	4	4.52E-54	NP_510177.1	69	214.927	213	148	hypothetical protein F17H10.1
Locus_19096_Transcript_1/1_Conf_1.000	1051	20	1.37E-91	XP_002633254.1	78	340.887	275	217	C. briggsae CBR-NDX-9 protein
Locus_19097_Transcript_1/1_Conf_1.000	137	0							
Locus_19098_Transcript_1/1_Conf_1.000	902	20	4.41E-45	EFO20748.1	58	186.037	269	157	MBOAT family protein
Locus_19099_Transcript_1/1_Conf_1.000	378	20	8.92E-35	XP_002640827.1	78	149.828	126	99	Hypothetical protein CBG15714
Locus_191_Transcript_1/1_Conf_1.000	1799	20	1.91E-26	AAO63577.1	48	125.561	266	129	secreted protein 5 precursor
Locus_1910_Transcript_1/3_Conf_0.286	308	15	2.67E-07	EFO20587.1	54	58.5362	96	52	groucho protein GRG1-S
Locus_1910_Transcript_2/3_Conf_0.714	1611	6	2.49E-06	EFO20587.1	54	58.5362	96	52	groucho protein GRG1-S
Locus_1910_Transcript_3/3_Conf_0.714	1611	6	2.49E-06	EFO20587.1	54	58.5362	96	52	groucho protein GRG1-S

Locus_19100_Transcript_1/1_Conf_1.000	207	20	1.05E-19	NP_510290.1	80	99.7525	68	55	Germinal Center Kinase family member (gck-4)
Locus_19101_Transcript_1/1_Conf_1.000	153	0							
Locus_19102_Transcript_1/1_Conf_1.000	131	0							
Locus_19103_Transcript_1/1_Conf_1.000	271	0							
Locus_19104_Transcript_1/1_Conf_1.000	144	0							
Locus_19105_Transcript_1/1_Conf_1.000	379	20	4.29E-29	NP_506019.2	81	130.954	90	73	aspartyl(D) tRNA Synthetase family member (drs-2)
Locus_19106_Transcript_1/1_Conf_1.000	210	0							
Locus_19107_Transcript_1/1_Conf_1.000	232	0							
Locus_19108_Transcript_1/1_Conf_1.000	213	2	1.73E-06	XP_002631789.1	60	55.8398	73	44	Hypothetical protein CBG21003
Locus_19109_Transcript_1/1_Conf_1.000	255	4	3.22E-24	XP_001898469.1	93	114.775	59	55	Zinc knuckle family protein
Locus_1911_Transcript_1/1_Conf_1.000	261	20	7.92E-07	XP_001495113.2	52	56.9954	85	45	PREDICTED: BUB1 budding uninhibited by benzimidazoles 1 homolog (yeast)
Locus_19110_Transcript_1/1_Conf_1.000	321	15	7.39E-13	CAA73142.1	67	77.0258	100	67	VPS34 homologue

Locus_19111_Transcript_1/1_Conf_1.000	387	10	6.54E-30	Q93890.5	82	133.65	103	85	UDP-galactose/UDP-N-acetylglucosamine transporter srf-3
Locus_19112_Transcript_1/1_Conf_1.000	379	2	2.14E-04	EFO63420.1	47	48.9062	117	56	Hypothetical protein GLP15_3736
Locus_19113_Transcript_1/1_Conf_1.000	285	0							
Locus_19114_Transcript_1/1_Conf_1.000	697	20	7.13E-78	XP_002634797.1	88	294.278	230	203	Hypothetical protein CBG13900
Locus_19115_Transcript_1/1_Conf_1.000	201	0							
Locus_19116_Transcript_1/1_Conf_1.000	322	0							
Locus_19117_Transcript_1/1_Conf_1.000	395	0							
Locus_19118_Transcript_1/1_Conf_1.000	158	0							
Locus_19119_Transcript_1/1_Conf_1.000	229	0							
Locus_1912_Transcript_1/2_Conf_1.000	416	3	2.94E-06	NP_740842.1	62	55.0694	77	48	protein KINase family member (kin-32)
Locus_1912_Transcript_2/2_Conf_1.000	1501	20	2.53E-106	NP_740842.1	64	390.578	470	303	protein KINase family member (kin-32)
Locus_19120_Transcript_1/1_Conf_1.000	329	20	2.98E-14	XP_002642974.1	79	81.6481	58	46	Hypothetical protein CBG15258
Locus_19121_Transcript_1/1_Conf_1.000	297	0							
Locus_19122_Transcript_1/1_Conf_1.000	214	0							
Locus_19123_Transcript_1/1_Conf_1.000	387	0							
Locus_19124_Transcript_1/1_Conf_1.000	366	0							
Locus_19125_Transcript_1/1_Conf_1.000	466	20	8.99E-27	NP_508866.1	54	123.25	153	83	Unidentified Vitellogenin-linked Transcript family member (uvt-3)

Locus_19126_Transcript_1/1_Conf_1.000	236	20	2.20E-33	XP_002639036.1	96	145.206	78	75	C. briggsae CBR-DYB-1 protein
Locus_19127_Transcript_1/1_Conf_1.000	292	1	7.66E-10	EFO25846.1	54	67.0106	96	52	hypothetical protein LOAG_02637
Locus_19128_Transcript_1/1_Conf_1.000	895	2	4.10E-27	XP_002640906.1	78	126.331	84	66	Hypothetical protein CBG00463
Locus_19129_Transcript_1/1_Conf_1.000	422	20	8.77E-06	ABK42176.1	53	53.5286	98	52	Chk2
Locus_1913_Transcript_1/2_Conf_1.000	590	0							
Locus_1913_Transcript_2/2_Conf_1.000	379	0							
Locus_19130_Transcript_1/1_Conf_1.000	433	4	2.58E-18	NP_497051.1	76	95.1301	72	55	hypothetical protein K02B7.3
Locus_19131_Transcript_1/1_Conf_1.000	339	0							
Locus_19132_Transcript_1/1_Conf_1.000	186	2	7.48E-05	XP_002639088.1	72	50.447	40	29	C. briggsae CBR-TAG-83 protein
Locus_19133_Transcript_1/1_Conf_1.000	244	0							
Locus_19134_Transcript_1/1_Conf_1.000	324	3	3.78E-09	XP_002633677.1	76	64.6994	65	50	C. briggsae CBR-LAD-2 protein
Locus_19135_Transcript_1/1_Conf_1.000	135	0							
Locus_19136_Transcript_1/1_Conf_1.000	219	0							
Locus_19137_Transcript_1/1_Conf_1.000	532	0							
Locus_19138_Transcript_1/1_Conf_1.000	533	4	3.40E-52	NP_499313.2	80	207.994	177	142	hypothetical protein F43D9.1

Locus_19139_Transcript_1/1_Conf_1.000	367	20	4.99E-22	XP_002637375.1	60	107.457	120	72	C. briggsae CBR-MYS-1 protein
Locus_1914_Transcript_1/1_Conf_1.000	494	0							
Locus_19140_Transcript_1/1_Conf_1.000	149	0							
Locus_19141_Transcript_1/1_Conf_1.000	420	20	3.91E-30	NP_001033350.1	68	134.42	132	90	hypothetical protein C23G10.7
Locus_19142_Transcript_1/1_Conf_1.000	151	0							
Locus_19143_Transcript_1/1_Conf_1.000	528	2	1.24E-06	XP_001901497.1	53	56.6102	104	56	Zinc finger, C2H2 type family protein
Locus_19144_Transcript_1/1_Conf_1.000	407	2	3.75E-17	EFO18996.1	60	91.2781	126	76	hypothetical protein LOAG_09500
Locus_19145_Transcript_1/1_Conf_1.000	243	0							
Locus_19146_Transcript_1/1_Conf_1.000	209	4	6.61E-06	NP_001129925.1	85	53.9138	35	30	High Incidence of Males (increased X chromosome loss) family member (him-4)
Locus_19147_Transcript_1/1_Conf_1.000	211	0							
Locus_19148_Transcript_1/1_Conf_1.000	218	20	6.96E-24	EFO28414.1	86	113.62	72	62	hypothetical protein LOAG_00060
Locus_19149_Transcript_1/1_Conf_1.000	434	0							

Locus_1915_Transcript_1/1_Conf_1.000	244	20	2.75E-28	CAV31817.1	87	128.257	79	69	C. elegans protein Y54G9A.3b, partially confirmed by transcript evidence
Locus_19150_Transcript_1/1_Conf_1.000	196	20	1.70E-17	XP_002719350.1	100	92.4337	48	48	PREDICTED: prohibitin
Locus_19151_Transcript_1/1_Conf_1.000	201	0							
Locus_19152_Transcript_1/1_Conf_1.000	151	2	1.90E-08	NP_492763.1	69	62.3882	49	34	hypothetical protein F25H2.7
Locus_19153_Transcript_1/1_Conf_1.000	129	0							
Locus_19154_Transcript_1/2_Conf_1.000	1470	20	6.22E-126	NP_508552.1	62	455.677	501	314	hypothetical protein F40F4.6
Locus_19154_Transcript_2/2_Conf_1.000	1470	20	3.65E-126	NP_508552.1	62	456.447	501	314	hypothetical protein F40F4.6
Locus_19155_Transcript_1/1_Conf_1.000	446	20	6.84E-51	XP_002638900.1	87	203.371	148	129	Hypothetical protein CBG22126

Locus_19156_Transcript_1/1_Conf_1.000	219	20	1.72E-25	XP_002645154.1	86	119.013	69	60	C. briggsae CBR-SEM-5 protein
Locus_19157_Transcript_1/1_Conf_1.000	820	20	2.46E-97	NP_509408.1	74	359.377	275	205	hypothetical protein R09F10.1
Locus_19158_Transcript_1/1_Conf_1.000	217	0							
Locus_19159_Transcript_1/2_Conf_1.000	403	1	6.24E-04	NP_505787.1	52	47.3654	103	54	hypothetical protein F58B4.4
Locus_19159_Transcript_2/2_Conf_1.000	403	0							
Locus_1916_Transcript_1/1_Conf_1.000	2977	20	0	XP_002630360.1	84	1172.53	880	743	C. briggsae CBR-TSN-1 protein
Locus_19160_Transcript_1/1_Conf_1.000	355	0							
Locus_19161_Transcript_1/1_Conf_1.000	151	0							
Locus_19162_Transcript_1/1_Conf_1.000	153	0							
Locus_19163_Transcript_1/1_Conf_1.000	397	20	4.03E-43	NP_506047.5	83	177.563	132	110	abnormal CHEmotaxis family member (che-11)
Locus_19164_Transcript_1/1_Conf_1.000	340	4	9.74E-18	XP_002636415.1	84	93.2041	113	96	Hypothetical protein CBG23068
Locus_19165_Transcript_1/1_Conf_1.000	371	20	2.22E-41	CAX65065.1	85	171.785	111	95	C. elegans protein K01A6.2c, partially confirmed by transcript evidence
Locus_19166_Transcript_1/1_Conf_1.000	640	20	4.49E-78	ADD13547.1	80	294.664	222	179	Inositol triphosphate receptor protein 1, isoform h

Locus_19167_Transcript_1/1_Conf_1.000	213	2	1.01E-14	XP_002632364.1	83	83.1889	53	44	Hypothetical protein CBG00382
Locus_19168_Transcript_1/1_Conf_1.000	348	3	1.03E-22	XP_002647656.1	62	109.768	117	73	Hypothetical protein CBG06763
Locus_19169_Transcript_1/1_Conf_1.000	251	0							
Locus_1917_Transcript_1/1_Conf_1.000	2068	20	0	XP_002637841.1	96	1114.37	603	581	C. briggsae CBR-VHA-13 protein
Locus_19170_Transcript_1/1_Conf_1.000	242	0							
Locus_19171_Transcript_1/1_Conf_1.000	791	20	1.54E-117	NP_493910.2	87	426.402	263	230	Sodium:Neurotransmitter symporter Family family member (snf-3)
Locus_19172_Transcript_1/1_Conf_1.000	213	0							
Locus_19173_Transcript_1/1_Conf_1.000	266	3	9.32E-08	XP_002636556.1	67	60.077	58	39	Hypothetical protein CBG23246
Locus_19174_Transcript_1/1_Conf_1.000	311	0							
Locus_19175_Transcript_1/1_Conf_1.000	174	0							
Locus_19176_Transcript_1/1_Conf_1.000	217	0							
Locus_19177_Transcript_1/2_Conf_1.000	596	4	1.49E-18	NP_490990.2	65	96.6709	155	102	hypothetical protein Y23H5B.5
Locus_19177_Transcript_2/2_Conf_1.000	437	3	1.28E-09	NP_490990.2	64	66.2402	85	55	hypothetical protein Y23H5B.5
Locus_19178_Transcript_1/1_Conf_1.000	295	20	6.88E-19	XP_001895786.1	85	97.0561	61	52	Fringe-like family protein
Locus_19179_Transcript_1/1_Conf_1.000	182	20	9.81E-13	NP_493967.1	83	76.6406	55	46	Conserved Oligomeric Golgi (COG) Component family member (cogc-4)
Locus_1918_Transcript_1/1_Conf_1.000	2006	20	0	NP_001022876.1	73	734.947	587	429	GLYcosylation related family member (gly-9)
Locus_19180_Transcript_1/1_Conf_1.000	298	0							
Locus_19181_Transcript_1/1_Conf_1.000	364	0							
Locus_19182_Transcript_1/1_Conf_1.000	341	0							
Locus_19183_Transcript_1/1_Conf_1.000	158	0							
Locus_19184_Transcript_1/1_Conf_1.000	192	2	1.35E-06	XP_002643956.1	57	56.225	61	35	Hypothetical protein CBG17315
Locus_19185_Transcript_1/1_Conf_1.000	168	0							

Locus_19186_Transcript_1/1_Conf_1.000	135	10	1.91E-13	XP_002637401.1	95	78.9518	43	41	C. briggsae CBR-FKB-3 protein
Locus_19187_Transcript_1/1_Conf_1.000	650	20	6.26E-91	NP_510336.2	85	337.421	215	184	TBC (Tre-2/Bub2/Cdc16) domain family member (tbc-19)
Locus_19188_Transcript_1/1_Conf_1.000	565	20	2.49E-62	XP_002634543.1	76	241.891	188	143	C. briggsae CBR-CED-5 protein
Locus_19189_Transcript_1/1_Conf_1.000	134	0							
Locus_1919_Transcript_1/1_Conf_1.000	1198	20	1.57E-97	XP_001901770.1	85	360.918	220	189	translation initiation factor 4E
Locus_19190_Transcript_1/1_Conf_1.000	228	0							
Locus_19191_Transcript_1/1_Conf_1.000	380	20	2.98E-22	NP_497538.1	63	108.227	130	82	hypothetical protein Y54F10BM.13
Locus_19192_Transcript_1/1_Conf_1.000	227	0							
Locus_19193_Transcript_1/1_Conf_1.000	361	2	9.14E-32	XP_001899472.1	70	139.813	128	90	Patatin-like phospholipase family protein
Locus_19194_Transcript_1/1_Conf_1.000	387	20	3.32E-66	XP_002630534.1	97	254.218	128	125	Hypothetical protein CBG12974
Locus_19195_Transcript_1/1_Conf_1.000	358	20	8.04E-28	NP_507683.2	83	126.716	80	67	tryptophanyl (W) tRNA Synthetase family member (wrs-1)
Locus_19196_Transcript_1/1_Conf_1.000	260	0							
Locus_19197_Transcript_1/1_Conf_1.000	179	0							
Locus_19198_Transcript_1/1_Conf_1.000	426	20	8.96E-19	XP_394116.3	65	96.6709	113	74	PREDICTED: similar to Aromatic-L-amino-acid decarboxylase (AADC) (DOPA decarboxylase) (DDC)
Locus_19199_Transcript_1/1_Conf_1.000	153	20	2.55E-13	NP_510672.2	90	78.5666	50	45	Nematode AStacin protease family member (nas-39)
Locus_192_Transcript_1/1_Conf_1.000	1244	20	2.85E-73	NP_494789.1	60	280.411	379	228	hypothetical protein W06B4.2
Locus_1920_Transcript_1/1_Conf_1.000	559	4	1.69E-23	XP_001899166.1	68	112.849	106	73	GTP-ase activating protein for Arf containing protein
Locus_19200_Transcript_1/1_Conf_1.000	133	0							

Locus_19201_Transcript_1/1_Conf_1.000	237	6	1.75E-06	XP_002644145.1	71	55.8398	53	38	C. briggsae CBR-DAF-12 protein
Locus_19202_Transcript_1/1_Conf_1.000	303	4	2.87E-09	XP_002641939.1	60	65.0846	95	57	C. briggsae CBR-MIG-10 protein
Locus_19203_Transcript_1/1_Conf_1.000	321	0							
Locus_19204_Transcript_1/1_Conf_1.000	182	0							
Locus_19205_Transcript_1/1_Conf_1.000	692	0							
Locus_19206_Transcript_1/1_Conf_1.000	822	20	4.73E-56	XP_001902208.1	71	222.246	177	127	TM2 domain containing protein
Locus_19207_Transcript_1/1_Conf_1.000	167	0							
Locus_19208_Transcript_1/1_Conf_1.000	142	0							
Locus_19209_Transcript_1/1_Conf_1.000	188	20	1.96E-21	XP_002113675.1	93	105.531	62	58	hypothetical protein TRIADDRAFT_50388
Locus_1921_Transcript_1/2_Conf_1.000	672	20	4.54E-79	NP_502655.1	87	298.13	187	164	Ribosomal Protein, Large subunit family member (rpl-18)
Locus_1921_Transcript_2/2_Conf_1.000	672	20	1.01E-78	NP_502655.1	87	296.975	187	164	Ribosomal Protein, Large subunit family member (rpl-18)
Locus_19210_Transcript_1/1_Conf_1.000	234	0							
Locus_19211_Transcript_1/1_Conf_1.000	387	20	3.36E-26	NP_502803.3	95	121.324	63	60	EPS (human endocytosis) related family member (eps-8)
Locus_19212_Transcript_1/1_Conf_1.000	298	3	3.90E-06	XP_002643075.1	60	54.6842	65	39	Hypothetical protein CBG22995
Locus_19213_Transcript_1/1_Conf_1.000	694	19	2.30E-36	EFO26231.1	67	156.377	165	111	hypothetical protein LOAG_02257

Locus_19214_Transcript_1/1_Conf_1.000	422	20	2.89E-33	XP_002645074.1	65	144.821	138	91	Hypothetical protein CBG16737
Locus_19215_Transcript_1/1_Conf_1.000	227	20	3.68E-12	CAR63556.1	68	74.7146	64	44	putative Lin-5 (Five) Interacting protein
Locus_19216_Transcript_1/1_Conf_1.000	214	20	3.59E-12	XP_002633609.1	71	74.7146	69	49	Hypothetical protein CBG05491
Locus_19217_Transcript_1/1_Conf_1.000	216	20	4.84E-09	XP_002594167.1	59	64.3142	66	39	hypothetical protein BRAFLDRAFT_195549
Locus_19218_Transcript_1/1_Conf_1.000	194	3	7.90E-23	NP_492730.2	90	110.153	64	58	hypothetical protein Y106G6H.5
Locus_19219_Transcript_1/1_Conf_1.000	227	20	1.22E-31	NP_499205.1	94	139.428	75	71	hypothetical protein K03H1.5
Locus_1922_Transcript_1/1_Conf_1.000	1067	20	2.66E-82	XP_001895586.1	63	310.071	377	238	N terminus of Rad21 / Rec8 like protein
Locus_19220_Transcript_1/1_Conf_1.000	183	20	3.57E-31	BAG60662.1	100	137.887	60	60	unnamed protein product
Locus_19221_Transcript_1/1_Conf_1.000	709	20	5.90E-11	ABB53347.1	45	72.0182	170	78	secreted protein 5 precursor
Locus_19222_Transcript_1/1_Conf_1.000	282	0							
Locus_19223_Transcript_1/1_Conf_1.000	317	20	3.33E-29	NP_497990.1	76	131.339	106	81	hypothetical protein C38D4.9
Locus_19224_Transcript_1/1_Conf_1.000	497	0							
Locus_19225_Transcript_1/1_Conf_1.000	148	17	1.01E-09	XP_002638772.1	81	66.6254	48	39	C. briggsae CBR-PTR-21 protein
Locus_19226_Transcript_1/1_Conf_1.000	310	20	3.72E-09	XP_002631206.1	90	64.6994	41	37	Hypothetical protein CBG03002
Locus_19227_Transcript_1/1_Conf_1.000	150	0							
Locus_19228_Transcript_1/1_Conf_1.000	222	20	4.50E-26	NP_492127.1	87	120.939	74	65	Low-density lipoprotein Receptor Related family member (lrp-1)
Locus_19229_Transcript_1/1_Conf_1.000	215	2	3.47E-15	NP_495787.1	74	84.7297	70	52	hypothetical protein M05D6.2
Locus_1923_Transcript_1/3_Conf_0.400	790	0							

Locus_1923_Transcript_2/3_Conf_0.600	2129	20	9.49E-52	EFO20470.1	64	209.92	300	192	hypothetical protein LOAG_08019
Locus_1923_Transcript_3/3_Conf_0.400	1559	20	8.29E-76	EFO20470.1	65	289.271	375	245	hypothetical protein LOAG_08019
Locus_19230_Transcript_1/1_Conf_1.000	186	0							
Locus_19231_Transcript_1/1_Conf_1.000	143	20	2.83E-17	ABW80082.1	95	91.6633	47	45	immunoglobulin heavy chain variable region
Locus_19232_Transcript_1/1_Conf_1.000	187	0							
Locus_19233_Transcript_1/1_Conf_1.000	213	0							
Locus_19234_Transcript_1/1_Conf_1.000	181	0							
Locus_19235_Transcript_1/1_Conf_1.000	200	0							
Locus_19236_Transcript_1/1_Conf_1.000	140	20	4.11E-08	XP_002644355.1	78	61.2326	46	36	C. briggsae CBR-DDR-2 protein
Locus_19237_Transcript_1/1_Conf_1.000	157	0							
Locus_19238_Transcript_1/1_Conf_1.000	229	0							
Locus_19239_Transcript_1/1_Conf_1.000	164	0							
Locus_1924_Transcript_1/1_Conf_1.000	280	20	4.51E-31	NP_502553.2	93	137.502	83	78	Cleavage and Polyadenylation Specificity Factor family member (cpsf-3)
Locus_19240_Transcript_1/1_Conf_1.000	254	0							

Locus_19241_Transcript_1/2_Conf_1.000	315	20	1.49E-37	XP_001175663.1	100	159.073	79	79	PREDICTED: similar to H4 histone protein
Locus_19241_Transcript_2/2_Conf_1.000	378	20	1.20E-39	XP_001175663.1	100	166.007	83	83	PREDICTED: similar to H4 histone protein
Locus_19242_Transcript_1/1_Conf_1.000	153	0							
Locus_19243_Transcript_1/1_Conf_1.000	284	20	1.23E-31	XP_513264.2	100	139.428	94	94	PREDICTED: similar to KIAA0085
Locus_19244_Transcript_1/1_Conf_1.000	190	2	2.17E-04	XP_002634109.1	62	48.9062	64	40	C. briggsae CBR-CSN-3 protein
Locus_19245_Transcript_1/1_Conf_1.000	156	0							
Locus_19246_Transcript_1/1_Conf_1.000	218	0							
Locus_19247_Transcript_1/1_Conf_1.000	322	2	3.31E-21	NP_499330.2	80	104.76	63	51	hypothetical protein Y45F3A.4
Locus_19248_Transcript_1/1_Conf_1.000	423	0							
Locus_19249_Transcript_1/1_Conf_1.000	213	20	7.44E-34	3GQY	100	146.747	70	70	Activator-BoundStructure Of Human Pyruvate Kinase M2

Locus_1925_Transcript_1/1_Conf_1.000	243	4	1.47E-05	NP_872245.2	65	52.7582	58	38	protein KINase family member (kin-25)
Locus_19250_Transcript_1/1_Conf_1.000	164	20	2.14E-12	CAA30855.1	97	75.485	41	40	myosin heavy chain 2
Locus_19251_Transcript_1/1_Conf_1.000	200	0							
Locus_19252_Transcript_1/1_Conf_1.000	273	20	1.16E-26	NP_510130.2	82	122.865	90	74	hypothetical protein F22E10.5
Locus_19253_Transcript_1/1_Conf_1.000	214	0							
Locus_19254_Transcript_1/1_Conf_1.000	292	6	8.16E-20	XP_001896152.1	73	100.138	73	54	hypothetical protein Bm1_23475
Locus_19255_Transcript_1/1_Conf_1.000	769	0							
Locus_19256_Transcript_1/1_Conf_1.000	231	20	2.01E-10	XP_002639913.1	70	68.9366	60	42	Hypothetical protein CBG08243
Locus_19257_Transcript_1/1_Conf_1.000	141	0							

Locus_19258_Transcript_1/1_Conf_1.000	265	20	1.30E-17	XP_001096144.2	100	92.8189	42	42	PREDICTED: hypothetical protein LOC707717
Locus_19259_Transcript_1/1_Conf_1.000	492	0							
Locus_1926_Transcript_1/2_Conf_1.000	2083	20	0	NP_001122877.1	82	909.442	642	531	hypothetical protein C39F7.2
Locus_1926_Transcript_2/2_Conf_1.000	2083	20	0	NP_001122877.1	82	909.442	642	531	hypothetical protein C39F7.2
Locus_19260_Transcript_1/1_Conf_1.000	206	0							
Locus_19261_Transcript_1/1_Conf_1.000	162	20	8.13E-12	NP_506047.5	79	73.559	53	42	abnormal CHEmotaxis family member (che-11)
Locus_19262_Transcript_1/1_Conf_1.000	138	0							

Locus_19263_Transcript_1/1_Conf_1.000	213	20	5.52E-29	NP_001040912.1	94	130.568	70	66	hypothetical protein C08F8.2
Locus_19264_Transcript_1/1_Conf_1.000	261	1	1.54E-10	NP_503410.3	62	69.3218	74	46	hypothetical protein R12A1.3
Locus_19265_Transcript_1/1_Conf_1.000	328	10	1.16E-34	NP_509899.3	77	149.443	110	85	Organic Cation Transporter family member (oct-2)
Locus_19266_Transcript_1/1_Conf_1.000	525	0							
Locus_19267_Transcript_1/1_Conf_1.000	289	20	5.65E-37	NP_509002.2	82	157.147	96	79	INneXin family member (inx-3)
Locus_19268_Transcript_1/1_Conf_1.000	353	0							
Locus_19269_Transcript_1/1_Conf_1.000	287	20	1.40E-19	XP_002647861.1	72	99.3673	95	69	Hypothetical protein CBG23659
Locus_1927_Transcript_1/1_Conf_1.000	832	13	1.40E-71	CBL43423.1	69	273.863	275	190	C. elegans protein C53A5.13b, partially confirmed by transcript evidence
Locus_19270_Transcript_1/1_Conf_1.000	432	0							
Locus_19271_Transcript_1/1_Conf_1.000	578	20	3.83E-77	NP_001022641.1	83	291.197	192	161	LEThal family member (let-805)
Locus_19272_Transcript_1/1_Conf_1.000	712	4	1.11E-57	XP_002645171.1	65	227.254	240	156	Hypothetical protein CBG16891
Locus_19273_Transcript_1/1_Conf_1.000	134	5	3.74E-09	XP_002630796.1	86	64.6994	38	33	Hypothetical protein CBG02494
Locus_19274_Transcript_1/1_Conf_1.000	958	20	1.52E-46	XP_002641058.1	61	191.045	320	196	Hypothetical protein CBG22469
Locus_19275_Transcript_1/1_Conf_1.000	139	0							
Locus_19276_Transcript_1/1_Conf_1.000	128	0							
Locus_19277_Transcript_1/1_Conf_1.000	133	0							
Locus_19278_Transcript_1/1_Conf_1.000	365	0							
Locus_19279_Transcript_1/1_Conf_1.000	470	20	1.99E-34	ACI49192.1	69	148.673	160	111	hypothetical protein Csp3_JD03.001
Locus_1928_Transcript_1/1_Conf_1.000	818	20	4.25E-25	XP_001897581.1	53	119.398	279	150	hypothetical protein Bm1_30710

Locus_19280_Transcript_1/1_Conf_1.000	139	3	5.03E-06	XP_002647772.1	83	54.299	36	30	Hypothetical protein CBG20529
Locus_19281_Transcript_1/1_Conf_1.000	128	0							
Locus_19282_Transcript_1/1_Conf_1.000	259	20	7.61E-42	XP_002920355.1	100	173.326	85	85	PREDICTED: proteasome subunit alpha type-7-like
Locus_19283_Transcript_1/1_Conf_1.000	372	11	1.49E-29	AAK31453.4	71	132.494	123	88	Hypothetical protein C25H3.8
Locus_19284_Transcript_1/1_Conf_1.000	372	20	9.06E-11	XP_001032930.1	70	70.0922	51	36	DHHC zinc finger domain containing protein
Locus_19285_Transcript_1/1_Conf_1.000	192	0							
Locus_19286_Transcript_1/1_Conf_1.000	237	0							
Locus_19287_Transcript_1/1_Conf_1.000	182	0							
Locus_19288_Transcript_1/1_Conf_1.000	158	0							
Locus_19289_Transcript_1/1_Conf_1.000	216	0							
Locus_1929_Transcript_1/1_Conf_1.000	491	20	1.16E-58	XP_002639025.1	83	229.18	150	125	C. briggsae CBR-RPS-10 protein
Locus_19290_Transcript_1/1_Conf_1.000	266	0							
Locus_19291_Transcript_1/1_Conf_1.000	332	0							
Locus_19292_Transcript_1/1_Conf_1.000	223	0							
Locus_19293_Transcript_1/1_Conf_1.000	216	0							
Locus_19294_Transcript_1/1_Conf_1.000	842	0							
Locus_19295_Transcript_1/1_Conf_1.000	525	20	3.48E-62	NP_001022870.1	79	241.121	169	134	hypothetical protein Y43F4A.1
Locus_19296_Transcript_1/1_Conf_1.000	132	0							
Locus_19297_Transcript_1/1_Conf_1.000	259	3	3.93E-22	XP_002642486.1	83	107.842	86	72	Hypothetical protein CBG06905
Locus_19298_Transcript_1/1_Conf_1.000	752	20	1.81E-32	EFO22295.1	88	143.665	123	109	hypothetical protein LOAG_06193
Locus_19299_Transcript_1/1_Conf_1.000	220	0							
Locus_193_Transcript_1/2_Conf_1.000	1078	20	1.95E-64	CAR63652.1	95	250.751	135	129	hypothetical protein
Locus_193_Transcript_2/2_Conf_1.000	2045	20	5.69E-139	NP_497804.2	69	499.59	441	306	PleXin Domain containing family member (pxd-1)

Locus_1930_Transcript_1/1_Conf_1.000	572	20	1.69E-21	NP_492370.1	96	106.301	56	54	Transport to INner mitochondrial membrane (yeast TIM) family member (tin-13)
Locus_19300_Transcript_1/1_Conf_1.000	155	0							
Locus_19301_Transcript_1/1_Conf_1.000	411	0							
Locus_19302_Transcript_1/1_Conf_1.000	722	20	1.50E-33	NP_510576.2	57	147.132	242	139	hypothetical protein K09E9.1
Locus_19303_Transcript_1/1_Conf_1.000	227	0							
Locus_19304_Transcript_1/1_Conf_1.000	209	20	3.99E-19	NP_497420.1	89	97.8265	69	62	hypothetical protein Y67D2.6
Locus_19305_Transcript_1/1_Conf_1.000	150	0							
Locus_19306_Transcript_1/1_Conf_1.000	332	20	4.25E-37	XP_001899111.1	88	157.532	96	85	Mitochondrial carrier protein
Locus_19307_Transcript_1/1_Conf_1.000	377	20	4.01E-43	XP_002636973.1	76	177.563	125	95	Hypothetical protein CBG09454
Locus_19308_Transcript_1/2_Conf_1.000	884	20	8.20E-81	CAA22108.2	74	304.679	295	219	C. elegans protein Y75B8A.24, partially confirmed by transcript evidence
Locus_19308_Transcript_2/2_Conf_1.000	578	20	7.26E-60	CAA22108.2	74	233.802	204	152	C. elegans protein Y75B8A.24, partially confirmed by transcript evidence
Locus_19309_Transcript_1/1_Conf_1.000	355	20	1.51E-50	AAF59456.4	91	202.216	118	108	Smg-associated and lethal protein 2, partially confirmed by transcript evidence
Locus_1931_Transcript_1/1_Conf_1.000	1692	20	6.40E-93	ACV31107.1	66	346.28	413	273	Hypothetical protein F22D3.2b
Locus_19310_Transcript_1/1_Conf_1.000	334	20	1.37E-11	NP_505133.1	58	72.7886	80	47	ASpartyl Protease family member (asp-6)
Locus_19311_Transcript_1/1_Conf_1.000	281	0							

Locus_19312_Transcript_1/2_Conf_1.000	588	20	4.20E-26	XP_002640523.1	83	121.709	80	67	Hypothetical protein CBG18685
Locus_19312_Transcript_2/2_Conf_1.000	588	20	4.20E-26	XP_002640523.1	83	121.709	80	67	Hypothetical protein CBG18685
Locus_19313_Transcript_1/1_Conf_1.000	290	0							
Locus_19314_Transcript_1/1_Conf_1.000	353	20	2.27E-38	XP_002632772.1	83	161.77	117	98	C. briggsae CBR-TAT-2 protein
Locus_19315_Transcript_1/1_Conf_1.000	157	0							
Locus_19316_Transcript_1/1_Conf_1.000	212	20	5.19E-19	NP_504373.2	72	97.4413	70	51	hypothetical protein F14F9.5
Locus_19317_Transcript_1/1_Conf_1.000	191	20	1.60E-23	ACJ65180.1	90	112.464	63	57	Gaba b receptor subunit protein 2, confirmed by transcript evidence
Locus_19318_Transcript_1/1_Conf_1.000	278	0							
Locus_19319_Transcript_1/1_Conf_1.000	211	0							
Locus_1932_Transcript_1/1_Conf_1.000	543	20	7.93E-84	BAG52420.1	100	313.153	180	180	unnamed protein product
Locus_19320_Transcript_1/1_Conf_1.000	423	0							
Locus_19321_Transcript_1/1_Conf_1.000	178	0							
Locus_19322_Transcript_1/1_Conf_1.000	152	0							
Locus_19323_Transcript_1/1_Conf_1.000	138	3	4.71E-04	CAX12927.1	68	47.7506	45	31	novel protein similar to human major facilitator superfamily domain containing 5 (MFSD5, zgc:91807)
Locus_19324_Transcript_1/1_Conf_1.000	131	0							
Locus_19325_Transcript_1/1_Conf_1.000	184	20	1.78E-14	XP_002630326.1	75	82.4185	58	44	Hypothetical protein CBG04253
Locus_19326_Transcript_1/1_Conf_1.000	150	0							

Locus_19327_Transcript_1/1_Conf_1.000	614	20	6.55E-44	XP_002648449.1	63	181.03	204	129	Hypothetical protein CBG24724
Locus_19328_Transcript_1/1_Conf_1.000	165	20	3.28E-21	XP_002927427.1	100	104.76	53	53	PREDICTED: transcription elongation factor SPT5-like isoform 2
Locus_19329_Transcript_1/1_Conf_1.000	484	4	1.69E-41	AAQ11020.1	69	172.17	172	120	mesocentin
Locus_1933_Transcript_1/1_Conf_1.000	2006	20	0	XP_002646154.1	82	826.624	627	516	C. briggsae CBR-CYN-15 protein
Locus_19330_Transcript_1/1_Conf_1.000	222	20	3.56E-23	NP_508805.3	94	111.309	53	50	hypothetical protein K02G10.1
Locus_19331_Transcript_1/1_Conf_1.000	423								
Locus_19332_Transcript_1/1_Conf_1.000	130	20	4.15E-08	XP_002600688.1	88	61.2326	34	30	hypothetical protein BRAFLDRAFT_118551
Locus_19333_Transcript_1/1_Conf_1.000	447	20	7.86E-15	NP_505516.1	55	83.5741	129	71	hypothetical protein F58E6.5
Locus_19334_Transcript_1/1_Conf_1.000	357	0							
Locus_19335_Transcript_1/1_Conf_1.000	254	20	8.51E-25	XP_002645934.1	83	116.701	83	69	Hypothetical protein CBG07699
Locus_19336_Transcript_1/1_Conf_1.000	397	20	1.64E-44	NP_492590.1	83	182.185	123	103	hypothetical protein K02A11.3

Locus_19337_Transcript_1/1_Conf_1.000	130	1	7.55E-18	XP_001171837.1	95	93.5893	43	41	PREDICTED: DnaJ (Hsp40) homolog, subfamily B, member 1 isoform 1
Locus_19338_Transcript_1/1_Conf_1.000	637	2	9.67E-33	NP_509416.2	72	144.05	178	129	hypothetical protein F55E10.7
Locus_19339_Transcript_1/1_Conf_1.000	291	20	7.57E-50	XP_002928507.1	100	199.904	97	97	PREDICTED: elongation factor 2-like
Locus_1934_Transcript_1/1_Conf_1.000	650	0							
Locus_19340_Transcript_1/1_Conf_1.000	618	20	7.42E-51	NP_505595.2	68	204.142	203	139	UDP-GlucuronosylTransferase family member (ugt-47)
Locus_19341_Transcript_1/1_Conf_1.000	205	0							
Locus_19342_Transcript_1/1_Conf_1.000	740	20	4.14E-58	NP_492244.1	66	228.794	226	150	hypothetical protein ZK265.3
Locus_19343_Transcript_1/1_Conf_1.000	213	0							
Locus_19344_Transcript_1/1_Conf_1.000	310	0							
Locus_19345_Transcript_1/1_Conf_1.000	1214	20	2.23E-99	EFO22512.1	66	367.081	415	274	hypothetical protein LOAG_05973
Locus_19346_Transcript_1/1_Conf_1.000	439	0							
Locus_19347_Transcript_1/1_Conf_1.000	280	0							
Locus_19348_Transcript_1/1_Conf_1.000	218	3	4.24E-05	NP_500978.1	62	51.2174	56	35	EXOSome (multiexonuclease complex) component family member (exos-2)
Locus_19349_Transcript_1/1_Conf_1.000	194	0							
Locus_1935_Transcript_1/4_Conf_0.556	1543	2	1.02E-09	XP_001902753.1	54	69.707	126	69	RhoGAP domain containing protein
Locus_1935_Transcript_2/4_Conf_0.444	1429	2	7.05E-10	XP_001902753.1	50	70.0922	161	81	RhoGAP domain containing protein
Locus_1935_Transcript_3/4_Conf_0.333	1480	2	7.41E-10	XP_001902753.1	50	70.0922	161	81	RhoGAP domain containing protein
Locus_1935_Transcript_4/4_Conf_0.556	1594	2	8.15E-10	XP_001902753.1	50	70.0922	161	81	RhoGAP domain containing protein
Locus_19350_Transcript_1/1_Conf_1.000	335	0							
Locus_19351_Transcript_1/1_Conf_1.000	494	20	1.01E-38	EFO17779.1	66	162.925	159	105	hypothetical protein LOAG_10719
Locus_19352_Transcript_1/1_Conf_1.000	142	0							
Locus_19353_Transcript_1/1_Conf_1.000	234	1	1.17E-10	NP_001129775.1	79	69.707	54	43	GLoBin family member (glb-18)
Locus_19354_Transcript_1/1_Conf_1.000	208	0							

Locus_19355_Transcript_1/1_Conf_1.000	158	20	8.17E-20	XP_002647071.1	90	100.138	52	47	C. briggsae CBR-NIT-1 protein
Locus_19356_Transcript_1/1_Conf_1.000	141	0							
Locus_19357_Transcript_1/1_Conf_1.000	452	20	9.22E-24	NP_001041088.1	60	113.235	146	89	hypothetical protein C27A7.3
Locus_19358_Transcript_1/1_Conf_1.000	669	0							
Locus_19359_Transcript_1/1_Conf_1.000	488	17	7.38E-13	XP_001898480.1	62	77.0258	122	76	integrin cytoplasmic domain-associated protein 1, isoform 1, putative
Locus_1936_Transcript_1/4_Conf_0.444	473	20	4.39E-50	EFO20496.1	72	200.675	157	114	hypothetical protein LOAG_07994
Locus_1936_Transcript_2/4_Conf_0.667	1144	20	3.60E-88	EFO20496.1	56	329.717	432	245	hypothetical protein LOAG_07994
Locus_1936_Transcript_3/4_Conf_0.222	498	8	2.25E-14	XP_001893982.1	47	82.0333	190	91	Mitotic control protein dis3
Locus_1936_Transcript_4/4_Conf_0.444	473	20	7.49E-50	EFO20496.1	72	199.904	157	114	hypothetical protein LOAG_07994
Locus_19360_Transcript_1/1_Conf_1.000	172	5	2.19E-09	CBL43415.1	68	65.4698	54	37	C. elegans protein C32C4.2b, partially confirmed by transcript evidence
Locus_19361_Transcript_1/1_Conf_1.000	1228	20	5.24E-96	NP_001022767.1	72	355.91	345	251	protein KINase family member (kin-18)
Locus_19362_Transcript_1/1_Conf_1.000	274	0							

Locus_19363_Transcript_1/1_Conf_1.000	505	20	1.58E-26	NP_495701.2	75	122.479	97	73	IRE1 kinase related family member (ire-1)
Locus_19364_Transcript_1/1_Conf_1.000	267	20	8.65E-22	XP_001742234.1	74	106.686	89	66	hypothetical protein
Locus_19365_Transcript_1/1_Conf_1.000	255	0							
Locus_19366_Transcript_1/1_Conf_1.000	495	20	5.52E-45	EFO23518.1	72	183.726	160	116	serine/threonine protein kinase
Locus_19367_Transcript_1/1_Conf_1.000	465	2	1.03E-06	XP_001900574.1	47	56.6102	121	58	hypothetical protein Bm1_45555
Locus_19368_Transcript_1/1_Conf_1.000	168	3	1.43E-08	NP_496184.2	79	62.7734	49	39	Location Of Vulva defective family member (lov-1)
Locus_19369_Transcript_1/1_Conf_1.000	133	20	1.24E-12	NP_504553.1	95	76.2554	42	40	Adenylyl CYclase family member (acy-2)
Locus_1937_Transcript_1/1_Conf_1.000	565	0							
Locus_19370_Transcript_1/1_Conf_1.000	182	0							
Locus_19371_Transcript_1/1_Conf_1.000	145	0							
Locus_19372_Transcript_1/1_Conf_1.000	218	0							
Locus_19373_Transcript_1/1_Conf_1.000	201	3	5.42E-24	XP_002641473.1	88	114.005	67	59	Hypothetical protein CBG09762
Locus_19374_Transcript_1/1_Conf_1.000	141	0							
Locus_19375_Transcript_1/1_Conf_1.000	490	4	8.03E-52	NP_871810.1	80	206.453	164	132	HAlF transporter (PGP related) family member (haf-9)

Locus_19376_Transcript_1/1_Conf_1.000	382	20	6.56E-46	XP_002647328.1	88	186.808	106	94	C. briggsae CBR-EGL-8 protein
Locus_19377_Transcript_1/1_Conf_1.000	250	0							
Locus_19378_Transcript_1/1_Conf_1.000	535	20	8.04E-62	NP_492067.1	85	239.965	148	127	hypothetical protein F13G3.11
Locus_19379_Transcript_1/1_Conf_1.000	595	20	2.21E-38	EFO23010.1	65	162.54	167	109	hypothetical protein LOAG_05474
Locus_1938_Transcript_1/10_Conf_0.208	750	20	2.47E-114	XP_002594632.1	93	415.616	242	227	hypothetical protein BRAFLDRAFT_217516

Locus_1938_Transcript_10/10_Conf_0.333	1691	20	0	XP_002594632.1	84	669.848	458	386	hypothetical protein BRAFLDRAFT_217516
Locus_1938_Transcript_2/10_Conf_0.208	792	20	6.09E-114	XP_002594632.1	93	414.461	244	228	hypothetical protein BRAFLDRAFT_217516

Locus_1938_Transcript_3/10_Conf_0.250	1175	20	0	XP_002594632.1	91	647.121	384	352	hypothetical protein BRAFLDRAFT_217516
Locus_1938_Transcript_4/10_Conf_0.250	1175	20	0	XP_002594632.1	91	649.818	384	352	hypothetical protein BRAFLDRAFT_217516

Locus_1938_Transcript_5/10_Conf_0.333	1703	20	0	XP_002594632.1	83	664.07	462	386	hypothetical protein BRAFLDRAFT_217516
Locus_1938_Transcript_6/10_Conf_0.333	1703	20	0	XP_002594632.1	83	661.374	462	386	hypothetical protein BRAFLDRAFT_217516

Locus_1938_Transcript_7/10_Conf_0.333	1691	20	0	XP_002594632.1	84	667.152	458	386	hypothetical protein BRAFLDRAFT_217516
Locus_1938_Transcript_8/10_Conf_0.333	1691	20	0	XP_002594632.1	84	669.848	458	386	hypothetical protein BRAFLDRAFT_217516

Locus_1938_Transcript_9/10_Conf_0.333	1691	20	0	XP_002594632.1	84	667.152	458	386	hypothetical protein BRAFLDRAFT_217516
Locus_19380_Transcript_1/1_Conf_1.000	139	0							
Locus_19381_Transcript_1/1_Conf_1.000	530	20	4.96E-64	XP_002633755.1	81	247.284	172	140	Hypothetical protein CBG03442
Locus_19382_Transcript_1/3_Conf_0.600	928	20	6.94E-49	XP_002633289.1	61	198.749	302	185	Hypothetical protein CBG06018
Locus_19382_Transcript_2/3_Conf_0.600	911	20	2.73E-42	XP_002633289.1	60	176.792	280	170	Hypothetical protein CBG06018
Locus_19382_Transcript_3/3_Conf_0.600	910	20	1.22E-42	XP_002633289.1	60	177.948	283	171	Hypothetical protein CBG06018
Locus_19383_Transcript_1/1_Conf_1.000	721	20	1.04E-18	EFO20401.1	59	97.8265	155	92	sex-determining protein fem-1
Locus_19384_Transcript_1/1_Conf_1.000	173	20	1.98E-10	XP_001900530.1	75	68.9366	57	43	manba-prov protein
Locus_19385_Transcript_1/1_Conf_1.000	377	0							
Locus_19386_Transcript_1/1_Conf_1.000	418	20	5.26E-59	AAM82167.1	89	230.335	138	123	gamma-butyrobetaine,2-oxoglutarate dioxygenase
Locus_19387_Transcript_1/1_Conf_1.000	1034	2	5.40E-24	XP_001897079.1	46	116.316	372	172	Protein kinase domain containing protein

Locus_19388_Transcript_1/1_Conf_1.000	956	20	1.49E-78	NP_501489.2	68	297.36	311	212	hypothetical protein F08B4.5
Locus_19389_Transcript_1/1_Conf_1.000	280	5	2.76E-04	NP_741176.2	56	48.521	95	54	hypothetical protein C23G10.7
Locus_1939_Transcript_1/1_Conf_1.000	430	20	1.09E-16	CAR63597.1	70	89.7373	80	56	hypothetical protein
Locus_19390_Transcript_1/1_Conf_1.000	786	6	1.99E-16	NP_492861.1	68	90.5077	93	64	hypothetical protein Y52B11A.10
Locus_19391_Transcript_1/2_Conf_0.500	395	0							
Locus_19391_Transcript_2/2_Conf_0.750	116	0							
Locus_19392_Transcript_1/1_Conf_1.000	230	0							
Locus_19393_Transcript_1/1_Conf_1.000	359	8	8.61E-22		50	106.686	155	78	hypothetical protein H19M22.2 - Caenorhabditis elegans
Locus_19394_Transcript_1/1_Conf_1.000	542	0							
Locus_19395_Transcript_1/1_Conf_1.000	153	0							
Locus_19396_Transcript_1/1_Conf_1.000	133	0							
Locus_19397_Transcript_1/1_Conf_1.000	158	20	3.21E-16	XP_001897126.1	94	88.1965	51	48	DnaJ domain containing protein
Locus_19398_Transcript_1/1_Conf_1.000	181	0							
Locus_19399_Transcript_1/1_Conf_1.000	428	6	5.10E-06	EFO23325.1	50	54.299	97	49	hypothetical protein LOAG_05163
Locus_194_Transcript_1/1_Conf_1.000	754	20	5.76E-55	CAR63677.1	89	218.394	124	111	putative SPErmatogenesis family member
Locus_1940_Transcript_1/1_Conf_1.000	258	0							
Locus_19400_Transcript_1/1_Conf_1.000	153	0							

Locus_19401_Transcript_1/1_Conf_1.000	571	20	3.05E-55	XP_002640062.1	81	218.394	161	131	C. briggsae CBR-UNC-120 protein
Locus_19402_Transcript_1/1_Conf_1.000	345	0							
Locus_19403_Transcript_1/1_Conf_1.000	357	3	1.38E-11	XP_002647203.1	72	72.7886	66	48	Hypothetical protein CBG22379
Locus_19404_Transcript_1/1_Conf_1.000	498	2	1.08E-16	NP_497632.1	64	89.7373	159	102	Kruppel-Like Factor (zinc finger protein) family member (klf-1)
Locus_19405_Transcript_1/1_Conf_1.000	447	10	1.29E-33	XP_002639456.1	69	145.976	152	106	Hypothetical protein CBG04051
Locus_19406_Transcript_1/1_Conf_1.000	263	0							
Locus_19407_Transcript_1/1_Conf_1.000	140	0							
Locus_19408_Transcript_1/1_Conf_1.000	144	0							
Locus_19409_Transcript_1/1_Conf_1.000	843	20	1.53E-97	CAA94853.5	79	360.147	266	211	C. elegans protein ZK856.5, confirmed by transcript evidence
Locus_1941_Transcript_1/2_Conf_1.000	3143	1	7.96E-05	XP_001902928.1	49	54.6842	137	68	hypothetical protein Bm1_57350
Locus_1941_Transcript_2/2_Conf_1.000	3120	1	7.89E-05	XP_001902928.1	49	54.6842	137	68	hypothetical protein Bm1_57350
Locus_19410_Transcript_1/1_Conf_1.000	249	0							
Locus_19411_Transcript_1/1_Conf_1.000	190	1	4.83E-04	XP_002646300.1	67	47.7506	49	33	Hypothetical protein CBG12007
Locus_19412_Transcript_1/1_Conf_1.000	362	20	1.50E-42	XP_001901345.1	84	175.637	120	101	Beta-tubulin cofactor D family protein

Locus_19413_Transcript_1/1_Conf_1.000	453	20	3.86E-22	NP_495283.2	57	107.842	139	80	C-type LECtin family member (clec-88)
Locus_19414_Transcript_1/1_Conf_1.000	385	0							
Locus_19415_Transcript_1/1_Conf_1.000	206	0							
Locus_19416_Transcript_1/1_Conf_1.000	671	20	3.96E-51	NP_505312.1	70	205.297	205	144	hypothetical protein T19A5.1
Locus_19417_Transcript_1/1_Conf_1.000	282	20	4.37E-21	XP_002642049.1	71	104.375	82	59	Hypothetical protein CBG17986
Locus_19418_Transcript_1/1_Conf_1.000	458	20	5.28E-72	XP_002638687.1	90	273.478	152	138	Hypothetical protein CBG11882
Locus_19419_Transcript_1/1_Conf_1.000	499	20	1.09E-85	XP_002644182.1	95	318.931	166	158	C. briggsae CBR-UNC-115 protein
Locus_1942_Transcript_1/1_Conf_1.000	667	20	1.15E-26	ACM08416.1	65	124.02	103	67	Lipopolysaccharide-induced tumor necrosis factor-alpha factor homolog
Locus_19420_Transcript_1/1_Conf_1.000	299	17	3.64E-20	EFO24930.1	66	101.293	106	70	hypothetical protein LOAG_03557
Locus_19421_Transcript_1/1_Conf_1.000	252	2	4.23E-16	XP_002630540.1	86	87.8113	66	57	Hypothetical protein CBG12981
Locus_19422_Transcript_1/1_Conf_1.000	174	20	3.48E-23	NP_498516.2	92	111.309	57	53	LEThal family member (let-711)
Locus_19423_Transcript_1/1_Conf_1.000	455	20	2.09E-68	NP_741692.1	88	261.536	151	133	TetraSPanin family member (tsp-13)
Locus_19424_Transcript_1/1_Conf_1.000	280	8	3.97E-11	XP_001894571.1	70	71.2478	62	44	UBX domain containing protein
Locus_19425_Transcript_1/1_Conf_1.000	263	0							
Locus_19426_Transcript_1/1_Conf_1.000	151	1	2.57E-05	XP_002647048.1	70	51.9878	51	36	Hypothetical protein CBG03566
Locus_19427_Transcript_1/1_Conf_1.000	168	0							
Locus_19428_Transcript_1/1_Conf_1.000	500	0							
Locus_19429_Transcript_1/1_Conf_1.000	332	4	4.26E-29	NP_496755.1	74	130.954	110	82	DAF-16/FOXO Controlled, germline Tumor affecting family member (dct-18)
Locus_1943_Transcript_1/1_Conf_1.000	142	0							
Locus_19430_Transcript_1/1_Conf_1.000	762	20	3.15E-40	XP_795502.1	57	169.474	217	124	PREDICTED: similar to ENSANGP00000010363
Locus_19431_Transcript_1/1_Conf_1.000	171	0							
Locus_19432_Transcript_1/1_Conf_1.000	383	20	7.01E-48	XP_002647751.1	84	193.356	126	106	C. briggsae CBR-CYP-42A1 protein
Locus_19433_Transcript_1/1_Conf_1.000	170	0							
Locus_19434_Transcript_1/1_Conf_1.000	294	20	1.74E-38	NP_505616.2	91	162.155	97	89	CULlin family member (cul-5)

Locus_19435_Transcript_1/1_Conf_1.000	165	0							
Locus_19436_Transcript_1/1_Conf_1.000	514	20	3.66E-42	NP_496592.1	82	174.481	120	99	hypothetical protein Y57A10A.15
Locus_19437_Transcript_1/1_Conf_1.000	137	1	3.61E-04	NP_504648.1	71	48.1358	45	32	Carnitine Palmitoyl Transferase family member (cpt-6)
Locus_19438_Transcript_1/1_Conf_1.000	146	0							
Locus_19439_Transcript_1/1_Conf_1.000	213	20	1.67E-17	P30646.4	75	92.4337	70	53	Uncharacterized sugar kinase R08D7.7
Locus_1944_Transcript_1/1_Conf_1.000	1698	20	0	XP_002632610.1	93	766.918	444	414	C. briggsae CBR-GDI-1 protein
Locus_19440_Transcript_1/1_Conf_1.000	253	0							
Locus_19441_Transcript_1/1_Conf_1.000	201	20	2.97E-22	NP_498931.2	84	108.227	65	55	SMAll family member (sma-2)
Locus_19442_Transcript_1/1_Conf_1.000	327	6	3.64E-12	XP_002629686.1	65	74.7146	82	54	Hypothetical protein CBG00906
Locus_19443_Transcript_1/1_Conf_1.000	338	20	1.19E-39	XP_002636314.1	89	166.007	111	99	Hypothetical protein CBG08607
Locus_19444_Transcript_1/1_Conf_1.000	235	0							
Locus_19445_Transcript_1/1_Conf_1.000	795	20	5.27E-65	NP_501024.1	67	251.906	264	179	hypothetical protein C01B10.8
Locus_19446_Transcript_1/1_Conf_1.000	233	20	3.19E-24	XP_002643253.1	96	114.775	59	57	C. briggsae CBR-AEX-3 protein

Locus_19447_Transcript_1/1_Conf_1.000	385	20	9.76E-42	NP_508178.2	79	172.94	128	102	TBC (Tre-2/Bub2/Cdc16) domain family member (tbc-11)
Locus_19448_Transcript_1/1_Conf_1.000	256	0							
Locus_19449_Transcript_1/1_Conf_1.000	1002	20	4.00E-85	NP_505523.1	71	319.316	308	219	hypothetical protein F46B6.4
Locus_1945_Transcript_1/2_Conf_1.000	1387	20	1.11E-36	NP_508806.1	73	159.073	187	137	DNaJ domain (prokaryotic heat shock protein) family member (dnj-14)
Locus_1945_Transcript_2/2_Conf_1.000	1388	20	1.11E-36	NP_508806.1	73	159.073	187	137	DNaJ domain (prokaryotic heat shock protein) family member (dnj-14)
Locus_19450_Transcript_1/1_Conf_1.000	423	20	2.27E-54	EFO24223.1	90	214.927	130	117	hypothetical protein LOAG_04263
Locus_19451_Transcript_1/1_Conf_1.000	147	6	3.04E-06	NP_741010.1	78	55.0694	38	30	hypothetical protein C56C10.7
Locus_19452_Transcript_1/2_Conf_1.000	257	0							
Locus_19452_Transcript_2/2_Conf_1.000	257	0							
Locus_19453_Transcript_1/1_Conf_1.000	142	20	2.10E-12	XP_002433850.1	85	75.485	47	40	conserved hypothetical protein
Locus_19454_Transcript_1/1_Conf_1.000	188	7	9.08E-27	XP_002640720.1	100	123.25	62	62	C. briggsae CBR-TAG-301 protein
Locus_19455_Transcript_1/1_Conf_1.000	804	20	5.82E-35	NP_508178.2	68	152.14	166	114	TBC (Tre-2/Bub2/Cdc16) domain family member (tbc-11)
Locus_19457_Transcript_1/1_Conf_1.000	437	0							
Locus_19458_Transcript_1/1_Conf_1.000	271	0							
Locus_19459_Transcript_1/1_Conf_1.000	362	0							
Locus_1946_Transcript_1/1_Conf_1.000	1208	20	1.24E-134	XP_002641737.1	78	484.182	362	284	C. briggsae CBR-DRS-1 protein
Locus_19460_Transcript_1/1_Conf_1.000	172	0							
Locus_19461_Transcript_1/1_Conf_1.000	725	0							
Locus_19462_Transcript_1/1_Conf_1.000	148	20	2.65E-18	NP_498711.1	85	95.1301	49	42	TYRosinase family member (tyr-1)
Locus_19463_Transcript_1/1_Conf_1.000	213	2	4.11E-08	NP_492278.2	71	61.2326	70	50	hypothetical protein T19A6.4
Locus_19464_Transcript_1/1_Conf_1.000	235	0							
Locus_19465_Transcript_1/1_Conf_1.000	172	0							
Locus_19466_Transcript_1/1_Conf_1.000	217	3	2.24E-14	XP_002634811.1	50	82.0333	112	57	Hypothetical protein CBG13916
Locus_19467_Transcript_1/1_Conf_1.000	248	0							
Locus_19468_Transcript_1/1_Conf_1.000	211	20	8.88E-11	EFO22061.1	61	70.0922	65	40	hypothetical protein LOAG_06422
Locus_19469_Transcript_1/1_Conf_1.000	194	2	4.35E-05	XP_001902022.1	56	51.2174	67	38	hypothetical protein Bm1_52770

Locus_1947_Transcript_1/1_Conf_1.000	1371	2	2.76E-80	XP_002634154.1	64	303.908	395	256	Hypothetical protein CBG01718
Locus_19470_Transcript_1/1_Conf_1.000	185	0							
Locus_19471_Transcript_1/1_Conf_1.000	540	20	3.41E-55	NP_498671.2	82	218.009	179	147	Uridine PhosPhorylase family member (upp-1)
Locus_19472_Transcript_1/1_Conf_1.000	144	20	3.48E-10	XP_002639439.1	78	68.1662	47	37	Hypothetical protein CBG04032
Locus_19473_Transcript_1/1_Conf_1.000	207	0							
Locus_19474_Transcript_1/1_Conf_1.000	138	20	1.67E-17	XP_002812491.1	100	92.4337	45	45	PREDICTED: c-X-C chemokine receptor type 4-like
Locus_19475_Transcript_1/1_Conf_1.000	299	0							
Locus_19476_Transcript_1/1_Conf_1.000	319	0							
Locus_19477_Transcript_1/1_Conf_1.000	1371	0							
Locus_19478_Transcript_1/1_Conf_1.000	424	20	5.26E-27	EFO24868.1	62	124.02	141	88	hypothetical protein LOAG_03617
Locus_19479_Transcript_1/1_Conf_1.000	235	4	5.44E-16	XP_002630800.1	90	87.4261	53	48	Hypothetical protein CBG02500
Locus_1948_Transcript_1/1_Conf_1.000	1671	20	3.76E-138	NP_001122655.1	64	496.508	566	367	Motor AXon guidance family member (max-2)

Locus_19480_Transcript_1/1_Conf_1.000	220	20	3.94E-38	XP_002831505.1	100	160.999	72	72	PREDICTED: diamine acetyltransferase 1-like
Locus_19481_Transcript_1/1_Conf_1.000	360	0							
Locus_19482_Transcript_1/1_Conf_1.000	139	0							
Locus_19483_Transcript_1/1_Conf_1.000	291	20	6.04E-23	XP_002645698.1	75	110.538	96	72	Hypothetical protein CBG07358
Locus_19484_Transcript_1/1_Conf_1.000	853	20	2.43E-66	NP_503353.2	68	256.529	264	180	hypothetical protein H24K24.4
Locus_19485_Transcript_1/1_Conf_1.000	133	4	4.14E-08	ABN08628.1	72	61.2326	43	31	RNA-directed DNA polymerase (Reverse transcriptase)
Locus_19486_Transcript_1/1_Conf_1.000	639	0							
Locus_19487_Transcript_1/1_Conf_1.000	256	0							
Locus_19488_Transcript_1/1_Conf_1.000	278	20	5.18E-27	EFO19307.1	87	124.02	93	81	hypothetical protein LOAG_09187
Locus_19489_Transcript_1/1_Conf_1.000	632	20	1.75E-79	XP_002640158.1	95	299.286	165	157	C. briggsae CBR-TRE-1 protein
Locus_1949_Transcript_1/1_Conf_1.000	398	4	2.23E-17	XP_001892578.1	57	92.0485	134	77	hypothetical protein Bm1_05520
Locus_19490_Transcript_1/1_Conf_1.000	156	3	2.38E-11	AAF66614.1	76	72.0182	52	40	AF142441_1H+/oligopeptide symporter OPT-3

Locus_19491_Transcript_1/1_Conf_1.000	139	20	2.75E-20	BAH13895.1	100	101.679	46	46	unnamed protein product
Locus_19492_Transcript_1/1_Conf_1.000	248	0							
Locus_19493_Transcript_1/1_Conf_1.000	308	5	7.99E-28	XP_001894154.1	75	126.716	101	76	hypothetical protein Bm1_13445
Locus_19494_Transcript_1/1_Conf_1.000	393	2	2.10E-07	XP_001895343.1	52	58.9214	106	56	hypothetical protein Bm1_19440
Locus_19495_Transcript_1/1_Conf_1.000	288	2	2.83E-04	NP_505787.1	61	48.521	77	47	hypothetical protein F58B4.4
Locus_19496_Transcript_1/1_Conf_1.000	165	0							
Locus_19497_Transcript_1/1_Conf_1.000	620	0							
Locus_19498_Transcript_1/1_Conf_1.000	305	0							
Locus_19499_Transcript_1/1_Conf_1.000	495	20	1.72E-38	XP_002642587.1	74	162.155	150	111	Hypothetical protein CBG09137
Locus_195_Transcript_1/1_Conf_1.000	942								

Locus_1950_Transcript_1/1_Conf_1.000	415	20	3.35E-18	NP_492436.1	56	94.7449	119	67	hypothetical protein W06D4.4
Locus_19500_Transcript_1/1_Conf_1.000	138	0							
Locus_19501_Transcript_1/1_Conf_1.000	433	20	1.32E-30	NP_498582.1	79	135.961	130	103	PreFolDin (molecular chaperone) family member (pfd-5)
Locus_19502_Transcript_1/1_Conf_1.000	386	8	1.20E-07	NP_492045.1	53	59.6918	128	68	hypothetical protein R06C7.1
Locus_19503_Transcript_1/1_Conf_1.000	132	0							
Locus_19504_Transcript_1/1_Conf_1.000	154	0							
Locus_19505_Transcript_1/1_Conf_1.000	1039	20	4.39E-50	XP_002638395.1	59	202.986	284	170	Hypothetical protein CBG18604

Locus_19506_Transcript_1/1_Conf_1.000	341	20	1.45E-37	NP_506256.3	82	159.073	113	93	CaDHerin family member (cdh-6)
Locus_19507_Transcript_1/1_Conf_1.000	953	20	1.82E-68	XP_002642783.1	70	263.848	273	193	C. briggsae CBR-GRO-1 protein
Locus_19508_Transcript_1/1_Conf_1.000	154	0							
Locus_19509_Transcript_1/1_Conf_1.000	205	0							
Locus_1951_Transcript_1/1_Conf_1.000	1290	20	1.52E-101	XP_002630594.1	68	374.4	400	275	C. briggsae CBR-AKA-1 protein
Locus_19510_Transcript_1/1_Conf_1.000	273	20	5.53E-45	NP_001021681.1	94	183.726	90	85	hypothetical protein Y105E8A.10
Locus_19511_Transcript_1/1_Conf_1.000	473	0							
Locus_19512_Transcript_1/1_Conf_1.000	352	0							
Locus_19513_Transcript_1/1_Conf_1.000	260	20	2.81E-28	XP_002412610.1	82	128.257	84	69	sorbitol dehydrogenase, putative
Locus_19514_Transcript_1/1_Conf_1.000	236	20	4.75E-20	NP_001023038.1	87	100.908	56	49	EXCretory canal abnormal family member (exc-5)
Locus_19515_Transcript_1/1_Conf_1.000	737	20	1.61E-70	NP_498895.1	77	270.011	245	191	RNA HelicAse family member (rha-2)
Locus_19516_Transcript_1/1_Conf_1.000	277	5	5.37E-16	EFO21774.1	87	87.4261	55	48	hypothetical protein LOAG_06715

Locus_19517_Transcript_1/1_Conf_1.000	241	0							
Locus_19518_Transcript_1/1_Conf_1.000	142	0							
Locus_19519_Transcript_1/1_Conf_1.000	238	0							
Locus_1952_Transcript_1/1_Conf_1.000	1130	20	2.88E-122	NP_502383.1	83	442.965	284	238	hypothetical protein C25G4.11
Locus_19520_Transcript_1/1_Conf_1.000	217	11	8.80E-19	XP_002640798.1	81	96.6709	71	58	C. briggsae CBR-NHR-121 protein
Locus_19521_Transcript_1/1_Conf_1.000	259	14	1.77E-14	XP_002635781.1	68	82.4185	88	60	Hypothetical protein CBG10437
Locus_19522_Transcript_1/1_Conf_1.000	180	0							
Locus_19523_Transcript_1/1_Conf_1.000	369	0							
Locus_19524_Transcript_1/1_Conf_1.000	421	20	3.78E-33	XP_002637497.1	69	144.436	137	95	Hypothetical protein CBG19218
Locus_19525_Transcript_1/1_Conf_1.000	335	4	2.51E-05	NP_740989.1	56	51.9878	76	43	hypothetical protein F07F6.8
Locus_19526_Transcript_1/1_Conf_1.000	319	20	9.91E-50	XP_002631126.1	97	199.519	106	103	C. briggsae CBR-SHN-1 protein
Locus_19527_Transcript_1/1_Conf_1.000	233	2	6.89E-19	NP_497911.1	72	97.0561	79	57	INSulin related family member (ins-17)
Locus_19528_Transcript_1/1_Conf_1.000	377	4	1.17E-26	NP_509961.1	80	122.865	100	80	hypothetical protein C44C10.9
Locus_19529_Transcript_1/1_Conf_1.000	295	20	6.44E-17	XP_002631513.1	64	90.5077	99	64	C. briggsae CBR-HDA-6 protein
Locus_1953_Transcript_1/1_Conf_1.000	554	0							
Locus_19530_Transcript_1/1_Conf_1.000	355	0							
Locus_19531_Transcript_1/1_Conf_1.000	606	20	5.80E-21	XP_002637610.1	61	104.76	142	87	C. briggsae CBR-TAG-120 protein
Locus_19532_Transcript_1/1_Conf_1.000	294	0							
Locus_19533_Transcript_1/1_Conf_1.000	234	1	2.52E-21	CAA26321.1	100	105.145	48	48	unnamed protein product
Locus_19534_Transcript_1/1_Conf_1.000	205	5	7.83E-07	XP_002644215.1	77	56.9954	44	34	C. briggsae CBR-ACL-5 protein

Locus_19535_Transcript_1/1_Conf_1.000	134	0							
Locus_19536_Transcript_1/1_Conf_1.000	320	2	8.78E-06	NP_510289.1	50	53.5286	67	34	hypothetical protein C04A11.2
Locus_19537_Transcript_1/1_Conf_1.000	406	2	8.67E-06	XP_001152656.1	49	53.5286	77	38	PREDICTED: similar to P antigen family, member 2B
Locus_19538_Transcript_1/1_Conf_1.000	262	0							
Locus_19539_Transcript_1/1_Conf_1.000	362	20	1.15E-34	NP_497034.1	74	149.443	119	89	hypothetical protein C01G12.8
Locus_1954_Transcript_1/2_Conf_1.000	425	0							
Locus_1954_Transcript_2/2_Conf_1.000	1076	20	1.83E-38	XP_002631192.1	87	164.466	98	86	C. briggsae CBR-CEY-1 protein
Locus_19540_Transcript_1/1_Conf_1.000	294	6	3.32E-05	CBM41222.1	50	51.6026	69	35	C. elegans protein T10G3.3b, partially confirmed by transcript evidence
Locus_19541_Transcript_1/1_Conf_1.000	327	0							
Locus_19542_Transcript_1/1_Conf_1.000	132	0							
Locus_19543_Transcript_1/1_Conf_1.000	508	20	6.96E-62	XP_002631239.1	81	239.965	164	133	C. briggsae CBR-LACT-3 protein
Locus_19544_Transcript_1/1_Conf_1.000	291	20	2.28E-38	XP_002634996.1	91	161.77	95	87	C. briggsae CBR-KGB-1 protein
Locus_19545_Transcript_1/2_Conf_1.000	454	0							
Locus_19545_Transcript_2/2_Conf_1.000	406	0							
Locus_19546_Transcript_1/1_Conf_1.000	242	0							
Locus_19547_Transcript_1/1_Conf_1.000	129	0							
Locus_19548_Transcript_1/1_Conf_1.000	162	0							
Locus_19549_Transcript_1/1_Conf_1.000	223	0							
Locus_1955_Transcript_1/1_Conf_1.000	380	0							
Locus_19550_Transcript_1/1_Conf_1.000	187	0							
Locus_19551_Transcript_1/1_Conf_1.000	359	0							
Locus_19552_Transcript_1/1_Conf_1.000	158	0							
Locus_19553_Transcript_1/1_Conf_1.000	566	4	3.30E-14	EFO21208.1	75	82.0333	69	52	hypothetical protein LOAG_07284
Locus_19554_Transcript_1/1_Conf_1.000	1027	7	7.43E-42	XP_002636786.1	63	175.637	176	112	Hypothetical protein CBG23525
Locus_19555_Transcript_1/1_Conf_1.000	494	20	8.25E-57	NP_506862.2	78	223.016	155	121	hypothetical protein F21A3.3

Locus_19556_Transcript_1/1_Conf_1.000	130	6	1.58E-07	XP_002633573.1	74	59.3066	43	32	Hypothetical protein CBG05447
Locus_19557_Transcript_1/1_Conf_1.000	226	0							
Locus_19558_Transcript_1/1_Conf_1.000	347	20	6.07E-15	EFA13656.1	60	83.9593	91	55	hypothetical protein TcasGA2_TC016129
Locus_19559_Transcript_1/1_Conf_1.000	222	0							
Locus_1956_Transcript_1/1_Conf_1.000	1180	20	2.71E-78	NP_503386.1	100	296.975	149	149	CalModulin family member (cmd-1)
Locus_19560_Transcript_1/1_Conf_1.000	490	20	9.23E-32	XP_002635570.1	67	139.813	122	82	Hypothetical protein CBG20553
Locus_19561_Transcript_1/1_Conf_1.000	133	0							
Locus_19562_Transcript_1/1_Conf_1.000	258	0							
Locus_19563_Transcript_1/2_Conf_1.000	369	0							
Locus_19563_Transcript_2/2_Conf_1.000	811	0							
Locus_19564_Transcript_1/1_Conf_1.000	200	4	2.80E-04	EFO16977.1	60	48.521	58	35	protein-tyrosine phosphatase

Locus_19565_Transcript_1/1_Conf_1.000	245	0							
Locus_19566_Transcript_1/1_Conf_1.000	227	20	3.80E-25	NP_491658.2	87	117.857	73	64	PaTched Related family member (ptr-10)
Locus_19567_Transcript_1/1_Conf_1.000	130	0							
Locus_19568_Transcript_1/1_Conf_1.000	354	20	1.57E-23	XP_002646774.1	74	112.464	116	86	Hypothetical protein CBG18418
Locus_19569_Transcript_1/1_Conf_1.000	319	20	1.26E-36	NP_509884.1	84	155.992	105	89	hypothetical protein F38B2.4
Locus_1957_Transcript_1/1_Conf_1.000	464	20	1.80E-59	NP_741743.1	84	231.876	152	129	Propionyl Coenzyme A Carboxylase Beta subunit family member (pccb-1)
Locus_19570_Transcript_1/1_Conf_1.000	139	0							
Locus_19571_Transcript_1/1_Conf_1.000	142	0							
Locus_19572_Transcript_1/1_Conf_1.000	158	0							
Locus_19573_Transcript_1/1_Conf_1.000	144	0							
Locus_19574_Transcript_1/1_Conf_1.000	702	20	1.17E-59	EFO26681.1	72	233.802	199	145	glycosyl transferase
Locus_19575_Transcript_1/1_Conf_1.000	417	20	3.42E-42	NP_495445.2	86	174.481	111	96	hypothetical protein ZK1127.5
Locus_19576_Transcript_1/1_Conf_1.000	148	0							
Locus_19577_Transcript_1/1_Conf_1.000	195	0							
Locus_19578_Transcript_1/1_Conf_1.000	313	1	1.01E-09	EDL06708.1	71	66.6254	56	40	mCG51545
Locus_19579_Transcript_1/1_Conf_1.000	219	0							
Locus_1958_Transcript_1/1_Conf_1.000	961	20	1.47E-65	NP_001129880.1	61	254.218	299	185	Nuclear eXport Factor family member (nxf-1)
Locus_19580_Transcript_1/1_Conf_1.000	321	20	1.21E-23	XP_002636926.1	84	112.849	83	70	Hypothetical protein CBG09393

Locus_19581_Transcript_1/1_Conf_1.000	253	20	3.94E-30	XP_001110023.2	100	134.42	82	82	PREDICTED: sentrin-specific protease 3-like isoform 3
Locus_19582_Transcript_1/1_Conf_1.000	492	2	1.38E-11	XP_001900176.1	50	72.7886	199	101	LIM domain containing protein
Locus_19583_Transcript_1/1_Conf_1.000	328	20	3.76E-17	EFN66847.1	60	91.2781	111	67	hypothetical protein EAG_08499
Locus_19584_Transcript_1/1_Conf_1.000	324	20	8.42E-09	XP_001960086.1	55	63.5438	98	54	GF13190
Locus_19585_Transcript_1/1_Conf_1.000	259	0							
Locus_19586_Transcript_1/1_Conf_1.000	429	0							
Locus_19587_Transcript_1/1_Conf_1.000	819	20	2.02E-83	XP_002643051.1	71	313.153	263	189	C. briggsae CBR-DPF-6 protein
Locus_19588_Transcript_1/1_Conf_1.000	247	7	3.46E-31	XP_002633317.1	85	137.887	82	70	Hypothetical protein CBG06054
Locus_19589_Transcript_1/1_Conf_1.000	480	20	6.47E-46	NP_510258.2	68	186.808	158	108	hypothetical protein F18H3.1
Locus_1959_Transcript_1/2_Conf_1.000	1056	20	1.69E-97	XP_002630503.1	81	360.533	291	238	Hypothetical protein CBG24740
Locus_1959_Transcript_2/2_Conf_1.000	1056	20	1.69E-97	XP_002630503.1	81	360.533	291	238	Hypothetical protein CBG24740
Locus_19590_Transcript_1/1_Conf_1.000	153	0							

Locus_19591_Transcript_1/1_Conf_1.000	151	5	4.68E-07	NP_500822.2	70	57.7658	48	34	UNCoordinated family member (unc-5)
Locus_19592_Transcript_1/1_Conf_1.000	132	0							
Locus_19593_Transcript_1/1_Conf_1.000	246	12	3.58E-28	XP_002633158.1	95	127.872	82	78	C. briggsae CBR-UNC-82 protein
Locus_19594_Transcript_1/1_Conf_1.000	214	20	9.44E-21	XP_002644316.1	88	103.219	71	63	C. briggsae CBR-TAG-312 protein
Locus_19595_Transcript_1/1_Conf_1.000	139	2	3.97E-11	NP_506168.2	91	71.2478	45	41	hypothetical protein K10C8.2
Locus_19596_Transcript_1/1_Conf_1.000	263	20	1.26E-28	BAE38513.1	98	129.413	66	65	unnamed protein product
Locus_19597_Transcript_1/1_Conf_1.000	238	0							
Locus_19598_Transcript_1/1_Conf_1.000	333	18	3.38E-10	XP_002637287.1	49	68.1662	117	58	Hypothetical protein CBG18974
Locus_19599_Transcript_1/1_Conf_1.000	291	2	1.59E-23	NP_508764.2	73	112.464	111	82	hypothetical protein F15A8.7
Locus_196_Transcript_1/1_Conf_1.000	597	4	5.39E-08	XP_002641461.1	44	61.6178	179	79	C. briggsae CBR-WAH-1 protein
Locus_1960_Transcript_1/4_Conf_0.222	253	0							
Locus_1960_Transcript_2/4_Conf_0.667	1220	8	8.46E-14	XP_002630572.1	44	82.8037	216	97	Hypothetical protein CBG13025
Locus_1960_Transcript_3/4_Conf_0.444	1126	9	7.54E-14	XP_002630572.1	44	82.8037	216	97	Hypothetical protein CBG13025
Locus_1960_Transcript_4/4_Conf_0.667	1130	9	7.57E-14	XP_002630572.1	44	82.8037	216	97	Hypothetical protein CBG13025
Locus_19600_Transcript_1/1_Conf_1.000	128	0							
Locus_19601_Transcript_1/1_Conf_1.000	651	20	4.12E-74	NP_510485.2	81	281.567	196	160	TBC (Tre-2/Bub2/Cdc16) domain family member (tbc-1)
Locus_19602_Transcript_1/1_Conf_1.000	161	0							
Locus_19603_Transcript_1/1_Conf_1.000	741	0							
Locus_19604_Transcript_1/1_Conf_1.000	134	0							
Locus_19605_Transcript_1/1_Conf_1.000	181	0							
Locus_19606_Transcript_1/1_Conf_1.000	294	20	1.34E-30	XP_002648619.1	78	135.961	98	77	Hypothetical protein CBG24962

Locus_19607_Transcript_1/1_Conf_1.000	484	14	4.95E-25	NP_492859.1	59	117.472	128	76	hypothetical protein Y52B11A.8
Locus_19608_Transcript_1/1_Conf_1.000	602	20	1.02E-62	NP_500647.2	83	243.432	156	131	Dipeptidyl Peptidase Four (IV) family member (dpf-5)
Locus_19609_Transcript_1/1_Conf_1.000	194	7	5.86E-18	NP_001021449.1	77	93.9745	62	48	hypothetical protein F35C12.3
Locus_1961_Transcript_1/1_Conf_1.000	2263	20	0	NP_506192.1	82	798.89	621	512	ABC transporter, class F family member (abcf-1)
Locus_19610_Transcript_1/1_Conf_1.000	134	0							
Locus_19611_Transcript_1/1_Conf_1.000	417	20	2.72E-23	AAK29933.3	81	111.694	83	68	Rab connectin related protein 2, partially confirmed by transcript evidence
Locus_19612_Transcript_1/1_Conf_1.000	370	0							
Locus_19613_Transcript_1/1_Conf_1.000	230	20	1.21E-39	ADN38247.1	100	166.007	76	76	MHC class I antigen
Locus_19614_Transcript_1/1_Conf_1.000	142	0							
Locus_19615_Transcript_1/1_Conf_1.000	418	4	2.31E-22	NP_508552.1	57	108.612	142	81	hypothetical protein F40F4.6
Locus_19616_Transcript_1/1_Conf_1.000	261	0							
Locus_19617_Transcript_1/1_Conf_1.000	777	9	3.41E-29	EFO22071.1	56	132.88	212	120	hypothetical protein LOAG_06415
Locus_19618_Transcript_1/1_Conf_1.000	139	0							
Locus_19619_Transcript_1/1_Conf_1.000	128	0							
Locus_1962_Transcript_1/1_Conf_1.000	378	20	7.28E-45	XP_002634052.1	87	183.341	124	108	C. briggsae CBR-VHA-5 protein
Locus_19620_Transcript_1/1_Conf_1.000	184	0							
Locus_19621_Transcript_1/1_Conf_1.000	136	2	1.47E-05	NP_505920.1	74	52.7582	43	32	hypothetical protein C55A6.6
Locus_19622_Transcript_1/1_Conf_1.000	563	20	1.04E-20	XP_001898035.1	53	103.605	156	84	hypothetical protein Bm1_32955

Locus_19623_Transcript_1/1_Conf_1.000	135	20	3.49E-07	EFO27090.1	82	58.151	35	29	WD repeats and SOF1 domain-containing protein
Locus_19624_Transcript_1/1_Conf_1.000	791	20	7.07E-38	XP_002631305.1	59	161.77	219	131	C. briggsae CBR-DNA-2 protein
Locus_19625_Transcript_1/1_Conf_1.000	320	20	1.34E-46	ACJ65168.1	93	189.119	106	99	Uncoordinated protein 68, isoform b, partially confirmed by transcript evidence
Locus_19626_Transcript_1/1_Conf_1.000	301	0							
Locus_19627_Transcript_1/1_Conf_1.000	167	11	6.63E-14	NP_497035.3	83	80.4925	55	46	Trehalose 6-Phosphate Synthase family member (tps-2)
Locus_19628_Transcript_1/1_Conf_1.000	281	0							
Locus_19629_Transcript_1/1_Conf_1.000	245	0							
Locus_1963_Transcript_1/1_Conf_1.000	474	3	2.58E-26	CAA85490.2	62	121.709	146	91	C. elegans protein C14B1.9, confirmed by transcript evidence
Locus_19630_Transcript_1/1_Conf_1.000	153	0							
Locus_19631_Transcript_1/1_Conf_1.000	141	0							
Locus_19632_Transcript_1/1_Conf_1.000	160	0							
Locus_19633_Transcript_1/1_Conf_1.000	142	3	3.96E-11	NP_505399.1	86	71.2478	44	38	hypothetical protein ZK682.2
Locus_19634_Transcript_1/1_Conf_1.000	372	0							
Locus_19635_Transcript_1/1_Conf_1.000	410	20	2.43E-08	XP_001198782.1	65	62.003	58	38	PREDICTED: similar to ENSANGP00000010363
Locus_19636_Transcript_1/1_Conf_1.000	154	2	2.73E-07	XP_002646320.1	64	58.5362	51	33	C. briggsae CBR-SMP-2 protein
Locus_19637_Transcript_1/1_Conf_1.000	312	2	1.16E-05	XP_002646277.1	67	53.1434	43	29	Hypothetical protein CBG11982
Locus_19638_Transcript_1/1_Conf_1.000	131	0							
Locus_19639_Transcript_1/1_Conf_1.000	188	20	1.04E-22	XP_002194320.1	100	109.768	51	51	PREDICTED: putative eukaryotic translation initiation factor 1 variant 1

Locus_1964_Transcript_1/1_Conf_1.000	410	5	9.83E-18	XP_002640436.1	61	93.2041	127	78	Hypothetical protein CBG08488
Locus_19640_Transcript_1/1_Conf_1.000	225	20	2.02E-18	EFO25092.1	78	95.5153	73	57	hypothetical protein LOAG_03389
Locus_19641_Transcript_1/1_Conf_1.000	363	20	1.27E-33	XP_002647703.1	89	145.976	86	77	Hypothetical protein CBG17892
Locus_19642_Transcript_1/1_Conf_1.000	140	0							
Locus_19643_Transcript_1/1_Conf_1.000	188	20	1.32E-09	EFO20907.1	74	66.2402	50	37	MH2 domain-containing protein
Locus_19644_Transcript_1/1_Conf_1.000	200	20	2.37E-11	XP_001901231.1	78	72.0182	60	47	cation channel family protein
Locus_19645_Transcript_1/1_Conf_1.000	279	20	5.31E-48	NP_502119.2	94	193.741	92	87	hypothetical protein F54D1.6
Locus_19646_Transcript_1/1_Conf_1.000	136	0							
Locus_19647_Transcript_1/1_Conf_1.000	321	0							
Locus_19648_Transcript_1/1_Conf_1.000	545	20	2.10E-60	NP_495125.1	80	235.343	181	146	hypothetical protein C18A3.3
Locus_19649_Transcript_1/1_Conf_1.000	157	0							
Locus_1965_Transcript_1/2_Conf_1.000	769	7	2.89E-49	NP_497909.1	64	199.519	208	134	Prion-like-(Q/N-rich)-domain-bearing protein family member (pqn-45)

Locus_1965_Transcript_2/2_Conf_1.000	781	8	6.02E-50	NP_497909.1	64	201.83	211	136	Prion-like-(Q/N-rich)-domain-bearing protein family member (pqn-45)
Locus_19650_Transcript_1/1_Conf_1.000	409	20	5.40E-16	NP_509229.1	58	87.4261	106	62	SUppressor of activated let-60 Ras family member (sur-5)
Locus_19651_Transcript_1/1_Conf_1.000	272	20	3.38E-26	CBW48351.1	80	121.324	86	69	Hypothetical protein C56A3.7c
Locus_19652_Transcript_1/1_Conf_1.000	235	0							
Locus_19653_Transcript_1/1_Conf_1.000	209	0							
Locus_19654_Transcript_1/1_Conf_1.000	158	0							
Locus_19655_Transcript_1/1_Conf_1.000	190	20	1.18E-18	EFO24000.1	87	96.2857	63	55	hypothetical protein LOAG_04486
Locus_19656_Transcript_1/1_Conf_1.000	380	0							
Locus_19657_Transcript_1/1_Conf_1.000	314	4	8.83E-14	NP_505776.1	89	80.1073	47	42	hypothetical protein F32D8.5
Locus_19658_Transcript_1/1_Conf_1.000	304	2	7.31E-05	NP_499025.1	81	50.447	44	36	hypothetical protein ZK512.2
Locus_19659_Transcript_1/1_Conf_1.000	434	20	7.25E-29	XP_001898123.1	70	130.183	145	102	hypothetical protein Bm1_33390

Locus_1966_Transcript_1/1_Conf_1.000	988	20	7.53E-113	XP_002633752.1	83	411.379	261	219	C. briggsae CBR-PYP-1 protein
Locus_19660_Transcript_1/1_Conf_1.000	394	0							
Locus_19661_Transcript_1/1_Conf_1.000	604	20	6.94E-43	NP_495318.2	72	177.563	186	135	hypothetical protein C56E6.2
Locus_19662_Transcript_1/1_Conf_1.000	198	0							
Locus_19663_Transcript_1/1_Conf_1.000	422	5	1.22E-23		63	112.849	141	90	myoblast city protein homolog
Locus_19664_Transcript_1/1_Conf_1.000	194	0							
Locus_19665_Transcript_1/1_Conf_1.000	284	20	6.73E-30	ABC69943.1	76	133.65	94	72	STIP
Locus_19666_Transcript_1/1_Conf_1.000	166	20	4.59E-15	XP_002632772.1	93	84.3445	47	44	C. briggsae CBR-TAT-2 protein
Locus_19667_Transcript_1/1_Conf_1.000	156	1	4.35E-05	EFO17124.1	59	51.2174	49	29	hypothetical protein LOAG_11376
Locus_19668_Transcript_1/1_Conf_1.000	200	0							
Locus_19669_Transcript_1/1_Conf_1.000	674	20	8.89E-59	XP_002642250.1	67	230.72	228	155	Hypothetical protein CBG18235

Locus_1967_Transcript_1/1_Conf_1.000	821	20	1.25E-133	XP_002646763.1	90	479.945	273	246	C. briggsae CBR-GSY-1 protein
Locus_19670_Transcript_1/1_Conf_1.000	573	4	2.31E-34	XP_002644385.1	56	149.058	187	106	Hypothetical protein CBG14225
Locus_19671_Transcript_1/1_Conf_1.000	420	0							
Locus_19672_Transcript_1/1_Conf_1.000	449	0							
Locus_19673_Transcript_1/1_Conf_1.000	309	20	2.85E-09	XP_002130995.1	87	65.0846	40	35	PREDICTED: transcription factor protein
Locus_19674_Transcript_1/2_Conf_1.000	307	20	2.85E-17	XP_002634069.1	65	91.6633	98	64	Hypothetical protein CBG01611
Locus_19674_Transcript_2/2_Conf_1.000	307	20	7.50E-18	XP_002634069.1	65	93.5893	98	64	Hypothetical protein CBG01611
Locus_19675_Transcript_1/1_Conf_1.000	629	20	3.05E-84	ACJ65168.1	87	315.079	208	182	Uncoordinated protein 68, isoform b, partially confirmed by transcript evidence
Locus_19676_Transcript_1/1_Conf_1.000	173	0							
Locus_19677_Transcript_1/1_Conf_1.000	517	20	2.43E-33	XP_001892323.1	80	145.206	109	88	Histone deacetylase family protein
Locus_19678_Transcript_1/1_Conf_1.000	259	0							
Locus_19679_Transcript_1/1_Conf_1.000	138	0							
Locus_1968_Transcript_1/1_Conf_1.000	1263	20	7.66E-58	XP_002631476.1	62	229.18	295	183	Hypothetical protein CBG20630
Locus_19680_Transcript_1/1_Conf_1.000	322	20	1.69E-41	NP_506927.2	91	172.17	106	97	HAlF transporter (PGP related) family member (haf-3)
Locus_19681_Transcript_1/1_Conf_1.000	670	1	2.27E-06	CAR63599.1	41	56.6102	169	70	hypothetical protein
Locus_19682_Transcript_1/1_Conf_1.000	258	4	6.71E-14	XP_002630479.1	78	80.4925	60	47	Hypothetical protein CBG11215
Locus_19683_Transcript_1/1_Conf_1.000	466	20	2.43E-48	NP_500129.1	77	194.897	152	118	hypothetical protein Y41D4A.6
Locus_19684_Transcript_1/1_Conf_1.000	521	12	4.56E-19	EFO16455.1	57	97.8265	156	89	hypothetical protein LOAG_12053

Locus_19685_Transcript_1/1_Conf_1.000	379	20	6.41E-25	XP_002642701.1	65	117.087	127	83	C. briggsae CBR-GLY-3 protein
Locus_19686_Transcript_1/1_Conf_1.000	294	8	5.11E-14	XP_002633220.1	84	80.8777	64	54	C. briggsae CBR-FLP-13 protein
Locus_19687_Transcript_1/1_Conf_1.000	283	0							
Locus_19688_Transcript_1/1_Conf_1.000	173	0							
Locus_19689_Transcript_1/1_Conf_1.000	253	5	1.72E-09	EFO26102.1	63	65.855	82	52	hypothetical protein LOAG_02385
Locus_1969_Transcript_1/1_Conf_1.000	591	0							
Locus_19690_Transcript_1/1_Conf_1.000	595	20	2.78E-49	XP_002634817.1	71	198.749	184	131	C. briggsae CBR-CLP-7 protein
Locus_19691_Transcript_1/1_Conf_1.000	316	0							
Locus_19692_Transcript_1/1_Conf_1.000	408	0							
Locus_19693_Transcript_1/1_Conf_1.000	608	0							
Locus_19694_Transcript_1/1_Conf_1.000	255	0							
Locus_19695_Transcript_1/1_Conf_1.000	227	4	1.83E-11	NP_501363.1	70	72.4034	61	43	P38 Map Kinase family member (pmk-3)
Locus_19696_Transcript_1/1_Conf_1.000	139	0							
Locus_19697_Transcript_1/1_Conf_1.000	146	20	2.18E-12	XP_002641621.1	75	75.485	48	36	Hypothetical protein CBG09941
Locus_19698_Transcript_1/1_Conf_1.000	529	2	7.02E-18	NP_001022051.1	56	93.9745	126	71	SREBP Cleavage activating Protein (SCAP) homolog family member (scp-1)
Locus_19699_Transcript_1/1_Conf_1.000	315	0							
Locus_197_Transcript_1/1_Conf_1.000	1044	20	5.64E-138	NP_001023493.1	84	494.967	327	277	hypothetical protein Y54G2A.2
Locus_1970_Transcript_1/1_Conf_1.000	673	0							
Locus_19700_Transcript_1/1_Conf_1.000	161	3	5.47E-08	NP_501376.2	71	60.8474	53	38	hypothetical protein C07G1.2
Locus_19701_Transcript_1/1_Conf_1.000	930	0							
Locus_19702_Transcript_1/2_Conf_1.000	338	4	5.93E-15	NP_493617.2	92	83.9593	50	46	hypothetical protein F33H2.6
Locus_19702_Transcript_2/2_Conf_1.000	338	4	5.93E-15	NP_493617.2	92	83.9593	50	46	hypothetical protein F33H2.6
Locus_19703_Transcript_1/1_Conf_1.000	817	20	2.09E-56	XP_001897058.1	81	223.402	182	149	SH3 domain-binding protein 5
Locus_19704_Transcript_1/1_Conf_1.000	282	0							
Locus_19705_Transcript_1/1_Conf_1.000	338	0							

Locus_19706_Transcript_1/1_Conf_1.000	565	20	7.32E-30	EAW75449.1	98	134.035	59	58	cathepsin Z, isoform CRA_b
Locus_19707_Transcript_1/1_Conf_1.000	351	20	5.46E-08	XP_001892050.1	56	60.8474	91	51	hypothetical protein Bm1_02740
Locus_19708_Transcript_1/1_Conf_1.000	266	0							
Locus_19709_Transcript_1/1_Conf_1.000	285	0							
Locus_1971_Transcript_1/1_Conf_1.000	734	20	1.79E-05	XP_002646359.1	47	53.9138	118	56	Hypothetical protein CBG12076
Locus_19710_Transcript_1/1_Conf_1.000	411	20	3.21E-69	AAC38987.1	97	264.233	136	133	P-glycoprotein
Locus_19711_Transcript_1/1_Conf_1.000	485	20	3.52E-55	NP_505451.1	80	217.624	161	129	hypothetical protein F11A3.1
Locus_19712_Transcript_1/1_Conf_1.000	252	12	1.66E-20	XP_002639310.1	82	102.449	68	56	Hypothetical protein CBG03881
Locus_19713_Transcript_1/1_Conf_1.000	425	1	3.00E-06	EFO20252.1	50	55.0694	132	67	hypothetical protein LOAG_08240
Locus_19714_Transcript_1/1_Conf_1.000	173	0							
Locus_19715_Transcript_1/1_Conf_1.000	264	0							
Locus_19716_Transcript_1/1_Conf_1.000	298	13	1.93E-13	XP_001901002.1	59	78.9518	99	59	WD40 associated region in TFIID subunit family protein
Locus_19717_Transcript_1/1_Conf_1.000	899	20	1.99E-122	NP_001041015.1	92	442.965	257	238	hypothetical protein Y116A8C.28

Locus_19718_Transcript_1/1_Conf_1.000	603	20	1.11E-48	NP_741839.2	50	196.823	242	123	hypothetical protein F48E3.8
Locus_19719_Transcript_1/1_Conf_1.000	145	0							
Locus_1972_Transcript_1/1_Conf_1.000	1429	20	6.15E-139	NP_694762.1	73	498.819	476	350	5-oxoprolinase
Locus_19720_Transcript_1/1_Conf_1.000	232	20	4.59E-39	ACX42645.1	100	164.081	76	76	MHC class I antigen
Locus_19721_Transcript_1/1_Conf_1.000	151	20	4.37E-13	XP_002635372.1	83	77.7962	42	35	C. briggsae CBR-RPM-1 protein
Locus_19722_Transcript_1/1_Conf_1.000	212	0							
Locus_19723_Transcript_1/1_Conf_1.000	198	20	3.53E-15	CBX25138.1	78	84.7297	64	50	Hypothetical protein C25A1.7a
Locus_19724_Transcript_1/1_Conf_1.000	137	0							
Locus_19725_Transcript_1/1_Conf_1.000	1016	20	7.30E-34	AAK84527.3	87	149.058	87	76	U40420_8Prion-like-(q/n-rich)-domain-bearing protein protein 37, partially confirmed by transcript evidence
Locus_19726_Transcript_1/1_Conf_1.000	130	0							
Locus_19727_Transcript_1/1_Conf_1.000	140	0							
Locus_19728_Transcript_1/1_Conf_1.000	1082	0							
Locus_19729_Transcript_1/1_Conf_1.000	207	0							
Locus_1973_Transcript_1/1_Conf_1.000	339	7	2.49E-05	EFO20860.1	42	51.9878	92	39	hypothetical protein LOAG_07630
Locus_19730_Transcript_1/1_Conf_1.000	227	20	1.82E-19	NP_495578.2	82	98.9821	63	52	hypothetical protein ZK1290.5
Locus_19731_Transcript_1/1_Conf_1.000	206	0							
Locus_19732_Transcript_1/1_Conf_1.000	270	20	7.28E-29	NP_504415.2	92	130.183	68	63	Na/Ca eXchangers family member (ncx-2)
Locus_19733_Transcript_1/1_Conf_1.000	359	5	4.28E-13	XP_002644294.1	74	77.7962	63	47	C. briggsae CBR-DPY-3 protein
Locus_19734_Transcript_1/1_Conf_1.000	195	20	1.75E-30	XP_002832361.1	100	135.576	64	64	PREDICTED: plexin-A3-like

Locus_19735_Transcript_1/1_Conf_1.000	249	3	4.38E-10	NP_493741.1	94	67.781	38	36	hypothetical protein W10D9.5
Locus_19736_Transcript_1/1_Conf_1.000	272	1	2.79E-04	NP_496062.3	68	48.521	50	34	Temporarily Assigned Gene name family member (tag-124)
Locus_19737_Transcript_1/1_Conf_1.000	236	2	7.09E-24	NP_500395.2	73	113.62	78	57	hypothetical protein Y37E11AL.6
Locus_19738_Transcript_1/1_Conf_1.000	343	3	8.18E-36	NP_501400.1	86	153.295	115	100	hypothetical protein F35H10.10
Locus_19739_Transcript_1/1_Conf_1.000	137	0							
Locus_1974_Transcript_1/1_Conf_1.000	1706	20	0	AAK17768.1	96	803.127	516	500	cytochrome c oxidase subunit I
Locus_19740_Transcript_1/1_Conf_1.000	372	0							
Locus_19741_Transcript_1/1_Conf_1.000	346	0							
Locus_19742_Transcript_1/1_Conf_1.000	199	0							
Locus_19743_Transcript_1/1_Conf_1.000	516	20	5.01E-71	CBL43419.1	92	270.396	153	141	C. elegans protein C47E8.4b, confirmed by transcript evidence
Locus_19744_Transcript_1/1_Conf_1.000	136	0							
Locus_19745_Transcript_1/1_Conf_1.000	178	20	4.10E-16	NP_509760.2	87	87.8113	56	49	Temporarily Assigned Gene name family member (tag-289)
Locus_19746_Transcript_1/1_Conf_1.000	548	20	7.68E-34	XP_002629803.1	62	147.132	185	115	Hypothetical protein CBG01047
Locus_19747_Transcript_1/1_Conf_1.000	414	9	1.01E-22	NP_509029.1	65	109.768	103	67	hypothetical protein C15H9.9

Locus_19748_Transcript_1/1_Conf_1.000	419	0								
Locus_19749_Transcript_1/1_Conf_1.000	297	20	7.08E-24	XP_002630866.1	82	113.62	79	65	C. briggsae CBR-HST-3.1 protein	
Locus_1975_Transcript_1/1_Conf_1.000	1557	20	0	NP_499778.1	88	767.303	521	460	CU (copper) ATPase family member (cua-1)	
Locus_19750_Transcript_1/1_Conf_1.000	527	6	1.02E-21	NP_001023604.1	88	106.686	75	66	hypothetical protein ZC477.3	
Locus_19751_Transcript_1/1_Conf_1.000	226	3	1.00E-09	CAR64255.1	93	66.6254	48	45	protein RIO1 kinase	
Locus_19752_Transcript_1/1_Conf_1.000	261	0								
Locus_19753_Transcript_1/1_Conf_1.000	142	20	7.96E-20	DAA21858.1	100	100.138	47	47	eukaryotic translation initiation factor 3 subunit M	
Locus_19754_Transcript_1/1_Conf_1.000	411	0								
Locus_19755_Transcript_1/1_Conf_1.000	139	0								
Locus_19756_Transcript_1/1_Conf_1.000	212	0								
Locus_19757_Transcript_1/1_Conf_1.000	270	0								

Locus_19758_Transcript_1/1_Conf_1.000	182	20	1.51E-29	BAG65408.1	100	132.494	60	60	unnamed protein product
Locus_19759_Transcript_1/1_Conf_1.000	303	20	1.42E-16	XP_002642179.1	62	89.3521	99	62	C. briggsae CBR-CUTC-1 protein
Locus_1976_Transcript_1/3_Conf_0.500	2042	20	7.90E-40	NP_495036.3	69	170.244	172	120	human DAZ (Deleted in Azoospermia) homolog family member (daz-1)
Locus_1976_Transcript_2/3_Conf_0.667	1754	20	6.53E-40	NP_495036.3	69	170.244	172	120	human DAZ (Deleted in Azoospermia) homolog family member (daz-1)
Locus_1976_Transcript_3/3_Conf_0.667	1766	20	6.59E-40	NP_495036.3	69	170.244	172	120	human DAZ (Deleted in Azoospermia) homolog family member (daz-1)
Locus_19760_Transcript_1/1_Conf_1.000	167	0							
Locus_19761_Transcript_1/1_Conf_1.000	189	2	3.69E-12	XP_002634423.1	84	74.7146	59	50	C. briggsae CBR-PTR-14 protein
Locus_19762_Transcript_1/1_Conf_1.000	175	0							
Locus_19763_Transcript_1/1_Conf_1.000	129	0							
Locus_19764_Transcript_1/1_Conf_1.000	238	0							
Locus_19765_Transcript_1/1_Conf_1.000	356	0							
Locus_19766_Transcript_1/1_Conf_1.000	542	20	6.07E-20	XP_002637670.1	55	100.908	161	89	C. briggsae CBR-RDE-1 protein
Locus_19767_Transcript_1/1_Conf_1.000	258	20	8.13E-44	XP_002810702.1	100	179.874	79	79	PREDICTED: protein CYR61-like isoform 3
Locus_19768_Transcript_1/1_Conf_1.000	471	0							
Locus_19769_Transcript_1/1_Conf_1.000	141	0							
Locus_1977_Transcript_1/1_Conf_1.000	449	0							

Locus_19770_Transcript_1/1_Conf_1.000	382	20	1.37E-27	NP_001021054.1	70	125.946	120	85	Suppressor with Morphological effect on Genitalia family member (smg-1)
Locus_19771_Transcript_1/1_Conf_1.000	146	0							
Locus_19772_Transcript_1/1_Conf_1.000	164	20	1.14E-13	XP_002647593.1	93	79.7221	45	42	C. briggsae CBR-TTR-44 protein
Locus_19773_Transcript_1/1_Conf_1.000	260	0							
Locus_19774_Transcript_1/1_Conf_1.000	174	0							
Locus_19775_Transcript_1/1_Conf_1.000	465	20	6.79E-75	A8XJL7.2	90	283.108	154	140	Serine/threonine-protein kinase sax-1
Locus_19776_Transcript_1/1_Conf_1.000	332	20	1.72E-54	NP_498645.1	93	215.312	110	103	MUscle Positioning family member (mup-4)
Locus_19777_Transcript_1/1_Conf_1.000	173	0							
Locus_19778_Transcript_1/1_Conf_1.000	186	0							
Locus_19779_Transcript_1/1_Conf_1.000	646	20	1.30E-64	AAK62032.1	69	249.98	209	145	metalloprotease 1 precursor
Locus_1978_Transcript_1/2_Conf_1.000	1440	20	7.17E-111	NP_498930.1	75	405.601	345	260	UDP-Galactose Transporter Protein family member (ugtp-1)
Locus_1978_Transcript_2/2_Conf_1.000	1440	20	7.17E-111	NP_498930.1	75	405.601	345	260	UDP-Galactose Transporter Protein family member (ugtp-1)
Locus_19780_Transcript_1/1_Conf_1.000	181	5	1.47E-08	XP_002641239.1	73	62.7734	56	41	Hypothetical protein CBG09106
Locus_19781_Transcript_1/1_Conf_1.000	233	20	5.83E-18	Q17678.6	80	93.9745	71	57	UPF0632 protein C05G5.5
Locus_19782_Transcript_1/1_Conf_1.000	210	20	1.57E-07	NP_500129.1	75	59.3066	45	34	hypothetical protein Y41D4A.6
Locus_19783_Transcript_1/1_Conf_1.000	357	3	9.87E-10	XP_002642316.1	59	66.6254	88	52	Hypothetical protein CBG18309
Locus_19784_Transcript_1/1_Conf_1.000	236	0							
Locus_19785_Transcript_1/1_Conf_1.000	465	0							
Locus_19786_Transcript_1/1_Conf_1.000	159	0							
Locus_19787_Transcript_1/1_Conf_1.000	233	0							
Locus_19788_Transcript_1/1_Conf_1.000	380	6	2.36E-11	NP_001021785.2	53	72.0182	108	58	hypothetical protein Y52B11A.3
Locus_19789_Transcript_1/1_Conf_1.000	817	20	7.22E-41	XP_002629747.1	58	171.785	259	151	Hypothetical protein CBG00981
Locus_1979_Transcript_1/1_Conf_1.000	568	0							
Locus_19790_Transcript_1/1_Conf_1.000	420	1	1.76E-14	ABV03827.1	84	82.4185	51	43	hypothetical protein

Locus_19791_Transcript_1/1_Conf_1.000	414	0							
Locus_19792_Transcript_1/1_Conf_1.000	401	20	4.60E-31	NP_496981.2	86	137.502	115	99	hypothetical protein Y54G11A.9
Locus_19793_Transcript_1/1_Conf_1.000	308	2	3.59E-28	NP_495419.1	78	127.872	100	78	hypothetical protein B0252.1
Locus_19794_Transcript_1/1_Conf_1.000	283	4	3.98E-06	NP_501765.1	60	54.6842	73	44	hypothetical protein K07F5.7
Locus_19795_Transcript_1/1_Conf_1.000	176	0							
Locus_19796_Transcript_1/1_Conf_1.000	159	0							
Locus_19797_Transcript_1/1_Conf_1.000	236	5	5.81E-18	XP_002644134.1	74	93.9745	78	58	C. briggsae CBR-SYD-2 protein
Locus_19798_Transcript_1/1_Conf_1.000	163	0							
Locus_19799_Transcript_1/1_Conf_1.000	367	20	1.47E-25	NP_493353.3	92	86.6557	41	38	Specificity Protein) Transcription Factor family member (sptf-3)
Locus_198_Transcript_1/1_Conf_1.000	1850	20	0	XP_002640154.1	85	803.127	514	441	Hypothetical protein CBG12654
Locus_1980_Transcript_1/1_Conf_1.000	513	0							
Locus_19800_Transcript_1/1_Conf_1.000	777	20	6.42E-28	XP_001896097.1	58	128.642	229	133	Nsp1-like C-terminal region family protein
Locus_19801_Transcript_1/1_Conf_1.000	276	10	4.11E-24	XP_002637177.1	76	114.39	94	72	Hypothetical protein CBG09695
Locus_19802_Transcript_1/1_Conf_1.000	280	0							
Locus_19803_Transcript_1/1_Conf_1.000	223	20	3.82E-17	EFO14895.1	75	91.2781	73	55	CMGC/CDK/CDK9 protein kinase
Locus_19804_Transcript_1/1_Conf_1.000	1239	20	1.25E-145	NP_495680.1	76	520.776	419	319	Nose Resistant to Fluoxetine family member (nrf-6)
Locus_19805_Transcript_1/1_Conf_1.000	280	20	5.18E-19	XP_002645153.1	79	97.4413	69	55	C. briggsae CBR-SFXN-2 protein
Locus_19806_Transcript_1/1_Conf_1.000	446	13	8.34E-57	NP_503543.1	85	223.016	149	128	hypothetical protein T08B1.1

Locus_19807_Transcript_1/1_Conf_1.000	276	20	1.61E-20	XP_001893670.1	75	102.449	92	69	Conserved oligomeric Golgi complex component 6
Locus_19808_Transcript_1/1_Conf_1.000	184	0							
Locus_19809_Transcript_1/1_Conf_1.000	182	0							
Locus_1981_Transcript_1/1_Conf_1.000	1694	20	3.60E-120	XP_002639089.1	89	436.802	269	241	C. briggsae CBR-TAG-163 protein
Locus_19810_Transcript_1/1_Conf_1.000	276	0							
Locus_19811_Transcript_1/1_Conf_1.000	263	0							
Locus_19812_Transcript_1/1_Conf_1.000	325	20	1.69E-25	XP_970656.1	71	119.013	108	77	PREDICTED: similar to Histone deacetylase 11 (HD11)
Locus_19813_Transcript_1/1_Conf_1.000	773	20	1.22E-47	EFO28238.1	82	194.126	141	117	rho GDP-dissociation inhibitor 2
Locus_19814_Transcript_1/1_Conf_1.000	800	13	1.24E-13	NP_504836.1	47	81.2629	254	120	GEX Interacting protein family member (gei-6)
Locus_19815_Transcript_1/1_Conf_1.000	190	20	4.98E-17	AAI43755.1	100	90.8929	63	63	FN1 protein
Locus_19816_Transcript_1/1_Conf_1.000	501	20	3.42E-42	XP_002630693.1	75	174.481	157	118	C. briggsae CBR-SRG-69 protein
Locus_19817_Transcript_1/1_Conf_1.000	325	20	7.79E-47	ADN00780.1	89	189.889	107	96	class 3 lipase protein
Locus_19818_Transcript_1/1_Conf_1.000	462	8	4.64E-23	CAR81373.1	66	110.923	153	102	C. elegans protein F58H1.8, confirmed by transcript evidence
Locus_19819_Transcript_1/1_Conf_1.000	564	11	2.70E-16	XP_002635081.1	50	88.9669	178	90	Hypothetical protein CBG11298
Locus_1982_Transcript_1/1_Conf_1.000	956	6	5.94E-35	NP_001040920.1	54	152.525	217	119	hypothetical protein C35B1.2
Locus_19820_Transcript_1/1_Conf_1.000	202	0							
Locus_19821_Transcript_1/1_Conf_1.000	336	0							

Locus_19822_Transcript_1/1_Conf_1.000	501	4	1.59E-39	NP_871808.3	62	165.622	192	120	hypothetical protein ZC123.4
Locus_19823_Transcript_1/1_Conf_1.000	211	0							
Locus_19824_Transcript_1/1_Conf_1.000	176	0							
Locus_19825_Transcript_1/1_Conf_1.000	147	0							
Locus_19826_Transcript_1/1_Conf_1.000	237	6	2.60E-18	NP_001022476.1	69	95.1301	86	60	Daf-16-Dependent Longevity (WT but not daf-16 lifespan increased) family member (ddl-3)
Locus_19827_Transcript_1/1_Conf_1.000	220	1	4.86E-04	EFO20896.1	67	47.7506	53	36	hypothetical protein LOAG_07593
Locus_19828_Transcript_1/1_Conf_1.000	208	0							
Locus_19829_Transcript_1/1_Conf_1.000	287	0							
Locus_1983_Transcript_1/1_Conf_1.000	1354	20	4.22E-158	XP_002630362.1	86	562.377	406	352	C. briggsae CBR-RPN-5 protein
Locus_19830_Transcript_1/1_Conf_1.000	134	20	1.98E-10	CAG09075.1	81	68.9366	44	36	unnamed protein product
Locus_19831_Transcript_1/1_Conf_1.000	291	0							
Locus_19832_Transcript_1/1_Conf_1.000	284	20	1.36E-30	Q22141.2	81	135.961	91	74	Post-GPI attachment to proteins factor 2
Locus_19833_Transcript_1/1_Conf_1.000	346	0							
Locus_19834_Transcript_1/1_Conf_1.000	201	0							
Locus_19835_Transcript_1/1_Conf_1.000	139	2	4.26E-05	NP_499273.3	77	51.2174	36	28	hypothetical protein R01H10.7
Locus_19836_Transcript_1/1_Conf_1.000	150	0							
Locus_19837_Transcript_1/1_Conf_1.000	274	1	2.78E-04	XP_002640131.1	60	48.521	84	51	Hypothetical protein CBG12630
Locus_19838_Transcript_1/1_Conf_1.000	321	1	4.80E-04	NP_495712.1	57	47.7506	66	38	abnormal EMBroygenesis family member (emb-27)
Locus_19839_Transcript_1/1_Conf_1.000	139	7	8.57E-06	XP_002646356.1	69	53.5286	46	32	Hypothetical protein CBG12070
Locus_1984_Transcript_1/1_Conf_1.000	772	20	2.58E-13	NP_501245.1	57	80.1073	155	89	HMG family member (hmg-5)
Locus_19840_Transcript_1/1_Conf_1.000	276	0							

Locus_19841_Transcript_1/1_Conf_1.000	498	20	1.89E-37	CAA10033.1	66	158.688	163	108	DYS-1 protein
Locus_19842_Transcript_1/1_Conf_1.000	170	1	3.64E-04	XP_002642935.1	68	48.1358	54	37	C. briggsae CBR-NSH-1 protein
Locus_19843_Transcript_1/1_Conf_1.000	294	0							
Locus_19844_Transcript_1/1_Conf_1.000	431	20	9.88E-10	ABG23688.1	50	66.6254	150	76	RNA-dependent RNA polymerase
Locus_19845_Transcript_1/1_Conf_1.000	260	0							
Locus_19846_Transcript_1/1_Conf_1.000	222	4	1.11E-24	XP_001902169.1	89	116.316	73	65	myotactin form B
Locus_19847_Transcript_1/1_Conf_1.000	289	20	2.99E-46	AAY56742.1	96	187.963	96	93	casein kinase I delta isoform
Locus_19848_Transcript_1/1_Conf_1.000	135	1	6.15E-12	EDL23991.1	89	73.9442	38	34	transmembrane protein 147, isoform CRA_a
Locus_19849_Transcript_1/1_Conf_1.000	161	0							
Locus_1985_Transcript_1/1_Conf_1.000	1235	20	3.49E-23	XP_001897562.1	55	114.005	181	101	TSC-22/dip/bun family protein
Locus_19850_Transcript_1/1_Conf_1.000	135	6	2.67E-15	AAA18259.1	90	85.1149	44	40	protein kinase C
Locus_19851_Transcript_1/1_Conf_1.000	168	20	1.85E-24	XP_002816715.1	100	115.546	53	53	PREDICTED: complement C4-B isoform 2

Locus_19852_Transcript_1/1_Conf_1.000	386	20	2.51E-13	XP_002636676.1	54	78.5666	114	62	Hypothetical protein CBG23390
Locus_19853_Transcript_1/1_Conf_1.000	129	0							
Locus_19854_Transcript_1/1_Conf_1.000	298	20	1.16E-50	2BDW	98	202.601	98	97	CrystalStructure OfThe Auto-Inhibited Kinase Domain Of CalciumCALMODULIN ACTIVATED KINASE II
Locus_19855_Transcript_1/1_Conf_1.000	185	0							
Locus_19856_Transcript_1/1_Conf_1.000	145	4	8.58E-09	EFO19864.1	72	63.5438	47	34	hypothetical protein LOAG_08632
Locus_19857_Transcript_1/1_Conf_1.000	415	0							
Locus_19858_Transcript_1/1_Conf_1.000	391	0							
Locus_19859_Transcript_1/1_Conf_1.000	164	0							
Locus_1986_Transcript_1/1_Conf_1.000	1046	20	2.53E-21	NP_496326.1	48	107.457	221	108	hypothetical protein R06F6.6
Locus_19860_Transcript_1/1_Conf_1.000	309	0							

Locus_19861_Transcript_1/1_Conf_1.000	645	20	9.10E-26	XP_001627842.1	69	120.939	117	81	predicted protein
Locus_19862_Transcript_1/1_Conf_1.000	154	0							
Locus_19863_Transcript_1/1_Conf_1.000	226	0							
Locus_19864_Transcript_1/1_Conf_1.000	366	0							
Locus_19865_Transcript_1/1_Conf_1.000	238	20	3.62E-28	CAA30855.1	90	127.872	76	69	myosin heavy chain 2
Locus_19866_Transcript_1/1_Conf_1.000	371	3	1.77E-22	XP_002639247.1	73	108.997	101	74	Hypothetical protein CBG03806
Locus_19867_Transcript_1/1_Conf_1.000	258	0							
Locus_19868_Transcript_1/1_Conf_1.000	230	0							
Locus_19869_Transcript_1/1_Conf_1.000	177	6	3.97E-11	XP_002640845.1	74	71.2478	58	43	C. briggsae CBR-CDH-1 protein
Locus_1987_Transcript_1/1_Conf_1.000	672	20	5.80E-34	CAA21654.2	67	148.288	177	120	C. elegans protein Y48A6C.4, confirmed by transcript evidence
Locus_19870_Transcript_1/1_Conf_1.000	275	0							
Locus_19871_Transcript_1/1_Conf_1.000	211	20	1.12E-21	NP_001090349.1	81	106.301	70	57	sideroflexin 1
Locus_19872_Transcript_1/1_Conf_1.000	306	0							

Locus_19873_Transcript_1/1_Conf_1.000	138	0							
Locus_19874_Transcript_1/1_Conf_1.000	153	5	2.09E-07	CAH04706.3	74	58.9214	47	35	C. elegans protein K04H4.2c, partially confirmed by transcript evidence
Locus_19875_Transcript_1/1_Conf_1.000	378	0							
Locus_19876_Transcript_1/1_Conf_1.000	243	0							
Locus_19877_Transcript_1/1_Conf_1.000	527	20	6.37E-48	XP_002633219.1	71	193.741	178	127	C. briggsae CBR-ITR-1 protein
Locus_19878_Transcript_1/1_Conf_1.000	149	0							
Locus_19879_Transcript_1/1_Conf_1.000	430	20	1.78E-59	ADL62853.1	86	231.876	140	121	thioredoxin reductase 2
Locus_1988_Transcript_1/1_Conf_1.000	893	20	4.50E-26	EFO21777.1	100	122.865	53	53	zinc finger protein
Locus_19880_Transcript_1/1_Conf_1.000	268	20	1.69E-17	NP_501552.1	81	92.4337	91	74	hypothetical protein D1046.3
Locus_19881_Transcript_1/1_Conf_1.000	239	0							
Locus_19882_Transcript_1/1_Conf_1.000	303	20	1.68E-17	NP_491872.1	56	92.4337	103	58	hypothetical protein C55B7.3
Locus_19883_Transcript_1/1_Conf_1.000	224	0							
Locus_19884_Transcript_1/1_Conf_1.000	129	0							
Locus_19885_Transcript_1/1_Conf_1.000	167	4	1.80E-19	XP_002639744.1	85	98.9821	54	46	C. briggsae CBR-LAM-3 protein
Locus_19886_Transcript_1/1_Conf_1.000	276	0							
Locus_19887_Transcript_1/1_Conf_1.000	390	20	7.41E-42	NP_496968.1	86	173.326	109	94	hypothetical protein Y48B6A.12
Locus_19888_Transcript_1/1_Conf_1.000	245	0							
Locus_19889_Transcript_1/1_Conf_1.000	330	0							
Locus_1989_Transcript_1/1_Conf_1.000	821	20	1.99E-22	XP_001894821.1	67	110.538	100	67	MGC80968 protein
Locus_19890_Transcript_1/1_Conf_1.000	302	0							
Locus_19891_Transcript_1/1_Conf_1.000	250	20	6.30E-25	NP_776251.1	100	117.087	83	83	keratinocyte-associated protein 2

Locus_19892_Transcript_1/1_Conf_1.000	353	20	6.01E-23	XP_002648880.1	69	110.538	101	70	C. briggsae CBR-CYP-33A1 protein
Locus_19893_Transcript_1/1_Conf_1.000	355	4	4.42E-34	NP_001033358.2	73	147.517	118	87	DAF-16/FOXO Controlled, germline Tumor affecting family member (dct-6)
Locus_19894_Transcript_1/1_Conf_1.000	812	20	3.69E-122	XP_002646641.1	87	441.81	270	235	Hypothetical protein CBG11073
Locus_19895_Transcript_1/1_Conf_1.000	140	0							
Locus_19896_Transcript_1/1_Conf_1.000	244	0							
Locus_19897_Transcript_1/1_Conf_1.000	357	0							
Locus_19898_Transcript_1/1_Conf_1.000	166	0							
Locus_19899_Transcript_1/1_Conf_1.000	400	20	2.58E-66	NP_501549.1	99	254.603	130	129	RAP homolog (vertebrate Rap GTPase family) family member (rap-1)
Locus_199_Transcript_1/2_Conf_1.000	814	0							
Locus_199_Transcript_2/2_Conf_1.000	813	0							
Locus_1990_Transcript_1/1_Conf_1.000	2284	20	1.31E-139	XP_001901168.1	72	501.901	470	342	Groucho/TLE N-terminal Q-rich domain containing protein
Locus_19900_Transcript_1/1_Conf_1.000	141	0							
Locus_19901_Transcript_1/1_Conf_1.000	254	0							
Locus_19902_Transcript_1/1_Conf_1.000	219	0							
Locus_19903_Transcript_1/1_Conf_1.000	560	0							
Locus_19904_Transcript_1/1_Conf_1.000	350	20	1.87E-32	NP_490888.1	76	142.124	116	89	Mitochondrial Processing Peptidase Alpha family member (mppa-1)
Locus_19905_Transcript_1/1_Conf_1.000	293	20	2.00E-34	NP_502428.1	83	148.673	97	81	hypothetical protein C27D8.1
Locus_19906_Transcript_1/1_Conf_1.000	427	0							

Locus_19907_Transcript_1/1_Conf_1.000	471	20	3.03E-59	NP_001040658.2	95	231.106	129	123	High Incidence of Males (increased X chromosome loss) family member (him-1)
Locus_19908_Transcript_1/1_Conf_1.000	219	20	5.88E-34	XP_001110023.2	100	147.132	73	73	PREDICTED: sentrin-specific protease 3-like isoform 3
Locus_19909_Transcript_1/1_Conf_1.000	139	0							
Locus_1991_Transcript_1/1_Conf_1.000	1973	20	0	NP_001022469.1	84	809.29	541	456	Eukaryotic Initiation Factor family member (eif-3.B)
Locus_19910_Transcript_1/1_Conf_1.000	205	20	6.60E-22	NP_501860.1	88	107.071	68	60	Mitochondrial Sorting of Proteins (yeast MSP) in Nematode family member (mspn-1)
Locus_19911_Transcript_1/1_Conf_1.000	281	20	3.59E-12	XP_002631607.1	94	74.7146	39	37	Hypothetical protein CBG20788

Locus_19912_Transcript_1/1_Conf_1.000	189	7	4.66E-15	XP_002630336.1	77	84.3445	62	48	Hypothetical protein CBG04265
Locus_19913_Transcript_1/1_Conf_1.000	262	0							
Locus_19914_Transcript_1/1_Conf_1.000	377	0							
Locus_19915_Transcript_1/1_Conf_1.000	136	10	1.33E-06	NP_001183823.1	70	56.225	41	29	hypothetical protein LOC100502416
Locus_19916_Transcript_1/1_Conf_1.000	222	20	5.66E-37	NP_001122684.1	100	157.147	74	74	UNCoordinated family member (unc-103)
Locus_19917_Transcript_1/1_Conf_1.000	400	4	3.42E-18	XP_001892604.1	62	94.7449	135	85	Phosphatidylinositol 3- and 4-kinase family protein
Locus_19918_Transcript_1/1_Conf_1.000	306	0							
Locus_19919_Transcript_1/1_Conf_1.000	240	0							
Locus_1992_Transcript_1/1_Conf_1.000	2187	20	9.98E-89	NP_740833.1	58	332.798	472	276	hypothetical protein F53F10.2
Locus_19920_Transcript_1/1_Conf_1.000	342	20	9.33E-40	NP_501928.1	83	166.392	114	95	hypothetical protein F13B12.4
Locus_19921_Transcript_1/1_Conf_1.000	288	0							
Locus_19922_Transcript_1/1_Conf_1.000	259	0							
Locus_19923_Transcript_1/1_Conf_1.000	423	20	2.26E-70	XP_001142154.1	98	268.085	136	134	PREDICTED: similar to Thioredoxin (ATL-derived factor) (ADF) (Surface-associated sulphydryl protein) (SASP)
Locus_19924_Transcript_1/2_Conf_1.000	477	0							
Locus_19924_Transcript_2/2_Conf_1.000	439	0							
Locus_19925_Transcript_1/1_Conf_1.000	298	0							
Locus_19926_Transcript_1/1_Conf_1.000	221	0							
Locus_19927_Transcript_1/1_Conf_1.000	575	5	4.24E-20	NP_001021744.1	68	101.679	103	71	abnormal cell MIGration family member (mig-1)

Locus_19928_Transcript_1/1_Conf_1.000	210	19	1.01E-30	NP_506637.1	98	136.346	68	67	One IG domain family member (oig-3)
Locus_19929_Transcript_1/1_Conf_1.000	291	20	6.22E-36	EAW47423.1	100	153.68	78	78	proteasome (prosome, macropain) subunit, beta type, 1, isoform CRA_d
Locus_1993_Transcript_1/1_Conf_1.000	434	0							
Locus_19930_Transcript_1/1_Conf_1.000	245	0							
Locus_19931_Transcript_1/1_Conf_1.000	559	0							
Locus_19932_Transcript_1/1_Conf_1.000	477	8	2.04E-15	EFO24135.1	72	85.5001	80	58	hypothetical protein LOAG_04354
Locus_19933_Transcript_1/1_Conf_1.000	318	2	4.66E-07	NP_494276.2	63	57.7658	98	62	Variable ABnormal morphology family member (vab-19)
Locus_19934_Transcript_1/1_Conf_1.000	690	20	1.83E-94	XP_001895028.1	88	349.362	226	199	cytoplasmic polyadenylation element binding protein 4
Locus_19935_Transcript_1/1_Conf_1.000	234	0							
Locus_19936_Transcript_1/2_Conf_1.000	901	20	4.28E-32	XP_002640503.1	61	142.895	282	174	Hypothetical protein CBG13642
Locus_19936_Transcript_2/2_Conf_1.000	910	20	4.36E-32	XP_002640503.1	61	142.895	282	174	Hypothetical protein CBG13642
Locus_19937_Transcript_1/1_Conf_1.000	324	0							
Locus_19938_Transcript_1/1_Conf_1.000	217	0							
Locus_19939_Transcript_1/1_Conf_1.000	317	20	1.35E-30	XP_001899898.1	89	135.961	104	93	LMBR1-like conserved region family protein
Locus_1994_Transcript_1/1_Conf_1.000	645	20	4.58E-86	NP_741348.2	88	321.242	214	190	hypothetical protein Y94H6A.5

Locus_19940_Transcript_1/1_Conf_1.000	285	20	4.34E-37	NP_496968.1	92	157.532	92	85	hypothetical protein Y48B6A.12
Locus_19941_Transcript_1/1_Conf_1.000	150	0							
Locus_19942_Transcript_1/1_Conf_1.000	129	2	5.42E-08	XP_001899872.1	78	60.8474	41	32	Protein-tyrosine phosphatase containing protein
Locus_19943_Transcript_1/1_Conf_1.000	538	20	4.88E-14	NP_001021851.2	79	81.2629	54	43	hypothetical protein ZC434.9
Locus_19944_Transcript_1/1_Conf_1.000	133	0							
Locus_19945_Transcript_1/1_Conf_1.000	129	0							
Locus_19946_Transcript_1/1_Conf_1.000	180	0							
Locus_19947_Transcript_1/1_Conf_1.000	305	12	9.52E-13	NP_004490.2	100	76.6406	78	78	heterogeneous nuclear ribonucleoprotein A/B isoform b
Locus_19948_Transcript_1/1_Conf_1.000	371	11	1.60E-07	ABB53347.1	44	59.3066	121	54	secreted protein 5 precursor
Locus_19949_Transcript_1/1_Conf_1.000	291	0							
Locus_1995_Transcript_1/2_Conf_1.000	518	20	1.74E-39	NP_492576.1	89	165.622	120	107	Ribosomal Protein, Large subunit family member (rpl-14)
Locus_1995_Transcript_2/2_Conf_1.000	218	20	1.83E-16	XP_002639221.1	87	88.9669	55	48	C. briggsae CBR-RPL-14 protein
Locus_19950_Transcript_1/1_Conf_1.000	219	0							
Locus_19951_Transcript_1/1_Conf_1.000	201	0							

Locus_19952_Transcript_1/1_Conf_1.000	325	20	2.87E-33	AAD09564.1	86	144.821	104	90	cyclophilin
Locus_19953_Transcript_1/1_Conf_1.000	188	0							
Locus_19954_Transcript_1/1_Conf_1.000	136	0							
Locus_19955_Transcript_1/1_Conf_1.000	369	11	5.82E-50	CBI63216.1	91	200.29	121	111	C. elegans protein F40F8.8b, partially confirmed by transcript evidence
Locus_19956_Transcript_1/1_Conf_1.000	1163	20	7.02E-55	NP_001021782.1	66	219.164	245	163	hypothetical protein Y52B11A.2
Locus_19957_Transcript_1/1_Conf_1.000	245	0							
Locus_19958_Transcript_1/1_Conf_1.000	849	0							
Locus_19959_Transcript_1/2_Conf_1.000	660	20	7.40E-79	XP_002646242.1	95	297.36	162	154	C. briggsae CBR-UNC-13 protein
Locus_19959_Transcript_2/2_Conf_1.000	383	20	2.40E-64	XP_002646242.1	96	248.054	128	123	C. briggsae CBR-UNC-13 protein
Locus_1996_Transcript_1/1_Conf_1.000	371	20	2.82E-20	XP_002643656.1	78	101.679	76	60	Hypothetical protein CBG16408
Locus_19960_Transcript_1/1_Conf_1.000	230	0							
Locus_19961_Transcript_1/1_Conf_1.000	171	20	5.05E-14	XP_002634908.1	76	80.8777	56	43	Hypothetical protein CBG22506
Locus_19962_Transcript_1/1_Conf_1.000	585	20	7.91E-41	NP_495933.1	69	170.629	163	114	hypothetical protein T24B8.2

Locus_19963_Transcript_1/1_Conf_1.000	261	20	7.38E-21	XP_002639570.1	87	103.605	63	55	C. briggsae CBR-ARX-7 protein
Locus_19964_Transcript_1/1_Conf_1.000	402	0							
Locus_19965_Transcript_1/1_Conf_1.000	463	20	1.03E-22	NP_496607.1	75	109.768	88	66	hypothetical protein Y57A10A.29
Locus_19966_Transcript_1/1_Conf_1.000	485	14	1.26E-12	XP_002633316.1	55	76.2554	167	92	C. briggsae CBR-GTL-2 protein
Locus_19967_Transcript_1/1_Conf_1.000	460	11	1.23E-12	NP_510606.1	74	76.2554	59	44	ACyLtransferase-like family member (acl-1)
Locus_19968_Transcript_1/1_Conf_1.000	319	1	2.64E-10	XP_001141980.1	61	68.5514	67	41	PREDICTED: ephrin A1
Locus_19969_Transcript_1/1_Conf_1.000	745	20	3.08E-101	NP_741172.2	91	372.089	220	201	protein KINase family member (kin-18)
Locus_1997_Transcript_1/1_Conf_1.000	198	13	3.19E-24	ACY39994.1	90	114.775	66	60	C. elegans FLN-2 protein, isoform d
Locus_19970_Transcript_1/1_Conf_1.000	182	0							
Locus_19971_Transcript_1/1_Conf_1.000	297	8	2.53E-05	XP_002646337.1	44	51.9878	95	42	Hypothetical protein CBG12050
Locus_19972_Transcript_1/1_Conf_1.000	140	20	3.97E-11	NP_500586.2	82	71.2478	46	38	hypothetical protein ZK185.2
Locus_19973_Transcript_1/1_Conf_1.000	190	0							
Locus_19974_Transcript_1/2_Conf_1.000	509	20	2.56E-56	XP_002632133.1	79	221.476	168	134	Hypothetical protein CBG06989
Locus_19974_Transcript_2/2_Conf_1.000	444	20	4.43E-50	XP_002632133.1	78	200.675	147	116	Hypothetical protein CBG06989

Locus_19975_Transcript_1/1_Conf_1.000	174	1	8.05E-04	XP_001944330.1	73	46.9802	34	25	PREDICTED: similar to CG8213 CG8213-PA
Locus_19976_Transcript_1/1_Conf_1.000	134	0							
Locus_19977_Transcript_1/1_Conf_1.000	425	20	1.59E-07	NP_733845.1	51	59.3066	110	57	nonstructural polyprotein
Locus_19978_Transcript_1/1_Conf_1.000	469	20	6.37E-49	NP_496736.1	85	196.823	121	103	Glycogen SYnthase family member (gsy-1)
Locus_19979_Transcript_1/1_Conf_1.000	322	6	2.55E-05	BAD94515.1	49	51.9878	67	33	peroxisome proliferator-activated receptor gamma
Locus_1998_Transcript_1/4_Conf_0.375	390	20	3.11E-48	NP_495823.1	91	194.512	108	99	Ribosomal Protein, Large subunit family member (rpl-26)
Locus_1998_Transcript_2/4_Conf_0.375	348	20	2.06E-47	NP_871965.1	93	191.815	103	96	Ribosomal Protein, Large subunit family member (rpl-26)
Locus_1998_Transcript_3/4_Conf_0.375	1184	20	2.24E-64	XP_002630145.1	92	250.751	139	129	C. briggsae CBR-RPL-26 protein
Locus_1998_Transcript_4/4_Conf_0.375	1184	20	2.24E-64	XP_002630145.1	92	250.751	139	129	C. briggsae CBR-RPL-26 protein
Locus_19980_Transcript_1/1_Conf_1.000	140	0							
Locus_19981_Transcript_1/1_Conf_1.000	257	0							

Locus_19982_Transcript_1/1_Conf_1.000	147	0							
Locus_19983_Transcript_1/1_Conf_1.000	265	0							
Locus_19984_Transcript_1/1_Conf_1.000	144	0							
Locus_19985_Transcript_1/1_Conf_1.000	143	0							
Locus_19986_Transcript_1/1_Conf_1.000	132	2	8.34E-09	NP_502336.1	89	63.5438	39	35	hypothetical protein C42C1.12
Locus_19987_Transcript_1/1_Conf_1.000	213	20	1.08E-16	EFO20785.1	77	89.7373	67	52	TKL/MLK/HH498 protein kinase
Locus_19988_Transcript_1/1_Conf_1.000	272	0							
Locus_19989_Transcript_1/1_Conf_1.000	347	0							
Locus_1999_Transcript_1/1_Conf_1.000	1887	20	1.43E-96	XP_001897626.1	67	358.607	353	238	Myosin head containing protein
Locus_19990_Transcript_1/1_Conf_1.000	479	2	1.73E-14	NP_509587.1	66	82.4185	110	73	hypothetical protein ZK899.1
Locus_19991_Transcript_1/1_Conf_1.000	162	20	4.46E-10	EDM11526.1	75	67.781	53	40	lactamase, beta 2
Locus_19992_Transcript_1/1_Conf_1.000	178	6	1.23E-20	XP_001896988.1	87	102.834	58	51	UTP--ammonia ligase
Locus_19993_Transcript_1/1_Conf_1.000	564	20	3.38E-51	NP_498172.3	70	204.912	192	136	hypothetical protein R02F2.2
Locus_19994_Transcript_1/1_Conf_1.000	301	20	8.05E-28	XP_002639522.1	76	126.716	99	76	Hypothetical protein CBG04128
Locus_19995_Transcript_1/1_Conf_1.000	554	2	5.38E-06	NP_741780.1	57	54.6842	90	52	hypothetical protein H28G03.2
Locus_19996_Transcript_1/1_Conf_1.000	673	0							

Locus_19997_Transcript_1/1_Conf_1.000	210	20	5.01E-30	AAC19161.1	98	134.035	69	68	unknown
Locus_19998_Transcript_1/1_Conf_1.000	206	20	7.55E-18	NP_496500.1	75	93.5893	66	50	hypothetical protein VF13D12L.3
Locus_19999_Transcript_1/1_Conf_1.000	229	0							
Locus_2_Transcript_1/1_Conf_1.000	285	0							
Locus_20_Transcript_1/1_Conf_1.000	535	7	3.92E-08	XP_002641747.1	63	61.6178	63	40	Hypothetical protein CBG10086
Locus_200_Transcript_1/1_Conf_1.000	730	20	5.66E-36	XP_002631322.1	58	155.221	197	115	C. briggsae CBR-TRS-1 protein
Locus_2000_Transcript_1/1_Conf_1.000	1265	20	6.72E-163	XP_002646193.1	80	578.17	421	341	Hypothetical protein CBG24498
Locus_20000_Transcript_1/1_Conf_1.000	271	2	1.38E-11	XP_002642828.1	68	72.7886	80	55	Hypothetical protein CBG21226
Locus_20001_Transcript_1/1_Conf_1.000	177	20	2.40E-24	BAG59482.1	100	115.161	58	58	unnamed protein product
Locus_20002_Transcript_1/1_Conf_1.000	184	0							

Locus_20003_Transcript_1/1_Conf_1.000	230	0							
Locus_20004_Transcript_1/1_Conf_1.000	621	20	1.22E-45	XP_002637227.1	72	186.808	147	106	Hypothetical protein CBG18895
Locus_20005_Transcript_1/1_Conf_1.000	452	8	7.54E-26	XP_002631168.1	76	120.168	92	70	C. briggsae CBR-PQM-1 protein
Locus_20006_Transcript_1/1_Conf_1.000	240	0							
Locus_20007_Transcript_1/1_Conf_1.000	318	4	1.95E-13	EFO19864.1	64	78.9518	100	64	hypothetical protein LOAG_08632
Locus_20008_Transcript_1/1_Conf_1.000	293	20	2.01E-26	NP_497035.3	78	122.094	97	76	Trehalose 6-Phosphate Synthase family member (tps-2)
Locus_20009_Transcript_1/1_Conf_1.000	348	0							
Locus_2001_Transcript_1/1_Conf_1.000	1103	20	8.64E-116	ACI48996.1	79	421.394	333	264	hypothetical protein Cbre_JD01.003
Locus_20010_Transcript_1/1_Conf_1.000	380	0							
Locus_20011_Transcript_1/1_Conf_1.000	817	0							
Locus_20012_Transcript_1/1_Conf_1.000	385	20	3.58E-44	XP_002633893.1	79	181.03	125	99	C. briggsae CBR-ELO-5 protein
Locus_20013_Transcript_1/1_Conf_1.000	248	20	2.74E-28	XP_001898240.1	84	128.257	82	69	XPA-binding protein 2
Locus_20014_Transcript_1/1_Conf_1.000	120	0							

Locus_20015_Transcript_1/1_Conf_1.000	258	20	6.25E-28	BAG60492.1	100	127.102	59	59	unnamed protein product
Locus_20016_Transcript_1/1_Conf_1.000	338	20	4.53E-23	NP_504239.2	66	110.923	106	71	hypothetical protein Y61A9LA.8
Locus_20017_Transcript_1/1_Conf_1.000	167	0							
Locus_20018_Transcript_1/1_Conf_1.000	188	0							
Locus_20019_Transcript_1/1_Conf_1.000	197	0							
Locus_2002_Transcript_1/1_Conf_1.000	2412	20	0	XP_002637276.1	91	1036.17	591	543	Hypothetical protein CBG18959
Locus_20020_Transcript_1/1_Conf_1.000	221	4	2.84E-12	NP_001076754.1	79	75.0998	73	58	hypothetical protein C14H10.2
Locus_20021_Transcript_1/1_Conf_1.000	392	20	4.64E-44	NP_504292.2	87	180.644	115	101	hypothetical protein C37H5.5
Locus_20022_Transcript_1/1_Conf_1.000	467	20	2.99E-22	EFO27494.1	61	108.227	125	77	hypothetical protein LOAG_00986
Locus_20023_Transcript_1/1_Conf_1.000	735	20	1.90E-63	XP_002646726.1	69	246.514	219	153	Hypothetical protein CBG13109
Locus_20024_Transcript_1/1_Conf_1.000	412	2	1.55E-47	NP_001122963.1	83	192.2	134	112	hypothetical protein F58E6.1
Locus_20025_Transcript_1/1_Conf_1.000	173	0							
Locus_20026_Transcript_1/1_Conf_1.000	129	20	1.47E-13	NP_741632.2	97	79.337	41	40	SMAll family member (sma-1)
Locus_20027_Transcript_1/1_Conf_1.000	312	20	2.84E-12	NP_956980.1	83	75.0998	54	45	mitochondrial fission protein MTP18
Locus_20028_Transcript_1/1_Conf_1.000	209	0							

Locus_20029_Transcript_1/1_Conf_1.000	163	0							
Locus_2003_Transcript_1/1_Conf_1.000	450	20	4.25E-45	XP_002641573.1	81	184.111	138	112	C. briggsae CBR-TTLL-4 protein
Locus_20030_Transcript_1/1_Conf_1.000	260	0							
Locus_20031_Transcript_1/1_Conf_1.000	290	1	6.30E-04	XP_002633908.1	45	47.3654	87	40	Hypothetical protein CBG19972
Locus_20032_Transcript_1/1_Conf_1.000	720	20	9.26E-84	NP_509174.1	83	313.923	206	173	hypothetical protein C16E9.2
Locus_20033_Transcript_1/1_Conf_1.000	333	20	1.12E-21	NP_500647.2	65	106.301	115	75	Dipeptidyl Peptidase Four (IV) family member (dpf-5)
Locus_20034_Transcript_1/1_Conf_1.000	258	8	1.39E-35	NP_502016.2	94	152.525	85	80	hypothetical protein F13E9.1
Locus_20035_Transcript_1/1_Conf_1.000	390	0							
Locus_20036_Transcript_1/1_Conf_1.000	236	0							
Locus_20037_Transcript_1/1_Conf_1.000	306	0							
Locus_20038_Transcript_1/1_Conf_1.000	214	0							
Locus_20039_Transcript_1/1_Conf_1.000	228	20	5.65E-21	EFO26545.1	77	103.99	76	59	kelch domain-containing protein family protein
Locus_2004_Transcript_1/1_Conf_1.000	1174	20	1.76E-53	NP_493375.2	69	214.542	220	153	AuTophaGy (yeast Atg homolog) family member (atg-4.1)
Locus_20040_Transcript_1/1_Conf_1.000	217	1	2.75E-04	YP_001044988.1	53	48.521	56	30	hypothetical protein Rsph17029_3116
Locus_20041_Transcript_1/1_Conf_1.000	464	0							
Locus_20042_Transcript_1/1_Conf_1.000	338	20	1.82E-56	XP_002747692.1	100	221.861	112	112	PREDICTED: 40S ribosomal protein S7-like
Locus_20043_Transcript_1/1_Conf_1.000	222	0							
Locus_20044_Transcript_1/1_Conf_1.000	1103	20	6.31E-50	NP_498604.2	71	202.601	180	129	hypothetical protein B0361.7
Locus_20045_Transcript_1/1_Conf_1.000	247	4	2.49E-13	XP_002645089.1	66	78.5666	93	62	Hypothetical protein CBG16766
Locus_20046_Transcript_1/1_Conf_1.000	513	0							
Locus_20047_Transcript_1/1_Conf_1.000	221	20	3.81E-33	NP_506269.1	100	144.436	73	73	EATing: abnormal pharyngeal pumping family member (eat-6)
Locus_20048_Transcript_1/1_Conf_1.000	134	0							
Locus_20049_Transcript_1/1_Conf_1.000	437	0							

Locus_2005_Transcript_1/1_Conf_1.000	2109	20	0	XP_002638594.1	89	784.252	479	427	C. briggsae CBR-GCK-3 protein
Locus_20050_Transcript_1/1_Conf_1.000	451	2	5.41E-24	XP_002643963.1	60	114.005	130	79	Hypothetical protein CBG17334
Locus_20051_Transcript_1/1_Conf_1.000	240	0							
Locus_20052_Transcript_1/1_Conf_1.000	436	3	8.56E-14	XP_002642011.1	57	80.1073	103	59	Hypothetical protein CBG17944
Locus_20053_Transcript_1/1_Conf_1.000	173	0							
Locus_20054_Transcript_1/1_Conf_1.000	165	0							
Locus_20055_Transcript_1/1_Conf_1.000	299	3	2.21E-09	AAO63577.1	54	65.4698	102	56	secreted protein 5 precursor
Locus_20056_Transcript_1/1_Conf_1.000	241	0							
Locus_20057_Transcript_1/1_Conf_1.000	145	3	1.85E-11	NP_498063.1	86	72.4034	46	40	hypothetical protein R144.11
Locus_20058_Transcript_1/1_Conf_1.000	139	0							
Locus_20059_Transcript_1/1_Conf_1.000	921	20	5.16E-113	ACJ65180.1	78	411.764	304	239	Gaba b receptor subunit protein 2, confirmed by transcript evidence
Locus_2006_Transcript_1/1_Conf_1.000	1118	4	3.93E-31	XP_002638907.1	69	140.198	158	110	Hypothetical protein CBG22133
Locus_20060_Transcript_1/1_Conf_1.000	372	20	2.70E-39	NP_509899.3	89	164.851	118	106	Organic Cation Transporter family member (oct-2)
Locus_20061_Transcript_1/1_Conf_1.000	276	0							
Locus_20062_Transcript_1/1_Conf_1.000	151	0							
Locus_20063_Transcript_1/1_Conf_1.000	161	0							
Locus_20064_Transcript_1/1_Conf_1.000	215	0							
Locus_20065_Transcript_1/1_Conf_1.000	302	0							
Locus_20066_Transcript_1/1_Conf_1.000	302	4	4.15E-16	XP_002641354.1	66	87.8113	96	64	Hypothetical protein CBG13208
Locus_20067_Transcript_1/1_Conf_1.000	311	0							
Locus_20068_Transcript_1/1_Conf_1.000	133	1	2.13E-04	XP_002634830.1	77	48.9062	44	34	Hypothetical protein CBG13938
Locus_20069_Transcript_1/1_Conf_1.000	338	20	3.98E-11	XP_001898083.1	63	71.2478	79	50	hypothetical protein
Locus_2007_Transcript_1/1_Conf_1.000	170	0							
Locus_20070_Transcript_1/1_Conf_1.000	230	0							

Locus_20071_Transcript_1/1_Conf_1.000	466	2	2.81E-04	NP_493556.1	49	48.521	97	48	hypothetical protein Y54E5A.8
Locus_20072_Transcript_1/1_Conf_1.000	147	0							
Locus_20073_Transcript_1/1_Conf_1.000	418	13	2.48E-08	CAR97807.1	48	62.003	115	56	C. elegans protein C23H4.4b, partially confirmed by transcript evidence
Locus_20074_Transcript_1/1_Conf_1.000	304	0							
Locus_20075_Transcript_1/1_Conf_1.000	173	0							
Locus_20076_Transcript_1/1_Conf_1.000	621	20	3.20E-54	NP_508866.1	73	215.312	186	137	Unidentified Vitellogenin-linked Transcript family member (uvt-3)
Locus_20077_Transcript_1/1_Conf_1.000	380	20	4.01E-27	EFO27289.1	69	124.405	123	85	actin family protein
Locus_20078_Transcript_1/1_Conf_1.000	203	0							
Locus_20079_Transcript_1/1_Conf_1.000	294	6	3.20E-16	NP_495366.2	76	88.1965	72	55	DumPY : shorter than wild-type family member (dpy-10)
Locus_2008_Transcript_1/1_Conf_1.000	2313	20	9.50E-154	XP_002631266.1	74	548.895	455	340	C. briggsae CBR-MNK-1 protein
Locus_20080_Transcript_1/1_Conf_1.000	305	0							
Locus_20081_Transcript_1/1_Conf_1.000	260	0							
Locus_20082_Transcript_1/1_Conf_1.000	559	0							
Locus_20083_Transcript_1/1_Conf_1.000	345	1	2.15E-28	BAB93497.1	100	128.642	73	73	OK/SW-CL.24
Locus_20084_Transcript_1/1_Conf_1.000	131	3	3.40E-10	XP_002632407.1	86	68.1662	43	37	Hypothetical protein CBG00431
Locus_20085_Transcript_1/1_Conf_1.000	210	20	2.33E-19	AAN11401.1	74	98.5969	66	49	metalloprotease 1 precursor
Locus_20086_Transcript_1/1_Conf_1.000	524	0							
Locus_20087_Transcript_1/1_Conf_1.000	172	0							

Locus_20088_Transcript_1/1_Conf_1.000	143	10	1.27E-17	BAD93019.1	100	92.8189	46	46	ubiquitin C variant
Locus_20089_Transcript_1/1_Conf_1.000	144	0							
Locus_2009_Transcript_1/1_Conf_1.000	884	0							
Locus_20090_Transcript_1/1_Conf_1.000	336	0							
Locus_20091_Transcript_1/1_Conf_1.000	133	2	1.25E-04	NP_508432.1	74	49.6766	47	35	hypothetical protein F47G3.1
Locus_20092_Transcript_1/1_Conf_1.000	263	4	6.23E-28	XP_002637429.1	84	127.102	83	70	Hypothetical protein CBG19140
Locus_20093_Transcript_1/1_Conf_1.000	391	0							
Locus_20094_Transcript_1/1_Conf_1.000	317	20	7.90E-39	XP_002631100.1	85	163.31	104	89	Hypothetical protein CBG02874
Locus_20095_Transcript_1/1_Conf_1.000	195	0							
Locus_20096_Transcript_1/1_Conf_1.000	159	0							
Locus_20097_Transcript_1/1_Conf_1.000	321	0							
Locus_20098_Transcript_1/1_Conf_1.000	380	0							
Locus_20099_Transcript_1/1_Conf_1.000	445	20	4.17E-32	XP_001897051.1	66	140.969	148	99	RNA polymerase Rpb1, domain 1 family protein
Locus_201_Transcript_1/1_Conf_1.000	906	0							
Locus_2010_Transcript_1/1_Conf_1.000	1579	20	1.77E-166	NP_501365.1	89	590.497	348	313	P38 Map Kinase family member (pmk-1)
Locus_20100_Transcript_1/1_Conf_1.000	545	2	7.07E-24	NP_495419.1	60	114.005	160	96	hypothetical protein B0252.1
Locus_20101_Transcript_1/1_Conf_1.000	157	0							
Locus_20102_Transcript_1/1_Conf_1.000	225	0							
Locus_20103_Transcript_1/1_Conf_1.000	151	0							

Locus_20104_Transcript_1/1_Conf_1.000	769	20	7.77E-95	NP_505613.1	79	350.903	257	204	hypothetical protein C51E3.6
Locus_20105_Transcript_1/1_Conf_1.000	354	20	1.42E-32	NP_505071.1	70	142.51	118	83	hypothetical protein ZC513.5
Locus_20106_Transcript_1/1_Conf_1.000	397	0							
Locus_20107_Transcript_1/1_Conf_1.000	297	20	5.42E-24	NP_492498.1	91	114.005	70	64	hypothetical protein F16A11.2
Locus_20108_Transcript_1/1_Conf_1.000	255	3	2.10E-07	AAA83446.5	70	58.9214	74	52	Hypothetical protein F44A2.3
Locus_20109_Transcript_1/1_Conf_1.000	444	20	1.62E-68	NP_498777.2	92	261.922	147	136	hypothetical protein C14B9.8
Locus_2011_Transcript_1/1_Conf_1.000	706	20	8.35E-42	XP_002642125.1	64	174.481	251	161	Hypothetical protein CBG18072
Locus_20110_Transcript_1/1_Conf_1.000	483	20	3.91E-30	NP_493165.3	60	134.42	156	95	hypothetical protein T27F6.1
Locus_20111_Transcript_1/1_Conf_1.000	350	0							
Locus_20112_Transcript_1/1_Conf_1.000	134	5	3.28E-05	EFA00585.1	70	51.6026	44	31	hypothetical protein TcasGA2_TC003455
Locus_20113_Transcript_1/1_Conf_1.000	261	20	8.96E-43	XP_002811879.1	100	176.407	87	87	PREDICTED: macrophage-capping protein-like isoform 4
Locus_20114_Transcript_1/1_Conf_1.000	437	0							
Locus_20115_Transcript_1/1_Conf_1.000	311	4	1.01E-14	NP_741925.2	81	83.1889	86	70	DYstrophin-like phenotype and CAPON related family member (dyc-1)

Locus_20116_Transcript_1/1_Conf_1.000	180	20	4.86E-20	AAK68292.3	92	100.908	54	50	U28943_4Clc-type chloride channel protein 3, isoform b, confirmed by transcript evidence
Locus_20117_Transcript_1/1_Conf_1.000	357	9	1.20E-15	XP_002633446.1	55	86.2705	120	67	Hypothetical protein CBG06214
Locus_20118_Transcript_1/1_Conf_1.000	290	0							
Locus_20119_Transcript_1/1_Conf_1.000	248	20	1.04E-35	NP_509689.1	96	152.91	82	79	hypothetical protein ZC373.4
Locus_2012_Transcript_1/1_Conf_1.000	1101	20	9.89E-112	NP_497785.1	78	407.912	301	237	TIR (Toll and Interleukin 1 Receptor) domain protein family member (tir-1)
Locus_20120_Transcript_1/1_Conf_1.000	187	0							
Locus_20121_Transcript_1/1_Conf_1.000	147	0							
Locus_20122_Transcript_1/1_Conf_1.000	225	0							
Locus_20123_Transcript_1/1_Conf_1.000	348	0							
Locus_20124_Transcript_1/1_Conf_1.000	369	13	1.27E-20	XP_002641615.1	82	102.834	76	63	Hypothetical protein CBG09935
Locus_20125_Transcript_1/1_Conf_1.000	396	20	1.18E-26	XP_002631717.1	64	122.865	137	88	Hypothetical protein CBG20918
Locus_20126_Transcript_1/1_Conf_1.000	418	7	4.37E-05	XP_002125751.1	40	51.2174	136	55	PREDICTED: similar to Thrombospondin type 1 domain containing protein
Locus_20127_Transcript_1/1_Conf_1.000	326	20	2.20E-33	NP_001022572.1	77	145.206	108	84	Elongation Factor Kinase family member (efk-1)
Locus_20128_Transcript_1/1_Conf_1.000	175	0							
Locus_20129_Transcript_1/1_Conf_1.000	135	0							
Locus_2013_Transcript_1/1_Conf_1.000	1587	20	3.17E-139	NP_001021806.1	72	499.975	494	360	Conserved Oligomeric Golgi (COG) Component family member (cogc-3)
Locus_20130_Transcript_1/1_Conf_1.000	151	0							
Locus_20131_Transcript_1/1_Conf_1.000	356	0							
Locus_20132_Transcript_1/1_Conf_1.000	163	0							
Locus_20133_Transcript_1/2_Conf_1.000	500	0							
Locus_20133_Transcript_2/2_Conf_1.000	511	0							
Locus_20134_Transcript_1/1_Conf_1.000	1114	20	0	NP_492498.1	93	664.84	369	345	hypothetical protein F16A11.2

Locus_20135_Transcript_1/1_Conf_1.000	432	20	1.61E-36	NP_001022174.1	74	155.606	147	110	hypothetical protein F45E10.2
Locus_20136_Transcript_1/1_Conf_1.000	236	0							
Locus_20137_Transcript_1/1_Conf_1.000	482	3	5.91E-15	XP_002641730.1	75	83.9593	62	47	Hypothetical protein CBG10069
Locus_20138_Transcript_1/1_Conf_1.000	654	20	6.48E-51	NP_001022694.1	69	204.527	195	135	hypothetical protein M142.8
Locus_20139_Transcript_1/1_Conf_1.000	158	0							
Locus_2014_Transcript_1/1_Conf_1.000	183	0							
Locus_20140_Transcript_1/1_Conf_1.000	177	0							
Locus_20141_Transcript_1/1_Conf_1.000	175	3	3.49E-07	NP_001022619.1	71	58.151	52	37	NorDiHydroGuaiaretic acid resistant family member (ndg-4)
Locus_20142_Transcript_1/1_Conf_1.000	166	0							
Locus_20143_Transcript_1/1_Conf_1.000	287	0							
Locus_20144_Transcript_1/1_Conf_1.000	294	20	6.02E-23	NP_001024958.1	82	110.538	97	80	hypothetical protein Y34B4A.4
Locus_20145_Transcript_1/2_Conf_1.000	466	0							
Locus_20145_Transcript_2/2_Conf_1.000	367	0							
Locus_20146_Transcript_1/1_Conf_1.000	205	0							
Locus_20147_Transcript_1/1_Conf_1.000	370	0							
Locus_20148_Transcript_1/1_Conf_1.000	268	0							
Locus_20149_Transcript_1/1_Conf_1.000	223	3	8.82E-06	NP_501039.1	63	53.5286	60	38	hypothetical protein C17H12.2
Locus_2015_Transcript_1/3_Conf_0.750	726	0							
Locus_2015_Transcript_2/3_Conf_0.750	818	0							
Locus_2015_Transcript_3/3_Conf_0.750	797	0							
Locus_20150_Transcript_1/1_Conf_1.000	145	2	3.04E-14	XP_002634423.1	89	81.6481	48	43	C. briggsae CBR-PTR-14 protein
Locus_20151_Transcript_1/1_Conf_1.000	138	20	6.12E-20	XP_002816317.1	100	100.523	46	46	PREDICTED: vesicular integral-membrane protein VIP36-like isoform 2

Locus_20152_Transcript_1/1_Conf_1.000	249	3	5.90E-23	NP_497970.1	88	110.538	68	60	Adenylyl CYclase family member (acy-1)
Locus_20153_Transcript_1/1_Conf_1.000	200	20	4.76E-20	NP_496968.1	87	100.908	66	58	hypothetical protein Y48B6A.12
Locus_20154_Transcript_1/1_Conf_1.000	213	20	3.96E-19	NP_500412.1	82	97.8265	69	57	Variable ABnormal morphology family member (vab-2)
Locus_20155_Transcript_1/1_Conf_1.000	371	2	4.97E-25	NP_492185.2	65	117.472	126	82	SMG-associated and Lethal family member (smgl-1)
Locus_20156_Transcript_1/2_Conf_1.000	360	0							
Locus_20156_Transcript_2/2_Conf_1.000	270	0							
Locus_20157_Transcript_1/1_Conf_1.000	231	1	1.44E-08	XP_002634119.1	65	62.7734	72	47	Hypothetical protein CBG01673
Locus_20158_Transcript_1/1_Conf_1.000	148	0							
Locus_20159_Transcript_1/1_Conf_1.000	134	0							
Locus_2016_Transcript_1/1_Conf_1.000	1281	20	1.24E-79	NP_492371.1	74	301.597	258	193	nuclear LaMiN family member (lmn-1)
Locus_20160_Transcript_1/1_Conf_1.000	198	20	1.58E-23	NP_001122833.1	89	112.464	65	58	hypothetical protein ZC518.1
Locus_20161_Transcript_1/1_Conf_1.000	229	20	3.21E-16	NP_741383.1	73	88.1965	72	53	tYrosinyl tRNA Synthetase family member (yrs-1)
Locus_20162_Transcript_1/1_Conf_1.000	211	0							
Locus_20163_Transcript_1/1_Conf_1.000	340	0							
Locus_20165_Transcript_1/1_Conf_1.000	492	20	9.51E-29	XP_002130984.1	63	129.798	145	92	PREDICTED: similar to MGC80593 protein
Locus_20166_Transcript_1/1_Conf_1.000	327	0							
Locus_20167_Transcript_1/1_Conf_1.000	153	0							
Locus_20168_Transcript_1/1_Conf_1.000	275	0							
Locus_20169_Transcript_1/1_Conf_1.000	169	0							

Locus_2017_Transcript_1/1_Conf_1.000	456	20	3.70E-33	XP_002642667.1	95	144.436	73	70	C. briggsae CBR-SNR-5 protein
Locus_20170_Transcript_1/1_Conf_1.000	198	0							
Locus_20171_Transcript_1/1_Conf_1.000	185	20	2.25E-17	NP_497008.1	83	92.0485	61	51	hypothetical protein K10H10.2
Locus_20172_Transcript_1/1_Conf_1.000	304	0							
Locus_20173_Transcript_1/1_Conf_1.000	210	0							
Locus_20174_Transcript_1/1_Conf_1.000	272	0							
Locus_20175_Transcript_1/1_Conf_1.000	146	0							
Locus_20176_Transcript_1/1_Conf_1.000	205	20	1.20E-23	NP_740900.1	88	112.849	68	60	hypothetical protein T08G11.1
Locus_20177_Transcript_1/1_Conf_1.000	318	0							
Locus_20178_Transcript_1/1_Conf_1.000	140	0							
Locus_20179_Transcript_1/1_Conf_1.000	360	20	3.83E-38	XP_002642021.1	93	160.999	90	84	C. briggsae CBR-COQ-8 protein
Locus_2018_Transcript_1/1_Conf_1.000	2111	20	6.94E-172	XP_001899259.1	75	608.986	504	381	Conserved hypothetical protein
Locus_20180_Transcript_1/1_Conf_1.000	427	20	1.48E-21	NP_499961.2	60	105.916	145	87	Nuclear Hormone Receptor family member (nhr-76)
Locus_20181_Transcript_1/1_Conf_1.000	236	0							
Locus_20182_Transcript_1/1_Conf_1.000	309	0							
Locus_20183_Transcript_1/1_Conf_1.000	1127	20	1.07E-84	ADI24662.1	71	318.161	322	230	Hypothetical protein Y110A2AL.12a
Locus_20184_Transcript_1/1_Conf_1.000	131	0							
Locus_20185_Transcript_1/1_Conf_1.000	137	0							
Locus_20186_Transcript_1/1_Conf_1.000	173	0							
Locus_20187_Transcript_1/2_Conf_1.000	318	20	4.15E-56	A8Y236.2	98	220.705	104	102	Putative polypeptide N-acetylgalactosaminyltransferase 10

Locus_20187_Transcript_2/2_Conf_1.000	318	20	4.15E-56	A8Y236.2	98	220.705	104	102	Putative polypeptide N-acetylgalactosaminyltransferase 10
Locus_20188_Transcript_1/1_Conf_1.000	361	6	1.51E-34	XP_002632694.1	79	149.058	111	88	Hypothetical protein CBG21625
Locus_20189_Transcript_1/1_Conf_1.000	144	0							
Locus_2019_Transcript_1/3_Conf_0.357	144	0							
Locus_2019_Transcript_2/3_Conf_0.429	210	0							
Locus_2019_Transcript_3/3_Conf_0.571	407	2	2.79E-04	CAC17797.1	52	48.521	74	39	SXC1 protein
Locus_20190_Transcript_1/1_Conf_1.000	189	4	1.96E-05	NP_001122780.1	73	52.373	56	41	hypothetical protein F41H10.6
Locus_20191_Transcript_1/1_Conf_1.000	209	0							
Locus_20192_Transcript_1/1_Conf_1.000	267	0							
Locus_20193_Transcript_1/1_Conf_1.000	164	9	8.97E-19	XP_002630203.1	92	96.6709	54	50	Hypothetical protein CBG00613
Locus_20194_Transcript_1/1_Conf_1.000	1183	20	1.70E-96	XP_001897426.1	76	357.451	301	231	Rab5 GDP/GTP exchange factor
Locus_20195_Transcript_1/1_Conf_1.000	129	0							
Locus_20196_Transcript_1/2_Conf_1.000	187	0							
Locus_20196_Transcript_2/2_Conf_1.000	264	0							
Locus_20197_Transcript_1/1_Conf_1.000	175	0							
Locus_20198_Transcript_1/1_Conf_1.000	215	12	3.13E-24	NP_493493.2	88	114.775	70	62	hypothetical protein F09C3.2
Locus_20199_Transcript_1/1_Conf_1.000	142	0							
Locus_202_Transcript_1/1_Conf_1.000	227	0							
Locus_2020_Transcript_1/1_Conf_1.000	316	20	5.13E-30	XP_002642349.1	75	134.035	103	78	Hypothetical protein CBG18346
Locus_20200_Transcript_1/1_Conf_1.000	364	0							
Locus_20201_Transcript_1/1_Conf_1.000	135	0							
Locus_20202_Transcript_1/1_Conf_1.000	146	0							
Locus_20203_Transcript_1/1_Conf_1.000	424	0							
Locus_20204_Transcript_1/1_Conf_1.000	142	0							
Locus_20205_Transcript_1/1_Conf_1.000	537	0							
Locus_20206_Transcript_1/1_Conf_1.000	225	20	1.31E-17	NP_510666.1	96	92.8189	50	48	hypothetical protein F01G12.6
Locus_20207_Transcript_1/1_Conf_1.000	627	20	2.13E-61	NP_741746.1	83	239.195	171	142	Kinesin-Like Protein family member (klp-4)

Locus_20208_Transcript_1/1_Conf_1.000	210	20	1.46E-21	XP_002637044.1	84	105.916	69	58	C. briggsae CBR-CUL-5 protein
Locus_20209_Transcript_1/1_Conf_1.000	479	20	3.01E-51	XP_002630906.1	76	204.527	159	121	Hypothetical protein CBG02630
Locus_2021_Transcript_1/1_Conf_1.000	141	0							
Locus_20210_Transcript_1/1_Conf_1.000	379	20	2.43E-32	XP_002643095.1	77	141.739	127	98	C. briggsae CBR-GLY-11 protein
Locus_20211_Transcript_1/1_Conf_1.000	250	20	5.92E-07	XP_001641433.1	57	57.3806	75	43	predicted protein
Locus_20212_Transcript_1/1_Conf_1.000	274	0							
Locus_20213_Transcript_1/1_Conf_1.000	221	0							
Locus_20214_Transcript_1/1_Conf_1.000	964	20	1.05E-71	XP_002643853.1	67	274.633	331	223	Hypothetical protein CBG02083
Locus_20215_Transcript_1/1_Conf_1.000	391	0							
Locus_20216_Transcript_1/1_Conf_1.000	141	0							
Locus_20217_Transcript_1/1_Conf_1.000	177	4	2.26E-06	EFO14006.1	73	55.4546	49	36	hypothetical protein LOAG_14520
Locus_20218_Transcript_1/1_Conf_1.000	189	0							
Locus_20219_Transcript_1/1_Conf_1.000	190	20	2.46E-24	Q60V90.3	95	115.161	60	57	Bifunctional heparan sulfate N-deacetylase/N-sulfotransferase 1
Locus_2022_Transcript_1/1_Conf_1.000	1196	20	1.74E-72	NP_499801.1	70	277.715	278	197	hypothetical protein Y76A2B.5
Locus_20220_Transcript_1/2_Conf_1.000	311	0							
Locus_20220_Transcript_2/2_Conf_1.000	311	0							
Locus_20221_Transcript_1/1_Conf_1.000	197	0							
Locus_20222_Transcript_1/1_Conf_1.000	165	1	8.13E-04	NP_505666.2	68	46.9802	51	35	UDP-GlucuronosylTransferase family member (ugt-49)
Locus_20223_Transcript_1/1_Conf_1.000	210	3	7.89E-19	NP_492219.1	86	91.2781	51	44	hypothetical protein K04G2.9
Locus_20224_Transcript_1/1_Conf_1.000	146	0							
Locus_20225_Transcript_1/1_Conf_1.000	179	20	1.85E-08	XP_002577284.1	66	62.3882	60	40	nuclear factor Y transcription factor subunit B homolog

Locus_20226_Transcript_1/1_Conf_1.000	137	7	1.01E-14	XP_002632396.1	97	83.1889	40	39	C. briggsae CBR-UAF-2 protein
Locus_20227_Transcript_1/1_Conf_1.000	234	6	4.17E-08	NP_001041050.1	58	61.2326	78	46	hypothetical protein Y57G11C.3
Locus_20228_Transcript_1/1_Conf_1.000	264	19	1.18E-10	XP_002639499.1	61	69.707	85	52	C. briggsae CBR-ITX-1 protein
Locus_20229_Transcript_1/1_Conf_1.000	645	1	8.85E-05	EFO26655.1	42	51.2174	133	57	hypothetical protein LOAG_01835
Locus_2023_Transcript_1/1_Conf_1.000	583	0							
Locus_20230_Transcript_1/1_Conf_1.000	194	0							
Locus_20231_Transcript_1/1_Conf_1.000	161	0							
Locus_20232_Transcript_1/1_Conf_1.000	179	0							
Locus_20233_Transcript_1/1_Conf_1.000	206	20	6.41E-09	XP_001745498.1	76	63.929	56	43	hypothetical protein
Locus_20234_Transcript_1/1_Conf_1.000	323	0							
Locus_20235_Transcript_1/1_Conf_1.000	165	0							
Locus_20236_Transcript_1/1_Conf_1.000	166	20	1.93E-21	XP_002644000.1	100	105.531	55	55	C. briggsae CBR-IFA-1 protein
Locus_20237_Transcript_1/1_Conf_1.000	176	0							
Locus_20238_Transcript_1/1_Conf_1.000	268	0							
Locus_20239_Transcript_1/1_Conf_1.000	645	20	2.01E-49	NP_505419.3	70	199.519	210	147	hypothetical protein K07B1.7

Locus_2024_Transcript_1/1_Conf_1.000	1841	20	5.40E-109	XP_002639032.1	57	399.823	606	349	Hypothetical protein CBG22281
Locus_20240_Transcript_1/1_Conf_1.000	297	20	5.24E-27	NP_504827.2	84	124.02	85	72	hypothetical protein F09G2.1
Locus_20241_Transcript_1/1_Conf_1.000	132	0							
Locus_20242_Transcript_1/1_Conf_1.000	276	20	2.85E-17	AAN61521.1	65	91.6633	92	60	301KDa_2 protein
Locus_20243_Transcript_1/1_Conf_1.000	367	0							
Locus_20244_Transcript_1/1_Conf_1.000	312	0							
Locus_20245_Transcript_1/1_Conf_1.000	370	20	1.26E-44	XP_002826560.1	100	182.57	93	93	PREDICTED: protein FAM96B-like
Locus_20246_Transcript_1/1_Conf_1.000	230	20	5.11E-22	NP_569731.2	100	107.457	52	52	putative NPIP-like protein KIAA0220-like
Locus_20247_Transcript_1/1_Conf_1.000	312	0							
Locus_20248_Transcript_1/1_Conf_1.000	257	0							
Locus_20249_Transcript_1/1_Conf_1.000	198	0							
Locus_2025_Transcript_1/1_Conf_1.000	1513	20	3.20E-141	NP_740900.1	75	506.523	507	385	hypothetical protein T08G11.1
Locus_20250_Transcript_1/1_Conf_1.000	251	0							
Locus_20251_Transcript_1/1_Conf_1.000	280	20	1.45E-29	XP_002634888.1	80	132.494	88	71	Hypothetical protein CBG10559
Locus_20252_Transcript_1/1_Conf_1.000	200	20	9.62E-13	XP_001900297.1	75	76.6406	65	49	Importin-beta N-terminal domain containing protein
Locus_20253_Transcript_1/1_Conf_1.000	228	0							
Locus_20254_Transcript_1/1_Conf_1.000	234	0							
Locus_20255_Transcript_1/1_Conf_1.000	520	4	9.14E-36	XP_002631027.1	70	153.295	172	121	Hypothetical protein CBG02784
Locus_20256_Transcript_1/1_Conf_1.000	156	20	5.48E-16	AAV41897.1	92	87.4261	52	48	SAX-2
Locus_20257_Transcript_1/1_Conf_1.000	187	0							
Locus_20258_Transcript_1/1_Conf_1.000	280	20	8.53E-22	NP_001041277.1	81	106.686	69	56	hypothetical protein PDB1.1
Locus_20259_Transcript_1/1_Conf_1.000	262	0							
Locus_2026_Transcript_1/1_Conf_1.000	567	0							
Locus_20260_Transcript_1/1_Conf_1.000	467	5	3.79E-17	XP_001892434.1	58	91.2781	111	65	hypothetical protein Bm1_04780
Locus_20261_Transcript_1/1_Conf_1.000	269	0							

Locus_20262_Transcript_1/1_Conf_1.000	602	10	1.39E-19	EFO17301.1	56	100.138	192	108	hypothetical protein LOAG_11198
Locus_20263_Transcript_1/1_Conf_1.000	223	0							
Locus_20264_Transcript_1/1_Conf_1.000	154	5	1.95E-13	EFO27571.1	76	78.9518	51	39	hypothetical protein LOAG_00907
Locus_20265_Transcript_1/1_Conf_1.000	790	5	2.70E-13	XP_002640068.1	53	80.1073	163	88	Hypothetical protein CBG12552
Locus_20266_Transcript_1/1_Conf_1.000	143	20	3.35E-18	NP_505810.2	95	94.7449	47	45	P21-Activated Kinase family member (pak-2)
Locus_20267_Transcript_1/1_Conf_1.000	410	3	3.39E-10	NP_741003.1	95	68.1662	41	39	hypothetical protein C18A3.4
Locus_20268_Transcript_1/1_Conf_1.000	220	0							
Locus_20269_Transcript_1/1_Conf_1.000	202	0							
Locus_2027_Transcript_1/1_Conf_1.000	1071	3	3.24E-11	NP_497269.1	43	73.9442	230	100	bZIP transcription factor family member (zip-2)
Locus_20270_Transcript_1/1_Conf_1.000	275	0							
Locus_20271_Transcript_1/1_Conf_1.000	226	20	2.30E-22	XP_002640668.1	95	108.612	60	57	C. briggsae CBR-UNC-54 protein
Locus_20272_Transcript_1/1_Conf_1.000	283	20	1.40E-27	NP_497791.1	83	125.946	93	78	NITrilase family member (nit-1)
Locus_20273_Transcript_1/1_Conf_1.000	183	16	5.70E-29	XP_002342629.1	100	130.568	61	61	PREDICTED: hypothetical protein XP_002342629
Locus_20274_Transcript_1/1_Conf_1.000	206	0							
Locus_20275_Transcript_1/1_Conf_1.000	129	0							
Locus_20276_Transcript_1/1_Conf_1.000	213	0							
Locus_20277_Transcript_1/1_Conf_1.000	336	4	7.25E-13	ACM46022.1	62	77.0258	102	64	Troponin t protein 3, isoform d
Locus_20278_Transcript_1/1_Conf_1.000	517	0							

Locus_20279_Transcript_1/1_Conf_1.000	159	0							
Locus_2028_Transcript_1/1_Conf_1.000	881	20	3.98E-136	A8X7H5.2	89	488.419	279	250	Adenylosuccinate synthetase
Locus_20280_Transcript_1/1_Conf_1.000	266	1	3.92E-06	XP_002641855.1	56	54.6842	89	50	Hypothetical protein CBG16529
Locus_20281_Transcript_1/1_Conf_1.000	311	20	9.11E-24	XP_002640257.1	74	113.235	101	75	Hypothetical protein CBG12782
Locus_20282_Transcript_1/1_Conf_1.000	471	20	5.96E-31	NP_001022174.1	68	137.117	129	88	hypothetical protein F45E10.2
Locus_20283_Transcript_1/1_Conf_1.000	610	20	3.65E-55	EFO26301.1	62	218.394	235	148	transcriptional enhancer factor TEF-3
Locus_20284_Transcript_1/1_Conf_1.000	429	20	7.76E-39	XP_002636280.1	78	163.31	128	100	Hypothetical protein CBG08570
Locus_20285_Transcript_1/1_Conf_1.000	294	20	2.69E-39	XP_002632893.1	88	164.851	98	87	Hypothetical protein CBG15101
Locus_20286_Transcript_1/1_Conf_1.000	472	4	2.49E-45	NP_495471.1	78	184.882	144	113	hypothetical protein F10E7.6
Locus_20287_Transcript_1/1_Conf_1.000	584	0							
Locus_20288_Transcript_1/1_Conf_1.000	311	20	7.20E-29	EFO24438.1	78	130.183	102	80	hypothetical protein LOAG_04046
Locus_20289_Transcript_1/1_Conf_1.000	156	0							
Locus_2029_Transcript_1/4_Conf_0.250	534	0							
Locus_2029_Transcript_2/4_Conf_0.250	576	0							
Locus_2029_Transcript_3/4_Conf_0.375	1644	20	0	NP_505657.1	95	692.575	394	377	ARp2/3 compleX component family member (arx-2)

Locus_2029_Transcript_4/4_Conf_0.375	1644	20	0	NP_505657.1	95	694.886	394	377	ARp2/3 compleX component family member (arx-2)
Locus_20290_Transcript_1/1_Conf_1.000	277	0							
Locus_20291_Transcript_1/1_Conf_1.000	401	2	6.91E-11	NP_495928.2	52	70.4774	136	71	hypothetical protein F37B12.3
Locus_20292_Transcript_1/1_Conf_1.000	222	20	7.94E-23	XP_002639618.1	80	110.153	71	57	Hypothetical protein CBG12331
Locus_20293_Transcript_1/1_Conf_1.000	144	0							
Locus_20294_Transcript_1/1_Conf_1.000	229	20	7.38E-21	XP_002811972.1	100	103.605	51	51	PREDICTED: small nuclear ribonucleoprotein G-like isoform 1
Locus_20295_Transcript_1/1_Conf_1.000	373	0							
Locus_20296_Transcript_1/1_Conf_1.000	132	0							
Locus_20297_Transcript_1/1_Conf_1.000	349	20	2.07E-23	EFO20853.1	64	112.079	119	77	aldehyde dehydrogenase 4
Locus_20298_Transcript_1/1_Conf_1.000	210	20	6.32E-33	XP_002800695.1	100	143.665	70	70	PREDICTED: protein disulfide-isomerase-like
Locus_20299_Transcript_1/1_Conf_1.000	243	9	5.21E-11	XP_002636805.1	72	70.8626	81	59	C. briggsae CBR-TTN-1 protein
Locus_203_Transcript_1/1_Conf_1.000	557	5	2.72E-05	XP_002646659.1	49	52.373	85	42	C. briggsae CBR-NURF-1 protein
Locus_2030_Transcript_1/3_Conf_0.500	736	20	5.28E-106	NP_509854.1	90	387.882	230	208	hypothetical protein W04G3.5

Locus_2030_Transcript_2/3_Conf_0.667	2018	20	5.24E-137	NP_509854.1	95	493.041	286	273	hypothetical protein W04G3.5
Locus_2030_Transcript_3/3_Conf_0.667	1622	20	4.83E-167	NP_509854.1	94	592.423	350	330	hypothetical protein W04G3.5
Locus_20300_Transcript_1/1_Conf_1.000	171	0							
Locus_20301_Transcript_1/1_Conf_1.000	140	0							
Locus_20302_Transcript_1/1_Conf_1.000	288	0							
Locus_20303_Transcript_1/1_Conf_1.000	570	4	1.92E-41	NP_741200.1	65	172.555	190	124	DIsplaced Gonad family member (dig-1)
Locus_20304_Transcript_1/1_Conf_1.000	250	20	1.71E-30	XP_002424532.1	89	135.576	83	74	GTP-binding protein Di-Ras2, putative
Locus_20305_Transcript_1/1_Conf_1.000	588	5	1.49E-39	NP_001024333.1	62	166.392	206	128	Olfactory LeaRNing defective family member (olrn-1)
Locus_20306_Transcript_1/1_Conf_1.000	169	0							
Locus_20307_Transcript_1/1_Conf_1.000	408	4	6.78E-35	NP_490772.2	77	150.214	136	105	hypothetical protein Y18H1A.3
Locus_20308_Transcript_1/1_Conf_1.000	143	0							
Locus_20309_Transcript_1/1_Conf_1.000	400	4	3.43E-10	XP_002647225.1	52	68.1662	131	69	Hypothetical protein CBG23815
Locus_2031_Transcript_1/2_Conf_1.000	1332	20	1.45E-86	XP_414473.2	69	324.709	312	217	PREDICTED: similar to multiple ankyrin repeats single KH domain protein
Locus_2031_Transcript_2/2_Conf_1.000	1332	20	1.45E-86	XP_414473.2	69	324.709	312	217	PREDICTED: similar to multiple ankyrin repeats single KH domain protein
Locus_20310_Transcript_1/1_Conf_1.000	138	0							
Locus_20311_Transcript_1/1_Conf_1.000	377	19	2.34E-43	XP_002338056.1	100	144.821	67	67	predicted protein
Locus_20312_Transcript_1/1_Conf_1.000	331	20	3.47E-47	XP_002637345.1	90	191.045	109	99	C. briggsae CBR-UNC-68 protein
Locus_20313_Transcript_1/1_Conf_1.000	397	0							
Locus_20314_Transcript_1/1_Conf_1.000	405	20	9.47E-45	NP_503758.4	79	182.956	134	106	KETtiN (Drosophila actin-binding) homolog family member (ketn-1)

Locus_20315_Transcript_1/1_Conf_1.000	137	1	3.26E-13	EAW60097.1	100	78.1814	36	36	myosin, heavy polypeptide 9, non-muscle, isoform CRA_b
Locus_20316_Transcript_1/1_Conf_1.000	377	9	6.89E-19	XP_002638736.1	68	97.0561	97	66	C. briggsae CBR-EGO-1 protein
Locus_20317_Transcript_1/1_Conf_1.000	231	3	9.31E-16	NP_496235.2	78	86.6557	78	61	Zinc finger Transcription Factor family member (ztf-27)
Locus_20318_Transcript_1/1_Conf_1.000	487	0							
Locus_20319_Transcript_1/1_Conf_1.000	164	20	4.16E-24	XP_002917683.1	100	114.39	54	54	PREDICTED: LOW QUALITY PROTEIN: dolichyl-diphosphooligosaccharide--protein glycosyltransferase subunit 1-like
Locus_2032_Transcript_1/2_Conf_1.000	994	20	1.80E-21	XP_002642942.1	46	107.842	283	132	Hypothetical protein CBG15220
Locus_2032_Transcript_2/2_Conf_1.000	985	20	1.04E-21	XP_002642942.1	46	108.612	283	131	Hypothetical protein CBG15220
Locus_20320_Transcript_1/1_Conf_1.000	404	0							
Locus_20321_Transcript_1/1_Conf_1.000	312	0							
Locus_20322_Transcript_1/1_Conf_1.000	342	0							
Locus_20323_Transcript_1/1_Conf_1.000	406	4	6.20E-12	NP_001021074.1	54	73.9442	133	73	Aboc, EXpulsion defective family member (aex-1)
Locus_20324_Transcript_1/1_Conf_1.000	312	20	7.44E-29	NP_493968.1	78	130.183	103	81	hypothetical protein Y51H7C.9
Locus_20325_Transcript_1/1_Conf_1.000	164	1	8.71E-06	XP_002639187.1	73	53.5286	38	28	Hypothetical protein CBG03729

Locus_20326_Transcript_1/1_Conf_1.000	404	20	3.26E-53	ADL62853.1	88	211.075	134	119	thioredoxin reductase 2
Locus_20327_Transcript_1/1_Conf_1.000	189	0							
Locus_20328_Transcript_1/1_Conf_1.000	313	5	7.20E-24	ABS50363.1	86	113.62	94	81	LIM domain protein variant
Locus_20329_Transcript_1/1_Conf_1.000	245	0							
Locus_2033_Transcript_1/1_Conf_1.000	581	20	6.90E-58	P51535.1	84	227.254	154	130	Myoglobin
Locus_20330_Transcript_1/1_Conf_1.000	146	0							
Locus_20331_Transcript_1/1_Conf_1.000	269	0							
Locus_20332_Transcript_1/1_Conf_1.000	156	8	4.35E-05	XP_002634715.1	65	51.2174	47	31	Hypothetical protein CBG24051
Locus_20333_Transcript_1/1_Conf_1.000	344	0							
Locus_20334_Transcript_1/1_Conf_1.000	344	20	1.55E-26	XP_002630719.1	82	122.479	80	66	C. briggsae CBR-DHS-5 protein
Locus_20335_Transcript_1/1_Conf_1.000	289	0							
Locus_20336_Transcript_1/1_Conf_1.000	258	4	9.69E-13	Q60V67.2	81	76.6406	49	40	Histone-H3-lysine-36 demethylase 1
Locus_20337_Transcript_1/1_Conf_1.000	299	16	2.52E-13	XP_002632937.1	71	78.5666	64	46	Hypothetical protein CBG21692
Locus_20338_Transcript_1/1_Conf_1.000	177	0							
Locus_20339_Transcript_1/1_Conf_1.000	294	20	2.52E-29	ACI49172.1	82	131.724	97	80	hypothetical protein Csp3_JD01.004

Locus_2034_Transcript_1/2_Conf_1.000	1859	20	0	XP_002631329.1	76	653.67	522	401	Hypothetical protein CBG03152
Locus_2034_Transcript_2/2_Conf_1.000	1734	20	2.64E-126	NP_495860.1	78	457.218	338	265	hypothetical protein K08F8.1
Locus_20340_Transcript_1/1_Conf_1.000	133	0							
Locus_20341_Transcript_1/1_Conf_1.000	152	0							
Locus_20342_Transcript_1/1_Conf_1.000	438	3	1.39E-64	NP_495881.2	86	248.825	145	126	hypothetical protein F46C5.2
Locus_20343_Transcript_1/1_Conf_1.000	230	14	5.68E-05	XP_002577435.1	64	50.8322	64	41	nonsense-mediated mRNA decay protein 1 (rent1)
Locus_20344_Transcript_1/1_Conf_1.000	230	20	2.80E-28	XP_002645190.1	89	128.257	75	67	C. briggsae CBR-PPK-3 protein
Locus_20345_Transcript_1/1_Conf_1.000	232	6	7.63E-10	NP_001021653.1	59	67.0106	74	44	FANCI (Fanconi anemia complex component I) homolog family member (fnci-1)
Locus_20346_Transcript_1/1_Conf_1.000	164	0							
Locus_20347_Transcript_1/1_Conf_1.000	182	20	2.04E-18	NP_491138.2	90	95.5153	60	54	SULfate Permease family member (sulp-6)

Locus_20348_Transcript_1/1_Conf_1.000	180	20	2.66E-26	XP_002801767.1	100	121.709	60	60	PREDICTED: sodium/potassium-transporting ATPase subunit alpha-1
Locus_20349_Transcript_1/1_Conf_1.000	272	5	1.80E-11	NP_493664.2	62	72.4034	89	56	hypothetical protein K10B4.3
Locus_2035_Transcript_1/1_Conf_1.000	1141	20	3.02E-103	NP_500218.2	76	379.793	362	277	Sex Peptide Receptor (Drosophila) Related family member (sprr-3)
Locus_20350_Transcript_1/1_Conf_1.000	151	0							
Locus_20351_Transcript_1/1_Conf_1.000	450	0							
Locus_20352_Transcript_1/1_Conf_1.000	160	0							
Locus_20353_Transcript_1/1_Conf_1.000	242	3	9.55E-05	EFO27722.1	56	50.0618	75	42	zinc finger protein
Locus_20354_Transcript_1/1_Conf_1.000	156	0							
Locus_20355_Transcript_1/1_Conf_1.000	468	20	1.98E-50	XP_002632055.1	90	201.83	132	120	C. briggsae CBR-VAB-19 protein
Locus_20356_Transcript_1/1_Conf_1.000	855	20	3.94E-24	P41234.4	48	116.316	321	155	ATP-binding cassette sub-family A member 2

Locus_20357_Transcript_1/1_Conf_1.000	543	4	2.07E-07	NP_494349.1	48	59.3066	152	74	hypothetical protein F29A7.6
Locus_20358_Transcript_1/1_Conf_1.000	604	20	2.49E-08	XP_415310.2	41	62.7734	197	81	PREDICTED: similar to heavy neurofilament protein
Locus_20359_Transcript_1/1_Conf_1.000	178	2	2.04E-07	XP_001894688.1	75	58.9214	41	31	Protein-tyrosine phosphatase containing protein
Locus_2036_Transcript_1/1_Conf_1.000	1530	20	1.33E-126	NP_001041105.1	74	457.988	377	282	EGg Laying defective family member (egl-9)
Locus_20360_Transcript_1/1_Conf_1.000	517	20	9.86E-43	NP_492839.4	74	176.407	171	127	Leucine-rich repeats, Ras-like domain, Kinase family member (lrk-1)
Locus_20361_Transcript_1/1_Conf_1.000	420	4	2.15E-12	NP_504235.2	67	75.485	83	56	Drosophila SOS homolog family member (sos-1)
Locus_20362_Transcript_1/1_Conf_1.000	265	0							
Locus_20363_Transcript_1/1_Conf_1.000	114	0							
Locus_20364_Transcript_1/1_Conf_1.000	250	5	6.31E-17	NP_504166.2	73	90.5077	76	56	hypothetical protein H43I07.2

Locus_20365_Transcript_1/1_Conf_1.000	233	4	1.58E-23	NP_504708.1	80	112.464	80	64	hypothetical protein T25F10.3
Locus_20366_Transcript_1/1_Conf_1.000	183	0							
Locus_20367_Transcript_1/1_Conf_1.000	238	0							
Locus_20368_Transcript_1/1_Conf_1.000	418	0							
Locus_20369_Transcript_1/1_Conf_1.000	276	20	5.37E-16	XP_002637140.1	100	87.4261	43	43	C. briggsae CBR-CAT-4 protein
Locus_2037_Transcript_1/1_Conf_1.000	205	20	1.57E-15	NP_495222.2	85	85.8853	67	57	hypothetical protein F21H12.1
Locus_20370_Transcript_1/1_Conf_1.000	219	0							
Locus_20371_Transcript_1/1_Conf_1.000	155	0							
Locus_20372_Transcript_1/1_Conf_1.000	665	0							
Locus_20373_Transcript_1/1_Conf_1.000	130	20	2.59E-18	BAH14542.1	100	95.1301	43	43	unnamed protein product
Locus_20374_Transcript_1/1_Conf_1.000	399	3	1.76E-06	XP_002423450.1	44	55.8398	127	56	conserved hypothetical protein
Locus_20375_Transcript_1/1_Conf_1.000	303	3	3.06E-11	XP_002642310.1	80	71.633	41	33	Hypothetical protein CBG18302
Locus_20376_Transcript_1/1_Conf_1.000	558	20	2.21E-15	XP_001894167.1	53	85.8853	175	93	Rad9 family protein
Locus_20377_Transcript_1/1_Conf_1.000	382	20	2.94E-54	XP_002640633.1	89	214.542	125	112	C. briggsae CBR-CATP-1 protein
Locus_20378_Transcript_1/1_Conf_1.000	224	0							
Locus_20379_Transcript_1/1_Conf_1.000	169	1	1.63E-04	XP_002632056.1	61	49.2914	54	33	Hypothetical protein CBG20437
Locus_2038_Transcript_1/2_Conf_1.000	2132	9	2.76E-59	XP_002639052.1	50	234.958	542	276	C. briggsae CBR-PQN-59.2 protein
Locus_2038_Transcript_2/2_Conf_1.000	2123	9	2.74E-59	XP_002639052.1	50	234.958	542	276	C. briggsae CBR-PQN-59.2 protein
Locus_20380_Transcript_1/1_Conf_1.000	299	0							
Locus_20381_Transcript_1/1_Conf_1.000	203	0							
Locus_20382_Transcript_1/1_Conf_1.000	251	0							

Locus_20383_Transcript_1/1_Conf_1.000	1124	20	4.21E-166	AAC38987.1	89	588.571	369	329	P-glycoprotein
Locus_20384_Transcript_1/1_Conf_1.000	137	0							
Locus_20385_Transcript_1/1_Conf_1.000	396	20	5.10E-38	ACJ65168.1	98	160.614	75	74	Uncoordinated protein 68, isoform b, partially confirmed by transcript evidence
Locus_20386_Transcript_1/1_Conf_1.000	242	20	1.79E-19	XP_002637220.1	76	98.9821	77	59	Hypothetical protein CBG18886
Locus_20387_Transcript_1/1_Conf_1.000	501	0							
Locus_20388_Transcript_1/1_Conf_1.000	271	0							
Locus_20389_Transcript_1/1_Conf_1.000	299	0							
Locus_2039_Transcript_1/1_Conf_1.000	1598	20	1.38E-166	NP_500596.2	71	590.882	503	362	hypothetical protein F19C7.4
Locus_20390_Transcript_1/1_Conf_1.000	298	0							
Locus_20391_Transcript_1/1_Conf_1.000	160	0							
Locus_20392_Transcript_1/1_Conf_1.000	206	0							
Locus_20393_Transcript_1/1_Conf_1.000	203	20	7.36E-05	EFO21349.1	70	50.447	67	47	hypothetical protein LOAG_07141
Locus_20394_Transcript_1/1_Conf_1.000	330	20	1.28E-41	NP_495662.2	90	172.555	105	95	PaTChed family member (ptc-1)

Locus_20395_Transcript_1/1_Conf_1.000	427	20	1.43E-24	XP_002641664.1	62	115.931	143	90	Hypothetical protein CBG09992
Locus_20396_Transcript_1/1_Conf_1.000	425	20	1.21E-23	CAA90256.2	79	112.849	112	89	C. elegans protein F54C9.11, confirmed by transcript evidence
Locus_20397_Transcript_1/1_Conf_1.000	154	0							
Locus_20398_Transcript_1/1_Conf_1.000	889	4	5.31E-11	NP_499003.1	50	72.7886	184	92	hypothetical protein R107.5
Locus_20399_Transcript_1/1_Conf_1.000	226	0							
Locus_204_Transcript_1/1_Conf_1.000	3308	20	4.04E-15	NP_491244.1	37	88.9669	678	252	HoloCentric chromosome binding Protein family member (hcp-4)
Locus_2040_Transcript_1/1_Conf_1.000	1213	20	2.23E-99	XP_002635668.1	72	367.081	345	249	Hypothetical protein CBG21865
Locus_20400_Transcript_1/1_Conf_1.000	566	0							
Locus_20401_Transcript_1/1_Conf_1.000	508	0							
Locus_20402_Transcript_1/1_Conf_1.000	913	20	1.62E-10	NP_491784.1	45	71.2478	268	123	hypothetical protein T05E7.3
Locus_20403_Transcript_1/1_Conf_1.000	285	20	3.82E-17	XP_001896129.1	68	91.2781	98	67	Hypothetical 216.3 kDa protein R06F6.8 in chromosome II, putative
Locus_20404_Transcript_1/1_Conf_1.000	199	0							
Locus_20405_Transcript_1/1_Conf_1.000	189	0							
Locus_20406_Transcript_1/1_Conf_1.000	329	0							
Locus_20407_Transcript_1/1_Conf_1.000	497	5	3.36E-34	NP_001023038.1	79	147.902	166	132	EXCretory canal abnormal family member (exc-5)
Locus_20408_Transcript_1/1_Conf_1.000	146	3	4.71E-07	NP_498918.3	80	57.7658	40	32	hypothetical protein B0303.7
Locus_20409_Transcript_1/1_Conf_1.000	227	0							
Locus_2041_Transcript_1/1_Conf_1.000	1385	4	1.12E-20	EFO20584.1	62	105.916	127	79	hypothetical protein LOAG_07907
Locus_20410_Transcript_1/1_Conf_1.000	275	0							
Locus_20411_Transcript_1/1_Conf_1.000	242	1	9.86E-10	NP_502377.2	72	66.6254	54	39	hypothetical protein C25G4.3
Locus_20412_Transcript_1/1_Conf_1.000	306	0							

Locus_20413_Transcript_1/1_Conf_1.000	153	20	9.68E-21	XP_002761135.1	100	103.219	50	50	PREDICTED: proteasome subunit beta type-10-like
Locus_20414_Transcript_1/1_Conf_1.000	288	20	3.11E-27	NP_001129887.1	82	124.79	91	75	SEC(selenocysteine)-tRNA Synthase family member (secs-1)
Locus_20415_Transcript_1/1_Conf_1.000	361	20	1.56E-31	XP_002633437.1	78	139.043	119	93	C. briggsae CBR-UNC-22 protein
Locus_20416_Transcript_1/1_Conf_1.000	811	20	8.96E-52	XP_001892617.1	57	207.994	272	156	Protein kinase domain containing protein
Locus_20417_Transcript_1/1_Conf_1.000	420	9	6.92E-19	XP_002634184.1	57	97.0561	138	80	C. briggsae CBR-QUI-1 protein
Locus_20418_Transcript_1/1_Conf_1.000	340	20	1.01E-14	AAD31839.1	56	83.1889	97	55	AF132291_1ancylostoma-secreted protein 1 precursor
Locus_20419_Transcript_1/1_Conf_1.000	294	20	1.05E-51	BAG60276.1	100	206.068	98	98	unnamed protein product
Locus_2042_Transcript_1/1_Conf_1.000	988	20	1.12E-116	NP_497726.2	77	424.091	332	256	hypothetical protein M01F1.7
Locus_20420_Transcript_1/1_Conf_1.000	533	0							

Locus_20421_Transcript_1/1_Conf_1.000	419	20	4.94E-41	NP_499920.1	81	170.629	119	97	hypothetical protein T07A9.8
Locus_20422_Transcript_1/1_Conf_1.000	135	5	2.95E-14	NP_492521.1	95	81.6481	45	43	SynTrophiN family member (stn-1)
Locus_20423_Transcript_1/1_Conf_1.000	206	7	1.93E-05	EFO24901.1	61	52.373	47	29	hypothetical protein LOAG_03581
Locus_20424_Transcript_1/1_Conf_1.000	162	0							
Locus_20425_Transcript_1/1_Conf_1.000	139	20	1.19E-15	XP_002917678.1	100	86.2705	43	43	PREDICTED: protein transport protein Sec61 subunit alpha isoform 1-like
Locus_20426_Transcript_1/1_Conf_1.000	219	8	1.19E-10	XP_002630904.1	67	69.707	65	44	Hypothetical protein CBG02628
Locus_20427_Transcript_1/1_Conf_1.000	467	3	9.33E-16	XP_002641334.1	59	86.6557	126	75	C. briggsae CBR-HMT-1 protein
Locus_20428_Transcript_1/1_Conf_1.000	305	20	4.13E-16	XP_002643868.1	71	87.8113	81	58	Hypothetical protein CBG02104
Locus_20429_Transcript_1/1_Conf_1.000	307	0							
Locus_2043_Transcript_1/1_Conf_1.000	686	0							
Locus_20430_Transcript_1/1_Conf_1.000	286	5	1.65E-36	NP_740906.1	90	155.606	90	81	hypothetical protein F26E4.7
Locus_20431_Transcript_1/1_Conf_1.000	297	0							
Locus_20432_Transcript_1/1_Conf_1.000	135	20	3.49E-07	NP_491634.1	82	58.151	45	37	hypothetical protein M01E11.2

Locus_20433_Transcript_1/1_Conf_1.000	199	20	2.00E-26	CAB00098.2	92	122.094	66	61	C. elegans protein F57F5.1, confirmed by transcript evidence
Locus_20434_Transcript_1/1_Conf_1.000	290	20	5.87E-18	NP_001024053.1	56	93.9745	116	65	UNCoordinated family member (unc-70)
Locus_20435_Transcript_1/1_Conf_1.000	197	20	3.10E-19	XP_002629656.1	89	98.2117	57	51	Hypothetical protein CBG00863
Locus_20436_Transcript_1/2_Conf_1.000	448	20	1.57E-47	XP_002631186.1	75	192.2	150	113	C. briggsae CBR-UNC-53 protein
Locus_20436_Transcript_2/2_Conf_1.000	459	20	6.92E-48	XP_002631186.1	75	193.356	153	115	C. briggsae CBR-UNC-53 protein
Locus_20437_Transcript_1/1_Conf_1.000	162	2	4.18E-08	NP_505894.2	86	61.2326	37	32	hypothetical protein K03B8.6
Locus_20438_Transcript_1/1_Conf_1.000	447	20	9.87E-34	XP_001898089.1	69	146.362	148	103	uridine monophosphate kinase
Locus_20439_Transcript_1/1_Conf_1.000	169	1	6.20E-04	XP_001896579.1	75	47.3654	53	40	hypothetical protein Bm1_25600
Locus_2044_Transcript_1/1_Conf_1.000	1097	20	8.97E-73	XP_002639582.1	80	278.485	225	180	C. briggsae CBR-TRAP-1 protein
Locus_20440_Transcript_1/1_Conf_1.000	232	0							
Locus_20441_Transcript_1/1_Conf_1.000	870	4	1.00E-38	NP_508466.2	68	164.851	155	106	hypothetical protein H01M10.1
Locus_20442_Transcript_1/1_Conf_1.000	178	0							
Locus_20443_Transcript_1/1_Conf_1.000	152	0							
Locus_20444_Transcript_1/1_Conf_1.000	225	0							
Locus_20445_Transcript_1/1_Conf_1.000	399	0							
Locus_20446_Transcript_1/1_Conf_1.000	262	0							
Locus_20447_Transcript_1/1_Conf_1.000	363	20	1.56E-23	XP_001899525.1	65	112.464	126	83	C-terminal binding protein

Locus_20448_Transcript_1/1_Conf_1.000	790	20	4.24E-75	NP_498392.1	70	285.419	265	186	THO Complex (transcription factor/nuclear export) subunit family member (thoc-2)
Locus_20449_Transcript_1/1_Conf_1.000	252	2	9.11E-19	XP_002640627.1	74	96.6709	75	56	C. briggsae CBR-UNC-95 protein
Locus_2045_Transcript_1/2_Conf_1.000	2189	20	4.56E-142	NP_001024503.1	68	509.99	515	355	hypothetical protein C55B6.1
Locus_2045_Transcript_2/2_Conf_1.000	1601	20	2.23E-132	NP_001024503.1	69	477.248	470	326	hypothetical protein C55B6.1
Locus_20450_Transcript_1/1_Conf_1.000	186	0							
Locus_20451_Transcript_1/1_Conf_1.000	184	0							
Locus_20452_Transcript_1/1_Conf_1.000	146	0							
Locus_20453_Transcript_1/1_Conf_1.000	906	20	1.24E-79	NP_001122707.1	75	300.827	247	187	hypothetical protein K02D10.1
Locus_20454_Transcript_1/1_Conf_1.000	258	0							
Locus_20455_Transcript_1/1_Conf_1.000	227	0							
Locus_20456_Transcript_1/1_Conf_1.000	578	4	1.39E-10	NP_001129872.1	53	70.0922	143	76	hypothetical protein T23B5.3
Locus_20457_Transcript_1/1_Conf_1.000	487	0							
Locus_20458_Transcript_1/1_Conf_1.000	400	0							
Locus_20459_Transcript_1/1_Conf_1.000	155	0							
Locus_2046_Transcript_1/2_Conf_1.000	648	2	4.15E-10	ABB53347.1	42	68.9366	186	79	secreted protein 5 precursor
Locus_2046_Transcript_2/2_Conf_1.000	222	0							
Locus_20460_Transcript_1/1_Conf_1.000	517	0							
Locus_20461_Transcript_1/1_Conf_1.000	266	20	1.09E-40	NP_510020.2	88	169.474	88	78	hypothetical protein W03G11.3
Locus_20462_Transcript_1/1_Conf_1.000	173	0							
Locus_20463_Transcript_1/1_Conf_1.000	177	20	8.53E-22	AAV32848.1	91	106.686	59	54	egl-20
Locus_20464_Transcript_1/1_Conf_1.000	251	4	1.01E-06	NP_501580.3	60	56.6102	71	43	LiPoate Ligase family member (lpl-1)
Locus_20465_Transcript_1/1_Conf_1.000	439	4	1.35E-43	NP_510514.2	76	179.104	153	117	hypothetical protein R07D5.2

Locus_20466_Transcript_1/1_Conf_1.000	200	20	1.14E-13	XP_002639317.1	85	79.7221	49	42	C. briggsae CBR-ABT-2 protein
Locus_20467_Transcript_1/1_Conf_1.000	327	20	3.36E-58	NP_509585.1	100	227.639	108	108	Tubulin, Beta family member (tbb-4)
Locus_20468_Transcript_1/1_Conf_1.000	301	0							
Locus_20469_Transcript_1/1_Conf_1.000	159	20	1.18E-18	NP_491028.2	88	96.2857	52	46	abnormal cell LINeage family member (lin-17)
Locus_2047_Transcript_1/1_Conf_1.000	1489	20	0	XP_002638927.1	83	650.588	498	416	Hypothetical protein CBG22153
Locus_20470_Transcript_1/1_Conf_1.000	335	1	6.17E-04	XP_002638631.1	54	47.3654	97	53	Hypothetical protein CBG05684
Locus_20471_Transcript_1/1_Conf_1.000	428	7	7.34E-13	XP_002641828.1	50	77.0258	155	79	C. briggsae CBR-PRK-1 protein
Locus_20472_Transcript_1/1_Conf_1.000	163	4	7.61E-10	NP_001033357.2	82	67.0106	45	37	DAF-16/FOXO Controlled, germline Tumor affecting family member (dct-6)
Locus_20473_Transcript_1/1_Conf_1.000	157	0							
Locus_20474_Transcript_1/1_Conf_1.000	153	0							
Locus_20475_Transcript_1/1_Conf_1.000	271	20	1.74E-22	XP_002646294.1	78	108.997	89	70	C. briggsae CBR-RIC-19 protein
Locus_20476_Transcript_1/1_Conf_1.000	225	0							
Locus_20477_Transcript_1/1_Conf_1.000	210	0							

Locus_20478_Transcript_1/1_Conf_1.000	418	20	5.49E-24	NP_491115.1	64	114.005	137	89	hypothetical protein Y54E10A.6
Locus_20479_Transcript_1/1_Conf_1.000	302	0							
Locus_2048_Transcript_1/1_Conf_1.000	903	20	1.11E-96	NP_001122845.1	87	357.451	234	204	Protein Phosphatase 2A (Two A) Regulatory subunit family member (pptr-2)
Locus_20480_Transcript_1/1_Conf_1.000	148	0							
Locus_20481_Transcript_1/1_Conf_1.000	167	0							
Locus_20482_Transcript_1/1_Conf_1.000	162	0							
Locus_20483_Transcript_1/1_Conf_1.000	129	0							
Locus_20484_Transcript_1/1_Conf_1.000	357	2	1.90E-53	XP_002647011.1	94	211.846	104	98	Hypothetical protein CBG23797
Locus_20485_Transcript_1/1_Conf_1.000	345	0							
Locus_20486_Transcript_1/1_Conf_1.000	502	20	1.13E-77	XP_002637163.1	92	292.352	167	154	C. briggsae CBR-SQV-4 protein

Locus_20487_Transcript_1/1_Conf_1.000	141	20	2.25E-06	XP_002429811.1	76	55.4546	42	32	class A rhodopsin-like G-protein coupled receptor GPRfsh, putative
Locus_20488_Transcript_1/1_Conf_1.000	280	0							
Locus_20489_Transcript_1/1_Conf_1.000	167	0							
Locus_2049_Transcript_1/1_Conf_1.000	668	20	1.51E-111	XP_002640980.1	90	405.986	221	201	Hypothetical protein CBG11728
Locus_20490_Transcript_1/1_Conf_1.000	205	0							
Locus_20491_Transcript_1/1_Conf_1.000	207	20	2.67E-23	NP_496968.1	95	111.694	67	64	hypothetical protein Y48B6A.12
Locus_20492_Transcript_1/1_Conf_1.000	163	1	6.67E-06	XP_002643614.1	72	53.9138	43	31	C. briggsae CBR-SER-1 protein
Locus_20493_Transcript_1/1_Conf_1.000	639	20	1.58E-99	NP_510361.1	89	365.925	212	189	SYnthetic lethal with Mec family member (sym-4)
Locus_20494_Transcript_1/1_Conf_1.000	172	0							
Locus_20495_Transcript_1/1_Conf_1.000	137	0							
Locus_20496_Transcript_1/1_Conf_1.000	294	4	1.53E-18	NP_506256.3	67	95.9005	96	65	CaDHerin family member (cdh-6)
Locus_20497_Transcript_1/1_Conf_1.000	140	0							

Locus_20498_Transcript_1/1_Conf_1.000	396	0							
Locus_20499_Transcript_1/1_Conf_1.000	224	10	4.36E-21	NP_001129803.1	82	104.375	74	61	Leucyl tRNA Synthetase family member (lrs-2)
Locus_205_Transcript_1/1_Conf_1.000	762	20	4.09E-56	NP_496817.1	74	222.246	191	142	hypothetical protein Y48C3A.10
Locus_2050_Transcript_1/1_Conf_1.000	320	0							
Locus_20500_Transcript_1/1_Conf_1.000	230	2	3.56E-07	XP_001893874.1	75	58.151	52	39	Smr domain containing protein
Locus_20501_Transcript_1/1_Conf_1.000	614	20	4.26E-27	XP_002643092.1	62	125.176	211	132	Hypothetical protein CBG23016
Locus_20502_Transcript_1/1_Conf_1.000	484	20	2.04E-87	P13498.2	100	324.709	157	157	Cytochrome b-245 light chain
Locus_20503_Transcript_1/1_Conf_1.000	237	20	7.31E-21	ACI49169.1	78	103.605	76	60	hypothetical protein Csp3_JD01.001
Locus_20504_Transcript_1/1_Conf_1.000	270	0							
Locus_20505_Transcript_1/1_Conf_1.000	157	0							
Locus_20506_Transcript_1/1_Conf_1.000	211	20	1.79E-19	XP_001894872.1	79	98.9821	69	55	hypothetical protein Bm1_17075
Locus_20507_Transcript_1/1_Conf_1.000	194	5	1.49E-13	XP_002637665.1	77	79.337	57	44	C. briggsae CBR-APT-10 protein
Locus_20508_Transcript_1/1_Conf_1.000	133	5	1.98E-10	XP_001893497.1	92	68.9366	41	38	Viral A-type inclusion protein repeat containing protein
Locus_20509_Transcript_1/1_Conf_1.000	346	6	5.13E-22	CAD11605.1	79	107.457	115	91	metalloprotease

Locus_2051_Transcript_1/1_Conf_1.000	202	20	6.00E-15	XP_002637392.1	76	83.9593	67	51	Hypothetical protein CBG19099
Locus_20510_Transcript_1/1_Conf_1.000	551	20	6.85E-30	XP_001900782.1	62	134.035	144	90	mKIAA2025 protein
Locus_20511_Transcript_1/1_Conf_1.000	421	20	4.05E-27	NP_505989.1	72	124.405	140	102	SULfate Permease family member (sulp-4)
Locus_20512_Transcript_1/1_Conf_1.000	549	5	3.65E-15	XP_002633339.1	57	85.1149	110	63	Hypothetical protein CBG06078
Locus_20513_Transcript_1/1_Conf_1.000	367	20	2.17E-17	XP_002643252.1	78	92.0485	74	58	C. briggsae CBR-TRAP-2 protein
Locus_20514_Transcript_1/1_Conf_1.000	148	0							
Locus_20515_Transcript_1/1_Conf_1.000	792	20	1.53E-48	XP_002641022.1	86	197.208	119	103	C. briggsae CBR-GLRX-5 protein
Locus_20516_Transcript_1/1_Conf_1.000	432	20	3.55E-76	CAM84506.1	96	287.345	143	138	serine/threonine phosphatase
Locus_20517_Transcript_1/1_Conf_1.000	203	0							
Locus_20518_Transcript_1/1_Conf_1.000	269	20	3.50E-31	NP_502314.1	91	137.887	73	67	hypothetical protein F35G2.1
Locus_20519_Transcript_1/1_Conf_1.000	265	20	3.64E-28		83	127.872	89	74	myoblast city protein homolog

Locus_2052_Transcript_1/2_Conf_1.000	716	20	3.18E-20	XP_001896583.1	87	102.834	65	57	high mobility group protein
Locus_2052_Transcript_2/2_Conf_1.000	470	0							
Locus_20520_Transcript_1/1_Conf_1.000	204	20	2.28E-06	XP_002181319.1	65	55.4546	63	41	predicted protein
Locus_20521_Transcript_1/1_Conf_1.000	353	2	9.64E-05	EFO18076.1	64	50.0618	59	38	DNA-dependent RNA polymerase
Locus_20522_Transcript_1/1_Conf_1.000	327	20	4.87E-41	NP_492476.1	91	170.629	106	97	hypothetical protein F14B4.3
Locus_20523_Transcript_1/1_Conf_1.000	219	0							
Locus_20524_Transcript_1/1_Conf_1.000	145	0							
Locus_20525_Transcript_1/1_Conf_1.000	140	0							
Locus_20526_Transcript_1/1_Conf_1.000	135	0							
Locus_20527_Transcript_1/1_Conf_1.000	129	0							

Locus_20528_Transcript_1/1_Conf_1.000	414	20	3.00E-59	EFO21414.1	89	231.106	137	122	hypothetical protein LOAG_07072
Locus_20529_Transcript_1/1_Conf_1.000	220	0							
Locus_2053_Transcript_1/1_Conf_1.000	511	20	2.11E-18	XP_001893088.1	61	95.5153	112	69	Glutaredoxin family protein
Locus_20530_Transcript_1/1_Conf_1.000	137	20	6.57E-14	NP_492593.2	93	80.4925	45	42	hypothetical protein F26E4.3
Locus_20531_Transcript_1/1_Conf_1.000	282	0							
Locus_20532_Transcript_1/1_Conf_1.000	415	5	3.24E-21	XP_002630781.1	62	104.76	121	76	Hypothetical protein CBG02477
Locus_20533_Transcript_1/1_Conf_1.000	335	0							
Locus_20534_Transcript_1/1_Conf_1.000	130	0							
Locus_20535_Transcript_1/1_Conf_1.000	165	0							
Locus_20536_Transcript_1/1_Conf_1.000	197	20	9.30E-24	XP_001088441.2	100	113.235	55	55	PREDICTED: transcription elongation factor B polypeptide 2-like
Locus_20537_Transcript_1/1_Conf_1.000	219	0							
Locus_20538_Transcript_1/1_Conf_1.000	504	20	3.62E-47	XP_002633316.1	75	191.045	166	126	C. briggsae CBR-GTL-2 protein
Locus_20539_Transcript_1/1_Conf_1.000	193	0							
Locus_2054_Transcript_1/1_Conf_1.000	1193	7	1.03E-32	XP_002638536.1	45	145.591	430	197	C. briggsae CBR-NUM-1 protein
Locus_20540_Transcript_1/1_Conf_1.000	276	0							
Locus_20541_Transcript_1/1_Conf_1.000	174	0							
Locus_20542_Transcript_1/1_Conf_1.000	350	0							
Locus_20543_Transcript_1/1_Conf_1.000	685	9	3.45E-21	XP_002581843.1	55	105.916	161	90	hypothetical protein

Locus_20544_Transcript_1/1_Conf_1.000	291	20	1.81E-43	NP_496205.2	93	178.718	96	90	PhosphoLipase C family member (plc-3)
Locus_20545_Transcript_1/1_Conf_1.000	273	20	2.18E-25	XP_002638629.1	77	118.627	88	68	C. briggsae CBR-TWK-33 protein
Locus_20546_Transcript_1/1_Conf_1.000	130	0							
Locus_20547_Transcript_1/1_Conf_1.000	204	0							
Locus_20548_Transcript_1/1_Conf_1.000	167	4	1.29E-09	XP_002641345.1	76	66.2402	55	42	Hypothetical protein CBG13198
Locus_20549_Transcript_1/1_Conf_1.000	228	1	7.15E-16	BAG59555.1	100	87.0409	38	38	unnamed protein product
Locus_2055_Transcript_1/1_Conf_1.000	2231	20	7.19E-151	EFO27499.1	75	539.265	473	357	hypothetical protein LOAG_00977
Locus_20550_Transcript_1/1_Conf_1.000	158	0							
Locus_20551_Transcript_1/1_Conf_1.000	225	0							
Locus_20552_Transcript_1/1_Conf_1.000	271	12	2.98E-06	XP_002660579.1	54	55.0694	86	47	PREDICTED: hypothetical protein
Locus_20553_Transcript_1/1_Conf_1.000	247	1	5.03E-06	NP_495345.1	61	54.299	78	48	hypothetical protein F54H5.3
Locus_20554_Transcript_1/1_Conf_1.000	148	0							

Locus_20555_Transcript_1/1_Conf_1.000	346	20	7.89E-31	XP_002635068.1	78	136.732	89	70	C. briggsae CBR-MOM-2 protein
Locus_20556_Transcript_1/1_Conf_1.000	239	3	2.44E-08	NP_001129867.1	69	62.003	72	50	hypothetical protein T05E11.2
Locus_20557_Transcript_1/1_Conf_1.000	373	5	1.27E-04	ZP_02210846.1	40	49.6766	100	40	hypothetical protein CLOBAR_00414
Locus_20558_Transcript_1/1_Conf_1.000	294	20	3.51E-39	NP_496448.1	87	164.466	98	86	hypothetical protein W02B12.10
Locus_20559_Transcript_1/1_Conf_1.000	207	2	1.13E-05	XP_001900715.1	57	53.1434	69	40	hypothetical protein
Locus_2056_Transcript_1/1_Conf_1.000	435	17	1.97E-10	XP_001900570.1	64	68.9366	85	55	ThiS family protein
Locus_20560_Transcript_1/1_Conf_1.000	380	2	4.44E-26	NP_496074.2	74	120.939	109	81	hypothetical protein ZK1307.7
Locus_20561_Transcript_1/1_Conf_1.000	454	20	1.67E-41	XP_002642775.1	78	172.17	132	104	C. briggsae CBR-NEX-1 protein
Locus_20562_Transcript_1/1_Conf_1.000	168	0							
Locus_20563_Transcript_1/1_Conf_1.000	212	0							
Locus_20564_Transcript_1/1_Conf_1.000	222	20	7.81E-31	EAW91906.1	100	87.4261	39	39	hCG2008737
Locus_20565_Transcript_1/1_Conf_1.000	148	0							
Locus_20566_Transcript_1/1_Conf_1.000	137	0							
Locus_20567_Transcript_1/1_Conf_1.000	582	20	1.71E-88	CAR63545.1	91	328.946	196	179	putative Hsp90 protein
Locus_20568_Transcript_1/1_Conf_1.000	275	20	6.78E-35	XP_002644234.1	90	150.214	91	82	C. briggsae CBR-MIG-15 protein
Locus_20569_Transcript_1/1_Conf_1.000	280	0							
Locus_2057_Transcript_1/1_Conf_1.000	2326	20	9.20E-56	NP_001122456.1	49	223.402	500	245	Protein Phosphatase Four Regulatory subunit family member (ppfr-1)
Locus_20570_Transcript_1/1_Conf_1.000	266	20	3.08E-27	XP_002641517.1	82	124.79	88	73	Hypothetical protein CBG09813
Locus_20571_Transcript_1/1_Conf_1.000	324	20	4.74E-36	NP_491855.1	84	154.066	109	92	Nuclear Pore complex Protein family member (npp-12)

Locus_20572_Transcript_1/1_Conf_1.000	172	20	6.17E-12	XP_863776.1	83	73.9442	55	46	PREDICTED: similar to Poly [ADP-ribose] polymerase-1 (PARP-1) (ADPRT) (NAD(+) ADP-ribosyltransferase-1) (Poly[ADP-ribose] synthetase-1) isoform 2
Locus_20573_Transcript_1/1_Conf_1.000	175	0							
Locus_20574_Transcript_1/1_Conf_1.000	343	0							
Locus_20575_Transcript_1/1_Conf_1.000	149	0							
Locus_20576_Transcript_1/1_Conf_1.000	182	16	2.04E-10	XP_002631174.1	71	68.9366	60	43	Hypothetical protein CBG02960
Locus_20577_Transcript_1/1_Conf_1.000	362	20	2.18E-25	XP_002642982.1	66	118.627	117	78	Hypothetical protein CBG15266
Locus_20578_Transcript_1/1_Conf_1.000	130	1	6.63E-06	NP_509023.1	88	53.9138	34	30	hypothetical protein C15H9.7
Locus_20579_Transcript_1/1_Conf_1.000	177	5	5.74E-10	NP_492476.1	75	67.3958	58	44	hypothetical protein F14B4.3
Locus_2058_Transcript_1/1_Conf_1.000	879	20	1.14E-74	XP_002647128.1	95	284.263	163	155	Hypothetical protein CBG23912
Locus_20580_Transcript_1/1_Conf_1.000	245	5	3.50E-07	XP_002636960.1	70	58.151	61	43	Hypothetical protein CBG09439
Locus_20581_Transcript_1/1_Conf_1.000	255	0							
Locus_20582_Transcript_1/1_Conf_1.000	211	0							
Locus_20583_Transcript_1/1_Conf_1.000	357	0							
Locus_20584_Transcript_1/1_Conf_1.000	261	20	1.88E-08	EFO16652.1	58	62.3882	68	40	hypothetical protein LOAG_11852
Locus_20585_Transcript_1/1_Conf_1.000	575	11	1.22E-51	NP_498645.1	86	206.453	192	167	MUscle Positioning family member (mup-4)
Locus_20586_Transcript_1/1_Conf_1.000	160	0							
Locus_20587_Transcript_1/1_Conf_1.000	196	0							
Locus_20588_Transcript_1/1_Conf_1.000	174	0							

Locus_20589_Transcript_1/1_Conf_1.000	270	18	1.99E-18	XP_002642615.1	59	95.5153	89	53	C. briggsae CBR-MUP-4 protein
Locus_2059_Transcript_1/1_Conf_1.000	559	5	5.82E-08	NP_001022759.1	53	61.2326	100	53	hypothetical protein T12D8.9
Locus_20590_Transcript_1/1_Conf_1.000	709								
Locus_20591_Transcript_1/1_Conf_1.000	260	0							
Locus_20592_Transcript_1/1_Conf_1.000	302	0							
Locus_20593_Transcript_1/1_Conf_1.000	178	0							
Locus_20594_Transcript_1/1_Conf_1.000	144	0							
Locus_20595_Transcript_1/1_Conf_1.000	149	0							
Locus_20596_Transcript_1/1_Conf_1.000	698	20	3.96E-28	XP_001899546.1	54	129.028	241	131	hypothetical protein
Locus_20597_Transcript_1/1_Conf_1.000	215	3	2.58E-10	XP_002632188.1	70	68.5514	70	49	C. briggsae CBR-SGCB-1 protein
Locus_20598_Transcript_1/1_Conf_1.000	274	19	2.35E-11	XP_002648597.1	63	72.0182	90	57	Hypothetical protein CBG24931
Locus_20599_Transcript_1/1_Conf_1.000	246	20	5.55E-13	NP_001041088.1	71	77.411	63	45	hypothetical protein C27A7.3
Locus_206_Transcript_1/5_Conf_0.450	391	14	1.61E-12	XP_002484510.1	64	75.8702	74	48	hypothetical protein TSTA_040370
Locus_206_Transcript_2/5_Conf_0.400	395	14	1.66E-12	XP_002484510.1	64	75.8702	74	48	hypothetical protein TSTA_040370
Locus_206_Transcript_3/5_Conf_0.650	446	12	1.67E-14	XP_002484510.1	64	75.8702	74	48	hypothetical protein TSTA_040370
Locus_206_Transcript_5/5_Conf_0.750	497	15	1.65E-14	XP_002484510.1	64	75.8702	74	48	hypothetical protein TSTA_040370
Locus_2060_Transcript_1/2_Conf_1.000	1414	0							
Locus_2060_Transcript_2/2_Conf_1.000	1393	0							
Locus_20600_Transcript_1/1_Conf_1.000	278	2	1.46E-13	NP_491385.2	59	79.337	89	53	hypothetical protein Y119C1B.9
Locus_20601_Transcript_1/1_Conf_1.000	132	0							
Locus_20602_Transcript_1/1_Conf_1.000	308	0							
Locus_20603_Transcript_1/1_Conf_1.000	223	11	7.67E-26	XP_002634505.1	90	120.168	74	67	Hypothetical protein CBG08295
Locus_20604_Transcript_1/1_Conf_1.000	180	19	1.08E-19	NP_001065270.1	100	99.7525	46	46	NADH dehydrogenase ubiquinone 1 beta subcomplex subunit 7
Locus_20605_Transcript_1/1_Conf_1.000	385	20	2.18E-33	XP_001893379.1	76	145.206	102	78	Malate/L-lactate dehydrogenase family protein
Locus_20606_Transcript_1/1_Conf_1.000	183	20	5.35E-19	AAD48989.1	86	97.4413	59	51	P-glycoprotein
Locus_20607_Transcript_1/1_Conf_1.000	527	0							
Locus_20608_Transcript_1/1_Conf_1.000	1108	20	1.08E-73	NP_509318.1	62	281.567	315	198	ADAM (disintegrin plus metalloprotease) family member (adm-4)
Locus_20609_Transcript_1/1_Conf_1.000	251	0							
Locus_2061_Transcript_1/1_Conf_1.000	527	1	2.53E-04	NP_001028814.1	41	48.9062	108	45	C4b-binding protein alpha chain precursor

Locus_20610_Transcript_1/1_Conf_1.000	192	0								
Locus_20611_Transcript_1/1_Conf_1.000	372	3	5.67E-13	NP_506162.1	62	77.411	97	61	abnormal cell LINeage family member (lin-25)
Locus_20612_Transcript_1/1_Conf_1.000	312	20	1.36E-30	CAR63696.1	92	135.961	77	71	putative Uncoordinated protein 23
Locus_20613_Transcript_1/1_Conf_1.000	532	20	6.61E-64	XP_002633391.1	83	246.899	161	135	Hypothetical protein CBG06150
Locus_20614_Transcript_1/1_Conf_1.000	146	0								
Locus_20615_Transcript_1/1_Conf_1.000	381	0								
Locus_20616_Transcript_1/1_Conf_1.000	287	0								
Locus_20617_Transcript_1/1_Conf_1.000	647	20	4.81E-35	XP_002633591.1	83	151.754	129	108	Hypothetical protein CBG05468
Locus_20618_Transcript_1/1_Conf_1.000	348	0								
Locus_20619_Transcript_1/1_Conf_1.000	228	0								
Locus_2062_Transcript_1/1_Conf_1.000	1630	20	1.52E-144	NP_498913.2	83	517.694	403	338	FLI-I (Drosophila flightless) homolog family member (fli-1)
Locus_20620_Transcript_1/1_Conf_1.000	159	0								
Locus_20621_Transcript_1/1_Conf_1.000	323	3	1.95E-05	XP_001892569.1	78	52.373	38	30	exonuclease family protein
Locus_20622_Transcript_1/1_Conf_1.000	443	3	4.66E-07	NP_501744.1	51	57.7658	100	51	SM Y trans-splicing-associated snRNA family member (sna-2)
Locus_20623_Transcript_1/1_Conf_1.000	186	20	2.09E-23	XP_002639867.1	95	112.079	62	59	Hypothetical protein CBG12222
Locus_20624_Transcript_1/1_Conf_1.000	204	0								
Locus_20625_Transcript_1/1_Conf_1.000	605	5	3.19E-80	NP_001123175.1	82	301.597	199	165	ImmunoGlobulin-like Cell adhesion Molecule family member (igcm-3)
Locus_20626_Transcript_1/1_Conf_1.000	144	0								
Locus_20627_Transcript_1/1_Conf_1.000	209	0								
Locus_20628_Transcript_1/1_Conf_1.000	149	20	1.90E-24	BAG64777.1	100	115.546	49	49	unnamed protein product
Locus_20629_Transcript_1/1_Conf_1.000	228	0								
Locus_2063_Transcript_1/1_Conf_1.000	859	20	1.39E-77	NP_491892.1	84	293.893	186	157	hypothetical protein F57B10.5

Locus_20630_Transcript_1/1_Conf_1.000	359	20	4.89E-17	XP_002633888.1	74	90.8929	66	49	Hypothetical protein CBG19950
Locus_20631_Transcript_1/1_Conf_1.000	293	20	7.64E-18	EFO13202.1	72	93.5893	93	67	hypothetical protein LOAG_15329
Locus_20632_Transcript_1/1_Conf_1.000	202	20	1.33E-30	XP_002922306.1	100	135.961	66	66	PREDICTED: triosephosphate isomerase-like, partial
Locus_20633_Transcript_1/1_Conf_1.000	179	0							
Locus_20634_Transcript_1/1_Conf_1.000	232	0							
Locus_20635_Transcript_1/1_Conf_1.000	184	20	7.23E-16	EFO27126.1	85	87.0409	61	52	hypothetical protein LOAG_01361
Locus_20636_Transcript_1/1_Conf_1.000	207	0							
Locus_20637_Transcript_1/1_Conf_1.000	396	20	1.49E-13	EFO22336.1	60	79.337	86	52	WW domain-containing protein
Locus_20638_Transcript_1/1_Conf_1.000	562	20	1.45E-38	XP_002641968.1	69	162.925	168	117	Hypothetical protein CBG16675
Locus_20639_Transcript_1/1_Conf_1.000	133	0							
Locus_2064_Transcript_1/1_Conf_1.000	597	20	1.12E-29	EFO20527.1	55	133.65	188	105	hypothetical protein LOAG_07962
Locus_20640_Transcript_1/1_Conf_1.000	256	0							
Locus_20641_Transcript_1/1_Conf_1.000	196	20	5.64E-21	XP_002639870.1	78	103.99	65	51	C. briggsae CBR-IRS-2 protein
Locus_20642_Transcript_1/1_Conf_1.000	155	20	1.14E-21	XP_002914618.1	100	106.301	51	51	PREDICTED: LOW QUALITY PROTEIN: eukaryotic translation initiation factor 3 subunit L-like
Locus_20643_Transcript_1/1_Conf_1.000	195	0							
Locus_20644_Transcript_1/1_Conf_1.000	143	0							

Locus_20645_Transcript_1/1_Conf_1.000	168	2	3.89E-06	XP_002639999.1	68	54.6842	45	31	Hypothetical protein CBG10829
Locus_20646_Transcript_1/1_Conf_1.000	152	0							
Locus_20647_Transcript_1/1_Conf_1.000	309	0							
Locus_20648_Transcript_1/1_Conf_1.000	151	0							
Locus_20649_Transcript_1/1_Conf_1.000	383	0							
Locus_2065_Transcript_1/1_Conf_1.000	1585	8	7.75E-45	XP_002635375.1	75	186.422	141	106	Hypothetical protein CBG00762
Locus_20650_Transcript_1/1_Conf_1.000	261	0							
Locus_20651_Transcript_1/1_Conf_1.000	753	0							
Locus_20652_Transcript_1/1_Conf_1.000	330	0							
Locus_20653_Transcript_1/1_Conf_1.000	283	0							
Locus_20654_Transcript_1/1_Conf_1.000	246	0							
Locus_20655_Transcript_1/1_Conf_1.000	302	20	1.05E-35	NP_001040798.1	87	152.91	100	87	PhosphoDiEsterase family member (pde-4)
Locus_20656_Transcript_1/1_Conf_1.000	408	0							
Locus_20657_Transcript_1/1_Conf_1.000	268	20	1.24E-36	NP_491915.2	89	155.992	89	80	hypothetical protein ZC581.9
Locus_20658_Transcript_1/1_Conf_1.000	121	0							
Locus_20659_Transcript_1/1_Conf_1.000	588	20	4.19E-34	AAK62032.1	61	148.288	206	127	metalloprotease 1 precursor
Locus_2066_Transcript_1/1_Conf_1.000	2578	0							
Locus_20660_Transcript_1/1_Conf_1.000	151	0							
Locus_20661_Transcript_1/1_Conf_1.000	471	20	3.26E-45	NP_508877.1	80	184.496	124	100	hypothetical protein T03G11.4
Locus_20662_Transcript_1/1_Conf_1.000	167	0							

Locus_20663_Transcript_1/1_Conf_1.000	221	6	9.78E-05	XP_001700925.1	65	50.0618	52	34	flagellar associated protein
Locus_20664_Transcript_1/1_Conf_1.000	204	5	3.40E-10	CAA10033.1	71	68.1662	66	47	DYS-1 protein
Locus_20665_Transcript_1/1_Conf_1.000	264	0							
Locus_20666_Transcript_1/1_Conf_1.000	150	0							
Locus_20667_Transcript_1/1_Conf_1.000	150	0							
Locus_20668_Transcript_1/1_Conf_1.000	208	20	7.53E-18	XP_002629821.1	77	93.5893	67	52	C. briggsae CBR-SQV-8 protein
Locus_20669_Transcript_1/1_Conf_1.000	154	0							
Locus_2067_Transcript_1/1_Conf_1.000	533	0							
Locus_20670_Transcript_1/2_Conf_1.000	631	2	1.37E-07	NP_496506.1	60	60.4622	151	91	hypothetical protein F13D12.8
Locus_20670_Transcript_2/2_Conf_1.000	367	0							
Locus_20671_Transcript_1/1_Conf_1.000	549	20	7.98E-79	CAH04706.3	79	296.59	183	146	C. elegans protein K04H4.2c, partially confirmed by transcript evidence
Locus_20672_Transcript_1/1_Conf_1.000	629	4	3.33E-06	NP_001022632.1	48	55.8398	174	85	Gamma-tubulin Interacting Protein family member (gip-1)

Locus_20673_Transcript_1/1_Conf_1.000	430	20	9.19E-32	NP_001021301.1	87	139.813	88	77	hypothetical protein C06A6.4
Locus_20674_Transcript_1/1_Conf_1.000	150	0							
Locus_20675_Transcript_1/1_Conf_1.000	195	0							
Locus_20676_Transcript_1/1_Conf_1.000	217	20	2.02E-23	XP_002632923.1	84	112.079	71	60	C. briggsae CBR-HIM-6 protein
Locus_20677_Transcript_1/1_Conf_1.000	168	0							
Locus_20678_Transcript_1/1_Conf_1.000	195	1	6.27E-04	XP_001512638.1	63	47.3654	60	38	PREDICTED: similar to phospholipase A2 receptor 1
Locus_20679_Transcript_1/1_Conf_1.000	218	20	2.17E-17	CAX65056.1	74	92.0485	67	50	C. elegans protein F25C8.3d, partially confirmed by transcript evidence
Locus_2068_Transcript_1/1_Conf_1.000	1176	20	1.50E-145	CAR63540.1	85	520.39	355	305	putative Uncharacterized GTP-binding protein
Locus_20680_Transcript_1/1_Conf_1.000	233	20	1.21E-23	NP_497616.1	88	112.849	76	67	hypothetical protein Y71H2AM.6
Locus_20681_Transcript_1/1_Conf_1.000	284	20	1.19E-18	XP_002083879.1	67	96.2857	90	61	GD13963
Locus_20682_Transcript_1/1_Conf_1.000	145	20	1.51E-13	NP_493616.1	89	79.337	47	42	hypothetical protein F33H2.5
Locus_20683_Transcript_1/1_Conf_1.000	131	0							
Locus_20684_Transcript_1/1_Conf_1.000	246	0							
Locus_20685_Transcript_1/1_Conf_1.000	208	0							
Locus_20686_Transcript_1/1_Conf_1.000	669	0							
Locus_20687_Transcript_1/1_Conf_1.000	165	0							
Locus_20688_Transcript_1/1_Conf_1.000	157	1	3.33E-05	XP_001895392.1	66	51.6026	51	34	hypothetical protein Bm1_19685

Locus_20689_Transcript_1/1_Conf_1.000	152	0							
Locus_2069_Transcript_1/1_Conf_1.000	462	20	2.81E-20	EFO21743.1	68	101.679	88	60	hypothetical protein LOAG_06744
Locus_20690_Transcript_1/1_Conf_1.000	268	20	6.63E-22	EFO20564.1	76	107.071	81	62	hypothetical protein LOAG_07924
Locus_20691_Transcript_1/1_Conf_1.000	327	7	2.88E-09	NP_498217.2	82	65.0846	46	38	abnormal embryonic PARtitioning of cytoplasm family member (par-3)
Locus_20692_Transcript_1/1_Conf_1.000	187	0							
Locus_20693_Transcript_1/1_Conf_1.000	253	20	2.32E-22	XP_002630114.1	77	108.612	84	65	C. briggsae CBR-PYR-1 protein
Locus_20694_Transcript_1/1_Conf_1.000	1111	0							
Locus_20695_Transcript_1/1_Conf_1.000	334	4	4.40E-18	NP_495032.1	68	94.3597	89	61	hypothetical protein F07F6.1
Locus_20696_Transcript_1/1_Conf_1.000	239	0							
Locus_20697_Transcript_1/1_Conf_1.000	135	9	1.08E-16	XP_002811006.1	100	89.7373	45	45	PREDICTED: LOW QUALITY PROTEIN: nuclease-sensitive element-binding protein 1-like
Locus_20698_Transcript_1/1_Conf_1.000	230	0							
Locus_20699_Transcript_1/1_Conf_1.000	272	0							
Locus_207_Transcript_10/20_Conf_1.000	183	0							
Locus_207_Transcript_11/20_Conf_0.125	786	19	4.57E-13	NP_508612.1	57	79.337	157	90	VITellogenin structural genes (yolk protein genes) family member (vit-4)
Locus_207_Transcript_12/20_Conf_0.125	786	19	2.05E-13	XP_002644391.1	56	80.4925	157	88	C. briggsae CBR-VIT-5 protein
Locus_207_Transcript_13/20_Conf_0.179	429	14	1.20E-15	NP_508612.1	67	86.2705	91	61	VITellogenin structural genes (yolk protein genes) family member (vit-4)
Locus_207_Transcript_14/20_Conf_0.054	151	0							
Locus_207_Transcript_15/20_Conf_0.161	821	14	3.17E-28	NP_508612.1	65	129.798	146	96	VITellogenin structural genes (yolk protein genes) family member (vit-4)

Locus_207_Transcript_16/20_Conf_0.161	863	19	1.62E-33	NP_508612.1	67	147.517	160	108	VITellogenin structural genes (yolk protein genes) family member (vit-4)
Locus_207_Transcript_17/20_Conf_0.196	678	19	3.14E-35	NP_508612.1	63	152.525	224	142	VITellogenin structural genes (yolk protein genes) family member (vit-4)
Locus_207_Transcript_18/20_Conf_0.107	228	5	7.41E-05	NP_509305.1	53	50.447	71	38	VITellogenin structural genes (yolk protein genes) family member (vit-1)
Locus_207_Transcript_19/20_Conf_0.107	228	14	1.22E-07	NP_001123117.1	60	59.6918	73	44	VITellogenin structural genes (yolk protein genes) family member (vit-2)
Locus_207_Transcript_20/20_Conf_0.196	636	14	2.63E-30	NP_508612.1	62	135.961	210	131	VITellogenin structural genes (yolk protein genes) family member (vit-4)
Locus_207_Transcript_7/20_Conf_1.000	183	0							
Locus_207_Transcript_8/20_Conf_1.000	229	0							
Locus_207_Transcript_9/20_Conf_1.000	229	0							
Locus_2070_Transcript_1/1_Conf_1.000	538	20	2.09E-65	XP_001894445.1	80	251.906	177	143	amine oxidase, flavin-containing family protein
Locus_20700_Transcript_1/1_Conf_1.000	258	0							
Locus_20701_Transcript_1/1_Conf_1.000	347	20	1.75E-46	NP_490776.2	93	188.734	103	96	hypothetical protein Y18H1A.9
Locus_20702_Transcript_1/1_Conf_1.000	546	0							
Locus_20703_Transcript_1/1_Conf_1.000	364	2	1.46E-05	NP_499886.1	47	52.7582	107	51	hypothetical protein R02D3.7
Locus_20704_Transcript_1/1_Conf_1.000	152	0							
Locus_20705_Transcript_1/2_Conf_1.000	472	0							
Locus_20705_Transcript_2/2_Conf_1.000	479	0							
Locus_20706_Transcript_1/1_Conf_1.000	409	20	3.14E-40	NP_495209.1	73	167.933	142	104	hypothetical protein R12C12.1
Locus_20707_Transcript_1/1_Conf_1.000	275	0							
Locus_20708_Transcript_1/1_Conf_1.000	129	0							
Locus_20709_Transcript_1/1_Conf_1.000	678	20	4.46E-98	XP_002640633.1	89	361.303	225	201	C. briggsae CBR-CATP-1 protein
Locus_2071_Transcript_1/2_Conf_1.000	1110	20	3.35E-91	XP_002634069.1	67	339.732	337	229	Hypothetical protein CBG01611
Locus_2071_Transcript_2/2_Conf_1.000	1103	20	1.10E-86	XP_002634069.1	70	324.709	298	209	Hypothetical protein CBG01611
Locus_20710_Transcript_1/1_Conf_1.000	204	20	3.87E-22	XP_002647042.1	88	107.842	61	54	Hypothetical protein CBG03560
Locus_20711_Transcript_1/1_Conf_1.000	416	1	8.56E-06	EFO19153.1	57	53.5286	107	61	hypothetical protein LOAG_09341
Locus_20712_Transcript_1/1_Conf_1.000	482	0							

Locus_20713_Transcript_1/1_Conf_1.000	772	0							
Locus_20714_Transcript_1/1_Conf_1.000	187	0							
Locus_20715_Transcript_1/1_Conf_1.000	227	0							
Locus_20716_Transcript_1/1_Conf_1.000	222	0							
Locus_20717_Transcript_1/1_Conf_1.000	333	0							
Locus_20718_Transcript_1/1_Conf_1.000	598	0							
Locus_20719_Transcript_1/1_Conf_1.000	144	0							
Locus_2072_Transcript_1/1_Conf_1.000	1126	20	1.02E-119	XP_002643477.1	84	434.491	300	252	Hypothetical protein CBG16139
Locus_20720_Transcript_1/1_Conf_1.000	788	17	3.62E-127	XP_002633468.1	93	458.373	261	244	C. briggsae CBR-LIN-24 protein
Locus_20721_Transcript_1/1_Conf_1.000	166	0							
Locus_20722_Transcript_1/1_Conf_1.000	439	20	1.82E-48	XP_002637101.1	75	195.282	144	109	Hypothetical protein CBG09600
Locus_20723_Transcript_1/1_Conf_1.000	133	0							
Locus_20724_Transcript_1/1_Conf_1.000	187	0							
Locus_20725_Transcript_1/1_Conf_1.000	231	2	1.82E-11	NP_498172.3	67	72.4034	85	57	hypothetical protein R02F2.2
Locus_20726_Transcript_1/1_Conf_1.000	196	2	5.67E-05	NP_495410.4	61	50.8322	62	38	hypothetical protein C44B7.11
Locus_20727_Transcript_1/1_Conf_1.000	246	20	2.24E-38	NP_497027.1	100	161.77	81	81	Masculinisation Of Germline family member (mog-4)
Locus_20728_Transcript_1/1_Conf_1.000	223	0							
Locus_20729_Transcript_1/2_Conf_1.000	444	0							
Locus_20729_Transcript_2/2_Conf_1.000	327	0							
Locus_2073_Transcript_1/1_Conf_1.000	3304	20	0	NP_498468.2	89	1436.4	930	836	yeast ISW (imitation SWI) homolog family member (isw-1)
Locus_20730_Transcript_1/1_Conf_1.000	186	0							
Locus_20731_Transcript_1/1_Conf_1.000	191	0							
Locus_20732_Transcript_1/1_Conf_1.000	340	4	4.68E-12	ACI49172.1	61	74.3294	112	69	hypothetical protein Csp3_JD01.004
Locus_20733_Transcript_1/2_Conf_1.000	386	0							
Locus_20733_Transcript_2/2_Conf_1.000	386	0							
Locus_20734_Transcript_1/1_Conf_1.000	327	0							
Locus_20735_Transcript_1/1_Conf_1.000	565	20	4.91E-26	NP_495451.1	64	121.324	129	83	High Incidence of Males (increased X chromosome loss) family member (him-14)
Locus_20736_Transcript_1/1_Conf_1.000	134	0							
Locus_20737_Transcript_1/1_Conf_1.000	405	0							

Locus_20738_Transcript_1/1_Conf_1.000	134	5	3.87E-06	NP_001024204.1	74	54.6842	43	32	TiTiN family member (ttn-1)
Locus_20739_Transcript_1/1_Conf_1.000	463	20	3.19E-48	EFO22683.1	77	194.512	153	119	STE/STE11/MEKK2 protein kinase
Locus_2074_Transcript_1/1_Conf_1.000	608	20	3.71E-68	NP_498858.2	91	261.536	157	143	hypothetical protein ZK353.1
Locus_20740_Transcript_1/1_Conf_1.000	205	0							
Locus_20741_Transcript_1/1_Conf_1.000	215	0							
Locus_20742_Transcript_1/1_Conf_1.000	189	0							
Locus_20743_Transcript_1/1_Conf_1.000	570	0							
Locus_20744_Transcript_1/1_Conf_1.000	409	20	1.19E-47	EFO19846.1	83	192.586	136	114	cleavage and polyadenylation specificity factor subunit 2
Locus_20745_Transcript_1/1_Conf_1.000	419	16	7.14E-40	XP_002640063.1	90	166.777	121	109	Hypothetical protein CBG12545
Locus_20746_Transcript_1/1_Conf_1.000	379	0							
Locus_20747_Transcript_1/1_Conf_1.000	209	0							
Locus_20748_Transcript_1/1_Conf_1.000	298	20	5.24E-27	XP_002629701.1	84	124.02	99	84	C. briggsae CBR-SEC-5 protein
Locus_20749_Transcript_1/1_Conf_1.000	248	0							
Locus_2075_Transcript_1/1_Conf_1.000	647	0							
Locus_20750_Transcript_1/1_Conf_1.000	236	0							
Locus_20751_Transcript_1/1_Conf_1.000	521	20	5.45E-89	XP_002630203.1	94	330.102	173	164	Hypothetical protein CBG00613
Locus_20752_Transcript_1/1_Conf_1.000	685	20	2.46E-35	NP_001023694.1	56	152.91	223	125	UDP-GlucuronosylTransferase family member (ugt-35)

Locus_20753_Transcript_1/1_Conf_1.000	355	20	5.80E-18	XP_002643945.1	90	93.9745	60	54	C. briggsae CBR-MIG-2 protein
Locus_20754_Transcript_1/1_Conf_1.000	161	0							
Locus_20755_Transcript_1/1_Conf_1.000	150	5	4.22E-16	CAN82657.1	100	87.8113	38	38	hypothetical protein
Locus_20756_Transcript_1/1_Conf_1.000	379	20	3.86E-46	XP_002632193.1	83	187.578	126	105	Hypothetical protein CBG07059
Locus_20757_Transcript_1/1_Conf_1.000	381	20	9.85E-26	NP_509122.1	78	119.783	95	75	hypothetical protein F38B6.4
Locus_20758_Transcript_1/1_Conf_1.000	209	0							
Locus_20759_Transcript_1/1_Conf_1.000	345	20	5.60E-61	XP_002807879.1	100	236.884	115	115	PREDICTED: LOW QUALITY PROTEIN: heterogeneous nuclear ribonucleoprotein L-like
Locus_2076_Transcript_1/1_Conf_1.000	530	5	2.50E-07	EFO26720.1	47	58.9214	184	87	hypothetical protein LOAG_01771
Locus_20760_Transcript_1/1_Conf_1.000	274	5	5.57E-21	NP_509311.1	70	103.99	95	67	DEGeneration of certain neurons family member (deg-1)
Locus_20761_Transcript_1/1_Conf_1.000	454	5	7.53E-18	EFO21092.1	71	93.5893	80	57	hypothetical protein LOAG_07396
Locus_20762_Transcript_1/1_Conf_1.000	970	20	3.65E-88	XP_002639255.1	69	329.331	325	226	C. briggsae CBR-OCRL-1 protein
Locus_20763_Transcript_1/1_Conf_1.000	181	20	1.79E-14	XP_002640378.1	85	82.4185	60	51	Hypothetical protein CBG20286

Locus_20764_Transcript_1/1_Conf_1.000	316	20	8.73E-38	XP_002644918.1	86	159.844	91	79	C. briggsae CBR-NHR-173 protein
Locus_20765_Transcript_1/1_Conf_1.000	423	4	5.65E-13	NP_493601.2	52	77.411	147	77	FERM domain (protein4.1-ezrin-radixin-moesin) family member (frm-1)
Locus_20766_Transcript_1/1_Conf_1.000	573	20	5.48E-52	AAS78778.1	81	207.608	144	117	thioredoxin
Locus_20767_Transcript_1/1_Conf_1.000	179	0							
Locus_20768_Transcript_1/1_Conf_1.000	435	4	6.14E-12	XP_001892264.1	58	73.9442	89	52	hypothetical protein Bm1_03880
Locus_20769_Transcript_1/1_Conf_1.000	244	0							
Locus_2077_Transcript_1/1_Conf_1.000	1141	20	4.66E-104	NP_001041026.1	81	382.489	296	240	hypothetical protein Y41E3.7
Locus_20770_Transcript_1/1_Conf_1.000	149	0							
Locus_20771_Transcript_1/1_Conf_1.000	186	0							
Locus_20772_Transcript_1/1_Conf_1.000	618	20	2.84E-26	EFO23582.1	54	122.479	192	104	hypothetical protein LOAG_04900
Locus_20773_Transcript_1/1_Conf_1.000	187	0							
Locus_20774_Transcript_1/1_Conf_1.000	135	0							
Locus_20775_Transcript_1/1_Conf_1.000	201	20	2.51E-29	XP_002726116.1	100	131.724	64	64	PREDICTED: ribosomal protein L8-like
Locus_20776_Transcript_1/1_Conf_1.000	304	4	4.56E-23	AAR30497.1	78	110.923	88	69	RhoGEF
Locus_20777_Transcript_1/1_Conf_1.000	604	20	1.83E-27	EFA05576.1	77	126.331	93	72	hypothetical protein TcasGA2_TC015765
Locus_20778_Transcript_1/1_Conf_1.000	246	3	4.68E-28	NP_492219.1	86	127.487	80	69	hypothetical protein K04G2.9
Locus_20779_Transcript_1/1_Conf_1.000	263	3	6.06E-07	XP_002647600.1	55	57.3806	83	46	Hypothetical protein CBG06690

Locus_2078_Transcript_1/1_Conf_1.000	3247	20	0	XP_002641511.1	93	1892.09	1065	998	C. briggsae CBR-CHC-1 protein
Locus_20780_Transcript_1/1_Conf_1.000	484	0							
Locus_20781_Transcript_1/1_Conf_1.000	473	20	2.59E-34	CAN99691.2	70	148.288	154	108	C. elegans protein F54B3.1b, partially confirmed by transcript evidence
Locus_20782_Transcript_1/1_Conf_1.000	264	20	1.14E-13	EFO26936.1	81	79.7221	58	47	DIX domain-containing protein
Locus_20783_Transcript_1/1_Conf_1.000	258	0							
Locus_20784_Transcript_1/1_Conf_1.000	238	0							
Locus_20785_Transcript_1/1_Conf_1.000	269	0							
Locus_20786_Transcript_1/1_Conf_1.000	312	20	9.43E-16	CAQ48388.2	86	86.6557	58	50	C. elegans protein C26H9A.1b, confirmed by transcript evidence
Locus_20787_Transcript_1/1_Conf_1.000	673	20	2.05E-63	NP_496845.1	70	246.128	226	159	hypothetical protein Y48E1B.2
Locus_20788_Transcript_1/1_Conf_1.000	259	0							
Locus_20789_Transcript_1/1_Conf_1.000	193	0							
Locus_2079_Transcript_1/5_Conf_0.417	658	20	2.34E-40	NP_491825.2	72	169.474	153	111	hypothetical protein F26B1.2
Locus_2079_Transcript_2/5_Conf_0.667	843	20	4.97E-52	XP_002640100.1	71	153.68	147	105	Hypothetical protein CBG12588
Locus_2079_Transcript_3/5_Conf_0.167	308	11	3.26E-13	NP_871833.1	72	78.1814	72	52	hypothetical protein F26B1.2
Locus_2079_Transcript_4/5_Conf_0.250	397	20	4.06E-19	XP_002640100.1	80	97.8265	78	63	Hypothetical protein CBG12588

Locus_2079_Transcript_5/5_Conf_0.500	713	20	2.74E-40	NP_491825.2	72	169.474	153	111	hypothetical protein F26B1.2
Locus_20790_Transcript_1/1_Conf_1.000	252	0							
Locus_20791_Transcript_1/1_Conf_1.000	351	0							
Locus_20792_Transcript_1/1_Conf_1.000	271	12	1.52E-26	ACI49247.1	78	122.479	89	70	hypothetical protein Csp3_JD06.013
Locus_20793_Transcript_1/1_Conf_1.000	675	3	9.61E-61	CAR63560.1	79	237.269	188	149	hypothetical protein
Locus_20794_Transcript_1/1_Conf_1.000	306	20	7.25E-21	XP_001629579.1	73	103.605	86	63	predicted protein
Locus_20795_Transcript_1/1_Conf_1.000	200	5	1.21E-15	NP_495375.3	74	86.2705	63	47	hypothetical protein T14B4.1
Locus_20796_Transcript_1/1_Conf_1.000	186	0							
Locus_20797_Transcript_1/1_Conf_1.000	252	20	1.00E-33	XP_002640129.1	93	146.362	83	78	C. briggsae CBR-SNT-4 protein
Locus_20798_Transcript_1/1_Conf_1.000	291	3	5.14E-06	NP_001122477.1	70	54.299	50	35	FERtilization defective (abnormal sperm) family member (fer-1)
Locus_20799_Transcript_1/1_Conf_1.000	139	0							
Locus_208_Transcript_1/1_Conf_1.000	455	20	1.07E-72	NP_508537.1	100	275.789	151	151	SPeCtrin family member (spc-1)
Locus_2080_Transcript_1/1_Conf_1.000	697	20	3.24E-30	XP_002646089.1	73	135.961	116	85	C. briggsae CBR-RSR-1 protein
Locus_20800_Transcript_1/1_Conf_1.000	136	4	5.21E-11	NP_498923.1	84	70.8626	44	37	hypothetical protein B0303.15
Locus_20801_Transcript_1/1_Conf_1.000	426	3	1.09E-32	XP_002631054.1	68	142.895	150	102	C. briggsae CBR-BTF-1 protein
Locus_20802_Transcript_1/1_Conf_1.000	416	20	5.89E-31	EFO16181.1	83	137.117	101	84	hypothetical protein LOAG_12327
Locus_20803_Transcript_1/1_Conf_1.000	478	20	2.11E-12	YP_003357881.1	52	75.485	154	81	DNA repair and recombination protein RadB
Locus_20804_Transcript_1/1_Conf_1.000	744	6	4.85E-30	XP_001897043.1	54	135.576	263	143	Dedicator of cytokinesis family protein
Locus_20805_Transcript_1/1_Conf_1.000	279	20	1.32E-22	NP_504644.1	71	109.383	89	64	ACyLtransferase-like family member (acl-9)

Locus_20806_Transcript_1/1_Conf_1.000	180	20	1.12E-24	EFO22809.1	96	116.316	60	58	hypothetical protein LOAG_05677
Locus_20807_Transcript_1/1_Conf_1.000	133	0							
Locus_20808_Transcript_1/1_Conf_1.000	394	20	4.22E-16	XP_002647917.1	61	87.8113	104	64	Hypothetical protein CBG23791
Locus_20809_Transcript_1/1_Conf_1.000	432	20	1.08E-24	NP_497756.2	65	116.316	126	82	hypothetical protein F34D10.2
Locus_2081_Transcript_1/1_Conf_1.000	804	0							
Locus_20810_Transcript_1/1_Conf_1.000	166	20	1.29E-25	BAG63585.1	100	119.398	55	55	unnamed protein product
Locus_20811_Transcript_1/1_Conf_1.000	348	0							
Locus_20812_Transcript_1/1_Conf_1.000	355	0							
Locus_20813_Transcript_1/1_Conf_1.000	218	0							
Locus_20814_Transcript_1/1_Conf_1.000	889	20	8.57E-86	XP_002638772.1	69	321.242	292	203	C. briggsae CBR-PTR-21 protein
Locus_20815_Transcript_1/1_Conf_1.000	194	20	1.89E-08	XP_001902614.1	78	62.3882	46	36	Elongation factor Tu GTP binding domain containing protein
Locus_20816_Transcript_1/1_Conf_1.000	130	9	5.79E-10	EFO27462.1	89	67.3958	37	33	hypothetical protein LOAG_01021
Locus_20817_Transcript_1/1_Conf_1.000	166	20	3.08E-11	ACI49172.1	70	71.633	54	38	hypothetical protein Csp3_JD01.004
Locus_20818_Transcript_1/1_Conf_1.000	447	0							
Locus_20819_Transcript_1/1_Conf_1.000	276	20	2.49E-21	XP_002631303.1	92	105.145	65	60	C. briggsae CBR-CLH-1 protein
Locus_2082_Transcript_1/2_Conf_1.000	831	0							

Locus_2082_Transcript_2/2_Conf_1.000	780	0								
Locus_20820_Transcript_1/1_Conf_1.000	155	0								
Locus_20821_Transcript_1/1_Conf_1.000	234	20	2.98E-14	XP_002633412.1	73	81.6481	73	54	Hypothetical protein CBG06177
Locus_20822_Transcript_1/1_Conf_1.000	178	0								
Locus_20823_Transcript_1/1_Conf_1.000	283	20	4.83E-28	EFO28229.1	77	127.487	94	73	hypothetical protein LOAG_00257
Locus_20824_Transcript_1/1_Conf_1.000	188	2	3.94E-30	BAC11651.1	98	134.42	60	59	unnamed protein product
Locus_20825_Transcript_1/1_Conf_1.000	132	0								
Locus_20826_Transcript_1/1_Conf_1.000	643	20	4.51E-102	A8X3A7.2	91	374.4	214	196	Integrin alpha pat-2
Locus_20827_Transcript_1/1_Conf_1.000	241	20	1.57E-23	XP_002643333.1	82	112.464	68	56	C. briggsae CBR-VAP-1 protein
Locus_20828_Transcript_1/1_Conf_1.000	131	0								
Locus_20829_Transcript_1/1_Conf_1.000	285	20	2.31E-30	NP_502582.2	81	135.191	95	77	hypothetical protein Y62E10A.13
Locus_2083_Transcript_1/3_Conf_0.714	1199	20	4.84E-123	XP_002635314.1	77	445.662	361	278	Hypothetical protein CBG01477

Locus_2083_Transcript_2/3_Conf_0.571	691	20	5.18E-73	XP_002635314.1	76	278.1	224	172	Hypothetical protein CBG01477
Locus_2083_Transcript_3/3_Conf_0.714	1199	20	4.84E-123	XP_002635314.1	77	445.662	361	278	Hypothetical protein CBG01477
Locus_20830_Transcript_1/1_Conf_1.000	230	2	1.18E-10	NP_740794.1	67	69.707	76	51	hypothetical protein Y51F10.10
Locus_20831_Transcript_1/1_Conf_1.000	187	0							
Locus_20832_Transcript_1/1_Conf_1.000	407	2	4.30E-05	XP_001898007.1	67	51.2174	53	36	Origin recognition complex subunit 5
Locus_20833_Transcript_1/1_Conf_1.000	185	0							
Locus_20834_Transcript_1/1_Conf_1.000	366	0							
Locus_20835_Transcript_1/1_Conf_1.000	152	0							
Locus_20836_Transcript_1/1_Conf_1.000	1263	20	0	EFO25412.1	95	653.67	378	360	hypothetical protein LOAG_03069
Locus_20837_Transcript_1/1_Conf_1.000	140	20	3.59E-12	NP_502549.2	85	74.7146	47	40	TransThyretin-Related family domain family member (ttr-51)
Locus_20838_Transcript_1/1_Conf_1.000	323	0							
Locus_20839_Transcript_1/1_Conf_1.000	427	20	5.95E-47	XP_002644986.1	87	190.274	113	99	C. briggsae CBR-TAG-320 protein
Locus_2084_Transcript_1/1_Conf_1.000	3760	20	0	NP_495221.1	60	741.88	1073	650	hypothetical protein F21H12.6
Locus_20840_Transcript_1/1_Conf_1.000	131	0							
Locus_20841_Transcript_1/1_Conf_1.000	165	0							
Locus_20842_Transcript_1/1_Conf_1.000	294	0							

Locus_20843_Transcript_1/1_Conf_1.000	202	5	1.58E-07	XP_002637497.1	68	59.3066	54	37	Hypothetical protein CBG19218
Locus_20844_Transcript_1/1_Conf_1.000	131	0							
Locus_20845_Transcript_1/1_Conf_1.000	204	4	6.39E-17	XP_002647777.1	85	90.5077	60	51	C. briggsae CBR-TSP-13 protein
Locus_20846_Transcript_1/1_Conf_1.000	455	0							
Locus_20847_Transcript_1/1_Conf_1.000	133	20	1.98E-10	NP_001129928.1	90	68.9366	43	39	CHoline Transporter-Like family member (chtl-1)
Locus_20848_Transcript_1/1_Conf_1.000	166	0							
Locus_20849_Transcript_1/1_Conf_1.000	142	0							
Locus_2085_Transcript_1/1_Conf_1.000	364	20	6.08E-44	NP_496492.1	86	180.259	121	105	hypothetical protein C47D12.2
Locus_20850_Transcript_1/1_Conf_1.000	268	0							
Locus_20851_Transcript_1/1_Conf_1.000	505	4	5.64E-16	NP_496906.2	55	87.4261	127	70	hypothetical protein Y51H1A.2
Locus_20852_Transcript_1/1_Conf_1.000	172	20	1.12E-21	NP_498528.1	94	106.301	53	50	Pim (mammalian oncogene) Related Kinase family member (prk-1)
Locus_20853_Transcript_1/1_Conf_1.000	239	2	1.69E-09	EFO24512.1	63	65.855	76	48	hypothetical protein LOAG_03972
Locus_20854_Transcript_1/1_Conf_1.000	346	2	4.35E-13	NP_506285.1	61	77.7962	111	68	hypothetical protein F57B1.5
Locus_20855_Transcript_1/1_Conf_1.000	287	0							
Locus_20856_Transcript_1/1_Conf_1.000	144	0							
Locus_20857_Transcript_1/1_Conf_1.000	206	20	3.07E-19	NP_500987.3	85	98.2117	68	58	hypothetical protein Y73B6BL.1
Locus_20858_Transcript_1/1_Conf_1.000	136	0							
Locus_20859_Transcript_1/1_Conf_1.000	173	0							
Locus_2086_Transcript_1/1_Conf_1.000	986	20	2.91E-40	NP_001171768.1	70	170.244	180	126	hypothetical protein LOC100376325
Locus_20860_Transcript_1/1_Conf_1.000	297	5	4.28E-37	XP_002643254.1	88	157.532	98	87	Hypothetical protein CBG08123
Locus_20861_Transcript_1/1_Conf_1.000	183	0							
Locus_20862_Transcript_1/1_Conf_1.000	236	2	3.41E-10	XP_002631009.1	63	68.1662	80	51	Hypothetical protein CBG02761

Locus_20863_Transcript_1/1_Conf_1.000	412	20	5.55E-21	XP_002636677.1	60	103.99	133	81	Hypothetical protein CBG23391
Locus_20864_Transcript_1/1_Conf_1.000	240	20	1.05E-27	XP_002639713.1	88	126.331	79	70	C. briggsae CBR-RAD-54 protein
Locus_20865_Transcript_1/1_Conf_1.000	144	0							
Locus_20866_Transcript_1/1_Conf_1.000	264	0							
Locus_20867_Transcript_1/1_Conf_1.000	188	0							
Locus_20868_Transcript_1/1_Conf_1.000	188	0							
Locus_20869_Transcript_1/1_Conf_1.000	277	2	1.85E-16	XP_002638587.1	67	88.9669	91	61	Hypothetical protein CBG05635
Locus_2087_Transcript_1/1_Conf_1.000	1131	20	2.61E-131	NP_001021405.1	90	473.011	292	263	asparagiNyl tRNA Synthetase family member (nrs-1)
Locus_20870_Transcript_1/2_Conf_1.000	519	20	2.26E-18	NP_001022032.1	55	95.5153	173	96	TRRAP-like (transcription/transformation domain-associated protein) family member (trr-1)
Locus_20870_Transcript_2/2_Conf_1.000	519	20	1.01E-18	EFO19589.1	50	96.6709	170	86	hypothetical protein LOAG_08898
Locus_20871_Transcript_1/1_Conf_1.000	151	0							
Locus_20872_Transcript_1/1_Conf_1.000	249	0							
Locus_20873_Transcript_1/1_Conf_1.000	214	0							
Locus_20874_Transcript_1/1_Conf_1.000	572	20	9.31E-12	EFO20177.1	55	73.9442	107	59	hypothetical protein LOAG_08313
Locus_20875_Transcript_1/1_Conf_1.000	414	20	1.07E-32	XP_002645073.1	90	142.895	71	64	C. briggsae CBR-ADT-2 protein
Locus_20876_Transcript_1/1_Conf_1.000	154	0							

Locus_20877_Transcript_1/1_Conf_1.000	313	0							
Locus_20878_Transcript_1/1_Conf_1.000	154	0							
Locus_20879_Transcript_1/1_Conf_1.000	290	6	2.15E-28	XP_002642067.1	94	128.642	67	63	Hypothetical protein CBG18006
Locus_2088_Transcript_1/2_Conf_1.000	2677	20	4.73E-35	XP_684510.2	43	154.836	718	313	PREDICTED: im:7150991
Locus_2088_Transcript_2/2_Conf_1.000	2317	20	4.00E-35	XP_684510.2	43	154.836	718	313	PREDICTED: im:7150991
Locus_20880_Transcript_1/1_Conf_1.000	284	0							
Locus_20881_Transcript_1/1_Conf_1.000	155	20	4.81E-12	XP_001893740.1	88	74.3294	45	40	ATP-dependent helicase DDX1
Locus_20882_Transcript_1/1_Conf_1.000	280	0							
Locus_20883_Transcript_1/1_Conf_1.000	425	5	1.59E-07	EFO27260.1	80	59.3066	41	33	RNA recognition domain-containing protein domain-containing protein
Locus_20884_Transcript_1/1_Conf_1.000	194	20	1.35E-22	P27442.1	90	109.383	64	58	GMP reductase
Locus_20885_Transcript_1/1_Conf_1.000	1807	20	6.28E-86	NP_495931.2	52	323.168	642	336	hypothetical protein T24B8.7
Locus_20886_Transcript_1/1_Conf_1.000	333	20	3.35E-42	ACY39994.1	92	174.481	95	88	C. elegans FLN-2 protein, isoform d
Locus_20887_Transcript_1/1_Conf_1.000	241	0							
Locus_20888_Transcript_1/1_Conf_1.000	175	5	1.51E-10	EFO21331.1	72	69.3218	58	42	expressed sequence
Locus_20889_Transcript_1/1_Conf_1.000	139	0							
Locus_2089_Transcript_1/4_Conf_0.667	842	9	8.79E-21	NP_510235.2	46	105.145	281	132	hypothetical protein C29F7.2
Locus_2089_Transcript_2/4_Conf_0.222	394	4	1.15E-13	NP_510234.1	54	79.7221	135	73	hypothetical protein C29F7.1
Locus_2089_Transcript_3/4_Conf_0.667	860	10	1.41E-21	XP_002645614.1	49	107.842	278	138	Hypothetical protein CBG05337

Locus_2089_Transcript_4/4_Conf_0.667	827	12	1.71E-21	XP_002645614.1	49	107.457	278	138	Hypothetical protein CBG05337
Locus_20890_Transcript_1/1_Conf_1.000	293	0							
Locus_20891_Transcript_1/1_Conf_1.000	139	1	1.12E-05	EDL24903.1	78	53.1434	37	29	mCG14133
Locus_20892_Transcript_1/1_Conf_1.000	198	0							
Locus_20893_Transcript_1/1_Conf_1.000	368	0							
Locus_20894_Transcript_1/1_Conf_1.000	381	20	5.21E-35	NP_499648.3	71	150.599	127	91	ELongator complex Protein Component family member (elpc-2)
Locus_20895_Transcript_1/1_Conf_1.000	719	0							
Locus_20896_Transcript_1/1_Conf_1.000	251	20	2.32E-11	EFO20608.1	62	72.0182	83	52	ran-binding protein 7
Locus_20897_Transcript_1/1_Conf_1.000	221	0							
Locus_20898_Transcript_1/1_Conf_1.000	147	0							
Locus_20899_Transcript_1/1_Conf_1.000	213	10	1.50E-34	NP_872018.1	100	149.058	70	70	Anion/Bicarbonate TranSporter family member (abts-3)
Locus_209_Transcript_1/1_Conf_1.000	1555	20	0	NP_490816.4	88	669.848	432	382	related to yeast Vacuolar Protein Sorting factor family member (vps-4)

Locus_2090_Transcript_1/1_Conf_1.000	583	5	7.81E-33	NP_509903.1	72	144.05	179	129	TweeTY transmembrane/cell surface protein Homolog family member (ttyh-1)
Locus_20900_Transcript_1/1_Conf_1.000	141	0							
Locus_20901_Transcript_1/1_Conf_1.000	789	0							
Locus_20902_Transcript_1/1_Conf_1.000	247	0							
Locus_20903_Transcript_1/1_Conf_1.000	181	20	2.66E-18	BAG61688.1	100	95.1301	46	46	unnamed protein product
Locus_20904_Transcript_1/1_Conf_1.000	422	0							
Locus_20905_Transcript_1/1_Conf_1.000	161	0							
Locus_20906_Transcript_1/1_Conf_1.000	131	0							
Locus_20907_Transcript_1/1_Conf_1.000	173	3	5.77E-10	XP_002639131.1	72	67.3958	58	42	Hypothetical protein CBG14951
Locus_20908_Transcript_1/1_Conf_1.000	458	20	6.93E-56	NP_508379.1	82	219.935	151	125	ACyLtransferase-like family member (acl-4)
Locus_20909_Transcript_1/1_Conf_1.000	305	0							
Locus_2091_Transcript_1/1_Conf_1.000	647	2	1.12E-07	XP_002634773.1	70	60.8474	57	40	Hypothetical protein CBG13872
Locus_20910_Transcript_1/1_Conf_1.000	155	20	6.29E-12	XP_002637345.1	82	73.9442	47	39	C. briggsae CBR-UNC-68 protein
Locus_20911_Transcript_1/1_Conf_1.000	505	20	2.69E-34	NP_504148.1	61	148.288	138	85	PHAryngeal gland Toxin-related family member (phat-5)
Locus_20912_Transcript_1/1_Conf_1.000	132	0							
Locus_20913_Transcript_1/1_Conf_1.000	205	0							
Locus_20914_Transcript_1/1_Conf_1.000	263	8	3.32E-13	XP_002638527.1	72	78.1814	87	63	C. briggsae CBR-UNC-80 protein
Locus_20915_Transcript_1/1_Conf_1.000	217	0							
Locus_20916_Transcript_1/1_Conf_1.000	136	0							
Locus_20917_Transcript_1/1_Conf_1.000	143	0							
Locus_20918_Transcript_1/1_Conf_1.000	313	0							
Locus_20919_Transcript_1/1_Conf_1.000	136	0							
Locus_2092_Transcript_1/1_Conf_1.000	767	20	2.28E-30	XP_002641311.1	94	136.732	94	89	C. briggsae CBR-UNC-69 protein
Locus_20920_Transcript_1/1_Conf_1.000	168	0							
Locus_20921_Transcript_1/1_Conf_1.000	328	0							
Locus_20922_Transcript_1/1_Conf_1.000	197	0							
Locus_20923_Transcript_1/1_Conf_1.000	374	0							
Locus_20924_Transcript_1/1_Conf_1.000	310	0							

Locus_20925_Transcript_1/1_Conf_1.000	372	20	2.99E-38	NP_502432.1	77	161.384	124	96	RBD (RNA binding domain) protein family member (rbd-1)
Locus_20926_Transcript_1/1_Conf_1.000	290	0							
Locus_20927_Transcript_1/1_Conf_1.000	269	0							
Locus_20928_Transcript_1/1_Conf_1.000	178	0							
Locus_20929_Transcript_1/1_Conf_1.000	267	0							
Locus_2093_Transcript_1/2_Conf_1.000	1210	2	2.43E-13	NP_505850.1	88	81.2629	51	45	Prion-like-(Q/N-rich)-domain-bearing protein family member (pqn-70)
Locus_2093_Transcript_2/2_Conf_1.000	1176	2	2.33E-13	NP_505850.1	88	81.2629	51	45	Prion-like-(Q/N-rich)-domain-bearing protein family member (pqn-70)
Locus_20930_Transcript_1/1_Conf_1.000	566	20	2.68E-40	XP_001894156.1	69	168.703	191	132	MutS domain V family protein
Locus_20931_Transcript_1/2_Conf_1.000	232	0							
Locus_20931_Transcript_2/2_Conf_1.000	231	0							
Locus_20932_Transcript_1/1_Conf_1.000	183	0							
Locus_20933_Transcript_1/1_Conf_1.000	322	0							
Locus_20934_Transcript_1/1_Conf_1.000	147	0							
Locus_20935_Transcript_1/1_Conf_1.000	130	2	7.33E-05	XP_001893489.1	70	50.447	41	29	Hepatocellular carcinoma-associated antigen 66
Locus_20936_Transcript_1/1_Conf_1.000	199	20	3.76E-25	NP_509205.2	93	117.857	66	62	P-GlycoProtein related family member (pgp-10)
Locus_20937_Transcript_1/2_Conf_1.000	817	4	2.32E-39	NP_508552.1	62	166.777	202	126	hypothetical protein F40F4.6
Locus_20937_Transcript_2/2_Conf_1.000	817	4	2.32E-39	NP_508552.1	62	166.777	202	126	hypothetical protein F40F4.6
Locus_20938_Transcript_1/1_Conf_1.000	229	0							
Locus_20939_Transcript_1/1_Conf_1.000	310	0							
Locus_2094_Transcript_1/1_Conf_1.000	475	7	3.39E-10	NP_499022.1	46	68.1662	151	70	hypothetical protein ZK512.5
Locus_20940_Transcript_1/1_Conf_1.000	164	0							
Locus_20941_Transcript_1/1_Conf_1.000	291	0							
Locus_20942_Transcript_1/1_Conf_1.000	141	0							
Locus_20943_Transcript_1/1_Conf_1.000	395	0							
Locus_20944_Transcript_1/1_Conf_1.000	228	20	1.44E-16	CBW44372.1	76	89.3521	72	55	C. elegans protein F21A10.2e, partially confirmed by transcript evidence

Locus_20945_Transcript_1/1_Conf_1.000	203	20	5.99E-23	XP_002643622.1	84	110.538	66	56	C. briggsae CBR-NAS-39 protein
Locus_20946_Transcript_1/1_Conf_1.000	202	0							
Locus_20947_Transcript_1/1_Conf_1.000	249	0							
Locus_20948_Transcript_1/1_Conf_1.000	614	3	3.50E-13	NP_497273.2	53	78.9518	166	89	Fatty Acid/Retinol binding protein family member (far-8)
Locus_20949_Transcript_1/1_Conf_1.000	343	2	5.85E-42	AAQ18782.1	80	173.711	115	92	GATA transcription factor
Locus_2095_Transcript_1/1_Conf_1.000	846	20	7.51E-36	NP_506009.1	55	155.221	274	152	hypothetical protein C52E4.7
Locus_20950_Transcript_1/1_Conf_1.000	387	20	1.95E-50	XP_002645256.1	84	201.83	129	109	Hypothetical protein CBG00135
Locus_20951_Transcript_1/1_Conf_1.000	232	0							
Locus_20952_Transcript_1/1_Conf_1.000	157	0							
Locus_20953_Transcript_1/1_Conf_1.000	150	20	8.24E-12	NP_741684.2	87	73.559	48	42	PROximal proliferation in germline family member (pro-3)
Locus_20954_Transcript_1/1_Conf_1.000	141	10	3.58E-12	CAA92456.3	91	74.7146	47	43	C. elegans protein C53B4.4b, partially confirmed by transcript evidence
Locus_20955_Transcript_1/1_Conf_1.000	227	1	2.89E-41	EAW61183.1	100	171.4	75	75	basigin (Ok blood group), isoform CRA_d
Locus_20956_Transcript_1/1_Conf_1.000	207	0							

Locus_20957_Transcript_1/1_Conf_1.000	214	20	2.75E-20	EFO18082.1	94	101.679	70	66	hypothetical protein LOAG_10417
Locus_20958_Transcript_1/1_Conf_1.000	284	0							
Locus_20959_Transcript_1/1_Conf_1.000	646	9	2.22E-56	NP_509677.1	70	222.631	216	153	hypothetical protein F19C6.2
Locus_2096_Transcript_1/1_Conf_1.000	278	0							
Locus_20960_Transcript_1/1_Conf_1.000	646	6	1.70E-48	XP_002636775.1	68	196.438	190	131	Hypothetical protein CBG23509
Locus_20961_Transcript_1/1_Conf_1.000	188	0							
Locus_20962_Transcript_1/1_Conf_1.000	140	0							
Locus_20963_Transcript_1/1_Conf_1.000	246	20	1.61E-12	NP_505977.1	73	75.8702	67	49	PYruvate Carboxylase family member (pyc-1)
Locus_20964_Transcript_1/1_Conf_1.000	137	20	4.87E-09	XP_797921.2	90	64.3142	43	39	PREDICTED: similar to MGC82686 protein
Locus_20965_Transcript_1/1_Conf_1.000	302	20	3.87E-22	NP_491200.1	72	107.842	102	74	hypothetical protein D1037.1
Locus_20966_Transcript_1/1_Conf_1.000	686	0							
Locus_20967_Transcript_1/1_Conf_1.000	191	20	5.49E-24	XP_002632893.1	95	114.005	63	60	Hypothetical protein CBG15101
Locus_20968_Transcript_1/1_Conf_1.000	164	4	3.65E-12	ACJ65180.1	86	74.7146	45	39	Gaba b receptor subunit protein 2, confirmed by transcript evidence

Locus_20969_Transcript_1/1_Conf_1.000	357	20	3.48E-39	EFO23704.1	80	164.466	117	94	hypothetical protein LOAG_04778
Locus_2097_Transcript_1/1_Conf_1.000	2466	20	0	NP_492319.2	84	820.846	590	497	RSK-pNinety (RSK-p90 kinase) homolog family member (rskn-1)
Locus_20970_Transcript_1/1_Conf_1.000	479	0							
Locus_20971_Transcript_1/1_Conf_1.000	191	0							
Locus_20972_Transcript_1/1_Conf_1.000	161	20	8.16E-12	NP_503578.1	84	73.559	45	38	hypothetical protein F22F7.7
Locus_20973_Transcript_1/1_Conf_1.000	743	20	1.13E-63	ACI49160.1	70	247.284	231	162	hypothetical protein Cbre_JD21.007
Locus_20974_Transcript_1/1_Conf_1.000	172	0							
Locus_20975_Transcript_1/1_Conf_1.000	351	0							
Locus_20976_Transcript_1/1_Conf_1.000	133	20	8.32E-17	ACJ65168.1	100	90.1225	44	44	Uncoordinated protein 68, isoform b, partially confirmed by transcript evidence
Locus_20977_Transcript_1/1_Conf_1.000	242	15	2.19E-17	ADI88500.1	71	92.0485	77	55	DRAG-1
Locus_20978_Transcript_1/1_Conf_1.000	153	0							
Locus_20979_Transcript_1/1_Conf_1.000	130	0							

Locus_2098_Transcript_1/1_Conf_1.000	564	20	9.80E-59	NP_492601.1	89	229.95	133	119	Cytochrome C Oxidase family member (cco-1)
Locus_20980_Transcript_1/1_Conf_1.000	157	1	1.65E-04	EFO17639.1	68	49.2914	35	24	hypothetical protein LOAG_10859
Locus_20981_Transcript_1/1_Conf_1.000	276	0							
Locus_20982_Transcript_1/1_Conf_1.000	179	20	6.55E-14	NP_496814.1	80	80.4925	60	48	Member of AAA family binding CED-4 family member (mac-1)
Locus_20983_Transcript_1/1_Conf_1.000	562	0							
Locus_20984_Transcript_1/1_Conf_1.000	134	20	1.47E-13	NP_510705.1	93	79.337	44	41	hypothetical protein C10E2.6
Locus_20985_Transcript_1/1_Conf_1.000	685	20	1.86E-83	XP_002645520.1	85	312.768	206	176	C. briggsae CBR-NHR-14 protein
Locus_20986_Transcript_1/1_Conf_1.000	304	20	1.56E-39	CAX65056.1	92	165.622	101	93	C. elegans protein F25C8.3d, partially confirmed by transcript evidence
Locus_20987_Transcript_1/1_Conf_1.000	225	0							

Locus_20988_Transcript_1/1_Conf_1.000	281	20	6.74E-27	NP_501743.1	77	123.635	92	71	CAVeolin family member (cav-1)
Locus_20989_Transcript_1/1_Conf_1.000	129	1	4.75E-04	XP_002937788.1	69	47.7506	39	27	PREDICTED: collagen alpha-6(VI) chain-like, partial
Locus_2099_Transcript_1/2_Conf_1.000	861	7	1.98E-23	NP_492047.1	55	114.005	139	77	Chondroitin ProteoGlycan family member (cpg-3)
Locus_2099_Transcript_2/2_Conf_1.000	716	7	1.38E-23	NP_492047.1	55	114.005	139	77	Chondroitin ProteoGlycan family member (cpg-3)
Locus_20990_Transcript_1/1_Conf_1.000	319	0							
Locus_20991_Transcript_1/1_Conf_1.000	129	0							
Locus_20992_Transcript_1/1_Conf_1.000	242	0							
Locus_20993_Transcript_1/1_Conf_1.000	128	0							
Locus_20994_Transcript_1/1_Conf_1.000	160	0							
Locus_20995_Transcript_1/2_Conf_1.000	476	0							
Locus_20995_Transcript_2/2_Conf_1.000	464	0							
Locus_20996_Transcript_1/1_Conf_1.000	245	3	2.67E-15	XP_002643880.1	69	85.1149	83	58	Hypothetical protein CBG02118
Locus_20997_Transcript_1/1_Conf_1.000	511	20	7.87E-82	XP_002645216.1	94	306.22	169	159	Hypothetical protein CBG00074
Locus_20998_Transcript_1/1_Conf_1.000	583	20	1.16E-52	NP_741293.1	69	209.92	204	142	Enhanced RNAI (RNA interference) family member (eri-1)

Locus_20999_Transcript_1/1_Conf_1.000	153	20	1.40E-11	EFO25550.1	78	72.7886	50	39	nucleolar protein 10
Locus_21_Transcript_1/1_Conf_1.000	1484	20	5.83E-156	NP_502269.1	96	555.444	350	336	hypothetical protein ZK829.7
Locus_210_Transcript_1/3_Conf_0.375	562	8	7.13E-62	ADN00782.1	80	240.35	175	141	parasitic stage specific protein 1
Locus_210_Transcript_2/3_Conf_0.500	851	20	1.47E-39	XP_002637073.1	56	167.548	207	117	C. briggsae CBR-LYS-1 protein
Locus_210_Transcript_3/3_Conf_0.375	696	8	4.04E-73	ADN00782.1	79	278.485	204	162	parasitic stage specific protein 1
Locus_2100_Transcript_1/9_Conf_0.692	3844	20	0	NP_491069.1	86	1819.67	1231	1059	hypothetical protein Y71F9AL.17
Locus_2100_Transcript_2/9_Conf_0.692	3844	20	0	NP_491069.1	86	1819.67	1231	1059	hypothetical protein Y71F9AL.17

Locus_2100_Transcript_3/9_Conf_0.692	3844	20	0	NP_491069.1	86	1819.67	1231	1059	hypothetical protein Y71F9AL.17
Locus_2100_Transcript_4/9_Conf_0.692	3844	20	0	NP_491069.1	86	1819.67	1231	1059	hypothetical protein Y71F9AL.17
Locus_2100_Transcript_5/9_Conf_0.692	3844	20	0	NP_491069.1	86	1819.67	1231	1059	hypothetical protein Y71F9AL.17

Locus_2100_Transcript_6/9_Conf_0.692	3844	20	0	NP_491069.1	86	1819.67	1231	1059	hypothetical protein Y71F9AL.17
Locus_2100_Transcript_7/9_Conf_0.692	3844	20	0	NP_491069.1	86	1819.67	1231	1059	hypothetical protein Y71F9AL.17
Locus_2100_Transcript_8/9_Conf_0.692	3844	20	0	NP_491069.1	86	1819.67	1231	1059	hypothetical protein Y71F9AL.17

Locus_2100_Transcript_9/9_Conf_0.692	3844	20	0	NP_491069.1	86	1819.67	1231	1059	hypothetical protein Y71F9AL.17
Locus_21000_Transcript_1/1_Conf_1.000	577	20	3.61E-51	NP_499668.1	71	204.912	184	131	hypothetical protein Y37D8A.2
Locus_21001_Transcript_1/1_Conf_1.000	233	0							
Locus_21002_Transcript_1/1_Conf_1.000	175	0							
Locus_21003_Transcript_1/1_Conf_1.000	226	0							
Locus_21004_Transcript_1/1_Conf_1.000	282	4	4.24E-16	NP_500204.2	70	87.8113	94	66	CaDHerin family member (cdh-10)
Locus_21005_Transcript_1/1_Conf_1.000	150	0							
Locus_21006_Transcript_1/1_Conf_1.000	329	20	7.59E-10	XP_002640649.1	60	67.0106	70	42	Hypothetical protein CBG19707
Locus_21007_Transcript_1/1_Conf_1.000	218	0							
Locus_21008_Transcript_1/1_Conf_1.000	489	0							
Locus_21009_Transcript_1/1_Conf_1.000	252	0							
Locus_2101_Transcript_1/2_Conf_1.000	1582	20	8.84E-158	EFO22806.1	77	561.607	441	341	N-myristoyltransferase 2
Locus_2101_Transcript_2/2_Conf_1.000	1582	20	8.84E-158	EFO22806.1	77	561.607	441	341	N-myristoyltransferase 2
Locus_21010_Transcript_1/1_Conf_1.000	191	0							
Locus_21011_Transcript_1/1_Conf_1.000	515	1	5.32E-17	EFO16507.1	56	90.8929	162	91	hypothetical protein LOAG_12000
Locus_21012_Transcript_1/1_Conf_1.000	270	20	7.27E-37	NP_497786.2	95	156.762	89	85	MethylMalonylCoA Mutase homolog family member (mmcm-1)

Locus_21013_Transcript_1/1_Conf_1.000	254	0							
Locus_21014_Transcript_1/1_Conf_1.000	162	10	1.34E-06	ZP_06280939.1	58	56.225	53	31	LPXTG-motif cell wall anchor domain protein
Locus_21015_Transcript_1/1_Conf_1.000	241	20	2.43E-08	XP_002155142.1	75	62.003	48	36	PREDICTED: similar to ezrin-binding partner PACE-1
Locus_21016_Transcript_1/1_Conf_1.000	349	2	1.26E-20	NP_506224.1	59	102.834	118	70	hypothetical protein R90.1
Locus_21017_Transcript_1/1_Conf_1.000	288	1	1.96E-05	XP_002642656.1	74	52.373	35	26	Hypothetical protein CBG00040
Locus_21018_Transcript_1/1_Conf_1.000	236	2	3.66E-04	NP_740912.2	46	48.1358	86	40	SCP(Small C-terminal domain Phosphatase)-Like phosphatase family member (scpl-1)
Locus_21019_Transcript_1/1_Conf_1.000	197	0							
Locus_2102_Transcript_1/1_Conf_1.000	1094	4	6.52E-07	XP_002631720.1	54	59.6918	120	65	C. briggsae CBR-PQN-87 protein
Locus_21020_Transcript_1/1_Conf_1.000	292	0							
Locus_21021_Transcript_1/1_Conf_1.000	467	4	2.78E-52	NP_492775.2	80	207.994	154	124	LAMinin related. See also lmb- family member (lam-3)
Locus_21022_Transcript_1/1_Conf_1.000	331	0							
Locus_21023_Transcript_1/1_Conf_1.000	220	0							
Locus_21024_Transcript_1/1_Conf_1.000	141	0							
Locus_21025_Transcript_1/1_Conf_1.000	134	0							
Locus_21026_Transcript_1/1_Conf_1.000	183	0							
Locus_21027_Transcript_1/1_Conf_1.000	187	4	4.37E-13	NP_491022.2	83	77.7962	61	51	hypothetical protein F32B5.7
Locus_21028_Transcript_1/1_Conf_1.000	159	0							
Locus_21029_Transcript_1/1_Conf_1.000	157	20	2.08E-23	ACV89490.1	100	112.079	52	52	MHC class II antigen
Locus_2103_Transcript_1/1_Conf_1.000	596	20	4.47E-23	EFO22749.1	61	111.694	157	97	hypothetical protein LOAG_05738
Locus_21030_Transcript_1/1_Conf_1.000	246	20	1.12E-21	NP_501999.2	78	106.301	80	63	hypothetical protein T25B9.10
Locus_21031_Transcript_1/1_Conf_1.000	227	10	3.32E-21	XP_002644127.1	77	104.76	75	58	C. briggsae CBR-TAG-289 protein
Locus_21032_Transcript_1/1_Conf_1.000	350	0							
Locus_21033_Transcript_1/1_Conf_1.000	143	11	5.35E-08	NP_496800.1	71	60.8474	45	32	hypothetical protein W09H1.5

Locus_21034_Transcript_1/1_Conf_1.000	154	20	1.00E-17	NP_491165.2	94	93.2041	50	47	human SPG (spastic paraplegia) family member (spg-7)
Locus_21035_Transcript_1/1_Conf_1.000	500	2	2.33E-11	NP_740794.1	47	72.0182	168	79	hypothetical protein Y51F10.10
Locus_21036_Transcript_1/1_Conf_1.000	481	20	5.12E-51	XP_002646593.1	89	203.756	131	117	C. briggsae CBR-PRPF-4 protein
Locus_21037_Transcript_1/1_Conf_1.000	363	0							
Locus_21038_Transcript_1/1_Conf_1.000	156	0							
Locus_21039_Transcript_1/1_Conf_1.000	154	0							
Locus_2104_Transcript_1/1_Conf_1.000	2329	20	0	CAX32485.1	68	697.582	790	539	C. elegans protein T20D3.11b, partially confirmed by transcript evidence
Locus_21040_Transcript_1/1_Conf_1.000	416	20	2.75E-12	XP_001894417.1	59	75.0998	100	59	Nuclear hormone receptor family member nhr-25
Locus_21041_Transcript_1/1_Conf_1.000	197	0							
Locus_21042_Transcript_1/1_Conf_1.000	204	20	2.78E-12	XP_002645018.1	69	75.0998	68	47	C. briggsae CBR-SUR-5 protein
Locus_21043_Transcript_1/1_Conf_1.000	196	0							
Locus_21044_Transcript_1/1_Conf_1.000	263	20	1.87E-32	NP_491529.2	87	142.124	87	76	hypothetical protein Y110A7A.6
Locus_21045_Transcript_1/1_Conf_1.000	129	0							
Locus_21046_Transcript_1/1_Conf_1.000	220	2	6.90E-06	XP_001892872.1	61	50.0618	52	32	hypothetical protein Bm1_07015
Locus_21047_Transcript_1/1_Conf_1.000	447	0							
Locus_21048_Transcript_1/1_Conf_1.000	177	20	5.72E-18	NP_493618.1	87	93.9745	56	49	Deletions Of G-rich DNA family member (dog-1)
Locus_21049_Transcript_1/1_Conf_1.000	213	4	1.61E-28	NP_495365.1	97	129.028	70	68	RaPsYn family member (rpy-1)

Locus_2105_Transcript_1/1_Conf_1.000	1036	20	7.53E-42	XP_002639656.1	58	175.637	239	141	Hypothetical protein CBG12372
Locus_21050_Transcript_1/1_Conf_1.000	192	0							
Locus_21051_Transcript_1/1_Conf_1.000	272	20	2.41E-40	NP_001122445.1	96	168.318	89	86	hypothetical protein F08A10.1
Locus_21052_Transcript_1/1_Conf_1.000	222	0							
Locus_21053_Transcript_1/1_Conf_1.000	210	20	3.59E-28	NP_502747.1	94	127.872	69	65	hypothetical protein F55B11.1
Locus_21054_Transcript_1/1_Conf_1.000	161	0							
Locus_21055_Transcript_1/1_Conf_1.000	318	0							
Locus_21056_Transcript_1/1_Conf_1.000	136	0							
Locus_21057_Transcript_1/1_Conf_1.000	161	2	1.03E-14	EFO27604.1	83	83.1889	53	44	hypothetical protein LOAG_00873
Locus_21058_Transcript_1/1_Conf_1.000	375	20	6.84E-43	XP_001900176.1	77	176.792	135	104	LIM domain containing protein
Locus_21059_Transcript_1/1_Conf_1.000	406	20	2.26E-46	ACZ13335.1	90	188.348	133	120	60S ribosomal protein L5
Locus_2106_Transcript_1/4_Conf_0.667	1634	20	1.57E-173	XP_002637767.1	87	613.994	400	349	C. briggsae CBR-UNC-61 protein
Locus_2106_Transcript_2/4_Conf_0.667	1639	20	6.61E-172	XP_002637767.1	86	608.601	407	351	C. briggsae CBR-UNC-61 protein

Locus_2106_Transcript_3/4_Conf_0.667	1627	20	6.55E-172	XP_002637767.1	86	608.601	403	349	C. briggsae CBR-UNC-61 protein
Locus_2106_Transcript_4/4_Conf_0.667	1618	20	1.55E-173	XP_002637767.1	87	613.994	400	349	C. briggsae CBR-UNC-61 protein
Locus_21060_Transcript_1/1_Conf_1.000	145	0							
Locus_21061_Transcript_1/1_Conf_1.000	402	17	4.15E-32	NP_502894.1	68	140.969	122	84	hypothetical protein Y105C5B.5
Locus_21062_Transcript_1/1_Conf_1.000	155	0							
Locus_21063_Transcript_1/1_Conf_1.000	450	0							
Locus_21064_Transcript_1/1_Conf_1.000	386	15	8.32E-17	NP_509219.1	55	90.1225	138	76	hypothetical protein F46H5.4
Locus_21065_Transcript_1/1_Conf_1.000	268	2	1.06E-11	XP_002637628.1	72	73.1738	65	47	Hypothetical protein CBG19373
Locus_21066_Transcript_1/1_Conf_1.000	138	20	1.90E-21	XP_002800676.1	100	105.531	45	45	PREDICTED: galectin-3-binding protein isoform 3
Locus_21067_Transcript_1/1_Conf_1.000	502	5	1.15E-13	XP_002636935.1	55	79.7221	145	81	C. briggsae CBR-UIG-1 protein
Locus_21068_Transcript_1/1_Conf_1.000	351	20	5.62E-21	XP_002629947.1	85	103.99	71	61	C. briggsae CBR-PFD-2 protein

Locus_21069_Transcript_1/1_Conf_1.000	308	20	1.90E-29	EFO24692.1	75	132.109	100	75	protein-tyrosine phosphatase
Locus_2107_Transcript_1/1_Conf_1.000	751	20	1.05E-88	XP_001899666.1	82	330.487	235	194	Putative activator 1 36 kDa subunit
Locus_21070_Transcript_1/1_Conf_1.000	131	4	5.60E-13	NP_501176.1	95	77.411	41	39	hypothetical protein T12B3.3
Locus_21071_Transcript_1/2_Conf_1.000	297	0							
Locus_21071_Transcript_2/2_Conf_1.000	195	0							
Locus_21072_Transcript_1/1_Conf_1.000	397	20	4.48E-26	XP_002643199.1	69	120.939	113	79	C. briggsae CBR-TTR-37 protein
Locus_21073_Transcript_1/1_Conf_1.000	436	0							
Locus_21074_Transcript_1/1_Conf_1.000	348	0							
Locus_21075_Transcript_1/1_Conf_1.000	440	20	5.50E-29	XP_002648469.1	73	130.568	110	81	Hypothetical protein CBG24757
Locus_21076_Transcript_1/1_Conf_1.000	165	20	1.69E-17	CAX65056.1	78	92.4337	66	52	C. elegans protein F25C8.3d, partially confirmed by transcript evidence
Locus_21077_Transcript_1/1_Conf_1.000	233	20	1.43E-24	NP_492944.2	87	115.931	70	61	hypothetical protein F32B4.5
Locus_21078_Transcript_1/1_Conf_1.000	141	5	1.84E-08	XP_002641945.1	85	62.3882	35	30	C. briggsae CBR-TAG-198 protein
Locus_21079_Transcript_1/1_Conf_1.000	163	0							
Locus_2108_Transcript_1/1_Conf_1.000	328	9	7.26E-45	XP_002632886.1	89	183.341	109	98	Hypothetical protein CBG15094
Locus_21080_Transcript_1/1_Conf_1.000	308	20	1.02E-06	NP_001082522.1	65	56.6102	60	39	UTP14, U3 small nucleolar ribonucleoprotein, homolog A

Locus_21081_Transcript_1/1_Conf_1.000	634	20	9.42E-81	XP_002638487.1	80	303.523	211	170	Hypothetical protein CBG12917
Locus_21082_Transcript_1/1_Conf_1.000	275	6	1.51E-26	NP_001122724.1	86	122.479	67	58	hypothetical protein Y22D7AL.6
Locus_21083_Transcript_1/1_Conf_1.000	170	20	9.88E-10	XP_002642493.1	77	66.6254	58	45	Hypothetical protein CBG06913
Locus_21084_Transcript_1/1_Conf_1.000	418	20	1.31E-25	XP_002430986.1	56	119.398	146	82	zinc finger protein DHHC domain containing protein, putative
Locus_21085_Transcript_1/1_Conf_1.000	522	20	2.99E-50	XP_002643964.1	71	201.445	173	124	C. briggsae CBR-HUM-4 protein
Locus_21086_Transcript_1/1_Conf_1.000	224	0							
Locus_21087_Transcript_1/1_Conf_1.000	168	0							
Locus_21088_Transcript_1/1_Conf_1.000	342	0							
Locus_21089_Transcript_1/1_Conf_1.000	209	0							
Locus_2109_Transcript_1/1_Conf_1.000	4169	20	0	XP_002638675.1	61	1021.15	1304	799	C. briggsae CBR-PBRM-1 protein
Locus_21090_Transcript_1/1_Conf_1.000	441	0							
Locus_21091_Transcript_1/1_Conf_1.000	408	20	2.85E-25	NP_001022060.1	56	118.242	165	94	CLC-type chloride cHannel family member (clh-3)
Locus_21092_Transcript_1/1_Conf_1.000	280	0							
Locus_21093_Transcript_1/1_Conf_1.000	604	20	2.90E-65	XP_002629799.1	80	251.906	204	165	Hypothetical protein CBG01042
Locus_21094_Transcript_1/1_Conf_1.000	363	4	4.85E-17	XP_001900557.1	57	90.8929	130	75	Bromodomain containing protein
Locus_21095_Transcript_1/1_Conf_1.000	185	0							
Locus_21096_Transcript_1/1_Conf_1.000	319	0							

Locus_21097_Transcript_1/1_Conf_1.000	463	2	1.26E-28	NP_492185.2	69	129.413	153	107	SMG-associated and Lethal family member (smgl-1)
Locus_21098_Transcript_1/1_Conf_1.000	506	0							
Locus_21099_Transcript_1/1_Conf_1.000	242	2	3.28E-05	NP_503409.1	71	51.6026	73	52	SyNapTotagmin family member (snt-5)
Locus_211_Transcript_1/5_Conf_0.588	459	20	8.77E-35	Q94637.1	70	149.828	156	110	Vitellogenin-6
Locus_211_Transcript_2/5_Conf_0.529	314	20	6.74E-22	Q94637.1	72	107.071	108	78	Vitellogenin-6
Locus_211_Transcript_3/5_Conf_0.353	231	20	3.66E-12	NP_001023274.1	65	74.7146	78	51	VITellogenin structural genes (yolk protein genes) family member (vit-6)
Locus_211_Transcript_4/5_Conf_0.471	311	20	4.23E-21	Q94637.1	71	104.375	107	77	Vitellogenin-6
Locus_211_Transcript_5/5_Conf_0.765	551	20	8.07E-39	Q94637.1	68	163.696	188	128	Vitellogenin-6
Locus_2110_Transcript_1/1_Conf_1.000	558	20	3.27E-43	NP_491843.3	88	178.333	100	88	hypothetical protein ZC328.2
Locus_21100_Transcript_1/1_Conf_1.000	163	1	2.60E-26	AAG23764.1	100	121.709	54	54	AF258561_1PP3686
Locus_21101_Transcript_1/1_Conf_1.000	167	20	5.43E-16	XP_002640833.1	87	87.4261	55	48	C. briggsae CBR-KLP-20 protein
Locus_21102_Transcript_1/1_Conf_1.000	297	0							
Locus_21103_Transcript_1/1_Conf_1.000	239	20	8.34E-25	CAR63597.1	87	116.701	70	61	hypothetical protein
Locus_21104_Transcript_1/1_Conf_1.000	175	0							
Locus_21105_Transcript_1/1_Conf_1.000	365	0							

Locus_21106_Transcript_1/1_Conf_1.000	237	0							
Locus_21107_Transcript_1/1_Conf_1.000	275	20	5.54E-37	XP_002646729.1	89	157.147	87	78	C. briggsae CBR-PRX-13 protein
Locus_21108_Transcript_1/1_Conf_1.000	198	0							
Locus_21109_Transcript_1/1_Conf_1.000	309	0							
Locus_2111_Transcript_1/2_Conf_1.000	301	20	3.06E-27	AAT40500.1	72	124.79	94	68	Putative reverse transcriptase, identical
Locus_2111_Transcript_2/2_Conf_1.000	170	15	6.19E-12	ABN08556.1	81	73.9442	53	43	Polyprotein, putative
Locus_21110_Transcript_1/1_Conf_1.000	144	1	1.67E-04	NP_510249.2	61	49.2914	47	29	TREhalase family member (tre-4)
Locus_21111_Transcript_1/1_Conf_1.000	326	0							
Locus_21112_Transcript_1/1_Conf_1.000	276	0							
Locus_21113_Transcript_1/1_Conf_1.000	232	0							
Locus_21114_Transcript_1/1_Conf_1.000	385	20	1.46E-29	XP_002646129.1	84	132.494	86	73	Hypothetical protein CBG08008
Locus_21115_Transcript_1/1_Conf_1.000	353	0							
Locus_21116_Transcript_1/1_Conf_1.000	351	3	1.44E-08	XP_002641791.1	66	62.7734	71	47	Hypothetical protein CBG10142
Locus_21117_Transcript_1/1_Conf_1.000	332	20	1.45E-61	XP_002801767.1	100	238.81	110	110	PREDICTED: sodium/potassium-transporting ATPase subunit alpha-1
Locus_21118_Transcript_1/1_Conf_1.000	191	0							
Locus_21119_Transcript_1/1_Conf_1.000	229	0							

Locus_2112_Transcript_1/2_Conf_1.000	829	20	5.35E-23	EFO19187.1	100	112.464	54	54	ets-domain-containing protein
Locus_2112_Transcript_2/2_Conf_1.000	829	20	5.35E-23	EFO19187.1	100	112.464	54	54	ets-domain-containing protein
Locus_21120_Transcript_1/1_Conf_1.000	447	20	7.80E-39	XP_002661248.1	74	163.31	147	109	PREDICTED: ATP-binding cassette, sub-family C, member 1
Locus_21121_Transcript_1/1_Conf_1.000	254	0							
Locus_21122_Transcript_1/1_Conf_1.000	178	0							

Locus_21123_Transcript_1/1_Conf_1.000	284	20	4.53E-18	XP_001895789.1	73	94.3597	91	67	Timeless protein
Locus_21124_Transcript_1/1_Conf_1.000	358	0							
Locus_21125_Transcript_1/1_Conf_1.000	270	20	7.52E-34	XP_002630757.1	84	146.747	90	76	Hypothetical protein CBG02451
Locus_21126_Transcript_1/1_Conf_1.000	148	0							
Locus_21127_Transcript_1/1_Conf_1.000	305	0							
Locus_21128_Transcript_1/1_Conf_1.000	491	20	1.02E-46	NP_506182.2	79	189.504	149	118	DeHydrogenases, Short chain family member (dhs-21)
Locus_21129_Transcript_1/1_Conf_1.000	259	9	1.60E-15	NP_499482.1	69	85.8853	85	59	defective SPErmatogenesis family member (spe-6)
Locus_2113_Transcript_1/2_Conf_1.000	1452	3	4.50E-28	XP_002630981.1	55	130.568	206	114	Hypothetical protein CBG02720
Locus_2113_Transcript_2/2_Conf_1.000	1454	3	4.50E-28	XP_002630981.1	55	130.568	206	114	Hypothetical protein CBG02720
Locus_21130_Transcript_1/1_Conf_1.000	157	0							
Locus_21131_Transcript_1/1_Conf_1.000	167	0							

Locus_21132_Transcript_1/1_Conf_1.000	188	20	5.68E-29	XP_001109998.2	100	130.568	62	62	PREDICTED: glucose-6-phosphate isomerase
Locus_21133_Transcript_1/1_Conf_1.000	264	0							
Locus_21134_Transcript_1/1_Conf_1.000	133	12	3.17E-08	NP_001041301.1	89	61.6178	37	33	hypothetical protein ZK54.1
Locus_21135_Transcript_1/1_Conf_1.000	139	0							
Locus_21136_Transcript_1/1_Conf_1.000	236	2	2.07E-07	EFO26611.1	65	58.9214	72	47	hypothetical protein LOAG_01867
Locus_21137_Transcript_1/1_Conf_1.000	219	4	1.51E-13	ACI49219.1	90	79.337	42	38	hypothetical protein Csp3_JD05.004
Locus_21138_Transcript_1/1_Conf_1.000	148	0							
Locus_21139_Transcript_1/1_Conf_1.000	174	0							
Locus_2114_Transcript_1/1_Conf_1.000	351	4	1.94E-13	XP_002640867.1	73	78.9518	60	44	Hypothetical protein CBG15758
Locus_21140_Transcript_1/1_Conf_1.000	139	0							
Locus_21141_Transcript_1/1_Conf_1.000	195	0							
Locus_21142_Transcript_1/1_Conf_1.000	262	0							
Locus_21143_Transcript_1/1_Conf_1.000	189	0							
Locus_21144_Transcript_1/1_Conf_1.000	192	0							
Locus_21145_Transcript_1/1_Conf_1.000	190	0							
Locus_21146_Transcript_1/1_Conf_1.000	187	20	1.15E-29	EAW90757.1	100	132.88	61	61	microsomal glutathione S-transferase 3, isoform CRA_d
Locus_21147_Transcript_1/1_Conf_1.000	136	20	1.67E-09	XP_002104105.1	84	65.855	44	37	GD18625
Locus_21148_Transcript_1/1_Conf_1.000	197	0							

Locus_21149_Transcript_1/1_Conf_1.000	197	0							
Locus_2115_Transcript_1/1_Conf_1.000	2245	20	2.00E-100	NP_501805.1	59	371.703	694	413	hypothetical protein F44D12.1
Locus_21150_Transcript_1/1_Conf_1.000	271	0							
Locus_21151_Transcript_1/1_Conf_1.000	469	1	1.25E-12	NP_500548.1	52	76.2554	162	85	hypothetical protein W03B1.9
Locus_21152_Transcript_1/1_Conf_1.000	343	20	5.66E-37	ACI49016.1	83	157.147	112	94	hypothetical protein Cbre_JD04.001
Locus_21153_Transcript_1/1_Conf_1.000	205	0							
Locus_21154_Transcript_1/1_Conf_1.000	204	20	2.77E-20	NP_503846.1	82	101.679	67	55	CYtochrome P450 family member (cyp-33C9)
Locus_21155_Transcript_1/1_Conf_1.000	182	0							
Locus_21156_Transcript_1/1_Conf_1.000	274	2	8.89E-19	NP_001024902.1	68	96.6709	90	62	hypothetical protein T10B10.4
Locus_21157_Transcript_1/1_Conf_1.000	302	0							
Locus_21158_Transcript_1/1_Conf_1.000	505	20	2.51E-40	EFO24958.1	71	168.318	151	108	hypothetical protein LOAG_03524
Locus_21159_Transcript_1/1_Conf_1.000	169	0							
Locus_2116_Transcript_1/1_Conf_1.000	284	0							
Locus_21160_Transcript_1/1_Conf_1.000	193	0							
Locus_21161_Transcript_1/1_Conf_1.000	136	3	1.98E-10	NP_491382.1	90	68.9366	44	40	hypothetical protein Y119C1B.5
Locus_21162_Transcript_1/1_Conf_1.000	323	5	5.12E-14	3N9L	60	80.8777	110	66	Cekdm7a
Locus_21163_Transcript_1/1_Conf_1.000	418	0							
Locus_21164_Transcript_1/1_Conf_1.000	128	0							
Locus_21165_Transcript_1/1_Conf_1.000	149	0							
Locus_21166_Transcript_1/1_Conf_1.000	322	20	1.53E-42	NP_505019.2	81	175.637	107	87	hypothetical protein C37C3.7
Locus_21167_Transcript_1/1_Conf_1.000	187	0							
Locus_21168_Transcript_1/1_Conf_1.000	229	13	1.93E-37	EAW74622.1	100	158.688	75	75	protein tyrosine phosphatase, receptor type, C-associated protein
Locus_21169_Transcript_1/1_Conf_1.000	210	0							
Locus_2117_Transcript_1/1_Conf_1.000	503	20	1.43E-48	NP_497411.2	76	195.667	170	130	hypothetical protein Y82E9BR.18
Locus_21170_Transcript_1/1_Conf_1.000	339	0							
Locus_21171_Transcript_1/1_Conf_1.000	288	0							
Locus_21172_Transcript_1/1_Conf_1.000	197	5	2.38E-11	NP_001122672.1	76	72.0182	63	48	Regulator of G protein Signaling family member (rgs-5)
Locus_21173_Transcript_1/1_Conf_1.000	274	5	3.87E-06	XP_002595725.1	55	54.6842	90	50	hypothetical protein BRAFLDRAFT_200329
Locus_21174_Transcript_1/1_Conf_1.000	202	0							
Locus_21175_Transcript_1/1_Conf_1.000	155	0							
Locus_21176_Transcript_1/1_Conf_1.000	140	15	1.97E-10	BAH14685.1	100	68.9366	46	46	unnamed protein product
Locus_21177_Transcript_1/2_Conf_1.000	560	11	5.15E-89	XP_001900400.1	87	330.487	187	163	Zinc finger, C2H2 type family protein

Locus_21177_Transcript_2/2_Conf_1.000	560	11	5.15E-89	XP_001900400.1	87	330.487	187	163	Zinc finger, C2H2 type family protein
Locus_21178_Transcript_1/1_Conf_1.000	419	20	6.47E-33	XP_002640010.1	72	143.665	128	93	Hypothetical protein CBG12480
Locus_21179_Transcript_1/1_Conf_1.000	346	0							
Locus_2118_Transcript_1/1_Conf_1.000	835	4	2.44E-23	NP_492051.1	49	113.62	250	124	yeast BUB homolog family member (bub-1)
Locus_21180_Transcript_1/1_Conf_1.000	397	20	6.72E-06	XP_002871971.1	49	53.9138	91	45	PAZ domain-containing protein
Locus_21181_Transcript_1/1_Conf_1.000	210	5	2.75E-20	XP_002641791.1	85	101.679	70	60	Hypothetical protein CBG10142
Locus_21182_Transcript_1/1_Conf_1.000	136	0							
Locus_21183_Transcript_1/1_Conf_1.000	260	20	1.82E-19	XP_002646198.1	69	98.9821	86	60	Hypothetical protein CBG04359
Locus_21184_Transcript_1/1_Conf_1.000	252	20	4.53E-10	XP_001897797.1	82	67.781	46	38	Ets-domain containing protein
Locus_21185_Transcript_1/1_Conf_1.000	364	18	9.15E-16	XP_002647704.1	59	86.6557	108	64	Hypothetical protein CBG17893
Locus_21186_Transcript_1/1_Conf_1.000	139	0							
Locus_21187_Transcript_1/1_Conf_1.000	153	2	6.51E-09	XP_002634130.1	80	63.929	45	36	Hypothetical protein CBG01688
Locus_21188_Transcript_1/1_Conf_1.000	149	0							
Locus_21189_Transcript_1/1_Conf_1.000	377	20	2.62E-10	XP_002415173.1	61	68.5514	71	44	sentrin/sumo-specific protease, putative
Locus_2119_Transcript_1/1_Conf_1.000	256	20	1.27E-20	EFO17214.1	78	102.834	71	56	DOMON domain-containing protein
Locus_21190_Transcript_1/1_Conf_1.000	245	0							
Locus_21191_Transcript_1/1_Conf_1.000	150	0							
Locus_21192_Transcript_1/1_Conf_1.000	328	20	7.05E-32	NP_505775.3	77	140.198	104	81	hypothetical protein F32D8.4
Locus_21193_Transcript_1/1_Conf_1.000	212	0							
Locus_21194_Transcript_1/1_Conf_1.000	145	0							
Locus_21195_Transcript_1/1_Conf_1.000	205	20	5.59E-21	EFO25626.1	96	103.99	56	54	hypothetical protein LOAG_02863
Locus_21196_Transcript_1/1_Conf_1.000	477								
Locus_21197_Transcript_1/1_Conf_1.000	374	20	6.47E-17	XP_001356645.1	58	90.5077	124	73	GA20218

Locus_21198_Transcript_1/1_Conf_1.000	137	0								
Locus_21199_Transcript_1/1_Conf_1.000	537	0								
Locus_212_Transcript_1/1_Conf_1.000	449	20	3.75E-33	XP_001900551.1	63	144.436	148	94	Protein kinase domain containing protein
Locus_2120_Transcript_1/1_Conf_1.000	619	20	1.54E-64	NP_001122891.1	75	249.595	213	160	hypothetical protein C53A5.2
Locus_21200_Transcript_1/1_Conf_1.000	226	20	2.47E-16	XP_001894892.1	76	88.5817	75	57	RhoGAP domain containing protein
Locus_21201_Transcript_1/1_Conf_1.000	415									
Locus_21202_Transcript_1/1_Conf_1.000	232	20	2.00E-18	NP_499164.1	76	95.5153	75	57	SQuashed Vulva family member (sqv-3)
Locus_21203_Transcript_1/1_Conf_1.000	135	0								
Locus_21204_Transcript_1/1_Conf_1.000	138	0								
Locus_21205_Transcript_1/1_Conf_1.000	165	20	2.28E-14	EFO21603.1	85	82.0333	55	47	hypothetical protein LOAG_06885
Locus_21206_Transcript_1/1_Conf_1.000	130	0								
Locus_21207_Transcript_1/1_Conf_1.000	165	0								
Locus_21208_Transcript_1/1_Conf_1.000	618	0								
Locus_21209_Transcript_1/1_Conf_1.000	492	0								
Locus_2121_Transcript_1/1_Conf_1.000	1563	20	4.01E-62	NP_496095.1	63	243.817	276	174	WEE homolog family member (wee-1.3)
Locus_21210_Transcript_1/1_Conf_1.000	356	0								
Locus_21211_Transcript_1/1_Conf_1.000	338	0								
Locus_21212_Transcript_1/1_Conf_1.000	131	0								
Locus_21213_Transcript_1/1_Conf_1.000	171	0								
Locus_21214_Transcript_1/1_Conf_1.000	162	5	1.49E-05	NP_492318.1	80	52.7582	40	32	hypothetical protein T01H8.2

Locus_21215_Transcript_1/1_Conf_1.000	429	20	7.53E-26	XP_001901553.1	60	120.168	146	89	Protein-tyrosine phosphatase containing protein
Locus_21216_Transcript_1/1_Conf_1.000	199	20	7.08E-32	BAG60652.1	100	140.198	66	66	unnamed protein product
Locus_21217_Transcript_1/1_Conf_1.000	350	0							
Locus_21218_Transcript_1/1_Conf_1.000	337	4	1.85E-08	XP_002641335.1	60	62.3882	88	53	Hypothetical protein CBG13183
Locus_21219_Transcript_1/1_Conf_1.000	204	0							
Locus_2122_Transcript_1/1_Conf_1.000	1418	20	1.26E-144	NP_494771.1	77	517.694	420	325	hypothetical protein B0286.3
Locus_21220_Transcript_1/1_Conf_1.000	299	2	2.44E-16	NP_492118.1	68	88.5817	98	67	hypothetical protein D2030.3
Locus_21221_Transcript_1/1_Conf_1.000	479	20	3.13E-40	NP_500629.1	86	167.933	104	90	CYtochrome C family member (cyc-2.1)
Locus_21222_Transcript_1/1_Conf_1.000	180	0							
Locus_21223_Transcript_1/1_Conf_1.000	359	20	2.74E-52	XP_002638749.1	92	207.994	119	110	Hypothetical protein CBG18552
Locus_21224_Transcript_1/1_Conf_1.000	169	0							

Locus_21225_Transcript_1/1_Conf_1.000	237	20	4.73E-28	NP_500379.1	79	127.487	78	62	hypothetical protein Y37E11B.5
Locus_21226_Transcript_1/1_Conf_1.000	319	20	1.03E-22	NP_494841.3	69	109.768	91	63	hypothetical protein F10G7.10
Locus_21227_Transcript_1/1_Conf_1.000	221	0							
Locus_21228_Transcript_1/1_Conf_1.000	201	4	5.07E-22	CAR63710.1	88	107.457	67	59	putative C2 domain containing protein
Locus_21229_Transcript_1/1_Conf_1.000	172	20	7.52E-18	XP_002639780.1	85	93.5893	56	48	Hypothetical protein CBG02227
Locus_2123_Transcript_1/1_Conf_1.000	623	20	1.16E-88	XP_002639761.1	89	329.717	200	179	C. briggsae CBR-PAB-1 protein
Locus_21230_Transcript_1/1_Conf_1.000	391	0							
Locus_21231_Transcript_1/1_Conf_1.000	405	20	8.04E-36	2OS5	82	153.295	114	94	Macrophage Migration Inhibitory Factor
Locus_21232_Transcript_1/1_Conf_1.000	200	0							

Locus_21233_Transcript_1/1_Conf_1.000	717	20	6.35E-85	NP_497203.1	81	317.775	228	185	Metaphase-to-Anaphase Transition defect family member (mat-3)
Locus_21234_Transcript_1/1_Conf_1.000	311	0							
Locus_21235_Transcript_1/1_Conf_1.000	228	20	2.62E-26	NP_001022450.1	92	121.709	75	69	hypothetical protein Y48E1A.1
Locus_21236_Transcript_1/1_Conf_1.000	278	0							
Locus_21237_Transcript_1/1_Conf_1.000	266	0							
Locus_21238_Transcript_1/1_Conf_1.000	197	20	5.29E-11	XP_001894671.1	79	70.8626	59	47	Protein kinase domain containing protein
Locus_21239_Transcript_1/1_Conf_1.000	159	0							
Locus_2124_Transcript_1/1_Conf_1.000	187	0							
Locus_21240_Transcript_1/1_Conf_1.000	148	0							
Locus_21241_Transcript_1/1_Conf_1.000	260	0							
Locus_21242_Transcript_1/1_Conf_1.000	239	1	2.52E-05	XP_002630993.1	63	51.9878	57	36	Hypothetical protein CBG02738
Locus_21243_Transcript_1/1_Conf_1.000	163	20	8.97E-19	NP_505312.1	90	96.6709	54	49	hypothetical protein T19A5.1
Locus_21244_Transcript_1/1_Conf_1.000	169	20	3.63E-12	NP_741501.1	70	74.7146	55	39	hypothetical protein F11E6.1
Locus_21245_Transcript_1/1_Conf_1.000	152	0							
Locus_21246_Transcript_1/1_Conf_1.000	133	0							

Locus_21247_Transcript_1/1_Conf_1.000	180	0							
Locus_21248_Transcript_1/1_Conf_1.000	166	0							
Locus_21249_Transcript_1/1_Conf_1.000	165	0							
Locus_2125_Transcript_1/3_Conf_0.714	1468	2	2.97E-19	XP_002634618.1	41	101.293	523	217	Hypothetical protein CBG18472
Locus_2125_Transcript_2/3_Conf_0.714	1468	2	1.13E-18	NP_501436.1	41	99.3673	526	216	hypothetical protein T26A8.1
Locus_2125_Transcript_3/3_Conf_0.714	1468	2	1.13E-18	NP_501436.1	41	99.3673	526	216	hypothetical protein T26A8.1
Locus_21250_Transcript_1/1_Conf_1.000	248	17	2.67E-07	XP_517936.2	80	58.5362	40	32	PREDICTED: PHD finger protein 15
Locus_21251_Transcript_1/1_Conf_1.000	207	0							
Locus_21252_Transcript_1/1_Conf_1.000	261	0							
Locus_21253_Transcript_1/1_Conf_1.000	848	20	2.50E-31	AAO63576.1	55	140.198	183	101	secreted protein 4 precursor
Locus_21254_Transcript_1/1_Conf_1.000	393	20	1.22E-39	EFO19650.1	75	166.007	129	98	NAK/GAK protein kinase
Locus_21255_Transcript_1/1_Conf_1.000	203	0							
Locus_21256_Transcript_1/1_Conf_1.000	142	0							
Locus_21257_Transcript_1/1_Conf_1.000	509	0							
Locus_21258_Transcript_1/1_Conf_1.000	393	20	3.30E-53	XP_002645892.1	92	211.075	118	109	Hypothetical protein CBG07642
Locus_21259_Transcript_1/1_Conf_1.000	237	20	7.31E-21	NP_001123159.1	74	103.605	78	58	SYnaptoGenesis abnormal family member (syg-1)

Locus_2126_Transcript_1/1_Conf_1.000	2907	20	3.03E-168	EFO26291.1	57	597.43	911	520	hypothetical protein LOAG_02191
Locus_21260_Transcript_1/1_Conf_1.000	268	20	1.38E-35	ACZ64269.1	98	152.525	88	87	astacin metalloprotease b
Locus_21261_Transcript_1/1_Conf_1.000	147	0							
Locus_21262_Transcript_1/1_Conf_1.000	253	20	1.04E-14	XP_002629772.1	63	83.1889	86	55	Hypothetical protein CBG01012
Locus_21263_Transcript_1/1_Conf_1.000	137	0							
Locus_21264_Transcript_1/1_Conf_1.000	144	20	1.67E-12	AAF82410.1	87	75.8702	47	41	AF221132_1diacylglycerol acyltransferase
Locus_21265_Transcript_1/1_Conf_1.000	147	20	3.70E-20	XP_002809166.1	100	101.293	48	48	PREDICTED: antigen peptide transporter 1-like
Locus_21266_Transcript_1/1_Conf_1.000	305	3	1.86E-08	NP_497061.1	62	62.3882	66	41	hypothetical protein Y54E2A.5
Locus_21267_Transcript_1/1_Conf_1.000	142	20	3.70E-17	NP_497809.1	97	91.2781	47	46	Heavy chain, Unconventional Myosin family member (hum-5)

Locus_21268_Transcript_1/1_Conf_1.000	244	20	1.41E-24	NP_491242.2	86	115.931	80	69	hypothetical protein T03F1.7
Locus_21269_Transcript_1/1_Conf_1.000	176	4	1.02E-06	EFO23768.1	66	56.6102	60	40	hypothetical protein LOAG_04713
Locus_2127_Transcript_1/1_Conf_1.000	145	0							
Locus_21270_Transcript_1/1_Conf_1.000	301	0							
Locus_21271_Transcript_1/1_Conf_1.000	190	0							
Locus_21272_Transcript_1/1_Conf_1.000	218	20	3.57E-20	NP_001181828.1	100	101.293	44	44	cathepsin B
Locus_21273_Transcript_1/1_Conf_1.000	132	0							
Locus_21274_Transcript_1/1_Conf_1.000	251	0							
Locus_21275_Transcript_1/1_Conf_1.000	470	20	8.28E-65	NP_500551.2	89	249.595	155	138	hypothetical protein F55F10.1
Locus_21276_Transcript_1/1_Conf_1.000	203	20	1.17E-18	NP_509676.1	98	96.2857	67	66	G-protein-coupled Receptor Kinase family member (grk-1)
Locus_21277_Transcript_1/1_Conf_1.000	814	0							

Locus_21278_Transcript_1/1_Conf_1.000	161	16	9.64E-13	NP_001122528.1	78	76.6406	52	41	Cation transporting ATPase family member (catp-1)
Locus_21279_Transcript_1/1_Conf_1.000	250	20	4.67E-20	NP_500531.1	82	100.908	81	67	hypothetical protein F15E6.6
Locus_2128_Transcript_1/4_Conf_0.429	1076	15	1.96E-24	XP_002630067.1	89	117.857	74	66	C. briggsae CBR-IPLA-1 protein
Locus_2128_Transcript_2/4_Conf_0.571	1204	20	5.36E-29	XP_002630067.1	77	133.265	111	86	C. briggsae CBR-IPLA-1 protein
Locus_2128_Transcript_3/4_Conf_0.571	2720	20	0	XP_002630067.1	73	731.865	664	490	C. briggsae CBR-IPLA-1 protein
Locus_2128_Transcript_4/4_Conf_0.571	2754	20	0	XP_002630067.1	73	731.865	664	490	C. briggsae CBR-IPLA-1 protein
Locus_21280_Transcript_1/1_Conf_1.000	289	0							
Locus_21281_Transcript_1/1_Conf_1.000	330	1	2.43E-08	NP_741467.1	67	62.003	59	40	hypothetical protein C09G4.2
Locus_21282_Transcript_1/1_Conf_1.000	323	20	2.29E-22	EFO17955.1	73	108.612	102	75	hypothetical protein LOAG_10542
Locus_21283_Transcript_1/1_Conf_1.000	216	5	1.84E-08	NP_741348.2	68	62.3882	66	45	hypothetical protein Y94H6A.5
Locus_21284_Transcript_1/1_Conf_1.000	274	0							
Locus_21285_Transcript_1/1_Conf_1.000	166	5	8.94E-19	NP_001122868.1	92	96.6709	54	50	GLIoTactin (Drosophila neuroligin-like) homolog family member (nrx-1)
Locus_21286_Transcript_1/1_Conf_1.000	218	8	1.01E-06	XP_001900229.1	62	56.6102	67	42	FAT domain containing protein
Locus_21287_Transcript_1/1_Conf_1.000	208	2	7.31E-05	XP_001901751.1	61	50.447	68	42	mKIAA0368 protein
Locus_21288_Transcript_1/1_Conf_1.000	232	6	4.05E-11	NP_492132.1	67	71.2478	71	48	Enhancer of Glp-One (glp-1) family member (ego-1)
Locus_21289_Transcript_1/1_Conf_1.000	276	0							

Locus_2129_Transcript_1/1_Conf_1.000	361	20	7.93E-60	XP_001156335.1	100	233.032	113	113	PREDICTED: similar to proliferation associated gene (pag) isoform 1
Locus_21290_Transcript_1/1_Conf_1.000	166	0							
Locus_21291_Transcript_1/1_Conf_1.000	291	12	1.49E-13	XP_002637289.1	69	79.337	96	67	Hypothetical protein CBG18976
Locus_21292_Transcript_1/1_Conf_1.000	167	0							
Locus_21293_Transcript_1/1_Conf_1.000	236	0							
Locus_21294_Transcript_1/1_Conf_1.000	283	0							
Locus_21295_Transcript_1/1_Conf_1.000	314	20	1.00E-33	XP_002643878.1	73	146.362	102	75	Hypothetical protein CBG02116
Locus_21296_Transcript_1/1_Conf_1.000	224	0							
Locus_21297_Transcript_1/1_Conf_1.000	457	20	1.84E-24	EFO15194.1	54	115.546	175	95	hypothetical protein LOAG_13318
Locus_21298_Transcript_1/1_Conf_1.000	129	2	5.98E-15	NP_491129.1	95	83.9593	42	40	hypothetical protein Y54E10A.16
Locus_21299_Transcript_1/1_Conf_1.000	230	0							
Locus_213_Transcript_1/6_Conf_0.727	1047	20	7.12E-40	XP_002639074.1	83	169.088	114	95	C. briggsae CBR-CEY-2 protein
Locus_213_Transcript_2/6_Conf_0.727	1047	20	1.59E-39	XP_002639074.1	85	167.933	109	93	C. briggsae CBR-CEY-2 protein
Locus_213_Transcript_3/6_Conf_0.409	1026	20	1.15E-42	XP_002639788.1	73	178.333	145	107	Hypothetical protein CBG02238

Locus_213_Transcript_4/6_Conf_0.409	1068	20	5.44E-43	XP_002639788.1	72	179.489	150	109	Hypothetical protein CBG02238
Locus_213_Transcript_5/6_Conf_0.409	1155	20	6.11E-43	XP_002639788.1	72	179.489	150	109	Hypothetical protein CBG02238
Locus_213_Transcript_6/6_Conf_0.409	1155	20	1.36E-42	XP_002639788.1	73	178.333	145	107	Hypothetical protein CBG02238
Locus_2130_Transcript_1/1_Conf_1.000	1047	20	2.01E-26	XP_002636810.1	49	124.405	247	123	C. briggsae CBR-MUT-14 protein
Locus_21300_Transcript_1/1_Conf_1.000	220	0							
Locus_21301_Transcript_1/1_Conf_1.000	303	20	1.52E-18	NP_741664.1	82	95.9005	76	63	hypothetical protein Y39B6A.33
Locus_21302_Transcript_1/1_Conf_1.000	381	0							
Locus_21303_Transcript_1/1_Conf_1.000	443	20	6.21E-52	AAN11401.1	75	206.838	147	111	metalloprotease 1 precursor
Locus_21304_Transcript_1/1_Conf_1.000	275	20	1.56E-39	XP_002811594.1	100	165.622	82	82	PREDICTED: protein DJ-1-like isoform 1
Locus_21305_Transcript_1/1_Conf_1.000	230	0							
Locus_21306_Transcript_1/1_Conf_1.000	336	0							
Locus_21307_Transcript_1/1_Conf_1.000	558	0							

Locus_21308_Transcript_1/1_Conf_1.000	224	20	7.66E-34	NP_001040798.1	98	146.747	74	73	PhosphoDiEsterase family member (pde-4)
Locus_21309_Transcript_1/1_Conf_1.000	247	6	8.29E-09	XP_001896275.1	71	63.5438	64	46	Ubiquitin carboxyl-terminal hydrolase family protein
Locus_2131_Transcript_1/1_Conf_1.000	723	20	1.10E-108	ABS45067.1	87	396.741	239	208	latrophilin-like protein 2
Locus_21310_Transcript_1/1_Conf_1.000	194	20	1.65E-12	XP_002630862.1	82	75.8702	58	48	C. briggsae CBR-RRT-2 protein
Locus_21311_Transcript_1/1_Conf_1.000	558	20	3.61E-50	XP_002631065.1	75	201.445	185	140	C. briggsae CBR-SFXN-1.4 protein
Locus_21312_Transcript_1/1_Conf_1.000	189	3	4.08E-11	NP_490976.1	70	71.2478	62	44	hypothetical protein F23C8.8
Locus_21313_Transcript_1/1_Conf_1.000	237	0							
Locus_21314_Transcript_1/1_Conf_1.000	280	0							
Locus_21315_Transcript_1/1_Conf_1.000	193	20	1.65E-20	XP_001896049.1	95	102.449	63	60	Patched family protein
Locus_21316_Transcript_1/1_Conf_1.000	132	0							
Locus_21317_Transcript_1/1_Conf_1.000	175	0							
Locus_21318_Transcript_1/1_Conf_1.000	223	0							
Locus_21319_Transcript_1/1_Conf_1.000	230	0							
Locus_2132_Transcript_1/1_Conf_1.000	260	20	1.70E-33	EFO18852.1	94	145.591	84	79	RNA polymerase III 128kD subunit-PA
Locus_21320_Transcript_1/1_Conf_1.000	172	0							
Locus_21321_Transcript_1/1_Conf_1.000	156	0							
Locus_21322_Transcript_1/1_Conf_1.000	207	9	3.38E-18	AAX36081.1	84	94.7449	69	58	transforming growth factor beta
Locus_21323_Transcript_1/1_Conf_1.000	461	10	1.08E-16	NP_498049.2	77	89.7373	66	51	FLYWCH zinc finger transcription factor homolog family member (flh-2)

Locus_21324_Transcript_1/1_Conf_1.000	446	20	7.86E-31	ABJ99064.1	67	136.732	145	98	Hypothetical protein W03A3.2
Locus_21325_Transcript_1/1_Conf_1.000	245	0							
Locus_21326_Transcript_1/1_Conf_1.000	197	0							
Locus_21327_Transcript_1/1_Conf_1.000	203	0							
Locus_21328_Transcript_1/1_Conf_1.000	183	20	3.83E-17	XP_002912747.1	100	91.2781	60	60	PREDICTED: tropomyosin alpha-1 chain-like isoform 2
Locus_21329_Transcript_1/1_Conf_1.000	158	1	3.54E-23	AAQ15254.1	100	111.309	52	52	AF370418_1PP14214
Locus_2133_Transcript_1/2_Conf_1.000	1256	20	8.58E-86	XP_002633413.1	81	322.013	250	203	C. briggsae CBR-ECH-2 protein
Locus_2133_Transcript_2/2_Conf_1.000	1132	20	2.92E-90	XP_002633413.1	80	336.65	266	214	C. briggsae CBR-ECH-2 protein
Locus_21330_Transcript_1/1_Conf_1.000	281	0							
Locus_21331_Transcript_1/1_Conf_1.000	423	0							
Locus_21332_Transcript_1/1_Conf_1.000	228	19	6.23E-28	NP_505844.2	90	127.102	75	68	hypothetical protein K10D6.2
Locus_21333_Transcript_1/1_Conf_1.000	244	0							
Locus_21334_Transcript_1/1_Conf_1.000	259	0							
Locus_21335_Transcript_1/1_Conf_1.000	391	20	6.12E-12	NP_492367.1	49	73.9442	139	69	Glycogen Synthase Kinase Alpha subunit family member (gska-3)
Locus_21336_Transcript_1/1_Conf_1.000	137	0							
Locus_21337_Transcript_1/1_Conf_1.000	249	3	6.10E-20	NP_493486.2	84	100.523	72	61	hypothetical protein C49A1.10

Locus_21338_Transcript_1/1_Conf_1.000	303	0							
Locus_21339_Transcript_1/1_Conf_1.000	168	0							
Locus_2134_Transcript_1/1_Conf_1.000	524	1	3.24E-04	XP_001639273.1	42	48.521	109	46	predicted protein
Locus_21340_Transcript_1/1_Conf_1.000	154	20	1.14E-21	XP_002745917.1	100	106.301	50	50	PREDICTED: heterogeneous nuclear ribonucleoproteins A2/B1-like
Locus_21341_Transcript_1/2_Conf_1.000	299	4	2.80E-04	EDL38626.1	73	48.521	49	36	multiple coagulation factor deficiency 2, isoform CRA_b
Locus_21341_Transcript_2/2_Conf_1.000	282	4	2.85E-04	EDL38626.1	73	48.521	49	36	multiple coagulation factor deficiency 2, isoform CRA_b
Locus_21342_Transcript_1/1_Conf_1.000	261	0							
Locus_21343_Transcript_1/1_Conf_1.000	378	20	1.29E-17	XP_002634314.1	72	92.8189	74	54	Hypothetical protein CBG17655
Locus_21344_Transcript_1/1_Conf_1.000	168	9	1.09E-24	ACR27076.1	100	116.316	56	56	transforming growth protein 2-like protein
Locus_21345_Transcript_1/1_Conf_1.000	241	2	4.41E-26	XP_002634586.1	88	120.939	79	70	Hypothetical protein CBG08397
Locus_21346_Transcript_1/1_Conf_1.000	154	20	1.07E-19	XP_002929569.1	100	99.7525	49	49	PREDICTED: transcription factor BTF3-like
Locus_21347_Transcript_1/1_Conf_1.000	261	7	1.26E-04	XP_002413556.1	61	49.6766	60	37	RB13-6 antigen, putative
Locus_21348_Transcript_1/1_Conf_1.000	194	0							
Locus_21349_Transcript_1/1_Conf_1.000	141	17	5.16E-19	XP_002820885.1	100	97.4413	46	46	PREDICTED: DNA damage-inducible transcript 4 protein-like isoform 1

Locus_2135_Transcript_1/1_Conf_1.000	436	20	5.87E-47	NP_508413.1	98	190.274	117	115	Ribosomal Protein, Large subunit family member (rpl-11.2)
Locus_21350_Transcript_1/1_Conf_1.000	458	20	5.94E-15	XP_001896061.1	52	83.9593	146	77	hypothetical protein Bm1_23020
Locus_21351_Transcript_1/1_Conf_1.000	371	0							
Locus_21352_Transcript_1/1_Conf_1.000	138	0							
Locus_21353_Transcript_1/1_Conf_1.000	194	0							
Locus_21354_Transcript_1/1_Conf_1.000	333	20	5.38E-16	NP_506227.1	54	87.4261	111	61	TransThyretin-Related family domain family member (ttr-29)
Locus_21355_Transcript_1/1_Conf_1.000	235	20	7.07E-32	XP_002632463.1	96	140.198	78	75	Hypothetical protein CBG13687
Locus_21356_Transcript_1/1_Conf_1.000	130	0							
Locus_21357_Transcript_1/1_Conf_1.000	281	1	3.60E-04	XP_002630436.1	70	48.1358	41	29	Hypothetical protein CBG11167
Locus_21358_Transcript_1/1_Conf_1.000	225	0							
Locus_21359_Transcript_1/1_Conf_1.000	465	20	3.76E-41	NP_001024271.1	69	171.014	152	105	CHeckpoint Kinase family member (chk-2)
Locus_2136_Transcript_1/1_Conf_1.000	2284	20	0	NP_741567.1	84	1033.86	710	601	eXPOrtin (nuclear export receptor) family member (xpo-1)
Locus_21360_Transcript_1/1_Conf_1.000	202	0							

Locus_21361_Transcript_1/1_Conf_1.000	219	10	1.37E-06	XP_001901212.1	52	56.225	61	32	Furin-like cysteine rich region family protein
Locus_21362_Transcript_1/1_Conf_1.000	907	20	4.00E-78	CAB16487.2	79	295.819	260	206	C. elegans protein Y53C12A.3, confirmed by transcript evidence
Locus_21363_Transcript_1/1_Conf_1.000	160	0							
Locus_21364_Transcript_1/1_Conf_1.000	294	3	1.54E-10	NP_491682.1	59	69.3218	101	60	MTK1/MEKK4 homolog family member (mtk-1)
Locus_21365_Transcript_1/1_Conf_1.000	281	20	4.83E-25	XP_002647206.1	73	117.472	91	67	Hypothetical protein CBG22384
Locus_21366_Transcript_1/1_Conf_1.000	165	0							
Locus_21367_Transcript_1/1_Conf_1.000	271	0							
Locus_21368_Transcript_1/1_Conf_1.000	186	0							
Locus_21369_Transcript_1/1_Conf_1.000	252	0							
Locus_2137_Transcript_1/1_Conf_1.000	667	20	1.55E-31	XP_001898376.1	61	140.198	215	132	corneal wound healing-related protein
Locus_21370_Transcript_1/1_Conf_1.000	376	20	4.17E-24	XP_002640523.1	82	114.39	78	64	Hypothetical protein CBG18685
Locus_21371_Transcript_1/1_Conf_1.000	165	20	8.96E-11	EFO27900.1	71	70.0922	57	41	ATP-dependent DNA helicase
Locus_21372_Transcript_1/1_Conf_1.000	110	0							
Locus_21373_Transcript_1/1_Conf_1.000	351	0							
Locus_21374_Transcript_1/1_Conf_1.000	269	0							
Locus_21375_Transcript_1/1_Conf_1.000	154	0							

Locus_21376_Transcript_1/1_Conf_1.000	186	1	4.85E-04	XP_002632226.1	66	47.7506	50	33	Hypothetical protein CBG07097
Locus_21377_Transcript_1/1_Conf_1.000	299	0							
Locus_21378_Transcript_1/1_Conf_1.000	252	20	2.46E-40	NP_001024999.1	97	168.318	80	78	Nuclear Hormone Receptor family member (nhr-48)
Locus_21379_Transcript_1/1_Conf_1.000	333	20	2.75E-28	EFO24634.1	71	128.257	110	79	hypothetical protein LOAG_03853
Locus_2138_Transcript_1/1_Conf_1.000	901	20	3.01E-102	ACN93881.1	88	375.941	254	224	putative glutamate-cysteine ligase modifier subunit
Locus_21380_Transcript_1/2_Conf_1.000	383	20	1.99E-10	XP_002637015.1	59	68.9366	87	52	Hypothetical protein CBG09507
Locus_21380_Transcript_2/2_Conf_1.000	407	20	1.29E-17	NP_507628.1	59	92.8189	124	74	TransThyretin-Related family domain family member (ttr-24)
Locus_21381_Transcript_1/1_Conf_1.000	539	0							
Locus_21382_Transcript_1/1_Conf_1.000	130	0							
Locus_21383_Transcript_1/1_Conf_1.000	283	18	8.86E-06	NP_504810.2	75	53.5286	36	27	UDP-GlucuronosylTransferase family member (ugt-40)
Locus_21384_Transcript_1/1_Conf_1.000	276	0							
Locus_21385_Transcript_1/1_Conf_1.000	313	20	1.32E-17	NP_500737.1	64	92.8189	97	63	hypothetical protein W03F8.3
Locus_21386_Transcript_1/2_Conf_1.000	779	20	2.05E-50	EFO28439.1	62	203.371	250	157	transcription factor E2F-4

Locus_21386_Transcript_2/2_Conf_1.000	764	20	9.77E-50	EFO28439.1	62	201.06	249	155	transcription factor E2F-4
Locus_21387_Transcript_1/1_Conf_1.000	238	20	2.51E-21	XP_002646431.1	81	105.145	79	64	Hypothetical protein CBG18813
Locus_21388_Transcript_1/1_Conf_1.000	314	9	1.15E-13	XP_001895480.1	60	79.7221	100	60	Immunoglobulin I-set domain containing protein
Locus_21389_Transcript_1/1_Conf_1.000	349	8	3.67E-12	NP_001021225.1	62	74.7146	98	61	CEll Death abnormality family member (ced-7)
Locus_2139_Transcript_1/1_Conf_1.000	1861	0							
Locus_21390_Transcript_1/1_Conf_1.000	242	20	3.37E-26	XP_002644773.1	86	121.324	67	58	Hypothetical protein CBG14786
Locus_21391_Transcript_1/1_Conf_1.000	381	20	7.47E-58	XP_002643489.1	94	226.483	127	120	C. briggsae CBR-APA-2 protein
Locus_21392_Transcript_1/1_Conf_1.000	281	20	1.05E-11	XP_002629730.1	87	73.1738	48	42	Hypothetical protein CBG00961
Locus_21393_Transcript_1/1_Conf_1.000	171	0							
Locus_21394_Transcript_1/1_Conf_1.000	559	2	7.59E-16	XP_002643026.1	54	87.4261	192	104	Hypothetical protein CBG22933
Locus_21395_Transcript_1/1_Conf_1.000	283	20	5.02E-09	XP_002595424.1	55	64.3142	94	52	hypothetical protein BRAFLDRAFT_69255
Locus_21396_Transcript_1/1_Conf_1.000	237	0							
Locus_21397_Transcript_1/1_Conf_1.000	366	0							

Locus_21398_Transcript_1/1_Conf_1.000	156	0							
Locus_21399_Transcript_1/1_Conf_1.000	172	0							
Locus_214_Transcript_1/1_Conf_1.000	793	0							
Locus_2140_Transcript_1/1_Conf_1.000	1934	20	1.91E-96	XP_001900109.1	78	358.221	266	210	SPRY domain-containing SOCS box protein SSB-4
Locus_21400_Transcript_1/1_Conf_1.000	366	20	1.30E-46	NP_001122650.1	90	189.119	121	109	PaTChed family member (ptc-3)
Locus_21401_Transcript_1/1_Conf_1.000	306	20	7.74E-23	XP_002631676.1	74	110.153	100	74	Hypothetical protein CBG20869
Locus_21402_Transcript_1/1_Conf_1.000	363	3	8.52E-30	NP_499313.2	67	133.265	119	80	hypothetical protein F43D9.1
Locus_21403_Transcript_1/1_Conf_1.000	380	1	8.14E-04	NP_504527.2	61	46.9802	54	33	hypothetical protein F26F12.3
Locus_21404_Transcript_1/1_Conf_1.000	419	0							
Locus_21405_Transcript_1/1_Conf_1.000	505	20	2.19E-52	XP_002635088.1	79	208.379	156	124	C. briggsae CBR-ASP-6 protein
Locus_21406_Transcript_1/1_Conf_1.000	242	0							
Locus_21407_Transcript_1/1_Conf_1.000	293	1	1.49E-21	NP_498957.1	72	105.916	97	70	hypothetical protein F44E2.9
Locus_21408_Transcript_1/1_Conf_1.000	296	20	5.08E-30	NP_491653.2	85	134.035	92	79	hypothetical protein F55F8.3
Locus_21409_Transcript_1/1_Conf_1.000	129	3	1.29E-09	NP_508449.1	79	66.2402	43	34	hypothetical protein R04B3.3
Locus_2141_Transcript_1/1_Conf_1.000	309	0							
Locus_21410_Transcript_1/1_Conf_1.000	132	0							

Locus_21411_Transcript_1/1_Conf_1.000	171	20	6.14E-28	EAX10909.1	98	127.102	57	56	collagen, type III, alpha 1 (Ehlers-Danlos syndrome type IV, autosomal dominant), isoform CRA_a
Locus_21412_Transcript_1/1_Conf_1.000	254	0							
Locus_21413_Transcript_1/1_Conf_1.000	155	0							
Locus_21414_Transcript_1/1_Conf_1.000	422	2	4.20E-16	XP_002631299.1	67	87.8113	98	66	Hypothetical protein CBG03113
Locus_21415_Transcript_1/1_Conf_1.000	240	0							
Locus_21416_Transcript_1/1_Conf_1.000	339	0							
Locus_21417_Transcript_1/1_Conf_1.000	173	0							
Locus_21418_Transcript_1/1_Conf_1.000	231	0							
Locus_21419_Transcript_1/1_Conf_1.000	143	0							
Locus_2142_Transcript_1/2_Conf_1.000	1427	20	1.15E-52	AAD13340.1	48	212.231	450	218	ancylostoma secreted protein 1 precursor
Locus_2142_Transcript_2/2_Conf_1.000	322	1	4.80E-04	AAD13339.1	57	47.7506	83	48	ancylostoma-secreted protein 1 precursor
Locus_21420_Transcript_1/1_Conf_1.000	458	15	1.61E-20	NP_491122.2	59	102.449	119	71	hypothetical protein Y54E10A.17
Locus_21421_Transcript_1/1_Conf_1.000	243	0							
Locus_21422_Transcript_1/1_Conf_1.000	149	0							
Locus_21423_Transcript_1/1_Conf_1.000	171	7	9.85E-10	NP_501376.2	67	66.6254	55	37	hypothetical protein C07G1.2
Locus_21424_Transcript_1/1_Conf_1.000	191	20	4.81E-20	XP_002635377.1	82	100.908	63	52	Hypothetical protein CBG00768
Locus_21425_Transcript_1/1_Conf_1.000	2573	20	0	NP_497274.2	92	1009.59	591	545	hypothetical protein K02F3.2

Locus_21426_Transcript_1/1_Conf_1.000	135	0							
Locus_21427_Transcript_1/1_Conf_1.000	335	2	5.57E-13	XP_002630454.1	65	77.411	86	56	Hypothetical protein CBG11187
Locus_21428_Transcript_1/1_Conf_1.000	289	20	7.90E-31	NP_492594.2	83	136.732	95	79	hypothetical protein F26E4.5
Locus_21429_Transcript_1/1_Conf_1.000	307	7	1.32E-14	NP_001021268.1	71	82.8037	84	60	UNCoordinated family member (unc-44)
Locus_2143_Transcript_1/1_Conf_1.000	1279	20	0	EFO27018.1	94	720.694	408	387	hypothetical protein LOAG_01458
Locus_21430_Transcript_1/1_Conf_1.000	334	20	2.34E-11	AAN05752.1	65	72.0182	107	70	heat shock protein 20
Locus_21431_Transcript_1/1_Conf_1.000	407	0							
Locus_21432_Transcript_1/1_Conf_1.000	450	0							
Locus_21433_Transcript_1/1_Conf_1.000	341	0							
Locus_21434_Transcript_1/1_Conf_1.000	344	20	6.95E-19	CAO03461.1	61	97.0561	112	69	SET binding factor 1
Locus_21435_Transcript_1/1_Conf_1.000	166	20	3.51E-23	BAD92403.1	100	111.309	55	55	major histocompatibility complex, class II, DR alpha precursor variant
Locus_21436_Transcript_1/1_Conf_1.000	148	0							
Locus_21437_Transcript_1/1_Conf_1.000	171	7	2.05E-07	NP_001122684.1	90	58.9214	40	36	UNCoordinated family member (unc-103)
Locus_21438_Transcript_1/1_Conf_1.000	207	20	2.41E-32	XP_001899245.1	100	141.739	68	68	Dumpy : shorter than wild-type protein 23, isoform b

Locus_21439_Transcript_1/1_Conf_1.000	199	20	4.45E-26	AAV38526.1	100	120.939	58	58	proteasome (prosome, macropain) subunit, beta type, 3
Locus_2144_Transcript_1/3_Conf_0.714	1075	20	2.36E-86	EFO18208.1	88	323.553	199	177	hypothetical protein LOAG_10291
Locus_2144_Transcript_2/3_Conf_0.714	1079	20	2.37E-86	EFO18208.1	88	323.553	199	177	hypothetical protein LOAG_10291
Locus_2144_Transcript_3/3_Conf_0.714	1081	20	2.38E-86	EFO18208.1	88	323.553	199	177	hypothetical protein LOAG_10291
Locus_21440_Transcript_1/1_Conf_1.000	163	17	8.38E-25	BAF84240.1	100	116.701	54	54	unnamed protein product

Locus_21441_Transcript_1/1_Conf_1.000	237	15	6.00E-07	XP_002116347.1	60	57.3806	66	40	hypothetical protein TRIADDRAFT_64268
Locus_21442_Transcript_1/1_Conf_1.000	135	0							
Locus_21443_Transcript_1/1_Conf_1.000	138	0							
Locus_21444_Transcript_1/1_Conf_1.000	185	0							
Locus_21445_Transcript_1/1_Conf_1.000	314	0							
Locus_21446_Transcript_1/1_Conf_1.000	189	0							
Locus_21447_Transcript_1/1_Conf_1.000	355	20	6.85E-19	NP_001090394.1	64	97.0561	115	74	putative deoxyribonuclease tatdn3-A
Locus_21448_Transcript_1/1_Conf_1.000	140	19	2.95E-06	XP_001113618.2	100	55.0694	44	44	PREDICTED: CD82 antigen isoform 2
Locus_21449_Transcript_1/1_Conf_1.000	129	0							
Locus_2145_Transcript_1/1_Conf_1.000	456	0							
Locus_21450_Transcript_1/1_Conf_1.000	526	0							
Locus_21451_Transcript_1/1_Conf_1.000	140	0							
Locus_21452_Transcript_1/1_Conf_1.000	143	3	3.47E-07	NP_498645.1	66	58.151	48	32	MUscle Positioning family member (mup-4)

Locus_21453_Transcript_1/1_Conf_1.000	537	20	2.38E-69	XP_001894059.1	87	265.003	177	154	SMC family, C-terminal domain containing protein
Locus_21454_Transcript_1/1_Conf_1.000	202	20	1.87E-08	XP_001011065.1	54	62.3882	66	36	LIM domain containing protein
Locus_21455_Transcript_1/1_Conf_1.000	176	5	1.37E-11	NP_001022925.1	68	72.7886	58	40	hypothetical protein Y56A3A.36
Locus_21456_Transcript_1/1_Conf_1.000	176	0							
Locus_21457_Transcript_1/1_Conf_1.000	339	8	3.55E-44	ABS45067.1	87	181.03	113	99	latrophilin-like protein 2
Locus_21458_Transcript_1/1_Conf_1.000	175	0							
Locus_21459_Transcript_1/1_Conf_1.000	326	7	4.00E-43	XP_002646632.1	92	177.563	108	100	C. briggsae CBR-UNC-52 protein
Locus_2146_Transcript_1/1_Conf_1.000	1454	20	1.88E-42	XP_001895237.1	62	178.333	222	139	hypothetical protein
Locus_21460_Transcript_1/1_Conf_1.000	243	0							
Locus_21461_Transcript_1/1_Conf_1.000	230	0							
Locus_21462_Transcript_1/1_Conf_1.000	760	5	8.15E-73	XP_002645124.1	75	277.715	237	179	Hypothetical protein CBG16818
Locus_21463_Transcript_1/1_Conf_1.000	159	0							
Locus_21464_Transcript_1/1_Conf_1.000	257	5	7.71E-10	EFO23218.1	66	67.0106	59	39	hypothetical protein LOAG_05263
Locus_21465_Transcript_1/1_Conf_1.000	498	0							
Locus_21466_Transcript_1/1_Conf_1.000	323	0							
Locus_21467_Transcript_1/1_Conf_1.000	515	20	2.30E-28	XP_001899869.1	57	128.642	171	99	hypothetical protein Bm1_42035

Locus_21468_Transcript_1/1_Conf_1.000	165	2	1.25E-04	NP_509647.1	70	49.6766	51	36	hypothetical protein F47A4.5
Locus_21469_Transcript_1/1_Conf_1.000	133	0							
Locus_2147_Transcript_1/2_Conf_1.000	417	0							
Locus_2147_Transcript_2/2_Conf_1.000	413	0							
Locus_21470_Transcript_1/1_Conf_1.000	188	3	3.95E-22	XP_002633497.1	95	107.842	61	58	Hypothetical protein CBG06269
Locus_21471_Transcript_1/1_Conf_1.000	157	0							
Locus_21472_Transcript_1/1_Conf_1.000	242	0							
Locus_21473_Transcript_1/1_Conf_1.000	199	0							
Locus_21474_Transcript_1/1_Conf_1.000	321	3	1.10E-24	XP_002635291.1	67	116.316	105	71	Hypothetical protein CBG11538
Locus_21475_Transcript_1/1_Conf_1.000	271	0							
Locus_21476_Transcript_1/1_Conf_1.000	149	2	2.57E-05	NP_491716.1	78	51.9878	41	32	hypothetical protein B0207.7
Locus_21477_Transcript_1/1_Conf_1.000	182	0							
Locus_21478_Transcript_1/1_Conf_1.000	179	0							
Locus_21479_Transcript_1/1_Conf_1.000	149	0							
Locus_2148_Transcript_1/1_Conf_1.000	1485	20	2.19E-94	NP_496624.2	59	350.903	471	282	hypothetical protein Y57A10B.1
Locus_21480_Transcript_1/1_Conf_1.000	481	20	2.55E-34	XP_001893384.1	65	148.288	160	104	Plasmid Maintenance Protein containing protein
Locus_21481_Transcript_1/1_Conf_1.000	470	0							
Locus_21482_Transcript_1/1_Conf_1.000	151	0							
Locus_21483_Transcript_1/1_Conf_1.000	335	20	1.08E-32	NP_502386.1	74	142.895	111	83	hypothetical protein T04A11.2
Locus_21484_Transcript_1/1_Conf_1.000	518	20	6.17E-45	XP_002630506.1	70	183.726	167	118	C. briggsae CBR-HIM-14 protein
Locus_21485_Transcript_1/1_Conf_1.000	522	0							
Locus_21486_Transcript_1/1_Conf_1.000	475	2	1.47E-21	NP_502182.2	77	105.916	86	67	hypothetical protein F12F6.1
Locus_21487_Transcript_1/1_Conf_1.000	491	0							
Locus_21488_Transcript_1/1_Conf_1.000	314	1	7.70E-26	AAB02411.1	100	120.168	55	55	CoxII/D-loop DNA fusion protein

Locus_21489_Transcript_1/1_Conf_1.000	133	20	2.76E-20	3MQL	100	101.679	43	43	CrystalStructure OfThe Fibronectin 6fni1-2fnii7fni Fragment
Locus_2149_Transcript_1/1_Conf_1.000	644	7	6.66E-21	CBI83239.1	54	104.76	130	71	C. elegans protein W06F12.2e, confirmed by transcript evidence
Locus_21490_Transcript_1/1_Conf_1.000	154	1	1.66E-04	NP_001076620.1	60	49.2914	45	27	hypothetical protein Y34D9A.8
Locus_21491_Transcript_1/1_Conf_1.000	242	20	4.55E-31	NP_001021650.1	90	137.502	80	72	HaMmeRhead embryonic lethal family member (hmr-1)
Locus_21492_Transcript_1/1_Conf_1.000	775	0							
Locus_21493_Transcript_1/1_Conf_1.000	297	0							
Locus_21494_Transcript_1/1_Conf_1.000	377	4	5.81E-34	NP_493078.2	73	147.132	125	92	hypothetical protein R05D7.5
Locus_21495_Transcript_1/1_Conf_1.000	398	0							
Locus_21496_Transcript_1/1_Conf_1.000	194	0							
Locus_21497_Transcript_1/1_Conf_1.000	281	3	2.33E-11	NP_499961.2	65	72.0182	93	61	Nuclear Hormone Receptor family member (nhr-76)
Locus_21498_Transcript_1/1_Conf_1.000	170	0							
Locus_21499_Transcript_1/1_Conf_1.000	137	0							
Locus_215_Transcript_1/2_Conf_1.000	983	16	4.01E-58	CAR63537.1	67	229.565	202	137	putative Lipid Binding Protein
Locus_215_Transcript_2/2_Conf_1.000	854	20	1.74E-64	CAR63537.1	86	250.366	159	137	putative Lipid Binding Protein
Locus_2150_Transcript_1/1_Conf_1.000	342	20	5.13E-30	NP_494366.2	78	134.035	111	87	hypothetical protein F08D12.1
Locus_21500_Transcript_1/1_Conf_1.000	384	0							
Locus_21501_Transcript_1/1_Conf_1.000	137	0							
Locus_21502_Transcript_1/1_Conf_1.000	309	0							

Locus_21503_Transcript_1/1_Conf_1.000	328	20	3.28E-29	NP_496542.1	76	131.339	108	83	hypothetical protein ZK930.1
Locus_21504_Transcript_1/1_Conf_1.000	189	1	1.83E-11	NP_499637.2	77	72.4034	61	47	hypothetical protein Y111B2A.8
Locus_21505_Transcript_1/1_Conf_1.000	621	0							
Locus_21506_Transcript_1/1_Conf_1.000	213	4	3.15E-08	XP_002636889.1	62	61.6178	67	42	C. briggsae CBR-HEX-2 protein
Locus_21507_Transcript_1/1_Conf_1.000	271	0							
Locus_21508_Transcript_1/1_Conf_1.000	128	0							
Locus_21509_Transcript_1/1_Conf_1.000	168	0							
Locus_2151_Transcript_1/1_Conf_1.000	773	20	1.75E-86	XP_002640077.1	81	323.168	253	206	Hypothetical protein CBG12562
Locus_21510_Transcript_1/1_Conf_1.000	444	0							
Locus_21511_Transcript_1/1_Conf_1.000	134	0							
Locus_21512_Transcript_1/1_Conf_1.000	178	0							
Locus_21513_Transcript_1/1_Conf_1.000	129	0							
Locus_21514_Transcript_1/1_Conf_1.000	177	20	5.92E-15	NP_499784.2	84	83.9593	58	49	KELch-repeat containing protein family member (kel-3)
Locus_21515_Transcript_1/1_Conf_1.000	201	0							
Locus_21516_Transcript_1/1_Conf_1.000	227	0							
Locus_21517_Transcript_1/1_Conf_1.000	160	0							
Locus_21518_Transcript_1/1_Conf_1.000	137	11	1.46E-13	NP_871654.2	93	79.337	45	42	GEX Interacting protein family member (gei-4)
Locus_21519_Transcript_1/1_Conf_1.000	315	6	1.45E-16	AAV41897.1	69	89.3521	94	65	SAX-2
Locus_2152_Transcript_1/1_Conf_1.000	2478	20	0	NP_498283.1	77	983.786	817	632	hypothetical protein C28H8.3
Locus_21520_Transcript_1/1_Conf_1.000	209	0							
Locus_21521_Transcript_1/1_Conf_1.000	155	0							
Locus_21522_Transcript_1/1_Conf_1.000	219	0							

Locus_21523_Transcript_1/1_Conf_1.000	138	20	6.13E-12	EFO27870.1	89	73.9442	46	41	CMGC/CDK/CDK7 protein kinase
Locus_21524_Transcript_1/1_Conf_1.000	306	4	1.84E-24	NP_508923.1	77	115.546	98	76	hypothetical protein C41A3.1
Locus_21525_Transcript_1/1_Conf_1.000	273	20	7.79E-15	XP_001900299.1	60	83.5741	87	53	Protein-tyrosine phosphatase containing protein
Locus_21526_Transcript_1/1_Conf_1.000	554	0							
Locus_21527_Transcript_1/1_Conf_1.000	142	11	7.71E-15	ACJ65168.1	93	83.5741	44	41	Uncoordinated protein 68, isoform b, partially confirmed by transcript evidence
Locus_21528_Transcript_1/1_Conf_1.000	131	0							
Locus_21529_Transcript_1/1_Conf_1.000	180	0							
Locus_2153_Transcript_1/1_Conf_1.000	443	0							
Locus_21530_Transcript_1/1_Conf_1.000	356	2	3.30E-05	XP_001901347.1	55	51.6026	76	42	DNA excision repair protein ERCC-6
Locus_21531_Transcript_1/1_Conf_1.000	324	0							
Locus_21532_Transcript_1/1_Conf_1.000	264	0							
Locus_21533_Transcript_1/2_Conf_1.000	731	0							
Locus_21533_Transcript_2/2_Conf_1.000	731	0							
Locus_21534_Transcript_1/1_Conf_1.000	277	2	3.27E-05	EFO19241.1	53	51.6026	88	47	hypothetical protein LOAG_09252
Locus_21535_Transcript_1/1_Conf_1.000	173	0							
Locus_21536_Transcript_1/1_Conf_1.000	223	20	1.27E-20	XP_002645688.1	83	102.834	62	52	C. briggsae CBR-ELT-3 protein

Locus_21537_Transcript_1/1_Conf_1.000	170	17	1.74E-06	NP_001165114.1	70	55.8398	54	38	collagen
Locus_21538_Transcript_1/1_Conf_1.000	542	20	4.95E-30	XP_002636579.1	75	134.42	103	78	Hypothetical protein CBG23273
Locus_21539_Transcript_1/1_Conf_1.000	152	4	1.89E-16	EFO25124.1	89	88.9669	47	42	bromodomain containing protein
Locus_2154_Transcript_1/1_Conf_1.000	1064	20	7.66E-98	NP_494772.1	74	361.688	281	209	NOT-Like (yeast CCR4/NOT complex component) family member (ntl-2)
Locus_21540_Transcript_1/1_Conf_1.000	411	20	8.84E-27	XP_002114490.1	65	123.25	127	83	hypothetical protein TRIADDRAFT_27462
Locus_21541_Transcript_1/1_Conf_1.000	171	0							
Locus_21542_Transcript_1/1_Conf_1.000	645	1	1.61E-06	XP_002634576.1	60	56.9954	75	45	C. briggsae CBR-SRB-17 protein
Locus_21543_Transcript_1/1_Conf_1.000	332	1	4.15E-08	XP_002640024.1	66	61.2326	115	77	Hypothetical protein CBG12496
Locus_21544_Transcript_1/1_Conf_1.000	302	20	3.86E-30	NP_500647.2	78	134.42	99	78	Dipeptidyl Peptidase Four (IV) family member (dpf-5)
Locus_21545_Transcript_1/1_Conf_1.000	860	20	9.45E-26	XP_001893874.1	48	121.709	284	139	Smr domain containing protein
Locus_21546_Transcript_1/1_Conf_1.000	151	20	1.27E-20	XP_001107614.2	100	102.834	49	49	PREDICTED: inorganic pyrophosphatase-like
Locus_21547_Transcript_1/1_Conf_1.000	145	0							
Locus_21548_Transcript_1/1_Conf_1.000	323	20	7.11E-32	XP_002630378.1	77	140.198	109	84	Hypothetical protein CBG04317
Locus_21549_Transcript_1/1_Conf_1.000	143	0							
Locus_2155_Transcript_1/2_Conf_1.000	295	0							
Locus_2155_Transcript_2/2_Conf_1.000	293	0							
Locus_21550_Transcript_1/1_Conf_1.000	258	4	4.65E-15	XP_002646447.1	69	84.3445	73	51	Hypothetical protein CBG18852
Locus_21551_Transcript_1/1_Conf_1.000	151	0							
Locus_21552_Transcript_1/1_Conf_1.000	232	0							

Locus_21553_Transcript_1/1_Conf_1.000	139	0							
Locus_21554_Transcript_1/1_Conf_1.000	155	6	2.17E-04	XP_002648446.1	68	48.9062	45	31	Hypothetical protein CBG24721
Locus_21555_Transcript_1/1_Conf_1.000	132	0							
Locus_21556_Transcript_1/1_Conf_1.000	524	20	7.42E-25	XP_001894749.1	59	117.087	162	97	galactokinase family protein
Locus_21557_Transcript_1/1_Conf_1.000	187	0							
Locus_21558_Transcript_1/1_Conf_1.000	590	20	1.24E-78	XP_002633788.1	83	296.204	189	157	C. briggsae CBR-FAT-4 protein
Locus_21559_Transcript_1/1_Conf_1.000	231	0							
Locus_2156_Transcript_1/2_Conf_1.000	1078	12	6.52E-44	XP_002640218.1	51	182.57	276	142	C. briggsae CBR-ZTF-4 protein
Locus_2156_Transcript_2/2_Conf_1.000	336	0							
Locus_21560_Transcript_1/1_Conf_1.000	263	0							
Locus_21561_Transcript_1/1_Conf_1.000	171	0							
Locus_21562_Transcript_1/1_Conf_1.000	298	0							
Locus_21563_Transcript_1/1_Conf_1.000	187	20	8.79E-22	XP_001898118.1	94	106.686	59	56	FYVE finger-containing phosphoinositide kinase
Locus_21564_Transcript_1/1_Conf_1.000	193	0							
Locus_21565_Transcript_1/1_Conf_1.000	279	0							
Locus_21566_Transcript_1/1_Conf_1.000	129	0							
Locus_21567_Transcript_1/2_Conf_1.000	198	0							
Locus_21567_Transcript_2/2_Conf_1.000	272	0							
Locus_21568_Transcript_1/1_Conf_1.000	378	20	8.07E-36	XP_002642916.1	91	153.295	87	80	C. briggsae CBR-TAG-168 protein
Locus_21569_Transcript_1/1_Conf_1.000	489	2	8.70E-06	XP_002634144.1	64	53.5286	48	31	Hypothetical protein CBG01705
Locus_2157_Transcript_1/2_Conf_1.000	1640	20	0	NP_001021071.1	83	675.626	452	378	hypothetical protein D1007.5
Locus_2157_Transcript_2/2_Conf_1.000	1640	20	0	NP_001021071.1	83	675.626	452	378	hypothetical protein D1007.5

Locus_21570_Transcript_1/1_Conf_1.000	229	20	2.22E-17	EFO19956.1	77	92.0485	68	53	hypothetical protein LOAG_08535
Locus_21571_Transcript_1/1_Conf_1.000	497	20	1.77E-51	XP_002636314.1	81	205.297	166	135	Hypothetical protein CBG08607
Locus_21572_Transcript_1/1_Conf_1.000	158	0							
Locus_21573_Transcript_1/1_Conf_1.000	168	0							
Locus_21574_Transcript_1/1_Conf_1.000	149	2	2.66E-10	NP_497053.1	83	68.5514	48	40	hypothetical protein C09F9.2
Locus_21575_Transcript_1/1_Conf_1.000	178	0							
Locus_21576_Transcript_1/1_Conf_1.000	329	0							
Locus_21577_Transcript_1/1_Conf_1.000	318	0							
Locus_21578_Transcript_1/1_Conf_1.000	149	20	8.83E-14	XP_002920932.1	100	80.1073	35	35	PREDICTED: lipolysis-stimulated lipoprotein receptor-like
Locus_21579_Transcript_1/1_Conf_1.000	259	20	3.67E-20	NP_493023.1	80	101.293	67	54	hypothetical protein R09B3.3
Locus_2158_Transcript_1/2_Conf_1.000	581	12	9.31E-63	XP_002637005.1	87	243.432	166	146	Hypothetical protein CBG09497
Locus_2158_Transcript_2/2_Conf_1.000	581	12	9.31E-63	XP_002637005.1	87	243.432	166	146	Hypothetical protein CBG09497
Locus_21580_Transcript_1/1_Conf_1.000	132	0							
Locus_21581_Transcript_1/1_Conf_1.000	330	0							
Locus_21582_Transcript_1/1_Conf_1.000	485	20	6.91E-27	NP_491361.1	59	123.635	161	96	hypothetical protein ZK973.11
Locus_21583_Transcript_1/1_Conf_1.000	291	20	9.04E-11	XP_970144.1	63	70.0922	96	61	PREDICTED: similar to conserved hypothetical protein
Locus_21584_Transcript_1/1_Conf_1.000	426	12	3.35E-74	XP_002633317.1	95	280.796	141	134	Hypothetical protein CBG06054

Locus_21585_Transcript_1/1_Conf_1.000	184	0								
Locus_21586_Transcript_1/1_Conf_1.000	366	20	5.68E-34	XP_002639355.1	73	147.132	122	90	Hypothetical protein CBG03934
Locus_21587_Transcript_1/1_Conf_1.000	483	0							
Locus_21588_Transcript_1/1_Conf_1.000	130	1	2.13E-04	XP_002646581.1	77	48.9062	44	34	Hypothetical protein CBG20463
Locus_21589_Transcript_1/1_Conf_1.000	234	20	5.97E-39	BAG58796.1	100	163.696	78	78	unnamed protein product
Locus_2159_Transcript_1/1_Conf_1.000	1025	8	3.34E-18	XP_002637670.1	40	97.0561	360	145	C. briggsae CBR-RDE-1 protein
Locus_21590_Transcript_1/1_Conf_1.000	136	2	7.52E-10	NP_495168.1	84	67.0106	45	38	hypothetical protein H41C03.1
Locus_21591_Transcript_1/1_Conf_1.000	197	0							
Locus_21592_Transcript_1/1_Conf_1.000	220	20	7.99E-15	NP_494994.1	75	83.5741	73	55	hypothetical protein ZK622.1
Locus_21593_Transcript_1/1_Conf_1.000	150	0							
Locus_21594_Transcript_1/1_Conf_1.000	190	0							
Locus_21595_Transcript_1/1_Conf_1.000	132	0							
Locus_21596_Transcript_1/1_Conf_1.000	198	1	6.25E-04	XP_001915506.1	74	47.3654	35	26	PREDICTED: similar to alpha-2-glycoprotein 1, zinc-binding
Locus_21597_Transcript_1/1_Conf_1.000	605	20	8.23E-72	NP_503138.1	82	210.305	151	125	hypothetical protein W03F9.1
Locus_21598_Transcript_1/1_Conf_1.000	141	20	7.96E-20	BAD96655.1	100	100.138	46	46	transcription factor-like 1 variant
Locus_21599_Transcript_1/1_Conf_1.000	503	0							

Locus_216_Transcript_1/1_Conf_1.000	891	20	8.35E-81	XP_002636473.1	80	304.679	220	177	Hypothetical protein CBG23144
Locus_2160_Transcript_1/1_Conf_1.000	569	20	9.35E-49	NP_001040840.1	75	196.823	179	136	hypothetical protein C02D5.2
Locus_21600_Transcript_1/1_Conf_1.000	192	0							
Locus_21601_Transcript_1/1_Conf_1.000	504	20	7.77E-58	NP_501991.2	80	226.483	153	123	hypothetical protein T25B9.1
Locus_21602_Transcript_1/1_Conf_1.000	129	0							
Locus_21603_Transcript_1/1_Conf_1.000	1342	4	9.34E-25	XP_002645538.1	71	119.398	91	65	Hypothetical protein CBG22805
Locus_21604_Transcript_1/1_Conf_1.000	130	0							
Locus_21605_Transcript_1/1_Conf_1.000	803	20	2.03E-72	NP_509284.2	76	276.559	190	146	UNCoordinated family member (unc-98)
Locus_21606_Transcript_1/1_Conf_1.000	273	7	2.25E-38	XP_002633317.1	91	161.77	90	82	Hypothetical protein CBG06054
Locus_21607_Transcript_1/1_Conf_1.000	648	20	4.29E-15	NP_491348.1	88	85.5001	68	60	SCP(Small C-terminal domain Phosphatase)-Like phosphatase family member (scpl-3)
Locus_21608_Transcript_1/1_Conf_1.000	241	20	3.05E-27	NP_500943.1	82	124.79	76	63	hypothetical protein F58F9.7
Locus_21609_Transcript_1/1_Conf_1.000	520	20	1.31E-58	XP_002629752.1	86	229.18	145	126	Hypothetical protein CBG00986

Locus_2161_Transcript_1/2_Conf_1.000	568	20	6.35E-05	ZP_01908754.1	60	51.2174	70	42	putative transcriptional regulator
Locus_2161_Transcript_2/2_Conf_1.000	692	20	4.31E-11	XP_002191349.1	59	72.4034	110	65	PREDICTED: hypothetical protein, partial
Locus_21610_Transcript_1/1_Conf_1.000	322	0							
Locus_21611_Transcript_1/1_Conf_1.000	363	0							
Locus_21612_Transcript_1/1_Conf_1.000	188	0							
Locus_21613_Transcript_1/1_Conf_1.000	130	20	8.36E-09	XP_001899525.1	80	63.5438	42	34	C-terminal binding protein
Locus_21614_Transcript_1/1_Conf_1.000	180	1	5.94E-18	NP_510548.2	100	93.9745	43	43	hypothetical protein F39D8.3
Locus_21615_Transcript_1/1_Conf_1.000	284	20	6.32E-20	NP_501792.2	74	100.523	93	69	abnormal GONad development family member (gon-1)
Locus_21616_Transcript_1/1_Conf_1.000	156	20	4.79E-20	NP_001021901.1	98	100.908	51	50	CLC-type chloride cHannel family member (clh-2)
Locus_21617_Transcript_1/1_Conf_1.000	145	3	1.85E-11	NP_001021685.1	83	72.4034	48	40	ZO-1 (Zonula Occludens tight junctional protein) Ortholog family member (zoo-1)
Locus_21618_Transcript_1/1_Conf_1.000	354	20	1.87E-08	EFO14379.1	80	62.3882	42	34	hypothetical protein LOAG_14143
Locus_21619_Transcript_1/1_Conf_1.000	281	2	9.78E-10	NP_001032983.1	74	66.6254	78	58	hypothetical protein W05F2.4

Locus_2162_Transcript_1/1_Conf_1.000	1136	20	7.53E-62	NP_505962.2	60	242.276	336	202	Nuclear Pore complex Protein family member (npp-22)
Locus_21620_Transcript_1/1_Conf_1.000	168	0							
Locus_21621_Transcript_1/1_Conf_1.000	447	0							
Locus_21622_Transcript_1/1_Conf_1.000	242	20	4.00E-19	EFO22882.1	86	97.8265	61	53	TK/FER protein kinase
Locus_21623_Transcript_1/1_Conf_1.000	144	3	1.62E-07	XP_002639976.1	83	59.3066	48	40	C. briggsae CBR-KIN-32 protein
Locus_21624_Transcript_1/1_Conf_1.000	159	0							
Locus_21625_Transcript_1/1_Conf_1.000	132	0							
Locus_21626_Transcript_1/1_Conf_1.000	241	20	1.38E-11	XP_002630247.1	72	72.7886	79	57	Hypothetical protein CBG00666
Locus_21627_Transcript_1/1_Conf_1.000	195	0							
Locus_21628_Transcript_1/1_Conf_1.000	291	1	2.72E-15	NP_001076618.1	73	85.1149	76	56	hypothetical protein Y110A7A.21
Locus_21629_Transcript_1/1_Conf_1.000	603	20	5.72E-21	XP_002632199.1	52	104.76	180	95	C. briggsae CBR-NOL-1 protein
Locus_2163_Transcript_1/1_Conf_1.000	162	0							
Locus_21630_Transcript_1/1_Conf_1.000	267	20	2.36E-19	XP_002633705.1	80	98.5969	77	62	C. briggsae CBR-ECH-9 protein
Locus_21631_Transcript_1/1_Conf_1.000	157	0							
Locus_21632_Transcript_1/1_Conf_1.000	232	1	4.33E-05	NP_490917.3	45	51.2174	87	40	hypothetical protein Y20F4.2
Locus_21633_Transcript_1/1_Conf_1.000	141	20	2.25E-06	BAG60188.1	100	55.4546	46	46	unnamed protein product
Locus_21634_Transcript_1/1_Conf_1.000	289	0							
Locus_21635_Transcript_1/1_Conf_1.000	216	0							

Locus_21636_Transcript_1/1_Conf_1.000	392	9	1.07E-32	XP_002640798.1	75	142.895	131	99	C. briggsae CBR-NHR-121 protein
Locus_21637_Transcript_1/1_Conf_1.000	309	0							
Locus_21638_Transcript_1/1_Conf_1.000	195	20	6.44E-25	XP_002829151.1	100	117.087	53	53	PREDICTED: tubulin-folding cofactor B-like
Locus_21639_Transcript_1/1_Conf_1.000	206	20	3.07E-19	XP_002633255.1	82	98.2117	68	56	Hypothetical protein CBG05980
Locus_2164_Transcript_1/2_Conf_1.000	330	0							
Locus_2164_Transcript_2/2_Conf_1.000	273	0							
Locus_21640_Transcript_1/1_Conf_1.000	332	0							
Locus_21641_Transcript_1/1_Conf_1.000	295	20	3.78E-17	EFO25770.1	100	91.2781	61	61	hypothetical protein LOAG_02719
Locus_21642_Transcript_1/1_Conf_1.000	324	3	2.80E-04	XP_002128277.1	50	48.521	77	39	PREDICTED: hypothetical protein
Locus_21643_Transcript_1/1_Conf_1.000	322	0							
Locus_21644_Transcript_1/1_Conf_1.000	153	0							
Locus_21645_Transcript_1/1_Conf_1.000	162	0							
Locus_21646_Transcript_1/1_Conf_1.000	305	2	2.96E-14	NP_498845.2	78	81.6481	55	43	hypothetical protein R05D3.9
Locus_21647_Transcript_1/1_Conf_1.000	408	20	5.87E-71	XP_002705104.1	100	270.011	135	135	PREDICTED: ribosomal protein L9-like

Locus_21648_Transcript_1/1_Conf_1.000	353	20	4.62E-07	XP_001636445.1	45	57.7658	137	63	predicted protein
Locus_21649_Transcript_1/1_Conf_1.000	186	20	5.15E-22	XP_002639246.1	83	107.457	62	52	Hypothetical protein CBG03805
Locus_2165_Transcript_1/2_Conf_0.500	351	20	7.31E-37	NP_501862.1	67	156.762	141	95	GABA TransAminase family member (gta-1)
Locus_2165_Transcript_2/2_Conf_0.750	891	20	1.96E-130	NP_501862.1	87	469.544	286	251	GABA TransAminase family member (gta-1)
Locus_21650_Transcript_1/1_Conf_1.000	341	0							
Locus_21651_Transcript_1/1_Conf_1.000	267	0							
Locus_21652_Transcript_1/1_Conf_1.000	255	0							
Locus_21653_Transcript_1/1_Conf_1.000	153	0							
Locus_21654_Transcript_1/2_Conf_1.000	714	8	1.93E-09	AAT40504.2	50	67.0106	121	61	Polyprotein, putative
Locus_21654_Transcript_2/2_Conf_1.000	714	8	1.93E-09	AAT40504.2	50	67.0106	121	61	Polyprotein, putative
Locus_21655_Transcript_1/1_Conf_1.000	181	0							

Locus_21656_Transcript_1/1_Conf_1.000	240	20	7.80E-15	CAD44516.1	73	83.5741	78	57	VAB-10B protein
Locus_21657_Transcript_1/1_Conf_1.000	149	0							
Locus_21658_Transcript_1/1_Conf_1.000	579								
Locus_21659_Transcript_1/1_Conf_1.000	196	0							
Locus_2166_Transcript_1/1_Conf_1.000	696	5	7.03E-09	XP_002639759.1	47	65.0846	93	44	Hypothetical protein CBG02205
Locus_21660_Transcript_1/1_Conf_1.000	291	0							
Locus_21661_Transcript_1/1_Conf_1.000	175	0							
Locus_21662_Transcript_1/1_Conf_1.000	181	0							
Locus_21663_Transcript_1/1_Conf_1.000	219	20	5.36E-11	XP_002639380.1	65	70.8626	70	46	Hypothetical protein CBG03966
Locus_21664_Transcript_1/1_Conf_1.000	1792	20	1.42E-98	EFO26157.1	59	365.155	561	333	type III restriction enzyme
Locus_21665_Transcript_1/1_Conf_1.000	145	0							
Locus_21666_Transcript_1/1_Conf_1.000	983	20	4.08E-119	XP_002639292.1	76	432.18	338	258	C. briggsae CBR-NHR-62 protein
Locus_21667_Transcript_1/1_Conf_1.000	219	0							
Locus_21668_Transcript_1/1_Conf_1.000	298	0							
Locus_21669_Transcript_1/1_Conf_1.000	249	7	1.55E-23	NP_491915.2	83	112.464	77	64	hypothetical protein ZC581.9
Locus_2167_Transcript_1/1_Conf_1.000	1206	20	8.23E-54	EFO26832.1	70	215.698	179	126	hypothetical protein LOAG_01653

Locus_21670_Transcript_1/1_Conf_1.000	142	20	2.32E-19	XP_002923631.1	100	98.5969	47	47	PREDICTED: sodium/potassium-transporting ATPase subunit alpha-1-like isoform 2
Locus_21671_Transcript_1/1_Conf_1.000	338	0							
Locus_21672_Transcript_1/1_Conf_1.000	297	0							
Locus_21673_Transcript_1/1_Conf_1.000	140	0							
Locus_21674_Transcript_1/1_Conf_1.000	344	20	7.64E-42	ACT52846.1	84	173.326	112	95	GMP reductase
Locus_21675_Transcript_1/1_Conf_1.000	180	0							
Locus_21676_Transcript_1/1_Conf_1.000	156	2	2.63E-10	NP_492746.2	82	68.5514	50	41	Temporarily Assigned Gene name family member (tag-68)
Locus_21677_Transcript_1/1_Conf_1.000	446	0							
Locus_21678_Transcript_1/1_Conf_1.000	487	20	6.63E-38	NP_498761.1	68	160.229	166	114	DiCer Related family member (dcr-1)
Locus_21679_Transcript_1/1_Conf_1.000	360	0							
Locus_2168_Transcript_1/1_Conf_1.000	155	0							
Locus_21680_Transcript_1/1_Conf_1.000	140	7	8.01E-12	XP_002634930.1	91	73.559	46	42	Hypothetical protein CBG22529
Locus_21681_Transcript_1/1_Conf_1.000	224	0							
Locus_21682_Transcript_1/1_Conf_1.000	406	6	1.13E-13	NP_501855.1	61	79.7221	100	61	TransThyretin-Related family domain family member (ttr-54)

Locus_21683_Transcript_1/1_Conf_1.000	431	20	6.55E-46	EFO19591.1	92	186.808	107	99	hypothetical protein LOAG_08901
Locus_21684_Transcript_1/1_Conf_1.000	138	2	4.11E-08	XP_511489.2	79	61.2326	39	31	PREDICTED: similar to isolog of yeast sui1 and rice gos2; putative
Locus_21685_Transcript_1/1_Conf_1.000	156	20	3.43E-18	EFO22882.1	92	94.7449	52	48	TK/FER protein kinase
Locus_21686_Transcript_1/1_Conf_1.000	195	20	4.18E-24	BAJ21087.1	100	114.39	56	56	transcription elongation factor B (SIII), polypeptide 2
Locus_21687_Transcript_1/1_Conf_1.000	189	0							
Locus_21688_Transcript_1/1_Conf_1.000	182	20	9.78E-21	XP_002639101.1	88	103.219	60	53	Hypothetical protein CBG14920
Locus_21689_Transcript_1/1_Conf_1.000	370	20	2.82E-20	XP_001894671.1	66	101.679	113	75	Protein kinase domain containing protein
Locus_2169_Transcript_1/1_Conf_1.000	2527	20	0	XP_002630210.1	71	853.203	829	596	C. briggsae CBR-ZYG-9 protein
Locus_21690_Transcript_1/1_Conf_1.000	180	0							
Locus_21691_Transcript_1/1_Conf_1.000	693	20	3.25E-107	XP_001902629.1	87	391.734	230	201	Zinc finger, C2H2 type family protein

Locus_21692_Transcript_1/1_Conf_1.000	145	20	2.04E-10	NP_495516.2	85	68.9366	48	41	hypothetical protein F32A5.8
Locus_21693_Transcript_1/1_Conf_1.000	139	20	1.28E-17	XP_002750292.1	100	92.8189	46	46	PREDICTED: alpha-enolase isoform 1
Locus_21694_Transcript_1/1_Conf_1.000	360	0							
Locus_21695_Transcript_1/1_Conf_1.000	289	20	8.17E-28	NP_496501.2	77	126.716	94	73	hypothetical protein F13D12.9
Locus_21696_Transcript_1/1_Conf_1.000	128	0							
Locus_21697_Transcript_1/1_Conf_1.000	173	0							
Locus_21698_Transcript_1/1_Conf_1.000	403	20	3.89E-22	XP_002648413.1	76	107.842	77	59	Hypothetical protein CBG24676
Locus_21699_Transcript_1/1_Conf_1.000	174	20	2.11E-20	XP_002636607.1	90	102.064	53	48	C. briggsae CBR-DHS-20 protein
Locus_217_Transcript_1/2_Conf_1.000	195	2	1.35E-06	XP_002425519.1	64	56.225	64	41	conserved hypothetical protein
Locus_217_Transcript_2/2_Conf_1.000	223	4	4.36E-13	XP_002425519.1	68	77.7962	73	50	conserved hypothetical protein
Locus_2170_Transcript_1/1_Conf_1.000	320	0							
Locus_21700_Transcript_1/1_Conf_1.000	224	0							
Locus_21701_Transcript_1/1_Conf_1.000	334	15	9.46E-29	XP_002638532.1	84	129.798	92	78	Hypothetical protein CBG05566
Locus_21702_Transcript_1/1_Conf_1.000	701	20	6.89E-113	XP_002638687.1	90	410.609	233	212	Hypothetical protein CBG11882
Locus_21703_Transcript_1/1_Conf_1.000	154	0							
Locus_21704_Transcript_1/1_Conf_1.000	196	20	1.22E-15	XP_002630450.1	75	86.2705	64	48	C. briggsae CBR-ASM-1 protein
Locus_21705_Transcript_1/1_Conf_1.000	149	0							

Locus_21706_Transcript_1/1_Conf_1.000	274	0							
Locus_21707_Transcript_1/1_Conf_1.000	314	0							
Locus_21708_Transcript_1/1_Conf_1.000	264	0							
Locus_21709_Transcript_1/1_Conf_1.000	351	20	6.04E-07	ZP_04769408.1	49	57.3806	109	54	gamma-glutamyltransferase
Locus_2171_Transcript_1/1_Conf_1.000	990	20	1.12E-31	XP_002731938.1	51	141.739	281	146	PREDICTED: sestrin 1-like
Locus_21710_Transcript_1/1_Conf_1.000	132	5	1.09E-08	XP_001901288.1	80	63.1586	42	34	hypothetical protein Bm1_49115
Locus_21711_Transcript_1/1_Conf_1.000	217	0							
Locus_21712_Transcript_1/1_Conf_1.000	148	20	8.24E-20	XP_001099130.2	100	100.138	48	48	PREDICTED: collagen alpha-2(VI) chain-like isoform 3
Locus_21713_Transcript_1/1_Conf_1.000	345	5	1.54E-34	NP_492157.2	79	149.058	112	89	hypothetical protein D2005.6
Locus_21714_Transcript_1/1_Conf_1.000	377	20	1.62E-60	NP_506269.1	99	235.343	125	124	EATing: abnormal pharyngeal pumping family member (eat-6)
Locus_21715_Transcript_1/1_Conf_1.000	138	0							
Locus_21716_Transcript_1/1_Conf_1.000	172	0							
Locus_21717_Transcript_1/1_Conf_1.000	470	4	2.78E-36	A8Y3Q1.2	81	154.836	101	82	Putative 28S ribosomal protein S5
Locus_21718_Transcript_1/1_Conf_1.000	207	20	1.80E-11	XP_002632023.1	65	72.4034	63	41	Hypothetical protein CBG10312
Locus_21719_Transcript_1/1_Conf_1.000	183	0							

Locus_2172_Transcript_1/1_Conf_1.000	1676	20	0	AAX36933.1	100	877.856	432	432	eukaryotic translation elongation factor 1 alpha 1
Locus_21720_Transcript_1/1_Conf_1.000	175	0							
Locus_21721_Transcript_1/1_Conf_1.000	373	20	4.46E-34	XP_001898170.1	75	147.517	121	91	Ubiquitin-conjugating enzyme family protein
Locus_21722_Transcript_1/1_Conf_1.000	178	0							
Locus_21723_Transcript_1/1_Conf_1.000	320	4	5.86E-18	NP_495792.1	68	93.9745	95	65	hypothetical protein M05D6.6
Locus_21724_Transcript_1/1_Conf_1.000	288	0							
Locus_21725_Transcript_1/1_Conf_1.000	377	0							
Locus_21726_Transcript_1/1_Conf_1.000	201	0							
Locus_21727_Transcript_1/1_Conf_1.000	157	20	2.15E-20	XP_002822113.1	100	102.064	51	51	PREDICTED: eukaryotic translation initiation factor 3 subunit F-like isoform 3
Locus_21728_Transcript_1/1_Conf_1.000	433	20	4.24E-37	NP_502676.3	70	157.532	143	101	hypothetical protein Y37A1A.2
Locus_21729_Transcript_1/1_Conf_1.000	523	20	4.05E-39	XP_002631431.1	75	164.466	161	121	C. briggsae CBR-TAG-341 protein
Locus_2173_Transcript_1/2_Conf_1.000	456	20	2.40E-24	NP_741102.1	84	115.161	119	100	UBiquitin-Like family member (ubl-1)
Locus_2173_Transcript_2/2_Conf_1.000	144	0							
Locus_21730_Transcript_1/1_Conf_1.000	239	0							

Locus_21731_Transcript_1/1_Conf_1.000	145	1	1.28E-04	XP_002638613.1	86	49.6766	43	37	C. briggsae CBR-PGP-9 protein
Locus_21732_Transcript_1/1_Conf_1.000	231	0							
Locus_21733_Transcript_1/2_Conf_1.000	219	0							
Locus_21733_Transcript_2/2_Conf_1.000	219	0							
Locus_21734_Transcript_1/1_Conf_1.000	508	20	2.58E-32	XP_002640010.1	77	141.739	132	102	Hypothetical protein CBG12480
Locus_21735_Transcript_1/1_Conf_1.000	213	0							
Locus_21736_Transcript_1/1_Conf_1.000	421	13	5.11E-30	AAK31453.4	64	134.035	151	97	Hypothetical protein C25H3.8
Locus_21737_Transcript_1/1_Conf_1.000	411	0							
Locus_21738_Transcript_1/1_Conf_1.000	218	20	3.35E-10	XP_002647313.1	68	68.1662	66	45	Hypothetical protein CBG06354
Locus_21739_Transcript_1/1_Conf_1.000	147	20	4.68E-15	NP_500551.2	87	84.3445	49	43	hypothetical protein F55F10.1
Locus_2174_Transcript_1/1_Conf_1.000	877	20	4.01E-88	XP_002631317.1	78	328.946	233	183	Hypothetical protein CBG03139
Locus_21740_Transcript_1/1_Conf_1.000	299	20	1.16E-34	XP_002645875.1	82	149.443	99	82	Hypothetical protein CBG07619
Locus_21741_Transcript_1/1_Conf_1.000	230	3	2.38E-11	NP_494819.3	89	72.0182	47	42	abnormal DYe Filling family member (dyf-14)
Locus_21742_Transcript_1/1_Conf_1.000	223	0							
Locus_21743_Transcript_1/1_Conf_1.000	147	0							
Locus_21744_Transcript_1/1_Conf_1.000	257	2	6.75E-06	XP_001898661.1	61	53.9138	71	44	MGC88946 protein
Locus_21745_Transcript_1/1_Conf_1.000	410	0							

Locus_21746_Transcript_1/1_Conf_1.000	128	3	2.79E-04	NP_001129839.1	66	48.521	42	28	hypothetical protein R05D3.12
Locus_21747_Transcript_1/1_Conf_1.000	231	0							
Locus_21748_Transcript_1/1_Conf_1.000	420	0							
Locus_21749_Transcript_1/1_Conf_1.000	216	0							
Locus_2175_Transcript_1/1_Conf_1.000	798	20	4.59E-109	ADL62852.1	84	398.282	266	225	thioredoxin reductase 1
Locus_21750_Transcript_1/1_Conf_1.000	285	0							
Locus_21751_Transcript_1/1_Conf_1.000	170	0							
Locus_21752_Transcript_1/1_Conf_1.000	249	0							
Locus_21753_Transcript_1/1_Conf_1.000	175	19	7.50E-18	XP_002811165.1	100	93.5893	57	57	PREDICTED: MARCKS-related protein-like
Locus_21754_Transcript_1/1_Conf_1.000	161	0							
Locus_21755_Transcript_1/1_Conf_1.000	149	0							
Locus_21756_Transcript_1/1_Conf_1.000	130	0							
Locus_21757_Transcript_1/1_Conf_1.000	151	0							
Locus_21758_Transcript_1/1_Conf_1.000	233	4	2.70E-31	NP_492127.1	89	138.272	77	69	Low-density lipoprotein Receptor Related family member (lrp-1)
Locus_21759_Transcript_1/1_Conf_1.000	435	20	1.00E-46	XP_002632693.1	81	189.504	144	117	Hypothetical protein CBG21624
Locus_2176_Transcript_1/1_Conf_1.000	650	20	2.91E-88	NP_497035.3	86	328.561	216	186	Trehalose 6-Phosphate Synthase family member (tps-2)
Locus_21760_Transcript_1/1_Conf_1.000	203	0							
Locus_21761_Transcript_1/1_Conf_1.000	195	16	1.70E-09	NP_001021671.2	75	65.855	44	33	hypothetical protein W09G3.7
Locus_21762_Transcript_1/1_Conf_1.000	443	0							

Locus_21763_Transcript_1/1_Conf_1.000	268	20	8.34E-33	NP_496251.1	96	143.28	76	73	TropoNin C family member (tnc-2)
Locus_21764_Transcript_1/1_Conf_1.000	401	20	2.61E-34	NP_496813.1	77	148.288	131	101	hypothetical protein Y48C3A.3
Locus_21765_Transcript_1/1_Conf_1.000	228	0							
Locus_21766_Transcript_1/1_Conf_1.000	408	17	5.53E-45	NP_502746.1	84	183.726	133	113	hypothetical protein Y67A10A.9
Locus_21767_Transcript_1/1_Conf_1.000	271	20	8.09E-12	XP_002631859.1	60	73.559	87	53	Hypothetical protein CBG07809
Locus_21768_Transcript_1/1_Conf_1.000	347	20	2.64E-10	XP_969308.1	77	68.5514	49	38	PREDICTED: similar to required for meiotic nuclear division 5 homolog A
Locus_21769_Transcript_1/1_Conf_1.000	365	20	8.56E-14	EFO27901.1	64	80.1073	106	68	hypothetical protein LOAG_00581
Locus_2177_Transcript_1/1_Conf_1.000	415	4	4.08E-24	XP_002629793.1	77	114.39	95	74	Hypothetical protein CBG01035
Locus_21770_Transcript_1/1_Conf_1.000	129	4	5.07E-06	XP_002647771.1	85	54.299	42	36	C. briggsae CBR-PPH-5 protein
Locus_21771_Transcript_1/1_Conf_1.000	244	20	4.25E-21	AAK31453.4	83	104.375	71	59	Hypothetical protein C25H3.8
Locus_21772_Transcript_1/1_Conf_1.000	135	0							
Locus_21773_Transcript_1/1_Conf_1.000	148	20	2.40E-19	XP_001249905.2	100	98.5969	49	49	PREDICTED: endothelial differentiation-related factor 1
Locus_21774_Transcript_1/1_Conf_1.000	353	1	8.16E-04	NP_508782.2	61	46.9802	39	24	hypothetical protein C05E11.6
Locus_21775_Transcript_1/1_Conf_1.000	237	0							
Locus_21776_Transcript_1/1_Conf_1.000	409	0							
Locus_21777_Transcript_1/1_Conf_1.000	146	0							
Locus_21778_Transcript_1/1_Conf_1.000	281	0							
Locus_21779_Transcript_1/1_Conf_1.000	154	0							
Locus_2178_Transcript_1/1_Conf_1.000	899	20	1.26E-84	XP_002642931.1	69	317.39	293	204	C. briggsae CBR-ALH-11 protein

Locus_21780_Transcript_1/1_Conf_1.000	188	2	3.96E-06	NP_508797.3	71	54.6842	52	37	Nuclear Hormone Receptor family member (nhr-45)
Locus_21781_Transcript_1/3_Conf_0.667	456	0							
Locus_21781_Transcript_2/3_Conf_0.667	475	0							
Locus_21781_Transcript_3/3_Conf_0.667	469	0							
Locus_21782_Transcript_1/1_Conf_1.000	145	0							
Locus_21783_Transcript_1/1_Conf_1.000	246	1	6.15E-04	NP_001002222.1	75	47.3654	36	27	serine/threonine-protein kinase PAK 4
Locus_21784_Transcript_1/1_Conf_1.000	151	0							
Locus_21785_Transcript_1/1_Conf_1.000	268	0							
Locus_21786_Transcript_1/1_Conf_1.000	133	0							
Locus_21787_Transcript_1/1_Conf_1.000	201	2	2.61E-10	NP_001022224.1	76	68.5514	63	48	hypothetical protein H20J04.9
Locus_21788_Transcript_1/1_Conf_1.000	295	20	2.51E-45	XP_002646290.1	90	184.882	97	88	C. briggsae CBR-INX-13 protein
Locus_21789_Transcript_1/1_Conf_1.000	140	0							
Locus_2179_Transcript_1/2_Conf_1.000	808	20	6.61E-79	NP_502295.1	75	298.13	265	200	yeast MON (monensin-resistant) homolog family member (mon-2)
Locus_2179_Transcript_2/2_Conf_1.000	3007	20	0	NP_502295.1	72	1091.64	1027	743	yeast MON (monensin-resistant) homolog family member (mon-2)
Locus_21790_Transcript_1/1_Conf_1.000	438	4	9.73E-18	CAN99691.2	53	93.2041	162	87	C. elegans protein F54B3.1b, partially confirmed by transcript evidence
Locus_21791_Transcript_1/2_Conf_1.000	426	0							
Locus_21791_Transcript_2/2_Conf_1.000	426	0							
Locus_21792_Transcript_1/1_Conf_1.000	535	0							

Locus_21793_Transcript_1/1_Conf_1.000	179	20	2.83E-25	XP_002918048.1	100	118.242	59	59	PREDICTED: signal transducer and activator of transcription 1-like
Locus_21794_Transcript_1/1_Conf_1.000	277	0							
Locus_21795_Transcript_1/1_Conf_1.000	294	0							
Locus_21796_Transcript_1/1_Conf_1.000	259	20	2.82E-12	NP_495376.1	64	75.0998	77	50	EXOSome (multiexonuclease complex) component family member (exos-8)
Locus_21797_Transcript_1/1_Conf_1.000	135	0							
Locus_21798_Transcript_1/1_Conf_1.000	265	0							
Locus_21799_Transcript_1/1_Conf_1.000	350	0							
Locus_218_Transcript_1/2_Conf_1.000	3101	20	1.52E-157	EFO23451.1	66	561.992	580	388	hypothetical protein LOAG_05033
Locus_218_Transcript_2/2_Conf_1.000	3101	20	1.52E-157	EFO23451.1	66	561.992	580	388	hypothetical protein LOAG_05033
Locus_2180_Transcript_1/1_Conf_1.000	919	0							
Locus_21800_Transcript_1/1_Conf_1.000	335	0							
Locus_21801_Transcript_1/1_Conf_1.000	258	0							
Locus_21802_Transcript_1/1_Conf_1.000	157	1	2.55E-05	NP_497340.2	62	51.9878	50	31	CaDHerin family member (cdh-12)

Locus_21803_Transcript_1/1_Conf_1.000	207	20	3.26E-29	XP_002646095.1	95	131.339	68	65	C. briggsae CBR-HMR-1 protein
Locus_21804_Transcript_1/1_Conf_1.000	143	0							
Locus_21805_Transcript_1/1_Conf_1.000	366	12	3.95E-19	EFO24904.1	59	97.8265	122	73	hypothetical protein LOAG_03584
Locus_21806_Transcript_1/1_Conf_1.000	395	3	2.56E-13	EFO20675.1	78	78.5666	66	52	hypothetical protein LOAG_07811
Locus_21807_Transcript_1/1_Conf_1.000	194	5	2.55E-05	XP_002648112.1	63	51.9878	52	33	C. briggsae CBR-PQN-83 protein
Locus_21808_Transcript_1/1_Conf_1.000	228	0							
Locus_21809_Transcript_1/1_Conf_1.000	202	0							
Locus_2181_Transcript_1/1_Conf_1.000	531	0							
Locus_21810_Transcript_1/1_Conf_1.000	234	0							
Locus_21811_Transcript_1/1_Conf_1.000	157	0							
Locus_21812_Transcript_1/1_Conf_1.000	271	4	8.94E-11	XP_002642332.1	67	70.0922	61	41	Hypothetical protein CBG18327
Locus_21813_Transcript_1/1_Conf_1.000	220	20	5.53E-16	XP_001850222.1	78	87.4261	66	52	phosphatidylethanolamine-binding protein
Locus_21814_Transcript_1/1_Conf_1.000	151	0							
Locus_21815_Transcript_1/1_Conf_1.000	128	20	5.61E-13	YP_002725704.1	97	77.411	42	41	NADH dehydrogenase subunit 1
Locus_21816_Transcript_1/1_Conf_1.000	407	0							
Locus_21817_Transcript_1/1_Conf_1.000	169	2	7.83E-07	XP_001899881.1	67	56.9954	55	37	cytochrome P450
Locus_21818_Transcript_1/1_Conf_1.000	231	5	1.10E-16	XP_001900525.1	76	89.7373	75	57	Lung seven transmembrane receptor family protein
Locus_21819_Transcript_1/1_Conf_1.000	233	6	6.24E-12	NP_500144.2	73	73.9442	71	52	Erythroid-Like Transcription factor family member (elt-6)
Locus_2182_Transcript_1/1_Conf_1.000	333	0							
Locus_21820_Transcript_1/1_Conf_1.000	151	20	1.60E-15	XP_002647214.1	94	85.8853	50	47	Hypothetical protein CBG22398
Locus_21821_Transcript_1/1_Conf_1.000	149	19	1.90E-16	XP_002633114.1	85	88.9669	49	42	C. briggsae CBR-UNC-8 protein
Locus_21822_Transcript_1/1_Conf_1.000	218	7	2.40E-08	XP_002630441.1	59	62.003	72	43	Hypothetical protein CBG11172
Locus_21823_Transcript_1/1_Conf_1.000	239	0							

Locus_21824_Transcript_1/1_Conf_1.000	330	20	8.26E-49	CAA21622.4	94	196.438	110	104	C. elegans protein Y43F8C.12, partially confirmed by transcript evidence
Locus_21825_Transcript_1/1_Conf_1.000	257	0							
Locus_21826_Transcript_1/1_Conf_1.000	290	6	4.31E-45	NP_001022641.1	95	184.111	96	92	LEThal family member (let-805)
Locus_21827_Transcript_1/1_Conf_1.000	154	15	3.02E-14	NP_076949.1	100	81.6481	34	34	structure-specific endonuclease subunit SLX1 isoform 1
Locus_21828_Transcript_1/1_Conf_1.000	201	0							
Locus_21829_Transcript_1/1_Conf_1.000	137	1	1.02E-06	NP_501709.2	77	56.6102	44	34	Membrane Calcium ATPase family member (mca-1)
Locus_2183_Transcript_1/1_Conf_1.000	339	20	6.98E-16	AAD51334.1	66	87.0409	80	53	Kunitz type serine protease inhibitor
Locus_21830_Transcript_1/1_Conf_1.000	251	20	3.58E-12	EFO23283.1	71	74.7146	67	48	hypothetical protein LOAG_05201
Locus_21831_Transcript_1/1_Conf_1.000	438	20	1.95E-34	NP_499155.1	65	148.673	144	95	hypothetical protein T05G5.5
Locus_21832_Transcript_1/1_Conf_1.000	510	20	3.94E-49	CAA97764.2	76	197.593	160	122	C. elegans protein B0564.2, confirmed by transcript evidence
Locus_21833_Transcript_1/1_Conf_1.000	249	0							

Locus_21834_Transcript_1/1_Conf_1.000	208	20	8.34E-09	EFO17055.1	64	63.5438	70	45	thiamin pyrophosphokinase
Locus_21835_Transcript_1/1_Conf_1.000	299	4	5.82E-10	NP_496134.1	58	67.3958	96	56	hypothetical protein C08H9.1
Locus_21836_Transcript_1/1_Conf_1.000	146	0							
Locus_21837_Transcript_1/1_Conf_1.000	169	11	4.15E-08	NP_001024812.1	75	61.2326	37	28	hypothetical protein M163.8
Locus_21838_Transcript_1/1_Conf_1.000	184	20	2.40E-19	NP_503175.1	83	98.5969	61	51	ABC Transporter family member (abt-4)
Locus_21839_Transcript_1/1_Conf_1.000	339	0							
Locus_2184_Transcript_1/1_Conf_1.000	184	0							
Locus_21840_Transcript_1/1_Conf_1.000	275	0							
Locus_21841_Transcript_1/1_Conf_1.000	180	20	8.87E-14	NP_495410.4	81	80.1073	60	49	hypothetical protein C44B7.11
Locus_21842_Transcript_1/1_Conf_1.000	325	20	1.17E-18	Q21012.3	75	96.2857	93	70	Enolase-phosphatase E1
Locus_21843_Transcript_1/1_Conf_1.000	259	4	2.16E-12	NP_001040741.1	54	75.485	84	46	hypothetical protein C15F1.5
Locus_21844_Transcript_1/1_Conf_1.000	185	3	7.48E-13	NP_495697.2	94	77.0258	34	32	Lim and Transglutaminase Domain family member (ltd-1)
Locus_21845_Transcript_1/1_Conf_1.000	139	0							
Locus_21846_Transcript_1/1_Conf_1.000	273	0							
Locus_21847_Transcript_1/1_Conf_1.000	170	0							
Locus_21848_Transcript_1/1_Conf_1.000	240	0							
Locus_21849_Transcript_1/1_Conf_1.000	161	0							
Locus_2185_Transcript_1/4_Conf_0.667	2646	20	0	NP_499080.3	78	730.324	588	462	SR Protein Kinase family member (spk-1)
Locus_2185_Transcript_2/4_Conf_0.667	2736	20	0	NP_499080.3	76	729.169	617	471	SR Protein Kinase family member (spk-1)

Locus_2185_Transcript_3/4_Conf_0.667	2847	20	0	NP_001021134.1	73	744.577	671	495	SR Protein Kinase family member (spk-1)
Locus_2185_Transcript_4/4_Conf_0.667	2697	20	0	NP_499080.3	77	731.865	604	467	SR Protein Kinase family member (spk-1)
Locus_21850_Transcript_1/1_Conf_1.000	139	0							
Locus_21851_Transcript_1/1_Conf_1.000	138	20	7.49E-10	XP_001510147.1	80	67.0106	46	37	PREDICTED: hypothetical protein, partial
Locus_21852_Transcript_1/1_Conf_1.000	609	0							
Locus_21853_Transcript_1/1_Conf_1.000	348	20	1.88E-16	NP_502195.3	72	88.9669	77	56	hypothetical protein F58B3.4
Locus_21854_Transcript_1/1_Conf_1.000	277	0							
Locus_21855_Transcript_1/1_Conf_1.000	147	0							
Locus_21856_Transcript_1/1_Conf_1.000	554	20	6.06E-58	NP_498895.1	75	227.254	184	138	RNA HelicAse family member (rha-2)
Locus_21857_Transcript_1/1_Conf_1.000	172	0							
Locus_21858_Transcript_1/1_Conf_1.000	132	0							
Locus_21859_Transcript_1/1_Conf_1.000	142	0							
Locus_2186_Transcript_1/1_Conf_1.000	147	0							
Locus_21860_Transcript_1/1_Conf_1.000	199	0							
Locus_21861_Transcript_1/1_Conf_1.000	128	0							
Locus_21862_Transcript_1/1_Conf_1.000	446	0							
Locus_21863_Transcript_1/1_Conf_1.000	226	1	1.27E-04	EFO19311.1	55	49.6766	67	37	hypothetical protein LOAG_09182
Locus_21864_Transcript_1/1_Conf_1.000	361	0							
Locus_21865_Transcript_1/1_Conf_1.000	243	0							
Locus_21866_Transcript_1/1_Conf_1.000	180	0							

Locus_21867_Transcript_1/1_Conf_1.000	406	20	4.01E-19	NP_498361.1	64	97.8265	104	67	hypothetical protein T12A2.8
Locus_21868_Transcript_1/1_Conf_1.000	1355	20	5.05E-119	CBK19468.1	66	432.565	463	308	C. elegans protein R11A8.4b, partially confirmed by transcript evidence
Locus_21869_Transcript_1/1_Conf_1.000	278	0							
Locus_2187_Transcript_1/1_Conf_1.000	1325	20	1.81E-81	NP_493544.2	68	307.76	362	249	hypothetical protein Y105E8B.3
Locus_21870_Transcript_1/1_Conf_1.000	208	20	8.34E-09	XP_002644943.1	61	63.5438	67	41	Hypothetical protein CBG10898
Locus_21871_Transcript_1/1_Conf_1.000	130	0							
Locus_21872_Transcript_1/1_Conf_1.000	188	20	3.47E-10	EFO27462.1	61	68.1662	70	43	hypothetical protein LOAG_01021
Locus_21873_Transcript_1/1_Conf_1.000	218	4	1.72E-14	XP_002638865.1	80	82.4185	72	58	Hypothetical protein CBG22082
Locus_21874_Transcript_1/1_Conf_1.000	655	0							
Locus_21875_Transcript_1/1_Conf_1.000	201	16	6.21E-12	NP_501217.2	85	73.9442	67	57	hypothetical protein R05G6.10
Locus_21876_Transcript_1/1_Conf_1.000	199	0							
Locus_21877_Transcript_1/1_Conf_1.000	255	20	5.87E-26	NP_499922.1	85	120.553	76	65	Kinase, GLH-Binding family member (kgb-1)
Locus_21878_Transcript_1/1_Conf_1.000	184	0							
Locus_21879_Transcript_1/1_Conf_1.000	199	0							

Locus_2188_Transcript_1/3_Conf_0.600	566	20	7.31E-38	XP_002637185.1	68	160.614	163	112	Hypothetical protein CBG09704
Locus_2188_Transcript_2/3_Conf_0.600	603	20	4.65E-39	XP_002637185.1	69	164.851	163	113	Hypothetical protein CBG09704
Locus_2188_Transcript_3/3_Conf_0.600	603	20	8.78E-38	XP_002637185.1	68	160.614	163	112	Hypothetical protein CBG09704
Locus_21880_Transcript_1/1_Conf_1.000	166	0							
Locus_21881_Transcript_1/1_Conf_1.000	216	0							
Locus_21882_Transcript_1/1_Conf_1.000	132	0							
Locus_21883_Transcript_1/1_Conf_1.000	171	0							
Locus_21884_Transcript_1/1_Conf_1.000	143	0							
Locus_21885_Transcript_1/1_Conf_1.000	429	20	1.24E-36	XP_001901596.1	74	155.992	143	106	oxidoreductase, short chain dehydrogenase/reductase family protein
Locus_21886_Transcript_1/1_Conf_1.000	214	0							
Locus_21887_Transcript_1/1_Conf_1.000	137	0							
Locus_21888_Transcript_1/1_Conf_1.000	147	0							
Locus_21889_Transcript_1/1_Conf_1.000	228	0							
Locus_2189_Transcript_1/2_Conf_1.000	967	20	1.17E-70	Q95PP1.1	86	271.166	227	197	Aspartyl protease inhibitor
Locus_2189_Transcript_2/2_Conf_1.000	961	20	1.16E-70	Q95PP1.1	86	271.166	227	197	Aspartyl protease inhibitor
Locus_21890_Transcript_1/1_Conf_1.000	167	0							
Locus_21891_Transcript_1/1_Conf_1.000	136	0							
Locus_21892_Transcript_1/1_Conf_1.000	207	2	1.80E-11	XP_002633609.1	68	72.4034	67	46	Hypothetical protein CBG05491
Locus_21893_Transcript_1/2_Conf_1.000	292	0							
Locus_21893_Transcript_2/2_Conf_1.000	291	0							
Locus_21894_Transcript_1/1_Conf_1.000	462	20	1.18E-26	XP_002630318.1	62	122.865	115	72	Hypothetical protein CBG04241
Locus_21895_Transcript_1/1_Conf_1.000	443	0							
Locus_21896_Transcript_1/1_Conf_1.000	150	0							
Locus_21897_Transcript_1/1_Conf_1.000	134	0							
Locus_21898_Transcript_1/1_Conf_1.000	189	20	8.18E-28	XP_002750079.1	100	126.716	63	63	PREDICTED: ATP synthase subunit gamma, mitochondrial-like
Locus_21899_Transcript_1/1_Conf_1.000	134	0							
Locus_219_Transcript_1/1_Conf_1.000	593	20	8.20E-70	XP_002645843.1	78	266.929	201	158	C. briggsae CBR-TAG-53 protein
Locus_2190_Transcript_1/2_Conf_1.000	1427	20	3.74E-112	XP_002639282.1	82	409.838	338	279	C. briggsae CBR-NHR-69 protein

Locus_2190_Transcript_2/2_Conf_1.000	1427	20	3.74E-112	XP_002639282.1	82	409.838	338	279	C. briggsae CBR-NHR-69 protein
Locus_21900_Transcript_1/1_Conf_1.000	151	2	3.35E-05	XP_002641207.1	66	51.6026	50	33	Hypothetical protein CBG09068
Locus_21901_Transcript_1/1_Conf_1.000	970	20	3.70E-40	EFO23919.1	57	169.859	319	185	hypothetical protein LOAG_04568
Locus_21902_Transcript_1/1_Conf_1.000	277	20	2.50E-13	AAY99638.1	90	78.5666	51	46	mca-1
Locus_21903_Transcript_1/1_Conf_1.000	274	3	2.05E-07	ABD39381.1	72	58.9214	36	26	putative galectin protein 10
Locus_21904_Transcript_1/1_Conf_1.000	415	0							
Locus_21905_Transcript_1/1_Conf_1.000	554	0							
Locus_21906_Transcript_1/1_Conf_1.000	277	1	8.60E-06	EFN73640.1	85	53.5286	34	29	Probable phospholipid-transporting ATPase ID
Locus_21907_Transcript_1/1_Conf_1.000	421	5	1.75E-30	ACI49226.1	77	135.576	121	94	hypothetical protein Csp3_JD05.011
Locus_21908_Transcript_1/1_Conf_1.000	145	20	1.67E-12	EFN69115.1	87	75.8702	47	41	Peroxisome biogenesis factor 1
Locus_21909_Transcript_1/1_Conf_1.000	333	0							
Locus_2191_Transcript_1/1_Conf_1.000	1559	20	6.02E-143	XP_002634371.1	70	512.301	521	367	C. briggsae CBR-RIG-4 protein
Locus_21910_Transcript_1/1_Conf_1.000	141	0							
Locus_21911_Transcript_1/1_Conf_1.000	309	0							
Locus_21912_Transcript_1/1_Conf_1.000	185	0							
Locus_21913_Transcript_1/1_Conf_1.000	152	0							
Locus_21914_Transcript_1/1_Conf_1.000	209	0							
Locus_21915_Transcript_1/1_Conf_1.000	244	20	1.50E-42	ADN00778.1	97	175.637	81	79	LON-1 protein
Locus_21916_Transcript_1/1_Conf_1.000	226	0							

Locus_21917_Transcript_1/1_Conf_1.000	289	1	1.50E-05	NP_501999.2	48	52.7582	95	46	hypothetical protein T25B9.10
Locus_21918_Transcript_1/1_Conf_1.000	294	20	8.40E-25	XP_002644815.1	82	116.701	73	60	Hypothetical protein CBG14833
Locus_21919_Transcript_1/1_Conf_1.000	204	5	3.52E-07	NP_507251.2	56	58.151	67	38	Proline HYdroxylase family member (phy-3)
Locus_2192_Transcript_1/1_Conf_1.000	516	20	1.29E-42	NP_493258.1	80	176.022	131	105	ProFiliN family member (pfn-1)
Locus_21920_Transcript_1/1_Conf_1.000	166	20	4.45E-10	NP_498037.2	87	67.781	40	35	yeast ULA (ubiquitin activating) homolog family member (ula-1)
Locus_21921_Transcript_1/1_Conf_1.000	161	0							
Locus_21922_Transcript_1/1_Conf_1.000	416	0							
Locus_21923_Transcript_1/1_Conf_1.000	147	0							
Locus_21924_Transcript_1/1_Conf_1.000	372	3	2.71E-23	CAC35853.2	62	111.694	124	78	C. elegans protein Y111B2A.24, partially confirmed by transcript evidence
Locus_21925_Transcript_1/2_Conf_1.000	407	3	3.40E-10	NP_491390.2	52	68.1662	103	54	hypothetical protein D1007.15
Locus_21925_Transcript_2/2_Conf_1.000	407	3	4.02E-11	NP_491390.2	52	71.2478	106	56	hypothetical protein D1007.15
Locus_21926_Transcript_1/1_Conf_1.000	382	0							
Locus_21927_Transcript_1/1_Conf_1.000	188	0							
Locus_21928_Transcript_1/1_Conf_1.000	274	20	8.04E-20	XP_001896429.1	84	100.138	89	75	Hypothetical WD-repeat protein F21H12.1 in chromosome II, putative
Locus_21929_Transcript_1/1_Conf_1.000	248	0							
Locus_2193_Transcript_1/1_Conf_1.000	511	0							
Locus_21930_Transcript_1/1_Conf_1.000	318	20	4.48E-26	NP_491734.1	84	120.939	86	73	UNCoordinated family member (unc-94)
Locus_21931_Transcript_1/1_Conf_1.000	261	0							
Locus_21932_Transcript_1/1_Conf_1.000	165	0							
Locus_21933_Transcript_1/1_Conf_1.000	238	20	7.56E-18	XP_002632693.1	73	93.5893	78	57	Hypothetical protein CBG21624

Locus_21934_Transcript_1/1_Conf_1.000	272	20	8.09E-12	XP_002638675.1	63	73.559	83	53	C. briggsae CBR-PBRM-1 protein
Locus_21935_Transcript_1/1_Conf_1.000	259	20	4.95E-25	NP_499717.1	91	117.472	67	61	ABC transporter, class E family member (abce-1)
Locus_21936_Transcript_1/1_Conf_1.000	571	4	1.94E-09	XP_002642383.1	52	66.2402	130	68	Hypothetical protein CBG18387
Locus_21937_Transcript_1/1_Conf_1.000	231	0							
Locus_21938_Transcript_1/1_Conf_1.000	170	0							
Locus_21939_Transcript_1/1_Conf_1.000	240	0							
Locus_2194_Transcript_1/1_Conf_1.000	2311	0							
Locus_21940_Transcript_1/1_Conf_1.000	363	1	1.67E-17	NP_001041053.1	68	92.4337	92	63	hypothetical protein Y62E10A.20
Locus_21941_Transcript_1/1_Conf_1.000	412	0							
Locus_21942_Transcript_1/1_Conf_1.000	154	0							
Locus_21943_Transcript_1/1_Conf_1.000	194	0							
Locus_21944_Transcript_1/1_Conf_1.000	360	12	3.36E-26	XP_002639929.1	82	121.324	106	87	Hypothetical protein CBG08265
Locus_21945_Transcript_1/1_Conf_1.000	312	20	8.78E-30	ACV73031.1	94	133.265	71	67	AHA-1

Locus_21946_Transcript_1/1_Conf_1.000	254	15	4.09E-19	XP_001898877.1	79	97.8265	84	67	oxidoreductase, short chain dehydrogenase/reductase family protein
Locus_21947_Transcript_1/1_Conf_1.000	206	0							
Locus_21948_Transcript_1/1_Conf_1.000	429	20	3.18E-08	EFO25642.1	56	61.6178	85	48	hypothetical protein LOAG_02841
Locus_21949_Transcript_1/1_Conf_1.000	304	0							
Locus_2195_Transcript_1/1_Conf_1.000	823	20	1.03E-119	XP_002636728.1	86	433.721	268	231	C. briggsae CBR-FRM-7 protein
Locus_21950_Transcript_1/1_Conf_1.000	303	0							
Locus_21951_Transcript_1/1_Conf_1.000	263	0							
Locus_21952_Transcript_1/1_Conf_1.000	311	20	1.82E-48	NP_001033411.1	96	195.282	102	98	hypothetical protein F53H1.1
Locus_21953_Transcript_1/1_Conf_1.000	207	0							
Locus_21954_Transcript_1/2_Conf_1.000	409	0							
Locus_21954_Transcript_2/2_Conf_1.000	409	0							
Locus_21955_Transcript_1/1_Conf_1.000	315	20	6.97E-11	NP_491270.2	51	70.4774	101	52	Low-density lipoprotein Receptor Related family member (lrp-2)
Locus_21956_Transcript_1/1_Conf_1.000	254	0							
Locus_21957_Transcript_1/1_Conf_1.000	602	1	2.08E-07	NP_491543.1	51	59.6918	133	68	FANCM (Fanconi anemia complex component M) homolog family member (fncm-1)
Locus_21958_Transcript_1/1_Conf_1.000	158	1	2.16E-04	XP_001842842.1	62	48.9062	54	34	plasma glutamate carboxypeptidase
Locus_21959_Transcript_1/1_Conf_1.000	281	0							
Locus_2196_Transcript_1/2_Conf_1.000	1525	20	1.89E-141	EFO24230.1	67	507.294	527	358	hypothetical protein LOAG_04252
Locus_2196_Transcript_2/2_Conf_1.000	1525	20	1.89E-141	EFO24230.1	67	507.294	527	358	hypothetical protein LOAG_04252
Locus_21960_Transcript_1/1_Conf_1.000	137	0							
Locus_21961_Transcript_1/1_Conf_1.000	190	1	6.48E-25	XP_002751331.1	90	117.087	62	56	PREDICTED: stathmin-like
Locus_21962_Transcript_1/1_Conf_1.000	195	2	1.03E-06	XP_002641391.1	67	56.6102	62	42	C. briggsae CBR-GLY-8 protein
Locus_21963_Transcript_1/1_Conf_1.000	209	2	3.36E-34	EAW76268.1	100	147.902	69	69	hCG38498, isoform CRA_b
Locus_21964_Transcript_1/1_Conf_1.000	174	0							
Locus_21965_Transcript_1/1_Conf_1.000	137	0							
Locus_21966_Transcript_1/1_Conf_1.000	130	0							
Locus_21967_Transcript_1/1_Conf_1.000	514	0							

Locus_21968_Transcript_1/1_Conf_1.000	170	20	8.92E-19	NP_494991.1	89	96.6709	56	50	Phosphoethanolamine MethyTransferase family member (pmt-1)
Locus_21969_Transcript_1/1_Conf_1.000	176	0							
Locus_2197_Transcript_1/1_Conf_1.000	1071	20	7.66E-146	NP_493279.1	85	521.161	356	303	Suppressor of Mec and Unc defects family member (smu-1)
Locus_21970_Transcript_1/1_Conf_1.000	289	14	5.69E-13	EFO20293.1	65	77.411	89	58	PDZ domain-containing protein
Locus_21971_Transcript_1/1_Conf_1.000	197	0							
Locus_21972_Transcript_1/1_Conf_1.000	392	2	1.27E-33	NP_495168.1	77	145.976	117	91	hypothetical protein H41C03.1
Locus_21973_Transcript_1/1_Conf_1.000	142	3	9.13E-08	NP_497765.2	78	60.077	42	33	Adenylyl CYclase family member (acy-3)
Locus_21974_Transcript_1/1_Conf_1.000	330	20	1.13E-13	NP_496307.1	60	79.7221	103	62	hypothetical protein C09G5.2
Locus_21975_Transcript_1/1_Conf_1.000	214	20	4.25E-13	XP_002634303.1	82	77.7962	62	51	C. briggsae CBR-NCX-3 protein
Locus_21976_Transcript_1/1_Conf_1.000	342	6	6.95E-19	XP_002631746.1	61	97.0561	111	68	C. briggsae CBR-TAG-280 protein
Locus_21977_Transcript_1/1_Conf_1.000	456	0							
Locus_21978_Transcript_1/1_Conf_1.000	125	0							
Locus_21979_Transcript_1/1_Conf_1.000	262	0							
Locus_2198_Transcript_1/2_Conf_1.000	899	20	6.46E-89	EFO20496.1	73	331.643	304	223	hypothetical protein LOAG_07994
Locus_2198_Transcript_2/2_Conf_1.000	899	20	6.46E-89	EFO20496.1	73	331.643	304	223	hypothetical protein LOAG_07994
Locus_21980_Transcript_1/1_Conf_1.000	168	0							
Locus_21981_Transcript_1/1_Conf_1.000	579	20	2.73E-38	NP_001022032.1	67	162.155	187	126	TRRAP-like (transcription/transformation domain-associated protein) family member (trr-1)
Locus_21982_Transcript_1/1_Conf_1.000	129	0							
Locus_21983_Transcript_1/1_Conf_1.000	132	5	9.85E-10	NP_500647.2	82	66.6254	41	34	Dipeptidyl Peptidase Four (IV) family member (dpf-5)
Locus_21984_Transcript_1/1_Conf_1.000	200	4	9.31E-08	XP_001900237.1	64	60.077	64	41	mucosa associated lymphoid tissue lymphoma translocation protein 1, isoform b
Locus_21985_Transcript_1/1_Conf_1.000	540	0							
Locus_21986_Transcript_1/1_Conf_1.000	186	1	1.67E-04	XP_002632203.1	71	49.2914	45	32	Hypothetical protein CBG07070
Locus_21987_Transcript_1/1_Conf_1.000	499	5	1.72E-22	XP_002642061.1	55	108.997	167	93	Hypothetical protein CBG17999
Locus_21988_Transcript_1/1_Conf_1.000	389	0							
Locus_21989_Transcript_1/1_Conf_1.000	143	0							
Locus_2199_Transcript_1/6_Conf_0.357	409	20	2.95E-30	NP_497777.1	72	134.806	124	90	hypothetical protein C36A4.4
Locus_2199_Transcript_2/6_Conf_0.500	1473	20	4.92E-139	NP_497777.1	71	499.204	454	324	hypothetical protein C36A4.4

Locus_2199_Transcript_3/6_Conf_0.429	1181	20	2.43E-119	NP_497777.1	74	433.335	364	270	hypothetical protein C36A4.4
Locus_2199_Transcript_4/6_Conf_0.500	1473	20	4.16E-138	NP_497777.1	71	496.123	454	323	hypothetical protein C36A4.4
Locus_2199_Transcript_5/6_Conf_0.429	1188	20	1.59E-118	NP_497777.1	74	430.639	360	268	hypothetical protein C36A4.4
Locus_2199_Transcript_6/6_Conf_0.429	1181	20	5.41E-119	NP_497777.1	74	432.18	364	270	hypothetical protein C36A4.4
Locus_21990_Transcript_1/1_Conf_1.000	139	0							
Locus_21991_Transcript_1/1_Conf_1.000	279	0							
Locus_21992_Transcript_1/1_Conf_1.000	361	20	6.78E-27	XP_001896664.1	69	123.635	119	83	Protein kinase domain containing protein
Locus_21993_Transcript_1/1_Conf_1.000	400	0							
Locus_21994_Transcript_1/1_Conf_1.000	252	19	4.38E-13	XP_002640231.1	75	77.7962	52	39	Hypothetical protein CBG12746
Locus_21995_Transcript_1/1_Conf_1.000	129	0							
Locus_21996_Transcript_1/1_Conf_1.000	176	20	8.29E-17	ACI49101.1	88	90.1225	54	48	hypothetical protein Cbre_JD13.003
Locus_21997_Transcript_1/1_Conf_1.000	300	20	2.77E-28	NP_496066.2	90	128.257	86	78	Nuclear Hormone Receptor family member (nhr-19)
Locus_21998_Transcript_1/1_Conf_1.000	206	0							

Locus_21999_Transcript_1/1_Conf_1.000	151	5	3.96E-06	ACI49239.1	85	54.6842	35	30	hypothetical protein Csp3_JD06.005
Locus_22_Transcript_1/1_Conf_1.000	2388	16	6.91E-06	AAH84352.1	35	57.7658	562	197	LOC495149 protein
Locus_220_Transcript_1/2_Conf_1.000	1383	20	7.76E-123	CAJ45481.1	67	445.277	424	286	legumain
Locus_220_Transcript_2/2_Conf_1.000	1059	20	6.90E-99	XP_002636781.1	69	365.155	332	231	Hypothetical protein CBG23516
Locus_2200_Transcript_1/2_Conf_1.000	523	20	3.08E-63	NP_500191.3	86	244.588	175	151	ParaPleGiN AAA protease family member (ppgn-1)
Locus_2200_Transcript_2/2_Conf_1.000	523	20	3.08E-63	NP_500191.3	86	244.588	175	151	ParaPleGiN AAA protease family member (ppgn-1)

Locus_22000_Transcript_1/1_Conf_1.000	450	1	5.25E-11	CAR63594.1	55	70.8626	127	71	hypothetical protein
Locus_22001_Transcript_1/1_Conf_1.000	166	0							
Locus_22002_Transcript_1/1_Conf_1.000	302	20	6.79E-43	EFO27430.1	95	176.792	93	89	GTP-binding protein GTR1
Locus_22003_Transcript_1/1_Conf_1.000	256	3	6.97E-11	AAF82410.1	77	70.4774	53	41	AF221132_1diacylglycerol acyltransferase
Locus_22004_Transcript_1/1_Conf_1.000	145	2	9.81E-05	XP_002635479.1	73	50.0618	45	33	C. briggsae CBR-PMT-2 protein
Locus_22005_Transcript_1/1_Conf_1.000	132	0							
Locus_22006_Transcript_1/1_Conf_1.000	228	14	5.14E-06	EFN62076.1	68	54.299	38	26	hypothetical protein EAG_04210
Locus_22007_Transcript_1/1_Conf_1.000	325	0							
Locus_22008_Transcript_1/1_Conf_1.000	212	0							
Locus_22009_Transcript_1/1_Conf_1.000	294	0							
Locus_2201_Transcript_1/1_Conf_1.000	275	0							
Locus_22010_Transcript_1/1_Conf_1.000	160	20	5.10E-22	XP_002913146.1	100	107.457	53	53	PREDICTED: transmembrane emp24 domain-containing protein 2-like isoform 1
Locus_22011_Transcript_1/1_Conf_1.000	340	20	5.33E-24	XP_001899060.1	66	114.005	112	75	acetyltransferase, GNAT family protein
Locus_22012_Transcript_1/1_Conf_1.000	132	0							
Locus_22013_Transcript_1/1_Conf_1.000	151	8	9.40E-16	CAB00107.2	85	86.6557	49	42	C. elegans protein F58H1.4, confirmed by transcript evidence

Locus_22014_Transcript_1/1_Conf_1.000	233	0							
Locus_22015_Transcript_1/1_Conf_1.000	144	0							
Locus_22016_Transcript_1/1_Conf_1.000	343	20	3.09E-59	ABU93237.1	97	231.106	114	111	ligand-gated ion channel subunit
Locus_22017_Transcript_1/1_Conf_1.000	138	0							
Locus_22018_Transcript_1/1_Conf_1.000	373	0							
Locus_22019_Transcript_1/1_Conf_1.000	197	20	1.64E-20	BAG65407.1	100	102.449	50	50	unnamed protein product
Locus_2202_Transcript_1/1_Conf_1.000	341	0							
Locus_22020_Transcript_1/1_Conf_1.000	146	0							
Locus_22021_Transcript_1/1_Conf_1.000	380	20	9.93E-10	NP_495244.1	77	66.6254	48	37	Transbilayer Amphipath Transporters (subfamily IV P-type ATPase) family member (tat-4)
Locus_22022_Transcript_1/1_Conf_1.000	349	20	8.67E-38	AAK62032.1	74	159.844	116	86	metalloprotease 1 precursor
Locus_22023_Transcript_1/1_Conf_1.000	217	0							
Locus_22024_Transcript_1/1_Conf_1.000	206	4	4.00E-19	NP_504292.2	89	97.8265	68	61	hypothetical protein C37H5.5
Locus_22025_Transcript_1/1_Conf_1.000	160	20	3.54E-15	XP_001896329.1	90	84.7297	53	48	calcium ATPase
Locus_22026_Transcript_1/1_Conf_1.000	312	7	1.96E-21	NP_001021268.1	87	105.531	78	68	UNCoordinated family member (unc-44)

Locus_22027_Transcript_1/1_Conf_1.000	234	3	9.93E-10	A8X3A7.2	85	66.6254	75	64	Integrin alpha pat-2
Locus_22028_Transcript_1/1_Conf_1.000	147	0							
Locus_22029_Transcript_1/1_Conf_1.000	235	20	4.44E-26	NP_498128.1	96	120.939	65	63	CLocK (biological timing) abnormality family member (clk-1)
Locus_2203_Transcript_1/1_Conf_1.000	854	20	2.54E-132	XP_002634111.1	94	475.707	280	264	Hypothetical protein CBG01664
Locus_22030_Transcript_1/1_Conf_1.000	353	0							
Locus_22031_Transcript_1/1_Conf_1.000	267	20	4.72E-44	XP_002808235.1	100	180.644	88	88	PREDICTED: LOW QUALITY PROTEIN: calpain small subunit 1-like
Locus_22032_Transcript_1/1_Conf_1.000	144	0							
Locus_22033_Transcript_1/1_Conf_1.000	165	20	1.69E-17	EFO18251.1	87	92.4337	55	48	hypothetical protein LOAG_10243
Locus_22034_Transcript_1/1_Conf_1.000	164	20	2.06E-23	XP_002799431.1	100	112.079	54	54	PREDICTED: pyruvate kinase isozymes M1/M2 isoform 2

Locus_22035_Transcript_1/1_Conf_1.000	154	20	3.81E-17	EFO24654.1	92	91.2781	51	47	RNA polymerase Rpb1
Locus_22036_Transcript_1/1_Conf_1.000	176	0							
Locus_22037_Transcript_1/1_Conf_1.000	156	3	4.20E-08	NP_001022683.1	67	61.2326	55	37	hypothetical protein M01A8.2
Locus_22038_Transcript_1/1_Conf_1.000	418	20	5.64E-45	NP_001023958.1	85	183.726	124	106	Heat Shock Protein family member (hsp-17)
Locus_22039_Transcript_1/1_Conf_1.000	439	0							
Locus_2204_Transcript_1/1_Conf_1.000	681	20	1.42E-59	EFO25906.1	68	233.417	206	142	steroid hormone receptor
Locus_22040_Transcript_1/1_Conf_1.000	396	4	5.87E-10	EFO16079.1	55	67.3958	129	72	hypothetical protein LOAG_12429
Locus_22041_Transcript_1/1_Conf_1.000	199	0							
Locus_22042_Transcript_1/1_Conf_1.000	164	20	1.58E-23	NP_498711.1	96	112.464	54	52	TYRosinase family member (tyr-1)
Locus_22043_Transcript_1/1_Conf_1.000	478	0							
Locus_22044_Transcript_1/1_Conf_1.000	215	0							
Locus_22045_Transcript_1/1_Conf_1.000	145	0							
Locus_22046_Transcript_1/1_Conf_1.000	323	0							
Locus_22047_Transcript_1/1_Conf_1.000	132	2	8.63E-06	NP_491776.1	75	53.5286	44	33	hypothetical protein T09B4.7
Locus_22048_Transcript_1/1_Conf_1.000	345	4	2.23E-09	EFO23701.1	55	65.4698	103	57	TPR Domain containing protein
Locus_22049_Transcript_1/1_Conf_1.000	325	1	7.41E-16	CAR63646.1	80	84.7297	46	37	putative Homeobox family member
Locus_2205_Transcript_1/1_Conf_1.000	491	5	1.58E-23	CAB04203.2	62	112.464	156	98	C. elegans protein F26H9.2, confirmed by transcript evidence
Locus_22050_Transcript_1/1_Conf_1.000	172	0							
Locus_22051_Transcript_1/1_Conf_1.000	976	1	9.61E-04	CBI61474.1	45	48.9062	150	68	unnamed protein product
Locus_22052_Transcript_1/1_Conf_1.000	142	1	8.55E-06	EFO27222.1	72	53.5286	47	34	hypothetical protein LOAG_01266
Locus_22053_Transcript_1/1_Conf_1.000	455	20	1.56E-39	XP_001896270.1	66	165.622	149	99	Zinc finger, C2H2 type family protein
Locus_22054_Transcript_1/1_Conf_1.000	298	20	4.31E-13	EFO28436.1	54	77.7962	92	50	zinc finger protein
Locus_22055_Transcript_1/1_Conf_1.000	313	0							

Locus_22056_Transcript_1/1_Conf_1.000	352	20	1.94E-13	XP_002637085.1	77	78.9518	58	45	Hypothetical protein CBG09584
Locus_22057_Transcript_1/1_Conf_1.000	505	0							
Locus_22058_Transcript_1/1_Conf_1.000	139	0							
Locus_22059_Transcript_1/1_Conf_1.000	164	0							
Locus_2206_Transcript_1/1_Conf_1.000	345	20	5.12E-22	XP_002630467.1	86	107.457	74	64	C. briggsae CBR-UNC-104 protein
Locus_22060_Transcript_1/1_Conf_1.000	129	0							
Locus_22061_Transcript_1/1_Conf_1.000	405	0							
Locus_22062_Transcript_1/1_Conf_1.000	432	20	4.84E-41	NP_504578.1	76	170.629	131	100	hypothetical protein F17A9.4
Locus_22063_Transcript_1/1_Conf_1.000	565	0							
Locus_22064_Transcript_1/1_Conf_1.000	240	0							
Locus_22065_Transcript_1/1_Conf_1.000	129	0							
Locus_22066_Transcript_1/1_Conf_1.000	236	3	4.58E-31	NP_500132.3	87	137.502	78	68	hypothetical protein Y41D4A.7
Locus_22067_Transcript_1/1_Conf_1.000	321	20	3.31E-21	NP_593736.1	70	104.76	105	74	histone acetyltransferase Mst2
Locus_22068_Transcript_1/1_Conf_1.000	232	0							
Locus_22069_Transcript_1/1_Conf_1.000	234	20	8.41E-09	NP_504512.1	71	63.5438	74	53	hypothetical protein F44E7.2
Locus_2207_Transcript_1/2_Conf_1.000	1526	3	5.54E-08	NP_492411.1	46	63.929	167	77	GEX Interacting protein family member (gei-11)
Locus_2207_Transcript_2/2_Conf_1.000	2627	3	1.07E-07	NP_492411.1	46	63.929	167	77	GEX Interacting protein family member (gei-11)
Locus_22070_Transcript_1/1_Conf_1.000	214	20	4.09E-24	XP_002646410.1	91	114.39	67	61	Hypothetical protein CBG18783
Locus_22071_Transcript_1/1_Conf_1.000	481	0							
Locus_22072_Transcript_1/1_Conf_1.000	146	0							
Locus_22073_Transcript_1/1_Conf_1.000	623	0							

Locus_22074_Transcript_1/1_Conf_1.000	492	0							
Locus_22075_Transcript_1/1_Conf_1.000	210	20	3.98E-19	NP_509186.3	78	97.8265	71	56	Carbonic AnHydrase family member (cah-5)
Locus_22076_Transcript_1/1_Conf_1.000	208	2	1.20E-07	XP_002642021.1	65	59.6918	69	45	C. briggsae CBR-COQ-8 protein
Locus_22077_Transcript_1/1_Conf_1.000	264	20	5.10E-14	XP_002642804.1	72	80.8777	75	54	Hypothetical protein CBG21200
Locus_22078_Transcript_1/1_Conf_1.000	341	20	1.90E-21	CAB07368.3	67	105.531	113	76	C. elegans protein F22G12.4, confirmed by transcript evidence
Locus_22079_Transcript_1/1_Conf_1.000	143	20	6.53E-14	XP_002640379.1	87	80.4925	47	41	C. briggsae CBR-OPT-3 protein
Locus_2208_Transcript_1/2_Conf_1.000	4847	20	0	NP_001024981.1	79	1048.88	844	674	Lin-5 (Five) Interacting protein family member (lfi-1)
Locus_2208_Transcript_2/2_Conf_1.000	4847	20	0	NP_001024981.1	79	1044.65	843	673	Lin-5 (Five) Interacting protein family member (lfi-1)
Locus_22080_Transcript_1/1_Conf_1.000	302	3	5.43E-08	XP_002645016.1	62	60.8474	81	51	Hypothetical protein CBG11012
Locus_22081_Transcript_1/1_Conf_1.000	307	20	9.77E-26	NP_498284.1	75	119.783	93	70	hypothetical protein C28H8.11
Locus_22082_Transcript_1/1_Conf_1.000	154	0							

Locus_22083_Transcript_1/1_Conf_1.000	250	2	1.15E-10	NP_505353.1	94	69.707	39	37	hypothetical protein T27E4.7
Locus_22084_Transcript_1/1_Conf_1.000	346	0							
Locus_22085_Transcript_1/1_Conf_1.000	432	0							
Locus_22086_Transcript_1/1_Conf_1.000	319	20	1.43E-48	XP_002633294.1	95	195.667	94	90	C. briggsae CBR-NHR-67 protein
Locus_22087_Transcript_1/1_Conf_1.000	167	20	5.07E-22	NP_500379.1	98	107.457	53	52	hypothetical protein Y37E11B.5
Locus_22088_Transcript_1/1_Conf_1.000	142	0							
Locus_22089_Transcript_1/1_Conf_1.000	362	20	2.96E-06	XP_001361765.2	43	55.0694	116	51	GA21047
Locus_2209_Transcript_1/1_Conf_1.000	782	20	1.64E-55	XP_002642013.1	85	220.32	185	159	Hypothetical protein CBG17947
Locus_22090_Transcript_1/1_Conf_1.000	182	20	2.84E-28	XP_002641773.1	98	128.257	60	59	Hypothetical protein CBG10119
Locus_22091_Transcript_1/1_Conf_1.000	404	2	1.70E-09	NP_496236.1	57	65.855	124	71	hypothetical protein T09F3.2
Locus_22092_Transcript_1/1_Conf_1.000	219	0							
Locus_22093_Transcript_1/1_Conf_1.000	212	0							
Locus_22094_Transcript_1/1_Conf_1.000	190	0							
Locus_22095_Transcript_1/1_Conf_1.000	524	0							
Locus_22096_Transcript_1/1_Conf_1.000	147	0							
Locus_22097_Transcript_1/1_Conf_1.000	173	0							
Locus_22098_Transcript_1/1_Conf_1.000	154	0							
Locus_22099_Transcript_1/1_Conf_1.000	273	0							
Locus_221_Transcript_1/3_Conf_0.571	588	1	3.01E-16	EFO15932.1	65	88.9669	112	73	hypothetical protein LOAG_12576
Locus_221_Transcript_2/3_Conf_0.429	588	1	3.01E-16	EFO15932.1	65	88.9669	112	73	hypothetical protein LOAG_12576
Locus_221_Transcript_3/3_Conf_0.571	2214	20	1.02E-72	EFO15932.1	67	279.641	286	192	hypothetical protein LOAG_12576
Locus_2210_Transcript_1/3_Conf_0.400	1914	20	0	XP_002636622.1	83	884.019	643	537	C. briggsae CBR-SMA-1 protein
Locus_2210_Transcript_2/3_Conf_0.400	1905	20	0	XP_002636622.1	85	883.248	625	532	C. briggsae CBR-SMA-1 protein

Locus_2210_Transcript_3/3_Conf_0.600	1954	20	0	XP_002636622.1	85	882.478	623	531	C. briggsae CBR-SMA-1 protein
Locus_22100_Transcript_1/1_Conf_1.000	319	0							
Locus_22101_Transcript_1/1_Conf_1.000	167	0							
Locus_22102_Transcript_1/1_Conf_1.000	169	20	1.24E-28	XP_002913159.1	100	129.413	56	56	PREDICTED: serine/threonine-protein phosphatase PP1-gamma catalytic subunit-like
Locus_22103_Transcript_1/1_Conf_1.000	181	0							
Locus_22104_Transcript_1/1_Conf_1.000	162	0							
Locus_22105_Transcript_1/1_Conf_1.000	668	20	7.73E-07	XP_001894189.1	53	58.151	149	79	hypothetical protein
Locus_22106_Transcript_1/1_Conf_1.000	179	0							
Locus_22107_Transcript_1/1_Conf_1.000	407	5	1.74E-14	NP_492265.1	66	82.4185	77	51	MYST family histone acetyltransferase-like family member (mys-4)
Locus_22108_Transcript_1/1_Conf_1.000	393	6	1.90E-08	XP_002645850.1	58	62.3882	73	43	Hypothetical protein CBG07586
Locus_22109_Transcript_1/1_Conf_1.000	191	0							
Locus_2211_Transcript_1/1_Conf_1.000	903	5	3.79E-12	NP_496595.1	73	76.6406	67	49	SR protein related family member (rsr-2)
Locus_22110_Transcript_1/1_Conf_1.000	250	0							

Locus_22111_Transcript_1/1_Conf_1.000	441	20	7.85E-47	EFO21836.1	77	189.889	146	113	hypothetical protein LOAG_06649
Locus_22112_Transcript_1/1_Conf_1.000	301	3	2.13E-04	XP_002646170.1	59	48.9062	72	43	C. briggsae CBR-TAF-5 protein
Locus_22113_Transcript_1/1_Conf_1.000	762	20	6.82E-27	XP_002131847.1	73	125.176	127	93	PREDICTED: similar to riboflavin kinase
Locus_22114_Transcript_1/1_Conf_1.000	157	2	2.23E-09	XP_002634423.1	75	65.4698	52	39	C. briggsae CBR-PTR-14 protein
Locus_22115_Transcript_1/1_Conf_1.000	513	20	5.99E-69	NP_741582.2	86	263.462	170	147	hypothetical protein H14N18.4
Locus_22116_Transcript_1/1_Conf_1.000	131	0							
Locus_22117_Transcript_1/1_Conf_1.000	305	0							
Locus_22118_Transcript_1/1_Conf_1.000	292	20	3.41E-34	XP_002629844.1	89	147.902	97	87	C. briggsae CBR-SCP-1 protein
Locus_22119_Transcript_1/1_Conf_1.000	302	0							
Locus_2212_Transcript_1/1_Conf_1.000	889	20	9.52E-69	NP_493412.2	68	264.618	316	218	hypothetical protein Y37H9A.3
Locus_22120_Transcript_1/1_Conf_1.000	130	0							
Locus_22121_Transcript_1/1_Conf_1.000	633	20	7.79E-43	NP_504651.1	75	177.563	152	114	MOlybdenum Cofactor biosynthesis family member (moc-2)
Locus_22122_Transcript_1/1_Conf_1.000	361	0							
Locus_22123_Transcript_1/1_Conf_1.000	148	0							
Locus_22124_Transcript_1/1_Conf_1.000	197	6	2.22E-09	EFO16773.1	69	65.4698	66	46	hypothetical protein LOAG_11731
Locus_22125_Transcript_1/1_Conf_1.000	286	0							
Locus_22126_Transcript_1/1_Conf_1.000	614	20	1.49E-88	1LLQ	89	329.331	201	180	CrystalStructure Of Malic Enzyme From Ascaris Suum Complexed With Nicotinamide Adenine Dinucleotide
Locus_22127_Transcript_1/1_Conf_1.000	262	0							
Locus_22128_Transcript_1/1_Conf_1.000	129	0							
Locus_22129_Transcript_1/1_Conf_1.000	379	9	3.19E-16	XP_002638736.1	71	88.1965	84	60	C. briggsae CBR-EGO-1 protein

Locus_2213_Transcript_1/1_Conf_1.000	2658	20	0	XP_002645233.1	76	946.421	777	597	C. briggsae CBR-TAG-147 protein
Locus_22130_Transcript_1/1_Conf_1.000	173	20	2.59E-10	YP_001413705.1	71	68.5514	57	41	D-lactate dehydrogenase (cytochrome)
Locus_22131_Transcript_1/1_Conf_1.000	222	20	2.64E-18	NP_497641.2	83	95.1301	68	57	CaDHerin family member (cdh-1)
Locus_22132_Transcript_1/1_Conf_1.000	288	0							
Locus_22133_Transcript_1/1_Conf_1.000	359	1	1.68E-17	EFO25272.1	58	92.4337	116	68	hypothetical protein LOAG_03210
Locus_22134_Transcript_1/1_Conf_1.000	284	0							
Locus_22135_Transcript_1/1_Conf_1.000	502	20	1.93E-53	NP_741714.1	80	211.846	167	134	related to yeast Vacuolar Protein Sorting factor family member (vps-45)
Locus_22136_Transcript_1/1_Conf_1.000	373	0							
Locus_22137_Transcript_1/1_Conf_1.000	133	0							
Locus_22138_Transcript_1/1_Conf_1.000	165	0							
Locus_22139_Transcript_1/1_Conf_1.000	174	17	5.04E-06	XP_002740793.1	62	54.299	54	34	PREDICTED: hypothetical protein
Locus_2214_Transcript_1/2_Conf_1.000	1072	20	2.79E-63	EFO18993.1	56	246.899	362	203	hypothetical protein LOAG_09502
Locus_2214_Transcript_2/2_Conf_1.000	1033	20	4.37E-66	EFO18993.1	58	256.144	349	203	hypothetical protein LOAG_09502
Locus_22140_Transcript_1/1_Conf_1.000	169	2	7.31E-13	NP_491499.1	80	77.0258	56	45	hypothetical protein F54C1.1
Locus_22141_Transcript_1/1_Conf_1.000	172	0							
Locus_22142_Transcript_1/1_Conf_1.000	157	0							
Locus_22143_Transcript_1/1_Conf_1.000	155	0							
Locus_22144_Transcript_1/1_Conf_1.000	251	2	8.00E-04	NP_503880.1	58	46.9802	50	29	hypothetical protein Y73C8B.1
Locus_22145_Transcript_1/1_Conf_1.000	260	0							
Locus_22146_Transcript_1/1_Conf_1.000	168	0							
Locus_22147_Transcript_1/1_Conf_1.000	276	0							
Locus_22148_Transcript_1/2_Conf_1.000	276	4	3.05E-11	NP_495478.1	82	71.633	50	41	hypothetical protein T07F8.4
Locus_22148_Transcript_2/2_Conf_1.000	270	2	1.43E-08	NP_495478.1	78	62.7734	50	39	hypothetical protein T07F8.4

Locus_22149_Transcript_1/1_Conf_1.000	291	20	2.29E-22	NP_061932.1	100	108.612	51	51	mitochondrial import receptor subunit TOM7 homolog
Locus_2215_Transcript_1/2_Conf_1.000	1365	2	1.44E-04	XP_001751006.1	30	52.373	325	98	hypothetical protein
Locus_2215_Transcript_2/2_Conf_1.000	1365	4	7.63E-06	XP_001751006.1	29	56.6102	325	95	hypothetical protein
Locus_22150_Transcript_1/1_Conf_1.000	206	4	1.74E-14	NP_496984.1	72	82.4185	68	49	hypothetical protein R06A4.8
Locus_22151_Transcript_1/1_Conf_1.000	229	0							
Locus_22152_Transcript_1/1_Conf_1.000	319	20	1.34E-46	XP_002831374.1	99	189.119	106	105	PREDICTED: ADP/ATP translocase 3-like, partial
Locus_22153_Transcript_1/1_Conf_1.000	410	1	4.28E-05	NP_504468.2	61	51.2174	72	44	hypothetical protein C18C4.7
Locus_22154_Transcript_1/1_Conf_1.000	213	2	4.54E-15	XP_002638798.1	76	84.3445	71	54	C. briggsae CBR-PIF-1 protein
Locus_22155_Transcript_1/1_Conf_1.000	251	20	1.83E-24	XP_002643979.1	95	115.546	68	65	C. briggsae CBR-PGP-3 protein
Locus_22156_Transcript_1/1_Conf_1.000	148	15	7.24E-08	XP_002630579.1	75	60.4622	48	36	C. briggsae CBR-NHX-2 protein
Locus_22157_Transcript_1/1_Conf_1.000	263	0							
Locus_22158_Transcript_1/1_Conf_1.000	248	0							
Locus_22159_Transcript_1/1_Conf_1.000	327	20	4.43E-26	NP_502164.1	86	120.939	105	91	WHiTe (Drosophila) related ABC transporte family member (wht-2)
Locus_2216_Transcript_1/2_Conf_1.000	2128	20	2.68E-147	NP_505969.1	91	527.324	309	284	HRD (yeast Hmg-coa Reductase Degradation) homolog family member (hrd-1)

Locus_2216_Transcript_2/2_Conf_1.000	2053	20	2.57E-147	NP_505969.1	91	527.324	309	284	HRD (yeast Hmg-coa Reductase Degradation) homolog family member (hrd-1)
Locus_22160_Transcript_1/1_Conf_1.000	372	2	4.35E-05	NP_506364.2	50	51.2174	122	62	hypothetical protein C50B8.5
Locus_22161_Transcript_1/1_Conf_1.000	184	0							
Locus_22162_Transcript_1/1_Conf_1.000	151	0							
Locus_22163_Transcript_1/1_Conf_1.000	473	20	2.26E-46	XP_001895190.1	74	188.348	157	117	Receptor family ligand binding region containing protein
Locus_22164_Transcript_1/1_Conf_1.000	156	0							
Locus_22165_Transcript_1/1_Conf_1.000	225	20	3.21E-24	XP_001100810.2	100	114.775	72	72	PREDICTED: 60S ribosomal protein L5-like isoform 3
Locus_22166_Transcript_1/1_Conf_1.000	167	0							
Locus_22167_Transcript_1/1_Conf_1.000	169	0							
Locus_22168_Transcript_1/1_Conf_1.000	300	0							
Locus_22169_Transcript_1/1_Conf_1.000	923	20	9.69E-11	XP_001464299.1	42	72.0182	293	124	kinesin K39
Locus_2217_Transcript_1/1_Conf_1.000	1332	20	6.06E-101	NP_498411.1	81	372.474	281	228	related to yeast Vacuolar Protein Sorting factor family member (vps-16)
Locus_22170_Transcript_1/1_Conf_1.000	120	0							

Locus_22171_Transcript_1/1_Conf_1.000	174	20	2.33E-19	XP_002643811.1	94	98.5969	57	54	C. briggsae CBR-NMY-1 protein
Locus_22172_Transcript_1/1_Conf_1.000	246	4	8.84E-19	NP_496968.1	77	96.6709	74	57	hypothetical protein Y48B6A.12
Locus_22173_Transcript_1/1_Conf_1.000	134	0							
Locus_22174_Transcript_1/1_Conf_1.000	284	20	1.23E-15	ABJ99064.1	64	86.2705	96	62	Hypothetical protein W03A3.2
Locus_22175_Transcript_1/1_Conf_1.000	210	20	2.75E-20	EFO25043.1	86	101.679	69	60	hypothetical protein LOAG_03439
Locus_22176_Transcript_1/1_Conf_1.000	197	20	5.83E-26	XP_002640668.1	96	120.553	65	63	C. briggsae CBR-UNC-54 protein
Locus_22177_Transcript_1/1_Conf_1.000	215	2	1.91E-05	XP_002636455.1	65	52.373	47	31	Hypothetical protein CBG23119
Locus_22178_Transcript_1/1_Conf_1.000	574	20	1.76E-58	CAR63670.1	84	229.18	152	129	hypothetical protein
Locus_22179_Transcript_1/1_Conf_1.000	211	0							
Locus_2218_Transcript_1/1_Conf_1.000	324	0							
Locus_22180_Transcript_1/1_Conf_1.000	177	0							
Locus_22181_Transcript_1/1_Conf_1.000	359	0							
Locus_22182_Transcript_1/1_Conf_1.000	255	0							
Locus_22183_Transcript_1/1_Conf_1.000	374	20	5.42E-56	XP_002642222.1	90	220.32	122	111	C. briggsae CBR-FSN-1 protein
Locus_22184_Transcript_1/1_Conf_1.000	345	10	3.20E-40	XP_002631802.1	84	167.933	115	97	Hypothetical protein CBG21021
Locus_22185_Transcript_1/1_Conf_1.000	273	20	2.12E-20	NP_492278.2	69	102.064	91	63	hypothetical protein T19A6.4
Locus_22186_Transcript_1/1_Conf_1.000	331	4	2.52E-05	XP_001204017.1	53	51.9878	111	59	PREDICTED: similar to Pdcd6ip protein
Locus_22187_Transcript_1/1_Conf_1.000	552	5	2.03E-37	ACM46022.1	70	159.073	150	105	Troponin t protein 3, isoform d
Locus_22188_Transcript_1/1_Conf_1.000	141	0							
Locus_22189_Transcript_1/1_Conf_1.000	136	0							
Locus_2219_Transcript_1/1_Conf_1.000	242	0							
Locus_22190_Transcript_1/1_Conf_1.000	346	20	2.79E-44	NP_498014.2	86	181.415	115	100	COenzyme Q (ubiquinone) biosynthesis family member (coq-8)

Locus_22191_Transcript_1/1_Conf_1.000	379	20	1.92E-29	XP_002634543.1	71	132.109	118	84	C. briggsae CBR-CED-5 protein
Locus_22192_Transcript_1/1_Conf_1.000	128	0							
Locus_22193_Transcript_1/1_Conf_1.000	497	0							
Locus_22194_Transcript_1/1_Conf_1.000	143	20	4.22E-21	3JYH	100	104.375	47	47	Human Dipeptidyl Peptidase Dpp7
Locus_22195_Transcript_1/1_Conf_1.000	230	7	1.14E-05	B4XT00.1	60	53.1434	71	43	C-type lectin B1
Locus_22196_Transcript_1/1_Conf_1.000	255	0							
Locus_22197_Transcript_1/1_Conf_1.000	496	20	3.47E-39	XP_001899820.1	85	164.466	132	113	Hexokinase family protein
Locus_22198_Transcript_1/1_Conf_1.000	132	9	2.27E-06	CBA11992.1	80	55.4546	41	33	endonuclease-reverse transcriptase HmRTE-e01
Locus_22199_Transcript_1/1_Conf_1.000	311	0							
Locus_222_Transcript_1/3_Conf_0.625	1227	20	1.16E-127	P54813.2	83	461.07	323	270	Protein YME1 homolog
Locus_222_Transcript_2/3_Conf_0.750	2500	20	0	P54813.2	86	921.383	636	551	Protein YME1 homolog
Locus_222_Transcript_3/3_Conf_0.750	2518	20	0	P54813.2	87	935.636	636	556	Protein YME1 homolog

Locus_2220_Transcript_1/1_Conf_1.000	2000	20	0	NP_498887.2	91	1072.77	628	574	GLutamate Receptor family (AMPA) family member (glr-1)
Locus_22200_Transcript_1/1_Conf_1.000	349	0							
Locus_22201_Transcript_1/1_Conf_1.000	146	1	6.36E-04	EFO24319.1	64	47.3654	48	31	hypothetical protein LOAG_04165
Locus_22202_Transcript_1/1_Conf_1.000	162	0							
Locus_22203_Transcript_1/1_Conf_1.000	291	4	1.59E-31	XP_002642450.1	83	139.043	92	77	Hypothetical protein CBG06858
Locus_22204_Transcript_1/1_Conf_1.000	261	20	2.01E-18	NP_740780.1	94	95.5153	71	67	AdaPtin, Small chain (clathrin associated complex) family member (aps-3)
Locus_22205_Transcript_1/1_Conf_1.000	376	0							
Locus_22206_Transcript_1/1_Conf_1.000	462	0							
Locus_22207_Transcript_1/1_Conf_1.000	163	0							
Locus_22208_Transcript_1/1_Conf_1.000	452	20	1.85E-32	XP_002636579.1	76	142.124	103	79	Hypothetical protein CBG23273
Locus_22209_Transcript_1/1_Conf_1.000	206	0							
Locus_2221_Transcript_1/1_Conf_1.000	864	0							
Locus_22210_Transcript_1/1_Conf_1.000	174	20	5.93E-23	BAG11032.1	100	110.538	52	52	FERM, RhoGEF and pleckstrin domain-containing protein 1
Locus_22211_Transcript_1/1_Conf_1.000	152	0							
Locus_22212_Transcript_1/1_Conf_1.000	229	0							

Locus_22213_Transcript_1/1_Conf_1.000	206	0							
Locus_22214_Transcript_1/1_Conf_1.000	149	0							
Locus_22215_Transcript_1/1_Conf_1.000	413	20	9.70E-50	NP_501127.2	85	199.519	126	108	RNA Polymerase, Class III (C) family member (rpc-1)
Locus_22216_Transcript_1/1_Conf_1.000	317	20	1.76E-38	NP_495009.1	90	162.155	96	87	hypothetical protein F09E5.3
Locus_22217_Transcript_1/1_Conf_1.000	270	20	4.01E-19	NP_490706.2	64	97.8265	90	58	hypothetical protein Y48G1BM.1
Locus_22218_Transcript_1/1_Conf_1.000	219	20	1.46E-16	XP_001894229.1	84	89.3521	58	49	Variant SH3 domain containing protein
Locus_22219_Transcript_1/1_Conf_1.000	159	0							
Locus_2222_Transcript_1/2_Conf_1.000	344	4	5.15E-14	XP_002640676.1	89	80.8777	47	42	Hypothetical protein CBG19738
Locus_2222_Transcript_2/2_Conf_1.000	340	2	6.53E-14	XP_002640676.1	85	80.4925	70	60	Hypothetical protein CBG19738
Locus_22220_Transcript_1/1_Conf_1.000	281	1	2.04E-07	NP_502613.2	62	58.9214	91	57	QUInine non-avoider family member (qui-1)
Locus_22221_Transcript_1/1_Conf_1.000	364	0							
Locus_22222_Transcript_1/1_Conf_1.000	158	0							
Locus_22223_Transcript_1/1_Conf_1.000	229	1	8.18E-12	XP_001896987.1	63	73.559	79	50	Zinc finger, C2H2 type family protein
Locus_22224_Transcript_1/1_Conf_1.000	327	1	2.98E-14	NP_508445.1	60	81.6481	82	50	hypothetical protein T07D1.3
Locus_22225_Transcript_1/1_Conf_1.000	227	0							
Locus_22226_Transcript_1/1_Conf_1.000	175	0							
Locus_22227_Transcript_1/1_Conf_1.000	243	0							
Locus_22228_Transcript_1/1_Conf_1.000	544	7	2.52E-05	CAB55122.3	56	52.373	51	29	C. elegans protein Y116A8C.16a, partially confirmed by transcript evidence
Locus_22229_Transcript_1/1_Conf_1.000	407	20	1.32E-62	XP_002642870.1	89	242.276	135	121	Hypothetical protein CBG15140
Locus_2223_Transcript_1/1_Conf_1.000	2261	20	0	NP_496721.2	87	1045.42	664	578	Carnitine Palmitoyl Transferase family member (cpt-1)

Locus_22230_Transcript_1/1_Conf_1.000	319	20	4.94E-33	NP_504750.2	80	144.05	91	73	hypothetical protein K11C4.2
Locus_22231_Transcript_1/1_Conf_1.000	145	20	1.97E-21	BAG60853.1	100	105.531	47	47	unnamed protein product
Locus_22232_Transcript_1/1_Conf_1.000	329	0							
Locus_22233_Transcript_1/1_Conf_1.000	224	0							
Locus_22234_Transcript_1/1_Conf_1.000	289	20	4.19E-32	NP_491653.2	84	140.969	95	80	hypothetical protein F55F8.3
Locus_22235_Transcript_1/1_Conf_1.000	312	0							
Locus_22236_Transcript_1/1_Conf_1.000	422	0							
Locus_22237_Transcript_1/1_Conf_1.000	160	0							
Locus_22238_Transcript_1/1_Conf_1.000	837	20	5.54E-92	XP_002630799.1	72	341.658	285	208	Hypothetical protein CBG02498
Locus_22239_Transcript_1/1_Conf_1.000	435	5	1.97E-26	NP_503502.2	75	122.094	86	65	hypothetical protein F36H9.7
Locus_2224_Transcript_1/2_Conf_1.000	2466	20	2.43E-38	XP_002640658.1	39	165.622	746	298	Hypothetical protein CBG19716
Locus_2224_Transcript_2/2_Conf_1.000	2403	20	5.28E-38	XP_002640658.1	39	164.466	731	286	Hypothetical protein CBG19716
Locus_22240_Transcript_1/1_Conf_1.000	247	0							
Locus_22241_Transcript_1/1_Conf_1.000	289	20	1.23E-07	NP_001037879.1	69	59.6918	63	44	CTF8, chromosome transmission fidelity factor 8 homolog
Locus_22242_Transcript_1/1_Conf_1.000	163	0							
Locus_22243_Transcript_1/1_Conf_1.000	267	20	3.89E-14	NP_505919.3	63	81.2629	86	55	hypothetical protein C55A6.3
Locus_22244_Transcript_1/1_Conf_1.000	140	2	5.94E-07	XP_002641999.1	78	57.3806	46	36	C. briggsae CBR-ACY-3 protein
Locus_22245_Transcript_1/1_Conf_1.000	204	18	4.13E-24	XP_002641899.1	89	114.39	68	61	C. briggsae CBR-NHR-10 protein
Locus_22246_Transcript_1/1_Conf_1.000	152	0							
Locus_22247_Transcript_1/1_Conf_1.000	438	0							
Locus_22248_Transcript_1/1_Conf_1.000	164	0							
Locus_22249_Transcript_1/1_Conf_1.000	133	0							

Locus_2225_Transcript_1/1_Conf_1.000	2231	20	0	XP_002633925.1	88	749.584	551	487	C. briggsae CBR-SOC-2 protein
Locus_22250_Transcript_1/1_Conf_1.000	351	2	6.64E-22	XP_002638987.1	75	107.071	113	85	Hypothetical protein CBG22233
Locus_22251_Transcript_1/1_Conf_1.000	959	20	2.68E-43	XP_002630256.1	53	180.259	308	166	Hypothetical protein CBG00675
Locus_22252_Transcript_1/1_Conf_1.000	236	0							
Locus_22253_Transcript_1/1_Conf_1.000	177	20	7.97E-28	XP_002811367.1	100	126.716	58	58	PREDICTED: complement C1q subcomponent subunit C-like isoform 1
Locus_22254_Transcript_1/1_Conf_1.000	322	16	7.30E-53	NP_001129826.1	98	209.92	106	104	hypothetical protein T13H5.1
Locus_22255_Transcript_1/1_Conf_1.000	131	0							
Locus_22256_Transcript_1/1_Conf_1.000	132	20	8.89E-19	XP_002802333.1	100	96.6709	43	43	PREDICTED: eukaryotic translation initiation factor 3 subunit I-like
Locus_22257_Transcript_1/1_Conf_1.000	143	20	1.23E-20	XP_002915235.1	100	102.834	47	47	PREDICTED: spectrin alpha chain, brain-like isoform 2
Locus_22258_Transcript_1/1_Conf_1.000	262	0							

Locus_22259_Transcript_1/1_Conf_1.000	237	20	1.57E-39	ADI78884.1	100	165.622	79	79	plasma membrane citrate carrier
Locus_2226_Transcript_1/1_Conf_1.000	576	4	5.82E-17	XP_001899796.1	70	91.2781	82	58	Uncoordinated protein 40
Locus_22260_Transcript_1/1_Conf_1.000	147	0							
Locus_22261_Transcript_1/1_Conf_1.000	130	0							
Locus_22262_Transcript_1/1_Conf_1.000	273	20	2.11E-28	XP_002644906.1	92	128.642	88	81	Hypothetical protein CBG10845
Locus_22263_Transcript_1/1_Conf_1.000	208	0							
Locus_22264_Transcript_1/1_Conf_1.000	264	0							
Locus_22265_Transcript_1/1_Conf_1.000	210	2	1.09E-08	NP_001023357.2	67	63.1586	68	46	hypothetical protein T04C4.1
Locus_22266_Transcript_1/1_Conf_1.000	145	0							
Locus_22267_Transcript_1/1_Conf_1.000	295	20	2.52E-29	XP_002631704.1	80	131.724	96	77	Hypothetical protein CBG20903
Locus_22268_Transcript_1/1_Conf_1.000	206	0							
Locus_22269_Transcript_1/1_Conf_1.000	210	0							
Locus_2227_Transcript_1/1_Conf_1.000	188	1	6.11E-07	XP_001898414.1	85	57.3806	35	30	collagen col-34
Locus_22270_Transcript_1/1_Conf_1.000	173	20	7.79E-15	XP_002636770.1	85	83.5741	56	48	Hypothetical protein CBG23501
Locus_22271_Transcript_1/1_Conf_1.000	767	20	1.78E-75	XP_002630049.1	73	286.574	238	176	C. briggsae CBR-APN-1 protein
Locus_22272_Transcript_1/1_Conf_1.000	382	20	2.83E-65	XP_002646694.1	95	251.136	126	120	C. briggsae CBR-SPON-1 protein
Locus_22273_Transcript_1/1_Conf_1.000	204	0							
Locus_22274_Transcript_1/1_Conf_1.000	182	0							
Locus_22275_Transcript_1/1_Conf_1.000	328	0							
Locus_22276_Transcript_1/1_Conf_1.000	278	0							
Locus_22277_Transcript_1/1_Conf_1.000	307	9	5.93E-23	NP_499482.1	78	110.538	83	65	defective SPErmatogenesis family member (spe-6)
Locus_22278_Transcript_1/1_Conf_1.000	167	0							
Locus_22279_Transcript_1/1_Conf_1.000	464	20	9.54E-69	XP_002629875.1	90	262.692	154	139	C. briggsae CBR-LET-75 protein
Locus_2228_Transcript_1/1_Conf_1.000	549	0							
Locus_22280_Transcript_1/1_Conf_1.000	247	20	5.51E-37	XP_002641343.1	96	157.147	81	78	C. briggsae CBR-DPY-18 protein

Locus_22281_Transcript_1/1_Conf_1.000	286	20	3.93E-30	NP_501419.2	84	134.42	95	80	hypothetical protein D2096.3
Locus_22282_Transcript_1/1_Conf_1.000	188	0							
Locus_22283_Transcript_1/1_Conf_1.000	423	0							
Locus_22284_Transcript_1/1_Conf_1.000	160	3	4.63E-07	NP_509676.1	68	57.7658	54	37	G-protein-coupled Receptor Kinase family member (grk-1)
Locus_22285_Transcript_1/1_Conf_1.000	158	0							
Locus_22286_Transcript_1/1_Conf_1.000	133	3	1.09E-08	EFO24958.1	79	63.1586	43	34	hypothetical protein LOAG_03524
Locus_22287_Transcript_1/1_Conf_1.000	357	20	1.24E-28	NP_001021200.1	70	129.413	116	82	tryptophanyl (W) tRNA Synthetase family member (wrs-2)
Locus_22288_Transcript_1/1_Conf_1.000	138	0							
Locus_22289_Transcript_1/1_Conf_1.000	287	0							
Locus_2229_Transcript_1/2_Conf_1.000	2011	20	0	NP_498777.2	84	619.772	437	370	hypothetical protein C14B9.8
Locus_2229_Transcript_2/2_Conf_1.000	1987	20	0	NP_498777.2	84	619.772	437	370	hypothetical protein C14B9.8
Locus_22290_Transcript_1/1_Conf_1.000	327	0							
Locus_22291_Transcript_1/1_Conf_1.000	172	0							
Locus_22292_Transcript_1/1_Conf_1.000	337	20	4.35E-50	XP_002632347.1	92	200.675	113	105	Hypothetical protein CBG00360
Locus_22293_Transcript_1/1_Conf_1.000	186	20	3.81E-25	NP_509174.1	96	117.857	61	59	hypothetical protein C16E9.2
Locus_22294_Transcript_1/1_Conf_1.000	321	20	6.90E-19	XP_001893826.1	66	97.0561	104	69	Protein-tyrosine phosphatase containing protein
Locus_22295_Transcript_1/1_Conf_1.000	325	0							
Locus_22296_Transcript_1/1_Conf_1.000	235	20	4.58E-31	XP_002927413.1	100	137.502	67	67	PREDICTED: eukaryotic translation initiation factor 3 subunit C-like
Locus_22297_Transcript_1/1_Conf_1.000	188	0							
Locus_22298_Transcript_1/1_Conf_1.000	147	0							
Locus_22299_Transcript_1/1_Conf_1.000	172	0							

Locus_223_Transcript_1/1_Conf_1.000	2106	20	0	XP_001894294.1	87	899.427	592	517	protein phosphatase PP2A regulatory subunit
Locus_2230_Transcript_1/1_Conf_1.000	916	0							
Locus_22300_Transcript_1/1_Conf_1.000	368	0							
Locus_22301_Transcript_1/1_Conf_1.000	140	0							
Locus_22302_Transcript_1/1_Conf_1.000	433	20	6.05E-76	BAG60350.1	100	286.574	144	144	unnamed protein product
Locus_22303_Transcript_1/1_Conf_1.000	293	20	1.18E-18	XP_002632873.1	64	96.2857	97	63	Hypothetical protein CBG15077
Locus_22304_Transcript_1/1_Conf_1.000	145	0							
Locus_22305_Transcript_1/1_Conf_1.000	367	0							
Locus_22306_Transcript_1/1_Conf_1.000	218	8	5.70E-18	NP_501928.1	80	93.9745	71	57	hypothetical protein F13B12.4
Locus_22307_Transcript_1/1_Conf_1.000	139	0							

Locus_22308_Transcript_1/1_Conf_1.000	381	20	1.57E-23	NP_494994.1	67	112.464	129	87	hypothetical protein ZK622.1
Locus_22309_Transcript_1/1_Conf_1.000	350	1	4.06E-11	EFO24286.1	76	71.2478	50	38	hypothetical protein LOAG_04203
Locus_2231_Transcript_1/1_Conf_1.000	563	20	1.95E-43	CAR63593.1	83	179.104	110	92	putative PQ loop repeat family protein
Locus_22310_Transcript_1/2_Conf_1.000	423	0							
Locus_22310_Transcript_2/2_Conf_1.000	475	0							
Locus_22311_Transcript_1/1_Conf_1.000	493	3	1.48E-05	XP_002633421.1	53	52.7582	94	50	C. briggsae CBR-NHR-43 protein
Locus_22312_Transcript_1/1_Conf_1.000	131	20	4.29E-13	NP_505370.2	97	77.7962	43	42	hypothetical protein B0222.2
Locus_22313_Transcript_1/1_Conf_1.000	637	20	4.57E-83	XP_002641813.1	95	311.227	180	171	C. briggsae CBR-INF-1 protein
Locus_22314_Transcript_1/1_Conf_1.000	283	0							
Locus_22315_Transcript_1/1_Conf_1.000	787	7	2.55E-72		74	276.174	255	189	hypothetical protein Y105C5A.r
Locus_22316_Transcript_1/1_Conf_1.000	458	2	6.16E-04	NP_001024104.1	61	47.3654	59	36	hypothetical protein T05B11.7
Locus_22317_Transcript_1/1_Conf_1.000	312	0							
Locus_22318_Transcript_1/1_Conf_1.000	407	0							
Locus_22319_Transcript_1/1_Conf_1.000	164	5	1.70E-09	NP_495484.1	80	65.855	50	40	hypothetical protein C27H5.2

Locus_2232_Transcript_1/1_Conf_1.000	500	20	4.85E-17	NP_500336.1	89	90.8929	57	51	Mitochondrial ATPase Inhibitor family member (mai-2)
Locus_22320_Transcript_1/1_Conf_1.000	379	0							
Locus_22321_Transcript_1/1_Conf_1.000	351	0							
Locus_22322_Transcript_1/1_Conf_1.000	305	2	4.29E-28	EAW71418.1	100	92.0485	57	57	RNA binding motif protein 8A, isoform CRA_a
Locus_22323_Transcript_1/1_Conf_1.000	143	0							
Locus_22324_Transcript_1/1_Conf_1.000	290	0							
Locus_22325_Transcript_1/1_Conf_1.000	175	0							
Locus_22326_Transcript_1/1_Conf_1.000	154	0							
Locus_22327_Transcript_1/1_Conf_1.000	347	20	1.26E-36	EFO24516.1	73	155.992	115	85	TBL1X protein
Locus_22328_Transcript_1/1_Conf_1.000	194	4	1.27E-04	XP_002639323.1	59	49.6766	67	40	C. briggsae CBR-HUM-7 protein
Locus_22329_Transcript_1/1_Conf_1.000	147	0							
Locus_2233_Transcript_1/1_Conf_1.000	256	20	7.65E-34	NP_001129862.1	90	146.747	85	77	UNCoordinated family member (unc-26)
Locus_22330_Transcript_1/1_Conf_1.000	269	0							

Locus_22331_Transcript_1/1_Conf_1.000	162	0							
Locus_22332_Transcript_1/1_Conf_1.000	417	20	2.71E-39	NP_505978.1	69	164.851	138	96	hypothetical protein D2023.4
Locus_22333_Transcript_1/1_Conf_1.000	206	11	1.33E-14	ADD13547.1	91	82.8037	68	62	Inositol triphosphate receptor protein 1, isoform h
Locus_22334_Transcript_1/1_Conf_1.000	393	20	2.99E-54	NP_001022450.1	89	214.542	130	116	hypothetical protein Y48E1A.1
Locus_22335_Transcript_1/1_Conf_1.000	260	4	7.39E-29	NP_510438.1	95	130.183	67	64	CalSeQuestrin family member (csq-1)
Locus_22336_Transcript_1/1_Conf_1.000	255	20	4.83E-12	NP_509417.2	71	74.3294	67	48	hypothetical protein F40B5.2
Locus_22337_Transcript_1/1_Conf_1.000	163	0							
Locus_22338_Transcript_1/1_Conf_1.000	261	0							
Locus_22339_Transcript_1/1_Conf_1.000	343	1	1.55E-10	NP_001041292.1	71	69.3218	59	42	hypothetical protein T23E7.6
Locus_2234_Transcript_1/1_Conf_1.000	2782	20	0	XP_002633929.1	86	1461.82	878	756	C. briggsae CBR-LAM-1 protein
Locus_22340_Transcript_1/1_Conf_1.000	243	0							
Locus_22341_Transcript_1/1_Conf_1.000	370	0							
Locus_22342_Transcript_1/1_Conf_1.000	283	20	6.78E-06	ZP_04074401.1	64	53.9138	53	34	hypothetical protein bthur0013_47340
Locus_22343_Transcript_1/1_Conf_1.000	131	0							
Locus_22344_Transcript_1/1_Conf_1.000	186	0							
Locus_22345_Transcript_1/1_Conf_1.000	540	2	2.74E-04	NP_001033432.1	65	48.9062	47	31	hypothetical protein T19E7.6
Locus_22346_Transcript_1/1_Conf_1.000	312	20	2.17E-20	NP_502560.1	67	102.064	81	55	FATty acid desaturase family member (fat-2)
Locus_22347_Transcript_1/1_Conf_1.000	143	0							
Locus_22348_Transcript_1/1_Conf_1.000	587	0							
Locus_22349_Transcript_1/1_Conf_1.000	203	0							
Locus_2235_Transcript_1/1_Conf_1.000	1801	20	1.11E-58	ACI32657.1	53	232.646	274	147	Delta protein
Locus_22350_Transcript_1/1_Conf_1.000	354	20	1.73E-46	XP_002638742.1	92	188.734	117	108	C. briggsae CBR-PDE-6 protein
Locus_22351_Transcript_1/1_Conf_1.000	139	0							

Locus_22352_Transcript_1/1_Conf_1.000	609	20	2.28E-65	NP_001021681.1	77	252.292	209	162	hypothetical protein Y105E8A.10
Locus_22353_Transcript_1/1_Conf_1.000	346	0							
Locus_22354_Transcript_1/1_Conf_1.000	206	20	5.07E-14	XP_002633760.1	68	80.8777	58	40	C. briggsae CBR-GON-1 protein
Locus_22355_Transcript_1/1_Conf_1.000	389	0							
Locus_22356_Transcript_1/1_Conf_1.000	263	20	7.15E-16	CAR63522.1	72	87.0409	86	62	putative heat shock protein
Locus_22357_Transcript_1/1_Conf_1.000	181	20	8.57E-17	XP_002637669.1	88	90.1225	51	45	Hypothetical protein CBG19425
Locus_22358_Transcript_1/1_Conf_1.000	133	1	6.18E-04	XP_002637804.1	70	47.3654	37	26	Hypothetical protein CBG04590
Locus_22359_Transcript_1/1_Conf_1.000	180	1	1.68E-04	NP_499757.2	60	49.2914	51	31	hypothetical protein T03F6.4
Locus_2236_Transcript_1/5_Conf_0.444	342	0							
Locus_2236_Transcript_2/5_Conf_0.222	475	0							
Locus_2236_Transcript_3/5_Conf_0.333	333	0							
Locus_2236_Transcript_4/5_Conf_0.333	1724	20	7.87E-123	XP_002637706.1	77	445.662	339	264	Hypothetical protein CBG11571
Locus_2236_Transcript_5/5_Conf_0.222	342	0							
Locus_22360_Transcript_1/1_Conf_1.000	199	2	1.94E-05	XP_001891532.1	62	52.373	66	41	hypothetical protein Bm1_00020
Locus_22361_Transcript_1/1_Conf_1.000	374	20	2.31E-06	EFN78575.1	57	55.4546	83	48	Histone-lysine N-methyltransferase SETMAR
Locus_22362_Transcript_1/1_Conf_1.000	147	0							
Locus_22363_Transcript_1/1_Conf_1.000	274	0							
Locus_22364_Transcript_1/1_Conf_1.000	139	0							
Locus_22365_Transcript_1/1_Conf_1.000	215	0							
Locus_22366_Transcript_1/1_Conf_1.000	248	0							

Locus_22367_Transcript_1/1_Conf_1.000	275	6	2.59E-10	NP_001033378.1	67	68.5514	73	49	Acyl CoA DeHydrogenase family member (acdh-11)
Locus_22368_Transcript_1/1_Conf_1.000	167	0							
Locus_22369_Transcript_1/1_Conf_1.000	460	20	3.22E-37	CAB55118.2	73	157.918	143	105	C. elegans protein Y48C3A.18a, confirmed by transcript evidence
Locus_2237_Transcript_1/1_Conf_1.000	399	20	8.68E-30	XP_001895262.1	90	133.265	66	60	Hypothetical UPF0222 protein Y54G11A.11 in chromosome II, putative
Locus_22370_Transcript_1/1_Conf_1.000	145	1	6.36E-04	NP_491653.2	65	47.3654	47	31	hypothetical protein F55F8.3
Locus_22371_Transcript_1/1_Conf_1.000	312	0							
Locus_22372_Transcript_1/1_Conf_1.000	172	6	5.05E-14	NP_490755.1	87	80.8777	54	47	Heavy chain, Unconventional Myosin family member (hum-7)
Locus_22373_Transcript_1/1_Conf_1.000	467	14	1.59E-15	NP_001024064.1	53	85.8853	123	66	hypothetical protein R02C2.7
Locus_22374_Transcript_1/1_Conf_1.000	165	9	4.44E-18	XP_002647278.1	87	94.3597	55	48	C. briggsae CBR-KETN-1 protein
Locus_22375_Transcript_1/1_Conf_1.000	322	0							
Locus_22376_Transcript_1/1_Conf_1.000	169	0							
Locus_22377_Transcript_1/1_Conf_1.000	198	20	3.65E-20	XP_002634787.1	80	101.293	65	52	C. briggsae CBR-NPP-20 protein
Locus_22378_Transcript_1/1_Conf_1.000	268	0							
Locus_22379_Transcript_1/1_Conf_1.000	172	0							
Locus_2238_Transcript_1/1_Conf_1.000	438	6	3.45E-23	XP_002646549.1	63	111.309	144	91	Hypothetical protein CBG20406
Locus_22380_Transcript_1/1_Conf_1.000	497	20	6.42E-102	NP_492020.1	99	372.859	165	164	hypothetical protein K10D3.4
Locus_22381_Transcript_1/1_Conf_1.000	134	0							
Locus_22382_Transcript_1/1_Conf_1.000	301	0							
Locus_22383_Transcript_1/1_Conf_1.000	256	20	4.83E-12	XP_002646299.1	63	74.3294	77	49	Hypothetical protein CBG12006

Locus_22384_Transcript_1/1_Conf_1.000	485	20	1.58E-55	ACD61693.1	81	218.779	164	133	UDP-galactose transporter-related protein
Locus_22385_Transcript_1/1_Conf_1.000	205	6	1.63E-12	XP_002639055.1	71	75.8702	70	50	C. briggsae CBR-GCY-28 protein
Locus_22386_Transcript_1/1_Conf_1.000	129	0							
Locus_22387_Transcript_1/1_Conf_1.000	236	0							
Locus_22388_Transcript_1/1_Conf_1.000	177	0							
Locus_22389_Transcript_1/1_Conf_1.000	450	3	1.93E-13	ACH87166.1	54	78.9518	142	78	GLO-3
Locus_2239_Transcript_1/1_Conf_1.000	898	20	3.82E-57	XP_001898424.1	68	226.098	241	165	DEAD/H
Locus_22390_Transcript_1/1_Conf_1.000	191	0							
Locus_22391_Transcript_1/1_Conf_1.000	173	0							
Locus_22392_Transcript_1/1_Conf_1.000	340	20	6.07E-36	NP_502129.1	84	153.68	113	95	hypothetical protein B0035.5
Locus_22393_Transcript_1/1_Conf_1.000	513	1	3.58E-05	XP_002640597.1	42	51.6026	168	71	C. briggsae CBR-DLK-1 protein
Locus_22394_Transcript_1/2_Conf_1.000	345	0							
Locus_22394_Transcript_2/2_Conf_1.000	345	0							
Locus_22395_Transcript_1/1_Conf_1.000	145	0							
Locus_22396_Transcript_1/1_Conf_1.000	377	0							
Locus_22397_Transcript_1/1_Conf_1.000	179	0							
Locus_22398_Transcript_1/1_Conf_1.000	137	0							
Locus_22399_Transcript_1/1_Conf_1.000	134	0							
Locus_224_Transcript_1/2_Conf_1.000	2197	20	0	NP_502350.1	80	701.049	543	439	hypothetical protein F19B6.1
Locus_224_Transcript_2/2_Conf_1.000	2197	20	0	NP_502350.1	80	701.049	543	439	hypothetical protein F19B6.1
Locus_2240_Transcript_1/1_Conf_1.000	708	0							
Locus_22400_Transcript_1/1_Conf_1.000	187	0							

Locus_22401_Transcript_1/1_Conf_1.000	133	20	2.68E-15	XP_002744859.1	100	85.1149	43	43	PREDICTED: L-lactate dehydrogenase B chain-like isoform 1
Locus_22402_Transcript_1/1_Conf_1.000	142	0							
Locus_22403_Transcript_1/1_Conf_1.000	188	20	1.27E-12	CAO72177.1	79	76.2554	62	49	hexosaminidase
Locus_22404_Transcript_1/1_Conf_1.000	144	0							
Locus_22405_Transcript_1/1_Conf_1.000	164	0							
Locus_22406_Transcript_1/1_Conf_1.000	310	0							
Locus_22407_Transcript_1/1_Conf_1.000	146	3	6.15E-07	XP_002633895.1	70	57.3806	48	34	C. briggsae CBR-SAND-1 protein
Locus_22408_Transcript_1/1_Conf_1.000	265	20	6.44E-17	EFO24933.1	75	90.5077	86	65	AGC/PKN protein kinase
Locus_22409_Transcript_1/1_Conf_1.000	284	0							
Locus_2241_Transcript_1/1_Conf_1.000	149	0							
Locus_22410_Transcript_1/1_Conf_1.000	296	0							
Locus_22411_Transcript_1/1_Conf_1.000	260	20	6.07E-15	XP_786956.2	64	83.9593	81	52	PREDICTED: similar to MGC140497 protein
Locus_22412_Transcript_1/1_Conf_1.000	206	0							
Locus_22413_Transcript_1/1_Conf_1.000	320	2	2.82E-12	XP_001893183.1	53	75.0998	115	61	Zinc finger, C2H2 type family protein
Locus_22414_Transcript_1/1_Conf_1.000	288	20	5.14E-14	XP_002635477.1	85	80.8777	48	41	Hypothetical protein CBG08772
Locus_22415_Transcript_1/1_Conf_1.000	130	0							
Locus_22416_Transcript_1/1_Conf_1.000	194	20	6.26E-20	NP_501657.1	84	100.523	64	54	hypothetical protein F56D5.3
Locus_22417_Transcript_1/1_Conf_1.000	743	20	6.27E-38	XP_002637600.1	67	161.77	179	120	C. briggsae CBR-SULP-8 protein
Locus_22418_Transcript_1/1_Conf_1.000	173	0							
Locus_22419_Transcript_1/1_Conf_1.000	282	0							
Locus_2242_Transcript_1/1_Conf_1.000	881	0							
Locus_22420_Transcript_1/1_Conf_1.000	160	20	3.66E-12	XP_002641552.1	80	74.7146	51	41	C. briggsae CBR-RBC-2 protein
Locus_22421_Transcript_1/1_Conf_1.000	317	3	1.77E-14	NP_492775.2	62	82.4185	108	67	LAMinin related. See also lmb- family member (lam-3)
Locus_22422_Transcript_1/1_Conf_1.000	251	0							
Locus_22423_Transcript_1/1_Conf_1.000	212	0							
Locus_22424_Transcript_1/1_Conf_1.000	236	20	3.77E-17	EFO15057.1	66	91.2781	77	51	DNA polymerase epsilon catalytic subunit
Locus_22425_Transcript_1/1_Conf_1.000	264	0							

Locus_22426_Transcript_1/1_Conf_1.000	252	0							
Locus_22427_Transcript_1/1_Conf_1.000	153	0							
Locus_22428_Transcript_1/1_Conf_1.000	179	0							
Locus_22429_Transcript_1/1_Conf_1.000	312	0							
Locus_2243_Transcript_1/4_Conf_0.154	715	8	6.01E-11	NP_741488.1	40	72.0182	227	93	abnormal cell LINeage family member (lin-3)
Locus_2243_Transcript_2/4_Conf_0.692	1505	9	3.75E-17	NP_741489.1	45	94.3597	246	111	abnormal cell LINeage family member (lin-3)
Locus_2243_Transcript_3/4_Conf_0.769	1505	9	3.75E-17	NP_741489.1	45	94.3597	246	111	abnormal cell LINeage family member (lin-3)
Locus_2243_Transcript_4/4_Conf_0.692	1505	9	3.75E-17	NP_741489.1	45	94.3597	246	111	abnormal cell LINeage family member (lin-3)
Locus_22430_Transcript_1/1_Conf_1.000	218	20	8.76E-35	XP_002917081.1	100	149.828	72	72	PREDICTED: alpha-actinin-1-like
Locus_22431_Transcript_1/1_Conf_1.000	182	0							
Locus_22432_Transcript_1/1_Conf_1.000	312	20	2.47E-32	NP_001040998.1	83	141.739	103	86	hypothetical protein W07G9.2
Locus_22433_Transcript_1/1_Conf_1.000	192	0							
Locus_22434_Transcript_1/1_Conf_1.000	512	20	1.85E-14	EFO23916.1	55	82.4185	177	98	transcription accessory protein
Locus_22435_Transcript_1/1_Conf_1.000	267	0							
Locus_22436_Transcript_1/1_Conf_1.000	328	0							
Locus_22437_Transcript_1/1_Conf_1.000	463	0							
Locus_22438_Transcript_1/1_Conf_1.000	129	0							
Locus_22439_Transcript_1/1_Conf_1.000	135	0							
Locus_2244_Transcript_1/1_Conf_1.000	443	20	1.88E-32	NP_871953.1	86	142.124	90	78	hypothetical protein T05H10.6
Locus_22440_Transcript_1/1_Conf_1.000	163	7	8.12E-20	XP_002644337.1	98	100.138	52	51	C. briggsae CBR-KLP-4 protein
Locus_22441_Transcript_1/1_Conf_1.000	185	20	2.03E-26	AAH05159.2	100	122.094	61	61	COL6A1 protein

Locus_22442_Transcript_1/1_Conf_1.000	319	0							
Locus_22443_Transcript_1/1_Conf_1.000	145	0							
Locus_22444_Transcript_1/1_Conf_1.000	141	0							
Locus_22445_Transcript_1/1_Conf_1.000	249	0							
Locus_22446_Transcript_1/1_Conf_1.000	241	20	3.61E-28	XP_002632693.1	83	127.872	80	67	Hypothetical protein CBG21624
Locus_22447_Transcript_1/1_Conf_1.000	308	0							
Locus_22448_Transcript_1/1_Conf_1.000	893	20	1.29E-89	NP_001040689.1	71	333.954	301	216	Prolyl tRNA Synthetase family member (prs-2)
Locus_22449_Transcript_1/1_Conf_1.000	218	0							
Locus_2245_Transcript_1/1_Conf_1.000	480	20	4.76E-73	XP_002756083.1	99	276.944	159	158	PREDICTED: fructose-bisphosphate aldolase A isoform 1
Locus_22450_Transcript_1/1_Conf_1.000	203	0							
Locus_22451_Transcript_1/1_Conf_1.000	130	0							
Locus_22452_Transcript_1/1_Conf_1.000	221	0							
Locus_22453_Transcript_1/1_Conf_1.000	139	0							
Locus_22454_Transcript_1/1_Conf_1.000	197	20	2.71E-15	XP_002635154.1	77	85.1149	62	48	C. briggsae CBR-VRS-1 protein
Locus_22455_Transcript_1/1_Conf_1.000	229	0							
Locus_22456_Transcript_1/1_Conf_1.000	202	0							
Locus_22457_Transcript_1/1_Conf_1.000	414	2	4.10E-16	NP_510476.2	75	87.8113	68	51	hypothetical protein Y70D2A.1
Locus_22458_Transcript_1/1_Conf_1.000	173	0							
Locus_22459_Transcript_1/1_Conf_1.000	425	0							
Locus_2246_Transcript_1/1_Conf_1.000	221	0							

Locus_22460_Transcript_1/1_Conf_1.000	319	1	5.15E-06	NP_492051.1	54	54.299	105	57	yeast BUB homolog family member (bub-1)
Locus_22461_Transcript_1/1_Conf_1.000	141	0							
Locus_22462_Transcript_1/1_Conf_1.000	145	20	2.26E-09	EFO26209.1	83	65.4698	43	36	hypothetical protein LOAG_02281
Locus_22463_Transcript_1/1_Conf_1.000	486	20	2.00E-34	XP_002641244.1	66	148.673	156	103	Hypothetical protein CBG09112
Locus_22464_Transcript_1/1_Conf_1.000	268	0							
Locus_22465_Transcript_1/1_Conf_1.000	204	3	5.08E-06	AAZ82850.2	67	54.299	62	42	Hypothetical protein C01B10.11
Locus_22466_Transcript_1/1_Conf_1.000	271	0							
Locus_22467_Transcript_1/2_Conf_1.000	256	0							
Locus_22467_Transcript_2/2_Conf_1.000	256	0							
Locus_22468_Transcript_1/1_Conf_1.000	270	0							
Locus_22469_Transcript_1/1_Conf_1.000	187	1	1.46E-08	EFO26588.1	70	62.7734	62	44	hypothetical protein LOAG_01892
Locus_2247_Transcript_1/1_Conf_1.000	500	20	1.94E-74	XP_001135183.1	99	281.567	138	137	PREDICTED: similar to ubiquitin A-52 residue ribosomal protein fusion product 1
Locus_22470_Transcript_1/1_Conf_1.000	271	0							
Locus_22471_Transcript_1/1_Conf_1.000	228	0							
Locus_22472_Transcript_1/1_Conf_1.000	321	0							
Locus_22473_Transcript_1/1_Conf_1.000	233	0							
Locus_22474_Transcript_1/1_Conf_1.000	483	0							
Locus_22475_Transcript_1/1_Conf_1.000	138	1	2.76E-04	NP_493647.1	70	48.521	44	31	hypothetical protein C23H3.5
Locus_22476_Transcript_1/1_Conf_1.000	295	0							

Locus_22477_Transcript_1/1_Conf_1.000	703	2	6.07E-08	XP_001898083.1	50	62.003	173	87	hypothetical protein
Locus_22478_Transcript_1/1_Conf_1.000	182	2	4.41E-05	XP_001897754.1	58	51.2174	62	36	Poly
Locus_22479_Transcript_1/1_Conf_1.000	260	0							
Locus_2248_Transcript_1/1_Conf_1.000	1524	20	1.32E-110	NP_495161.1	86	404.831	280	241	FZO (Fzo mitochondrial fusion protein) related family member (fzo-1)
Locus_22480_Transcript_1/1_Conf_1.000	189	0							
Locus_22481_Transcript_1/1_Conf_1.000	222	9	1.36E-06	XP_002648400.1	62	56.225	62	39	C. briggsae CBR-DPF-1 protein
Locus_22482_Transcript_1/1_Conf_1.000	137	0							
Locus_22483_Transcript_1/1_Conf_1.000	416	0							
Locus_22484_Transcript_1/1_Conf_1.000	203	2	5.63E-05	XP_002646533.1	64	50.8322	48	31	C. briggsae CBR-SPTF-3 protein
Locus_22485_Transcript_1/1_Conf_1.000	284	20	5.87E-48	NP_001034936.1	100	127.487	57	57	phospholipid hydroperoxide glutathione peroxidase, mitochondrial isoform B precursor
Locus_22486_Transcript_1/1_Conf_1.000	199	0							
Locus_22487_Transcript_1/1_Conf_1.000	487	5	7.64E-10	XP_002647866.1	55	67.0106	93	52	C. briggsae CBR-PRS-2 protein
Locus_22488_Transcript_1/1_Conf_1.000	255	2	4.67E-07	XP_001900629.1	63	57.7658	73	46	hypothetical protein Bm1_45825
Locus_22489_Transcript_1/1_Conf_1.000	185	0							
Locus_2249_Transcript_1/1_Conf_1.000	746	20	4.30E-103	NP_500125.1	94	378.252	219	207	Proteasome Beta Subunit family member (pbs-1)

Locus_22490_Transcript_1/1_Conf_1.000	282	20	2.04E-10	NP_502532.1	55	68.9366	79	44	SCP-Like extracellular protein family member (scl-14)
Locus_22491_Transcript_1/1_Conf_1.000	545	0							
Locus_22492_Transcript_1/1_Conf_1.000	433	20	1.31E-62	NP_505810.2	91	242.276	143	131	P21-Activated Kinase family member (pak-2)
Locus_22493_Transcript_1/1_Conf_1.000	138	0							
Locus_22494_Transcript_1/1_Conf_1.000	216	20	1.35E-35	XP_001164037.1	100	152.525	71	71	PREDICTED: similar to macrophage inflammatory protein 3 beta isoform 1
Locus_22495_Transcript_1/1_Conf_1.000	323	2	3.55E-07	NP_502859.2	62	58.151	69	43	hypothetical protein Y73F8A.24
Locus_22496_Transcript_1/1_Conf_1.000	206	5	9.25E-08	NP_505281.1	59	60.077	62	37	hypothetical protein F07B7.12
Locus_22497_Transcript_1/1_Conf_1.000	147	0							
Locus_22498_Transcript_1/1_Conf_1.000	234	0							
Locus_22499_Transcript_1/1_Conf_1.000	193	0							
Locus_225_Transcript_1/1_Conf_1.000	651	8	2.47E-111	NP_510149.1	93	405.216	216	203	hypothetical protein C34E7.4
Locus_2250_Transcript_1/1_Conf_1.000	480	0							
Locus_22500_Transcript_1/1_Conf_1.000	197	0							
Locus_22501_Transcript_1/1_Conf_1.000	198	5	8.40E-17	NP_499157.2	84	90.1225	65	55	hypothetical protein T05G5.8
Locus_22502_Transcript_1/1_Conf_1.000	129	0							
Locus_22503_Transcript_1/1_Conf_1.000	363	5	1.91E-05	XP_001945045.1	50	52.373	89	45	PREDICTED: similar to conserved hypothetical protein
Locus_22504_Transcript_1/1_Conf_1.000	236	0							
Locus_22505_Transcript_1/1_Conf_1.000	184	0							

Locus_22506_Transcript_1/1_Conf_1.000	135	20	1.73E-14	NP_496037.1	93	82.4185	44	41	Guanylyl CYclase family member (gcy-3)
Locus_22507_Transcript_1/1_Conf_1.000	164	0							
Locus_22508_Transcript_1/1_Conf_1.000	148	20	1.01E-09	NP_001122772.1	77	66.6254	45	35	Poly(ADP-ribose) Metabolism Enzyme family member (pme-3)
Locus_22509_Transcript_1/1_Conf_1.000	434	5	1.13E-05	NP_001021813.1	55	53.1434	89	49	abnormal cell LINeage family member (lin-17)
Locus_2251_Transcript_1/1_Conf_1.000	259	0							
Locus_22510_Transcript_1/1_Conf_1.000	410	20	2.95E-22	P34569.4	69	108.227	94	65	Kelch repeat-containing protein kel-10
Locus_22511_Transcript_1/1_Conf_1.000	185	20	3.70E-28	XP_002931439.1	100	127.872	61	61	PREDICTED: heat shock 70 kDa protein 1A/1B-like, partial
Locus_22512_Transcript_1/1_Conf_1.000	240	0							
Locus_22513_Transcript_1/1_Conf_1.000	141	0							
Locus_22514_Transcript_1/1_Conf_1.000	140	0							
Locus_22515_Transcript_1/1_Conf_1.000	249	0							
Locus_22516_Transcript_1/1_Conf_1.000	128	0							
Locus_22517_Transcript_1/1_Conf_1.000	185	0							
Locus_22518_Transcript_1/1_Conf_1.000	408	10	1.41E-32	XP_002645844.1	75	142.51	131	99	C. briggsae CBR-TSP-8 protein
Locus_22519_Transcript_1/1_Conf_1.000	363	1	2.11E-04	XP_002643891.1	49	48.9062	102	50	Hypothetical protein CBG02143

Locus_2252_Transcript_1/1_Conf_1.000	444	20	9.19E-64	EFO27339.1	90	246.128	148	134	dynein heavy chain protein 1
Locus_22520_Transcript_1/1_Conf_1.000	184	3	3.71E-12	NP_491803.1	87	74.7146	48	42	SaPosin-like Protein family member (spp-15)
Locus_22521_Transcript_1/1_Conf_1.000	284	2	8.27E-12	NP_001023948.1	62	73.559	70	44	hypothetical protein F48F5.6
Locus_22522_Transcript_1/1_Conf_1.000	413	3	1.05E-11	XP_002638813.1	61	73.1738	98	60	C. briggsae CBR-ATM-1 protein
Locus_22523_Transcript_1/1_Conf_1.000	150	20	2.02E-18	CAC43204.1	96	95.5153	50	48	immunoglobulin heavy chain variable region
Locus_22524_Transcript_1/1_Conf_1.000	176	20	1.42E-08	XP_002633529.1	70	62.7734	54	38	Hypothetical protein CBG05395
Locus_22525_Transcript_1/1_Conf_1.000	159	0							
Locus_22526_Transcript_1/1_Conf_1.000	186	0							
Locus_22527_Transcript_1/1_Conf_1.000	128	6	1.21E-07	EFO13288.1	80	59.6918	41	33	hypothetical protein LOAG_15242
Locus_22528_Transcript_1/1_Conf_1.000	208	20	1.24E-20	NP_496814.1	82	102.834	70	58	Member of AAA family binding CED-4 family member (mac-1)
Locus_22529_Transcript_1/1_Conf_1.000	293	0							
Locus_2253_Transcript_1/2_Conf_1.000	924	20	4.14E-86	NP_498620.1	84	322.398	227	191	hypothetical protein F08F8.7
Locus_2253_Transcript_2/2_Conf_1.000	904	20	3.97E-86	NP_498620.1	84	322.398	227	191	hypothetical protein F08F8.7
Locus_22530_Transcript_1/1_Conf_1.000	518	4	3.75E-42	XP_001898170.1	73	174.481	172	126	Ubiquitin-conjugating enzyme family protein
Locus_22531_Transcript_1/1_Conf_1.000	313	0							
Locus_22532_Transcript_1/1_Conf_1.000	567	20	7.96E-32	XP_002634048.1	59	140.584	197	118	Hypothetical protein CBG01587

Locus_22533_Transcript_1/1_Conf_1.000	174	20	1.36E-27	XP_614269.5	100	125.946	58	58	PREDICTED: filamin A, alpha
Locus_22534_Transcript_1/1_Conf_1.000	158	0							
Locus_22535_Transcript_1/1_Conf_1.000	222	3	1.23E-07	NP_502249.3	64	59.6918	73	47	TRPA cation channel homolog family member (trpa-1)
Locus_22536_Transcript_1/1_Conf_1.000	337	0							
Locus_22537_Transcript_1/1_Conf_1.000	466	20	2.07E-23	NP_001086849.1	66	112.079	154	102	dolichyl pyrophosphate Man9GlcNAc2 alpha-1,3-glucosyltransferase
Locus_22538_Transcript_1/1_Conf_1.000	136	0							
Locus_22539_Transcript_1/1_Conf_1.000	303	0							
Locus_2254_Transcript_1/1_Conf_1.000	714	9	7.10E-12	EFO26833.1	56	75.0998	117	66	hypothetical protein LOAG_01654
Locus_22540_Transcript_1/1_Conf_1.000	197	0							
Locus_22541_Transcript_1/1_Conf_1.000	255	0							
Locus_22542_Transcript_1/1_Conf_1.000	567	0							
Locus_22543_Transcript_1/1_Conf_1.000	359	5	4.71E-28	XP_002645442.1	72	127.487	115	83	C. briggsae CBR-TAG-241 protein
Locus_22544_Transcript_1/1_Conf_1.000	145	0							
Locus_22545_Transcript_1/1_Conf_1.000	437	20	9.36E-53	XP_002639554.1	83	209.534	148	123	Hypothetical protein CBG04185
Locus_22546_Transcript_1/1_Conf_1.000	442	0							
Locus_22547_Transcript_1/1_Conf_1.000	147	0							
Locus_22548_Transcript_1/1_Conf_1.000	162	20	5.26E-19	XP_002637506.1	86	97.4413	53	46	C. briggsae CBR-INX-10 protein
Locus_22549_Transcript_1/1_Conf_1.000	275	1	2.97E-06	NP_502390.2	52	55.0694	85	45	High Incidence of Males (increased X chromosome loss) family member (him-6)

Locus_2255_Transcript_1/1_Conf_1.000	1317	20	4.49E-157	NP_499188.2	89	558.91	346	309	Glycerol-3-Phosphate DeHydrogenase family member (gpdh-2)
Locus_22550_Transcript_1/1_Conf_1.000	208	3	1.25E-04	NP_491050.3	58	49.6766	73	43	InTestinal Neurexin-like family member (itx-1)
Locus_22551_Transcript_1/1_Conf_1.000	168	20	3.75E-17	NP_497990.1	83	91.2781	56	47	hypothetical protein C38D4.9
Locus_22552_Transcript_1/1_Conf_1.000	149	0							
Locus_22553_Transcript_1/1_Conf_1.000	129	0							
Locus_22554_Transcript_1/1_Conf_1.000	163	0							
Locus_22555_Transcript_1/1_Conf_1.000	280	6	9.14E-16	XP_002640853.1	62	86.6557	88	55	Hypothetical protein CBG15741
Locus_22556_Transcript_1/1_Conf_1.000	188	3	6.53E-09	NP_741241.1	66	63.929	60	40	Dipeptidyl Peptidase Four (IV) family member (dpf-6)
Locus_22557_Transcript_1/1_Conf_1.000	355	17	6.63E-14	EFO22315.1	60	80.4925	105	64	hypothetical protein LOAG_06169
Locus_22558_Transcript_1/1_Conf_1.000	145	0							
Locus_22559_Transcript_1/1_Conf_1.000	269	1	1.48E-05	ACI49192.1	59	52.7582	81	48	hypothetical protein Csp3_JD03.001
Locus_2256_Transcript_1/2_Conf_1.000	893	11	3.83E-09	XP_002733735.1	47	66.6254	138	65	PREDICTED: receptor for egg jelly 6-like
Locus_2256_Transcript_2/2_Conf_1.000	902	12	7.86E-10	NP_001021459.2	40	68.9366	188	76	hypothetical protein F36H2.3
Locus_22560_Transcript_1/1_Conf_1.000	283	20	1.88E-48	NP_492127.1	92	195.282	94	87	Low-density lipoprotein Receptor Related family member (lrp-1)
Locus_22561_Transcript_1/1_Conf_1.000	167	0							
Locus_22562_Transcript_1/1_Conf_1.000	258	0							
Locus_22563_Transcript_1/1_Conf_1.000	149	4	6.78E-06	XP_001894015.1	68	53.9138	47	32	hypothetical protein Bm1_12730
Locus_22564_Transcript_1/1_Conf_1.000	288	0							
Locus_22565_Transcript_1/1_Conf_1.000	150	0							
Locus_22566_Transcript_1/1_Conf_1.000	191	1	1.36E-06	XP_002643591.1	76	56.225	63	48	Hypothetical protein CBG16316
Locus_22567_Transcript_1/1_Conf_1.000	183	20	3.84E-09	NP_001023008.1	66	64.6994	57	38	CULlin family member (cul-2)

Locus_22568_Transcript_1/1_Conf_1.000	401	20	2.19E-57	NP_001022438.1	96	224.942	127	122	Ligand-Gated ion Channel family member (lgc-35)
Locus_22569_Transcript_1/1_Conf_1.000	164	0							
Locus_2257_Transcript_1/1_Conf_1.000	1700	20	5.45E-84	CAD44516.1	59	316.62	564	334	VAB-10B protein
Locus_22570_Transcript_1/1_Conf_1.000	276	0							
Locus_22571_Transcript_1/1_Conf_1.000	276	0							
Locus_22572_Transcript_1/1_Conf_1.000	332	0							
Locus_22573_Transcript_1/1_Conf_1.000	430	4	4.71E-36	NP_505948.2	72	154.066	143	104	hypothetical protein T27F2.2
Locus_22574_Transcript_1/1_Conf_1.000	271	0							
Locus_22575_Transcript_1/1_Conf_1.000	349	0							
Locus_22576_Transcript_1/1_Conf_1.000	185	20	8.52E-25	XP_002641621.1	90	116.701	61	55	Hypothetical protein CBG09941
Locus_22577_Transcript_1/1_Conf_1.000	219	0							
Locus_22578_Transcript_1/1_Conf_1.000	369	20	2.46E-24	NP_495529.1	73	115.161	91	67	SCP(Small C-terminal domain Phosphatase)-Like phosphatase family member (scpl-2)
Locus_22579_Transcript_1/1_Conf_1.000	611	20	2.05E-82	NP_497814.3	89	308.916	203	181	UNCoordinated family member (unc-79)
Locus_2258_Transcript_1/2_Conf_1.000	685	20	4.77E-55	ACX55058.1	92	218.394	127	117	glutathione peroxidase
Locus_2258_Transcript_2/2_Conf_1.000	679	20	1.45E-56	ACX55058.1	93	223.402	125	117	glutathione peroxidase
Locus_22580_Transcript_1/1_Conf_1.000	161	20	1.87E-24	EFO18724.1	98	115.546	53	52	voltage and ligand gated potassium channel
Locus_22581_Transcript_1/1_Conf_1.000	271	0							
Locus_22582_Transcript_1/1_Conf_1.000	146	0							
Locus_22583_Transcript_1/1_Conf_1.000	230	20	1.53E-34	XP_002710954.1	100	149.058	72	72	PREDICTED: small nuclear ribonucleoprotein polypeptide B/B-like
Locus_22584_Transcript_1/1_Conf_1.000	221	0							
Locus_22585_Transcript_1/1_Conf_1.000	283	0							
Locus_22586_Transcript_1/1_Conf_1.000	149	4	1.41E-11	XP_002644189.1	85	72.7886	49	42	Hypothetical protein CBG17172

Locus_22587_Transcript_1/1_Conf_1.000	287	11	1.66E-12	EFO24938.1	78	75.8702	87	68	hypothetical protein LOAG_03545
Locus_22588_Transcript_1/1_Conf_1.000	313	20	1.07E-35	NP_495634.1	84	152.91	104	88	hypothetical protein C06A8.6
Locus_22589_Transcript_1/1_Conf_1.000	293	20	3.65E-36	CAN70790.1	97	154.451	80	78	hypothetical protein
Locus_2259_Transcript_1/1_Conf_1.000	1021	0							
Locus_22590_Transcript_1/1_Conf_1.000	175	6	2.50E-13	EAW66059.1	100	78.5666	47	47	neural precursor cell expressed, developmentally down-regulated 8
Locus_22591_Transcript_1/1_Conf_1.000	166	0							
Locus_22592_Transcript_1/1_Conf_1.000	342	0							
Locus_22593_Transcript_1/1_Conf_1.000	246	20	1.78E-27	NP_502312.2	86	125.561	81	70	Neurexin Like receptor family member (nlr-1)
Locus_22594_Transcript_1/1_Conf_1.000	179	0							
Locus_22595_Transcript_1/1_Conf_1.000	138	0							
Locus_22596_Transcript_1/1_Conf_1.000	215	0							
Locus_22597_Transcript_1/1_Conf_1.000	284	4	1.16E-05	EFO13647.1	59	53.1434	67	40	hypothetical protein LOAG_14881
Locus_22598_Transcript_1/1_Conf_1.000	448	8	2.27E-38	XP_002636678.1	75	161.77	145	109	C. briggsae CBR-CHE-11 protein
Locus_22599_Transcript_1/1_Conf_1.000	131	20	5.24E-11	AAF82410.1	88	70.8626	43	38	AF221132_1diacylglycerol acyltransferase
Locus_226_Transcript_1/1_Conf_1.000	379	0							
Locus_2260_Transcript_1/2_Conf_1.000	609	20	7.94E-18	EFO17801.1	100	94.3597	47	47	hypothetical protein LOAG_10696
Locus_2260_Transcript_2/2_Conf_1.000	609	20	1.77E-17	EFO17801.1	97	93.2041	47	46	hypothetical protein LOAG_10696

Locus_22600_Transcript_1/1_Conf_1.000	137	19	6.35E-17	XP_002647760.1	97	90.5077	45	44	Hypothetical protein CBG20513
Locus_22601_Transcript_1/1_Conf_1.000	228	0							
Locus_22602_Transcript_1/1_Conf_1.000	238	0							
Locus_22603_Transcript_1/1_Conf_1.000	152	1	2.17E-04	XP_001896667.1	63	48.9062	46	29	Zinc finger, C2H2 type family protein
Locus_22604_Transcript_1/1_Conf_1.000	145	20	2.04E-10	EFO20586.1	80	68.9366	47	38	anion exchange protein
Locus_22605_Transcript_1/1_Conf_1.000	145	9	5.56E-08	NP_001020988.1	71	60.8474	45	32	UNCoordinated family member (unc-89)
Locus_22606_Transcript_1/1_Conf_1.000	184	0							
Locus_22607_Transcript_1/1_Conf_1.000	248	0							
Locus_22608_Transcript_1/1_Conf_1.000	202	0							
Locus_22609_Transcript_1/1_Conf_1.000	138	0							
Locus_2261_Transcript_1/1_Conf_1.000	444	20	3.52E-39	P27613.1	81	164.466	125	102	Globin-like host-protective antigen
Locus_22610_Transcript_1/1_Conf_1.000	301	2	7.09E-08	NP_499962.2	63	60.4622	88	56	hypothetical protein K11H12.9
Locus_22611_Transcript_1/1_Conf_1.000	164	20	1.87E-16	NP_506256.3	88	88.9669	54	48	CaDHerin family member (cdh-6)
Locus_22612_Transcript_1/1_Conf_1.000	249	2	2.94E-06	EFO23679.1	61	55.0694	94	58	hypothetical protein LOAG_04806
Locus_22613_Transcript_1/1_Conf_1.000	235	20	2.96E-38	BAG51482.1	100	161.384	78	78	unnamed protein product
Locus_22614_Transcript_1/1_Conf_1.000	142	0							
Locus_22615_Transcript_1/1_Conf_1.000	468	20	5.05E-46	NP_508419.1	76	187.193	139	107	hypothetical protein T10H10.2
Locus_22616_Transcript_1/1_Conf_1.000	158	0							
Locus_22617_Transcript_1/1_Conf_1.000	189	20	1.44E-24	NP_001024984.1	89	115.931	59	53	DVE (Defective proVEntriculus in Drosophila) homolog family member (dve-1)
Locus_22618_Transcript_1/1_Conf_1.000	349	20	2.53E-29	NP_510434.2	82	131.724	76	63	hypothetical protein F54E4.4
Locus_22619_Transcript_1/1_Conf_1.000	156	0							

Locus_2262_Transcript_1/1_Conf_1.000	3456	20	0	XP_001895813.1	61	806.594	1073	659	F/Y-rich N-terminus family protein
Locus_22620_Transcript_1/1_Conf_1.000	163	0							
Locus_22621_Transcript_1/1_Conf_1.000	328	0							
Locus_22622_Transcript_1/1_Conf_1.000	320	20	1.31E-17	NP_505966.1	82	92.8189	69	57	hypothetical protein T04F3.4
Locus_22623_Transcript_1/1_Conf_1.000	440	0							
Locus_22624_Transcript_1/1_Conf_1.000	167	0							
Locus_22625_Transcript_1/1_Conf_1.000	211	20	2.75E-28	XP_002831615.1	100	128.257	64	64	PREDICTED: LOW QUALITY PROTEIN: cell division protein kinase 16-like
Locus_22626_Transcript_1/1_Conf_1.000	401	0							
Locus_22627_Transcript_1/1_Conf_1.000	252	0							
Locus_22628_Transcript_1/1_Conf_1.000	195	0							
Locus_22629_Transcript_1/1_Conf_1.000	141	20	2.17E-17	XP_001110023.2	100	92.0485	46	46	PREDICTED: sentrin-specific protease 3-like isoform 3
Locus_2263_Transcript_1/1_Conf_1.000	219	0							
Locus_22630_Transcript_1/1_Conf_1.000	172	3	8.63E-06	NP_001122749.1	66	53.5286	57	38	hypothetical protein ZK643.3
Locus_22631_Transcript_1/1_Conf_1.000	196	20	2.45E-16	XP_426592.2	95	88.5817	42	40	PREDICTED: hypothetical protein
Locus_22632_Transcript_1/1_Conf_1.000	334	4	2.05E-07	XP_002642528.1	69	58.9214	66	46	Hypothetical protein CBG20151
Locus_22633_Transcript_1/1_Conf_1.000	316	20	2.23E-25	NP_510713.1	72	118.627	113	82	Cyclase ASsociated protein Homolog family member (cas-1)
Locus_22634_Transcript_1/1_Conf_1.000	406	5	1.01E-46	ACY39996.1	83	189.504	133	111	Hypothetical protein C30F12.5a
Locus_22635_Transcript_1/1_Conf_1.000	178	0							
Locus_22636_Transcript_1/1_Conf_1.000	229	0							
Locus_22637_Transcript_1/1_Conf_1.000	184	0							
Locus_22638_Transcript_1/1_Conf_1.000	155	0							
Locus_22639_Transcript_1/1_Conf_1.000	137	0							
Locus_2264_Transcript_1/1_Conf_1.000	462	0							
Locus_22640_Transcript_1/1_Conf_1.000	135	0							
Locus_22641_Transcript_1/1_Conf_1.000	168	0							

Locus_22642_Transcript_1/1_Conf_1.000	207	0							
Locus_22643_Transcript_1/1_Conf_1.000	387	0							
Locus_22644_Transcript_1/1_Conf_1.000	275	20	9.22E-08	XP_002585751.1	57	60.077	80	46	hypothetical protein BRAFLDRAFT_111201
Locus_22645_Transcript_1/1_Conf_1.000	145	0							
Locus_22646_Transcript_1/1_Conf_1.000	408	0							
Locus_22647_Transcript_1/1_Conf_1.000	196	2	3.93E-06	XP_001895789.1	66	54.6842	65	43	Timeless protein
Locus_22648_Transcript_1/1_Conf_1.000	365	5	1.61E-28	XP_002638930.1	87	129.028	121	106	C. briggsae CBR-CPX-1 protein
Locus_22649_Transcript_1/1_Conf_1.000	132	0							
Locus_2265_Transcript_1/1_Conf_1.000	1188	20	3.15E-66	XP_002429272.1	55	256.914	353	197	G-protein signaling modulator, putative
Locus_22650_Transcript_1/1_Conf_1.000	202	0							
Locus_22651_Transcript_1/1_Conf_1.000	187	0							
Locus_22652_Transcript_1/1_Conf_1.000	136	2	1.98E-10	NP_502231.1	81	68.9366	44	36	LiPocalin-Related protein family member (lpr-2)
Locus_22653_Transcript_1/1_Conf_1.000	176	0							
Locus_22654_Transcript_1/1_Conf_1.000	544	20	2.17E-65	NP_500551.2	86	251.906	172	148	hypothetical protein F55F10.1
Locus_22655_Transcript_1/1_Conf_1.000	134	0							
Locus_22656_Transcript_1/1_Conf_1.000	312	20	3.23E-32	EFO15665.1	85	141.354	89	76	hypothetical protein LOAG_12844
Locus_22657_Transcript_1/1_Conf_1.000	251	0							
Locus_22658_Transcript_1/1_Conf_1.000	166	20	3.88E-22	XP_002630655.1	96	107.842	55	53	C. briggsae CBR-MPK-2 protein
Locus_22659_Transcript_1/1_Conf_1.000	173	0							
Locus_2266_Transcript_1/1_Conf_1.000	2199	20	0	EFO25169.1	84	907.131	625	526	trehalose 6-phosphate synthase 1
Locus_22660_Transcript_1/1_Conf_1.000	160	20	6.04E-15	XP_002630143.1	88	83.9593	50	44	Hypothetical protein CBG00545
Locus_22661_Transcript_1/1_Conf_1.000	206	2	3.52E-07	XP_001895139.1	63	58.151	65	41	MGC84124 protein
Locus_22662_Transcript_1/1_Conf_1.000	285	0							

Locus_22663_Transcript_1/1_Conf_1.000	252	20	1.50E-29	CBW48349.1	85	132.494	84	72	Hypothetical protein C52E4.5b
Locus_22664_Transcript_1/1_Conf_1.000	175	0							
Locus_22665_Transcript_1/1_Conf_1.000	144	20	3.98E-14	NP_490693.2	86	81.2629	46	40	ATM (ataxia telangectasia mutated) family member (atm-1)
Locus_22666_Transcript_1/1_Conf_1.000	243	17	1.42E-08	XP_002634032.1	55	62.7734	85	47	Hypothetical protein CBG01569
Locus_22667_Transcript_1/1_Conf_1.000	184	0							
Locus_22668_Transcript_1/1_Conf_1.000	542	20	1.10E-53	Q60QP4.2	80	213.001	150	120	UPF0359 membrane protein CBG21730
Locus_22669_Transcript_1/1_Conf_1.000	226	0							
Locus_2267_Transcript_1/1_Conf_1.000	546	0							
Locus_22670_Transcript_1/1_Conf_1.000	313	0							
Locus_22671_Transcript_1/1_Conf_1.000	372	2	2.30E-14	NP_498398.1	71	82.0333	125	89	LEThal family member (let-716)
Locus_22672_Transcript_1/1_Conf_1.000	155	20	1.31E-09	XP_002633495.1	74	66.2402	51	38	Hypothetical protein CBG06267
Locus_22673_Transcript_1/1_Conf_1.000	328	0							
Locus_22674_Transcript_1/1_Conf_1.000	315	0							
Locus_22675_Transcript_1/1_Conf_1.000	158	0							
Locus_22676_Transcript_1/1_Conf_1.000	390	0							

Locus_22677_Transcript_1/1_Conf_1.000	218	20	8.80E-19	NP_508493.1	81	96.6709	64	52	protein KINase family member (kin-29)
Locus_22678_Transcript_1/1_Conf_1.000	313	0							
Locus_22679_Transcript_1/1_Conf_1.000	242	0							
Locus_2268_Transcript_1/1_Conf_1.000	809	2	1.11E-17	NP_509937.1	64	94.7449	156	100	hypothetical protein F52D10.2
Locus_22680_Transcript_1/1_Conf_1.000	313	0							
Locus_22681_Transcript_1/1_Conf_1.000	134	0							
Locus_22682_Transcript_1/1_Conf_1.000	379	20	2.88E-25	XP_001901557.1	66	118.242	113	75	Protein kinase domain containing protein
Locus_22683_Transcript_1/1_Conf_1.000	192	3	2.01E-18	XP_002633133.1	77	95.5153	68	53	C. briggsae CBR-GRL-4 protein
Locus_22684_Transcript_1/2_Conf_1.000	542	2	2.48E-05	NP_001122966.1	50	52.373	124	63	hypothetical protein F59A1.11
Locus_22684_Transcript_2/2_Conf_1.000	651	4	6.07E-09	NP_001122966.1	52	65.0846	153	80	hypothetical protein F59A1.11
Locus_22685_Transcript_1/1_Conf_1.000	280	0							
Locus_22686_Transcript_1/1_Conf_1.000	331	20	2.27E-14	EFO16615.1	68	82.0333	83	57	hypothetical protein LOAG_11891
Locus_22687_Transcript_1/1_Conf_1.000	353	0							
Locus_22688_Transcript_1/1_Conf_1.000	136	0							
Locus_22689_Transcript_1/1_Conf_1.000	157	0							
Locus_2269_Transcript_1/1_Conf_1.000	1064	20	1.38E-38	CAA86058.2	52	164.851	210	111	C. elegans protein B0393.5, partially confirmed by transcript evidence
Locus_22690_Transcript_1/1_Conf_1.000	128	5	5.09E-06	NP_001021995.1	76	54.299	42	32	hypothetical protein C27D6.11
Locus_22691_Transcript_1/1_Conf_1.000	129	20	9.55E-13	A8WRV1.2	83	76.6406	42	35	Serine/threonine-protein kinase kin-29
Locus_22692_Transcript_1/1_Conf_1.000	323	20	5.99E-47	CAR97828.1	88	190.274	106	94	C. elegans protein F44G4.8b, confirmed by transcript evidence

Locus_22693_Transcript_1/1_Conf_1.000	207	0							
Locus_22694_Transcript_1/1_Conf_1.000	182	0							
Locus_22695_Transcript_1/1_Conf_1.000	241	0							
Locus_22696_Transcript_1/1_Conf_1.000	162	0							
Locus_22697_Transcript_1/1_Conf_1.000	269	0							
Locus_22698_Transcript_1/1_Conf_1.000	299	0							
Locus_22699_Transcript_1/1_Conf_1.000	437	20	2.17E-33	NP_490828.3	73	145.206	123	91	HAlF transporter (PGP related) family member (haf-6)
Locus_227_Transcript_1/4_Conf_0.500	2580	20	0	NP_500329.1	74	724.931	707	528	mammalian ELKS/CAST/ERC/Rab6 interacting protein homolog family member (elks-1)
Locus_227_Transcript_2/4_Conf_0.400	842	3	3.13E-10	XP_002634742.1	63	70.0922	79	50	C. briggsae CBR-ELKS-1 protein
Locus_227_Transcript_3/4_Conf_0.700	3093	20	0	NP_500329.1	69	750.355	819	573	mammalian ELKS/CAST/ERC/Rab6 interacting protein homolog family member (elks-1)
Locus_227_Transcript_4/4_Conf_0.700	3093	20	0	NP_500329.1	69	750.355	819	573	mammalian ELKS/CAST/ERC/Rab6 interacting protein homolog family member (elks-1)
Locus_2270_Transcript_1/1_Conf_1.000	1071	0							
Locus_22700_Transcript_1/1_Conf_1.000	320	1	5.65E-29	CAR63539.1	77	130.568	102	79	hypothetical protein
Locus_22701_Transcript_1/1_Conf_1.000	344	0							
Locus_22702_Transcript_1/1_Conf_1.000	342	20	9.73E-13	XP_002647591.1	51	76.6406	95	49	Hypothetical protein CBG06679
Locus_22703_Transcript_1/1_Conf_1.000	154	0							
Locus_22704_Transcript_1/1_Conf_1.000	312	3	2.25E-09	NP_741925.2	62	65.4698	100	62	DYstrophin-like phenotype and CAPON related family member (dyc-1)
Locus_22705_Transcript_1/1_Conf_1.000	132	0							
Locus_22706_Transcript_1/1_Conf_1.000	261	20	8.75E-06	NP_001087602.1	58	53.5286	67	39	zinc finger, MIZ-type containing 2
Locus_22707_Transcript_1/1_Conf_1.000	237	20	1.74E-14	NP_741251.1	68	82.4185	76	52	hypothetical protein C02F5.6
Locus_22708_Transcript_1/1_Conf_1.000	270	3	1.63E-04	XP_001194422.1	52	49.2914	72	38	PREDICTED: similar to contactin associated protein
Locus_22709_Transcript_1/1_Conf_1.000	182	0							
Locus_2271_Transcript_1/1_Conf_1.000	819	20	6.76E-63	NP_001129866.1	71	244.973	212	152	Kinesin-Like Protein family member (klp-12)

Locus_22710_Transcript_1/1_Conf_1.000	431	20	2.98E-06	EFN51338.1	82	55.0694	39	32	hypothetical protein CHLNCDRAFT_141133
Locus_22711_Transcript_1/1_Conf_1.000	156	0							
Locus_22712_Transcript_1/1_Conf_1.000	423	14	4.17E-24	CAR63608.1	72	114.39	74	54	hypothetical protein
Locus_22713_Transcript_1/1_Conf_1.000	387	0							
Locus_22714_Transcript_1/1_Conf_1.000	373	20	2.22E-25	XP_518438.2	66	118.627	123	82	PREDICTED: copine V isoform 3
Locus_22715_Transcript_1/1_Conf_1.000	309	20	4.25E-05	XP_002108615.1	48	51.2174	72	35	hypothetical protein TRIADDRAFT_19102
Locus_22716_Transcript_1/1_Conf_1.000	161	0							
Locus_22717_Transcript_1/1_Conf_1.000	225	20	2.15E-28	EFO21576.1	86	128.642	74	64	hypothetical protein LOAG_06912
Locus_22718_Transcript_1/1_Conf_1.000	314	3	1.77E-22	NP_508833.1	86	108.997	67	58	hypothetical protein T07F12.4
Locus_22719_Transcript_1/1_Conf_1.000	180	0							
Locus_2272_Transcript_1/2_Conf_1.000	734	20	5.56E-15	ABH88210.1	65	85.5001	103	67	saposin-like protein 1
Locus_2272_Transcript_2/2_Conf_1.000	739	20	5.66E-15	ABH88210.1	65	85.5001	103	67	saposin-like protein 1
Locus_22720_Transcript_1/1_Conf_1.000	554	20	1.01E-28	XP_002630751.1	63	130.183	133	85	Hypothetical protein CBG02443

Locus_22721_Transcript_1/1_Conf_1.000	270	20	1.84E-48	XP_002803216.1	100	195.282	89	89	PREDICTED: amyloid beta A4 protein-like, partial
Locus_22722_Transcript_1/1_Conf_1.000	361	20	8.87E-19	XP_001900618.1	63	96.6709	117	74	Leucine Rich Repeat family protein
Locus_22723_Transcript_1/1_Conf_1.000	177	20	2.25E-14	CAR63659.1	82	82.0333	57	47	putative Collagen protein 20
Locus_22724_Transcript_1/1_Conf_1.000	242	0							
Locus_22725_Transcript_1/1_Conf_1.000	521	0							
Locus_22726_Transcript_1/1_Conf_1.000	165	0							
Locus_22727_Transcript_1/1_Conf_1.000	177	0							
Locus_22728_Transcript_1/1_Conf_1.000	242	6	3.88E-06	XP_002630353.1	61	54.6842	57	35	Hypothetical protein CBG04283
Locus_22729_Transcript_1/1_Conf_1.000	718	3	1.75E-34	NP_498060.2	57	150.214	244	140	hypothetical protein R144.5
Locus_2273_Transcript_1/1_Conf_1.000	1338	20	9.65E-14	XP_002664350.1	69	82.8037	59	41	PREDICTED: hypothetical protein LOC322903
Locus_22730_Transcript_1/1_Conf_1.000	128	20	2.88E-09	NP_001024131.1	76	65.0846	42	32	Peroxisomal Membrane Protein related family member (pmp-5)
Locus_22731_Transcript_1/1_Conf_1.000	128	0							

Locus_22732_Transcript_1/1_Conf_1.000	148	20	7.72E-18	XP_002823344.1	100	93.5893	49	49	PREDICTED: keratin, type I cytoskeletal 18-like
Locus_22733_Transcript_1/1_Conf_1.000	228	20	9.32E-24	AAQ06435.1	96	113.235	76	73	ABC6 protein
Locus_22734_Transcript_1/1_Conf_1.000	157	0							
Locus_22735_Transcript_1/1_Conf_1.000	203	2	3.90E-06	EFO23663.1	61	54.6842	68	42	myosin xviiia
Locus_22736_Transcript_1/1_Conf_1.000	194	0							
Locus_22737_Transcript_1/2_Conf_1.000	303	20	6.58E-22	XP_002632609.1	72	107.071	100	72	C. briggsae CBR-COQ-3 protein
Locus_22737_Transcript_2/2_Conf_1.000	309	20	2.10E-20	XP_002632609.1	70	102.064	102	72	C. briggsae CBR-COQ-3 protein
Locus_22738_Transcript_1/1_Conf_1.000	640	20	2.11E-27	NP_509145.2	68	126.331	104	71	AuTophaGy (yeast Atg homolog) family member (atg-2)
Locus_22739_Transcript_1/1_Conf_1.000	504	20	3.49E-58	XP_001894372.1	75	227.639	180	136	Immunoglobulin I-set domain containing protein
Locus_2274_Transcript_1/1_Conf_1.000	1028	20	4.97E-46	XP_001893845.1	57	189.504	303	173	Mov34/MPN/PAD-1 family protein
Locus_22740_Transcript_1/1_Conf_1.000	220	0							
Locus_22741_Transcript_1/1_Conf_1.000	368	0							
Locus_22742_Transcript_1/1_Conf_1.000	135	0							
Locus_22743_Transcript_1/1_Conf_1.000	525	20	2.48E-76	XP_002644087.1	90	288.115	173	157	Hypothetical protein CBG17560
Locus_22744_Transcript_1/1_Conf_1.000	172	0							

Locus_22745_Transcript_1/1_Conf_1.000	330	20	6.61E-14	XP_002643183.1	60	80.4925	98	59	Hypothetical protein CBG24152
Locus_22746_Transcript_1/1_Conf_1.000	168	0							
Locus_22747_Transcript_1/1_Conf_1.000	381	20	3.74E-25	XP_002639036.1	75	117.857	122	92	C. briggsae CBR-DYB-1 protein
Locus_22748_Transcript_1/1_Conf_1.000	178	0							
Locus_22749_Transcript_1/1_Conf_1.000	138	0							
Locus_2275_Transcript_1/2_Conf_1.000	566	6	4.17E-17	XP_002633345.1	52	91.6633	163	86	Hypothetical protein CBG06087
Locus_2275_Transcript_2/2_Conf_1.000	560	6	2.36E-17	XP_002633345.1	53	92.4337	161	86	Hypothetical protein CBG06087
Locus_22750_Transcript_1/1_Conf_1.000	137	0							
Locus_22751_Transcript_1/1_Conf_1.000	355	20	4.44E-18	EFO24692.1	65	94.3597	120	79	protein-tyrosine phosphatase
Locus_22752_Transcript_1/1_Conf_1.000	139	1	2.75E-12	NP_001122963.1	86	75.0998	46	40	hypothetical protein F58E6.1
Locus_22753_Transcript_1/1_Conf_1.000	216	0							
Locus_22754_Transcript_1/1_Conf_1.000	448	0							
Locus_22755_Transcript_1/1_Conf_1.000	148	0							
Locus_22756_Transcript_1/1_Conf_1.000	196	0							
Locus_22757_Transcript_1/1_Conf_1.000	198	0							
Locus_22758_Transcript_1/1_Conf_1.000	245	0							
Locus_22759_Transcript_1/1_Conf_1.000	207	20	8.57E-30	XP_002818459.1	100	133.265	67	67	PREDICTED: hypothetical protein LOC100441808
Locus_2276_Transcript_1/4_Conf_0.500	854	20	1.64E-38	AAG36874.1	76	164.081	178	136	AF242767_1SF2
Locus_2276_Transcript_3/4_Conf_0.375	566	9	7.36E-14	AAG36874.1	69	80.8777	134	93	AF242767_1SF2

Locus_2276_Transcript_4/4_Conf_0.500	970	20	2.03E-38	AAG36874.1	76	164.081	178	136	AF242767_1SF2
Locus_22760_Transcript_1/1_Conf_1.000	180	0							
Locus_22761_Transcript_1/1_Conf_1.000	188	2	2.18E-04	NP_500597.1	62	48.9062	35	22	hypothetical protein F19C7.1
Locus_22762_Transcript_1/1_Conf_1.000	165	0							
Locus_22763_Transcript_1/1_Conf_1.000	319	20	2.72E-15	XP_002641345.1	63	85.1149	103	65	Hypothetical protein CBG13198
Locus_22764_Transcript_1/1_Conf_1.000	179	1	1.46E-05	AAG43143.1	100	52.7582	53	53	AF061732_1My029 protein
Locus_22765_Transcript_1/1_Conf_1.000	179	1	2.76E-04	XP_001895772.1	59	48.521	57	34	hypothetical protein Bm1_21590
Locus_22766_Transcript_1/1_Conf_1.000	166	0							
Locus_22767_Transcript_1/1_Conf_1.000	686	20	4.64E-34	NP_495011.1	87	148.673	90	79	Protein Kinase C family member (pkc-3)
Locus_22768_Transcript_1/1_Conf_1.000	423	1	1.54E-10	EFO21344.1	56	69.3218	91	51	hypothetical protein LOAG_07145
Locus_22769_Transcript_1/1_Conf_1.000	210	0							
Locus_2277_Transcript_1/1_Conf_1.000	1707	20	9.80E-158	XP_002640778.1	74	561.607	491	365	C. briggsae CBR-FRL-1 protein

Locus_22770_Transcript_1/1_Conf_1.000	361	20	1.45E-45	EFO25590.1	84	185.652	120	101	hypothetical protein LOAG_02898
Locus_22771_Transcript_1/1_Conf_1.000	147	0							
Locus_22772_Transcript_1/1_Conf_1.000	232	0							
Locus_22773_Transcript_1/1_Conf_1.000	353	0							
Locus_22774_Transcript_1/1_Conf_1.000	298	20	2.06E-23	XP_002427603.1	84	112.079	88	74	mitochondrial 2-oxoglutarate/malate carrier protein, putative
Locus_22775_Transcript_1/1_Conf_1.000	311	20	2.66E-07	XP_001897863.1	76	58.5362	56	43	rab6
Locus_22776_Transcript_1/1_Conf_1.000	403	3	2.60E-26	NP_500870.2	80	121.709	112	90	hypothetical protein C06E7.2
Locus_22777_Transcript_1/1_Conf_1.000	280	0							
Locus_22778_Transcript_1/1_Conf_1.000	325	0							
Locus_22779_Transcript_1/1_Conf_1.000	153	20	9.37E-16	EFO27858.1	90	86.6557	50	45	hypothetical protein LOAG_00620
Locus_2278_Transcript_1/1_Conf_1.000	587	0							
Locus_22780_Transcript_1/1_Conf_1.000	179	0							
Locus_22781_Transcript_1/1_Conf_1.000	583	20	1.20E-33	NP_500530.1	61	146.747	191	118	Nuclear Hormone Receptor family member (nhr-78)
Locus_22782_Transcript_1/1_Conf_1.000	170	3	3.29E-05	AAA82423.2	62	51.6026	58	36	Hypothetical protein F10E7.9
Locus_22783_Transcript_1/1_Conf_1.000	180	0							

Locus_22784_Transcript_1/1_Conf_1.000	131	0							
Locus_22785_Transcript_1/1_Conf_1.000	228	20	6.68E-22	NP_497205.1	89	107.071	75	67	UNCoordinated family member (unc-45)
Locus_22786_Transcript_1/1_Conf_1.000	404	20	3.76E-25	XP_002639857.1	68	117.857	134	92	Hypothetical protein CBG12210
Locus_22787_Transcript_1/1_Conf_1.000	203	0							
Locus_22788_Transcript_1/2_Conf_1.000	320	0							
Locus_22788_Transcript_2/2_Conf_1.000	294	0							
Locus_22789_Transcript_1/1_Conf_1.000	302	0							
Locus_2279_Transcript_1/1_Conf_1.000	1312	20	1.47E-176	XP_002639838.1	87	623.624	401	351	Hypothetical protein CBG12186
Locus_22790_Transcript_1/1_Conf_1.000	141	0							
Locus_22791_Transcript_1/1_Conf_1.000	134	0							
Locus_22792_Transcript_1/1_Conf_1.000	240	20	6.15E-28	NP_001122569.1	86	127.102	81	70	hypothetical protein ZK849.2
Locus_22793_Transcript_1/1_Conf_1.000	304	20	3.58E-52	EAW85022.1	100	207.608	100	100	px19-like protein, isoform CRA_b
Locus_22794_Transcript_1/1_Conf_1.000	326	0							
Locus_22795_Transcript_1/1_Conf_1.000	188	5	5.18E-06	CBK19511.1	68	54.299	47	32	C. elegans protein Y67H2A.10b, partially confirmed by transcript evidence
Locus_22796_Transcript_1/1_Conf_1.000	289	0							
Locus_22797_Transcript_1/1_Conf_1.000	182	5	5.04E-09	CAE47474.2	72	64.3142	54	39	C. elegans protein Y79H2A.3b, partially confirmed by transcript evidence
Locus_22798_Transcript_1/1_Conf_1.000	423	20	7.86E-23	XP_002630155.1	58	110.153	154	90	Hypothetical protein CBG00558
Locus_22799_Transcript_1/1_Conf_1.000	256	3	5.68E-29	NP_493358.1	90	130.568	83	75	hypothetical protein C01A2.2
Locus_228_Transcript_1/1_Conf_1.000	1269	20	3.77E-105	ACO15793.1	85	386.341	253	217	Hypothetical protein Y71G12B.23a
Locus_2280_Transcript_1/1_Conf_1.000	1611	20	2.29E-177	XP_002633188.1	78	626.706	449	354	C. briggsae CBR-PAM-1 protein
Locus_22800_Transcript_1/1_Conf_1.000	194	0							
Locus_22801_Transcript_1/1_Conf_1.000	172	20	1.20E-23	AAW29117.1	96	112.849	57	55	AF1 non-allergic IgE heavy chain VH3-30-3

Locus_22802_Transcript_1/1_Conf_1.000	364	20	1.08E-16	NP_506711.1	58	89.7373	127	74	DNaJ domain (prokaryotic heat shock protein) family member (dnj-3)
Locus_22803_Transcript_1/1_Conf_1.000	297	20	1.17E-18	EFO22690.1	80	96.2857	62	50	4-hydroxybenzoate polyprenyl transferase
Locus_22804_Transcript_1/1_Conf_1.000	140	0							
Locus_22805_Transcript_1/1_Conf_1.000	409	0							
Locus_22806_Transcript_1/1_Conf_1.000	355	4	1.25E-04	ACP31595.1	62	49.6766	72	45	Hypothetical protein Y32H12A.2b
Locus_22807_Transcript_1/1_Conf_1.000	175	0							
Locus_22808_Transcript_1/1_Conf_1.000	317	0							
Locus_22809_Transcript_1/1_Conf_1.000	187	0							
Locus_2281_Transcript_1/1_Conf_1.000	2804	20	0	XP_002644134.1	81	1070.84	835	684	C. briggsae CBR-SYD-2 protein
Locus_22810_Transcript_1/1_Conf_1.000	190	0							
Locus_22811_Transcript_1/1_Conf_1.000	261	2	5.48E-08	NP_505736.2	75	60.8474	48	36	yeast MRE recombination/repair homolog family member (mre-11)
Locus_22812_Transcript_1/1_Conf_1.000	168	0							
Locus_22813_Transcript_1/1_Conf_1.000	354	11	1.48E-13	EFO21218.1	97	79.337	40	39	CAMK/CAMKL/NUAK protein kinase
Locus_22814_Transcript_1/1_Conf_1.000	195	0							
Locus_22815_Transcript_1/1_Conf_1.000	146	0							
Locus_22816_Transcript_1/1_Conf_1.000	183	0							
Locus_22817_Transcript_1/1_Conf_1.000	377	20	3.55E-07	XP_700974.4	60	58.151	65	39	PREDICTED: transmembrane protein 67
Locus_22818_Transcript_1/1_Conf_1.000	134	0							
Locus_22819_Transcript_1/1_Conf_1.000	350	0							

Locus_2282_Transcript_1/1_Conf_1.000	278	20	1.90E-37	ABC40752.1	94	158.688	88	83	myosin heavy chain
Locus_22820_Transcript_1/1_Conf_1.000	434	20	1.27E-49	AAH13586.2	100	199.134	108	108	PRKCSH protein
Locus_22821_Transcript_1/1_Conf_1.000	130	4	1.43E-08	NP_490840.2	87	62.7734	41	36	DAP (Death-Associated Protein) Kinase homolog family member (dapk-1)
Locus_22822_Transcript_1/1_Conf_1.000	180	0							
Locus_22823_Transcript_1/1_Conf_1.000	364	0							
Locus_22824_Transcript_1/1_Conf_1.000	246	8	6.32E-25	XP_002634052.1	87	117.087	82	72	C. briggsae CBR-VHA-5 protein
Locus_22825_Transcript_1/1_Conf_1.000	176	0							
Locus_22826_Transcript_1/1_Conf_1.000	129	0							
Locus_22827_Transcript_1/1_Conf_1.000	346	20	4.78E-36	NP_001022673.1	85	154.066	111	95	NADC (Na+-coupled dicarboxylate transporter) family member (nac-3)
Locus_22828_Transcript_1/1_Conf_1.000	259	20	3.66E-36	XP_002632226.1	90	154.451	85	77	Hypothetical protein CBG07097
Locus_22829_Transcript_1/1_Conf_1.000	295	20	1.53E-34	ADL62853.1	84	149.058	98	83	thioredoxin reductase 2

Locus_2283_Transcript_1/1_Conf_1.000	241	20	1.56E-31	XP_002636221.1	88	139.043	80	71	Hypothetical protein CBG12142
Locus_22830_Transcript_1/1_Conf_1.000	309	20	1.19E-23	NP_500418.1	70	112.849	96	68	hypothetical protein M02B7.2
Locus_22831_Transcript_1/1_Conf_1.000	219	0							
Locus_22832_Transcript_1/1_Conf_1.000	224	20	8.48E-25	NP_001022123.1	86	116.701	76	66	hypothetical protein F28C6.4
Locus_22833_Transcript_1/1_Conf_1.000	205	0							
Locus_22834_Transcript_1/1_Conf_1.000	1423	20	1.44E-164	XP_002636960.1	82	583.948	439	362	Hypothetical protein CBG09439
Locus_22835_Transcript_1/1_Conf_1.000	141	0							
Locus_22836_Transcript_1/1_Conf_1.000	199	0							
Locus_22837_Transcript_1/1_Conf_1.000	232	0							
Locus_22838_Transcript_1/1_Conf_1.000	154	0							
Locus_22839_Transcript_1/1_Conf_1.000	417	0							
Locus_2284_Transcript_1/2_Conf_0.500	3023	20	0	XP_002635006.1	86	1407.89	935	811	Hypothetical protein CBG13546
Locus_2284_Transcript_2/2_Conf_0.500	925	20	2.42E-102	XP_001900408.1	98	376.326	194	192	GTP-binding nuclear protein RAN/TC4

Locus_22840_Transcript_1/1_Conf_1.000	263	20	2.70E-39	XP_002633776.1	89	164.851	87	78	C. briggsae CBR-NHR-4 protein
Locus_22841_Transcript_1/1_Conf_1.000	280	0							
Locus_22842_Transcript_1/1_Conf_1.000	257	0							
Locus_22843_Transcript_1/1_Conf_1.000	201	0							
Locus_22844_Transcript_1/1_Conf_1.000	246	20	3.46E-31	XP_001093980.2	100	137.887	60	60	PREDICTED: hypothetical protein LOC705622
Locus_22845_Transcript_1/1_Conf_1.000	282	1	9.79E-05	EFO22051.1	58	50.0618	78	46	hypothetical protein LOAG_06433
Locus_22846_Transcript_1/1_Conf_1.000	140	4	2.41E-08	XP_001901959.1	79	62.003	39	31	hypothetical protein
Locus_22847_Transcript_1/1_Conf_1.000	324	0							
Locus_22848_Transcript_1/1_Conf_1.000	160	20	2.70E-23	XP_002810196.1	100	111.694	53	53	PREDICTED: protein S100-A4-like isoform 2
Locus_22849_Transcript_1/1_Conf_1.000	281	0							
Locus_2285_Transcript_1/1_Conf_1.000	212	0							
Locus_22850_Transcript_1/1_Conf_1.000	653	7	2.62E-28	NP_498592.1	55	129.413	216	119	hypothetical protein R151.8
Locus_22851_Transcript_1/1_Conf_1.000	187	0							
Locus_22852_Transcript_1/1_Conf_1.000	275	0							
Locus_22853_Transcript_1/1_Conf_1.000	338	0							
Locus_22854_Transcript_1/1_Conf_1.000	133	0							
Locus_22855_Transcript_1/1_Conf_1.000	204	0							
Locus_22856_Transcript_1/1_Conf_1.000	138	0							
Locus_22857_Transcript_1/1_Conf_1.000	235	0							
Locus_22858_Transcript_1/1_Conf_1.000	523	20	8.72E-42	AAO18224.1	95	173.326	92	88	FMRFamide-like prepropeptide

Locus_22859_Transcript_1/1_Conf_1.000	388	20	1.14E-58	XP_002637345.1	92	229.18	128	118	C. briggsae CBR-UNC-68 protein
Locus_2286_Transcript_1/1_Conf_1.000	660	4	7.26E-26	NP_501779.1	52	121.324	198	103	hypothetical protein F32B6.4
Locus_22860_Transcript_1/1_Conf_1.000	199	0							
Locus_22861_Transcript_1/1_Conf_1.000	610	0							
Locus_22862_Transcript_1/1_Conf_1.000	297	0							
Locus_22863_Transcript_1/1_Conf_1.000	209	20	6.58E-22	XP_001899774.1	88	107.071	68	60	Guanine nucleotide exchange factor for Ras-like GTPases; N-terminal motif family protein
Locus_22864_Transcript_1/1_Conf_1.000	360	20	4.23E-45	NP_490690.1	86	184.111	104	90	hypothetical protein F56C11.3
Locus_22865_Transcript_1/1_Conf_1.000	161	0							
Locus_22866_Transcript_1/1_Conf_1.000	182	9	3.05E-14	NP_001020988.1	79	81.6481	62	49	UNCoordinated family member (unc-89)
Locus_22867_Transcript_1/1_Conf_1.000	241	0							
Locus_22868_Transcript_1/1_Conf_1.000	339	2	1.15E-10	NP_500240.1	91	69.707	36	33	hypothetical protein Y69A2AR.31
Locus_22869_Transcript_1/1_Conf_1.000	278	8	9.15E-24	XP_002639434.1	79	113.235	91	72	Hypothetical protein CBG04027
Locus_2287_Transcript_1/4_Conf_0.625	1135	20	2.06E-43	NP_506352.1	82	181.03	137	113	TransThyretin-Related family domain family member (ttr-44)
Locus_2287_Transcript_2/4_Conf_0.625	1094	20	1.94E-43	NP_506352.1	82	181.03	137	113	TransThyretin-Related family domain family member (ttr-44)
Locus_2287_Transcript_3/4_Conf_0.375	532	20	2.21E-51	EFO24957.1	84	205.297	122	103	hypothetical protein LOAG_03532
Locus_2287_Transcript_4/4_Conf_0.625	1135	20	2.06E-43	NP_506352.1	82	181.03	137	113	TransThyretin-Related family domain family member (ttr-44)
Locus_22870_Transcript_1/1_Conf_1.000	173	20	5.20E-27	XP_002825204.1	100	124.02	57	57	PREDICTED: apoptosis regulatory protein Siva-like
Locus_22871_Transcript_1/1_Conf_1.000	191	0							

Locus_22872_Transcript_1/1_Conf_1.000	161	20	7.87E-23	NP_001024865.1	100	110.153	53	53	DumPY : shorter than wild-type family member (dpy-23)
Locus_22873_Transcript_1/1_Conf_1.000	217	7	9.13E-08	NP_502367.2	69	60.077	59	41	hypothetical protein F28D1.9
Locus_22874_Transcript_1/1_Conf_1.000	191	0							
Locus_22875_Transcript_1/1_Conf_1.000	136	0							
Locus_22876_Transcript_1/1_Conf_1.000	188	5	7.99E-07	NP_504937.3	57	56.9954	56	32	hypothetical protein C54F6.6
Locus_22877_Transcript_1/1_Conf_1.000	305	20	1.57E-23	ABD75715.1	95	112.464	80	76	vacuolar protein sorting factor
Locus_22878_Transcript_1/1_Conf_1.000	142	0							
Locus_22879_Transcript_1/1_Conf_1.000	242	20	6.60E-14	CAA96571.1	74	80.4925	79	59	parasite pepsinogen
Locus_2288_Transcript_1/1_Conf_1.000	1890	20	0	NP_499949.2	82	783.097	558	459	Gut on EXterior family member (gex-2)
Locus_22880_Transcript_1/1_Conf_1.000	324	0							
Locus_22881_Transcript_1/1_Conf_1.000	159	0							
Locus_22882_Transcript_1/1_Conf_1.000	265	20	4.32E-13	NP_001024930.1	68	77.7962	79	54	Choline Kinase C family member (ckc-1)
Locus_22883_Transcript_1/1_Conf_1.000	152	0							
Locus_22884_Transcript_1/1_Conf_1.000	230	20	2.45E-24	NP_872193.1	80	115.161	76	61	hypothetical protein K07C11.7
Locus_22885_Transcript_1/1_Conf_1.000	161	0							
Locus_22886_Transcript_1/1_Conf_1.000	364	20	9.15E-16	NP_508641.2	82	86.6557	73	60	Temporarily Assigned Gene name family member (tag-81)
Locus_22887_Transcript_1/1_Conf_1.000	243	0							
Locus_22888_Transcript_1/1_Conf_1.000	172	0							
Locus_22889_Transcript_1/1_Conf_1.000	217	0							

Locus_2289_Transcript_1/4_Conf_0.700	2494	20	0	EFO23774.1	80	902.123	740	594	replication licensing factor MCM7
Locus_2289_Transcript_2/4_Conf_0.700	2494	20	0	EFO23774.1	80	902.123	740	594	replication licensing factor MCM7

Locus_2289_Transcript_3/4_Conf_0.700	2494	20	0	EFO23774.1	80	901.353	740	593	replication licensing factor MCM7
Locus_2289_Transcript_4/4_Conf_0.700	2494	20	0	EFO23774.1	80	902.123	740	594	replication licensing factor MCM7
Locus_22890_Transcript_1/1_Conf_1.000	431	0							
Locus_22891_Transcript_1/1_Conf_1.000	145	1	6.78E-14	NP_001021158.1	83	80.4925	48	40	UDP-GlucuronosylTransferase family member (ugt-60)
Locus_22892_Transcript_1/1_Conf_1.000	499	0							
Locus_22893_Transcript_1/1_Conf_1.000	206	0							
Locus_22894_Transcript_1/1_Conf_1.000	151	0							
Locus_22895_Transcript_1/1_Conf_1.000	203	0							
Locus_22896_Transcript_1/1_Conf_1.000	248	0							
Locus_22897_Transcript_1/1_Conf_1.000	137	11	4.40E-18	CAH73152.1	100	94.3597	45	45	golgi membrane protein 1
Locus_22898_Transcript_1/1_Conf_1.000	339	0							
Locus_22899_Transcript_1/1_Conf_1.000	354	10	1.25E-12	XP_001895198.1	57	76.2554	111	64	PAZ domain containing protein

Locus_229_Transcript_1/1_Conf_1.000	1740	20	0	XP_002642779.1	94	870.922	511	482	C. briggsae CBR-ATP-2 protein
Locus_2290_Transcript_1/1_Conf_1.000	613	20	2.94E-44	ACD88894.1	73	182.185	138	102	transthyretin-like protein
Locus_22900_Transcript_1/1_Conf_1.000	148	20	7.73E-10	NP_001122772.1	77	67.0106	45	35	Poly(ADP-ribose) Metabolism Enzyme family member (pme-3)
Locus_22901_Transcript_1/1_Conf_1.000	182	0							
Locus_22902_Transcript_1/1_Conf_1.000	257	20	6.97E-11	NP_494994.1	70	70.4774	60	42	hypothetical protein ZK622.1
Locus_22903_Transcript_1/1_Conf_1.000	190	2	7.20E-08	NP_498149.1	69	60.4622	63	44	hypothetical protein Y32H12A.6
Locus_22904_Transcript_1/1_Conf_1.000	420	0							
Locus_22905_Transcript_1/1_Conf_1.000	150	0							
Locus_22906_Transcript_1/1_Conf_1.000	306	20	4.23E-37	1UH6	98	157.532	75	74	SolutionStructure OfThe Murine Ubiquitin-Like 5 Protein From Riken Cdna 0610031k06
Locus_22907_Transcript_1/1_Conf_1.000	243	0							
Locus_22908_Transcript_1/1_Conf_1.000	147	16	1.08E-19	XP_002831355.1	100	99.7525	48	48	PREDICTED: kelch domain-containing protein 7B-like
Locus_22909_Transcript_1/1_Conf_1.000	360	20	2.12E-12	XP_002402660.1	59	75.485	77	46	engulfment and cell motility, putative
Locus_2291_Transcript_1/1_Conf_1.000	620	19	1.93E-83	XP_002646662.1	84	312.383	206	174	C. briggsae CBR-DDL-3 protein

Locus_22910_Transcript_1/1_Conf_1.000	182	20	3.05E-14	XP_001901250.1	83	81.6481	56	47	MGC80217 protein
Locus_22911_Transcript_1/1_Conf_1.000	170	0							
Locus_22912_Transcript_1/1_Conf_1.000	233	20	4.78E-12	AAC39118.1	72	74.3294	74	54	high-affinity peptide transporter
Locus_22913_Transcript_1/1_Conf_1.000	132	0							
Locus_22914_Transcript_1/1_Conf_1.000	292	20	4.47E-26	NP_498248.2	82	120.939	89	73	hypothetical protein Y37B11A.2
Locus_22915_Transcript_1/1_Conf_1.000	231	14	8.40E-25	AAZ42352.1	76	116.701	76	58	degeneration of certain neurons protein 1
Locus_22916_Transcript_1/1_Conf_1.000	214	0							
Locus_22917_Transcript_1/1_Conf_1.000	264	20	3.64E-28	NP_502011.1	81	127.872	83	68	hypothetical protein T11G6.3
Locus_22918_Transcript_1/1_Conf_1.000	133	0							
Locus_22919_Transcript_1/1_Conf_1.000	230	3	2.55E-05	NP_001022134.1	60	51.9878	64	39	hypothetical protein F32A5.1
Locus_2292_Transcript_1/1_Conf_1.000	319	0							
Locus_22920_Transcript_1/1_Conf_1.000	151	0							
Locus_22921_Transcript_1/1_Conf_1.000	199	20	5.26E-19	XP_002636781.1	83	97.4413	66	55	Hypothetical protein CBG23516
Locus_22922_Transcript_1/1_Conf_1.000	140	0							
Locus_22923_Transcript_1/1_Conf_1.000	132	0							
Locus_22924_Transcript_1/1_Conf_1.000	148	0							

Locus_22925_Transcript_1/1_Conf_1.000	130	20	3.39E-18	XP_002914592.1	100	94.7449	43	43	PREDICTED: casein kinase I isoform epsilon-like
Locus_22926_Transcript_1/1_Conf_1.000	157	0							
Locus_22927_Transcript_1/1_Conf_1.000	224	0							
Locus_22928_Transcript_1/1_Conf_1.000	147	0							
Locus_22929_Transcript_1/1_Conf_1.000	138	0							
Locus_2293_Transcript_1/1_Conf_1.000	1402	20	1.20E-163	XP_002633437.1	77	580.867	470	364	C. briggsae CBR-UNC-22 protein
Locus_22930_Transcript_1/1_Conf_1.000	138	0							
Locus_22931_Transcript_1/1_Conf_1.000	173	0							
Locus_22932_Transcript_1/1_Conf_1.000	225	3	3.94E-14	NP_496398.2	76	81.2629	55	42	hypothetical protein T06D8.1
Locus_22933_Transcript_1/1_Conf_1.000	330	0							
Locus_22934_Transcript_1/1_Conf_1.000	266	2	9.96E-10	NP_741424.1	86	66.6254	38	33	hypothetical protein Y73B6BL.35
Locus_22935_Transcript_1/1_Conf_1.000	218	2	1.15E-10	NP_505963.3	70	69.707	61	43	hypothetical protein T04F3.1
Locus_22936_Transcript_1/1_Conf_1.000	262	0							
Locus_22937_Transcript_1/1_Conf_1.000	292	2	2.31E-06	XP_002645672.1	53	55.4546	102	55	Hypothetical protein CBG07319
Locus_22938_Transcript_1/1_Conf_1.000	185	0							
Locus_22939_Transcript_1/1_Conf_1.000	131	0							
Locus_2294_Transcript_1/1_Conf_1.000	195	20	5.11E-14	XP_002633575.1	79	80.8777	62	49	C. briggsae CBR-EXOS-2 protein
Locus_22940_Transcript_1/1_Conf_1.000	284	20	3.14E-11	XP_002630279.1	58	71.633	72	42	Hypothetical protein CBG00710
Locus_22941_Transcript_1/1_Conf_1.000	358	20	6.80E-27	XP_002638874.1	87	123.635	100	87	Hypothetical protein CBG22093
Locus_22942_Transcript_1/1_Conf_1.000	207	0							
Locus_22943_Transcript_1/1_Conf_1.000	241	0							
Locus_22944_Transcript_1/1_Conf_1.000	432	5	2.42E-08	XP_001897081.1	62	62.003	94	59	hypothetical protein
Locus_22945_Transcript_1/1_Conf_1.000	416	20	1.22E-60	XP_002638579.1	88	235.728	137	121	Hypothetical protein CBG05625
Locus_22946_Transcript_1/1_Conf_1.000	159	0							
Locus_22947_Transcript_1/1_Conf_1.000	324	20	4.43E-34	XP_002639978.1	85	147.517	107	91	C. briggsae CBR-FHOD-1 protein

Locus_22948_Transcript_1/1_Conf_1.000	192	20	4.95E-25	NP_502317.1	95	117.472	60	57	Proline HYdroxylase family member (phy-2)
Locus_22949_Transcript_1/1_Conf_1.000	309	20	9.13E-16	NP_493574.2	71	86.6557	67	48	hypothetical protein F39B2.5
Locus_2295_Transcript_1/1_Conf_1.000	1397	20	1.24E-19	NP_001012918.1	45	102.449	328	148	G2/M phase-specific E3 ubiquitin-protein ligase
Locus_22950_Transcript_1/1_Conf_1.000	183	20	1.27E-20	XP_002637401.1	88	102.834	59	52	C. briggsae CBR-FKB-3 protein
Locus_22951_Transcript_1/1_Conf_1.000	289	20	3.80E-25	XP_002648446.1	72	117.857	92	67	Hypothetical protein CBG24721
Locus_22952_Transcript_1/1_Conf_1.000	277	5	1.12E-21	XP_002629678.1	81	106.301	83	68	Hypothetical protein CBG00897
Locus_22953_Transcript_1/1_Conf_1.000	204	0							
Locus_22954_Transcript_1/1_Conf_1.000	231	0							
Locus_22955_Transcript_1/1_Conf_1.000	283	0							
Locus_22956_Transcript_1/1_Conf_1.000	199	20	1.03E-14	NP_505237.1	71	83.1889	66	47	human CLN (neuronal ceroid lipofuscinosis) related family member (cln-3.3)
Locus_22957_Transcript_1/1_Conf_1.000	508	20	2.13E-10	AAD13339.1	45	68.9366	167	76	ancylostoma-secreted protein 1 precursor

Locus_22958_Transcript_1/1_Conf_1.000	199	20	3.19E-24	XP_002634309.1	89	114.775	65	58	Hypothetical protein CBG17650
Locus_22959_Transcript_1/1_Conf_1.000	142	0							
Locus_2296_Transcript_1/1_Conf_1.000	788	20	1.26E-79	NP_506570.1	73	300.442	250	183	DeHydrogenases, Short chain family member (dhs-22)
Locus_22960_Transcript_1/1_Conf_1.000	298	20	1.14E-05	XP_001523646.1	60	53.1434	78	47	conserved hypothetical protein
Locus_22961_Transcript_1/1_Conf_1.000	137	0							
Locus_22962_Transcript_1/1_Conf_1.000	150	0							
Locus_22963_Transcript_1/1_Conf_1.000	135	0							
Locus_22964_Transcript_1/1_Conf_1.000	157	0							
Locus_22965_Transcript_1/1_Conf_1.000	129	0							
Locus_22966_Transcript_1/1_Conf_1.000	245	0							
Locus_22967_Transcript_1/1_Conf_1.000	142	20	1.45E-13	XP_002631429.1	85	79.337	47	40	Hypothetical protein CBG03286
Locus_22968_Transcript_1/1_Conf_1.000	383	6	9.89E-10	NP_510686.4	63	66.6254	116	74	hypothetical protein F59C12.3
Locus_22969_Transcript_1/1_Conf_1.000	244	0							
Locus_2297_Transcript_1/6_Conf_0.556	1657	20	3.85E-18	EEE52502.1	34	97.8265	374	129	hypothetical protein OsJ_34704
Locus_2297_Transcript_2/6_Conf_0.389	1133	20	1.34E-18	EEE52502.1	35	98.5969	370	130	hypothetical protein OsJ_34704
Locus_2297_Transcript_3/6_Conf_0.167	445	0							
Locus_2297_Transcript_4/6_Conf_0.333	922	20	8.18E-18	YP_068003.1	36	95.5153	355	130	LF3
Locus_2297_Transcript_5/6_Conf_0.500	2112	20	2.11E-19	XP_001427121.1	39	102.449	415	164	hypothetical protein
Locus_2297_Transcript_6/6_Conf_0.556	1675	20	3.91E-18	EEE52502.1	34	97.8265	374	129	hypothetical protein OsJ_34704
Locus_22970_Transcript_1/1_Conf_1.000	343	0							

Locus_22971_Transcript_1/1_Conf_1.000	279	0							
Locus_22972_Transcript_1/1_Conf_1.000	134	20	4.27E-13	CBL87054.2	97	77.7962	44	43	C. elegans protein F54E4.1, partially confirmed by transcript evidence
Locus_22973_Transcript_1/1_Conf_1.000	305	20	7.24E-37	XP_002640083.1	89	156.762	101	90	C. briggsae CBR-FRK-1 protein
Locus_22974_Transcript_1/1_Conf_1.000	254	20	2.09E-31	XP_396338.2	98	138.658	84	83	PREDICTED: similar to Tubulin alpha-1 chain
Locus_22975_Transcript_1/1_Conf_1.000	228	2	2.16E-04	NP_001041050.1	65	48.9062	55	36	hypothetical protein Y57G11C.3
Locus_22976_Transcript_1/1_Conf_1.000	187	0							
Locus_22977_Transcript_1/1_Conf_1.000	130	0							
Locus_22978_Transcript_1/1_Conf_1.000	473	0							
Locus_22979_Transcript_1/1_Conf_1.000	199	0							
Locus_2298_Transcript_1/1_Conf_1.000	708	20	6.93E-28	NP_492608.2	74	128.257	119	89	2 (Zwei) IG-domain protein family member (zig-1)
Locus_22980_Transcript_1/1_Conf_1.000	137	0							
Locus_22981_Transcript_1/1_Conf_1.000	229	0							
Locus_22982_Transcript_1/1_Conf_1.000	130	1	5.79E-10	NP_506678.2	80	67.3958	41	33	hypothetical protein W09D12.1
Locus_22983_Transcript_1/1_Conf_1.000	161	11	1.18E-18	NP_491003.2	94	96.2857	53	50	Amino Acid Transporter family member (aat-5)

Locus_22984_Transcript_1/1_Conf_1.000	179	2	4.39E-10	NP_492730.2	79	67.781	49	39	hypothetical protein Y106G6H.5
Locus_22985_Transcript_1/1_Conf_1.000	269	20	3.64E-20	NP_001024342.1	84	101.293	76	64	Aboc, EXpulsion defective family member (aex-3)
Locus_22986_Transcript_1/1_Conf_1.000	181	0							
Locus_22987_Transcript_1/1_Conf_1.000	148	17	1.72E-09	A8X3A7.2	78	65.855	50	39	Integrin alpha pat-2
Locus_22988_Transcript_1/1_Conf_1.000	128	0							
Locus_22989_Transcript_1/1_Conf_1.000	321	0							
Locus_2299_Transcript_1/1_Conf_1.000	1902	20	4.54E-58	XP_001899184.1	75	230.72	208	158	SWIM zinc finger family protein
Locus_22990_Transcript_1/1_Conf_1.000	217	6	1.23E-12	NP_001021463.1	65	76.2554	69	45	SUppressor of activated let-60 Ras family member (sur-2)
Locus_22991_Transcript_1/1_Conf_1.000	269	0							
Locus_22992_Transcript_1/1_Conf_1.000	297	4	3.28E-29	XP_002644323.1	81	131.339	93	76	C. briggsae CBR-SEC-3 protein
Locus_22993_Transcript_1/1_Conf_1.000	357	20	8.04E-28	XP_002631304.1	81	126.716	85	69	Hypothetical protein CBG03118
Locus_22994_Transcript_1/1_Conf_1.000	184	0							
Locus_22995_Transcript_1/1_Conf_1.000	196	0							
Locus_22996_Transcript_1/1_Conf_1.000	440	20	7.35E-66	XP_002637345.1	89	253.062	146	130	C. briggsae CBR-UNC-68 protein
Locus_22997_Transcript_1/1_Conf_1.000	137	0							
Locus_22998_Transcript_1/1_Conf_1.000	252	0							
Locus_22999_Transcript_1/1_Conf_1.000	468	20	1.17E-18	EFO22869.1	81	96.2857	92	75	hypothetical protein LOAG_05613

Locus_23_Transcript_1/1_Conf_1.000	1555	20	0	XP_002645954.1	92	862.833	482	446	C. briggsae CBR-GAS-1 protein
Locus_230_Transcript_1/1_Conf_1.000	1380	16	2.66E-14	XP_001901969.1	49	84.7297	216	106	hypothetical protein
Locus_2300_Transcript_1/1_Conf_1.000	1112	20	7.98E-93	EFO19759.1	67	345.125	354	240	hypothetical protein LOAG_08726
Locus_23000_Transcript_1/1_Conf_1.000	388	0							
Locus_23001_Transcript_1/1_Conf_1.000	177	0							
Locus_23002_Transcript_1/1_Conf_1.000	262	20	5.66E-13	XP_002631622.1	62	77.411	87	54	Hypothetical protein CBG20808
Locus_23003_Transcript_1/1_Conf_1.000	136	0							
Locus_23004_Transcript_1/1_Conf_1.000	308	20	1.04E-35	XP_002629821.1	84	152.91	101	85	C. briggsae CBR-SQV-8 protein
Locus_23005_Transcript_1/1_Conf_1.000	266	0							
Locus_23006_Transcript_1/1_Conf_1.000	432	20	7.73E-39	EFO17002.1	80	163.31	122	98	rab5-interacting protein
Locus_23007_Transcript_1/1_Conf_1.000	374	5	4.61E-39	NP_001123075.1	86	164.081	123	106	hypothetical protein ZC455.1
Locus_23008_Transcript_1/1_Conf_1.000	351	0							
Locus_23009_Transcript_1/1_Conf_1.000	167	20	4.30E-13	XP_002635372.1	86	77.7962	51	44	C. briggsae CBR-RPM-1 protein
Locus_2301_Transcript_1/1_Conf_1.000	3724	20	0	NP_497506.3	85	1672.14	1246	1066	hypothetical protein Y48G9A.3
Locus_23010_Transcript_1/1_Conf_1.000	182	11	9.48E-16	XP_002648958.1	77	86.6557	61	47	Hypothetical protein CBG21275
Locus_23011_Transcript_1/1_Conf_1.000	175	0							

Locus_23012_Transcript_1/1_Conf_1.000	185	0							
Locus_23013_Transcript_1/1_Conf_1.000	152	0							
Locus_23014_Transcript_1/1_Conf_1.000	208	1	1.25E-04	NP_497103.1	62	49.6766	61	38	hypothetical protein Y53F4B.18
Locus_23015_Transcript_1/1_Conf_1.000	146	6	8.86E-14	NP_495550.1	82	80.1073	51	42	BTB and MATH domain containing family member (bath-44)
Locus_23016_Transcript_1/1_Conf_1.000	156	0							
Locus_23017_Transcript_1/1_Conf_1.000	222	4	9.73E-13	EFO20262.1	88	76.6406	53	47	zinc finger protein
Locus_23018_Transcript_1/1_Conf_1.000	145	20	2.66E-18	XP_001369724.1	93	95.1301	46	43	PREDICTED: similar to LOC539180 protein
Locus_23019_Transcript_1/1_Conf_1.000	299	0							
Locus_2302_Transcript_1/1_Conf_1.000	2013	20	6.32E-175	NP_505638.1	69	619.002	593	413	RO (Ro) ribonucleoProtein family member (rop-1)
Locus_23020_Transcript_1/1_Conf_1.000	283	0							
Locus_23021_Transcript_1/1_Conf_1.000	204	0							
Locus_23022_Transcript_1/1_Conf_1.000	169	0							
Locus_23023_Transcript_1/1_Conf_1.000	243	12	4.57E-07	EFO17581.1	76	57.7658	63	48	bestrophin family protein
Locus_23024_Transcript_1/1_Conf_1.000	537	5	2.84E-38	XP_002647112.1	73	161.77	146	107	C. briggsae CBR-FRM-8 protein
Locus_23025_Transcript_1/1_Conf_1.000	780	1	2.64E-21	EFO16834.1	64	106.686	136	88	hypothetical protein LOAG_11670
Locus_23026_Transcript_1/1_Conf_1.000	267	8	1.09E-24	NP_001022643.1	76	116.316	88	67	LEThal family member (let-805)

Locus_23027_Transcript_1/1_Conf_1.000	284	20	9.42E-24	XP_002634423.1	79	113.235	88	70	C. briggsae CBR-PTR-14 protein
Locus_23028_Transcript_1/2_Conf_1.000	384	20	3.74E-09	ZP_05656155.1	47	64.6994	87	41	predicted protein
Locus_23028_Transcript_2/2_Conf_1.000	293	20	3.80E-09	ZP_05656155.1	47	64.6994	87	41	predicted protein
Locus_23029_Transcript_1/1_Conf_1.000	280	0							
Locus_2303_Transcript_1/1_Conf_1.000	552	0							
Locus_23030_Transcript_1/1_Conf_1.000	385	20	1.31E-54	CAR63666.1	90	215.698	128	116	putative Zinc finger C-x8-C-x5-C-x3-H type
Locus_23031_Transcript_1/1_Conf_1.000	166	3	1.06E-11	CAR63674.1	85	73.1738	54	46	putative PeRoXisome assembly factor
Locus_23032_Transcript_1/1_Conf_1.000	198	20	6.23E-12	XP_002645757.1	88	73.9442	44	39	Hypothetical protein CBG07432
Locus_23033_Transcript_1/1_Conf_1.000	253	0							
Locus_23034_Transcript_1/1_Conf_1.000	188	1	8.25E-12	CAJ45481.1	76	73.559	60	46	legumain
Locus_23035_Transcript_1/1_Conf_1.000	218	0							
Locus_23036_Transcript_1/1_Conf_1.000	235	0							
Locus_23037_Transcript_1/1_Conf_1.000	312	1	5.73E-05	XP_002642902.1	45	50.8322	105	48	Hypothetical protein CBG15176
Locus_23038_Transcript_1/1_Conf_1.000	144	20	1.01E-17	EFO25442.1	95	93.2041	48	46	TWK-7 protein

Locus_23039_Transcript_1/1_Conf_1.000	145	4	1.46E-08	XP_002640845.1	76	62.7734	46	35	C. briggsae CBR-CDH-1 protein
Locus_2304_Transcript_1/1_Conf_1.000	284	0							
Locus_23040_Transcript_1/1_Conf_1.000	141	0							
Locus_23041_Transcript_1/1_Conf_1.000	224	20	2.31E-22	NP_498057.1	89	108.612	67	60	hypothetical protein C27F2.10
Locus_23042_Transcript_1/1_Conf_1.000	221	0							
Locus_23043_Transcript_1/1_Conf_1.000	254	0							
Locus_23044_Transcript_1/2_Conf_1.000	271	0							
Locus_23044_Transcript_2/2_Conf_1.000	424	0							
Locus_23045_Transcript_1/1_Conf_1.000	146	0							
Locus_23046_Transcript_1/1_Conf_1.000	256	9	7.20E-16	NP_499308.1	70	87.0409	77	54	hypothetical protein D2045.5
Locus_23047_Transcript_1/1_Conf_1.000	181	2	7.53E-05	XP_002631385.1	61	50.447	57	35	Hypothetical protein CBG03227
Locus_23048_Transcript_1/1_Conf_1.000	203	0							
Locus_23049_Transcript_1/1_Conf_1.000	162	0							
Locus_2305_Transcript_1/1_Conf_1.000	1074	4	3.48E-122	XP_002631137.1	75	442.58	328	248	Hypothetical protein CBG02921
Locus_23050_Transcript_1/1_Conf_1.000	312	20	7.64E-50	NP_505708.2	94	199.904	102	96	hypothetical protein F32G8.4
Locus_23051_Transcript_1/1_Conf_1.000	141	0							
Locus_23052_Transcript_1/1_Conf_1.000	163	0							
Locus_23053_Transcript_1/1_Conf_1.000	395	0							
Locus_23054_Transcript_1/1_Conf_1.000	133	0							
Locus_23055_Transcript_1/1_Conf_1.000	181	0							
Locus_23056_Transcript_1/1_Conf_1.000	135	4	2.85E-17	ABU93237.1	100	91.6633	44	44	ligand-gated ion channel subunit
Locus_23057_Transcript_1/1_Conf_1.000	253	2	4.83E-20	XP_002719154.1	73	100.908	83	61	PREDICTED: alpha 1 type I collagen
Locus_23058_Transcript_1/1_Conf_1.000	132	0							
Locus_23059_Transcript_1/1_Conf_1.000	185	20	5.52E-24	XP_001640743.1	95	114.005	60	57	predicted protein

Locus_2306_Transcript_1/2_Conf_1.000	1217	20	4.42E-55	XP_002633667.1	67	219.935	230	156	Hypothetical protein CBG03342
Locus_2306_Transcript_2/2_Conf_1.000	2247	20	0	O18023.4	72	824.313	761	548	Ig-like and fibronectin type-III domain-containing protein C25G4.10
Locus_23060_Transcript_1/1_Conf_1.000	180	1	1.05E-06	XP_001083984.1	75	56.6102	45	34	PREDICTED: hypothetical protein LOC695354
Locus_23061_Transcript_1/1_Conf_1.000	298	20	9.18E-48	XP_001901913.1	95	192.971	97	93	F-box only protein 11
Locus_23062_Transcript_1/1_Conf_1.000	165	0							
Locus_23063_Transcript_1/1_Conf_1.000	215	20	1.85E-08	XP_002742403.1	72	62.3882	68	49	PREDICTED: TIP30-like
Locus_23064_Transcript_1/1_Conf_1.000	256	0							
Locus_23065_Transcript_1/1_Conf_1.000	179	4	1.08E-08	XP_001892742.1	83	63.1586	56	47	hypothetical protein
Locus_23066_Transcript_1/1_Conf_1.000	468	20	1.51E-58	XP_002633219.1	84	228.794	152	128	C. briggsae CBR-ITR-1 protein
Locus_23067_Transcript_1/1_Conf_1.000	184	17	7.25E-08	NP_998128.1	94	60.4622	34	32	disco-interacting protein 2 homolog B
Locus_23068_Transcript_1/1_Conf_1.000	137	0							
Locus_23069_Transcript_1/1_Conf_1.000	325	20	7.56E-34	XP_002645153.1	84	146.747	99	84	C. briggsae CBR-SFXN-2 protein
Locus_2307_Transcript_1/2_Conf_1.000	2933	20	2.70E-23	EFO22438.1	54	115.931	206	112	R3H domain-containing protein

Locus_2307_Transcript_2/2_Conf_1.000	2933	20	6.01E-23	XP_001900693.1	44	114.775	327	147	R3H domain containing protein
Locus_23070_Transcript_1/1_Conf_1.000	297	0							
Locus_23071_Transcript_1/1_Conf_1.000	240	0							
Locus_23072_Transcript_1/1_Conf_1.000	165	0							
Locus_23073_Transcript_1/1_Conf_1.000	247	0							
Locus_23074_Transcript_1/1_Conf_1.000	156	11	2.81E-12	XP_002633999.1	90	75.0998	43	39	C. briggsae CBR-TNI-4 protein
Locus_23075_Transcript_1/1_Conf_1.000	358	20	1.01E-30	XP_001899166.1	74	136.346	116	86	GTP-ase activating protein for Arf containing protein
Locus_23076_Transcript_1/1_Conf_1.000	143	0							
Locus_23077_Transcript_1/1_Conf_1.000	138	0							
Locus_23078_Transcript_1/1_Conf_1.000	298	4	6.64E-14	XP_002638288.1	61	80.4925	89	55	Hypothetical protein CBG22835
Locus_23079_Transcript_1/1_Conf_1.000	184	0							
Locus_2308_Transcript_1/1_Conf_1.000	1637	20	0	P50719.1	100	843.188	440	440	Tubulin alpha chain
Locus_23080_Transcript_1/1_Conf_1.000	339	20	3.47E-07	BAC36271.1	51	58.151	104	54	unnamed protein product
Locus_23081_Transcript_1/1_Conf_1.000	224	0							
Locus_23082_Transcript_1/1_Conf_1.000	137	0							
Locus_23083_Transcript_1/1_Conf_1.000	286	0							
Locus_23084_Transcript_1/1_Conf_1.000	213	0							
Locus_23085_Transcript_1/1_Conf_1.000	145	19	8.29E-12	XP_002640829.1	91	73.559	48	44	C. briggsae CBR-ADS-1 protein
Locus_23086_Transcript_1/1_Conf_1.000	415	0							

Locus_23087_Transcript_1/1_Conf_1.000	181	2	4.41E-05	NP_502795.2	68	51.2174	51	35	Homolog of Hedgehog AcylTransferase family member (hhat-2)
Locus_23088_Transcript_1/1_Conf_1.000	251	1	3.84E-06	XP_001897820.1	61	54.6842	81	50	hypothetical protein Bm1_31875
Locus_23089_Transcript_1/1_Conf_1.000	193	8	1.14E-13	EFO12672.1	79	79.7221	64	51	hypothetical protein LOAG_15862
Locus_2309_Transcript_1/1_Conf_1.000	762	20	3.25E-45	NP_499537.2	61	186.037	267	164	hypothetical protein Y56A3A.2
Locus_23090_Transcript_1/1_Conf_1.000	165	0							
Locus_23091_Transcript_1/1_Conf_1.000	187	0							
Locus_23092_Transcript_1/1_Conf_1.000	232	0							
Locus_23093_Transcript_1/1_Conf_1.000	132	3	7.06E-08	NP_508964.2	75	60.4622	45	34	Regulator of G protein Signaling family member (rgs-6)
Locus_23094_Transcript_1/1_Conf_1.000	172	2	3.28E-05	XP_002630912.1	63	51.6026	57	36	Hypothetical protein CBG02636
Locus_23095_Transcript_1/1_Conf_1.000	544	4	5.25E-11	NP_001021653.1	45	71.2478	191	87	FANCI (Fanconi anemia complex component I) homolog family member (fnci-1)
Locus_23096_Transcript_1/1_Conf_1.000	168	0							
Locus_23097_Transcript_1/1_Conf_1.000	322	20	1.14E-21	XP_001899134.1	73	106.301	102	75	hypothetical protein Bm1_38405
Locus_23098_Transcript_1/1_Conf_1.000	228	2	7.64E-18	XP_002632364.1	93	93.5893	75	70	Hypothetical protein CBG00382
Locus_23099_Transcript_1/1_Conf_1.000	129	0							
Locus_231_Transcript_1/2_Conf_1.000	536	20	1.87E-34	XP_002698333.1	90	149.058	103	93	PREDICTED: hypothetical protein
Locus_231_Transcript_2/2_Conf_1.000	497	20	1.05E-27	XP_002698333.1	89	126.331	92	82	PREDICTED: hypothetical protein

Locus_2310_Transcript_1/4_Conf_0.429	194	4	2.82E-04	XP_002641892.1	62	48.521	50	31	Hypothetical protein CBG16586
Locus_2310_Transcript_2/4_Conf_0.571	297	4	2.61E-10	XP_002641892.1	60	68.5514	76	46	Hypothetical protein CBG16586
Locus_2310_Transcript_3/4_Conf_0.571	238	4	1.34E-06	NP_001021172.1	69	56.225	46	32	PDF Receptor homolog family member (pdfr-1)
Locus_2310_Transcript_4/4_Conf_0.571	289	4	2.64E-10	XP_002641892.1	60	68.5514	76	46	Hypothetical protein CBG16586
Locus_23100_Transcript_1/1_Conf_1.000	137	0							
Locus_23101_Transcript_1/1_Conf_1.000	237	20	4.27E-29	NP_496802.2	89	130.954	79	71	BTAF (TBP-associated factor) homolog family member (btf-1)
Locus_23102_Transcript_1/1_Conf_1.000	135	0							
Locus_23103_Transcript_1/1_Conf_1.000	465	5	8.17E-12	XP_002642202.1	47	73.559	123	59	Hypothetical protein CBG18175
Locus_23104_Transcript_1/1_Conf_1.000	177	0							
Locus_23105_Transcript_1/1_Conf_1.000	204	20	2.05E-23	NP_502390.2	84	112.079	66	56	High Incidence of Males (increased X chromosome loss) family member (him-6)
Locus_23106_Transcript_1/1_Conf_1.000	545	20	5.94E-47	NP_001129830.1	62	190.66	182	113	LEThal family member (let-23)

Locus_23107_Transcript_1/1_Conf_1.000	237	20	2.43E-24	CAR63538.1	79	115.161	78	62	putative MFP2
Locus_23108_Transcript_1/1_Conf_1.000	142	20	6.10E-20	XP_002828013.1	100	100.523	46	46	PREDICTED: tRNA-dihydrouridine synthase 1-like isoform 2
Locus_23109_Transcript_1/1_Conf_1.000	248	0							
Locus_2311_Transcript_1/1_Conf_1.000	1074	20	1.06E-62	NP_496320.3	63	244.973	337	213	Cell Death Lethal family member (cdl-1)
Locus_23110_Transcript_1/1_Conf_1.000	177	1	5.56E-05	XP_002640523.1	79	50.8322	44	35	Hypothetical protein CBG18685
Locus_23111_Transcript_1/1_Conf_1.000	147	3	1.37E-06	EFO17834.1	76	56.225	47	36	hypothetical protein LOAG_10662
Locus_23112_Transcript_1/1_Conf_1.000	551	0							
Locus_23113_Transcript_1/1_Conf_1.000	140	0							
Locus_23114_Transcript_1/1_Conf_1.000	144	0							
Locus_23115_Transcript_1/1_Conf_1.000	478	20	2.03E-39	XP_002735023.1	75	165.236	157	119	PREDICTED: CG8129-like
Locus_23116_Transcript_1/1_Conf_1.000	221	0							
Locus_23117_Transcript_1/1_Conf_1.000	188	0							
Locus_23118_Transcript_1/1_Conf_1.000	200	0							
Locus_23119_Transcript_1/1_Conf_1.000	165	20	9.56E-21	AAH18938.4	100	103.219	45	45	NSMCE1 protein
Locus_2312_Transcript_1/2_Conf_1.000	2781	20	0	XP_002642644.1	85	1073.15	796	678	C. briggsae CBR-EGL-45 protein
Locus_2312_Transcript_2/2_Conf_1.000	2835	20	0	XP_002642644.1	85	1073.15	796	678	C. briggsae CBR-EGL-45 protein
Locus_23120_Transcript_1/1_Conf_1.000	155	0							
Locus_23121_Transcript_1/1_Conf_1.000	212	0							
Locus_23122_Transcript_1/1_Conf_1.000	189	0							

Locus_23123_Transcript_1/1_Conf_1.000	342	20	2.45E-48	XP_002637013.1	82	194.897	113	93	C. briggsae CBR-NID-1 protein
Locus_23124_Transcript_1/1_Conf_1.000	151	0							
Locus_23125_Transcript_1/1_Conf_1.000	369	20	3.32E-37	NP_001024306.1	78	157.918	114	90	Poly(ADP-ribose) Metabolism Enzyme family member (pme-5)
Locus_23126_Transcript_1/1_Conf_1.000	375	7	9.29E-24	XP_002640437.1	68	113.235	113	77	Hypothetical protein CBG08489
Locus_23127_Transcript_1/1_Conf_1.000	625	20	1.72E-63	NP_001040789.1	84	246.128	178	150	hypothetical protein H20J04.4
Locus_23128_Transcript_1/1_Conf_1.000	269	0							
Locus_23129_Transcript_1/1_Conf_1.000	225	7	3.33E-13	XP_001894370.1	69	78.1814	72	50	Immunoglobulin I-set domain containing protein
Locus_2313_Transcript_1/1_Conf_1.000	551	20	1.45E-96	BAE90788.1	100	355.525	183	183	unnamed protein product
Locus_23130_Transcript_1/1_Conf_1.000	327	8	2.34E-43	ACI49226.1	87	178.333	109	95	hypothetical protein Csp3_JD05.011
Locus_23131_Transcript_1/1_Conf_1.000	157	0							
Locus_23132_Transcript_1/1_Conf_1.000	498	0							
Locus_23133_Transcript_1/1_Conf_1.000	146	0							
Locus_23134_Transcript_1/1_Conf_1.000	191	2	3.23E-08	XP_002634150.1	71	61.6178	39	28	Hypothetical protein CBG01711
Locus_23135_Transcript_1/1_Conf_1.000	227	20	4.05E-35	XP_002828602.1	100	150.984	75	75	PREDICTED: trafficking protein particle complex subunit 5-like isoform 1
Locus_23136_Transcript_1/1_Conf_1.000	396	20	9.02E-27	NP_493980.1	64	123.25	132	85	hypothetical protein H17B01.4
Locus_23137_Transcript_1/1_Conf_1.000	291	13	8.18E-12	XP_002646276.1	63	73.559	66	42	C. briggsae CBR-ABF-2 protein
Locus_23138_Transcript_1/1_Conf_1.000	180	0							
Locus_23139_Transcript_1/1_Conf_1.000	169	0							
Locus_2314_Transcript_1/1_Conf_1.000	1573	20	9.32E-59	NP_494334.3	59	232.646	255	152	SET (trithorax/polycomb) domain containing family member (set-11)
Locus_23140_Transcript_1/1_Conf_1.000	203	0							
Locus_23141_Transcript_1/1_Conf_1.000	181	0							
Locus_23142_Transcript_1/1_Conf_1.000	488	1	2.22E-09	XP_001900122.1	58	65.4698	90	53	hypothetical protein Bm1_43305
Locus_23143_Transcript_1/1_Conf_1.000	187	5	1.11E-24	ACD85816.1	95	116.316	61	58	forkhead transcription factor DAF-16

Locus_23144_Transcript_1/1_Conf_1.000	157	17	4.06E-11	XP_002635426.1	92	71.2478	51	47	Hypothetical protein CBG00823
Locus_23145_Transcript_1/1_Conf_1.000	167	0							
Locus_23146_Transcript_1/1_Conf_1.000	175	5	4.56E-07	NP_001041091.2	67	57.7658	55	37	Motor AXon guidance family member (max-1)
Locus_23147_Transcript_1/1_Conf_1.000	251	0							
Locus_23148_Transcript_1/1_Conf_1.000	191	20	2.81E-28	XP_002642963.1	96	128.257	63	61	Hypothetical protein CBG15247
Locus_23149_Transcript_1/1_Conf_1.000	152	0							
Locus_2315_Transcript_1/1_Conf_1.000	551	20	6.96E-91	NP_506055.2	95	336.65	183	175	ELongator complex Protein Component family member (elpc-3)
Locus_23150_Transcript_1/1_Conf_1.000	169	0							
Locus_23151_Transcript_1/1_Conf_1.000	741	10	7.44E-23	XP_002633684.1	53	111.694	209	112	C. briggsae CBR-EEL-1 protein
Locus_23152_Transcript_1/1_Conf_1.000	151	0							
Locus_23153_Transcript_1/1_Conf_1.000	214	0							
Locus_23154_Transcript_1/1_Conf_1.000	344								
Locus_23155_Transcript_1/1_Conf_1.000	169	0							
Locus_23156_Transcript_1/1_Conf_1.000	173	0							
Locus_23157_Transcript_1/1_Conf_1.000	175	8	7.48E-26	XP_001893452.1	98	120.168	58	57	basement membrane proteoglycan
Locus_23158_Transcript_1/1_Conf_1.000	202	0							
Locus_23159_Transcript_1/1_Conf_1.000	200	0							
Locus_2316_Transcript_1/1_Conf_1.000	1085	20	7.44E-80	XP_001510286.1	59	301.982	364	217	PREDICTED: similar to Phosphoenolpyruvate carboxykinase, cytosolic [GTP] (Phosphoenolpyruvate carboxylase) (PEPCK-C) [Ornithorhynchus anatinus]

Locus_23160_Transcript_1/2_Conf_1.000	778	20	4.44E-37	NP_491390.2	65	159.073	217	142	hypothetical protein D1007.15
Locus_23160_Transcript_2/2_Conf_1.000	778	20	4.44E-37	NP_491390.2	65	159.073	217	142	hypothetical protein D1007.15
Locus_23161_Transcript_1/1_Conf_1.000	154	0							
Locus_23162_Transcript_1/1_Conf_1.000	243	6	9.50E-13	CBX25138.1	69	76.6406	69	48	Hypothetical protein C25A1.7a
Locus_23163_Transcript_1/1_Conf_1.000	181	0							
Locus_23164_Transcript_1/1_Conf_1.000	318	0							
Locus_23165_Transcript_1/1_Conf_1.000	277	0							
Locus_23166_Transcript_1/1_Conf_1.000	185	0							
Locus_23167_Transcript_1/1_Conf_1.000	212	0							
Locus_23168_Transcript_1/1_Conf_1.000	448	20	1.62E-60	XP_002635876.1	89	235.343	149	133	C. briggsae CBR-PTR-16 protein
Locus_23169_Transcript_1/1_Conf_1.000	187	3	1.97E-05	EFO13949.1	72	52.373	58	42	hypothetical protein LOAG_14577
Locus_2317_Transcript_1/1_Conf_1.000	1249	3	5.27E-43	XP_002646302.1	49	179.874	429	214	Hypothetical protein CBG12009
Locus_23170_Transcript_1/1_Conf_1.000	176	0							
Locus_23171_Transcript_1/1_Conf_1.000	160	20	2.99E-22	NP_502576.1	100	108.227	53	53	RAB family member (rab-19)
Locus_23172_Transcript_1/1_Conf_1.000	220	0							
Locus_23173_Transcript_1/1_Conf_1.000	298	0							
Locus_23174_Transcript_1/1_Conf_1.000	227	0							
Locus_23175_Transcript_1/1_Conf_1.000	141	0							
Locus_23176_Transcript_1/1_Conf_1.000	173	0							
Locus_23177_Transcript_1/1_Conf_1.000	131	20	9.86E-18	XP_002813192.1	100	93.2041	43	43	PREDICTED: plexin-D1-like

Locus_23178_Transcript_1/1_Conf_1.000	299	20	2.34E-51	NP_001024972.1	100	204.912	99	99	abnormal cell MIGration family member (mig-15)
Locus_23179_Transcript_1/1_Conf_1.000	625	20	4.85E-50	XP_002646339.1	75	201.445	174	131	C. briggsae CBR-INX-16 protein
Locus_2318_Transcript_1/2_Conf_1.000	2402	20	5.84E-29	AAK18978.3	47	134.42	267	128	Hypothetical protein T20F7.1
Locus_2318_Transcript_2/2_Conf_1.000	2381	20	2.87E-28	AAK18978.3	47	132.109	263	126	Hypothetical protein T20F7.1
Locus_23180_Transcript_1/1_Conf_1.000	154	3	2.64E-10	XP_001900332.1	97	68.5514	34	33	hypothetical protein
Locus_23181_Transcript_1/1_Conf_1.000	343	0							
Locus_23182_Transcript_1/1_Conf_1.000	225	0							
Locus_23183_Transcript_1/1_Conf_1.000	791	20	4.85E-71	XP_002635154.1	74	271.937	230	172	C. briggsae CBR-VRS-1 protein
Locus_23184_Transcript_1/1_Conf_1.000	179	2	4.55E-07	NP_505266.1	73	57.7658	65	48	hypothetical protein C01B7.5
Locus_23185_Transcript_1/1_Conf_1.000	249	6	1.27E-17	AAV41897.1	77	92.8189	70	54	SAX-2
Locus_23186_Transcript_1/1_Conf_1.000	250	0							
Locus_23187_Transcript_1/1_Conf_1.000	150	0							
Locus_23188_Transcript_1/1_Conf_1.000	235	0							
Locus_23189_Transcript_1/1_Conf_1.000	299	0							
Locus_2319_Transcript_1/1_Conf_1.000	830	20	5.86E-54	NP_741763.1	62	215.312	276	172	Homeodomain interacting Protein Kinase family member (hpk-1)
Locus_23190_Transcript_1/1_Conf_1.000	213	0							
Locus_23191_Transcript_1/1_Conf_1.000	207	20	6.56E-30	NP_508833.1	94	133.65	68	64	hypothetical protein T07F12.4
Locus_23192_Transcript_1/1_Conf_1.000	214	0							
Locus_23193_Transcript_1/1_Conf_1.000	662	20	1.01E-35	XP_002639186.1	69	154.066	146	102	C. briggsae CBR-LEM-3 protein

Locus_23194_Transcript_1/1_Conf_1.000	239	4	1.80E-27	XP_002638826.1	84	125.561	79	67	Hypothetical protein CBG22031
Locus_23195_Transcript_1/1_Conf_1.000	461	0							
Locus_23196_Transcript_1/1_Conf_1.000	163	20	9.28E-16	NP_501335.1	92	86.6557	53	49	UNCoordinated family member (unc-24)
Locus_23197_Transcript_1/1_Conf_1.000	148	0							
Locus_23198_Transcript_1/1_Conf_1.000	237	5	2.98E-06	XP_002637807.1	64	55.0694	71	46	Hypothetical protein CBG04593
Locus_23199_Transcript_1/1_Conf_1.000	130	2	1.25E-04	NP_001023038.1	77	49.6766	40	31	EXCretory canal abnormal family member (exc-5)
Locus_232_Transcript_1/1_Conf_1.000	1047	20	1.18E-34	NP_505179.1	54	151.754	251	137	hypothetical protein T23B12.4
Locus_2320_Transcript_1/1_Conf_1.000	1692	20	8.96E-79	NP_498203.2	57	299.286	510	292	CEll Death abnormality family member (ced-6)
Locus_23200_Transcript_1/1_Conf_1.000	276	20	3.97E-27	XP_002638736.1	81	124.405	92	75	C. briggsae CBR-EGO-1 protein
Locus_23201_Transcript_1/1_Conf_1.000	248	20	1.27E-41	XP_002823653.1	100	172.555	82	82	PREDICTED: hypothetical protein LOC100451758
Locus_23202_Transcript_1/1_Conf_1.000	234	0							
Locus_23203_Transcript_1/1_Conf_1.000	259	0							
Locus_23204_Transcript_1/1_Conf_1.000	148	5	8.85E-06	NP_493023.1	85	53.5286	34	29	hypothetical protein R09B3.3
Locus_23205_Transcript_1/1_Conf_1.000	195	7	9.34E-08	XP_002636803.1	68	60.077	63	43	Hypothetical protein CBG09244
Locus_23206_Transcript_1/1_Conf_1.000	133	3	2.51E-05	XP_002639821.1	79	51.9878	39	31	C. briggsae CBR-ERS-2 protein

Locus_23207_Transcript_1/1_Conf_1.000	131	20	4.14E-16	EFN87947.1	100	87.8113	43	43	26S protease regulatory subunit 8
Locus_23208_Transcript_1/1_Conf_1.000	138	0							
Locus_23209_Transcript_1/1_Conf_1.000	264	4	1.94E-05	NP_001023935.1	56	52.373	67	38	hypothetical protein F46B6.5
Locus_2321_Transcript_1/1_Conf_1.000	1394	20	0	XP_002643863.1	86	710.294	449	390	C. briggsae CBR-HID-1 protein
Locus_23210_Transcript_1/1_Conf_1.000	310	20	7.42E-42	EAW52759.1	100	173.326	82	82	transgelin 2, isoform CRA_a
Locus_23211_Transcript_1/1_Conf_1.000	227	5	4.98E-09	CBW48343.1	86	64.3142	38	33	Hypothetical protein C29A12.4d
Locus_23212_Transcript_1/1_Conf_1.000	317	1	6.32E-04	EFO26583.1	53	47.3654	98	52	hypothetical protein LOAG_01905
Locus_23213_Transcript_1/1_Conf_1.000	326	0							
Locus_23214_Transcript_1/1_Conf_1.000	184	0							
Locus_23215_Transcript_1/1_Conf_1.000	383	20	2.66E-47	NP_001024720.1	88	191.43	126	111	Na/H eXchanger family member (nhx-5)
Locus_23216_Transcript_1/1_Conf_1.000	188	0							
Locus_23217_Transcript_1/1_Conf_1.000	288	0							
Locus_23218_Transcript_1/1_Conf_1.000	165	8	2.88E-09	CAA46190.1	80	65.0846	51	41	P-glycoprotein A
Locus_23219_Transcript_1/1_Conf_1.000	141	1	1.72E-06	EAW50361.1	88	55.8398	44	39	hCG2041296
Locus_2322_Transcript_1/1_Conf_1.000	758	20	1.09E-85	NP_503105.1	80	320.472	255	204	Peroxisomal Membrane Protein related family member (pmp-4)
Locus_23220_Transcript_1/1_Conf_1.000	167	0							
Locus_23221_Transcript_1/1_Conf_1.000	192	2	4.81E-04	XP_002637532.1	80	47.7506	36	29	Hypothetical protein CBG19257
Locus_23222_Transcript_1/1_Conf_1.000	300	0							
Locus_23223_Transcript_1/1_Conf_1.000	181	0							

Locus_23224_Transcript_1/1_Conf_1.000	346	20	3.54E-31	NP_501928.1	70	137.887	116	82	hypothetical protein F13B12.4
Locus_23225_Transcript_1/1_Conf_1.000	135	0							
Locus_23226_Transcript_1/1_Conf_1.000	212	3	2.41E-16	XP_002636913.1	75	88.5817	70	53	Hypothetical protein CBG09377
Locus_23227_Transcript_1/1_Conf_1.000	199	3	3.78E-09	XP_002638395.1	67	64.6994	61	41	Hypothetical protein CBG18604
Locus_23228_Transcript_1/1_Conf_1.000	221	7	2.48E-16	EFO21619.1	76	88.5817	71	54	DOMON domain-containing protein
Locus_23229_Transcript_1/1_Conf_1.000	169	20	2.19E-25	XP_002828392.1	100	118.627	55	55	PREDICTED: LOW QUALITY PROTEIN: lipid phosphate phosphohydrolase 2-like, partial
Locus_2323_Transcript_1/1_Conf_1.000	523	0							
Locus_23230_Transcript_1/1_Conf_1.000	168								
Locus_23231_Transcript_1/1_Conf_1.000	168	8	3.88E-14	XP_001894372.1	78	81.2629	56	44	Immunoglobulin I-set domain containing protein
Locus_23232_Transcript_1/1_Conf_1.000	151	0							
Locus_23233_Transcript_1/1_Conf_1.000	129	0							
Locus_23234_Transcript_1/1_Conf_1.000	330	5	2.32E-51	CAR63666.1	94	204.912	110	104	putative Zinc finger C-x8-C-x5-C-x3-H type
Locus_23235_Transcript_1/1_Conf_1.000	140	0							
Locus_23236_Transcript_1/1_Conf_1.000	157	2	2.15E-12	XP_002634505.1	82	75.485	52	43	Hypothetical protein CBG08295
Locus_23237_Transcript_1/1_Conf_1.000	202	0							
Locus_23238_Transcript_1/1_Conf_1.000	128	20	1.06E-11	NP_502846.2	90	73.1738	42	38	hypothetical protein Y73F8A.11
Locus_23239_Transcript_1/1_Conf_1.000	132	0							
Locus_2324_Transcript_1/2_Conf_1.000	345	20	1.03E-14	NP_504416.1	68	83.1889	51	35	hypothetical protein C10G8.4
Locus_2324_Transcript_2/2_Conf_1.000	462	20	1.04E-14	NP_504416.1	68	83.1889	51	35	hypothetical protein C10G8.4
Locus_23240_Transcript_1/1_Conf_1.000	144	0							
Locus_23241_Transcript_1/1_Conf_1.000	270	0							
Locus_23242_Transcript_1/1_Conf_1.000	153	0							
Locus_23243_Transcript_1/1_Conf_1.000	200	2	1.26E-04	EFO16396.1	60	49.6766	66	40	hypothetical protein LOAG_12108
Locus_23244_Transcript_1/1_Conf_1.000	385	20	3.94E-59	CAB55030.2	96	230.72	128	123	C. elegans protein Y57A10A.10, partially confirmed by transcript evidence

Locus_23245_Transcript_1/1_Conf_1.000	146	20	2.33E-22	BAH13667.1	100	108.612	48	48	unnamed protein product
Locus_23246_Transcript_1/1_Conf_1.000	548	6	2.31E-30	EFO19771.1	59	135.576	189	113	hypothetical protein LOAG_08718
Locus_23247_Transcript_1/1_Conf_1.000	319	20	2.71E-31	NP_495273.1	79	138.272	106	84	hypothetical protein C32D5.6
Locus_23248_Transcript_1/1_Conf_1.000	186	3	3.83E-09	NP_495267.1	85	64.6994	35	30	hypothetical protein C29F5.1
Locus_23249_Transcript_1/1_Conf_1.000	270	20	3.75E-17	NP_505501.1	68	91.2781	85	58	Temporarily Assigned Gene name family member (tag-120)
Locus_2325_Transcript_1/2_Conf_1.000	766	20	2.04E-63	NP_741181.1	74	246.514	213	158	hypothetical protein T12A2.15
Locus_2325_Transcript_2/2_Conf_1.000	743	20	1.93E-63	NP_741181.1	73	246.514	213	157	hypothetical protein T12A2.15
Locus_23250_Transcript_1/1_Conf_1.000	163	0							
Locus_23251_Transcript_1/1_Conf_1.000	337	0							
Locus_23252_Transcript_1/1_Conf_1.000	170	0							
Locus_23253_Transcript_1/1_Conf_1.000	132	0							
Locus_23254_Transcript_1/1_Conf_1.000	317	0							
Locus_23255_Transcript_1/1_Conf_1.000	262	0							
Locus_23256_Transcript_1/1_Conf_1.000	428	20	2.29E-06	XP_002648766.1	45	55.4546	146	66	Hypothetical protein CBG10699
Locus_23257_Transcript_1/1_Conf_1.000	184	20	5.35E-19	CAR63646.1	98	97.4413	60	59	putative Homeobox family member
Locus_23258_Transcript_1/1_Conf_1.000	205	0							

Locus_23259_Transcript_1/1_Conf_1.000	154	0							
Locus_2326_Transcript_1/1_Conf_1.000	356	20	1.06E-19	ACI49172.1	60	99.7525	121	73	hypothetical protein Csp3_JD01.004
Locus_23260_Transcript_1/1_Conf_1.000	138	0							
Locus_23261_Transcript_1/1_Conf_1.000	137	0							
Locus_23262_Transcript_1/1_Conf_1.000	474	0							
Locus_23263_Transcript_1/1_Conf_1.000	489	20	1.93E-21	XP_001636445.1	49	105.531	180	89	predicted protein
Locus_23264_Transcript_1/1_Conf_1.000	338	20	5.35E-24	EFO26090.1	72	114.005	111	80	hypothetical protein LOAG_02402
Locus_23265_Transcript_1/1_Conf_1.000	153	0							
Locus_23266_Transcript_1/1_Conf_1.000	156	0							
Locus_23267_Transcript_1/1_Conf_1.000	222	20	1.77E-14	NP_506322.1	81	82.4185	55	45	hypothetical protein F53F1.2
Locus_23268_Transcript_1/1_Conf_1.000	407	20	8.61E-30	XP_002638851.1	69	133.265	136	94	C. briggsae CBR-IMB-5 protein
Locus_23269_Transcript_1/1_Conf_1.000	239	0							
Locus_2327_Transcript_1/1_Conf_1.000	128	0							
Locus_23270_Transcript_1/1_Conf_1.000	187	0							
Locus_23271_Transcript_1/1_Conf_1.000	188	20	3.13E-11	BAG10454.1	100	71.633	62	62	ribosome-binding protein 1
Locus_23272_Transcript_1/1_Conf_1.000	199	1	3.91E-06	NP_001022861.1	76	54.6842	43	33	CREB Homolog family member (crh-1)
Locus_23273_Transcript_1/1_Conf_1.000	140	0							
Locus_23274_Transcript_1/1_Conf_1.000	135	0							
Locus_23275_Transcript_1/1_Conf_1.000	265	0							

Locus_23276_Transcript_1/1_Conf_1.000	133	7	2.34E-11	XP_002634396.1	86	72.0182	44	38	Hypothetical protein CBG04396
Locus_23277_Transcript_1/1_Conf_1.000	274	5	2.77E-12	EFO24919.1	64	75.0998	90	58	RGC/RGC protein kinase
Locus_23278_Transcript_1/1_Conf_1.000	142	0							
Locus_23279_Transcript_1/1_Conf_1.000	143	20	5.34E-16	XP_002342425.1	100	87.4261	41	41	PREDICTED: hypothetical protein XP_002342425
Locus_2328_Transcript_1/2_Conf_1.000	570	0							
Locus_2328_Transcript_2/2_Conf_1.000	438	0							
Locus_23280_Transcript_1/1_Conf_1.000	159	8	6.02E-23	XP_002632635.1	98	110.538	51	50	C. briggsae CBR-DMD-6 protein
Locus_23281_Transcript_1/1_Conf_1.000	244	0							
Locus_23282_Transcript_1/1_Conf_1.000	219	0							
Locus_23283_Transcript_1/1_Conf_1.000	221	3	4.24E-08	XP_002636414.1	61	61.2326	72	44	Hypothetical protein CBG23067
Locus_23284_Transcript_1/1_Conf_1.000	202	0							
Locus_23285_Transcript_1/1_Conf_1.000	492	0							
Locus_23286_Transcript_1/2_Conf_1.000	409	9	1.51E-34	NP_496430.1	90	149.058	82	74	hypothetical protein C33B4.2
Locus_23286_Transcript_2/2_Conf_1.000	409	7	5.19E-35	NP_496430.1	91	150.599	82	75	hypothetical protein C33B4.2
Locus_23287_Transcript_1/1_Conf_1.000	240	3	2.96E-14	XP_002634119.1	69	81.6481	79	55	Hypothetical protein CBG01673
Locus_23288_Transcript_1/1_Conf_1.000	164	20	1.29E-25	2DDY	100	119.398	54	54	SolutionStructure Of Matrilysin (Mmp-7) Complexed To Constraint Conformational Sulfonamide Inhibitor
Locus_23289_Transcript_1/1_Conf_1.000	263	20	7.39E-13	XP_001897247.1	65	77.0258	85	56	Strictosidine synthase family protein
Locus_2329_Transcript_1/1_Conf_1.000	226	4	1.76E-22	XP_002631267.1	81	108.997	75	61	C. briggsae CBR-PRO-1 protein
Locus_23290_Transcript_1/1_Conf_1.000	159	0							
Locus_23291_Transcript_1/1_Conf_1.000	193	0							
Locus_23292_Transcript_1/1_Conf_1.000	324	0							
Locus_23293_Transcript_1/1_Conf_1.000	168	7	2.13E-04	XP_002848567.1	73	48.9062	41	30	ADP-ribosylation factor
Locus_23294_Transcript_1/1_Conf_1.000	328	1	1.09E-16	AAB32534.1	100	89.7373	39	39	histone H1 transcription factor large subunit 2A
Locus_23295_Transcript_1/1_Conf_1.000	212	0							
Locus_23296_Transcript_1/1_Conf_1.000	134	0							
Locus_23297_Transcript_1/1_Conf_1.000	198	0							
Locus_23298_Transcript_1/1_Conf_1.000	130	0							
Locus_23299_Transcript_1/1_Conf_1.000	349	0							
Locus_233_Transcript_1/1_Conf_1.000	506	20	8.67E-25	XP_002644823.1	59	116.701	153	91	C. briggsae CBR-TAG-275 protein
Locus_2330_Transcript_1/2_Conf_1.000	600	0							
Locus_2330_Transcript_2/2_Conf_1.000	596	0							

Locus_23300_Transcript_1/1_Conf_1.000	176	0							
Locus_23301_Transcript_1/1_Conf_1.000	329	5	4.75E-12	XP_002630535.1	57	74.3294	89	51	C. briggsae CBR-TAG-308 protein
Locus_23302_Transcript_1/1_Conf_1.000	199	0							
Locus_23303_Transcript_1/1_Conf_1.000	287	20	1.26E-36	ACI49226.1	87	155.992	95	83	hypothetical protein Csp3_JD05.011
Locus_23304_Transcript_1/1_Conf_1.000	405	0							
Locus_23305_Transcript_1/1_Conf_1.000	286	0							
Locus_23306_Transcript_1/1_Conf_1.000	150	0							
Locus_23307_Transcript_1/1_Conf_1.000	240	0							
Locus_23308_Transcript_1/1_Conf_1.000	255	1	9.75E-05	CBH39183.1	57	50.0618	73	42	conserved hypothetical protein, DUF255 family
Locus_23309_Transcript_1/1_Conf_1.000	194	20	7.65E-18	XP_002637256.1	77	93.5893	63	49	Hypothetical protein CBG18935
Locus_2331_Transcript_1/1_Conf_1.000	1737	20	4.28E-76	XP_002735266.1	50	290.426	542	271	PREDICTED: lethal giant larvae homolog 1-like
Locus_23310_Transcript_1/1_Conf_1.000	143	0							
Locus_23311_Transcript_1/1_Conf_1.000	158	0							
Locus_23312_Transcript_1/1_Conf_1.000	283	0							
Locus_23313_Transcript_1/1_Conf_1.000	256	4	6.06E-31	XP_002643867.1	91	137.117	83	76	Hypothetical protein CBG02103
Locus_23314_Transcript_1/1_Conf_1.000	221	0							
Locus_23315_Transcript_1/1_Conf_1.000	181	13	7.03E-11	XP_002648535.1	77	70.4774	59	46	Hypothetical protein CBG24833
Locus_23316_Transcript_1/1_Conf_1.000	306	20	5.01E-30	NP_497814.3	77	134.035	101	78	UNCoordinated family member (unc-79)
Locus_23317_Transcript_1/1_Conf_1.000	380	11	1.29E-17	XP_001896168.1	62	92.8189	108	68	SET domain containing protein
Locus_23318_Transcript_1/1_Conf_1.000	164	1	1.64E-04	EFO14283.1	69	49.2914	52	36	hypothetical protein LOAG_14239
Locus_23319_Transcript_1/1_Conf_1.000	231	0							
Locus_2332_Transcript_1/1_Conf_1.000	1141	20	1.29E-154	XP_002642936.1	90	550.436	341	310	C. briggsae CBR-MDH-1 protein
Locus_23320_Transcript_1/1_Conf_1.000	263	0							
Locus_23321_Transcript_1/1_Conf_1.000	145	0							
Locus_23322_Transcript_1/1_Conf_1.000	134	0							
Locus_23323_Transcript_1/1_Conf_1.000	199	20	2.88E-25	NP_496219.1	90	118.242	66	60	Guanylyl CYclase family member (gcy-5)
Locus_23324_Transcript_1/1_Conf_1.000	406	0							

Locus_23325_Transcript_1/1_Conf_1.000	143	1	2.25E-06	NP_001021657.2	77	55.4546	36	28	hypothetical protein W03F11.5
Locus_23326_Transcript_1/1_Conf_1.000	255	0							
Locus_23327_Transcript_1/1_Conf_1.000	184	0							
Locus_23328_Transcript_1/1_Conf_1.000	189	20	7.18E-16	EFN89112.1	82	87.0409	57	47	F-actin-capping protein subunit alpha
Locus_23329_Transcript_1/1_Conf_1.000	195	0							
Locus_2333_Transcript_1/1_Conf_1.000	1837	20	1.64E-65	XP_002642511.1	54	255.373	430	233	Hypothetical protein CBG06936
Locus_23330_Transcript_1/1_Conf_1.000	444	0							
Locus_23331_Transcript_1/1_Conf_1.000	159	5	4.19E-08	AAB42328.2	70	61.2326	51	36	Hypothetical protein C32E8.11
Locus_23332_Transcript_1/1_Conf_1.000	155	0							
Locus_23333_Transcript_1/1_Conf_1.000	251	0							
Locus_23334_Transcript_1/1_Conf_1.000	129	20	2.77E-20	XP_002924566.1	100	101.679	42	42	PREDICTED: LIM domain-containing protein 2-like
Locus_23335_Transcript_1/1_Conf_1.000	147	0							
Locus_23336_Transcript_1/1_Conf_1.000	151	0							
Locus_23337_Transcript_1/1_Conf_1.000	186	0							
Locus_23338_Transcript_1/1_Conf_1.000	208	0							
Locus_23339_Transcript_1/1_Conf_1.000	134	20	2.86E-17	XP_002919669.1	100	91.6633	44	44	PREDICTED: n-alpha-acetyltransferase 20, NatB catalytic subunit-like isoform 3
Locus_2334_Transcript_1/1_Conf_1.000	323	0							
Locus_23340_Transcript_1/1_Conf_1.000	189	20	1.40E-11	CAX65070.1	81	72.7886	53	43	C. elegans protein R74.4b, confirmed by transcript evidence
Locus_23341_Transcript_1/1_Conf_1.000	141	20	8.24E-17	NP_510700.3	91	90.1225	47	43	Histone DeAcetylase family member (hda-4)
Locus_23342_Transcript_1/1_Conf_1.000	133	0							
Locus_23343_Transcript_1/1_Conf_1.000	297	20	1.58E-23	XP_002634426.1	67	112.464	98	66	Hypothetical protein CBG04437
Locus_23344_Transcript_1/1_Conf_1.000	139	0							

Locus_23345_Transcript_1/1_Conf_1.000	136	16	6.15E-12	NP_510520.4	91	73.9442	45	41	Osm-9 and Capsaicin receptor-Related family member (ocr-3)
Locus_23346_Transcript_1/1_Conf_1.000	179								
Locus_23347_Transcript_1/1_Conf_1.000	160	0							
Locus_23348_Transcript_1/1_Conf_1.000	140	0							
Locus_23349_Transcript_1/1_Conf_1.000	305	0							
Locus_2335_Transcript_1/1_Conf_1.000	1570	1	2.48E-11	XP_001902673.1	44	75.0998	396	175	hypothetical protein Bm1_56075
Locus_23350_Transcript_1/1_Conf_1.000	236	0							
Locus_23351_Transcript_1/1_Conf_1.000	128	0							
Locus_23352_Transcript_1/1_Conf_1.000	933	4	5.99E-24	NP_509471.2	59	115.931	233	138	hypothetical protein F53A9.4
Locus_23353_Transcript_1/1_Conf_1.000	325	20	3.75E-41	NP_508154.2	89	171.014	93	83	Nematode AStacin protease family member (nas-15)
Locus_23354_Transcript_1/1_Conf_1.000	255	0							
Locus_23355_Transcript_1/1_Conf_1.000	148	7	7.24E-08	ACJ65168.1	77	60.4622	49	38	Uncoordinated protein 68, isoform b, partially confirmed by transcript evidence
Locus_23356_Transcript_1/1_Conf_1.000	157	20	3.00E-22	EFO27462.1	100	108.227	52	52	hypothetical protein LOAG_01021
Locus_23357_Transcript_1/1_Conf_1.000	187	0							
Locus_23358_Transcript_1/1_Conf_1.000	129	20	1.09E-16	XP_002829423.1	100	89.7373	42	42	PREDICTED: LOW QUALITY PROTEIN: mitochondrial import receptor subunit TOM40 homolog
Locus_23359_Transcript_1/1_Conf_1.000	363	0							
Locus_2336_Transcript_1/2_Conf_1.000	139	0							
Locus_2336_Transcript_2/2_Conf_1.000	132	0							

Locus_23360_Transcript_1/1_Conf_1.000	251	20	3.23E-21	XP_002640311.1	79	104.76	82	65	C. briggsae CBR-LET-363 protein
Locus_23361_Transcript_1/1_Conf_1.000	131	0							
Locus_23362_Transcript_1/1_Conf_1.000	159	0							
Locus_23363_Transcript_1/1_Conf_1.000	250	0							
Locus_23364_Transcript_1/1_Conf_1.000	259	20	2.72E-23	XP_002636068.1	76	111.694	84	64	Hypothetical protein CBG01308
Locus_23365_Transcript_1/1_Conf_1.000	263	5	5.30E-11	NP_497641.2	72	70.8626	86	62	CaDHerin family member (cdh-1)
Locus_23366_Transcript_1/1_Conf_1.000	232	5	3.31E-13	Q60UZ5.2	71	78.1814	77	55	Mediator of RNA polymerase II transcription subunit 23
Locus_23367_Transcript_1/1_Conf_1.000	235	0							
Locus_23368_Transcript_1/1_Conf_1.000	214	20	9.74E-26	XP_001891908.1	98	119.783	66	65	SNF7 family protein
Locus_23369_Transcript_1/1_Conf_1.000	172	4	8.59E-22	NP_001022641.1	91	106.686	57	52	LEThal family member (let-805)
Locus_2337_Transcript_1/1_Conf_1.000	534	20	1.58E-41	XP_002629939.1	89	172.555	103	92	Hypothetical protein CBG03659
Locus_23370_Transcript_1/1_Conf_1.000	178	0							

Locus_23371_Transcript_1/1_Conf_1.000	130	0							
Locus_23372_Transcript_1/1_Conf_1.000	163	14	4.62E-07	AAK64493.1	67	57.7658	49	33	AF388311_1C-type lectin CTL-2 precursor
Locus_23373_Transcript_1/1_Conf_1.000	314	2	2.56E-21	ACE63491.1	67	105.145	97	65	GABAB2 receptor subunit
Locus_23374_Transcript_1/1_Conf_1.000	306	0							
Locus_23375_Transcript_1/1_Conf_1.000	148	0							
Locus_23376_Transcript_1/1_Conf_1.000	141	0							
Locus_23377_Transcript_1/1_Conf_1.000	156	20	2.54E-13	XP_002918275.1	100	78.5666	35	35	PREDICTED: collagen alpha-2(I) chain-like
Locus_23378_Transcript_1/1_Conf_1.000	176	0							
Locus_23379_Transcript_1/1_Conf_1.000	223	0							
Locus_2338_Transcript_1/2_Conf_1.000	510	0							
Locus_2338_Transcript_2/2_Conf_1.000	510	0							
Locus_23380_Transcript_1/1_Conf_1.000	171	2	2.43E-08	XP_002641539.1	70	62.003	54	38	C. briggsae CBR-SET-27 protein
Locus_23381_Transcript_1/1_Conf_1.000	336	0							
Locus_23382_Transcript_1/1_Conf_1.000	163	0							
Locus_23383_Transcript_1/1_Conf_1.000	156	0							
Locus_23384_Transcript_1/1_Conf_1.000	184	20	9.42E-24	XP_002919957.1	100	113.235	53	53	PREDICTED: activated RNA polymerase II transcriptional coactivator p15-like
Locus_23385_Transcript_1/1_Conf_1.000	149	2	5.92E-10	XP_002643350.1	77	67.3958	45	35	Hypothetical protein CBG15947
Locus_23386_Transcript_1/1_Conf_1.000	243	0							
Locus_23387_Transcript_1/1_Conf_1.000	288	0							
Locus_23388_Transcript_1/1_Conf_1.000	160	0							
Locus_23389_Transcript_1/1_Conf_1.000	318	7	8.20E-12	NP_497229.1	70	73.559	93	66	hypothetical protein W06E11.1
Locus_2339_Transcript_1/1_Conf_1.000	1339	20	1.14E-22	EFO18116.1	46	112.464	463	215	api5-prov protein
Locus_23390_Transcript_1/1_Conf_1.000	158	0							

Locus_23391_Transcript_1/1_Conf_1.000	147	0							
Locus_23392_Transcript_1/1_Conf_1.000	149	0							
Locus_23393_Transcript_1/1_Conf_1.000	163	0							
Locus_23394_Transcript_1/1_Conf_1.000	160	20	1.76E-06	NP_490949.3	69	55.8398	53	37	ABC Transporter family member (abt-2)
Locus_23395_Transcript_1/1_Conf_1.000	262	0							
Locus_23396_Transcript_1/1_Conf_1.000	365	9	3.13E-32	AAA83446.5	80	141.354	109	88	Hypothetical protein F44A2.3
Locus_23397_Transcript_1/1_Conf_1.000	270	0							
Locus_23398_Transcript_1/1_Conf_1.000	152	0							
Locus_23399_Transcript_1/1_Conf_1.000	142	20	3.03E-19	XP_002921914.1	100	98.2117	47	47	PREDICTED: eukaryotic translation initiation factor 3 subunit I-like
Locus_234_Transcript_1/1_Conf_1.000	568	20	2.80E-61	NP_506256.3	67	238.424	187	126	CaDHerin family member (cdh-6)
Locus_2340_Transcript_1/1_Conf_1.000	666	6	1.21E-60	NP_492439.1	83	236.884	160	133	hypothetical protein T06D10.1
Locus_23400_Transcript_1/1_Conf_1.000	140	0							
Locus_23401_Transcript_1/1_Conf_1.000	229	0							

Locus_23402_Transcript_1/1_Conf_1.000	410	20	6.98E-48	XP_002639323.1	78	193.356	134	105	C. briggsae CBR-HUM-7 protein
Locus_23403_Transcript_1/1_Conf_1.000	137	4	3.99E-11	XP_002646095.1	86	71.2478	45	39	C. briggsae CBR-HMR-1 protein
Locus_23404_Transcript_1/1_Conf_1.000	183	1	4.25E-08	XP_002641351.1	73	61.2326	60	44	C. briggsae CBR-TEG-1 protein
Locus_23405_Transcript_1/1_Conf_1.000	214	0							
Locus_23406_Transcript_1/1_Conf_1.000	655	0							
Locus_23407_Transcript_1/1_Conf_1.000	319	0							
Locus_23408_Transcript_1/1_Conf_1.000	658	0							
Locus_23409_Transcript_1/1_Conf_1.000	240	20	4.71E-28	XP_002632772.1	82	127.487	79	65	C. briggsae CBR-TAT-2 protein
Locus_2341_Transcript_1/1_Conf_1.000	348	0							
Locus_23410_Transcript_1/1_Conf_1.000	284	0							
Locus_23411_Transcript_1/1_Conf_1.000	147	0							
Locus_23412_Transcript_1/1_Conf_1.000	278	9	6.31E-41	NP_001022641.1	89	170.244	92	82	LEThal family member (let-805)
Locus_23413_Transcript_1/1_Conf_1.000	174	20	5.74E-18	AAF36480.1	87	93.9745	56	49	glutathione S-transferase 2
Locus_23414_Transcript_1/1_Conf_1.000	172	20	5.38E-24	XP_002834167.1	100	114.005	56	56	PREDICTED: peptidyl-tRNA hydrolase 2, mitochondrial-like isoform 2
Locus_23415_Transcript_1/1_Conf_1.000	240	20	1.23E-44	NP_001182611.1	100	182.57	78	78	metalloproteinase inhibitor 3
Locus_23416_Transcript_1/1_Conf_1.000	338	20	1.11E-45	XP_002642615.1	81	186.037	112	91	C. briggsae CBR-MUP-4 protein

Locus_23417_Transcript_1/1_Conf_1.000	160	0							
Locus_23418_Transcript_1/1_Conf_1.000	386	4	8.91E-11	NP_001021653.1	61	70.0922	126	78	FANCI (Fanconi anemia complex component I) homolog family member (fnci-1)
Locus_23419_Transcript_1/1_Conf_1.000	683	20	9.46E-72	NP_499173.1	82	273.863	206	169	hypothetical protein ZK632.3
Locus_2342_Transcript_1/1_Conf_1.000	374	20	9.61E-37	XP_002643252.1	78	156.377	114	89	C. briggsae CBR-TRAP-2 protein
Locus_23420_Transcript_1/1_Conf_1.000	137	0							
Locus_23421_Transcript_1/1_Conf_1.000	290	3	7.18E-16	XP_002637525.1	74	87.0409	75	56	Hypothetical protein CBG19248
Locus_23422_Transcript_1/1_Conf_1.000	157	0							
Locus_23423_Transcript_1/1_Conf_1.000	212	0							
Locus_23424_Transcript_1/1_Conf_1.000	172	0							
Locus_23425_Transcript_1/1_Conf_1.000	169	20	2.51E-13	NP_001024724.2	80	78.5666	56	45	EGg Laying defective family member (egl-15)
Locus_23426_Transcript_1/1_Conf_1.000	182	0							
Locus_23427_Transcript_1/1_Conf_1.000	151	0							
Locus_23428_Transcript_1/1_Conf_1.000	152	0							
Locus_23429_Transcript_1/1_Conf_1.000	137	0							
Locus_2343_Transcript_1/2_Conf_1.000	1864	20	6.27E-113	XP_002630571.1	70	412.92	403	285	Hypothetical protein CBG13024
Locus_2343_Transcript_2/2_Conf_1.000	1788	20	5.94E-113	XP_002630571.1	70	412.92	403	285	Hypothetical protein CBG13024
Locus_23430_Transcript_1/1_Conf_1.000	187	0							
Locus_23431_Transcript_1/1_Conf_1.000	306	3	2.50E-05	XP_002642803.1	82	51.9878	40	33	C. briggsae CBR-TAG-164 protein
Locus_23432_Transcript_1/1_Conf_1.000	136	0							
Locus_23433_Transcript_1/1_Conf_1.000	255	0							
Locus_23434_Transcript_1/1_Conf_1.000	194	20	9.66E-21	CAA86821.1	87	103.219	64	56	excretory/secretory antigen
Locus_23435_Transcript_1/1_Conf_1.000	261	0							
Locus_23436_Transcript_1/1_Conf_1.000	209	20	2.18E-25	XP_002634716.1	95	118.627	69	66	C. briggsae CBR-EXC-5 protein
Locus_23437_Transcript_1/1_Conf_1.000	261	0							
Locus_23438_Transcript_1/1_Conf_1.000	157	20	4.63E-23	NP_001034792.3	100	110.923	52	52	neuroblastoma breakpoint family member 10
Locus_23439_Transcript_1/1_Conf_1.000	165	4	7.10E-08	XP_001893303.1	67	60.4622	53	36	ribosomal RNA adenine dimethylase family protein
Locus_2344_Transcript_1/2_Conf_1.000	1084	20	3.64E-10	XP_002641794.1	65	70.4774	61	40	Hypothetical protein CBG10147
Locus_2344_Transcript_2/2_Conf_1.000	1095	20	3.71E-10	XP_002641794.1	65	70.4774	61	40	Hypothetical protein CBG10147
Locus_23440_Transcript_1/1_Conf_1.000	159	20	1.35E-14	XP_002640523.1	84	82.8037	51	43	Hypothetical protein CBG18685
Locus_23441_Transcript_1/1_Conf_1.000	301	0							

Locus_23442_Transcript_1/1_Conf_1.000	169	0							
Locus_23443_Transcript_1/1_Conf_1.000	504	20	1.32E-65	NP_490772.2	88	252.292	159	141	hypothetical protein Y18H1A.3
Locus_23444_Transcript_1/1_Conf_1.000	165	20	4.02E-11	XP_002639959.1	86	71.2478	46	40	Hypothetical protein CBG10779
Locus_23445_Transcript_1/1_Conf_1.000	330	0							
Locus_23446_Transcript_1/1_Conf_1.000	274	0							
Locus_23447_Transcript_1/1_Conf_1.000	161	0							
Locus_23448_Transcript_1/1_Conf_1.000	165	20	1.38E-19	NP_004137.2	100	99.3673	42	42	NADH dehydrogenase ubiquinone 1 beta subcomplex subunit 7
Locus_23449_Transcript_1/1_Conf_1.000	352	0							
Locus_2345_Transcript_1/1_Conf_1.000	1819	20	0	NP_001040881.1	71	650.588	582	414	WHiTe (Drosophila) related ABC transporte family member (wht-7)
Locus_23450_Transcript_1/1_Conf_1.000	198	2	2.70E-15	NP_001076730.1	86	85.1149	52	45	hypothetical protein Y41E3.22
Locus_23451_Transcript_1/1_Conf_1.000	139	2	2.26E-06	XP_002630900.1	79	55.4546	43	34	Hypothetical protein CBG02622
Locus_23452_Transcript_1/1_Conf_1.000	202	0							
Locus_23453_Transcript_1/1_Conf_1.000	159	0							
Locus_23454_Transcript_1/1_Conf_1.000	308	0							
Locus_23455_Transcript_1/1_Conf_1.000	221	0							
Locus_23456_Transcript_1/1_Conf_1.000	184	20	8.52E-25	NP_509350.2	90	116.701	61	55	hypothetical protein C34D10.2
Locus_23457_Transcript_1/1_Conf_1.000	162	0							
Locus_23458_Transcript_1/1_Conf_1.000	134	0							
Locus_23459_Transcript_1/1_Conf_1.000	382	1	4.76E-04	EFO21065.1	50	47.7506	89	45	hypothetical protein LOAG_07427
Locus_2346_Transcript_1/2_Conf_1.000	4361	20	7.65E-118	NP_498678.3	42	430.639	1286	548	abnormal cell LINeage family member (lin-13)
Locus_2346_Transcript_2/2_Conf_1.000	2584	20	9.64E-86	NP_498678.3	46	323.168	703	329	abnormal cell LINeage family member (lin-13)
Locus_23460_Transcript_1/1_Conf_1.000	132	0							
Locus_23461_Transcript_1/1_Conf_1.000	389	0							

Locus_23462_Transcript_1/1_Conf_1.000	307	20	1.91E-13	NP_001006002.1	67	78.9518	67	45	galactokinase
Locus_23463_Transcript_1/1_Conf_1.000	332	0							
Locus_23464_Transcript_1/1_Conf_1.000	201	0							
Locus_23465_Transcript_1/1_Conf_1.000	300	6	8.07E-20	AAX48771.1	95	100.138	60	57	FMRF amide-like protein 7
Locus_23466_Transcript_1/1_Conf_1.000	505	0							
Locus_23467_Transcript_1/1_Conf_1.000	334	20	1.98E-10	XP_001901255.1	60	68.9366	106	64	COMM domain containing 4
Locus_23468_Transcript_1/1_Conf_1.000	320	0							
Locus_23469_Transcript_1/1_Conf_1.000	313	20	6.11E-15	XP_002633416.1	56	83.9593	125	70	Hypothetical protein CBG06181
Locus_2347_Transcript_1/1_Conf_1.000	2252	20	0	NP_502420.2	77	916.376	760	591	hypothetical protein C26H9A.2
Locus_23470_Transcript_1/1_Conf_1.000	263	1	8.19E-04	XP_002613981.1	45	46.9802	70	32	hypothetical protein BRAFLDRAFT_118457
Locus_23471_Transcript_1/1_Conf_1.000	278	7	6.53E-43	XP_002644616.1	91	170.244	87	80	Hypothetical protein CBG14575
Locus_23472_Transcript_1/1_Conf_1.000	412	20	5.51E-45	NP_495209.1	81	183.726	138	113	hypothetical protein R12C12.1
Locus_23473_Transcript_1/1_Conf_1.000	235	20	1.75E-14	XP_001524562.1	72	82.4185	77	56	26S proteasome regulatory subunit RPN6
Locus_23474_Transcript_1/1_Conf_1.000	158	0							
Locus_23475_Transcript_1/1_Conf_1.000	354	2	4.45E-10	XP_001899872.1	55	67.781	87	48	Protein-tyrosine phosphatase containing protein

Locus_23476_Transcript_1/1_Conf_1.000	190	20	1.35E-30	XP_002913719.1	100	135.961	63	63	PREDICTED: actin-related protein 2/3 complex subunit 2-like
Locus_23477_Transcript_1/1_Conf_1.000	308	20	8.29E-17	NP_500127.2	60	90.1225	102	62	hypothetical protein K08D12.2
Locus_23478_Transcript_1/1_Conf_1.000	232	3	4.63E-07	XP_002635372.1	68	57.7658	73	50	C. briggsae CBR-RPM-1 protein
Locus_23479_Transcript_1/1_Conf_1.000	136	0							
Locus_2348_Transcript_1/2_Conf_1.000	1264	20	1.03E-62	XP_002643530.1	55	245.358	400	223	C. briggsae CBR-CAB-1 protein
Locus_2348_Transcript_2/2_Conf_1.000	1188	20	2.20E-46	XP_002643530.1	86	115.161	75	65	C. briggsae CBR-CAB-1 protein
Locus_23480_Transcript_1/1_Conf_1.000	312	0							
Locus_23481_Transcript_1/1_Conf_1.000	222	0							
Locus_23482_Transcript_1/1_Conf_1.000	576	20	2.64E-94	NP_503410.3	92	348.206	176	162	hypothetical protein R12A1.3
Locus_23483_Transcript_1/1_Conf_1.000	144	0							
Locus_23484_Transcript_1/1_Conf_1.000	181	4	2.26E-17	XP_002648388.1	84	92.0485	57	48	Hypothetical protein CBG24638
Locus_23485_Transcript_1/1_Conf_1.000	284	0							
Locus_23486_Transcript_1/1_Conf_1.000	347	20	1.10E-48	XP_001897519.1	91	196.052	115	105	symbol
Locus_23487_Transcript_1/1_Conf_1.000	179	0							
Locus_23488_Transcript_1/1_Conf_1.000	135	3	5.58E-05	NP_506407.1	73	50.8322	42	31	hypothetical protein F20G2.2
Locus_23489_Transcript_1/1_Conf_1.000	147	4	1.01E-09	XP_002644860.1	86	66.6254	46	40	Hypothetical protein CBG05033
Locus_2349_Transcript_1/1_Conf_1.000	432	2	3.38E-10	XP_002646300.1	66	68.1662	78	52	Hypothetical protein CBG12007
Locus_23490_Transcript_1/1_Conf_1.000	477	10	1.37E-19	NP_494916.1	68	99.3673	106	73	SHC (Src Homology domain C-terminal) adaptor homolog family member (shc-2)
Locus_23491_Transcript_1/1_Conf_1.000	237	0							
Locus_23492_Transcript_1/1_Conf_1.000	211	0							
Locus_23493_Transcript_1/1_Conf_1.000	156	0							
Locus_23494_Transcript_1/1_Conf_1.000	394	0							

Locus_23495_Transcript_1/1_Conf_1.000	567	20	2.63E-59	NP_491658.2	74	231.876	190	141	PaTched Related family member (ptr-10)
Locus_23496_Transcript_1/1_Conf_1.000	162	20	1.03E-14	XP_002632718.1	75	83.1889	54	41	Hypothetical protein CBG18748
Locus_23497_Transcript_1/1_Conf_1.000	168	0							
Locus_23498_Transcript_1/1_Conf_1.000	286	20	3.35E-13	XP_002642338.1	70	78.1814	81	57	C. briggsae CBR-DHS-11 protein
Locus_23499_Transcript_1/2_Conf_1.000	175	0							
Locus_23499_Transcript_2/2_Conf_1.000	187	0							
Locus_235_Transcript_1/1_Conf_1.000	1048	7	4.65E-15	XP_001892092.1	72	86.6557	94	68	reticulocyte binding-like protein 2b
Locus_2350_Transcript_1/1_Conf_1.000	258	20	1.65E-20	NP_500837.1	72	102.449	85	62	hypothetical protein C39H7.1
Locus_23500_Transcript_1/1_Conf_1.000	216	0							
Locus_23501_Transcript_1/1_Conf_1.000	319	20	1.03E-30	EFO26545.1	79	136.346	105	83	kelch domain-containing protein family protein
Locus_23502_Transcript_1/1_Conf_1.000	143	1	1.56E-07	NP_001123118.1	80	59.3066	35	28	hypothetical protein C44C10.9
Locus_23503_Transcript_1/1_Conf_1.000	158	2	9.69E-05	NP_496542.1	83	50.0618	43	36	hypothetical protein ZK930.1
Locus_23504_Transcript_1/1_Conf_1.000	358	0							
Locus_23505_Transcript_1/1_Conf_1.000	665	2	1.10E-29	NP_499472.1	67	134.035	125	84	Sterol regulatory element Binding Protein family member (sbp-1)
Locus_23506_Transcript_1/1_Conf_1.000	158	20	2.54E-21	XP_002829216.1	96	105.145	52	50	PREDICTED: eukaryotic translation initiation factor 3 subunit K-like
Locus_23507_Transcript_1/1_Conf_1.000	212	0							
Locus_23508_Transcript_1/1_Conf_1.000	228	0							
Locus_23509_Transcript_1/1_Conf_1.000	145	0							
Locus_2351_Transcript_1/1_Conf_1.000	443	20	1.81E-43	EFO27551.1	76	178.718	147	112	hypothetical protein LOAG_00931
Locus_23510_Transcript_1/1_Conf_1.000	339	12	4.95E-46	NP_506408.1	85	187.193	113	97	hypothetical protein F20G2.3
Locus_23511_Transcript_1/1_Conf_1.000	186	0							
Locus_23512_Transcript_1/1_Conf_1.000	182	0							

Locus_23513_Transcript_1/1_Conf_1.000	157	6	2.46E-08	NP_001021110.1	70	62.003	51	36	CAlSYntenin/Alcadein homolog family member (casy-1)
Locus_23514_Transcript_1/1_Conf_1.000	168	20	5.41E-16	NP_493642.1	92	87.4261	56	52	hypothetical protein F23F1.6
Locus_23515_Transcript_1/1_Conf_1.000	381	20	2.86E-25	XP_002645261.1	80	118.242	86	69	Hypothetical protein CBG00142
Locus_23516_Transcript_1/1_Conf_1.000	163	0							
Locus_23517_Transcript_1/1_Conf_1.000	195	0							
Locus_23518_Transcript_1/1_Conf_1.000	197	0							
Locus_23519_Transcript_1/1_Conf_1.000	163	0							
Locus_2352_Transcript_1/1_Conf_1.000	554	0							
Locus_23520_Transcript_1/1_Conf_1.000	144	3	7.26E-08	EFO24290.1	73	60.4622	46	34	hypothetical protein LOAG_04198
Locus_23521_Transcript_1/1_Conf_1.000	178	0							
Locus_23522_Transcript_1/1_Conf_1.000	194	2	9.37E-08	XP_002630446.1	61	60.077	60	37	Hypothetical protein CBG11179
Locus_23523_Transcript_1/1_Conf_1.000	205	20	1.63E-20	XP_002633097.1	79	102.449	68	54	C. briggsae CBR-RPC-1 protein
Locus_23524_Transcript_1/1_Conf_1.000	164	0							
Locus_23525_Transcript_1/1_Conf_1.000	318	3	3.22E-08	EFO16242.1	53	61.6178	83	44	hypothetical protein LOAG_12266
Locus_23526_Transcript_1/1_Conf_1.000	180	2	7.53E-05	XP_002648586.1	52	50.447	91	48	Hypothetical protein CBG24916
Locus_23527_Transcript_1/1_Conf_1.000	179	0							
Locus_23528_Transcript_1/1_Conf_1.000	174	0							
Locus_23529_Transcript_1/1_Conf_1.000	141	0							
Locus_2353_Transcript_1/3_Conf_0.600	721	20	8.76E-42	NP_001041048.1	59	174.481	204	122	EPS (human endocytosis) related family member (eps-8)
Locus_2353_Transcript_2/3_Conf_0.400	751	20	7.62E-31	NP_502803.3	53	138.272	213	113	EPS (human endocytosis) related family member (eps-8)
Locus_2353_Transcript_3/3_Conf_0.600	698	20	8.23E-42	NP_001041048.1	59	174.481	204	122	EPS (human endocytosis) related family member (eps-8)
Locus_23530_Transcript_1/1_Conf_1.000	277	20	2.10E-28	EFO24787.1	89	128.642	83	74	ATP-dependent Clp protease

Locus_23531_Transcript_1/1_Conf_1.000	313	20	4.05E-51	XP_002822113.1	100	204.142	104	104	PREDICTED: eukaryotic translation initiation factor 3 subunit F-like isoform 3
Locus_23532_Transcript_1/1_Conf_1.000	128	0							
Locus_23533_Transcript_1/1_Conf_1.000	255	20	1.27E-20	XP_002633417.1	84	102.834	76	64	C. briggsae CBR-TRPA-1 protein
Locus_23534_Transcript_1/1_Conf_1.000	208	20	7.75E-31	NP_001023038.1	98	136.732	69	68	EXCretory canal abnormal family member (exc-5)
Locus_23535_Transcript_1/1_Conf_1.000	279	20	4.49E-47	XP_002803216.1	100	190.66	93	93	PREDICTED: amyloid beta A4 protein-like, partial
Locus_23536_Transcript_1/1_Conf_1.000	155	0							
Locus_23537_Transcript_1/1_Conf_1.000	1347	20	4.79E-162	XP_002641814.1	80	575.474	437	351	C. briggsae CBR-BYN-1 protein

Locus_23538_Transcript_1/1_Conf_1.000	130	20	4.89E-17	XP_002931287.1	100	90.8929	43	43	PREDICTED: filamin-A-like, partial
Locus_23539_Transcript_1/1_Conf_1.000	187								
Locus_2354_Transcript_1/2_Conf_1.000	1575	20	1.62E-119	XP_001898426.1	56	434.491	575	325	Bromodomain containing protein
Locus_2354_Transcript_2/2_Conf_1.000	499	20	5.14E-43	NP_509770.2	76	177.178	135	103	hypothetical protein F57C7.1
Locus_23540_Transcript_1/1_Conf_1.000	174	1	9.51E-05	NP_499670.1	59	50.0618	57	34	hypothetical protein Y37D8A.4
Locus_23541_Transcript_1/1_Conf_1.000	213	0							
Locus_23542_Transcript_1/1_Conf_1.000	371	0							
Locus_23543_Transcript_1/1_Conf_1.000	169	20	1.92E-21	NP_001122771.1	88	105.531	53	47	hypothetical protein F13H10.4
Locus_23544_Transcript_1/1_Conf_1.000	261	0							
Locus_23545_Transcript_1/1_Conf_1.000	744	4	3.36E-31	EFO25848.1	69	139.428	175	122	zinc finger protein
Locus_23546_Transcript_1/1_Conf_1.000	149	0							
Locus_23547_Transcript_1/1_Conf_1.000	324	0							
Locus_23548_Transcript_1/1_Conf_1.000	228	0							
Locus_23549_Transcript_1/1_Conf_1.000	333	0							

Locus_2355_Transcript_1/1_Conf_1.000	348	20	6.83E-51	AAZ42325.1	98	203.371	103	101	translation elongation factor 1-alpha
Locus_23550_Transcript_1/1_Conf_1.000	810	20	9.95E-35	NP_501226.2	67	151.369	157	106	hypothetical protein F49E8.1
Locus_23551_Transcript_1/1_Conf_1.000	366	20	1.78E-11	XP_001899513.1	67	72.4034	68	46	AIDA-1b
Locus_23552_Transcript_1/1_Conf_1.000	214	1	2.76E-04	XP_002634850.1	65	48.521	60	39	Hypothetical protein CBG13967
Locus_23553_Transcript_1/1_Conf_1.000	321	20	1.13E-37	NP_492438.1	87	159.458	106	93	RADiation sensitivity abnormal/yeast RAD-related family member (rad-54)
Locus_23554_Transcript_1/1_Conf_1.000	131	0							
Locus_23555_Transcript_1/1_Conf_1.000	146	0							
Locus_23556_Transcript_1/1_Conf_1.000	206	0							
Locus_23557_Transcript_1/1_Conf_1.000	358	7	1.05E-11	NP_001021268.1	71	73.1738	115	82	UNCoordinated family member (unc-44)
Locus_23558_Transcript_1/1_Conf_1.000	181	20	9.16E-19	NP_491003.2	91	96.6709	60	55	Amino Acid Transporter family member (aat-5)
Locus_23559_Transcript_1/1_Conf_1.000	249	20	2.65E-23	NP_500943.1	86	111.694	65	56	hypothetical protein F58F9.7
Locus_2356_Transcript_1/3_Conf_0.714	1423	20	4.21E-47	NP_001018536.1	44	193.741	421	188	drebrin-like protein
Locus_2356_Transcript_2/3_Conf_0.714	1423	20	7.17E-47	NP_001018536.1	44	192.971	424	190	drebrin-like protein

Locus_2356_Transcript_3/3_Conf_0.714	1423	20	4.21E-47	NP_001018536.1	44	193.741	421	188	drebrin-like protein
Locus_23560_Transcript_1/1_Conf_1.000	323	4	3.50E-55	XP_002629930.1	96	217.624	105	101	Hypothetical protein CBG21978
Locus_23561_Transcript_1/1_Conf_1.000	260	4	3.94E-06	XP_002641855.1	62	54.6842	66	41	Hypothetical protein CBG16529
Locus_23562_Transcript_1/1_Conf_1.000	147	3	4.69E-07	XP_002639522.1	76	57.7658	43	33	Hypothetical protein CBG04128
Locus_23563_Transcript_1/1_Conf_1.000	150	0							
Locus_23564_Transcript_1/1_Conf_1.000	243	2	8.32E-09	XP_002634329.1	79	63.5438	48	38	C. briggsae CBR-TAG-321 protein
Locus_23565_Transcript_1/1_Conf_1.000	202	0							
Locus_23566_Transcript_1/1_Conf_1.000	174	20	9.77E-26	XP_001115060.2	100	119.783	57	57	PREDICTED: heat shock 70 kDa protein 1A/1B, partial
Locus_23567_Transcript_1/1_Conf_1.000	235	9	7.05E-40	ADN00778.1	97	166.777	77	75	LON-1 protein
Locus_23568_Transcript_1/1_Conf_1.000	412	20	1.31E-38	NP_496802.2	73	162.54	133	98	BTAF (TBP-associated factor) homolog family member (btf-1)
Locus_23569_Transcript_1/1_Conf_1.000	197	0							
Locus_2357_Transcript_1/1_Conf_1.000	774	20	2.10E-63	XP_001895436.1	66	246.514	268	177	Micro-fibrillar-associated protein 1 C-terminus containing protein
Locus_23570_Transcript_1/1_Conf_1.000	157	2	1.65E-12	CBL43426.1	84	75.8702	52	44	C. elegans protein C54E10.2b, confirmed by transcript evidence
Locus_23571_Transcript_1/1_Conf_1.000	136	20	7.52E-10	XP_002171431.1	86	67.0106	45	39	vacuolar ATP synthase subunit B
Locus_23572_Transcript_1/1_Conf_1.000	180	20	8.00E-23	EFO22245.1	96	110.153	59	57	ubiquitin-conjugating enzyme family protein

Locus_23573_Transcript_1/1_Conf_1.000	241	3	1.20E-23	NP_001022992.1	80	112.849	80	64	CYtoKinesis defect family member (cyk-3)
Locus_23574_Transcript_1/1_Conf_1.000	156	0							
Locus_23575_Transcript_1/1_Conf_1.000	190	0							
Locus_23576_Transcript_1/1_Conf_1.000	166	0							
Locus_23577_Transcript_1/1_Conf_1.000	212	0							
Locus_23578_Transcript_1/1_Conf_1.000	229	0							
Locus_23579_Transcript_1/1_Conf_1.000	129	0							
Locus_2358_Transcript_1/1_Conf_1.000	1379	5	1.54E-38	NP_872024.1	63	165.236	211	134	hypothetical protein F18C5.10
Locus_23580_Transcript_1/1_Conf_1.000	439	20	2.21E-62	XP_002752629.1	100	241.506	140	140	PREDICTED: CD63 antigen-like isoform 1
Locus_23581_Transcript_1/1_Conf_1.000	503	0							
Locus_23582_Transcript_1/1_Conf_1.000	158	0							
Locus_23583_Transcript_1/1_Conf_1.000	170	13	1.42E-24	BAG65491.1	100	115.931	56	56	unnamed protein product
Locus_23584_Transcript_1/1_Conf_1.000	334	20	3.47E-39	NP_491529.2	89	164.466	101	90	hypothetical protein Y110A7A.6
Locus_23585_Transcript_1/1_Conf_1.000	164	0							
Locus_23586_Transcript_1/1_Conf_1.000	204	20	4.28E-21	EFO18824.1	83	104.375	66	55	hypothetical protein LOAG_09672
Locus_23587_Transcript_1/1_Conf_1.000	210	20	3.14E-24	EFO21237.1	87	114.775	66	58	hypothetical protein LOAG_07254
Locus_23588_Transcript_1/1_Conf_1.000	196	0							
Locus_23589_Transcript_1/1_Conf_1.000	233	0							
Locus_2359_Transcript_1/2_Conf_1.000	1640	20	1.60E-125	NP_741885.1	77	454.521	366	284	NUCleoBindin homolog family member (nucb-1)

Locus_2359_Transcript_2/2_Conf_1.000	1601	20	5.69E-120	XP_002644192.1	76	436.032	375	285	C. briggsae CBR-NUCB-1 protein
Locus_23590_Transcript_1/1_Conf_1.000	662	6	1.41E-37	XP_002638375.1	67	160.229	190	128	Hypothetical protein CBG18580
Locus_23591_Transcript_1/1_Conf_1.000	318	0							
Locus_23592_Transcript_1/1_Conf_1.000	345	0							
Locus_23593_Transcript_1/1_Conf_1.000	141	0							
Locus_23594_Transcript_1/1_Conf_1.000	329	0							
Locus_23595_Transcript_1/1_Conf_1.000	129	20	4.89E-17	XP_002831617.1	100	90.8929	43	43	PREDICTED: serine/threonine-protein kinase A-Raf-like
Locus_23596_Transcript_1/1_Conf_1.000	216	0							
Locus_23597_Transcript_1/1_Conf_1.000	237	0							
Locus_23598_Transcript_1/1_Conf_1.000	296	0							
Locus_23599_Transcript_1/1_Conf_1.000	159	20	1.44E-16	XP_002639507.1	84	89.3521	52	44	C. briggsae CBR-LIN-17 protein
Locus_236_Transcript_1/4_Conf_0.250	844	20	2.94E-16	EFO27939.1	71	90.1225	85	61	hypothetical protein LOAG_00532
Locus_236_Transcript_2/4_Conf_0.500	976	20	3.76E-16	EFO27939.1	71	90.1225	85	61	hypothetical protein LOAG_00532
Locus_236_Transcript_3/4_Conf_0.500	1137	20	4.80E-16	EFO27939.1	71	90.1225	85	61	hypothetical protein LOAG_00532
Locus_236_Transcript_4/4_Conf_0.375	1156	20	5.39E-15	EFO27939.1	75	86.6557	74	56	hypothetical protein LOAG_00532
Locus_2360_Transcript_1/4_Conf_0.444	1248	20	1.59E-156	NP_503064.2	78	556.984	409	323	Amino acid Transporter GlycoProtein subunit family member (atgp-1)
Locus_2360_Transcript_2/4_Conf_0.667	1905	20	0	NP_503064.2	74	756.518	617	461	Amino acid Transporter GlycoProtein subunit family member (atgp-1)
Locus_2360_Transcript_3/4_Conf_0.667	1905	20	0	NP_503064.2	74	756.133	617	461	Amino acid Transporter GlycoProtein subunit family member (atgp-1)
Locus_2360_Transcript_4/4_Conf_0.667	1905	20	0	NP_503064.2	74	756.133	617	461	Amino acid Transporter GlycoProtein subunit family member (atgp-1)
Locus_23600_Transcript_1/1_Conf_1.000	150	20	1.23E-15	EFO17879.1	87	86.2705	48	42	hypothetical protein LOAG_10616
Locus_23601_Transcript_1/1_Conf_1.000	189	0							

Locus_23602_Transcript_1/1_Conf_1.000	225	20	1.39E-27	XP_002634807.1	85	125.946	74	63	Hypothetical protein CBG13912
Locus_23603_Transcript_1/1_Conf_1.000	155	0							
Locus_23604_Transcript_1/1_Conf_1.000	241	0							
Locus_23605_Transcript_1/1_Conf_1.000	169	0							
Locus_23606_Transcript_1/1_Conf_1.000	159	0							
Locus_23607_Transcript_1/1_Conf_1.000	187	20	2.17E-12	XP_002648059.1	78	75.485	60	47	Hypothetical protein CBG24048
Locus_23608_Transcript_1/1_Conf_1.000	199	20	3.19E-16	EDL39643.1	100	88.1965	55	55	small nuclear ribonucleoprotein E, isoform CRA_a
Locus_23609_Transcript_1/1_Conf_1.000	184	0							
Locus_2361_Transcript_1/1_Conf_1.000	1589	20	1.49E-96	XP_002642323.1	60	358.221	526	317	C. briggsae CBR-RIB-2 protein
Locus_23610_Transcript_1/1_Conf_1.000	321	0							
Locus_23611_Transcript_1/1_Conf_1.000	275	20	7.03E-24	XP_002647685.1	76	113.62	91	70	Hypothetical protein CBG17873
Locus_23612_Transcript_1/1_Conf_1.000	170	20	1.05E-19	CAB57891.2	91	99.7525	56	51	C. elegans protein Y47D3A.11, partially confirmed by transcript evidence
Locus_23613_Transcript_1/1_Conf_1.000	254	0							
Locus_23614_Transcript_1/1_Conf_1.000	230	0							
Locus_23615_Transcript_1/1_Conf_1.000	128	0							
Locus_23616_Transcript_1/1_Conf_1.000	155	0							
Locus_23617_Transcript_1/1_Conf_1.000	131	2	1.09E-16	NP_001023062.1	100	89.7373	43	43	Ligand-Gated ion Channel family member (lgc-43)
Locus_23618_Transcript_1/1_Conf_1.000	248	12	7.02E-08	EFO19578.1	72	60.4622	72	52	hypothetical protein LOAG_08912
Locus_23619_Transcript_1/1_Conf_1.000	159	0							
Locus_2362_Transcript_1/1_Conf_1.000	618	20	4.68E-29	NP_501149.1	57	131.724	198	113	hypothetical protein D2024.2
Locus_23620_Transcript_1/1_Conf_1.000	197	0							

Locus_23621_Transcript_1/1_Conf_1.000	233	20	1.64E-12	NP_503528.2	82	75.8702	64	53	SeRPin family member (srp-3)
Locus_23622_Transcript_1/1_Conf_1.000	174	0							
Locus_23623_Transcript_1/1_Conf_1.000	289	13	3.44E-18	NP_001041303.1	65	94.7449	93	61	Trehalose 6-Phosphate Synthase family member (tps-1)
Locus_23624_Transcript_1/1_Conf_1.000	131	1	4.75E-04	XP_001121351.1	79	47.7506	39	31	PREDICTED: similar to SET domain and mariner transposase fusion
Locus_23625_Transcript_1/1_Conf_1.000	761	20	3.10E-72	NP_500284.2	81	275.789	213	174	Enhancer of EfL-1 mutant phenotype family member (eel-1)
Locus_23626_Transcript_1/2_Conf_1.000	390	3	3.84E-06	XP_002642918.1	57	54.6842	107	61	Hypothetical protein CBG15194
Locus_23626_Transcript_2/2_Conf_1.000	417	6	7.20E-08	NP_498462.3	52	60.4622	112	59	hypothetical protein C13B9.2
Locus_23627_Transcript_1/1_Conf_1.000	179	0							
Locus_23628_Transcript_1/1_Conf_1.000	454	0							
Locus_23629_Transcript_1/1_Conf_1.000	143	0							
Locus_2363_Transcript_1/1_Conf_1.000	1280	20	2.94E-81	EFO22000.1	60	306.99	392	239	hypothetical protein LOAG_06484
Locus_23630_Transcript_1/1_Conf_1.000	160	0							
Locus_23631_Transcript_1/1_Conf_1.000	295	0							
Locus_23632_Transcript_1/1_Conf_1.000	147	7	2.49E-08	AAN61521.1	82	62.003	47	39	301KDa_2 protein
Locus_23633_Transcript_1/1_Conf_1.000	178	0							
Locus_23634_Transcript_1/2_Conf_1.000	668	20	2.72E-44	XP_002643134.1	67	182.57	205	139	Hypothetical protein CBG15311

Locus_23634_Transcript_2/2_Conf_1.000	668	20	2.72E-44	XP_002643134.1	67	182.57	205	139	Hypothetical protein CBG15311
Locus_23635_Transcript_1/1_Conf_1.000	355	20	2.51E-21	CAB40412.1	73	105.145	93	68	hexokinase
Locus_23636_Transcript_1/1_Conf_1.000	144	3	4.41E-05	NP_501448.1	68	51.2174	45	31	hypothetical protein C28C12.4
Locus_23637_Transcript_1/1_Conf_1.000	147	0							
Locus_23638_Transcript_1/1_Conf_1.000	211	12	5.38E-16	XP_002639999.1	82	87.4261	68	56	Hypothetical protein CBG10829
Locus_23639_Transcript_1/1_Conf_1.000	140	20	1.08E-08	XP_002926200.1	100	63.1586	46	46	PREDICTED: l-lactate dehydrogenase A chain-like
Locus_2364_Transcript_1/1_Conf_1.000	270	0							
Locus_23640_Transcript_1/1_Conf_1.000	333	0							
Locus_23641_Transcript_1/1_Conf_1.000	206	0							
Locus_23642_Transcript_1/1_Conf_1.000	549	20	3.61E-55	NP_491390.2	74	218.009	181	135	hypothetical protein D1007.15

Locus_23643_Transcript_1/1_Conf_1.000	445	20	5.60E-45	XP_002634229.1	77	183.726	146	113	C. briggsae CBR-UNC-26 protein
Locus_23644_Transcript_1/1_Conf_1.000	148	0							
Locus_23645_Transcript_1/1_Conf_1.000	432	2	2.77E-12	XP_002634200.1	53	75.0998	135	72	C. briggsae CBR-NOL-9 protein
Locus_23646_Transcript_1/1_Conf_1.000	134	20	4.13E-16	NP_506269.1	100	87.8113	44	44	EATing: abnormal pharyngeal pumping family member (eat-6)
Locus_23647_Transcript_1/1_Conf_1.000	265	0							
Locus_23648_Transcript_1/1_Conf_1.000	208	20	3.87E-14	NP_504523.1	76	81.2629	67	51	LEThal family member (let-418)
Locus_23649_Transcript_1/1_Conf_1.000	207	0							

Locus_2365_Transcript_1/1_Conf_1.000	1466	20	4.23E-167	CAJ43810.1	74	592.423	483	361	astacin-like metalloprotease
Locus_23650_Transcript_1/1_Conf_1.000	165	0							
Locus_23651_Transcript_1/1_Conf_1.000	335	0							
Locus_23652_Transcript_1/1_Conf_1.000	219	20	3.58E-15	CAB01717.3	84	84.7297	51	43	C. elegans protein C07A4.1, partially confirmed by transcript evidence
Locus_23653_Transcript_1/1_Conf_1.000	274	0							
Locus_23654_Transcript_1/1_Conf_1.000	202	0							
Locus_23655_Transcript_1/1_Conf_1.000	138	13	2.18E-17	XP_002630534.1	97	92.0485	45	44	Hypothetical protein CBG12974
Locus_23656_Transcript_1/1_Conf_1.000	340	20	8.83E-11	XP_001902350.1	56	70.0922	103	58	Myosin XVIIIA
Locus_23657_Transcript_1/1_Conf_1.000	314	20	2.81E-52	XP_002346307.1	99	207.994	104	103	PREDICTED: similar to complement component 4B (Childo blood group), partial
Locus_23658_Transcript_1/1_Conf_1.000	371	1	4.83E-04	XP_002641128.1	46	47.7506	130	60	Hypothetical protein CBG08978
Locus_23659_Transcript_1/1_Conf_1.000	186	20	1.83E-19	NP_501991.2	83	98.9821	61	51	hypothetical protein T25B9.1
Locus_2366_Transcript_1/2_Conf_1.000	2579	20	0	XP_001901889.1	68	675.241	712	487	Kelch motif family protein
Locus_2366_Transcript_2/2_Conf_1.000	2573	20	0	XP_001901889.1	68	676.011	712	488	Kelch motif family protein
Locus_23660_Transcript_1/1_Conf_1.000	219	0							
Locus_23661_Transcript_1/1_Conf_1.000	198	1	4.33E-05	NP_497340.2	55	51.2174	65	36	CaDHerin family member (cdh-12)
Locus_23662_Transcript_1/1_Conf_1.000	147	0							
Locus_23663_Transcript_1/1_Conf_1.000	244	0							
Locus_23664_Transcript_1/1_Conf_1.000	359	0							

Locus_23665_Transcript_1/1_Conf_1.000	390	20	2.25E-06	XP_418071.2	48	55.4546	117	57	PREDICTED: similar to mannose receptor, C type 2
Locus_23666_Transcript_1/1_Conf_1.000	157	0							
Locus_23667_Transcript_1/1_Conf_1.000	240	1	8.09E-04	NP_498765.1	54	46.9802	79	43	hypothetical protein K06H7.3
Locus_23668_Transcript_1/1_Conf_1.000	129	0							
Locus_23669_Transcript_1/1_Conf_1.000	287	0							
Locus_2367_Transcript_1/3_Conf_0.375	1482	20	2.08E-145	Q24702.1	77	520.39	417	325	DVA-1 polyprotein
Locus_2367_Transcript_3/3_Conf_0.375	226	0							
Locus_23670_Transcript_1/1_Conf_1.000	140	1	1.01E-06	NP_502390.2	73	56.6102	46	34	High Incidence of Males (increased X chromosome loss) family member (him-6)
Locus_23671_Transcript_1/1_Conf_1.000	206	20	6.18E-20	BAG63346.1	100	100.523	48	48	unnamed protein product
Locus_23672_Transcript_1/1_Conf_1.000	148	4	8.85E-06	XP_001901521.1	70	53.5286	47	33	Protein kinase domain containing protein
Locus_23673_Transcript_1/1_Conf_1.000	166	20	9.23E-24	BAG63075.1	100	113.235	55	55	unnamed protein product
Locus_23674_Transcript_1/1_Conf_1.000	448	20	1.74E-30	NP_502434.1	69	135.576	147	102	hypothetical protein C36H8.1

Locus_23675_Transcript_1/1_Conf_1.000	287	20	7.68E-26	XP_002633316.1	74	120.168	99	74	C. briggsae CBR-GTL-2 protein
Locus_23676_Transcript_1/1_Conf_1.000	630	20	4.59E-96	NP_492775.2	87	354.369	213	187	LAMinin related. See also lmb- family member (lam-3)
Locus_23677_Transcript_1/1_Conf_1.000	138	1	6.57E-06	XP_002635510.1	75	53.9138	44	33	Hypothetical protein CBG08814
Locus_23678_Transcript_1/1_Conf_1.000	137	0							
Locus_23679_Transcript_1/1_Conf_1.000	278	0							
Locus_2368_Transcript_1/1_Conf_1.000	342	20	5.67E-29	EEZ99728.1	77	130.568	89	69	hypothetical protein TcasGA2_TC002495
Locus_23680_Transcript_1/1_Conf_1.000	351	0							
Locus_23681_Transcript_1/1_Conf_1.000	175	0							
Locus_23682_Transcript_1/1_Conf_1.000	332	20	2.18E-33	NP_001022190.1	85	145.206	95	81	hypothetical protein F53A10.2
Locus_23683_Transcript_1/1_Conf_1.000	141	0							
Locus_23684_Transcript_1/1_Conf_1.000	295	0							
Locus_23685_Transcript_1/1_Conf_1.000	244	20	1.56E-31	XP_002719808.1	100	139.043	64	64	PREDICTED: actin related protein 2/3 complex subunit 3-like
Locus_23686_Transcript_1/1_Conf_1.000	493	20	1.16E-42	XP_002642732.1	75	176.022	132	100	C. briggsae CBR-KIN-18 protein
Locus_23687_Transcript_1/1_Conf_1.000	247	20	1.51E-18	XP_001898877.1	79	95.9005	81	64	oxidoreductase, short chain dehydrogenase/reductase family protein
Locus_23688_Transcript_1/1_Conf_1.000	264	0							
Locus_23689_Transcript_1/1_Conf_1.000	220	0							
Locus_2369_Transcript_1/2_Conf_1.000	1648	5	2.60E-68	XP_002638678.1	56	179.874	274	156	Hypothetical protein CBG11870
Locus_2369_Transcript_2/2_Conf_1.000	1598	5	8.30E-71	NP_492101.1	57	272.707	479	274	hypothetical protein T28F4.4
Locus_23690_Transcript_1/1_Conf_1.000	177	0							
Locus_23691_Transcript_1/1_Conf_1.000	242	0							
Locus_23692_Transcript_1/1_Conf_1.000	203	0							
Locus_23693_Transcript_1/1_Conf_1.000	389	0							

Locus_23694_Transcript_1/1_Conf_1.000	215	20	5.33E-32	XP_002807990.1	100	140.584	68	68	PREDICTED: LOW QUALITY PROTEIN: ATP-dependent DNA helicase 2 subunit 1-like
Locus_23695_Transcript_1/1_Conf_1.000	168	0							
Locus_23696_Transcript_1/1_Conf_1.000	194	2	1.15E-05	XP_002644087.1	74	53.1434	39	29	Hypothetical protein CBG17560
Locus_23697_Transcript_1/1_Conf_1.000	162	20	1.75E-22	BAG59422.1	100	108.997	53	53	unnamed protein product
Locus_23698_Transcript_1/1_Conf_1.000	276	0							
Locus_23699_Transcript_1/1_Conf_1.000	197	20	1.21E-23	NP_506269.1	89	112.849	65	58	EATing: abnormal pharyngeal pumping family member (eat-6)

Locus_237_Transcript_1/1_Conf_1.000	1085	10	6.02E-05	ZP_04598122.1	45	53.1434	174	80	immunoglobulin A1 protease
Locus_2370_Transcript_1/1_Conf_1.000	229	7	3.22E-08	EFO22616.1	72	61.6178	59	43	TK/ABL protein kinase
Locus_23700_Transcript_1/1_Conf_1.000	166	20	7.57E-18	NP_001040798.1	87	93.5893	55	48	PhosphoDiEsterase family member (pde-4)
Locus_23701_Transcript_1/1_Conf_1.000	198	5	2.54E-05	A8WRJ2.2	61	51.9878	71	44	GTPase-activating protein rrc-1
Locus_23702_Transcript_1/1_Conf_1.000	304	20	1.78E-51	XP_002928507.1	100	205.297	101	101	PREDICTED: elongation factor 2-like
Locus_23703_Transcript_1/1_Conf_1.000	291	20	1.89E-08	XP_001641575.1	58	62.3882	70	41	predicted protein
Locus_23704_Transcript_1/1_Conf_1.000	203	20	1.25E-28	NP_497796.1	95	129.413	67	64	hypothetical protein F37A8.5
Locus_23705_Transcript_1/1_Conf_1.000	246	0							
Locus_23706_Transcript_1/1_Conf_1.000	215	9	3.04E-11	XP_002630557.1	71	71.633	71	51	Hypothetical protein CBG13002
Locus_23707_Transcript_1/1_Conf_1.000	137	0							
Locus_23708_Transcript_1/1_Conf_1.000	174	5	1.91E-13	ABU49430.1	82	78.9518	57	47	PRO-2

Locus_23709_Transcript_1/1_Conf_1.000	292	6	7.64E-18	ADJ37280.1	70	93.5893	99	70	Hypothetical protein F26G1.2a
Locus_2371_Transcript_1/1_Conf_1.000	3023	20	0	XP_002636622.1	79	1228.77	1002	794	C. briggsae CBR-SMA-1 protein
Locus_23710_Transcript_1/1_Conf_1.000	138	20	5.36E-16	ACT68329.1	86	87.4261	45	39	cysteine proteinase
Locus_23711_Transcript_1/1_Conf_1.000	174	0							
Locus_23712_Transcript_1/1_Conf_1.000	229	0							
Locus_23713_Transcript_1/1_Conf_1.000	880	20	2.13E-105	NP_001022916.1	82	386.341	292	242	STIM (mammalian STromal Interaction Molecule) homolog family member (stim-1)
Locus_23714_Transcript_1/1_Conf_1.000	184	0							
Locus_23715_Transcript_1/1_Conf_1.000	144	5	2.76E-07	EFO26737.1	85	58.5362	48	41	hypothetical protein LOAG_01753
Locus_23716_Transcript_1/1_Conf_1.000	161	3	4.34E-05	XP_002647546.1	71	51.2174	45	32	C. briggsae CBR-PME-5 protein
Locus_23717_Transcript_1/1_Conf_1.000	357	20	8.04E-28	XP_002637952.1	76	126.716	117	90	Hypothetical protein CBG04769
Locus_23718_Transcript_1/1_Conf_1.000	197	1	8.19E-04	XP_001898052.1	62	46.9802	54	34	Importin-beta N-terminal domain containing protein
Locus_23719_Transcript_1/1_Conf_1.000	142	20	2.84E-09	EFO25060.1	82	65.0846	45	37	UDP-glucoronosyl and UDP-glucosyl transferase
Locus_2372_Transcript_1/1_Conf_1.000	253	0							
Locus_23720_Transcript_1/1_Conf_1.000	308	20	1.27E-33	XP_002634145.1	85	145.976	101	86	Hypothetical protein CBG01706
Locus_23721_Transcript_1/1_Conf_1.000	128	2	7.85E-07	XP_002634289.1	87	56.9954	41	36	Hypothetical protein CBG17623
Locus_23722_Transcript_1/1_Conf_1.000	150	20	1.96E-13	AAZ82849.1	87	78.9518	49	43	Cam kinase kinase protein 1, isoform b

Locus_23723_Transcript_1/1_Conf_1.000	266	13	7.33E-29	XP_002631169.1	92	130.183	88	81	Hypothetical protein CBG02955
Locus_23724_Transcript_1/1_Conf_1.000	211	5	1.33E-06	XP_001946729.1	59	56.225	61	36	PREDICTED: similar to DNA ligase IV
Locus_23725_Transcript_1/1_Conf_1.000	244	0							
Locus_23726_Transcript_1/1_Conf_1.000	208	0							
Locus_23727_Transcript_1/1_Conf_1.000	439	0							
Locus_23728_Transcript_1/1_Conf_1.000	213	6	8.85E-11	NP_001021303.1	76	70.0922	67	51	Nuclear Hormone Receptor family member (nhr-105)
Locus_23729_Transcript_1/1_Conf_1.000	270	20	2.33E-51	XP_540489.1	100	204.912	85	85	PREDICTED: similar to anaphase promoting complex subunit 11 homolog
Locus_2373_Transcript_1/1_Conf_1.000	2283	20	2.07E-177	AAD45354.1	91	627.476	348	319	AF159950_1GSK-3

Locus_23730_Transcript_1/1_Conf_1.000	302	20	1.46E-37	XP_002639432.1	81	159.073	100	81	C. briggsae CBR-TYR-4 protein
Locus_23731_Transcript_1/1_Conf_1.000	151	0							
Locus_23732_Transcript_1/1_Conf_1.000	146	0							
Locus_23733_Transcript_1/1_Conf_1.000	189	20	6.27E-28	XP_002914618.1	100	127.102	62	62	PREDICTED: LOW QUALITY PROTEIN: eukaryotic translation initiation factor 3 subunit L-like
Locus_23734_Transcript_1/1_Conf_1.000	148	0							
Locus_23735_Transcript_1/1_Conf_1.000	159	20	3.92E-06	XP_002735062.1	60	54.6842	48	29	PREDICTED: Alk-like, partial
Locus_23736_Transcript_1/1_Conf_1.000	239	20	2.21E-09	XP_002639475.1	64	65.4698	75	48	Hypothetical protein CBG04073
Locus_23737_Transcript_1/1_Conf_1.000	645	20	2.15E-43	XP_002629985.1	79	179.489	137	109	Hypothetical protein CBG13347
Locus_23738_Transcript_1/1_Conf_1.000	267	0							
Locus_23739_Transcript_1/1_Conf_1.000	200	0							
Locus_2374_Transcript_1/3_Conf_0.571	477	0							
Locus_2374_Transcript_2/3_Conf_0.571	1546	20	1.18E-82		63	311.997	329	208	hypothetical protein Y105C5B.t - Caenorhabditis elegans
Locus_2374_Transcript_3/3_Conf_0.714	1461	20	1.10E-82		63	311.997	329	208	hypothetical protein Y105C5B.t - Caenorhabditis elegans
Locus_23740_Transcript_1/1_Conf_1.000	229	0							
Locus_23741_Transcript_1/1_Conf_1.000	128	0							
Locus_23742_Transcript_1/1_Conf_1.000	214	20	1.15E-34	ACJ65168.1	100	149.443	71	71	Uncoordinated protein 68, isoform b, partially confirmed by transcript evidence
Locus_23743_Transcript_1/1_Conf_1.000	130	0							

Locus_23744_Transcript_1/1_Conf_1.000	254	20	9.37E-40	EFO24654.1	96	166.392	84	81	RNA polymerase Rpb1
Locus_23745_Transcript_1/1_Conf_1.000	138	20	1.51E-18	XP_002924501.1	100	95.9005	45	45	PREDICTED: eukaryotic translation initiation factor 5A-1-like
Locus_23746_Transcript_1/1_Conf_1.000	615	20	4.23E-99	NP_001122963.1	94	364.385	204	193	hypothetical protein F58E6.1
Locus_23747_Transcript_1/1_Conf_1.000	287	5	5.33E-19	NP_001122928.1	74	97.4413	79	59	hypothetical protein F23H12.3
Locus_23748_Transcript_1/1_Conf_1.000	228	20	2.70E-31	XP_002745237.1	100	138.272	68	68	PREDICTED: 40S ribosomal protein S13-like

Locus_23749_Transcript_1/1_Conf_1.000	243	0							
Locus_2375_Transcript_1/1_Conf_1.000	2868	20	0	XP_001899340.1	63	783.867	990	624	c-MYC promoter-binding protein IRLB
Locus_23750_Transcript_1/1_Conf_1.000	132	2	1.13E-05	EFO21756.1	70	53.1434	44	31	PAN domain-containing protein
Locus_23751_Transcript_1/1_Conf_1.000	182	18	2.66E-18	XP_002642115.1	82	95.1301	57	47	C. briggsae CBR-NDG-4 protein
Locus_23752_Transcript_1/1_Conf_1.000	305	20	9.16E-32	XP_002644203.1	81	139.813	101	82	Hypothetical protein CBG17194
Locus_23753_Transcript_1/1_Conf_1.000	135	20	1.05E-11	NP_493662.1	90	73.1738	44	40	hypothetical protein C50D2.2
Locus_23754_Transcript_1/1_Conf_1.000	138	0							
Locus_23755_Transcript_1/1_Conf_1.000	199	20	9.00E-11	NP_503517.1	66	70.0922	65	43	hypothetical protein T10B5.7
Locus_23756_Transcript_1/1_Conf_1.000	161	0							
Locus_23757_Transcript_1/1_Conf_1.000	324	20	1.68E-41	NP_001122686.1	100	172.17	103	103	UNCoordinated family member (unc-103)
Locus_23758_Transcript_1/1_Conf_1.000	309	0							
Locus_23759_Transcript_1/1_Conf_1.000	179	2	5.94E-07	NP_508708.2	71	57.3806	45	32	hypothetical protein ZK470.1
Locus_2376_Transcript_1/3_Conf_0.600	922	20	1.26E-10	NP_495957.1	77	71.633	57	44	vertebrate transcription factor DP-Like family member (dpl-1)
Locus_2376_Transcript_2/3_Conf_0.400	2018	20	9.73E-75	XP_001897870.1	78	286.189	235	184	transcription factor DP1

Locus_2376_Transcript_3/3_Conf_0.400	1648	20	1.83E-137	XP_001897504.1	76	494.197	413	316	SNF2 family N-terminal domain containing protein
Locus_23760_Transcript_1/1_Conf_1.000	129	0							
Locus_23761_Transcript_1/1_Conf_1.000	235	0							
Locus_23762_Transcript_1/1_Conf_1.000	227	0							
Locus_23763_Transcript_1/1_Conf_1.000	205	20	3.07E-11	XP_001381283.1	73	71.633	56	41	PREDICTED: similar to haloacid dehalogenase-like hydrolase domain containing 1A
Locus_23764_Transcript_1/1_Conf_1.000	277	20	8.56E-22	XP_002642776.1	79	106.686	77	61	C. briggsae CBR-WRS-2 protein
Locus_23765_Transcript_1/1_Conf_1.000	152	0							
Locus_23766_Transcript_1/1_Conf_1.000	140	4	3.26E-05	XP_002641225.1	82	51.6026	35	29	C. briggsae CBR-SMA-4 protein
Locus_23767_Transcript_1/1_Conf_1.000	282	0							

Locus_23768_Transcript_1/1_Conf_1.000	249	20	4.82E-25	XP_002633897.1	82	117.472	80	66	Hypothetical protein CBG19959
Locus_23769_Transcript_1/1_Conf_1.000	284	0							
Locus_2377_Transcript_1/1_Conf_1.000	593	20	1.91E-34	XP_002637821.1	70	149.443	179	126	Hypothetical protein CBG04610
Locus_23770_Transcript_1/1_Conf_1.000	455	0							
Locus_23771_Transcript_1/1_Conf_1.000	140	0							
Locus_23772_Transcript_1/1_Conf_1.000	213	20	4.82E-33	A8Y3Q1.2	100	144.05	70	70	Putative 28S ribosomal protein S5
Locus_23773_Transcript_1/1_Conf_1.000	171	0							
Locus_23774_Transcript_1/1_Conf_1.000	321	18	1.75E-22	EFO19513.1	75	108.997	88	66	hypothetical protein LOAG_08980

Locus_23775_Transcript_1/1_Conf_1.000	323	20	5.64E-21	NP_741839.2	58	103.99	104	61	hypothetical protein F48E3.8
Locus_23776_Transcript_1/1_Conf_1.000	292	20	9.04E-11	EFO25944.1	56	70.0922	95	54	hypothetical protein LOAG_02539
Locus_23777_Transcript_1/1_Conf_1.000	140	0							
Locus_23778_Transcript_1/1_Conf_1.000	143	2	2.11E-04	NP_490886.2	70	48.9062	48	34	hypothetical protein Y71G12B.11
Locus_23779_Transcript_1/1_Conf_1.000	149	0							
Locus_2378_Transcript_1/1_Conf_1.000	957	16	5.07E-26	XP_002639372.1	54	122.865	296	160	C. briggsae CBR-RPA-2 protein
Locus_23780_Transcript_1/1_Conf_1.000	207	20	7.27E-21	XP_002640853.1	77	103.605	67	52	Hypothetical protein CBG15741
Locus_23781_Transcript_1/1_Conf_1.000	258	0							
Locus_23782_Transcript_1/1_Conf_1.000	161	20	2.01E-18	XP_002636919.1	88	95.5153	51	45	Hypothetical protein CBG09383
Locus_23783_Transcript_1/1_Conf_1.000	187	10	1.90E-08	XP_001901648.1	72	62.3882	61	44	Conserved hypothetical protein
Locus_23784_Transcript_1/1_Conf_1.000	274	0							
Locus_23785_Transcript_1/1_Conf_1.000	171	0							
Locus_23786_Transcript_1/1_Conf_1.000	198	2	1.21E-15	NP_496015.1	80	86.2705	65	52	hypothetical protein M176.5
Locus_23787_Transcript_1/1_Conf_1.000	277	20	2.25E-30	NP_493616.1	85	135.191	91	78	hypothetical protein F33H2.5

Locus_23788_Transcript_1/1_Conf_1.000	266	20	3.51E-39	CAX51641.1	92	164.466	88	81	C. elegans protein F11E6.8b, partially confirmed by transcript evidence
Locus_23789_Transcript_1/1_Conf_1.000	232	0							
Locus_2379_Transcript_1/1_Conf_1.000	1394	20	1.05E-119	NP_491213.1	69	434.876	400	277	INneXin family member (inx-12)
Locus_23790_Transcript_1/1_Conf_1.000	129	0							
Locus_23791_Transcript_1/1_Conf_1.000	317	18	4.14E-49	NP_001002032.1	100	177.178	78	78	hematological and neurological expressed 1 protein isoform 2
Locus_23792_Transcript_1/1_Conf_1.000	196	0							
Locus_23793_Transcript_1/1_Conf_1.000	534	2	7.85E-33	NP_498567.1	70	143.665	164	116	hypothetical protein K04G7.1
Locus_23794_Transcript_1/1_Conf_1.000	137	0							
Locus_23795_Transcript_1/1_Conf_1.000	265	0							
Locus_23796_Transcript_1/1_Conf_1.000	186	20	3.23E-24	XP_002631133.1	96	114.775	61	59	C. briggsae CBR-CLH-2 protein
Locus_23797_Transcript_1/1_Conf_1.000	620	20	4.35E-51	XP_002637127.1	75	204.912	205	154	Hypothetical protein CBG09629
Locus_23798_Transcript_1/1_Conf_1.000	131	20	6.19E-12	NP_510155.3	93	73.9442	43	40	PIP Kinase family member (ppk-3)
Locus_23799_Transcript_1/1_Conf_1.000	198	0							
Locus_238_Transcript_1/1_Conf_1.000	608	0							
Locus_2380_Transcript_1/1_Conf_1.000	802	0							
Locus_23800_Transcript_1/1_Conf_1.000	278	0							
Locus_23801_Transcript_1/1_Conf_1.000	446	0							

Locus_23802_Transcript_1/1_Conf_1.000	154	20	1.44E-24	XP_002737593.1	100	115.931	51	51	PREDICTED: protein phosphatase 4, catalytic subunit-like
Locus_23803_Transcript_1/1_Conf_1.000	201	0							
Locus_23804_Transcript_1/1_Conf_1.000	234	0							
Locus_23805_Transcript_1/1_Conf_1.000	174	0							
Locus_23806_Transcript_1/1_Conf_1.000	347	0							
Locus_23807_Transcript_1/1_Conf_1.000	338	0							
Locus_23808_Transcript_1/1_Conf_1.000	143	0							
Locus_23809_Transcript_1/1_Conf_1.000	237	0							
Locus_2381_Transcript_1/1_Conf_1.000	2060	20	2.81E-77	Q09275.2	67	294.664	328	223	Putative thiol protease ulp-4
Locus_23810_Transcript_1/1_Conf_1.000	143	0							
Locus_23811_Transcript_1/1_Conf_1.000	279	4	1.11E-21	NP_501142.1	70	106.301	94	66	GLoBin family member (glb-23)
Locus_23812_Transcript_1/1_Conf_1.000	144	0							
Locus_23813_Transcript_1/1_Conf_1.000	288	0							
Locus_23814_Transcript_1/1_Conf_1.000	357	1	5.61E-05	XP_002641620.1	60	50.8322	53	32	C. briggsae CBR-BUB-1 protein
Locus_23815_Transcript_1/1_Conf_1.000	163	0							
Locus_23816_Transcript_1/1_Conf_1.000	134	0							
Locus_23817_Transcript_1/1_Conf_1.000	162	0							
Locus_23818_Transcript_1/1_Conf_1.000	137	0							
Locus_23819_Transcript_1/1_Conf_1.000	218	20	8.80E-19	XP_001891673.1	86	96.6709	65	56	hypothetical protein Bm1_00760
Locus_2382_Transcript_1/3_Conf_0.714	4636	20	0	XP_002630260.1	81	1592.4	1126	913	Hypothetical protein CBG00683
Locus_2382_Transcript_2/3_Conf_0.714	4612	20	0	XP_002630260.1	80	1583.93	1126	911	Hypothetical protein CBG00683
Locus_2382_Transcript_3/3_Conf_0.714	4618	20	0	XP_002630260.1	81	1592.4	1126	913	Hypothetical protein CBG00683
Locus_23820_Transcript_1/1_Conf_1.000	292	20	2.07E-39	XP_002630350.1	87	165.236	97	85	Hypothetical protein CBG04280
Locus_23821_Transcript_1/1_Conf_1.000	136	8	1.57E-07	XP_002639522.1	74	59.3066	43	32	Hypothetical protein CBG04128

Locus_23822_Transcript_1/1_Conf_1.000	206	20	8.07E-20	XP_002917175.1	100	100.138	68	68	PREDICTED: proteasome subunit beta type-2-like
Locus_23823_Transcript_1/1_Conf_1.000	208	2	3.39E-10	XP_002638587.1	78	68.1662	55	43	Hypothetical protein CBG05635
Locus_23824_Transcript_1/1_Conf_1.000	208	0							
Locus_23825_Transcript_1/1_Conf_1.000	205	0							
Locus_23826_Transcript_1/1_Conf_1.000	153	20	3.33E-21	CAR63641.1	100	104.76	50	50	putative 26S proteasome regulatory chain 4
Locus_23827_Transcript_1/1_Conf_1.000	323	0							
Locus_23828_Transcript_1/1_Conf_1.000	384	20	1.55E-47	NP_491030.1	91	192.2	128	117	DNaJ domain (prokaryotic heat shock protein) family member (dnj-30)
Locus_23829_Transcript_1/1_Conf_1.000	151	0							
Locus_2383_Transcript_1/3_Conf_0.667	1039	20	1.44E-101	XP_001902124.1	73	374.015	317	233	sterol carrier protein
Locus_2383_Transcript_2/3_Conf_0.667	918	20	7.71E-85	XP_001902124.1	71	318.161	277	197	sterol carrier protein

Locus_2383_Transcript_3/3_Conf_0.667	1039	20	1.10E-101	XP_001902124.1	73	374.4	317	233	sterol carrier protein
Locus_23830_Transcript_1/1_Conf_1.000	155	0							
Locus_23831_Transcript_1/1_Conf_1.000	234	0							
Locus_23832_Transcript_1/1_Conf_1.000	322	0							
Locus_23833_Transcript_1/1_Conf_1.000	166	0							
Locus_23834_Transcript_1/1_Conf_1.000	178	0							
Locus_23835_Transcript_1/1_Conf_1.000	128	5	4.45E-10	NP_509586.1	92	67.781	42	39	GOlgi Snap Receptor complex member family member (gosr-2.1)
Locus_23836_Transcript_1/1_Conf_1.000	179	4	5.01E-14	XP_002637214.1	75	80.8777	60	45	Hypothetical protein CBG09740
Locus_23837_Transcript_1/1_Conf_1.000	232	20	7.58E-34	NP_741181.1	97	146.747	77	75	hypothetical protein T12A2.15
Locus_23838_Transcript_1/1_Conf_1.000	353	20	7.29E-45	NP_490755.1	89	183.341	120	107	Heavy chain, Unconventional Myosin family member (hum-7)
Locus_23839_Transcript_1/1_Conf_1.000	145	0							
Locus_2384_Transcript_1/2_Conf_1.000	1699	20	8.68E-90	XP_001897752.1	95	335.88	182	173	Ubiquitin-conjugating enzyme E2 1

Locus_2384_Transcript_2/2_Conf_1.000	511	20	1.81E-78	1Z3D	98	295.049	146	144	ProteinCrystal Growth Improvement Leading ToThe 2.5aCrystallographicStructure Of Ubiquitin-Conjugating Enzyme (Ubc-1) From Caenorhabditis Elegans
Locus_23840_Transcript_1/1_Conf_1.000	304	5	2.05E-07	NP_499641.2	53	58.9214	73	39	hypothetical protein Y111B2A.10
Locus_23841_Transcript_1/1_Conf_1.000	179	0							
Locus_23842_Transcript_1/1_Conf_1.000	390	13	7.25E-05	XP_652875.1	54	50.447	116	63	hypothetical protein
Locus_23843_Transcript_1/1_Conf_1.000	251	0							

Locus_23844_Transcript_1/1_Conf_1.000	162	20	2.70E-15	XP_002832350.1	100	85.1149	53	53	PREDICTED: LOW QUALITY PROTEIN: filamin-A-like
Locus_23845_Transcript_1/1_Conf_1.000	363	20	1.22E-52	XP_002633950.1	85	209.149	120	102	C. briggsae CBR-FBL-1 protein
Locus_23846_Transcript_1/1_Conf_1.000	152	20	2.56E-13	XP_002703534.1	100	78.5666	50	50	PREDICTED: signal recognition particle receptor subunit alpha-like
Locus_23847_Transcript_1/1_Conf_1.000	212	0							
Locus_23848_Transcript_1/1_Conf_1.000	386	20	1.60E-68	ADF56012.1	97	261.922	128	125	nicotinic acetylcholine receptor non-alpha subunit LEV-1
Locus_23849_Transcript_1/1_Conf_1.000	191	20	8.78E-14	XP_002642807.1	79	80.1073	63	50	C. briggsae CBR-KEL-3 protein
Locus_2385_Transcript_1/1_Conf_1.000	913	20	9.29E-91	NP_498012.1	83	337.806	263	219	hypothetical protein C35D10.10
Locus_23850_Transcript_1/1_Conf_1.000	130	5	3.18E-08	NP_501599.1	80	61.6178	41	33	Prolyl Carboxy Peptidase like family member (pcp-2)
Locus_23851_Transcript_1/1_Conf_1.000	192	0							
Locus_23852_Transcript_1/1_Conf_1.000	212	0							

Locus_23853_Transcript_1/1_Conf_1.000	141	0							
Locus_23854_Transcript_1/1_Conf_1.000	277	20	1.50E-34	NP_501127.2	89	149.058	92	82	RNA Polymerase, Class III (C) family member (rpc-1)
Locus_23855_Transcript_1/1_Conf_1.000	158	0							
Locus_23856_Transcript_1/1_Conf_1.000	143	0							
Locus_23857_Transcript_1/1_Conf_1.000	171	0							
Locus_23858_Transcript_1/1_Conf_1.000	128	18	4.02E-11	NP_494777.1	90	71.2478	42	38	MoLTing defective family member (mlt-7)
Locus_23859_Transcript_1/1_Conf_1.000	136	4	2.19E-09	NP_500615.1	81	65.4698	44	36	hypothetical protein T22B11.2
Locus_2386_Transcript_1/1_Conf_1.000	1394	20	3.97E-137	NP_492839.4	81	347.436	271	220	Leucine-rich repeats, Ras-like domain, Kinase family member (lrk-1)
Locus_23860_Transcript_1/1_Conf_1.000	133	0							
Locus_23861_Transcript_1/1_Conf_1.000	299	2	1.34E-06	XP_001899503.1	56	56.225	107	60	hypothetical protein Bm1_40230
Locus_23862_Transcript_1/1_Conf_1.000	182	0							
Locus_23863_Transcript_1/1_Conf_1.000	191	0							
Locus_23864_Transcript_1/1_Conf_1.000	356	0							
Locus_23865_Transcript_1/1_Conf_1.000	272	0							
Locus_23866_Transcript_1/1_Conf_1.000	204	0							
Locus_23867_Transcript_1/1_Conf_1.000	274	0							
Locus_23868_Transcript_1/1_Conf_1.000	507	0							
Locus_23869_Transcript_1/1_Conf_1.000	173	0							
Locus_2387_Transcript_1/3_Conf_0.571	792	0							
Locus_2387_Transcript_2/3_Conf_0.714	1343	0							
Locus_2387_Transcript_3/3_Conf_0.714	1382	0							
Locus_23870_Transcript_1/1_Conf_1.000	171	0							
Locus_23871_Transcript_1/1_Conf_1.000	165	20	4.14E-24	AAH46345.1	100	114.39	55	55	AURKAIP1 protein

Locus_23872_Transcript_1/1_Conf_1.000	164	20	1.87E-16	XP_002929659.1	100	88.9669	43	43	PREDICTED: GTP-binding nuclear protein Ran-like, partial
Locus_23873_Transcript_1/1_Conf_1.000	170	0							
Locus_23874_Transcript_1/1_Conf_1.000	389	0							
Locus_23875_Transcript_1/1_Conf_1.000	439	0							
Locus_23876_Transcript_1/1_Conf_1.000	171	0							
Locus_23877_Transcript_1/1_Conf_1.000	277	0							
Locus_23878_Transcript_1/1_Conf_1.000	163	20	2.99E-14	NP_509229.1	90	81.6481	51	46	SUppressor of activated let-60 Ras family member (sur-5)
Locus_23879_Transcript_1/1_Conf_1.000	174	0							
Locus_2388_Transcript_1/1_Conf_1.000	1053	20	9.22E-104	NP_500892.1	85	381.333	287	244	hypothetical protein K02B2.3
Locus_23880_Transcript_1/1_Conf_1.000	320	20	3.41E-42	XP_001891823.1	90	174.481	106	96	2-amino-3-ketobutyrate coenzyme A ligase, mitochondrial precursor
Locus_23881_Transcript_1/1_Conf_1.000	159	0							
Locus_23882_Transcript_1/1_Conf_1.000	233	0							
Locus_23883_Transcript_1/1_Conf_1.000	189	0							
Locus_23884_Transcript_1/1_Conf_1.000	196	0							
Locus_23885_Transcript_1/1_Conf_1.000	176	0							
Locus_23886_Transcript_1/1_Conf_1.000	263	0							
Locus_23887_Transcript_1/1_Conf_1.000	138	0							
Locus_23888_Transcript_1/1_Conf_1.000	128	6	1.48E-05	EFO20090.1	86	52.7582	37	32	hypothetical protein LOAG_08398
Locus_23889_Transcript_1/1_Conf_1.000	160	0							

Locus_2389_Transcript_1/2_Conf_1.000	991	20	7.28E-15	EFO27344.1	43	85.8853	314	138	double-stranded RNA binding domain-containing protein family protein
Locus_2389_Transcript_2/2_Conf_1.000	995	20	7.32E-15	EFO27344.1	43	85.8853	314	138	double-stranded RNA binding domain-containing protein family protein
Locus_23890_Transcript_1/1_Conf_1.000	155	0							
Locus_23891_Transcript_1/1_Conf_1.000	192	0							
Locus_23892_Transcript_1/1_Conf_1.000	135	0							
Locus_23893_Transcript_1/1_Conf_1.000	206	0							
Locus_23894_Transcript_1/1_Conf_1.000	192	20	1.64E-28	XP_002828455.1	100	129.028	63	63	PREDICTED: LOW QUALITY PROTEIN: ornithine decarboxylase antizyme 1-like
Locus_23895_Transcript_1/1_Conf_1.000	551	20	3.09E-14	NP_507654.1	46	82.0333	169	79	SCP-Like extracellular protein family member (scl-19)
Locus_23896_Transcript_1/1_Conf_1.000	187	0							
Locus_23897_Transcript_1/1_Conf_1.000	392	0							
Locus_23898_Transcript_1/1_Conf_1.000	129	0							

Locus_23899_Transcript_1/1_Conf_1.000	284	20	3.02E-30	CAF97585.1	80	134.806	88	71	unnamed protein product
Locus_239_Transcript_1/1_Conf_1.000	180	0							
Locus_2390_Transcript_1/1_Conf_1.000	498	20	6.90E-64	CAG25499.1	88	246.514	144	128	heat shock protein 20
Locus_23900_Transcript_1/1_Conf_1.000	133	1	3.63E-04	XP_002642828.1	64	48.1358	42	27	Hypothetical protein CBG21226
Locus_23901_Transcript_1/1_Conf_1.000	205	20	5.96E-31	BAG64784.1	100	137.117	68	68	unnamed protein product
Locus_23902_Transcript_1/1_Conf_1.000	208	20	4.11E-32	XP_002920795.1	100	140.969	59	59	PREDICTED: LOW QUALITY PROTEIN: SPARC-like
Locus_23903_Transcript_1/1_Conf_1.000	227	0							
Locus_23904_Transcript_1/1_Conf_1.000	171	20	1.67E-25	XP_002810873.1	100	119.013	57	57	PREDICTED: nardilysin-like isoform 2
Locus_23905_Transcript_1/1_Conf_1.000	742	0							
Locus_23906_Transcript_1/1_Conf_1.000	370	20	3.33E-21	NP_503012.2	67	104.76	102	69	hypothetical protein Y116A8C.13
Locus_23907_Transcript_1/1_Conf_1.000	356	0							
Locus_23908_Transcript_1/1_Conf_1.000	164	0							
Locus_23909_Transcript_1/1_Conf_1.000	423	7	2.12E-60	CAA92681.2	89	234.958	125	112	C. elegans protein K08E4.3, confirmed by transcript evidence

Locus_2391_Transcript_1/1_Conf_1.000	730	20	2.62E-41	NP_492270.1	58	172.94	235	137	hypothetical protein F16D3.4
Locus_23910_Transcript_1/1_Conf_1.000	212	0							
Locus_23911_Transcript_1/1_Conf_1.000	395	20	1.17E-50	NP_497814.3	84	202.601	131	111	UNCoordinated family member (unc-79)
Locus_23912_Transcript_1/1_Conf_1.000	470	10	5.59E-37	NP_499042.2	71	157.147	156	112	hypothetical protein T23G5.6
Locus_23913_Transcript_1/1_Conf_1.000	345	2	6.28E-12	NP_494997.1	67	73.9442	76	51	VH1 dual-specificity phosphatase family member (vhp-1)
Locus_23914_Transcript_1/1_Conf_1.000	317	2	7.95E-15	NP_001024845.1	69	83.5741	89	62	hypothetical protein R08B4.5
Locus_23915_Transcript_1/1_Conf_1.000	174	6	5.56E-13	XP_523970.2	90	77.411	42	38	PREDICTED: similar to Finkel-Biskis-Reilly murine sarcoma virus (FBR-MuSV) ubiquitously expressed (fox derived) isoform 2
Locus_23916_Transcript_1/1_Conf_1.000	183	0							
Locus_23917_Transcript_1/1_Conf_1.000	226	3	6.47E-25	NP_740934.2	93	117.087	75	70	UNCoordinated family member (unc-95)
Locus_23918_Transcript_1/1_Conf_1.000	459	20	3.84E-06	XP_002130348.1	48	54.6842	128	62	PREDICTED: similar to protease, serine, 7 (enterokinase)
Locus_23919_Transcript_1/1_Conf_1.000	304	11	7.55E-10		54	67.0106	100	54	hypothetical protein T28B8.4
Locus_2392_Transcript_1/1_Conf_1.000	2200	20	2.86E-160	XP_002641169.1	66	570.466	528	353	C. briggsae CBR-FRM-2 protein
Locus_23920_Transcript_1/1_Conf_1.000	147	0							
Locus_23921_Transcript_1/1_Conf_1.000	522	20	9.64E-41	NP_502553.2	79	169.859	162	128	Cleavage and Polyadenylation Specificity Factor family member (cpsf-3)
Locus_23922_Transcript_1/1_Conf_1.000	268	0							
Locus_23923_Transcript_1/1_Conf_1.000	389	0							
Locus_23924_Transcript_1/1_Conf_1.000	235	0							
Locus_23925_Transcript_1/1_Conf_1.000	169	0							
Locus_23926_Transcript_1/1_Conf_1.000	290	20	1.82E-19	NP_493592.1	68	98.9821	104	71	hypothetical protein F32A7.4
Locus_23927_Transcript_1/1_Conf_1.000	418	0							
Locus_23928_Transcript_1/1_Conf_1.000	254	0							

Locus_23929_Transcript_1/1_Conf_1.000	239	20	2.35E-27	AAM11895.1	86	125.176	79	68	high-affinity dicarboxylate transporter INDY3
Locus_2393_Transcript_1/1_Conf_1.000	451	20	1.66E-57	NP_497721.1	87	225.328	138	121	Ribosomal Protein, Large subunit family member (rpl-16)
Locus_23930_Transcript_1/1_Conf_1.000	376	0							
Locus_23931_Transcript_1/1_Conf_1.000	132	0							
Locus_23932_Transcript_1/1_Conf_1.000	226	0							
Locus_23933_Transcript_1/1_Conf_1.000	226	0							
Locus_23934_Transcript_1/1_Conf_1.000	242	0							
Locus_23935_Transcript_1/1_Conf_1.000	152	0							
Locus_23936_Transcript_1/1_Conf_1.000	278	20	3.25E-29	EFO19658.1	82	131.339	92	76	hypothetical protein LOAG_08833
Locus_23937_Transcript_1/1_Conf_1.000	187	0							
Locus_23938_Transcript_1/1_Conf_1.000	320	20	2.14E-36	XP_002643176.1	87	155.221	99	87	Hypothetical protein CBG24142
Locus_23939_Transcript_1/1_Conf_1.000	367	20	4.08E-24	XP_002646775.1	66	114.39	116	77	Hypothetical protein CBG18419
Locus_2394_Transcript_1/2_Conf_1.000	1214	0							
Locus_2394_Transcript_2/2_Conf_1.000	621	0							
Locus_23940_Transcript_1/1_Conf_1.000	347	20	8.43E-33	XP_002637479.1	78	143.28	112	88	Hypothetical protein CBG19196

Locus_23941_Transcript_1/1_Conf_1.000	340	19	1.04E-19	NP_497235.2	94	99.7525	57	54	G-protein-coupled Receptor Kinase family member (grk-2)
Locus_23942_Transcript_1/1_Conf_1.000	154	0							
Locus_23943_Transcript_1/1_Conf_1.000	143	0							
Locus_23944_Transcript_1/1_Conf_1.000	133	0							
Locus_23945_Transcript_1/1_Conf_1.000	332	0							
Locus_23946_Transcript_1/1_Conf_1.000	207	0							
Locus_23947_Transcript_1/1_Conf_1.000	350	0							
Locus_23948_Transcript_1/1_Conf_1.000	155	0							
Locus_23949_Transcript_1/1_Conf_1.000	240	0							
Locus_2395_Transcript_1/1_Conf_1.000	808	20	3.70E-122	NP_492775.2	80	441.81	265	213	LAMinin related. See also lmb- family member (lam-3)
Locus_23950_Transcript_1/1_Conf_1.000	136	1	4.72E-04	NP_491418.1	76	47.7506	39	30	hypothetical protein B0041.5
Locus_23951_Transcript_1/1_Conf_1.000	154	0							
Locus_23952_Transcript_1/1_Conf_1.000	426	7	5.74E-58	Q20548.2	89	226.868	141	126	Probable RING finger protein 207 homolog
Locus_23953_Transcript_1/1_Conf_1.000	226	0							
Locus_23954_Transcript_1/1_Conf_1.000	135	0							
Locus_23955_Transcript_1/1_Conf_1.000	217	0							
Locus_23956_Transcript_1/1_Conf_1.000	377	20	1.18E-18	EFO24235.1	60	96.2857	118	71	hypothetical protein LOAG_04250
Locus_23957_Transcript_1/1_Conf_1.000	189	0							
Locus_23958_Transcript_1/1_Conf_1.000	178	4	8.85E-11	NP_500790.1	82	70.0922	57	47	hypothetical protein F41H10.4
Locus_23959_Transcript_1/1_Conf_1.000	197	0							
Locus_2396_Transcript_1/1_Conf_1.000	1005	1	7.75E-04	NP_504247.2	38	49.2914	226	87	hypothetical protein F54D11.2

Locus_23960_Transcript_1/1_Conf_1.000	1207	20	1.97E-156	NP_497007.1	81	556.599	403	327	hypothetical protein K10H10.1
Locus_23961_Transcript_1/1_Conf_1.000	239	20	3.40E-10	EFO26171.1	68	68.1662	60	41	tyrosyl-DNA phosphodiesterase
Locus_23962_Transcript_1/1_Conf_1.000	455	20	2.32E-43	NP_502044.1	76	178.333	150	115	AQuaPorin or aquaglyceroporin related family member (aqp-3)
Locus_23963_Transcript_1/1_Conf_1.000	147	3	4.38E-13	XP_001897744.1	91	77.7962	48	44	ABC transporter family protein
Locus_23964_Transcript_1/1_Conf_1.000	153	20	1.15E-13	EAX10112.1	82	79.7221	47	39	hCG2018900
Locus_23965_Transcript_1/1_Conf_1.000	208	20	1.68E-17	XP_002642043.1	73	92.4337	72	53	C. briggsae CBR-PAL-1 protein
Locus_23966_Transcript_1/1_Conf_1.000	201	0							
Locus_23967_Transcript_1/1_Conf_1.000	222	0							
Locus_23968_Transcript_1/1_Conf_1.000	313	0							
Locus_23969_Transcript_1/1_Conf_1.000	168	0							

Locus_2397_Transcript_1/1_Conf_1.000	2178	20	4.59E-102	EFO15014.1	73	377.096	314	230	CAMK/PIM protein kinase
Locus_23970_Transcript_1/1_Conf_1.000	311	4	1.23E-28	NP_500851.2	79	129.413	101	80	hypothetical protein H35B03.1
Locus_23971_Transcript_1/1_Conf_1.000	143	0							
Locus_23972_Transcript_1/1_Conf_1.000	192	1	9.70E-05	XP_002633562.1	63	50.0618	65	41	Hypothetical protein CBG05433
Locus_23973_Transcript_1/1_Conf_1.000	324	0							
Locus_23974_Transcript_1/1_Conf_1.000	368	20	2.61E-63	XP_002926784.1	100	244.588	122	122	PREDICTED: heat shock protein HSP 90-alpha-like
Locus_23975_Transcript_1/1_Conf_1.000	163	20	3.53E-15	XP_002631757.1	86	84.7297	53	46	Hypothetical protein CBG20965
Locus_23976_Transcript_1/1_Conf_1.000	390	7	5.54E-13	XP_001901143.1	70	77.411	93	66	Protein kinase domain containing protein

Locus_23977_Transcript_1/1_Conf_1.000	196	20	2.46E-08	XP_001900312.1	80	62.003	66	53	Krr1 family protein
Locus_23978_Transcript_1/1_Conf_1.000	593	12	1.08E-45	XP_002644583.1	74	186.808	150	112	C. briggsae CBR-GLIT-1 protein
Locus_23979_Transcript_1/1_Conf_1.000	276	20	5.54E-29	NP_509689.1	84	130.568	95	80	hypothetical protein ZC373.4
Locus_2398_Transcript_1/1_Conf_1.000	468	5	3.91E-06	AAR03713.1	49	54.6842	103	51	secreted protein 4 precursor
Locus_23980_Transcript_1/1_Conf_1.000	261	20	2.46E-16	XP_002634885.1	75	88.5817	76	57	Hypothetical protein CBG10553
Locus_23981_Transcript_1/1_Conf_1.000	194	9	7.16E-16	NP_503758.4	77	87.0409	63	49	KETtiN (Drosophila actin-binding) homolog family member (ketn-1)
Locus_23982_Transcript_1/1_Conf_1.000	198	20	3.91E-06	YP_779412.1	60	54.6842	64	39	malonyl-CoA synthase
Locus_23983_Transcript_1/1_Conf_1.000	269	20	9.25E-24	EFO16116.1	79	113.235	88	70	hypothetical protein LOAG_12392
Locus_23984_Transcript_1/1_Conf_1.000	138	0							
Locus_23985_Transcript_1/1_Conf_1.000	755	20	3.51E-36	XP_002636125.1	52	155.992	257	136	Hypothetical protein CBG01375
Locus_23986_Transcript_1/1_Conf_1.000	276	0							
Locus_23987_Transcript_1/1_Conf_1.000	304	19	6.82E-11	NP_502296.1	58	70.4774	92	54	hypothetical protein M18.3
Locus_23988_Transcript_1/1_Conf_1.000	183	0							
Locus_23989_Transcript_1/1_Conf_1.000	229	0							
Locus_2399_Transcript_1/1_Conf_1.000	2816	20	0	XP_002644820.1	87	1172.15	864	757	C. briggsae CBR-NNT-1 protein
Locus_23990_Transcript_1/1_Conf_1.000	186	0							
Locus_23991_Transcript_1/1_Conf_1.000	192	0							
Locus_23992_Transcript_1/1_Conf_1.000	148	20	3.47E-10	YP_466652.1	83	68.1662	43	36	L-threonine ammonia-lyase

Locus_23993_Transcript_1/1_Conf_1.000	132	3	9.54E-05		67	50.0618	43	29	ypothetical protein Y39B6B.h
Locus_23994_Transcript_1/1_Conf_1.000	298	4	1.06E-27	XP_002638375.1	80	126.331	97	78	Hypothetical protein CBG18580
Locus_23995_Transcript_1/1_Conf_1.000	145	1	4.87E-04	NP_494953.3	72	47.7506	40	29	Nematode AStacin protease family member (nas-29)
Locus_23996_Transcript_1/1_Conf_1.000	497	0							
Locus_23997_Transcript_1/1_Conf_1.000	417	0							
Locus_23998_Transcript_1/1_Conf_1.000	219	0							
Locus_23999_Transcript_1/1_Conf_1.000	186	0							
Locus_24_Transcript_1/1_Conf_1.000	315	0							
Locus_240_Transcript_1/1_Conf_1.000	2181	20	0	NP_496273.1	96	1174.07	702	674	Cell Division Cycle related family member (cdc-48.1)
Locus_2400_Transcript_1/1_Conf_1.000	506	20	1.57E-42	XP_002631189.1	90	175.637	103	93	C. briggsae CBR-SDHD-1 protein
Locus_24000_Transcript_1/1_Conf_1.000	172	0							
Locus_24001_Transcript_1/1_Conf_1.000	131	0							
Locus_24002_Transcript_1/1_Conf_1.000	161	0							
Locus_24003_Transcript_1/1_Conf_1.000	138	0							
Locus_24004_Transcript_1/1_Conf_1.000	188	0							
Locus_24005_Transcript_1/1_Conf_1.000	164	0							
Locus_24006_Transcript_1/1_Conf_1.000	187	20	1.19E-18	Q03566.2	88	96.2857	51	45	Probable G-protein coupled receptor tkr-1
Locus_24007_Transcript_1/1_Conf_1.000	317	20	9.68E-29	XP_002630524.1	69	129.798	105	73	Hypothetical protein CBG12962

Locus_24008_Transcript_1/1_Conf_1.000	183	1	6.35E-04	XP_002641413.1	65	47.3654	60	39	Hypothetical protein CBG13279
Locus_24009_Transcript_1/1_Conf_1.000	170	0							
Locus_2401_Transcript_1/1_Conf_1.000	620	5	7.64E-64	NP_001123059.1	76	247.284	204	156	hypothetical protein Y61A9LA.11
Locus_24010_Transcript_1/1_Conf_1.000	330	0							
Locus_24011_Transcript_1/1_Conf_1.000	156	6	3.01E-06	EFO20752.1	73	55.0694	41	30	AMOP domain-containing protein
Locus_24012_Transcript_1/1_Conf_1.000	684	20	7.13E-51	XP_001897894.1	66	204.527	247	165	ancient conserved domain protein 4
Locus_24013_Transcript_1/1_Conf_1.000	152	0							
Locus_24014_Transcript_1/1_Conf_1.000	211	20	1.16E-18	XP_002640591.1	77	96.2857	68	53	Hypothetical protein CBG15874
Locus_24015_Transcript_1/1_Conf_1.000	242	20	5.19E-35	EFO28104.1	97	150.599	76	74	hypothetical protein LOAG_00390
Locus_24016_Transcript_1/1_Conf_1.000	161	0							
Locus_24017_Transcript_1/1_Conf_1.000	411	2	1.79E-11	NP_497340.2	59	72.4034	139	83	CaDHerin family member (cdh-12)
Locus_24018_Transcript_1/1_Conf_1.000	265	0							
Locus_24019_Transcript_1/1_Conf_1.000	301	0							
Locus_2402_Transcript_1/1_Conf_1.000	281	8	3.26E-05	NP_498849.3	57	51.6026	84	48	hypothetical protein R05D3.1
Locus_24020_Transcript_1/1_Conf_1.000	204	0							
Locus_24021_Transcript_1/1_Conf_1.000	284	0							

Locus_24022_Transcript_1/1_Conf_1.000	214	20	1.36E-27	NP_503175.1	92	125.946	71	66	ABC Transporter family member (abt-4)
Locus_24023_Transcript_1/1_Conf_1.000	193	20	1.65E-12	NP_500785.1	70	75.8702	64	45	hypothetical protein Y4C6B.6
Locus_24024_Transcript_1/1_Conf_1.000	210	20	2.25E-30	NP_004532.1	100	135.191	62	62	NADH dehydrogenase ubiquinone 1 alpha subcomplex subunit 1
Locus_24025_Transcript_1/1_Conf_1.000	250	0							
Locus_24026_Transcript_1/1_Conf_1.000	235	0							
Locus_24027_Transcript_1/1_Conf_1.000	225	20	5.12E-30	BAI49947.1	85	134.035	74	63	GLI family zinc finger protein
Locus_24028_Transcript_1/1_Conf_1.000	344	0							
Locus_24029_Transcript_1/1_Conf_1.000	320	5	5.29E-27	NP_001024085.2	95	124.02	71	68	hypothetical protein R186.8
Locus_2403_Transcript_1/1_Conf_1.000	1475	20	1.24E-166	NP_502560.1	82	590.882	376	311	FATty acid desaturase family member (fat-2)
Locus_24030_Transcript_1/1_Conf_1.000	133	0							
Locus_24031_Transcript_1/1_Conf_1.000	226	2	6.49E-17	XP_002634566.1	85	90.5077	64	55	Hypothetical protein CBG08372
Locus_24032_Transcript_1/1_Conf_1.000	228	0							

Locus_24033_Transcript_1/1_Conf_1.000	135	2	1.73E-06	NP_504508.1	76	55.8398	47	36	hypothetical protein K09H11.1
Locus_24034_Transcript_1/1_Conf_1.000	136	0							
Locus_24035_Transcript_1/1_Conf_1.000	165	0							
Locus_24036_Transcript_1/1_Conf_1.000	183	2	8.82E-22	NP_001122963.1	88	106.686	60	53	hypothetical protein F58E6.1
Locus_24037_Transcript_1/1_Conf_1.000	232	20	1.48E-37	BAG62774.1	100	159.073	77	77	unnamed protein product
Locus_24038_Transcript_1/1_Conf_1.000	162	9	1.34E-06	AAO63576.1	66	56.225	53	35	secreted protein 4 precursor
Locus_24039_Transcript_1/1_Conf_1.000	275	2	1.29E-09	NP_491915.2	81	66.2402	43	35	hypothetical protein ZC581.9
Locus_2404_Transcript_1/1_Conf_1.000	2087	20	1.81E-39	XP_002640179.1	52	169.088	411	217	Hypothetical protein CBG12682
Locus_24040_Transcript_1/1_Conf_1.000	141	0							
Locus_24041_Transcript_1/1_Conf_1.000	139	0							
Locus_24042_Transcript_1/1_Conf_1.000	376	20	9.93E-26	NP_871661.1	75	119.783	106	80	hypothetical protein F54C8.7
Locus_24043_Transcript_1/1_Conf_1.000	237	0							
Locus_24044_Transcript_1/1_Conf_1.000	252	0							
Locus_24045_Transcript_1/1_Conf_1.000	546	20	1.90E-08	ADI61829.1	76	62.7734	56	43	endonuclease-reverse transcriptase
Locus_24046_Transcript_1/1_Conf_1.000	183	0							
Locus_24047_Transcript_1/1_Conf_1.000	301	0							
Locus_24048_Transcript_1/1_Conf_1.000	169	20	2.87E-09	XP_002648446.1	67	65.0846	53	36	Hypothetical protein CBG24721
Locus_24049_Transcript_1/1_Conf_1.000	241	2	2.35E-11	NP_494213.2	84	72.0182	51	43	hypothetical protein Y59C2A.1
Locus_2405_Transcript_1/4_Conf_0.600	721	20	2.48E-20	AAO63578.1	46	103.219	204	94	secreted protein 6 precursor

Locus_2405_Transcript_2/4_Conf_0.500	416	20	2.48E-13	NP_502498.1	52	78.5666	99	52	SCP-Like extracellular protein family member (scl-10)
Locus_2405_Transcript_3/4_Conf_0.600	766	20	4.77E-20	AAO63578.1	45	102.449	204	92	secreted protein 6 precursor
Locus_2405_Transcript_4/4_Conf_0.700	766	20	8.14E-20	AAO63578.1	45	101.679	204	93	secreted protein 6 precursor
Locus_24050_Transcript_1/1_Conf_1.000	275	1	8.08E-04	ZP_05532439.1	50	46.9802	85	43	hypothetical protein SvirD4_18528
Locus_24051_Transcript_1/1_Conf_1.000	145	5	1.91E-08	CAA10033.1	75	62.3882	48	36	DYS-1 protein
Locus_24052_Transcript_1/1_Conf_1.000	176	2	5.57E-05	NP_508178.2	63	50.8322	58	37	TBC (Tre-2/Bub2/Cdc16) domain family member (tbc-11)
Locus_24053_Transcript_1/1_Conf_1.000	145	0							
Locus_24054_Transcript_1/1_Conf_1.000	320	20	3.79E-25	NP_001022327.2	67	117.857	105	71	CalEXcitin family member (cex-2)
Locus_24055_Transcript_1/1_Conf_1.000	254	20	3.83E-17	XP_002647073.1	66	91.2781	84	56	Hypothetical protein CBG03599
Locus_24056_Transcript_1/1_Conf_1.000	142	0							
Locus_24057_Transcript_1/1_Conf_1.000	155	0							
Locus_24058_Transcript_1/1_Conf_1.000	184	4	1.19E-10	AAK31527.3	74	69.707	58	43	Neuronal symmetry protein 1, partially confirmed by transcript evidence
Locus_24059_Transcript_1/1_Conf_1.000	226	0							
Locus_2406_Transcript_1/3_Conf_0.714	1282	4	3.45E-05	AAH48067.1	44	54.299	201	89	Nolc1l protein
Locus_2406_Transcript_2/3_Conf_0.714	1306	4	1.59E-05	YP_708893.1	43	55.4546	216	93	hypothetical protein RHA1_ro11088
Locus_2406_Transcript_3/3_Conf_0.714	1306	4	4.63E-05	YP_708893.1	42	53.9138	216	92	hypothetical protein RHA1_ro11088
Locus_24060_Transcript_1/1_Conf_1.000	160	0							
Locus_24061_Transcript_1/1_Conf_1.000	151	0							
Locus_24062_Transcript_1/1_Conf_1.000	285	0							
Locus_24063_Transcript_1/1_Conf_1.000	263	0							
Locus_24064_Transcript_1/1_Conf_1.000	236	20	4.31E-13	XP_002640318.1	64	77.7962	76	49	C. briggsae CBR-CLN-3.2 protein
Locus_24065_Transcript_1/1_Conf_1.000	190	0							
Locus_24066_Transcript_1/1_Conf_1.000	131	0							
Locus_24067_Transcript_1/1_Conf_1.000	187	0							
Locus_24068_Transcript_1/1_Conf_1.000	176	0							
Locus_24069_Transcript_1/1_Conf_1.000	259	0							
Locus_2407_Transcript_1/1_Conf_1.000	160	0							
Locus_24070_Transcript_1/1_Conf_1.000	285	20	3.45E-18	NP_491128.4	72	94.7449	84	61	Conserved Oligomeric Golgi (COG) Component family member (cogc-1)
Locus_24071_Transcript_1/1_Conf_1.000	155	5	8.79E-06	CAD44090.2	80	53.5286	46	37	C. elegans protein C27H6.4b, confirmed by transcript evidence
Locus_24072_Transcript_1/1_Conf_1.000	402	0							

Locus_24073_Transcript_1/1_Conf_1.000	139	0							
Locus_24074_Transcript_1/1_Conf_1.000	129	0							
Locus_24075_Transcript_1/1_Conf_1.000	308	20	9.47E-21	NP_491496.1	68	103.219	103	71	Downstream Of Mes (in same operon) family member (dom-3)
Locus_24076_Transcript_1/1_Conf_1.000	132	0							
Locus_24077_Transcript_1/1_Conf_1.000	144	20	2.41E-11	XP_002639452.1	92	72.0182	42	39	Hypothetical protein CBG04047
Locus_24078_Transcript_1/1_Conf_1.000	172	0							
Locus_24079_Transcript_1/1_Conf_1.000	201	0							
Locus_2408_Transcript_1/1_Conf_1.000	160	0							
Locus_24080_Transcript_1/1_Conf_1.000	335	3	1.62E-12	EFO17724.1	55	75.8702	109	61	hypothetical protein LOAG_10773
Locus_24081_Transcript_1/1_Conf_1.000	234	0							
Locus_24082_Transcript_1/1_Conf_1.000	147	3	1.12E-08	XP_002642237.1	82	63.1586	40	33	Hypothetical protein CBG18219
Locus_24083_Transcript_1/1_Conf_1.000	221	20	7.47E-13	ACI49160.1	67	77.0258	74	50	hypothetical protein Cbre_JD21.007
Locus_24084_Transcript_1/1_Conf_1.000	375	20	1.34E-30	NP_509266.1	73	135.961	125	92	hypothetical protein F48E3.2
Locus_24085_Transcript_1/1_Conf_1.000	473	13	3.71E-57	NP_001040915.1	84	224.172	144	121	hypothetical protein C27D8.3
Locus_24086_Transcript_1/1_Conf_1.000	194	20	2.63E-18	EFO22374.1	83	95.1301	62	52	hypothetical protein LOAG_06111
Locus_24087_Transcript_1/1_Conf_1.000	280	0							
Locus_24088_Transcript_1/1_Conf_1.000	179	9	1.01E-22	NP_492127.1	89	109.768	59	53	Low-density lipoprotein Receptor Related family member (lrp-1)
Locus_24089_Transcript_1/1_Conf_1.000	184	0							
Locus_2409_Transcript_1/1_Conf_1.000	1275	20	7.04E-168	NP_492451.2	93	594.734	333	313	hypothetical protein ZK858.6
Locus_24090_Transcript_1/1_Conf_1.000	411	0							
Locus_24091_Transcript_1/1_Conf_1.000	369	5	1.71E-17	AAA97973.2	79	92.4337	78	62	Hypothetical protein F21C10.7
Locus_24092_Transcript_1/1_Conf_1.000	248	0							
Locus_24093_Transcript_1/1_Conf_1.000	372	0							
Locus_24094_Transcript_1/1_Conf_1.000	152	0							
Locus_24095_Transcript_1/1_Conf_1.000	141	0							
Locus_24096_Transcript_1/1_Conf_1.000	139	20	1.51E-10	XP_002596899.1	78	69.3218	46	36	hypothetical protein BRAFLDRAFT_76408
Locus_24097_Transcript_1/1_Conf_1.000	190	0							

Locus_24098_Transcript_1/1_Conf_1.000	233	2	4.32E-13	NP_493385.2	85	77.7962	56	48	hypothetical protein Y87G2A.13
Locus_24099_Transcript_1/1_Conf_1.000	309	20	5.72E-18	XP_002636521.1	67	93.9745	96	65	Hypothetical protein CBG23202
Locus_241_Transcript_1/2_Conf_1.000	1101	20	4.03E-97	NP_498934.1	74	359.377	303	226	abnormal cell MIGration family member (mig-22)
Locus_241_Transcript_2/2_Conf_1.000	1053	20	2.77E-100	NP_498934.1	78	369.777	287	226	abnormal cell MIGration family member (mig-22)
Locus_2410_Transcript_1/1_Conf_1.000	311	0							
Locus_24100_Transcript_1/1_Conf_1.000	136	0							
Locus_24101_Transcript_1/1_Conf_1.000	136	20	1.97E-18	BAD92458.1	100	95.5153	44	44	hypothetical protein FLJ41407 variant
Locus_24102_Transcript_1/1_Conf_1.000	150	0							
Locus_24103_Transcript_1/1_Conf_1.000	384	0							
Locus_24104_Transcript_1/1_Conf_1.000	201	20	4.03E-11	NP_001122921.1	66	71.2478	68	45	hypothetical protein F17C11.12
Locus_24105_Transcript_1/1_Conf_1.000	242	0							
Locus_24106_Transcript_1/1_Conf_1.000	158	0							

Locus_24107_Transcript_1/1_Conf_1.000	191	20	3.00E-30	XP_002916731.1	100	134.806	63	63	PREDICTED: u4/U6.U5 tri-snRNP-associated protein 1-like
Locus_24108_Transcript_1/1_Conf_1.000	134	20	6.61E-06	DAA27257.1	95	53.9138	44	42	SPARC
Locus_24109_Transcript_1/1_Conf_1.000	218	20	2.16E-25	NP_502044.1	86	118.627	72	62	AQuaPorin or aquaglyceroporin related family member (aqp-3)
Locus_2411_Transcript_1/1_Conf_1.000	2614	20	2.92E-106	XP_001902470.1	55	391.349	585	326	SET domain containing protein
Locus_24110_Transcript_1/1_Conf_1.000	254	0							
Locus_24111_Transcript_1/1_Conf_1.000	137	0							
Locus_24112_Transcript_1/1_Conf_1.000	175	20	1.84E-24	NP_497696.2	96	115.546	58	56	Pim (mammalian oncogene) Related Kinase family member (prk-2)
Locus_24113_Transcript_1/1_Conf_1.000	403								
Locus_24114_Transcript_1/1_Conf_1.000	263	0							
Locus_24115_Transcript_1/1_Conf_1.000	269	0							
Locus_24116_Transcript_1/1_Conf_1.000	135	0							
Locus_24117_Transcript_1/1_Conf_1.000	139	0							

Locus_24118_Transcript_1/1_Conf_1.000	251	20	2.55E-34	NP_001001392.1	98	148.288	70	69	CD44 antigen isoform 5 precursor
Locus_24119_Transcript_1/1_Conf_1.000	140	1	6.15E-04	XP_002640263.1	63	47.3654	46	29	Hypothetical protein CBG12788
Locus_2412_Transcript_1/1_Conf_1.000	1672	20	2.59E-163	AAF63404.1	99	580.096	285	283	AF105967_1galectin
Locus_24120_Transcript_1/1_Conf_1.000	206	0							
Locus_24121_Transcript_1/1_Conf_1.000	272	0							
Locus_24122_Transcript_1/1_Conf_1.000	388	2	1.98E-10	XP_002634119.1	56	68.9366	116	65	Hypothetical protein CBG01673
Locus_24123_Transcript_1/1_Conf_1.000	347	20	1.10E-24	EFO26647.1	73	116.316	114	84	FYVE zinc finger family protein
Locus_24124_Transcript_1/1_Conf_1.000	332	20	2.35E-46	EAX00616.1	100	110.538	46	46	chromosome 2 open reading frame 28, isoform CRA_b
Locus_24125_Transcript_1/1_Conf_1.000	364	20	1.08E-32	XP_001901809.1	70	142.895	118	83	Serine/threonine protein phosphatase PP1-1

Locus_24126_Transcript_1/1_Conf_1.000	296	0							
Locus_24127_Transcript_1/1_Conf_1.000	294	20	2.37E-19	NP_741638.1	70	98.5969	92	65	CYtochrome P450 family member (cyp-35C1)
Locus_24128_Transcript_1/1_Conf_1.000	492	20	2.41E-48	XP_002642154.1	76	194.897	161	123	Hypothetical protein CBG18111
Locus_24129_Transcript_1/1_Conf_1.000	144	20	1.16E-13	XP_001628405.1	93	79.7221	45	42	predicted protein
Locus_2413_Transcript_1/1_Conf_1.000	1443	0							
Locus_24130_Transcript_1/1_Conf_1.000	184	2	9.80E-05	AAN10061.1	71	50.0618	38	27	Kunitz-like protease inhibitor precursor
Locus_24131_Transcript_1/1_Conf_1.000	173	0							
Locus_24132_Transcript_1/1_Conf_1.000	459	1	2.75E-04	XP_002647046.1	56	48.521	73	41	Hypothetical protein CBG03564
Locus_24133_Transcript_1/1_Conf_1.000	321	7	4.33E-13	XP_001901084.1	66	77.7962	77	51	hypothetical protein Bm1_48110
Locus_24134_Transcript_1/1_Conf_1.000	174	0							
Locus_24135_Transcript_1/1_Conf_1.000	218	0							
Locus_24136_Transcript_1/1_Conf_1.000	140	0							
Locus_24137_Transcript_1/1_Conf_1.000	128	0							
Locus_24138_Transcript_1/1_Conf_1.000	236	2	2.21E-17	NP_492944.2	73	92.0485	75	55	hypothetical protein F32B4.5
Locus_24139_Transcript_1/1_Conf_1.000	244	20	1.98E-18	NP_510410.1	75	95.5153	77	58	HIStone family member (his-24)
Locus_2414_Transcript_1/2_Conf_1.000	1323	20	3.46E-40	XP_002641351.1	72	170.629	148	108	C. briggsae CBR-TEG-1 protein
Locus_2414_Transcript_2/2_Conf_1.000	820	20	1.09E-20	NP_510410.1	73	104.76	89	65	HIStone family member (his-24)

Locus_24140_Transcript_1/1_Conf_1.000	141	20	8.80E-19	XP_002829265.1	100	96.6709	47	47	PREDICTED: rRNA 2'-O-methyltransferase fibrillarin-like
Locus_24141_Transcript_1/1_Conf_1.000	236	0							
Locus_24142_Transcript_1/1_Conf_1.000	174	20	2.57E-26	AAA18259.1	100	121.709	57	57	protein kinase C
Locus_24143_Transcript_1/1_Conf_1.000	328	20	7.59E-10	XP_001184423.1	51	67.0106	106	55	PREDICTED: similar to alpha-2-macroglobulin receptor
Locus_24144_Transcript_1/1_Conf_1.000	133	0							
Locus_24145_Transcript_1/1_Conf_1.000	246	20	6.78E-11	XP_001990298.1	71	70.4774	67	48	GH18314
Locus_24146_Transcript_1/1_Conf_1.000	130	0							

Locus_24147_Transcript_1/1_Conf_1.000	379	20	6.62E-30	XP_002639618.1	72	133.65	118	85	Hypothetical protein CBG12331
Locus_24148_Transcript_1/1_Conf_1.000	151	0							
Locus_24149_Transcript_1/1_Conf_1.000	339	0							
Locus_2415_Transcript_1/1_Conf_1.000	1170	20	0	XP_002642268.1	98	711.064	375	370	C. briggsae CBR-ACT-5 protein
Locus_24150_Transcript_1/1_Conf_1.000	206	20	1.13E-21	XP_001099076.2	100	106.301	49	49	PREDICTED: hypothetical protein LOC710457
Locus_24151_Transcript_1/1_Conf_1.000	347	0							
Locus_24152_Transcript_1/1_Conf_1.000	343	6	3.32E-37	AAK09440.1	93	157.918	86	80	AF339097_1fasciclin-like precursor protein
Locus_24153_Transcript_1/1_Conf_1.000	160	0							
Locus_24154_Transcript_1/1_Conf_1.000	128	0							
Locus_24155_Transcript_1/1_Conf_1.000	278	0							
Locus_24156_Transcript_1/1_Conf_1.000	328	0							
Locus_24157_Transcript_1/1_Conf_1.000	323	20	1.53E-34	NP_492266.2	84	149.058	106	90	hypothetical protein C34B7.2
Locus_24158_Transcript_1/1_Conf_1.000	237	0							
Locus_24159_Transcript_1/1_Conf_1.000	200	20	9.95E-10	NP_499001.1	70	66.6254	57	40	hypothetical protein R107.2
Locus_2416_Transcript_1/1_Conf_1.000	1155	20	9.92E-102	NP_492781.2	81	374.785	277	226	hypothetical protein B0511.7

Locus_24160_Transcript_1/1_Conf_1.000	450	20	6.19E-20	EFO19584.1	51	100.523	156	80	histone acetyltransferase MYST1
Locus_24161_Transcript_1/1_Conf_1.000	153	20	8.48E-17	XP_002643253.1	94	90.1225	50	47	C. briggsae CBR-AEX-3 protein
Locus_24162_Transcript_1/1_Conf_1.000	407	0							
Locus_24163_Transcript_1/1_Conf_1.000	314	20	1.89E-32	XP_002429721.1	75	142.124	104	79	low-density lipoprotein receptor, putative

Locus_24164_Transcript_1/1_Conf_1.000	285	20	3.79E-41	CAD44516.1	93	171.014	95	89	VAB-10B protein
Locus_24165_Transcript_1/1_Conf_1.000	132	0							
Locus_24166_Transcript_1/1_Conf_1.000	153	0							
Locus_24167_Transcript_1/1_Conf_1.000	228	0							
Locus_24168_Transcript_1/1_Conf_1.000	219	0							
Locus_24169_Transcript_1/1_Conf_1.000	217	18	1.04E-19	XP_002643289.1	82	99.7525	68	56	C. briggsae CBR-DVE-1 protein
Locus_2417_Transcript_1/2_Conf_1.000	896	0							
Locus_2417_Transcript_2/2_Conf_1.000	887	0							
Locus_24170_Transcript_1/1_Conf_1.000	138	0							
Locus_24171_Transcript_1/1_Conf_1.000	179	0							
Locus_24172_Transcript_1/1_Conf_1.000	198	0							
Locus_24173_Transcript_1/1_Conf_1.000	368	2	2.10E-12	XP_002630024.1	76	75.485	51	39	Hypothetical protein CBG13391
Locus_24174_Transcript_1/1_Conf_1.000	351	0							
Locus_24175_Transcript_1/1_Conf_1.000	131	0							
Locus_24176_Transcript_1/1_Conf_1.000	251	20	8.49E-30	XP_002634445.1	81	133.265	83	68	Hypothetical protein CBG04460
Locus_24177_Transcript_1/1_Conf_1.000	281	0							
Locus_24178_Transcript_1/1_Conf_1.000	234	20	7.83E-23	XP_002603816.1	81	110.153	76	62	hypothetical protein BRAFLDRAFT_124685
Locus_24179_Transcript_1/1_Conf_1.000	331	4	6.61E-14	EFO19179.1	61	80.4925	115	71	hypothetical protein LOAG_09315
Locus_2418_Transcript_1/1_Conf_1.000	485	20	2.37E-27	NP_497641.2	60	125.176	150	91	CaDHerin family member (cdh-1)

Locus_24180_Transcript_1/1_Conf_1.000	359	14	6.82E-19	NP_505989.1	57	97.0561	135	78	SULfate Permease family member (sulp-4)
Locus_24181_Transcript_1/1_Conf_1.000	291	0							
Locus_24182_Transcript_1/1_Conf_1.000	155	0							
Locus_24183_Transcript_1/1_Conf_1.000	203	0							
Locus_24184_Transcript_1/1_Conf_1.000	208	0							
Locus_24185_Transcript_1/1_Conf_1.000	208	20	7.49E-34	XP_002809493.1	100	146.747	65	65	PREDICTED: bifunctional aminoacyl-tRNA synthetase
Locus_24186_Transcript_1/1_Conf_1.000	166	0							
Locus_24187_Transcript_1/1_Conf_1.000	306	2	1.98E-10	NP_509809.2	60	68.9366	88	53	hypothetical protein C05A9.2
Locus_24188_Transcript_1/1_Conf_1.000	245	0							
Locus_24189_Transcript_1/1_Conf_1.000	164	0							
Locus_2419_Transcript_1/1_Conf_1.000	1171	20	3.20E-47	XP_002645722.1	63	193.741	234	149	C. briggsae CBR-ADM-2 protein
Locus_24190_Transcript_1/1_Conf_1.000	206	0							
Locus_24191_Transcript_1/1_Conf_1.000	166	20	3.28E-21	AAI00854.1	100	104.76	52	52	Cytochrome c oxidase subunit VIIa polypeptide 2 (liver)
Locus_24192_Transcript_1/1_Conf_1.000	154	20	3.33E-13	XP_002642408.1	84	78.1814	50	42	C. briggsae CBR-NHL-1 protein
Locus_24193_Transcript_1/1_Conf_1.000	178	0							
Locus_24194_Transcript_1/1_Conf_1.000	312	20	3.23E-24	XP_001901686.1	72	114.775	99	72	Cohesin subunit SA-1
Locus_24195_Transcript_1/1_Conf_1.000	279	0							
Locus_24196_Transcript_1/1_Conf_1.000	286	0							
Locus_24197_Transcript_1/1_Conf_1.000	146	0							
Locus_24198_Transcript_1/1_Conf_1.000	432	20	1.40E-64	NP_871810.1	93	248.825	143	133	HAlF transporter (PGP related) family member (haf-9)
Locus_24199_Transcript_1/1_Conf_1.000	264	20	1.81E-27	NP_495790.1	81	125.561	87	71	beta-LACTamase domain containing family member (lact-4)
Locus_242_Transcript_1/1_Conf_1.000	464	20	1.48E-37	NP_001021606.1	72	159.073	131	95	hypothetical protein T03F1.12
Locus_2420_Transcript_1/1_Conf_1.000	296	0							
Locus_24200_Transcript_1/1_Conf_1.000	144	5	2.11E-07	NP_001129925.1	74	58.9214	47	35	High Incidence of Males (increased X chromosome loss) family member (him-4)

Locus_24201_Transcript_1/1_Conf_1.000	240	20	9.53E-13	EFO15112.1	69	76.6406	76	53	hypothetical protein LOAG_13403
Locus_24202_Transcript_1/1_Conf_1.000	131	0							
Locus_24203_Transcript_1/1_Conf_1.000	216	0							
Locus_24204_Transcript_1/1_Conf_1.000	311	0							
Locus_24205_Transcript_1/1_Conf_1.000	150	20	1.19E-10	XP_002634930.1	82	69.707	46	38	Hypothetical protein CBG22529
Locus_24206_Transcript_1/1_Conf_1.000	138	0							
Locus_24207_Transcript_1/1_Conf_1.000	194	4	6.32E-14	XP_002631791.1	77	68.1662	48	37	Hypothetical protein CBG21009
Locus_24208_Transcript_1/1_Conf_1.000	168	14	1.47E-13	XP_002644186.1	85	79.337	54	46	Hypothetical protein CBG17168
Locus_24209_Transcript_1/1_Conf_1.000	522	20	6.05E-35	XP_002632055.1	61	150.599	181	111	C. briggsae CBR-VAB-19 protein
Locus_2421_Transcript_1/1_Conf_1.000	882	20	4.08E-72	XP_001900701.1	65	275.789	286	187	hypothetical protein Bm1_46195
Locus_24210_Transcript_1/1_Conf_1.000	265	20	1.29E-41	XP_002633950.1	87	172.555	88	77	C. briggsae CBR-FBL-1 protein
Locus_24211_Transcript_1/1_Conf_1.000	292	0							
Locus_24212_Transcript_1/1_Conf_1.000	163	0							
Locus_24213_Transcript_1/1_Conf_1.000	137	0							
Locus_24214_Transcript_1/1_Conf_1.000	159	20	5.64E-21	XP_002923631.1	100	103.99	52	52	PREDICTED: sodium/potassium-transporting ATPase subunit alpha-1-like isoform 2

Locus_24215_Transcript_1/1_Conf_1.000	270	10	1.21E-15	EFO20077.1	75	86.2705	72	54	hypothetical protein LOAG_08413
Locus_24216_Transcript_1/1_Conf_1.000	253	0							
Locus_24217_Transcript_1/1_Conf_1.000	405	1	2.70E-07	YP_002000086.1	45	58.5362	97	44	virulence-associated lipoprotein MIA
Locus_24218_Transcript_1/1_Conf_1.000	184	1	3.72E-04	AAO63576.1	53	48.1358	62	33	secreted protein 4 precursor
Locus_24219_Transcript_1/1_Conf_1.000	379	4	7.85E-15	XP_002631397.1	56	83.5741	121	68	C. briggsae CBR-DEP-1 protein
Locus_2422_Transcript_1/1_Conf_1.000	360	0							
Locus_24220_Transcript_1/1_Conf_1.000	375	20	4.75E-36	XP_002635372.1	80	154.066	118	95	C. briggsae CBR-RPM-1 protein
Locus_24221_Transcript_1/1_Conf_1.000	151	20	3.95E-14	ADM44660.1	82	81.2629	51	42	immunoglobulin epsilon heavy chain variable region
Locus_24222_Transcript_1/1_Conf_1.000	139	0							
Locus_24223_Transcript_1/1_Conf_1.000	195	20	9.94E-26	NP_495986.3	98	119.783	65	64	EATing: abnormal pharyngeal pumping family member (eat-3)
Locus_24224_Transcript_1/1_Conf_1.000	136	0							
Locus_24225_Transcript_1/1_Conf_1.000	298	0							
Locus_24226_Transcript_1/1_Conf_1.000	142	20	9.41E-21	XP_001713909.1	100	103.219	46	46	PREDICTED: similar to MHC class II antigen
Locus_24227_Transcript_1/1_Conf_1.000	253	7	5.91E-18	NP_494897.3	66	93.9745	80	53	hypothetical protein R05F9.12
Locus_24228_Transcript_1/1_Conf_1.000	221	0							
Locus_24229_Transcript_1/1_Conf_1.000	244	0							

Locus_2423_Transcript_1/2_Conf_1.000	1601	20	1.59E-138	XP_002637274.1	81	497.664	356	291	Hypothetical protein CBG18957
Locus_2423_Transcript_2/2_Conf_1.000	1541	20	6.60E-142	XP_002637274.1	86	508.834	336	289	Hypothetical protein CBG18957
Locus_24230_Transcript_1/1_Conf_1.000	266	20	1.53E-18	EFO24692.1	70	95.9005	86	61	protein-tyrosine phosphatase
Locus_24231_Transcript_1/1_Conf_1.000	265	12	5.64E-13	XP_002640224.1	68	77.411	83	57	C. briggsae CBR-SMG-1 protein
Locus_24232_Transcript_1/1_Conf_1.000	129	0							
Locus_24233_Transcript_1/1_Conf_1.000	132	0							
Locus_24234_Transcript_1/1_Conf_1.000	162	20	1.58E-23	DAA31575.1	100	112.464	53	53	cold shock domain containing E1, RNA-binding
Locus_24235_Transcript_1/1_Conf_1.000	520	0							
Locus_24236_Transcript_1/1_Conf_1.000	221	20	3.95E-30	NP_741812.1	89	134.42	73	65	hypothetical protein T07H6.1
Locus_24237_Transcript_1/1_Conf_1.000	200	20	2.00E-18	NP_872022.1	80	95.5153	65	52	CysTiNoSin (lysosomal protein) homolog family member (ctns-1)
Locus_24238_Transcript_1/1_Conf_1.000	149	0							
Locus_24239_Transcript_1/1_Conf_1.000	234	0							
Locus_2424_Transcript_1/2_Conf_1.000	1973	20	3.29E-152	NP_491664.1	61	543.502	685	423	UNCoordinated family member (unc-40)
Locus_2424_Transcript_2/2_Conf_1.000	1793	20	3.67E-171	NP_491664.1	69	606.29	625	432	UNCoordinated family member (unc-40)
Locus_24240_Transcript_1/1_Conf_1.000	313	0							
Locus_24241_Transcript_1/1_Conf_1.000	418	0							
Locus_24242_Transcript_1/1_Conf_1.000	357	3	6.18E-12	NP_001023197.1	64	73.9442	111	72	hypothetical protein F38E11.6
Locus_24243_Transcript_1/1_Conf_1.000	181	2	4.12E-11	XP_001898554.1	72	71.2478	59	43	DOMON domain containing protein

Locus_24244_Transcript_1/1_Conf_1.000	150	20	6.09E-15	XP_002640668.1	97	83.9593	45	44	C. briggsae CBR-UNC-54 protein
Locus_24245_Transcript_1/1_Conf_1.000	249	0							
Locus_24246_Transcript_1/1_Conf_1.000	280	0							
Locus_24247_Transcript_1/1_Conf_1.000	212	20	1.41E-24	NP_508573.1	85	115.931	70	60	Discoidin Domain Receptor family member (ddr-2)
Locus_24248_Transcript_1/1_Conf_1.000	220	0							
Locus_24249_Transcript_1/1_Conf_1.000	415	0							

Locus_2425_Transcript_1/1_Conf_1.000	1947	20	0	NP_490866.4	95	833.558	463	442	SRC oncogene related family member (src-1)
Locus_24250_Transcript_1/1_Conf_1.000	412	20	3.37E-18	XP_002631639.1	62	94.7449	116	73	Hypothetical protein CBG20828
Locus_24251_Transcript_1/1_Conf_1.000	169	0							
Locus_24252_Transcript_1/1_Conf_1.000	165	0							
Locus_24253_Transcript_1/1_Conf_1.000	195	20	2.53E-21	NP_495697.2	90	105.145	64	58	Lim and Transglutaminase Domain family member (ltd-1)
Locus_24254_Transcript_1/1_Conf_1.000	246	0							
Locus_24255_Transcript_1/1_Conf_1.000	174	2	9.19E-08	XP_002646794.1	75	60.077	53	40	C. briggsae CBR-RHGF-2 protein
Locus_24256_Transcript_1/1_Conf_1.000	219	0							
Locus_24257_Transcript_1/1_Conf_1.000	131	0							
Locus_24258_Transcript_1/1_Conf_1.000	208	2	4.89E-09	NP_490719.1	69	64.3142	53	37	hypothetical protein Y48G1A.3
Locus_24259_Transcript_1/1_Conf_1.000	600	6	1.06E-35	CAR63655.1	94	153.68	73	69	hypothetical protein
Locus_2426_Transcript_1/1_Conf_1.000	502	20	1.71E-09	EFO21554.1	56	65.855	81	46	hypothetical protein LOAG_06935
Locus_24260_Transcript_1/1_Conf_1.000	297	20	7.28E-45	NP_498931.2	91	183.341	101	92	SMAll family member (sma-2)
Locus_24261_Transcript_1/1_Conf_1.000	142	0							
Locus_24262_Transcript_1/1_Conf_1.000	204	0							
Locus_24263_Transcript_1/1_Conf_1.000	168	0							
Locus_24264_Transcript_1/1_Conf_1.000	181	0							

[truncated: 630,610 more chars]
