# Supplementary material for: Proteomic Analysis of Excretory-Secretory Products of Heligmosomoides polygyrus Assessed with Next-Generation Sequencing Transcriptomic Information
Source: PLoS Negl Trop Dis. 2011 Oct 25;5(10):e1370. doi: 10.1371/journal.pntd.0001370 (PMC3201918; doi:10.1371/journal.pntd.0001370)
Supplement: Table S3 — Proteins identified in ESP from H. polygyrus through search on conceptual translation from RNA-seq assembly. (PDF) [file pntd.0001370.s003.pdf]

Table S3. Proteins identified in ESP from *H. polygyrus* through search on conceptual translation from RNA-seq assembly.

Protein name	Blast2GO Seq. Description	Protein accession numbers	Protein identification probability
**Locus_272_Transcript_4/4_Confidence_0.700_3**	venom-allergen-like protein family member (vap-1)	Locus_272_Transcript_4_0.700_3	100.00%
**Locus_566_Transcript_2/6_Confidence_0.476_3**	globin	Locus_566_Transcript_2_0.476_3,Locus_566_Transcript_3_0.571_3,Locus_566_Transcript_4_0.524_3,Locus_566_Transcript_5_0.762_2,Locus_566_Transcript_6_0.762_3	100.00%
**Locus_120_Transcript_1/8_Confidence_0.410_1**	secreted protein asp-2	Locus_120_Transcript_1_0.410_1,Locus_120_Transcript_5_0.436_1,Locus_120_Transcript_8_0.462_1	100.00%
**Locus_384_Transcript_8/9_Confidence_0.306_5**	globin	Locus_384_Transcript_8_0.306_5	100.00%
**Locus_2405_Transcript_4/4_Confidence_0.700_4**	secreted protein asp-2	Locus_2405_Transcript_4_0.700_4	100.00%
**Locus_2261_Transcript_1/1_Confidence_1.000_4**	globin	Locus_2261_Transcript_1_1.000_4	100.00%
**Locus_73_Transcript_5/5_Confidence_0.619_4**	vit-2	Locus_73_Transcript_5_0.619_4	100.00%
**Locus_94_Transcript_3/4_Confidence_0.429_4**	elegans protein confirmed by transcript evidence	Locus_94_Transcript_3_0.429_4,Locus_94_Transcript_4_0.429_1	100.00%
**Locus_122_Transcript_10/10_Confidence_0.312_3**	vitellogenin structural genes (yolk protein genes) family member (vit-2)	Locus_122_Transcript_10_0.312_3	100.00%
**Locus_2367_Transcript_1/3_Confidence_0.375_1**	ll20 15kda ladder antigen	Locus_2367_Transcript_1_0.375_1	100.00%
**Locus_93_Transcript_5/6_Confidence_0.444_2**	vitellogenin structural genes (yolk protein genes) family member (vit-2)	Locus_93_Transcript_5_0.444_2	100.00%
**Locus_207_Transcript_17/20_Confidence_0.196_1**	briggsae cbr-vit-5 protein	Locus_207_Transcript_17_0.196_1	100.00%
**Locus_290_Transcript_3/3_Confidence_0.833_3**	briggsae cbr-vit-2 protein	Locus_290_Transcript_3_0.833_3	100.00%
**Locus_211_Transcript_5/5_Confidence_0.765_5**	vitellogenin structural genes (yolk protein genes) family member (vit-6)	Locus_211_Transcript_5_0.765_5	100.00%

**Locus_3_Transcript_3/3_Confidence_0.875_4**	vitellogenin structural genes (yolk protein genes) family member (vit-2)	Locus_3_Transcript_3_0.875_4	100.00%
**Locus_2733_Transcript_1/1_Confidence_1.000_3**	globin	Locus_2733_Transcript_1_1.000_3	100.00%
**Locus_6303_Transcript_1/1_Confidence_1.000_1**	ancylostoma-secreted protein 1 precursor	Locus_6303_Transcript_1_1.000_1	99.80%
**Locus_927_Transcript_1/2_Confidence_1.000_6**	---NA---	Locus_927_Transcript_1_1.000_6,Locus_927_Transcript_2_1.000_4	99.80%
**Locus_160_Transcript_5/5_Confidence_0.733_4**	secreted protein asp-2	Locus_160_Transcript_5_0.733_4	100.00%
**Locus_407_Transcript_1/2_Confidence_1.000_5**	vitellogenin structural genes (yolk protein genes) family member (vit-6)	Locus_407_Transcript_1_1.000_5	100.00%
**Locus_7106_Transcript_1/1_Confidence_1.000_2**	acetylcholinesterase 2	Locus_7106_Transcript_1_1.000_2	100.00%
**Locus_21253_Transcript_1/1_Confidence_1.000_5**	secreted protein 5 precursor	Locus_21253_Transcript_1_1.000_5	100.00%
**Locus_420_Transcript_1/1_Confidence_1.000_1**	venom-allergen-like protein family member (vap-1)	Locus_420_Transcript_1_1.000_1	100.00%
**Locus_2033_Transcript_1/1_Confidence_1.000_4**	globin	Locus_2033_Transcript_1_1.000_4	100.00%
**Locus_391_Transcript_1/2_Confidence_1.000_3**	large subunit ribosomal protein 6	Locus_391_Transcript_1_1.000_3,Locus_391_Transcript_2_1.000_2	100.00%
**Locus_1550_Transcript_1/1_Confidence_1.000_1**	briggsae cbr-vit-6 protein	Locus_1550_Transcript_1_1.000_1,Locus_51_Transcript_1_1.000_5	100.00%
**Locus_1900_Transcript_4/6_Confidence_0.667_6**	calcium activated nucleotidase 1	Locus_1900_Transcript_4_0.667_6,Locus_1900_Transcript_6_0.667_6	100.00%
**Locus_633_Transcript_3/4_Confidence_0.444_5**	briggsae cbr-vit-6 protein	Locus_633_Transcript_3_0.444_5	100.00%
**Locus_4583_Transcript_1/1_Confidence_1.000_5**	venom-allergen-like protein family member (vap-1)	Locus_4583_Transcript_1_1.000_5	100.00%

**Locus_541_Transcript_1/4_Confidence_0.692_4**	enolase	Locus_541_Transcript_1_0.692_4,Locus_541_Transcript_3_0.692_4,Locus_541_Transcript_4_0.692_4	100.00%
**Locus_5904_Transcript_1/1_Confidence_1.000_4**	secreted protein asp-2	Locus_5904_Transcript_1_1.000_4	100.00%
**Locus_6562_Transcript_1/1_Confidence_1.000_4**	---NA---	Locus_6562_Transcript_1_1.000_4	99.80%
**Locus_7877_Transcript_1/1_Confidence_1.000_4**	---NA---	Locus_7877_Transcript_1_1.000_4	100.00%
**Locus_97_Transcript_13/17_Confidence_0.302_6**	secreted protein 5 precursor	Locus_97_Transcript_13_0.302_6	100.00%
**Locus_13795_Transcript_1/1_Confidence_1.000_4**	nematode polyprotein allergen related family member (npa-1)	Locus_13795_Transcript_1_1.000_4	100.00%
**Locus_1878_Transcript_2/2_Confidence_1.000_2**	elegans protein confirmed by transcript evidence	Locus_1878_Transcript_2_1.000_2	100.00%
**Locus_2046_Transcript_1/2_Confidence_1.000_1**	secreted protein 5 precursor	Locus_2046_Transcript_1_1.000_1	100.00%
**Locus_3463_Transcript_1/2_Confidence_1.000_1**	elegans protein confirmed by transcript evidence	Locus_3463_Transcript_1_1.000_1	100.00%
**Locus_207_Transcript_19/20_Confidence_0.107_3**	vit-2	Locus_207_Transcript_19_0.107_3	99.80%
**Locus_2879_Transcript_1/1_Confidence_1.000_2**	---NA---	Locus_2879_Transcript_1_1.000_2	100.00%
**Locus_3116_Transcript_1/1_Confidence_1.000_4**	annexin a13	Locus_3116_Transcript_1_1.000_4	100.00%
**Locus_313_Transcript_1/2_Confidence_1.000_2**	briggsae cbr-vit-6 protein	Locus_313_Transcript_1_1.000_2	100.00%
**Locus_4418_Transcript_1/1_Confidence_1.000_4**	secreted protein asp-2	Locus_4418_Transcript_1_1.000_4	100.00%
**Locus_564_Transcript_6/7_Confidence_0.167_2**	parasitic stage specific protein 2	Locus_564_Transcript_6_0.167_2	100.00%
**Locus_630_Transcript_1/3_Confidence_0.375_5**	briggsae cbr-vit-6 protein	Locus_630_Transcript_1_0.375_5,Locus_630_Transcript_2_0.625_5,Locus_630_Transcript_3_0.750_6	99.80%
**Locus_8762_Transcript_1/1_Confidence_1.000_4**	elegans protein partially confirmed by transcript evidence	Locus_8762_Transcript_1_1.000_4	100.00%
**Locus_1002_Transcript_1/1_Confidence_1.000_4**	secreted protein 6 precursor	Locus_1002_Transcript_1_1.000_4	99.80%
**Locus_11_Transcript_14/14_Confidence_0.176_5**	vitellogenin structural genes (yolk protein genes) family member (vit-6)	Locus_11_Transcript_14_0.176_5,Locus_11_Transcript_7_0.176_5	99.80%
**Locus_215_Transcript_2/2_Confidence_1.000_3**	fatty acid-binding protein homolog	Locus_215_Transcript_2_1.000_3	100.00%

**Locus_2656_Transcript_1/4_Confidence_0.625_3**	acetylcholinesterase 2	Locus_2656_Transcript_1_0.625_3,Locus_2656_Transcript_3_0.625_2,Locus_2656_Transcript_4_0.625_2	100.00%
**Locus_498_Transcript_1/2_Confidence_1.000_2**	14-3-3 protein	Locus_498_Transcript_1_1.000_2	100.00%
**Locus_1319_Transcript_1/2_Confidence_1.000_3**	elegans protein confirmed by transcript evidence	Locus_1319_Transcript_1_1.000_3,Locus_1319_Transcript_2_1.000_3	100.00%
**Locus_1358_Transcript_5/6_Confidence_0.357_4**	aldolase	Locus_1358_Transcript_5_0.357_4,Locus_1358_Transcript_6_0.357_2	100.00%
**Locus_169_Transcript_1/2_Confidence_1.000_2**	fatty acid and retinol-binding protein 1	Locus_169_Transcript_1_1.000_2	100.00%
**Locus_186_Transcript_3/3_Confidence_0.700_1**	---NA---	Locus_186_Transcript_3_0.700_1	100.00%
**Locus_3619_Transcript_1/1_Confidence_1.000_1**	zinc metallopeptidase	Locus_3619_Transcript_1_1.000_1	100.00%
**Locus_5886_Transcript_1/1_Confidence_1.000_2**	secreted protein asp-2	Locus_5886_Transcript_1_1.000_2	100.00%
**Locus_153_Transcript_2/3_Confidence_0.833_5**	briggsae cbr-vit-4 protein	Locus_153_Transcript_2_0.833_5,Locus_153_Transcript_3_0.833_5	100.00%
**Locus_3236_Transcript_1/1_Confidence_1.000_6**	---NA---	Locus_3236_Transcript_1_1.000_6	99.80%
**Locus_347_Transcript_1/1_Confidence_1.000_1**	---NA---	Locus_347_Transcript_1_1.000_1	100.00%
**Locus_3624_Transcript_1/1_Confidence_1.000_2**	cytosolic glutathione s-transferase 1	Locus_3624_Transcript_1_1.000_2	100.00%
**Locus_5942_Transcript_1/1_Confidence_1.000_2**	---NA---	Locus_5942_Transcript_1_1.000_2	100.00%
**Locus_633_Transcript_1/4_Confidence_0.556_2**	briggsae cbr-vit-2 protein	Locus_633_Transcript_1_0.556_2,Locus_633_Transcript_2_0.556_2,Locus_633_Transcript_4_0.444_2	99.80%
**Locus_7308_Transcript_3/3_Confidence_0.400_6**	serine or cysteine protease inhibitor	Locus_7308_Transcript_3_0.400_6	100.00%
**Locus_8124_Transcript_1/1_Confidence_1.000_6**	zinc metallopeptidase	Locus_8124_Transcript_1_1.000_6	100.00%
**Locus_8688_Transcript_1/1_Confidence_1.000_4**	venom-allergen-like protein family member (vap-1)	Locus_8688_Transcript_1_1.000_4	100.00%

**Locus_975_Transcript_1/1_Confidence_1.000_2**	vitellogenin structural genes (yolk protein genes) family member (vit-2)	Locus_975_Transcript_1_1.000_2	99.80%
**Locus_1267_Transcript_1/2_Confidence_1.000_2**	isoform cra_a	Locus_1267_Transcript_1_1.000_2	100.00%
**Locus_2061_Transcript_1/1_Confidence_1.000_2**	c4b-binding protein alpha chain precursor	Locus_2061_Transcript_1_1.000_2	100.00%
**Locus_2142_Transcript_1/2_Confidence_1.000_6**	ancylostoma-secreted protein 1 precursor	Locus_2142_Transcript_1_1.000_6	100.00%
**Locus_2405_Transcript_3/4_Confidence_0.600_4**	elegans protein partially confirmed by transcript evidence	Locus_2405_Transcript_3_0.600_4	99.80%
**Locus_4423_Transcript_1/1_Confidence_1.000_3**	---NA---	Locus_4423_Transcript_1_1.000_3	100.00%
**Locus_7205_Transcript_1/1_Confidence_1.000_5**	---NA---	Locus_7205_Transcript_1_1.000_5	100.00%
**Locus_800_Transcript_3/4_Confidence_0.375_3**	---NA---	Locus_800_Transcript_3_0.375_3,Locus_800_Transcript_4_0.375_4	99.90%
**Locus_970_Transcript_3/4_Confidence_0.750_3**	thiamine pyrophosphate central domain containing protein	Locus_970_Transcript_3_0.750_3	99.80%
**Locus_10349_Transcript_1/1_Confidence_1.000_6**	novel protein containing multiple sushi domains (scr repeat)	Locus_10349_Transcript_1_1.000_6	100.00%
**Locus_11_Transcript_11/14_Confidence_0.147_5**	vitellogenin structural genes (yolk protein genes) family member (vit-6)	Locus_11_Transcript_11_0.147_5,Locus_11_Transcript_12_0.147_3	99.80%
**Locus_153_Transcript_1/3_Confidence_0.333_5**	---NA---	Locus_153_Transcript_1_0.333_5,Locus_153_Transcript_2_0.833_4,Locus_153_Transcript_3_0.833_4	99.80%
**Locus_1737_Transcript_1/5_Confidence_0.333_1**	translationally controlled tumor protein	Locus_1737_Transcript_1_0.333_1,Locus_1737_Transcript_3_0.417_2,Locus_1737_Transcript_4_0.500_2	100.00%
**Locus_22057_Transcript_1/1_Confidence_1.000_4**	---NA---	Locus_22057_Transcript_1_1.000_4	100.00%
**Locus_2417_Transcript_2/2_Confidence_1.000_1**	---NA---	Locus_2417_Transcript_2_1.000_1	100.00%

**Locus_2724_Transcript_1/2_Confidence_1.000_3**	hypothetical protein [Angiostrongylus cantonensis]	Locus_2724_Transcript_1_1.000_3,Locus_2724_Transcript_1_1.000_6,Locus_2724_Transcript_2_1.000_3,Locus_2724_Transcript_2_1.000_6	100.00%
**Locus_2728_Transcript_1/2_Confidence_1.000_3**	briggsae cbr-cpi-2 protein	Locus_2728_Transcript_1_1.000_3,Locus_2728_Transcript_2_1.000_3	100.00%
**Locus_3858_Transcript_1/5_Confidence_0.500_3**	serine protease	Locus_3858_Transcript_1_0.500_3,Locus_3858_Transcript_3_0.500_3,Locus_3858_Transcript_5_0.500_3	100.00%
**Locus_3914_Transcript_1/1_Confidence_1.000_6**	briggsae cbr-ttr-16 protein	Locus_3914_Transcript_1_1.000_6	100.00%
**Locus_3925_Transcript_1/1_Confidence_1.000_2**	---NA---	Locus_3925_Transcript_1_1.000_2	99.80%
**Locus_5527_Transcript_1/1_Confidence_1.000_1**	secreted protein asp-2	Locus_5527_Transcript_1_1.000_1	100.00%
**Locus_825_Transcript_1/2_Confidence_1.000_6**	calcium activated nucleotidase isoform cra_b	Locus_825_Transcript_1_1.000_6	100.00%
**Locus_100_Transcript_1/1_Confidence_1.000_6**	globin	Locus_100_Transcript_1_1.000_6	100.00%
**Locus_1081_Transcript_1/2_Confidence_1.000_5**	---NA---	Locus_1081_Transcript_1_1.000_5	99.90%
**Locus_1213_Transcript_3/4_Confidence_0.500_1**	elegans protein confirmed by transcript evidence	Locus_1213_Transcript_3_0.500_1,Locus_1213_Transcript_4_0.500_1	100.00%
**Locus_1216_Transcript_1/1_Confidence_1.000_4**	placental protein 11 related	Locus_1216_Transcript_1_1.000_4	100.00%
**Locus_1500_Transcript_1/1_Confidence_1.000_2**	actin	Locus_1500_Transcript_1_1.000_2,Locus_314_Transcript_2_0.625_6,Locus_314_Transcript_3_0.625_6	100.00%
**Locus_1908_Transcript_1/2_Confidence_1.000_1**	cysteine proteinase	Locus_1908_Transcript_1_1.000_1	100.00%
**Locus_2287_Transcript_1/4_Confidence_0.625_3**	elegans protein confirmed by transcript evidence	Locus_2287_Transcript_1_0.625_3,Locus_2287_Transcript_2_0.625_1,Locus_2287_Transcript_4_0.625_3	100.00%
**Locus_2684_Transcript_2/2_Confidence_1.000_1**	---NA---	Locus_2684_Transcript_2_1.000_1	100.00%
**Locus_3465_Transcript_2/3_Confidence_0.812_4**	---NA---	Locus_3465_Transcript_2_0.812_4	100.00%
**Locus_4068_Transcript_1/1_Confidence_1.000_3**	---NA---	Locus_4068_Transcript_1_1.000_3	100.00%

**Locus_412_Transcript_1/6_Confidence_0.625_3**	peroxiredoxin 1 variant 2	Locus_412_Transcript_1_0.625_3,Locus_412_Transcript_1_0.625_4,Locus_412_Transcript_2_0.625_3,Locus_412_Transcript_2_0.625_4,Locus_412_Transcript_5_0.375_5,Locus_412_Transcript_6_0.375_3	100.00%
**Locus_5744_Transcript_4/4_Confidence_0.625_3**	---NA---	Locus_5744_Transcript_4_0.625_3	100.00%
**Locus_73_Transcript_4/5_Confidence_0.095_3**	globin	Locus_73_Transcript_4_0.095_3	100.00%
**Locus_785_Transcript_1/7_Confidence_0.444_3**	---NA---	Locus_785_Transcript_1_0.444_3,Locus_785_Transcript_3_0.389_3,Locus_785_Transcript_6_0.500_3,Locus_785_Transcript_7_0.556_3	100.00%
**Locus_844_Transcript_1/1_Confidence_1.000_6**	---NA---	Locus_844_Transcript_1_1.000_6	99.80%
**Locus_9150_Transcript_1/1_Confidence_1.000_5**	hypothetical protein K02F3.9 [Caenorhabditis elegans]	Locus_9150_Transcript_1_1.000_5	99.80%
**Locus_949_Transcript_1/1_Confidence_1.000_6**	venom allergen antigen-like protein 1	Locus_949_Transcript_1_1.000_6	100.00%
**Locus_11_Transcript_10/14_Confidence_0.176_2**	vitellogenin structural genes (yolk protein genes) family member (vit-6)	Locus_11_Transcript_10_0.176_2,Locus_11_Transcript_13_0.176_2	99.80%
**Locus_1311_Transcript_1/1_Confidence_1.000_6**	secreted protein 5 precursor	Locus_1311_Transcript_1_1.000_6	99.80%
**Locus_1600_Transcript_1/1_Confidence_1.000_4**	paz piwi domain-containing family member (ppw-1)	Locus_1600_Transcript_1_1.000_4	100.00%
**Locus_16128_Transcript_1/2_Confidence_1.000_2**	calcium activated nucleotidase isoform cra_b	Locus_16128_Transcript_1_1.000_2,Locus_16128_Transcript_2_1.000_2	100.00%
**Locus_1798_Transcript_1/3_Confidence_0.750_4**	c-type lectin family member (clec-266)	Locus_1798_Transcript_1_0.750_4,Locus_1798_Transcript_2_0.750_4,Locus_1798_Transcript_3_0.750_4	100.00%
**Locus_2189_Transcript_1/2_Confidence_1.000_3**	aspartyl protease inhibitor 1	Locus_2189_Transcript_1_1.000_3,Locus_2189_Transcript_2_1.000_3	100.00%

**Locus_2287_Transcript_3/4_Confidence_0.375_3**	elegans protein confirmed by transcript evidence	Locus_2287_Transcript_3_0.375_3	99.80%
**Locus_2365_Transcript_1/1_Confidence_1.000_1**	metalloprotease 1 precursor	Locus_2365_Transcript_1_1.000_1	100.00%
**Locus_2423_Transcript_1/2_Confidence_1.000_2**	malate cytoplasmic	Locus_2423_Transcript_1_1.000_2,Locus_2423_Transcript_2_1.000_2	100.00%
**Locus_3123_Transcript_1/2_Confidence_1.000_2**	elegans protein confirmed by transcript evidence	Locus_3123_Transcript_1_1.000_2,Locus_3123_Transcript_1_1.000_5,Locus_3123_Transcript_2_1.000_2,Locus_3123_Transcript_2_1.000_5	100.00%
**Locus_38_Transcript_1/1_Confidence_1.000_2**	elegans protein partially confirmed by transcript evidence	Locus_38_Transcript_1_1.000_2	100.00%
**Locus_5855_Transcript_1/1_Confidence_1.000_2**	elegans protein partially confirmed by transcript evidence	Locus_5855_Transcript_1_1.000_2	99.80%
**Locus_6538_Transcript_1/1_Confidence_1.000_1**	elegans protein confirmed by transcript evidence	Locus_6538_Transcript_1_1.000_1	99.80%
**Locus_6601_Transcript_1/1_Confidence_1.000_5**	calcium activated nucleotidase isoform cra_b	Locus_6601_Transcript_1_1.000_5	100.00%
**Locus_7004_Transcript_1/1_Confidence_1.000_1**	elegans protein confirmed by transcript evidence	Locus_7004_Transcript_1_1.000_1	99.80%
**Locus_7986_Transcript_2/2_Confidence_1.000_5**	---NA---	Locus_7986_Transcript_2_1.000_5	100.00%
**Locus_8223_Transcript_1/1_Confidence_1.000_4**	---NA---	Locus_8223_Transcript_1_1.000_4	100.00%
**Locus_8678_Transcript_1/1_Confidence_1.000_1**	triosephosphate isomerase	Locus_8678_Transcript_1_1.000_1	100.00%
**Locus_1079_Transcript_1/1_Confidence_1.000_3**	ppib protein	Locus_1079_Transcript_1_1.000_3	100.00%
**Locus_11813_Transcript_1/1_Confidence_1.000_4**	ll20 15kda ladder antigen	Locus_11813_Transcript_1_1.000_4	99.80%

**Locus_12666_Transcript_1/1_Confidence_1.000_2**	---NA---	Locus_12666_Transcript_1_1.000_2	100.00%
**Locus_13497_Transcript_1/1_Confidence_1.000_3**	---NA---	Locus_13497_Transcript_1_1.000_3	99.80%
**Locus_16079_Transcript_1/1_Confidence_1.000_1**	elegans protein partially confirmed by transcript evidence	Locus_16079_Transcript_1_1.000_1	99.90%
**Locus_2530_Transcript_1/7_Confidence_0.409_4**	metalloprotease 1 precursor	Locus_2530_Transcript_1_0.409_4,Locus_2530_Transcript_3_0.409_4,Locus_2530_Transcript_4_0.364_4,Locus_2530_Transcript_5_0.545_4,Locus_2530_Transcript_7_0.409_4	100.00%
**Locus_3111_Transcript_1/2_Confidence_1.000_4**	phosphatidylethanolamine-binding protein	Locus_3111_Transcript_1_1.000_4,Locus_3111_Transcript_2_1.000_4	100.00%
**Locus_3209_Transcript_2/6_Confidence_0.438_6**	elegans protein partially confirmed by transcript evidence	Locus_3209_Transcript_2_0.438_6	99.80%
**Locus_3370_Transcript_1/1_Confidence_1.000_4**	ribonucleotide reductase m2 polypeptide	Locus_3370_Transcript_1_1.000_4	100.00%
**Locus_3502_Transcript_1/2_Confidence_1.000_5**	---NA---	Locus_3502_Transcript_1_1.000_5,Locus_3502_Transcript_2_1.000_2	99.80%
**Locus_361_Transcript_1/1_Confidence_1.000_3**	elegans protein partially confirmed by transcript evidence	Locus_361_Transcript_1_1.000_3	100.00%
**Locus_3626_Transcript_5/5_Confidence_0.600_3**	novel protein hatching enzymes	Locus_3626_Transcript_5_0.600_3	100.00%

**Locus_404_Transcript_5/6_Confidence_0.250_4**	elegans protein confirmed by transcript evidence	Locus_404_Transcript_5_0.250_4,Locus_404_Transcript_6_0.250_3	99.80%
**Locus_4720_Transcript_1/1_Confidence_1.000_1**	---NA---	Locus_4720_Transcript_1_1.000_1	100.00%
**Locus_4863_Transcript_1/1_Confidence_1.000_2**	elegans protein confirmed by transcript evidence	Locus_4863_Transcript_1_1.000_2	100.00%
**Locus_541_Transcript_1/4_Confidence_0.692_5**	enolase	Locus_541_Transcript_1_0.692_5,Locus_541_Transcript_3_0.692_5,Locus_541_Transcript_4_0.692_5	100.00%
**Locus_624_Transcript_3/3_Confidence_0.625_2**	tissue factor pathway inhibitor	Locus_624_Transcript_3_0.625_2	100.00%
**Locus_6250_Transcript_1/1_Confidence_1.000_3**	lysozyme	Locus_6250_Transcript_1_1.000_3	100.00%
**Locus_6760_Transcript_1/1_Confidence_1.000_1**	---NA---	Locus_6760_Transcript_1_1.000_1	100.00%
**Locus_7281_Transcript_1/1_Confidence_1.000_3**	secreted protein asp-2	Locus_7281_Transcript_1_1.000_3	100.00%
**Locus_8509_Transcript_1/1_Confidence_1.000_2**	zinc metallopeptidase	Locus_8509_Transcript_1_1.000_2	100.00%
**Locus_9113_Transcript_1/1_Confidence_1.000_1**	elegans protein confirmed by transcript evidence	Locus_9113_Transcript_1_1.000_1	100.00%
**Locus_106_Transcript_1/1_Confidence_1.000_3**	protein disulfide isomerase	Locus_106_Transcript_1_1.000_3	100.00%
**Locus_10663_Transcript_1/1_Confidence_1.000_2**	tissue factor pathway inhibitor 2-like	Locus_10663_Transcript_1_1.000_2	99.80%

**Locus_1117_Transcript_1/6_Confidence_0.250_2**	---NA---	Locus_1117_Transcript_1_0.250_2,Locus_1117_Transcript_3_0.708_2,Locus_1117_Transcript_3_0.708_5,Locus_1117_Transcript_4_0.542_2,Locus_1117_Transcript_5_0.667_2,Locus_1117_Transcript_5_0.667_5,Locus_1117_Transcript_6_0.667_2,Locus_1117_Transcript_6_0.667_5	100.00%
**Locus_11537_Transcript_1/1_Confidence_1.000_4**	l3 es protein	Locus_11537_Transcript_1_1.000_4	100.00%
**Locus_123_Transcript_7/8_Confidence_0.400_6**	heat shock protein 70	Locus_123_Transcript_7_0.400_6,Locus_123_Transcript_8_0.400_2	99.80%
**Locus_1247_Transcript_1/2_Confidence_1.000_4**	major sperm protein	Locus_1247_Transcript_1_1.000_4,Locus_1247_Transcript_2_1.000_4	99.80%
**Locus_12822_Transcript_1/1_Confidence_1.000_3**	peptidase s9 prolyl oligopeptidase active site domain protein	Locus_12822_Transcript_1_1.000_3	100.00%
**Locus_13707_Transcript_1/1_Confidence_1.000_3**	---NA---	Locus_13707_Transcript_1_1.000_3	99.80%
**Locus_1814_Transcript_1/1_Confidence_1.000_6**	nucleoside diphosphate kinase b	Locus_1814_Transcript_1_1.000_6	99.80%
**Locus_18853_Transcript_1/1_Confidence_1.000_5**	---NA---	Locus_18853_Transcript_1_1.000_5	99.80%
**Locus_20948_Transcript_1/1_Confidence_1.000_2**	briggsae cbr-far-8 protein	Locus_20948_Transcript_1_1.000_2	100.00%
**Locus_23434_Transcript_1/1_Confidence_1.000_4**	ladder protein	Locus_23434_Transcript_1_1.000_4	99.80%
**Locus_24_Transcript_1/1_Confidence_1.000_3**	---NA---	Locus_24_Transcript_1_1.000_3	99.80%
**Locus_2902_Transcript_2/3_Confidence_0.667_5**	secreted protein asp-2	Locus_2902_Transcript_2_0.667_5	100.00%

**Locus_3209_Transcript_3/6_Confidence_0.375_5**	elegans protein partially confirmed by transcript evidence	Locus_3209_Transcript_3_0.375_5,Locus_3209_Transcript_4_0.375_6	99.80%
**Locus_3220_Transcript_1/2_Confidence_1.000_4**	ferritin	Locus_3220_Transcript_1_1.000_4,Locus_3220_Transcript_2_1.000_4	100.00%
**Locus_4325_Transcript_1/1_Confidence_1.000_2**	mannose c type 1	Locus_4325_Transcript_1_1.000_2	100.00%
**Locus_4404_Transcript_1/1_Confidence_1.000_3**	zinc metallopeptidase	Locus_4404_Transcript_1_1.000_3	100.00%
**Locus_5289_Transcript_2/3_Confidence_0.667_2**	anis1_anisi ame: full=major allergen ani s 1 ame: full=21 kda allergen ame: full=excretory gland allergen ans1 ame: allergen=ani s 1 flags: precursor	Locus_5289_Transcript_2_0.667_2	100.00%
**Locus_55_Transcript_1/2_Confidence_0.889_2**	briggsae cbr-cpg-4 protein	Locus_55_Transcript_1_0.889_2,Locus_55_Transcript_2_0.889_2	99.80%
**Locus_5598_Transcript_1/1_Confidence_1.000_6**	elegans protein confirmed by transcript evidence	Locus_5598_Transcript_1_1.000_6	99.80%
**Locus_6242_Transcript_1/1_Confidence_1.000_6**	briggsae cbr-gst- protein	Locus_6242_Transcript_1_1.000_6	100.00%
**Locus_7093_Transcript_1/1_Confidence_1.000_2**	---NA---	Locus_7093_Transcript_1_1.000_2	99.80%
**Locus_7740_Transcript_1/1_Confidence_1.000_4**	metalloprotease 1 precursor	Locus_7740_Transcript_1_1.000_4	100.00%

**Locus_8012_Transcript_1/1_Confidence_1.000_4**	fatty acid binding protein 4	Locus_8012_Transcript_1_1.000_4	100.00%
**Locus_85_Transcript_3/3_Confidence_0.714_2**	elegans protein confirmed by transcript evidence	Locus_85_Transcript_3_0.714_2	99.80%
**Locus_9275_Transcript_1/1_Confidence_1.000_6**	heat-responsive protein 12	Locus_9275_Transcript_1_1.000_6	99.80%
**Locus_97_Transcript_14/17_Confidence_0.321_5**	secreted protein 5 precursor	Locus_97_Transcript_14_0.321_5,Locus_97_Transcript_15_0.377_5,Locus_97_Transcript_17_0.321_5,Locus_97_Transcript_8_0.264_4	99.80%
**Locus_9926_Transcript_1/2_Confidence_1.000_1**	adenosine deaminase	Locus_9926_Transcript_1_1.000_1,Locus_9926_Transcript_2_1.000_1	99.80%
**Locus_999_Transcript_3/3_Confidence_0.400_2**	profilin iii	Locus_999_Transcript_3_0.400_2	100.00%
**Locus_1034_Transcript_1/1_Confidence_1.000_2**	aspartyl protease family member (asp-2)	Locus_1034_Transcript_1_1.000_2	99.80%
**Locus_11179_Transcript_2/3_Confidence_0.600_2**	elegans protein confirmed by transcript evidence	Locus_11179_Transcript_2_0.600_2, Locus_11179_Transcript_3_0.600_2	99.80%
**Locus_1253_Transcript_1/2_Confidence_1.000_4**	---NA---	Locus_1253_Transcript_1_1.000_4,Locus_1253_Transcript_2_1.000_4	99.80%
**Locus_1302_Transcript_1/2_Confidence_1.000_3**	---NA---	Locus_1302_Transcript_1_1.000_3	99.80%
**Locus_13077_Transcript_1/1_Confidence_1.000_4**	glutathione s-transferase	Locus_13077_Transcript_1_1.000_4	99.80%
**Locus_1316_Transcript_1/1_Confidence_1.000_3**	protein disulfide isomerase	Locus_1316_Transcript_1_1.000_3	99.80%

**Locus_14802_Transcript_1/2_Confidence_1.000_2**	aminopeptidase n	Locus_14802_Transcript_1_1.000_2, Locus_14802_Transcript_2_1.000_3	99.80%
**Locus_15246_Transcript_1/1_Confidence_1.000_5**	secreted protein asp-2	Locus_15246_Transcript_1_1.000_5	99.80%
**Locus_15549_Transcript_1/1_Confidence_1.000_5**	acylamino-acid-releasing enzyme	Locus_15549_Transcript_1_1.000_5	99.90%
**Locus_158_Transcript_1/3_Confidence_0.727_2**	briggsae cbr-cpg-2 protein	Locus_158_Transcript_1_0.727_2,Locus_158_Transcript_2_0.636_1,Locus_158_Transcript_3_0.818_1	99.80%
**Locus_2192_Transcript_1/1_Confidence_1.000_5**	profilin	Locus_2192_Transcript_1_1.000_5	99.80%
**Locus_220_Transcript_1/2_Confidence_1.000_4**	legumain	Locus_220_Transcript_1_1.000_4,Locus_220_Transcript_2_1.000_4	99.80%
**Locus_2510_Transcript_1/1_Confidence_1.000_5**	elegans protein partially confirmed by transcript evidence	Locus_2510_Transcript_1_1.000_5	99.80%
**Locus_2529_Transcript_1/1_Confidence_1.000_1**	aspartyl protease family member (asp-2)	Locus_2529_Transcript_1_1.000_1	99.80%
**Locus_2535_Transcript_1/1_Confidence_1.000_4**	elegans protein partially confirmed by transcript evidence	Locus_2535_Transcript_1_1.000_4	99.80%
**Locus_2709_Transcript_1/1_Confidence_1.000_3**	secreted protein 5 precursor	Locus_2709_Transcript_1_1.000_3	99.80%
**Locus_2717_Transcript_1/1_Confidence_1.000_3**	---NA---	Locus_2717_Transcript_1_1.000_3	99.80%
**Locus_2756_Transcript_1/1_Confidence_1.000_5**	immunogenic protein 3	Locus_2756_Transcript_1_1.000_5	99.80%
**Locus_2825_Transcript_1/1_Confidence_1.000_6**	elegans protein confirmed by transcript evidence	Locus_2825_Transcript_1_1.000_6	99.80%
**Locus_3574_Transcript_1/1_Confidence_1.000_4**	---NA---	Locus_3574_Transcript_1_1.000_4	99.80%
**Locus_3751_Transcript_1/1_Confidence_1.000_2**	sxc2 protein	Locus_3751_Transcript_1_1.000_2	99.80%

**Locus_3946_Transcript_1/1_Confidence_1.000_6**	briggsae cbr-fum-1 protein	Locus_3946_Transcript_1_1.000_6	99.80%
**Locus_3970_Transcript_1/1_Confidence_1.000_2**	macrophage migration inhibitory factor	Locus_3970_Transcript_1_1.000_2	99.80%
**Locus_4193_Transcript_1/1_Confidence_1.000_2**	glutamate dehydrogenase	Locus_4193_Transcript_1_1.000_2	99.80%
**Locus_421_Transcript_1/2_Confidence_1.000_5**	elegans protein confirmed by transcript evidence	Locus_421_Transcript_1_1.000_5,Locus_421_Transcript_2_1.000_5	99.80%
**Locus_4977_Transcript_1/1_Confidence_1.000_4**	elegans protein confirmed by transcript evidence	Locus_4977_Transcript_1_1.000_4	99.80%
**Locus_53_Transcript_1/1_Confidence_1.000_6**	c-type lectin-1	Locus_53_Transcript_1_1.000_6	99.80%
**Locus_606_Transcript_1/1_Confidence_1.000_3**	hypothetical protein F48E3.4 [Caenorhabditis elegans]	Locus_606_Transcript_1_1.000_3	99.80%
**Locus_6800_Transcript_1/1_Confidence_1.000_1**	glutathione s-transferase	Locus_6800_Transcript_1_1.000_1	99.80%

**Locus_731_Transcript_1/3_Confidence_0.667_4**	fumarate hydratase	Locus_731_Transcript_1_0.667_4,Locus_731_Transcript_2_0.667_4,Locus_731_Transcript_3_0.667_4	99.80%
**Locus_8097_Transcript_2/3_Confidence_0.750_1**	metalloprotease 1 precursor	Locus_8097_Transcript_2_0.750_1	99.80%
**Locus_883_Transcript_1/2_Confidence_1.000_4**	hypothetical protein F42A10.7 [Caenorhabditis elegans]	Locus_883_Transcript_1_1.000_4,Locus_883_Transcript_2_1.000_4	99.80%
**Locus_902_Transcript_1/1_Confidence_1.000_4**	metalloprotease 1 precursor	Locus_902_Transcript_1_1.000_4	99.80%
**Locus_9573_Transcript_2/3_Confidence_0.333_6**	suppression of tumorigenicity 14 (colon carcinoma)	Locus_9573_Transcript_2_0.333_6	100.00%

Number of total spectra	Number of unique peptides	Percentage of total spectra	Percentage sequence coverage	Blastx Closest Hit description	Specie
132	19	3.96%	56.70%	two-domain activation associated secreted protein ASP4 precursor	Ostertagia ostertagi
82	5	2.46%	34.60%	Globin-like host-protective antigen	Trichostrongylus colubriformis
74	6	2.22%	46.40%	secreted protein 4 precursor	Ancylostoma caninum
73	10	2.19%	45.80%	Globin-like host-protective antigen	Trichostrongylus colubriformis
53	11	1.59%	52.00%	secreted protein 6 precursor	Ancylostoma caninum
47	7	1.41%	45.20%	Globin-like host-protective antigen	Trichostrongylus colubriformis
46	10	1.38%	50.90%	C. briggsae CBR-VIT-6 protein	Caenorhabditis briggsae
46	8	1.38%	45.10%	LYSozyme family member (lys-8)	Caenorhabditis elegans
45	10	1.35%	50.80%	VITellogenin structural genes (yolk protein genes) family member (vit-1)	Caenorhabditis elegans
44	17	1.32%	40.90%	DVA-1 polyprotein	Dictyocaulus viviparus
43	10	1.29%	47.80%	VITellogenin structural genes (yolk protein genes) family member (vit-2)	Caenorhabditis elegans
41	7	1.23%	36.30%	VITellogenin structural genes (yolk protein genes) family member (vit-4)	Caenorhabditis elegans
35	4	1.05%	31.10%	C. briggsae CBR-VIT-2 protein	Caenorhabditis briggsae
30	7	0.90%	40.20%	Vitellogenin-6	Oscheius sp. (strain CEW1)

28	7	0.84%	51.30%	Vitellogenin-6	Oscheius sp. (strain CEW1)
27	7	0.81%	41.00%	Globin-like host-protective antigen	Trichostrongylus colubriformis
27	2	0.81%	46.80%	ancylostoma-secreted protein 1 precursor	Ancylostoma duodenale
25	2	0.75%	23.20%		
23	7	0.69%	44.70%	secreted protein 5 precursor	Ancylostoma caninum
23	3	0.69%	28.40%	Vitellogenin-6	Oscheius sp. (strain CEW1)
23	14	0.69%	42.80%	putative neuromuscular acetylcholinesterase	Dictyocaulus viviparus
21	8	0.63%	37.50%	secreted protein 4 precursor	Ancylostoma caninum
21	11	0.63%	36.70%	AF132291_1ancylostoma-secreted protein 1 precursor	Ancylostoma caninum
19	3	0.57%	44.50%	Myoglobin	Nippostrongylus brasiliensis
19	3	0.57%	27.10%	C. briggsae CBR-RPL-6 protein	Caenorhabditis briggsae
18	3	0.54%	34.70%	C. briggsae CBR-VIT-6 protein	Caenorhabditis briggsae
17	12	0.51%	48.10%	calcium-activated apyrase	Teladorsagia circumcincta
16	5	0.48%	42.10%	AF305957_1vitellogenin	Haemonchus contortus
15	6	0.45%	50.90%	two-domain activation associated secreted protein ASP4 precursor	Ostertagia ostertagi

15	12	0.45%	37.70%	enolase	Haemonchus contortus
14	4	0.42%	27.70%	secreted protein 4 precursor	Ancylostoma caninum
14	2	0.42%	23.60%		
14	5	0.42%	73.50%		
14	5	0.42%	22.90%	secreted-protein 1 precursor	Ancylostoma ceylanicum
13	8	0.39%	17.80%	DVA-1 polyprotein	Dictyocaulus viviparus
13	5	0.39%	28.60%	C. briggsae CBR-TTR-19 protein	Caenorhabditis briggsae
13	2	0.39%	18.50%	secreted protein 5 precursor	Ancylostoma ceylanicum
13	8	0.39%	53.70%	C. briggsae CBR-TTR-15 protein	Caenorhabditis briggsae
12	2	0.36%	26.30%	VITellogenin structural genes (yolk protein genes) family member (vit-2)	Caenorhabditis elegans
12	4	0.36%	35.80%		
12	10	0.36%	40.00%	C. briggsae CBR-NEX-1 protein	Caenorhabditis briggsae
12	3	0.36%	26.40%	C. briggsae CBR-VIT-6 protein	Caenorhabditis briggsae
12	5	0.36%	15.30%	Ancylostoma secreted protein	Ancylostoma caninum
12	3	0.36%	20.60%	parasitic stage specific protein 2	Haemonchus contortus
12	2	0.36%	34.60%	C. briggsae CBR-VIT-6 protein	Caenorhabditis briggsae
12	6	0.36%	40.60%	AF273705_1zinc metallopeptidase 2 MEP2	Ancylostoma caninum
11	2	0.33%	70.60%		Ancylostoma caninum
11	2	0.33%	27.50%	Vitellogenin-6	Oscheius sp. (strain CEW1)
11	7	0.33%	25.30%	putative Lipid Binding Protein	Angiostrongylus cantonensis

11	10	0.33%	20.70%	neuronal acetylcholinesterase precursor	Dictyocaulus viviparus
11	7	0.33%	22.80%	14-3-3 protein isoform 2	Ancylostoma caninum
10	9	0.30%	26.30%	C. briggsae CBR-LEC-5 protein	Caenorhabditis briggsae
10	8	0.30%	33.90%	hypothetical protein F01F1.12	Caenorhabditis elegans
10	7	0.30%	26.60%	putative ES protein F7	Ostertagia ostertagi
10	5	0.30%	23.80%		
10	5	0.30%	18.40%	putative zinc metallopeptidase	Haemonchus contortus
10	5	0.30%	76.10%	secreted protein 6 precursor	Ancylostoma caninum
9	4	0.27%	30.80%	VITellogenin structural genes (yolk protein genes) family member (vit-4)	Caenorhabditis elegans
9	2	0.27%	47.80%		
9	4	0.27%	32.50%		
9	8	0.27%	55.80%	Glutathione Transferase-2	Heligmosomoides polygyrus
9	4	0.27%	43.70%		
9	2	0.27%	20.20%	AF305957_1vitellogenin	Haemonchus contortus
9	8	0.27%	30.40%	Hypothetical protein CBG06352	Caenorhabditis briggsae
9	8	0.27%	19.70%	putative zinc metallopeptidase	Haemonchus contortus
9	4	0.27%	26.80%	secreted protein 6 precursor	Ancylostoma caninum

9	2	0.27%	40.80%	put. vitellogenin	Caenorhabditis elegans
8	7	0.24%	14.00%	C. briggsae CBR-HSP-60 protein	Caenorhabditis briggsae
8	5	0.24%	28.00%	C4b-binding protein alpha chain precursor	Gallus gallus
8	6	0.24%	16.20%	ancylostoma-secreted protein 1 precursor	Ancylostoma duodenale
8	2	0.24%	54.70%	secreted protein 6 precursor	Ancylostoma caninum
8	6	0.24%	31.70%		
8	5	0.24%	34.10%		
8	2	0.24%	48.00%		
8	2	0.24%	20.60%	Hypothetical protein CBG03380	Caenorhabditis briggsae
7	4	0.21%	13.70%	novel protein containing multiple sushi domains (SCR repeat)	Danio rerio
7	2	0.21%	38.00%	VITellogenin structural genes (yolk protein genes) family member (vit-6)	Caenorhabditis elegans
7	2	0.21%	16.70%		
7	5	0.21%	21.00%	translationally controlled tumor protein	Ostertagia ostertagi
7	5	0.21%	34.10%		
7	5	0.21%	17.30%		

7	4	0.21%	10.30%	hypothetical protein	Angiostrongylus cantonensis
7	4	0.21%	26.50%	nippocystatin	Nippostrongylus brasiliensis
7	4	0.21%	17.50%	GJ10405	Drosophila virilis
7	5	0.21%	45.10%	hypothetical protein LOAG_12508	Loa loa
7	2	0.21%	35.00%		
7	6	0.21%	18.60%	secreted-protein 1 precursor	Ancylostoma ceylanicum
7	7	0.21%	38.90%	calcium-activated apyrase	Teladorsagia circumcincta
6	3	0.18%	20.50%	Myoglobin	Nippostrongylus brasiliensis
6	2	0.18%	24.40%		
6	5	0.18%	26.40%	C. briggsae CBR-LYS-8 protein	Caenorhabditis briggsae
6	5	0.18%	12.20%	hypothetical protein M60.2	Caenorhabditis elegans
6	4	0.18%	19.00%	ACTin family member (act-4)	Caenorhabditis elegans
6	6	0.18%	26.70%	cysteine proteinase	Ancylostoma ceylanicum
6	4	0.18%	13.50%	TransThyretin-Related family domain family member (ttr-44)	Caenorhabditis elegans
6	5	0.18%	10.90%		
6	3	0.18%	18.90%		
6	3	0.18%	30.70%		

6	4	0.18%	19.40%	peroxiredoxin	Haemonchus contortus
6	4	0.18%	51.10%		
6	3	0.18%	20.20%	Myoglobin	Nippostrongylus brasiliensis
6	3	0.18%	9.03%		
6	2	0.18%	20.80%		
6	2	0.18%	26.30%	hypothetical protein K02F3.9	Caenorhabditis elegans
6	4	0.18%	11.30%	secreted protein 4 precursor	Ancylostoma caninum
5	2	0.15%	21.00%	VITellogenin structural genes (yolk protein genes) family member (vit-6)	Caenorhabditis elegans
5	2	0.15%	16.30%	secreted protein 5 precursor	Ancylostoma ceylanicum
5	4	0.15%	6.10%	Synthetic secondary siRNA-deficient ArGOnaute mutant family member (sago-2)	Caenorhabditis elegans
5	4	0.15%	19.10%	calcium-dependent apyrase	Ostertagia ostertagi
5	4	0.15%	10.10%	c-type lectin 1	Ancylostoma caninum
5	5	0.15%	17.80%	Aspartyl protease inhibitor	Ostertagia ostertagi

5	2	0.15%	9.20%	hypothetical protein LOAG_03532	Loa loa
5	4	0.15%	7.79%	astacin-like metalloprotease	Haemonchus contortus
5	4	0.15%	11.90%	Hypothetical protein CBG18957	Caenorhabditis briggsae
5	4	0.15%	13.90%	putative retinol-binding protein	Heligmosomoides polygyrus
5	4	0.15%	4.98%	hypothetical protein T25C12.3	Caenorhabditis elegans
5	2	0.15%	30.60%	hypothetical protein ZC373.2	Caenorhabditis elegans
5	2	0.15%	10.80%	hypothetical protein Y52B11A.8	Caenorhabditis elegans
5	5	0.15%	19.20%	calcium-activated apyrase	Teladorsagia circumcincta
5	2	0.15%	16.30%	putative retinol-binding protein	Heligmosomoides polygyrus
5	3	0.15%	28.40%		
5	4	0.15%	43.30%		
5	4	0.15%	17.60%	C. briggsae CBR-TPI-1 protein	Caenorhabditis briggsae
4	3	0.12%	9.82%	cyclophilin-type peptidyl-prolyl cis-trans isomerase-15, Bmcyp-5	Brugia malayi
4	2	0.12%	11.90%	excretory/secretory antigen	Ostertagia ostertagi

4	3	0.12%	37.20%		
4	2	0.12%	25.80%		
4	2	0.12%	15.60%	hypothetical protein F58B4.4	Caenorhabditis elegans
4	4	0.12%	17.40%	metalloprotease 1 precursor	Ancylostoma ceylanicum
4	4	0.12%	29.70%	hypothetical protein F40A3.3	Caenorhabditis elegans
4	2	0.12%	17.40%	Nematode AStacin protease family member (nas-5)	Caenorhabditis elegans
4	4	0.12%	13.40%	C. briggsae CBR-RNR-2 protein	Caenorhabditis briggsae
4	2	0.12%	14.10%		
4	2	0.12%	7.01%	C. elegans protein F54F11.2b, partially confirmed by transcript evidence	Caenorhabditis elegans
4	3	0.12%	39.40%	Nematode AStacin protease family member (nas-5)	Caenorhabditis elegans

4	2	0.12%	13.50%	C. briggsae CBR-LYS-8 protein	Caenorhabditis briggsae
4	3	0.12%	29.70%		
4	2	0.12%	22.00%	C. briggsae CBR-TTR-15 protein	Caenorhabditis briggsae
4	3	0.12%	9.35%	enolase	Haemonchus contortus
4	3	0.12%	30.40%	Kunitz type serine protease inhibitor	Ancylostoma ceylanicum
4	3	0.12%	13.90%	Invertebrate LYSozyme family member (ilys-5)	Caenorhabditis elegans
4	4	0.12%	27.10%		
4	4	0.12%	28.20%	two-domain activation associated secreted protein ASP4 precursor	Ostertagia ostertagi
4	4	0.12%	24.00%	AF273084_1zinc metallopeptidase 1	Ancylostoma caninum
4	4	0.12%	31.70%	hypothetical protein M163.8	Caenorhabditis elegans
3	3	0.09%	4.72%	protein disulfide isomerase	Teladorsagia circumcincta
3	2	0.09%	23.70%	Textilinin-2	Pseudonaja textilis

3	3	0.09%	31.00%		
3	3	0.09%	29.70%	putative L3 ES protein	Ostertagia ostertagi
3	2	0.09%	5.77%	Heat Shock Protein family member (hsp-1)	Caenorhabditis elegans
3	2	0.09%	20.30%	major sperm protein	Dictyocaulus viviparus
3	3	0.09%	17.20%	Dipeptidyl Peptidase Four (IV) family member (dpf-6)	Caenorhabditis elegans
3	2	0.09%	28.70%		
3	2	0.09%	17.10%	hypothetical protein F25H2.5	Caenorhabditis elegans
3	2	0.09%	19.80%		
3	3	0.09%	24.50%	Fatty Acid/Retinol binding protein family member (far-8)	Caenorhabditis elegans
3	2	0.09%	39.10%	excretory/secretory antigen	Ostertagia ostertagi
3	2	0.09%	33.30%		
3	3	0.09%	9.43%	secreted protein 4 precursor	Ancylostoma caninum

3	2	0.09%	24.20%	Nematode AStacin protease family member (nas-5)	Caenorhabditis elegans
3	3	0.09%	19.90%	FerriTiN family member (ftn-2)	Caenorhabditis elegans
3	3	0.09%	11.10%	PREDICTED: mannose receptor C type 1-like	Saccoglossus kowalevskii
3	3	0.09%	31.40%	hypothetical protein T25B6.2	Caenorhabditis elegans
3	3	0.09%	21.70%	Hypothetical protein CBG06772	Caenorhabditis briggsae
3	2	0.09%	11.90%	Hypothetical protein CBG13025	Caenorhabditis briggsae
3	2	0.09%	8.61%	hypothetical protein T19B10.2	Caenorhabditis elegans
3	3	0.09%	35.80%	glutathione S-transferase-3	Necator americanus
3	2	0.09%	18.90%		
3	3	0.09%	9.79%	astacin-like metalloprotease	Haemonchus contortus

3	3	0.09%	36.80%	Lipid Binding Protein family member (lbp-9)	Caenorhabditis elegans
3	2	0.09%	12.10%	C. briggsae CBR-CYN-3 protein	Caenorhabditis briggsae
3	2	0.09%	12.60%	Hypothetical protein CBG24261	Caenorhabditis briggsae
3	2	0.09%	8.06%	secreted protein 5 precursor	Ancylostoma ceylanicum
3	2	0.09%	7.01%	hypothetical protein C06G3.5	Caenorhabditis elegans
3	3	0.09%	16.90%	C. briggsae CBR-PFN-1 protein	Caenorhabditis briggsae
2	2	0.06%	5.91%	parasite pepsinogen	Haemonchus contortus
2	2	0.06%	10.40%	putative MFP2	Angiostrongylus cantonensis
2	2	0.06%	11.80%		
2	2	0.06%	7.01%		
2	2	0.06%	31.20%	glutathione S-transferase 2	Heligmosomoides polygyrus
2	2	0.06%	4.56%	protein disulfide isomerase 1	Ostertagia ostertagi

2	2	0.06%	7.71%	antigen h11	Haemonchus contortus
2	2	0.06%	21.40%	two-domain activation associated secreted protein ASP4 precursor	Ostertagia ostertagi
2	2	0.06%	9.91%	Dipeptidyl Peptidase Four (IV) family member (dpf-6)	Caenorhabditis elegans
2	2	0.06%	15.70%	Hypothetical protein CBG05204	Caenorhabditis briggsae
2	2	0.06%	23.50%	ProFiliN family member (pfn-1)	Caenorhabditis elegans
2	2	0.06%	7.93%	legumain	Haemonchus contortus
2	2	0.06%	13.40%	AF273705_1zinc metallopeptidase 2 MEP2	Ancylostoma caninum
2	2	0.06%	14.50%	parasite pepsinogen	Haemonchus contortus
2	2	0.06%	2.96%	Hypothetical protein CBG20766	Caenorhabditis briggsae
2	2	0.06%	14.00%	ancylostoma-secreted protein 1 precursor	Ancylostoma duodenale
2	2	0.06%	13.40%		
2	2	0.06%	22.50%	hypothetical protein ZK856.7	Caenorhabditis elegans
2	2	0.06%	11.60%	C. briggsae CBR-LEC-4 protein	Caenorhabditis briggsae
2	2	0.06%	12.60%		
2	2	0.06%	42.20%	SXC2 protein	Ostertagia ostertagi

2	2	0.06%	10.90%	C. briggsae CBR-FUM-1 protein	Caenorhabditis briggsae
2	2	0.06%	20.60%	macrophage migration inhibitory factor	Ancylostoma ceylanicum
2	2	0.06%	4.74%	glutamate dehydrogenase	Haemonchus contortus
2	2	0.06%	14.80%	TransThyretin-Related family domain family member (ttr-2)	Caenorhabditis elegans
2	2	0.06%	6.07%	hypothetical protein C04G2.9	Caenorhabditis elegans
2	2	0.06%	12.60%	C-type lectin-1	Heligmosomoides polygyrus
2	2	0.06%	12.10%	hypothetical protein F48E3.4	Caenorhabditis elegans
2	2	0.06%	11.40%	glutathione S-transferase-3	Necator americanus

2	2	0.06%	15.40%	FUMarase family member (fum-1)	Caenorhabditis elegans
2	2	0.06%	6.24%	metalloprotease 1 precursor	Ancylostoma ceylanicum
2	2	0.06%	37.00%	hypothetical protein F42A10.7	Caenorhabditis elegans
2	2	0.06%	21.10%	metalloprotease 1 precursor	Ancylostoma ceylanicum
2	2	0.06%	13.50%	PREDICTED: similar to epithin	Monodelphis domestica

Accession Number	Closest Blastx Hit eValue	#GOs (Annotated)	GOs (Annotated)	#GOs (when mapped but not annotated)	Mapped but not annotated	Enzyme Codes	InterProScan
CAO00417.1	1.957E-89	0		3	P:GO:0040011; P:GO:0006898; C:GO:0005576		IPR001283; IPR002413; IPR014044; IPR018244; PTHR10334:SF11 (PANTHER)
P27613.1	2.936E-30	6	F:GO:0020037; C:GO:0005576; C:GO:0005737; F:GO:0005344; P:GO:0015671; F:GO:0019825	-	-		IPR009548
AAO63576.1	3.028E-26	0		2	P:GO:0040011; C:GO:0005576		IPR001283; IPR014044; PTHR10334:SF11 (PANTHER)
P27613.1	3.102E-55	6	F:GO:0020037; C:GO:0005615; C:GO:0005737; F:GO:0005344; P:GO:0015671; F:GO:0019825	-	-		IPR000971; IPR009050; IPR012085; IPR012292; SignalP (SIGNALP)
AAO63578.1	8.137E-20	0		2	P:GO:0040011; C:GO:0005576		SignalP (SIGNALP)
P27613.1	3.518E-39	6	F:GO:0020037; C:GO:0005615; C:GO:0005737; F:GO:0005344; P:GO:0015671; F:GO:0019825	-	-		no IPS match
XP_002634040.1	6.073E-37	0		9	F:GO:0045735; F:GO:0005319; F:GO:0008565; P:GO:0006869; P:GO:0009792; C:GO:0005576; P:GO:0006886; P:GO:0008340; P:GO:0040010		no IPS match
NP_495083.1	4.647E-30	1	F:GO:0005515	-	-		IPR013781; IPR017853; PTHR23208 (PANTHER), PTHR23208:SF13 (PANTHER), SignalP (SIGNALP)
NP_509305.1	1.001E-45	4	P:GO:0009792; F:GO:0005515; P:GO:0040010; P:GO:0008340	-	-		IPR015255; IPR015819; PTHR23345 (PANTHER)
Q24702.1	2.08E-145	1	F:GO:0008289	-	-		PTHR21693 (PANTHER)
NP_001123117.1	3.69E-53	4	P:GO:0009792; F:GO:0005515; P:GO:0040010; P:GO:0008340	-	-		IPR001747; IPR015816; IPR015819; PTHR23345 (PANTHER)
NP_508612.1	3.144E-35	4	P:GO:0009792; F:GO:0005515; P:GO:0040010; P:GO:0008340	-	-		PTHR23345 (PANTHER)
XP_002644638.1	4.109E-32	4	P:GO:0009792; F:GO:0005515; P:GO:0040010; P:GO:0008340	-	-		IPR011030; IPR015255; IPR015817; IPR015819; PTHR23345 (PANTHER)
Q94637.1	8.07E-39	6	P:GO:0009792; F:GO:0005319; F:GO:0005515; P:GO:0040010; P:GO:0006869; P:GO:0008340	-	-		no IPS match

Q94637.1	5.987E-31	4	P:GO:0009792; F:GO:0005515; P:GO:0040010; P:GO:0008340	-	-		SignalP (SIGNALP)
P27613.1	4.944E-56	6	F:GO:0020037; C:GO:0005615; C:GO:0005737; F:GO:0005344; P:GO:0015671; F:GO:0019825	-	-		IPR000971; IPR009050; IPR012085; IPR012292; SignalP (SIGNALP)
AAD13339.1	0.0001661	0		1	C:GO:0005576		IPR014044
		0					no IPS match
AAO63577.1	2.788E-38	0		4	F:GO:0009055; P:GO:0019646; P:GO:0006898; C:GO:0005576		IPR001283; IPR014044; IPR018244; PTHR10334:SF11 (PANTHER)
Q94637.1	3.544E-15	6	P:GO:0009792; F:GO:0005515; P:GO:0006810; P:GO:0040010; P:GO:0033036; P:GO:0008340	-	-		SignalP (SIGNALP)
AAS49411.1	6.37E-169	5	P:GO:0001507; F:GO:0003990; C:GO:0005623; P:GO:0040012; F:GO:0042803	-	-	EC:3.1.1.7	IPR000997; IPR002018; IPR019826; G3DSA:3.40.50.1820 (GENE3D), PTHR11559:SF33 (PANTHER), SignalP (SIGNALP), SSF53474 (SUPERFAMILY)
AAO63576.1	2.5E-31	0		2	P:GO:0040011; C:GO:0005576		IPR001283; IPR002413; IPR014044; PTHR10334:SF11 (PANTHER)
AAD31839.1	4.282E-49	0		3	P:GO:0040011; P:GO:0006898; C:GO:0005576		IPR001283; IPR013032; IPR014044; IPR018244; PTHR10334:SF11 (PANTHER), SignalP (SIGNALP)
P51535.1	6.905E-58	6	F:GO:0020037; C:GO:0005576; C:GO:0005737; F:GO:0005344; P:GO:0015671; F:GO:0019825	-	-		IPR000971; IPR009050; IPR012085; IPR012292; SignalP (SIGNALP)
XP_002641923.1	1.076E-79	3	C:GO:0005840; F:GO:0003735; P:GO:0006412	-	-	EC:3.6.5.3	IPR001747; IPR011030; PTHR23345 (PANTHER), SSF48431 (SUPERFAMILY)
XP_002634040.1	8.945E-11	0		9	F:GO:0045735; F:GO:0005319; F:GO:0008565; P:GO:0006869; P:GO:0009792; C:GO:0005576; P:GO:0006886; P:GO:0008340; P:GO:0040010		IPR001747; IPR011030; SSF48431 (SUPERFAMILY)
CBW38507.1	2.36E-89	5	P:GO:0040010; P:GO:0008340; F:GO:0005515; F:GO:0016462; C:GO:0005789	-	-		no IPS match
AAL09375.1	2.41E-39	6	P:GO:0009792; F:GO:0005319; F:GO:0005515; P:GO:0040010; P:GO:0006869; P:GO:0008340	-	-		IPR015255
CAO00417.1	1.272E-17	0		2	P:GO:0040011; C:GO:0005576		IPR001283; IPR014044; PTHR10334:SF9 (PANTHER), SignalP (SIGNALP)

ADK47524.1	0	12	P:GO:0040010; P:GO:0008340; P:GO:0040035; C:GO:0000015; C:GO:0009986; P:GO:0002119; F:GO:0000287; P:GO:0006096; P:GO:0006898; P:GO:0009792; P:GO:0040018; F:GO:0004634	-	-	EC:4.2.1.11	IPR000941; IPR020809; IPR020810; G3DSA:3.20.20.120 (GENE3D), PTHR11902 (PANTHER), PS51257 (PROFILE), SSF51604 (SUPERFAMILY), SSF54826 (SUPERFAMILY)
AAO63576.1	5.012E-22	0		1	C:GO:0005576		IPR001283; IPR014044; PTHR10334:SF11 (PANTHER)
		0					SignalP (SIGNALP)
		0					no IPS match
AAN11402.1	4.525E-11	0		1	C:GO:0005576		IPR014044
Q24702.1	9.32E-133	2	P:GO:0008340; F:GO:0008289	-	-		PTHR21693 (PANTHER), SignalP (SIGNALP)
XP_002637058.1	5.239E-43	0					IPR001534; SignalP (SIGNALP)
ABB53347.1	4.151E-10	0		1	C:GO:0005576		IPR014044
XP_002641305.1	2.209E-33	0		1	P:GO:0016539		IPR001534; SignalP (SIGNALP)
NP_001123117.1	1.222E-07	1	F:GO:0005515	-	-		no IPS match
		0					no IPS match
XP_002642775.1	2.26E-128	4	C:GO:0044464; F:GO:0005544; F:GO:0005509; F:GO:0005515	-	-		IPR001464; IPR018252; IPR018502; PTHR10502:SF30 (PANTHER)
XP_002634040.1	1.562E-23	0		6	P:GO:0006869; F:GO:0005319; F:GO:0045735; P:GO:0006886; C:GO:0005576; F:GO:0008565		IPR001747; IPR015816; IPR015819
Q16937.1	1.551E-45	0		2	P:GO:0040011; C:GO:0005576		IPR001283; IPR014044; IPR018244; PTHR10334:SF11 (PANTHER)
ADN00784.1	1.054E-38	0					no IPS match
XP_002634040.1	2.235E-09	3	P:GO:0006810; F:GO:0022892; P:GO:0033036	-	-		no IPS match
AAG29105.1	3.599E-85	2	P:GO:0008152; F:GO:0008237	-	-		IPR000718; IPR008753; G3DSA:3.40.390.10 (GENE3D), PTHR11733:SF15 (PANTHER), SSF55486 (SUPERFAMILY)
AAO63578.1	0.0004719	0		1	C:GO:0005576		no IPS match
Q94637.1	4.359E-15	5	P:GO:0006869; F:GO:0005319; P:GO:0006886; F:GO:0008565; F:GO:0005515	-	-		IPR001747; IPR015816; PTHR23345 (PANTHER)
CAR63537.1	4.014E-58	1	F:GO:0005515	-	-		IPR000463; IPR011038; IPR012674; PTHR22725 (PANTHER), SignalP (SIGNALP)

AAO62949.1	3.93E-157	5	P:GO:0001507; F:GO:0003990; C:GO:0005623; P:GO:0040012; F:GO:0042803	-	-	EC:3.1.1.7	SignalP (SIGNALP)
ACO59962.1	4.14E-124	7	C:GO:0005737; F:GO:0019904; P:GO:0018991; P:GO:0040010; P:GO:0008340; P:GO:0006898; P:GO:0043053	-	-		IPR000308
XP_002630817.1	6.04E-142	3	F:GO:0005515; F:GO:0005529; P:GO:0000003	-	-		IPR001079; IPR008985; IPR013320; PTHR11346:SF29 (PANTHER), SignalP (SIGNALP)
NP_001021240.1	6.04E-167	7	F:GO:0042802; P:GO:0009792; P:GO:0018991; P:GO:0040010; P:GO:0040011; P:GO:0006096; F:GO:0004332	-	-	EC:4.1.2.13	IPR000741; IPR013785; SSF51569 (SUPERFAMILY)
CAD20464.1	1.781E-64	4	C:GO:0005576; F:GO:0005504; F:GO:0005515; F:GO:0019841	-	-		IPR008632; SignalP (SIGNALP)
		0					IPR000436; SignalP (SIGNALP)
AAC31568.1	2.832E-66	2	P:GO:0008152; F:GO:0008237	-	-		IPR000718; IPR008753; G3DSA:1.10.1380.10 (GENE3D), PTHR11733:SF15 (PANTHER), SignalP (SIGNALP), SSF55486 (SUPERFAMILY)
AAO63578.1	5.818E-10	0		2	P:GO:0040011; C:GO:0005576		IPR014044
NP_508612.1	2.586E-15	4	P:GO:0009792; F:GO:0005515; P:GO:0040010; P:GO:0008340	-	-		IPR001846; PTHR11339 (PANTHER), PTHR11339:SF43 (PANTHER)
		0					IPR000276
		0					no IPS match
1TW9	1.13E-101	4	P:GO:0008340; P:GO:0040010; F:GO:0005515; F:GO:0016740	-	-		IPR004045; IPR004046; IPR010987; IPR012335; IPR012336; IPR017933; PTHR11571 (PANTHER), PTHR11571:SF6 (PANTHER)
		0					SignalP (SIGNALP)
AAL09375.1	3.665E-30	6	P:GO:0006869; F:GO:0005319; P:GO:0009792; P:GO:0008340; P:GO:0040010; F:GO:0005515	-	-		IPR000372
XP_002647311.1	8.59E-37	2	F:GO:0005488; P:GO:0010955	-	-		IPR000215; G3DSA:2.30.39.10 (GENE3D), G3DSA:3.30.497.10 (GENE3D)
AAC28740.1	2.64E-130	1	F:GO:0003824	-	-		IPR000718; IPR008753; G3DSA:1.10.1380.10 (GENE3D), PTHR11733:SF15 (PANTHER), SignalP (SIGNALP), SSF55486 (SUPERFAMILY)
AAO63578.1	3.439E-08	0		2	P:GO:0040011; C:GO:0005576		IPR001283; IPR014044

CAA26849.1	9.487E-13	4	P:GO:0009792; P:GO:0008340; P:GO:0040010; F:GO:0005515	-	-		IPR011030; SignalP (SIGNALP)
XP_002640959.1	0	55	C:GO:0009986; P:GO:0032755; F:GO:0051087; C:GO:0046696; P:GO:0042026; P:GO:0032735; P:GO:0032729; C:GO:0005791; F:GO:0051082; P:GO:0043032; P:GO:0042542; P:GO:0032733; P:GO:0043065; C:GO:0005829; F:GO:0001530; P:GO:0032727; P:GO:0051085; C:GO:0005759; C:GO:0005615; C:GO:0045121; F:GO:0002039; P:GO:0042100; P:GO:0006986; F:GO:0002020; P:GO:0001666; P:GO:0006919; C:GO:0044459; F:GO:0043559; P:GO:0002755; C:GO:0019907; P:GO:0042220; P:GO:0002842; P:GO:0009408; C:GO:0042588; F:GO:0032403; C:GO:0005769; C:GO:0005794; P:GO:0043627; F:GO:0005524; F:GO:0051787; F:GO:0043498; P:GO:0045768; P:GO:0002368; F:GO:0046982; P:GO:0050821; P:GO:0050729; P:GO:0050870; C:GO:0030135; P:GO:0042493; C:GO:0030061; C:GO:0005905; P:GO:0048291; P:GO:0002236; P:GO:0032496; P:GO:0033198	-	-		SignalP (SIGNALP)
NP_001028814.1	0.0002535	0					IPR000436; IPR016060; PTHR19325 (PANTHER), PTHR19325:SF51 (PANTHER), SignalP (SIGNALP)
AAD13339.1	0.0004799	0		1	C:GO:0005576		IPR014044; SignalP (SIGNALP)
AAO63578.1	4.77E-20	0		2	P:GO:0040011; C:GO:0005576		SignalP (SIGNALP)
		0					SignalP (SIGNALP)
		0					SignalP (SIGNALP)
		0					no IPS match
XP_002633697.1	5.245E-26	6	F:GO:0030976; C:GO:0016021; P:GO:0009792; P:GO:0008152; F:GO:0000287; F:GO:0016740	-	-		IPR014044; SignalP (SIGNALP)
CAP09614.1	1.093E-05	0					IPR000436; IPR016060; SignalP (SIGNALP)
NP_001023275.1	3.859E-06	0		6	P:GO:0006869; F:GO:0005319; F:GO:0045735; P:GO:0006886; C:GO:0005576; F:GO:0008565		IPR015819; SignalP (SIGNALP)
		0					no IPS match
CAT00091.1	2.808E-51	8	C:GO:0005737; P:GO:0009792; P:GO:0002119; F:GO:0005515; P:GO:0000003; P:GO:0040011; P:GO:0040007; P:GO:0006898	-	-		no IPS match
		0					SignalP (SIGNALP)
		0					IPR000436; IPR016060; SignalP (SIGNALP)

CAR63643.1	5.697E-23	0					no IPS match
BAB59011.1	1.776E-50	2	F:GO:0004869; P:GO:0000003	-	-		no IPS match
XP_002056110.1	1.456E-12	0		7	P:GO:0006508; F:GO:0004252; F:GO:0008236; P:GO:0006911; F:GO:0016787; F:GO:0003824; F:GO:0008233		no IPS match
EFO15999.1	3.943E-06	0		1	P:GO:0016539		IPR001534; SignalP (SIGNALP)
		0					SignalP (SIGNALP)
AAN11402.1	1.861E-23	0		3	P:GO:0040011; P:GO:0006898; C:GO:0005576		IPR001283; IPR014044; SignalP (SIGNALP)
CBW38507.1	1.866E-82	8	P:GO:0040010; P:GO:0008340; F:GO:0004871; P:GO:0043123; F:GO:0017110; F:GO:0005515; P:GO:0008152; C:GO:0005789	-	-	EC:3.6.1.6	IPR009283
P51535.1	1.953E-64	6	F:GO:0020037; C:GO:0005615; C:GO:0005737; F:GO:0005344; P:GO:0015671; F:GO:0019825	-	-		IPR009050; IPR012292
		0					SignalP (SIGNALP)
XP_002630755.1	4.177E-60	4	F:GO:0005515; P:GO:0008340; P:GO:0008152; F:GO:0003824	-	-		IPR013781; IPR017853; PTHR23208 (PANTHER), PTHR23208:SF13 (PANTHER), SignalP (SIGNALP)
NP_509391.1	1.18E-124	4	F:GO:0005488; F:GO:0004519; P:GO:0019538; C:GO:0044424	-	-		IPR018998; PTHR12439 (PANTHER), SignalP (SIGNALP), SSF142877 (SUPERFAMILY)
NP_508841.1	0	18	P:GO:0007126; P:GO:0007108; P:GO:0040007; P:GO:0007517; P:GO:0000281; P:GO:0040035; P:GO:0030036; C:GO:0005865; F:GO:0005524; C:GO:0005938; P:GO:0002119; F:GO:0016887; F:GO:0005515; C:GO:0005884; F:GO:0005200; P:GO:0040039; P:GO:0006898; P:GO:0009792	-	-		IPR004000; IPR004001; IPR020902; G3DSA:2.30.36.70 (GENE3D), G3DSA:3.30.420.40 (GENE3D), G3DSA:3.90.640.10 (GENE3D), SSF53067 (SUPERFAMILY)
AAM82155.1	5.63E-102	7	P:GO:0009792; P:GO:0002119; F:GO:0008233; F:GO:0005515; P:GO:0040011; P:GO:0040007; P:GO:0006898	-	-		IPR000169; IPR000668; IPR013128; IPR015643; G3DSA:3.90.70.10 (GENE3D), SignalP (SIGNALP), SSF54001 (SUPERFAMILY)
NP_506352.1	2.061E-43	0					IPR001534; SignalP (SIGNALP)
		0					no IPS match
		0					no IPS match
		0					no IPS match

AAT28331.1	2.92E-102	13	P:GO:0032872; P:GO:0042744; F:GO:0005515; P:GO:0034101; P:GO:0042267; P:GO:0008283; P:GO:0019430; F:GO:0008379; P:GO:0045454; P:GO:0042345; P:GO:0055114; C:GO:0005634; C:GO:0005739	-	-		IPR000866; IPR012335; IPR012336; IPR017936; IPR019479; G3DSA:3.30.1020.10 (GENE3D), PTHR10681 (PANTHER), PTHR10681:SF8 (PANTHER)
		0					no IPS match
P51535.1	2.143E-71	6	F:GO:0020037; C:GO:0005615; C:GO:0005737; F:GO:0005344; P:GO:0015671; F:GO:0019825	-	-		
		0					SignalP (SIGNALP)
		0					SignalP (SIGNALP)
NP_497272.1	2.604E-26	0					no IPS match
AAO63576.1	5.229E-32	0		2	P:GO:0040011; C:GO:0005576		IPR001283; IPR002413; IPR013032; IPR014044; IPR018244; PTHR10334:SF11 (PANTHER), SignalP (SIGNALP)
NP_001023275.1	4.777E-09	0		7	P:GO:0006869; F:GO:0005319; P:GO:0006886; F:GO:0045735; F:GO:0005515; C:GO:0005576; F:GO:0008565		IPR015816
ABB53347.1	4.943E-17	0		3	P:GO:0040011; P:GO:0006898; C:GO:0005576		IPR014044
NP_490758.1	4.132E-96	0		3	F:GO:0003676; P:GO:0000003; P:GO:0016246		IPR003100; IPR003165; IPR012337; G3DSA:2.170.260.10 (GENE3D), PTHR22891 (PANTHER), SSF101690 (SUPERFAMILY)
ADG63133.1	2.898E-82	9	F:GO:0004871; P:GO:0043123; F:GO:0017110; C:GO:0044446; C:GO:0044425; C:GO:0031090; C:GO:0012505; C:GO:0005783; P:GO:0009191	-	-	EC:3.6.1.6	IPR009283
ADK91433.1	2.794E-42	0		27	F:GO:0001874; P:GO:0050766; F:GO:0001872; F:GO:0005529; P:GO:0016337; P:GO:0008037; P:GO:0032760; F:GO:0003674; F:GO:0030247; C:GO:0016021; P:GO:0006911; C:GO:0016020; P:GO:0006910; P:GO:0002238; F:GO:0030169; F:GO:0008329; F:GO:0004872; P:GO:0008150; P:GO:0006955; F:GO:0005488; P:GO:0007165; C:GO:0009897; P:GO:0001879; P:GO:0001878; C:GO:0005575; F:GO:0001846; P:GO:0002752		SignalP (SIGNALP)
Q95PP1.1	1.172E-70	3	C:GO:0005576; F:GO:0008233; F:GO:0019828	-	-		IPR010480; SignalP (SIGNALP), SSF55149 (SUPERFAMILY)

EFO24957.1	2.206E-51	0						no IPS match
CAJ43810.1	4.23E-167	2	F:GO:0046872; F:GO:0008237	-	-			IPR000859; IPR001506; IPR006026; IPR013032; G3DSA:3.40.390.10 (GENE3D), PTHR10127 (PANTHER), PTHR10127:SF65 (PANTHER), SSF55486 (SUPERFAMILY)
XP_002637274.1	6.6E-142	8	F:GO:0005488; C:GO:0005811; F:GO:0030060; P:GO:0006108; P:GO:0055114; P:GO:0006099; P:GO:0006096; C:GO:0005829	-	-	EC:1.1.1.37		IPR001236; IPR001252; IPR001557; IPR010945; IPR011274; IPR015955; IPR016040; IPR022383; SSF51735 (SUPERFAMILY)
AAK57805.1	1.061E-88	4	C:GO:0005576; F:GO:0005515; F:GO:0019842; F:GO:0005501	-	-			no IPS match
NP_509919.2	0	1	F:GO:0005488	-	-			IPR001304; IPR002035; IPR013032; IPR016186; IPR016187; G3DSA:3.40.50.410 (GENE3D), PTHR22802 (PANTHER), PTHR22802:SF6 (PANTHER), SSF53300 (SUPERFAMILY), SSF57196 (SUPERFAMILY)
NP_509687.1	2.41E-08	1	P:GO:0002119	-	-			no IPS match
NP_492859.1	1.506E-17	0		3	C:GO:0016021; P:GO:0016042; P:GO:0006644			IPR013090; IPR016090; SignalP (SIGNALP)
CBW38507.1	4.979E-89	9	P:GO:0040010; C:GO:0043231; P:GO:0008340; F:GO:0004871; P:GO:0043123; F:GO:0005515; C:GO:0044444; F:GO:0016787; C:GO:0016020	-	-			IPR009283; SignalP (SIGNALP)
AAK57805.1	8.964E-34	2	F:GO:0005515; F:GO:0008289	-	-			IPR008632; SignalP (SIGNALP)
		0						SignalP (SIGNALP)
		0						no IPS match
XP_002631798.1	1.05E-105	6	F:GO:0004807; P:GO:0044283; P:GO:0044249; P:GO:0032787; P:GO:0008610; P:GO:0006007	-	-	EC:5.3.1.1		IPR000652; IPR013785; IPR020861
XP_001896264.1	1.052E-66	7	P:GO:0006457; C:GO:0005788; F:GO:0005515; C:GO:0042470; P:GO:0051169; F:GO:0042277; F:GO:0003755	-	-	EC:5.2.1.8		IPR002130; IPR015891; IPR020892; PTHR11071 (PANTHER), SignalP (SIGNALP)
CAA86821.1	9.927E-74	1	F:GO:0019841	-	-			no IPS match

		0					IPR001283; IPR014044; PTHR10334:SF4 (PANTHER)
		0					SignalP (SIGNALP)
NP_505787.1	4.712E-10	0					SignalP (SIGNALP)
AAN11401.1	6.69E-139	2	F:GO:0046872; F:GO:0008237	-	-		IPR000859; IPR001506; IPR006026; IPR006210; IPR013032; G3DSA:3.40.390.10 (GENE3D), PTHR10127 (PANTHER), PTHR10127:SF65 (PANTHER), SSF55486 (SUPERFAMILY)
NP_001023903.1	2.272E-79	0					IPR001858; IPR008914; PTHR11362 (PANTHER), SignalP (SIGNALP)
NP_492616.1	1.362E-39	2	F:GO:0008233; P:GO:0007413	-	-		IPR001506; IPR006026; G3DSA:3.40.390.10 (GENE3D), PTHR10127 (PANTHER), PTHR10127:SF73 (PANTHER), SSF55486 (SUPERFAMILY)
XP_002642568.1	1.05E-156	9	C:GO:0005737; P:GO:0009263; P:GO:0006260; F:GO:0046914; F:GO:0005515; P:GO:0055114; P:GO:0051290; P:GO:0009186; F:GO:0004748	-	-	EC:1.17.4.1	IPR000358; IPR009078; IPR012348
		0					no IPS match
CAR97838.1	3.49E-141	10	P:GO:0009056; P:GO:0048856; P:GO:0016486; F:GO:0004222; P:GO:0006508; C:GO:0044444; P:GO:0007275; F:GO:0042803; P:GO:0044237; C:GO:0016020	-	-	EC:3.4.24.0	IPR000718; IPR008753; IPR018497; G3DSA:3.40.390.10 (GENE3D), PTHR11733:SF15 (PANTHER), SSF55486 (SUPERFAMILY)
NP_492616.1	4.28E-21	2	F:GO:0016787; P:GO:0007413	-	-		IPR001506; IPR006026; G3DSA:3.40.390.10 (GENE3D), PTHR10127 (PANTHER), PTHR10127:SF73 (PANTHER), SSF55486 (SUPERFAMILY)

XP_002630755.1	7.76E-53	7	P:GO:0009056; P:GO:0005975; F:GO:0043169; F:GO:0005515; P:GO:0008340; F:GO:0003796; P:GO:0043170	-	-	EC:3.2.1.17	IPR013781; IPR017853; PTHR23208 (PANTHER), PTHR23208:SF13 (PANTHER)
		0					no IPS match
XP_002641305.1	1.838E-24	0		1	P:GO:0016539		IPR001534; SignalP (SIGNALP)
ADK47524.1	0	12	P:GO:0040010; P:GO:0008340; P:GO:0040035; C:GO:0000015; C:GO:0009986; P:GO:0002119; F:GO:0000287; P:GO:0006096; P:GO:0006898; P:GO:0009792; P:GO:0040018; F:GO:0004634	-	-	EC:4.2.1.11	IPR000941; IPR020809; IPR020810; G3DSA:3.20.20.120 (GENE3D), PTHR11902 (PANTHER), PS51257 (PROFILE), SSF51604 (SUPERFAMILY), SSF54826 (SUPERFAMILY)
AAD51334.1	1.489E-13	1	F:GO:0030414	-	-		IPR002223; IPR020901; PTHR10083 (PANTHER), PTHR10083:SF39 (PANTHER), SignalP (SIGNALP)
NP_001024594.1	1.602E-44	5	C:GO:0005576; P:GO:0050830; P:GO:0009792; F:GO:0003796; P:GO:0050829	-	-	EC:3.2.1.17	IPR008597; PTHR11195:SF7 (PANTHER), SignalP (SIGNALP), SSF53955 (SUPERFAMILY)
		0					SignalP (SIGNALP)
CAO00417.1	7.183E-46	1	P:GO:0040011	-	-		IPR001283; IPR002413; IPR014044; IPR018244; PTHR10334:SF11 (PANTHER), SignalP (SIGNALP)
AAG29103.2	5.901E-52	1	F:GO:0008233	-	-		IPR000718; IPR018497; G3DSA:3.40.390.10 (GENE3D), PTHR11733:SF15 (PANTHER), SSF55486 (SUPERFAMILY)
NP_001024812.1	2.043E-07	0					no IPS match
ABC86956.1	0	4	P:GO:0045454; F:GO:0003756; C:GO:0016020; C:GO:0005783	-	-	EC:5.3.4.1	no IPS match
Q90WA0.1	4.582E-07	0		17	F:GO:0004222; P:GO:0018298; P:GO:0006810; F:GO:0008270; C:GO:0005792; C:GO:0005578; F:GO:0008237; C:GO:0031012; P:GO:0010951; C:GO:0005576; F:GO:0030414; F:GO:0008233; P:GO:0009405; F:GO:0004867; P:GO:0051604; F:GO:0005488; P:GO:0010466		no IPS match

		0					no IPS match
CAH23216.1	0.0006187	0					no IPS match
NP_503068.1	0	5	F:GO:0005524; P:GO:0055114; P:GO:0006950; F:GO:0005515; F:GO:0032440	-	-	EC:1.3.1.74	IPR001023; IPR013126; IPR018181; G3DSA:2.60.34.10 (GENE3D), G3DSA:3.30.30.30 (GENE3D), G3DSA:3.30.420.40 (GENE3D), G3DSA:3.90.640.10 (GENE3D), PTHR19375:SF1 (PANTHER), SSF100920 (SUPERFAMILY), SSF100934 (SUPERFAMILY), SSF53067 (SUPERFAMILY)
ABJ97284.1	3.579E-60	5	P:GO:0019915; C:GO:0005856; C:GO:0005737; F:GO:0005198; C:GO:0031143	-	-		IPR000535; IPR008962; PTHR22920 (PANTHER)
NP_741242.1	1.132E-25	1	F:GO:0008236	-	-		no IPS match
		0					SignalP (SIGNALP)
NP_492761.1	2.933E-74	29	C:GO:0030027; F:GO:0005515; P:GO:0002762; P:GO:0007155; P:GO:0040007; F:GO:0004550; P:GO:0006898; P:GO:0006241; P:GO:0006355; P:GO:0030819; C:GO:0005882; F:GO:0046872; P:GO:0006165; C:GO:0001726; P:GO:0009792; F:GO:0003677; P:GO:0045618; P:GO:0006183; P:GO:0002119; P:GO:0006228; P:GO:0050679; F:GO:0003700; C:GO:0048471; C:GO:0005886; C:GO:0005634; F:GO:0005524; P:GO:0043066; P:GO:0040035; C:GO:0005739	-	-	EC:2.7.4.6	IPR001564; IPR023005; SignalP (SIGNALP)
		0					no IPS match
NP_497273.2	3.503E-13	0		1	F:GO:0008289		IPR008632
CAA86821.1	9.657E-21	1	F:GO:0019841	-	-		no IPS match
		0					IPR000436
AAO63576.1	5.463E-19	0		2	P:GO:0040011; C:GO:0005576		IPR001283; IPR014044; PTHR10334:SF11 (PANTHER), SignalP (SIGNALP)

NP_492616.1	2.473E-29	4	F:GO:0046872; F:GO:0008233; P:GO:0000003; P:GO:0007413	-	-		IPR001506; G3DSA:3.40.390.10 (GENE3D), PTHR10127 (PANTHER), PTHR10127:SF73 (PANTHER), SSF55486 (SUPERFAMILY)
NP_491198.1	4.638E-46	5	F:GO:0042802; P:GO:0006879; F:GO:0008199; P:GO:0040010; P:GO:0006826	-	-		no IPS match
XP_002740738.1	8.174E-08	0		13	C:GO:0009986; F:GO:0004888; C:GO:0016021; F:GO:0005529; P:GO:0006897; C:GO:0005576; P:GO:0007165; F:GO:0004872; P:GO:0009405; P:GO:0008150; P:GO:0007155; F:GO:0005537; F:GO:0005488		IPR001304; IPR016186; IPR016187; PTHR22802 (PANTHER), PTHR22802:SF5 (PANTHER), SignalP (SIGNALP)
NP_509528.1	2.297E-67	6	C:GO:0016021; F:GO:0004222; F:GO:0003676; P:GO:0032259; P:GO:0006508; F:GO:0008168	-	-	EC:3.4.24.0; EC:2.1.1.0	IPR000718; IPR002052; IPR018497; G3DSA:3.40.390.10 (GENE3D), PTHR11733:SF15 (PANTHER), SSF55486 (SUPERFAMILY)
XP_002647662.1	1.649E-31	0		4	P:GO:0006915; F:GO:0030414; F:GO:0004867; C:GO:0005576		IPR002223; IPR020901; PTHR10083 (PANTHER), PTHR10083:SF39 (PANTHER), SignalP (SIGNALP)
XP_002630572.1	5.836E-11	0					SignalP (SIGNALP)
NP_505848.1	4.43E-141	6	P:GO:0002009; P:GO:0018996; P:GO:0008340; P:GO:0000003; P:GO:0040010; P:GO:0002119	-	-		IPR000276; SignalP (SIGNALP)
ACX53263.1	2.447E-48	4	P:GO:0008340; P:GO:0040010; F:GO:0005515; F:GO:0016740	-	-		IPR004045; IPR012335; IPR012336; PTHR11571 (PANTHER), PTHR11571:SF6 (PANTHER)
		0					SignalP (SIGNALP)
CAJ43810.1	1.96E-160	2	F:GO:0046872; F:GO:0008237	-	-		IPR001506; IPR006026; IPR013032; G3DSA:3.40.390.10 (GENE3D), PTHR10127 (PANTHER), PTHR10127:SF65 (PANTHER), SSF55486 (SUPERFAMILY)

NP_001033512.1	1.839E-40	16	P:GO:0050872; P:GO:0008340; F:GO:0005515; P:GO:0006469; P:GO:0050873; C:GO:0005829; P:GO:0051384; P:GO:0001816; P:GO:0008284; P:GO:0006631; P:GO:0071285; P:GO:0042493; F:GO:0008289; P:GO:0016481; P:GO:0050729; P:GO:0042632	-	-		no IPS match
XP_002638373.1	1.887E-79	3	P:GO:0006457; P:GO:0009792; F:GO:0003755	-	-	EC:5.2.1.8	no IPS match
XP_002648175.1	8.411E-52	4	F:GO:0004519; P:GO:0006449; C:GO:0005634; C:GO:0005739	-	-		IPR006056; IPR006175; IPR013813; IPR019897
ABB53347.1	9.284E-16	0		2	P:GO:0006898; C:GO:0005576		IPR014044; SignalP (SIGNALP)
NP_501087.1	1E-153	15	P:GO:0034656; P:GO:0046700; P:GO:0048534; P:GO:0009168; P:GO:0002684; F:GO:0004000; P:GO:0009792; P:GO:0030154; P:GO:0048523; P:GO:0048522; P:GO:0050896; P:GO:0042278; P:GO:0046649; P:GO:0051239; C:GO:0016020	-	-	EC:3.5.4.4	SignalP (SIGNALP)
XP_002646204.1	5.188E-46	18	P:GO:0009653; P:GO:0008340; P:GO:0003006; P:GO:0048610; C:GO:0015629; P:GO:0007283; P:GO:0040011; P:GO:0048468; C:GO:0005737; P:GO:0051179; F:GO:0003779; P:GO:0045927; P:GO:0009792; P:GO:0008361; P:GO:0007015; P:GO:0048477; P:GO:0007399; P:GO:0000910	-	-		no IPS match
CAA96571.1	4.75E-122	4	P:GO:0008219; F:GO:0016787; F:GO:0005515; P:GO:0040011	-	-		IPR001461; IPR001969; IPR009007; IPR021109; PTHR13683:SF53 (PANTHER), SignalP (SIGNALP)
CAR63538.1	7.07E-103	0					IPR021010; SSF141739 (SUPERFAMILY)
		0					no IPS match
		0					SignalP (SIGNALP)
AAF36480.1	9.571E-21	4	P:GO:0008340; P:GO:0040010; F:GO:0005515; F:GO:0016740	-	-		IPR004046; IPR010987; IPR017933; PTHR11571 (PANTHER), PTHR11571:SF6 (PANTHER)
CAD29445.1	0	8	P:GO:0006457; C:GO:0005875; F:GO:0008415; F:GO:0016757; C:GO:0005811; P:GO:0045454; F:GO:0003756; C:GO:0005783	-	-	EC:5.3.4.1	no IPS match

AAV68383.1	4.94E-168	5	C:GO:0016021; F:GO:0008237; P:GO:0006508; F:GO:0008270; F:GO:0004177	-	-	EC:3.4.11.0	IPR001930; IPR014782; PTHR11533:SF29 (PANTHER), SSF55486 (SUPERFAMILY), SSF63737 (SUPERFAMILY)
CAO00417.1	2.228E-55	1	P:GO:0040011	-	-		IPR001283; IPR014044; IPR018244; PTHR10334:SF11 (PANTHER)
NP_741241.1	8.05E-57	1	F:GO:0008236	-	-		no IPS match
XP_002641290.1	5.458E-16	2	P:GO:0032502; P:GO:0032501	-	-		IPR002557; G3DSA:2.170.140.10 (GENE3D), PTHR11177 (PANTHER), PTHR11177:SF37 (PANTHER)
NP_493258.1	1.287E-42	9	C:GO:0005737; P:GO:0009792; F:GO:0003779; P:GO:0000910; P:GO:0000003; P:GO:0008340; P:GO:0040011; C:GO:0015629; P:GO:0030036	-	-		no IPS match
CAJ45481.1	7.76E-123	8	C:GO:0005737; P:GO:0009792; F:GO:0008233; P:GO:0040015; C:GO:0045177; C:GO:0043231; P:GO:0000003; P:GO:0006508	-	-		IPR001096; PTHR12000:SF4 (PANTHER), SignalP (SIGNALP)
AAG29105.1	2.55E-100	3	C:GO:0016021; F:GO:0004222; P:GO:0006508	-	-	EC:3.4.24.0	IPR000718; IPR018497; G3DSA:3.40.390.10 (GENE3D), PTHR11733:SF15 (PANTHER), SSF55486 (SUPERFAMILY)
CAA96571.1	5.487E-67	4	P:GO:0008219; F:GO:0016787; F:GO:0005515; P:GO:0040011	-	-		IPR001461; IPR009007; IPR021109; PTHR13683:SF53 (PANTHER)
XP_002631588.1	0	1	F:GO:0003824	-	-		IPR000718; IPR008753; IPR018497; G3DSA:3.40.390.10 (GENE3D), PTHR11733:SF15 (PANTHER), SignalP (SIGNALP), SSF55486 (SUPERFAMILY)
AAD13339.1	4.324E-12	0		3	P:GO:0040011; P:GO:0006898; C:GO:0005576		IPR001283; IPR014044; SignalP (SIGNALP)
		0					no IPS match
NP_505622.1	7.147E-53	0					SignalP (SIGNALP)
XP_002641997.1	2.272E-50	1	F:GO:0005488	-	-		IPR001079; IPR008985; IPR013320; PTHR11346:SF2 (PANTHER)
		0					no IPS match
CAD20812.1	9.479E-05	0					IPR003582

XP_002642617.1	4.97E-127	9	P:GO:0009792; C:GO:0045239; F:GO:0005515; P:GO:0006106; P:GO:0040010; P:GO:0000003; P:GO:0006099; P:GO:0006898; F:GO:0004333	-	-	EC:4.2.1.2	IPR000362; IPR008948; IPR018951; IPR020557; IPR022761; G3DSA:1.10.40.30 (GENE3D), G3DSA:1.20.200.10 (GENE3D), PTHR11444 (PANTHER), PTHR11444:SF1 (PANTHER)
ACC54555.1	1.284E-52	16	P:GO:0035468; P:GO:0042325; P:GO:0006955; P:GO:0009966; P:GO:0002684; P:GO:0042127; P:GO:0051047; P:GO:0032268; F:GO:0004167; P:GO:0048523; P:GO:0048583; N:GO:0035556; P:GO:0006950; P:GO:0033036; F:GO:0005488; P:GO:0048598	-	-	EC:5.3.3.12	IPR001398; IPR014347; PTHR11954:SF4 (PANTHER), SSF55331 (SUPERFAMILY)
ACT34056.1	0	17	P:GO:0000003; F:GO:0070403; F:GO:0070728; F:GO:0004353; P:GO:0040010; C:GO:0005759; F:GO:0043531; F:GO:0005525; C:GO:0005743; P:GO:0006537; P:GO:0007616; F:GO:0004352; P:GO:0019459; P:GO:0055114; F:GO:0019899; F:GO:0005524; P:GO:0032024	-	-	EC:1.4.1.3; EC:1.4.1.2	IPR006095; IPR006096; IPR006097; IPR014362; IPR016040; G3DSA:1.10.287.140 (GENE3D), G3DSA:3.40.192.10 (GENE3D), PTHR11606 (PANTHER), SSF51735 (SUPERFAMILY), SSF53223 (SUPERFAMILY)
NP_499203.1	2.339E-52	0					no IPS match
NP_501838.2	3.65E-153	0					IPR021010; SSF141739 (SUPERFAMILY)
ACS37721.1	1.154E-61	1	F:GO:0005529	-	-		IPR001304; IPR016186; IPR016187; PTHR22801 (PANTHER), PTHR22801:SF9 (PANTHER)
NP_509267.2	3.204E-26	0		1	F:GO:0003824		IPR001254; IPR009003; G3DSA:2.40.10.10 (GENE3D), PTHR19355 (PANTHER), PTHR19355:SF135 (PANTHER), SignalP (SIGNALP)
ACX53263.1	1.88E-40	3	F:GO:0016740; F:GO:0005515; P:GO:0008340	-	-		IPR004046; IPR010987; IPR017933; PTHR11571 (PANTHER), PTHR11571:SF6 (PANTHER)

NP_498642.1	2.17E-108	12	P:GO:0040010; P:GO:0048873; P:GO:0000003; P:GO:0006108; P:GO:0006099; F:GO:0004333; F:GO:0005515; C:GO:0045239; C:GO:0005759; P:GO:0006898; P:GO:0009792; P:GO:0006106	-	-	EC:4.2.1.2	IPR000362; IPR008948; IPR022761; G3DSA:1.10.275.10 (GENE3D), G3DSA:1.20.200.10 (GENE3D), PTHR11444 (PANTHER), PTHR11444:SF1 (PANTHER), SignalP (SIGNALP)
AAN11401.1	2.43E-117	1	F:GO:0008233	-	-		IPR001506; IPR003582; IPR006026; IPR013032; G3DSA:3.40.390.10 (GENE3D), PTHR10127 (PANTHER), PTHR10127:SF65 (PANTHER), SSF55486 (SUPERFAMILY)
NP_498341.1	2.416E-24	0					SignalP (SIGNALP)
AAN11401.1	3.819E-50	2	F:GO:0046872; F:GO:0008237	-	-		IPR000859; IPR013032; PTHR10127 (PANTHER), PTHR10127:SF60 (PANTHER)
XP_001373707.1	2.398E-07	0		17	P:GO:0016477; F:GO:0004252; C:GO:0016021; C:GO:0016020; F:GO:0016787; C:GO:0019897; F:GO:0008236; F:GO:0003824; C:GO:0005576; F:GO:0005044; F:GO:0008233; C:GO:0016323; P:GO:0006508; P:GO:0008152; C:GO:0005887; C:GO:0005886; C:GO:0005615		IPR001254; IPR001314; IPR009003; IPR018114; G3DSA:2.40.10.10 (GENE3D), PTHR19355 (PANTHER), PTHR19355:SF174 (PANTHER)
