# Supplementary material for: Proteomic Analysis of Excretory-Secretory Products of Heligmosomoides polygyrus Assessed with Next-Generation Sequencing Transcriptomic Information
Source: PLoS Negl Trop Dis. 2011 Oct 25;5(10):e1370. doi: 10.1371/journal.pntd.0001370 (PMC3201918; doi:10.1371/journal.pntd.0001370)
Supplement: Table S4 — Enrichment analysis of GO terms in the ESP dataset when compared to the transcriptome dataset. (PDF) [file pntd.0001370.s004.pdf]

Table S4. Enrichment analysis of GO terms in the ESP dataset when compared to the transcriptome dataset.

	GO	Name	FDR	FWER	p-Value	# Seq ESP	#Seqs Total	% ESP	%Transcriptome	Fold increase
**Molecular Function**	GO:0005344	oxygen transporter activity	2.4E-07	1.2E-07	3.9E-10	7	16	6.2	0.1	55.8
	GO:0019825	oxygen binding	5.2E-07	6.4E-07	2.4E-09	7	22	6.2	0.2	40.6
	GO:0008233	peptidase activity	4.9E-06	7.4E-06	1.9E-08	20	544	17.7	3.8	4.7
	GO:0008289	lipid binding	2.0E-04	3.4E-04	8.6E-07	10	156	8.8	1.1	8.2
	GO:0005501	retinoid binding	2.8E-04	6.4E-04	1.8E-06	4	8	3.5	0.1	63.8
	GO:0019840	isoprenoid binding	4.5E-04	1.1E-03	2.7E-06	4	9	3.5	0.1	56.7
	GO:0008237	metallopeptidase activity	5.2E-04	1.4E-03	3.9E-06	10	186	8.8	1.3	6.9
	GO:0020037	heme binding	5.2E-04	1.6E-03	4.4E-06	7	76	6.2	0.5	11.7
	GO:0046906	tetrapyrrole binding	1.0E-03	3.3E-03	9.5E-06	7	86	6.2	0.6	10.4
	GO:0005506	iron ion binding	3.7E-03	1.3E-02	4.0E-05	8	150	7.1	1.0	6.8
	GO:0019841	retinol binding	3.9E-03	1.5E-02	4.1E-05	3	6	2.7	0.0	63.8
	GO:0070011	peptidase activity, acting on L-amino acid peptides	2.5E-02	1.1E-01	2.1E-04	12	427	10.6	3.0	3.6
	GO:0016860	intramolecular oxidoreductase activity	2.5E-02	1.1E-01	2.1E-04	4	33	3.5	0.2	15.5
	GO:0009132	nucleoside diphosphate metabolic process	2.5E-02	1.2E-01	2.1E-04	3	12	2.7	0.1	31.9
**Biological Process**	GO:0008340	determination of adult lifespan	1.2E-07	3.0E-08	0.0E+00	28	613	24.8	4.3	5.8
	GO:0015671	oxygen transport	4.1E-07	3.1E-07	1.0E-09	7	19	6.2	0.1	47.0
	GO:0015669	gas transport	5.2E-07	6.4E-07	2.4E-09	7	22	6.2	0.2	40.6
	GO:0032501	multicellular organismal process	2.8E-04	5.6E-04	1.8E-06	33	1730	29.2	12.0	2.4
	GO:0040010	positive regulation of growth rate	1.4E-02	5.9E-02	1.3E-04	26	1487	23.0	10.3	2.2
	GO:0040009	regulation of growth rate	1.4E-02	5.9E-02	1.3E-04	26	1489	23.0	10.3	2.2
	GO:0045927	positive regulation of growth	2.9E-02	1.4E-01	3.4E-04	27	1675	23.9	11.6	2.1
	GO:0040008	regulation of growth	4.1E-02	2.0E-01	4.0E-04	28	1784	24.8	12.4	2.0

Summary of the ESP gene ontology (GO) terms enrichment analysis using the statistical framework GOSSIP feature of BLAST2GO. Fisher`s exact test with testing correction using False Discovery Rate (FDR) values of 0.05 to identify enriched GO terms in the ESP when compared to the total transcriptome annotation.
