# Supplementary material for: Proteomic Analysis of Excretory-Secretory Products of Heligmosomoides polygyrus Assessed with Next-Generation Sequencing Transcriptomic Information
Source: PLoS Negl Trop Dis. 2011 Oct 25;5(10):e1370. doi: 10.1371/journal.pntd.0001370 (PMC3201918; doi:10.1371/journal.pntd.0001370)
Supplement: Table S5 — Enrichment analysis of InterPro domains and families in the ESP dataset versus the transcriptome dataset. (PDF) [file pntd.0001370.s005.pdf]

Table S5. Enrichment analysis of InterPro domains and families in the ESP dataset versus the transcriptome dataset.

	InterPro	InterPro Entry Name	FDR	p-Value	#Seq ESP	#Seq Total	%ESP	% Transcriptome	Fold increase
Domains	IPR014044	CAP domain	3.95E-26	8.69E-30	25	136	12.02	0.40	29.7
	IPR018497	Peptidase M13, neprilysin, C-terminal	7.83E-06	1.03E-08	5	13	2.4	0.04	62.1
	IPR001747	Lipid transport protein, N-terminal	1.04E-05	1.60E-08	5	14	2.4	0.04	57.7
	IPR008753	Peptidase M13	1.96E-05	3.45E-08	5	16	2.4	0.05	50.5
	IPR015819	Lipid transport protein, beta-sheet shell	4.12E-05	9.05E-08	5	19	2.4	0.06	42.5
	IPR006026	Peptidase, metallopeptidase	1.10E-04	2.91E-07	6	44	2.88	0.13	22.0
	IPR011030	Vitellinogen, superhelical	1.10E-04	2.83E-07	4	10	1.92	0.03	64.6
	IPR001506	Peptidase M12A, astacin	1.21E-04	3.46E-07	7	73	3.37	0.22	15.5
	IPR015816	Vitellinogen, beta-sheet N-terminal	9.39E-04	3.10E-06	4	17	1.92	0.05	38.0
	IPR009050	Globin-like	1.13E-03	3.96E-06	4	18	1.92	0.05	35.9
	IPR012292	Globin	1.34E-03	4.99E-06	4	19	1.92	0.06	34.0
	IPR015255	Vitellinogen, open beta-sheet	7.52E-03	3.64E-05	3	11	1.44	0.03	44.0
	IPR004046	Glutathione S-transferase, C-terminal	9.55E-03	4.83E-05	3	12	1.44	0.04	40.4
	IPR000436	Sushi/SCR/CCP	2.37E-02	1.30E-04	5	79	2.4	0.23	10.2
	IPR017933	Glutathione S-transferase/chloride channel, C-terminal	2.55E-02	1.46E-04	3	17	1.44	0.05	28.5
Families	IPR001283	Allergen V5/Tpx-1-related	1.77E-19	7.80E-23	18	86	8.65	0.26	33.8
	IPR000718	Peptidase M13, neprilysin	4.06E-09	3.57E-12	8	27	3.85	0.08	48.0
	IPR002413	Ves allergen	3.47E-07	3.81E-10	6	16	2.88	0.05	60.6
	IPR012085	Globin, nematode	3.16E-03	1.25E-05	3	8	1.44	0.02	60.6
	IPR008632	Nematode fatty acid retinoid binding	4.47E-03	1.87E-05	3	9	1.44	0.03	53.8
	IPR001534	Transthyretin-like	4.70E-03	2.07E-05	5	54	2.4	0.16	15.0
	IPR009283	Apyrase	7.52E-03	3.64E-05	3	11	1.44	0.03	44.0
	IPR000971	Globin, subset	1.87E-02	9.85E-05	3	15	1.44	0.04	32.3

Fisher`s exact test with testing correction using False Discovery Rate (FDR) values <0.05 were used to identify enriched Interpro domains and families in the ESP when compared to the total transcriptome annotation.
