# Supplementary material for: Proteomic Analysis of Excretory-Secretory Products of Heligmosomoides polygyrus Assessed with Next-Generation Sequencing Transcriptomic Information
Source: PLoS Negl Trop Dis. 2011 Oct 25;5(10):e1370. doi: 10.1371/journal.pntd.0001370 (PMC3201918; doi:10.1371/journal.pntd.0001370)
Supplement: Table S1 — Proteins identified in H. polygyrus ESP through search in UniProt database taxon identifier 6231, Nematoda. (PDF) [file pntd.0001370.s001.pdf]

Table S1. Proteins identified in *H. polygyrus* ESP through search in UniProt database taxon identifier 6231, Nematoda.

Protein name	Uniprot Protein accession numbers	Protein molecular weight (kDa)	Protein identification probability	Number of unique peptides	Number of total spectra	Percentage of total spectra	Percentage sequence coverage
GLB2_NIPBR Myoglobin OS=Nippostrongylus brasiliensis GN=GLBB PE=2 SV=1|6231	P51535	17.3	100.00%	3	4	0.03%	19.50%
A8XPS2_CAEBR C. briggsae CBR-VIT-1 protein OS=Caenorhabditis briggsae GN=cbr-vit-1 PE=4 SV=1|6231	A8XPS2	187.9	99.80%	2	12	0.09%	1.18%
C7TNU5_9BILA Putative Heat Shock Protein (Fragment) OS=Angiostrongylus cantonensis GN=hsp-60 PE=2 SV=1|6231	C7TNU5	44.1	99.80%	2	2	0.02%	6.01%
Q6JKL1_MELIC 14-3-3b protein OS=Meloidogyne incognita PE=2 SV=1|6231	Q6JKL1	28.6	100.00%	4	9	0.07%	21.90%
A8X7W5_CAEBR Fructose-bisphosphate aldolase OS=Caenorhabditis briggsae GN=cbr-aldo-2 PE=3 SV=1|6231	A8X7W5	38.9	100.00%	3	4	0.03%	14.50%
A7XXV6_BURXY Actin OS=Bursaphelenchus xylophilus PE=2 SV=1|6231	A7XXV6,B0LJD2,D1MBR7,D1MX15,P10983,Q6A8K1,Q8MTG0,Q95ZL1	41.8	100.00%	5	6	0.05%	22.60%
A8PKM4_BRUMA Triosephosphate isomerase OS=Brugia malayi GN=Bm1_29130 PE=3 SV=1|6231	A8PKM4	27.1	100.00%	3	4	0.03%	13.80%
D3KZ67_NIPBR Vitellogenin-1 (Fragment) OS=Nippostrongylus brasiliensis GN=vit-1 PE=2 SV=1|6231	D3KZ67	14.9	100.00%	2	8	0.06%	10.20%
D3KZ66_NIPBR Major sperm protein 1 (Fragment) OS=Nippostrongylus brasiliensis GN=msp1 PE=2 SV=1|6231	D3KZ66	13.2	99.80%	2	3	0.02%	24.80%
Q23426_CAEEL Galectin LEC-5 OS=Caenorhabditis elegans GN=lec-5 PE=1 SV=2|6231	Q23426	35.4	99.90%	2	2	0.02%	6.05%
ENO_CAEEL Enolase OS=Caenorhabditis elegans GN=enol-1 PE=1 SV=3|6231	Q27527	46.6	100.00%	5	6	0.05%	19.60%
Q8WQ12_OSTOS Putative ES protein F7 (Fragment) OS=Ostertagia ostertagi PE=2 SV=1|6231	Q8WQ12	19.8	99.80%	2	4	0.03%	14.90%
A8X3V3_CAEBR Peptidyl-prolyl cis-trans isomerase OS=Caenorhabditis briggsae GN=CBG07031 PE=3 SV=1|6231	A8X3V3	18.6	99.80%	2	3	0.02%	15.10%
Q95WS8_HAECO Vitellogenin (Fragment) OS=Haemonchus contortus GN=VIT PE=2 SV=1|6231	Q95WS8	24.6	99.80%	2	6	0.05%	5.29%
Q962H2_9BILA Putative retinol-binding protein OS=Heligmosomoides polygyrus PE=2 SV=1|6231	Q962H2	19.4	100.00%	5	6	0.05%	38.80%
Q2HZY3_9BILA Protein disulfide isomerase (Fragment) OS=Teladorsagia circumcincta PE=2 SV=1|6231	Q2HZY3,Q95PP0	55.0	100.00%	2	2	0.02%	3.85%
A8NSV0_BRUMA Ubiquitin, putative OS=Brugia malayi GN=Bm1_09050 PE=4 SV=1|6231	A8NSV0,A8PH75,A8PJI9,A8QFW7,A8X7Y8,A8XMR9,B3GD96,P0CG71,P49632,Q25400,Q45UA3,Q8MYQ4,Q8T4K9,Q8T4L0,Q8T4L5	29.1	99.90%	2	2	bl	6.98%
A8X890_CAEBR C. briggsae CBR-FUM-1 protein OS=Caenorhabditis briggsae GN=cbr-fum-1 PE=4 SV=1|6231	A8X890,O17214	53.6	99.80%	2	2	0.02%	5.99%
Q9NJQ6_9BILA Glutathione S-transferase 2 (Fragment) OS=Heligmosomoides polygyrus PE=1 SV=1|6231	Q9NJQ6	23.1	100.00%	7	8	0.06%	52.50%
Q6J3P3_HAECO Peroxiredoxin OS=Haemonchus contortus PE=2 SV=1|6231	Q6J3P3	21.9	100.00%	4	6	0.05%	21.40%
